# Diverse human astrocyte and microglial transcriptional responses to Alzheimer’s pathology

**DOI:** 10.1007/s00401-021-02372-6

**Published:** 2021-11-12

**Authors:** Amy M. Smith, Karen Davey, Stergios Tsartsalis, Combiz Khozoie, Nurun Fancy, See Swee Tang, Eirini Liaptsi, Maria Weinert, Aisling McGarry, Robert C. J. Muirhead, Steve Gentleman, David R. Owen, Paul M. Matthews

**Affiliations:** 1 UK Dementia Research Institute, Imperial College London, London, UK; 2 E515, Department of Brain Sciences, Imperial College London, Hammersmith Hospital, DuCane Road, London, WC12 0NN UK; 3 Department of Psychiatry, University of Geneva, Geneva, Switzerland

**Keywords:** Alzheimer’s disease, Microglia, Astrocytes, Amyloid-beta, Tau, snRNA sequencing

## Abstract

To better define roles that astrocytes and microglia play in Alzheimer’s disease (AD), we used single-nuclei RNA-sequencing to comprehensively characterise transcriptomes in astrocyte and microglia nuclei selectively enriched during isolation *post-mortem* from neuropathologically defined AD and control brains with a range of amyloid-beta and phospho-tau (pTau) pathology. Significant differences in glial gene expression (including AD risk genes expressed in both the astrocytes [*CLU*, *MEF2C*, *IQCK*] and microglia [*APOE*, *MS4A6A*, *PILRA*]) were correlated with tissue amyloid or pTau expression. The differentially expressed genes were distinct between with the two cell types and pathologies, although common (but cell-type specific) gene sets were enriched with both pathologies in each cell type. Astrocytes showed enrichment for proteostatic, inflammatory and metal ion homeostasis pathways. Pathways for phagocytosis, inflammation and proteostasis were enriched in microglia and perivascular macrophages with greater tissue amyloid, but IL1-related pathway enrichment was found specifically in association with pTau. We also found distinguishable sub-clusters in the astrocytes and microglia characterised by transcriptional signatures related to either homeostatic functions or disease pathology. Gene co-expression analyses revealed potential functional associations of soluble biomarkers of AD in astrocytes (*CLU*) and microglia (*GPNMB*). Our work highlights responses of both astrocytes and microglia for pathological protein clearance and inflammation, as well as glial transcriptional diversity in AD.

**Supplementary Information:**

The online version contains supplementary material available at 10.1007/s00401-021-02372-6.

## Introduction

While Alzheimer’s disease (AD) is classically characterised by the presence of amyloid plaques, phosphorylated tau tangles in neurons, and neurodegeneration, it has become clear in recent years that astrocytes and microglia play important and potentially causal roles in the disease [3]. Activated astrocytes and microglia are found around amyloid plaques [52] and genes associated with AD risk are enriched in both cell types, particularly in microglia [12, 56]. However, the mechanisms by which microglia and astrocytes contribute to disease genesis, progression, and response still are poorly defined. Mouse transgenic models have suggested pathways that may drive processes central to amyloid or pTau pathologies, but a recent meta-analysis suggested that the diversity of glial responses in late-onset sporadic Alzheimer’s disease is not well captured by mouse models [70].

Single-nuclei RNA-sequencing (snRNASeq) from *post-mortem* brain tissue [30, 32] is transforming the potential to characterise the molecular neuropathology of AD at the level of single cells [18, 36, 76]. However, because of their lower cellular abundance in the brain, microglia and astrocytes have been poorly represented in most studies published to date (e.g., 449–3982 microglia, representing only 1–3% of the total nuclei annotated [18, 36, 41]), limiting the depth to which they can be characterised. Astrocytes and microglia also have highly heterogeneous phenotypes [2, 11, 49]. To address these limitations, we employed a robust negative-selection approach (closely related to that used in a recent report [16]) that enriches for them in nuclei isolated from *post-mortem* tissue. This allowed us to characterise snRNASeq transcriptomes from much larger numbers of these nuclei (52,706 astrocytes and 27,592 microglia) efficiently. After quantitative assessments of neuropathological features in each brain region, we generated transcriptional signatures associated with amyloid-beta or pTau pathology in non-disease control (NDC) and AD brains. These data allowed us to develop comprehensive descriptions of gene co-expression networks that provide both further insights into the different pathology-specific transcriptional responses of astrocytes and microglia in AD and evidence for cell-type specific functions of genes associated with risks for AD. Transcription factors potentially responsible for the differential gene expression with pathology were identified from these co-expression modules. We confirmed our major observations by re-analyses of data from four previously reported AD snRNASeq studies. Our work provides new insights into linked glial-specific responses mediating pathological protein clearance and inflammation, highlighting evidence for distinct and diverse roles of astrocytes and microglia in AD.

## Methods

### Brain tissue

This study was carried out in accordance with the Regional Ethics Committee and Imperial College Use of Human Tissue guidelines. Cases were selected from the London Neurodegeneration (King’s College London) and Parkinson’s UK (Imperial College London) Brain Banks. Entorhinal and somatosensory cortex from 6 non-diseased control (NDC) cases (Braak stage 0–II) and 6 AD cases (Braak stage III–VI) were used (total of 24 brain samples) (Supplementary Table 1, online resource). Cortical samples from two regions were prepared from each brain to characterise transcript expression with both higher (entorhinal cortex) and lower (somatosensory cortex) tissue densities of pTau in neurofibrillary tangles and amyloid-beta plaques. Brains used for this study excluded cases with clinical or pathological evidence for small vessel disease, stroke, cerebral amyloid angiopathy, diabetes, Lewy body pathology (TDP-43), or other neurological diseases. Where the information was available, cases were selected for a brain pH greater than 6 and all but one had a post-mortem delay of less than 24 h (Table 1).

**Table 1:** Cohort information

	M:F ratio	Age at death (years, mean ± SD)	Post-mortem delay (hr, mean ± SD)	RIN (mean ± SD)
Non-diseased controls (Braak 0–II)	4:2	79.3 ± 6.5	18 ± 6.9	4.9 ± 2.0
Alzheimer’s disease (Braak III–VI)	4:2	81 ± 6.8	22.1 ± 15.9	7.1 ± 0.7

### Immunohistochemistry

Immunohistochemical staining was performed on formalin-fixed paraffin-embedded sections from homologous regions of each brain used for snRNASeq. Standard immunohistochemical procedures were followed using the ImmPRESS Polymer (Vector Laboratories) and SuperSensitive Polymer-HRP (Biogenex) kits (Table 2). Briefly, endogenous peroxidase activity and non-specific binding were blocked with 0.3% H_2_O_2_ and 10% normal horse serum, respectively. Primary antibodies were incubated overnight at 4 °C. Species-specific ImmPRESS or SuperSensitive kits and DAB were used for antibody visualisation. Counter-staining for nuclei was performed by incubating tissue sections in haematoxylin (TCS Biosciences) for 2 min. AD pathology was assessed by amyloid plaque (4G8, BioLegend 17–24) and pTau (AT8, NBS Biologics) staining. GPNMB staining in microglia was assessed using R&D antibody AF2550.

**Table 2:** List of antibodies and immunostaining methods

Antigen	Antibody	Dilution	Antigen retrieval	IHC staining kit
Amyloid-β	4G8 BioLegend (800702)	1:15,000	Citrate buffer, in steamer	Supersensitive Kit
pTau	AT8 Invitrogen (MN1020)	1:1600	Citrate buffer, in steamer	Supersensitive Kit
GPNMB	R&D (AF2550)	1:500	Citrate buffer, in steamer	ImmPRESS Kit

### Quantitative image analysis

Labelled tissue sections were imaged using a Leica Aperio AT2 Brightfield Scanner (Leica Biosystems). Images were analysed using HALO software (Indica Labs, Version 2.3.2989.34). The following image analysis macros were used for the study: area quantification macro (amyloid), multiplex macro (pTau), and microglia macro (GPNMB). GraphPad Prism version 8 for Windows (GraphPad Software, La Jolla, CA, USA) was used for plotting immunohistochemical results and performing statistical analysis. A Mann–Whitney test was used to test for the significance of differences between NDC and AD.

We found that densities of pTau staining were greater in brains from the higher Braak stages, and that pTau and amyloid pathology both were found in the regions studied even in brains with lower Braak stages (Extended Data Fig. 1, online resource). This variability of local tissue pathology within similar Braak stages emphasises the potential importance of matching regional neuropathology with transcript expression for each brain individually.

### Nuclei isolation and selective glial enrichment

Fresh-frozen entorhinal and somatosensory cortical tissue blocks were sectioned to 80 μm on a cryostat and grey matter separated by scoring the tissue with sharp forceps to collect ~ 200 mg grey matter in an RNAse-free Eppendorf tube. Nuclei from NDC and AD samples were isolated in parallel using a protocol based on Krishnaswami et al*.* [30]. All steps were carried out on ice or at 4 °C. Tissue was homogenised in a 2 ml glass douncer containing homogenization buffer (0.1% Triton-X + 0.4 U/μl RNAseIn + 0.2 U/μl SUPERaseIn). The tissue homogenate was centrifuged at 1000 g for 8 min, and the majority of supernatant removed without disturbing the tissue pellet. Homogenised tissue was filtered through a 70 μm filter and centrifuged in an Optiprep (Sigma) density gradient at 13,000*g* for 40 min to remove myelin and cellular debris. The nuclei pellet was washed and filtered twice in PBS buffer (PBS + 1% BSA + 0.2 U/ml RNAseIn). These nuclei were used directly for 10X processing (see below) for analysis of the unenriched astrocytes and microglia. To enrich for these glia, separate preparations of nuclei were labelled in suspension in 1 ml PBS buffer with 1:500 anti-NeuN antibody (Millipore, MAB377, mouse) and 1:250 anti-Sox10 antibody (R&D, AF2864, goat) for 1 h on ice. Nuclei were washed twice with PBS buffer and centrifuged at 500*g* for 5 min. Nuclei were incubated with Alexa-fluor secondary antibodies at 1:1000 (goat-anti-mouse-647 and donkey-anti-goat-488) and Dapi (1:1000) for 30 min on ice and washed twice. Nuclei were FACS-sorted on a BD Aria II, using BD FACSDiva software, gating first for Dapi + ve nuclei, then singlets, and then Sox10- and NeuN-negative nuclei. A minimum of 150,000 double-negative nuclei were collected.

### Single nucleus capture and snRNA sequencing

Sorted nuclei were centrifuged at 500 g, resuspended in 50 μl PBS buffer, and counted on a LUNA-FL Dual Fluorescence Cell Counter (Logos Biosystems, L20001) using Acridine Orange dye to stain nuclei. Sufficient nuclei were added for a target of 7,000 nuclei for each library prepared. Barcoding, cDNA synthesis, and library preparation were performed using 10X Genomics Single Cell 3’ Gene Expression kit v3 with 8 cycles of cDNA amplification, after which up to 25 ng of cDNA was taken through to the fragmentation reaction and a final indexing PCR was carried out to 14 cycles. cDNA concentrations were measured using Qubit dsDNA HS Assay Kit (ThermoFisher, Q32851), and cDNA and library preparations were assessed using the Bioanalyzer High-Sensitivity DNA Kit (Agilent, 5067-4627). Samples were pooled to equimolar concentrations and the pool sequenced across 24 lanes of an Illumina HiSeq 4000 according to the standard 10X Genomics protocol. The snRNAseq data will be made available for download from the Gene Expression Omnibus (GEO) database (https://www.ncbi.nlm.nih.gov/geo/) under accession number GSE160936.

### Processing of FASTQ files, dimensionality reduction, and clustering

snRNASeq data were pre-processed and clustered using 10X Genomics Cell Ranger and Seurat analysis tools [17, 57, 76]. Illumina sequencing files were aligned to the genomic sequence (introns and exons) using GRCh38 annotation in Cell Ranger v3.1. Nuclei were identified above background by the Cell Ranger software. Filtered gene matrices from CellRanger were loaded into R where Seurat v3 single-cell analysis package was used for analysis [61]. Genes that were expressed in three or more nuclei were used for further analysis. Further QC was performed to exclude nuclei with less than 200 genes or greater than 6000 genes or 25,000 UMIs, which likely represent low quality or doublet nuclei, respectively. Nuclei with greater than 5% mitochondrial genes were also excluded. Mitochondrial gene reads were excluded. The 24 samples had an average of 3819 nuclei per sample after passing QC.

Data was normalised and scaled using the *NormalizeData* function with normalisation.method = “LogNormalize” and scale.factor = 10,000. Variable genes then were identified using the *FindVariableFeatures* function with nfeatures (number of variable genes) set to 2000. To integrate the data from all samples, *FindIntegrationAnchors* (dims = 1:20) and *IntegrateData* (dims = 1:20) functions were used. PCA analysis was run, using variable genes, for the top 30 components. Clusters were identified using *FindClusters* (resolution = 0.5) and UMAP was used for 2D visualisation of clusters (with the top 15 PCs, based on the ranking of PCs by the variance explained by each).

**Fig. 1:**
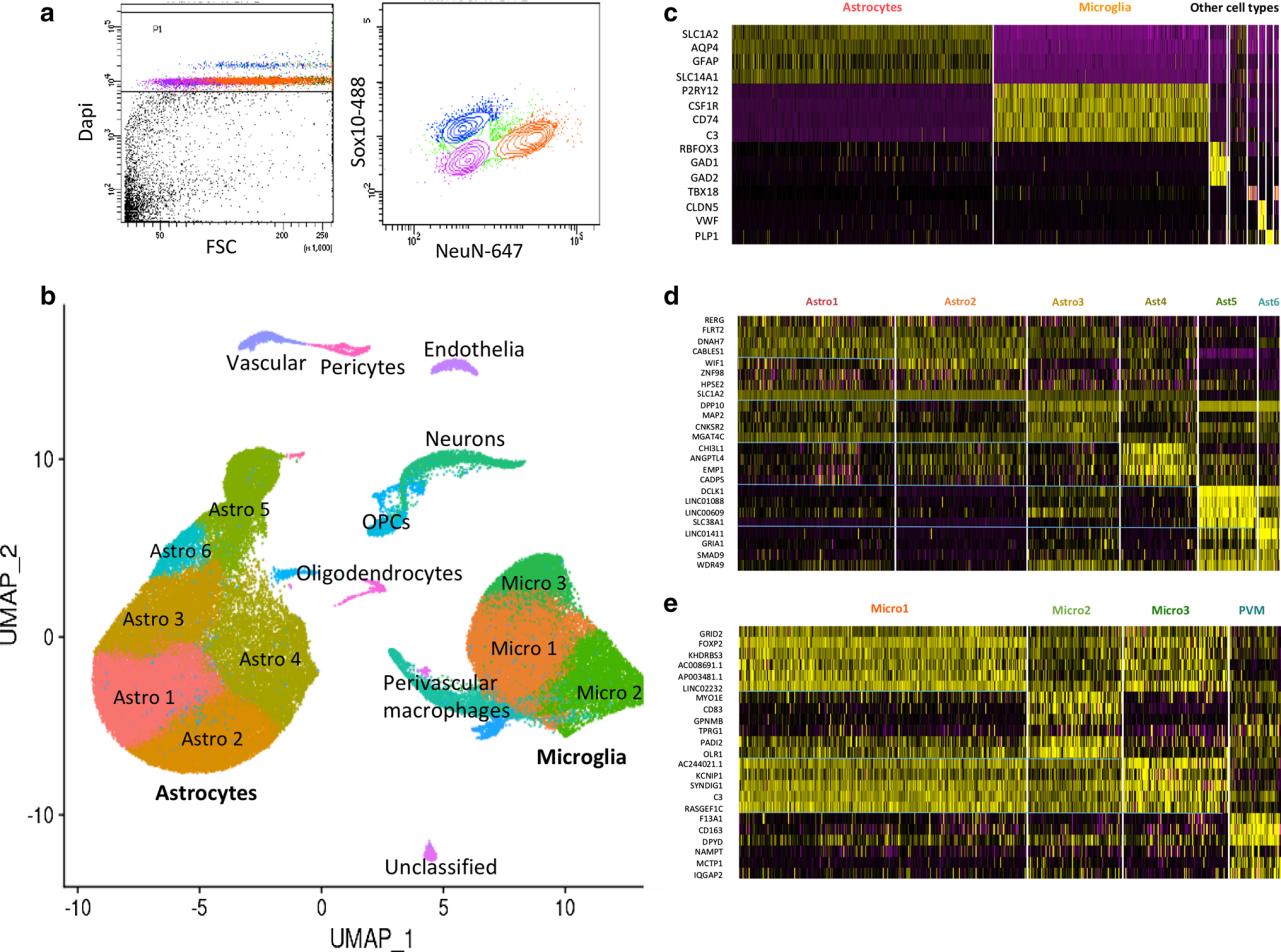
Analysis of human brain microglia and astrocytes from low and high AD pathology brains by single-nuclei RNASeq. **a** FACS gating method for sorting of human brain nuclei to enrich for microglia and astrocyte double-negative population (pink; mean for 24 samples = 17.9%, standard deviation = 6.2%). Dapi + ve nuclei were selected first, followed by separation based on NeuN and Sox10 staining. **b** UMAP 2D visualisation of clustering of 91,655 single nuclei from the 24 brain samples (average of 3819 nuclei per sample) including 52,706 (58%) astrocytes and 27,592 (30%) microglia. Smaller numbers of nuclei from neurons, oligodendrocytes, and vascular cells also were found (Extended Data Fig. 2, online resource), but these cell types formed distinct UMAP clusters that were not analysed further. **c** Heatmap showing cell type-specific marker gene expression in the nuclei clusters. **d** Heatmap of top differentially expressed genes in each microglial cluster. **e** Heatmap of top differentially expressed genes in each astrocyte cluster

Cell-type identification of clusters was performed using AUCell (see below) with cell-type specific genes identified by previous human brain snRNASeq studies [51]. Cell-type annotation was confirmed by visual inspection of key marker genes (Fig. 1 and Extended Data Fig. 2, online resource). Glial cluster-specific genes were identified using the *FindMarkers* function. All clusters were composed of nuclei from all samples and did not represent a single case or disease group. A small number of nuclei that either did not express any major cell-type markers, or expressed a combination of cell-type markers, were categorised as ‘unclassified’. We used the thresholding method described above for identification and removal of unclassified clusters.

### Integration and cell-type specific clustering of the unenriched samples

Gene-cell matrices for the samples without enrichment were processed using similar qc parameters to the samples with enrichment. Nuclei with less than 200 genes were excluded. Any nuclei with more than 5% mitochondrial counts were removed. Mitochondrial genes were removed from the final gene-cell count matrices. Integration was performed using LIGER (Linked Inference of Genomic Experimental Relationships) with *k* = 20 and *λ* = 5. The LIGER factors were then used for uniform manifold approximation and projection (UMAP) and a Leiden algorithm was applied to detect the clusters using the UMAP embeddings as the input. Clusters were annotated for cell types using EWCE (weighted cell type enrichment) algorithm against reference datasets previously generated with EWCE. Astrocyte and microglial clusters were then separated from the rest of the cell population to compare to that of enriched clusters.

### Comparison of astrocyte and microglial nuclei gene expression before and after enrichment

The top 10% most variable genes were calculated separately from astrocytes and microglia clusters from the unenriched and enriched samples prepared independently from the same regions of the same brains. The normalised counts of these genes were first pseudobulked by sample in both datasets. A Pearson correlation was then calculated between the gene counts in samples from the unenriched and enriched sets.

### Differential gene expression analysis

MAST was used to identify genes differentially expressed (associated) with histopathological features (using pTau or amyloid-beta as markers) [14, 59] to perform zero-inflated regression analysis by fitting a mixed model. The model specification was zlm(~ histopath_marker + (1|sample) + cngeneson + pc_mito + sex + brain_region, sca, method = "glmer", ebayes = F). The fixed effect term pc_mito accounts for the percentage of counts mapping to mitochondrial genes. The term cngeneson is the cellular detection rate. Each nuclei preparation was considered as a distinct sample for the mixed effect. Models were fit with and without the dependent variable and compared using a likelihood ratio test. Units for differential expression are defined as log_2_ fold difference/% pTau-positive cells (or log_2_ fold difference/% amyloid area), i.e., a one unit change in immunohistochemically defined pTau (or amyloid density) is associated with one log_2_ fold change in gene expression. Genes expressed in at least 10% of nuclei from each cell type (either total microglia or total astrocytes) were tested. Genes with a log_2_ fold change of at least 0.25 and adjusted *p* value < 0.05 were defined as meaningfully differentially expressed. As an additional filter, the percentage of the inter-individual variance in expression between the NDC subjects was calculated for each gene and three genes with unusually high (> 2 standard deviations) variance (*LINGO1*, *SLC26A3* and *RASGEF1B*) in one or two samples were excluded.

### Gene ontology and pathway enrichment analysis

The gene ontology (GO) enrichment and the pathway enrichments analysis were carried out using the R package enrichR (v 3.0), which uses Fisher's exact test (Benjamini–Hochberg FDR < 0.1) [5, 9]. Gene sets with minimum and maximum genes of 10 and 500, respectively, were considered. To improve biological interpretation of functionally related gene ontology and pathway terms and to reduce the number of redundant gene sets, we first calculated a pairwise distance matrix using Cohen’s kappa statistics based on the overlapping genes between the enriched terms and then performed hierarchical clustering of the enriched terms [22]. Pathways relating specifically to cancer pathology were excluded.

### Gene set enrichment analyses (GSEA)

AUCell [1] (R package v1.6.1) was used to quantify the expression of published gene set signatures (Supplementary Table 2, online resource). Mouse genes were converted to human orthologues where applicable using bioDBnet. Normalised data were processed in AUCell using the *AUCell_buildRankings* function. The resulting rankings, along with the gene lists of interest, were then put into the function *AUCell_calcAUC* (aucMaxRank set to 1% of the number of input genes). Resulting AUC scores were scaled across clusters.

We also used AUCell to test for enrichment of the gene sets that we identified on previously published human single-nuclei data (Supplementary Table 3, online resource) [16, 18, 36, 76]. Where possible for the AUCell tests for enrichment of gene sets from previously reported data, the cell type annotations from the published data were used. Filtered matrices were processed and cell types identified using the methods described above for the Zhou et al*.*’s [76] data set. The scFlow single-cell analysis pipeline [28] was employed for analysis of the Gerrits et al*.* data set. In brief, quality control was performed using the same criteria as described above. Sample integration was performed using Liger [72] and dimension reduction using PCA and UMAP, and finally, clustering was performed using the Leiden algorithm [65]. Microglia and astrocytes were then identified using the same sets of marker genes used for the primary analysis (Fig. 1). AUCell was then run separately on microglia and astrocyte populations from each study using lists of our significantly up-regulated and down-regulated genes with pTau and amyloid pathology, from microglia and astrocytes (thresholded as described above). The aucMaxRank term was set to 200 genes. LogFC values between Control and AD samples were estimated using the limma package in R [45], using the default configuration and the following linear model: ~ diagnosis + nFeature, where nFeature is the total number of distinct features expressed in each nucleus (to account for the fact that nuclei that express a higher number of features may have higher AUCell scores).

Over-representation analyses of literature gene sets in our glial sub-clusters and differentially expressed gene lists were performed by Fisher's exact test using the "enrichment" function of the R package "bc3net" (https://github.com/cran/bc3net). The p values associated with the Fisher's exact test correspond to the probability that the overlap between the literature gene sets and the sub-cluster markers/differentially regressed genes from our dataset has occurred by chance.

### Cell–cell communication, gene co-expression (module), regulatory network (regulon), and enrichment analyses

Cell–cell communication analysis was performed using CellChat [26]. CellChat employs a curated database of potential signalling ligand–receptor pairs from the literature. Amongst all these potential ligand–receptor pairs, cell–cell interactions are identified based on mass action models, along with differential expression analysis and statistical tests on cell groups. The CellChat algorithm with default parameters (unless otherwise specified) was applied to the sub-set of the dataset that corresponded to microglia and astrocytes, separately on the AD and NDC samples. The human CellChat database was used for the ligand–receptor pairs. Communications that involved less than 100 nuclei were filtered out (using the min.cells argument in the filterCommunication function). The results of the cell–cell communication analysis were integrated with the results of the differential gene expression analysis to prioritise interactions that are most likely altered with advancing pathology. The ligand–receptor interactions where at least one of the interacting partners corresponds to genes that are significantly associated with amyloid-beta or pTau are thus highlighted in Supplementary Table 10.

Gene co-expression module and hub-gene identification analysis were performed separately for microglial and astrocyte populations using the MEGENA (v1.3.7) package [58]. The top 15% most variably expressed genes were used as input [2]. We evaluated the mean expression of each of these genes across all the nuclei in the expression matrices: all the genes in both astrocyte and microglia matrices were amongst the top 25% most highly expressed genes, confirming that the choice to filter the expression matrix based on variability did not bias the inclusion towards genes with a particularly low level of expression. In addition, we verified that the filtered expression matrices for both astrocytes and microglia included a substantial proportion of the differentially expressed genes: over 90% for astrocytes and all but two of the microglial differentially expressed genes were included in the respective filtered expression matrices, suggesting that the filtered expression matrices contained a biologically meaningful gene set. The MEGENA pipeline then was applied using default parameters, using Pearson’s correlations and a minimum module size of 10 genes. Parent modules were produced from which a sub-set of genes form smaller child modules (Supplementary Tables 4 and 5, online resource). For downstream analysis, interpretation, and presentation of results, modules with > 20 genes were retained. Co-expression modules were represented graphically using Cytoscape software (Mac OS version 3.8.0) [53] with hub genes represented with a triangle and nodes with a circle with a diameter proportional to the node degree [58]. Genes previously associated with AD as defined by Kunkle et al*.* (Table 1 [31], 23 genes), Jansen et al*.* (Table 1 [25], a further 17 genes) and Andrews et al*.* (Table 1 [3], a further 25 genes), for a total of 65 genes, were annotated in the co-expression networks.

Gene regulatory networks were built using pySCENIC (0.10.3) [39, 67] package default parameters. The inputs for the pySCENIC gene regulatory network analyses were the same filtered expression matrices as for the MEGENA gene co-expression module analyses. Correlations between a list of 1390 human transcription factors (TFs) curated by Lambert et al. [33] and the genes in the expression matrix were evaluated and co-expression modules with a minimum size of 20 genes were defined. From these, regulons (gene modules sharing a common association with a TF) were built after removing the genes without a recognition motif (based on the hg19-500 bp-upstream-7species.mc9nr and hg19-tss-centred-10 kb-7species.mc9nr databases provided in the pySCENIC package) for the correlated TF. Only regulons with activator-TFs were retained [67]. Regulons including 50 or more genes were retained for downstream analyses and Cytoscape software (Mac OS version 3.8.0) [53] was used for their graphical representation.

To evaluate module and regulon enrichment with AD pathology, the AUCell scores for each gene co-expression module or regulon in each nucleus were calculated (aucMaxRank set to 5% of the number of input genes). The statistical analysis was performed using the limma package in R [45], using the default configuration and the following linear model: ~ pathology + nFeature + pc_mito, where pathology is the average immunohistochemistry quantification value for Aβ or pTau, nFeatures is the total number of distinct features expressed in each nucleus (to account for the fact that nuclei that express a higher number of features may have higher AUCell scores), and pc_mito is the percentage of counts mapping to mitochondrial genes. We also corrected for a potential pseudoreplication bias [77], using the duplicateCorrelation function of the limma package with the sample as the “blocking” variable. To assess if the module and regulon enrichment with AD pathology differed with respect to the brain region (EC and SSC), we repeated the limma analysis separately in each brain region and compared the results by means of a linear regression analysis.

Sub-cluster-specificity of modules and regulons were estimated using the regulon_specificity_scores function in pySCENIC [63]. Briefly, the module/regulon specificity score employs the Jensen–Shannon divergence, a metric previously used to assess cell-type specificity of transcripts [6] and regulons [63]. Modules and regulons with the highest specificity score may be considered sub-cluster-specific. A specificity score of 1 indicates a gene set that is only expressed in one sub-cluster, while a specificity score of 0 indicates an evenly expressed gene set across all sub-clusters.

## Results

### Selective astrocyte and microglia transcriptome sequencing

The proportions of microglia and astrocytes defined by snRNASeq of nuclei isolated from the human brain *post-mortem* are low and variable [18, 36]. To enable comprehensive analyses of differential transcript expression in astrocytes and microglia with AD pathology, we enriched for these glia by selectively removing neuronal (NeuN-positive [32]) and oligodendrocyte (Sox10-positive [73]) nuclei using FACS (Fig. 1 and Extended Data Fig. 2, online resource). For this, we isolated nuclei from each of two cortical regions [entorhinal (EC) and somatosensory (SSC) cortex] taken from six brains with low levels of AD neuropathology provided by donors without reported cognitive impairment and from six brains with high levels of AD pathology (Extended Data Fig. 1). Nuclei were well mixed with respect to disease (AD vs NDC), brain region (EC vs SSC), and donor sex after integration (Extended Data Fig. 3a–d, online resource).

We used nuclear markers that are highly specific for oligodendroglia and neurons to separate these nuclei from those isolated to enrich for astrocytes and microglia (Extended Data Fig. 4a, online resource). We also confirmed that the transcriptomes expressed in samples enriched from each sample were not biased through the enrichment process by showing that the top 10% most variable genes expressed in nuclei without and with enrichment obtained from paired sections from each subject and brain region were correlated. For astrocyte nuclei, correlations between variable genes from the same individual ranged between 0.99 and 1.00, with similarly strong correlations between the enriched and unenriched microglial nuclei (0.89–1.00) (Extended Data Fig. 4b, c, online resource). Individual proportions of nuclei clustered as astrocytes and microglia varied between subjects, but the same proportions of nuclei from the two cell types were isolated from male and female donors (Extended Data Fig. 3e, f, online resource). There was a small trend for a relative astrogliosis with AD (Extended Data Fig. 3g, online resource).

Astrocyte nuclei had a mean unique molecular identifier (UMI) count of 8775 with an average of 3166 distinct genes and microglial nuclei had a mean UMI count of 4808 with an average of 2132 genes. Amongst the 52,706 astrocyte nuclei, we found expression of 90% of astrocyte transcripts previously reported from human brain astrocytes (500 genes) [75]. 16/65 AD risk genes were represented in the astrocyte co-expression network (Extended Data Fig. 5a, online resource). The 27,592 total microglial nuclei included expression of 96% of the recently described microglial “core” consensus transcriptome (249 genes) [44]. Microglia also were highly enriched in genes associated previously with genetic risk for AD (27/65) [3, 25, 31] (Extended Data Fig. 6a, online resource).

### Increased expression of genes related to metal ion homeostasis, proteostasis, and inflammation in astrocytes with AD pathology

**Fig. 2:**
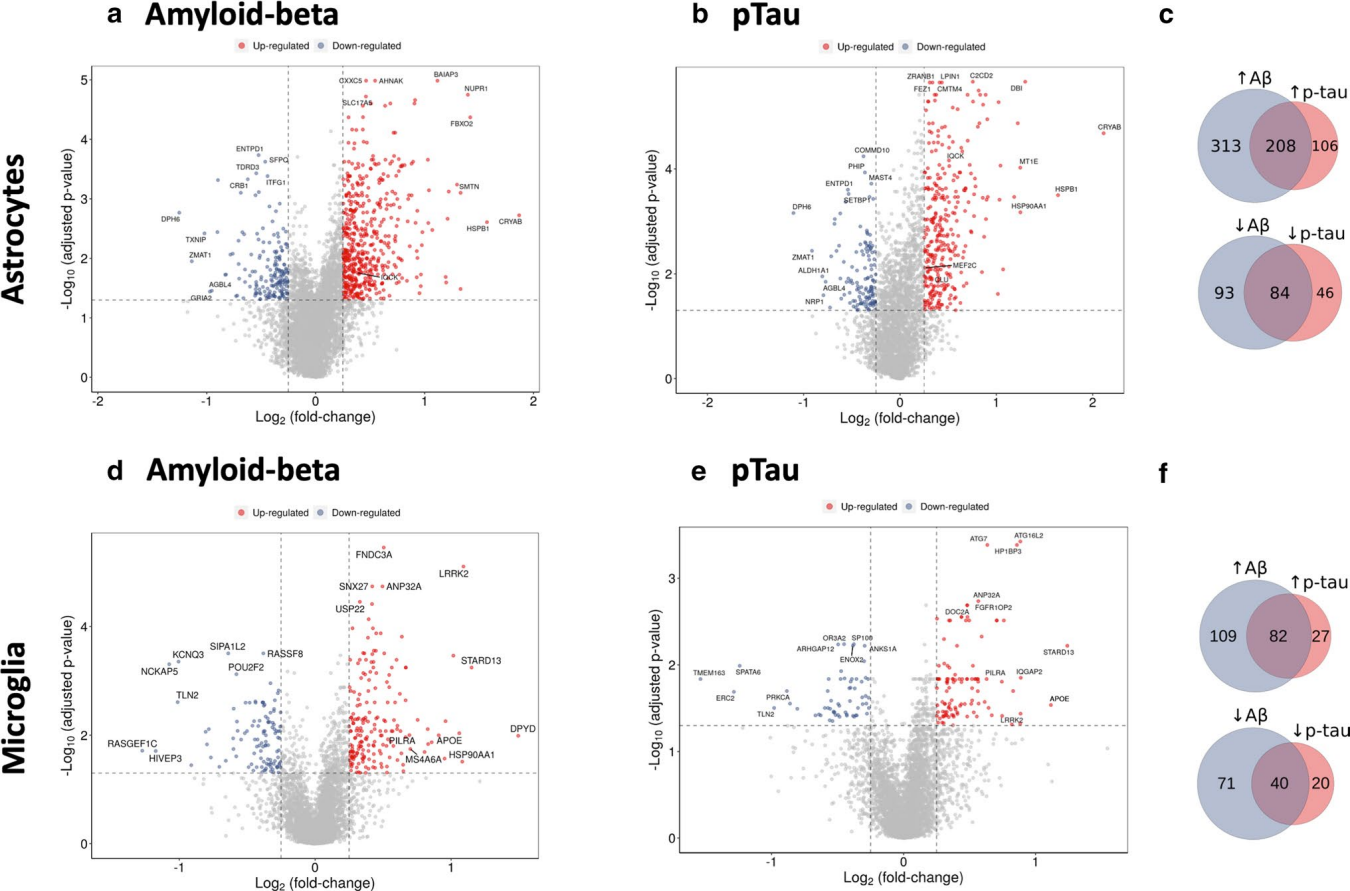
Differential gene expression in astrocytes and microglia with amyloid-beta and pTau pathology. Volcano plot of transcripts differentially expressed in astrocyte nuclei (threshold of ≤ 0.05 adjusted *p* value, abs logFC ≥ 0.25, omitting the top three most variable genes between samples) with immunohistochemically defined tissue amyloid-beta (**a**) and pTau (**b**) density. **c** Venn diagram illustrating the number of genes positively correlated (top) and negatively correlated (bottom) with amyloid-beta (blue) and pTau pathology (pink) in astrocytes. Volcano plot of transcripts differentially expressed in microglial nuclei (threshold of ≤ 0.05 adjusted *p* value, abs logFC ≥ 0.25, omitting the top three most variable genes between samples) with immunohistochemically defined tissue amyloid-beta (**d**) and pTau (**e**) density. **f** Venn diagram illustrating the number of genes positively correlated (top) and negatively correlated (bottom) with amyloid-beta (blue) and pTau (pink) pathology in microglia

**Fig. 3:**
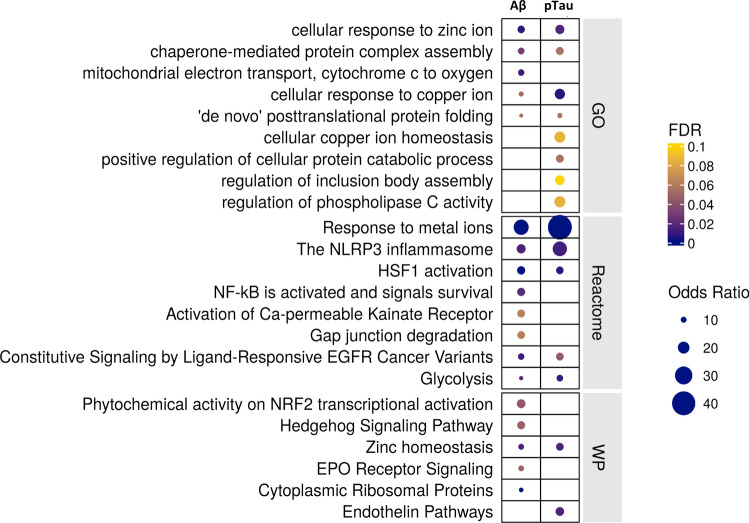
Functional enrichment of differential gene expression with amyloid-beta (left) and pTau (right) pathology in astrocytes. The plots describe the significant functionally enriched pathways in astrocytes obtained using enrichR (see “Methods”) from Gene Ontology (GO), Reactome, and Wikipathways (WP) databases

Gene expression associated with extracellular amyloid plaques or intraneuronal neurofibrillary tangles (pTau) (Fig. 2 and Supplementary Tables 6 and 7, online resource) were discovered by regressing gene expression against amyloid-beta (expressed as log_2_ fold difference/% area stained) or pTau (expressed as log_2_ fold difference/% pTau-positive cells) densities in sections prepared from homologous regions of the contralateral hemispheres for each of the brains. Half of the significantly positively associated genes expressed were correlated with both amyloid-beta and pTau pathology, but almost threefold more transcripts were associated uniquely with amyloid-beta (313 genes) expression relative to pTau (106 genes) (Fig. 2). We found significant astroglial functional enrichment for pathways involved in the ‘cellular response to zinc ion’, ‘cellular response to copper ion’, and ‘response to metal ions’ with both amyloid-beta and pTau expression (Fig. 3 and Supplementary Table 8, online resource); genes encoding proteins involved in metal ion homeostasis (*MT1G, MT1F*, *MT1E*, *MT2A*, *MT3,* and *FTL*) were amongst the top transcripts most highly *positively* associated with pathology in astrocytes (Supplementary Table 6, online resource). Transcripts involved in ‘chaperone-mediated protein complex assembly’ and ‘response to unfolded protein’ pathways, such as *CRYAB*, *HSPB1*, *HSPH1,* and *HSP90AA1,* also were positively differentially expressed. Increased expression of the AD risk gene *CLU* was associated with pTau pathology in astrocytes (Extended Data Fig. 7a, online resource). Expression of the AD risk gene *IQCK* was positively associated with both amyloid-beta and pTau (Extended Data Fig. 7a, online resource). Pathways involved in inflammatory processes also were significantly enriched (‘NLRP3 inflammasome’ and ‘NFkB is activated and signals survival’). By contrast, “core” or homeostatic astrocyte transcripts, such as those for glutamate transporters *SLC1A3* and *SLC1A2* or for *IL-33* (CSF1R ligand, which promotes microglial synaptic remodelling [69]), were down-regulated. The AD risk-associated *MEF2C* transcription factor, as well as *MAFG*, *JUND*, *CEBPB*, *MAF*, and *LHX2* were up-regulated, suggesting roles for these transcription factors in the regulation of responses to AD pathology.

We confirmed with gene set enrichment (AUCell) that transcripts positively differentially expressed with pathology also were significantly enriched in nuclei with human AD pathology reported in previously published snRNASeq studies [16, 18, 36, 76], albeit with very low log fold changes in one [16] out of the four datasets analysed (Supplementary Table 3, online resource).

### Expression of genes related to autophagy, phagocytosis, and proteostasis in microglia with AD pathology

**Fig. 4:**
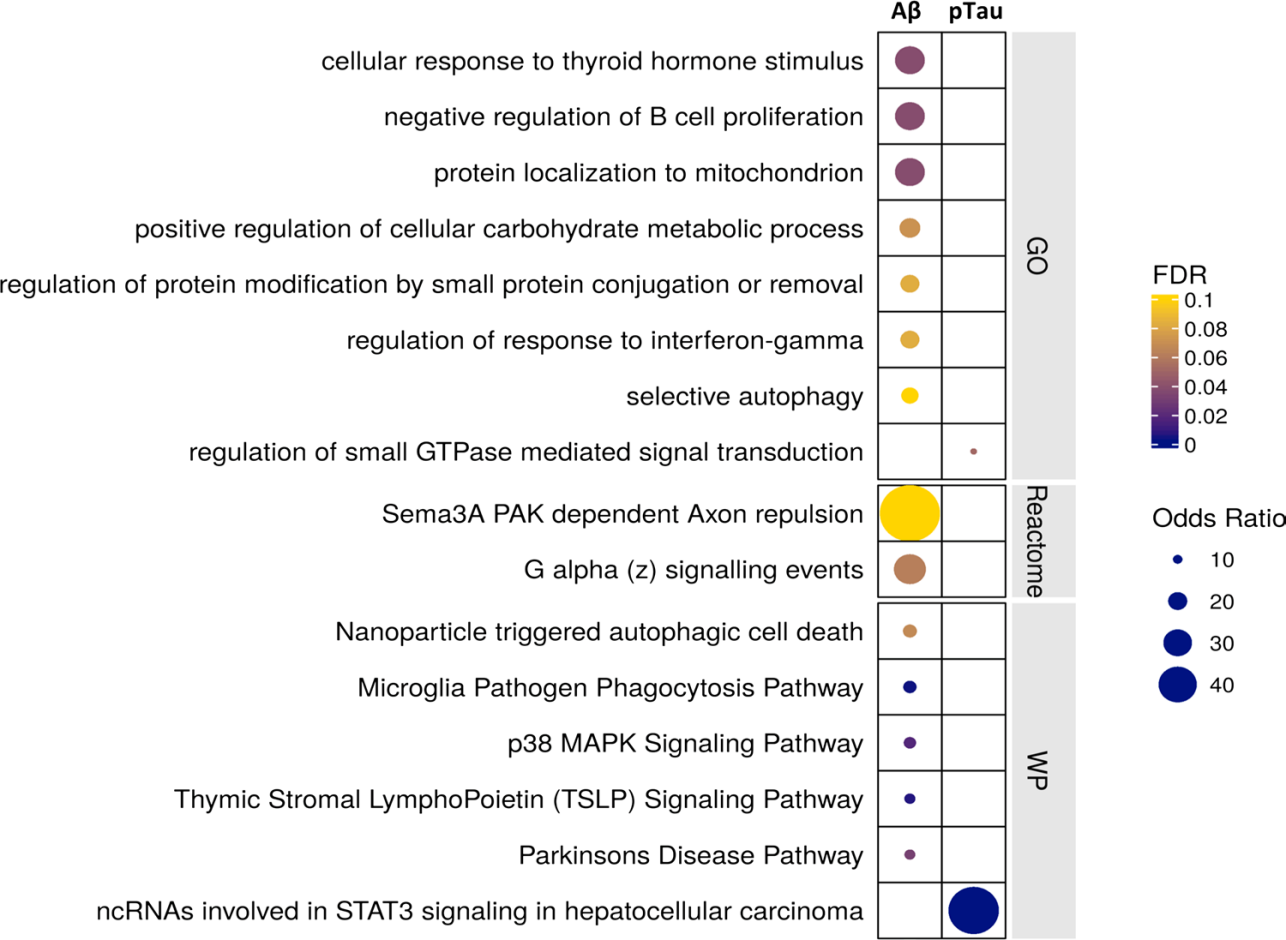
Functional enrichment of differential gene expression with amyloid-beta (left) and pTau (right) pathology in microglia. The plots describe the significant functionally enriched pathways in microglia obtained using enrichR (see “Methods”) from Gene Ontology (GO), Reactome, and Wikipathways (WP) databases

Microglial transcripts most highly *positively* associated with tissue amyloid-beta and tissue pTau density included those for genes associated with AD risk (*APOE*, *MS4A6A* and *PILRA*, Fig. 2 and Extended Data Fig. 7c,d, online resource), as well as those for genes associated with risks for other neurodegenerative disorders (*LRRK2, SNCA* and *GPNMB*, associated with Parkinson’s disease [8], and *GRN*, associated with ceroid lipofuscinosis [71] and frontotemporal dementia [4]). Fourfold more transcripts were associated uniquely with amyloid-beta (109 genes) expression relative to pTau (27 genes), while 60% of the significantly positively associated genes expressed were correlated with both amyloid-beta and pTau pathology (Fig. 2). Differentially expressed transcripts were functionally enriched in ‘selective autophagy’ and ‘microglia pathogen phagocytosis’ pathways (Fig. 4 and Supplementary Table 9, online resource); *ASAH1*, *ATG7*, *STARD13*, and *MYO1E* were amongst the most strongly positively associated genes (Supplementary Table 7, online resource). Perivascular macrophages (PVM) also showed functional enrichment in ‘selective autophagy’, as well as several CCT/TriC molecular chaperone complex pathways involved in proteostasis and actin/tubulin folding (Extended Data Fig. 8, online resource and Supplementary Table 9, online resource). Up-regulation of the transcription factors *MAFG*, *MITF*, and *JUND* with amyloid-beta or pTau pathology suggests their involvement in transcriptional regulation of these microglial and PVM responses to pathology.

We confirmed that transcripts positively differentially expressed in microglia were significantly enriched in nuclei with human AD pathology in previously published snRNASeq studies [16, 18, 36, 76] (Supplementary Table 3, online resource). Toll-like receptors (TLR2 and TLR10), HK2 (hexokinase 2), JAK2 (Janus kinase 2), and ITGAM (CD11b) were amongst the smaller number of transcripts in these cells that were significantly *negatively* associated with tissue amyloid-beta or pTau.

We found that astrocytes and microglia display differences in the genes whose expression is specifically differentially associated with amyloid-beta and pTau pathology. Astrocytes show greater expression of mitochondrial oxidative phosphorylation and potential neuroprotective Nrf2 activation pathways associated specifically with amyloid-beta, while cell junctional and catabolic pathway up-regulation is associated specifically with pTau pathology, with shared up-regulation of pro-inflammatory NLRP3 inflammasome and metal ion response pathways (Extended Data Fig. 9a, online resource). By contrast, microglia show increased expression of carbohydrate metabolic processes, responses to unfolded protein, the MAPK cascade, and the TYROBP causal network are amongst pathways showing greater expression with amyloid-beta, with a sparser response to IL1-related pathway up-regulation associated with pTau (Extended Data Fig. 9b, online resource).

### Exploration of ligand–receptor interactions between astrocytes and microglia

We applied CellChat [26] in conjunction with the jointly generated astrocyte and microglial transcriptomes to explore correlations between inferred ligand–receptor interactions with greater amyloid-beta or tau pathology (Supplementary Table 10, online resource). Both amyloid-beta and pTau pathology were associated with increased expression of genes implicated in integrin interactions with laminin and fibrillin, the APP (astrocyte)–CD74 (microglia) ligand pair, which can suppress amyloid-beta production [37], and inferred CD99 (astrocyte)–PILRA (microglia) interactions that may inhibit inflammatory responses [62]. By contrast, adenosine signalling via microglial ENTPD1 nucleosidase products interacting with astrocytic ADORA2B was less prominent with advancing pathology (the *ENTPD1* gene was negatively associated with amyloid-beta and pTau pathology). Independent of this, amyloid-beta pathology alone was associated with higher probabilities of CXCL12 (microglial)–CXCR7 (astrocytic) interactions implicated in neurogenesis and learning [38, 66], BMP4 (microglial)–BMP1RA (astrocyte) ligand–receptor pairing [74], and neuroprotective *Wnt* signalling with expression of WNT by microglia and its receptors on astrocytes [24]. This interaction was only present in AD samples; it was non-significant in NDC. Microglial integrin interactions with the astrocytic glycoprotein GP1B and ICAM1 became less likely, as the relative expression of integrin genes was found to be negatively associated with amyloid-beta. Finally, greater tissue pTau density was singularly associated with a higher probability of both increased C3 and C4A complement interactions with C3AR1 on microglia; interaction was only identified in AD samples (it was non-significant in NDC).

### Gene co-expression modules suggest glial cell-specific functional roles of AD GWAS genes

#### The EC and SSC show similar co-expression signatures

Co-expression network analyses were used to characterise gene expression modules (MEGENA) in astrocytes and microglia, suggesting potential functional relationships. We found consistent gene co-expression signatures for the EC and SSC; strong correlations were found between MEGENA modules differentially expressed with greater amyloid-beta or pTau in nuclei in the two regions (for astrocytes, *r* = 0.96 with pTau and *r* = 0.86 for amyloid-beta; for microglia, *r* = 0.86 with pTau and *r* = 0.87 for amyloid-beta) (Supplementary Data Table 11, online resource). The patterns of regulon expression related to increasing pTau or amyloid-beta inferred using SCENIC for nuclei from EC and SSC also were similar; strong correlations were found between the differential activity (logFC) inferred with SCENIC for nuclei from the EC and SSC (for astrocytes, *r* = 0.89 with pTau and *r* = 0.76 for amyloid-beta; for microglia, *r* = 0.85 with pTau and *r* = 0.71 for amyloid-beta) (Supplementary Data Table 11, online resource). This suggests that similar cell responses are associated with amyloid-beta and pTau pathological features in the two brain regions.

#### Evidence for involvement of CLU in astrocyte metal ion homeostasis and proteostasis pathways with AD

AD GWAS genes *CLU* and *IQCK* were co-expressed in an astrocyte module (module 9; Supplementary Table 4, online resource) which was amongst the most strongly positively correlated with both amyloid-beta and pTau density. Both *CLU* and *GJA1* (Gap Junction Protein Alpha 1; Connexin-43) are hub genes in this module, which was functionally enriched in transcripts for proteins involved in metal ion homeostasis (e.g., ‘metallothioneins bind metals’ and ‘response to metal ions’) and proteostasis (‘HSF1 activation’, ‘response to unfolded protein’, and ‘chaperone-mediated protein complex assembly’) (Supplementary Table 4, online resource). They also were hub genes in the related child module 30, which includes genes in pathways for ‘ceramide transport’ and ‘gap junction assembly’ (Extended Data Fig. 5b, online resource). *CLU* was associated with the astrocyte regulons identified using SCENIC (for transcription factors MAF, MAFG, JUND, and CEBPB) that had the strongest correlations with pTau and amyloid-beta (Supplementary Table 12, online resource). *CLU*-containing modules and the associated regulons also were enriched in AD nuclei reported in earlier studies (Supplementary Tables 4 and 12, online resource).

#### Evidence for a cell-specific role for APOE in microglia linking phagocytic, complement, and inflammatory activation pathways in AD

*APOE*, the largest genetic risk factor for AD, was up-regulated in microglia with both pTau and amyloid-beta pathology. *APOE* was a hub gene in microglia co-expressed both with *TREM2* and inflammatory activation and response genes (e.g., *C1QB, C1QC*, *CD74*, *CTSB*) in a module functionally enriched for pathways including the ‘endosomal/vacuolar pathway’, ‘microglia pathogen phagocytosis’, and ‘antigen processing—cross presentation’ (module 19) (Extended Data Fig. 6b, online resource and Supplementary Table 5, online resource). Regulons inferred to be responsible for microglial *APOE* expression included those for transcription factors MXD4, MITF, PBX3, and JUND (Supplementary Table 14, online resource). *APOE* expression in astrocytes was not significantly correlated with either amyloid-beta or pTau pathology and co-expression relationships suggested a different functional role for *APOE* in astrocytes as a hub gene in a module functionally enriched for ‘dermatan sulphate biosynthesis’, ‘extracellular matrix organisation’, and ‘ferroptosis’ (module 13) (Supplementary Table 4, online resource).

#### Microglial and PVM GPNMB are up-regulated with AD pathology in modules related to lipid homeostasis

**Fig. 5:**
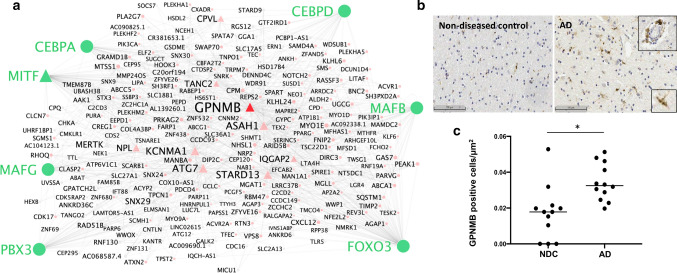
*GPNMB* is a hub gene in microglial gene co-expression modules up-regulated with AD pathology. **a** Graph of microglial gene co-expression module 11 enriched with amyloid-beta and pTau pathology for which *GPNMB* is a hub gene (triangles = module hub genes). *GPNMB* is expressed in regulons identified by their transcription factors (in green). **b** Immunohistochemical staining of GPNMB in post-mortem human brain tissue from representative NDC and AD brains with nuclear counterstain. Staining is present in both microglia and perivascular macrophages (insets). Scale = 100 μm. **c** Quantification of density of GPNMB-positive cells in cortical tissue by automated image analysis showing an increase in GPNMB-positive cells per μm^2^ for AD cases compared to NDC (*p* = 0.0023; Mann–Whitney test, two-tailed; *N* = 12). Each point represents a single sample and the horizontal bar indicates the median

Glycoprotein nonmetastatic melanoma protein B (GPNMB) is elevated in plasma and CSF with AD and has been proposed as a biomarker of disease [23]. We found that *GPNMB* is up-regulated in microglia with amyloid-beta and pTau pathology and in PVMs with pTau pathology (Supplementary Table 7, online resource). Consistent with this, *GPNMB* was found in association with the MAFG, JUND, MAFB, CEBPD, and CEBPA transcription factor regulons which showed strong correlations to amyloid-beta and pTau in microglia (Fig. 5a and Supplementary Table 14, online resource). *GPNMB* also is a hub gene in microglial co-expression modules 11 and 34, which are strongly associated with amyloid-beta and pTau expression (Supplementary Table 5, online resource). As well as *GPNMB*, hub genes for module 11 also include *ASAH1, ATG7, STARD13, IQGAP2, CPVL, TANC2,* and *MITF*, all of which were positively differentially expressed with one or both of the AD pathologies (Fig. 5a). We found that module 11 was enriched in AD (relative to control) samples from three out of four previous human snRNASeq studies analysed [16, 18, 76] (Supplementary Table 5, online resource). Pathways involving the differentially expressed genes in module 11 suggest a functional role in cholesterol homeostasis (‘regulation of cholesterol storage’). We found that the smaller module 34 was enriched in AD samples from two out of four previous studies [16, 36]. Functional pathways enriched in the *GPNMB* hub-gene module 34 relate to phospholipid and lipoprotein homeostasis (‘phospholipid efflux’, ‘phospholipid homeostasis’, and ‘lipoprotein metabolism’).

Consistent with the expected GPNMB expression in PVM, immunohistochemistry for GPNMB showed prominent staining around blood vessels, as well as in a sub-set of parenchymal microglia (Fig. 5b). There was a mean ~ 1.8-fold increase in GPNMB-positive cells/μm^2^ in AD (relative to NDC) in the cortical tissue studied here (Fig. 5c). GPNMB staining density in sections of samples was positively correlated both with tissue pTau (*R* = 0.39) and with expression of modules 11 and 34 (*R* = 0.507 and 0.576, respectively) defined by snRNASeq of the same region in the contralateral hemisphere.

### Transcriptional heterogeneity associated with AD pathology in sub-sets of human astrocytes and microglia

Our snRNASeq data reduction identified six distinguishable clusters that expressed the core set of astrocyte genes. Each also expressed distinct sets of genes that suggested sub-types of astrocytes (Fig. 1 and Supplementary Table 13, online resource). Astro1 and Astro2 expressed higher levels of genes involved in core astrocyte functions, such as *SLC1A2* (*GLT1*) and glutamine synthetase (*GLUL*) (Supplementary Table 13, online resource) and Astro2 was enriched for pathways including ‘neurotransmitter uptake’, ‘glutamatergic synapses’, and ‘amino acid import’. In contrast, Astro4 and Astro5 were characterised by relative expression of genes involved in extracellular matrix formation and functions [40] and were enriched for pathways including ‘carbohydrate binding’ and ‘cell–matrix adhesion’. Astro4 was distinguished from Astro5 by relatively high expression of *VEGFA*, while Astro5 was enriched uniquely for immune response pathways, such as the Toll-like receptor cascade and the activated astrocyte marker *GFAP*. Transcripts in Astro6 were enriched for metallothionein genes. Astro5 and Astro6 showed the greatest specificity for regulons most highly up-regulated with pTau (for transcription factors JUND, MAF, and CEBPB) (Supplementary Table 12, online resource).

We found consistent trends for increases in the numbers of nuclei in Astro5 and (although less prominently for nuclei from the SSC) in Astro6 sub-sets with both increased local amyloid-beta and pTau pathology (Extended Data Fig. 10a, b, online resource). This was associated with a consistent trend to decreased Astro1 nuclear numbers with greater pTau expression and a similar trend in the EC with greater amyloid-beta expression.

The disease associated astrocyte (DAA) expression signature defined in a mouse model [19] was represented in all astrocyte clusters other than Astro3 (Supplementary Table 16, online resource). 4.6% of DAA genes were up-regulated with amyloid-beta and 9.2% with pTau. Although the A1 (12/15 genes expressed) or A2 (13/13 genes expressed) gene sets [35] associated previously with injury-responsive or homeostatic astrocytes in rodent models, respectively, were represented in the astrocyte clusters, neither A1 nor A2 gene sets were enriched significantly in the total astrocyte nuclei or in any of the clusters in agreement with several rodent and human studies that fail to replicate distinct ‘A1’ and ‘A2’ astrocyte populations [13].

Clustering parameters selected to distinguish PVM [identified by markers such as *CD163*, *MRC1* (*CD206*), and *MSR1* [29]] also identified three clusters of nuclei (designated Micro1, 2, and 3) expressing different sets of microglial marker genes (Fig. 1 and Supplementary Table 15, online resource). Micro1 was most highly enriched in transcripts for human ‘core’ [15] homeostatic genes, Micro2 showed a relative functional enrichment for the ‘TYROBP causal network’ and ‘ferroptosis’ pathways and Micro3 expressed lower levels of both homeostatic and activation genes, but had higher expression of *C3* and *LPAR6* (Supplementary Table 15, online resource). Micro2 showed the greatest specificity for the MAFG, CEBPA, JUND, CEBPD, and MITF regulons found here to be highly correlated with amyloid-beta and pTau (Supplementary Table 14, online resource). Plaque-induced (PIG [10]), disease associated (DAM [27]), activated response (ARM [48]), and interferon response microglial (IRM [47, 48]) gene sets identified in rodent amyloid models were expressed in all of the clusters, with relative enrichment of DAM, ARM, and, to a lesser degree, IRM gene sets in Micro2 (Extended Data Fig. 11, online resource and Supplementary Tables 2 and 16, online resource). PVMs were also relatively enriched in these gene sets related to microglial activation, as well as in a gene set associated with human microglial ageing [43]. Trends towards increased numbers of nuclei in the Micro2 and PVM sub-clusters were found with both greater amyloid-beta and pTau expression (Extended Data Fig. 10c, d, online resource).

## Discussion

Our results describe neuroprotective gene up-regulation for proteostasis, phagocytosis, and protein clearance with amyloid-beta and pTau pathology in both astrocytes and microglia, extending similar observations reported recently [34]. While 50–60% of the significantly up-regulated genes were associated with both pathological proteins, a 3–4-fold greater number of genes were uniquely differentially expressed with amyloid-beta relative to pTau, mirroring observations with microglia in preclinical transgenic models overexpressing the two proteins [55]. Differentially expressed genes in astrocytes were associated with increased expression of genes for the regulation of metal ion homeostasis that may both protect cells from oxidative injury and facilitate redox-dependent chaperone clearance of abnormal proteins by astrocytes [46]. However, astrocytes also expressed inflammasomes and inflammatory activation pathways commonly found in microglia, both of which may promote neurodegeneration. Our data show that this transcriptional functional diversity reflects distinguishable sub-types of human astrocytes and microglia enriched in gene sets related to (but distinct from) those previously described in transgenic amyloid or tau mouse models or with ageing [19, 27, 43, 48]. Moreover, co-expression networks suggest cell-specific functional roles for genes associated with AD risk, most notably highlighting *APOE* as a hub gene in microglial co-expression modules linking gene expression subserving phagocytic, complement and inflammatory activation pathways.

We extended previous approaches to enrich our *post-mortem* brain snRNASeq data for microglia and astrocytes in our study. Several recent publications have described approaches for selective glial nuclei isolation from biopsy and rapid post-mortem delay tissue, including the use of antibodies to microglial transcription factor PU.1 [42], a combination of NeuN and Olig2 [17], and IRF8 [68]. Our negative-selection approach is distinguished by relying on nuclear markers that we have found to be robust to post-mortem delay, reliably at least to 24 h. Our analytical approach for addressing heterogeneity in the samples also differs from most prior studies (although it is not without similar precedents [7, 41]). Categorical descriptions of brains by Braak stage do not directly reflect tissue pathology in the local regions studied, so we associated each glial nuclear preparation with quantitative measures of pTau and amyloid-beta density specific to the regions studied in each of the brains. This has allowed us to characterise both similarities and differences in the astrocyte and microglia transcriptomic signatures with AD pathology. Even with use of a conservative, linear mixed regression model to account for uncontrolled sources of variance related to each sample [14], we found highly statistically significant associations between transcripts and pathological measures in both astrocytes and microglia (Supplementary Tables 6 and 7, online resource).

Our analyses relied on integration of transcriptomic data from the EC and SSC. The numbers of significantly differentially expressed genes in the regressions against pathology were different for the EC and SSC. However, this reflects multiple factors including variance in their expression in each region. The strong correlations observed between MEGENA modules and regulons by SCENIC suggests similar transcriptional responses to amyloid-beta and pTau pathology in the two brain regions, despite their differences in pathology load. They also support our approach with its integration of data across the two brain regions, although future work could explore any differences in transcriptional signatures that related to, e.g., differences in when the pathology first developed relative to the time of death.

Astrocytes expressed genes suggesting functional roles for proteostasis with amyloid-beta and pTau-associated functional enrichment for HSF1 activation and chaperone pathways. This was accompanied by expression of genes for pro-inflammatory NF-kB and inflammasome pathways. Greater regional amyloid-beta and pTau also was associated with enrichment of astrocytes for metal ion homeostasis pathways and the expression of metallothioneins. The latter proteins modulate neuroprotective superoxide dismutases through their regulation of Cu and Zn concentrations in extra- and intracellular compartments [20, 21, 54]. Identification of *CLU* as a hub gene in astrocyte co-expression modules including genes for metal ion homeostasis and proteostasis may reflect dependence of clusterin chaperone functions on the local redox environment [46]. Consistent with clinical studies providing evidence that amyloid potentiates clusterin expression, we found increased expression of the astrocyte *CLU* module with greater amyloid or pTau.

Co-expression analysis in microglia identified *APOE* as a hub gene for a module including complement genes (*C1QA, C1QB, and C1QC*) and functionally enriched in phagocytosis pathways. Microglia may contribute to astrocyte activation through C1q expression and C1q and other complement cascade proteins co-localise with amyloid plaques in AD [50]. These analyses also highlighted potential functional relationships of *GPNMB* (concentrations of the protein product of which are increased in brain samples and cerebrospinal fluid of sporadic AD patients [23]) with a diverse group of genes connected to transcription factors whose regulons were up-regulated with amyloid and pTau, suggesting that GPNMB is a biomarker for a human microglial neurodegenerative activation state central to pathological responses in AD.

Our results also highlight a diversity of astrocyte and microglial responses to AD pathology that, while related to sub-types previously defined in preclinical models, also had distinct features. We tested the relevance of microglial sub-cluster expression signatures defined in mouse transgenic models of AD using gene set enrichment analyses (Extended Data Fig. 11, online resource). We found evidence for similar, moderate levels of relative enrichment for inflammatory activation gene sets (DAM and ARM) in Micro2 and PVM [27, 48]. The relatively low expression of both homeostatic and activation genes in Micro3 corresponded to patterns associated with ‘dystrophic’, immuno-senescent microglia [60]. Transcripts from the disease associated astrocyte (DAA) gene set were up-regulated with amyloid-beta and pTau, but not restricted to any particular astrocyte sub-set, consistent with their involvement in a general transcriptional programme [19]. Overall, our results highlight distinct features and a greater functional diversity amongst microglia and astrocytes in the human disease relative to related preclinical models.

We recognise limitations of our data, as well as their considerable promise for further exploration. First, our data are limited to post-mortem tissue. However, although restricted to end-stage tissue, we attempted to maximise the dynamic range of pathology sampled by evaluating correlations with quantitative pathological measures across two anatomical regions and brains of different Braak stages. There are limitations to the sensitivity of the 3’-sequencing method used. We tried to compensate for the sparse sampling of the transcriptome by increasing the number of glial nuclei of interest and provided evidence for generalisability of our results by demonstration that major co-expression modules, regulons, and gene expression analyses discovered here also were represented in previous human *post-mortem* snRNASeq datasets [16, 18, 36, 76]. Even so, the nuclear transcriptome may be biased relative to that from a whole cell, potentially reducing the power to detect some genes reported from studies of related pathologies in mouse models [64]. The need to maximise detection sensitivity motivated us to enrich our sample for microglia and astrocytes for maximising the number of nuclei characterised and to make use of co-expression-based analyses, which rely less on detection of absolute expression levels than do single gene differential expression analyses.

With these caveats, we have extended previous work defining AD-associated molecular pathology of glial cells substantively by describing proteostasis, metal ion homeostasis, and inflammatory mechanisms in astrocytes and phagocytotic, proteostatic, and autophagic pathways in microglia. We found that gene sets described in transgenic mouse models are variably represented in AD, but in the context of more complex glial phenotypes. Our data also re-emphasise that there are functionally distinct sub-types of astrocytes and microglia, with particular diversity amongst the former, in which we distinguished enrichments for gene sets associated with synaptic function, extracellular matrix formation, immune responses, and control of metal ion homeostasis/redox state. The relative activation of PVM with pathology may make this cell type of particular interest given potential roles of PVM both in amyloid-beta clearance and neurovascular dysfunction. While this diversity suggests multiple potential targets for therapeutic modulation, the complexity of human astroglial and microglial phenotypes simultaneously expressed in AD also needs to be taken into account.

## Supplementary Information

Below is the link to the electronic supplementary material.

Supplementary file1 Extended Data Fig. 1: Measures of pathology in brains used for snRNASeq. a) Immunohistochemical (IHC) stains for pTau (AT8 antibody) of sections of representative formalin-fixed and paraffin embedded tissue from the entorhinal cortex in the contralateral hemisphere of typical AD (Braak stage III-VI) and NDC (Braak stage 0-II) cortical samples used in this study. Quantitative IHC measures of pTau in neurofibrillary tangles (% positive cells) (AT8 antibody, orange) and amyloid-beta plaques (% area stained) (4G8 antibody, blue) in somatosensory or entorhinal cortical sections (b) from NDC (low Braak 0-II) and c) AD (high Braak III-VI) samples. Each bar describes IHC results from a different cortical sample in either of the two regions from brains used in this study. The sample data for both (b) and (c) are ordered with respect to increasing pTau pathology load. Extended Data Fig. 2: Cell-type clusters identified by snRNASeq of NeuN and Sox10-depleted human brain nuclei. a. UMAP feature plots demonstrating astrocyte-specific (SLC1A2, GFAP, AQP4) and microglia-specific (CSF1R, C3, CD74) marker transcripts. b. UMAP feature plots for markers of cell types other than microglia and astrocytes in the glial-enriched samples: i. neurons, ii. oligodendrocytes, iii. endothelial cells, iv. oligodendroglial progenitor cells (OPC), v. pericytes, and vi. unclassified nuclei. Extended Data Fig. 3: Good integration of nuclei across all of the enriched samples is illustrated by the good mixing of nuclei from a. AD and NDC donor brains, b. entorhinal (EC) and somatosensory (SSC) cortical tissues, c. female (F) and male (M) donors and d. the 24 individual samples. Relative numbers of nuclei in the clusters (astrocytes, red; microglia, blue; PVM, green) from each sample (e). No significant differences were found between those from female (F) and male (M) donors. There was a small trend to a smaller proportion of microglial nuclei in the AD samples relative to NDC (g), although it was not statistically significant. Extended Data Fig. 4: To further validate the glial nuclear enrichment methods, we demonstrated the selectivity of gene expression for the neuronal (RBFOX3, the gene encoding NeuN) and the oligodendroglial (SOX10) markers in nuclei prepared from the cortical tissue and clustered for cell-type annotation (a). Pearson correlation matrices showed that the top 10% of most variable genes expressed in astrocyte (b) and microglial (c) clusters from nuclei prepared from the same tissues with and without FACS enrichments were highly correlated. Extended Data Fig. 5: AD GWAS genes represented in astrocyte gene co-expression modules. a. Astrocyte global gene co-expression network produced using MEGENA. Each grey point represents a co-expressed gene (Supplementary Table 4, online resource). AD GWAS genes are highlighted in red. b. Astrocyte co-expression module including AD GWAS genes CLU (hub gene) and IQCK (module 30), expression of which showed strong positive correlations to amyloid-beta and pTau pathology. Triangles indicate module hub genes. Extended Data Fig. 6: AD GWAS genes represented in microglial gene co-expression modules. a. Microglial global gene co-expression network produced using MEGENA. Each grey point represents a co-expressed gene (Supplementary Table 5, online resource). AD GWAS genes are highlighted in red. b. Microglial co-expression module including AD GWAS genes APOE (hub gene) and TREM2 co-expressed with genes involved in inflammation, complement and phagocytosis pathways (module 19). Triangles indicate module hub genes. Extended Data Fig. 7: Graphical display of significant correlations of AD GWAS genes in astrocytes and microglia with amyloid-beta and pTau pathology. a,b. Correlations of AD GWAS gene expression in astrocytes with amyloid (IQCK) and pTau (CLU, MEF2C, IQCK) pathology. c,d. Correlations of AD GWAS gene expression in microglia with amyloid (APOE, MS4A6A, PILRA) and pTau (APOE, PILRA) pathology. Extended Data Fig. 8: Differential gene expression in perivascular macrophages (PVMs) with amyloid-beta and pTau pathology. a,b. Volcano plot of transcripts differentially expressed in PVM nuclei (threshold of <0.05 adjusted p-value, abs logFC> 0.25, omitting the top three most variable genes between samples) with immunohistochemically-defined tissue amyloid-beta (a) and pTau (b). c. Functional pathway enrichment of gene sets differentially expressed with amyloid-beta and pTau pathology in PVMs. d. Venn diagram illustrating the number of genes positively (top) and negatively (bottom) correlated with amyloid-beta (blue) and pTau (pink) pathology in PVMs. Extended Data Fig. 9: Astrocyte (a) and microglial (b) nuclei are enriched in pathways uniquely expressed with greater amyloid-beta or pTau. Extended Data Fig. 10: Some astrocyte (a, b) and microglial (c,d) sub-clusters show trends for differences in relative abundance with greater amyloid-beta or pTau. Extended Data Fig. 11: Glial nuclei clusters are enriched (AUCell) in gene sets previously defined in preclinical models and human microglia. Enrichment of microglial gene sets in Micro1, Micro2, Micro3 and PVM nuclei clusters (the scaled average AUCell enrichment score, see Methods). Rodent DAM[27] and ARM[48] microglia transcripts are relatively enriched in Micro2 and PVMs. Aging human microglia transcripts[43] are relatively enriched in Micro2, Micro3 and PVMs. Human microglia core transcripts[15] are relatively enriched in Micro1. Cohen’s d effect sizes are provided in Supplementary Table 2, online resource (PDF 1851 kb)

Supplementary file2 (PDF 22369 kb)

## References

[1] Aibar S, Gonzalez-Blas CB, Moerman T, Huynh-Thu VA, Imrichova H, Hulselmans G, Rambow F, Marine JC, Geurts P, Aerts J (2017). SCENIC: single-cell regulatory network inference and clustering. Nat Methods.

[2] Al-Dalahmah O, Sosunov AA, Shaik A, Ofori K, Liu Y, Vonsattel JP, Adorjan I, Menon V, Goldman JE (2020). Single-nucleus RNA-seq identifies Huntington disease astrocyte states. Acta Neuropathol Commun.

[3] Andrews SJ, Fulton-Howard B, Goate A (2020). Interpretation of risk loci from genome-wide association studies of Alzheimer's disease. Lancet Neurol.

[4] Baker M, Mackenzie IR, Pickering-Brown SM, Gass J, Rademakers R, Lindholm C, Snowden J, Adamson J, Sadovnick AD, Rollinson S (2006). Mutations in progranulin cause tau-negative frontotemporal dementia linked to chromosome 17. Nature.

[5] Benjamini Y, Hochberg Y (1995). Controlling the false discovery rate: a practical and powerful approach to multiple testing. J R Stat Soc: Ser B (Methodol).

[6] Cabili MN, Trapnell C, Goff L, Koziol M, Tazon-Vega B, Regev A, Rinn JL (2011). Integrative annotation of human large intergenic noncoding RNAs reveals global properties and specific subclasses. Genes Dev.

[7] Castanho I, Murray TK, Hannon E, Jeffries A, Walker E, Laing E, Baulf H, Harvey J, Bradshaw L, Randall A (2020). Transcriptional signatures of tau and amyloid neuropathology. Cell Rep.

[8] Chang D, Nalls MA, Hallgrímsdóttir IB, Hunkapiller J, van der Brug M, Cai F, Kerchner GA, Ayalon G, Bingol B, Sheng M (2017). A meta-analysis of genome-wide association studies identifies 17 new Parkinson's disease risk loci. Nat Genet.

[9] Chen EY, Tan CM, Kou Y, Duan Q, Wang Z, Meirelles GV, Clark NR, Ma’ayan A (2013). Enrichr: interactive and collaborative HTML5 gene list enrichment analysis tool. BMC Bioinform.

[10] Chen W-T, Lu A, Craessaerts K, Pavie B, Sala Frigerio C, Corthout N, Qian X, Laláková J, Kühnemund M, Voytyuk I (2020). Spatial transcriptomics and in situ sequencing to study Alzheimer’s disease. Cell.

[11] Das S, Li Z, Noori A, Hyman BT, Serrano-Pozo A (2020). Meta-analysis of mouse transcriptomic studies supports a context-dependent astrocyte reaction in acute CNS injury versus neurodegeneration. J Neuroinflamm.

[12] Efthymiou AG, Goate AM (2017). Late onset Alzheimer's disease genetics implicates microglial pathways in disease risk. Mol Neurodegener.

[13] Escartin C, Galea E, Lakatos A, O’Callaghan JP, Petzold GC, Serrano-Pozo A, Steinhäuser C, Volterra A, Carmignoto G, Agarwal A (2021). Reactive astrocyte nomenclature, definitions, and future directions. Nat Neurosci.

[14] Finak G, McDavid A, Yajima M, Deng J, Gersuk V, Shalek AK, Slichter CK, Miller HW, McElrath MJ, Prlic M (2015). MAST: a flexible statistical framework for assessing transcriptional changes and characterizing heterogeneity in single-cell RNA sequencing data. Genome Biol.

[15] Galatro TF, Holtman IR, Lerario AM, Vainchtein ID, Brouwer N, Sola PR, Veras MM, Pereira TF, Leite REP, Möller T (2017). Transcriptomic analysis of purified human cortical microglia reveals age-associated changes. Nat Neurosci.

[16] Gerrits E, Brouwer N, Kooistra SM, Woodbury ME, Vermeiren Y, Lambourne M, Mulder J, Kummer M, Möller T, Biber K (2021). Distinct amyloid-β and tau-associated microglia profiles in Alzheimer’s disease. Acta Neuropathol.

[17] Gerrits E, Heng Y, Boddeke EWGM, Eggen BJL (2020). Transcriptional profiling of microglia; current state of the art and future perspectives. Glia.

[18] Grubman A, Chew G, Ouyang JF, Sun G, Choo XY, McLean C, Simmons RK, Buckberry S, Vargas-Landin DB, Poppe D (2019). A single-cell atlas of entorhinal cortex from individuals with Alzheimer's disease reveals cell-type-specific gene expression regulation. Nat Neurosci.

[19] Habib N, McCabe C, Medina S, Varshavsky M, Kitsberg D, Dvir-Szternfeld R, Green G, Dionne D, Nguyen L, Marshall JL (2020). Disease-associated astrocytes in Alzheimer’s disease and aging. Nat Neurosci.

[20] Hao Q, Maret W (2005). Imbalance between pro-oxidant and pro-antioxidant functions of zinc in disease. J Alzheimers Dis.

[21] Hozumi I (2013). Roles and therapeutic potential of metallothioneins in neurodegenerative diseases. Curr Pharm Biotechnol.

[22] Huang DW, Sherman BT, Tan Q, Collins JR, Alvord WG, Roayaei J, Stephens R, Baseler MW, Lane HC, Lempicki RA (2007). The DAVID Gene Functional Classification Tool: a novel biological module-centric algorithm to functionally analyze large gene lists. Genome Biol.

[23] Hüttenrauch M, Ogorek I, Klafki H, Otto M, Stadelmann C, Weggen S, Wiltfang J, Wirths O (2018). Glycoprotein NMB: a novel Alzheimer's disease associated marker expressed in a subset of activated microglia. Acta Neuropathol Commun.

[24] Inestrosa NC, Toledo EM (2008). The role of Wnt signaling in neuronal dysfunction in Alzheimer's disease. Mol Neurodegener.

[25] Jansen IE, Savage JE, Watanabe K, Bryois J, Williams DM, Steinberg S, Sealock J, Karlsson IK, Hägg S, Athanasiu L (2019). Genome-wide meta-analysis identifies new loci and functional pathways influencing Alzheimer’s disease risk. Nat Genet.

[26] Jin S, Guerrero-Juarez CF, Zhang L, Chang I, Ramos R, Kuan CH, Myung P, Plikus MV, Nie Q (2021). Inference and analysis of cell-cell communication using Cell Chat. Nat Commun.

[27] Keren-Shaul H, Spinrad A, Weiner A, Matcovitch-Natan O, Dvir-Szternfeld R, Ulland TK, David E, Baruch K, Lara-Astaiso D, Toth B (2017). A unique microglia type associated with restricting development of Alzheimer's disease. Cell.

[28] Khozoie CF, Nurun M, Marjaneh M, Murphy AE, Matthews PM, Skene N (2021) scFlow: a scalable and reproducible analysis pipeline for single-cell RNA sequencing data. bioRxiv 2021.08.16.456499. 10.1101/2021.08.16.456499

[29] Kim W-K, Alvarez X, Fisher J, Bronfin B, Westmoreland S, McLaurin J, Williams K (2006). CD163 identifies perivascular macrophages in normal and viral encephalitic brains and potential precursors to perivascular macrophages in blood. Am J Pathol.

[30] Krishnaswami SR, Grindberg RV, Novotny M, Venepally P, Lacar B, Bhutani K, Linker SB, Pham S, Erwin JA, Miller JA (2016). Using single nuclei for RNA-seq to capture the transcriptome of postmortem neurons. Nat Protoc.

[31] Kunkle BW, Grenier-Boley B, Sims R, Bis JC, Damotte V, Naj AC, Boland A, Vronskaya M, van der Lee SJ, Amlie-Wolf A (2019). Genetic meta-analysis of diagnosed Alzheimer’s disease identifies new risk loci and implicates Aβ, tau, immunity and lipid processing. Nat Genet.

[32] Lake BB, Ai R, Kaeser GE, Salathia NS, Yung YC, Liu R, Wildberg A, Gao D, Fung H-L, Chen S (2016). Neuronal subtypes and diversity revealed by single-nucleus RNA sequencing of the human brain. Science.

[33] Lambert SA, Jolma A, Campitelli LF, Das PK, Yin Y, Albu M, Chen X, Taipale J, Hughes TR, Weirauch MT (2018). The human transcription factors. Cell.

[34] Lau S-F, Cao H, Fu AKY, Ip NY (2020). Single-nucleus transcriptome analysis reveals dysregulation of angiogenic endothelial cells and neuroprotective glia in Alzheimer’s disease. Proc Natl Acad Sci.

[35] Liddelow SA, Guttenplan KA, Clarke LE, Bennett FC, Bohlen CJ, Schirmer L, Bennett ML, Münch AE, Chung W-S, Peterson TC (2017). Neurotoxic reactive astrocytes are induced by activated microglia. Nature.

[36] Mathys H, Davila-Velderrain J, Peng Z, Gao F, Mohammadi S, Young JZ, Menon M, He L, Abdurrob F, Jiang X (2019). Single-cell transcriptomic analysis of Alzheimer's disease. Nature.

[37] Matsuda S, Matsuda Y, D'Adamio L (2009). CD74 interacts with APP and suppresses the production of Abeta. Mol Neurodegener.

[38] Meyers EA, Kessler JA (2017). TGF-beta family signaling in neural and neuronal differentiation, development, and function. Cold Spring Harb Perspect Biol.

[39] Moerman T, Aibar Santos S, Bravo Gonzalez-Blas C, Simm J, Moreau Y, Aerts J, Aerts S (2019). GRNBoost2 and Arboreto: efficient and scalable inference of gene regulatory networks. Bioinformatics.

[40] Naba A, Clauser KR, Hoersch S, Liu H, Carr SA, Hynes RO (2012). The matrisome: in silico definition and in vivo characterization by proteomics of normal and tumor extracellular matrices. Mol Cell Proteom.

[41] Nguyen AT, Wang K, Hu G, Wang X, Miao Z, Azevedo JA, Suh E, Van Deerlin VM, Choi D, Roeder K (2020). APOE and TREM2 regulate amyloid-responsive microglia in Alzheimer's disease. Acta Neuropathol.

[42] Nott A, Holtman IR, Coufal NG, Schlachetzki JCM, Yu M, Hu R, Han CZ, Pena M, Xiao J, Wu Y (2019). Brain cell type-specific enhancer-promoter interactome maps and disease-risk association. Science (New York, NY).

[43] Olah M, Patrick E, Villani A-C, Xu J, White CC, Ryan KJ, Piehowski P, Kapasi A, Nejad P, Cimpean M (2018). A transcriptomic atlas of aged human microglia. Nat Commun.

[44] Patir A, Shih B, McColl BW, Freeman TC (2019). A core transcriptional signature of human microglia: Derivation and utility in describing region-dependent alterations associated with Alzheimer's disease. Glia.

[45] Ritchie ME, Phipson B, Wu D, Hu Y, Law CW, Shi W, Smyth GK (2015). limma powers differential expression analyses for RNA-sequencing and microarray studies. Nucleic Acids Res.

[46] Rohne P, Prochnow H, Wolf S, Renner B, Koch-Brandt C (2014). The chaperone activity of clusterin is dependent on glycosylation and redox environment. Cell Physiol Biochem.

[47] Roy ER, Wang B, Wan Y-w, Chiu G, Cole A, Yin Z, Propson NE, Xu Y, Jankowsky JL, Liu Z (2020). Type I interferon response drives neuroinflammation and synapse loss in Alzheimer disease. J Clin Investig.

[48] Sala Frigerio C, Wolfs L, Fattorelli N, Thrupp N, Voytyuk I, Schmidt I, Mancuso R, Chen W-T, Woodbury ME, Srivastava G (2019). The major risk factors for alzheimer's disease: age, sex, and genes modulate the microglia response to Aβ plaques. Cell Rep.

[49] Sankowski R, Böttcher C, Masuda T, Geirsdottir L, Sagar SE, Seredenina T, Muhs A, Scheiwe C, Shah MJ (2019). Mapping microglia states in the human brain through the integration of high-dimensional techniques. Nat Neurosci.

[50] Schartz ND, Tenner AJ (2020). The good, the bad, and the opportunities of the complement system in neurodegenerative disease. J Neuroinflamm.

[51] Schirmer L, Velmeshev D, Holmqvist S, Kaufmann M, Werneburg S, Jung D, Vistnes S, Stockley JH, Young A, Steindel M (2019). Neuronal vulnerability and multilineage diversity in multiple sclerosis. Nature.

[52] Serrano-Pozo A, Muzikansky A, Gómez-Isla T, Growdon JH, Betensky RA, Frosch MP, Hyman BT (2013). Differential relationships of reactive astrocytes and microglia to fibrillar amyloid deposits in Alzheimer disease. J Neuropathol Exp Neurol.

[53] Shannon P, Markiel A, Ozier O, Baliga NS, Wang JT, Ramage D, Amin N, Schwikowski B, Ideker T (2003). Cytoscape: a software environment for integrated models of biomolecular interaction networks. Genome Res.

[54] Sharma S, Ebadi M (2014). Significance of metallothioneins in aging brain. Neurochem Int.

[55] Sierksma A, Lu A, Mancuso R, Fattorelli N, Thrupp N, Salta E, Zoco J, Blum D, Buée L, De Strooper B (2020). Novel Alzheimer risk genes determine the microglia response to amyloid-β but not to TAU pathology. EMBO Mol Med.

[56] Skene NG, Grant SGN (2016). Identification of vulnerable cell types in major brain disorders using single cell transcriptomes and expression weighted cell type enrichment. Front Neurosci.

[57] Smillie CS, Biton M, Ordovas-Montanes J, Sullivan KM, Burgin G, Graham DB, Herbst RH, Rogel N, Slyper M, Waldman J (2019). Intra- and inter-cellular rewiring of the human colon during ulcerative colitis. Cell.

[58] Song WM, Zhang B (2015). Multiscale embedded gene co-expression network analysis. PLoS Comput Biol.

[59] Sorrells SF, Paredes MF, Velmeshev D, Herranz-Pérez V, Sandoval K, Mayer S, Chang EF, Insausti R, Kriegstein AR, Rubenstein JL (2019). Immature excitatory neurons develop during adolescence in the human amygdala. Nat Commun.

[60] Streit WJ, Braak H, Xue Q-S, Bechmann I (2009). Dystrophic (senescent) rather than activated microglial cells are associated with tau pathology and likely precede neurodegeneration in Alzheimer's disease. Acta Neuropathol.

[61] Stuart T, Butler A, Hoffman P, Hafemeister C, Papalexi E, Mauck Iii WM, Hao Y, Stoeckius M, Smibert P, Satija R (2019). Comprehensive integration of single-cell data. Cell.

[62] Sun Y, Senger K, Baginski TK, Mazloom A, Chinn Y, Pantua H, Hamidzadeh K, Ramani SR, Luis E, Tom I (2012). Evolutionarily conserved paired immunoglobulin-like receptor alpha (PILRalpha) domain mediates its interaction with diverse sialylated ligands. J Biol Chem.

[63] Suo S, Zhu Q, Saadatpour A, Fei L, Guo G, Yuan GC (2018). Revealing the critical regulators of cell identity in the mouse cell atlas. Cell Rep.

[64] Thrupp N, Frigerio CS, Wolfs L, Skene NG, Poovathingal S, Fourne Y, Matthews PM, Theys T, Mancuso R, de Strooper Bet al (2020) Single nucleus sequencing fails to detect microglial activation in human tissue. bioRxiv: 2020.2004.2013.035386–032020.035304.035313.035386. 10.1101/2020.04.13.035386

[65] Traag VA, Waltman L, van Eck NJ (2019). From Louvain to Leiden: guaranteeing well-connected communities. Sci Rep.

[66] Trousse F, Jemli A, Silhol M, Garrido E, Crouzier L, Naert G, Maurice T, Rossel M (2019). Knockdown of the CXCL12/CXCR7 chemokine pathway results in learning deficits and neural progenitor maturation impairment in mice. Brain Behav Immun.

[67] Van de Sande B, Flerin C, Davie K, De Waegeneer M, Hulselmans G, Aibar S, Seurinck R, Saelens W, Cannoodt R, Rouchon Q (2020). A scalable SCENIC workflow for single-cell gene regulatory network analysis. Nat Protoc.

[68] van der Poel M, Ulas T, Mizee MR, Hsiao C-C, Miedema SSM, Adelia SKG, Helder B, Tas SW, Schultze JL (2019). Transcriptional profiling of human microglia reveals grey-white matter heterogeneity and multiple sclerosis-associated changes. Nat Commun.

[69] Vilalta A, Brown GC (2018). Neurophagy, the phagocytosis of live neurons and synapses by glia, contributes to brain development and disease. FEBS J.

[70] Wan Y-W, Al-Ouran R, Mangleburg CG, Perumal TM, Lee TV, Allison K, Swarup V, Funk CC, Gaiteri C, Allen M (2020). Meta-analysis of the Alzheimer's disease human brain transcriptome and functional dissection in mouse models. Cell Rep.

[71] Ward ME, Chen R, Huang H-Y, Ludwig C, Telpoukhovskaia M, Taubes A, Boudin H, Minami SS, Reichert M, Albrecht P (2017). Individuals with progranulin haploinsufficiency exhibit features of neuronal ceroid lipofuscinosis. Sci Transl Med.

[72] Welch JD, Kozareva V, Ferreira A, Vanderburg C, Martin C, Macosko EZ (2019). Single-cell multi-omic integration compares and contrasts features of brain cell identity. Cell.

[73] Yeung MSY, Djelloul M, Steiner E, Bernard S, Salehpour M, Possnert G, Brundin L, Frisén J (2019). Dynamics of oligodendrocyte generation in multiple sclerosis. Nature.

[74] Zhang X, Li J, Ma L, Xu H, Cao Y, Liang W, Ma J, Wang ZP, Li Y (2021). BMP4 overexpression induces the upregulation of APP/Tau and memory deficits in Alzheimer's disease. Cell Death Discov.

[75] Zhang Y, Sloan SA, Clarke LE, Caneda C, Plaza CA, Blumenthal PD, Vogel H, Steinberg GK, Edwards MS, Li G (2016). Purification and characterization of progenitor and mature human astrocytes reveals transcriptional and functional differences with mouse. Neuron.

[76] Zhou Y, Song WM, Andhey PS, Swain A, Levy T, Miller KR, Poliani PL, Cominelli M, Grover S, Gilfillan S (2020). Human and mouse single-nucleus transcriptomics reveal TREM2-dependent and TREM2-independent cellular responses in Alzheimer's disease. Nat Med.

[77] Zimmerman KD, Espeland MA, Langefeld CD (2021). A practical solution to pseudoreplication bias in single-cell studies. Nat Commun.

