# Supplementary material for: Diverse human astrocyte and microglial transcriptional responses to Alzheimer’s pathology
Source: Acta Neuropathol. 2021 Nov 12;143(1):75–91. doi: 10.1007/s00401-021-02372-6 (PMC8732962; doi:10.1007/s00401-021-02372-6)

Ext Data Fig 1

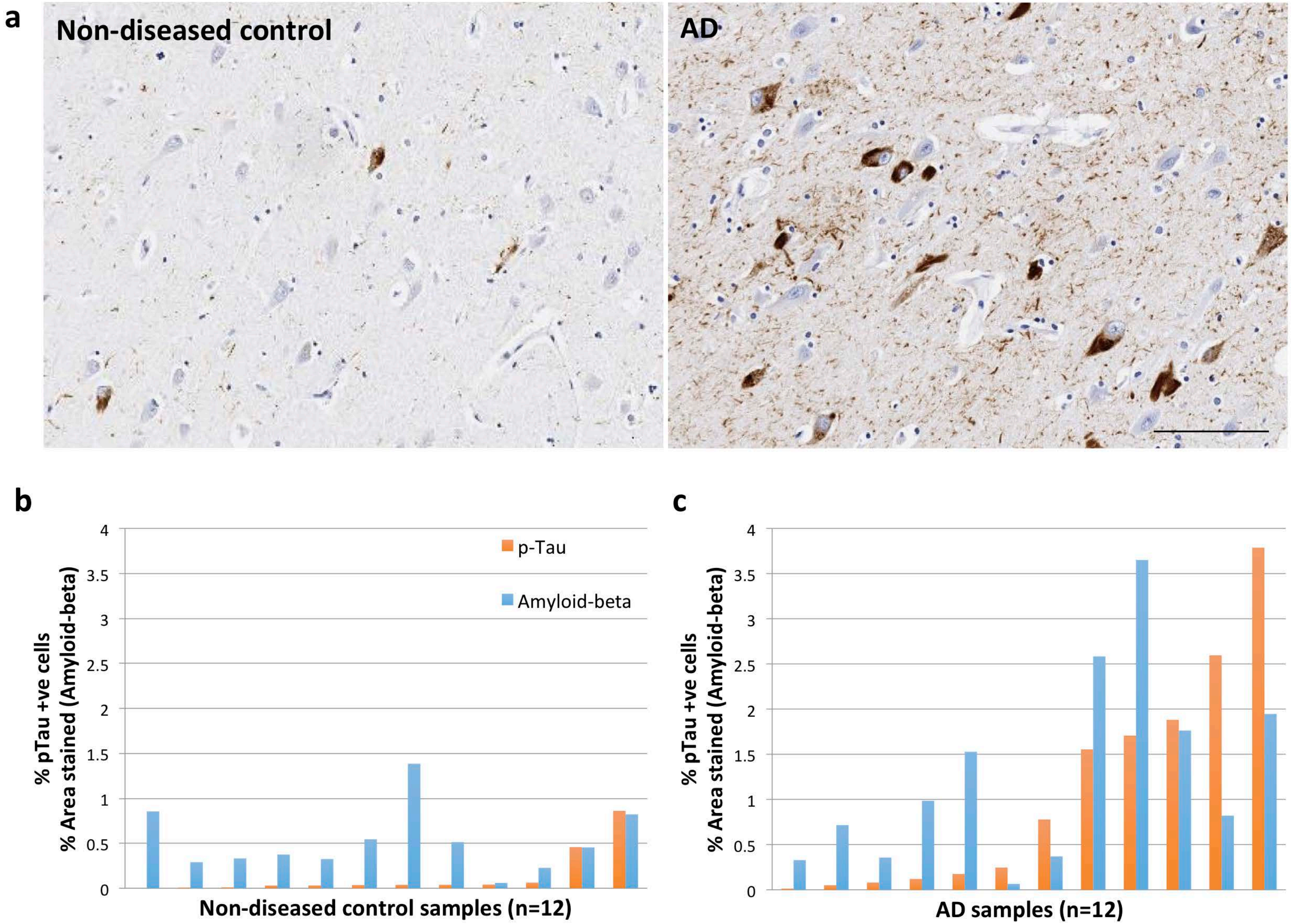

Ext Data Fig 2

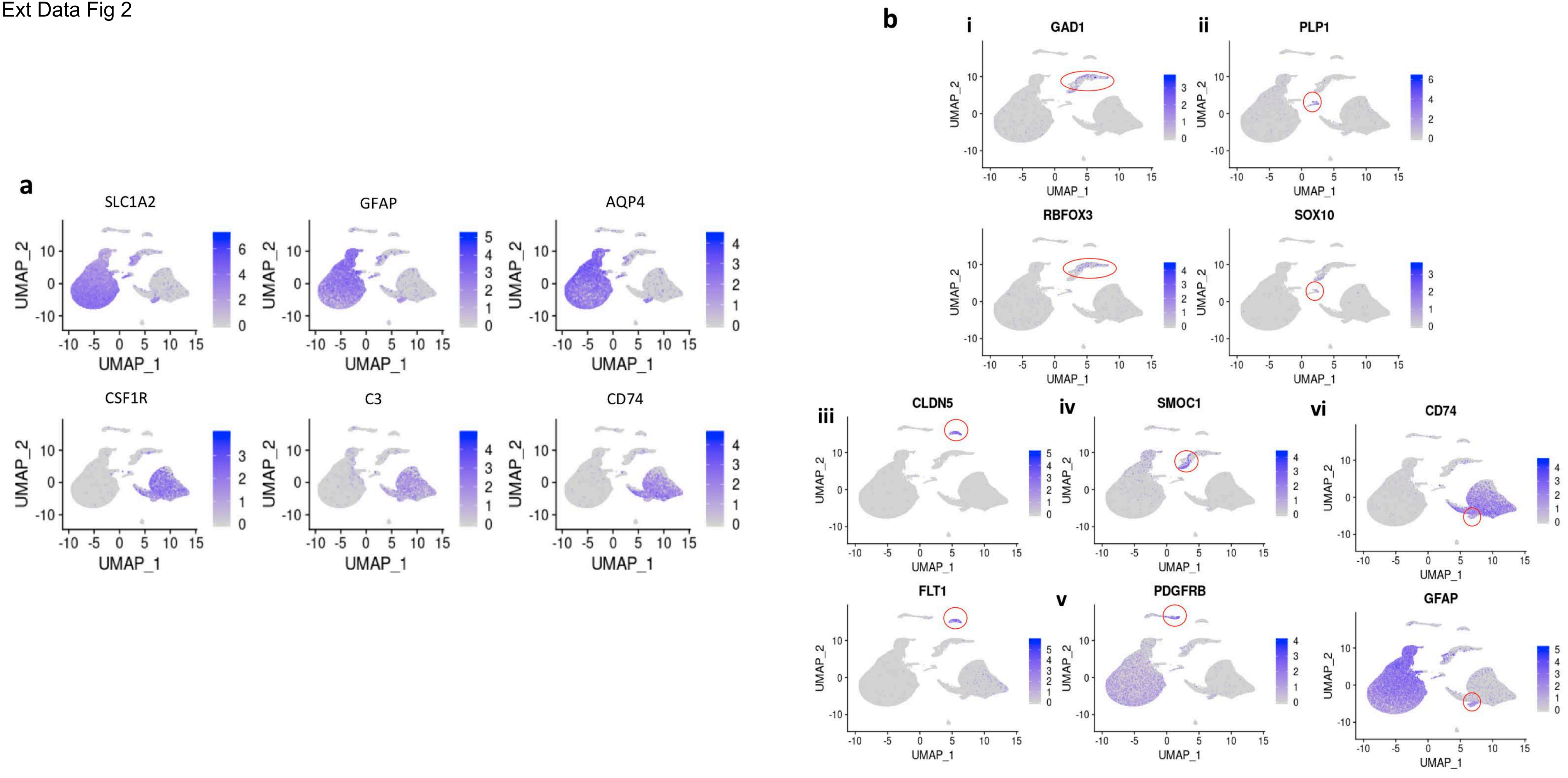

Ext Data Fig 3

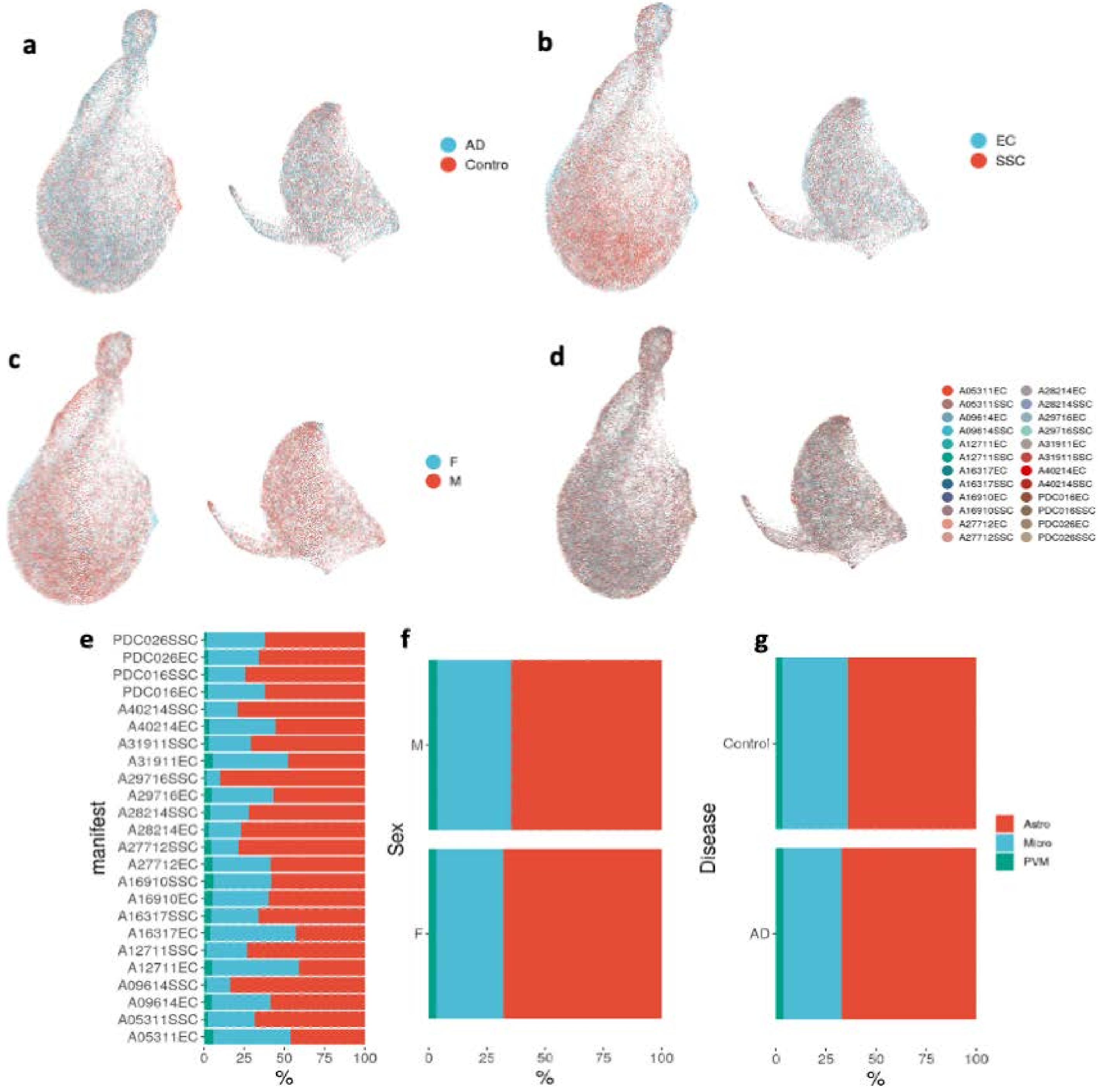

Ext Data Fig 4

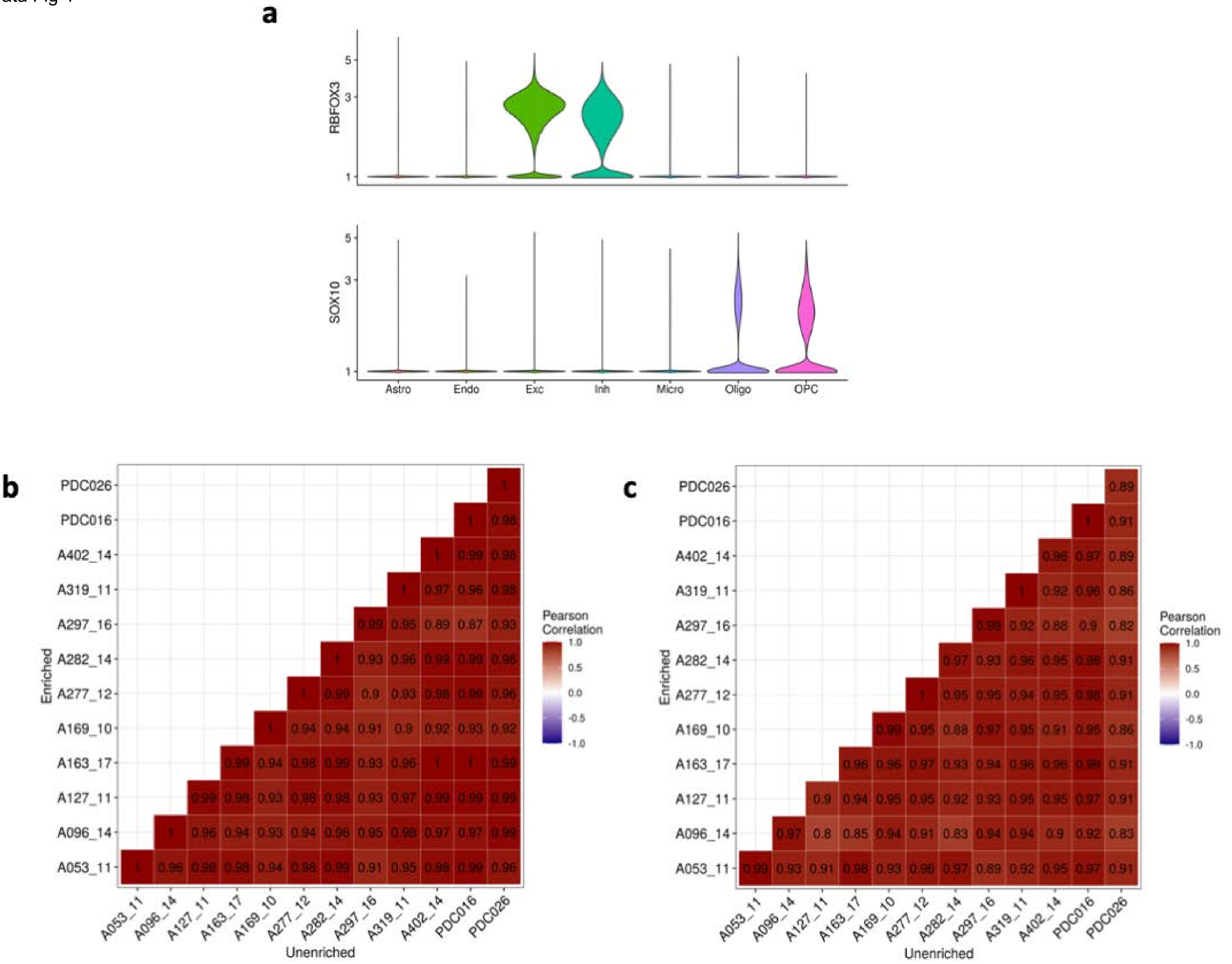

Ext Data Fig 5

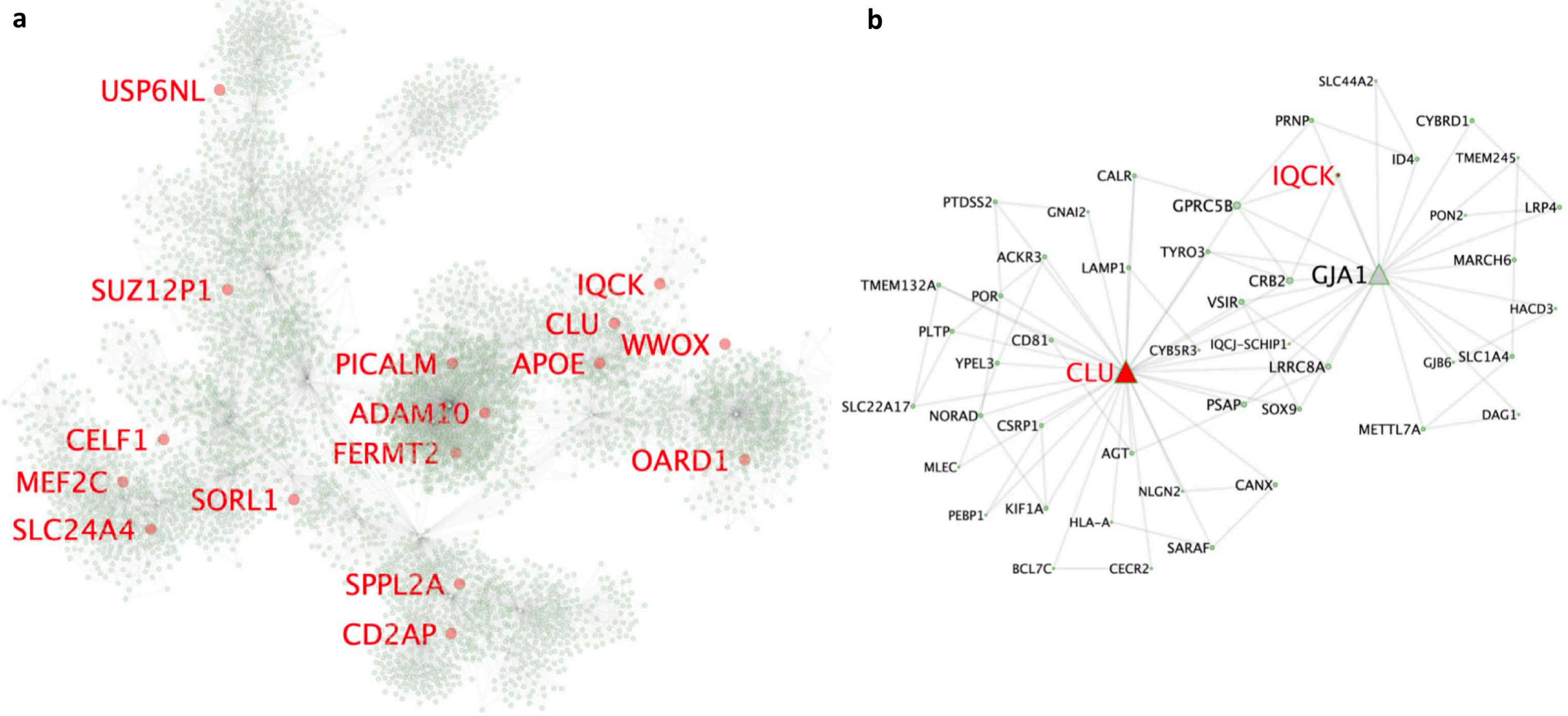

Ext Data Fig 6

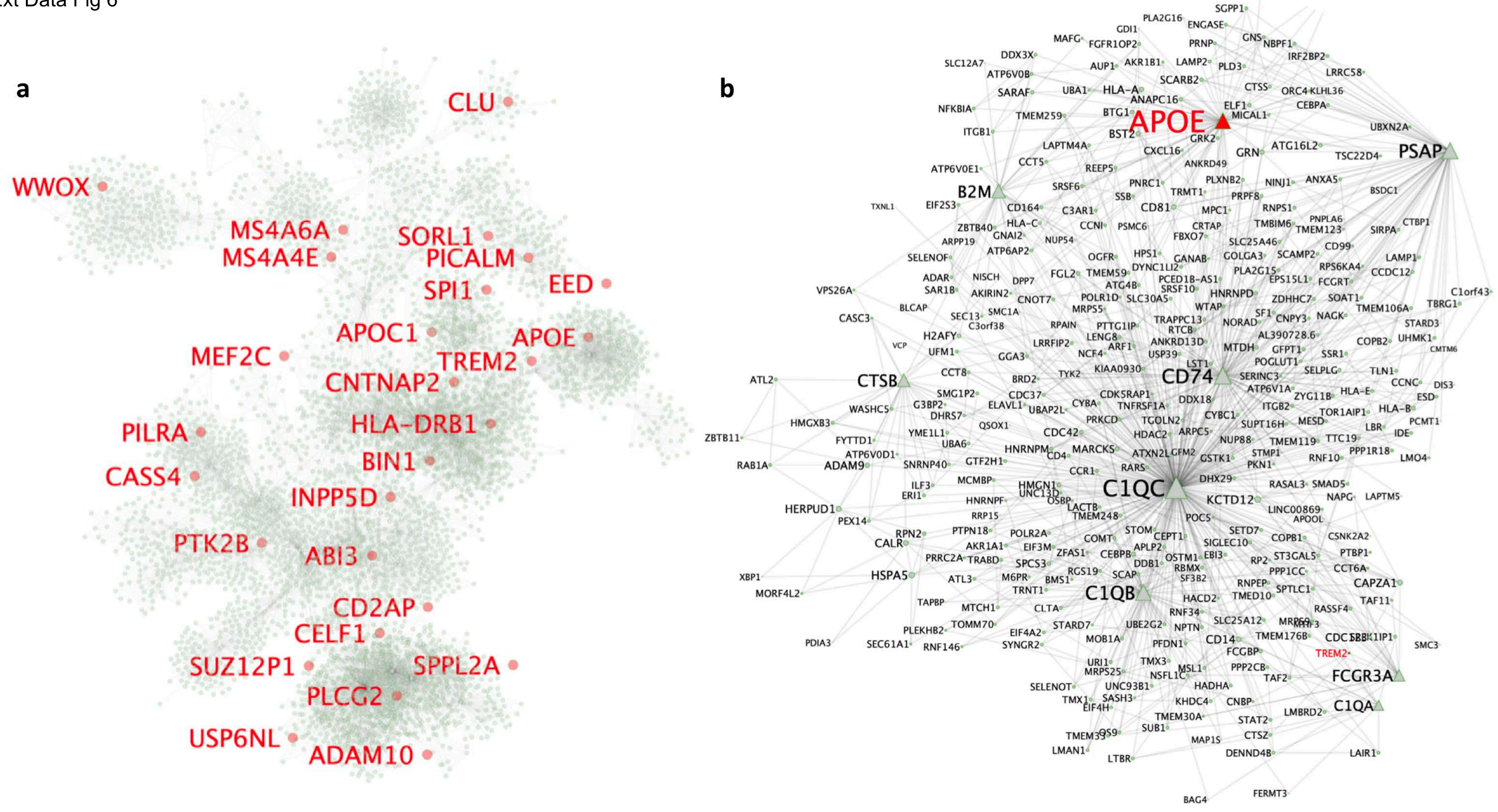

Ext Data Fig 7

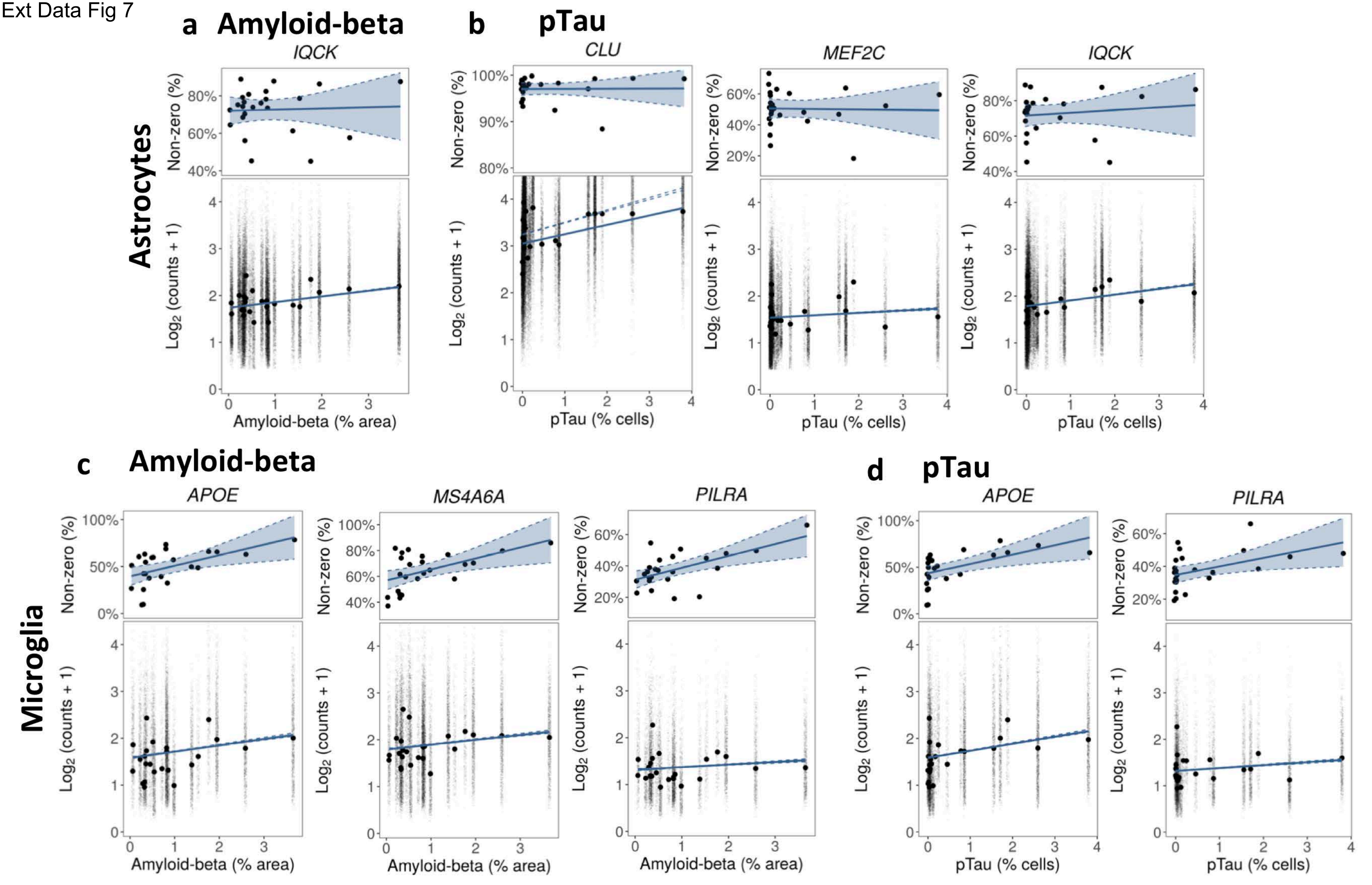

Ext Data Fig 8

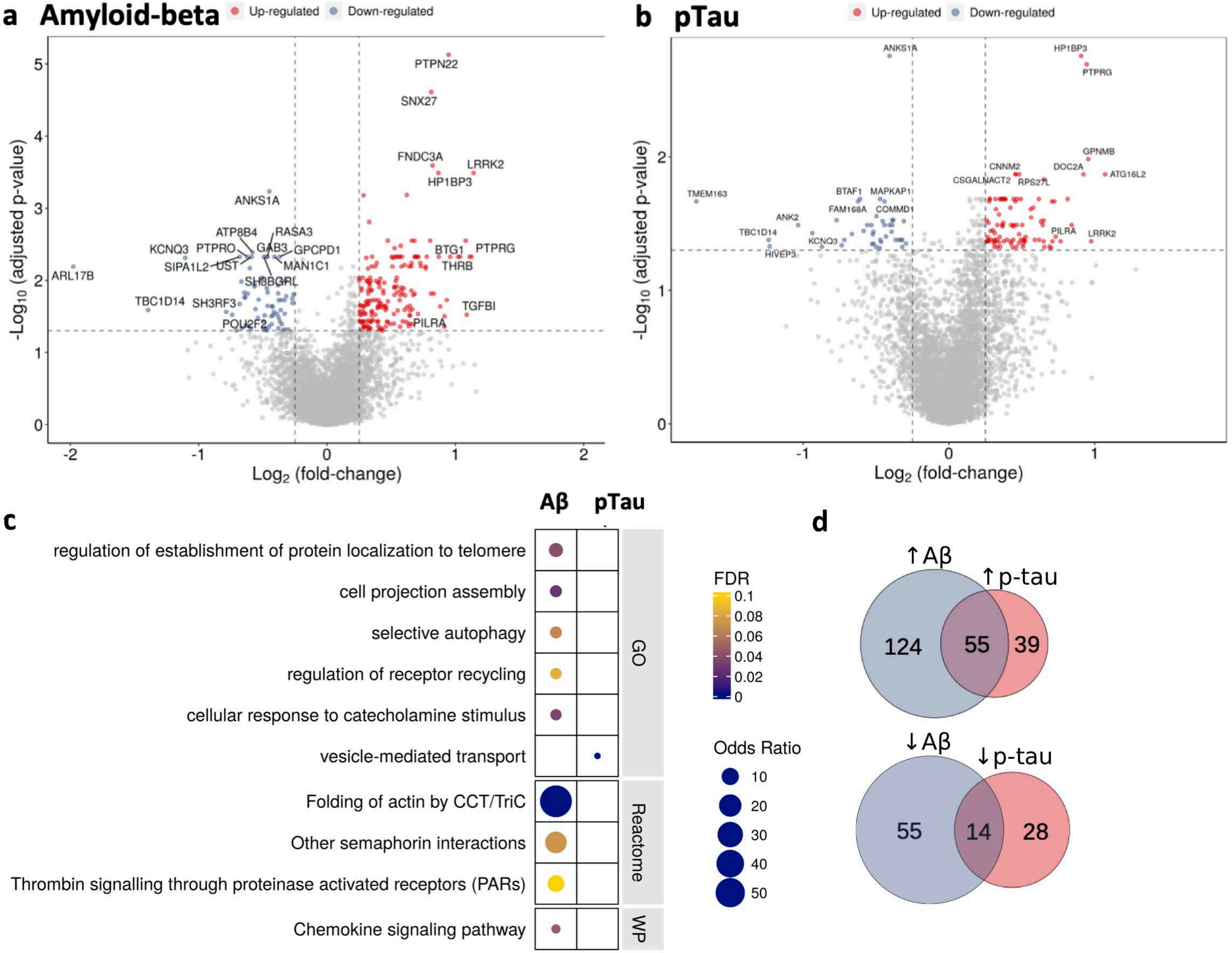

Ext Data Fig 9

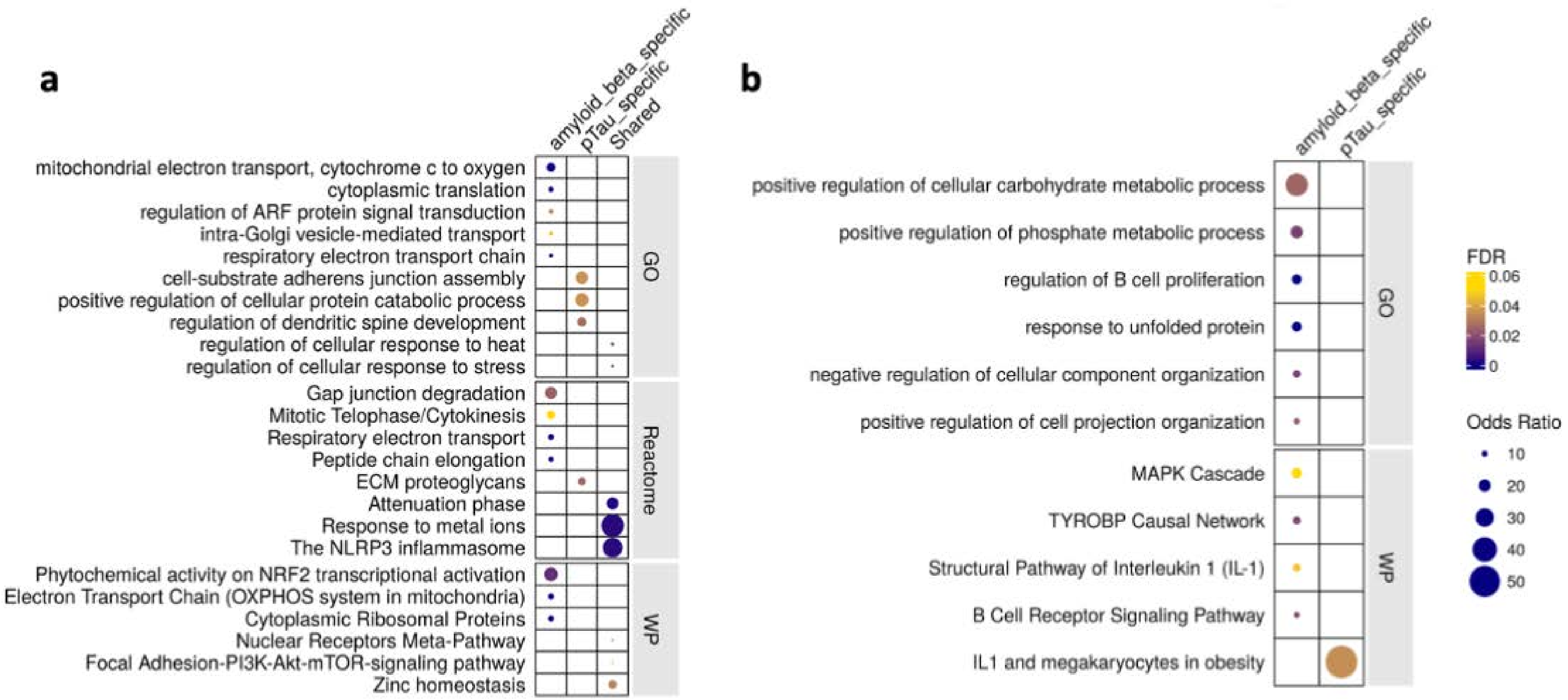

Ext Data Fig 10

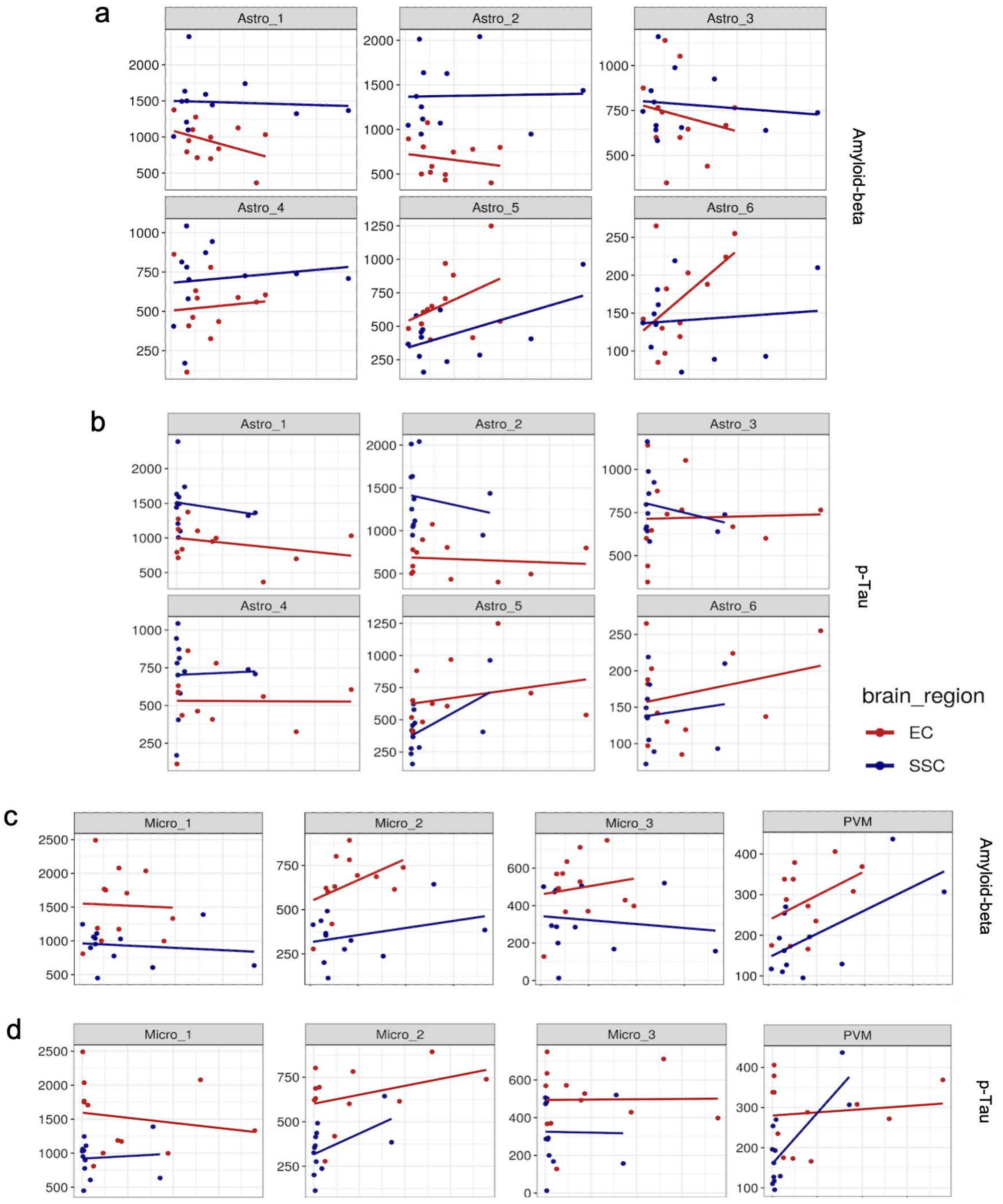

Ext Data Fig 11

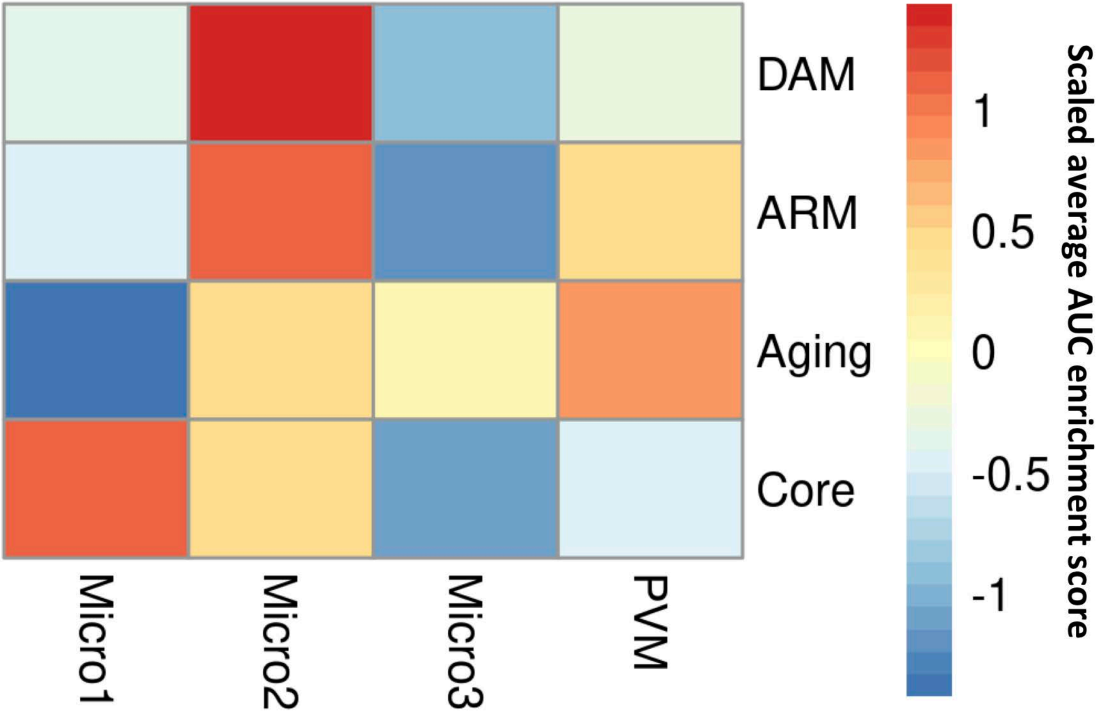

Supplement: Supplementary file 1 — Supplementary file1 Extended Data Fig. 1: Measures of pathology in brains used for snRNASeq. a) Immunohistochemical (IHC) stains for pTau (AT8 antibody) of sections of representative formalin-fixed and paraffin embedded tissue from the entorhinal cortex in the contralateral hemisphere of typical AD (Braak stage III-VI) and NDC (Braak stage 0-II) cortical samples used in this study. Quantitative IHC measures of pTau in neurofibrillary tangles (% positive cells) (AT8 antibody, orange) and amyloid-beta plaques (% area stained) (4G8 antibody, blue) in somatosensory or entorhinal cortical sections (b) from NDC (low Braak 0-II) and c) AD (high Braak III-VI) samples. Each bar describes IHC results from a different cortical sample in either of the two regions from brains used in this study. The sample data for both (b) and (c) are ordered with respect to increasing pTau pathology load. Extended Data Fig. 2: Cell-type clusters identified by snRNASeq of NeuN and Sox10-depleted human brain nuclei. a. UMAP feature plots demonstrating astrocyte-specific (SLC1A2, GFAP, AQP4) and microglia-specific (CSF1R, C3, CD74) marker transcripts. b. UMAP feature plots for markers of cell types other than microglia and astrocytes in the glial-enriched samples: i. neurons, ii. oligodendrocytes, iii. endothelial cells, iv. oligodendroglial progenitor cells (OPC), v. pericytes, and vi. unclassified nuclei. Extended Data Fig. 3: Good integration of nuclei across all of the enriched samples is illustrated by the good mixing of nuclei from a. AD and NDC donor brains, b. entorhinal (EC) and somatosensory (SSC) cortical tissues, c. female (F) and male (M) donors and d. the 24 individual samples. Relative numbers of nuclei in the clusters (astrocytes, red; microglia, blue; PVM, green) from each sample (e). No significant differences were found between those from female (F) and male (M) donors. There was a small trend to a smaller proportion of microglial nuclei in the AD samples relative to NDC (g), a [file 401_2021_2372_MOESM1_ESM.pdf]
