# Supplementary material for: Diverse human astrocyte and microglial transcriptional responses to Alzheimer’s pathology
Source: Acta Neuropathol. 2021 Nov 12;143(1):75–91. doi: 10.1007/s00401-021-02372-6 (PMC8732962; doi:10.1007/s00401-021-02372-6)
Supplement: Supplementary file 2 — Supplementary file2 (PDF 22369 kb) [file 401_2021_2372_MOESM2_ESM.pdf]

Supplementary Table 1_Sample data and sequencing template

METADATA TEMPLATE

# High-throughput sequencing metadata template. (Version: April 28, 2020)
# All fields in this template must be completed.
# See METADATA EXAMPLES in tabs at the foot of this page.
# Field names (in blue on this page) should not be edited. Hover over cells containing field names to view field content guidelines.
# Human data. If there are patient privacy concerns regarding making data fully public through GEO, please submit to NCBI's dbGaP (http://www.ncbi.nlm.nih.gov/gap/) database. dbGaP has controlled access mechanisms and is an appropriate resource for hosting sensitive patient data.

SERIES

This section describes the overall study. Complete all fields.

**title**	Diverse human astrocyte and microglial transcriptional responses to Alzheimer's pathology
**summary**	To better define roles that astrocytes and microglia play in Alzheimer's disease (AD), we used single-nuclei RNA sequencing to comprehensively characterize transcriptomes in astrocyte and microglia nuclei isolated post mortem from neuropathologically-defined AD and control brains with a range of amyloid-beta and phospho-tau (pTau) pathology. Significant differences in glial gene expression (including AD risk genes expressed in astrocytes [CLU, MEF2C, IQCK] and microglia [APOE, MS4A6A, PILRA]) were correlated with tissue amyloid and pTau expression. Astrocytes were enriched for proteostatic, inflammatory and metal ion homeostasis pathways. Pathways for phagocytosis, proteostasis and autophagy were highly enriched in microglia and perivascular macrophages. Gene co-expression analyses revealed potential functional associations of soluble biomarkers of AD in astrocytes (CLU) and microglia (GPNMB). Our work highlights responses of both astrocytes and microglia for pathological protein clearance and inflammation, as well as glial transcriptional diversity in AD.
**overall design**	Single-nuclei RNA-sequencing of microglia and astrocyte nuclei isolated *post mortem* from non-diseased control and neuropathologically-defined AD brains.
**contributor**	Amy, M, Smith
contributor	Karen, Davey
contributor	Stergios, Tsartsalis
contributor	Combiz, Khozoie
contributor	Nurun, Fancy
contributor	See Swee, Tang
contributor	Eirini, Liaptsi
contributor	Maria, Weinert
contributor	Aisling, McGarry
contributor	Robert, CJ, Muirhead
contributor	Steve, Gentleman
contributor	David, R, Owen
contributor	Paul, M, Matthews
supplementary file	

SAMPLES

Thoroughly describe each of the biological Samples under investigation. **Tissue/cell type/cell line** information is required.
# Include all biological and experimental variables in characteristics columns (for example, characteristics: tissue, characteristics: cell type, characteristics: treatment, characteristics: genotype, characteristics: disease state or any clinical variables, etc...)
# -> -> KEEP SCROLLING TO THE RIGHT FOR IMPORTANT FIELDS; RAW AND PROCESSED FILES MUST BE LISTED IN THIS SECTION -> ->

Sample name	title	source name	organism	characteristics: Disease categ	characteristics: Braak tangle sta	characteristics: Brain regi	characteristics: Ag	characteristics: Se	characteristics: RI	characteristics: pTau immunosta	characteristics: Amyloid immunc	molecule	description	processed data file	raw file	raw file
IGF112633	A127/11 SSC	Human post-mortem cortex	Homo sapiens	Non-disease control	0	SSC	73	male	6.3	0.004665811	0.289797	nuclear RNA		IGF112633_filtered_feature_bc_matrix.tar.gz	IGF112633_HISEQ4000_cellranger_count_HG38_20191005.cram	IGF112633_HISEQ4000_cellranger_count_HG38_20191005.cram.crai
IGF112634	A053/11 SSC	Human post-mortem cortex	Homo sapiens	Non-disease control	0	SSC	77	male	6.7	0.031438067	0.3253	nuclear RNA		IGF112634_filtered_feature_bc_matrix.tar.gz	IGF112634_HISEQ4000_cellranger_count_HG38_20191006.cram	IGF112634_HISEQ4000_cellranger_count_HG38_20191006.cram.crai
IGF112635	A282/14 SSC	Human post-mortem cortex	Homo sapiens	AD	3	SSC	68	female	8.1	0.014272971	0.329722	nuclear RNA		IGF112635_filtered_feature_bc_matrix.tar.gz	IGF112635_HISEQ4000_cellranger_count_HG38_20191005.cram	IGF112635_HISEQ4000_cellranger_count_HG38_20191005.cram.crai
IGF112636	A127/11 EC	Human post-mortem cortex	Homo sapiens	Non-disease control	0	EC	73	male	6	0.010239607	0.331774	nuclear RNA		IGF112636_filtered_feature_bc_matrix.tar.gz	IGF112636_HISEQ4000_cellranger_count_HG38_20191005.cram	IGF112636_HISEQ4000_cellranger_count_HG38_20191005.cram.crai
IGF112637	A282/14 EC	Human post-mortem cortex	Homo sapiens	AD	3	EC	68	female	8.2	0.247326689	0.066141	nuclear RNA		IGF112637_filtered_feature_bc_matrix.tar.gz	IGF112637_HISEQ4000_cellranger_count_HG38_20191005.cram	IGF112637_HISEQ4000_cellranger_count_HG38_20191005.cram.crai
IGF112638	A096/14 SSC	Human post-mortem cortex	Homo sapiens	AD	4	SSC	87	male	6.9	0.176706404	1.52959	nuclear RNA		IGF112638_filtered_feature_bc_matrix.tar.gz	IGF112638_HISEQ4000_cellranger_count_HG38_20191006.cram	IGF112638_HISEQ4000_cellranger_count_HG38_20191006.cram.crai
IGF112639	A163/17 SSC	Human post-mortem cortex	Homo sapiens	AD	5	SSC	83	male	7.6	0.081283908	0.357876	nuclear RNA		IGF112639_filtered_feature_bc_matrix.tar.gz	IGF112639_HISEQ4000_cellranger_count_HG38_20191005.cram	IGF112639_HISEQ4000_cellranger_count_HG38_20191005.cram.crai
IGF112640	A096/14 EC	Human post-mortem cortex	Homo sapiens	AD	4	EC	87	male	6	0.780064092	0.370786	nuclear RNA		IGF112640_filtered_feature_bc_matrix.tar.gz	IGF112640_HISEQ4000_cellranger_count_HG38_20191005.cram	IGF112640_HISEQ4000_cellranger_count_HG38_20191005.cram.crai
IGF112641	A163/17 EC	Human post-mortem cortex	Homo sapiens	AD	5	EC	83	male	7.7	2.597481599	0.821893	nuclear RNA		IGF112641_filtered_feature_bc_matrix.tar.gz	IGF112641_HISEQ4000_cellranger_count_HG38_20191006.cram	IGF112641_HISEQ4000_cellranger_count_HG38_20191006.cram.crai
IGF112642	A402/14 SSC	Human post-mortem cortex	Homo sapiens	AD	3	SSC	83	male	6.8	0.052077306	0.717751	nuclear RNA		IGF112642_filtered_feature_bc_matrix.tar.gz	IGF112642_HISEQ4000_cellranger_count_HG38_20191006.cram	IGF112642_HISEQ4000_cellranger_count_HG38_20191006.cram.crai
IGF112643	A169/10 SSC	Human post-mortem cortex	Homo sapiens	AD	5	SSC	85	female	7.1	1.555636545	2.58539	nuclear RNA		IGF112643_filtered_feature_bc_matrix.tar.gz	IGF112643_HISEQ4000_cellranger_count_HG38_20191006.cram	IGF112643_HISEQ4000_cellranger_count_HG38_20191006.cram.crai
IGF112644	A053/11 EC	Human post-mortem cortex	Homo sapiens	Non-disease control	0	EC	77	male	6.8	0.036788375	0.5444	nuclear RNA		IGF112644_filtered_feature_bc_matrix.tar.gz	IGF112644_HISEQ4000_cellranger_count_HG38_20191005.cram	IGF112644_HISEQ4000_cellranger_count_HG38_20191005.cram.crai
IGF112645	A402/14 EC	Human post-mortem cortex	Homo sapiens	AD	3	EC	83	male	6.9	0.12159934	0.987485	nuclear RNA		IGF112645_filtered_feature_bc_matrix.tar.gz	IGF112645_HISEQ4000_cellranger_count_HG38_20191005.cram	IGF112645_HISEQ4000_cellranger_count_HG38_20191005.cram.crai
IGF112646	A169/10 EC	Human post-mortem cortex	Homo sapiens	AD	5	EC	85	female	6.6	1.883817427	1.76432	nuclear RNA		IGF112646_filtered_feature_bc_matrix.tar.gz	IGF112646_HISEQ4000_cellranger_count_HG38_20191005.cram	IGF112646_HISEQ4000_cellranger_count_HG38_20191005.cram.crai
IGF112647	A297/16 SSC	Human post-mortem cortex	Homo sapiens	Non-disease control	1	SSC	81	male	2.7	0.030622244	0.374616	nuclear RNA		IGF112647_filtered_feature_bc_matrix.tar.gz	IGF112647_HISEQ4000_cellranger_count_HG38_20191005.cram	IGF112647_HISEQ4000_cellranger_count_HG38_20191005.cram.crai
IGF112648	A297/16 EC	Human post-mortem cortex	Homo sapiens	Non-disease control	1	EC	81	male	3.4	0.0390064	0.513866	nuclear RNA		IGF112648_filtered_feature_bc_matrix.tar.gz	IGF112648_HISEQ4000_cellranger_count_HG38_20191005.cram	IGF112648_HISEQ4000_cellranger_count_HG38_20191005.cram.crai
IGF112649	A319/11 EC	Human post-mortem cortex	Homo sapiens	Non-disease control	1	EC	74	male	4.6	0.0386487	1.38531	nuclear RNA		IGF112649_filtered_feature_bc_matrix.tar.gz	IGF112649_HISEQ4000_cellranger_count_HG38_20191006.cram	IGF112649_HISEQ4000_cellranger_count_HG38_20191006.cram.crai
IGF112650	A277/12 SSC	Human post-mortem cortex	Homo sapiens	AD	6	SSC	79	male	7.7	1.70910729	3.65359	nuclear RNA		IGF112650_filtered_feature_bc_matrix.tar.gz	IGF112650_HISEQ4000_cellranger_count_HG38_20191006.cram	IGF112650_HISEQ4000_cellranger_count_HG38_20191006.cram.crai
IGF112651	A277/12 EC	Human post-mortem cortex	Homo sapiens	AD	6	EC	79	male	6.3	3.790097878	1.94794	nuclear RNA		IGF112651_filtered_feature_bc_matrix.tar.gz	IGF112651_HISEQ4000_cellranger_count_HG38_20191006.cram	IGF112651_HISEQ4000_cellranger_count_HG38_20191006.cram.crai
IGF112652	A319/11 SSC	Human post-mortem cortex	Homo sapiens	Non-disease control	1	SSC	74	male	6.3	0	0.854616	nuclear RNA		IGF112652_filtered_feature_bc_matrix.tar.gz	IGF112652_HISEQ4000_cellranger_count_HG38_20191006.cram	IGF112652_HISEQ4000_cellranger_count_HG38_20191006.cram.crai
IGF112653	PDC016 EC	Human post-mortem cortex	Homo sapiens	Non-disease control	1	EC	91	female	6.6	0.861503	0.821825	nuclear RNA		IGF112653_filtered_feature_bc_matrix.tar.gz	IGF112653_HISEQ4000_cellranger_count_HG38_20191006.cram	IGF112653_HISEQ4000_cellranger_count_HG38_20191006.cram.crai
IGF112654	PDC016 SSC	Human post-mortem cortex	Homo sapiens	Non-disease control	1	SSC	91	female	7.9	0.0628615	0.226991	nuclear RNA		IGF112654_filtered_feature_bc_matrix.tar.gz	IGF112654_HISEQ4000_cellranger_count_HG38_20191007.cram	IGF112654_HISEQ4000_cellranger_count_HG38_20191007.cram.crai
IGF112655	PDC026 EC	Human post-mortem cortex	Homo sapiens	Non-disease control	1	EC	80	female	4.1	0.457716	0.454358	nuclear RNA		IGF112655_filtered_feature_bc_matrix.tar.gz	IGF112655_HISEQ4000_cellranger_count_HG38_20191007.cram	IGF112655_HISEQ4000_cellranger_count_HG38_20191007.cram.crai
IGF112656	PDC026 SSC	Human post-mortem cortex	Homo sapiens	Non-disease control	1	SSC	80	female	5.3	0.040833	0.059688	nuclear RNA		IGF112656_filtered_feature_bc_matrix.tar.gz	IGF112656_HISEQ4000_cellranger_count_HG38_20191006.cram	IGF112656_HISEQ4000_cellranger_count_HG38_20191006.cram.crai

PROTOCOLS

Protocols applicable to only a subset of Samples can be included as additional columns of the SAMPLES section above instead.

growth protocol	
treatment protocol	Post-mortem delay < 24hr. Grey matter isolated (white matter removed).
**extract protocol**	Nuclei were isolated from fresh frozen cortical grey matter from AD and control brains using a protocol based on Krishnaswami et al. (2016). Single-nuclei capture was performed using 10X Genomics Single Cell Gene Expression kit v3
**library construction protocol**	10X Genomics Single Cell 3' Gene Expression kit v3
**library strategy**	RNASeq

DATA PROCESSING PIPELINE

Data processing steps include base-calling, alignment, filtering, peak-calling, generation of normalized abundance measurements etc…
For each step provide a description, as well as software name, version, parameters, if applicable.

**data processing step**	Illumina sequencing files were aligned to the genomic sequence (introns and exons) using GRCh38 (gencode v30) annotation in Cell Ranger v3.0.2
**data processing step**	
**data processing step**	
**data processing step**	
**data processing step**	
**genome build**	GRCh38_gencode_v30_pre_mRNA
**processed data files format and content**	Filtered Feature-Barcode Matrix Generated by Cell Ranger Version 3.0.2

For each file listed in the "processed data file" columns of the SAMPLES section, provide additional information below.

PROCESSED DATA FILES

file name	file type	file checksum
IGF112633_filtered_feature_bc_matrix.tar.gz	Filtered Feature-Barcode Matrix	b35d911a38dc0e6672f5ac8a473380e8
IGF112634_filtered_feature_bc_matrix.tar.gz	Filtered Feature-Barcode Matrix	599cedd5ef0ee7f8be12197d29531f3b
IGF112635_filtered_feature_bc_matrix.tar.gz	Filtered Feature-Barcode Matrix	9fba50752cd33d720fabf4edda1ba239
IGF112636_filtered_feature_bc_matrix.tar.gz	Filtered Feature-Barcode Matrix	384934f548d8f2e03c06428352f4e41b
IGF112637_filtered_feature_bc_matrix.tar.gz	Filtered Feature-Barcode Matrix	d9ec65da48a3e1b7453088ee8d451953
IGF112638_filtered_feature_bc_matrix.tar.gz	Filtered Feature-Barcode Matrix	72f8af50b8b4740e3bbe75d15c4d7bf8
IGF112639_filtered_feature_bc_matrix.tar.gz	Filtered Feature-Barcode Matrix	a3fef005d400f62856e2dc808fff1b7c
IGF112640_filtered_feature_bc_matrix.tar.gz	Filtered Feature-Barcode Matrix	1ec6363a59216345da0b09f76bf3d711
IGF112641_filtered_feature_bc_matrix.tar.gz	Filtered Feature-Barcode Matrix	254416ef1988a21805613491a965fda1
IGF112642_filtered_feature_bc_matrix.tar.gz	Filtered Feature-Barcode Matrix	1d3fe01e27afd744f7b0e2690c33da76
IGF112643_filtered_feature_bc_matrix.tar.gz	Filtered Feature-Barcode Matrix	de5ce73954597a6b65f39fc2e748429c
IGF112644_filtered_feature_bc_matrix.tar.gz	Filtered Feature-Barcode Matrix	e71237f84af7608947b459e4682df3cb
IGF112645_filtered_feature_bc_matrix.tar.gz	Filtered Feature-Barcode Matrix	5a861608ac1d7c24e5742f55b2114a8b
IGF112646_filtered_feature_bc_matrix.tar.gz	Filtered Feature-Barcode Matrix	5bdad11d2af57d8736c0ea2e5d86914e
IGF112647_filtered_feature_bc_matrix.tar.gz	Filtered Feature-Barcode Matrix	ecea0cab89a31355097ec4d67626a348
IGF112648_filtered_feature_bc_matrix.tar.gz	Filtered Feature-Barcode Matrix	722f6750ef535c3d82d9b00ba698a8c7
IGF112649_filtered_feature_bc_matrix.tar.gz	Filtered Feature-Barcode Matrix	161d484a1d3366ea5b33439bb6db2cf9
IGF112650_filtered_feature_bc_matrix.tar.gz	Filtered Feature-Barcode Matrix	32d8f85ef047695dad956d7d3469e29d
IGF112651_filtered_feature_bc_matrix.tar.gz	Filtered Feature-Barcode Matrix	398d8aac663ed614339bdd25d1923de9
IGF112652_filtered_feature_bc_matrix.tar.gz	Filtered Feature-Barcode Matrix	00d4ae2d47cf8b1cdf9b16e0f56c1e18
IGF112653_filtered_feature_bc_matrix.tar.gz	Filtered Feature-Barcode Matrix	39b0a7e9cfff09fceefabc529463c0b0
IGF112654_filtered_feature_bc_matrix.tar.gz	Filtered Feature-Barcode Matrix	823f0071e1159f9794dd6ea5ecabbf44
IGF112655_filtered_feature_bc_matrix.tar.gz	Filtered Feature-Barcode Matrix	1247f0d86bb7aec37bde145388942fe4
IGF112656_filtered_feature_bc_matrix.tar.gz	Filtered Feature-Barcode Matrix	2f206664b6a8404e5f3bf7232c3dd25e

instrument m	single or paired-end

For each file listed in the "raw file" columns of the SAMPLES section above, provide additional information below.

RAW FILES

file name	file type	file checksum
IGF112633_HISEQ4000_cellranger_count_HG38_20191005.cram.crai	CRAM Index	54c4f7e807105736232ddb9f6161ec18
IGF112633_HISEQ4000_cellranger_count_HG38_20191005.cram	CRAM	d76a3170020793080dfd682a650f2057
IGF112634_HISEQ4000_cellranger_count_HG38_20191006.cram.crai	CRAM Index	bd7b1777b4da58e12d6fffd589f9f44d
IGF112634_HISEQ4000_cellranger_count_HG38_20191006.cram	CRAM	407cfc57e92c3efaa799601e09497a1d
IGF112635_HISEQ4000_cellranger_count_HG38_20191005.cram.crai	CRAM Index	43754c9efede4ea3629840b9c769e86c
IGF112635_HISEQ4000_cellranger_count_HG38_20191005.cram	CRAM	a5989b72f529a55aa42aaa2a46ac7e1b
IGF112636_HISEQ4000_cellranger_count_HG38_20191005.cram.crai	CRAM Index	a8e0fdd6d107af17ff9837ba3bf73a65
IGF112636_HISEQ4000_cellranger_count_HG38_20191005.cram	CRAM	d40ed4d5bc4e06aeb3d26712956406a1
IGF112637_HISEQ4000_cellranger_count_HG38_20191005.cram.crai	CRAM Index	2999ce416c4973024496eb3ae7fded6e
IGF112637_HISEQ4000_cellranger_count_HG38_20191005.cram	CRAM	df870ccb13932c1fdaad951bd9076cb3
IGF112638_HISEQ4000_cellranger_count_HG38_20191006.cram.crai	CRAM Index	848c41591aa2fe5bc89996a99cb64bd3
IGF112638_HISEQ4000_cellranger_count_HG38_20191006.cram	CRAM	bb5783249ce07d8852537f6fc1d9343c
IGF112639_HISEQ4000_cellranger_count_HG38_20191005.cram.crai	CRAM Index	5139fccc6d36e7498f74e790314eace8
IGF112639_HISEQ4000_cellranger_count_HG38_20191005.cram	CRAM	e47fda0fc4b6094baf7a7e94c6f9f956
IGF112640_HISEQ4000_cellranger_count_HG38_20191005.cram.crai	CRAM Index	e94ccc2d728d0fa8b593ddda15f935bb
IGF112640_HISEQ4000_cellranger_count_HG38_20191005.cram	CRAM	3bb70f26ccd9dfe30eb49ef33f83f5b6
IGF112641_HISEQ4000_cellranger_count_HG38_20191006.cram.crai	CRAM Index	6d443cf9735d85c279af900d9bb4755c
IGF112641_HISEQ4000_cellranger_count_HG38_20191006.cram	CRAM	69e8f790f9351b0500ff6982ee0ab756
IGF112642_HISEQ4000_cellranger_count_HG38_20191006.cram.crai	CRAM Index	5d3d667609e403033ca54a55fae64e5d
IGF112642_HISEQ4000_cellranger_count_HG38_20191006.cram	CRAM	92b4082083b5daa24c6c941fef647fb3
IGF112643_HISEQ4000_cellranger_count_HG38_20191006.cram.crai	CRAM Index	c13bb12d4263be9ff002adaa3b685617
IGF112643_HISEQ4000_cellranger_count_HG38_20191006.cram	CRAM	13ddfd34971abf42aee09ccf4deadefb
IGF112644_HISEQ4000_cellranger_count_HG38_20191005.cram.crai	CRAM Index	6bf6995464607937f3aa13ecda5894d6
IGF112644_HISEQ4000_cellranger_count_HG38_20191005.cram	CRAM	138cd001d12bb7e638a4cb67e57764d9
IGF112645_HISEQ4000_cellranger_count_HG38_20191005.cram.crai	CRAM Index	c0afd77bbc77a43cec5b5e5e64aac87a
IGF112645_HISEQ4000_cellranger_count_HG38_20191005.cram	CRAM	708018b8f31f9258f59e5e85a87911be
IGF112646_HISEQ4000_cellranger_count_HG38_20191005.cram.crai	CRAM Index	1d57bf78d37338e275e032364b5b8385
IGF112646_HISEQ4000_cellranger_count_HG38_20191005.cram	CRAM	997640676951152b8d00ed4e2c439b61
IGF112647_HISEQ4000_cellranger_count_HG38_20191005.cram.crai	CRAM Index	d0ca2858f02190f43fb0892663e9b34e
IGF112647_HISEQ4000_cellranger_count_HG38_20191005.cram	CRAM	52a3c996a2c0814f8963e3248e176154
IGF112648_HISEQ4000_cellranger_count_HG38_20191005.cram.crai	CRAM Index	5377a3daf571f20425753b9eb847c7e8
IGF112648_HISEQ4000_cellranger_count_HG38_20191005.cram	CRAM	8957da89969403fc962aedd6830db57b
IGF112649_HISEQ4000_cellranger_count_HG38_20191006.cram.crai	CRAM Index	64d3f2e28ad7b977792770bd4b5f4f81
IGF112649_HISEQ4000_cellranger_count_HG38_20191006.cram	CRAM	576568f86e7c4708d7dd4e063314f5db
IGF112650_HISEQ4000_cellranger_count_HG38_20191006.cram.crai	CRAM Index	bbd993fced81f531189c0817aba8089a
IGF112650_HISEQ4000_cellranger_count_HG38_20191006.cram	CRAM	500f2b6fe7837518c7129bafa354bbe5
IGF112651_HISEQ4000_cellranger_count_HG38_20191006.cram.crai	CRAM Index	942abd2a5650bb907e4702e09ea10e42
IGF112651_HISEQ4000_cellranger_count_HG38_20191006.cram	CRAM	b17647269d80519937a5aace30038179
IGF112652_HISEQ4000_cellranger_count_HG38_20191006.cram.crai	CRAM Index	747f74e492713223a68e27ee6afd3ae8
IGF112652_HISEQ4000_cellranger_count_HG38_20191006.cram	CRAM	6c34cada7b4a6435a4f95b7d91f5129e
IGF112653_HISEQ4000_cellranger_count_HG38_20191006.cram.crai	CRAM Index	11aac98d97461fb46afa2ae23bea80a8
IGF112653_HISEQ4000_cellranger_count_HG38_20191006.cram	CRAM	d22628ff598867f5f8e4cb730de9b5c3
IGF112654_HISEQ4000_cellranger_count_HG38_20191007.cram.crai	CRAM Index	2b2295093aa5c331938161a2f93069ec
IGF112654_HISEQ4000_cellranger_count_HG38_20191007.cram	CRAM	ec05edeee38c59d85b25f18d826840e1
IGF112655_HISEQ4000_cellranger_count_HG38_20191007.cram.crai	CRAM Index	ba01c035a32c8b5e71078daffdadb9b9
IGF112655_HISEQ4000_cellranger_count_HG38_20191007.cram	CRAM	0298c82cccfacf136c1f90f591bf68af
IGF112656_HISEQ4000_cellranger_count_HG38_20191006.cram.crai	CRAM Index	b276a786c548e5b17c77279621460eea
IGF112656_HISEQ4000_cellranger_count_HG38_20191006.cram	CRAM	1c10e4a5e428cbd7cd7eac536a8c3e6f

For paired-end experiments, list the 2 (or more) fastq files from the same run. For SOLiD experiments, list the 4 file names (include "file name 3" and "file name 4" columns).

PAIRED-END EXPERIMENTS

file name 1	file name 2

Supplementary Table 1_Sample data and sequencing template
EXAMPLE 1

# High-throughput sequencing metadata template.
# All fields in this template must be completed.
# See METADATA EXAMPLES in tabs at the foot of this page.
# Field names (in blue on this page) should not be edited. Hover over cells containing field names to view field content guidelines.
# Human data. If there are patient privacy concerns regarding making data fully public through GEO, please submit to NCBI's dbGaP (http://www.ncbi.nlm.nih.gov/gap/) database. dbGaP has controlled access mechanisms and is an appropriate resource for hosting sensitive patient data.

SERIES

This section describes the overall study. Complete all fields.

**title**	Genome-wide maps of chromatin state in pluripotent and lineage-committed cells.
**summary**	We report the application of single-molecule-based sequencing technology for high-throughput profiling of histone modifications in mammalian cells. By obtaining over four billion bases of sequence from chromatin immunoprecipitated DNA, we generated genome-wide chromatin-state maps of mouse embryonic stem cells, neural progenitor cells and embryonic fibroblasts. We find that lysine 4 and lysine 27 trimethylation effectively discriminates genes that are expressed, poised for expression, or stably repressed, and therefore reflect cell state and lineage potential. Lysine 36 trimethylation marks primary coding and non-coding transcripts, facilitating gene annotation. Trimethylation of lysine 9 and lysine 20 is detected at satellite, telomeric and active long-terminal repeats, and can spread into proximal unique sequences. Lysine 4 and lysine 9 trimethylation marks imprinting control regions. Finally, we show that chromatin state can be read in an allele-specific manner by using single nucleotide polymorphisms. This study provides a framework for the application of comprehensive chromatin profiling towards characterization of diverse mammalian cell populations.
**overall design**	Examination of 2 different histone modifications in Neural progenitor cells.
**contributor**	John,B,Goode
**contributor**	Bradley,Smith
supplementary file	

SAMPLES

Thoroughly describe each of the biological Samples under investigation. **Tissue/cell type/cell line** information is required.
# Include all biological and experimental variables in characteristics columns (for example, characteristics: tissue, characteristics: cell type, characteristics: treatment, characteristics: genotype, characteristics: disease state or any clinical variables, etc...)
# -> -> KEEP SCROLLING TO THE RIGHT FOR IMPORTANT FIELDS; RAW AND PROCESSED FILES MUST BE LISTED IN THIS SECTION -> ->

**Sample name**	**title**	**source name**	**organism**	**characteristics: cell type**	**characteristics: strain**	**characteristics: ChIP antibody**	**characteristics: treatment**	**characteristics: cell line**	**molecule**	**description**	**processed data file**	**processed data file**	**raw file**	**raw file**
Sample 1	Neural progenitor cells - H3K4me2_rep1	Neural progenitor cells	Mus musculus	ES-derived neural progenitor cells	C57BL/6	H3K4me2 (Millipore, 07-030, lot 12:	untreated		genomic DNA		H3K4me2.peaks_rep1.txt	H3K4me2_rep1.wig	080716_BI-EAS46_0001_209DH_L1.fastq.gz	
Sample 2	Neural progenitor cells - H3K4me2_rep2	Neural progenitor cells	Mus musculus	ES-derived neural progenitor cells	C57BL/6	H3K4me2 (Millipore, 07-030, lot 12:	untreated		genomic DNA		H3K4me2.peaks_rep2.txt	H3K4me2_rep2.wig	080716_BI-EAS46_0001_209DH_L4.fastq.gz	
Sample 3	Neural progenitor cells - input DNA	Neural progenitor cells	Mus musculus	ES-derived neural progenitor cells	C57BL/6	none	untreated		genomic DNA			H3K4me2_input.wig	080717_BI-EAS46_0001_20DH_L5.fastq.gz	
Sample 4	Neural progenitor cells - H3K4me2 - ABX-treated	Neural progenitor cells	Mus musculus	ES-derived neural progenitor cells	C57BL/6	H3K4me2 (Millipore, 07-030, lot 12:	treated with ABX for 24 hours		genomic DNA		H3K4me2.peaks_ABX.txt	H3K4me2_rABX.wig	080716_BI-EAS46_0001_209DH_L6.fastq.gz	

PROTOCOLS

Protocols applicable to only a subset of Samples can be included as additional columns of the SAMPLES section above instead.

growth protocol	ES cell–derived NS cells were routinely generated by re-plating d 7 adherent neural differentiation cultures (typically 2–3 × 106 cells into a T75 flask) on uncoated plastic in NS-A medium (Euroclone, Milan, Italy) supplemented with modified N2 and 10 ng/ml of both EGF and FGF-2 (NS expansion medium).
treatment protocol	One replicate was ABX treated for 24 hours.
**extract protocol**	Lysates were clarified from sonicated nuclei and histone-DNA complexes were isolated with antibody.
**library construction protocol**	Libraries were prepared according to Illumina's instructions accompanying the DNA Sample Kit (Part# 0801-0303). Briefly, DNA was end-repaired using a combination of T4 DNA polymerase, E. coli DNA Pol I large fragment (Klenow polymerase) and T4 polynucleotide kinase. The blunt, phosphorylated ends were treated with Klenow fragment (32 to 52 exo minus) and dATP to yield a protruding 3- 'A' base for ligation of Illumina's adapters which have a single 'T' base overhang at the 3' end. After adapter ligation DNA was PCR amplified with Illumina primers for 15 cycles and library fragments of ~250 bp (insert plus adaptor and PCR primer sequences) were band isolated from an agarose gel. The purified DNA was captured on an Illumina flow cell for cluster generation. Libraries were sequenced on the Illumina HiSeq 2500 following the manufacturer's protocols.
**library strategy**	ChIP-Seq

DATA PROCESSING PIPELINE

Data processing steps include base-calling, alignment, filtering, peak-calling, generation of normalized abundance measurements etc…
For each step provide a description, as well as software name, version, parameters, if applicable.

**data processing step**	Basecalls performed using CASAVA version 1.4
**data processing step**	ChIP-seq reads were aligned to the mm9 genome assembly using EasyAlign version 3.2 with the following configurations…
**data processing step**	Data were filtered using the following specifications…
**data processing step**	peaks were called using PeaksFind version 2.2 with the following setting: ChIP threshold (0.2), Enrichment Fold (2.5), Rescue Fold (3).
**data processing step**	
**genome build**	mm9
**processed data files format and content**	wig files were generated using …; Scores represent …
**processed data files format and content**	peak text files

For each file listed in the "processed data file" columns of the SAMPLES section, provide additional information below.

PROCESSED DATA FILES

**file name**	**file type**	file checksum
H3K4me2.peaks_rep1.txt	peak files	95cf1d1fa509d871b2ef0bb9fd734c3d
H3K4me2.peaks_rep2.txt	peak files	8ec6ee3cce10b970e5bfea4e35cdb231
H3K4me2.peaks_ABX.txt	peak files	f8fcd650914ff1a733956d6d06e8b543
H3K4me2_rep1.wig	wig	604fbb658413c559511eb6ad2bb1472e
H3K4me2_rep2.wig	wig	57cf1d1fa509d871b2ef0bb9fd734c3c
H3K4me2_input.wig	wig	8ec6ee3cce10b970e5bfea4e35cdb231
H3K4me2_rABX.wig	wig	f8fcd650914ff1a733956d6d06e8b543

For each file listed in the "raw file" columns of the SAMPLES section, provide additional information below.

RAW FILES

**file name**	**file type**	file checksum	**instrument model**	**single or paired-end**
080716_BI-EAS46_0001_209DH_L1.fastq.gz	fastq	6cc6ee3cce10b970e5bfea4e35cdb	Illumina HiSeq 2500	single
080716_BI-EAS46_0001_209DH_L4.fastq.gz	fastq	88ceb0e0d056dda9208a03acf9073	Illumina HiSeq 2500	single
080717_BI-EAS46_0001_20DH_L5.fastq.gz	fastq	f2786fedc5106789a2af4014a0e74f(	Illumina HiSeq 2500	single
080716_BI-EAS46_0001_209DH_L6.fastq.gz	fastq	9a2af4014a0e74f00f2786fedc5106	Illumina HiSeq 2500	single

For paired-end experiments, list the 2 (or more) fastq files from the same run. For SOLiD experiments, list the 4 file names (include "file name 3" and "file name 4" columns).

PAIRED-END EXPERIMENTS

**file name 1**	**file name 2**

Supplementary Table 1_Sample data and sequencing template
EXAMPLE 2

# High-throughput sequencing metadata template.
# All fields in this template must be completed.
# See METADATA EXAMPLES in tabs at the foot of this page.
# Field names (in blue on this page) should not be edited. Hover over cells containing field names to view field content guidelines.
# Human data. If there are patient privacy concerns regarding making data fully public through GEO, please submit to NCBI's dbGaP (http://www.ncbi.nlm.nih.gov/gap/) database. dbGaP has controlled access mechanisms and is an appropriate resource for hosting sensitive patient data.

SERIES
This section describes the overall study. Complete all fields.

**title**	Next Generation Sequencing Facilitates Quantitative Analysis of Wild Type and Nrl-/- Retinal Transcriptomes
**summary**	Purpose: Next-generation sequencing (NGS) has revolutionized systems-based analysis of cellular pathways. The goals of this study are to compare NGS-derived retinal transcriptome profiling (RNA-seq) to microarray and quantitative reverse transcription polymerase chain reaction (qRT–PCR) methods and to evaluate protocols for optimal high-throughput data analysis
**summary**	Methods: Retinal mRNA profiles of 21-day-old wild-type (WT) and neural retina leucine zipper knockout (Nrl−/−) mice were generated by deep sequencing, in triplicate, using Illumina GAIIx. The sequence reads that passed quality filters were analyzed at the transcript isoform level with two methods: Burrows–Wheeler Aligner (BWA) followed by ANOVA (ANOVA) and TopHat followed by Cufflinks. qRT–PCR validation was performed using TaqMan and SYBR Green assays
**summary**	Results: Using an optimized data analysis workflow, we mapped about 30 million sequence reads per sample to the mouse genome (build mm9) and identified 16,014 transcripts in the retinas of WT and Nrl−/− mice with BWA workflow and 34,115 transcripts with TopHat workflow. RNA-seq data confirmed stable expression of 25 known housekeeping genes, and 12 of these were validated with qRT–PCR. RNA-seq data had a linear relationship with qRT–PCR for more than four orders of magnitude and a goodness of fit (R2) of 0.8798. Approximately 10% of the transcripts showed differential expression between the WT and Nrl−/− retina, with a fold change ≥1.5 and p value <0.05. Altered expression of 25 genes was confirmed with qRT–PCR, demonstrating the high degree of sensitivity of the RNA-seq method. Hierarchical clustering of differentially expressed genes uncovered several as yet uncharacterized genes that may contribute to retinal function. Data analysis with BWA and TopHat workflows revealed a significant overlap yet provided complementary insights in transcriptome profiling.
**summary**	Conclusions: Our study represents the first detailed analysis of retinal transcriptomes, with biologic replicates, generated by RNA-seq technology. The optimized data analysis workflows reported here should provide a framework for comparative investigations of expression profiles. Our results show that NGS offers a comprehensive and more accurate quantitative and qualitative evaluation of mRNA content within a cell or tissue. We conclude that RNA-seq based transcriptome characterization would expedite genetic network analyses and permit the dissection of complex biologic functions.
**overall design**	Retinal mRNA profiles of 21-day old wild type (WT) and Nrl-/- mice
**contributor**	Rebecca,A,Smith
**contributor**	David,Doe
supplementary file	Raw_gene_counts_matrix.txt

SAMPLES
Thoroughly describe each of the biological Samples under investigation. **Tissue/cell type/cell line** information is required.
# Include all biological and experimental variables in characteristics columns (for example, characteristics: tissue, characteristics: cell type, characteristics: treatment, characteristics: genotype, characteristics: disease state or any clinical variables, etc...)
# -> -> KEEP SCROLLING TO THE RIGHT FOR IMPORTANT FIELDS; RAW AND PROCESSED FILES MUST BE LISTED IN THIS SECTION -> ->

Sample name	title	source name	organism	characteristics: strain	characteristics: tissue	characteristics: age	characteristics: genotype	characteristics: cell line	characteristics: treatmen	molecule	description	processed data file	raw file	raw file
Sample 1	Retina WT rep1	Retina	Mus musculus	C57BL/6	retina	post natal day 21	wild type			total RNA		WT1.txt	080716 BI-EAS46 0001 2	080716_BI-EAS46_0001_209DH_L1_R1.fastq.gz
Sample 2	Retina WT rep2	Retina	Mus musculus	C57BL/6	retina	post natal day 21	wild type			total RNA		WT2.txt	080716 BI-EAS46 0001 2	080716_BI-EAS46_0001_209DH_L2_R1.fastq.gz
Sample 3	Retina Nrl-KO rep1	Retina	Mus musculus	C57BL/6	retina	post natal day 21	Nrl-/-			total RNA		mutant1.txt	080717 BI-EAS46 1.fastq.	080717_BI-EAS46_2.fastq.gz
Sample 4	Retina Nrl-KO rep2	Retina	Mus musculus	C57BL/6	retina	post natal day 21	Nrl-/-			total RNA		mutant2.txt	080718 BI-EAS49 1.fastq.	080718_BI-EAS49_2.fastq.gz

PROTOCOLS
Protocols applicable to only a subset of Samples can be included as additional columns of the SAMPLES section above instead.

growth protocol	
treatment protocol	
**extract protocol**	Retinas were removed, flash frozen on dry ice, and RNA was harvested using Trizol reagent. Illumina TruSeq RNA Sample Prep Kit (Cat#FC-122-1001) was used with 1 ug of total RNA for the construction of sequencing libraries.
**library construction protocol**	RNA libraries were prepared for sequencing using standard Illumina protocols
**library strategy**	RNA-Seq

DATA PROCESSING PIPELINE
Data processing steps include base-calling, alignment, filtering, peak-calling, generation of normalized abundance measurements etc…
For each step provide a description, as well as software name, version, parameters, if applicable.

**data processing step**	Illumina Casava1.7 software used for basecalling.
**data processing step**	Sequenced reads were trimmed for adaptor sequence, and masked for low-complexity or low-quality sequence, then mapped to mm8 whole genome using bowtie v0.12.2 with parameters -q -p 4 -e 100 -y -a -m 10 --best --strata
**data processing step**	Reads Per Kilobase of exon per Megabase of library size (RPKM) were calculated using a protocol from Chepelev et al., Nucleic Acids Research, 2009. In short, exons from all isoforms of a gene were merged to create one meta-transcript. The number of reads falling in the exons of this meta-transcript were counted and normalized by the size of the meta-transcript and by the size of the library.
**data processing step**	
**data processing step**	
**genome build**	mm9
**processed data files format and content**	tab-delimited text files include FPKM values for each Sample ...
**processed data files format and content**	Matrix table with raw gene counts for every gene and every sample

For each file listed in the "processed data file" columns of the SAMPLES section, provide additional information below.
PROCESSED DATA FILES

file name	file type	file checksum
WT.txt	abundance measurements	d8fcd650914ff1a733956d6d06e8b091
WT2.txt	abundance measurements	abcdef123456789abc123456789abc
mutant1.txt	abundance measurements	95cf1d1fa509d871b2ef0bb9fd734c3d
mutant2.txt	abundance measurements	0wd6ee3cce10b970e5bfea4e35cdb987
Raw_gene_counts_matrix.txt	raw counts	57cf1d1fa509d871b2ef0bb9fd734c3c

For each file listed in the "raw file" columns of the SAMPLES section, provide additional information below.
RAW FILES

file name	file type	file checksum	instrument model	single or paired-end
080716 BI-EAS46 0001 209DH L1 R1.fast	fastq	6cc6ee3cce10b970e5bfea4	Illumina HiSeq 2000	paired-end
080716 BI-EAS46 0001 209DH L1 R2.fast	fastq	88ceb0e0d056dda9208a03	Illumina HiSeq 2000	paired-end
080716 BI-EAS46 0001 209DH L2 R1.fast	fastq	f2786fedc5106789a2af401	Illumina HiSeq 2000	paired-end
080716 BI-EAS46 0001 209DH L2 R2.fast	fastq	d8fcd650914ff1a733956d6	Illumina HiSeq 2000	paired-end
080717_BI-EAS46_1.fastq.gz	fastq	03839cca2e797b28b9f937	Illumina HiSeq 2000	paired-end
080717_BI-EAS46_2.fastq.gz	fastq	604fbb658413c559511eb6	Illumina HiSeq 2000	paired-end
080718_BI-EAS49_1.fastq.gz	fastq	57cf1d1fa509d871b2ef0bb	Illumina HiSeq 2000	paired-end
080718_BI-EAS49_2.fastq.gz	fastq	e5718e1a97690d410464f2	Illumina HiSeq 2000	paired-end

For paired-end experiments, list the 2 (or more) fastq files from the same run. For SOLiD experiments, list the 4 file names (include "file name 3" and "file name 4" columns).
PAIRED-END EXPERIMENTS

file name 1	file name 2
080716 BI-EAS46 0001 209DH L1 R1.fast	080716_BI-EAS46_0001_209DH_L1_R1.fastq.gz
080716 BI-EAS46 0001 209DH L2 R1.fast	080716_BI-EAS46_0001_209DH_L2_R1.fastq.gz
080717_BI-EAS46_1.fastq.gz	080717_BI-EAS46_2.fastq.gz
080718_BI-EAS49_1.fastq.gz	080718_BI-EAS49_2.fastq.gz

Supplementary Table 2_AUCell gene set enrichment
Contents

Sheet name	Description
Gene sets from literature	Gene sets used in the analyses
AUCell enrichment	Enrichment of literature gene lists in microglia clusters
Ext Data Fig 7 AUC values	Raw AUC enrichment values for Ext Data Fig 7

Analyses were performed using AUCell (R package v1.6.1)

Supplementary Table 2_AUCell gene set enrichment
Gene sets from literature

Core Microglial Genes
Galatro et al 2017 DOI: 10.1038/nn.4597
1289 upregulated genes

TNF
PIK3R5
MS4A14
ITGAL
ARRDC5
CIITA
PIK3R6
RHBDF2
CSF3R
ITGAX
PARVG
FGD2
RASAL3
PLVAP
CD300LF
IL1B
CD37
BLNK
XIRP1
HSPA6
LILRB2
LILRB1
CTD-2270F17.1
NLRC4
CD300A
FCGR1B
CD33
IRF8
ABI3
LPAR5
CYTH4
ITGAM
KLHL6
VENTX
SIGLEC7
ADGRE4P
TRAF3IP3
TREML1
VAV1
SRGAP2-AS1
PLCB2
RUNX1
DOCK2
SIGLEC9
PRAM1
BIN2
SELPLG
TMC8
IL18
FCGR1A
ITGB2-AS1
SIRPB2
CLEC5A
RASGRP4
MYO1F
MIR223
ALOX5
ARHGAP45
ADGRG5
RP11-153M7.3
HLA-DMB
CSF1R
TYMP
APBB1IP
CARD11
MILR1
CYTIP
DOCK8
SLC11A1
S100Z
FCGR2A
TLR2
SPN
LAIR1
SLC2A5
ADAM28
LINC01480
SH3TC1
SERPINA1
LILRB4
C16orf54
RBM47
APOBR
SIGLEC11
SIGLEC8
MIR142
FCAR
MYO1G
CCR1
TAGAP
FPR1
TNFRSF13C
SLC1A5
CSF2RA
LRRC25
CCR5
SPI1
TAL1
DEF6
CLEC7A
ARL11
IL10RA
AP000476.1
TBXAS1
CAPNS2
CXorf21
RHOH
DENND1C
INPP5D
IKZF1
ELF4
LAT2
SCIMP
CMTM7
PTPN6
LILRA4
RGS1
NCF1
FYB
BTK
ITGB2
STAB1
TGFB1
HCLS1
PTAFR
GPR132
P2RY13
CH17-118O6.3
IL1A
VSIG4
CASP1
FAM159A
TMEM106A
FCGR3A
CD84
LYL1
NCF4
LILRA1
AC018755.18
TNFSF8
FCGR3B
CSF2RB
HCK
AOAH-IT1
LINC01268
PTPRC
CCL3
SLC37A2
CD86
HIST2H3C
B3GNT5
HIST2H3A
GPR84
WAS
TMEM156
SASH3
MIR503HG
TLR7
SIGLEC5
ARHGAP15
RP11-483P21.2
TCIRG1
SYK
LILRA2
C3
SLC16A3
NCF1B
IFI30
MS4A7
STXBP2
SNX20
MEP1A
SIGLEC10
GAB3
LAIR2
AOAH
SLC7A7
PARP15
FGD3
SCIN
SUCNR1
MS4A6A
NCKAP1L
PLCG2
SAMSN1
ARHGAP30
EBI3
CX3CR1
PIK3AP1
PLD4
TREM2
NCF1C
KCNQ1
FCGBP
SLC17A9
C3AR1
RIN3
SIGLEC14
SLC16A5
RP11-365O16.3
HAMP
AC011899.9
RIPK3
COL8A2
PROX2
PTPN7
SLCO2B1
CMKLR1
ACPP
LST1
IRF5
CPED1
LGALS9
CASP8
OLFML3
P2RX1
HK3
NFAM1
TNFRSF1B
TRIM38
CYBB
PCED1B-AS1
CD180
CD74
TLR6
CLECL1
CYBA
MORC1
MPEG1
CEACAM1
ITGB3
MMRN1
NCF2
CD68
CLEC9A
TLR10
TMEM119
PLEK
TMIGD3
CD300C
HAAO
CCDC88B
NRROS
GAPT
CD69
LIPG
BCL2A1
FGL2
TOMA
MRC1
TLR5
GPR65
CH25H
ALOX5AP
CEACAM21
IL16
OSM
TYROBP
FERMT3
LPAR6
IL10
LMNTD2
ADAP2
CD14
NFATC2
GPR183
DHRS9
CCL3L3
UBA7
MSR1
RP11-552D4.1
GPR34
HK2
MLKL
LYVE1
CISH
TLR1
DISC1-IT1
PTGS1
PLAC8
FOLR2
MNDA
ATP2A3
ZMYND15
DAPP1
DLEU7-AS1
RNASET2
SPNS3
FCGR2B
HIST1H3I
C1QB
HLA-DMA
PALD1
LINC00528
TMIGD2
C1QC
PDCD1LG2
CARD9
HAVCR2
SOWAHD
MYBPH
KYNU
LY86
RGS18
DPEP2
PTGER4
CPVL
L3MBTL4-AS1
SLC15A3
ZFP36
IGSF6
SPINT1
HPGDS
CLEC17A
TNFRSF10C
ACY3
LCP1
LILRB3
RGS10
HHEX
SYTL3
AC002480.3
TNFRSF10D
SAP25
GMIP
RP11-137H2.4
ADRB2
AATBC
SIPA1
BNC2
C5AR1
CPA6
OLR1
CD4
FCER1G
C19orf38
RNASE6
CYTL1
KCNMB1
HLX
LINC01468
TFEC
GAL3ST4
HCG22
NLRP6
RPGRIP1
SLA
ARHGAP25
SP100
ARHGAP27
UCP2
PGM5P2
LINC01091
C9orf66
NLRP4
LINC01624
UNC93B1
ADCY7
RP11-344B5.2
TMEM236
TRIM34
GHRL
TMEM150B
SLAMF8
CD53
LAPTM5
[illegible]

Aging Genes
Olah et al 2018 DOI: 10.1038/s41467-018-02926-5
LogFC >0.25
padj <0.005
1604 upregulated genes

RPL36A
RPS28
RPS27
HLA-DRB1
MYO15B
RPL18A
APOE
HLA-DPB1
C9orf142
RPL9
SH3TC1
ARHGAP27
CTSD
RPS15
C19orf60
NEDD4
HLA-A
HLA-C
HMHA1
ARHGAP4
C1QA
CCS
C1QC
GAA
CD14
WASH5P
ATG16L2
HLA-F
AP1G2
CYBA
TCEB2
CORO1A
CYP27A1
ARAP1
LTBR
PSENEN
TNFRSF14
CXXC1
TRABD
NUDT14
MAN2C1
RHOT2
POLR2J3
TRAPPC5
C17orf62
CPNE1
HIST1H2AC
NAPSB
SIPA1
NDUFB7
PIEZO1
PLD4
RPL13
ARSA
TRIM27
SPSB3
TCIRG1
CORO7
PFKL
RPL10
ATP5D
ARHGEF1
TYK2
GRN
C1QB
MRPL55
C10orf10
COPE
MT1X
HLA-B
ARF5
TRMT2A
CIITA
CDK2AP2
PNPLA6
TMEM182
TSPO
NDUFV1
HIST2H2BF
QTRT1
FAM212A
SNHG9
NT5C
RPS19
RNF166
RPL23A
ARL6IP4
C9orf114
GUK1
NCF1B
IGFLR1
SIVA1
POR
TMC6
NLRC5
UBXN11
PLEKHM1P
SCNM1
LRCH4
SLC38A10
HLA-DQA1
TREX1
DNAJC4
RCN3
TAP1
B3GAT3
HSPB1
MTG1
LSP1
RGS12
TMEM175
GUSBP3
NDUFA11
FPGS
ATF6B
PGLS
FLOT1
SLC17A9
EIF3CL
TSC2
RPS18
MUTYH
PRMT7
HLA-DMB
HIST3H2A
NINJ1
RPL17
STAB1
STXBP2
CD37
NDUFA3
ANKZF1
ATP13A1
RPL27A
DDAH2
ROBO3
ABHD16A
POLR2E
HOOK2
FAM160B2
LRPAP1
RIC3
CDK10
RPL29
DDX11
TBRG4
GRAMD1A
ADRBK1
GSTP1
STX10
PLTP
IRF7
MCM5
ALKBH7
CENPT
ATG4B
MYO1F
ZNF692
HAMP
PRAM1
MAP2K2
HLA-DPA1
MSH5
HCG18
ATP5G2
HLA-DQB1
ACADVL
AIP
UQCRC1
ALDOA
EEF1D
RNPEPL1
NAT9
SERF2
VPS28
TIMM13
CPSF3L
ADAM15
APBB3
CAPN1
MAN2B1
CCDC130
SDF2L1
MRPL38
OTUB1
RPL36
UBA7
NCF1
NDUFS8
WASH3P
ORC6
LST1
KIAA1328
CLASRP
AP1M1
COMT
PLXND1
RBCK1
NOL12
HPS1
RPS7
PCBP1-AS1
FMNL1
RPS9
SDHAF1
ZBTB16
ARFGAP1
RABAC1
CPSF1
PLEKHM1
WDR46
PSMB8
ATHL1
RPSA
PLA2G4B
GADD45GIP1
DDX39B
HIST1H2AE
NDUFA13
SLC16A3
JUNB
STAG3L3
BAG6
MIF
CCDC57
ARL17A
NCAPH2
SGSH
SHKBP1
THOC6
MAGOH
PLOD3
RAB4B
MED24
JPX
TUBGCP6
RABGGTA
ABCC10
TRPM2
SIL1
GTF3C5
DENND3
CCM2
CHID1
NUDT22
YIF1A
RHBDF2
ENTPD6
C10orf54
FLII
PSMB9
REEP4
PLXNB2
RPL8
HMG20B
TMEM120A
NR1H2
SYNGR2
TTLL4
LENG8
RNH1
CALHM2
MCOLN1
WDPCP
MAP3K11
SLC39A13
VARS
TPTEP1
PIN1
RPL28
HLA-DMA
BCS1L
DHPS
GALE
BANP
CSNK2B
RPUSD3
VAMP5
LSM2
ALG3
MRPS18B
GAS6
SUGP1
MRPS34
HLA-E
UNC45A
STRA13
FAM193B
RECQL5
CDC37
VPS52
KIAA0195
GALT
DPM2
C9orf37
FLAD1
PYCARD
RPL12
DPH1
LSM7
TBCD
C20orf27
ING4
U2AF1L4
HIST1H2BJ
EIF4EBP1
ANAPC11
IFRD2
FAM3A
AGER
RPS26
DHX30
NUCB1
TWF2
TIMM44
TUBGCP2
POLR2J4
DHX16
KCNN4
LMF2
MRI1
BCKDHA
ANKMY1
NCF1C
NEIL1
RPL32P3
SHISA9
PLEKHJ1
NCOR2
BRAT1
PLA2G15
ENGASE
POLR2L
LMAN2
PHKG2
POLL
C6orf1
HDAC5
XRCC3
PLD3
UBXN6
TNFSF13
SFI1
SLC26A6
TCF25
EML3
PRRC2A
STAG3L1
SNX20
TECR
PACS2
TOP3B
ADRM1
AARSD1
TKT
CSF3R
AUP1
COMMD6
APRT
RPS21
JAK3
UBXN1
EXOSC5
LIG1
CD82
ZBTB48
[illegible]

DAM (Disease Associated Microglia)
Keren-Shaul et al 2017 DOI: 10.1016/j.cell.2017.05.018
1803 upregulated genes

ABCA2
ABCA3
ABCB6
ABCB8
ABCD2
ABCF1
ABHD10
ABHD11
ABHD12
ABHD16A
ABI1
ACACA
ACADL
ACADSB
ACADVL
ACD
ACER3
ACHE
ACOX3
ACSL5
ACTN4
ACY1
ADAM9
ADAMTS1
ADAR
ADARB1
ADCK1
ADCK5
ADIPOR1
ADIPOR2
ADO
AGA
AGL
AGPAT2
AGPAT5
AGTRAP
AHDC1
AIG1
AIMP1
AIP
AKAP8
AKAP8L
AKR1E2
AKR7A2
AKT1
AKT3
ALDH16A1
ALDOA
ALG1
ALG10
ALG10B
ALG1L
ALG1L2
ALPK1
ALS2
AMACR
AMBRA1
AMMECR1L
AMZ1
ANAPC5
ANGPTL7
ANKH
ANKIB1
ANKRA2
ANKRD12
ANKRD16
ANKRD40
ANXA3
ANXA4
AP2S1
AP3D1
AP3M1
AP4B1
APAF1
APBB2
APBB3
APEX1
APLP2
APOE
APRT
ARAF
ARAP1
ARAP2
ARF3
ARF5
ARFGEF2
ARHGAP26
ARHGDIA
ARHGEF7
ARID4B
ARIH1
ARL11
ARL15
ARL2BP
ARL3
ARL5A
ARL6IP5
ARMC10
ARMCX3
ARNT
ARPC1A
ARPC1B
ARPC2
ARPC3
ARPC5
ARPC5L
ARPP19
ARRDC2
ARRDC4
AS3MT
ASAP1
ASB10
ASB7
ASCC3
ASL
ASXL1
ATAD2B
ATAD3A
ATF3
ATF7
ATG2B
ATG4A
ATG5
ATG7
ATN1
ATOX1
ATP11C
ATP13A1
ATP1B3
ATP5F1A
ATP5F1B
ATP5F1C
ATP5F1D
ATP5F1E
ATP5ME
ATP5MF
ATP5PD
ATP5PF
ATP5PO
ATP6AP1
ATP6V0B
ATP6V0C
ATP6V0D1
ATP6V0E1
ATP6V1A
ATP6V1C1
ATP6V1E1
ATP6V1F
ATP6V1G1
ATP6V1H
ATP7A
ATP8B2
ATPAF2
ATRN
ATXN10
AURKAIP1
AXL
AZIN1
B2M
B3GALNT1
B3GAT3
BACH1
BAG1
BAIAP2L2
BANF1
BBIP1
BCAP31
BCDIN3D
BCKDK
BCL2
BCL2A1
BCORL1
BECN1
BIN3
BIRC5
BLOC1S1
BLOC1S2
BLVRA
BOLA2
BOLA2B
BPGM
BRCC3
BSCL2
BSG
BST2
BTAF1
BTBD3
BTBD7
C11orf96
C12orf29
C12orf57
C16orf70
C16orf87
C19orf53
C1orf131
C1orf198
C1orf216
C1orf43
C1orf54
C1QBP
C20orf144
C2CD3
C2orf42
C3AR1
C5AR1
C5orf15
C5orf51
C5orf63
C6orf226
C8orf76
C9orf64
CACYBP
CADM1
CALM1
CALM3
CALR
CAMP
CANT1
CAPG
CAPN3
CAPN7
CAPNS1
CARD16
CARD17
CARM1
CASP1
CAST
CASZ1
CBR4
CBX6
CBX7
CCAR1
CCDC107
CCDC126
CCDC34
CCDC59
CCDC85B
CCDC86
CCDC88A
CCL15
CCL2
CCL23
CCL3
CCL3L1
CCL3L3
CCL4
CCL4L1
CCL4L2
CCND3
CCNL1
CCNY
CCRL2
CCT2
CCT4
CCT5
CD14
CD151
CD180
CD22
CD300LD
CD34
CD37
CD48
CD52
CD63
CD74
CD83
CD84
CD9
CD99L2
CDC16
CDC42BPA
CDC42EP3
CDCA8
CDIPT
CDK10
CDK19
CDK2AP2
CDK4
CDK6
CDKN1B
CDKN2AIP
CEBPA
CENPB
CENPC
CENPE
CETN2
CFL1
CH25H
CHCHD1
CHD8
CHIC1
CHKB
CHMP2A
CHMP2B
CHMP3
CHMP4B
CHMP5
CHMP6
CHN2
CHORDC1
CHPF2
CHPT1
CHST1
CHST11
CHST12
CIB2
CIC
CIRBP
CKAP5
CLASRP
CLCC1
CLCN3
CLCN7
CLEC7A
CLIC1
CLINT1
CLIP1
CLIP2
CLN3
CLN5
CLN6
CLN8
CLPTM1L
CLTA
CLU
CMPK1
CMTM3
CNDP2
CNIH2
CNNM2
CNOT4
CNOT7
CNPY2
CNRIP1
COL4A1
COMMD3
COPA
COPE
COPS2
COPS3
COPS6
COPZ1
COQ5
CORO1C
CORO2A
COTL1
COX17
COX18
COX4I1
COX5A
COX6A1
COX6A2
COX6B1
COX6C
COX7A2
COX7A2L
COX7B
COX7C
COX8A
CPD
CPEB2
CPNE3
CPSF2
CPSF6
CRAT
[illegible]

ARM (Activated Response Microglia)
Sala Frigerio et al 2019 DOI: 10.1016/j.celrep.2019.03.099
LogFC >0.25
padj <0.005
254 upregulated genes

APOE
CST7
SPP1
LPL
CD74
CLEC7A
HLA-DQB1
LYZ
HLA-DQA1
ANKH
CCL4
AXL
LILRB4
HLA-DRB5
CCL3L3
CCL23
ITGAX
CD83
LGALS3BP
CD63
HLA-A
ATP1A3
CTSB
HIF1A
MYO1E
CAPG
IGF1
ANXA5
PDCD1
APLP2
CD9
CTSV
PLD3
PSAT1
NCEH1
FTH1
CTSZ
CH25H
CSF1
CD52
TLR2
TPI1
PKM
RAMP1
SERPINE2
ALDOA
CADM1
CTSD
PLAUR
TYROBP
B2M
FAM20C
HCAR2
ATF3
PLEK
NPC2
DPP7
EEF1A1
GUSB
MYO5A
LDHA
ST8SIA6
HCAR3
GAPDH
CXCL16
OR5V1
FABP5
LY9
HEXA
CD34
C3AR1
APBB2
SCPEP1
FTL
SLC11A1
ADSS1
GCNT2
TREM2
PEA15
LRPAP1
FXYD5
GPR84
MIF
LAG3
CPQ
TNFSF8
SOAT1
EPHX1
MILR1
SDF4
SLAMF9
SLC2A1
TSPO
SIGLEC8
IFI27L2
GPI
ARHGAP24
CTSA
ST14
GPR65
CD14
DGKZ
RTCB
RPLP0
NRP1
RPS5
RPS14
FAU
CD48
TPD52
CSF2RA
PLAU
RPL18A
CREG1
PRDX1
SLC16A3
GLB1
PLIN2
IFI16
IFITM3
RPL4
ENO1
RPL21
SYNGR1
TPM4
NAGLU
AKR1A1
LSP1
RPL13
P2RX4
PLPP1
PFKL
RPS4Y1
HLA-G
UAP1L1
RPL23
GM2A
RPLP1
COX6A2
GOT1
CRLF2
KLHL6
LY86
RPL10A
RPS3
SULF2
ASPH
KCNJ2
GNG12
DNAJB14
EEF1B2
KIAA1217
NPM1
PRDX4
HLA-E
RPL3
ATP6V1C1
SDC3
PFKFB3
ANXA4
RPS19
GAA
MT1E
HACD2
ABCD2
GLMP
TMEM176A
TMED3
DEGS1
PMP22
NDUFA1
RPL9
RPS2
ATP6V1B2
PLEKHM2
CSTB
PLEKHH2
COX4I1
LAMP1
CCL2
RENBP
RPS23
RPL19
CYB5R1
RPS24
RPS6
ACACA
RPL6
RPS27A
ATP6V1A
EEF2
HMOX1
GLTP
LAT2
SDF2L1
LAMP2
FUCA1
PLEKHA1
RPS20
MYO1F
BAIAP2L2
ECHS1
ATP1B3
TIMP2
RPL10
ATXN10
COLGALT1
ARL11
CNDP2
RACK1
MSMO1
PTPRC
RPS16
RPL14
CTSS
FGF13
SSR4
RPL11
SCARB1
GGH
GRN
EIF3F
SP140
GBA
UCP2
PLBD2
RPL12
GLIPR1
LIPA
RPL8
PPT1
SRGN
PLEKHB2
PLXDC2
VPS13C
CRIP1
OAS1
BST2
CTSE
SAMSN1
RPS28
CORO1C
SDCBP
HLA-DQB2
MT1A
IFITM2
IFITM1
CCL4L1
SIGLEC6
LOC112694756
CCL4L2
CCL3L1
CCL15
CCL3

IRM (Interferon Response Microglia)
Sala Frigerio et al 2019 DOI: 10.1016/j.celrep.2019.03.099
LogFC >0.25
padj <0.005
139 upregulated genes

LACC1
CD83
APLP2
SLAMF9
RPLP0
CNP
DPP7
GPR84
ACER3
CD9
PFKFB3
ANXA5
HLA-G
PIK3AP1
CASP4
CD63
MAP3K8
HCK
IL15RA
PTTG1
TMEM184B
CAPG
CXCL16
RNASE6
RNF114
NPC2
TLR3
CTSC
SLAMF8
TRIM5
TRAFD1
NMI
MILR1
APOBEC1
PTTG2
C19orf12
PSMB8
LGALS13
IRF1
CTSB
IRGM
APOBEC3B
CCDC86
DTX3L
SLC25A22
CTSZ
TMEM106A
PSMB9
UBA7
IRF9
ETNK1
GRN
ZC3HAV1
PSME2
LYZ
SAMHD1
LGALS16
PARP10
TSPO
LGALS9B
TAP1
CTSS
APOE
FTH1
PSME1
SP110
DCK
SHISA5
ZNFX1
NAMPT
FCGR1B
OGFR
EPSTI1
FCGR1A
FCGR3A
CD52
CSF1
FCGR3B
CLEC7A
PDCD1
CD74
PARP12
TRIM14
TRIM34
CST7
HLA-A
IFI35
IFIH1
HLA-E
PML
TRIM25
TRIM6-TRIM34
UBE2L6
GBP6
DDX58
TOR3A
SLFN13
PARP9
HERC6
ADAR
EIF2AK2
DDX60
SDC3
CCL2
PARP14
B2M
LY6E
BST2
DHX58
ISG20
FGL2
RNF213
PHF11
AXL
STAT2
LGALS3BP
SAMD9L
MX1
STAT1
NLRC5
SLFN12L
XAF1
SLFN12
SP140
IFITM2
SLFN5
IFIT2
IFIT3
IFITM3
RTP4
IRF7
IFITM1
IFI16
USP18
IFIT1B
IFI27L2
OAS1
OAS2
ZBP1

PIG (Plaque Induced Genes)
Chen et al 2020 DOI: 10.1016/j.cell.2020.06.038
56 upregulated genes

HLA-A
B2M
C4B
C4A
GFAP
SERPINA3
APOE
AXL
CD63
CD9
CTSB
CTSD
CTSV
CTSZ
HEXA
LGALS3BP
LYZ
NPC2
TREM2
TYROBP
ARPC1B
C1QA
C1QB
C1QC
CLU
CSF1R
CST3
CTSA
CTSS
CTSH
CX3CR1
CYBA
FCER1G
FCGR2A
FCRL2
GRN
GUSB
GNS
GPX4
HEXB
IGFBP5
ITGB5
ITM2B
LAPTM5
LGMN
LY86
MAN2B1
MPEG1
OLFML3
PLEK
PRDX6
FCRL3
S100A6
RPL18A
VSIR
FCGR2C

PLK3	STK19	DERL1
LINC00477	CACNA2D4	DGCR2
IKBKE	ZNF76	DGKD
TIMD4	COQ9	DGKQ
ERP27	UROD	DGKZ
LCP2	MUM1	DGUOK
PLAUR	FAM45A	DHCR7
CCRL2	IMP4	DHFR
HIST1H2AM	CHCHD5	DHRS3
HLA-DOA	SLC25A45	DHRS7
PYCARD	SLC25A6	DHRS7B
LTBR	BAX	DHX32
PLBD1	HLA-DRA	DHX40
SP140L	ORAOV1	DHX9
ALPK1	NEURL4	DIS3
REEP4	RPLP1	DIS3L2
TNFAIP3	SIN3B	DKC1
CYP2S1	RXRB	DLD
IFI16	ATP9B	DNAJB12
CYSLTR1	CAPG	DNAJB14
PCDH12	CLN3	DNAJC1
ODF3B	ILVBL	DNAJC13
IL1RN	BST2	DNAJC15
FPR2	PABPC1L	DNAJC5
LINC00954	EBI3	DNASE2
RGS16	PGS1	DNHD1
RP11-480C22.1	MAN1B1	DNMT3A
MMP14	LYPLA2	DOCK1
ITPRIP	TOM1	DOK1
PGGHG	PPOX	DPCD
LINC00996	CFD	DPH2
LYZ	EGLN2	DPM3
MB21D1	RPS2	DPP7
HMOX1	FAM73B	DPY19L1
MX2	CDK11A	DPY19L4
CD300LB	NDUFC2	DRAP1
FAM105A	TAPBP	DSTN
AIF1	STARD3	DTD1
ATP8B4	MSTO1	DTNBP1
MYO7A	GPI	DTYMK
CASS4	LMF1	DUSP12
SPTLC3	C9orf89	DUSP16
TIFAB	RNF40	DUSP3
P2RY8	TBC1D17	DYNC1LI2
IL17RA	ALG1	DYNLT3
RREB1	CISD3	DZIP3
FHOD1	ELMOD3	E2F6
B4GALT1	ABTB1	EBAG9
LY75	PWP2	EBPL
RNASE3	RPL7A	ECHS1
P2RY6	MCRS1	ECI2
LHFPL2	NAA10	EDEM2
OSCAR	EIF3G	EDF1
RCN3	CLPP	EEF1A1
NABP1	MPST	EEF1B2
HCST	MAP4K1	EEF1D
BTN2A3P	CERCAM	EEF1G
CTSS	SCYL1	EEF2
MIR3687-1	TMEM91	EFHD2
MSLN	DEF6	EFR3A
NFKBID	C5orf56	EGLN1
NLRC5	EGFL8	EGR1
CFD	TBCB	EGR2
NOD2	UQCR11	EID1
CD93	ASL	EIF1AX
HIST1H2BO	FGD2	EIF1AY
SPP1	METTL22	EIF2A
MIR378E	BAIAP2	EIF2AK2
MIR3153	ABHD14A	EIF2S1
CXCR2	STRN4	EIF2S3
FXYD5	ZNF677	EIF3A
RPS6KA1	FBXW4	EIF3B
LSP1	ENG	EIF3E
TMEM71	MICAL1	EIF3F
STEAP3	ARHGEF10L	EIF3H
SLC18A1	NMT1	EIF3I
UNC13D	NFIC	EIF3K
POU2F2	POLR2J	EIF3M
CD58	MED15	EIF4A1
TMEM102	PLB1	EIF4B
TMEM173	C12orf10	EIF4EBP1
RGS19	HINFP	EIF4EBP3
C1QA	BCKDK	EIF4ENIF1
DENND3	POLD2	EIF4H
HOXC4	BCAP31	EIF5
GIMAP4	WDR81	EIF5A
TMEM255B	ASCC2	EIF5AL1
ST6GAL1	PPP6R1	EIF5B
HIST1H2BH	CES2	ELAC1
IRF1	MRPL28	ELK3
MIR3648-1	ARID5A	ELL2
MIR3690	GPR108	ELMOD2
TSPAN32	RPS3	ELOVL5
LPCAT2	MLXIPL	EMP3
ITGB7	POU2F2	ENDOG
ABCC4	EDF1	ENPP1
CD99P1	NBPF9	ENPP2
SP140	CYTH4	EPM2A
FMNL3	NSUN5	EPRS1
RNU5F-1	ZNF467	EPS15
RP11-834C11.4	TUBG2	ERGIC3
MIR3687-2	TAPBPL	ERO1B
ARHGAP9	PSMB10	ERP29
GRAPL	MYL6B	ESD
FCN1	ENO3	EVI2A
STK10	TPT1-AS1	EXT1
ABCB4	C19orf10	FAF2
IL21R	MTX1	FAM120A
PATL2	TMEM219	FAM122A
CASP5	ACCS	FAM122B
MS4A4E	ACAA1	FAM13A
TNFRSF10B	SCAP	FAM174A
GIMAP8	HMBS	FAM193A
C10orf11	SH2D3C	FAM20C
MIR646HG	RAB11B	FAM3A
HLA-DRB5	ARFIP2	FAM3C
TBC1D10C	NUMA1	FAM50A
BMF	FDX1L	FAM53A
RP11-693J15.5	MOV10	FAM76A
PADI1	HEMK1	FAM98C
GIPC3	TUFM	FANCC
MFNG	POLR2J2	FANCE
ASCL2	ABCD4	FASTKD2
BATF	TNK2	FAT3
HPSE	ARHGAP9	FAU
PRKCD	DUS1L	FBXO22
SIGLEC12	AKNA	FBXW8
ARHGAP24	UQCRQ	FCER1G
BTBD19	LPCAT4	FCGR2A
IRAK3	TIMM17B	FCGR2B
HIST1H1B	GNB2	FCGR2C
APOC1P1	DEAF1	FCGR3A
TLR3	IRF9	FCGR3B
HLA-DPB1	RAD23A	FEM1A
FPR3	EEFSEC	FEN1
HIST1H2AI	TMEM109	FGD4
VNN2	DNASE1L1	FGGY
RNF135	C11orf31	FGL2
MIR4496	FLOT2	FKBP2
APOBEC3G	DNAJC17	FKBP8
CIDEB	FCGR3B	FMC1-LUC7L2
HCAR2	PHPT1	FMO5
RAB3IL1	GPS2	FNDC3A
AC079586.1	USP20	FNDC3B
RYR1	GBGT1	FOS
CD163	UCKL1	FRMD8
CCDC26	MIPOL1	FRRS1
PLAU	DOK3	FTH1
RUNX3	ARFGAP2	FTL
FBP1	AURKAIP1	FTO
IGSF10	CTC1	FUBP3
OXER1	BSG	FUCA1
LTC4S	CSK	FUCA2
HIST1H2AG	MRPL34	FUNDC2
APOBEC3C	HRH2	FUS
PYGL	IDH3G	FXYD5
MIR3143	DCXR	FXYD6
FCGRT	LRRC37A2	FYN
LY75-CD302	LGALS9	G6PC3
PPP1R18	RUVBL2	G6PD
MICB	GNRHR2	GAA
CCL8	LMBR1L	GABARAP
MIR3648-2	PQBP1	GALC
ZFP36L2	ATP6V0E2	GALK1
AGER	TRAF3IP3	GALNT3
RGL3	NHP2	GAMT
RUNX2	DHRSX	GANAB
CTSC	SNX8	GARRE1
CACNA2D4	RPL7	GARS1
LPAR2	TMEM150A	GBA
ZBP1	RPL32	GBE1
ENTPD1	SYMPK	GBP6
HIST1H4L	EPN2	GCC2
NCMAP	ZFPL1	GCH1
MYD88	C12orf57	GCSH
DDX60L	FAM127B	GDE1
MIR4420	DDX54	GDI2
EPSTI1	IDH2	GET3
CD40	AP2A1	GFER
IL12RB1	FERMT3	GFM1
C11orf21	KCTD13	GFUS
MCM5	GUSBP11	GGA3
MIR24-2	CCDC159	GGH
CEBPB	SPI1	GHITM
ARHGAP22	CTSH	GINS4
DOK3	TMCO6	GKAP1
SP110	MGRN1	GLB1
IL6R	RHBDD2	GLB1L
APOBEC3F	DOK1	GLIPR1
RP11-41O4.1	DMAP1	GLOD4
AKNA	ANKRD52	GLRX3
CCDC73	FAM96B	GLRX5
ZFP36L1	NCKAP5L	GLTP
CDKN1A	IRF3	GLUD1
SLFN12	EEF1G	GLUD2
ACSM5	IVD	GM2A
OAS1	LAMTOR2	GMPPA
CMTM3	PCIF1	GMPS
TRPC2	STK25	GNAQ
DISC1	MMS19	GNAS
HLA-DQA1	ETFB	GNG12
FLI1	LAIR1	GNG5
ADAMTSL4	MKS1	GNPDA2
ZC3H12A	CHCHD6	GNPTAB
C2	ITGB2	GNS
SOCS1	PNPLA2	GOLGA2
ZNF217	DHX58	GOLGA3
MMP8	ASMTL	GOLM2
TRPV4	NUP85	GOLT1B
ZC3H12D	TEX264	GON4L
ANXA9	CHMP1A	GORASP2
HIST1H2AH	FKBP2	GOT1
IRF7	TAX1BP3	GPATCH8
FOSL1	TRPT1	GPCPD1
DENND2D	PLD2	GPD2
IL13RA1	PRKCSH	GPI
C12orf75	HDAC6	GPNMB
SNAI3	SEMA4C	GPR180
RCSD1	SMUG1	GPR65
MIR6774	POLM	GPR84
RP11-231D20.2	ARPC1B	GPS2
STX11	ZMIZ2	GRAMD2B
LINC00264	SNRPA	GRHPR
SPATC1	PMPCA	GRINA
SLC2A9	LILRA2	GRN
KCNE3	CERS4	GSN
TMEM154	PCSK7	GTF2A2
LINC00243	PIGG	GTF2IRD1
THEMIS2	ATG7	GTF3C4
ARHGDIB	TXNDC17	GTPBP6
TGFBR2	MXD4	GUF1
RP11-8L8.2	SLC12A7	GUSB
RNVU1-15	COX17	GYS1
SLFN11	C11orf49	H1-0
OR52K2	C19orf53	H2AJ
RP11-1149O23.3	WRAP73	HADH
CDCP1	SYNGR1	HADHB
JAK3	ZNF385A	HAGHL
CD300E	SCAMP3	HARBI1
ZEB2-AS1	EHMT1	HARS2
AC073072.5	MKL1	HAVCR2
BEAN1-AS1	MGMT	HCFC1R1
RP11-317P15.4	CCDC22	HDAC11
FZD2	ATP6V0B	HDAC2
ACRBP	RGS19	HEATR3
ZFHX3	TMEM223	HERC6
KCNK13	POLR2F	HERPUD2
FAM3B	P4HB	HEXA
RAB20	SLC12A9	HIF1A
CXCR4	PTOV1	HINT1
MAN2B1	LSM4	HIPK1
MAP3K8	MDP1	HLA-A
AC226118.1	RPL21P28	HLA-DMA
SERPINE1	P2RX4	HLA-DOB
LINC01126	WDR27	HLA-DQA1
RNF122	RASSF4	HLA-DQB1
FGF20	GCDH	HLA-DQB2
FAM60A	FCGRT	HLA-DRB5
C19orf35	TUBB	HLA-E
PILRA	MARS	HLA-G
PTGIR	PMF1BP1	HMG20A
HLA-DQB1	LEPRE1	HMGXB4
GBP5	GAMT	HNRNPUL1
TNFRSF10A	BCAT2	HOMER1
MIR7848	SUN2	HPRT1
GSN-AS1	PIK3R6	HPS5
CECR1	AAAS	HSD17B10
AC008697.1	ERICH1	HSD17B12
GPR160	CROCC	HSD17B4
LINC01356	FAM131A	HSF1
GNLY	CDK18	HSP90B1
BHLHE41	SORBS3	HSPA9
SELL	PIH1D1	HSPBP1
C2orf91	ZNF706	HSPE1
C10orf54	SLC2A11	HTRA2
MIR4451	HEXA	HUS1
C5AR2	ZFYVE19	HVCN1
ILDR1	DDR1	HYI
SAMD9L	SNHG12	ICMT
TGFBR1	ZYX	ID2
FAM177B	TRIM3	IDH1
CASP10	HERC2P3	IDH3A
GIMAP2	PSMC3	IER3IP1
GCNT2	CNPY3	IFI16
EDA2R	COQ4	IFI27L2
ANKRD44-IT1	ARHGAP24	IFIT1B
PCED1B	GNPTG	IFIT2
LINC01426	WDR45	IFIT3
RP11-66N24.3	NUDCD3	IFNAR1
DOK1	NDUFC1	IFNAR2
SNORA55	RIPK3	IFRD1
FAM72A	CLK3	IFT52
TMCO4	SLC25A37	IGF1
SRGAP2	PLCD1	IGSF8
SNORD15A	NSL1	IKBIP
RUBCNL	RNF216P1	IKBKG
OLFML2B	ATG10	IL13RA1
MAF	SPCS2	IL1B
SRGN	CDK11B	IL4I1
GIMAP1	C1orf35	IL4R
PTCRA	SPINT1	IL6ST
GOLGA8H	UBA52	ILDR2
SNORD62A	DPP9	ILKAP
CEBPA	SPN	IMMP1L
CARD8	RPS27A	IMMT
GYPC	PPP1R18	IMPACT
ADPRH	GSN	INCENP
PIEZO1	HDAC4	INO80B
CHIT1	MAD2L2	INO80C
NFATC1	TNFRSF1A	INPP5A
NFKB2	UPP1	INPP5K
TEP1	DBNL	INTS1
PIGR	RRP1	INTS3
CYP26C1	PUS1	IPCEF1
HIST2H2BF	RGL3	IQGAP2
ICAM1	C1orf63	IRAG2
TMPRSS4-AS1	BATF3	IRAK1
FFAR2	TOLLIP	IRF7
SIRPD	MYO19	IRF8
PARP14	TEP1	ISCU
PSTPIP1	AGPAT1	ISG20
EFCAB13	BSDC1	ISG20L2
LINC01598	METTL21A	ISOC1
PRICKLE3	G6PC3	ISY1-RAB43
TMEM52B	CEP164	ISYNA1
IL3RA	CRTAP	ITFG1
FAM26F	KLHDC3	ITGA5
PGM5	MRPL23	ITGAX
SNORD14E	IL15RA	ITGB1BP1
NUP62CL	RIN3	ITGB2
RP1-111D6.4	DNPEP	ITGB5
SLED1	HSF1	ITM2B
TRIM6	FAM72A	ITM2C
MMP2	DHRS1	ITPKB
LINC01504	DDA1	ITPR1
RNF166	FAM133B	JAGN1
TGIF2	TRAP1	JMY
CARD16	UQCRB	JOSD1
EFNA4	LRP1	JTB
CARD6	NR1H3	KARS1
BATF3	PTPN6	KCNJ2
IL4I1	NPRL3	KDM4A
MIR6753	MGAT1	KEAP1
ITPRIPL2	TAF6	KHDRBS1
NXF3	NAGPA	KHSRP
GCSAM	STEAP3	KIAA0100
ULK4P1	AGPAT6	KIAA1217
SAMD9	IL15	KIDINS220
PPP3CB-AS1	ITIH4	KIF16B
CEBPE	CLDN15	KIF5A
GBGT1	CAMTA2	KIFAP3
RP11-356I2.4	KLC4	KITLG
ARHGEF1	GSTK1	KLHDC10
NKILA	ELOVL1	KLHL36
ASCL4	MICB	KLHL5
SH3BP2	TRPV2	KLHL6
A2M	SNX29P2	KPNA3
LTF	WIBG	KRIT1
LINC00278	NISCH	LACTB2
ABCC13	PSMC3IP	LAG3
FAM72B	TRIM69	LAMB2
SYT6	TMEM180	LAMP1
GIMAP5	TADA3	LAMP2
MS4A6E	FAM65A	LAP3
ENPP7	SLC29A1	LARP4B
SNORA65	SLC39A7	LARS1
MIR616	CD74	LAT2
PSMB8-AS1	MRPL45P2	LBX2
PIK3CD-AS1	KAT2A	LDHA
LILRB5	MAGED2	LEPROTL1
TTC7A	RHOC	LGALS3
FAM111A	SMARCB1	LGALS3BP
STAG3	DHX38	LGI2
FOS	EEF1B2	LHFPL2
NFKBIZ	LY6G5B	LIAS
ZNF321P	MAP4K2	LILRA4
PMAIP1	ZNF90	LILRA5
KCNJ5	SLC35C2	LILRA6
SNORD60	DYRK4	LILRB3
IKBIP	BTBD19	LILRB4
SLC43A3	TLE3	LILRB5
ARPC1B	TBC1D9B	LIMA1
RNU6-7	RPL3	LIMD1
NAGA	PORCN	LIN54
CYFIP1	CTBP1	LIN9
MKNK1	SLC25A35	LIPA
MIR5690	ROGDI	LITAF
S100A9	IGSF21	LMBRD1
SNORD26	TMEM8A	LMBRD2
TBX19	MEF2BNB	LMO2
GNGT2	SH3D19	LNPEP
AC068499.10	ALOX5	LOC102725035
IFNGR1	CEACAM1	LOC107987425
ESRRB	SGTA	LOC107987462
HIST2H2AA4	DNAJA3	LOC112694756
MLXIPL	SLC37A1	LPL
RP11-705C15.2	PPIE	LRPAP1
HIST1H2BI	MUS81	LRRC14
KLHDC7B	MECR	LRRC39
FUOM	C4orf36	LRRC57
ARID3A	NAPA	LRRC58
SNORD27	PSD4	LRRC59
GSDMD	C16orf58	LRRC8D
AC156455.1	DHRS3	LRRCC1
CD5L	ZNF324	LSP1
VAMP8	FIBP	LUC7L2
MTHFD2	NCKIPSD	LY86
SNORD62B	GUSBP2	LYAR
PSD4	TMED9	LYRM2
MIR6087	LRSAM1	LYVE1
SRGAP2B	RASGRP2	LYZ
LINC01176	PPP2R1A	LYZL4
HIST2H2AA3	C2orf68	LZIC
CYP19A1	TSPAN15	LZTFL1
TNFAIP8	MFNG	LZTR1
GDPD3	AP2A2	M6PR
ARL5C	HLA-DOA	MAGED1
RNA5-8S5	CST3	MAMDC2
MIR4802	GGA1	MAN1C1
RNA5-8S5	MED22	MAN2B1
MERTK	CABIN1	MANF
BRCA2	RHOG	MAP2K1
VASP	SLC25A11	MAP4K3
CDKN2A	SCAND1	MAPK3
RNA5-8S5	PRPF31	MAPRE2
PLXDC2	CIZ1	MBD2
ELF1	MS4A6A	MBLAC2
DAB2	COX5B	MBP
RNA5-8S5	MINOS1	MCEE
SERPINB9	PPP6R2	MCTP1
LINC00968	LILRB3	MDH2
APOC1	INPP5K	MEA1
FCMR	DNASE2	MEAF6
DTX2	ZNF250	MED11
COLGALT1	KCNK6	MED14
STAT5A	TAMM41	MED28

ENPP1
NUAK2
PLA2G4E-AS1
FLJ40194
RAB42
ATP8B3
RNVU1-14
FAM166B
PRDM1
HACD4
RNA5-8S5
RAB32
CCL2
RNA5-8S5
GNA15
PMEL
C9
LCN2
CFLAR-AS1
MEFV
HSPA1A
ZNF467
MARCO
RNVU1-19
PPP1R13L
NMI
IL1R2
RASGEF1C
TRG-AS1
MAML3
CLDN23
FAM87B
ST14
LINC00649
S100A8
NPL
HLA-DQB2
SNORD14D
SBNO2
CTSZ
FAM110A
SAT1
CLEC2D
MMP9
DCANP1
TNNI2
RPA4
MIR365B
SLC8B1
SLC12A7
C10orf128
C1orf162
RNU6-1
DDX43
FBXO47
ATG16L2
ENG
GOLGA8K
CHEK2
ZDHHC19
TRIM5
SLC29A3
CAPG
MOB3C
USP6
CTB-55O6.12
ACKR2
DSC2
LY96
ARAP1
CALCR
ERAP2
GIMAP1-GIMAP5
MIR6813
DOCK11
ZNF878
TNFRSF12A
ELK3
P2RX4
DUOX1
HLA-E
NAT1
FOXD2-AS1
LTBP2
LRP1-AS
GRAP
RP11-452F19.3
SCAMP2
CCL22
PPP1R3B
ACTL10
SNORD14C
CHRNE
PLEKHG6
HSPA1B
SQRDL
RP11-177H13.2
GBP2
SNORD99
NBEAL2
ALDH3B1
DUSP1
IL15RA
IL6
BCL2L12
TNFRSF1A
CCL4L2
CD7
NAIP
SH2B3
TREML2
SNORD57
FMNL1
DDB2
SPIB
CH507-154B10.2
CRACR2A
MIR34A
ARRB2
SENCR
PLB1
SFMBT2
FRRS1
SPATA13
SLFN13
RNA5-8S5
MIR5190
PIK3CD
C22orf34
RNA5-8S5
SNORD101
GMFG
ST8SIA4
KCTD12
AC025171.1
WNT5A
TGFBI
BTG2
GSTM2
FES
FAM212A
DUOX2
GIMAP6
LTB4R
STAC3
CEBPD
PDGFB
FAM72D
AP003774.4
SNORD45A
CTB-186G2.1
ABALON
MIR4489
DLGAP4-AS1
ANKRD44
C7orf77
SLC25A19
LILRA5
TMC6
ADGRG1
RNF213
PPP1R15A
HLA-B
IL23A
TNFAIP2
MIR6891
ZNF90
MICAL1
PTPN18
PDPN
MYC
AC002480.2
ELMO3
IRGM
SULT1C2
LRRC69
RP11-727F15.9
MYO9B
BIK
TRIB1
MIR4312
PLA2G4A
SNORD61
TMEM176B
ZNF710
PROCR
IL27
RNA5S9
MCEMP1
TRABD
SRGAP2C
CEP55
HCP5
CTSH
PTER
CH507-24F1.1
PRR15
HIST2H3D
GPR141
CD226
ADGRG3
DTX3L
LRG1
S100A12
MIR5194
FAM46A
CEACAM8
PARP12
MIR646
CD19
SNORD58A
PARP9
CARD8-AS1
C11orf45
SLC5A9
H1FNT
MTHFD1L
CNPY3
MSH5
LRCH4
CTD-2314G24.2
LRMP
ZNF816-ZNF321P
NOD1
SNORD34
LINC01353
CTLA4
MIR606
C10orf55
FAM72C
BAZ1A
RAB38
CYP27C1
MIR3653
HOXB3
AANAT
SMAD6
MIR17HG
SNHG12
IL18BP
MFSD7
F2RL1
ARHGEF40
RELT
EPHX3
KLHL33
GRN
ABHD15
GOLGA8J
EIF4EBP1
SNORD125
FGL1
JAML
MIR6797
BTN2A2
MAP7D3
RP11-160H22.3
FGR
SNORA24
CLIC1
SLC16A13
XAF1
ARID5A
KIFC1
HIST1H3H
MYO1A
MIR3161
RNU6-2
RP11-712B9.2
TRIM21
KCNN4
MIR7854
LINC00676
IL18RAP
CTB-60B18.10
BST2
SNORD48
HCAR3
HIST1H2AJ
CATIP
FCHSD1
OASL
GRHL3
HIST1H2BM
C17orf62
ARHGAP6
TMEM86A
OAS2
HIST1H2BF
MIR3183
AIM2
PCP2
RP11-417F21.1
CTD-3088G3.8
ARHGEF19
SERPINB1
SAG
FFAR3
CLEC2B
RP4-684O24.5
HIST1H2AK
PLSCR1
GBP3
SOCS6
PIM1
FAM78A
VASH1
MIR3922
GIMAP7
SAPCD1
PFKFB3
C15orf39
PABPC1L
SNORD83A
APOBEC3D
CD302
NINJ1
DLEC1
SNORA31
GSDMA
CDC42BPG
SLC23A3
ITPR2
TRIM50
LIPC
CDK2
LINC00977
MIS18BP1
SCARF1
GABRR2
TMEM176A
CTD-2006C1.2
HLA-F
ESR1
CTD-2521M24.13
OR52K1
DERL3
IFI44
LAMP3
GGTA1P
TP53I13
HIST2H2BC
PRKD3
CORO1A
PNP
TLE3
IKBKB
STAT6
HFE
MIR3190
MIR1976
WBSCR27
COTL1
CLEC12A
CSTA
CCR2
SECTM1
LINC00921
IQGAP2
PLXNB2
ADGRE3
ZNF469
TMEM51
CXCL8
SMAGP
VTRNA1-1
BMP2K
MIR941-2
HIST1H2AD
PRKCH
ANGPTL6
SERPINF2
CREB3L2
AC109333.10
DOK2
SHKBP1
DIP2A-IT1
CFB
PARP4
MIR619
SUSD6
LINC00324
CKLF
SLC12A9
MAP3K14
TMEM37
DUSP27
GPC2
STK32B
SNORA13
GPR82
SNORD32A
TP53I11
PARP10
SLC9A9
COQ2
FAM149A
ADPGK
PYCARD-AS1
RP11-563J2.2
BCL3
ASGR2
TRPV2
CCL20
ACP5
CXCL12
UPK3A
SNORA26
NEAT1
RP11-47I22.2
P3H2
TSGA10IP
ENGASE
APOC4
ATP11C
MYCL
CSK
HIST1H2BD
YTHDF3-AS1
CRISP3
LRRK1
ABCC6
MIR572
AXL
LINC00106
COL6A4P1
TREML4
IGFLR1
SYNGR2
RASSF1

NOTCH1
RPS6KA1
KRI1
NBPF1
C11orf24
MCM7
IKBKG
AKR7A2
ZNF335
HDAC7
LILRB1
COPS7B
ATG13
PARVG
NDUFAF3
CHFR
SPAG7
PI4KAP1
HAGH
OAZ1
IRF5
TMEM119
EIF3F
NDUFA10
PI4KAP2
RNASEH2C
SLC4A2
RPL13A
SUSD3
CCDC137
GPSM3
HCK
TREM2
NSFL1C
CTSA
ARHGAP22
RIMKLB
AKT1
SH3BGRL3
HARS2
LIMK1
TFPT
PTCD1
SNX29
TYROBP
WFS1
SURF6
PSMF1
RPS5
GUSBP1
TMUB2
TRIM11
CYB5R3
SIRPB2
FAM161B
BRD2
SGK494
NBPF15
SZT2
TXN2
IRAK1
PPP2R4
ZNF84
SPNS2
GYPC
CEPT1
SLC20A2
AAMP
GNB2L1
CCDC18
RNASEH2A
JUP
FUK
LILRB4
IDH3B
ZNF276
ABI3
C14orf159
CRAT
CLEC16A
POLR3E
RUSC2
NOP2
USF2
MRPS11
FAM160A2
BRD8
PILRB
NUP210
WASH2P
CCNDBP1
TMCO4
RBM42
FAHD2A
FAM127A
RPL39
KAT8
EIF2B4
CYTH2
CD68
RPS3A
IL16
CTSB
RPL34
FTL
USP21
AGAP4
TMSB4X
TMBIM4
TAF1D
FUS
HM13
ALPK1
RPL18
MPPE1
COX4I1
DDX56
DDX23
RPL6
NEU1
GFOD2
SNX17
RPS29
RMND5B
DTNB
NDUFS2
ATXN1
SUPT5H
RNF135
FAM49B
ARNTL
GAS5
PI4KA
RPL4
HCLS1
RPL26
ILKAP
B2M
SCPEP1

MED30
MED9
MEF2D
MEGF8
MEIS1
METTL14
METTL15
METTL2A
METTL2B
MFAP1
MFGE8
MFN2
MFSD1
MGAT5
MIB2
MICAL2
MICAL3
MIER1
MIER2
MIF
MIS12
MITF
MKRN2
MLF2
MLPH
MLX
MMADHC
MMGT1
MNT
MOBP
MON2
MOSPD2
MPDU1
MPEG1
MPHOSPH10
MPHOSPH9
MPP1
MPP6
MRAS
MRPL14
MRPL17
MRPL18
MRPL21
MRPL22
MRPL27
MRPL28
MRPL30
MRPL36
MRPL38
MRPL40
MRPL41
MRPL45
MRPL50
MRPL52
MRPL53
MRPL9
MRPS6
MRPS9
MRRF
MSH6
MSL3
MSRA
MT1A
MT1E
MTA1
MTAP
MTF2
MTHFS
MTMR10
MTMR2
MTMR6
MXI1
MYBBP1A
MYL12B
MYO1E
MYO1F
MYO9B
MYSM1
NAA30
NAA35
NAA50
NAALAD2
NAGK
NAGLU
NAIP
NAMPT
NAP1L1
NAPA
NAPG
NASP
NAT8L
NBR1
NCBP2AS2
NCEH1
NCOA7
NCSTN
NDE1
NDFIP2
NDRG3
NDUFA10
NDUFA11
NDUFA13
NDUFA2
NDUFA3
NDUFA6
NDUFA8
NDUFAF4
NDUFB10
NDUFB11
NDUFB5
NDUFB6
NDUFB7
NDUFB9
NDUFS2
NDUFS6
NDUFV3
NEDD8
NEIL1
NEK6
NEMF
NENF
NEURL3
NF1
NFATC1
NFATC3
NFE2L2
NFIC
NFS1
NFXL1
NFYA
NFYC
NHP2
NIBAN1
NIPSNAP3A
NKTR
NLRC5
NME1
NME2
NME3
NMI
NNT
NOL8
NOLC1
NOM1
NOP58
NPC2
NPEPPS
NPFF
NPM1
NPNT
NPTN
NQO2
NRAS
NRDC
NRGN
NRP1
NSMCE1
NSMCE2
NSMCE4A
NT5DC2
NUCB1
NUCB2
NUDC
NUDT4
NUDT4B
NUDT9
NUP205
NUP54
NUS1
NVL
OPA1
ORMDL1
ORMDL2
OS9
OSBPL2
OSBPL8
OST4
OSTM1
OXCT1
P2RX4
P2RY2
PABPC1
PACS2
PADI2
PAFAH1B3
PARD6A
PARK7
PARL
PARP1
PCBD2
PCBP2
PCMTD2
PCNA
PCNT
PCP4
PDCD2
PDCD4
PDCD6
PDE12
PDHB
PDK1
PDXK
PDZD11
PEA15
PEAR1
PEDS1
PELP1
PEX12
PEX16
PFDN5
PFKFB2
PFKFB3
PFKL
PFN1
PGAM5
PGAP1
PGAP6
PGD
PGK1
PGLS
PGM1
PGM2L1
PGRMC1
PHACTR4
PHC1
PHC2
PHF3
PHF7
PHKA2
PHKG2
PHLPP1
PHRF1
PIAS2
PIGK
PIGN
PIGO
PIGP
PIGS
PIK3R4
PIP4K2C
PIP5K1C
PITPNA
PITRM1
PKIB
PKN2
PLA2G12A
PLA2G4B
PLA2G6
PLBD2
PLCB2
PLCB3
PLD3
PLEK
PLEKHA1
PLEKHM2
PLEKHM3
PLIN2
PLXDC2
PLXNB3
PLXND1
PMP22
PNO1
PNPLA7
POFUT1
POGK
POGZ
POLD3
POLD4
POLR1H
POLR2G
POLR2J
POLR2J2
POLR2J3
POLR3K
POMP
POMT1
PON2
PON3
PPAN
PPAN-P2RY11
PPAT
PPFIA4
PPIB
PPIG
PPM1A
PPM1F
PPM1H
PPP1CB
PPP1R11
PPP1R14B
PPP1R3D
PPP2CA
PPP2R2D
PPT1
PRDM1
PRDX1
PRDX2
PRDX3
PRDX4
PRDX5
PRDX6
PRICKLE1
PRKCB
PRKDC
PRMT3
PROS1
PRPF19
PRPF4
PRPF40A
PRPF8
PRUNE2
PSAP
PSAT1
PSD4
PSIP1
PSMA3
PSMA6
PSMA7
PSMB1
PSMB2
PSMB7
PSMB8
PSMB9
PSMD10
PSMD2
PSME1
PSME2
PSMF1
PSMG2
PSMG4
PTDSS1
PTK2B
PTMA
PTOV1
PTPRA
PTPRC
PTPRM
PUS3
PYCR3
PYGL
PYROXD1
QARS1
R3HCC1
RAB11FIP5
RAB12
RAB18
RAB1B
RAB21
RAB27A
RAB31
RAB32
RAB5C
RAB8A
RABAC1
RABGAP1L
RABGGTB
RABL3
RAE1
RALGAPA1
RAMP1
RAN
RANBP1
RANBP6
RAP2A
RAP2B
RASA4
RASA4B
RASGRF2
RASGRP3
RASSF3
RBBP7
RBM10
RBM12
RBMS1
RCBTB1
RCBTB2
RCC2
RCOR1
REEP3
RENBP
REST
RETSAT
REXO2
RFC1
RFC2
RGS1
RHBDD2
RHBDF1
RHEB
RHOC
RING1
RINL
RMND5A
RNASEH2B
RNASEK
RNASET2
RNF103-CHMP3
RNF115
RNF149
RNF150
RNF166
RNF187
RNF40
RNF41
RNF7
RNFT1
RNPEP
RNPEPL1
ROMO1
ROPN1L
RPL10
RPL10A
RPL12
RPL13
RPL13A
RPL14
RPL15
RPL18A
RPL22
RPL23
RPL24
RPL27A
RPL30
RPL31
RPL34
RPL35
RPL35A
RPL36AL
RPL37
RPL37A
RPL38
RPL39
RPL4
RPL5
RPL6
RPL7

RPL8
RPLP0
RPLP1
RPLP2
RPN2
RPP14
RPRD1B
RPS10
RPS11
RPS14
RPS15
RPS15A
RPS16
RPS17
RPS18
RPS19
RPS2
RPS20
RPS21
RPS24
RPS25
RPS26
RPS27A
RPS27L
RPS28
RPS29
RPS3
RPS4Y1
RPS5
RPS6KC1
RPS8
RPS9
RRAGC
RRAS
RRAS2
RRM1
RRM2B
RRP1
RRP8
RTN3
RTP4
RTTN
RUFY1
RXRB
S1PR1
SACM1L
SAMM50
SAMSN1
SAP130
SAP25
SAP30BP
SARS1
SAT1
SBNO1
SCAF11
SCAI
SCAMP4
SCAND1
SCARB2
SCARF2
SCD
SCLT1
SCP2
SCPEP1
SCRN3
SCYL1
SDC3
SDCBP
SDF2L1
SDF4
SDHA
SDHAF1
SDHAF2
SEC13
SEC23A
SEC24A
SEC24B
SEC63
SEH1L
SEL1L
SENP1
SENP5
SENP7
SERP1
SERPINE2
SETD1A
SETD3
SETX
SF3B2
SF3B5
SFR1
SFT2D3
SGCB
SGPL1
SH3BGRL3
SH3KBP1
SHARPIN
SHC1
SHISA5
SHKBP1
SHMT1
SIAE
SIAH2
SIDT2
SIGLEC1
SIGLEC6
SIGLEC8
SIKE1
SIL1
SIN3B
SIPA1L1
SIPA1L2
SIRT2
SIVA1
SLAMF9
SLC11A1
SLC12A2
SLC15A2
SLC15A3
SLC16A10
SLC16A3
SLC17A5
SLC1A2
SLC23A2
SLC25A25
SLC25A28
SLC25A3
SLC25A32
SLC25A39
SLC25A5
SLC30A1
SLC30A7
SLC31A1
SLC35B3
SLC35C1
SLC35E1
SLC37A2
SLC38A1
SLC38A2
SLC39A1
SLC39A14
SLC44A1
SLC44A2
SLC6A6
SLC9A6
SLU7
SLX4
SMARCD1
SMARCD2
SMC1A
SMC6
SMG6
SMN1
SMN2
SMO
SMPD4
SMU1
SMYD5
SNAP47
SNAPC1
SNCAIP
SND1
SNF8
SNRNP200
SNRPB
SNRPD1
SNRPD2
SNRPD3
SNX1
SNX10
SNX16
SNX25
SNX3
SOAT1
SOCS6
SOD1
SORL1
SOS2
SP110
SP2
SPARCL1
SPC25
SPEG
SPG7
SPHK2
SPNS2
SPOCK1
SPOP
SPP1
SQSTM1
SRA1
SREBF2
SRGN
SRM
SRPK1
SRPK2
SRPRB
SRRD
SSH1
SSNA1
SSR4
SSU72
SSX2IP
ST14
ST20-MTHFS
ST6GAL1
ST7
ST8SIA6
STAG2
STAM2
STAT1
STAT2
STAT5B
STAU1
STAU2
STIM2
STRADA
STRN
STRN4
STUB1
STX12
STX6
STX7
SUGP2
SULF2
SUMF1
SUMF2
SUMO3
SUN2
SURF1
SWAP70
SWI5
SYCP3
SYNCRIP
SYNGR1
TAB1
TAF1C
TAF5
TAGLN2
TANK
TAOK3
TAPBP
TAZ
TBC1D13
TBC1D20
TBC1D8
TBCC
TBCD
TBRG1
TBXAS1
TCEA2
TCEAL1
TCEAL8
TCF19
TCIRG1
TCOF1
TEC
TECPR1
TECPR2
TECR
TEP1
TET2
TEX261
TF
TFPI
TGFBR2
THBS1
THUMPD1
THUMPD2
TIMM13
TIMM8B
TIMM9
TIMP2
TIPRL
TKT
TLCD2
TLK1
TLN2
TLR2
TM2D3
TM7SF3
TMBIM1
TMBIM4
TMC6
TMCC2
TMCC3
TMCO3
TMED2
TMED3
TMEM101
TMEM106A
TMEM123
TMEM147
TMEM14C
TMEM154
TMEM159
TMEM160
TMEM175
TMEM176A
TMEM18
TMEM184B
TMEM208
TMEM214
TMEM43
TMEM50B
TMEM65
TMF1
TMOD1
TMSB4Y
TNFAIP3
TNIP2
TNS3
TOLLIP
TOMM7
TOP2A
TOP3A
TOR1AIP2
TOR1B
TP53INP2
TPD52
TPI1
TPP1
TPR
TPRG1L
TPRN
TPT1
TRAPPC2L
TRAPPC4
TREM2
TRIM24
TRIM25
TRIM27
TRIM28
TRIM32
TRIM41
TRIOBP
TRMT2B
TRMT6
TSC2
TSEN2
TSFM
TSG101
TSGA10
TSPAN31
TSPO
TSPYL1
TSSC4
TTC17
TTC38
TTF1
TTF2
TTR
TUBA1C
TUBB
TULP4
TUSC2
TUSC3
TXLNA
TXN
TXNDC16
TXNDC17
TXNL4A
TYROBP
UAP1
UBA6
UBAC2
UBE2B
UBE2D2
UBE2D3
UBE2E1
UBE2G1
UBE2M
UBE2Q1
UBE2R2
UBE3B
UBE4B
UBL5
UBP1
UBQLN2
UBXN1
UBXN8
UCKL1
UCP2
UFC1
UHRF1BP1
UPF2
UPP2
UQCR10
UQCR11
UQCRB
UQCRFS1
UQCRHL
UQCRQ
URM1
USE1
USF2
USO1
USP12
USP16
USP30
USP32
USP34
USP47
USP5
VAMP3
VANGL2
VARS1
VBP1
VDAC1
VDAC2
VEGFB
VEZT
VKORC1
VMA21
VPS13C
VPS16
VPS18
VPS26A
VPS29
VPS37C
WASL
WDFY1
WDR12
WDR35
WDR36
WDR41
WDR53
WDR55
WDR82
WDR83
WDR91
WIPF2
WLS
WRNIP1
WWOX
WWP1

XAF1
XPA
XPNPEP1
XRCC4
XYLT2
YIF1A
YIPF5
YTHDC2
YY1AP1
ZBP1
ZBTB10
ZBTB22
ZBTB33
ZBTB34
ZBTB44
ZBTB49
ZC3H10
ZC3H12A
ZC3HAV1
ZC4H2
ZCCHC10
ZCCHC24
ZCWPW1
ZDHHC16
ZDHHC18
ZDHHC21
ZDHHC3
ZDHHC4
ZFAND2B
ZFP30
ZFP36
ZFR
ZFYVE21
ZHX1
ZHX1-C8orf76
ZMIZ2
ZMPSTE24
ZMYM4
ZNF106
ZNF189
ZNF195
ZNF236
ZNF260
ZNF266
ZNF274
ZNF280C
ZNF326
ZNF354A
ZNF394
ZNF397
ZNF420
ZNF426
ZNF519
ZNF524
ZNF641
ZNF654
ZNF655
ZNF692
ZNF747
ZNF764
ZNF830
ZNHIT1
ZNRF2
ZSWIM1
ZZZ3

Supplementary Table 2_AUCell gene set enrichment
AUCell enrichment

Gene List	Comparison Group	Test Group	p.adj	Cohen's d eff_size	Eff_size_term	Lower conf interval	Upper conf interval
Core Microglial Genes (Galatro)	Micro_1	Micro_2	2.10E-132	-0.4044782	(small)	-0.4352231	-0.3737334
Core Microglial Genes (Galatro)	Micro_1	Micro_3	0	-1.451992	(large)	-1.487855	-1.416128
Core Microglial Genes (Galatro)	Micro_1	PVM	3.30E-277	-0.8980096	(large)	-0.9394599	-0.8565594
Aging (Olah)	Micro_1	Micro_2	2.10E-57	0.2705161	(small)	0.2399132	0.3011191
Aging (Olah)	Micro_1	Micro_3	3.00E-47	0.2377403	(small)	0.2049114	0.2705692
Aging (Olah)	Micro_1	PVM	1.40E-43	0.3338133	(small)	0.2933249	0.3743017
DAM (Keren-Shaul)	Micro_1	Micro_2	0	0.7091486	(medium)	0.677877	0.7404203
DAM (Keren-Shaul)	Micro_1	Micro_3	0.000028	-0.06944977	(negligible)	-0.10219857	-0.03670096
DAM (Keren-Shaul)	Micro_1	PVM	5.70E-97	0.5059975	(medium)	0.4653071	0.5466879
ARM (Sala Frigerio)	Micro_1	Micro_2	7.10E-278	0.6517729	(medium)	0.6206217	0.6829242
ARM (Sala Frigerio)	Micro_1	Micro_3	1.10E-161	-0.3972333	(small)	-0.4302185	-0.3642481
ARM (Sala Frigerio)	Micro_1	PVM	2.50E-57	0.4119251	(small)	0.3713552	0.452495
IRM (Sala Frigerio)	Micro_1	Micro_2	4.10E-78	0.3127979	(small)	0.2821561	0.3434396
IRM (Sala Frigerio)	Micro_1	Micro_3	0.021	0.03815325	(negligible)	0.005409667	0.070896833
IRM (Sala Frigerio)	Micro_1	PVM	6.90E-68	0.4270782	(small)	0.3864905	0.4676659
PIG (Chen)	Micro_1	Micro_2	2.70E-17	0.1410731	(negligible)	0.1105541	0.1715922
PIG (Chen)	Micro_1	Micro_3	5.90E-69	-0.2689941	(small)	-0.3018475	-0.2361407
PIG (Chen)	Micro_1	PVM	0.022	0.05432948	(negligible)	0.01399327	0.09466569

Supplementary Table 2_AUCell gene set enrichment
Ext data figure 11 AUC values

**Gene List**	**Micro1**	**Micro2**	**Micro3**	**PVM**
DAM (Keren-Shaul)	0.1400868	0.15581126	0.13874868	0.1510533
ARM (Sala Frigerio)	0.03799058	0.04800219	0.03285514	0.0440068
Aging (Olah)	0.04465521	0.04894072	0.04802817	0.04979458
Core Microglial Genes (Galatro)	0.10716635	0.09945952	0.08286285	0.08964665

Supplementary Table 3_DGE enrichment in other datasets
Contents

Sheet name	Description
Astrocyte_DGE	Astrocyte DEG enrichment in samples reported in previous studies
Microglia_DGE	Microglia DEG enrichment in samples reported in previous studies

Terms:

DEGs_amyloid_UP	Genes found to be significantly positively associated with amyloid pathology in astrocytes or microglia (see Supplementary Tables 1 and 2 respectively)
DEGs_amyloid_DOWN	Genes found to be significantly negatively associated with amyloid pathology in astrocytes or microglia (see Supplementary Tables 1 and 2 respectively)
DEGs_ptau_UP	Genes found to be significantly positively associated with pTau pathology in astrocytes or microglia (see Supplementary Tables 1 and 2 respectively)
DEGs_ptau_DOWN	Genes found to be significantly negatively associated with pTau pathology in astrocytes or microglia (see Supplementary Tables 1 and 2 respectively)

Analyses were performed using AUCell and limma (see Methods)

Previous studies:	Grubman et al 2019, doi: 10.1038/s41593-019-0539-4
	Mathys et al 2019, doi: 10.1038/s41586-019-1195-2
	Zhou et al 2020, doi: 10.1038/s41591-019-0695-9
	Gerrits et al 2021, doi: 10.1007/s00401-021-02263-w

Supplementary Table 3_DGE enrichment in other datasets
Astrocyte DGE

**Grubman et al 2019**	**logFC**	**P.Value**	**adj.P.Val**
DEGs_amyloid_UP	0.43864158	5.80E-112	1.1603E-111
DEGs_amyloid_DOWN	-0.6633103	5.75E-213	2.2991E-212
DEGs_ptau_UP	0.33956493	4.91E-74	4.91345E-74
DEGs_ptau_DOWN	-0.5068653	5.07E-101	6.7615E-101

**Mathys et al 2019**	**logFC**	**P.Value**	**adj.P.Val**
DEGs_amyloid_UP	0.13023331	4.85E-32	9.70988E-32
DEGs_amyloid_DOWN	-0.1007316	3.97E-17	5.29454E-17
DEGs_ptau_UP	0.13588274	4.54E-33	1.8156E-32
DEGs_ptau_DOWN	-0.0955003	4.61E-13	4.61453E-13

**Zhou et al 2020**	**logFC**	**P.Value**	**adj.P.Val**
DEGs_amyloid_UP	0.20404415	7.52E-195	1.5049E-194
DEGs_amyloid_DOWN	-0.0810627	6.06E-18	6.05788E-18
DEGs_ptau_UP	0.25388578	1.50E-307	6.0061E-307
DEGs_ptau_DOWN	-0.1680302	1.73E-60	2.30681E-60

**Gerrits et al 2021**	**logFC**	**P.Value**	**adj.P.Val**
DEGs_amyloid_UP	0.01363858	2.09E-14	2.78474E-14
DEGs_amyloid_DOWN	-0.0169866	1.99E-20	7.95435E-20
DEGs_ptau_UP	0.00750133	0.00015475	0.000154753
DEGs_ptau_DOWN	-0.015916	5.28E-15	1.05699E-14

Supplementary Table 3_DGE enrichment in other datasets
Microglia DGE

**Grubman et al 2019**	**logFC**	**P.Value**	**adj.P.Val**
DEGs_amyloid_UP	0.37751126	2.63E-15	1.05233E-14
DEGs_amyloid_DOWN	-0.2022053	6.76E-05	0.000135176
DEGs_ptau_UP	0.13650833	0.01612692	0.021502553
DEGs_ptau_DOWN	0.15115991	0.02941246	0.029412464

**Mathys et al 2019**	**logFC**	**P.Value**	**adj.P.Val**
DEGs_amyloid_UP	0.09607165	1.89E-06	3.78594E-06
DEGs_amyloid_DOWN	-0.0535726	0.0347086	0.034708597
DEGs_ptau_UP	0.13853693	5.61E-08	2.24362E-07
DEGs_ptau_DOWN	-0.0595375	0.03117732	0.034708597

**Zhou et al 2020**	**logFC**	**P.Value**	**adj.P.Val**
DEGs_amyloid_UP	0.19889297	1.51E-40	2.01207E-40
DEGs_amyloid_DOWN	-0.0590511	0.00393719	0.003937194
DEGs_ptau_UP	0.36264581	4.33E-76	1.73373E-75
DEGs_ptau_DOWN	-0.3735525	4.98E-45	9.95415E-45

**Gerrits et al 2021**	**logFC**	**P.Value**	**adj.P.Val**
DEGs_amyloid_UP	0.1383819	0	0
DEGs_amyloid_DOWN	0.00788924	0.00012742	0.000127415
DEGs_ptau_UP	0.18163868	0	0
DEGs_ptau_DOWN	-0.028563	1.93E-32	2.56905E-32

Supplementary Table 4_Astrocyte MEGENA modules
Contents

Sheet name	Description
Module_list	MEGENA Gene Co-expression Modules in Astrocytes
Module_description	MEGENA Gene Co-expression Module description
Module 9 FE	Functional enrichment of Module 9 (CLU hub gene) genes by enrichR
Module 30 FE	Functional enrichment of Module 30 (CLU hub gene) genes by enrichR
Module 13 FE	Functional enrichment of Module 13 (APOE hub gene) genes by enrichR
Pathology_enrichment	logFC of the enrichment (AUCell and limma) of modules with amyloid-beta and pTau pathology
Enrichment_other_datasets	logFC of the enrichment (AUCell and limma) of modules in AD samples compared to Control samples from previous studies
MSS	Module specificity scores (MSS) in astrocytic subclusters

Supplementary Table 4_Astrocyte MEGENA modules
Module list

MEGENA Gene Co-expression Modules in Astrocytes

c1_3	c1_4	c1_5	c1_6	c1_8	c1_9	c1_10	c1_11	c1_12	c1_13	c1_16	c1_17	c1_18
APOE	SLC26A3	SLC1A2	SLC38A1	CACNB2	CRYAB	F3	ARHGEF3	HSPB1	APOE	FTH1	SLC26A3	XIST
FTH1	RASGEF1B	SLC1A3	NRXN1	HPSE2	HSPA1A	CHI3L1	MACF1	HSPH1	CKB	MALAT1	RASGEF1B	UTY
MALAT1	XIST	SNED1	DCLK1	SERPINI2	MT3	SAMD4A	DST	NEAT1	ITM2C	HEPN1	LINGO1	USP9Y
CKB	LINGO1	PTN	LINC01088	DPP10	MT2A	AHCYL1	SLC44A3-AS1	AC091826.2	CPE	PCDH9-AS2	INO80D	LINC00278
HEPN1	INO80D	SLC4A4	TNC	LINC00609	CLU	ITPR2	TPST1	LINC02060	GPR37L1	AP000462.1	ARL17B	ANOS2P
ITM2C	UTY	GLUL	KAZN	VCAN	UBC	ADGRG1	OSMR	AC002429.2	B4GALNT4	LINC00513	CSMD1	KANSL1
PCDH9-AS2	ARL17B	GRIA2	STXBP5L	WDR49	ID3	BCL6	PLSCR1	LINC01748	BCAN	CMAHP	FAM198B-AS1	STK33
AP000462.1	USP9Y	DGKG	GALNT15	CERS6	JUN	NRCAM	ZFYVE28	NPAS2	METRN	AC079385.1	C2orf72	ARAP2
MT1E	CSMD1	DNM3	ARHGAP24	VAV3	FOS	AC010655.4	FAM20C	PAMR1	PRKIP1	P2RY14	PABPN1	FHIT
CPE	LINC00278	LRRC3B	CADM1	SLC35F1	DBI	ATP1B2	ELL2	SLC7A11	SLC3A2	PNISR	MRPL41	LRMDA
LINC00513	ANOS2P	ATP1A2	CADM2	AQP1	HSP90AA1	DOCK4	CNTNAP3B	CRB1	ITM2B	AC093535.1	GSTM5	ABLIM1
CMAHP	FAM198B-AS1	SLC6A11	SYNPO2	COL5A3	PTGES3	PFKFB3	OSMR-AS1	AC021231.1	TSC22D4	AC092902.4	VWA8	AP001992.1
AC079385.1	KANSL1	MIR4300HG	LRP1B	AKAP6	HMGB1	EHBP1	CNN3	AC104596.1	NME3	FP671120.2	MAPT	ATG4B
P2RY14	C2orf72	FGFR3	GPC5	TRPM3	HSP90AB1	CADPS	SLC39A14	SQSTM1	EDNRB	HNRNPDL	ITGA7	LIFR
SRP14	PABPN1	PLD5	KCNN2	NR2F2-AS1	S100A1	ANGPTL4	DEPP1	AC125613.1	CSPG5	AP002336.2	ACADVL	LINC00923
PNISR	MRPL41	GABRA2	CD44	RIMS1	MT1F	RGL1	NAMPT	AZIN1-AS1	DDR1	SLC6A1-AS1	SDC3	AC092683.1
GPR37L1	STK33	HTRA1	KCNJ3	OLFM2	GJA1	PTPRZ1	BAALC-AS1	RFX3-AS1	GRINA	ACTG1	PATJ	SPATA6L
RPL32	GSTM5	F11-AS1	GRM3	CALN1	COPS9	LRIG1	SLC44A3	IQCA1	WFS1	SCRG1	PFKL	KDM6A
COX7C	VWA8	MAST4	MAN1C1	SPRY4-AS1	AGT	TRIO	SLC9A9	IFRD1	ENHO	RORA-AS1	AC093535.2	CAMK1D
RPS25	MAPT	C1orf61	ERBB4	B3GAT2	CD81	PRKCA	CNTNAP3	AC027117.2	TMEM259	INTU	MROH7-TTC4	GUSBP1
RPL11	ARAP2	DLC1	ADGRB3	KCTD8	PRDX1	CAMK2G	DPYSL2	AC108866.1	COL16A1	GNG12-AS1	AP006621.3	SLC25A29
AC093535.1	FHIT	SMAD9	LSAMP	KCNIP1	DYNLL1	IFITM3	DNER	NAALADL2	PEA15	NKTR	SNX19	IRAK1BP1
B4GALNT4	ITGA7	FRMD4A	LINC01411	RGS20	MT1M	CNTN1	PROS1	SPATA6	CTSF	HNRNPA2B1	BCOR	VPS50
AC092902.4	ACADVL	RNF19A	GPM6A	NDRG2	PSAP	NTRK3	PAM	CSGALNACT1	ANO8	DKK3	UHRF2	MEGF9
RPL30	LRMDA	NRP1	AC073941.1	SLC8A1	GAPDH	PTPN9	TMTC1	LINC00461	PKD1	PDE5A	CPSF7	TRA2A
FP671120.2	SDC3	PRRX1	ADAMTSL3	PLXDC2	CHORDC1	RALGDS	RFTN1	LINC01376	ANKRD9	AL078459.1	ARFGAP1	HERC4
BCAN	ABLIM1	ARHGEF4	PCDH9	PSD3	DNAJA1	TMBIM1	TAB2	UFL1-AS1	S1PR1	ZDHHC21	WDR6	TMEM144
HNRNPDL	PATJ	HS3ST5	GABRB1	ST6GALNAC3	TENM2	ACSS3	FAM189A2	GMDS-DT	FAIM2	AC244669.1	NFIC	CEP290
RPLP1	PFKL	LGR4	NFASC	EGFR	TPT1	LINC02649	HMBOX1	SLC38A2	ALDH2	HNRNPH1	ATP5MC2	RAB4A
AP002336.2	AC093535.2	EGF	DGKB	LINC00299	ZFP36L1	HES1	CTDSPL	TAPT1-AS1	SAT2	AC016831.7	CPEB2	TMEM178B
SLC6A1-AS1	AP001992.1	KCNE4	TTN	BNC2	SERF2	MFAP3L	NOD1	CAP2	LTBP3	ZNF638	SHC2	CEP83
METRN	MROH7-TTC4	TUBB2B	GRIA1	TPD52L1	FBXO2	FYN	AL354740.1	AL133257.1	SELENBP1	AC068587.4	MAPK8IP3	DOCK3
RPLP2	AP006621.3	PIK3C2A	GPC6	KCNQ3	HSPA4L	PDE4B	LIMS1	SIRPB3P	CYHR1	ZRANB2	MARK1	ERCC8
UQCRB	SNX19	KCNJ10	IGFBP5	LAMA4	HSPD1	PBX1	FGF2	AC003991.1	INPPL1	AHI1	LRRC37A2	PHF20L1
ACTG1	BCOR	ZNF273	SYNE1	DGKZ	VEGFA	NPL	ASAP3	CLIP2	DPP7	RAB11A	QTRT1	CCND2-AS1
SCRG1	ATG4B	AL365273.2	ADGRL3	FGFR2	DDIT4	ADAM12	ERBIN	GLUD1	DTX3	TUT4	ATE1-AS1	RNF169
RORA-AS1	UHRF2	39326	LINC01094	DCLK2	ATP5ME	BHLHE40	MFHAS1	TKT	NDUFS2	ARGLU1	ATP6V0E2	ZFX
[illegible]	[illegible]	[illegible]	[illegible]	[illegible]	[illegible]	[illegible]	[illegible]	[illegible]	[illegible]	[illegible]	[illegible]	[illegible]

c1_19	c1_21	c1_22	c1_23	c1_24	c1_25	c1_26	c1_27	c1_28	c1_29	c1_30	c1_32	c1_33	c1_34	c1_36	c1_37	c1_41	c1_46	c1_47	c1_51	c1_53	c1_54	c1_61	c1_62	c1_65	c1_66	c1_78	c1_80	c1_83	c1_86	c1_89	c1_92	c1_95	c1_96	c1_99	c1_106	c1_108	c1_112	c1_117	c1_119
[illegible]	[illegible]	[illegible]	[illegible]	[illegible]	[illegible]	[illegible]	[illegible]	[illegible]	[illegible]	[illegible]	[illegible]	[illegible]	[illegible]	[illegible]	[illegible]	[illegible]	[illegible]	[illegible]	[illegible]	[illegible]	[illegible]	[illegible]	[illegible]	[illegible]	[illegible]	[illegible]	[illegible]	[illegible]	[illegible]	[illegible]	[illegible]	[illegible]	[illegible]	[illegible]	[illegible]	[illegible]	[illegible]	[illegible]	[illegible]

WAPL	CD99L2	ALS12662.2	DENND2A	TMEM248	ZFP14	PIGF
USP48	VAPA	CC2D2A	BACH1	SLC41A2	POC1B-AS1	KATNAL1
GSAP	ZNF271P	VPS13B	LRCH1	SSR3	TLE3	CDK13
SLMAP	PIP4K2B	CBX5	FTO	SLC11A2	HSPA12A	MCM9
PRKCSH	CLASRP	CTIF	PPP6R2	LRP12	MARF1	SCAMP1
WBP4	MED13	PDLIM5	GNAL	HNRNPM	ATG14	WWP1
MAGI2-AS3	AC004076.1	RERE	AC074032.1	IPO7	ZNF549	CEP162
AFTPH	UBTD1	PLEKHM3	SAMD4B	ZBTB25	ATF1	RUFY2
CDC42SE1	NCOA3	PLA2G16	MTG2	NIM1K	WWP2	DEK
ARHGAP12	CDC27	RUNX1T1	MAP2K1	CHD8	YY1	ATP11B
GLMN	PHF12	FAM204A	GGT7	MMP24OS	TULP4	ZNF862
SLC17A5	FBXO28	HADHB	TTC38	KPNB1	ACAT1	KIF2A
CEP63	IQSEC2	PPP2R5A	SPESP1	POLR3A	RAB12	ZBTB41
NSRP1	RAB8B	XPR1	INPP5A	ARHGAP17		PHYKPL
ZMYND11	BLCAP	PKP4	AC008403.1	SRP14-AS1		PIKFYVE
EIF3A	ADIPOR2	ERC2	RBM10	SPTBN4		XRN1
GPR137B	PRPSAP2	MIR100HG	CCDC57	CALU		LARP4B
PRCP	TRAP1	WDFY3-AS2	KSR1	CLPB		CUL5
IQGAP1	APTR	CWC27	AC025682.1	SGPP1		SUPT3H
IQCB1	SOCS7	ATXN7L1	PKNOX1	MIATNB		C1GALT1
AGPAT5	MAP3K3	LINC00886	NDRG3	RRBP1		STX7
PNPT1	CEP89	GNAO1	SMARCA4	ARFGAP3		ZNF800
SEC63	NBR1	LRRFIP1	NUP50-DT	FAF2		ZBTB8OS
SERINC1	FBXO34	BBS9	BCL2L1	KCTD20		SCYL2
AC124319.1	GRB2	EXT1	HSCB	CREB3L2		CSTF3
BBX	ZCCHC8	WDR35	CBL	ELMSAN1		CLCN6
TFG		EML6	VPS26C	TRIM56		TRMT13
NAA15		PER2	PANK2	ZNF75A		KPNA3
XPA		CTNNA3	PHKA2	HM13		RPRD1B
C1orf54		SLC12A6	SPATS2	PTPN11		RIF1
PSIP1		AL162726.3	NDUFV3	ZFP36		UBE2E1
DHX9		CHIC2	ZNF791	AKAP13		TPR
AGFG1		ARHGEF26	RALY	TSC22D1		KDM1A
SYNGAP1		MVB12B	NF2	MIDN		ARL8B
DCTN4		AL160314.2	SLC25A53	YTHDF1		CAMSAP1
CDK17		HACD2	HNRNPUL1	SCML1		NUTM2A-AS1
TTC3		CMC1		GPM6B		GOLPH3
CLOCK		VPS45		AC005261.1		RNMT
GLRX3		TUBA1C		ITGB1		EIF4G2
TOP2B		AL355974.2		C1S		UVRAG
PRKAA2		KANSL1L		ZNF708		BBS4
PUM3		ZNF438		DPY19L3		PABPC1
DCUN1D4		RIDA		DAPK1		RBV1
LRPPRC		AC242426.2		TOM1L2		ASXL2
STK38		EXOC4		SERGEF		ARID1A
PCNP		DCP1B		EVA1C		RNF145
THRAP3		AFF3		RFX2		INVS
ZFAND6		ABTB2		MYH9		OSBPL8
UBA5		TBCK		PTCHD1		STRN
MAX		LRSAM1		PSMG4		SCAF11
CHD6		HEG1		CYBC1		TTBK2
COL19A1		CYFIP2		SPPL2A		AP1G1
WDR26		OXCT1		MTSS1L		ATP10D
MTDH		ADD3		CADM4		RNF180
FBXL4		FAM126B		DPH6-DT		EIF3H
NUP133		AMOTL1		AUH		WDR41
HLTF		ANKRD44		MTMR3		MINDY3
PLCD1		TTC7A		ZNF423		DENND6A
SLC25A13		AC087564.1		MRTFA		FKTN
NBPF12		ERI1		TRAF3IP2		PSMD14
ZDHHC17		SP4		SLAIN1		TXNRD1
PMS1		AC079447.1		TCF12		OXSR1
HACE1		MTA3		CDC37		RSBN1L
STXBP3		MNAT1		TPS3		SNX2
EPS8		XKR6		DDHD1		PSMD5
INTS11		SERP1		CABIN1		FGFR1OP2
XRN2		RHBDD1		ATP9B		MEF2A
[illegible]		[illegible]		[illegible]		[illegible]

RAPGEF1
HHIPL1
SH3BGRL
LINS2
DLG1
FIRRE
AC007495.1
NMI9
LINC00598
NSMCE2
SLC43A2
SLC10A7
UTP23
PPP2R1B
C15orf41
ARL3
GANC
NCBP1
LRRC37B
PDSS2
HLCS
FHOD3
EFHC2
TSC1
PKIG
SPG11
MGMT
HMG20A
NBDY
ZSCAN30
AL353743.1
KDM2B
NCOA5
INTS8
CYTH1
AC092338.1
PYGO1
ZNF710
RC3H2
LINC00662
PIN4
ERCC6
ZNF251
AC007216.4
WDR25
NEK4

[illegible]

CTCF
CLTC
NVL
MAPK9
PRPF8
SMARCAL1
PHLPP2
PPHLN1
UQCRC2
WASHC3
EXOC7
C2CD3
ZCCHC14
NR2C2
FBXO33
MRPL45P2
BTBD2
METTL16
TUBGCP3
AKAP11
SETD3
CHIC1
CWF19L2
CUL4A
FBXO22
ESD
WDR73
SUPT20H
ACTR10
TRIM13
ZSWIM7
RFX4
SRP54
AC098850.3
APLP2
ATL1
MAU2
MTIF3
KLC1
CEP95
SEC11A
RAB30
PCF11
XPOT
SCGB2B2
RSPRY1
VPS4B
VPS29
DZIP1
TTC3P1
PHF11
GGNBP2
SYCP2
LCMT1
CST3
PRKCA-AS1
AP000311.1
CSTB
RPS11
RPL10
UQCR10
TMEM47
SAT1
SLITRK2
ALOX12P2
PAXBP1
LINC02256
PTTG1IP
ANKRD12
PRMT2
HMGN1
CRLF3
RCN2
HSF4
LAMP2
THOC2
MPHOSPH8
SPAG9
ZFAS1
ATRX
KTN1
PLXNB2
VPS13C
AL139317.5
RGS11
TMSB4X
FOXG1
BRWD1
COL6A1
LAMA5
ARHGEF7
GAS2L1
URI1
PAN3
DDX3X
KATNBL1
HOOK2
MYEF2
ZSCAN18
HMGXB4
UBE2Q2P2
HERC2
MARK3
ATM
ZFC3H1
CLPTM1
OFD1
RBMX
UGGT2
VPS9D1
CDC16
ARID4A
RNF111
THOC1
SRSF6
SMARCA1
SMS
USP16
KLHDC2
CEP295
ZNF780B
CDC42EP4
RBCK1
CHM
LINC00685
TAF1
CSNK1E
EWSR1
SOX9-AS1
ILF3
TM9SF4
ZNF44
TRPM7
COG7
TTC28-AS1
CAND1
PIK3C3
SPART
TP53BP1
AP2B1
SLC26A11
BRWD3
TRIP11
RRAGB
ZNF334
ATP5F1A
ATG2B
ARID2
FHL1
PCMTD2
MCM3AP
GALC
LTN1
ADAM10
IQCH
RB1
RIOK3
ESF1
ZNF471
TMLHE
DEF8
ICE2
ZNF81
VPS53
UPF3B
ARHGEF40
AC138207.8
ELP2
HPS4
AQR
TRAPPC10
CDKL5
SNX1
CDC42BPB
LRCH2
TPP2
MBTPS1
MED14
ALG13
CASC4
GPATCH2L
FBXO7
WNK3
AAGAB
KRBOX4
APPBP2
NAE1
ZNF529
GSPT1
NAA16
ZNF155
AL162171.1
PITPNB
GRAMD1A
DIP2A
ACSL4
KDSR
AP3D1
PHF8
UBA2
GPALPP1
ARMCX5-GPRASP2
TMEM50B
CDS2
CDK12
SMAD4
SOS2
FBXW8
APOOL
TARSL2
RTTN
ZNF160
GABPA
NCBP3
ZNF24
SELENOO
GRIPAP1
DDX42
IFT81
DIDO1
PCNT
SAFB
CDK5RAP1
MAPK1
RABEP1
DPM1
FKBP3
FAN1
EEF1AKMT1
ZNF397
PRPF6
HKR1
MORC3
TBK1
PRR14L
TMOD3
ADARB1
SCAF4
AP4E1
IST1
ZFP90
KIAA0355
DCAF7
UBE2O
C16orf45
USP7
NUP88

TAF1
EWSR1
SOX9-AS1
ILF3
TM9SF4
ZNF44
TRPM7
COG7
TTC28-AS1
CAND1
PIK3C3
SPART
TP53BP1
AP2B1
BRWD3
TRIP11
RRAGB
ZNF334
ATP5F1A
ATG2B
ARID2
FHL1
PCMTD2
MCM3AP
GALC
LTN1
ADAM10
IQCH
RB1
RIOK3
ESF1
ZNF471
TMLHE
ICE2
ZNF81
VPS53
UPF3B
ARHGEF40
AC138207.8
ELP2
HPS4
AQR
TRAPPC10
CDKL5
SNX1
CDC42BPB
LRCH2
TPP2
MBTPS1
MED14
ALG13
CASC4
GPATCH2L
FBXO7
WNK3
AAGAB
KRBOX4
APPBP2
NAE1
ZNF529
GSPT1
NAA16
ZNF155
AL162171.1
PITPNB
DIP2A
KDSR
PHF8
UBA2
GPALPP1
ARMCX5-GPRASP2
TMEM50B
CDS2
CDK12
SMAD4
SOS2
FBXW8
APOOL
TARSL2
RTTN
ZNF160
GABPA
NCBP3
ZNF24
SELENOO
GRIPAP1
DDX42
IFT81
DIDO1
PCNT
SAFB
CDK5RAP1
MAPK1
RABEP1
DPM1
FKBP3
FAN1
EEF1AKMT1
ZNF397
PRPF6
HKR1
MORC3
TBK1
TMOD3
ADARB1
SCAF4
AP4E1
ZFP90
KIAA0355
DCAF7
UBE2O
C16orf45
USP7
NUP88

DDX42
IFT81
DIDO1
PCNT
SAFB
MAPK1
RABEP1
DPM1
FAN1
EEF1AKMT1
ZNF397
PRPF6
MORC3
TBK1
TMOD3
ADARB1
SCAF4
AP4E1
ZFP90
KIAA0355
DCAF7
UBE2O
C16orf45
USP7
NUP88

ZFP90
KIAA0355
DCAF7
UBE2O
C16orf45
USP7
NUP88

Supplementary Table 4_Astrocyte MEGENA modules
Module description

MEGENA Gene Co-expression Module description

Co-expression module	Module size	Parent module	Hub genes (number of edges in parentheses)
**c1_3**	1260	c1_1	MALAT1(965),FP236383.1(411),PCDH9-AS2(134),FTH1(129),HEPN1(129),AQP4(98),APOE(93),ZNF518A(89),ZDHHC21(68),PNISR(62),CKB(58),LUC7L3(32),ARGLU1(31),AC079385.1(26),SRSF5(26),AC012404.1(23),ITM2C(17),B4GALNT4(15),AP000462.1(14)
**c1_4**	157	c1_2	XIST(91),LINGO1(83),UTY(50),SLC26A3(39),RASGEF1B(25)
**c1_5**	530	c1_2	SLC1A2(213),ATP1A2(101),SLC1A3(87),SLC4A4(47),FGFR3(44),GRIA2(43),C1orf61(39),ANKDD1A(36),DLC1(32),PRODH(30),GLUL(26),MAST4(16),PIK3C2A(16),NAT8L(15),TPCN1(15),SMAD9(14),PLEKHA5(14),TTYH1(14),NRP1(13)
**c1_6**	907	c1_2	NRXN1(304),CADM2(158),DCLK1(148),GPM6A(93),CD44(74),SLC38A1(68),GPC5(48),LRP1B(47),FLRT2(30),ADGRB3(29),AC092691.1(26),GNA14(25),RORA(25),CABLES1(24),GFAP(24),CADM1(23),NCKAP5(23),ETNPPL(22),ERBB4(21),PLCB1(21),DNAH7(20),CLASP2(20),MAGI2(20),PCDH9(19),GABRB1(19),LSAMP(18),ASPH(18),LINC01088(17),LMO3(17),MEF2C-AS1(17),AC073941.1(16),FAM155A(16),RANBP3L(16),ARHGAP24(15),MAN1C1(15),ADAMTSL3(15),PDE4D(15),NKAIN3(15),KCND3(15),ADGRV1(15),TNC(14),SOX5(14),GPD2(14),EMP1(14)
**c1_8**	540	c1_2	ZNF98(197),DPP10(196),COL5A3(98),HPSE2(75),CACNB2(66),NDRG2(58),LINC00609(51),HIF3A(32),LINC00299(27),VCAN(23),TRPM3(22),RGS20(22),NR2F2-AS1(21),SLC39A11(21),WDR49(16),OLFM2(16),KCNIP1(16),ADCY2(16),ALDH1A1(15)
**c1_9**	307	c1_2	CRYAB(107),HSP90AA1(72),MT2A(68),CLU(60),HSPA1A(47),MT3(25),VEGFA(25),GJA1(23),UBC(20),FOS(15),HMGB1(15),GAPDH(15)
**c1_10**	688	c1_2	F3(252),CHI3L1(216),ITPR2(180),CAMK2G(72),ANGPTL4(69),DTNA(59),ATP1B2(53),CADPS(41),NRCAM(38),PTPRZ1(38),SAMD4A(35),APC(35),BCL6(29),ACOT11(23),PFKFB3(20),LINC02649(16),AHCYL1(14)
**c1_11**	224	c1_2	TPST1(164),ARHGEF3(72),SLC44A3-AS1(46),DST(30),FAM189A2(23),MACF1(21),BAALC-AS1(21),MAPRE2(20),OSMR(15)
**c1_12**	517	c1_2	NEAT1(424),HSPB1(109),AC091826.2(83),LINC01748(67),RFX3-AS1(47),NPAS2(39),SLC38A2(24),ZMAT1(24),AC138627.1(22),CRB1(19),AC104596.1(17),CIRBP(16),HSPH1(15),AC002429.2(15)
**c1_13**	100	c1_3	APOE(87),CKB(58),ITM2C(17),B4GALNT4(15)
**c1_16**	1127	c1_3	MALAT1(965),FP236383.1(411),PCDH9-AS2(134),HEPN1(128),AQP4(98),FTH1(92),ZNF518A(89),ZDHHC21(68),PNISR(62),LUC7L3(32),ARGLU1(31),AC079385.1(26),SRSF5(26),AC012404.1(23)
**c1_17**	83	c1_4	LINGO1(79),SLC26A3(36),RASGEF1B(18)
**c1_18**	74	c1_4	XIST(67),UTY(46)
**c1_19**	308	c1_5	SLC1A2(209),SLC4A4(47),GRIA2(43),DLC1(32),GLUL(18),PIK3C2A(16),SMAD9(14),PLEKHA5(14),CFAP54(12)
**c1_21**	76	c1_5	SLC1A3(62),MAST4(16),NRP1(12)
**c1_22**	96	c1_5	ATP1A2(86),C1orf61(26),FGFR3(15),TPCN1(15),TTYH1(14)
**c1_23**	45	c1_5	ANKDD1A(35),PRODH(24)
**c1_24**	201	c1_6	DCLK1(125),SLC38A1(66),GNA14(25),PLCB1(21),CABLES1(18),LINC01088(17),AC073941.1(16),ADAMTSL3(15),KCND3(15),TNC(14)
**c1_25**	706	c1_6	NRXN1(303),CADM2(158),GPM6A(93),CD44(73),LRP1B(47),GPC5(42),FLRT2(30),ADGRB3(29),AC092691.1(26),RORA(25),GFAP(24),NCKAP5(23),ETNPPL(22),ERBB4(21),CADM1(20),DNAH7(20),CLASP2(20),MAGI2(20),PCDH9(19),GABRB1(19),LSAMP(18),ASPH(18),LMO3(17),MEF2C-AS1(17),FAM155A(16),RANBP3L(16),PDE4D(15),NKAIN3(15),ADGRV1(15),ARHGAP24(14),MAN1C1(14),SOX5(14),GPD2(14),EMP1(14)
**c1_26**	312	c1_8	DPP10(187),COL5A3(68),LINC00609(51),CACNB2(41),VCAN(23),TRPM3(21),WDR49(16),ADCY2(16),OLFM2(15)
**c1_27**	183	c1_8	ZNF98(155),HPSE2(28),LINC00299(27),RGS20(22),NR2F2-AS1(21),ALDH1A1(15)
**c1_28**	45	c1_8	HIF3A(28),NDRG2(27),SLC39A11(21)
**c1_29**	236	c1_9	CRYAB(106),HSP90AA1(72),MT2A(67),HSPA1A(46),MT3(24),UBC(20),FOS(15),HMGB1(15)
**c1_30**	48	c1_9	CLU(33),GJA1(23)
**c1_32**	236	c1_10	F3(185),ATP1B2(53),ANGPTL4(52)
**c1_33**	300	c1_10	CHI3L1(213),CAMK2G(72),DTNA(39),CADPS(37),SAMD4A(35),ACOT11(23),BCL6(17)
**c1_34**	151	c1_10	ITPR2(143),NRCAM(37),APC(35),PTPRZ1(31)
**c1_36**	208	c1_11	TPST1(164),ARHGEF3(61),SLC44A3-AS1(46),DST(26),FAM189A2(22),BAALC-AS1(21),MAPRE2(20),MACF1(17)
**c1_37**	504	c1_12	NEAT1(424),HSPB1(109),AC091826.2(83),LINC01748(67),RFX3-AS1(47),NPAS2(33),SLC38A2(24),ZMAT1(24),AC138627.1(22),CRB1(19),AC104596.1(17),CIRBP(16),HSPH1(15),AC002429.2(15)
**c1_41**	39	c1_16	AC012404.1(20),LINC00513(11)
**c1_46**	1038	c1_16	MALAT1(965),FP236383.1(411),AQP4(98),PCDH9-AS2(96),ZNF518A(89),ZDHHC21(68),HEPN1(66),FTH1(65),PNISR(62),LUC7L3(32),ARGLU1(31),AC079385.1(23),SRSF5(23)
**c1_47**	282	c1_19	SLC1A2(209),SLC4A4(47),GRIA2(43),DLC1(31),PIK3C2A(16),PLEKHA5(14)
**c1_51**	188	c1_24	DCLK1(125),SLC38A1(66),PLCB1(21),CABLES1(18),LINC01088(17),AC073941.1(16),ADAMTSL3(15)
**c1_53**	550	c1_25	NRXN1(301),GPM6A(91),GPC5(41),CD44(30),ADGRB3(27),AC092691.1(25),RORA(25),GFAP(24),NCKAP5(23),ETNPPL(22),CADM1(20),DNAH7(20),PCDH9(19),GABRB1(19),ASPH(18),LMO3(17),MEF2C-AS1(17),RANBP3L(16),PDE4D(15),NKAIN3(15),FAM155A(15),ADGRV1(15),ARHGAP24(14),SOX5(14),GPD2(14),PREX2(13),ATP13A4(13),BMPR1B(13)
**c1_54**	105	c1_25	CADM2(83),LRP1B(29),FLRT2(29),CLASP2(14)
**c1_61**	231	c1_26	DPP10(186),CACNB2(40),LINC00609(26),VCAN(23),WDR49(16),ADCY2(15)
**c1_62**	46	c1_26	COL5A3(45)
**c1_65**	27	c1_28	HIF3A(26),NDRG2(15)
**c1_66**	139	c1_29	CRYAB(106),HSP90AA1(53),MT2A(35),HSPA1A(29),UBC(17)
**c1_78**	34	c1_30	CLU(32)
**c1_80**	211	c1_32	F3(185),ATP1B2(53),ANGPTL4(29)
**c1_83**	260	c1_33	CHI3L1(212),DTNA(39),CADPS(36),SAMD4A(35),CAMK2G(35),BCL6(17),CNTN1(13)
**c1_86**	203	c1_36	TPST1(164),ARHGEF3(61),SLC44A3-AS1(46),DST(26),FAM189A2(22),BAALC-AS1(21),MAPRE2(18),MACF1(17)
**c1_89**	27	c1_41	AC012404.1(20)
**c1_92**	1020	c1_46	MALAT1(965),FP236383.1(393),AQP4(98),PCDH9-AS2(96),ZNF518A(89),HEPN1(66),FTH1(65),PNISR(62),ZDHHC21(51),LUC7L3(32),ARGLU1(31),AC079385.1(23),SRSF5(23)
**c1_95**	28	c1_51	SLC38A1(22),DPP6(9)
**c1_96**	149	c1_51	DCLK1(121),AC073941.1(16),LINC01088(14),ADAMTSL3(14)
**c1_99**	483	c1_53	NRXN1(301),GPM6A(69),GPC5(41),CD44(30),ADGRB3(27),GFAP(24),NCKAP5(23),CADM1(20),DNAH7(20),PCDH9(19),GABRB1(19),ASPH(18),RORA(18),LMO3(17),PDE4D(15),NKAIN3(15),FAM155A(15),AC092691.1(15),ADGRV1(15),ARHGAP24(14),SOX5(14),ETNPPL(14),GPD2(14),PREX2(13),ATP13A4(13)
**c1_106**	224	c1_61	DPP10(186),CACNB2(40),LINC00609(26),VCAN(23),WDR49(16)
**c1_108**	132	c1_66	CRYAB(106),HSP90AA1(52),MT2A(35),HSPA1A(29)
**c1_112**	241	c1_83	CHI3L1(212),CADPS(36),CAMK2G(35),SAMD4A(31),DTNA(20),BCL6(17),CNTN1(13)
**c1_117**	1010	c1_92	MALAT1(965),FP236383.1(393),PCDH9-AS2(96),AQP4(96),ZNF518A(89),HEPN1(66),FTH1(65),PNISR(54),ZDHHC21(51),ARGLU1(31),LUC7L3(24),AC079385.1(23),SRSF5(23)
**c1_119**	437	c1_99	NRXN1(301),GPM6A(69),GPC5(35),CD44(30),NCKAP5(23),CADM1(20),ADGRB3(18),ASPH(18),GABRB1(17),DNAH7(17),LMO3(17),PDE4D(15),NKAIN3(15),GFAP(15),AC092691.1(15),ADGRV1(15),ARHGAP24(14),SOX5(14),ETNPPL(14),GPD2(14),ATP13A4(13)

Supplementary Table 4_Astrocyte MEGENA modules
Module 9 FE

geneset	description	size	overlap	odds_ratio	pval	FDR	database	X.Loe10.FDR.	genes
GO:0060050	positive regulation of protein glycosylation	6	3	64.77	0.000069	0.0033	GO	2.4815	GOLGA2;CHORDC1;RAMP1
R-HSA-5660526	Response to metal ions	11	5	54.32	0.00000035	0.000036	Reactome	4.444	MT2A;MT1M;MT1F;MT1G;MT3
R-HSA-5661231	Metallothioneins bind metals	11	5	54.32	0.00000035	0.000036	Reactome	4.444	MT2A;MT1M;MT1F;MT1G;MT3
GO:0051131	chaperone-mediated protein complex assembly	17	7	45.93	3.2E-09	0.00000086	GO	6.0655	HSP90AA1;HSP90AB1;HSPA4;PTGES3;CLU;HSPD1;HSPA1A
GO:0097201	negative regulation of transcription from RNA polymerase II promoter in response to stress	10	4	43.32	0.000011	0.00091	GO	3.041	DNAJB1;JUN;BAG3;HSPA1A
GO:0051085	chaperone mediated protein folding requiring cofactor	24	9	39.62	4.5E-11	3.2E-08	GO	7.4949	HSPA9:HSPA8:DNAJB1:HSPA5:DNAJC7:HSPA4:PTGES3:HSPA4L:HSPA1A
GO:0060049	regulation of protein glycosylation	12	4	32.48	0.000024	0.0015	GO	2.8239	GOLGA2;CHORDC1;MT3;RAMP1
GO:0090190	positive regulation of branching involved in ureteric bud morphogenesis	12	4	32.48	0.000024	0.0015	GO	2.8239	SMO;SOX9;AGT;VEGFA
R-HSA-3000484	Scavenging by Class F Receptors	6	2	32.28	0.0034	0.041	Reactome	1.387	HSP90AA1;CALR
R-HSA-5336415	Uptake and function of diphtheria toxin	6	2	32.28	0.0034	0.041	Reactome	1.387	HSP90AA1;HSP90AB1
WP3943	Robo4 and VEGF Signaling Pathways Crosstalk	6	2	32.28	0.0034	0.031	Wikipathway	1.5086	RAC1;VEGFA
R-HSA-3371511	HSF1 activation	29	9	29.71	3.2E-10	6.5E-08	Reactome	7.187	YWHAE;EEF1A1;DNAJB1;HSP90AA1;HSP90AB1;DNAJB6;UBB;PTGES3;HSPA1A
R-HSA-3371568	Attenuation phase	26	8	29.25	3.5E-09	0.00000056	Reactome	6.252	DNAJB1;HSPA8;HSP90AA1;HSP90AB1;DNAJB6;UBB;PTGES3;HSPA1A
GO:0051084	'de novo' posttranslational protein folding	30	9	28.29	4.5E-10	0.00000019	GO	6.7212	HSPA9;DNAJB1;HSPA8;HSPA5;DNAJC7;HSPA4;PTGES3;HSPA4L;HSPA1A
GO:0090084	negative regulation of inclusion body assembly	10	3	27.75	0.0004	0.013	GO	1.8861	DNAJB1;DNAJB6;HSPA1A
R-HSA-451308	Activation of Ca-permeable Kainate Receptor	10	3	27.75	0.0004	0.013	Reactome	1.886	GRIK5;GRIK2;CALM1
R-HSA-451306	Ionotropic activity of Kainate Receptors	10	3	27.75	0.0004	0.013	Reactome	1.886	GRIK5;GRIK2;CALM1
R-HSA-5663084	Diseases of carbohydrate metabolism	10	3	27.75	0.0004	0.013	Reactome	1.886	GYS1;UBB;UBC
R-HSA-3785653	Myoclonic epilepsy of Lafora	10	3	27.75	0.0004	0.013	Reactome	1.886	GYS1;UBB;UBC
R-HSA-3229121	Glycogen storage diseases	10	3	27.75	0.0004	0.013	Reactome	1.886	GYS1;UBB;UBC
R-HSA-390450	Folding of actin by CCT/TriC	10	3	27.75	0.0004	0.013	Reactome	1.886	CCT3;ACTB;CCT4
GO:0071280	cellular response to copper ion	21	6	26.15	0.00000056	0.000092	GO	4.0362	PRNP;MT2A;MT1M;MT1F;MT1G;MT3
GO:0034620	cellular response to unfolded protein	22	6	24.51	0.00000076	0.00012	GO	3.9208	HSPA9;HSPA8;HSPA5;HSPA4;HSPA4L;HSPA1A
GO:0090083	regulation of inclusion body assembly	11	3	24.28	0.00054	0.016	GO	1.7959	DNAJB1;DNAJB6;HSPA1A
GO:2001044	regulation of integrin-mediated signaling pathway	11	3	24.28	0.00054	0.016	GO	1.7959	CD63;FLNA;PRKD1
GO:0010560	positive regulation of glycoprotein biosynthetic process	11	3	24.28	0.00054	0.016	GO	1.7959	GOLGA2;CHORDC1;RAMP1
GO:0090189	regulation of branching involved in ureteric bud morphogenesis	15	4	23.62	0.000065	0.0032	GO	2.4949	SMO;SOX9;AGT;VEGFA
GO:0061213	positive regulation of mesonephros development	15	4	23.62	0.000065	0.0032	GO	2.4949	SMO;SOX9;AGT;VEGFA
GO:0071294	cellular response to zinc ion	19	5	23.27	0.000008	0.00072	GO	3.1427	MT2A;MT1M;MT1F;MT1G;MT3
GO:0006986	response to unfolded protein	43	11	22.83	3.5E-11	3.2E-08	GO	7.4949	HSPA9;DNAJA1;DNAJB1;HSPA8;HSP90AA1;HSP90AB1;HSPA5;HSPA4;HSPA4L;HSPD1;HSPA1A
WP1425	Bone Morphogenic Protein (BMP) Signalling and Regulation	12	3	21.58	0.00071	0.011	Wikipathway	1.9586	SMAD1;TOB2;TOB1
GO:0071276	cellular response to cadmium ion	29	7	20.86	0.00000022	0.000047	GO	4.3279	MT2A;JUN;MT1M;MT1F;MT1G;FOS;MT3
GO:0046688	response to copper ion	25	6	20.64	0.0000017	0.00025	GO	3.6021	PRNP;MT2A;MT1M;MT1F;MT1G;MT3
WP1528	Physiological and Pathological Hypertrophy of the Heart	25	6	20.64	0.0000017	0.00016	Wikipathway	3.7959	JUN;RAC1;FOS;CALM1;RHOA;AGT
R-HSA-3371571	HSF1-dependent transactivation	34	8	20.24	3.6E-08	0.0000049	Reactome	5.31	DNAJB1;HSPA8;HSP90AA1;HSP90AB1;DNAJB6;UBB;PTGES3;HSPA1A
GO:0010939	regulation of necrotic cell death	13	3	19.42	0.00091	0.024	GO	1.6198	UBB;UBC;MT3
GO:0046686	response to cadmium ion	31	7	19.12	0.00000036	0.00007	GO	4.1549	MT2A;JUN;MT1M;MT1F;MT1G;FOS;MT3
GO:0035967	cellular response to topologically incorrect protein	27	6	18.67	0.0000028	0.00038	GO	3.4202	HSPA9;HSPA8;HSPA5;HSPA4;HSPA4L;HSPA1A
GO:1905332	positive regulation of morphogenesis of an epithelium	18	4	18.56	0.00014	0.0058	GO	2.2366	SMO;SOX9;AGT;VEGFA
GO:1903320	regulation of protein modification by small protein conjugation or removal	14	3	17.66	0.0011	0.028	GO	1.5528	HSP90AA1;HSP90AB1;HSPA1A
R-HSA-1236382	Constitutive Signaling by Ligand-Responsive EGFR Cancer Variants	19	4	17.32	0.00018	0.0095	Reactome	2.022	HSP90AA1;UBB;UBC;SOS1
R-HSA-5637815	Signaling by Ligand-Responsive EGFR Variants in Cancer	19	4	17.32	0.00018	0.0095	Reactome	2.022	HSP90AA1;UBB;UBC;SOS1
R-HSA-1643713	Signaling by EGFR in Cancer	19	4	17.32	0.00018	0.0095	Reactome	2.022	HSP90AA1;UBB;UBC;SOS1
GO:0061620	glycolytic process through glucose-6-phosphate	25	5	16.29	0.000034	0.0019	GO	2.7212	GPI;PKM;PGK1;ENO1;GAPDH
GO:0061621	canonical glycolysis	25	5	16.29	0.000034	0.0019	GO	2.7212	GPI;PKM;PGK1;ENO1;GAPDH
GO:0061718	glucose catabolic process to pyruvate	25	5	16.29	0.000034	0.0019	GO	2.7212	GPI;PKM;PGK1;ENO1;GAPDH
GO:0071384	cellular response to corticosteroid stimulus	15	3	16.19	0.0014	0.033	GO	1.4815	HNRNPU;ZFP36L1;UBE2L3
R-HSA-69298	Association of licensing factors with the pre-replicative complex	15	3	16.19	0.0014	0.028	Reactome	1.553	UBB;UBC;E2F3
R-HSA-2691232	Constitutive Signaling by NOTCH1 HD Domain Mutants	15	3	16.19	0.0014	0.028	Reactome	1.553	UBB;UBC;MIB1
R-HSA-2691230	Signaling by NOTCH1 HD Domain Mutants in Cancer	15	3	16.19	0.0014	0.028	Reactome	1.553	UBB;UBC;MIB1
R-HSA-3322077	Glycogen synthesis	15	3	16.19	0.0014	0.028	Reactome	1.553	GYS1;UBB;UBC
GO:0055069	zinc ion homeostasis	31	6	15.68	0.0000066	0.00065	GO	3.1871	MT2A;MT1M;MT1F;MT1G;SLC30A1;MT3
GO:0006882	cellular zinc ion homeostasis	31	6	15.68	0.0000066	0.00065	GO	3.1871	MT2A;MT1M;MT1F;MT1G;SLC30A1;MT3
R-HSA-3371556	Cellular response to heat stress	96	18	15.66	6.3E-15	5.1E-12	Reactome	11.292	YWHAE;HSPA9;HSPA8;HSP90AA1;HSP90AB1;HSPA5;HSPA4;ST13;PTGES3;HSPA4L;EEF1A1;DNAJB1;DNAJC7;DNAJB6;UBB;POM121C;BAG3;HSPA1A
WP3614	Photodynamic therapy-induced HIF-1 survival signaling	37	7	15.29	0.0000013	0.00016	Wikipathway	3.7959	EGLN1;BNIP3L;PKM;BNIP3;PGK1;MCL1;VEGFA
GO:0010043	response to zinc ion	32	6	15.08	0.0000081	0.00072	GO	3.1427	MT2A;MT1M;MT1F;MT1G;SLC30A1;MT3
GO:1902229	regulation of intrinsic apoptotic signaling pathway in response to DNA damage	16	3	14.94	0.0017	0.038	GO	1.4202	ACKR3;CLU;TPT1
R-HSA-399954	Sema3A PAK dependent Axon repulsion	16	3	14.94	0.0017	0.028	Reactome	1.553	HSP90AA1;HSP90AB1;RAC1
R-HSA-5625900	RHO GTPases activate CIT	16	3	14.94	0.0017	0.028	Reactome	1.553	RAC1;RHOA;RHOB
R-HSA-2173791	TGF-beta receptor signaling in EMT (epithelial to mesenchymal transition)	16	3	14.94	0.0017	0.028	Reactome	1.553	UBB;UBC;RHOA
WP1946	Cori Cycle	16	3	14.94	0.0017	0.022	Wikipathway	1.6576	GPI;PGK1;GAPDH
R-HSA-3371453	Regulation of HSF1-mediated heat shock response	80	14	14.21	1.8E-11	4.9E-09	Reactome	8.31	YWHAE;HSPA9;HSPA8;HSPA5;HSPA4;ST13;HSPA4L;DNAJB1;DNAJC7;DNAJB6;UBB;POM121C;BAG3;HSPA1A
GO:0014074	response to purine-containing compound	17	3	13.87	0.0021	0.042	GO	1.3768	JUN;JUND;JUNB
GO:0071385	cellular response to glucocorticoid stimulus	17	3	13.87	0.0021	0.042	GO	1.3768	HNRNPU;ZFP36L1;UBE2L3
R-HSA-532668	N-glycan trimming in the ER and Calnexin/Calreticulin cycle	17	3	13.87	0.0021	0.03	Reactome	1.523	CANX;MLEC;CALR
GO:0050771	negative regulation of axonogenesis	23	4	13.67	0.00038	0.013	GO	1.8861	GDI1;LRP4;MT3;RHOA
GO:0034605	cellular response to heat	47	8	13.48	0.00000053	0.000092	GO	4.0362	YWHAE;HSPA9;HSPA8;HSPA5;BAG3;HSPA4;HSPA4L;HSPA1A
GO:1900034	regulation of cellular response to heat	78	13	13.35	1.8E-10	9.9E-08	GO	7.0044	YWHAE;HSPA8;HSP90AA1;HSP90AB1;PTGES3;DNAJB1;DNAJC7;DNAJB6;POM121C;BAG3;CHORDC1;CRYAB;HSPA1A
WP3529	Zinc homeostasis	36	6	13.07	0.000016	0.00076	Wikipathway	3.1192	MT2A;MT1M;MT1F;MT1G;SLC30A1;MT3
GO:0042398	cellular modified amino acid biosynthetic process	24	4	12.99	0.00045	0.015	GO	1.8239	PTDSS2;PLOD3;PLOD2;HAGH
GO:0009250	glucan biosynthetic process	18	3	12.95	0.0025	0.046	GO	1.3372	GYS1;UBB;UBC
GO:0005978	glycogen biosynthetic process	18	3	12.95	0.0025	0.046	GO	1.3372	GYS1;UBB;UBC
GO:0046683	response to organophosphorus	18	3	12.95	0.0025	0.046	GO	1.3372	JUN;JUND;JUNB
GO:0050849	negative regulation of calcium-mediated signaling	18	3	12.95	0.0025	0.046	GO	1.3372	PRNP;CHORDC1;CALM1
R-HSA-1234176	Oxygen-dependent proline hydroxylation of Hypoxia-inducible Factor Alpha	18	3	12.95	0.0025	0.033	Reactome	1.481	EGLN1;UBB;UBC
GO:0021762	substantia nigra development	43	7	12.74	0.0000038	0.00047	GO	3.3279	YWHAE;HSPA5;S100A1;DYNLL1;CALM1;ACTB;RHOA
R-HSA-983170	Antigen Presentation: Folding, assembly and peptide loading of class I MHC	25	4	12.37	0.00053	0.017	Reactome	1.77	HSPA5;CANX;HLA-A;CALR
GO:0034109	homotypic cell-cell adhesion	38	6	12.25	0.000023	0.0015	GO	2.8239	CSRP1;TYRO3;GNAS;FLNA;PLPP3;ACTB
WP4300	Extracellular vesicles in the crosstalk of cardiac cells	19	3	12.14	0.0029	0.029	Wikipathway	1.5376	CD63;CD81;SOD1
R-HSA-70171	Glycolysis	32	5	12.06	0.00012	0.0087	Reactome	2.06	GPI;PKM;PGK1;ENO1;GAPDH
GO:0043277	apoptotic cell clearance	26	4	11.8	0.00062	0.018	GO	1.7447	SCARB1;TYRO3;HMGB1;RAC1
R-HSA-1234174	Regulation of Hypoxia-inducible Factor (HIF) by oxygen	26	4	11.8	0.00062	0.017	Reactome	1.77	EGLN1;UBB;UBC;VEGFA
R-HSA-2262749	Cellular response to hypoxia	26	4	11.8	0.00062	0.017	Reactome	1.77	EGLN1;UBB;UBC;VEGFA
GO:0050768	negative regulation of neurogenesis	33	5	11.63	0.00014	0.0058	GO	2.2366	ID2;ID4;ID3;PAX6;CALR
GO:0070527	platelet aggregation	33	5	11.63	0.00014	0.0058	GO	2.2366	CSRP1;TYRO3;GNAS;FLNA;ACTB
GO:0051223	regulation of protein transport	27	4	11.29	0.00072	0.02	GO	1.699	SNX3;DNAJA1;HSPA8;CHORDC1
R-HSA-389957	Prefoldin mediated transfer of substrate to CCT/TriC	27	4	11.29	0.00072	0.02	Reactome	1.699	CCT3;TUBA1A;ACTB;CCT4
GO:0043392	negative regulation of DNA binding	34	5	11.23	0.00016	0.0063	GO	2.2007	JUN;SMO;ID2;ID4;ID3
GO:0046916	cellular transition metal ion homeostasis	93	13	10.84	1.8E-09	0.00000054	GO	6.2676	PRNP:MT1M:CYBRD1:SLC30A1:MT3:SOD1:MT2A:UBB:SLC22A17:UBC:MT1F:MT1G:FTL
R-HSA-2979096	NOTCH2 Activation and Transmission of Signal to the Nucleus	21	3	10.79	0.0039	0.043	Reactome	1.367	UBB;UBC;MIB1
R-HSA-5627123	RHO GTPases activate PAKs	21	3	10.79	0.0039	0.043	Reactome	1.367	FLNA;RAC1;CALM1
GO:0080135	regulation of cellular response to stress	104	14	10.41	6.9E-10	0.00000025	GO	6.6021	YWHAE;HSPA8;HSP90AA1;HSP90AB1;PTGES3;DNAJB1;POM121C;DNAJC7;DNAJB6;BAG3;CHORDC1;CRYAB;MCL1;HSPA1A
GO:0036003	positive regulation of transcription from RNA polymerase II promoter in response to stress	29	4	10.39	0.00096	0.024	GO	1.6198	CEBPB;HSPA5;MT3;VEGFA
WP3286	Copper homeostasis	52	7	10.19	0.000014	0.00076	Wikipathway	3.1192	PRNP;MT2A;JUN;MT1F;MT1G;MT3;SOD1
R-HSA-5620922	BBSome-mediated cargo-targeting to cilium	23	3	9.71	0.0051	0.049	Reactome	1.31	CCT3;SMO;CCT4
WP712	Estrogen signaling pathway	23	3	9.71	0.0051	0.04	Wikipathway	1.3979	JUN;GNAS;FOS
WP2526	PDGF Pathway	39	5	9.57	0.00031	0.0071	Wikipathway	2.1487	JUN;RAC1;FOS;SOS1;RHOA
GO:0010941	regulation of cell death	96	12	9.5	2.7E-08	0.0000065	GO	5.1871	BNIP3L;JUN;JUND;UBB;BNIP3;CHORDC1;PRKD1;CLU;JUNB;CRYAB;MT3;HSPA1A
GO:0036294	cellular response to decreased oxygen levels	64	8	9.38	0.0000059	0.00065	GO	3.1871	BNIP3L;FAM162A;BNIP3;HP1BP3;PGK1;MT3;ZFP36L1;VEGFA
R-HSA-70263	Gluconeogenesis	32	4	9.27	0.0014	0.028	Reactome	1.553	GPI;PGK1;ENO1;GAPDH
R-HSA-389958	Cooperation of Prefoldin and TriC/CCT in actin and tubulin folding	32	4	9.27	0.0014	0.028	Reactome	1.553	CCT3;TUBA1A;ACTB;CCT4
WP3612	Photodynamic therapy-induced NFE2L2 (NRF2) survival signaling	24	3	9.24	0.0057	0.043	Wikipathway	1.3665	JUN;EPHX1;FOS
GO:0050821	protein stabilization	152	18	9.09	2.2E-11	3.2E-08	GO	7.4949	CCT3;HSP90AA1;HSP90AB1;UBE2B;PTGES3;CLU;MT3;HSPD1;LAMP1;CHORDC1;FLNA;CALR;SUGT1;PLPP3;CRYAB;GAPDH;CCT4;HSPA1A
GO:0005977	glycogen metabolic process	33	4	8.95	0.0016	0.035	GO	1.4559	GYS1;UBB;UBC;CALM1
WP4290	Metabolic reprogramming in colon cancer	42	5	8.8	0.00044	0.0093	Wikipathway	2.0315	GPI;PKM;PGK1;ENO1;GAPDH
GO:0051248	negative regulation of protein metabolic process	43	5	8.56	0.00049	0.016	GO	1.7959	SNX3;PRNP;FLNA;CLU;CRYAB
GO:0051101	regulation of DNA binding	52	6	8.51	0.00014	0.0058	GO	2.2366	JUN;SMO;ID2;ID4;ID3;HMGB1
GO:0010595	positive regulation of endothelial cell migration	70	8	8.47	0.000012	0.00092	GO	3.0362	SCARB1;HMGB1;PRKD1;CALR;PLPP3;AGT;RHOB;VEGFA
R-HSA-70326	Glucose metabolism	79	9	8.47	0.0000034	0.0003	Reactome	3.523	GPI;GYS1;PKM;UBB;UBC;PGK1;ENO1;CALM1;GAPDH
WP2359	Parkin-Ubiquitin Proteasomal System pathway	70	8	8.47	0.000012	0.00076	Wikipathway	3.1192	HSPA9;HSPA8;TUBA1A;HSPA5;HSPA4;UBA1;UBE2L3;HSPA1A
GO:0051973	positive regulation of telomerase activity	35	4	8.37	0.002	0.041	GO	1.3872	HSP90AA1;HSP90AB1;PTGES3;CCT4
GO:2001243	negative regulation of intrinsic apoptotic signaling pathway	62	7	8.33	0.000045	0.0025	GO	2.6021	DNAJA1;ACKR3;ENO1;CLU;TPT1;MCL1;HSPA1A
R-HSA-1227986	Signaling by ERBB2	45	5	8.13	0.00061	0.017	Reactome	1.77	HSP90AA1;UBB;UBC;SOS1;RHOA
WP534	Glycolysis and Gluconeogenesis	45	5	8.13	0.00061	0.011	Wikipathway	1.9586	GPI;PKM;PGK1;ENO1;GAPDH
GO:0050999	regulation of nitric-oxide synthase activity	36	4	8.11	0.0022	0.043	GO	1.3665	SCARB1;HSP90AA1;S100A1;CALM1
WP2873	Aryl Hydrocarbon Receptor Pathway	46	5	7.94	0.00068	0.011	Wikipathway	1.9586	JUN;HSP90AA1;JUND;PTGES3;JUNB
GO:0006879	cellular iron ion homeostasis	56	6	7.83	0.00021	0.0079	GO	2.1024	UBB;SLC22A17;UBC;CYBRD1;FTL;SOD1
GO:0010942	positive regulation of cell death	57	6	7.68	0.00023	0.0084	GO	2.0757	PRNP;BNIP3;DDIT4;FOS;CLU;MT3
GO:1901216	positive regulation of neuron death	38	4	7.63	0.0027	0.049	GO	1.3098	PRNP;DDIT4;FOS;CLU
GO:0001895	retina homeostasis	38	4	7.63	0.0027	0.049	GO	1.3098	WHRN;PRDX1;ACTB;SOD1
WP524	G13 Signaling Pathway	39	4	7.41	0.0029	0.029	Wikipathway	1.5376	GNA13;RAC1;CALM1;RHOA
WP3611	Photodynamic therapy-induced AP-1 survival signaling.	50	5	7.23	0.00099	0.014	Wikipathway	1.8539	HSP90AA1;JUN;FOS;JUNB;MCL1
R-HSA-5620924	Intraflagellar transport	41	4	7.01	0.0035	0.042	Reactome	1.377	IFT140;IFT172;DYNLRB1;DYNLL1
GO:0055072	iron ion homeostasis	63	6	6.87	0.00041	0.013	GO	1.8861	UBB;SLC22A17;UBC;CYBRD1;FTL;SOD1
GO:0010634	positive regulation of epithelial cell migration	74	7	6.83	0.00014	0.0058	GO	2.2366	SCARB1;SOX9;PRKD1;CALR;AGT;RHOB;VEGFA
WP4018	Pathways in clear cell renal cell carcinoma	85	8	6.82	0.000049	0.0019	Wikipathway	2.7212	GPI;PKM;PGK1;PLOD2;PHGDH;ENO1;GAPDH;VEGFA
R-HSA-917937	Iron uptake and transport	43	4	6.65	0.0042	0.044	Reactome	1.357	ATP6V0E1;SLC22A17;CYBRD1;FTL
WP364	IL-6 signaling pathway	43	4	6.65	0.0042	0.034	Wikipathway	1.4685	RAC1;SOS1;JUNB;AGT
GO:0051129	negative regulation of cellular component organization	54	5	6.64	0.0014	0.033	GO	1.4815	DNAJB1;HSPA8;DNAJB6;PRKD1;HSPA1A
WP2374	Oncostatin M Signaling Pathway	65	6	6.63	0.00048	0.0093	Wikipathway	2.0315	CEBPB;JUND;FOS;SOS1;JUNB;VEGFA
GO:0071248	cellular response to metal ion	100	9	6.51	0.000024	0.0015	GO	2.8239	PRNP;MT2A;JUN;BNIP3;MT1M;MT1F;MT1G;FOS;MT3
GO:0071560	cellular response to transforming growth factor beta stimulus	100	9	6.51	0.000024	0.0015	GO	2.8239	SMAD1;WWOX;JUN;UBB;UBC;SOX9;FOS;RHOA;ZFP36L1
GO:2000573	positive regulation of DNA biosynthetic process	55	5	6.5	0.0015	0.035	GO	1.4559	CCT3;HSP90AA1;HSP90AB1;PTGES3;CCT4
R-HSA-2262752	Cellular responses to stress	367	31	6.47	1.4E-14	5.6E-12	Reactome	11.252	YWHAE;CEBPB;HSP90AB1;HSPA4L;DNAJB1;POM121C;UBB;DNAJB6;BAG3;PRDX1;UBC;E2F3;HIST1H2AC;EGLN1;HSPA9;HSPA8;JUN;HSP90AA1;HSPA5;ST13;HSPA4;PTGES3;ATG10;FOS;DYNLL1;PRDX6;VEGFA;SOD1;EEF1A1;DNAJC7;HSPA1A
WP138	Androgen receptor signaling pathway	90	8	6.4	0.000073	0.0025	Wikipathway	2.6021	JUN;UBE2I;PRDX1;FLNA;RAC1;CALR;RHOA;RHOB
GO:0010594	regulation of endothelial cell migration	69	6	6.21	0.00066	0.019	GO	1.7212	SCARB1;PRKD1;CALR;AGT;RHOB;VEGFA
WP2355	Corticotropin-releasing hormone signaling pathway	93	8	6.17	0.000093	0.0026	Wikipathway	2.585	GJA1;HSP90AA1;JUND;GNAS;FOS;JUNB;RHOA;GNAI2
GO:0048010	vascular endothelial growth factor receptor signaling pathway	70	6	6.11	0.00072	0.02	GO	1.699	HSP90AA1;RAC1;PRKD1;ACTB;RHOA;VEGFA
GO:0051100	negative regulation of binding	82	7	6.1	0.00027	0.0092	GO	2.0362	GOLGA2;JUN;SMO;ID2;ID4;ID3;ACTB
GO:0072001	renal system development	59	5	6.02	0.0021	0.042	GO	1.3768	SMO;IFT140;LRP4;AGT;VEGFA
GO:0034329	cell junction assembly	59	5	6.02	0.0021	0.042	GO	1.3768	CDH11;TBCD;FLNA;ACTB;RHOA
GO:0071456	cellular response to hypoxia	132	11	6.01	0.0000062	0.00065	GO	3.1871	EGLN1;BNIP3L;UBB;FAM162A;BNIP3;UBC;HP1BP3;PGK1;MT3;ZFP36L1;VEGFA
GO:1901214	regulation of neuron death	72	6	5.93	0.00083	0.022	GO	1.6576	PRNP;UBB;CHORDC1;DDIT4;FOS;CLU
R-HSA-168180	TRAF6 Mediated Induction of proinflammatory cytokines	72	6	5.93	0.00083	0.022	Reactome	1.658	JUN;UBB;UBC;HMGB1;FOS;S100B
GO:0000302	response to reactive oxygen species	60	5	5.91	0.0023	0.044	GO	1.3565	EGLN1;JUN;FOS;APTX;SOD1
R-HSA-5625740	RHO GTPases activate PKNs	60	5	5.91	0.0023	0.032	Reactome	1.495	YWHAE;RAC1;HIST1H2AC;RHOA;RHOB
WP410	Exercise-induced Circadian Regulation	48	4	5.9	0.0062	0.044	Wikipathway	1.3565	DNAJA1;HSPA8;CEBPB;TOB1
GO:0034250	positive regulation of cellular amide metabolic process	61	5	5.81	0.0024	0.046	GO	1.3372	FXR1;RPS27L;VIM;CLU;ELAVL1
R-HSA-204998	Cell death signalling via NRAGE, NRIF and NADE	61	5	5.81	0.0024	0.033	Reactome	1.481	YWHAE;UBB;UBC;RAC1;SOS1
WP3646	Hepatitis C and Hepatocellular Carcinoma	49	4	5.76	0.0067	0.045	Wikipathway	1.3468	JUN;RAC1;SOS1;VEGFA
R-HSA-2565942	Regulation of PLK1 Activity at G2/M Transition	87	7	5.72	0.00038	0.013	Reactome	1.886	YWHAE;HSP90AA1;TUBA1A;UBB;UBC;ALMS1;DYNLL1
WP183	Proteasome Degradation	62	5	5.7	0.0026	0.028	Wikipathway	1.5528	UBB;UBE2B;UBC;UBA1;HLA-A
GO:0034249	negative regulation of cellular amide metabolic process	75	6	5.67	0.001	0.026	GO	1.585	FXR1;PRNP;CALR;CLU;GAPDH;TOB1
GO:0072503	cellular divalent inorganic cation homeostasis	101	8	5.64	0.00017	0.0063	GO	2.2007	MT2A;MT1M;MT1F;MT1G;SLC30A1;CALR;MT3;TPT1
GO:0007179	transforming growth factor beta receptor signaling pathway	76	6	5.59	0.0011	0.028	GO	1.5528	SMAD1;JUN;UBB;UBC;FOS;RHOA
R-HSA-2559582	Senescence-Associated Secretory Phenotype (SASP)	77	6	5.51	0.0012	0.028	Reactome	1.553	CEBPB;JUN;UBB;UBC;FOS;HIST1H2AC
GO:0032270	positive regulation of cellular protein metabolic process	91	7	5.45	0.0005	0.016	GO	1.7959	FXR1;HSP90AA1;ATG10;RPS27L;VIM;ELAVL1;AGT
WP1772	Apoptosis Modulation and Signaling	91	7	5.45	0.0005	0.0093	Wikipathway	2.0315	JUN;BAG3;BNIP3;FOS;PRKD1;MCL1;HSPA1A
R-HSA-114608	Platelet degranulation	105	8	5.41	0.00022	0.011	Reactome	1.959	CD63;HSPA5;PSAP;FLNA;CALM1;CLU;VEGFA;SOD1
WP2032	Human Thyroid Stimulating Hormone (TSH) signaling pathway	66	5	5.33	0.0034	0.031	Wikipathway	1.5086	GNA13;JUN;GNAS;FOS;GNAI2
GO:0043618	regulation of transcription from RNA polymerase II promoter in response to stress	93	7	5.32	0.00058	0.017	GO	1.7696	EGLN1;DNAJB1;UBB;BAG3;UBC;VEGFA;HSPA1A
GO:0032870	cellular response to hormone stimulus	80	6	5.28	0.0014	0.034	GO	1.4685	JUN;JUND;JUNB;RAMP1;UBE2L3;ZFP36L1
R-HSA-373755	Semaphorin interactions	67	5	5.24	0.0037	0.043	Reactome	1.367	HSP90AA1;HSP90AB1;RAC1;RHOA;RHOB
GO:0060548	negative regulation of cell death	81	6	5.21	0.0015	0.035	GO	1.4559	BNIP3L;BNIP3;PRKD1;CLU;MT3;HSPA1A
R-HSA-168142	Toll Like Receptor 10 (TLR10) Cascade	82	6	5.15	0.0016	0.028	Reactome	1.553	JUN;UBB;UBC;HMGB1;FOS;S100B
R-HSA-168176	Toll Like Receptor 5 (TLR5) Cascade	82	6	5.15	0.0016	0.028	Reactome	1.553	JUN;UBB;UBC;HMGB1;FOS;S100B
R-HSA-975871	MyD88 cascade initiated on plasma membrane	82	6	5.15	0.0016	0.028	Reactome	1.553	JUN;UBB;UBC;HMGB1;FOS;S100B
R-HSA-76005	Response to elevated platelet cytosolic Ca2+	110	8	5.14	0.0003	0.013	Reactome	1.886	CD63;HSPA5;PSAP;FLNA;CALM1;CLU;VEGFA;SOD1
R-HSA-975138	TRAF6 mediated induction of NFkB and MAP kinases upon TLR7/8 or 9 activation	83	6	5.08	0.0017	0.028	Reactome	1.553	JUN;UBB;UBC;HMGB1;FOS;S100B
R-HSA-193704	p75 NTR receptor-mediated signalling	83	6	5.08	0.0017	0.028	Reactome	1.553	YWHAE;UBB;UBC;RAC1;SOS1;RHOA
R-HSA-380259	Loss of Nlp from mitotic centrosomes	69	5	5.08	0.0042	0.044	Reactome	1.357	YWHAE;HSP90AA1;TUBA1A;ALMS1;DYNLL1
R-HSA-380284	Loss of proteins required for interphase microtubule organization?from the centrosome	69	5	5.08	0.0042	0.044	Reactome	1.357	YWHAE;HSP90AA1;TUBA1A;ALMS1;DYNLL1
GO:0045664	regulation of neuron differentiation	84	6	5.01	0.0019	0.04	GO	1.3979	GPRC5B;ID2;ID4;ID3;CALR;RHOA
R-HSA-5628897	TP53 Regulates Metabolic Genes	84	6	5.01	0.0019	0.03	Reactome	1.523	YWHAE;GPI;NDUFA4;COX4I1;PRDX1;DDIT4
GO:0043488	regulation of mRNA stability	128	9	4.97	0.00016	0.0063	GO	2.2007	FXR1;HSPA8;UBB;UBC;HNRNPU;VIM;ELAVL1;ZFP36L1;HSPA1A
R-HSA-975155	MyD88 dependent cascade initiated on endosome	85	6	4.95	0.002	0.03	Reactome	1.523	JUN;UBB;UBC;HMGB1;FOS;S100B
R-HSA-168181	Toll Like Receptor 7/8 (TLR7/8) Cascade	85	6	4.95	0.002	0.03	Reactome	1.523	JUN;UBB;UBC;HMGB1;FOS;S100B
GO:0043623	cellular protein complex assembly	144	10	4.91	0.000079	0.0036	GO	2.4437	SMAD1;HSP90AA1;HSP90AB1;HSPA4;PTGES3;TBCD;PSMG2;CLU;HSPD1;HSPA1A
GO:0034599	cellular response to oxidative stress	115	8	4.9	0.0004	0.013	GO	1.8861	JUN;PRDX1;PRKD1;FOS;PRDX6;MT3;SOD1;HSPA1A
R-HSA-450531	Regulation of mRNA stability by proteins that bind AU-rich elements	86	6	4.89	0.0021	0.03	Reactome	1.523	HSPA8:UBB:UBC:ELAVL1:ZFP36L1:HSPA1A
GO:0031396	regulation of protein ubiquitination	101	7	4.87	0.00094	0.024	GO	1.6198	DNAJA1;HSP90AA1;HSP90AB1;UBB;CHORDC1;UBE2L3;HSPA1A
R-HSA-8854518	AURKA Activation by TPX2	72	5	4.85	0.005	0.049	Reactome	1.31	YWHAE;HSP90AA1;TUBA1A;ALMS1;DYNLL1
WP2884	NRF2 pathway	146	10	4.84	0.000088	0.0026	Wikipathway	2.585	DNAJB1;HSP90AA1;SLC6A8;HSP90AB1;MGST3;PRDX1;ABCC5;PRDX6;FTL;HSPA1A
WP2840	Hair Follicle Development: Cytodifferentiation (Part 3 of 3)	87	6	4.83	0.0022	0.026	Wikipathway	1.585	SMAD1;JUN;GJB6;NFATC1;SOX9;FOS
R-HSA-168138	Toll Like Receptor 9 (TLR9) Cascade	88	6	4.77	0.0024	0.033	Reactome	1.481	JUN;UBB;UBC;HMGB1;FOS;S100B
GO:0045926	negative regulation of growth	120	8	4.68	0.00053	0.016	GO	1.7959	MT2A;GJA1;MT1M;MT1F;MT1G;ENO1;MT3;HSPA1A
R-HSA-2559580	Oxidative Stress Induced Senescence	91	6	4.6	0.0028	0.037	Reactome	1.432	JUN;UBB;UBC;E2F3;FOS;HIST1H2AC
WP2037	Prolactin Signaling Pathway	76	5	4.58	0.0063	0.044	Wikipathway	1.3565	JUN;FLNA;RAC1;FOS;SOS1
R-HSA-166058	MyD88:Mal cascade initiated on plasma membrane	92	6	4.54	0.0029	0.037	Reactome	1.432	JUN;UBB;UBC;HMGB1;FOS;S100B
R-HSA-168179	Toll Like Receptor TLR1:TLR2 Cascade	92	6	4.54	0.0029	0.037	Reactome	1.432	JUN;UBB;UBC;HMGB1;FOS;S100B
R-HSA-168188	Toll Like Receptor TLR6:TLR2 Cascade	92	6	4.54	0.0029	0.037	Reactome	1.432	JUN;UBB;UBC;HMGB1;FOS;S100B
R-HSA-181438	Toll Like Receptor 2 (TLR2) Cascade	92	6	4.54	0.0029	0.037	Reactome	1.432	JUN;UBB;UBC;HMGB1;FOS;S100B
WP2882	Nuclear Receptors Meta-Pathway	319	20	4.52	0.00000012	0.000034	Wikipathway	4.4685	JUN:HSP90AA1:JUND:HSP90AB1:MGST3:PTGES3:ABCC5:DBI:PRDX6:DNAJB1:SLC6A8:DNAJC7:SCD:PRDX1:ACKR3:LRRC8A:JUNB:PLTP:HSPA1A:FTL
WP289	Myometrial Relaxation and Contraction Pathways	156	10	4.51	0.00015	0.0038	Wikipathway	2.4202	YWHAE;GJA1;JUN;GNAS;ACKR3;PRKD1;FOS;CALM1;RAMP1;ACTB
WP35	G Protein Signaling Pathways	93	6	4.49	0.0031	0.03	Wikipathway	1.5229	GNA13:GNAS:PRKD1:CALM1:RHOA:GNAI2
GO:0016055	Wnt signaling pathway	109	7	4.48	0.0015	0.034	GO	1.4685	SNX3;SMO;UBB;UBE2B;UBC;LRP4;PLPP3
GO:0031401	positive regulation of protein modification process	163	10	4.3	0.00022	0.0079	GO	2.1024	GOLGA2;CHORDC1;ATG10;SOX9;WDFY2;RAC1;CALM1;MT3;RAMP1;VEGFA
R-HSA-166166	MyD88-independent TLR3/TLR4 cascade	97	6	4.29	0.0038	0.043	Reactome	1.367	JUN;UBB;UBC;HMGB1;FOS;S100B
R-HSA-168164	Toll Like Receptor 3 (TLR3) Cascade	97	6	4.29	0.0038	0.043	Reactome	1.367	JUN;UBB;UBC;HMGB1;FOS;S100B
R-HSA-937061	TRIF-mediated TLR3/TLR4 signaling	97	6	4.29	0.0038	0.043	Reactome	1.367	JUN;UBB;UBC;HMGB1;FOS;S100B
WP4540	Pathways Regulating Hippo Signaling	98	6	4.25	0.004	0.034	Wikipathway	1.4685	LATS2;CDH11;GNAS;RAC1;RHOA;GNAI2
WP366	TGF-beta Signaling Pathway	132	8	4.22	0.001	0.014	Wikipathway	1.8539	JUN;UBE2I;JUND;RAC1;FOS;SOS1;JUNB;RHOA
R-HSA-5610787	Hedgehog 'off' state	99	6	4.2	0.0042	0.044	Reactome	1.357	SMO;UBB;IFT140;IFT172;UBC;GNAS
GO:0006338	chromatin remodeling	116	7	4.19	0.0021	0.042	GO	1.3768	CECR2;MORF4L2;ZBTB1;SOX9;HMGB1;NUDT5;ACTB
GO:0043068	positive regulation of programmed cell death	257	15	4.13	0.000011	0.00092	GO	3.0362	BNIP3L;JUN;BNIP3;HMGB1;RHOB;HSPD1;SOD1;GNA13;DNAJA1;LATS2;UBB;FAM162A;UBC;RAC1;SOS1
R-HSA-5617833	Assembly of the primary cilium	187	11	4.12	0.00015	0.0095	Reactome	2.022	YWHAE;CCT3;HSP90AA1;TUBA1A;SMO;IFT140;IFT172;ALMS1;DYNLRB1;DYNLL1;CCT4
WP2431	Spinal Cord Injury	118	7	4.12	0.0023	0.026	Wikipathway	1.585	GJA1;SOX9;RAC1;FOS;VIM;RHOA;RHOB
R-HSA-391251	Protein folding	101	6	4.11	0.0047	0.047	Reactome	1.328	CCT3;TUBA1A;TBCD;ACTB;GNAI2;CCT4
R-HSA-168255	Influenza Life Cycle	136	8	4.09	0.0012	0.028	Reactome	1.553	HSP90AA1;POM121C;RPL31;RPS29;CANX;RPL37;CALR;HSPA1A
WP111	Electron Transport Chain (OXPHOS system in mitochondria)	103	6	4.03	0.0051	0.04	Wikipathway	1.3979	ATP5PF;NDUFA4;COX4I1;UQCRH;ATP5F1E;ATP5ME
GO:0030182	neuron differentiation	139	8	4	0.0014	0.033	GO	1.4815	FXR1;TENM2;ID2;DDIT4;ID4;ID3;PAX6;VEGFA
GO:0045597	positive regulation of cell differentiation	194	11	3.96	0.00021	0.0079	GO	2.1024	CRB2;JUN;CEBPB;JUND;HSP90AB1;SOX9;PRKD1;WDFY2;JUNB;AGT;ZFP36L1
GO:0031328	positive regulation of cellular biosynthetic process	159	9	3.93	0.0008	0.022	GO	1.6576	FXR1;SCARB1;HSP90AA1;HSP90AB1;RPS27L;CALR;VIM;CLU;ELAVL1
R-HSA-76002	Platelet activation, signaling and aggregation	253	14	3.89	0.000039	0.0032	Reactome	2.495	CD63;HSPA5;CLU;RHOA;RHOB;GNAI2;VEGFA;SOD1;GNA13;PSAP;FLNA;RAC1;CALM1;SOS1
GO:0043123	positive regulation of I-kappaB kinase/NF-kappaB signaling	163	9	3.83	0.00096	0.024	GO	1.6198	GJA1;GPRC5B;UBB;SLC44A2;UBC;FLNA;PRKD1;S100B;RHOA
R-HSA-168254	Influenza Infection	147	8	3.76	0.002	0.03	Reactome	1.523	HSP90AA1;POM121C;RPL31;RPS29;CANX;RPL37;CALR;HSPA1A
WP4217	Ebola Virus Pathway on Host	129	7	3.74	0.0038	0.033	Wikipathway	1.4815	TYRO3;FLNA;HLA-A;RAC1;ACTB;RHOA;RHOB
WP536	Calcium Regulation in the Cardiac Cell	149	8	3.71	0.0022	0.026	Wikipathway	1.585	YWHAE;GJA1;GJB6;GNAS;PRKD1;CALR;CALM1;GNAI2
GO:0043065	positive regulation of apoptotic process	307	16	3.67	0.000023	0.0015	GO	2.8239	BNIP3L;PRNP;JUN;BNIP3;HMGB1;RHOB;HSPD1;SOD1;GNA13;DNAJA1;LATS2;UBB;FAM162A;UBC;RAC1;SOS1
R-HSA-453274	Mitotic G2-G2/M phases	175	9	3.55	0.0016	0.028	Reactome	1.553	YWHAE;HSP90AA1;HSP90AB1;TUBA1A;UBB;UBC;ALMS1;E2F3;DYNLL1
GO:0010506	regulation of autophagy	203	10	3.4	0.0012	0.03	GO	1.5229	GOLGA2;EEF1A1;ATP6V0E1;BNIP3;PSAP;PRKD1;HMGB1;MT3;GAPDH;MCL1
GO:1901990	regulation of mitotic cell cycle phase transition	184	9	3.37	0.0022	0.044	GO	1.3565	YWHAE;HSP90AA1;TUBA1A;UBB;ID2;UBC;ALMS1;DYNLL1;ZFP36L1
WP3888	VEGFA-VEGFR2 Signaling Pathway	236	11	3.22	0.0011	0.015	Wikipathway	1.8239	YWHAE;GJA1;JUN;HSP90AA1;ACKR3;NFATC1;PRKD1;RAC1;RHOA;VEGFA;HSPA1A
GO:0043066	negative regulation of apoptotic process	485	22	3.21	0.0000066	0.00065	GO	3.1871	HSPA9;YWHAE;BNIP3L;HSPA5;BNIP3;MT3;VEGFA;HSPD1;SOD1;DNAJA1;UBB;DNAJB6;BAG3;UBC;TYRO3;FLNA;SOX9;CRYAB;CRLF1;TPT1;HSPA1A;MCL1
GO:0051726	regulation of cell cycle	215	10	3.2	0.0019	0.04	GO	1.3979	JUN;JUND;ID2;ID4;HNRNPU;ID3;SOX9;CALR;JUNB;RHOB
GO:0043069	negative regulation of programmed cell death	408	18	3.08	0.000065	0.0032	GO	2.4949	HSPA9;BNIP3L;HSPA5;BNIP3;MT3;VEGFA;HSPD1;DNAJA1;UBB;BAG3;UBC;TYRO3;FLNA;SOX9;CRYAB;TPT1;HSPA1A;MCL1
GO:0043312	neutrophil degranulation	479	20	2.92	0.000056	0.003	GO	2.5229	GPI;CD63;HSPA8;HSP90AA1;HSP90AB1;SLC44A2;HMGB1;DYNLL1;PRDX6;RHOA;EEF1A1;CYB5R3;PKM;LAMP1;PSAP;MLEC;RAC1;METTL7A;HSPA1A;FTL
GO:0002283	neutrophil activation involved in immune response	483	20	2.89	0.000063	0.0032	GO	2.4949	GPI;CD63;HSPA8;HSP90AA1;HSP90AB1;SLC44A2;HMGB1;DYNLL1;PRDX6;RHOA;EEF1A1;CYB5R3;PKM;LAMP1;PSAP;MLEC;RAC1;METTL7A;HSPA1A;FTL
GO:0002446	neutrophil mediated immunity	487	20	2.87	0.00007	0.0033	GO	2.4815	GPI;CD63;HSPA8;HSP90AA1;HSP90AB1;SLC44A2;HMGB1;DYNLL1;PRDX6;RHOA;EEF1A1;CYB5R3;PKM;LAMP1;PSAP;MLEC;RAC1;METTL7A;HSPA1A;FTL
GO:0000122	negative regulation of transcription from RNA polymerase II promoter	565	23	2.86	0.000023	0.0015	GO	2.8239	WWOX;JUN;TENM2;CEBPB;JUND;UBE2I;CNBP;ZBTB1;HNRNPU;ENO1;HMGB1;VEGFA;DNAJB1;UBB;YEATS2;BAG3;SAP130;UBC;SOX9;CALR;MXD1;JUNB;HSPA1A
WP4352	Ciliary landscape	216	9	2.84	0.0063	0.044	Wikipathway	1.3565	GDI1;IFT140;WHRN;IFT172;RAC1;DYNLRB1;VIM;CALM1;DYNLL1
R-HSA-5663205	Infectious disease	348	14	2.77	0.001	0.026	Reactome	1.585	HSP90AA1;HSP90AB1;RPL31;HLA-A;POM121C;UBB;RPS29;CANX;UBC;RPL37;CALR;RAC1;CALM1;HSPA1A
GO:0071345	cellular response to cytokine stimulus	456	18	2.74	0.00026	0.0092	GO	2.0362	HSPA8;HSP90AA1;CEBPD;HNRNPU;FOS;RHOA;VEGFA;ZFP36L1;MT2A;UBB;UBC;SOX9;VIM;SOS1;JUNB;GAPDH;CRLF1;MCL1
R-HSA-71387	Metabolism of carbohydrates	282	11	2.66	0.0044	0.045	Reactome	1.347	GPI;GYS1;PKM;POM121C;UBB;ABCC5;UBC;PGK1;ENO1;CALM1;GAPDH
GO:0042981	regulation of apoptotic process	815	29	2.51	0.000025	0.0015	GO	2.8239	HMGB1;CLU;HSPD1;GNA13;UBB;FAM162A;BAG3;UBC;FLNA;SOX9;RAC1;MCL1;HSPA9;BNIP3L;JUN;HSPA5;BNIP3;MT3;RHOB;VEGFA;SOD1;DNAJA1;LATS2;TYRO3;CALR;SOS1;CRYAB;TPT1;HSPA1A
GO:0045892	negative regulation of transcription, DNA-templated	813	27	2.32	0.00015	0.0061	GO	2.2147	TENM2;CEBPB;ZBTB1;HNRNPU;ENO1;HMGB1;UBB;SAP130;UBC;FLNA;SOX9;JUNB;WWOX;HSPA8;JUN;JUND;UBE2I;SFMBT1;CNBP;MT3;VEGFA;YEATS2;ID2;ID4;ID3;CALR;MXD1
GO:0019221	cytokine-mediated signaling pathway	633	20	2.17	0.0019	0.041	GO	1.3872	HSPA9:HSPA8:HSP90AA1:CEBPD:HLA-A:FOS:MT3:IL17RB:VEGFA:SOD1:MT2A:UBB:CANX:UBC:ACKR3:VIM:SOS1:JUNB:CRLF1:MCL1
GO:0010628	positive regulation of gene expression	771	23	2.05	0.0019	0.041	GO	1.3872	SMAD1;JUN;CNBP;RPS27L;NFATC1;PAX6;FOS;MED4;CLU;MT3;ELAVL1;ACTB;AGT;VEGFA;FXR1;SMO;ID2;ITGB8;SOX9;PCBD2;VIM;ZNF398;HSPA1A

Supplementary Table 4_Astrocyte MEGENA modules
Module 30 FE

geneset	description	size	overlap	odds_ratio	pval	FDR	database	X.Log10.FDR.	genes
GO:0035627	ceramide transport	6	2	212.2	0.000088	0.015	GO	1.824	PSAP;PLTP
GO:1902430	negative regulation of amyloid-beta formation	9	2	121.24	0.00021	0.02	GO	1.699	PRNP;CLU
GO:0097104	postsynaptic membrane assembly	9	2	121.24	0.00021	0.02	GO	1.699	NLGN2;LRP4
GO:0097105	presynaptic membrane assembly	10	2	106.08	0.00026	0.02	GO	1.699	NLGN2;LRP4
GO:1902992	negative regulation of amyloid precursor protein catabolic process	11	2	94.29	0.00032	0.02	GO	1.699	PRNP;CLU
GO:0099068	postsynapse assembly	11	2	94.29	0.00032	0.02	GO	1.699	NLGN2;LRP4
GO:0097090	presynaptic membrane organization	11	2	94.29	0.00032	0.02	GO	1.699	NLGN2;LRP4
R-HSA-532668	N-glycan trimming in the ER and Calnexin/Calreticulin cycle	17	3	92.87	0.0000092	0.0017	Reactome	2.77	CANX;MLEC;CALR
GO:0090190	positive regulation of branching involved in ureteric bud morphogenesis	12	2	84.86	0.00038	0.022	GO	1.658	SOX9;AGT
GO:0010470	regulation of gastrulation	14	2	70.71	0.00053	0.024	GO	1.62	CRB2;DAG1
GO:0090189	regulation of branching involved in ureteric bud morphogenesis	15	2	65.26	0.0006	0.024	GO	1.62	SOX9;AGT
GO:0099054	presynapse assembly	15	2	65.26	0.0006	0.024	GO	1.62	NLGN2;LRP4
GO:0061213	positive regulation of mesonephros development	15	2	65.26	0.0006	0.024	GO	1.62	SOX9;AGT
R-HSA-901042	Calnexin/calreticulin cycle	15	2	65.26	0.0006	0.0363	Reactome	1.44	CANX;CALR
GO:0001941	postsynaptic membrane organization	16	2	60.6	0.00069	0.025	GO	1.602	NLGN2;LRP4
GO:1902229	regulation of intrinsic apoptotic signaling pathway in response to DNA damage	16	2	60.6	0.00069	0.025	GO	1.602	ACKR3;CLU
R-HSA-983170	Antigen Presentation: Folding, assembly and peptide loading of class I MHC	25	3	59.08	0.000031	0.0028	Reactome	2.55	CANX;HLA-A;CALR
GO:1902003	regulation of amyloid-beta formation	18	2	53.02	0.00088	0.025	GO	1.602	PRNP;CLU
GO:1905332	positive regulation of morphogenesis of an epithelium	18	2	53.02	0.00088	0.025	GO	1.602	SOX9;AGT
GO:0061098	positive regulation of protein tyrosine kinase activity	28	3	51.98	0.000043	0.013	GO	1.886	PRNP;GPRC5B;AGT
GO:0002474	antigen processing and presentation of peptide antigen via MHC class I	28	3	51.98	0.000043	0.013	GO	1.886	CANX;HLA-A;CALR
GO:0051353	positive regulation of oxidoreductase activity	19	2	49.9	0.00098	0.025	GO	1.602	POR;AGT
R-HSA-190861	Gap junction assembly	19	2	49.9	0.00098	0.0441	Reactome	1.36	GJA1;GJB6
GO:0061097	regulation of protein tyrosine kinase activity	31	3	46.4	0.000059	0.013	GO	1.886	PRNP;GPRC5B;AGT
GO:0071709	membrane assembly	23	2	40.39	0.0014	0.029	GO	1.538	NLGN2;LRP4
GO:1902230	negative regulation of intrinsic apoptotic signaling pathway in response to DNA damage	24	2	38.55	0.0016	0.03	GO	1.523	ACKR3;CLU
GO:2001021	negative regulation of response to DNA damage stimulus	28	2	32.61	0.0021	0.039	GO	1.409	ACKR3;CLU
GO:0048488	synaptic vesicle endocytosis	29	2	31.4	0.0023	0.039	GO	1.409	NLGN2;CANX
GO:0045995	regulation of embryonic development	29	2	31.4	0.0023	0.039	GO	1.409	CRB2;DAG1
GO:0036465	synaptic vesicle recycling	29	2	31.4	0.0023	0.039	GO	1.409	NLGN2;CANX
GO:0070527	platelet aggregation	33	2	27.34	0.003	0.048	GO	1.319	CSRP1;TYRO3
GO:0050768	negative regulation of neurogenesis	33	2	27.34	0.003	0.048	GO	1.319	ID4;CALR
GO:0045665	negative regulation of neuron differentiation	64	3	21.27	0.00052	0.024	GO	1.62	ID4;SOX9;CALR
GO:0010634	positive regulation of epithelial cell migration	74	3	18.26	0.00079	0.025	GO	1.602	SOX9;CALR;AGT
GO:0034249	negative regulation of cellular amide metabolic process	75	3	18.01	0.00082	0.025	GO	1.602	PRNP;CALR;CLU
GO:0045664	regulation of neuron differentiation	84	3	16	0.0011	0.025	GO	1.602	GPRC5B;ID4;CALR
GO:0050730	regulation of peptidyl-tyrosine phosphorylation	85	3	15.8	0.0012	0.025	GO	1.602	PRNP;CD81;AGT
GO:0050731	positive regulation of peptidyl-tyrosine phosphorylation	116	4	15.75	0.00019	0.02	GO	1.699	PRNP;GPRC5B;CD81;AGT
GO:0071702	organic substance transport	87	3	15.42	0.0013	0.026	GO	1.585	SLC22A17;PSAP;PLTP
GO:0046916	cellular transition metal ion homeostasis	93	3	14.39	0.0015	0.03	GO	1.523	PRNP;SLC22A17;CYBRD1
WP536	Calcium Regulation in the Cardiac Cell	149	4	12.14	0.00048	0.028	Wikipathway	1.55	GJA1;GJB6;CALR;GNAI2
GO:0045860	positive regulation of protein kinase activity	172	4	10.47	0.00083	0.025	GO	1.602	PRNP;GPRC5B;CLU;AGT
GO:0006898	receptor-mediated endocytosis	188	4	9.55	0.0012	0.025	GO	1.602	CD81;CANX;ACKR3;CALR
GO:1902533	positive regulation of intracellular signal transduction	479	6	5.75	0.0011	0.025	GO	1.602	GJA1;GPRC5B;SLC44A2;TYRO3;SOX9;AGT
GO:0043312	neutrophil degranulation	479	6	5.75	0.0011	0.025	GO	1.602	CYB5R3;LAMP1;SLC44A2;PSAP;MLEC;METTL7A
GO:0002283	neutrophil activation involved in immune response	483	6	5.7	0.0011	0.025	GO	1.602	CYB5R3;LAMP1;SLC44A2;PSAP;MLEC;METTL7A
GO:0002446	neutrophil mediated immunity	487	6	5.65	0.0012	0.025	GO	1.602	CYB5R3;LAMP1;SLC44A2;PSAP;MLEC;METTL7A

Supplementary Table 4_Astrocyte MEGENA modules
Module 13 FE

geneset	description	size	overlap	odds_ratio	pval	FDR	database	X.Log10.FDR.	genes
R-HSA-3595172	Defective CHST3 causes SEDCJD	7	2	80.38	0.00052	0.042	Reactome	1.377	BCAN;CSPG5
R-HSA-3595177	Defective CHSY1 causes TPBS	7	2	80.38	0.00052	0.042	Reactome	1.377	BCAN;CSPG5
R-HSA-3595174	Defective CHST14 causes EDS, musculocontractural type	7	2	80.38	0.00052	0.042	Reactome	1.377	BCAN;CSPG5
R-HSA-2022923	Dermatan sulfate biosynthesis	11	2	44.65	0.00135	0.078	Reactome	1.108	BCAN;CSPG5
WP4313	Ferroptosis	40	3	16.43	0.0011	0.07	Wikipathway	1.155	SLC3A2;SAT2;ACSL4
WP1591	Heart Development	44	3	14.83	0.0014	0.07	Wikipathway	1.155	NOTCH1;VEGFB;NFATC4
WP268	Notch Signaling	45	3	14.47	0.0015	0.07	Wikipathway	1.155	NOTCH1;DTX3;RFNG
R-HSA-1474244	Extracellular matrix organization	283	7	5.29	0.00058	0.042	Reactome	1.377	DDR1;BCAN;LAMA5;COL16A1;PTPRS;COL6A1;LTBP3

Supplementary Table 4_Astrocyte MEGENA modules
Pathology enrichment

Positive logFC values indicate a higher enrichment with pathology

Amyloid-pathology

Co-expression module	logFC	P.Value	adj.P.Val
c1_3	-0.06859778	0.770659255	0.875925579
c1_4	-0.293860665	0.420888066	0.587028092
c1_5	-0.305587773	0.273096821	0.54196649
c1_6	-0.079824225	0.694585651	0.818067544
c1_8	0.454540874	0.077108067	0.340560628
c1_9	1.559016384	0.000247637	0.003281187
c1_10	-0.396756475	0.07060206	0.34017356
c1_11	-0.400185388	0.313135127	0.54196649
c1_12	-0.641982034	0.022802997	0.151069854
c1_13	1.182116093	0.145035488	0.452169464
c1_16	-0.207024806	0.390488261	0.574885495
c1_17	-0.467720407	0.517766138	0.669307447
c1_18	-0.127940017	0.776764193	0.875925579
c1_19	-0.290918128	0.367318118	0.556224579
c1_21	-0.271745458	0.553188396	0.698071071
c1_22	0.046291632	0.945835147	0.945835147
c1_23	-1.234766179	0.192284314	0.536372033
c1_24	0.328108419	0.49800718	0.659859514
c1_25	-0.13622104	0.569094838	0.701442474
c1_26	0.544847304	0.045101098	0.239035817
c1_27	0.028805576	0.943497552	0.945835147
c1_28	1.866372275	0.037553386	0.22114772
c1_29	1.836336771	0.000131389	0.002573233
c1_30	0.914491167	0.241374039	0.54196649
c1_32	-1.094852605	0.005607146	0.04952979
c1_33	0.025093669	0.923994014	0.945835147
c1_34	-0.595811145	0.104036173	0.399445376
c1_36	-0.430635271	0.296555333	0.54196649
c1_37	-0.681094856	0.016719801	0.126592779
c1_41	1.424539976	0.337524278	0.542084447
c1_46	-0.27568285	0.259877663	0.54196649
c1_47	-0.333128973	0.320578586	0.54196649
c1_51	0.385137827	0.450845351	0.612687272
c1_53	-0.243333831	0.363890385	0.556224579
c1_54	0.309517354	0.32722505	0.54196649
c1_61	0.435492337	0.127393979	0.421992554
c1_62	1.65850416	0.110690437	0.399445376
c1_65	1.453411772	0.182186565	0.536372033
c1_66	2.669025097	0.000145655	0.002573233
c1_78	1.092524906	0.248128164	0.54196649
c1_80	-1.195901964	0.002970513	0.031487435
c1_83	-0.021896459	0.936105395	0.945835147
c1_86	-0.424631693	0.306445098	0.54196649
c1_89	1.077959421	0.589070715	0.709562452
c1_92	-0.271847814	0.271903138	0.54196649
c1_95	0.110383254	0.914865889	0.945835147
c1_96	0.464238962	0.402648742	0.576767117
c1_99	-0.283404927	0.296587568	0.54196649
c1_106	0.459532508	0.113050578	0.399445376
c1_108	2.776922194	8.47E-05	0.002573233
c1_112	0.036996988	0.897509781	0.945835147
c1_117	-0.27405863	0.269141455	0.54196649
c1_119	-0.312564613	0.265138855	0.54196649

pTau pathology

Co-expression module	logFC	P.Value	adj.P.Val
c1_3	0.209969455	0.29858848	0.494537171
c1_4	-0.10412534	0.739709466	0.891013674
c1_5	-0.374587327	0.117581474	0.250112001
c1_6	-0.233831533	0.180281995	0.353886879
c1_8	-0.006399877	0.976869411	0.976869411
c1_9	1.767123627	1.30E-06	1.73E-05
c1_10	-0.450575706	0.016758831	0.080747097
c1_11	-0.675226545	0.047410314	0.167516441
c1_12	-0.654586064	0.006844468	0.051822399
c1_13	1.726224321	0.013171821	0.070538104
c1_16	0.029044349	0.888386781	0.905471142
c1_17	0.279000683	0.653106788	0.821398545
c1_18	-0.260231335	0.501681526	0.699404505
c1_19	-0.477066394	0.085031213	0.204847921
c1_21	-0.488692757	0.214124495	0.391330974
c1_22	0.381131129	0.514656145	0.699404505
c1_23	-0.672258624	0.408266218	0.65570029
c1_24	0.243893344	0.55734765	0.738485636
c1_25	-0.305731317	0.136565391	0.278383298
c1_26	0.173749962	0.456660171	0.691513973
c1_27	-0.621283766	0.07494616	0.204847921
c1_28	1.568771222	0.041710681	0.158372208
c1_29	2.054629163	6.22E-07	1.10E-05
c1_30	1.284860386	0.055166692	0.182739667
c1_32	-0.893856771	0.008429792	0.055847371
c1_33	-0.097328938	0.666417688	0.821398545
c1_34	-0.778808534	0.013309076	0.070538104
c1_36	-0.720942079	0.041771851	0.158372208
c1_37	-0.682142356	0.005242011	0.046304427
c1_41	1.494312166	0.241202516	0.426124445
c1_46	-0.049693107	0.812978419	0.892042551
c1_47	-0.543206289	0.05918544	0.184519313
c1_51	0.307168593	0.483582004	0.699404505
c1_53	-0.375104864	0.102987235	0.237318411
c1_54	-0.050401422	0.85256374	0.892042551
c1_61	0.04375695	0.858380568	0.892042551
c1_62	1.175904721	0.187677162	0.355246057
c1_65	1.462043015	0.117977359	0.250112001
c1_66	3.107801305	2.57E-07	6.81E-06
c1_78	1.414253003	0.081576634	0.204847921
c1_80	-0.977743629	0.004663508	0.046304427
c1_83	-0.153025297	0.513980607	0.699404505
c1_86	-0.725363854	0.041834168	0.158372208
c1_89	1.34206982	0.433354868	0.675523765
c1_92	-0.041206877	0.846169426	0.892042551
c1_95	-0.494808375	0.576672413	0.745454582
c1_96	0.509790776	0.28434592	0.486139798
c1_99	-0.410030284	0.078537451	0.204847921
c1_106	0.053330337	0.830365737	0.892042551
c1_108	3.217133701	1.12E-07	5.96E-06
c1_112	-0.064023202	0.795121006	0.892042551
c1_117	-0.040425998	0.849397095	0.892042551
c1_119	-0.437095093	0.069472986	0.204559349

Supplementary Table 4_Astrocyte MEGENA modules
Enrichment other datasets

Positive logFC values indicate a higher enrichment in AD compared to Control

Grubman et al 2019, doi: 10.1038/s41593-019-0539-4

Co-expression module	logFC	P.Value	adj.P.Val
c1_3	0.049461881	4.00E-07	5.17E-07
c1_4	0.238267601	8.24E-18	1.75E-17
c1_5	-0.142380073	3.40E-22	1.00E-21
c1_6	-0.258733709	2.44E-155	3.23E-154
c1_8	-0.027240801	0.032764228	0.035438858
c1_9	0.253162063	5.01942E-28	1.77353E-27
c1_10	0.100150497	1.63E-17	3.33E-17
c1_11	0.321683462	2.56E-45	2.26E-44
c1_12	-0.08371632	1.60E-07	2.12E-07
c1_13	-0.086340282	0.012933065	0.014280259
c1_16	0.067950687	8.13E-11	1.31E-10
c1_17	0.572760706	3.98E-29	1.51E-28
c1_18	-0.031314602	0.450093306	0.450093306
c1_19	-0.221752679	1.03E-33	4.97E-33
c1_21	-0.099868959	0.001153473	0.001358535
c1_22	-0.124475013	0.001123915	0.001353806
c1_23	0.35381773	1.37E-07	1.86E-07
c1_24	0.040052847	0.181832557	0.19274251
c1_25	-0.292387868	3.10E-154	3.29E-153
c1_26	-0.054823547	0.000196345	0.000242007
c1_27	-0.185056692	2.37E-10	3.69E-10
c1_28	0.692081873	2.04E-32	8.31E-32
c1_29	0.270728909	8.89E-25	2.95E-24
c1_30	0.263092626	3.27318E-09	4.68861E-09
c1_32	0.252232245	5.49E-24	1.71E-23
c1_33	0.125287878	3.11E-14	5.50E-14
c1_34	-0.08239362	0.001265692	0.001458297
c1_36	0.311008328	3.72E-41	2.46E-40
c1_37	-0.089681739	1.89E-08	2.63E-08
c1_41	-0.515878938	4.39E-10	6.47E-10
c1_46	0.099216802	6.77E-20	1.79E-19
c1_47	-0.254638704	4.00E-40	2.36E-39
c1_51	0.03456916	0.266361621	0.276807175
c1_53	-0.348137387	1.03E-183	1.81E-182
c1_54	-0.14748805	6.43E-15	1.26E-14
c1_61	-0.116708848	1.27E-12	2.11E-12
c1_62	0.063607457	0.27574471	0.281047493
c1_65	0.561295942	2.47E-18	5.70E-18
c1_66	0.444145514	4.73E-34	2.51E-33
c1_78	0.428373651	1.24E-14	2.36E-14
c1_80	0.226974949	5.33E-18	1.18E-17
c1_83	0.133241722	1.33E-14	2.43E-14
c1_86	0.316057009	2.17E-41	1.64E-40
c1_89	-0.630317558	2.97E-10	4.50E-10
c1_92	0.099516636	8.29E-20	2.09E-19
c1_95	-0.285967326	1.03E-05	1.30E-05
c1_96	0.105991671	0.003183604	0.003590021
c1_99	-0.367766906	1.25E-194	3.31E-193
c1_106	-0.122860339	1.00E-13	1.71E-13
c1_108	0.439807834	1.28E-33	5.65E-33
c1_112	0.171113616	1.12E-20	3.13E-20
c1_117	0.097768607	4.06E-19	9.78E-19
c1_119	-0.397116145	1.41E-206	7.48E-205

Mathys et al 2019, doi: 10.1038/s41586-019-1195-2

Co-expression module	logFC	P.Value	adj.P.Val
c1_3	-0.006947811	0.283634747	0.37581604
c1_4	0.073123904	7.22E-07	3.83E-06
c1_5	-0.010214001	0.210054451	0.309246831
c1_6	-0.022982624	0.000708684	0.002209427
c1_8	-0.018062078	0.022823257	0.046524332
c1_9	0.085495915	1.59822E-10	2.82351E-09
c1_10	-0.017228138	0.028122846	0.055204105
c1_11	0.024980953	0.05652905	0.099867989
c1_12	-0.011312795	0.254581128	0.345969225
c1_13	0.049784241	0.013091206	0.030166692
c1_16	-0.024173045	0.000630718	0.002089252
c1_17	0.170902212	1.48E-13	7.86E-12
c1_18	-0.000761956	0.971416724	0.980747539
c1_19	-0.008624374	0.374478076	0.461566001
c1_21	-0.041997313	0.009825871	0.023671417
c1_22	0.023261411	0.206356253	0.309246831
c1_23	-0.096075718	0.006100189	0.015395715
c1_24	-0.00586585	0.680176164	0.767007163
c1_25	-0.025683157	0.000384102	0.001454101
c1_26	0.007511351	0.391967613	0.472142807
c1_27	-0.089833808	5.47E-11	1.45E-09
c1_28	0.029876575	0.248790751	0.345969225
c1_29	0.076114846	2.26E-07	2.00E-06
c1_30	0.117870058	5.46598E-07	3.21886E-06
c1_32	-0.009897032	0.450446206	0.530525532
c1_33	-0.010088313	0.300956422	0.389041229
c1_34	-0.04711322	0.00046515	0.001643529
c1_36	0.026865694	0.047919769	0.087577509
c1_37	-0.013258481	0.186213254	0.290273601
c1_41	-0.15860211	0.000276202	0.001219892
c1_46	-0.02046982	0.004924115	0.013048905
c1_47	-0.020347305	0.043543774	0.082422144
c1_51	0.003384027	0.818962345	0.868100085
c1_53	-0.032386483	2.41E-05	0.000115899
c1_54	-0.00478179	0.672464566	0.767007163
c1_61	0.01108666	0.24590283	0.345969225
c1_62	-0.053904452	0.066558467	0.113793508
c1_65	0.007337203	0.811484075	0.868100085
c1_66	0.122515222	2.22E-09	2.94E-08
c1_78	0.10338037	0.000308896	0.001259344
c1_80	0.000331038	0.980747539	0.980747539
c1_83	0.003400177	0.740960268	0.818143629
c1_86	0.022656167	0.09786834	0.162094439
c1_89	-0.119013441	0.016380381	0.034726408
c1_92	-0.020923555	0.004283068	0.011947505
c1_95	0.0848138	0.01614217	0.034726408
c1_96	-0.016589984	0.327237504	0.412942565
c1_99	-0.039291667	4.66E-07	3.14E-06
c1_106	0.014609767	0.13047484	0.209550501
c1_108	0.106191114	4.74E-07	3.14E-06
c1_112	-0.001391519	0.895534214	0.930653203
c1_117	-0.021114616	0.004073622	0.011947505
c1_119	-0.041790322	1.60E-07	1.69E-06

Zhou et al 2020, doi: 10.1038/s41591-019-0695-9

Co-expression module	logFC	P.Value	adj.P.Val
c1_3	0.045780677	9.97E-28	1.82E-27
c1_4	0.103627858	1.93E-20	3.29E-20
c1_5	0.143620904	3.00E-94	7.56E-94
c1_6	0.119473594	2.29E-83	5.52E-83
c1_8	0.164210011	8.78E-109	2.58E-108
c1_9	0.041606692	1.35769E-07	1.99882E-07
c1_10	-0.216313508	1.91E-268	2.53E-267
c1_11	-0.405628331	0	0
c1_12	0.064063064	2.46E-19	4.08E-19
c1_13	0.292242852	2.01E-166	9.70E-166
c1_16	0.007532299	0.129067951	0.159083753
c1_17	0.181272053	4.29E-31	8.12E-31
c1_18	0.000523756	0.972802243	0.972802243
c1_19	0.11083771	6.86E-47	1.45E-46
c1_21	-0.055436335	3.44E-05	4.68E-05
c1_22	0.372170104	1.03E-187	6.06E-187
c1_23	0.005893917	0.784486182	0.822245386
c1_24	-0.057942939	3.10E-07	4.32E-07
c1_25	0.147415696	7.60E-98	2.01E-97
c1_26	0.176093603	2.29E-101	6.37E-101
c1_27	-0.003155673	0.791217258	0.822245386
c1_28	0.508773306	3.00E-130	1.22E-129
c1_29	-0.001503693	0.871898548	0.888665827
c1_30	0.203383984	3.19236E-55	7.04981E-55
c1_32	-0.283850747	1.03E-254	1.09E-253
c1_33	-0.218015901	1.26E-163	5.58E-163
c1_34	-0.075782955	1.25E-13	1.96E-13
c1_36	-0.415588048	0	0
c1_37	0.063842304	8.49E-19	1.36E-18
c1_41	0.083872189	0.006284511	0.008326977
c1_46	-0.003667788	0.477957222	0.527744433
c1_47	0.131544926	2.83E-58	6.52E-58
c1_51	-0.025298488	0.031786282	0.04011126
c1_53	0.18474917	1.58E-125	5.99E-125
c1_54	0.116805472	4.89E-23	8.63E-23
c1_61	0.107475927	1.14E-34	2.24E-34
c1_62	0.561498226	2.11E-168	1.12E-167
c1_65	0.538029007	1.94E-115	6.43E-115
c1_66	0.01274467	0.241413692	0.284354636
c1_78	0.097858526	6.97E-13	1.06E-12
c1_80	-0.282075227	1.96E-234	1.73E-233
c1_83	-0.277714002	1.27E-221	9.65E-221
c1_86	-0.418538673	0	0
c1_89	0.191940738	1.40E-07	2.01E-07
c1_92	-0.004276834	0.408735148	0.460914103
c1_95	0.038785696	0.257723555	0.296942357
c1_96	-0.031324077	0.013993182	0.018088748
c1_99	0.178435848	3.77E-115	1.17E-114
c1_106	0.109012072	4.66E-35	9.51E-35
c1_108	0.005734277	0.604738863	0.654105301
c1_112	-0.284627632	7.38E-219	4.89E-218
c1_117	-0.0060824	0.241433181	0.284354636
c1_119	0.186579028	9.38E-120	3.32E-119

Gerrits et al 2021, doi: 10.1007/s00401-021-02263-w

Co-expression module	logFC	P.Value	adj.P.Val
c1_3	0.075916706	0	0
c1_4	0.016337584	2.26E-14	2.73E-14
c1_5	-0.058606949	0	0
c1_6	-0.04730356	0	0
c1_8	-0.09745998	0	0
c1_9	0.078383121	1.41547102464818e-313	3.00079857227589e-313
c1_10	0.034590295	1.45E-215	2.55E-215
c1_11	0.131502765	0	0
c1_12	-0.011737617	2.90E-18	3.75E-18
c1_13	0.003548397	0.416187693	0.424191303
c1_16	0.078699044	0	0
c1_17	-0.012018728	0.010078055	0.011127853
c1_18	0.030931929	1.53E-32	2.13E-32
c1_19	-0.04900626	1.66E-187	2.76E-187
c1_21	-0.017264937	1.65E-12	1.90E-12
c1_22	-0.196589443	0	0
c1_23	-0.078857166	5.68E-48	8.60E-48
c1_24	0.006095102	0.023645017	0.025063718
c1_25	-0.055292338	0	0
c1_26	-0.098517151	0	0
c1_27	-0.060445824	9.57E-162	1.54E-161
c1_28	-0.346598217	0	0
c1_29	0.106274546	0	0
c1_30	0.039346668	6.6953E-14	7.88558E-14
c1_32	0.085027649	2.10E-269	4.27E-269
c1_33	-0.001170824	0.394023522	0.409475425
c1_34	0.061686408	3.69E-253	6.99E-253
c1_36	0.142860632	0	0
c1_37	-0.011574435	1.95E-17	2.45E-17
c1_41	-0.030204861	3.65E-06	4.12E-06
c1_46	0.081521428	0	0
c1_47	-0.059693744	1.82E-251	3.33E-251
c1_51	0.000659386	0.815515666	0.815515666
c1_53	-0.073998199	0	0
c1_54	-0.023484961	2.67E-36	3.82E-36
c1_61	-0.076555442	0	0
c1_62	-0.283321687	0	0
c1_65	-0.350149602	0	0
c1_66	0.163116448	0	0
c1_78	0.069036167	4.01E-25	5.45E-25
c1_80	0.075848097	4.28E-199	7.31E-199
c1_83	0.018520326	6.30E-37	9.27E-37
c1_86	0.14167171	0	0
c1_89	0.025665927	0.020423319	0.022090528
c1_92	0.083949835	0	0
c1_95	-0.102079457	5.35E-58	8.35E-58
c1_96	0.024653859	3.41E-17	4.20E-17
c1_99	-0.074716093	0	0
c1_106	-0.080577714	0	0
c1_108	0.129076471	1.72E-253	3.38E-253
c1_112	0.014702304	1.67E-21	2.21E-21
c1_117	0.084609327	0	0
c1_119	-0.078920261	0	0

Supplementary Table 4_Astrocyte MEGENA modules
Module specificity scores

Module specificity scores (MSS) in astrocytic subclusters
A high module specificity score indicates a relatively specific enrichment of this module for a particular subcluster

Co-express	Astro1	Astro2	Astro3	Astro4	Astro5	Astro6
c1_3	0.399	0.374	0.329	0.316	0.297	0.215
c1_4	0.400	0.374	0.329	0.312	0.298	0.214
c1_5	0.407	0.388	0.326	0.315	0.279	0.211
c1_6	0.407	0.383	0.328	0.311	0.286	0.211
c1_8	0.403	0.385	0.326	0.313	0.288	0.211
c1_9	0.392	0.369	0.326	0.313	0.308	0.218
c1_10	0.399	0.377	0.325	0.324	0.290	0.212
c1_11	0.393	0.369	0.323	0.331	0.296	0.211
c1_12	0.403	0.375	0.332	0.310	0.295	0.213
c1_13	0.394	0.377	0.321	0.309	0.286	0.215
c1_16	0.399	0.373	0.329	0.316	0.297	0.215
c1_17	0.389	0.363	0.325	0.311	0.303	0.214
c1_18	0.402	0.376	0.329	0.310	0.293	0.213
c1_19	0.407	0.389	0.325	0.315	0.277	0.210
c1_21	0.406	0.382	0.327	0.318	0.278	0.210
c1_22	0.401	0.386	0.323	0.308	0.280	0.212
c1_23	0.395	0.374	0.323	0.311	0.290	0.212
c1_24	0.389	0.364	0.325	0.308	0.329	0.216
c1_25	0.409	0.385	0.329	0.311	0.280	0.210
c1_26	0.402	0.381	0.327	0.313	0.293	0.212
c1_27	0.405	0.391	0.322	0.316	0.279	0.209
c1_28	0.400	0.378	0.326	0.299	0.290	0.213
c1_29	0.389	0.363	0.326	0.311	0.315	0.219
c1_30	0.399	0.380	0.324	0.307	0.284	0.216
c1_32	0.395	0.375	0.323	0.329	0.289	0.212
c1_33	0.400	0.377	0.325	0.323	0.292	0.212
c1_34	0.401	0.378	0.326	0.320	0.286	0.212
c1_36	0.393	0.368	0.323	0.332	0.295	0.211
c1_37	0.403	0.375	0.332	0.309	0.294	0.213
c1_41	0.392	0.382	0.317	0.308	0.274	0.208
c1_46	0.399	0.373	0.329	0.316	0.298	0.215
c1_47	0.408	0.390	0.325	0.315	0.276	0.210
c1_51	0.388	0.363	0.325	0.306	0.333	0.216
c1_53	0.410	0.386	0.329	0.310	0.278	0.210
c1_54	0.408	0.384	0.327	0.312	0.282	0.210
c1_61	0.402	0.382	0.327	0.314	0.291	0.212
c1_62	0.397	0.387	0.317	0.311	0.274	0.211
c1_65	0.400	0.378	0.326	0.296	0.289	0.212
c1_66	0.379	0.354	0.323	0.309	0.325	0.221
c1_78	0.392	0.373	0.322	0.310	0.294	0.218
c1_80	0.397	0.377	0.323	0.328	0.287	0.212
c1_83	0.399	0.376	0.325	0.324	0.291	0.212
c1_86	0.392	0.368	0.323	0.332	0.295	0.211
c1_89	0.384	0.379	0.312	0.300	0.268	0.208
c1_92	0.399	0.373	0.329	0.316	0.298	0.215
c1_95	0.392	0.371	0.318	0.296	0.316	0.220
c1_96	0.385	0.359	0.326	0.308	0.338	0.216
c1_99	0.410	0.386	0.329	0.310	0.278	0.210
c1_106	0.402	0.382	0.326	0.314	0.290	0.212
c1_108	0.380	0.355	0.322	0.308	0.324	0.220
c1_112	0.398	0.376	0.325	0.324	0.293	0.212
c1_117	0.399	0.373	0.329	0.316	0.298	0.215
c1_119	0.410	0.387	0.329	0.310	0.277	0.210

Supplementary Table 5_Microglia MEGENA Modules
Contents

Sheet name	Description
Module_list	MEGENA Gene Co-expression Modules in Microglia
Module_description	MEGENA Gene Co-expression Module description
Module 11 FE	Functional enrichment of Module 11 (GPNMB hub gene) genes by enrichR
Module 34 FE	Functional enrichment of Module 34 (GPNMB hub gene) genes by enrichR
Module 19 FE	Functional enrichment of Module 19 (APOE hub gene) genes by enrichR
Pathology_enrichment	logFC of the enrichment (AUCell and limma) of modules with amyloid-beta and pTau pathology
Enrichment_other_datasets	logFC of the enrichment (AUCell and limma) of modules in AD samples compared to Control samples from previous studies
MSS	Module specificity scores (MSS) in microglial subclusters

Supplementary Table 5_Microglia MEGENA Modules
Module list

MEGENA Gene Co-expression Modules in Microglia

c1_2	c1_4	c1_5	c1_6	c1_7	c1_9	c1_10	c1_11	c1_12	c1_13	c1_15	c1_16	c1_17	c1_18	c1_19	c1_20	c1_21	c1_22	c1_23	c1_24	c1_25	c1_26	c1_27	c1_28	c1_29	c1_30	c1_31	c1_32	c1_33	c1_34	c1_35	c1_36	c1_37	c1_39	c1_41	c1_43	c1_45	c1_49	c1_55	c1_57	c1_59	c1_60	c1_61	c1_62	c1_64	c1_65	c1_66	c1_67	c1_68	c1_71	c1_75	c1_76	c1_78	c1_80	c1_84	c1_86	c1_92	c1_93	c1_95	c1_96	c1_97	c1_98	c1_99	c1_101	c1_102	c1_104	c1_107	c1_109	c1_115	c1_117	c1_123	c1_127	c1_129	c1_130	c1_133	c1_137	c1_145
FTH1	GLDN	FTH1	AC120193.1	SLC26A3	TMEM163	SRGAP2B	IQGAP2	GLDN	RGS1	DDIT4	DUSP1	HIF1A	FTH1	CD74	HSP90B1	AC120193.1	AP003086.2	N4BP2L2	SLC26A3	MSR1	CTNNA2	DOCK4	TMEM163	NCK2	SCFD2	SRGAP2B	ELMO1	IQGAP2	GPNMB	ATG7	ASAH1	KCNMA1	CPED1	RGS1	DUSP1	FTH1	AC120193.1	AP003086.2	XIST	MSR1	F13A1	HSPH1	DOCK4	ANKRD44	TMEM163	[illegible]	SLC2A3	PTPN2	NCK2	SCFD2	SRGAP2B	ANKS1A	ARID1A	ATG7	KCNMA1	MSR1	IPCEF1	F13A1	RASGEF1C	NAV3	DPYD	MGAT4A	HSPH1	AP001977.1	TMEM163	SLC2A3	SRGAP2B	ANKS1A	MGAT5	P2RY12	F13A1	RASGEF1C	FRMD4A	DPYD	TMEM163	ANKS1A
[illegible]	[illegible]	[illegible]	[illegible]	[illegible]	[illegible]	[illegible]	[illegible]	[illegible]	[illegible]	[illegible]	[illegible]	[illegible]	[illegible]	[illegible]	[illegible]	[illegible]	[illegible]	[illegible]	[illegible]	[illegible]	[illegible]	[illegible]	[illegible]	[illegible]	[illegible]	[illegible]	[illegible]	[illegible]	[illegible]	[illegible]	[illegible]	[illegible]	[illegible]	[illegible]	[illegible]	[illegible]	[illegible]	[illegible]	[illegible]	[illegible]	[illegible]	[illegible]	[illegible]	[illegible]	[illegible]	[illegible]	[illegible]	[illegible]	[illegible]	[illegible]	[illegible]	[illegible]	[illegible]	[illegible]	[illegible]	[illegible]	[illegible]	[illegible]	[illegible]	[illegible]	[illegible]	[illegible]	[illegible]	[illegible]	[illegible]	[illegible]	[illegible]	[illegible]	[illegible]	[illegible]	[illegible]	[illegible]	[illegible]	[illegible]	[illegible]	[illegible]

CSAR1	CMPK1	SMARCA5	CLASP1	ASPH	CHIC2
TRA2A	IDE	ITFG2	LRRC37A2	POM121	TSC22D2
CORO1C	ACADVL	SCRN1	UTP23	JAK3	NCOA2
ANP32A	PSMB1	PPIL4	LMBRD1	KSR1	KIAA0319L
STAT6	SLC12A7	RBM12B	SNAP23	DNM1L	LRIG2
CCNI	MRPS5	TAF7	POU2F2	RAB3GAP2	NFIC
ARPC5	ZBTB40	PWWP2A	ZDHHC14	PMM2	CLTC
TMEM144	CCT6A	MFSD8	AC098850.3	HNRNPR	STXBP3
C9orf72	VCP	FBXO28	FMNL3	HEATR1	THADA
MKLN1	ZUP1	ANXA11	ZFX	INO80	FNBP1L
MNDA	SLC25A46	TBC1D23	UBXN2B	SLC25A13	PSIP1
WNK1	QSOX1	CWC22	C11orf80	EIF3A	ANKIB1
B4GALT1	PABPC4	XPA	ARHGAP20	PPP2R5E	AC097376.2
KDM1B	TMEM41B	CDC40	DEPTOR	TANGO6	VGLL4
NR1D2	RASSF4	RBL2	TNFAIP8	FKBP15	CSTF3
PDK3	KLHL36	TMEM63A	TNIK	UGP2	PTAR1
DAZAP2	ATF4	ZNF84	S100Z	TCERG1	ATP11B
NAP1L1	POLR1D	ZC3H14	CLECL1	COMMD10	SERTAD2
CAPZA1	GFPT1	PATL1	ABR	SETX	FAM114A1
CDC42	AKIRIN2	DNASE1	SYT17	SARS	ZMYND8
BTG2	ATP6V1A	C1orf52	AUTS2	UIMC1	AL359762.3
CTSH	MTPN	ARL6IP6	RC3H1	PBRM1	RBM33
PPP6R3	UBAP2L	C8orf37-AS1	DST	EIF4E	ERI3
KDM4B	FANCC	TAMM41	ASXL2	OTUD4	YTHDC1
PRKCD	UNC93B1	TSTD2	LIPC	GSPT1	ATP2B1
C1orf162	CD4	MTERF4	HPS3	AL645568.1	CEP350
SSH1	ERI1	SLC35B3	BCYRN1	SH2B3	SCAF8
NUS1	DDX18	CTR9	SMIM35	LCLAT1	JMJD1C
HLA-DQB1	CNOT7	ABCB10	PACS1	NASP	RNF145
ARL17A	SELENOF	STX17	GAL3ST4	HK1	NUTM2A-AS1
CHSY3	HPS5	AC007038.1	USPL1	ARL8B	PPP4R3B
TBCA	PLA2G16	PIP4P2	VASH1	UBE2D2	FTCDNL1
EIF3H	TXNL1	PUS10	SLC38A2	VPS53	FAM13B
KIAA1147	EIF4EBP2	NPHP4	JARID2	ZNF277	XRCC5
LINC01736	LINC00869	DCP1A	SNX27	SLC20A1	KPNA4
HNRNPDL	FYTTD1	RCHY1	ELAVL4	G3BP1	RCOR3
CNBP	BSDC1	PPM1D	GAK	GPATCH2	CWC27
FRYL	SMAD5	LINC00174	AKAP13	ABCE1	SPOPL
ZNF207	HACD2	NUDT5	PDE6D	TMBIM1	C12orf4
M6PR	TRNT1	CYP20A1	SUCLG2-AS1	XRCC4	DAP
CREB3L2	C3orf38	NUP133	WLS	SRFBP1	UBE2K
IKBKB	LBR	MKRN1	AC090630.1	SELENOI	QSER1
ATP6V0B	UBXN2A	RO60	ELOVL5	SH3GLB1	CLINT1
HMGN3	POGLUT1	ZBTB43	KCNMB4	DCAF1	SRPK2
SRSF11	TRAPPC13	NEK11	BTBD9	GAPVD1	PLEKHM3
PPT1	APLP2	IDH1	INPP4B	TNKS	KIF13B
CMTM7	NISCH	RUFY1	MYO5A	ELP3	ANAPC1
RAF1	POMT1	TCAIM	KAT6A	TLK1	HERC3
PLEKHA2	SCAMP2	WASL	WDR26	ATP1A1	THRAP3

[illegible]

TTC1
MFSD14B
CACNB4
ARHGEF7
RNF19B
GRK2
SCAMP1
PTPN18
SEC62
GLG1
PLD3
AC008105.3
LNPK
GTPBP10
TUBGCP2
PIK3C2A
ARAP1
SLC46A3
ZNF106
WTAP
DOCK7
ZSWIM8
SMIM14
STK32B
KBTBD2
IP6K1
FERMT3
TBK1
RAB11A
SKP1
ELMOD3
DCAF10
SLC25A36
RPN1
DPP8
MSRA
TXNL4A
WBP1L
MTFR1
MTA3
YIPF4
UBE4B
CHFR
CARF
RSBN1L
RSRC2
INTS3
FDFT1
BCKDHB
[illegible]

GMIP
MPP5
BRD4
ARHGEF9
HKR1
SLC25A53
RBM23
MZF1
EPG5
DERA
SHOC2
CCDC130
STAT5A
NDUFV3
DDX42
SIN3B
BRD1
ZNF44
VPS26C
MAU2
SAFB2
ZFP14
XRCC6
TMEM164
CIAO2A
TERF2IP
CLPX
CSE1L
AC025682.1
CHEK2
COG7
CD99L2
ZNF337
NPRL3
NUP85
MARK4
ZNF611
PSMD5
PSMD12
PPM1F
LINC01278
AMFR
PRR12
RNF214
CDK12
TMEM62
ZNF235
TSPAN3
AFG3L1P
[illegible]

MACROD1
TMEM161B-AS1
TOM1
PIGG
MBD5
KCNMB1
PRIM2
DEAF1
ZFYVE9
USP12
SLC25A29
AHDC1
ZFAT
TNFSF8
SAMD9
ATF1
FGD3
CEP152
ITGA5
USF3
MINDY2
PTPN9
MAN2A1
ARHGEF12
TRIM4
IGF1
LIN54
HDX
LNCAROD
RAP2C-AS1
IRS2
CCT6P3
CASP8AP2
TNFSF13B
LINC00476
AL049828.1
AK9
PACRGL
AC124319.1
CUL4A
ZBTB8OS
SKP2
GABPB2
DLG2
NCAPD3
CCDC88A
CRYL1
ZRANB2-AS2
DPH6
[illegible]

ZNF248
FUBP3
MLLT1
MLH3
PDHX
MTMR2
KIAA0586
LIMK2
RUNX1
TYMP
ADGRE2
SETBP1
PREX1
SEC14L1
DDX21
NFATC2
HIVEP2
FAM20A
MBP
SMAD3
ALOX5
NLRC5
MIR646HG
SIGLEC9
PLAUR
CD93
RPS6KA2
PACSIN2
XRN2
CCDC26
CTSC
SLC11A2
TSPAN14
ACACA
TRPM2
LILRB1
PXN
SLA
RB1
B4GALT5
ANOS1
ATP11C
VTI1A
GRAMD1A
TLE3
CCSER2
DNAJC5
AARS
RASSF2
[illegible]

XRN1
EDEM3
KLHL20
SRRM1
DYNC1LI1
KPNA3
ATP6V1H
MTR
FOXN3
ZMYM5
LRP12
ZNF518A
PGGT1B
CTNND1
CEP192
TMEM168
TBC1D4
CSNK2A1
NEK4
CCM2
NADSYN1
MCCC1
B3GALNT2
TTF2
BTBD1
IPO8
AC007000.3
UBE3D
PDPR
ALMS1
CTIF
NDE1
REEP3
PRPF39
COG2
ZNF592
KIF2A
SHQ1
SIMC1
FAM185A
ZMYM1
SGK3
RECK
STAM
IPO9
FAM149B1
ECHDC1
PIGK
ZNF251
[illegible]

UACA
ZNF767P
AC005258.1
LMF1
RIMKLB
MNAT1
NBDY
NBR2
ACSS2
ZSCAN5A
MTMR1
NUP50-DT
ZNF24
VRK1
ARMCX5-GPRASP2
TERF2
ZNF43
BCLAF3
SLC25A43
RAB40C
CEP290
MBNL3
MICAL3
KIAA0825
PCID2
AC073529.1
STX5
AC009120.2

EZH1
BLZF1
NUP160
TMTC3
RNF115
EEA1
RNF170
MRPS31
KLHL9
KIAA2026
VPS45
ABHD18
ANO10
STX18-AS1
MTREX
C1GALT1
PHF14
SZT2
ARL14EP
RC3H2
BMT2
PNPT1
ARSB
AC079781.5
YY1AP1
SECISBP2
PIK3R4
LPGAT1
PANK2
TMEM245
GNPTAB
SVIL-AS1
ZMYND11
KRAS
NEK9
BTBD3
ZNF276
PTCD3
FBXW2
VAC14
PHF11
HECA
PIK3R5
DPY19L1
XRRA1
DYNC1I2
DCLRE1C
KCTD3
RIC8B
[illegible]

OSTF1
ZNF431
RALY
EMID1
BRWD1
BRAF
MLLT6
MAST3
MCM3AP
TPK1
NFATC3
RAP1GAP2
TNRC6A
YLPM1
CLCN5
EWSR1
LSM14A
PIAS2
ATRN
TAF1
TMLHE
MYO15B
RABGAP1
STK38L
SYNRG
MYH9
FAM133B
PITPNB
AP1G1
MED14
ARHGAP32
TTC13
RALGAPB
UHRF1BP1L
LINC01004
CASK
MAP2K4
ZNF641
MFSD11
SMC5
HECTD4
NOD1

38961

UQCRC2
ATG5
ZZEF1
MAPK1
SFSWAP
WWC2
[illegible]

DHX35
ZNF562
LRRC28
NNT
NEMF
ARMCX4
SYNJ1
TARBP1
GGNBP2
ZNF236
NEK3
LINC00894
TBCD
POLI
EFL1
METTL25
DIS3L
EIF2AK4
IFNAR1
TMEM260
MAP3K4
RFX7
SMARCA1
IST1
PCNX4
TCF20
RAB22A
RBM17
DGCR2
ARHGEF1
RPRD1B
FAM204A
G2E3
UPF1
THOC1
SIRT1
USP42
MGME1
DCP1B
ZNF81
AREL1
MAPK8IP3
CDC14B
NCBP3
MCM9
SMYD4
PIWIL4
CPSF2
PHF8
[illegible]

VPS35
ZNF138
AC132008.2
RNF169
DCAF17
CD4
ERI1
SRRM2
GOLGA1
DDX18
CNOT7
SELENOF
ZC3H18
HPS5
NIN
EMSY
IP6K2
ZNF141
ZCCHC17
KIAA0556
PLA2G16
ABCD3
VCL
ODF2L
TXNL1
NR2C1
SLC7A7
C6orf89
EIF4EBP2
LINC00869
VHL
ATP6V0A2
FYTTD1
DAB1
BSDC1
SPAG16
PDE6D
SMAD5
NPAT
LINC02649
ATG13
LRSAM1
RBM45
HACD2
PIK3R6
CCDC84
MIA3
TRNT1
USP47
SMG1P3
C3orf38
RALA
MAX
PNPLA8
LBR
ZNF720
UBXN2A
POLR3C
POGLUT1
TRAPPC13
DNHD1
FAM168B
ORC3
APLP2
NISCH
SBNO1
REST
TMEM268
PCMT1
SCAMP2
PDXDC1
MICAL1
HAT1
SEC13
NUP54
EPM2AIP1
GIT2
NCKAP5L
LINC01376
RSBN1
DPP7
TAOK1
TBC1D19
INPP5F
MSH2
AFG1L
HADHA
ANKLE2
TRIM56
CRTAP
ITPRID2
SLTM
PRKCI
P4HB
AASDH
SLC30A5
LRRC40
UBE3B
DSTYK
NKTR
SNRNP40
DNAJC9
RALB
RAD18
QTRT2
COPB2
GOLGA5
AKIRIN1
LACTB
ZBTB11
NFXL1
TSC1
SWT1
AP5M1
RRP15
SLC29A3
WDR6
DHX29
RNF168
DENND4B
CTBP1
SLC25A16
TMEM106B
PRPF3
CCDC82
POLN
SUPT5H
STARD7
FUT10
EIF2AK1
NPTN
LAMP1
DLD
CCR1
RUSC2
STYXL1
CDC123
GRSF1
KIFAP3
MPC1
TRIQK
TSC22D4
GABPB1-AS1
PCED1B-AS1
ZNF271P
PGK1
KDM2B
NARS2
SELENOT
CNDP2
VPS35L
EPM2A
QRICH1
SRPK1
LST1
LRP6
PSMD11
NFIC
CCDC186
LMO4
KHDC4
STOM
RNF150
G3BP2
TMEM176B
STAG3L2
SSR1
SNHG14
SMC6
ANKRD49
CDH23
AC245297.3
MTCH1
AL354744.1
PLA2G15
TMEM123
RPA1
TAF2
CLTA
TOR1AIP1
PKP4
CEBPZ
CNTNAP2
HSF1
IRAK4
EXOSC10
CNOT1
ARHGEF40
DUT
SMC3
SUGP2
ZNF708
SUB1
METTL6
TTF1
PEX13
TMEM33
ANKRD11
NRDE2
MAML1
VPS39
RARS
DHRS7
IMMP1L
CAMK2G
ATL3
OS9
SIPA1
RAB39A
RNF166
GAS6-AS1
BMS1
STK38
ZNF197
NPLOC4
ADCY9
RAB11FIP3
SLC7A6OS
PMEPA1
TRIP11
MAF
KAT6B
GANAB
TCAF1
PRDM10
YME1L1
PFDN1
FBXL3
DLST
WIPF2
NIFK-AS1
PREPL
TCF25
SETD7
GSTK1
RPN2
MGRN1
FBXO21
SART3
SIAH1
CDC37
PRKACA
TLN1
OSTM1
MRPS25
EIF4H
CERS4
BTN3A2
TMEM248
WASHC5
PTTG1IP
SIN3A
TTLL5
LMAN1
DDB1
GOLGA3
CHCHD7
SLC43A2
HDAC2
PPTC7
AC245060.5
BRD2
UFM1
SLC25A12
SUPT16H
RBBP1
ZNF675
DSC2
CATSPER2
SCYL2
ACTR10
LCMT1
RNPEP
PNN
HPS4
ZDHHC7
RNPS1
ACBD5
OTUD5
CYB5B
BLMH
CDK5RAP1
UBE2I
DNMT1
GPCPD1
SCAP
TTYH2
DYNC1LI2
RNF34
CMTR2
EPN2
RNF10
SRP54
MAPKAPK5
PRRC2A
CBX5
AC108866.1
STAT2
LMBRD2
ACSF3
MRPS9
IDH2
RPS6KA4
RPS11
AL009174.1
RPS4X
RPL28
RPL38
RPS15
RPL10
RPS9
RPL13A
FOS
AC134878.2
GPR34
GPR183
RPS29
ATP8B1
ZFP36
CST3
CEBPB
MAML2
CTSZ
ITGB2
CACNA1A
AP1B1
SESN3
TMBIM6
TBC1D22A
CCDC200
PKM
GNAS
SSH2
EPS8
VMP1
RAB31
LILRB4
SRSF5

RYR1
YPEL3
EP300
STAG2
ALG13
DOCK11
RELCH
LRBA
TTC28
LPXN
TTC17
ULK2
YTHDC2
PPP1R12A
HECTD2
MAP2K6
JAM2
CLEC2D
RBM26
RAP1B
SNRNP70
TRAPPC8
SMAD2
PDS5A
HELZ
GPATCH8
CD276
PHKB
ZDHHC21
C2CD5
GLCE
RPS27L
PAFAH1B1
ARID2
MAP2K1
NLK
LONP2
EFCAB13
MAP2K5
WDR7
IMMP2L
ZRANB1
BTBD7
ME2
CUL5
NPC1
HECTD1
HMBOX1
OSTF1
ZNF431
RALY
EMID1
KIZ
TRAF3IP2-AS1
BRWD1
BRAF
TTBK2
MLLT6
R3HDM2
GAA
MAST3
OSBPL1A
AXL
MCM3AP
NKAPP1
CDC27
FUS
TPK1
NOL4L
APAF1
NFATC3
RAP1GAP2
TNRC6A
SPG11
YLPM1
ZBTB44
CLCN5
EWSR1
LSM14A
PIAS2
RB1CC1
SOGA1
39508
ATRN
KIF16B
N4BP2L1
TAF1
TMLHE
MYO15B
RABGAP1
STK38L
SYNRG
MYH9
FAM133B
PITPNB
AP1G1
MED14
ZADH2
ARHGAP32
TTC13
RALGAPB
SLC38A10
UHRF1BP1L
LINC01004
CASK
MAP2K4
SLC35E3
ZNF641
MFSD11
SLC12A6
SMC5
HECTD4
MIRLET7BHG
NOD1
MARF1
38961
UQCRC2
ATG5
ZZEF1
MAPK1
DCAF7
SFSWAP
WWC2
KMT5B
ZBED5
CIRBP
MPRIP
CHM
PHKA
COA1
AP2B1
SLX4IP
NCOA6
SPPL2A
PHF6
WDR37
TBL1X
THOC2
CSK
ICE2
USP25
CUL2
SMG1P5
HMG20A
NDUFS4
RPRD1A
ASCC2
MMD
COX10
CTNNBL1
EVI2B
PHLPP1
WBP2NL
NFATC2IP
WASHC3
SUCLA2
ABCB7
RNASEH2B
FBXO34
ATAD1
OFD1
BTK
RMDN1
INTS6L
ANGEL1
RTF2
CCDC90B
ACSF2
UQCC1
TMEM87A
DHX35
ZNF562
LRRC28
PCSK7
NNT
NEMF
ARMCX4
SYNJ1
TBC1D15
TARBP1
GGNBP2
FAM122B
ZNF236
RBM41
ANKRD13A
NEK3
SINHCAF
PISD
NBR1
FBXO38
LINC00894
TBCD
POLI
EFL1
METTL25
DIS3L
NSF
EIF2AK4
APPBP2
WWP2
IFNAR1
TMEM260
MAP3K4
RFX7
SMARCA1
IST1
PCNX4
TCF20
RAB22A
RBM17
DGCR2
ARHGEF1
RPRD1B
VIRMA
PHACTR2
FAM204A
G2E3
ARHGAP45
UPF1
USP16
THOC1
SIRT1
PUDP
USP42
SLC39A9
BPTF
CTPS2
MPHOSPH9
MGME1
DCP1B
ZNF81
RFWD3
AREL1
KMT2A
ATG2B
IKBIP
MAPK8IP3
CDC14B
NCBP3
MCM9
SMYD4
PPARA
PIWIL4
CPSF2
PHF8
PPP6C
TNRC6C
MRE11
SNW1
CRYBB2P1
SMG9
FAM120C
METTL16
PPP2R2D
YY1
XPO7
PIP4K2B
MYO19
ZNF217
MAN2C1
IRF3
ANKRD26
SAP30BP
ADCK1
SMAD4
FDX1
CDKL5
PEX2
VTA1
TRIM37
ZNF33B
FMR1
TEX10
ESCO1
MIPOL1
ZNF107
CRADD
RAB4A
SAE1
YARS2
AL035587.1
RSPRY1
LINS2
AC078881.1
BAZ2A
RPS6KB1
COG6
SLC25A17
DPM1
CSAD
GABPA
ZBTB4
CEP57
NAPB
CWF19L2
SSBP4
FBXO22
KAT7
WDR59
GPALPP1
INPPL1
DAZAP1
KIAA0753
KMT2D
AKAP8L
SFMBT1
RRP7BP
TPTE2P5
TAF4
C16orf72
ZFP90
MOB3A
ASB7
RBBP6
AKAP11
AMN1
TBC1D22B
ECD
PIP5K1C
ZNF41
ZNF143
KLHL22
C16orf87
ZNF594
ZBTB41
DCAF12
ZFP64
AC091948.1
GNA15
TAB3
CCNT1
GABPB1
STXBP4
UBLCP1
CEP89

ARMCX4
SYNJ1
TARBP1
GGNBP2
FAM122B
ZNF236
RBM41
ANKRD13A
NEK3
SINHCAF
PISD
FBXO38
LINC00894
TBCD
POLI
EFL1
METTL25
DIS3L
NSF
EIF2AK4
APPBP2
WWP2
IFNAR1
TMEM260
MAP3K4
RFX7
SMARCA1
IST1
PCNX4
TCF20
RAB22A
RBM17
DGCR2
ARHGEF1
RPRD1B
VIRMA
PHACTR2
FAM204A
G2E3
ARHGAP45
UPF1
USP16
THOC1
SIRT1
PUDP
USP42
SLC39A9
MPHOSPH9
MGME1
DCP1B
ZNF81
RFWD3
AREL1
IKBIP
MAPK8IP3
CDC14B
NCBP3
MCM9
SMYD4
PIWIL4
CPSF2
PHF8
PPP6C
TNRC6C
MRE11
SNW1
CRYBB2P1
SMG9
FAM120C
METTL16
PPP2R2D
YY1
XPO7
PIP4K2B
MYO19
ZNF217
MAN2C1
SAP30BP
ADCK1
SMAD4
FDX1
CDKL5
PEX2
VTA1
TRIM37
FMR1
TEX10
ESCO1
MIPOL1
ZNF107
RAB4A
SAE1
YARS2
AL035587.1
RSPRY1
LINS2
AC078881.1
RPS6KB1
COG6
SLC25A17
DPM1
CSAD
GABPA
CEP57
NAPB
CWF19L2
SSBP4
FBXO22
KAT7
WDR59
GPALPP1
INPPL1
DAZAP1
KIAA0753
KMT2D
AKAP8L
SFMBT1
RRP7BP
TPTE2P5
TAF4
C16orf72
ZFP90
MOB3A
ASB7
RBBP6
AKAP11
AMN1
TBC1D22B
ECD
PIP5K1C
ZNF41
ZNF143
KLHL22
ZNF594
ZBTB41
DCAF12
ZFP64
AC091948.1
GNA15
TAB3
CCNT1
GABPB1
STXBP4
UBLCP1
CEP89
PHF2
ENTPD4
BTBD2
RPAP3
CEP135
AP003108.2
AMMECR1
HEXD
ZNF621
ZNF133
ACAD10
PHKA2
AP001992.1
LINC00662

RGS10
SELPLG
HMGN1
DDX17
RAB20
FTX
MALT1
SEL1L
CD226
USP9X
EBI3
SMURF2
RBM39
AKAP10
TCF4
CERS5
SF1
TMEM50B
RGS19
SRSF2
SYNGR2
MYCBP2
ACSM5
RNMT
XBP1
HMOX1
ABHD12
LAIR1
PPP1R18
ZFAS1
FCGRT
MYO18A
HNRNPM
CBWD5
RBMX
UBE2G2
CHPT1
MTMR4
TMEM106A
LENG8
SPTLC3
OGT
USP36
ARIH1
ARNTL
MAGT1
LAMP2
RNF114
MAP3K14
SIGLEC10
AC138207.8
ATP6AP2
ZC3H12C
SIRPA
HOOK2
IFNGR2
CLEC2B
ZNF83
MSI2
PRPF8
ELP2
SLC23A2
MPPE1
ZNF331
ZBTB46
SON
RTCB
CXCL16
TEFM
SMCHD1
ADAMTS10
MTMR3
DDX3X
PTPRO
GYS1
SCPEP1
URI1
JKAMP
TBRG1
ADA2
DIDO1
CABIN1
KRBOX4
CERK
KCNC3
RGL3
RBM3
MINK1
IQSEC2
SMAD7
PIK3C3
PAXBP1
TXNDC11
KIAA0355
NPEPPS
37316
SAFB
SS18
SLC8B1
PRNP
TMED10
MCMBP
PARN
GOLGA8A
NORAD
RASAL3
ERCC1
ZNF254
GGA3
BRD7
SLIRP
RRP1B
CHD2
ALG5
SPAG9
PPP4R1
CYBC1
EIF2S3
NCF4
MESD
LIPE-AS1
FRG1CP
PLXNB2
WAS
SASH3
HERC2P9
TMX1
GSE1
TPM4
FOXK2
STON2
ZNF100
FAM192A
RIOK3
TRABD
ZNF430
MBTPS1
SIK2
MSL3
C16orf70
CCT8
TMX3
RRAGB
COMT
HSCB
CUEDC1
RIPOR1
PSMG2
PDPK1
SREBF2
EIF3M
AC011477.1
40057
ITPK1
SRSF6
CD99
PPP4R1L
OGFR
TRMT1
TAZ
ZNF267
TTC19
ACSS1
KIAA0100
MTG2
TAPBP
MORF4L2
SMG1P2
ZNF397
ZNF91
NAPG
VPS4B
CXorf38
LEPROTL1
FBXO7
MSL1
UNK
KIAA0930
COPB1
ZNF160
UBE2L3
SLFN11
GMIP
ILF3
PPP1CC
SERINC3
NDEL1
MPP5
ATXN2L
EPS15L1
MAN2B1
BRD4
ARHGEF9
HKR1
STMP1
ELAVL1
SLC25A53
UBA1
RBM23
TRPC4AP
MZF1
EPG5
DERA
SHOC2
SPTLC1
CCDC130
NFATC1
STAT5A
RNF146
ZBTB25
NDUFV3
DDX42
SIN3B
BRD1
ZNF44
ATP6V0D1
ESD
UBE2Z
POLR2A
VPS26C
PPP2CB
OSBP
MAU2
SAFB2
GTF2H1
HMGXB4
TMEM30A
ZFP14
XRCC6
UNC13D
NUP88
TMEM164
CIAO2A
NSFL1C
BLCAP
POC5
RP2
EIF3J
CSNK1G2
PSMC6
TERF2IP
CLPX
CSE1L
AC025682.1
CHEK2
COG7
CASC3
NARF
CD99L2
APOOL
PTBP1
ARPP19
ANKRD12
ZNF337
NPRL3
NUP85
MARK4
ZNF611
ZCCHC14
PSMD5
PSMD12
PPM1F
LINC01278
AMFR
PRR12
PHF12
GFM2
MAP1S
PNPLA6
RPAIN
TMEM230
GDI1
ANKRD27
RNF214
CDK12
GRIPAP1
TMEM62
ZNF235
TSPAN3
AFG3L1P
SMC1A
EIF4ENIF1
RLIM
PKN1
TYK2
HPRT1
RBCK1
DIS3
XPNPEP3
CSNK2A2
TAF11
IPO5
STARD3
MAGED1
MTIF3
MIATNB
PAFAH1B2
ZNF75A
DDX24
IGBP1
FAN1
ST3GAL2
TRIOBP
SREK1IP1
UPF3B
PPIP5K1
LINC00685
PTS
ZSCAN30
VPS13A
RTF1
TPGS2
CHIC1
ATP6V1E1
BAG4
NMT1
FAM19A2

PHF2
ENTPD4
BTBD2
RALBP1
RPAP3
CEP135
AP003108.2
AMMECR1
HEXD
ZNF621
ZNF133
ACAD10
PHKA2
AP001992.1
LINC00662

Supplementary Table 5_Microglia MEGENA Modules
Module description

MEGENA Gene Co-expression Module description

Co-expression modul	Module size	Parent module	Hub genes (number of edges in parentheses)
**c1_2**	1664	c1_1	DLEU7(342),AC120193.1(285),C1QC(284),DDX5(211),AC044781.1(184),FTL(170),AP003086.2(129),CD74(120),TMSB4X(83),PSAP(66),C1QB(65),RAPGEF1(65),APOE(65),RPL19(63),HIF1A(63),RGS1(60),SRGN(60),MALAT1(52),HIF1A-AS2(50),PFKFB3(49),ACTB(46),B2M(42),ABHD15-AS1(38),CTSB(31),TTC7A(31),FTH1(28),ANKUB1(28),HSPA5(28),SAT1(27),HIST2H2BB(26),N4BP2L2(25),PGK1(25),TUBA1B(24),HSP90B1(24),DNAJC3(24),WSB1(24),AC016739.1(22),HNRNPA2B1(22),FAM177B(21),FCGR3A(21),STX7(21),NEAT1(20),FRG1HP(20),TPT1(19),CPED1(19),PPP6R3(18),RAB11A(17),AC073861.1(16),ENTPD1-AS1(16),PDE8A(16),C1QA(15),AC079793.1(15),HDAC9(15),ANKRD10(14)
**c1_4**	317	c1_2	RAPGEF1(65),RGS1(59),HIF1A(59),SRGN(56),MALAT1(52),HIF1A-AS2(50),PFKFB3(49),TTC7A(31),SAT1(27),PGK1(25),NEAT1(20),CPED1(19),PDE8A(16),HDAC9(15)
**c1_5**	610	c1_2	C1QC(284),FTL(168),CD74(99),TMSB4X(83),PSAP(66),C1QB(65),APOE(65),RPL19(63),ACTB(46),B2M(42),CTSB(31),HSPA5(28),TUBA1B(24),HSP90B1(24),DNAJC3(24),FTH1(23),AC016739.1(22),FCGR3A(21),TPT1(19),AC073861.1(16),C1QA(15)
**c1_6**	737	c1_2	DLEU7(341),AC120193.1(284),DDX5(211),AC044781.1(184),AP003086.2(129),ABHD15-AS1(38),ANKUB1(28),HIST2H2BB(26),N4BP2L2(25),WSB1(24),HNRNPA2B1(22),FAM177B(21),STX7(21),FRG1HP(20),PPP6R3(18),RAB11A(17),ENTPD1-AS1(16),AC079793.1(15),ANKRD10(14)
**c1_7**	983	c1_3	HSPH1(103),DPYD(92),PICALM(88),AP001977.1(87),P2RY12(78),RASGEF1C(73),NAV3(63),ANKRD44(60),CD163(57),FRMD4A(51),SORL1(46),IPCEF1(43),XIST(42),KCNQ3(39),PALD1(38),MGAT4A(36),LSAMP(33),LINGO1(32),F13A1(32),SYNDIG1(28),ACSL1(27),IFI44L(26),HSP90AA1(25),A2M(25),CHORDC1(24),AC008691.1(24),SOCS6(24),TLN2(24),EPSTI1(24),FRMD4B(23),UTY(22),ABCC4(22),JAZF1(22),LRRK2(21),CTNNA2(20),NTM(19),DOCK8(19),LINC02232(18),TGFBI(18),C3(18),OLR1(18),AC007262.2(18),LINC00278(16),AC011586.2(15),ZNF804A(15),SLC26A3(14),HSPB1(14),DOCK4(14)
**c1_9**	885	c1_3	DENND3(172),SLC11A1(158),GRB2(140),SND1(117),TMEM163(101),PTPN2(100),TGFBR1(79),SH3RF3(76),TBC1D14(67),SCFD2(62),NCK2(58),PVT1(50),PRKCA(46),ERC2(35),SUSD6(28),IARS(26),ZFAND3(26),BIRC6(26),LYN(25),RNF149(22),HIVEP3(20),WDFY3(19),GNAQ(18),AL034397.3(17),BACH1(17),MAPKAP1(16),VSIG4(16),PAG1(15),ETV6(15),IL4R(15),SLC2A3(14),AC011933.4(14)
**c1_10**	1362	c1_3	SRGAP2B(674),SRGAP2(394),SRGAP2C(347),CSGALNACT1(166),IL6ST(132),HNRNPC(97),TCF12(92),ITM2B(83),FGD2(79),MED13L(75),CX3CR1(37),HS3ST4(37),ARHGAP24(34),TENT2(33),ELMO1(31),PTPRJ(30),CCND3(27),SMAP2(27),KANSL1(25),ATP2C1(21),ANKS1A(21),ANKRD17(19),CSF1R(18),APBB1IP(17),CAB39(16),ZMAT3(15),MGAT5(15),BHLHE41(15),RASA1(15),ACTR2(14)
**c1_11**	237	c1_3	ASAH1(73),GPNMB(68),ATG7(65),KCNMA1(64),STARD13(61),NPL(28),IQGAP2(21),CPVL(18),TANC2(18),MERTK(17),MITF(16),SNX29(15)
**c1_12**	56	c1_4	MALAT1(42),CPED1(17),HDAC9(15)
**c1_13**	66	c1_4	PFKFB3(44),TTC7A(31),RGS1(29)
**c1_15**	51	c1_4	HIF1A-AS2(49),PGK1(25)
**c1_16**	64	c1_4	SRGN(50),SAT1(21)
**c1_17**	72	c1_4	RAPGEF1(64),HIF1A(28),NEAT1(20)
**c1_18**	231	c1_5	FTL(146),TMSB4X(80),RPL19(63),ACTB(46),TUBA1B(24),FTH1(23),AC016739.1(22),TPT1(19),AC073861.1(16)
**c1_19**	344	c1_5	C1QC(283),CD74(86),PSAP(66),APOE(65),C1QB(64),B2M(35),CTSB(27),FCGR3A(21),C1QA(14)
**c1_20**	34	c1_5	DNAJC3(23),HSP90B1(19)
**c1_21**	286	c1_6	AC120193.1(203),AC044781.1(116),ABHD15-AS1(38),FAM177B(21),ANKUB1(18),RAB11A(17),ENTPD1-AS1(16),HIST2H2BB(14),WSB1(14)
**c1_22**	366	c1_6	DLEU7(336),AP003086.2(78),AC079793.1(13),DLEU1(13)
**c1_23**	84	c1_6	DDX5(75),HNRNPA2B1(21),N4BP2L2(20),PPP6R3(17),STX7(16)
**c1_24**	87	c1_7	XIST(37),LINGO1(32),IFI44L(26),EPSTI1(24),UTY(22)
**c1_25**	699	c1_7	HSPH1(103),DPYD(92),AP001977.1(87),P2RY12(78),RASGEF1C(73),NAV3(62),CD163(57),IPCEF1(43),FRMD4A(42),KCNQ3(39),PALD1(38),MGAT4A(36),F13A1(32),ACSL1(27),SYNDIG1(26),HSP90AA1(25),A2M(25),CHORDC1(24),AC008691.1(24),SOCS6(24),TLN2(24),FRMD4B(23),JAZF1(22),LRRK2(21),DOCK8(19),TGFBI(18),C3(18),OLR1(18),AC007262.2(18),LINC02232(16),AC011586.2(15),ZNF804A(15),HSPB1(14)
**c1_26**	54	c1_7	LSAMP(33),NTM(19),CTNNA2(18)
**c1_27**	141	c1_7	PICALM(88),ANKRD44(57),SORL1(40)
**c1_28**	633	c1_9	DENND3(172),SLC11A1(158),GRB2(140),PTPN2(100),TMEM163(98),TBC1D14(67),SH3RF3(65),PRKCA(46),ERC2(33),SUSD6(28),ZFAND3(26),LYN(25),RNF149(22),HIVEP3(20),WDFY3(19),BACH1(16),ETV6(15),IL4R(15),SLC2A3(14),AC011933.4(14)
**c1_29**	109	c1_9	TGFBR1(76),NCK2(52),GNAQ(18),PAG1(15)
**c1_30**	143	c1_9	SND1(117),SCFD2(44),PVT1(29),BIRC6(26),IARS(20),MAPKAP1(16)
**c1_31**	1168	c1_10	SRGAP2B(674),SRGAP2(394),SRGAP2C(347),IL6ST(127),HNRNPC(97),TCF12(92),FGD2(79),MED13L(75),CX3CR1(37),TENT2(33),PTPRJ(30),KANSL1(25),ANKS1A(21),ANKRD17(19),CSF1R(18),APBB1IP(17),ATP2C1(17),CAB39(16),ZMAT3(15),MGAT5(15),BHLHE41(15),RASA1(15),ACTR2(14)
**c1_32**	194	c1_10	CSGALNACT1(147),ITM2B(60),ARHGAP24(34),ELMO1(31),HS3ST4(28),CCND3(27),SMAP2(22)
**c1_33**	26	c1_11	STARD13(20),IQGAP2(16)
**c1_34**	27	c1_11	GPNMB(23)
**c1_35**	81	c1_11	ATG7(62),NPL(24),MERTK(17)
**c1_36**	69	c1_11	ASAH1(67)
**c1_37**	34	c1_11	KCNMA1(25),CPVL(12)
**c1_39**	50	c1_12	MALAT1(42),CPED1(17)
**c1_41**	45	c1_13	PFKFB3(43),RGS1(20),TTC7A(20)
**c1_43**	57	c1_16	SRGN(49),SAT1(14)
**c1_45**	179	c1_18	FTL(144),RPL19(63),TMSB4X(42),FTH1(23),AC016739.1(22),TPT1(19),AC073861.1(16)
**c1_49**	258	c1_21	AC120193.1(203),AC044781.1(92),ABHD15-AS1(38),FAM177B(21),ANKUB1(18),RAB11A(17),ENTPD1-AS1(16),WSB1(14)
**c1_55**	345	c1_22	DLEU7(336),AP003086.2(57),AC079793.1(13),DLEU1(13)
**c1_57**	41	c1_24	XIST(30),UTY(20),LINC00278(14)
**c1_59**	170	c1_25	P2RY12(72),IPCEF1(38),A2M(25),SOCS6(24),JAZF1(22),OLR1(18),DOCK8(15),AC011586.2(13),CD83(13)
**c1_60**	381	c1_25	DPYD(92),RASGEF1C(68),CD163(57),NAV3(55),FRMD4A(42),PALD1(38),MGAT4A(36),KCNQ3(33),F13A1(32),ACSL1(27),SYNDIG1(26),TLN2(24),FRMD4B(23),LRRK2(21),TGFBI(18),C3(18),LINC02232(16),ZNF804A(15)
**c1_61**	148	c1_25	HSPH1(103),AP001977.1(84),CHORDC1(24),HSP90AA1(22),AC007262.2(18)
**c1_62**	39	c1_27	SORL1(29),FMNL3(12),RASAL2(11)
**c1_64**	95	c1_27	PICALM(87),ANKRD44(38)
**c1_65**	160	c1_28	TMEM163(90),SH3RF3(40),PRKCA(28),ERC2(23),HIVEP3(20),BACH1(15)
**c1_66**	173	c1_28	DENND3(148),EIF4G3(12)
**c1_67**	202	c1_28	SLC11A1(147),TBC1D14(45),RNF149(22),WDFY3(19),ETV6(15),SLC2A3(14)
**c1_68**	98	c1_28	GRB2(85),PTPN2(33),ZFAND3(20),SUSD6(18)
**c1_71**	82	c1_29	TGFBR1(76),NCK2(29),GNAQ(18),PAG1(15)
**c1_75**	122	c1_30	SND1(117),SCFD2(37),BIRC6(26),MAPKAP1(16)
**c1_76**	871	c1_31	SRGAP2B(674),SRGAP2(246),SRGAP2C(199),IL6ST(127),HNRNPC(97),MED13L(75),CX3CR1(36),TENT2(33),KANSL1(25),ATP2C1(17),RASA1(15),CAB39(15),BHLHE41(14),ACTR2(14)
**c1_78**	219	c1_31	TCF12(90),FGD2(77),PTPRJ(25),ANKS1A(19),ANKRD17(18),MGAT5(14),PIK3R1(11)
**c1_80**	32	c1_31	()
**c1_84**	66	c1_35	ATG7(61)
**c1_86**	26	c1_37	KCNMA1(22)
**c1_92**	110	c1_59	P2RY12(71),A2M(24),SOCS6(21),DOCK8(14),AC011586.2(13)
**c1_93**	44	c1_59	IPCEF1(30),SKAP2(9)
**c1_95**	45	c1_60	CD163(30),F13A1(28)
**c1_96**	128	c1_60	RASGEF1C(60),FRMD4A(36),KCNQ3(30),SYNDIG1(19),PALD1(18),C3(16)
**c1_97**	37	c1_60	NAV3(31)
**c1_98**	125	c1_60	DPYD(89),ACSL1(24),FRMD4B(23),LRRK2(21),TGFBI(17)
**c1_99**	30	c1_60	MGAT4A(27)
**c1_101**	80	c1_61	HSPH1(70),CHORDC1(21),HSP90AA1(17)
**c1_102**	60	c1_61	AP001977.1(56),AC007262.2(15)
**c1_104**	130	c1_65	TMEM163(90),PRKCA(28),ERC2(23),HIVEP3(20),SH3RF3(18),BACH1(15)
**c1_107**	185	c1_67	SLC11A1(147),TBC1D14(32),RNF149(22),WDFY3(16),ETV6(15),SLC2A3(14)
**c1_109**	784	c1_76	SRGAP2B(674),SRGAP2(246),SRGAP2C(199),HNRNPC(97),MED13L(75),IL6ST(48),TENT2(33),CX3CR1(29),KANSL1(25),RASA1(15),CAB39(15)
**c1_115**	96	c1_78	FGD2(77),PTPRJ(25),ANKS1A(11)
**c1_117**	105	c1_78	TCF12(89),ANKRD17(18),MGAT5(13)
**c1_123**	90	c1_92	P2RY12(71),A2M(18),DOCK8(14)
**c1_127**	28	c1_95	F13A1(24)
**c1_129**	78	c1_96	RASGEF1C(58),KCNQ3(20),SYNDIG1(15)
**c1_130**	26	c1_96	FRMD4A(23)
**c1_133**	114	c1_98	DPYD(89),FRMD4B(23),LRRK2(21),TGFBI(17)
**c1_137**	116	c1_104	TMEM163(90),ERC2(23),SH3RF3(18),PRKCA(16),BACH1(15)
**c1_145**	82	c1_115	FGD2(77)

Supplementary Table 5_Microglia MEGENA Modules
Module 11 FE

geneset	description	size	overlap	odds_ratio	pval	FDR	database	X.Log10.FDR.	genes
GO:0010885	regulation of cholesterol storage	12	4	42.21	0.000009	0.012	GO	1.92082	ABCA1;SCARB1;PPARG;ABCG1
GO:0010887	negative regulation of cholesterol storage	6	3	84.08	0.000032	0.022	GO	1.65758	ABCA1;PPARG;ABCG1

Supplementary Table 5_Microglia MEGENA Modules
Module 34 FE

geneset	description	size	overlap	odds_ratio	pval	FDR	database	X.Log10.FDR.	genes
GO:0010887	negative regulation of cholesterol storage	6	2	384	0.000028	0.0051	GO	2.29	ABCA1;ABCG1
GO:0032366	intracellular sterol transport	7	2	307.18	0.00004	0.0051	GO	2.29	ABCA1;ABCG1
GO:0055091	phospholipid homeostasis	8	2	255.97	0.000053	0.0051	GO	2.29	ABCA1;ABCG1
GO:0032367	intracellular cholesterol transport	10	2	191.96	0.000084	0.006	GO	2.22	ABCA1;ABCG1
GO:0010885	regulation of cholesterol storage	12	2	153.55	0.00012	0.006	GO	2.22	ABCA1;ABCG1
GO:0033700	phospholipid efflux	12	2	153.55	0.00012	0.006	GO	2.22	ABCA1;ABCG1
GO:0010745	negative regulation of macrophage derived foam cell differentiation	13	2	139.59	0.00015	0.0061	GO	2.21	ABCA1;ABCG1
GO:0010888	negative regulation of lipid storage	16	2	109.66	0.00022	0.0082	GO	2.09	ABCA1;ABCG1
GO:0043691	reverse cholesterol transport	17	2	102.34	0.00025	0.0083	GO	2.08	ABCA1;ABCG1
GO:0055081	anion homeostasis	18	2	95.94	0.00029	0.0084	GO	2.08	ABCA1;ABCG1
R-HSA-194223	HDL-mediated lipid transport	19	2	90.29	0.00032	0.00653	Reactome	2.185	ABCA1;ABCG1
GO:0033344	cholesterol efflux	23	2	73.08	0.00047	0.0125	GO	1.9	ABCA1;ABCG1
GO:0010743	regulation of macrophage derived foam cell differentiation	29	2	56.82	0.00075	0.0183	GO	1.74	ABCA1;ABCG1
GO:1902652	secondary alcohol metabolic process	31	2	52.9	0.00086	0.0193	GO	1.71	ABCA1;ABCG1
R-HSA-73923	Lipid digestion, mobilization, and transport	71	4	49.51	0.0000028	0.00011	Reactome	3.959	ABCA1;NCEH1;MGLL;ABCG1
WP299	Nuclear Receptors in Lipid Metabolism and Toxicity	33	2	49.48	0.00097	0.022	Wikipathway	1.66	ABCA1;ABCG1
R-HSA-174824	Lipoprotein metabolism	34	2	47.93	0.001	0.01409	Reactome	1.851	ABCA1;ABCG1
GO:0030301	cholesterol transport	44	2	36.5	0.0017	0.0361	GO	1.44	ABCA1;ABCG1
GO:0006690	icosanoid metabolic process	46	2	34.84	0.0019	0.0368	GO	1.43	LTA4H;MGLL
GO:0055088	lipid homeostasis	49	2	32.61	0.0021	0.0391	GO	1.41	ABCA1;ABCG1
GO:0015914	phospholipid transport	58	2	27.36	0.003	0.0451	GO	1.35	ABCA1;ABCG1
GO:0042632	cholesterol homeostasis	58	2	27.36	0.003	0.0451	GO	1.35	ABCA1;ABCG1
GO:0055092	sterol homeostasis	58	2	27.36	0.003	0.0451	GO	1.35	ABCA1;ABCG1
GO:0016125	sterol metabolic process	59	2	26.88	0.0031	0.0451	GO	1.35	ABCA1;ABCG1
R-HSA-556833	Metabolism of lipids and lipoproteins	659	5	6.42	0.002	0.02053	Reactome	1.688	ABCA1;NCEH1;LTA4H;MGLL;ABCG1

Supplementary Table 5_Microglia MEGENA Modules
Module 19 FE

geneset	description	size	overlap	odds_ratio	pval	FDR	database	X.Log10.FDR.	genes
GO:0002480	antigen processing and presentation of exogenous p	9	5	72.46	0.00000017	0.000043	GO	4.3665	HLA-B;HLA-C;HLA-A;B2M;HLA-E
R-HSA-12369	Endosomal/Vacuolar pathway	12	6	58.14	2.1E-08	0.0000027	Reactome	5.5686	HLA-B;HLA-C;HLA-A;B2M;CTSS;HLA-E
R-HSA-98317	Antigen Presentation: Folding, assembly and peptide	25	11	46.35	1.2E-13	9.3E-11	Reactome	10.0315	PDIA3;SEC13;HSPA5;SAR1B;HLA-B;HLA-C;HLA-A;CALR;B2M;TAPBP;HLA-E
GO:0050686	negative regulation of mRNA processing	15	6	38.75	0.00000011	0.000031	GO	4.5086	PTBP1;RNPS1;TMBIM6;SRSF6;RBMX;SRSF10
GO:0048025	negative regulation of mRNA splicing, via spliceosom	13	5	36.22	0.0000017	0.00034	GO	3.4685	PTBP1;RNPS1;SRSF6;RBMX;SRSF10
GO:1904871	positive regulation of protein localization to Cajal bo	8	3	34.58	0.00026	0.016	GO	1.7959	CCT6A;CCT8;CCT5
GO:1904869	regulation of protein localization to Cajal body	8	3	34.58	0.00026	0.016	GO	1.7959	CCT6A;CCT8;CCT5
GO:0002474	antigen processing and presentation of peptide anti	28	10	32.66	2E-11	0.00000004	GO	7.3979	PDIA3;SEC13;SAR1B;HLA-B;HLA-C;HLA-A;CALR;B2M;TAPBP;HLA-E
GO:0033119	negative regulation of RNA splicing	18	6	29.06	0.00000039	0.000086	GO	4.0655	PTBP1;RNPS1;TMBIM6;SRSF6;RBMX;SRSF10
GO:0036500	ATF6-mediated unfolded protein response	9	3	28.81	0.00039	0.018	GO	1.7447	XBP1;HSPA5;CALR
GO:0035437	maintenance of protein localization in endoplasmic r	9	3	28.81	0.00039	0.018	GO	1.7447	PDIA3;HSPA5;OS9
GO:1904851	positive regulation of establishment of protein locali	9	3	28.81	0.00039	0.018	GO	1.7447	CCT6A;CCT8;CCT5
R-HSA-16791	Trafficking and processing of endosomal TLR	13	4	25.68	0.000054	0.002	Reactome	2.699	UNC93B1;CNPY3;CTSS;CTSB
GO:0070203	regulation of establishment of protein localization to	10	3	24.7	0.00055	0.022	GO	1.6576	CCT6A;CCT8;CCT5
R-HSA-38118	ATF6-alpha activates chaperone genes	10	3	24.7	0.00055	0.012	Reactome	1.9208	XBP1;HSPA5;CALR
R-HSA-39045	Folding of actin by CCT/TriC	10	3	24.7	0.00055	0.012	Reactome	1.9208	CCT6A;CCT8;CCT5
GO:1904816	positive regulation of protein localization to chromo	11	3	21.61	0.00075	0.025	GO	1.6021	CCT6A;CCT8;CCT5
R-HSA-90104	Calnexin/calreticulin cycle	15	4	21.01	0.0001	0.0036	Reactome	2.4437	PDIA3;GANAB;OS9;CALR
GO:1903513	endoplasmic reticulum to cytosol transport	16	4	19.26	0.00013	0.011	GO	1.9586	VCP;OS9;AUP1;HERPUD1
GO:1904951	positive regulation of establishment of protein locali	12	3	19.21	0.00099	0.032	GO	1.4949	CCT6A;CCT8;CCT5
GO:0002183	cytoplasmic translational initiation	12	3	19.21	0.00099	0.032	GO	1.4949	EIF3M;EIF2S3;EIF4H
R-HSA-38103	ATF6-alpha activates chaperones	12	3	19.21	0.00099	0.019	Reactome	1.7212	XBP1;HSPA5;CALR
R-HSA-53266	N-glycan trimming in the ER and Calnexin/Calreticuli	17	4	17.78	0.00017	0.0053	Reactome	2.2757	PDIA3;GANAB;OS9;CALR
GO:0071404	cellular response to low-density lipoprotein particle	13	3	17.28	0.0013	0.035	GO	1.4559	ITGB1;CD81;ITGB2
GO:0046641	positive regulation of alpha-beta T cell proliferation	13	3	17.28	0.0013	0.035	GO	1.4559	EBI3;RASAL3;HLA-E
GO:0030970	retrograde protein transport, ER to cytosol	18	4	16.51	0.00022	0.016	GO	1.7959	VCP;OS9;AUP1;HERPUD1
GO:1902236	negative regulation of endoplasmic reticulum stress-	14	3	15.71	0.0016	0.04	GO	1.3979	XBP1;TMBIM6;HERPUD1
GO:0032527	protein exit from endoplasmic reticulum	19	4	15.4	0.00027	0.016	GO	1.7959	VCP;OS9;AUP1;HERPUD1
GO:1904874	positive regulation of telomerase RNA localization to	15	3	14.4	0.002	0.045	GO	1.3468	CCT6A;CCT8;CCT5
GO:1901797	negative regulation of signal transduction by p53 cla	15	3	14.4	0.002	0.045	GO	1.3468	CD74;RNF34;PTTG1IP
GO:0010955	negative regulation of protein processing	15	3	14.4	0.002	0.045	GO	1.3468	PRNP;TMEM59;CTSZ
R-HSA-16493	Nef-mediates down modulation of cell surface recep	21	4	13.59	0.00041	0.0097	Reactome	2.0132	ARF1;CD4;HLA-A;B2M
GO:0019079	viral genome replication	22	4	12.84	0.00049	0.02	GO	1.699	CDC42;VCP;GRK2;CTBP1
R-HSA-12225	ROS, RNS production in response to bacteria	34	6	12.44	0.000022	0.001	Reactome	3	ATP6V1A;ATP6V0B;ATP6V0E1;NCF4;CYBA;ATP6V0D1
WP3937	Microglia Pathogen Phagocytosis Pathway	40	7	12.35	0.0000048	0.00056	Wikipathway	3.252	C1QB;C1QA;ITGB2;NCF4;TREM2;CYBA;C1QC
R-HSA-12369	Antigen processing-Cross presentation	82	13	11.15	1.4E-09	0.00000028	Reactome	6.5528	PDIA3;NCF4;HLA-B;HLA-C;CYBA;HLA-A;CTSS;TAPBP;HLA-E;SEC61A1;PSMC6;CALR;B2M
R-HSA-12369	ER-Phagosome pathway	65	10	10.67	0.00000015	0.000012	Reactome	4.9208	PDIA3;SEC61A1;PSMC6;HLA-B;HLA-C;HLA-A;CALR;B2M;TAPBP;HLA-E
R-HSA-68035	FGFR2 alternative splicing	26	4	10.5	0.00095	0.019	Reactome	1.7212	PTBP1;HNRNPM;POLR2A;HNRNPF
R-HSA-77387	Insulin receptor recycling	26	4	10.5	0.00095	0.019	Reactome	1.7212	ATP6V1A;ATP6V0B;ATP6V0E1;ATP6V0D1
WP3941	Oxidative Damage	40	6	10.24	0.000058	0.0045	Wikipathway	2.347	C1QB;CDC42;C1QA;BAG4;C3AR1;C1QC
GO:0002479	antigen processing and presentation of exogenous p	75	11	10.11	6.1E-08	0.000024	GO	4.6198	PDIA3;PSMC6;NCF4;HLA-B;HLA-C;CYBA;HLA-A;CALR;B2M;TAPBP;HLA-E
GO:0090383	phagosome acidification	27	4	10.04	0.0011	0.032	GO	1.4949	ATP6V1A;ATP6V0B;ATP6V0E1;ATP6V0D1
R-HSA-38995	Prefoldin mediated transfer of substrate to CCT/TriC	27	4	10.04	0.0011	0.02	Reactome	1.699	CCT6A;PFDN1;CCT8;CCT5
GO:0007029	endoplasmic reticulum organization	34	5	9.98	0.00027	0.016	GO	1.7959	LMAN1;SEC61A1;ATL3;TMEM33;ATL2
GO:0042590	antigen processing and presentation of exogenous p	78	11	9.66	9.2E-08	0.000031	GO	4.5086	PDIA3;PSMC6;NCF4;HLA-B;HLA-C;CYBA;HLA-A;CALR;B2M;TAPBP;HLA-E
R-HSA-43272	Lysosome Vesicle Biogenesis	35	5	9.65	0.00031	0.0084	Reactome	2.0757	ARF1;CTSZ;M6PR;CLTA;GNS
R-HSA-16495	The role of Nef in HIV-1 replication and disease path	28	4	9.62	0.0013	0.022	Reactome	1.6576	ARF1;CD4;HLA-A;B2M
R-HSA-91797	Transferrin endocytosis and recycling	29	4	9.24	0.0015	0.024	Reactome	1.6198	ATP6V1A;ATP6V0B;ATP6V0E1;ATP6V0D1
GO:1903902	positive regulation of viral life cycle	44	6	9.16	0.0001	0.0084	GO	2.0757	DDB1;CD74;CD4;DDX3X;CNOT7;ADAR
GO:0072384	organelle transport along microtubule	31	4	8.55	0.0019	0.045	GO	1.3468	CDC42;RAB1A;MAP1S;PEX14
GO:0045070	positive regulation of viral genome replication	31	4	8.55	0.0019	0.045	GO	1.3468	DDB1;DDX3X;CNOT7;ADAR
GO:0000381	regulation of alternative mRNA splicing, via spliceosc	39	5	8.51	0.00052	0.021	GO	1.6778	PTBP1;WTAP;RNPS1;SRSF6;RBMX
R-HSA-38107	IRE1alpha activates chaperones	55	7	8.49	0.000042	0.0016	Reactome	2.7959	SEC61A1;XBP1;HSPA5;GFPT1;SSR1;TLN1;ATP6V0D1
GO:0090382	phagosome maturation	32	4	8.25	0.0021	0.047	GO	1.3279	ATP6V1A;ATP6V0B;ATP6V0E1;ATP6V0D1
R-HSA-38995	Cooperation of Prefoldin and TriC/CCT in actin and t	32	4	8.25	0.0021	0.033	Reactome	1.4815	CCT6A;PFDN1;CCT8;CCT5
GO:0071357	cellular response to type I interferon	65	8	8.19	0.000015	0.0017	GO	2.7696	BST2;STAT2;HLA-B;HLA-C;ADAR;TYK2;HLA-A;HLA-E
GO:0060337	type I interferon signaling pathway	65	8	8.19	0.000015	0.0017	GO	2.7696	BST2;STAT2;HLA-B;HLA-C;ADAR;TYK2;HLA-A;HLA-E
GO:0036498	IRE1-mediated unfolded protein response	57	7	8.14	0.000053	0.005	GO	2.301	SEC61A1;XBP1;HSPA5;GFPT1;SSR1;TLN1;ATP6V0D1
R-HSA-56945	Cargo concentration in the ER	33	4	7.96	0.0024	0.036	Reactome	1.4437	LMAN1;TMED10;SAR1B;CTSZ
R-HSA-68141	Cooperation of PDCL (PhLP1) and TRiC/CCT in G-prot	42	5	7.82	0.00074	0.016	Reactome	1.7959	CCT6A;CSNK2A2;CCT8;CCT5;GNAI2
R-HSA-90973	Interferon alpha/beta signaling	68	8	7.78	0.000021	0.001	Reactome	3	BST2;STAT2;HLA-B;HLA-C;ADAR;TYK2;HLA-A;HLA-E
R-HSA-38111	Unfolded Protein Response (UPR)	86	10	7.71	0.0000022	0.00013	Reactome	3.8861	SEC61A1;XBP1;HSPA5;DIS3;GFPT1;SSR1;CALR;TLN1;ATP6V0D1;HERPUD1
GO:0018279	protein N-linked glycosylation via asparagine	44	5	7.42	0.00092	0.03	GO	1.5229	LMAN1;VCP;RPN2;ST3GAL5;UBE2G2
GO:0090304	nucleic acid metabolic process	80	9	7.41	0.0000094	0.0013	GO	2.8861	PTBP1;HNRNPM;POLR2A;WTAP;ERI1;HNRNPF;HNRNPD;CASC3;RBMX
R-HSA-38103	XBP1(S) activates chaperone genes	53	6	7.41	0.00029	0.0083	Reactome	2.0809	SEC61A1;XBP1;GFPT1;SSR1;TLN1;ATP6V0D1
GO:0044257	cellular protein catabolic process	62	7	7.4	0.000091	0.0079	GO	2.1024	CTSZ;CLTA;YME1L1;IDE;UBE2G2;CTSS;CTSB
WP183	Proteasome Degradation	62	7	7.4	0.000091	0.0054	Wikipathway	2.268	PSMC6;RPN2;HLA-B;HLA-C;UBA1;HLA-A;HLA-E
GO:0018196	peptidyl-asparagine modification	45	5	7.23	0.001	0.032	GO	1.4949	LMAN1;VCP;RPN2;ST3GAL5;UBE2G2
GO:0035722	interleukin-12-mediated signaling pathway	46	5	7.06	0.0011	0.032	GO	1.4949	CDC42;ARF1;CAPZA1;HNRNPF;TYK2
GO:0071349	cellular response to interleukin-12	46	5	7.06	0.0011	0.032	GO	1.4949	CDC42;ARF1;CAPZA1;HNRNPF;TYK2
GO:0017038	protein import	56	6	6.96	0.00039	0.018	GO	1.7447	RPAIN;PDIA3;LAMP2;NUP88;PTTG1IP;APOE
R-HSA-72163	mRNA Splicing - Major Pathway	134	14	6.91	8.1E-08	0.0000079	Reactome	5.1024	SF3B2;CASC3;USP39;PRPF8;ELAVL1;HNRNPM;PTBP1;SNRNP40;POLR2A;HNRNPF;HNRNPD;RNPS1;SRSF6;RBMX
GO:0034976	response to endoplasmic reticulum stress	106	11	6.8	0.0000022	0.00039	GO	3.4089	PDIA3;VCP;CEBPB;UFM1;TMX3;HSPA5;OS9;TMEM259;TMX1;TMEM33;HERPUD1
WP411	mRNA Processing	126	13	6.79	0.00000027	0.000064	Wikipathway	4.194	SF3B2;TMED10;SMC1A;PRPF8;HNRNPM;PTBP1;SNRNP40;POLR2A;HNRNPD;RNPS1;SRSF6;SRSF10;RBMX
GO:0002221	pattern recognition receptor signaling pathway	48	5	6.73	0.0014	0.035	GO	1.4559	UNC93B1;CD14;CNPY3;CTSS;CTSB
GO:0030433	ubiquitin-dependent ERAD pathway	58	6	6.69	0.00047	0.02	GO	1.699	VCP;PSMC6;OS9;HSPA5;AUP1;UBE2G2
R-HSA-20400	COPII (Coat Protein 2) Mediated Vesicle Transport	68	7	6.67	0.00017	0.0053	Reactome	2.2757	RAB1A;LMAN1;SEC13;TMED10;SAR1B;CTSZ;NAPG
GO:0048024	regulation of mRNA splicing, via spliceosome	60	6	6.44	0.00057	0.022	GO	1.6576	PTBP1;WTAP;RNPS1;SRSF6;SRSF10;RBMX
R-HSA-72172	mRNA Splicing	144	14	6.37	0.0000002	0.000014	Reactome	4.8539	SF3B2;CASC3;USP39;PRPF8;ELAVL1;HNRNPM;PTBP1;SNRNP40;POLR2A;HNRNPF;HNRNPD;RNPS1;SRSF6;RBMX
WP3945	TYROBP Causal Network	61	6	6.33	0.00062	0.021	Wikipathway	1.678	CD4;ITGB2;PPP1R18;TMEM106A;CXCL16;C1QC
GO:2001243	negative regulation of intrinsic apoptotic signaling pa	62	6	6.21	0.00068	0.025	GO	1.6021	URI1;XBP1;DDX3X;TMBIM6;PTTG1IP;HERPUD1
R-HSA-72203	Processing of Capped Intron-Containing Pre-mRNA	193	18	6.15	6.9E-09	0.0000011	Reactome	5.9586	FYTTD1;SF3B2;WTAP;CASC3;USP39;PRPF8;ELAVL1;HNRNPM;PTBP1;SNRNP40;POLR2A;HNRNPF;HNRNPD;NUP88;RNPS1;NUP54;SRSF6;RBMX
R-HSA-72165	mRNA Splicing - Minor Pathway	52	5	6.15	0.002	0.031	Reactome	1.5086	SF3B2;SNRNP40;POLR2A;SRSF6;PRPF8
GO:0006901	vesicle coating	63	6	6.1	0.00074	0.025	GO	1.6021	RAB1A;LMAN1;SEC13;TMED10;SAR1B;CTSZ
GO:0048208	COPII vesicle coating	63	6	6.1	0.00074	0.025	GO	1.6021	RAB1A;LMAN1;SEC13;TMED10;SAR1B;CTSZ
GO:0048207	vesicle targeting, rough ER to cis-Golgi	63	6	6.1	0.00074	0.025	GO	1.6021	RAB1A;LMAN1;SEC13;TMED10;SAR1B;CTSZ
GO:0045069	regulation of viral genome replication	63	6	6.1	0.00074	0.025	GO	1.6021	DDB1;BST2;ILF3;DDX3X;CNOT7;ADAR
GO:0036503	ERAD pathway	77	7	5.81	0.00036	0.018	GO	1.7447	VCP;PSMC6;OS9;HSPA5;AUP1;UBE2G2;HERPUD1
WP2328	Allograft Rejection	89	8	5.75	0.00015	0.007	Wikipathway	2.155	C1QB;C1QA;CSNK2A2;HLA-B;HLA-C;HLA-A;HLA-E;C1QC
R-HSA-68078	COPI-mediated anterograde transport	78	7	5.73	0.00039	0.0097	Reactome	2.0132	RAB1A;COPB2;ARF1;DYNC1LI2;TMED10;COPB1;NAPG
GO:0032091	negative regulation of protein binding	67	6	5.7	0.001	0.032	GO	1.4949	HSPA5;CTSZ;PRKCD;TMBIM6;B2M;PEX14
GO:0090114	COPII-coated vesicle budding	68	6	5.61	0.0011	0.032	GO	1.4949	RAB1A;LMAN1;SEC13;TMED10;SAR1B;CTSZ
R-HSA-15923	Transport of Mature mRNA derived from an Intron-C	68	6	5.61	0.0011	0.02	Reactome	1.699	FYTTD1;NUP54;NUP88;RNPS1;CASC3;SRSF6
GO:0060333	interferon-gamma-mediated signaling pathway	70	6	5.43	0.0013	0.035	GO	1.4559	PRKCD;HLA-B;HLA-C;HLA-A;B2M;HLA-E
R-HSA-19999	trans-Golgi Network Vesicle Budding	70	6	5.43	0.0013	0.022	Reactome	1.6576	TGOLN2;ARF1;CTSZ;M6PR;CLTA;GNS
R-HSA-42183	Clathrin derived vesicle budding	70	6	5.43	0.0013	0.022	Reactome	1.6576	TGOLN2;ARF1;CTSZ;M6PR;CLTA;GNS
R-HSA-19997	ER to Golgi Anterograde Transport	131	11	5.38	0.000017	0.00094	Reactome	3.0269	COPB2;RAB1A;LMAN1;DYNC1LI2;ARF1;SEC13;TMED10;SAR1B;COPB1;CTSZ;NAPG
GO:0019886	antigen processing and presentation of exogenous p	97	8	5.23	0.00027	0.016	GO	1.7959	CD74;DYNC1LI2;ARF1;SEC13;SAR1B;CAPZA1;CLTA;CTSS
GO:0002478	antigen processing and presentation of exogenous p	97	8	5.23	0.00027	0.016	GO	1.7959	CD74;DYNC1LI2;ARF1;SEC13;SAR1B;CAPZA1;CLTA;CTSS
GO:0050730	regulation of peptidyl-tyrosine phosphorylation	85	7	5.21	0.00066	0.025	GO	1.6021	CD74;PRNP;CNOT7;CD81;ITGB2;PRKCD;TREM2
GO:0006487	protein N-linked glycosylation	73	6	5.19	0.0016	0.04	GO	1.3979	LMAN1;VCP;RPN2;GFPT1;ST3GAL5;UBE2G2
GO:0002495	antigen processing and presentation of peptide anti	98	8	5.18	0.00029	0.016	GO	1.7959	CD74;DYNC1LI2;ARF1;SEC13;SAR1B;CAPZA1;CLTA;CTSS
R-HSA-72202	Transport of Mature Transcript to Cytoplasm	74	6	5.11	0.0017	0.028	Reactome	1.5528	FYTTD1;NUP54;NUP88;RNPS1;CASC3;SRSF6
GO:0034249	negative regulation of cellular amide metabolic proc	75	6	5.04	0.0018	0.045	GO	1.3468	PRNP;ILF3;DDX3X;CNOT7;APOE;CALR
R-HSA-21322	MHC class II antigen presentation	103	8	4.9	0.00041	0.0097	Reactome	2.0132	CD74;DYNC1LI2;ARF1;SEC13;SAR1B;CLTA;CTSS;CTSB
R-HSA-19893	Immunoregulatory interactions between a Lymphoi	157	12	4.86	0.000018	0.00094	Reactome	3.0269	ITGB1;FCGR3A;CD81;SIGLEC10;ITGB2;HLA-B;HLA-C;TREM2;HLA-A;B2M;LAIR1;HLA-E
GO:0051603	proteolysis involved in cellular protein catabolic pro	93	7	4.73	0.0011	0.032	GO	1.4949	DDB1;VCP;CTSZ;YME1L1;IDE;CTSS;CTSB
R-HSA-44620	Asparagine N-linked glycosylation	259	19	4.73	0.00000013	0.000011	Reactome	4.9586	PDIA3;COPB2;RAB1A;ARF1;SEC13;TMED10;RPN2;SAR1B;COPB1;CTSZ;GFPT1;NAPG;LMAN1;DYNC1LI2;GANAB;NAGK;OS9;ST3GAL5;CALR
GO:0045055	regulated exocytosis	148	11	4.71	0.000052	0.005	GO	2.301	TMX3;TMED10;SYNGR2;APOOL;APLP2;LAMP2;PSAP;ANXA5;QSOX1;TLN1;FERMT3
GO:0019058	viral life cycle	107	8	4.7	0.00053	0.021	GO	1.6778	RAB1A;VCP;GRK2;CD81;CTBP1;NUP88;NUP54;APOE
GO:0016070	RNA metabolic process	191	14	4.67	0.000006	0.00091	GO	3.041	EIF4A2;DDX18;DDX3X;WTAP;DIS3;CASC3;HNRNPM;PTBP1;SNRNP40;POLR2A;HNRNPF;HNRNPD;DHX29;RBMX
R-HSA-68114	COPI-dependent Golgi-to-ER retrograde traffic	81	6	4.63	0.0027	0.039	Reactome	1.4089	RAB1A;COPB2;ARF1;TMED10;COPB1;NAPG
GO:0050821	protein stabilization	152	11	4.57	0.000066	0.006	GO	2.2218	CCT6A;TBRG1;CRTAP;LAMP1;CDC37;LAMP2;PRKCD;CALR;CCT8;NAPG;CCT5
GO:0002576	platelet degranulation	124	9	4.57	0.0003	0.016	GO	1.7959	TMX3;APOOL;APLP2;LAMP2;PSAP;ANXA5;QSOX1;TLN1;FERMT3
GO:0051247	positive regulation of protein metabolic process	96	7	4.57	0.0013	0.035	GO	1.4559	DDB1;NFKBIA;RAB1A;VCP;SCAP;IDE;APOE
GO:0034504	protein localization to nucleus	96	7	4.57	0.0013	0.035	GO	1.4559	RPAIN;PDIA3;TBRG1;NUP88;PTTG1IP;CALR;TOR1AIP1
R-HSA-73857	RNA Polymerase II Transcription	124	9	4.57	0.0003	0.0083	Reactome	2.0809	FYTTD1;SUPT16H;POLR2A;TAF11;RNPS1;GTF2H1;CASC3;SRSF6;TAF2
WP2806	Human Complement System	97	7	4.52	0.0014	0.042	Wikipathway	1.377	PRNP;FCGR3A;SELPLG;ITGB2;C3AR1;CALR;GNAI2
GO:0019941	modification-dependent protein catabolic process	141	10	4.46	0.00017	0.013	GO	1.8861	DDB1;RNF146;XBP1;RNF34;PSMC6;OS9;UBA6;UBA1;UBE2G2;FBXO7
GO:0071427	mRNA-containing ribonucleoprotein complex export	100	7	4.37	0.0017	0.042	GO	1.3768	FYTTD1;NUP88;RNPS1;NUP54;CASC3;SRSF6;SRSF10
WP4217	Ebola Virus Pathway on Host	129	9	4.37	0.0004	0.016	Wikipathway	1.796	BST2;ITGB1;CDC42;HLA-B;HLA-C;CLTA;HLA-A;HLA-E;CTSB
GO:0000377	RNA splicing, via transesterification reactions with bu	236	16	4.31	0.0000036	0.00059	GO	3.2291	SF3B2;CASC3;USP39;PRPF8;ELAVL1;HNRNPM;PTBP1;SNRNP40;POLR2A;HNRNPF;HNRNPD;RNPS1;SRSF6;SRSF10;RBMX;SF1
R-HSA-94802	Transport to the Golgi and subsequent modification	162	11	4.27	0.00012	0.004	Reactome	2.3979	COPB2;RAB1A;LMAN1;DYNC1LI2;ARF1;SEC13;TMED10;SAR1B;COPB1;CTSZ;NAPG
R-HSA-98316	Class I MHC mediated antigen processing & presenta	305	20	4.2	0.00000037	0.000024	Reactome	4.6198	PDIA3;SEC13;RNF34;HSPA5;SAR1B;UBA6;NCF4;HLA-B;HLA-C;CYBA;HLA-A;UBE2G2;CTSS;HLA-E;TAPBP;SEC61A1;PSMC6;UBA1;CALR;B2M
GO:0006405	RNA export from nucleus	119	8	4.19	0.0011	0.032	GO	1.4949	FYTTD1;SSB;NUP88;RNPS1;NUP54;CASC3;SRSF6;SRSF10
GO:0051028	mRNA transport	104	7	4.19	0.0021	0.047	GO	1.3279	FYTTD1;NUP88;RNPS1;NUP54;CASC3;SRSF6;SRSF10
R-HSA-91353	Interferon Signaling	196	13	4.18	0.000037	0.0016	Reactome	2.7959	EIF4A2;STAT2;PRKCD;HLA-B;HLA-C;ADAR;HLA-A;TYK2;HLA-E;BST2;NUP88;NUP54;B2M
GO:0006406	mRNA export from nucleus	105	7	4.15	0.0023	0.049	GO	1.3098	FYTTD1;NUP88;RNPS1;NUP54;CASC3;SRSF6;SRSF10
R-HSA-11460	Platelet degranulation	105	7	4.15	0.0023	0.035	Reactome	1.4559	TMX3;HSPA5;APLP2;LAMP2;PSAP;QSOX1;TLN1
GO:0007030	Golgi organization	121	8	4.12	0.0012	0.033	GO	1.4815	CDC42;NSFL1C;RAB1A;LMAN1;TMED10;ATL3;UBXN2A;ATL2
GO:0043312	neutrophil degranulation	479	30	4.09	1.2E-09	0.00000089	GO	6.0506	GRN;VCP;DDX3X;COPB1;CTSZ;ITGB2;FGL2;GNS;CTSS;DPP7;LAMP1;LAMP2;PSAP;C3AR1;SIRPA;STOM;QSOX1;CD14;CCT8;B2M;LAIR1;CTSB;TMEM30A;PRKCD;ATP6AP2;CMTM6;CYBA;ARPC5;UNC13D;BST2
GO:0002283	neutrophil activation involved in immune response	483	30	4.05	1.5E-09	0.00000089	GO	6.0506	GRN;VCP;DDX3X;COPB1;CTSZ;ITGB2;FGL2;GNS;CTSS;DPP7;LAMP1;LAMP2;PSAP;C3AR1;SIRPA;STOM;QSOX1;CD14;CCT8;B2M;LAIR1;CTSB;TMEM30A;PRKCD;ATP6AP2;CMTM6;CYBA;ARPC5;UNC13D;BST2
GO:0002446	neutrophil mediated immunity	487	30	4.01	1.8E-09	0.00000089	GO	6.0506	GRN;VCP;DDX3X;COPB1;CTSZ;ITGB2;FGL2;GNS;CTSS;DPP7;LAMP1;LAMP2;PSAP;C3AR1;SIRPA;STOM;QSOX1;CD14;CCT8;B2M;LAIR1;CTSB;TMEM30A;PRKCD;ATP6AP2;CMTM6;CYBA;ARPC5;UNC13D;BST2
R-HSA-88566	Golgi-to-ER retrograde transport	110	7	3.94	0.0029	0.039	Reactome	1.4089	COPB2;RAB1A;DYNC1LI2;ARF1;TMED10;COPB1;NAPG
R-HSA-76005	Response to elevated platelet cytosolic Ca2+	110	7	3.94	0.0029	0.039	Reactome	1.4089	TMX3;HSPA5;APLP2;LAMP2;PSAP;QSOX1;TLN1
GO:0000398	mRNA splicing, via spliceosome	261	16	3.86	0.000013	0.0016	GO	2.7959	SF3B2;CASC3;USP39;PRPF8;ELAVL1;HNRNPM;PTBP1;SNRNP40;POLR2A;HNRNPF;HNRNPD;RNPS1;SRSF6;SRSF10;RBMX;SF1
GO:0006397	mRNA processing	283	17	3.79	0.0000089	0.0013	GO	2.8861	SF3B2;GTF2H1;CASC3;USP39;PRPF8;ELAVL1;HNRNPM;PTBP1;SNRNP40;POLR2A;HNRNPF;HNRNPD;RNPS1;SRSF6;SRSF10;RBMX;SF1
GO:0006605	protein targeting	133	8	3.72	0.0022	0.047	GO	1.3279	TOMM70;SEC61A1;SPCS3;CDC37;LAMP2;ATG4B;ZDHHC7;FBXO7
GO:0006461	protein complex assembly	189	11	3.61	0.00045	0.02	GO	1.699	RAB1A;LMAN1;SEC13;CDC123;TMED10;SAR1B;CTSZ;AKR1B1;RBMX;FERMT3;TNFRSF1A
R-HSA-16889	Toll-Like Receptors Cascades	140	8	3.52	0.003	0.039	Reactome	1.4089	NFKBIA;PPP2CB;UNC93B1;ITGB2;CNPY3;CD14;CTSS;CTSB
GO:0006417	regulation of translation	213	12	3.5	0.00034	0.018	GO	1.7447	EIF4A2;CDC123;ILF3;CNOT7;DDX3X;POLR2A;HNRNPD;EIF4H;NUP88;NUP54;CALR;ELAVL1
R-HSA-16258	HIV Life Cycle	141	8	3.49	0.0031	0.04	Reactome	1.3979	SUPT16H;CD4;POLR2A;TAF11;NUP88;NUP54;GTF2H1;TAF2
GO:0016032	viral process	220	12	3.38	0.00045	0.02	GO	1.699	PTBP1;RAB1A;ARF1;VCP;CD4;GRK2;SSB;CTBP1;NUP88;NUP54;APOE;B2M
R-HSA-16290	HIV Infection	222	12	3.35	0.00049	0.011	Reactome	1.9586	ARF1;SUPT16H;CD4;PSMC6;POLR2A;TAF11;NUP88;GTF2H1;NUP54;HLA-A;B2M;TAF2
GO:0048193	Golgi vesicle transport	271	14	3.2	0.00027	0.016	GO	1.7959	COPB2;RAB1A;ARF1;TMED10;SAR1B;COPB1;CTSZ;NAPG;SCAMP2;TAPBP;GOLGA3;LMAN1;DYNC1LI2;CAPZA1
R-HSA-68114	Intra-Golgi and retrograde Golgi-to-ER traffic	179	9	3.08	0.0039	0.049	Reactome	1.3098	TGOLN2;COPB2;RAB1A;DYNC1LI2;ARF1;TMED10;COPB1;M6PR;NAPG
R-HSA-12802	Adaptive Immune System	762	36	3.05	4.5E-08	0.000005	Reactome	5.301	ITGB1;ARF1;RNF34;SAR1B;CD81;UBA6;NCF4;ITGB2;CLTA;TREM2;CTSS;CDC42;PPP2CB;SEC61A1;FCGR3A;B2M;LAIR1;CTSB;PDIA3;CD74;SEC13;HSPA5;SIGLEC10;HLA-B;HLA-C;CYBA;HLA-A;UBE2G2;HLA-
GO:0015031	protein transport	326	16	3.04	0.00018	0.013	GO	1.8861	ATP6V1A;CD74;COPB2;RAB1A;SEC13;ATP6V0B;ATP6V0E1;TMED10;UNC93B1;VPS26A;NAPG;SCAMP2;GGA3;LAMP2;APOE;ATP6V0D1
GO:0050776	regulation of immune response	251	12	2.94	0.0014	0.036	GO	1.4437	ITGB1;FCGR3A;CD81;ITGB2;HLA-B;HLA-C;TREM2;HLA-A;B2M;LAIR1;CD99;HLA-E
R-HSA-39249	Metabolism of proteins	1074	48	2.94	1.3E-09	0.00000028	Reactome	6.5528	EIF4A2;COPB2;RAB1A;ARF1;TMED10;RPN2;SAR1B;COPB1;CTSZ;SMC3;NAPG;HERPUD1;GNAI2;PCMT1;LMAN1;SEC61A1;GANAB;GGA3;OS9;EIF4H;ST3GAL5;SSR1;NUP88;CCT8;B2M;CCT5;PDIA3;XBP1;SEC13;HSPA5;D
GO:0006886	intracellular protein transport	347	16	2.85	0.00036	0.018	GO	1.7447	RPAIN;PDIA3;CD74;COPB2;RAB1A;SEC13;TMED10;UNC93B1;VPS26A;PTTG1IP;NAPG;GGA3;CDC37;LAMP2;NUP88;CALR
R-HSA-16825	Immune System	1547	64	2.8	3.7E-11	1.4E-08	Reactome	7.8539	EIF4A2;ITGB1;ARF1;CD81;NCF4;ITGB2;CLTA;TREM2;ADAR;CTSS;SEC61A1;FCGR3A;C3AR1;B2M;CTSB;PDIA3;ATP6V0B;SEC13;ATP6V0E1;PRKCD;HLA-B;HLA-C;CYBA;HLA-A;TYK2;CNPY3;HLA-
R-HSA-59759	Post-translational protein modification	521	23	2.76	0.000039	0.0016	Reactome	2.7959	PDIA3;COPB2;RAB1A;ARF1;SEC13;TMED10;RPN2;SAR1B;COPB1;CTSZ;GFPT1;SMC3;SMC1A;NAPG;LMAN1;DYNC1LI2;GANAB;NAGK;OS9;ST3GAL5;NUP88;NUP54;CALR
R-HSA-68886	M Phase	268	12	2.74	0.0024	0.036	Reactome	1.4437	PPP2CB;PPP1CC;RAB1A;SEC13;PSMC6;ANAPC16;CSNK2A2;NUP88;NUP54;SMC3;SMC1A;ARPP19
GO:0019221	cytokine-mediated signaling pathway	633	26	2.57	0.000041	0.0043	GO	2.3665	ITGB1;CEBPA;ARF1;EBI3;ITGB2;ADAR;RPS6KA4;CDC42;BAG4;B2M;CCR1;STAT2;PRKCD;HLA-B;HLA-C;HLA-A;TYK2;HLA-E;TNFRSF1A;BST2;NFKBIA;CD4;PSMC6;HNRNPF;CAPZA1;LTBR
R-HSA-56632	Infectious disease	348	14	2.45	0.003	0.039	Reactome	1.4089	ARF1;SUPT16H;TAF11;CLTA;GTF2H1;HLA-A;CD4;PSMC6;POLR2A;NUP88;NUP54;CALR;B2M;TAF2
GO:0010467	gene expression	411	16	2.38	0.0021	0.047	GO	1.3279	FYTTD1;CEBPA;AKR1B1;CASC3;MRPS5;MRPS9;SNRNP40;POLR2A;HNRNPF;HNRNPD;NUP88;RNPS1;NUP54;DHX29;SRSF6;SRSF10
R-HSA-16824	Innate Immune System	807	28	2.15	0.00036	0.0095	Reactome	2.0223	C1QB;C1QA;UNC93B1;ITGB2;TREM2;RASAL3;CTSS;PLD3;CDC42;PPP2CB;FCGR3A;C3AR1;CD14;B2M;CTSB;PRKCD;HLA-B;HLA-C;CNPY3;ARPC5;HLA-E;NFKBIA;CD4;PSMC6;CAPZA1;POLR1D;TLN1;C1QC
R-HSA-16436	Disease	725	25	2.12	0.00082	0.017	Reactome	1.7696	ARF1;VCP;HDAC2;CTBP1;CLTA;CCNC;PPP2CB;BAG4;OS9;POLR2A;FGFR1OP2;NUP88;CD14;B2M;SUPT16H;TAF11;GTF2H1;HLA-A;NFKBIA;CD4;PSMC6;CDC37;NUP54;CALR;TAF2
R-HSA-10958	Hemostasis	552	19	2.1	0.0034	0.044	Reactome	1.3565	ITGB1;CD74;HDAC2;SELPLG;HSPA5;APLP2;PRKCD;ITGB2;GNAI2;CDC42;PPP2CB;TMX3;CAPZA1;MAFG;LAMP2;PSAP;SIRPA;QSOX1;TLN1
R-HSA-12802	Cytokine Signaling in Immune system	620	21	2.07	0.0026	0.038	Reactome	1.4202	EIF4A2;STAT2;PRKCD;EBI3;HLA-B;HLA-C;ADAR;HLA-A;TYK2;RASAL3;HLA-E;TNFRSF1A;BST2;PPP2CB;CD4;PSMC6;NUP88;NUP54;LTBR;TLN1;B2M

Supplementary Table 5_Microglia MEGENA Modules
Pathology enrichment

Positive logFC values indicate a higher enrichment with pathology

Amyloid-pathology

Co-expression modul	logFC	P.Value	adj.P.Val
c1_2	0.2862	0.17193163	0.350524
c1_4	-0.0089	0.97167591	0.971676
c1_5	0.4025	0.37583873	0.615736
c1_6	0.4504	0.04977189	0.174202
c1_7	0.0574	0.75412301	0.855844
c1_9	-0.5935	0.01037905	0.049949
c1_10	0.1586	0.45077098	0.708354
c1_11	1.2345	0.00091194	0.007022
c1_12	0.0763	0.7940627	0.886128
c1_13	-0.7658	0.14624212	0.325059
c1_15	-0.7443	0.2690202	0.493204
c1_16	0.3291	0.56365235	0.789113
c1_17	0.5396	0.18209055	0.350524
c1_18	0.3157	0.67061233	0.849967
c1_19	0.5602	0.13789563	0.321756
c1_20	-0.4173	0.68899939	0.855544
c1_21	-0.0231	0.94032824	0.952701
c1_22	0.7117	0.01122234	0.050831
c1_23	0.8038	0.04326356	0.158633
c1_24	-1.0214	0.03473101	0.133714
c1_25	0.1946	0.32095102	0.545958
c1_26	-0.4033	0.73332301	0.855544
c1_27	0.0521	0.8534151	0.931046
c1_28	-0.7079	0.00280927	0.018026
c1_29	-0.1807	0.62973347	0.822396
c1_30	-0.2539	0.52163634	0.757849
c1_31	0.0386	0.85849742	0.931046
c1_32	0.712	0.02438046	0.098805
c1_33	2.3441	0.00808842	0.044486
c1_34	2.3215	0.05585215	0.186983
c1_35	0.7214	0.11630047	0.289663
c1_36	1.5864	0.00684973	0.040571
c1_37	1.0893	0.08093223	0.23333
c1_39	0.1268	0.6733502	0.849967
c1_41	-1.2076	0.06539507	0.201417
c1_43	0.3191	0.5933357	0.806371
c1_45	0.6445	0.47385214	0.715424
c1_49	-0.0356	0.91131458	0.94826
c1_55	0.7458	0.00886303	0.045497
c1_57	0.4382	0.53609073	0.764426
c1_59	-0.4567	0.13216366	0.318019
c1_60	-0.0835	0.70371253	0.855544
c1_61	2.22	1.05E-09	8.05E-08
c1_62	0.4326	0.32204191	0.545958
c1_64	-0.045	0.88798889	0.942329
c1_65	-1.1863	0.00030499	0.002936
c1_66	-0.6879	0.02315288	0.098805
c1_67	-0.4753	0.14775389	0.325059
c1_68	-0.4701	0.18128153	0.350524
c1_71	-0.1417	0.72050787	0.855544
c1_75	-0.272	0.50224359	0.743707
c1_76	0.12	0.59692436	0.806371
c1_78	-0.2823	0.32324416	0.545958
c1_80	-0.0459	0.94025148	0.952701
c1_84	0.9416	0.0599406	0.192309
c1_86	0.7017	0.32615653	0.545958
c1_92	-0.4899	0.16406002	0.350524
c1_93	-0.4911	0.25106085	0.471505
c1_95	0.4459	0.43798114	0.702595
c1_96	-1.0804	0.0027764	0.018026
c1_97	-0.8052	0.11661777	0.289663
c1_98	1.853	0.00015178	0.002337
c1_99	-0.4459	0.47297835	0.715424
c1_101	2.3098	2.74E-06	9.74E-05
c1_102	2.5592	3.80E-06	9.74E-05
c1_104	-1.2367	0.00028977	0.002936
c1_107	-0.4539	0.17627398	0.350524
c1_109	0.0783	0.73139242	0.855544
c1_115	-0.7152	0.08181691	0.23333
c1_117	-0.0454	0.89337692	0.942329
c1_123	-0.6332	0.09310672	0.247214
c1_127	0.2557	0.75581052	0.855844
c1_129	-1.7946	0.00012563	0.002337
c1_130	-0.1855	0.63014776	0.822396
c1_133	1.9017	0.0001845	0.002368
c1_137	-1.2536	0.00038373	0.003283
c1_145	-0.7661	0.08839628	0.24309

pTau pathology

Co-expression modul	logFC	P.Value	adj.P.Val
c1_2	0.38893	0.032	0.090824
c1_4	0.05243	0.809516	0.86403
c1_5	0.55452	0.158678	0.341184
c1_6	0.55676	0.005086	0.021755
c1_7	0.08567	0.589495	0.709236
c1_9	-0.6772	0.000729	0.00569
c1_10	0.26525	0.145133	0.338645
c1_11	1.26264	8.90E-05	0.001143
c1_12	0.11667	0.644889	0.741141
c1_13	-0.5553	0.223577	0.407647
c1_15	-0.4126	0.479068	0.636004
c1_16	0.31712	0.520437	0.664656
c1_17	0.49279	0.159245	0.341184
c1_18	0.63238	0.324997	0.532442
c1_19	0.68045	0.037343	0.099153
c1_20	-0.7045	0.435139	0.619661
c1_21	0.2054	0.442615	0.619661
c1_22	0.79207	0.001115	0.007152
c1_23	0.7339	0.033027	0.090824
c1_24	-0.9515	0.023068	0.070872
c1_25	0.17612	0.299553	0.501426
c1_26	-0.8815	0.389629	0.593074
c1_27	0.22199	0.363258	0.570834
c1_28	-0.7951	0.000106	0.001163
c1_29	-0.1884	0.561543	0.6974
c1_30	-0.4233	0.217166	0.407647
c1_31	0.15484	0.409317	0.601477
c1_32	0.8105	0.003075	0.015783
c1_33	1.91678	0.01236	0.047586
c1_34	2.37326	0.023931	0.070872
c1_35	0.90438	0.022932	0.070872
c1_36	1.67806	0.000952	0.006666
c1_37	0.92906	0.085496	0.21944
c1_39	0.11239	0.665917	0.743125
c1_41	-0.8579	0.130501	0.314018
c1_43	0.29317	0.570875	0.697737
c1_45	0.94652	0.224308	0.407647
c1_49	0.22599	0.414004	0.601477
c1_55	0.83237	0.000739	0.00569
c1_57	-0.4401	0.472831	0.636004
c1_59	-0.1236	0.6378	0.741141
c1_60	-0.1204	0.526546	0.664656
c1_61	1.70182	6.55E-08	5.04E-06
c1_62	0.6121	0.10551	0.262072
c1_64	0.12271	0.657034	0.743125
c1_65	-1.224	1.68E-05	0.000258
c1_66	-0.7553	0.003973	0.017994
c1_67	-0.6786	0.016957	0.059351
c1_68	-0.4282	0.159515	0.341184
c1_71	-0.1308	0.702829	0.773112
c1_75	-0.4162	0.235648	0.412384
c1_76	0.25411	0.195673	0.38633
c1_78	-0.2114	0.392815	0.593074
c1_80	0.04683	0.929668	0.95446
c1_84	1.05435	0.014951	0.054821
c1_86	0.74635	0.227647	0.407647
c1_92	-0.0697	0.819145	0.86403
c1_93	-0.3531	0.340375	0.546018
c1_95	0.00439	0.992964	0.996924
c1_96	-0.7983	0.010664	0.043217
c1_97	-0.3016	0.497226	0.648922
c1_98	1.22306	0.003875	0.017994
c1_99	-0.3982	0.45912	0.631289
c1_101	1.84425	1.52E-05	0.000258
c1_102	1.84132	0.000122	0.001177
c1_104	-1.3203	7.83E-06	0.000254
c1_107	-0.67	0.021118	0.070698
c1_109	0.20674	0.295319	0.501426
c1_115	-0.4888	0.169473	0.348666
c1_117	-0.0671	0.819061	0.86403
c1_123	-0.1638	0.615798	0.729483
c1_127	-0.0027	0.996924	0.996924
c1_129	-1.3101	0.001221	0.007234
c1_130	-0.0613	0.854066	0.88869
c1_133	1.31132	0.002896	0.015783
c1_137	-1.3504	9.91E-06	0.000254
c1_145	-0.5315	0.172069	0.348666

Supplementary Table 5_Microglia MEGENA Modules Enrichment other datasets

Positive logFC values indicate a higher enrichment in AD compared to Control

Grubman et al 2019, doi: 10.1038/s41593-019-0539-4

Co-expressio	logFC	P.Value	adj.P.Val
c1 2	-0.005767339	0.790952536	0.89563743
c1 4	-0.064816457	0.038244412	0.070114755
c1 5	0.104387362	0.049020284	0.083879152
c1 6	-0.05208393	0.040215433	0.070377008
c1 7	-0.155915153	1.35E-14	1.74E-13
c1 9	0.245963279	8.83E-18	1.70E-16
c1 10	-0.110149651	7.70E-08	3.30E-07
c1 11	0.120821508	0.01450547	0.029392663
c1 12	-0.0861973	0.050599635	0.08469939
c1 13	0.05151979	0.579293114	0.696962027
c1 15	0.041333687	0.744958882	0.856146775
c1 16	-0.365746243	0.000316928	0.000787209
c1 17	0.107822586	0.10118333	0.155822328
c1 18	0.120005636	0.12546088	0.189421329
c1 19	0.116401155	0.006973477	0.014512372
c1 20	0.612058699	6.55E-05	0.000180245
c1 21	0.015432659	0.685402111	0.811937885
c1 22	-0.121560444	0.000821298	0.001859998
c1 23	-0.069355147	0.356404903	0.473158234
c1 24	0.482708741	3.74E-07	1.37E-06
c1 25	-0.141230137	3.19E-09	1.53E-08
c1 26	-0.642954401	3.58E-09	1.62E-08
c1 27	-0.261819569	1.58E-11	1.22E-10
c1 28	0.26828931	5.63E-17	8.68E-16
c1 29	0.150026472	0.019174711	0.036911319
c1 30	0.161672438	0.01697926	0.033523153
c1 31	-0.098534694	1.44E-05	4.62E-05
c1 32	-0.159459084	1.12E-05	3.75E-05
c1 33	0.246656348	0.08471518	0.133123854
c1 34	0.033233276	0.856191377	0.915837372
c1 35	0.050615058	0.532743976	0.661633647
c1 36	0.143383095	0.152406321	0.221420505
c1 37	0.011944635	0.928710285	0.928710285
c1 39	-0.082903787	0.072530853	0.116351577
c1 41	0.01552144	0.891205286	0.915837372
c1 43	-0.425369363	5.22E-05	0.000148979
c1 45	0.161076096	0.059967785	0.098245094
c1 49	0.014964528	0.701160527	0.818020615
c1 55	-0.139283005	0.000214913	0.000570631
c1 57	0.121297994	0.368210079	0.480545357
c1 59	-0.550935346	4.60E-34	3.54E-32
c1 60	-0.004203644	0.891340507	0.915837372
c1 61	0.010808106	0.892049388	0.915837372
c1 62	-0.444795653	4.04E-12	3.45E-11
c1 64	-0.116429165	0.023210496	0.043590444
c1 65	0.166239759	0.003455266	0.007390429
c1 66	0.316727514	3.21E-10	2.06E-09
c1 67	0.350195974	1.64E-12	1.58E-11
c1 68	0.198747477	0.001674059	0.003682931
c1 71	0.101431872	0.145222163	0.215040511
c1 75	0.14171791	0.040201309	0.070377008
c1 76	-0.132192804	1.02E-07	4.13E-07
c1 78	0.006057765	0.87134735	0.915837372
c1 80	0.125545415	0.277040396	0.394669176
c1 84	0.09084341	0.309686479	0.425818908
c1 86	0.084981603	0.553380824	0.676354341
c1 92	-0.675703929	2.83E-33	1.09E-31
c1 93	-0.059911658	0.3332668	0.45020252
c1 95	0.676572803	1.50E-09	8.23E-09
c1 96	-0.400344022	6.04E-11	4.23E-10
c1 97	-0.270740811	0.000789999	0.001843331
c1 98	0.44527301	1.62E-09	8.32E-09
c1 99	-0.023409304	0.845324545	0.915837372
c1 101	0.087537347	0.419536889	0.529841828
c1 102	-0.106984099	0.419744825	0.529841828
c1 104	0.224948545	0.000281407	0.000722279
c1 107	0.361471296	7.94E-13	8.73E-12
c1 109	-0.104048887	4.66E-05	0.000137893
c1 115	0.008021808	0.910316447	0.922294295
c1 117	-0.053558997	0.281906554	0.394669176
c1 123	-0.709839841	5.67E-30	1.46E-28
c1 127	0.54498186	3.00E-05	9.25E-05
c1 129	-0.405534311	2.61E-07	1.01E-06
c1 130	-0.314652796	0.000692732	0.001666887
c1 133	0.494822105	1.00E-09	5.95E-09
c1 137	0.29034071	9.87E-06	3.45E-05
c1 145	0.018718522	0.804568686	0.897852011

Mathys et al 2019, doi: 10.1038/s41586-019-1195-2

Co-expressio	logFC	P.Value	adj.P.Val
c1 2	0.034646734	0.000185429	0.000751477
c1 4	-0.070372111	2.16E-06	1.11E-05
c1 5	0.128391242	1.70E-09	2.62E-08
c1 6	-0.01210198	0.304486437	0.450874148
c1 7	0.004645706	0.698390314	0.853588162
c1 9	-0.070620646	4.70E-08	3.62E-07
c1 10	0.008565222	0.418849043	0.56581362
c1 11	-0.022629207	0.300062582	0.450874148
c1 12	0.149158121	3.40E-08	3.46E-07
c1 13	-0.133169994	0.000264633	0.00097032
c1 15	-0.043944892	0.315930226	0.45899297
c1 16	-0.276923421	1.58E-12	1.22E-10
c1 17	-0.176174113	3.95E-08	3.46E-07
c1 18	0.160628462	2.42E-06	1.16E-05
c1 19	0.114056681	8.96E-10	1.85E-08
c1 20	0.145069845	0.016705924	0.045941292
c1 21	-0.042348602	0.01767002	0.04691695
c1 22	-0.00472834	0.771238734	0.899778523
c1 23	0.035581088	0.243381637	0.398731618
c1 24	-0.127409919	0.00144378	0.005053229
c1 25	0.025762297	0.051948787	0.118157051
c1 26	-0.33965159	9.61E-10	1.85E-08
c1 27	0.01056291	0.592302227	0.735601153
c1 28	-0.085924864	2.32E-09	2.98E-08
c1 29	0.000540952	0.983257889	0.983257889
c1 30	-0.050793137	0.052173243	0.118157051
c1 31	0.00620163	0.567176795	0.715944479
c1 32	0.023561881	0.220816357	0.377841321
c1 33	0.036752242	0.51905938	0.689096074
c1 34	0.151833243	0.011374381	0.032438048
c1 35	-0.019438734	0.542867257	0.696679647
c1 36	-0.045447175	0.264057892	0.414358
c1 37	-0.088444625	0.06499161	0.139009832
c1 39	0.138107866	1.20E-06	7.13E-06
c1 41	-0.195839086	1.73E-05	7.39E-05
c1 43	-0.282382685	6.04E-12	2.33E-10
c1 45	0.192592266	1.32E-06	7.27E-06
c1 49	-0.042673526	0.020297025	0.052095697
c1 55	-0.000515284	0.975286818	0.983257889
c1 57	0.082596939	0.182042443	0.32598298
c1 59	0.095829638	0.000235443	0.000906457
c1 60	-0.0031721	0.837837064	0.92162077
c1 61	0.030836698	0.269063636	0.414358
c1 62	0.061323309	0.111683058	0.226305144
c1 64	-0.0198519	0.357513902	0.499123761
c1 65	-0.042842429	0.067984807	0.141481896
c1 66	-0.064543183	0.002328762	0.007471445
c1 67	-0.118576779	4.04E-08	3.46E-07
c1 68	-0.129310581	3.52E-07	2.26E-06
c1 71	-0.006517818	0.820308469	0.92162077
c1 75	-0.072887107	0.007818197	0.023153892
c1 76	0.001638724	0.887554305	0.945401163
c1 78	-0.00353528	0.834759031	0.92162077
c1 80	-0.005846716	0.896289414	0.945401163
c1 84	0.010217062	0.784047756	0.901069809
c1 86	-0.062255065	0.230941519	0.386576021
c1 92	0.09092184	0.007355517	0.022654994
c1 93	0.163153721	3.72E-06	1.69E-05
c1 95	0.040804025	0.332372109	0.473938007
c1 96	-0.035347246	0.25743752	0.412972688
c1 97	0.054778338	0.193656871	0.338899524
c1 98	0.009187413	0.763603958	0.899778523
c1 99	0.044818021	0.362999099	0.499123761
c1 101	0.0135618	0.733617691	0.882633785
c1 102	0.102127748	0.041577872	0.100046754
c1 104	-0.049381803	0.057614198	0.126751236
c1 107	-0.12036072	6.30E-08	4.41E-07
c1 109	-0.00199335	0.86252598	0.935415499
c1 115	0.037783622	0.163808074	0.300314802
c1 117	-0.046622284	0.034119227	0.084747756
c1 123	0.120666658	0.00158214	0.005296729
c1 127	-0.002165025	0.9672635	0.983257889
c1 129	-0.02485596	0.528231664	0.689387087
c1 130	-0.057856063	0.138302255	0.259738382
c1 133	0.045142639	0.13044124	0.251099387
c1 137	-0.042051351	0.129393603	0.251099387
c1 145	0.001280324	0.96559324	0.983257889

Zhou et al 2020, doi: 10.1038/s41591-019-0695-9

Co-expressio	logFC	P.Value	adj.P.Val
c1 2	-0.006566331	0.444712429	0.450563909
c1 4	-0.044988423	6.33E-05	7.86E-05
c1 5	-0.073994888	3.94E-06	5.23E-06
c1 6	0.127993267	1.31E-32	4.04E-32
c1 7	0.126246789	2.95E-47	1.52E-46
c1 9	-0.251048286	1.53E-102	2.95E-101
c1 10	0.179513971	7.75E-74	6.63E-73
c1 11	0.156941605	8.90344E-19	2.07747E-18
c1 12	0.140085123	1.24E-18	2.73E-18
c1 13	-0.164385815	1.42E-10	2.37E-10
c1 15	-0.186013484	1.33E-07	1.89E-07
c1 16	-0.230360676	1.99E-21	5.27E-21
c1 17	0.060721063	0.003953731	0.004756832
c1 18	-0.204495839	7.86E-18	1.64E-17
c1 19	0.062268768	6.32E-06	8.11E-06
c1 20	-0.12568446	0.006650915	0.007759401
c1 21	0.079026606	9.44E-08	1.40E-07
c1 22	0.168721592	2.63E-38	9.64E-38
c1 23	0.154292099	1.88E-18	4.02E-18
c1 24	-0.363700586	3.12E-35	1.09E-34
c1 25	0.15654579	7.31E-58	4.33E-57
c1 26	-0.518252586	4.32E-19	1.04E-18
c1 27	0.297614661	4.31E-58	2.76E-57
c1 28	-0.283213983	3.57E-111	9.17E-110
c1 29	-0.098284928	2.80E-06	3.79E-06
c1 30	-0.207975984	1.05E-20	2.69E-20
c1 31	0.148682203	6.62E-50	3.64E-49
c1 32	0.360983289	9.58E-95	1.48E-93
c1 33	0.233937111	1.01E-05	1.28E-05
c1 34	-0.053473199	0.292606112	0.304468522
c1 35	0.135123023	1.06E-06	1.48E-06
c1 36	0.247202529	1.95E-14	3.57E-14
c1 37	0.119912092	0.011639016	0.012988467
c1 39	0.130977956	3.16E-15	5.93E-15
c1 41	-0.256092045	2.95E-16	5.67E-16
c1 43	-0.253942891	8.29E-23	2.36E-22
c1 45	-0.223111368	5.94E-17	1.20E-16
c1 49	0.083860359	5.72E-08	8.81E-08
c1 55	0.175719758	2.15E-39	8.29E-39
c1 57	-0.215186978	7.55E-09	1.21E-08
c1 59	0.407245131	1.01E-92	1.29E-91
c1 60	0.069221517	9.06E-09	1.42E-08
c1 61	0.040724268	0.019632122	0.021595334
c1 62	0.611050175	7.13E-62	4.99E-61
c1 64	0.2042953	1.58E-21	4.33E-21
c1 65	-0.269312409	5.36E-40	2.17E-39
c1 66	-0.207469907	5.64E-33	1.81E-32
c1 67	-0.446242346	7.13E-137	5.49E-135
c1 68	-0.193198319	1.81E-19	4.49E-19
c1 71	-0.121439778	1.23E-07	1.79E-07
c1 75	-0.18545602	1.27E-13	2.28E-13
c1 76	0.178776422	3.85E-64	2.97E-63
c1 78	-0.023697368	0.116672701	0.123065726
c1 80	0.14678083	8.04E-05	9.82E-05
c1 84	0.138430782	4.20E-06	5.48E-06
c1 86	0.111202045	0.032194457	0.034915115
c1 92	0.480117527	1.22E-86	1.18E-85
c1 93	0.353060834	8.72E-24	2.58E-23
c1 95	-0.186236705	2.07E-06	2.85E-06
c1 96	0.310020275	5.23E-33	1.75E-32
c1 97	0.276158661	1.48E-12	2.59E-12
c1 98	-0.156767073	1.80E-11	3.08E-11
c1 99	0.09126166	0.037971605	0.040608522
c1 101	0.005833176	0.792017321	0.792017321
c1 102	0.187645714	6.41E-08	9.68E-08
c1 104	-0.305917278	7.39E-43	3.16E-42
c1 107	-0.455183627	1.24E-133	4.78E-132
c1 109	0.148430927	2.45E-45	1.18E-44
c1 115	-0.01952669	0.377834544	0.387910132
c1 117	-0.059792988	0.004680369	0.005544437
c1 123	0.522014052	1.20E-87	1.32E-86
c1 127	-0.118454006	0.009124926	0.010486855
c1 129	0.247848864	1.40E-16	2.77E-16
c1 130	0.379589453	1.23E-18	2.73E-18
c1 133	-0.148593179	5.62E-09	9.20E-09
c1 137	-0.330954778	3.89E-45	1.76E-44
c1 145	-0.065732675	0.009471578	0.010725169

Gerrits et al 2021, doi: 10.1007/s00401-021-02263-w

Co-expressio	logFC	P.Value	adj.P.Val
c1 2	0.05628638	0	0
c1 4	0.030767446	6.93E-124	2.22E-123
c1 5	0.121359031	0	0
c1 6	0.045463605	0	0
c1 7	0.008090544	7.97E-19	1.12E-18
c1 9	0.001376427	0.285108383	0.296666831
c1 10	0.010444227	5.07E-27	8.48E-27
c1 11	0.110396665	0	0
c1 12	0.036018093	2.72E-81	6.76E-81
c1 13	-0.002397504	0.451902749	0.457848838
c1 15	0.070677079	2.37E-76	5.52E-76
c1 16	0.050093958	8.92E-38	1.60E-37
c1 17	0.012567914	5.02E-08	5.86E-08
c1 18	0.180331183	0	0
c1 19	0.105848757	0	0
c1 20	-0.033494497	1.38E-07	1.58E-07
c1 21	0.034536138	1.74E-106	4.79E-106
c1 22	0.051514347	6.48E-259	2.93E-258
c1 23	0.065193203	3.63E-126	1.22E-125
c1 24	0.142493557	0	0
c1 25	-0.000878743	0.39854517	0.409173042
c1 26	-0.026136557	2.80E-08	3.32E-08
c1 27	0.038995619	5.79E-122	1.78E-121
c1 28	-0.012388297	3.98E-18	5.37E-18
c1 29	0.058783947	8.77E-140	3.38E-139
c1 30	0.048204608	1.66E-96	4.41E-96
c1 31	0.015114906	2.61E-53	5.58E-53
c1 32	-0.012723637	5.80E-14	7.44E-14
c1 33	0.137428889	6.85E-128	2.40E-127
c1 34	0.321395381	0	0
c1 35	0.108200863	0	0
c1 36	0.115838987	9.97E-234	4.27E-233
c1 37	0.021136877	1.11E-08	1.34E-08
c1 39	0.014610627	6.88E-14	8.69E-14
c1 41	-0.032163005	1.22E-16	1.62E-16
c1 43	0.041315916	2.63E-22	3.97E-22
c1 45	0.242334366	0	0
c1 49	0.037066705	9.13E-113	2.70E-112
c1 55	0.053304873	1.77E-263	8.50E-263
c1 57	0.040078362	1.53E-18	2.10E-18
c1 59	0.030481173	3.36E-53	7.00E-53
c1 60	-0.009984274	7.22E-19	1.03E-18
c1 61	-0.026449958	1.87E-26	3.07E-26
c1 62	0.054663248	4.80E-77	1.15E-76
c1 64	0.029254606	1.95E-52	3.94E-52
c1 65	0.003071168	0.145473681	0.155468255
c1 66	-0.049863142	5.60E-139	2.05E-138
c1 67	0.026535418	2.90E-33	4.96E-33
c1 68	0.000767514	0.720496618	0.720496618
c1 71	0.066555582	3.21E-148	1.30E-147
c1 75	0.053873847	5.83E-108	1.66E-107
c1 76	0.015922531	2.74E-49	5.42E-49
c1 78	0.020144575	1.53E-42	2.81E-42
c1 80	-0.059438482	1.07E-55	2.42E-55
c1 84	0.173814837	0	0
c1 86	0.039723738	1.04E-20	1.54E-20
c1 92	0.033725394	1.85E-43	3.47E-43
c1 93	0.01789485	1.13E-09	1.38E-09
c1 95	0.057836775	8.14E-48	1.57E-47
c1 96	-0.113971866	0	0
c1 97	-0.053514003	2.07E-54	4.56E-54
c1 98	0.235505956	0	0
c1 99	-0.017212794	6.17E-05	6.98E-05
c1 101	-0.034050919	3.02E-19	4.39E-19
c1 102	-0.027253898	4.11E-11	5.10E-11
c1 104	-0.003311822	0.147391982	0.155468255
c1 107	0.0286692	1.57E-36	2.74E-36
c1 109	0.020692093	1.23E-83	3.15E-83
c1 115	0.017441959	9.32E-15	1.22E-14
c1 117	0.020893328	2.28E-25	3.66E-25
c1 123	0.02567295	9.36E-23	1.47E-22
c1 127	0.013508553	0.011719535	0.012891489
c1 129	-0.161834996	0	0
c1 130	-0.025459258	2.39E-22	3.68E-22
c1 133	0.259320304	0	0
c1 137	-0.006032744	0.013272173	0.014393765
c1 145	0.006817056	0.005291388	0.005904882

Supplementary Table 5_Microglia MEGENA Modules
Module specificity scores

Module specificity scores (MSS) in microglial subclusters

A high module specificity score indicates a relatively specific enrichment of this module for a particular subcluster

Co-express	Micro1	Micro2	Micro3	PVM
c1_2	0.549	0.370	0.303	0.282
c1_4	0.548	0.369	0.303	0.282
c1_5	0.535	0.373	0.299	0.284
c1_6	0.553	0.364	0.304	0.280
c1_7	0.559	0.359	0.308	0.276
c1_9	0.557	0.361	0.303	0.277
c1_10	0.563	0.360	0.299	0.277
c1_11	0.530	0.384	0.292	0.293
c1_12	0.555	0.357	0.314	0.273
c1_13	0.544	0.362	0.307	0.272
c1_15	0.534	0.368	0.295	0.277
c1_16	0.528	0.372	0.291	0.297
c1_17	0.541	0.376	0.293	0.285
c1_18	0.513	0.374	0.291	0.281
c1_19	0.542	0.370	0.302	0.284
c1_20	0.519	0.350	0.304	0.279
c1_21	0.555	0.362	0.304	0.277
c1_22	0.549	0.365	0.307	0.279
c1_23	0.552	0.364	0.295	0.284
c1_24	0.542	0.353	0.314	0.279
c1_25	0.559	0.360	0.307	0.276
c1_26	0.457	0.321	0.378	0.262
c1_27	0.563	0.360	0.298	0.275
c1_28	0.557	0.360	0.303	0.277
c1_29	0.556	0.365	0.298	0.274
c1_30	0.549	0.361	0.302	0.281
c1_31	0.563	0.361	0.299	0.276
c1_32	0.557	0.357	0.301	0.281
c1_33	0.506	0.377	0.284	0.307
c1_34	0.460	0.413	0.282	0.299
c1_35	0.533	0.382	0.289	0.291
c1_36	0.523	0.384	0.291	0.289
c1_37	0.541	0.363	0.298	0.283
c1_39	0.555	0.354	0.316	0.275
c1_41	0.540	0.359	0.306	0.268
c1_43	0.527	0.372	0.289	0.298
c1_45	0.497	0.374	0.284	0.282
c1_49	0.555	0.361	0.303	0.277
c1_55	0.549	0.365	0.307	0.279
c1_57	0.545	0.352	0.305	0.278
c1_59	0.565	0.361	0.305	0.263
c1_60	0.557	0.357	0.309	0.279
c1_61	0.542	0.360	0.300	0.286
c1_62	0.558	0.362	0.302	0.269
c1_64	0.563	0.361	0.294	0.277
c1_65	0.560	0.350	0.310	0.272
c1_66	0.565	0.355	0.302	0.272
c1_67	0.543	0.368	0.300	0.283
c1_68	0.552	0.365	0.298	0.279
c1_71	0.555	0.367	0.295	0.275
c1_75	0.549	0.361	0.300	0.281
c1_76	0.564	0.361	0.298	0.276
c1_78	0.558	0.362	0.299	0.279
c1_80	0.557	0.359	0.294	0.274
c1_84	0.530	0.382	0.290	0.293
c1_86	0.538	0.359	0.303	0.280
c1_92	0.567	0.357	0.306	0.260
c1_93	0.561	0.357	0.304	0.267
c1_95	0.530	0.356	0.292	0.317
c1_96	0.569	0.350	0.315	0.256
c1_97	0.572	0.349	0.312	0.253
c1_98	0.519	0.371	0.296	0.312
c1_99	0.559	0.350	0.303	0.272
c1_101	0.534	0.361	0.299	0.286
c1_102	0.540	0.354	0.300	0.284
c1_104	0.560	0.348	0.311	0.272
c1_107	0.543	0.368	0.299	0.283
c1_109	0.563	0.361	0.297	0.276
c1_115	0.558	0.360	0.301	0.274
c1_117	0.555	0.363	0.298	0.281
c1_123	0.569	0.355	0.306	0.257
c1_127	0.519	0.351	0.296	0.318
c1_129	0.571	0.345	0.316	0.249
c1_130	0.563	0.354	0.311	0.264
c1_133	0.517	0.371	0.296	0.313
c1_137	0.560	0.348	0.311	0.272
c1_145	0.557	0.358	0.301	0.275

Supplementary Table 6_Astrocyte DGE
Contents

Sheet name	Description
Astrocytes_pTau	DGE analysis in Total Astrocytes using MAST and p_tau as contrast
Astrocytes_beta_amyloid	DGE analysis in Total Astrocytes using MAST and beta_amyloid as contrast

ci.hi/lo: higher and lower confidence intervals of the logFC value, respectively

Supplementary Table 6_Astrocyte DGE
Astrocytes pTau

DGE analysis in Total Astrocytes using MAST and p_tau as contrast

ensembl_gene_id	pval	logFC	ci.hi	ci.lo	gene	padj	model	variance	variance_rank
ENSG00000109846	0.000	2.113	2.990	1.236	CRYAB	0.000	~p_tau+(1|manifest)+cngeneson+pc_mito+brain_region+Sex	16.907	79.000
ENSG00000106211	0.000	1.640	2.465	0.816	HSPB1	0.000	~p_tau+(1|manifest)+cngeneson+pc_mito+brain_region+Sex	18.250	65.000
ENSG00000155368	0.000	1.300	1.815	0.784	DBI	0.000	~p_tau+(1|manifest)+cngeneson+pc_mito+brain_region+Sex	4.177	861.000
ENSG00000080824	0.000	1.250	1.902	0.599	HSP90AA1	0.001	~p_tau+(1|manifest)+cngeneson+pc_mito+brain_region+Sex	16.142	84.000
ENSG00000169715	0.000	1.249	1.855	0.643	MT1E	0.000	~p_tau+(1|manifest)+cngeneson+pc_mito+brain_region+Sex	15.131	95.000
ENSG00000124172	0.000	1.223	1.768	0.677	ATP5F1E	0.000	~p_tau+(1|manifest)+cngeneson+pc_mito+brain_region+Sex	4.269	837.000
ENSG00000088986	0.000	1.186	1.845	0.527	DYNLL1	0.000	~p_tau+(1|manifest)+cngeneson+pc_mito+brain_region+Sex	17.981	68.000
ENSG00000110958	0.002	1.071	1.851	0.290	PTGES3	0.008	~p_tau+(1|manifest)+cngeneson+pc_mito+brain_region+Sex	23.169	36.000
ENSG00000125148	0.000	1.044	1.485	0.603	MT2A	0.000	~p_tau+(1|manifest)+cngeneson+pc_mito+brain_region+Sex	13.804	127.000
ENSG00000125144	0.000	1.033	1.653	0.414	MT1G	0.000	~p_tau+(1|manifest)+cngeneson+pc_mito+brain_region+Sex	6.539	480.000
ENSG00000178980	0.000	1.024	1.432	0.616	SELENOW	0.000	~p_tau+(1|manifest)+cngeneson+pc_mito+brain_region+Sex	3.193	1179.000
ENSG00000120694	0.007	1.017	1.944	0.089	HSPH1	0.024	~p_tau+(1|manifest)+cngeneson+pc_mito+brain_region+Sex	32.169	18.000
ENSG00000006652	0.000	0.912	1.392	0.431	IFRD1	0.000	~p_tau+(1|manifest)+cngeneson+pc_mito+brain_region+Sex	2.039	1813.000
ENSG00000161011	0.000	0.906	1.284	0.529	SQSTM1	0.000	~p_tau+(1|manifest)+cngeneson+pc_mito+brain_region+Sex	0.957	2684.000
ENSG00000096384	0.000	0.899	1.437	0.361	HSP90AB1	0.000	~p_tau+(1|manifest)+cngeneson+pc_mito+brain_region+Sex	4.756	736.000
ENSG00000150991	0.000	0.893	1.399	0.387	UBC	0.001	~p_tau+(1|manifest)+cngeneson+pc_mito+brain_region+Sex	4.153	871.000
ENSG00000087460	0.000	0.889	1.183	0.596	GNAS	0.000	~p_tau+(1|manifest)+cngeneson+pc_mito+brain_region+Sex	1.984	1854.000
ENSG00000102362	0.000	0.874	1.486	0.261	SYTL4	0.002	~p_tau+(1|manifest)+cngeneson+pc_mito+brain_region+Sex	7.557	386.000
ENSG00000100364	0.000	0.863	1.176	0.550	KIAA0930	0.000	~p_tau+(1|manifest)+cngeneson+pc_mito+brain_region+Sex	15.984	86.000
ENSG00000087086	0.000	0.859	1.269	0.450	FTL	0.000	~p_tau+(1|manifest)+cngeneson+pc_mito+brain_region+Sex	5.723	583.000
ENSG00000132002	0.002	0.851	1.519	0.184	DNAJB1	0.009	~p_tau+(1|manifest)+cngeneson+pc_mito+brain_region+Sex	15.836	87.000
ENSG00000163931	0.000	0.833	1.146	0.520	TKT	0.000	~p_tau+(1|manifest)+cngeneson+pc_mito+brain_region+Sex	1.798	1990.000
ENSG00000133112	0.000	0.824	1.207	0.441	TPT1	0.000	~p_tau+(1|manifest)+cngeneson+pc_mito+brain_region+Sex	4.050	897.000
ENSG00000196739	0.000	0.817	1.129	0.505	COL27A1	0.000	~p_tau+(1|manifest)+cngeneson+pc_mito+brain_region+Sex	2.621	1445.000
ENSG00000131748	0.000	0.809	1.146	0.471	STARD3	0.000	~p_tau+(1|manifest)+cngeneson+pc_mito+brain_region+Sex	4.402	818.000
ENSG00000158106	0.000	0.784	1.225	0.344	RHPN1	0.000	~p_tau+(1|manifest)+cngeneson+pc_mito+brain_region+Sex	4.406	814.000
ENSG00000108773	0.000	0.779	1.150	0.407	KAT2A	0.000	~p_tau+(1|manifest)+cngeneson+pc_mito+brain_region+Sex	6.711	465.000
ENSG00000140319	0.000	0.770	1.213	0.326	SRP14	0.000	~p_tau+(1|manifest)+cngeneson+pc_mito+brain_region+Sex	2.221	1690.000
ENSG00000105993	0.000	0.769	1.266	0.272	DNAJB6	0.001	~p_tau+(1|manifest)+cngeneson+pc_mito+brain_region+Sex	5.121	681.000
ENSG00000127483	0.000	0.759	1.031	0.486	HP1BP3	0.000	~p_tau+(1|manifest)+cngeneson+pc_mito+brain_region+Sex	1.022	2627.000
ENSG00000157617	0.000	0.757	1.058	0.455	C2CD2	0.000	~p_tau+(1|manifest)+cngeneson+pc_mito+brain_region+Sex	0.832	2799.000
ENSG00000158186	0.000	0.743	1.125	0.361	MRAS	0.002	~p_tau+(1|manifest)+cngeneson+pc_mito+brain_region+Sex	3.526	1052.000
ENSG00000143819	0.000	0.738	1.072	0.403	EPHX1	0.000	~p_tau+(1|manifest)+cngeneson+pc_mito+brain_region+Sex	2.985	1272.000
ENSG00000140044	0.000	0.737	1.262	0.212	JDP2	0.002	~p_tau+(1|manifest)+cngeneson+pc_mito+brain_region+Sex	8.442	327.000
ENSG00000119729	0.000	0.725	1.067	0.382	RHOQ	0.000	~p_tau+(1|manifest)+cngeneson+pc_mito+brain_region+Sex	1.042	2611.000
ENSG00000160307	0.000	0.712	1.162	0.261	S100B	0.000	~p_tau+(1|manifest)+cngeneson+pc_mito+brain_region+Sex	4.815	725.000
ENSG00000166295	0.000	0.703	1.001	0.406	ANAPC16	0.000	~p_tau+(1|manifest)+cngeneson+pc_mito+brain_region+Sex	3.192	1180.000
ENSG00000102547	0.000	0.702	0.964	0.441	CAB39L	0.000	~p_tau+(1|manifest)+cngeneson+pc_mito+brain_region+Sex	1.197	2486.000
ENSG00000127922	0.004	0.692	1.229	0.154	SEM1	0.015	~p_tau+(1|manifest)+cngeneson+pc_mito+brain_region+Sex	7.667	377.000
ENSG00000163531	0.000	0.682	1.037	0.327	NFASC	0.000	~p_tau+(1|manifest)+cngeneson+pc_mito+brain_region+Sex	3.633	1022.000
ENSG00000205364	0.000	0.681	1.214	0.147	MT1M	0.003	~p_tau+(1|manifest)+cngeneson+pc_mito+brain_region+Sex	5.169	669.000
ENSG00000178104	0.000	0.680	0.988	0.371	PDE4DIP	0.000	~p_tau+(1|manifest)+cngeneson+pc_mito+brain_region+Sex	3.218	1168.000
ENSG00000087250	0.000	0.676	0.965	0.387	MT3	0.001	~p_tau+(1|manifest)+cngeneson+pc_mito+brain_region+Sex	14.756	104.000
ENSG00000173812	0.000	0.675	1.098	0.252	EIF1	0.000	~p_tau+(1|manifest)+cngeneson+pc_mito+brain_region+Sex	3.526	1051.000
ENSG00000152137	0.000	0.664	0.955	0.373	HSPB8	0.000	~p_tau+(1|manifest)+cngeneson+pc_mito+brain_region+Sex	2.657	1429.000
ENSG00000170579	0.001	0.663	1.049	0.278	DLGAP1	0.004	~p_tau+(1|manifest)+cngeneson+pc_mito+brain_region+Sex	9.911	265.000
ENSG00000103260	0.004	0.662	1.223	0.100	METRN	0.017	~p_tau+(1|manifest)+cngeneson+pc_mito+brain_region+Sex	9.872	267.000
ENSG00000165795	0.001	0.654	1.019	0.289	NDRG2	0.005	~p_tau+(1|manifest)+cngeneson+pc_mito+brain_region+Sex	23.452	34.000
ENSG00000197756	0.000	0.647	0.946	0.347	RPL37A	0.000	~p_tau+(1|manifest)+cngeneson+pc_mito+brain_region+Sex	3.081	1225.000
ENSG00000106665	0.021	0.643	1.101	0.186	CLIP2	0.050	~p_tau+(1|manifest)+cngeneson+pc_mito+brain_region+Sex	0.996	2653.000
ENSG00000074964	0.000	0.636	0.903	0.369	ARHGEF10L	0.000	~p_tau+(1|manifest)+cngeneson+pc_mito+brain_region+Sex	3.844	953.000
ENSG00000198792	0.002	0.635	1.076	0.195	TMEM184B	0.009	~p_tau+(1|manifest)+cngeneson+pc_mito+brain_region+Sex	7.037	438.000
ENSG00000079841	0.013	0.627	1.158	0.096	RIMS1	0.036	~p_tau+(1|manifest)+cngeneson+pc_mito+brain_region+Sex	11.773	182.000
ENSG00000100490	0.000	0.625	0.963	0.287	CDKL1	0.000	~p_tau+(1|manifest)+cngeneson+pc_mito+brain_region+Sex	1.076	2587.000
ENSG00000198668	0.000	0.617	0.892	0.341	CALM1	0.000	~p_tau+(1|manifest)+cngeneson+pc_mito+brain_region+Sex	2.995	1268.000
ENSG00000196218	0.000	0.604	1.069	0.139	RYR1	0.002	~p_tau+(1|manifest)+cngeneson+pc_mito+brain_region+Sex	7.915	360.000
ENSG00000196924	0.001	0.603	1.069	0.137	FLNA	0.004	~p_tau+(1|manifest)+cngeneson+pc_mito+brain_region+Sex	5.114	682.000
ENSG00000130304	0.000	0.601	0.901	0.302	SLC27A1	0.000	~p_tau+(1|manifest)+cngeneson+pc_mito+brain_region+Sex	2.845	1340.000
ENSG00000156467	0.000	0.599	0.998	0.200	UQCRB	0.000	~p_tau+(1|manifest)+cngeneson+pc_mito+brain_region+Sex	3.944	921.000
ENSG00000089220	0.000	0.598	0.817	0.378	PEBP1	0.000	~p_tau+(1|manifest)+cngeneson+pc_mito+brain_region+Sex	2.866	1334.000
ENSG00000175745	0.000	0.596	0.900	0.291	NR2F1	0.000	~p_tau+(1|manifest)+cngeneson+pc_mito+brain_region+Sex	4.056	896.000
ENSG00000189403	0.096	0.590	1.198	-0.018	HMGB1	0.158	~p_tau+(1|manifest)+cngeneson+pc_mito+brain_region+Sex	38.973	8.000
ENSG00000178209	0.000	0.589	0.898	0.280	PLEC	0.001	~p_tau+(1|manifest)+cngeneson+pc_mito+brain_region+Sex	9.967	263.000
ENSG00000087903	0.000	0.580	0.863	0.297	RFX2	0.001	~p_tau+(1|manifest)+cngeneson+pc_mito+brain_region+Sex	4.693	751.000
ENSG00000112335	0.000	0.580	0.953	0.207	SNX3	0.000	~p_tau+(1|manifest)+cngeneson+pc_mito+brain_region+Sex	2.612	1449.000
ENSG00000049759	0.009	0.578	1.045	0.111	NEDD4L	0.028	~p_tau+(1|manifest)+cngeneson+pc_mito+brain_region+Sex	7.112	427.000
ENSG00000182272	0.021	0.578	1.165	-0.010	B4GALNT4	0.050	~p_tau+(1|manifest)+cngeneson+pc_mito+brain_region+Sex	16.308	82.000
ENSG00000069849	0.000	0.578	0.994	0.162	ATP1B3	0.003	~p_tau+(1|manifest)+cngeneson+pc_mito+brain_region+Sex	3.183	1184.000
ENSG00000148411	0.001	0.570	0.923	0.218	NACC2	0.004	~p_tau+(1|manifest)+cngeneson+pc_mito+brain_region+Sex	7.410	405.000
ENSG00000056736	0.000	0.570	0.898	0.243	IL17RB	0.000	~p_tau+(1|manifest)+cngeneson+pc_mito+brain_region+Sex	1.759	2013.000
ENSG00000159176	0.000	0.568	0.896	0.241	CSRP1	0.003	~p_tau+(1|manifest)+cngeneson+pc_mito+brain_region+Sex	3.724	1000.000
ENSG00000177807	0.005	0.568	1.028	0.107	KCNJ10	0.020	~p_tau+(1|manifest)+cngeneson+pc_mito+brain_region+Sex	3.787	972.000
ENSG00000165238	0.000	0.568	0.903	0.232	WNK2	0.001	~p_tau+(1|manifest)+cngeneson+pc_mito+brain_region+Sex	3.005	1262.000
ENSG00000101400	0.005	0.567	1.050	0.084	SNTA1	0.019	~p_tau+(1|manifest)+cngeneson+pc_mito+brain_region+Sex	9.646	277.000
ENSG00000116132	0.207	0.566	1.307	-0.176	PRRX1	0.282	~p_tau+(1|manifest)+cngeneson+pc_mito+brain_region+Sex	10.143	254.000
ENSG00000135916	0.010	0.565	1.036	0.095	ITM2C	0.030	~p_tau+(1|manifest)+cngeneson+pc_mito+brain_region+Sex	12.611	158.000
ENSG00000149403	0.000	0.564	0.826	0.302	GRIK4	0.000	~p_tau+(1|manifest)+cngeneson+pc_mito+brain_region+Sex	1.488	2231.000
ENSG00000148180	0.000	0.554	0.821	0.287	GSN	0.000	~p_tau+(1|manifest)+cngeneson+pc_mito+brain_region+Sex	7.007	444.000
ENSG00000196091	0.000	0.551	0.986	0.116	MYBPC1	0.003	~p_tau+(1|manifest)+cngeneson+pc_mito+brain_region+Sex	6.123	531.000
ENSG00000163703	0.000	0.549	0.824	0.274	CRELD1	0.001	~p_tau+(1|manifest)+cngeneson+pc_mito+brain_region+Sex	4.505	795.000
ENSG00000008710	0.000	0.548	0.866	0.231	PKD1	0.002	~p_tau+(1|manifest)+cngeneson+pc_mito+brain_region+Sex	6.764	462.000
ENSG00000132329	0.000	0.548	0.792	0.305	RAMP1	0.000	~p_tau+(1|manifest)+cngeneson+pc_mito+brain_region+Sex	0.953	2692.000
ENSG00000157216	0.000	0.547	0.780	0.315	SSBP3	0.000	~p_tau+(1|manifest)+cngeneson+pc_mito+brain_region+Sex	2.384	1585.000
ENSG00000164050	0.001	0.547	0.862	0.232	PLXNB1	0.004	~p_tau+(1|manifest)+cngeneson+pc_mito+brain_region+Sex	10.335	241.000
ENSG00000185950	0.005	0.546	0.927	0.165	IRS2	0.018	~p_tau+(1|manifest)+cngeneson+pc_mito+brain_region+Sex	4.056	895.000
ENSG00000052126	0.197	0.530	1.151	-0.090	PLEKHA5	0.272	~p_tau+(1|manifest)+cngeneson+pc_mito+brain_region+Sex	32.194	17.000
ENSG00000122756	0.000	0.530	0.878	0.182	CNTFR	0.001	~p_tau+(1|manifest)+cngeneson+pc_mito+brain_region+Sex	5.558	613.000
ENSG00000101752	0.008	0.527	0.965	0.089	MIB1	0.026	~p_tau+(1|manifest)+cngeneson+pc_mito+brain_region+Sex	7.677	375.000
ENSG00000158201	0.003	0.526	0.987	0.065	ABHD3	0.012	~p_tau+(1|manifest)+cngeneson+pc_mito+brain_region+Sex	5.919	552.000
ENSG00000166925	0.001	0.526	0.883	0.168	TSC22D4	0.005	~p_tau+(1|manifest)+cngeneson+pc_mito+brain_region+Sex	7.964	358.000
ENSG00000006118	0.000	0.522	0.888	0.156	TMEM132A	0.004	~p_tau+(1|manifest)+cngeneson+pc_mito+brain_region+Sex	1.949	1880.000
ENSG00000221932	0.014	0.521	1.175	-0.132	HEPN1	0.037	~p_tau+(1|manifest)+cngeneson+pc_mito+brain_region+Sex	10.935	214.000
ENSG00000115738	0.002	0.518	0.808	0.228	ID2	0.010	~p_tau+(1|manifest)+cngeneson+pc_mito+brain_region+Sex	4.194	859.000
ENSG00000117592	0.000	0.516	0.862	0.169	PRDX6	0.004	~p_tau+(1|manifest)+cngeneson+pc_mito+brain_region+Sex	4.750	737.000
ENSG00000130203	0.039	0.515	0.947	0.083	APOE	0.080	~p_tau+(1|manifest)+cngeneson+pc_mito+brain_region+Sex	20.738	47.000
ENSG00000067057	0.010	0.514	1.026	0.002	PFKP	0.030	~p_tau+(1|manifest)+cngeneson+pc_mito+brain_region+Sex	3.670	1011.000
ENSG00000275023	0.000	0.511	0.756	0.266	MLLT6	0.000	~p_tau+(1|manifest)+cngeneson+pc_mito+brain_region+Sex	4.967	706.000
ENSG00000132024	0.000	0.510	0.720	0.300	CC2D1A	0.000	~p_tau+(1|manifest)+cngeneson+pc_mito+brain_region+Sex	2.045	1810.000
ENSG00000058668	0.000	0.509	0.729	0.289	ATP2B4	0.000	~p_tau+(1|manifest)+cngeneson+pc_mito+brain_region+Sex	2.995	1269.000
ENSG00000147853	0.000	0.509	0.706	0.312	AK3	0.000	~p_tau+(1|manifest)+cngeneson+pc_mito+brain_region+Sex	2.194	1707.000
ENSG00000174628	0.000	0.508	0.712	0.305	IQCK	0.000	~p_tau+(1|manifest)+cngeneson+pc_mito+brain_region+Sex	3.351	1116.000
ENSG00000067113	0.001	0.507	0.792	0.222	PLPP1	0.007	~p_tau+(1|manifest)+cngeneson+pc_mito+brain_region+Sex	3.730	998.000
ENSG00000186350	0.000	0.504	0.823	0.184	RXRA	0.001	~p_tau+(1|manifest)+cngeneson+pc_mito+brain_region+Sex	3.667	1012.000
ENSG00000240583	0.143	0.503	1.056	-0.050	AQP1	0.213	~p_tau+(1|manifest)+cngeneson+pc_mito+brain_region+Sex	11.589	188.000
ENSG00000183864	0.000	0.503	0.872	0.134	TOB2	0.003	~p_tau+(1|manifest)+cngeneson+pc_mito+brain_region+Sex	5.605	602.000
ENSG00000079337	0.002	0.499	0.898	0.099	RAPGEF3	0.010	~p_tau+(1|manifest)+cngeneson+pc_mito+brain_region+Sex	6.676	469.000
ENSG00000117115	0.000	0.498	0.963	0.033	PADI2	0.003	~p_tau+(1|manifest)+cngeneson+pc_mito+brain_region+Sex	4.575	777.000
ENSG00000133195	0.004	0.498	0.806	0.190	SLC39A11	0.016	~p_tau+(1|manifest)+cngeneson+pc_mito+brain_region+Sex	15.754	90.000
ENSG00000168906	0.000	0.495	0.790	0.199	MAT2A	0.002	~p_tau+(1|manifest)+cngeneson+pc_mito+brain_region+Sex	6.365	502.000
ENSG00000170485	0.017	0.491	0.866	0.115	NPAS2	0.044	~p_tau+(1|manifest)+cngeneson+pc_mito+brain_region+Sex	1.206	2477.000
ENSG00000138326	0.000	0.491	0.801	0.180	RPS24	0.000	~p_tau+(1|manifest)+cngeneson+pc_mito+brain_region+Sex	4.125	881.000
ENSG00000172201	0.000	0.489	0.764	0.214	ID4	0.002	~p_tau+(1|manifest)+cngeneson+pc_mito+brain_region+Sex	5.580	609.000
ENSG00000038427	0.088	0.488	1.047	-0.070	VCAN	0.147	~p_tau+(1|manifest)+cngeneson+pc_mito+brain_region+Sex	6.996	445.000
ENSG00000106689	0.000	0.485	0.788	0.182	LHX2	0.001	~p_tau+(1|manifest)+cngeneson+pc_mito+brain_region+Sex	1.744	2026.000
ENSG00000111640	0.002	0.485	0.818	0.151	GAPDH	0.010	~p_tau+(1|manifest)+cngeneson+pc_mito+brain_region+Sex	6.521	484.000
ENSG00000072778	0.004	0.483	0.878	0.089	ACADVL	0.017	~p_tau+(1|manifest)+cngeneson+pc_mito+brain_region+Sex	19.036	59.000
ENSG00000137509	0.000	0.476	0.749	0.203	PRCP	0.000	~p_tau+(1|manifest)+cngeneson+pc_mito+brain_region+Sex	1.551	2169.000
ENSG00000214655	0.000	0.475	0.725	0.225	ZSWIM8	0.001	~p_tau+(1|manifest)+cngeneson+pc_mito+brain_region+Sex	4.798	730.000
ENSG00000126858	0.000	0.475	0.724	0.226	RHOT1	0.001	~p_tau+(1|manifest)+cngeneson+pc_mito+brain_region+Sex	2.196	1705.000
ENSG00000167191	0.000	0.467	0.615	0.320	GPRC5B	0.000	~p_tau+(1|manifest)+cngeneson+pc_mito+brain_region+Sex	6.414	497.000
ENSG00000148204	0.000	0.466	0.795	0.138	CRB2	0.003	~p_tau+(1|manifest)+cngeneson+pc_mito+brain_region+Sex	2.836	1345.000
ENSG00000132692	0.005	0.466	0.957	-0.025	BCAN	0.019	~p_tau+(1|manifest)+cngeneson+pc_mito+brain_region+Sex	7.108	428.000
ENSG00000151746	0.000	0.464	0.648	0.281	BICD1	0.000	~p_tau+(1|manifest)+cngeneson+pc_mito+brain_region+Sex	6.480	493.000
ENSG00000113721	0.005	0.464	0.887	0.041	PDGFRB	0.018	~p_tau+(1|manifest)+cngeneson+pc_mito+brain_region+Sex	10.433	237.000
ENSG00000111275	0.000	0.463	0.750	0.177	ALDH2	0.001	~p_tau+(1|manifest)+cngeneson+pc_mito+brain_region+Sex	3.616	1031.000
ENSG00000079459	0.000	0.461	0.714	0.207	FDFT1	0.000	~p_tau+(1|manifest)+cngeneson+pc_mito+brain_region+Sex	0.967	2671.000
ENSG00000123933	0.000	0.460	0.782	0.138	MXD4	0.003	~p_tau+(1|manifest)+cngeneson+pc_mito+brain_region+Sex	4.234	848.000
ENSG00000120306	0.004	0.458	0.807	0.109	CYSTM1	0.016	~p_tau+(1|manifest)+cngeneson+pc_mito+brain_region+Sex	2.162	1722.000
ENSG00000168397	0.000	0.455	0.768	0.143	ATG4B	0.002	~p_tau+(1|manifest)+cngeneson+pc_mito+brain_region+Sex	4.041	900.000
ENSG00000136160	0.001	0.455	0.807	0.104	EDNRB	0.007	~p_tau+(1|manifest)+cngeneson+pc_mito+brain_region+Sex	5.453	633.000
ENSG00000142192	0.006	0.455	0.875	0.035	APP	0.020	~p_tau+(1|manifest)+cngeneson+pc_mito+brain_region+Sex	4.081	890.000
ENSG00000120693	0.000	0.448	0.839	0.058	SMAD9	0.003	~p_tau+(1|manifest)+cngeneson+pc_mito+brain_region+Sex	0.529	2997.000
ENSG00000109906	0.000	0.446	0.651	0.241	ZBTB16	0.000	~p_tau+(1|manifest)+cngeneson+pc_mito+brain_region+Sex	1.092	2575.000
ENSG00000188039	0.050	0.445	1.028	-0.137	NWD1	0.095	~p_tau+(1|manifest)+cngeneson+pc_mito+brain_region+Sex	9.096	302.000
ENSG00000085733	0.000	0.445	0.704	0.187	CTTN	0.000	~p_tau+(1|manifest)+cngeneson+pc_mito+brain_region+Sex	5.762	579.000
ENSG00000197746	0.001	0.445	0.703	0.187	PSAP	0.005	~p_tau+(1|manifest)+cngeneson+pc_mito+brain_region+Sex	3.597	1036.000
ENSG00000120896	0.000	0.444	0.671	0.217	SORBS3	0.000	~p_tau+(1|manifest)+cngeneson+pc_mito+brain_region+Sex	2.610	1451.000
ENSG00000080573	0.026	0.441	0.806	0.076	COL5A3	0.059	~p_tau+(1|manifest)+cngeneson+pc_mito+brain_region+Sex	6.356	505.000
ENSG00000168209	0.096	0.441	1.023	-0.141	DDIT4	0.158	~p_tau+(1|manifest)+cngeneson+pc_mito+brain_region+Sex	10.792	220.000
ENSG00000143850	0.123	0.441	1.041	-0.160	PLEKHA6	0.190	~p_tau+(1|manifest)+cngeneson+pc_mito+brain_region+Sex	11.223	201.000
ENSG00000138735	0.000	0.440	0.802	0.077	PDE5A	0.001	~p_tau+(1|manifest)+cngeneson+pc_mito+brain_region+Sex	2.526	1491.000
ENSG00000138193	0.065	0.440	1.117	-0.237	PLCE1	0.118	~p_tau+(1|manifest)+cngeneson+pc_mito+brain_region+Sex	10.181	250.000
ENSG00000205517	0.000	0.439	0.685	0.194	RGL3	0.000	~p_tau+(1|manifest)+cngeneson+pc_mito+brain_region+Sex	3.228	1162.000
ENSG00000124942	0.000	0.438	0.639	0.237	AHNAK	0.000	~p_tau+(1|manifest)+cngeneson+pc_mito+brain_region+Sex	2.492	1518.000
ENSG00000130287	0.015	0.437	0.894	-0.020	NCAN	0.039	~p_tau+(1|manifest)+cngeneson+pc_mito+brain_region+Sex	5.893	556.000
ENSG00000050165	0.000	0.436	0.675	0.197	DKK3	0.004	~p_tau+(1|manifest)+cngeneson+pc_mito+brain_region+Sex	7.579	385.000
ENSG00000133083	0.201	0.436	0.976	-0.105	DCLK1	0.276	~p_tau+(1|manifest)+cngeneson+pc_mito+brain_region+Sex	4.200	857.000
ENSG00000163644	0.001	0.436	0.741	0.130	PPM1K	0.005	~p_tau+(1|manifest)+cngeneson+pc_mito+brain_region+Sex	2.587	1463.000
ENSG00000111906	0.000	0.435	0.645	0.226	HDDC2	0.000	~p_tau+(1|manifest)+cngeneson+pc_mito+brain_region+Sex	0.879	2756.000
ENSG00000185432	0.000	0.435	0.666	0.204	METTL7A	0.001	~p_tau+(1|manifest)+cngeneson+pc_mito+brain_region+Sex	2.449	1546.000
ENSG00000134324	0.000	0.434	0.565	0.303	LPIN1	0.000	~p_tau+(1|manifest)+cngeneson+pc_mito+brain_region+Sex	2.517	1500.000
ENSG00000119900	0.004	0.432	0.763	0.100	OGFRL1	0.017	~p_tau+(1|manifest)+cngeneson+pc_mito+brain_region+Sex	6.820	460.000
ENSG00000165416	0.001	0.430	0.833	0.027	SUGT1	0.007	~p_tau+(1|manifest)+cngeneson+pc_mito+brain_region+Sex	3.853	949.000
ENSG00000146122	0.015	0.428	0.804	0.053	DAAM2	0.039	~p_tau+(1|manifest)+cngeneson+pc_mito+brain_region+Sex	6.538	481.000
ENSG00000154380	0.000	0.428	0.698	0.158	ENAH	0.003	~p_tau+(1|manifest)+cngeneson+pc_mito+brain_region+Sex	3.114	1212.000
ENSG00000130294	0.000	0.428	0.646	0.209	KIF1A	0.000	~p_tau+(1|manifest)+cngeneson+pc_mito+brain_region+Sex	2.035	1817.000
ENSG00000135862	0.000	0.426	0.773	0.080	LAMC1	0.001	~p_tau+(1|manifest)+cngeneson+pc_mito+brain_region+Sex	2.404	1570.000
ENSG00000166689	0.000	0.426	0.661	0.191	PLEKHA7	0.002	~p_tau+(1|manifest)+cngeneson+pc_mito+brain_region+Sex	3.655	1019.000
ENSG00000026025	0.007	0.424	0.974	-0.127	VIM	0.023	~p_tau+(1|manifest)+cngeneson+pc_mito+brain_region+Sex	10.661	227.000
ENSG00000163346	0.002	0.423	0.734	0.111	PBXIP1	0.008	~p_tau+(1|manifest)+cngeneson+pc_mito+brain_region+Sex	3.769	983.000
ENSG00000107186	0.000	0.423	0.652	0.194	MPDZ	0.001	~p_tau+(1|manifest)+cngeneson+pc_mito+brain_region+Sex	0.258	3112.000
ENSG00000133872	0.000	0.420	0.688	0.152	SARAF	0.002	~p_tau+(1|manifest)+cngeneson+pc_mito+brain_region+Sex	1.897	1916.000
ENSG00000198756	0.000	0.420	0.691	0.149	COLGALT2	0.001	~p_tau+(1|manifest)+cngeneson+pc_mito+brain_region+Sex	2.236	1680.000
ENSG00000104695	0.000	0.420	0.617	0.222	PPP2CB	0.000	~p_tau+(1|manifest)+cngeneson+pc_mito+brain_region+Sex	2.577	1467.000
ENSG00000167996	0.124	0.419	0.839	0.000	FTH1	0.191	~p_tau+(1|manifest)+cngeneson+pc_mito+brain_region+Sex	8.576	317.000
ENSG00000101439	0.040	0.418	0.752	0.084	CST3	0.081	~p_tau+(1|manifest)+cngeneson+pc_mito+brain_region+Sex	9.828	269.000
ENSG00000100711	0.008	0.415	0.839	-0.010	ZFYVE21	0.026	~p_tau+(1|manifest)+cngeneson+pc_mito+brain_region+Sex	7.527	391.000
ENSG00000074800	0.000	0.414	0.673	0.155	ENO1	0.001	~p_tau+(1|manifest)+cngeneson+pc_mito+brain_region+Sex	1.374	2337.000
ENSG00000175029	0.000	0.413	0.658	0.169	CTBP2	0.001	~p_tau+(1|manifest)+cngeneson+pc_mito+brain_region+Sex	1.490	2229.000
ENSG00000255690	0.017	0.413	0.860	-0.034	TRIL	0.043	~p_tau+(1|manifest)+cngeneson+pc_mito+brain_region+Sex	7.652	378.000
ENSG00000183723	0.000	0.411	0.608	0.215	CMTM4	0.000	~p_tau+(1|manifest)+cngeneson+pc_mito+brain_region+Sex	0.239	3116.000
ENSG00000092931	0.000	0.411	0.622	0.199	MFSD11	0.000	~p_tau+(1|manifest)+cngeneson+pc_mito+brain_region+Sex	2.052	1797.000
ENSG00000141756	0.000	0.411	0.692	0.130	FKBP10	0.002	~p_tau+(1|manifest)+cngeneson+pc_mito+brain_region+Sex	4.148	873.000
ENSG00000105855	0.000	0.410	0.614	0.207	ITGB8	0.002	~p_tau+(1|manifest)+cngeneson+pc_mito+brain_region+Sex	0.497	3020.000
ENSG00000007372	0.000	0.408	0.605	0.212	PAX6	0.000	~p_tau+(1|manifest)+cngeneson+pc_mito+brain_region+Sex	1.851	1950.000
ENSG00000134569	0.011	0.406	0.694	0.119	LRP4	0.032	~p_tau+(1|manifest)+cngeneson+pc_mito+brain_region+Sex	12.032	173.000
ENSG00000168056	0.003	0.406	0.777	0.035	LTBP3	0.012	~p_tau+(1|manifest)+cngeneson+pc_mito+brain_region+Sex	7.859	367.000
ENSG00000139793	0.019	0.406	0.721	0.090	MBNL2	0.046	~p_tau+(1|manifest)+cngeneson+pc_mito+brain_region+Sex	10.957	211.000
ENSG00000118997	0.044	0.403	0.776	0.030	DNAH7	0.087	~p_tau+(1|manifest)+cngeneson+pc_mito+brain_region+Sex	6.357	504.000
ENSG00000183255	0.000	0.403	0.650	0.156	PTTG1IP	0.000	~p_tau+(1|manifest)+cngeneson+pc_mito+brain_region+Sex	2.404	1571.000
ENSG00000176533	0.000	0.402	0.722	0.083	GNG7	0.003	~p_tau+(1|manifest)+cngeneson+pc_mito+brain_region+Sex	2.416	1566.000
ENSG00000154721	0.003	0.401	0.725	0.076	JAM2	0.013	~p_tau+(1|manifest)+cngeneson+pc_mito+brain_region+Sex	1.521	2199.000
ENSG00000148175	0.000	0.400	0.702	0.098	STOM	0.003	~p_tau+(1|manifest)+cngeneson+pc_mito+brain_region+Sex	2.396	1578.000
ENSG00000110651	0.007	0.399	0.705	0.094	CD81	0.023	~p_tau+(1|manifest)+cngeneson+pc_mito+brain_region+Sex	4.401	819.000
ENSG00000168003	0.013	0.399	0.815	-0.017	SLC3A2	0.035	~p_tau+(1|manifest)+cngeneson+pc_mito+brain_region+Sex	6.047	543.000
ENSG00000064655	0.014	0.398	0.699	0.097	EYA2	0.037	~p_tau+(1|manifest)+cngeneson+pc_mito+brain_region+Sex	8.211	347.000
ENSG00000130254	0.002	0.398	0.712	0.084	SAFB2	0.008	~p_tau+(1|manifest)+cngeneson+pc_mito+brain_region+Sex	5.352	643.000
ENSG00000169045	0.000	0.397	0.637	0.157	HNRNPH1	0.000	~p_tau+(1|manifest)+cngeneson+pc_mito+brain_region+Sex	2.286	1647.000
ENSG00000138002	0.000	0.396	0.569	0.224	IFT172	0.000	~p_tau+(1|manifest)+cngeneson+pc_mito+brain_region+Sex	1.951	1877.000
ENSG00000173599	0.000	0.395	0.574	0.217	PC	0.000	~p_tau+(1|manifest)+cngeneson+pc_mito+brain_region+Sex	0.971	2669.000
ENSG00000179604	0.000	0.395	0.645	0.145	CDC42EP4	0.002	~p_tau+(1|manifest)+cngeneson+pc_mito+brain_region+Sex	3.322	1126.000
ENSG00000129422	0.004	0.395	0.661	0.129	MTUS1	0.017	~p_tau+(1|manifest)+cngeneson+pc_mito+brain_region+Sex	2.225	1687.000
ENSG00000171877	0.003	0.395	0.651	0.138	FRMD5	0.014	~p_tau+(1|manifest)+cngeneson+pc_mito+brain_region+Sex	2.907	1315.000
ENSG00000074696	0.000	0.394	0.538	0.250	HACD3	0.000	~p_tau+(1|manifest)+cngeneson+pc_mito+brain_region+Sex	1.367	2342.000
ENSG00000178719	0.002	0.394	0.717	0.071	GRINA	0.011	~p_tau+(1|manifest)+cngeneson+pc_mito+brain_region+Sex	2.584	1466.000
ENSG00000120963	0.000	0.391	0.620	0.163	ZNF706	0.000	~p_tau+(1|manifest)+cngeneson+pc_mito+brain_region+Sex	0.395	3066.000
ENSG00000132334	0.000	0.391	0.649	0.132	PTPRE	0.004	~p_tau+(1|manifest)+cngeneson+pc_mito+brain_region+Sex	2.488	1520.000
ENSG00000003989	0.059	0.390	0.824	-0.044	SLC7A2	0.109	~p_tau+(1|manifest)+cngeneson+pc_mito+brain_region+Sex	8.448	326.000
ENSG00000113013	0.000	0.390	0.653	0.127	HSPA9	0.002	~p_tau+(1|manifest)+cngeneson+pc_mito+brain_region+Sex	0.893	2739.000
ENSG00000099864	0.002	0.389	0.707	0.071	PALM	0.009	~p_tau+(1|manifest)+cngeneson+pc_mito+brain_region+Sex	10.051	256.000
ENSG00000166165	0.068	0.388	0.736	0.041	CKB	0.122	~p_tau+(1|manifest)+cngeneson+pc_mito+brain_region+Sex	20.366	49.000
ENSG00000165802	0.029	0.388	0.837	-0.062	NSMF	0.063	~p_tau+(1|manifest)+cngeneson+pc_mito+brain_region+Sex	9.678	274.000
ENSG00000154655	0.081	0.387	0.873	-0.098	L3MBTL4	0.139	~p_tau+(1|manifest)+cngeneson+pc_mito+brain_region+Sex	5.484	625.000
ENSG00000156642	0.000	0.386	0.631	0.140	NPTN	0.001	~p_tau+(1|manifest)+cngeneson+pc_mito+brain_region+Sex	0.886	2749.000
ENSG00000164070	0.030	0.386	0.887	-0.116	HSPA4L	0.065	~p_tau+(1|manifest)+cngeneson+pc_mito+brain_region+Sex	13.580	131.000
ENSG00000165566	0.016	0.386	0.818	-0.047	AMER2	0.041	~p_tau+(1|manifest)+cngeneson+pc_mito+brain_region+Sex	4.545	787.000
ENSG00000082014	0.000	0.384	0.672	0.095	SMARCD3	0.002	~p_tau+(1|manifest)+cngeneson+pc_mito+brain_region+Sex	3.874	935.000
ENSG00000105426	0.000	0.383	0.569	0.197	PTPRS	0.000	~p_tau+(1|manifest)+cngeneson+pc_mito+brain_region+Sex	3.777	977.000
ENSG00000136002	0.000	0.382	0.529	0.234	ARHGEF4	0.000	~p_tau+(1|manifest)+cngeneson+pc_mito+brain_region+Sex	6.205	523.000
ENSG00000172530	0.000	0.382	0.658	0.105	BANP	0.002	~p_tau+(1|manifest)+cngeneson+pc_mito+brain_region+Sex	2.416	1565.000
ENSG00000120063	0.000	0.379	0.595	0.163	GNA13	0.000	~p_tau+(1|manifest)+cngeneson+pc_mito+brain_region+Sex	0.392	3067.000
ENSG00000164188	0.055	0.377	0.692	0.062	RANBP3L	0.103	~p_tau+(1|manifest)+cngeneson+pc_mito+brain_region+Sex	8.577	316.000
ENSG00000114416	0.007	0.377	0.762	-0.009	FXR1	0.022	~p_tau+(1|manifest)+cngeneson+pc_mito+brain_region+Sex	3.009	1257.000
ENSG00000103365	0.000	0.376	0.509	0.244	GGA2	0.000	~p_tau+(1|manifest)+cngeneson+pc_mito+brain_region+Sex	0.630	2949.000
ENSG00000103769	0.001	0.373	0.648	0.097	RAB11A	0.007	~p_tau+(1|manifest)+cngeneson+pc_mito+brain_region+Sex	3.428	1084.000
ENSG00000136448	0.000	0.372	0.587	0.157	NMT1	0.000	~p_tau+(1|manifest)+cngeneson+pc_mito+brain_region+Sex	3.521	1055.000
ENSG00000168309	0.000	0.372	0.560	0.184	FAM107A	0.003	~p_tau+(1|manifest)+cngeneson+pc_mito+brain_region+Sex	2.498	1515.000
ENSG00000144711	0.000	0.372	0.580	0.163	IQSEC1	0.000	~p_tau+(1|manifest)+cngeneson+pc_mito+brain_region+Sex	1.811	1980.000
ENSG00000175115	0.000	0.371	0.543	0.198	PACS1	0.001	~p_tau+(1|manifest)+cngeneson+pc_mito+brain_region+Sex	2.021	1825.000
ENSG00000110237	0.000	0.370	0.598	0.143	ARHGEF17	0.001	~p_tau+(1|manifest)+cngeneson+pc_mito+brain_region+Sex	3.873	936.000
ENSG00000171310	0.069	0.370	0.756	-0.016	CHST11	0.123	~p_tau+(1|manifest)+cngeneson+pc_mito+brain_region+Sex	1.720	2053.000
ENSG00000178951	0.000	0.370	0.581	0.158	ZBTB7A	0.001	~p_tau+(1|manifest)+cngeneson+pc_mito+brain_region+Sex	1.839	1956.000
ENSG00000075624	0.018	0.370	0.790	-0.051	ACTB	0.045	~p_tau+(1|manifest)+cngeneson+pc_mito+brain_region+Sex	7.018	442.000
ENSG00000012660	0.001	0.369	0.614	0.125	ELOVL5	0.004	~p_tau+(1|manifest)+cngeneson+pc_mito+brain_region+Sex	1.181	2505.000
ENSG00000170345	0.102	0.367	1.108	-0.373	FOS	0.165	~p_tau+(1|manifest)+cngeneson+pc_mito+brain_region+Sex	5.139	676.000
ENSG00000137713	0.000	0.366	0.615	0.118	PPP2R1B	0.000	~p_tau+(1|manifest)+cngeneson+pc_mito+brain_region+Sex	2.770	1374.000
ENSG00000005893	0.000	0.366	0.576	0.155	LAMP2	0.001	~p_tau+(1|manifest)+cngeneson+pc_mito+brain_region+Sex	1.701	2066.000
ENSG00000147162	0.000	0.364	0.616	0.113	OGT	0.001	~p_tau+(1|manifest)+cngeneson+pc_mito+brain_region+Sex	0.908	2727.000
ENSG00000114796	0.000	0.364	0.551	0.178	KLHL24	0.000	~p_tau+(1|manifest)+cngeneson+pc_mito+brain_region+Sex	2.061	1792.000
ENSG00000171206	0.002	0.363	0.666	0.061	TRIM8	0.008	~p_tau+(1|manifest)+cngeneson+pc_mito+brain_region+Sex	3.628	1023.000
ENSG00000141556	0.000	0.363	0.539	0.186	TBCD	0.000	~p_tau+(1|manifest)+cngeneson+pc_mito+brain_region+Sex	0.381	3073.000
ENSG00000152894	0.001	0.362	0.579	0.146	PTPRK	0.005	~p_tau+(1|manifest)+cngeneson+pc_mito+brain_region+Sex	0.654	2935.000
ENSG00000156299	0.008	0.361	0.682	0.041	TIAM1	0.025	~p_tau+(1|manifest)+cngeneson+pc_mito+brain_region+Sex	2.205	1698.000
ENSG00000073849	0.024	0.360	0.864	-0.143	ST6GAL1	0.055	~p_tau+(1|manifest)+cngeneson+pc_mito+brain_region+Sex	4.624	763.000
ENSG00000170266	0.000	0.360	0.513	0.206	GLB1	0.000	~p_tau+(1|manifest)+cngeneson+pc_mito+brain_region+Sex	0.419	3053.000
ENSG00000175048	0.000	0.360	0.536	0.184	ZDHHC14	0.000	~p_tau+(1|manifest)+cngeneson+pc_mito+brain_region+Sex	0.767	2856.000
ENSG00000165895	0.004	0.358	0.644	0.072	ARHGAP42	0.017	~p_tau+(1|manifest)+cngeneson+pc_mito+brain_region+Sex	1.436	2275.000
ENSG00000101236	0.069	0.357	0.843	-0.128	RNF24	0.123	~p_tau+(1|manifest)+cngeneson+pc_mito+brain_region+Sex	2.761	1381.000
ENSG00000080546	0.053	0.357	0.754	-0.039	SESN1	0.101	~p_tau+(1|manifest)+cngeneson+pc_mito+brain_region+Sex	2.120	1756.000
ENSG00000104765	0.000	0.357	0.599	0.114	BNIP3L	0.000	~p_tau+(1|manifest)+cngeneson+pc_mito+brain_region+Sex	0.989	2659.000
ENSG00000070759	0.008	0.353	0.661	0.046	TESK2	0.026	~p_tau+(1|manifest)+cngeneson+pc_mito+brain_region+Sex	2.065	1790.000
ENSG00000144677	0.031	0.353	0.641	0.065	CTDSPL	0.066	~p_tau+(1|manifest)+cngeneson+pc_mito+brain_region+Sex	7.765	371.000
ENSG00000145088	0.021	0.352	0.723	-0.019	EAF2	0.051	~p_tau+(1|manifest)+cngeneson+pc_mito+brain_region+Sex	5.891	557.000
ENSG00000116675	0.001	0.351	0.628	0.074	DNAJC6	0.006	~p_tau+(1|manifest)+cngeneson+pc_mito+brain_region+Sex	7.494	394.000
ENSG00000173542	0.000	0.350	0.581	0.119	MOB1B	0.001	~p_tau+(1|manifest)+cngeneson+pc_mito+brain_region+Sex	0.327	3097.000
ENSG00000160445	0.000	0.350	0.512	0.187	ZER1	0.000	~p_tau+(1|manifest)+cngeneson+pc_mito+brain_region+Sex	2.569	1473.000
ENSG00000126804	0.000	0.350	0.562	0.137	ZBTB1	0.001	~p_tau+(1|manifest)+cngeneson+pc_mito+brain_region+Sex	2.181	1713.000
ENSG00000174705	0.005	0.349	0.588	0.109	SH3PXD2B	0.018	~p_tau+(1|manifest)+cngeneson+pc_mito+brain_region+Sex	2.775	1371.000
ENSG00000026508	0.747	0.348	1.311	-0.614	CD44	0.784	~p_tau+(1|manifest)+cngeneson+pc_mito+brain_region+Sex	20.284	50.000
ENSG00000151929	0.201	0.346	1.056	-0.364	BAG3	0.276	~p_tau+(1|manifest)+cngeneson+pc_mito+brain_region+Sex	15.805	89.000
ENSG00000151090	0.022	0.346	0.627	0.064	THRB	0.051	~p_tau+(1|manifest)+cngeneson+pc_mito+brain_region+Sex	2.640	1435.000
ENSG00000092820	0.001	0.345	0.617	0.072	EZR	0.006	~p_tau+(1|manifest)+cngeneson+pc_mito+brain_region+Sex	1.301	2400.000
ENSG00000106624	0.006	0.344	0.912	-0.224	AEBP1	0.020	~p_tau+(1|manifest)+cngeneson+pc_mito+brain_region+Sex	3.177	1187.000
ENSG00000114126	0.000	0.343	0.517	0.169	TFDP2	0.001	~p_tau+(1|manifest)+cngeneson+pc_mito+brain_region+Sex	2.872	1328.000
ENSG00000012171	0.000	0.343	0.665	0.021	SEMA3B	0.001	~p_tau+(1|manifest)+cngeneson+pc_mito+brain_region+Sex	3.454	1072.000
ENSG00000120907	0.008	0.340	0.642	0.039	ADRA1A	0.026	~p_tau+(1|manifest)+cngeneson+pc_mito+brain_region+Sex	3.656	1018.000
ENSG00000174720	0.000	0.340	0.491	0.189	LARP7	0.000	~p_tau+(1|manifest)+cngeneson+pc_mito+brain_region+Sex	0.482	3030.000
ENSG00000175287	0.000	0.340	0.589	0.090	PHYHD1	0.000	~p_tau+(1|manifest)+cngeneson+pc_mito+brain_region+Sex	3.353	1114.000
ENSG00000196233	0.006	0.339	0.629	0.050	LCOR	0.021	~p_tau+(1|manifest)+cngeneson+pc_mito+brain_region+Sex	1.564	2162.000
ENSG00000109458	0.018	0.339	0.636	0.041	GAB1	0.044	~p_tau+(1|manifest)+cngeneson+pc_mito+brain_region+Sex	2.873	1327.000
ENSG00000151012	0.051	0.338	0.612	0.064	SLC7A11	0.097	~p_tau+(1|manifest)+cngeneson+pc_mito+brain_region+Sex	18.086	66.000
ENSG00000173992	0.001	0.337	0.608	0.067	CCS	0.005	~p_tau+(1|manifest)+cngeneson+pc_mito+brain_region+Sex	5.476	626.000
ENSG00000100664	0.000	0.337	0.553	0.120	EIF5	0.001	~p_tau+(1|manifest)+cngeneson+pc_mito+brain_region+Sex	0.495	3021.000
ENSG00000149557	0.000	0.337	0.450	0.223	FEZ1	0.000	~p_tau+(1|manifest)+cngeneson+pc_mito+brain_region+Sex	0.727	2891.000
ENSG00000196730	0.010	0.335	0.592	0.078	DAPK1	0.031	~p_tau+(1|manifest)+cngeneson+pc_mito+brain_region+Sex	2.458	1544.000
ENSG00000125755	0.001	0.334	0.601	0.068	SYMPK	0.004	~p_tau+(1|manifest)+cngeneson+pc_mito+brain_region+Sex	2.806	1355.000
ENSG00000082781	0.000	0.333	0.507	0.159	ITGB5	0.000	~p_tau+(1|manifest)+cngeneson+pc_mito+brain_region+Sex	1.119	2556.000
ENSG00000131711	0.119	0.332	0.755	-0.091	MAP1B	0.185	~p_tau+(1|manifest)+cngeneson+pc_mito+brain_region+Sex	5.124	680.000
ENSG00000120162	0.001	0.331	0.632	0.031	MOB3B	0.004	~p_tau+(1|manifest)+cngeneson+pc_mito+brain_region+Sex	1.763	2006.000
ENSG00000176165	0.000	0.330	0.573	0.088	FOXG1	0.002	~p_tau+(1|manifest)+cngeneson+pc_mito+brain_region+Sex	3.674	1009.000
ENSG00000114805	0.003	0.330	0.572	0.089	PLCH1	0.012	~p_tau+(1|manifest)+cngeneson+pc_mito+brain_region+Sex	1.004	2645.000
ENSG00000101040	0.003	0.329	0.591	0.067	ZMYND8	0.013	~p_tau+(1|manifest)+cngeneson+pc_mito+brain_region+Sex	2.353	1598.000
ENSG00000144029	0.000	0.329	0.471	0.188	MRPS5	0.000	~p_tau+(1|manifest)+cngeneson+pc_mito+brain_region+Sex	1.453	2257.000
ENSG00000130402	0.000	0.328	0.478	0.177	ACTN4	0.000	~p_tau+(1|manifest)+cngeneson+pc_mito+brain_region+Sex	0.716	2901.000
ENSG00000137409	0.020	0.327	0.671	-0.017	MTCH1	0.048	~p_tau+(1|manifest)+cngeneson+pc_mito+brain_region+Sex	4.780	735.000
ENSG00000105223	0.000	0.325	0.529	0.122	PLD3	0.001	~p_tau+(1|manifest)+cngeneson+pc_mito+brain_region+Sex	3.177	1186.000
ENSG00000256463	0.006	0.325	0.666	-0.016	SALL3	0.022	~p_tau+(1|manifest)+cngeneson+pc_mito+brain_region+Sex	4.417	810.000
ENSG00000196781	0.030	0.324	0.631	0.017	TLE1	0.065	~p_tau+(1|manifest)+cngeneson+pc_mito+brain_region+Sex	3.832	958.000
ENSG00000239521	0.004	0.324	0.663	-0.016	CASTOR3	0.016	~p_tau+(1|manifest)+cngeneson+pc_mito+brain_region+Sex	2.141	1743.000
ENSG00000162944	0.055	0.322	0.647	-0.002	RFTN2	0.103	~p_tau+(1|manifest)+cngeneson+pc_mito+brain_region+Sex	7.679	374.000
ENSG00000152661	0.013	0.322	0.531	0.113	GJA1	0.036	~p_tau+(1|manifest)+cngeneson+pc_mito+brain_region+Sex	8.285	343.000
ENSG00000148219	0.029	0.322	0.561	0.083	ASTN2	0.063	~p_tau+(1|manifest)+cngeneson+pc_mito+brain_region+Sex	2.250	1667.000
ENSG00000179242	0.002	0.321	0.520	0.123	CDH4	0.008	~p_tau+(1|manifest)+cngeneson+pc_mito+brain_region+Sex	2.245	1672.000
ENSG00000174738	0.000	0.320	0.562	0.079	NR1D2	0.001	~p_tau+(1|manifest)+cngeneson+pc_mito+brain_region+Sex	1.904	1908.000
ENSG00000120885	0.003	0.320	0.515	0.124	CLU	0.012	~p_tau+(1|manifest)+cngeneson+pc_mito+brain_region+Sex	13.446	134.000
ENSG00000249209	0.001	0.318	0.582	0.054	AP000311.1	0.005	~p_tau+(1|manifest)+cngeneson+pc_mito+brain_region+Sex	4.075	891.000
ENSG00000077782	0.000	0.318	0.557	0.079	FGFR1	0.002	~p_tau+(1|manifest)+cngeneson+pc_mito+brain_region+Sex	0.720	2897.000
ENSG00000165458	0.009	0.317	0.641	-0.006	INPPL1	0.028	~p_tau+(1|manifest)+cngeneson+pc_mito+brain_region+Sex	4.318	832.000
ENSG00000184009	0.024	0.317	0.730	-0.096	ACTG1	0.056	~p_tau+(1|manifest)+cngeneson+pc_mito+brain_region+Sex	5.223	664.000
ENSG00000170365	0.092	0.315	0.760	-0.130	SMAD1	0.153	~p_tau+(1|manifest)+cngeneson+pc_mito+brain_region+Sex	5.584	608.000
ENSG00000163637	0.057	0.315	0.690	-0.060	PRICKLE2	0.106	~p_tau+(1|manifest)+cngeneson+pc_mito+brain_region+Sex	5.693	591.000
ENSG00000177119	0.242	0.315	0.733	-0.103	ANO6	0.321	~p_tau+(1|manifest)+cngeneson+pc_mito+brain_region+Sex	11.336	194.000
ENSG00000154122	0.000	0.314	0.506	0.122	ANKH	0.001	~p_tau+(1|manifest)+cngeneson+pc_mito+brain_region+Sex	0.463	3038.000
ENSG00000069702	0.008	0.311	0.578	0.045	TGFBR3	0.024	~p_tau+(1|manifest)+cngeneson+pc_mito+brain_region+Sex	4.088	888.000
ENSG00000172458	0.001	0.311	0.533	0.089	IL17D	0.004	~p_tau+(1|manifest)+cngeneson+pc_mito+brain_region+Sex	1.870	1939.000
ENSG00000123836	0.007	0.310	0.669	-0.048	PFKFB2	0.023	~p_tau+(1|manifest)+cngeneson+pc_mito+brain_region+Sex	4.805	728.000
ENSG00000147724	0.058	0.310	0.717	-0.096	FAM135B	0.108	~p_tau+(1|manifest)+cngeneson+pc_mito+brain_region+Sex	1.215	2469.000
ENSG00000155506	0.000	0.309	0.460	0.158	LARP1	0.000	~p_tau+(1|manifest)+cngeneson+pc_mito+brain_region+Sex	1.207	2475.000
ENSG00000130787	0.258	0.308	0.857	-0.240	HIP1R	0.339	~p_tau+(1|manifest)+cngeneson+pc_mito+brain_region+Sex	18.947	60.000
ENSG00000031081	0.003	0.308	0.529	0.088	ARHGAP31	0.012	~p_tau+(1|manifest)+cngeneson+pc_mito+brain_region+Sex	1.799	1989.000
ENSG00000019995	0.000	0.308	0.421	0.196	ZRANB1	0.000	~p_tau+(1|manifest)+cngeneson+pc_mito+brain_region+Sex	1.205	2478.000
ENSG00000136156	0.003	0.308	0.546	0.069	ITM2B	0.013	~p_tau+(1|manifest)+cngeneson+pc_mito+brain_region+Sex	2.713	1399.000
ENSG00000107736	0.024	0.307	0.712	-0.098	CDH23	0.055	~p_tau+(1|manifest)+cngeneson+pc_mito+brain_region+Sex	3.746	992.000
ENSG00000058404	0.014	0.307	0.783	-0.170	CAMK2B	0.037	~p_tau+(1|manifest)+cngeneson+pc_mito+brain_region+Sex	9.151	300.000
ENSG00000104859	0.000	0.306	0.420	0.192	CLASRP	0.000	~p_tau+(1|manifest)+cngeneson+pc_mito+brain_region+Sex	1.422	2283.000
ENSG00000177570	0.002	0.306	0.604	0.008	SAMD12	0.008	~p_tau+(1|manifest)+cngeneson+pc_mito+brain_region+Sex	3.309	1134.000
ENSG00000173406	0.100	0.304	0.766	-0.157	DAB1	0.162	~p_tau+(1|manifest)+cngeneson+pc_mito+brain_region+Sex	3.650	1020.000
ENSG00000147027	0.018	0.304	0.623	-0.015	TMEM47	0.044	~p_tau+(1|manifest)+cngeneson+pc_mito+brain_region+Sex	2.545	1481.000
ENSG00000162804	0.075	0.304	0.827	-0.219	SNED1	0.131	~p_tau+(1|manifest)+cngeneson+pc_mito+brain_region+Sex	7.116	426.000
ENSG00000161509	0.023	0.301	0.652	-0.050	GRIN2C	0.053	~p_tau+(1|manifest)+cngeneson+pc_mito+brain_region+Sex	6.612	475.000
ENSG00000104852	0.153	0.301	0.729	-0.127	SNRNP70	0.224	~p_tau+(1|manifest)+cngeneson+pc_mito+brain_region+Sex	18.258	64.000
ENSG00000113719	0.000	0.300	0.418	0.181	ERGIC1	0.000	~p_tau+(1|manifest)+cngeneson+pc_mito+brain_region+Sex	0.912	2723.000
ENSG00000065882	0.000	0.300	0.523	0.076	TBC1D1	0.003	~p_tau+(1|manifest)+cngeneson+pc_mito+brain_region+Sex	2.219	1691.000
ENSG00000213918	0.000	0.299	0.427	0.172	DNASE1	0.000	~p_tau+(1|manifest)+cngeneson+pc_mito+brain_region+Sex	1.371	2339.000
ENSG00000137802	0.001	0.299	0.521	0.077	MAPKBP1	0.006	~p_tau+(1|manifest)+cngeneson+pc_mito+brain_region+Sex	1.698	2068.000
ENSG00000185650	0.015	0.297	0.509	0.084	ZFP36L1	0.039	~p_tau+(1|manifest)+cngeneson+pc_mito+brain_region+Sex	2.520	1497.000
ENSG00000160310	0.000	0.295	0.487	0.104	PRMT2	0.000	~p_tau+(1|manifest)+cngeneson+pc_mito+brain_region+Sex	1.567	2159.000
ENSG00000185787	0.000	0.294	0.414	0.174	MORF4L1	0.000	~p_tau+(1|manifest)+cngeneson+pc_mito+brain_region+Sex	1.344	2362.000
ENSG00000182095	0.000	0.293	0.451	0.136	TNRC18	0.000	~p_tau+(1|manifest)+cngeneson+pc_mito+brain_region+Sex	1.395	2313.000
ENSG00000133318	0.000	0.293	0.453	0.133	RTN3	0.001	~p_tau+(1|manifest)+cngeneson+pc_mito+brain_region+Sex	0.684	2921.000
ENSG00000169826	0.000	0.292	0.473	0.111	[illegible]	0.001	~p_tau+(1|manifest)+cngeneson+pc_mito+brain_region+Sex	2.211	1695.000
ENSG00000164970	0.001	0.292	0.529	0.054	FAM219A	0.004	~p_tau+(1|manifest)+cngeneson+pc_mito+brain_region+Sex	1.269	2433.000
ENSG00000149177	0.005	0.291	0.656	-0.074	PTPRJ	0.017	~p_tau+(1|manifest)+cngeneson+pc_mito+brain_region+Sex	1.627	2119.000
ENSG00000070214	0.001	0.291	0.505	0.076	SLC44A1	0.007	~p_tau+(1|manifest)+cngeneson+pc_mito+brain_region+Sex	2.422	1563.000
ENSG00000104218	0.000	0.290	0.423	0.157	CSPP1	0.000	~p_tau+(1|manifest)+cngeneson+pc_mito+brain_region+Sex	0.966	2672.000
ENSG00000182103	0.011	0.290	0.618	-0.039	FAM181B	0.032	~p_tau+(1|manifest)+cngeneson+pc_mito+brain_region+Sex	3.018	1253.000
ENSG00000074855	0.114	0.289	0.742	-0.163	ANO8	0.180	~p_tau+(1|manifest)+cngeneson+pc_mito+brain_region+Sex	10.412	238.000
ENSG00000170234	0.000	0.288	0.460	0.117	PWWP2A	0.000	~p_tau+(1|manifest)+cngeneson+pc_mito+brain_region+Sex	0.537	2993.000
ENSG00000099910	0.000	0.288	0.444	0.133	KLHL22	0.000	~p_tau+(1|manifest)+cngeneson+pc_mito+brain_region+Sex	0.959	2677.000
ENSG00000175928	0.002	0.288	0.547	0.030	LRRN1	0.009	~p_tau+(1|manifest)+cngeneson+pc_mito+brain_region+Sex	0.942	2696.000
ENSG00000103148	0.000	0.288	0.425	0.151	NPRL3	0.000	~p_tau+(1|manifest)+cngeneson+pc_mito+brain_region+Sex	1.696	2071.000
ENSG00000013441	0.002	0.287	0.558	0.017	CLK1	0.009	~p_tau+(1|manifest)+cngeneson+pc_mito+brain_region+Sex	5.271	655.000
ENSG00000106780	0.001	0.287	0.467	0.107	MEGF9	0.005	~p_tau+(1|manifest)+cngeneson+pc_mito+brain_region+Sex	1.350	2357.000
ENSG00000139668	0.000	0.284	0.435	0.133	WDFY2	0.003	~p_tau+(1|manifest)+cngeneson+pc_mito+brain_region+Sex	0.360	3080.000
ENSG00000054965	0.000	0.283	0.440	0.125	FAM168A	0.004	~p_tau+(1|manifest)+cngeneson+pc_mito+brain_region+Sex	2.948	1291.000
ENSG00000107864	0.034	0.282	0.521	0.043	CPEB3	0.072	~p_tau+(1|manifest)+cngeneson+pc_mito+brain_region+Sex	3.421	1085.000
ENSG00000006704	0.000	0.282	0.503	0.061	GTF2IRD1	0.001	~p_tau+(1|manifest)+cngeneson+pc_mito+brain_region+Sex	3.025	1251.000
ENSG00000122884	0.150	0.282	0.649	-0.085	P4HA1	0.220	~p_tau+(1|manifest)+cngeneson+pc_mito+brain_region+Sex	3.371	1108.000
ENSG00000137074	0.000	0.281	0.456	0.105	APTX	0.001	~p_tau+(1|manifest)+cngeneson+pc_mito+brain_region+Sex	0.934	2703.000
ENSG00000185070	0.233	0.278	0.710	-0.154	FLRT2	0.311	~p_tau+(1|manifest)+cngeneson+pc_mito+brain_region+Sex	16.992	77.000
ENSG00000182087	0.075	0.278	0.737	-0.181	TMEM259	0.131	~p_tau+(1|manifest)+cngeneson+pc_mito+brain_region+Sex	9.280	296.000
ENSG00000285082	0.012	0.278	0.641	-0.085	AL160272.2	0.034	~p_tau+(1|manifest)+cngeneson+pc_mito+brain_region+Sex	5.212	667.000
ENSG00000148468	0.046	0.276	0.501	0.051	FAM171A1	0.090	~p_tau+(1|manifest)+cngeneson+pc_mito+brain_region+Sex	7.543	389.000
ENSG00000136193	0.001	0.275	0.490	0.060	SCRN1	0.006	~p_tau+(1|manifest)+cngeneson+pc_mito+brain_region+Sex	0.659	2932.000
ENSG00000182175	0.137	0.274	0.702	-0.154	RGMA	0.206	~p_tau+(1|manifest)+cngeneson+pc_mito+brain_region+Sex	6.631	473.000
ENSG00000131771	0.058	0.274	0.646	-0.099	PPP1R1B	0.108	~p_tau+(1|manifest)+cngeneson+pc_mito+brain_region+Sex	5.409	634.000
ENSG00000164877	0.078	0.273	0.614	-0.069	MICALL2	0.135	~p_tau+(1|manifest)+cngeneson+pc_mito+brain_region+Sex	10.973	210.000
ENSG00000144445	0.004	0.273	0.468	0.078	KANSL1L	0.017	~p_tau+(1|manifest)+cngeneson+pc_mito+brain_region+Sex	1.095	2572.000
ENSG00000111961	0.000	0.271	0.415	0.128	SASH1	0.002	~p_tau+(1|manifest)+cngeneson+pc_mito+brain_region+Sex	5.871	560.000
ENSG00000170370	0.104	0.271	0.648	-0.106	EMX2	0.168	~p_tau+(1|manifest)+cngeneson+pc_mito+brain_region+Sex	2.285	1648.000
ENSG00000100242	0.003	0.271	0.530	0.012	SUN2	0.012	~p_tau+(1|manifest)+cngeneson+pc_mito+brain_region+Sex	2.998	1267.000
ENSG00000143198	0.000	0.269	0.373	0.166	MGST3	0.000	~p_tau+(1|manifest)+cngeneson+pc_mito+brain_region+Sex	0.812	2820.000
ENSG00000137486	0.142	0.269	0.688	-0.150	ARRB1	0.211	~p_tau+(1|manifest)+cngeneson+pc_mito+brain_region+Sex	9.522	283.000
ENSG00000129521	0.313	0.267	0.899	-0.366	EGLN3	0.393	~p_tau+(1|manifest)+cngeneson+pc_mito+brain_region+Sex	14.109	120.000
ENSG00000183098	0.274	0.266	0.656	-0.123	GPC6	0.355	~p_tau+(1|manifest)+cngeneson+pc_mito+brain_region+Sex	2.510	1509.000
ENSG00000177606	0.106	0.266	0.775	-0.242	JUN	0.170	~p_tau+(1|manifest)+cngeneson+pc_mito+brain_region+Sex	3.497	1057.000
ENSG00000163453	0.361	0.266	0.643	-0.111	IGFBP7	0.439	~p_tau+(1|manifest)+cngeneson+pc_mito+brain_region+Sex	14.567	106.000
ENSG00000115904	0.003	0.266	0.459	0.072	SOS1	0.012	~p_tau+(1|manifest)+cngeneson+pc_mito+brain_region+Sex	0.313	3102.000
ENSG00000218336	0.073	0.264	0.667	-0.140	TENM3	0.129	~p_tau+(1|manifest)+cngeneson+pc_mito+brain_region+Sex	8.925	310.000
ENSG00000198053	0.015	0.262	0.469	0.055	SIRPA	0.039	~p_tau+(1|manifest)+cngeneson+pc_mito+brain_region+Sex	3.704	1003.000
ENSG00000172613	0.000	0.261	0.434	0.088	RAD9A	0.001	~p_tau+(1|manifest)+cngeneson+pc_mito+brain_region+Sex	2.182	1712.000
ENSG00000100412	0.001	0.261	0.473	0.050	ACO2	0.004	~p_tau+(1|manifest)+cngeneson+pc_mito+brain_region+Sex	2.517	1502.000
ENSG00000108231	0.085	0.261	0.542	-0.019	LGI1	0.144	~p_tau+(1|manifest)+cngeneson+pc_mito+brain_region+Sex	3.796	968.000
ENSG00000175662	0.004	0.261	0.421	0.101	TOM1L2	0.014	~p_tau+(1|manifest)+cngeneson+pc_mito+brain_region+Sex	7.106	430.000
ENSG00000172292	0.258	0.260	0.773	-0.253	CERS6	0.339	~p_tau+(1|manifest)+cngeneson+pc_mito+brain_region+Sex	1.738	2032.000
ENSG00000154856	0.103	0.260	0.681	-0.160	APCDD1	0.166	~p_tau+(1|manifest)+cngeneson+pc_mito+brain_region+Sex	6.265	512.000
ENSG00000141576	0.000	0.260	0.410	0.110	RNF157	0.000	~p_tau+(1|manifest)+cngeneson+pc_mito+brain_region+Sex	0.485	3028.000
ENSG00000083857	0.000	0.260	0.478	0.042	FAT1	0.004	~p_tau+(1|manifest)+cngeneson+pc_mito+brain_region+Sex	1.404	2307.000
ENSG00000187147	0.110	0.259	0.618	-0.100	RNF220	0.175	~p_tau+(1|manifest)+cngeneson+pc_mito+brain_region+Sex	3.943	922.000
ENSG00000123094	0.248	0.259	0.615	-0.096	RASSF8	0.326	~p_tau+(1|manifest)+cngeneson+pc_mito+brain_region+Sex	2.254	1666.000
ENSG00000141279	0.000	0.259	0.415	0.103	NPEPPS	0.000	~p_tau+(1|manifest)+cngeneson+pc_mito+brain_region+Sex	4.425	809.000
ENSG00000111907	0.013	0.259	0.424	0.094	TPD52L1	0.035	~p_tau+(1|manifest)+cngeneson+pc_mito+brain_region+Sex	0.781	2849.000
ENSG00000205531	0.000	0.259	0.459	0.058	NAP1L4	0.003	~p_tau+(1|manifest)+cngeneson+pc_mito+brain_region+Sex	1.616	2130.000
ENSG00000090060	0.020	0.258	0.632	-0.115	PAPOLA	0.049	~p_tau+(1|manifest)+cngeneson+pc_mito+brain_region+Sex	2.261	1664.000
ENSG00000081189	0.001	0.258	0.447	0.069	MEF2C	0.008	~p_tau+(1|manifest)+cngeneson+pc_mito+brain_region+Sex	1.254	2440.000
ENSG00000118816	0.000	0.258	0.374	0.141	CCNI	0.000	~p_tau+(1|manifest)+cngeneson+pc_mito+brain_region+Sex	0.883	2752.000
ENSG00000172985	0.017	0.258	0.575	-0.059	SH3RF3	0.042	~p_tau+(1|manifest)+cngeneson+pc_mito+brain_region+Sex	4.173	864.000
ENSG00000141458	0.000	0.257	0.487	0.028	NPC1	0.001	~p_tau+(1|manifest)+cngeneson+pc_mito+brain_region+Sex	3.077	1228.000
ENSG00000103197	0.004	0.256	0.524	-0.012	TSC2	0.017	~p_tau+(1|manifest)+cngeneson+pc_mito+brain_region+Sex	7.615	382.000
ENSG00000035115	0.000	0.255	0.413	0.097	SH3YL1	0.000	~p_tau+(1|manifest)+cngeneson+pc_mito+brain_region+Sex	1.036	2615.000
ENSG00000148660	0.208	0.255	0.550	-0.041	CAMK2G	0.283	~p_tau+(1|manifest)+cngeneson+pc_mito+brain_region+Sex	21.479	42.000
ENSG00000110328	0.045	0.254	0.539	-0.031	GALNT18	0.088	~p_tau+(1|manifest)+cngeneson+pc_mito+brain_region+Sex	2.146	1737.000
ENSG00000084636	0.127	0.253	0.701	-0.195	COL16A1	0.194	~p_tau+(1|manifest)+cngeneson+pc_mito+brain_region+Sex	10.536	232.000
ENSG00000171867	0.011	0.253	0.498	0.007	PRNP	0.032	~p_tau+(1|manifest)+cngeneson+pc_mito+brain_region+Sex	1.498	2220.000
ENSG00000162390	0.040	0.252	0.492	0.013	ACOT11	0.081	~p_tau+(1|manifest)+cngeneson+pc_mito+brain_region+Sex	15.072	99.000
ENSG00000197077	0.002	0.252	0.433	0.072	KIAA1671	0.011	~p_tau+(1|manifest)+cngeneson+pc_mito+brain_region+Sex	2.904	1316.000
ENSG00000119048	0.031	0.251	0.695	-0.192	UBE2B	0.066	~p_tau+(1|manifest)+cngeneson+pc_mito+brain_region+Sex	2.197	1704.000
ENSG00000105552	0.042	0.251	0.571	-0.068	BCAT2	0.084	~p_tau+(1|manifest)+cngeneson+pc_mito+brain_region+Sex	6.939	451.000
ENSG00000182348	0.040	0.251	0.479	0.024	ZNF804B	0.080	~p_tau+(1|manifest)+cngeneson+pc_mito+brain_region+Sex	6.081	539.000
ENSG00000196504	0.000	0.251	0.460	0.043	PRPF40A	0.002	~p_tau+(1|manifest)+cngeneson+pc_mito+brain_region+Sex	0.958	2680.000
ENSG00000103319	0.046	0.248	0.580	-0.084	EEF2K	0.090	~p_tau+(1|manifest)+cngeneson+pc_mito+brain_region+Sex	7.649	379.000
ENSG00000179364	0.000	0.247	0.372	0.121	PACS2	0.000	~p_tau+(1|manifest)+cngeneson+pc_mito+brain_region+Sex	2.629	1442.000
ENSG00000127946	0.002	0.246	0.440	0.053	HIP1	0.009	~p_tau+(1|manifest)+cngeneson+pc_mito+brain_region+Sex	1.394	2315.000
ENSG00000125398	0.024	0.246	0.486	0.006	SOX9	0.056	~p_tau+(1|manifest)+cngeneson+pc_mito+brain_region+Sex	3.451	1075.000
ENSG00000140526	0.030	0.246	0.523	-0.031	ABHD2	0.065	~p_tau+(1|manifest)+cngeneson+pc_mito+brain_region+Sex	0.777	2852.000
ENSG00000136802	0.015	0.244	0.487	0.001	LRRC8A	0.038	~p_tau+(1|manifest)+cngeneson+pc_mito+brain_region+Sex	2.007	1835.000
ENSG00000075142	0.002	0.244	0.486	0.003	SRI	0.009	~p_tau+(1|manifest)+cngeneson+pc_mito+brain_region+Sex	0.929	2705.000
ENSG00000092531	0.000	0.244	0.435	0.053	SNAP23	0.001	~p_tau+(1|manifest)+cngeneson+pc_mito+brain_region+Sex	0.468	3036.000
ENSG00000157985	0.002	0.243	0.415	0.071	AGAP1	0.011	~p_tau+(1|manifest)+cngeneson+pc_mito+brain_region+Sex	0.293	3107.000
ENSG00000185722	0.000	0.243	0.354	0.132	ANKFY1	0.000	~p_tau+(1|manifest)+cngeneson+pc_mito+brain_region+Sex	1.180	2508.000
ENSG00000100077	0.101	0.242	0.597	-0.113	GRK3	0.164	~p_tau+(1|manifest)+cngeneson+pc_mito+brain_region+Sex	4.405	815.000
ENSG00000139644	0.039	0.242	0.524	-0.040	TMBIM6	0.079	~p_tau+(1|manifest)+cngeneson+pc_mito+brain_region+Sex	3.751	991.000
ENSG00000150764	0.000	0.242	0.427	0.057	DIXDC1	0.000	~p_tau+(1|manifest)+cngeneson+pc_mito+brain_region+Sex	1.036	2617.000
ENSG00000073417	0.067	0.241	0.524	-0.041	PDE8A	0.120	~p_tau+(1|manifest)+cngeneson+pc_mito+brain_region+Sex	8.670	314.000
ENSG00000189079	0.000	0.241	0.370	0.112	ARID2	0.000	~p_tau+(1|manifest)+cngeneson+pc_mito+brain_region+Sex	1.560	2164.000
ENSG00000147526	0.000	0.241	0.373	0.109	TACC1	0.000	~p_tau+(1|manifest)+cngeneson+pc_mito+brain_region+Sex	1.808	1983.000
ENSG00000102786	0.000	0.241	0.416	0.066	INTS6	0.001	~p_tau+(1|manifest)+cngeneson+pc_mito+brain_region+Sex	0.828	2802.000
ENSG00000115548	0.002	0.240	0.419	0.062	KDM3A	0.009	~p_tau+(1|manifest)+cngeneson+pc_mito+brain_region+Sex	1.545	2175.000
ENSG00000102125	0.000	0.240	0.412	0.068	TAZ	0.002	~p_tau+(1|manifest)+cngeneson+pc_mito+brain_region+Sex	3.314	1131.000
ENSG00000152284	0.005	0.239	0.433	0.045	TCF7L1	0.018	~p_tau+(1|manifest)+cngeneson+pc_mito+brain_region+Sex	2.413	1567.000
ENSG00000156931	0.000	0.239	0.363	0.114	VPS8	0.000	~p_tau+(1|manifest)+cngeneson+pc_mito+brain_region+Sex	1.555	2167.000
ENSG00000187764	0.003	0.238	0.529	-0.052	SEMA4D	0.014	~p_tau+(1|manifest)+cngeneson+pc_mito+brain_region+Sex	3.567	1045.000
ENSG00000104450	0.001	0.238	0.477	-0.002	SPAG1	0.008	~p_tau+(1|manifest)+cngeneson+pc_mito+brain_region+Sex	1.040	2613.000
ENSG00000221968	0.068	0.237	0.625	-0.151	FADS3	0.122	~p_tau+(1|manifest)+cngeneson+pc_mito+brain_region+Sex	6.159	526.000
ENSG00000149091	0.136	0.237	0.620	-0.146	DGKZ	0.205	~p_tau+(1|manifest)+cngeneson+pc_mito+brain_region+Sex	4.587	773.000
ENSG00000124749	0.356	0.237	0.865	-0.392	COL21A1	0.435	~p_tau+(1|manifest)+cngeneson+pc_mito+brain_region+Sex	5.476	627.000
ENSG00000053254	0.028	0.236	0.452	0.020	FOXN3	0.063	~p_tau+(1|manifest)+cngeneson+pc_mito+brain_region+Sex	3.547	1048.000
ENSG00000140350	0.000	0.236	0.352	0.120	ANP32A	0.000	~p_tau+(1|manifest)+cngeneson+pc_mito+brain_region+Sex	0.505	3015.000
ENSG00000135424	0.062	0.235	0.572	-0.102	ITGA7	0.113	~p_tau+(1|manifest)+cngeneson+pc_mito+brain_region+Sex	10.707	225.000
ENSG00000160460	0.001	0.234	0.449	0.020	SPTBN4	0.007	~p_tau+(1|manifest)+cngeneson+pc_mito+brain_region+Sex	3.490	1060.000
ENSG00000126214	0.000	0.234	0.364	0.104	KLC1	0.001	~p_tau+(1|manifest)+cngeneson+pc_mito+brain_region+Sex	2.287	1646.000
ENSG00000102935	0.108	0.234	0.597	-0.130	ZNF423	0.172	~p_tau+(1|manifest)+cngeneson+pc_mito+brain_region+Sex	6.932	453.000
ENSG00000197111	0.004	0.233	0.410	0.057	PCBP2	0.017	~p_tau+(1|manifest)+cngeneson+pc_mito+brain_region+Sex	2.160	1725.000
ENSG00000145819	0.019	0.233	0.392	0.073	ARHGAP26	0.046	~p_tau+(1|manifest)+cngeneson+pc_mito+brain_region+Sex	7.515	392.000
ENSG00000139687	0.000	0.232	0.412	0.052	RB1	0.002	~p_tau+(1|manifest)+cngeneson+pc_mito+brain_region+Sex	0.703	2912.000
ENSG00000164916	0.000	0.232	0.429	0.035	FOXK1	0.002	~p_tau+(1|manifest)+cngeneson+pc_mito+brain_region+Sex	1.728	2045.000
ENSG00000133794	0.019	0.231	0.620	-0.157	ARNTL	0.046	~p_tau+(1|manifest)+cngeneson+pc_mito+brain_region+Sex	0.574	2973.000
ENSG00000101134	0.026	0.231	0.453	0.009	DOK5	0.058	~p_tau+(1|manifest)+cngeneson+pc_mito+brain_region+Sex	1.696	2072.000
ENSG00000075785	0.000	0.231	0.393	0.068	RAB7A	0.002	~p_tau+(1|manifest)+cngeneson+pc_mito+brain_region+Sex	2.807	1354.000
ENSG00000198700	0.000	0.231	0.394	0.068	IPO9	0.001	~p_tau+(1|manifest)+cngeneson+pc_mito+brain_region+Sex	1.678	2081.000
ENSG00000172380	0.000	0.231	0.422	0.039	GNG12	0.004	~p_tau+(1|manifest)+cngeneson+pc_mito+brain_region+Sex	0.390	3068.000
ENSG00000004799	0.190	0.231	0.602	-0.141	PDK4	0.265	~p_tau+(1|manifest)+cngeneson+pc_mito+brain_region+Sex	5.133	678.000
ENSG00000160305	0.000	0.230	0.389	0.072	DIP2A	0.003	~p_tau+(1|manifest)+cngeneson+pc_mito+brain_region+Sex	0.713	2903.000
ENSG00000162496	0.003	0.230	0.502	-0.042	DHRS3	0.013	~p_tau+(1|manifest)+cngeneson+pc_mito+brain_region+Sex	2.931	1301.000
ENSG00000070476	0.003	0.229	0.424	0.034	ZXDC	0.014	~p_tau+(1|manifest)+cngeneson+pc_mito+brain_region+Sex	3.545	1049.000
ENSG00000006831	0.000	0.229	0.355	0.103	ADIPOR2	0.000	~p_tau+(1|manifest)+cngeneson+pc_mito+brain_region+Sex	1.064	2602.000
ENSG00000120451	0.001	0.229	0.515	-0.057	SNX19	0.005	~p_tau+(1|manifest)+cngeneson+pc_mito+brain_region+Sex	4.723	742.000
ENSG00000107745	0.000	0.229	0.395	0.062	MICU1	0.001	~p_tau+(1|manifest)+cngeneson+pc_mito+brain_region+Sex	0.303	3104.000
ENSG00000178764	0.084	0.228	0.443	0.014	ZHX2	0.143	~p_tau+(1|manifest)+cngeneson+pc_mito+brain_region+Sex	9.472	289.000
ENSG00000117298	0.013	0.228	0.498	-0.042	ECE1	0.036	~p_tau+(1|manifest)+cngeneson+pc_mito+brain_region+Sex	3.779	975.000
ENSG00000148200	0.006	0.227	0.418	0.037	NR6A1	0.021	~p_tau+(1|manifest)+cngeneson+pc_mito+brain_region+Sex	0.135	3135.000
ENSG00000078549	0.010	0.227	0.484	-0.030	ADCYAP1R1	0.029	~p_tau+(1|manifest)+cngeneson+pc_mito+brain_region+Sex	3.366	1110.000
ENSG00000134278	0.010	0.226	0.385	0.068	SPIRE1	0.029	~p_tau+(1|manifest)+cngeneson+pc_mito+brain_region+Sex	0.528	2998.000
ENSG00000179832	0.000	0.226	0.354	0.098	MROH1	0.000	~p_tau+(1|manifest)+cngeneson+pc_mito+brain_region+Sex	1.434	2277.000
ENSG00000130653	0.101	0.226	0.617	-0.166	PNPLA7	0.163	~p_tau+(1|manifest)+cngeneson+pc_mito+brain_region+Sex	6.487	489.000
ENSG00000187079	0.019	0.225	0.379	0.072	TEAD1	0.046	~p_tau+(1|manifest)+cngeneson+pc_mito+brain_region+Sex	4.517	792.000
ENSG00000165029	0.022	0.225	0.487	-0.037	ABCA1	0.051	~p_tau+(1|manifest)+cngeneson+pc_mito+brain_region+Sex	1.789	1995.000
ENSG00000119772	0.000	0.225	0.416	0.035	DNMT3A	0.004	~p_tau+(1|manifest)+cngeneson+pc_mito+brain_region+Sex	1.418	2289.000
ENSG00000173064	0.000	0.225	0.329	0.121	HECTD4	0.000	~p_tau+(1|manifest)+cngeneson+pc_mito+brain_region+Sex	1.913	1901.000
ENSG00000109501	0.063	0.225	0.549	-0.100	WFS1	0.114	~p_tau+(1|manifest)+cngeneson+pc_mito+brain_region+Sex	2.827	1347.000
ENSG00000100207	0.000	0.225	0.354	0.095	TCF20	0.000	~p_tau+(1|manifest)+cngeneson+pc_mito+brain_region+Sex	0.190	3128.000
ENSG00000163162	0.000	0.225	0.379	0.070	RNF149	0.000	~p_tau+(1|manifest)+cngeneson+pc_mito+brain_region+Sex	2.822	1351.000
ENSG00000134775	0.008	0.224	0.434	0.015	FHOD3	0.026	~p_tau+(1|manifest)+cngeneson+pc_mito+brain_region+Sex	1.269	2431.000
ENSG00000182902	0.395	0.224	0.594	-0.146	SLC25A18	0.472	~p_tau+(1|manifest)+cngeneson+pc_mito+brain_region+Sex	12.521	162.000
ENSG00000160007	0.002	0.224	0.446	0.001	ARHGAP35	0.010	~p_tau+(1|manifest)+cngeneson+pc_mito+brain_region+Sex	0.423	3050.000
ENSG00000077713	0.000	0.223	0.421	0.025	SLC25A43	0.001	~p_tau+(1|manifest)+cngeneson+pc_mito+brain_region+Sex	0.716	2902.000
ENSG00000168434	0.000	0.223	0.317	0.129	COG7	0.000	~p_tau+(1|manifest)+cngeneson+pc_mito+brain_region+Sex	0.962	2675.000
ENSG00000155974	0.248	0.223	0.537	-0.091	GRIP1	0.326	~p_tau+(1|manifest)+cngeneson+pc_mito+brain_region+Sex	3.335	1122.000
ENSG00000182944	0.000	0.223	0.362	0.084	EWSR1	0.001	~p_tau+(1|manifest)+cngeneson+pc_mito+brain_region+Sex	0.958	2681.000
ENSG00000103994	0.000	0.222	0.378	0.067	ZNF106	0.001	~p_tau+(1|manifest)+cngeneson+pc_mito+brain_region+Sex	0.170	3131.000
ENSG00000062194	0.006	0.222	0.422	0.022	GPBP1	0.021	~p_tau+(1|manifest)+cngeneson+pc_mito+brain_region+Sex	0.315	3101.000
ENSG00000124422	0.000	0.222	0.349	0.094	USP22	0.000	~p_tau+(1|manifest)+cngeneson+pc_mito+brain_region+Sex	1.018	2632.000
ENSG00000087274	0.000	0.221	0.334	0.109	ADD1	0.003	~p_tau+(1|manifest)+cngeneson+pc_mito+brain_region+Sex	2.509	1510.000
ENSG00000124181	0.038	0.221	0.551	-0.110	PLCG1	0.078	~p_tau+(1|manifest)+cngeneson+pc_mito+brain_region+Sex	6.013	544.000

ENSG00000188026	0.000	0.220	0.410	0.031	RILPL1	0.001	~p_tau+(1|manifest)+cngeneson+pc_mito+brain_region+Sex	2.369	1593.000
ENSG00000152465	0.000	0.220	0.317	0.124	NMT2	0.000	~p_tau+(1|manifest)+cngeneson+pc_mito+brain_region+Sex	0.488	3024.000
ENSG00000107742	0.017	0.220	0.473	-0.033	SPOCK2	0.043	~p_tau+(1|manifest)+cngeneson+pc_mito+brain_region+Sex	3.169	1189.000
ENSG00000111666	0.002	0.220	0.336	0.104	CHPT1	0.009	~p_tau+(1|manifest)+cngeneson+pc_mito+brain_region+Sex	3.470	1066.000
ENSG00000109332	0.001	0.219	0.415	0.024	UBE2D3	0.007	~p_tau+(1|manifest)+cngeneson+pc_mito+brain_region+Sex	0.596	2962.000
ENSG00000138031	0.009	0.219	0.502	-0.064	ADCY3	0.026	~p_tau+(1|manifest)+cngeneson+pc_mito+brain_region+Sex	2.987	1271.000
ENSG00000088387	0.000	0.218	0.386	0.050	DOCK9	0.002	~p_tau+(1|manifest)+cngeneson+pc_mito+brain_region+Sex	1.533	2190.000
ENSG00000122566	0.006	0.218	0.377	0.059	HNRNPA2B1	0.020	~p_tau+(1|manifest)+cngeneson+pc_mito+brain_region+Sex	2.634	1439.000
ENSG00000114770	0.000	0.218	0.352	0.084	ABCC5	0.001	~p_tau+(1|manifest)+cngeneson+pc_mito+brain_region+Sex	0.563	2977.000
ENSG00000007047	0.000	0.218	0.353	0.082	MARK4	0.000	~p_tau+(1|manifest)+cngeneson+pc_mito+brain_region+Sex	0.848	2783.000
ENSG00000165164	0.024	0.218	0.552	-0.117	CFAP47	0.055	~p_tau+(1|manifest)+cngeneson+pc_mito+brain_region+Sex	1.969	1868.000
ENSG00000179241	0.002	0.217	0.420	0.014	LDLRAD3	0.010	~p_tau+(1|manifest)+cngeneson+pc_mito+brain_region+Sex	1.532	2191.000
ENSG00000167972	0.006	0.217	0.495	-0.062	ABCA3	0.022	~p_tau+(1|manifest)+cngeneson+pc_mito+brain_region+Sex	2.462	1542.000
ENSG00000101290	0.000	0.216	0.313	0.120	CDS2	0.000	~p_tau+(1|manifest)+cngeneson+pc_mito+brain_region+Sex	1.270	2430.000
ENSG00000154134	0.130	0.216	0.657	-0.224	ROBO3	0.199	~p_tau+(1|manifest)+cngeneson+pc_mito+brain_region+Sex	8.287	342.000
ENSG00000141905	0.001	0.216	0.416	0.016	NFIC	0.006	~p_tau+(1|manifest)+cngeneson+pc_mito+brain_region+Sex	2.837	1344.000
ENSG00000138640	0.070	0.215	0.566	-0.136	FAM13A	0.125	~p_tau+(1|manifest)+cngeneson+pc_mito+brain_region+Sex	3.256	1149.000
ENSG00000143970	0.001	0.215	0.371	0.059	ASXL2	0.004	~p_tau+(1|manifest)+cngeneson+pc_mito+brain_region+Sex	1.232	2456.000
ENSG00000164741	0.048	0.214	0.504	-0.075	DLC1	0.093	~p_tau+(1|manifest)+cngeneson+pc_mito+brain_region+Sex	5.883	558.000
ENSG00000141959	0.022	0.214	0.548	-0.120	PFKL	0.051	~p_tau+(1|manifest)+cngeneson+pc_mito+brain_region+Sex	9.021	304.000
ENSG00000106829	0.001	0.214	0.398	0.030	TLE4	0.005	~p_tau+(1|manifest)+cngeneson+pc_mito+brain_region+Sex	0.524	3002.000
ENSG00000075413	0.000	0.214	0.285	0.143	MARK3	0.000	~p_tau+(1|manifest)+cngeneson+pc_mito+brain_region+Sex	2.204	1699.000
ENSG00000133961	0.002	0.213	0.391	0.035	NUMB	0.011	~p_tau+(1|manifest)+cngeneson+pc_mito+brain_region+Sex	2.123	1753.000
ENSG00000006715	0.000	0.213	0.308	0.117	VPS41	0.000	~p_tau+(1|manifest)+cngeneson+pc_mito+brain_region+Sex	0.539	2992.000
ENSG00000132669	0.066	0.212	0.400	0.024	RIN2	0.119	~p_tau+(1|manifest)+cngeneson+pc_mito+brain_region+Sex	5.167	670.000
ENSG00000141298	0.000	0.212	0.350	0.074	SSH2	0.003	~p_tau+(1|manifest)+cngeneson+pc_mito+brain_region+Sex	1.300	2405.000
ENSG00000138834	0.001	0.212	0.446	-0.022	MAPK8IP3	0.006	~p_tau+(1|manifest)+cngeneson+pc_mito+brain_region+Sex	4.837	720.000
ENSG00000102858	0.000	0.212	0.335	0.089	MGRN1	0.000	~p_tau+(1|manifest)+cngeneson+pc_mito+brain_region+Sex	1.361	2346.000
ENSG00000106479	0.000	0.211	0.359	0.064	ZNF862	0.004	~p_tau+(1|manifest)+cngeneson+pc_mito+brain_region+Sex	2.159	1726.000
ENSG00000147679	0.006	0.211	0.482	-0.060	UTP23	0.021	~p_tau+(1|manifest)+cngeneson+pc_mito+brain_region+Sex	1.448	2266.000
ENSG00000121892	0.000	0.211	0.396	0.025	PDS5A	0.001	~p_tau+(1|manifest)+cngeneson+pc_mito+brain_region+Sex	0.150	3134.000
ENSG00000214595	0.096	0.210	0.451	-0.030	EML6	0.158	~p_tau+(1|manifest)+cngeneson+pc_mito+brain_region+Sex	1.930	1892.000
ENSG00000173402	0.001	0.210	0.368	0.052	DAG1	0.004	~p_tau+(1|manifest)+cngeneson+pc_mito+brain_region+Sex	0.533	2995.000
ENSG00000108100	0.013	0.210	0.411	0.009	CCNY	0.036	~p_tau+(1|manifest)+cngeneson+pc_mito+brain_region+Sex	1.280	2423.000
ENSG00000171475	0.002	0.210	0.389	0.030	WIPF2	0.011	~p_tau+(1|manifest)+cngeneson+pc_mito+brain_region+Sex	2.147	1734.000
ENSG00000164124	0.014	0.209	0.524	-0.107	TMEM144	0.038	~p_tau+(1|manifest)+cngeneson+pc_mito+brain_region+Sex	1.615	2133.000
ENSG00000068796	0.000	0.209	0.346	0.072	KIF2A	0.001	~p_tau+(1|manifest)+cngeneson+pc_mito+brain_region+Sex	1.198	2484.000
ENSG00000145439	0.000	0.208	0.341	0.075	CBR4	0.000	~p_tau+(1|manifest)+cngeneson+pc_mito+brain_region+Sex	3.238	1160.000
ENSG00000083642	0.011	0.208	0.429	-0.013	PDS5B	0.031	~p_tau+(1|manifest)+cngeneson+pc_mito+brain_region+Sex	0.475	3032.000
ENSG00000196151	0.000	0.208	0.394	0.022	WDSUB1	0.000	~p_tau+(1|manifest)+cngeneson+pc_mito+brain_region+Sex	0.623	2953.000
ENSG00000089486	0.026	0.208	0.493	-0.077	CDIP1	0.059	~p_tau+(1|manifest)+cngeneson+pc_mito+brain_region+Sex	2.422	1562.000
ENSG00000107738	0.090	0.207	0.554	-0.140	VSIR	0.150	~p_tau+(1|manifest)+cngeneson+pc_mito+brain_region+Sex	2.922	1307.000
ENSG00000162104	0.001	0.206	0.423	-0.011	ADCY9	0.004	~p_tau+(1|manifest)+cngeneson+pc_mito+brain_region+Sex	0.689	2920.000
ENSG00000196208	0.041	0.206	0.455	-0.043	GREB1	0.082	~p_tau+(1|manifest)+cngeneson+pc_mito+brain_region+Sex	3.938	924.000
ENSG00000170075	0.173	0.206	0.579	-0.168	GPR37L1	0.247	~p_tau+(1|manifest)+cngeneson+pc_mito+brain_region+Sex	2.790	1366.000
ENSG00000117906	0.014	0.206	0.471	-0.060	RCN2	0.037	~p_tau+(1|manifest)+cngeneson+pc_mito+brain_region+Sex	3.865	942.000
ENSG00000138801	0.001	0.205	0.391	0.019	PAPSS1	0.006	~p_tau+(1|manifest)+cngeneson+pc_mito+brain_region+Sex	0.417	3055.000
ENSG00000134030	0.033	0.205	0.421	-0.011	CTIF	0.069	~p_tau+(1|manifest)+cngeneson+pc_mito+brain_region+Sex	3.419	1088.000
ENSG00000171885	0.008	0.205	0.344	0.065	AQP4	0.026	~p_tau+(1|manifest)+cngeneson+pc_mito+brain_region+Sex	10.977	209.000
ENSG00000184349	0.331	0.205	0.500	-0.091	EFNA5	0.410	~p_tau+(1|manifest)+cngeneson+pc_mito+brain_region+Sex	3.106	1217.000
ENSG00000198853	0.000	0.205	0.303	0.106	RUSC2	0.000	~p_tau+(1|manifest)+cngeneson+pc_mito+brain_region+Sex	0.735	2882.000
ENSG00000074416	0.281	0.204	0.505	-0.096	MGLL	0.362	~p_tau+(1|manifest)+cngeneson+pc_mito+brain_region+Sex	2.000	1840.000
ENSG00000140391	0.003	0.204	0.404	0.004	TSPAN3	0.014	~p_tau+(1|manifest)+cngeneson+pc_mito+brain_region+Sex	1.818	1975.000
ENSG00000257923	0.000	0.203	0.332	0.075	CUX1	0.001	~p_tau+(1|manifest)+cngeneson+pc_mito+brain_region+Sex	1.028	2622.000
ENSG00000171530	0.000	0.203	0.372	0.034	TBCA	0.001	~p_tau+(1|manifest)+cngeneson+pc_mito+brain_region+Sex	0.770	2855.000
ENSG00000197119	0.001	0.203	0.554	-0.149	SLC25A29	0.005	~p_tau+(1|manifest)+cngeneson+pc_mito+brain_region+Sex	4.220	853.000
ENSG00000162923	0.000	0.203	0.325	0.081	WDR26	0.000	~p_tau+(1|manifest)+cngeneson+pc_mito+brain_region+Sex	0.525	3001.000
ENSG00000178252	0.030	0.202	0.530	-0.126	WDR6	0.065	~p_tau+(1|manifest)+cngeneson+pc_mito+brain_region+Sex	6.396	499.000
ENSG00000104290	0.011	0.201	0.450	-0.048	FZD3	0.031	~p_tau+(1|manifest)+cngeneson+pc_mito+brain_region+Sex	1.482	2238.000
ENSG00000106615	0.015	0.200	0.400	0.001	RHEB	0.039	~p_tau+(1|manifest)+cngeneson+pc_mito+brain_region+Sex	1.692	2074.000
ENSG00000076555	0.258	0.200	0.466	-0.066	ACACB	0.339	~p_tau+(1|manifest)+cngeneson+pc_mito+brain_region+Sex	14.487	109.000
ENSG00000141664	0.000	0.200	0.353	0.047	ZCCHC2	0.001	~p_tau+(1|manifest)+cngeneson+pc_mito+brain_region+Sex	0.210	3124.000
ENSG00000080845	0.000	0.200	0.394	0.006	DLGAP4	0.002	~p_tau+(1|manifest)+cngeneson+pc_mito+brain_region+Sex	1.407	2301.000
ENSG00000134873	0.006	0.199	0.501	-0.102	CLDN10	0.022	~p_tau+(1|manifest)+cngeneson+pc_mito+brain_region+Sex	1.001	2651.000
ENSG00000119950	0.055	0.199	0.417	-0.019	MXI1	0.104	~p_tau+(1|manifest)+cngeneson+pc_mito+brain_region+Sex	3.719	1001.000
ENSG00000115468	0.001	0.199	0.400	-0.002	EFHD1	0.007	~p_tau+(1|manifest)+cngeneson+pc_mito+brain_region+Sex	1.251	2442.000
ENSG00000160199	0.000	0.198	0.336	0.061	PKNOX1	0.001	~p_tau+(1|manifest)+cngeneson+pc_mito+brain_region+Sex	0.741	2873.000
ENSG00000144744	0.000	0.197	0.263	0.132	UBA3	0.000	~p_tau+(1|manifest)+cngeneson+pc_mito+brain_region+Sex	0.814	2818.000
ENSG00000107779	0.099	0.197	0.377	0.017	BMPR1A	0.161	~p_tau+(1|manifest)+cngeneson+pc_mito+brain_region+Sex	8.233	346.000
ENSG00000101605	0.023	0.197	0.600	-0.206	MYOM1	0.053	~p_tau+(1|manifest)+cngeneson+pc_mito+brain_region+Sex	4.356	829.000
ENSG00000118418	0.067	0.197	0.543	-0.150	HMGN3	0.120	~p_tau+(1|manifest)+cngeneson+pc_mito+brain_region+Sex	2.383	1586.000
ENSG00000040933	0.000	0.196	0.373	0.019	INPP4A	0.003	~p_tau+(1|manifest)+cngeneson+pc_mito+brain_region+Sex	0.839	2791.000
ENSG00000164684	0.011	0.195	0.400	-0.009	ZNF704	0.032	~p_tau+(1|manifest)+cngeneson+pc_mito+brain_region+Sex	0.905	2731.000
ENSG00000137177	0.015	0.195	0.368	0.022	KIF13A	0.040	~p_tau+(1|manifest)+cngeneson+pc_mito+brain_region+Sex	2.143	1740.000
ENSG00000184787	0.001	0.195	0.327	0.063	UBE2G2	0.004	~p_tau+(1|manifest)+cngeneson+pc_mito+brain_region+Sex	2.881	1324.000
ENSG00000165449	0.193	0.195	0.591	-0.202	SLC16A9	0.268	~p_tau+(1|manifest)+cngeneson+pc_mito+brain_region+Sex	2.132	1748.000
ENSG00000134504	0.010	0.194	0.369	0.020	KCTD1	0.030	~p_tau+(1|manifest)+cngeneson+pc_mito+brain_region+Sex	0.912	2724.000
ENSG00000075420	0.341	0.194	0.536	-0.148	FNDC3B	0.420	~p_tau+(1|manifest)+cngeneson+pc_mito+brain_region+Sex	5.473	628.000
ENSG00000034677	0.305	0.193	0.485	-0.098	RNF19A	0.386	~p_tau+(1|manifest)+cngeneson+pc_mito+brain_region+Sex	6.259	514.000
ENSG00000077549	0.000	0.193	0.318	0.069	CAPZB	0.001	~p_tau+(1|manifest)+cngeneson+pc_mito+brain_region+Sex	2.206	1697.000
ENSG00000160209	0.001	0.193	0.378	0.008	PDXK	0.005	~p_tau+(1|manifest)+cngeneson+pc_mito+brain_region+Sex	2.060	1793.000
ENSG00000151694	0.000	0.193	0.399	-0.013	ADAM17	0.000	~p_tau+(1|manifest)+cngeneson+pc_mito+brain_region+Sex	1.923	1898.000
ENSG00000184486	0.036	0.192	0.527	-0.142	POU3F2	0.074	~p_tau+(1|manifest)+cngeneson+pc_mito+brain_region+Sex	3.776	978.000
ENSG00000171132	0.038	0.192	0.379	0.005	PRKCE	0.077	~p_tau+(1|manifest)+cngeneson+pc_mito+brain_region+Sex	0.843	2787.000
ENSG00000133104	0.002	0.192	0.390	-0.006	SPART	0.011	~p_tau+(1|manifest)+cngeneson+pc_mito+brain_region+Sex	0.356	3087.000
ENSG00000127022	0.006	0.192	0.403	-0.020	CANX	0.020	~p_tau+(1|manifest)+cngeneson+pc_mito+brain_region+Sex	1.120	2555.000
ENSG00000147862	0.023	0.192	0.341	0.043	NFIB	0.053	~p_tau+(1|manifest)+cngeneson+pc_mito+brain_region+Sex	4.586	774.000
ENSG00000101493	0.199	0.191	0.522	-0.139	ZNF516	0.274	~p_tau+(1|manifest)+cngeneson+pc_mito+brain_region+Sex	3.013	1256.000
ENSG00000197555	0.016	0.191	0.319	0.063	SIPA1L1	0.041	~p_tau+(1|manifest)+cngeneson+pc_mito+brain_region+Sex	3.812	964.000
ENSG00000173517	0.019	0.191	0.393	-0.011	PEAK1	0.046	~p_tau+(1|manifest)+cngeneson+pc_mito+brain_region+Sex	3.870	938.000
ENSG00000109819	0.243	0.190	0.580	-0.199	PPARGC1A	0.322	~p_tau+(1|manifest)+cngeneson+pc_mito+brain_region+Sex	3.047	1245.000
ENSG00000141540	0.005	0.190	0.465	-0.084	TTYH2	0.017	~p_tau+(1|manifest)+cngeneson+pc_mito+brain_region+Sex	1.499	2219.000
ENSG00000148842	0.004	0.190	0.374	0.007	CNNM2	0.017	~p_tau+(1|manifest)+cngeneson+pc_mito+brain_region+Sex	1.464	2249.000
ENSG00000001084	0.010	0.190	0.407	-0.027	GCLC	0.029	~p_tau+(1|manifest)+cngeneson+pc_mito+brain_region+Sex	0.852	2777.000
ENSG00000100427	0.184	0.190	0.533	-0.154	MLC1	0.259	~p_tau+(1|manifest)+cngeneson+pc_mito+brain_region+Sex	5.219	665.000
ENSG00000107882	0.001	0.190	0.352	0.027	SUFU	0.007	~p_tau+(1|manifest)+cngeneson+pc_mito+brain_region+Sex	2.128	1749.000
ENSG00000078699	0.000	0.189	0.275	0.104	CBFA2T2	0.000	~p_tau+(1|manifest)+cngeneson+pc_mito+brain_region+Sex	1.247	2443.000
ENSG00000140090	0.373	0.189	0.524	-0.145	SLC24A4	0.450	~p_tau+(1|manifest)+cngeneson+pc_mito+brain_region+Sex	5.621	600.000
ENSG00000165626	0.000	0.189	0.341	0.037	BEND7	0.002	~p_tau+(1|manifest)+cngeneson+pc_mito+brain_region+Sex	0.164	3133.000
ENSG00000146005	0.537	0.189	0.666	-0.288	PSD2	0.600	~p_tau+(1|manifest)+cngeneson+pc_mito+brain_region+Sex	15.205	93.000
ENSG00000164543	0.000	0.189	0.354	0.024	STK17A	0.003	~p_tau+(1|manifest)+cngeneson+pc_mito+brain_region+Sex	1.485	2232.000
ENSG00000141568	0.000	0.188	0.339	0.037	FOXK2	0.001	~p_tau+(1|manifest)+cngeneson+pc_mito+brain_region+Sex	1.484	2235.000
ENSG00000179222	0.031	0.188	0.467	-0.092	MAGED1	0.066	~p_tau+(1|manifest)+cngeneson+pc_mito+brain_region+Sex	2.300	1637.000
ENSG00000112715	0.523	0.187	0.773	-0.398	VEGFA	0.589	~p_tau+(1|manifest)+cngeneson+pc_mito+brain_region+Sex	8.973	306.000
ENSG00000076716	0.002	0.187	0.423	-0.049	GPC4	0.011	~p_tau+(1|manifest)+cngeneson+pc_mito+brain_region+Sex	0.321	3098.000
ENSG00000132388	0.000	0.187	0.324	0.049	UBE2G1	0.000	~p_tau+(1|manifest)+cngeneson+pc_mito+brain_region+Sex	0.840	2790.000
ENSG00000136367	0.000	0.187	0.300	0.073	ZFHX2	0.000	~p_tau+(1|manifest)+cngeneson+pc_mito+brain_region+Sex	1.162	2521.000
ENSG00000071967	0.000	0.187	0.398	-0.025	CYBRD1	0.003	~p_tau+(1|manifest)+cngeneson+pc_mito+brain_region+Sex	0.520	3004.000
ENSG00000105221	0.009	0.186	0.385	-0.013	AKT2	0.027	~p_tau+(1|manifest)+cngeneson+pc_mito+brain_region+Sex	4.180	860.000
ENSG00000105088	0.270	0.186	0.578	-0.205	OLFM2	0.352	~p_tau+(1|manifest)+cngeneson+pc_mito+brain_region+Sex	5.468	630.000
ENSG00000112576	0.137	0.185	0.749	-0.378	CCND3	0.206	~p_tau+(1|manifest)+cngeneson+pc_mito+brain_region+Sex	8.338	337.000
ENSG00000126016	0.003	0.185	0.374	-0.003	AMOT	0.013	~p_tau+(1|manifest)+cngeneson+pc_mito+brain_region+Sex	0.652	2938.000
ENSG00000164828	0.011	0.185	0.379	-0.009	SUN1	0.033	~p_tau+(1|manifest)+cngeneson+pc_mito+brain_region+Sex	6.096	536.000
ENSG00000023228	0.000	0.184	0.312	0.057	NDUFS1	0.001	~p_tau+(1|manifest)+cngeneson+pc_mito+brain_region+Sex	1.076	2588.000
ENSG00000108256	0.038	0.184	0.454	-0.086	NUFIP2	0.078	~p_tau+(1|manifest)+cngeneson+pc_mito+brain_region+Sex	2.802	1357.000
ENSG00000137642	0.007	0.184	0.329	0.039	SORL1	0.022	~p_tau+(1|manifest)+cngeneson+pc_mito+brain_region+Sex	2.590	1461.000
ENSG00000125952	0.000	0.184	0.324	0.044	MAX	0.002	~p_tau+(1|manifest)+cngeneson+pc_mito+brain_region+Sex	2.240	1676.000
ENSG00000184792	0.001	0.183	0.334	0.033	OSBP2	0.008	~p_tau+(1|manifest)+cngeneson+pc_mito+brain_region+Sex	1.048	2610.000
ENSG00000123352	0.000	0.183	0.289	0.076	SPATS2	0.000	~p_tau+(1|manifest)+cngeneson+pc_mito+brain_region+Sex	0.858	2773.000
ENSG00000091409	0.015	0.182	0.398	-0.034	ITGA6	0.038	~p_tau+(1|manifest)+cngeneson+pc_mito+brain_region+Sex	5.504	623.000
ENSG00000061273	0.153	0.182	0.576	-0.213	HDAC7	0.224	~p_tau+(1|manifest)+cngeneson+pc_mito+brain_region+Sex	7.291	410.000
ENSG00000154188	0.740	0.180	0.724	-0.364	ANGPT1	0.778	~p_tau+(1|manifest)+cngeneson+pc_mito+brain_region+Sex	2.974	1276.000
ENSG00000185158	0.000	0.180	0.356	0.003	LRRC37B	0.003	~p_tau+(1|manifest)+cngeneson+pc_mito+brain_region+Sex	0.745	2869.000
ENSG00000127527	0.026	0.179	0.376	-0.017	EPS15L1	0.059	~p_tau+(1|manifest)+cngeneson+pc_mito+brain_region+Sex	4.028	905.000
ENSG00000259417	0.358	0.179	0.665	-0.307	CTXND1	0.436	~p_tau+(1|manifest)+cngeneson+pc_mito+brain_region+Sex	9.428	290.000
ENSG00000126705	0.000	0.179	0.399	-0.041	AHDC1	0.003	~p_tau+(1|manifest)+cngeneson+pc_mito+brain_region+Sex	2.218	1692.000
ENSG00000056998	0.049	0.179	0.513	-0.156	GYG2	0.095	~p_tau+(1|manifest)+cngeneson+pc_mito+brain_region+Sex	4.389	822.000
ENSG00000164830	0.007	0.178	0.357	0.000	OXR1	0.023	~p_tau+(1|manifest)+cngeneson+pc_mito+brain_region+Sex	2.795	1361.000
ENSG00000101680	0.211	0.178	0.515	-0.159	LAMA1	0.288	~p_tau+(1|manifest)+cngeneson+pc_mito+brain_region+Sex	4.621	765.000
ENSG00000012983	0.005	0.178	0.310	0.045	MAP4K5	0.020	~p_tau+(1|manifest)+cngeneson+pc_mito+brain_region+Sex	1.368	2341.000
ENSG00000145907	0.000	0.177	0.387	-0.033	G3BP1	0.003	~p_tau+(1|manifest)+cngeneson+pc_mito+brain_region+Sex	0.696	2916.000
ENSG00000162909	0.089	0.177	0.453	-0.098	CAPN2	0.149	~p_tau+(1|manifest)+cngeneson+pc_mito+brain_region+Sex	6.215	522.000
ENSG00000243156	0.002	0.177	0.411	-0.056	MICAL3	0.011	~p_tau+(1|manifest)+cngeneson+pc_mito+brain_region+Sex	2.463	1541.000
ENSG00000113384	0.000	0.177	0.348	0.007	GOLPH3	0.000	~p_tau+(1|manifest)+cngeneson+pc_mito+brain_region+Sex	0.468	3035.000
ENSG00000122741	0.000	0.177	0.284	0.070	DCAF10	0.001	~p_tau+(1|manifest)+cngeneson+pc_mito+brain_region+Sex	0.291	3108.000
ENSG00000196361	0.002	0.177	0.335	0.018	ELAVL3	0.008	~p_tau+(1|manifest)+cngeneson+pc_mito+brain_region+Sex	1.240	2447.000
ENSG00000148356	0.001	0.176	0.367	-0.014	LRSAM1	0.005	~p_tau+(1|manifest)+cngeneson+pc_mito+brain_region+Sex	2.226	1684.000
ENSG00000113657	0.152	0.176	0.518	-0.165	DPYSL3	0.223	~p_tau+(1|manifest)+cngeneson+pc_mito+brain_region+Sex	5.356	642.000
ENSG00000072134	0.039	0.176	0.372	-0.019	EPN2	0.078	~p_tau+(1|manifest)+cngeneson+pc_mito+brain_region+Sex	3.068	1236.000
ENSG00000101558	0.002	0.176	0.397	-0.045	VAPA	0.008	~p_tau+(1|manifest)+cngeneson+pc_mito+brain_region+Sex	0.253	3114.000
ENSG00000129116	0.288	0.176	0.394	-0.042	PALLD	0.369	~p_tau+(1|manifest)+cngeneson+pc_mito+brain_region+Sex	5.954	551.000
ENSG00000160191	0.008	0.176	0.406	-0.055	PDE9A	0.026	~p_tau+(1|manifest)+cngeneson+pc_mito+brain_region+Sex	2.325	1617.000
ENSG00000114023	0.041	0.174	0.373	-0.024	FAM162A	0.081	~p_tau+(1|manifest)+cngeneson+pc_mito+brain_region+Sex	1.137	2546.000
ENSG00000100650	0.137	0.174	0.521	-0.172	SRSF5	0.206	~p_tau+(1|manifest)+cngeneson+pc_mito+brain_region+Sex	12.410	165.000
ENSG00000179981	0.079	0.174	0.527	-0.178	TSHZ1	0.136	~p_tau+(1|manifest)+cngeneson+pc_mito+brain_region+Sex	3.808	966.000
ENSG00000134490	0.117	0.174	0.560	-0.212	TMEM241	0.183	~p_tau+(1|manifest)+cngeneson+pc_mito+brain_region+Sex	8.010	356.000
ENSG00000002586	0.001	0.174	0.377	-0.029	CD99	0.006	~p_tau+(1|manifest)+cngeneson+pc_mito+brain_region+Sex	4.225	851.000
ENSG00000215301	0.005	0.174	0.357	-0.009	DDX3X	0.019	~p_tau+(1|manifest)+cngeneson+pc_mito+brain_region+Sex	3.890	932.000
ENSG00000135720	0.000	0.173	0.284	0.063	DYNC1LI2	0.000	~p_tau+(1|manifest)+cngeneson+pc_mito+brain_region+Sex	1.369	2340.000
ENSG00000145794	0.161	0.173	0.412	-0.065	MEGF10	0.232	~p_tau+(1|manifest)+cngeneson+pc_mito+brain_region+Sex	1.902	1911.000
ENSG00000136158	0.020	0.173	0.417	-0.071	SPRY2	0.048	~p_tau+(1|manifest)+cngeneson+pc_mito+brain_region+Sex	3.202	1176.000
ENSG00000187109	0.011	0.173	0.396	-0.051	NAP1L1	0.032	~p_tau+(1|manifest)+cngeneson+pc_mito+brain_region+Sex	3.117	1210.000
ENSG00000177565	0.084	0.173	0.412	-0.066	TBL1XR1	0.143	~p_tau+(1|manifest)+cngeneson+pc_mito+brain_region+Sex	0.937	2699.000
ENSG00000198130	0.225	0.172	0.410	-0.066	HIBCH	0.301	~p_tau+(1|manifest)+cngeneson+pc_mito+brain_region+Sex	3.990	914.000
ENSG00000161813	0.006	0.172	0.438	-0.094	LARP4	0.022	~p_tau+(1|manifest)+cngeneson+pc_mito+brain_region+Sex	1.654	2096.000
ENSG00000120742	0.050	0.172	0.385	-0.041	SERP1	0.095	~p_tau+(1|manifest)+cngeneson+pc_mito+brain_region+Sex	1.376	2333.000
ENSG00000167216	0.078	0.172	0.418	-0.074	KATNAL2	0.135	~p_tau+(1|manifest)+cngeneson+pc_mito+brain_region+Sex	1.511	2207.000
ENSG00000164463	0.000	0.172	0.304	0.040	CREBRF	0.000	~p_tau+(1|manifest)+cngeneson+pc_mito+brain_region+Sex	0.216	3120.000
ENSG00000011295	0.081	0.172	0.425	-0.081	TTC19	0.138	~p_tau+(1|manifest)+cngeneson+pc_mito+brain_region+Sex	3.861	944.000
ENSG00000137449	0.115	0.171	0.530	-0.188	CPEB2	0.181	~p_tau+(1|manifest)+cngeneson+pc_mito+brain_region+Sex	2.272	1657.000
ENSG00000219481	0.010	0.171	0.389	-0.048	NBPF1	0.029	~p_tau+(1|manifest)+cngeneson+pc_mito+brain_region+Sex	1.795	1991.000
ENSG00000110075	0.000	0.170	0.278	0.063	PPP6R3	0.003	~p_tau+(1|manifest)+cngeneson+pc_mito+brain_region+Sex	1.144	2540.000
ENSG00000173041	0.028	0.170	0.453	-0.113	ZNF680	0.061	~p_tau+(1|manifest)+cngeneson+pc_mito+brain_region+Sex	1.218	2467.000
ENSG00000079805	0.000	0.170	0.351	-0.011	DNM2	0.000	~p_tau+(1|manifest)+cngeneson+pc_mito+brain_region+Sex	1.773	2000.000
ENSG00000188153	0.020	0.170	0.453	-0.112	COL4A5	0.048	~p_tau+(1|manifest)+cngeneson+pc_mito+brain_region+Sex	0.518	3005.000
ENSG00000104205	0.005	0.170	0.291	0.049	SGK3	0.019	~p_tau+(1|manifest)+cngeneson+pc_mito+brain_region+Sex	2.702	1404.000
ENSG00000132323	0.000	0.170	0.283	0.057	ILKAP	0.001	~p_tau+(1|manifest)+cngeneson+pc_mito+brain_region+Sex	1.201	2481.000
ENSG00000008441	0.078	0.170	0.440	-0.101	NFIX	0.135	~p_tau+(1|manifest)+cngeneson+pc_mito+brain_region+Sex	4.639	761.000
ENSG00000148400	0.015	0.169	0.433	-0.094	NOTCH1	0.040	~p_tau+(1|manifest)+cngeneson+pc_mito+brain_region+Sex	3.664	1016.000
ENSG00000257103	0.000	0.169	0.287	0.051	LSM14A	0.000	~p_tau+(1|manifest)+cngeneson+pc_mito+brain_region+Sex	1.290	2413.000
ENSG00000065000	0.000	0.168	0.316	0.021	AP3D1	0.004	~p_tau+(1|manifest)+cngeneson+pc_mito+brain_region+Sex	1.977	1860.000
ENSG00000171766	0.009	0.168	0.435	-0.099	GATM	0.028	~p_tau+(1|manifest)+cngeneson+pc_mito+brain_region+Sex	1.130	2550.000
ENSG00000105854	0.056	0.168	0.306	0.030	PON2	0.105	~p_tau+(1|manifest)+cngeneson+pc_mito+brain_region+Sex	5.820	563.000
ENSG00000064999	0.000	0.168	0.250	0.086	ANKS1A	0.000	~p_tau+(1|manifest)+cngeneson+pc_mito+brain_region+Sex	0.459	3041.000
ENSG00000185985	0.024	0.168	0.424	-0.089	SLITRK2	0.055	~p_tau+(1|manifest)+cngeneson+pc_mito+brain_region+Sex	2.503	1513.000
ENSG00000072110	0.548	0.167	0.541	-0.206	ACTN1	0.610	~p_tau+(1|manifest)+cngeneson+pc_mito+brain_region+Sex	8.245	345.000
ENSG00000135744	0.340	0.167	0.567	-0.232	AGT	0.418	~p_tau+(1|manifest)+cngeneson+pc_mito+brain_region+Sex	2.343	1602.000
ENSG00000049246	0.116	0.167	0.555	-0.221	PER3	0.182	~p_tau+(1|manifest)+cngeneson+pc_mito+brain_region+Sex	4.891	711.000
ENSG00000102172	0.004	0.167	0.390	-0.056	SMS	0.017	~p_tau+(1|manifest)+cngeneson+pc_mito+brain_region+Sex	0.811	2821.000
ENSG00000131095	0.530	0.167	0.574	-0.240	GFAP	0.594	~p_tau+(1|manifest)+cngeneson+pc_mito+brain_region+Sex	33.577	13.000
ENSG00000130939	0.000	0.166	0.283	0.049	UBE4B	0.000	~p_tau+(1|manifest)+cngeneson+pc_mito+brain_region+Sex	1.274	2426.000
ENSG00000077044	0.161	0.165	0.440	-0.110	DGKD	0.232	~p_tau+(1|manifest)+cngeneson+pc_mito+brain_region+Sex	5.802	568.000
ENSG00000168610	0.123	0.165	0.447	-0.117	STAT3	0.190	~p_tau+(1|manifest)+cngeneson+pc_mito+brain_region+Sex	4.552	785.000
ENSG00000105662	0.017	0.165	0.405	-0.076	CRTC1	0.042	~p_tau+(1|manifest)+cngeneson+pc_mito+brain_region+Sex	3.624	1027.000
ENSG00000115902	0.119	0.165	0.513	-0.184	SLC1A4	0.184	~p_tau+(1|manifest)+cngeneson+pc_mito+brain_region+Sex	4.709	745.000
ENSG00000157445	0.526	0.165	0.714	-0.385	CACNA2D3	0.591	~p_tau+(1|manifest)+cngeneson+pc_mito+brain_region+Sex	9.616	279.000
ENSG00000155324	0.009	0.163	0.269	0.058	GRAMD2B	0.028	~p_tau+(1|manifest)+cngeneson+pc_mito+brain_region+Sex	5.996	547.000
ENSG00000152795	0.017	0.163	0.433	-0.107	HNRNPDL	0.042	~p_tau+(1|manifest)+cngeneson+pc_mito+brain_region+Sex	5.303	650.000
ENSG00000076685	0.014	0.163	0.278	0.049	NT5C2	0.038	~p_tau+(1|manifest)+cngeneson+pc_mito+brain_region+Sex	1.380	2329.000
ENSG00000154928	0.399	0.163	0.471	-0.145	EPHB1	0.475	~p_tau+(1|manifest)+cngeneson+pc_mito+brain_region+Sex	8.553	320.000
ENSG00000173960	0.000	0.162	0.263	0.061	UBXN2A	0.003	~p_tau+(1|manifest)+cngeneson+pc_mito+brain_region+Sex	0.767	2857.000
ENSG00000138029	0.000	0.162	0.307	0.017	HADHB	0.003	~p_tau+(1|manifest)+cngeneson+pc_mito+brain_region+Sex	2.214	1693.000
ENSG00000029725	0.000	0.162	0.324	0.000	RABEP1	0.003	~p_tau+(1|manifest)+cngeneson+pc_mito+brain_region+Sex	1.951	1876.000
ENSG00000165185	0.045	0.162	0.321	0.002	KIAA1958	0.088	~p_tau+(1|manifest)+cngeneson+pc_mito+brain_region+Sex	2.960	1282.000
ENSG00000134243	0.000	0.162	0.272	0.052	SORT1	0.001	~p_tau+(1|manifest)+cngeneson+pc_mito+brain_region+Sex	0.260	3111.000
ENSG00000034053	0.072	0.162	0.491	-0.168	APBA2	0.126	~p_tau+(1|manifest)+cngeneson+pc_mito+brain_region+Sex	4.897	710.000
ENSG00000204842	0.000	0.161	0.237	0.085	ATXN2	0.003	~p_tau+(1|manifest)+cngeneson+pc_mito+brain_region+Sex	1.272	2429.000
ENSG00000171603	0.013	0.161	0.335	-0.013	CLSTN1	0.036	~p_tau+(1|manifest)+cngeneson+pc_mito+brain_region+Sex	0.555	2980.000
ENSG00000015153	0.011	0.161	0.327	-0.006	YAF2	0.032	~p_tau+(1|manifest)+cngeneson+pc_mito+brain_region+Sex	0.554	2981.000
ENSG00000146373	0.008	0.160	0.354	-0.033	RNF217	0.026	~p_tau+(1|manifest)+cngeneson+pc_mito+brain_region+Sex	1.098	2570.000
ENSG00000183049	0.065	0.160	0.435	-0.114	CAMK1D	0.117	~p_tau+(1|manifest)+cngeneson+pc_mito+brain_region+Sex	1.576	2155.000
ENSG00000148482	0.119	0.160	0.380	-0.060	SLC39A12	0.184	~p_tau+(1|manifest)+cngeneson+pc_mito+brain_region+Sex	2.119	1757.000
ENSG00000105738	0.001	0.160	0.331	-0.012	SIPA1L3	0.005	~p_tau+(1|manifest)+cngeneson+pc_mito+brain_region+Sex	0.674	2927.000
ENSG00000167323	0.003	0.159	0.333	-0.014	STIM1	0.012	~p_tau+(1|manifest)+cngeneson+pc_mito+brain_region+Sex	0.710	2905.000
ENSG00000088826	0.033	0.159	0.462	-0.143	SMOX	0.071	~p_tau+(1|manifest)+cngeneson+pc_mito+brain_region+Sex	4.402	817.000
ENSG00000141503	0.090	0.159	0.407	-0.088	MINK1	0.150	~p_tau+(1|manifest)+cngeneson+pc_mito+brain_region+Sex	5.113	683.000
ENSG00000148848	0.734	0.159	0.677	-0.359	ADAM12	0.772	~p_tau+(1|manifest)+cngeneson+pc_mito+brain_region+Sex	12.670	154.000
ENSG00000140396	0.040	0.159	0.286	0.031	NCOA2	0.081	~p_tau+(1|manifest)+cngeneson+pc_mito+brain_region+Sex	0.464	3037.000
ENSG00000160613	0.001	0.159	0.315	0.002	PCSK7	0.007	~p_tau+(1|manifest)+cngeneson+pc_mito+brain_region+Sex	2.072	1787.000
ENSG00000147459	0.108	0.158	0.453	-0.136	DOCK5	0.172	~p_tau+(1|manifest)+cngeneson+pc_mito+brain_region+Sex	2.716	1398.000
ENSG00000131149	0.017	0.157	0.458	-0.143	GSE1	0.042	~p_tau+(1|manifest)+cngeneson+pc_mito+brain_region+Sex	2.603	1455.000
ENSG00000135387	0.000	0.157	0.292	0.023	CAPRIN1	0.002	~p_tau+(1|manifest)+cngeneson+pc_mito+brain_region+Sex	0.546	2990.000
ENSG00000109472	0.036	0.157	0.279	0.035	CPE	0.075	~p_tau+(1|manifest)+cngeneson+pc_mito+brain_region+Sex	7.162	424.000
ENSG00000139990	0.000	0.157	0.327	-0.013	DCAF5	0.004	~p_tau+(1|manifest)+cngeneson+pc_mito+brain_region+Sex	0.955	2688.000
ENSG00000160953	0.081	0.157	0.420	-0.107	PWWP3A	0.139	~p_tau+(1|manifest)+cngeneson+pc_mito+brain_region+Sex	5.740	581.000
ENSG00000068724	0.000	0.155	0.297	0.014	TTC7A	0.002	~p_tau+(1|manifest)+cngeneson+pc_mito+brain_region+Sex	1.916	1900.000
ENSG00000147649	0.000	0.155	0.259	0.052	MTDH	0.001	~p_tau+(1|manifest)+cngeneson+pc_mito+brain_region+Sex	1.374	2336.000
ENSG00000154978	0.001	0.155	0.313	-0.003	VOPP1	0.006	~p_tau+(1|manifest)+cngeneson+pc_mito+brain_region+Sex	0.827	2804.000
ENSG00000163605	0.000	0.155	0.337	-0.028	PPP4R2	0.002	~p_tau+(1|manifest)+cngeneson+pc_mito+brain_region+Sex	0.881	2753.000
ENSG00000137710	0.057	0.155	0.422	-0.113	RDX	0.107	~p_tau+(1|manifest)+cngeneson+pc_mito+brain_region+Sex	4.660	755.000
ENSG00000099246	0.000	0.155	0.269	0.040	RAB18	0.001	~p_tau+(1|manifest)+cngeneson+pc_mito+brain_region+Sex	0.786	2843.000
ENSG00000185716	0.003	0.154	0.329	-0.020	MOSMO	0.014	~p_tau+(1|manifest)+cngeneson+pc_mito+brain_region+Sex	0.810	2824.000
ENSG00000165113	0.000	0.154	0.326	-0.018	GKAP1	0.002	~p_tau+(1|manifest)+cngeneson+pc_mito+brain_region+Sex	0.297	3105.000
ENSG00000079950	0.000	0.154	0.249	0.059	STX7	0.000	~p_tau+(1|manifest)+cngeneson+pc_mito+brain_region+Sex	0.409	3059.000
ENSG00000087258	0.173	0.153	0.334	-0.027	GNAO1	0.247	~p_tau+(1|manifest)+cngeneson+pc_mito+brain_region+Sex	2.787	1368.000
ENSG00000133030	0.002	0.153	0.278	0.028	MPRIP	0.008	~p_tau+(1|manifest)+cngeneson+pc_mito+brain_region+Sex	1.929	1894.000
ENSG00000119318	0.000	0.153	0.245	0.061	RAD23B	0.000	~p_tau+(1|manifest)+cngeneson+pc_mito+brain_region+Sex	0.642	2942.000
ENSG00000184384	0.034	0.152	0.288	0.015	MAML2	0.072	~p_tau+(1|manifest)+cngeneson+pc_mito+brain_region+Sex	3.930	925.000
ENSG00000148498	0.019	0.151	0.295	0.008	PARD3	0.047	~p_tau+(1|manifest)+cngeneson+pc_mito+brain_region+Sex	9.426	291.000
ENSG00000111676	0.134	0.151	0.406	-0.103	ATN1	0.202	~p_tau+(1|manifest)+cngeneson+pc_mito+brain_region+Sex	5.552	615.000
ENSG00000141441	0.264	0.151	0.330	-0.028	GAREM1	0.345	~p_tau+(1|manifest)+cngeneson+pc_mito+brain_region+Sex	6.502	487.000
ENSG00000147316	0.058	0.151	0.488	-0.185	MCPH1	0.107	~p_tau+(1|manifest)+cngeneson+pc_mito+brain_region+Sex	1.088	2576.000
ENSG00000149212	0.423	0.151	0.531	-0.228	SESN3	0.498	~p_tau+(1|manifest)+cngeneson+pc_mito+brain_region+Sex	5.086	689.000
ENSG00000011114	0.000	0.150	0.282	0.018	BTBD7	0.002	~p_tau+(1|manifest)+cngeneson+pc_mito+brain_region+Sex	2.009	1832.000
ENSG00000084733	0.000	0.150	0.255	0.045	RAB10	0.001	~p_tau+(1|manifest)+cngeneson+pc_mito+brain_region+Sex	0.511	3011.000
ENSG00000179912	0.003	0.150	0.358	-0.059	R3HDM2	0.012	~p_tau+(1|manifest)+cngeneson+pc_mito+brain_region+Sex	0.344	3091.000
ENSG00000088538	0.017	0.149	0.252	0.047	DOCK3	0.042	~p_tau+(1|manifest)+cngeneson+pc_mito+brain_region+Sex	1.531	2192.000
ENSG00000165476	0.000	0.149	0.229	0.069	REEP3	0.000	~p_tau+(1|manifest)+cngeneson+pc_mito+brain_region+Sex	0.675	2926.000
ENSG00000164904	0.041	0.149	0.356	-0.058	ALDH7A1	0.082	~p_tau+(1|manifest)+cngeneson+pc_mito+brain_region+Sex	4.835	721.000
ENSG00000081913	0.193	0.149	0.307	-0.010	PHLPP1	0.268	~p_tau+(1|manifest)+cngeneson+pc_mito+brain_region+Sex	5.732	582.000
ENSG00000090905	0.019	0.148	0.265	0.032	TNRC6A	0.046	~p_tau+(1|manifest)+cngeneson+pc_mito+brain_region+Sex	10.401	240.000
ENSG00000185238	0.003	0.148	0.359	-0.062	PRMT3	0.014	~p_tau+(1|manifest)+cngeneson+pc_mito+brain_region+Sex	0.405	3061.000
ENSG00000136205	0.158	0.148	0.523	-0.227	TNS3	0.229	~p_tau+(1|manifest)+cngeneson+pc_mito+brain_region+Sex	5.788	572.000
ENSG00000142089	0.080	0.148	0.795	-0.499	IFITM3	0.137	~p_tau+(1|manifest)+cngeneson+pc_mito+brain_region+Sex	5.631	597.000
ENSG00000134531	0.722	0.148	0.642	-0.347	EMP1	0.763	~p_tau+(1|manifest)+cngeneson+pc_mito+brain_region+Sex	18.069	67.000
ENSG00000170871	0.001	0.148	0.359	-0.063	KIAA0232	0.007	~p_tau+(1|manifest)+cngeneson+pc_mito+brain_region+Sex	1.151	2531.000
ENSG00000174099	0.013	0.148	0.353	-0.058	MSRB3	0.036	~p_tau+(1|manifest)+cngeneson+pc_mito+brain_region+Sex	1.330	2372.000
ENSG00000203780	0.000	0.148	0.356	-0.060	FANK1	0.002	~p_tau+(1|manifest)+cngeneson+pc_mito+brain_region+Sex	0.878	2758.000
ENSG00000076356	0.236	0.147	0.480	-0.185	PLXNA2	0.314	~p_tau+(1|manifest)+cngeneson+pc_mito+brain_region+Sex	5.620	601.000
ENSG00000196588	0.271	0.147	0.431	-0.136	MRTFA	0.353	~p_tau+(1|manifest)+cngeneson+pc_mito+brain_region+Sex	4.822	724.000
ENSG00000185420	0.192	0.147	0.312	-0.018	SMYD3	0.267	~p_tau+(1|manifest)+cngeneson+pc_mito+brain_region+Sex	2.854	1338.000
ENSG00000124570	0.000	0.147	0.248	0.046	SERPINB6	0.003	~p_tau+(1|manifest)+cngeneson+pc_mito+brain_region+Sex	1.753	2020.000
ENSG00000213096	0.001	0.147	0.295	-0.001	ZNF254	0.004	~p_tau+(1|manifest)+cngeneson+pc_mito+brain_region+Sex	0.732	2885.000
ENSG00000180891	0.001	0.147	0.328	-0.035	CUEDC1	0.004	~p_tau+(1|manifest)+cngeneson+pc_mito+brain_region+Sex	1.689	2076.000
ENSG00000087495	0.358	0.147	0.346	-0.053	PHACTR3	0.436	~p_tau+(1|manifest)+cngeneson+pc_mito+brain_region+Sex	14.231	116.000
ENSG00000078403	0.007	0.146	0.250	0.043	MLLT10	0.022	~p_tau+(1|manifest)+cngeneson+pc_mito+brain_region+Sex	1.588	2151.000
ENSG00000046653	0.097	0.146	0.294	-0.001	GPM6B	0.159	~p_tau+(1|manifest)+cngeneson+pc_mito+brain_region+Sex	8.280	344.000
ENSG00000128789	0.038	0.146	0.417	-0.126	PSMG2	0.077	~p_tau+(1|manifest)+cngeneson+pc_mito+brain_region+Sex	2.343	1604.000
ENSG00000133121	0.357	0.146	0.355	-0.063	STARD13	0.435	~p_tau+(1|manifest)+cngeneson+pc_mito+brain_region+Sex	4.232	849.000
ENSG00000082898	0.183	0.145	0.355	-0.065	XPO1	0.257	~p_tau+(1|manifest)+cngeneson+pc_mito+brain_region+Sex	2.301	1634.000
ENSG00000156639	0.062	0.145	0.263	0.028	ZFAND3	0.113	~p_tau+(1|manifest)+cngeneson+pc_mito+brain_region+Sex	2.500	1514.000
ENSG00000004897	0.000	0.145	0.231	0.059	CDC27	0.000	~p_tau+(1|manifest)+cngeneson+pc_mito+brain_region+Sex	1.170	2517.000
ENSG00000117643	0.848	0.145	0.750	-0.461	MAN1C1	0.868	~p_tau+(1|manifest)+cngeneson+pc_mito+brain_region+Sex	23.947	32.000
ENSG00000141068	0.002	0.145	0.309	-0.019	KSR1	0.009	~p_tau+(1|manifest)+cngeneson+pc_mito+brain_region+Sex	0.450	3045.000
ENSG00000174282	0.007	0.145	0.303	-0.014	ZBTB4	0.024	~p_tau+(1|manifest)+cngeneson+pc_mito+brain_region+Sex	2.817	1353.000
ENSG00000137942	0.001	0.144	0.305	-0.016	FNBP1L	0.005	~p_tau+(1|manifest)+cngeneson+pc_mito+brain_region+Sex	0.817	2815.000
ENSG00000137936	0.107	0.144	0.419	-0.131	BCAR3	0.171	~p_tau+(1|manifest)+cngeneson+pc_mito+brain_region+Sex	2.495	1516.000
ENSG00000162433	0.126	0.144	0.459	-0.171	AK4	0.194	~p_tau+(1|manifest)+cngeneson+pc_mito+brain_region+Sex	1.686	2078.000
ENSG00000067225	0.033	0.144	0.387	-0.099	PKM	0.070	~p_tau+(1|manifest)+cngeneson+pc_mito+brain_region+Sex	3.253	1150.000
ENSG00000127663	0.000	0.144	0.263	0.024	KDM4B	0.003	~p_tau+(1|manifest)+cngeneson+pc_mito+brain_region+Sex	1.523	2197.000
ENSG00000169851	0.614	0.143	0.437	-0.150	PCDH7	0.667	~p_tau+(1|manifest)+cngeneson+pc_mito+brain_region+Sex	5.104	685.000
ENSG00000112851	0.110	0.143	0.279	0.007	ERBIN	0.175	~p_tau+(1|manifest)+cngeneson+pc_mito+brain_region+Sex	6.988	446.000
ENSG00000054793	0.090	0.143	0.422	-0.135	ATP9A	0.150	~p_tau+(1|manifest)+cngeneson+pc_mito+brain_region+Sex	2.792	1365.000
ENSG00000124126	0.085	0.143	0.386	-0.100	PREX1	0.144	~p_tau+(1|manifest)+cngeneson+pc_mito+brain_region+Sex	3.971	918.000
ENSG00000156273	0.013	0.143	0.348	-0.061	BACH1	0.036	~p_tau+(1|manifest)+cngeneson+pc_mito+brain_region+Sex	2.185	1711.000
ENSG00000177879	0.000	0.143	0.289	-0.003	AP3S1	0.003	~p_tau+(1|manifest)+cngeneson+pc_mito+brain_region+Sex	0.082	3136.000
ENSG00000110318	0.000	0.143	0.396	-0.110	CEP126	0.001	~p_tau+(1|manifest)+cngeneson+pc_mito+brain_region+Sex	0.747	2867.000
ENSG00000115310	0.326	0.142	0.396	-0.111	RTN4	0.405	~p_tau+(1|manifest)+cngeneson+pc_mito+brain_region+Sex	6.524	483.000
ENSG00000198369	0.116	0.142	0.421	-0.136	SPRED2	0.182	~p_tau+(1|manifest)+cngeneson+pc_mito+brain_region+Sex	0.996	2655.000
ENSG00000127616	0.001	0.141	0.328	-0.047	SMARCA4	0.004	~p_tau+(1|manifest)+cngeneson+pc_mito+brain_region+Sex	2.101	1769.000
ENSG00000087884	0.000	0.141	0.240	0.041	AAMDC	0.001	~p_tau+(1|manifest)+cngeneson+pc_mito+brain_region+Sex	0.320	3100.000
ENSG00000071051	0.002	0.140	0.276	0.005	NCK2	0.009	~p_tau+(1|manifest)+cngeneson+pc_mito+brain_region+Sex	0.692	2919.000
ENSG00000148408	0.026	0.140	0.481	-0.200	CACNA1B	0.059	~p_tau+(1|manifest)+cngeneson+pc_mito+brain_region+Sex	2.793	1362.000
ENSG00000106771	0.000	0.140	0.229	0.052	TMEM245	0.000	~p_tau+(1|manifest)+cngeneson+pc_mito+brain_region+Sex	0.765	2858.000
ENSG00000173692	0.000	0.140	0.284	-0.003	PSMD1	0.003	~p_tau+(1|manifest)+cngeneson+pc_mito+brain_region+Sex	1.006	2643.000
ENSG00000177728	0.083	0.140	0.441	-0.161	TMEM94	0.142	~p_tau+(1|manifest)+cngeneson+pc_mito+brain_region+Sex	4.570	781.000
ENSG00000100836	0.297	0.140	0.616	-0.337	PABPN1	0.378	~p_tau+(1|manifest)+cngeneson+pc_mito+brain_region+Sex	12.512	163.000
ENSG00000187098	0.010	0.140	0.282	-0.003	MITF	0.029	~p_tau+(1|manifest)+cngeneson+pc_mito+brain_region+Sex	1.010	2639.000
ENSG00000131437	0.000	0.138	0.257	0.020	KIF3A	0.002	~p_tau+(1|manifest)+cngeneson+pc_mito+brain_region+Sex	0.796	2837.000
ENSG00000036257	0.000	0.138	0.293	-0.017	CUL3	0.003	~p_tau+(1|manifest)+cngeneson+pc_mito+brain_region+Sex	3.315	1129.000
ENSG00000132970	0.362	0.138	0.490	-0.214	WASF3	0.439	~p_tau+(1|manifest)+cngeneson+pc_mito+brain_region+Sex	6.255	516.000
ENSG00000101337	0.000	0.138	0.254	0.022	TM9SF4	0.001	~p_tau+(1|manifest)+cngeneson+pc_mito+brain_region+Sex	1.409	2298.000
ENSG00000101972	0.001	0.137	0.279	-0.004	STAG2	0.004	~p_tau+(1|manifest)+cngeneson+pc_mito+brain_region+Sex	0.825	2807.000
ENSG00000175309	0.018	0.137	0.367	-0.092	PHYKPL	0.045	~p_tau+(1|manifest)+cngeneson+pc_mito+brain_region+Sex	3.856	947.000
ENSG00000160710	0.001	0.137	0.295	-0.021	ADAR	0.006	~p_tau+(1|manifest)+cngeneson+pc_mito+brain_region+Sex	1.830	1963.000
ENSG00000102738	0.002	0.137	0.270	0.004	MRPS31	0.010	~p_tau+(1|manifest)+cngeneson+pc_mito+brain_region+Sex	0.497	3019.000
ENSG00000116641	0.029	0.137	0.236	0.039	DOCK7	0.063	~p_tau+(1|manifest)+cngeneson+pc_mito+brain_region+Sex	1.825	1968.000
ENSG00000104529	0.005	0.137	0.341	-0.067	EEF1D	0.018	~p_tau+(1|manifest)+cngeneson+pc_mito+brain_region+Sex	3.073	1231.000
ENSG00000152518	0.362	0.137	0.393	-0.120	ZFP36L2	0.439	~p_tau+(1|manifest)+cngeneson+pc_mito+brain_region+Sex	1.628	2117.000
ENSG00000145990	0.026	0.137	0.330	-0.056	GFOD1	0.058	~p_tau+(1|manifest)+cngeneson+pc_mito+brain_region+Sex	1.628	2116.000
ENSG00000167766	0.000	0.137	0.351	-0.078	ZNF83	0.002	~p_tau+(1|manifest)+cngeneson+pc_mito+brain_region+Sex	1.681	2079.000
ENSG00000170145	0.058	0.136	0.316	-0.043	SIK2	0.107	~p_tau+(1|manifest)+cngeneson+pc_mito+brain_region+Sex	3.152	1196.000
ENSG00000106086	0.002	0.136	0.287	-0.015	PLEKHA8	0.008	~p_tau+(1|manifest)+cngeneson+pc_mito+brain_region+Sex	0.499	3017.000
ENSG00000171456	0.000	0.136	0.293	-0.021	ASXL1	0.002	~p_tau+(1|manifest)+cngeneson+pc_mito+brain_region+Sex	1.405	2304.000
ENSG00000163406	0.324	0.136	0.524	-0.252	SLC15A2	0.403	~p_tau+(1|manifest)+cngeneson+pc_mito+brain_region+Sex	7.203	422.000
ENSG00000120690	0.007	0.135	0.322	-0.051	ELF1	0.023	~p_tau+(1|manifest)+cngeneson+pc_mito+brain_region+Sex	2.676	1416.000
ENSG00000185052	0.591	0.135	0.528	-0.258	SLC24A3	0.647	~p_tau+(1|manifest)+cngeneson+pc_mito+brain_region+Sex	3.800	967.000
ENSG00000184232	0.280	0.135	0.557	-0.287	OAF	0.361	~p_tau+(1|manifest)+cngeneson+pc_mito+brain_region+Sex	7.044	436.000
ENSG00000156298	0.202	0.135	0.466	-0.197	TSPAN7	0.278	~p_tau+(1|manifest)+cngeneson+pc_mito+brain_region+Sex	2.675	1418.000
ENSG00000149633	0.096	0.134	0.422	-0.153	KIAA1755	0.157	~p_tau+(1|manifest)+cngeneson+pc_mito+brain_region+Sex	6.594	477.000
ENSG00000168958	0.005	0.134	0.304	-0.035	MFF	0.018	~p_tau+(1|manifest)+cngeneson+pc_mito+brain_region+Sex	1.623	2124.000
ENSG00000111783	0.005	0.134	0.208	0.060	RFX4	0.017	~p_tau+(1|manifest)+cngeneson+pc_mito+brain_region+Sex	5.956	549.000
ENSG00000103091	0.000	0.134	0.281	-0.012	WDR59	0.002	~p_tau+(1|manifest)+cngeneson+pc_mito+brain_region+Sex	2.186	1710.000
ENSG00000101577	0.158	0.134	0.432	-0.164	LPIN2	0.229	~p_tau+(1|manifest)+cngeneson+pc_mito+brain_region+Sex	1.125	2551.000
ENSG00000145687	0.307	0.134	0.303	-0.035	SSBP2	0.388	~p_tau+(1|manifest)+cngeneson+pc_mito+brain_region+Sex	1.622	2125.000
ENSG00000156515	0.002	0.134	0.348	-0.080	HK1	0.010	~p_tau+(1|manifest)+cngeneson+pc_mito+brain_region+Sex	2.349	1601.000
ENSG00000047578	0.000	0.134	0.242	0.026	KIAA0556	0.001	~p_tau+(1|manifest)+cngeneson+pc_mito+brain_region+Sex	0.915	2719.000
ENSG00000078114	0.048	0.133	0.245	0.022	NEBL	0.093	~p_tau+(1|manifest)+cngeneson+pc_mito+brain_region+Sex	6.047	542.000
ENSG00000184828	0.167	0.133	0.443	-0.177	ZBTB7C	0.239	~p_tau+(1|manifest)+cngeneson+pc_mito+brain_region+Sex	3.251	1151.000
ENSG00000128487	0.115	0.133	0.464	-0.198	SPECC1	0.180	~p_tau+(1|manifest)+cngeneson+pc_mito+brain_region+Sex	2.690	1410.000
ENSG00000132155	0.001	0.133	0.246	0.020	RAF1	0.005	~p_tau+(1|manifest)+cngeneson+pc_mito+brain_region+Sex	2.033	1819.000
ENSG00000116106	0.126	0.133	0.464	-0.198	EPHA4	0.194	~p_tau+(1|manifest)+cngeneson+pc_mito+brain_region+Sex	3.782	973.000
ENSG00000005339	0.000	0.133	0.223	0.042	CREBBP	0.002	~p_tau+(1|manifest)+cngeneson+pc_mito+brain_region+Sex	0.962	2676.000
ENSG00000137269	0.062	0.133	0.343	-0.078	LRRC1	0.114	~p_tau+(1|manifest)+cngeneson+pc_mito+brain_region+Sex	1.490	2230.000
ENSG00000205268	0.030	0.132	0.342	-0.078	PDE7A	0.065	~p_tau+(1|manifest)+cngeneson+pc_mito+brain_region+Sex	2.144	1738.000
ENSG00000159023	0.017	0.132	0.333	-0.069	EPB41	0.044	~p_tau+(1|manifest)+cngeneson+pc_mito+brain_region+Sex	1.580	2154.000
ENSG00000178177	0.046	0.132	0.377	-0.113	LCORL	0.090	~p_tau+(1|manifest)+cngeneson+pc_mito+brain_region+Sex	1.405	2306.000
ENSG00000170500	0.001	0.132	0.335	-0.071	LONRF2	0.007	~p_tau+(1|manifest)+cngeneson+pc_mito+brain_region+Sex	1.330	2371.000
ENSG00000115183	0.212	0.132	0.458	-0.194	TANC1	0.288	~p_tau+(1|manifest)+cngeneson+pc_mito+brain_region+Sex	5.527	617.000
ENSG00000134108	0.000	0.132	0.290	-0.027	ARL8B	0.000	~p_tau+(1|manifest)+cngeneson+pc_mito+brain_region+Sex	0.969	2670.000
ENSG00000105926	0.275	0.132	0.404	-0.141	MPP6	0.357	~p_tau+(1|manifest)+cngeneson+pc_mito+brain_region+Sex	4.644	759.000
ENSG00000116668	0.000	0.132	0.226	0.037	SWT1	0.000	~p_tau+(1|manifest)+cngeneson+pc_mito+brain_region+Sex	0.190	3127.000
ENSG00000107372	0.064	0.131	0.346	-0.083	ZFAND5	0.116	~p_tau+(1|manifest)+cngeneson+pc_mito+brain_region+Sex	1.947	1882.000
ENSG00000108021	0.000	0.130	0.263	-0.003	TASOR2	0.002	~p_tau+(1|manifest)+cngeneson+pc_mito+brain_region+Sex	0.730	2887.000
ENSG00000028528	0.000	0.130	0.210	0.049	SNX1	0.001	~p_tau+(1|manifest)+cngeneson+pc_mito+brain_region+Sex	0.901	2735.000
ENSG00000060237	0.024	0.130	0.317	-0.058	WNK1	0.055	~p_tau+(1|manifest)+cngeneson+pc_mito+brain_region+Sex	2.306	1628.000
ENSG00000141002	0.009	0.130	0.477	-0.217	TCF25	0.028	~p_tau+(1|manifest)+cngeneson+pc_mito+brain_region+Sex	6.335	508.000
ENSG00000037637	0.004	0.129	0.307	-0.049	FBXO42	0.016	~p_tau+(1|manifest)+cngeneson+pc_mito+brain_region+Sex	0.415	3057.000
ENSG00000108510	0.004	0.129	0.264	-0.006	MED13	0.017	~p_tau+(1|manifest)+cngeneson+pc_mito+brain_region+Sex	1.617	2128.000
ENSG00000104219	0.027	0.129	0.341	-0.082	ZDHHC2	0.060	~p_tau+(1|manifest)+cngeneson+pc_mito+brain_region+Sex	2.934	1300.000
ENSG00000075391	0.290	0.129	0.391	-0.133	RASAL2	0.371	~p_tau+(1|manifest)+cngeneson+pc_mito+brain_region+Sex	6.291	511.000
ENSG00000138081	0.005	0.128	0.246	0.010	FBXO11	0.018	~p_tau+(1|manifest)+cngeneson+pc_mito+brain_region+Sex	0.645	2941.000
ENSG00000109180	0.001	0.128	0.298	-0.042	OCIAD1	0.004	~p_tau+(1|manifest)+cngeneson+pc_mito+brain_region+Sex	0.389	3069.000
ENSG00000115977	0.001	0.128	0.249	0.007	AAK1	0.007	~p_tau+(1|manifest)+cngeneson+pc_mito+brain_region+Sex	1.150	2532.000
ENSG00000124440	0.487	0.128	0.337	-0.081	HIF3A	0.555	~p_tau+(1|manifest)+cngeneson+pc_mito+brain_region+Sex	36.581	9.000
ENSG00000155897	0.264	0.128	0.356	-0.100	ADCY8	0.345	~p_tau+(1|manifest)+cngeneson+pc_mito+brain_region+Sex	4.791	732.000
ENSG00000092439	0.000	0.127	0.217	0.037	TRPM7	0.002	~p_tau+(1|manifest)+cngeneson+pc_mito+brain_region+Sex	1.149	2534.000
ENSG00000115459	0.000	0.127	0.205	0.049	ELMOD3	0.001	~p_tau+(1|manifest)+cngeneson+pc_mito+brain_region+Sex	1.281	2422.000
ENSG00000164924	0.000	0.126	0.267	-0.015	YWHAZ	0.001	~p_tau+(1|manifest)+cngeneson+pc_mito+brain_region+Sex	0.764	2859.000
ENSG00000134215	0.369	0.126	0.702	-0.450	VAV3	0.446	~p_tau+(1|manifest)+cngeneson+pc_mito+brain_region+Sex	5.796	570.000
ENSG00000072518	0.000	0.126	0.221	0.031	MARK2	0.001	~p_tau+(1|manifest)+cngeneson+pc_mito+brain_region+Sex	0.213	3122.000
ENSG00000198740	0.000	0.126	0.229	0.022	ZNF652	0.000	~p_tau+(1|manifest)+cngeneson+pc_mito+brain_region+Sex	0.192	3126.000
ENSG00000132466	0.060	0.125	0.249	0.001	ANKRD17	0.110	~p_tau+(1|manifest)+cngeneson+pc_mito+brain_region+Sex	0.797	2836.000
ENSG00000189337	0.607	0.125	0.410	-0.160	KAZN	0.661	~p_tau+(1|manifest)+cngeneson+pc_mito+brain_region+Sex	1.706	2062.000
ENSG00000140443	0.550	0.125	0.374	-0.124	IGF1R	0.612	~p_tau+(1|manifest)+cngeneson+pc_mito+brain_region+Sex	5.704	589.000
ENSG00000159788	0.101	0.125	0.373	-0.124	RGS12	0.164	~p_tau+(1|manifest)+cngeneson+pc_mito+brain_region+Sex	2.880	1325.000
ENSG00000136828	0.007	0.124	0.282	-0.035	RALGPS1	0.023	~p_tau+(1|manifest)+cngeneson+pc_mito+brain_region+Sex	1.386	2323.000
ENSG00000071242	0.129	0.124	0.269	-0.022	RPS6KA2	0.197	~p_tau+(1|manifest)+cngeneson+pc_mito+brain_region+Sex	14.459	110.000
ENSG00000083067	0.487	0.123	0.352	-0.105	TRPM3	0.555	~p_tau+(1|manifest)+cngeneson+pc_mito+brain_region+Sex	25.444	28.000
ENSG00000146243	0.009	0.123	0.364	-0.118	IRAK1BP1	0.028	~p_tau+(1|manifest)+cngeneson+pc_mito+brain_region+Sex	1.376	2332.000
ENSG00000166439	0.001	0.123	0.359	-0.112	RNF169	0.008	~p_tau+(1|manifest)+cngeneson+pc_mito+brain_region+Sex	0.633	2946.000
ENSG00000146648	0.411	0.123	0.369	-0.124	EGFR	0.486	~p_tau+(1|manifest)+cngeneson+pc_mito+brain_region+Sex	2.586	1464.000
ENSG00000172987	0.406	0.122	0.896	-0.652	HPSE2	0.482	~p_tau+(1|manifest)+cngeneson+pc_mito+brain_region+Sex	10.941	213.000
ENSG00000123552	0.000	0.122	0.206	0.038	USP45	0.000	~p_tau+(1|manifest)+cngeneson+pc_mito+brain_region+Sex	0.924	2708.000
ENSG00000138175	0.013	0.122	0.278	-0.034	ARL3	0.035	~p_tau+(1|manifest)+cngeneson+pc_mito+brain_region+Sex	3.014	1255.000
ENSG00000058272	0.006	0.122	0.230	0.014	PPP1R12A	0.021	~p_tau+(1|manifest)+cngeneson+pc_mito+brain_region+Sex	2.793	1363.000
ENSG00000116667	0.163	0.121	0.259	-0.017	C1orf21	0.235	~p_tau+(1|manifest)+cngeneson+pc_mito+brain_region+Sex	1.008	2641.000
ENSG00000108175	0.043	0.121	0.338	-0.096	ZMIZ1	0.085	~p_tau+(1|manifest)+cngeneson+pc_mito+brain_region+Sex	2.335	1607.000
ENSG00000004534	0.003	0.121	0.195	0.047	RBM6	0.012	~p_tau+(1|manifest)+cngeneson+pc_mito+brain_region+Sex	2.615	1447.000
ENSG00000168259	0.000	0.121	0.207	0.035	DNAJC7	0.000	~p_tau+(1|manifest)+cngeneson+pc_mito+brain_region+Sex	0.403	3063.000
ENSG00000065413	0.005	0.120	0.269	-0.028	ANKRD44	0.018	~p_tau+(1|manifest)+cngeneson+pc_mito+brain_region+Sex	0.550	2987.000
ENSG00000196935	0.247	0.120	0.270	-0.030	SRGAP1	0.326	~p_tau+(1|manifest)+cngeneson+pc_mito+brain_region+Sex	3.835	956.000
ENSG00000144560	0.056	0.120	0.260	-0.020	VGLL4	0.105	~p_tau+(1|manifest)+cngeneson+pc_mito+brain_region+Sex	0.380	3074.000
ENSG00000139631	0.002	0.120	0.266	-0.026	CSAD	0.010	~p_tau+(1|manifest)+cngeneson+pc_mito+brain_region+Sex	2.247	1669.000
ENSG00000179134	0.006	0.120	0.241	-0.001	SAMD4B	0.021	~p_tau+(1|manifest)+cngeneson+pc_mito+brain_region+Sex	1.439	2272.000
ENSG00000138101	0.001	0.120	0.247	-0.007	DTNB	0.004	~p_tau+(1|manifest)+cngeneson+pc_mito+brain_region+Sex	1.198	2483.000
ENSG00000140548	0.014	0.120	0.296	-0.056	ZNF710	0.037	~p_tau+(1|manifest)+cngeneson+pc_mito+brain_region+Sex	1.212	2472.000
ENSG00000184304	0.320	0.120	0.286	-0.046	PRKD1	0.399	~p_tau+(1|manifest)+cngeneson+pc_mito+brain_region+Sex	3.994	913.000
ENSG00000196498	0.073	0.120	0.359	-0.119	NCOR2	0.128	~p_tau+(1|manifest)+cngeneson+pc_mito+brain_region+Sex	2.998	1266.000
ENSG00000146067	0.008	0.120	0.273	-0.034	FAM193B	0.026	~p_tau+(1|manifest)+cngeneson+pc_mito+brain_region+Sex	2.674	1419.000
ENSG00000138303	0.000	0.120	0.253	-0.014	ASCC1	0.003	~p_tau+(1|manifest)+cngeneson+pc_mito+brain_region+Sex	0.784	2845.000
ENSG00000120008	0.189	0.119	0.318	-0.079	WDR11	0.264	~p_tau+(1|manifest)+cngeneson+pc_mito+brain_region+Sex	1.036	2616.000
ENSG00000182149	0.000	0.119	0.184	0.054	IST1	0.000	~p_tau+(1|manifest)+cngeneson+pc_mito+brain_region+Sex	0.847	2784.000
ENSG00000143603	0.486	0.118	0.315	-0.079	KCNN3	0.554	~p_tau+(1|manifest)+cngeneson+pc_mito+brain_region+Sex	14.177	118.000
ENSG00000147044	0.033	0.118	0.284	-0.048	CASK	0.070	~p_tau+(1|manifest)+cngeneson+pc_mito+brain_region+Sex	1.352	2351.000
ENSG00000118473	0.212	0.118	0.253	-0.017	SGIP1	0.288	~p_tau+(1|manifest)+cngeneson+pc_mito+brain_region+Sex	3.202	1175.000
ENSG00000165186	0.393	0.118	0.494	-0.259	PTCHD1	0.470	~p_tau+(1|manifest)+cngeneson+pc_mito+brain_region+Sex	7.027	441.000
ENSG00000153317	0.113	0.117	0.225	0.009	ASAP1	0.179	~p_tau+(1|manifest)+cngeneson+pc_mito+brain_region+Sex	2.517	1503.000
ENSG00000196814	0.105	0.116	0.358	-0.126	MVB12B	0.168	~p_tau+(1|manifest)+cngeneson+pc_mito+brain_region+Sex	2.200	1702.000
ENSG00000152936	0.855	0.116	0.529	-0.298	LMNTD1	0.875	~p_tau+(1|manifest)+cngeneson+pc_mito+brain_region+Sex	4.976	703.000
ENSG00000139910	0.179	0.115	0.278	-0.047	NOVA1	0.253	~p_tau+(1|manifest)+cngeneson+pc_mito+brain_region+Sex	3.250	1152.000
ENSG00000109689	0.243	0.115	0.389	-0.158	STIM2	0.321	~p_tau+(1|manifest)+cngeneson+pc_mito+brain_region+Sex	2.940	1295.000
ENSG00000182247	0.665	0.115	0.422	-0.192	UBE2E2	0.713	~p_tau+(1|manifest)+cngeneson+pc_mito+brain_region+Sex	3.626	1024.000
ENSG00000123983	0.041	0.115	0.268	-0.038	ACSL3	0.082	~p_tau+(1|manifest)+cngeneson+pc_mito+brain_region+Sex	1.004	2646.000
ENSG00000170558	0.306	0.115	0.289	-0.059	CDH2	0.387	~p_tau+(1|manifest)+cngeneson+pc_mito+brain_region+Sex	2.804	1356.000
ENSG00000073921	0.050	0.115	0.242	-0.012	PICALM	0.095	~p_tau+(1|manifest)+cngeneson+pc_mito+brain_region+Sex	1.809	1982.000
ENSG00000169155	0.003	0.114	0.235	-0.006	ZBTB43	0.014	~p_tau+(1|manifest)+cngeneson+pc_mito+brain_region+Sex	1.468	2247.000
ENSG00000141258	0.018	0.114	0.362	-0.134	SGSM2	0.045	~p_tau+(1|manifest)+cngeneson+pc_mito+brain_region+Sex	3.479	1063.000
ENSG00000151692	0.003	0.114	0.314	-0.086	RNF144A	0.013	~p_tau+(1|manifest)+cngeneson+pc_mito+brain_region+Sex	0.723	2893.000
ENSG00000116473	0.002	0.114	0.261	-0.034	RAP1A	0.011	~p_tau+(1|manifest)+cngeneson+pc_mito+brain_region+Sex	0.735	2881.000
ENSG00000087842	0.143	0.114	0.385	-0.158	PIR	0.212	~p_tau+(1|manifest)+cngeneson+pc_mito+brain_region+Sex	1.096	2571.000
ENSG00000197702	0.063	0.114	0.344	-0.117	PARVA	0.114	~p_tau+(1|manifest)+cngeneson+pc_mito+brain_region+Sex	1.209	2473.000
ENSG00000211455	0.001	0.113	0.225	0.002	STK38L	0.007	~p_tau+(1|manifest)+cngeneson+pc_mito+brain_region+Sex	1.100	2567.000
ENSG00000151067	0.164	0.113	0.341	-0.115	CACNA1C	0.236	~p_tau+(1|manifest)+cngeneson+pc_mito+brain_region+Sex	3.280	1143.000
ENSG00000140575	0.002	0.113	0.433	-0.207	IQGAP1	0.010	~p_tau+(1|manifest)+cngeneson+pc_mito+brain_region+Sex	2.885	1322.000
ENSG00000164548	0.012	0.113	0.272	-0.046	TRA2A	0.034	~p_tau+(1|manifest)+cngeneson+pc_mito+brain_region+Sex	2.925	1303.000
ENSG00000186591	0.415	0.112	0.402	-0.177	UBE2H	0.489	~p_tau+(1|manifest)+cngeneson+pc_mito+brain_region+Sex	3.573	1043.000
ENSG00000082996	0.000	0.112	0.212	0.012	RNF13	0.002	~p_tau+(1|manifest)+cngeneson+pc_mito+brain_region+Sex	1.618	2127.000
ENSG00000174238	0.002	0.112	0.210	0.014	PITPNA	0.008	~p_tau+(1|manifest)+cngeneson+pc_mito+brain_region+Sex	0.892	2743.000
ENSG00000163681	0.015	0.111	0.246	-0.023	SLMAP	0.038	~p_tau+(1|manifest)+cngeneson+pc_mito+brain_region+Sex	2.450	1545.000
ENSG00000102710	0.000	0.111	0.221	0.001	SUPT20H	0.003	~p_tau+(1|manifest)+cngeneson+pc_mito+brain_region+Sex	0.264	3110.000
ENSG00000140740	0.008	0.111	0.317	-0.095	UQCRC2	0.026	~p_tau+(1|manifest)+cngeneson+pc_mito+brain_region+Sex	2.949	1290.000
ENSG00000204084	0.000	0.111	0.238	-0.016	INPP5B	0.001	~p_tau+(1|manifest)+cngeneson+pc_mito+brain_region+Sex	0.676	2925.000
ENSG00000157540	0.108	0.111	0.285	-0.064	DYRK1A	0.172	~p_tau+(1|manifest)+cngeneson+pc_mito+brain_region+Sex	1.449	2262.000
ENSG00000064607	0.068	0.110	0.442	-0.222	SUGP2	0.122	~p_tau+(1|manifest)+cngeneson+pc_mito+brain_region+Sex	6.145	528.000
ENSG00000125388	0.020	0.110	0.241	-0.021	GRK4	0.048	~p_tau+(1|manifest)+cngeneson+pc_mito+brain_region+Sex	1.392	2317.000
ENSG00000167986	0.001	0.110	0.219	0.001	DDB1	0.006	~p_tau+(1|manifest)+cngeneson+pc_mito+brain_region+Sex	1.016	2633.000
ENSG00000105953	0.001	0.110	0.311	-0.091	OGDH	0.005	~p_tau+(1|manifest)+cngeneson+pc_mito+brain_region+Sex	1.998	1841.000
ENSG00000087053	0.000	0.109	0.291	-0.072	MTMR2	0.002	~p_tau+(1|manifest)+cngeneson+pc_mito+brain_region+Sex	1.348	2358.000
ENSG00000241973	0.007	0.109	0.281	-0.062	PI4KA	0.022	~p_tau+(1|manifest)+cngeneson+pc_mito+brain_region+Sex	2.952	1285.000
ENSG00000078369	0.005	0.109	0.260	-0.042	GNB1	0.018	~p_tau+(1|manifest)+cngeneson+pc_mito+brain_region+Sex	1.301	2401.000
ENSG00000177479	0.003	0.109	0.201	0.017	ARIH2	0.012	~p_tau+(1|manifest)+cngeneson+pc_mito+brain_region+Sex	1.734	2040.000
ENSG00000114573	0.001	0.109	0.230	-0.013	ATP6V1A	0.006	~p_tau+(1|manifest)+cngeneson+pc_mito+brain_region+Sex	0.878	2757.000
ENSG00000115677	0.001	0.108	0.263	-0.046	HDLBP	0.006	~p_tau+(1|manifest)+cngeneson+pc_mito+brain_region+Sex	0.844	2786.000
ENSG00000197959	0.667	0.108	0.412	-0.197	DNM3	0.714	~p_tau+(1|manifest)+cngeneson+pc_mito+brain_region+Sex	11.811	180.000
ENSG00000181790	0.113	0.108	0.534	-0.319	ADGRB1	0.179	~p_tau+(1|manifest)+cngeneson+pc_mito+brain_region+Sex	5.813	565.000
ENSG00000089876	0.001	0.108	0.284	-0.068	DHX32	0.005	~p_tau+(1|manifest)+cngeneson+pc_mito+brain_region+Sex	2.060	1794.000
ENSG00000171105	0.016	0.108	0.331	-0.116	INSR	0.042	~p_tau+(1|manifest)+cngeneson+pc_mito+brain_region+Sex	0.988	2662.000
ENSG00000135299	0.053	0.108	0.439	-0.224	ANKRD6	0.100	~p_tau+(1|manifest)+cngeneson+pc_mito+brain_region+Sex	0.398	3065.000
ENSG00000143797	0.113	0.107	0.282	-0.068	MBOAT2	0.179	~p_tau+(1|manifest)+cngeneson+pc_mito+brain_region+Sex	1.625	2121.000
ENSG00000143702	0.007	0.107	0.276	-0.062	CEP170	0.023	~p_tau+(1|manifest)+cngeneson+pc_mito+brain_region+Sex	1.951	1875.000
ENSG00000095787	0.001	0.107	0.222	-0.009	WAC	0.006	~p_tau+(1|manifest)+cngeneson+pc_mito+brain_region+Sex	2.172	1719.000
ENSG00000093167	0.005	0.106	0.228	-0.015	LRRFIP2	0.018	~p_tau+(1|manifest)+cngeneson+pc_mito+brain_region+Sex	1.136	2547.000
ENSG00000170456	0.002	0.106	0.198	0.014	DENND5B	0.011	~p_tau+(1|manifest)+cngeneson+pc_mito+brain_region+Sex	1.080	2582.000
ENSG00000103342	0.001	0.106	0.194	0.018	GSPT1	0.004	~p_tau+(1|manifest)+cngeneson+pc_mito+brain_region+Sex	0.586	2967.000
ENSG00000132478	0.001	0.105	0.234	-0.024	UNK	0.005	~p_tau+(1|manifest)+cngeneson+pc_mito+brain_region+Sex	0.833	2796.000
ENSG00000047849	0.190	0.105	0.250	-0.040	MAP4	0.264	~p_tau+(1|manifest)+cngeneson+pc_mito+brain_region+Sex	2.826	1348.000
ENSG00000134884	0.121	0.105	0.341	-0.132	ARGLU1	0.188	~p_tau+(1|manifest)+cngeneson+pc_mito+brain_region+Sex	10.626	228.000
ENSG00000149311	0.005	0.105	0.246	-0.037	ATM	0.019	~p_tau+(1|manifest)+cngeneson+pc_mito+brain_region+Sex	1.363	2345.000
ENSG00000164603	0.009	0.105	0.329	-0.120	BMT2	0.027	~p_tau+(1|manifest)+cngeneson+pc_mito+brain_region+Sex	0.866	2768.000
ENSG00000123908	0.008	0.104	0.301	-0.092	AGO2	0.025	~p_tau+(1|manifest)+cngeneson+pc_mito+brain_region+Sex	1.701	2064.000
ENSG00000135069	0.096	0.104	0.447	-0.239	PSAT1	0.157	~p_tau+(1|manifest)+cngeneson+pc_mito+brain_region+Sex	2.514	1506.000
ENSG00000164292	0.131	0.104	0.203	0.005	RHOBTB3	0.199	~p_tau+(1|manifest)+cngeneson+pc_mito+brain_region+Sex	4.128	879.000
ENSG00000114204	0.027	0.104	0.498	-0.290	SERPINI2	0.060	~p_tau+(1|manifest)+cngeneson+pc_mito+brain_region+Sex	3.856	946.000
ENSG00000137845	0.005	0.104	0.234	-0.026	ADAM10	0.018	~p_tau+(1|manifest)+cngeneson+pc_mito+brain_region+Sex	1.943	1885.000
ENSG00000046651	0.011	0.104	0.256	-0.049	OFD1	0.032	~p_tau+(1|manifest)+cngeneson+pc_mito+brain_region+Sex	0.560	2978.000
ENSG00000145246	0.003	0.104	0.191	0.017	ATP10D	0.012	~p_tau+(1|manifest)+cngeneson+pc_mito+brain_region+Sex	1.352	2353.000
ENSG00000072609	0.000	0.103	0.230	-0.023	CHFR	0.001	~p_tau+(1|manifest)+cngeneson+pc_mito+brain_region+Sex	1.042	2612.000
ENSG00000136720	0.015	0.103	0.226	-0.020	HS6ST1	0.039	~p_tau+(1|manifest)+cngeneson+pc_mito+brain_region+Sex	0.890	2744.000
ENSG00000092020	0.019	0.103	0.276	-0.069	PPP2R3C	0.047	~p_tau+(1|manifest)+cngeneson+pc_mito+brain_region+Sex	2.635	1437.000
ENSG00000066136	0.032	0.103	0.295	-0.089	NFYC	0.067	~p_tau+(1|manifest)+cngeneson+pc_mito+brain_region+Sex	1.448	2267.000
ENSG00000005483	0.002	0.102	0.227	-0.022	KMT2E	0.010	~p_tau+(1|manifest)+cngeneson+pc_mito+brain_region+Sex	0.631	2948.000
ENSG00000196367	0.002	0.102	0.254	-0.051	TRRAP	0.011	~p_tau+(1|manifest)+cngeneson+pc_mito+brain_region+Sex	1.067	2597.000
ENSG00000164466	0.006	0.102	0.236	-0.033	SFXN1	0.021	~p_tau+(1|manifest)+cngeneson+pc_mito+brain_region+Sex	1.670	2087.000
ENSG00000079335	0.279	0.102	0.327	-0.124	CDC14A	0.360	~p_tau+(1|manifest)+cngeneson+pc_mito+brain_region+Sex	0.571	2974.000
ENSG00000048828	0.045	0.101	0.230	-0.027	FAM120A	0.088	~p_tau+(1|manifest)+cngeneson+pc_mito+brain_region+Sex	2.305	1630.000
ENSG00000113742	0.324	0.101	0.231	-0.029	CPEB4	0.403	~p_tau+(1|manifest)+cngeneson+pc_mito+brain_region+Sex	4.530	789.000
ENSG00000158467	0.327	0.101	0.233	-0.032	AHCYL2	0.405	~p_tau+(1|manifest)+cngeneson+pc_mito+brain_region+Sex	3.638	1021.000
ENSG00000170085	0.002	0.101	0.240	-0.039	SIMC1	0.009	~p_tau+(1|manifest)+cngeneson+pc_mito+brain_region+Sex	0.484	3029.000
ENSG00000141367	0.000	0.100	0.204	-0.003	CLTC	0.003	~p_tau+(1|manifest)+cngeneson+pc_mito+brain_region+Sex	1.002	2649.000
ENSG00000141447	0.368	0.100	0.342	-0.142	OSBPL1A	0.445	~p_tau+(1|manifest)+cngeneson+pc_mito+brain_region+Sex	1.724	2049.000
ENSG00000177189	0.016	0.100	0.313	-0.113	RPS6KA3	0.042	~p_tau+(1|manifest)+cngeneson+pc_mito+brain_region+Sex	1.284	2420.000
ENSG00000100239	0.014	0.100	0.250	-0.051	PPP6R2	0.038	~p_tau+(1|manifest)+cngeneson+pc_mito+brain_region+Sex	2.869	1331.000
ENSG00000106723	0.244	0.099	0.319	-0.120	SPIN1	0.322	~p_tau+(1|manifest)+cngeneson+pc_mito+brain_region+Sex	2.795	1359.000
ENSG00000033627	0.000	0.099	0.268	-0.069	ATP6V0A1	0.003	~p_tau+(1|manifest)+cngeneson+pc_mito+brain_region+Sex	0.851	2778.000
ENSG00000196323	0.004	0.099	0.197	0.001	ZBTB44	0.017	~p_tau+(1|manifest)+cngeneson+pc_mito+brain_region+Sex	0.545	2991.000
ENSG00000263001	0.001	0.099	0.231	-0.032	GTF2I	0.007	~p_tau+(1|manifest)+cngeneson+pc_mito+brain_region+Sex	1.889	1923.000
ENSG00000176261	0.011	0.099	0.264	-0.066	ZBTB8OS	0.032	~p_tau+(1|manifest)+cngeneson+pc_mito+brain_region+Sex	1.304	2396.000
ENSG00000149179	0.073	0.099	0.235	-0.037	C11orf49	0.128	~p_tau+(1|manifest)+cngeneson+pc_mito+brain_region+Sex	0.679	2923.000
ENSG00000171914	0.316	0.099	0.389	-0.191	TLN2	0.395	~p_tau+(1|manifest)+cngeneson+pc_mito+brain_region+Sex	3.715	1002.000

ENSG00000054523	0.058	0.099	0.187	0.011	KIF1B	0.107	~p_tau+(1|manifest)+cngeneson+pc_mito+brain_region+Sex	7.877	363.000
ENSG00000134250	0.140	0.099	0.249	-0.051	NOTCH2	0.209	~p_tau+(1|manifest)+cngeneson+pc_mito+brain_region+Sex	1.263	2436.000
ENSG00000089057	0.001	0.098	0.245	-0.048	SLC23A2	0.006	~p_tau+(1|manifest)+cngeneson+pc_mito+brain_region+Sex	1.646	2101.000
ENSG00000187257	0.000	0.098	0.166	0.030	RSBN1L	0.001	~p_tau+(1|manifest)+cngeneson+pc_mito+brain_region+Sex	0.399	3064.000
ENSG00000169762	0.000	0.098	0.236	-0.040	TAPT1	0.002	~p_tau+(1|manifest)+cngeneson+pc_mito+brain_region+Sex	1.453	2258.000
ENSG00000069869	0.011	0.097	0.285	-0.092	NEDD4	0.031	~p_tau+(1|manifest)+cngeneson+pc_mito+brain_region+Sex	0.760	2861.000
ENSG00000083828	0.002	0.097	0.244	-0.051	ZNF586	0.009	~p_tau+(1|manifest)+cngeneson+pc_mito+brain_region+Sex	1.529	2194.000
ENSG00000153201	0.001	0.097	0.229	-0.036	RANBP2	0.007	~p_tau+(1|manifest)+cngeneson+pc_mito+brain_region+Sex	0.278	3109.000
ENSG00000018236	0.467	0.097	0.259	-0.066	CNTN1	0.536	~p_tau+(1|manifest)+cngeneson+pc_mito+brain_region+Sex	11.942	176.000
ENSG00000064042	0.310	0.096	0.361	-0.169	LIMCH1	0.390	~p_tau+(1|manifest)+cngeneson+pc_mito+brain_region+Sex	3.053	1242.000
ENSG00000118946	0.270	0.096	0.378	-0.185	PCDH17	0.352	~p_tau+(1|manifest)+cngeneson+pc_mito+brain_region+Sex	1.327	2373.000
ENSG00000164089	0.850	0.096	0.455	-0.263	ETNPPL	0.871	~p_tau+(1|manifest)+cngeneson+pc_mito+brain_region+Sex	19.565	55.000
ENSG00000134852	0.017	0.096	0.301	-0.109	CLOCK	0.042	~p_tau+(1|manifest)+cngeneson+pc_mito+brain_region+Sex	2.517	1499.000
ENSG00000172992	0.002	0.096	0.269	-0.077	DCAKD	0.009	~p_tau+(1|manifest)+cngeneson+pc_mito+brain_region+Sex	1.713	2055.000
ENSG00000151491	0.129	0.096	0.242	-0.051	EPS8	0.197	~p_tau+(1|manifest)+cngeneson+pc_mito+brain_region+Sex	0.472	3034.000
ENSG00000137073	0.020	0.095	0.218	-0.027	UBAP2	0.048	~p_tau+(1|manifest)+cngeneson+pc_mito+brain_region+Sex	1.243	2445.000
ENSG00000106459	0.034	0.095	0.241	-0.050	NRF1	0.071	~p_tau+(1|manifest)+cngeneson+pc_mito+brain_region+Sex	1.704	2063.000
ENSG00000162437	0.086	0.095	0.295	-0.104	RAVER2	0.144	~p_tau+(1|manifest)+cngeneson+pc_mito+brain_region+Sex	1.391	2319.000
ENSG00000244754	0.079	0.095	0.176	0.015	N4BP2L2	0.136	~p_tau+(1|manifest)+cngeneson+pc_mito+brain_region+Sex	1.068	2596.000
ENSG00000135525	0.414	0.095	0.407	-0.218	MAP7	0.489	~p_tau+(1|manifest)+cngeneson+pc_mito+brain_region+Sex	3.887	933.000
ENSG00000127124	0.272	0.095	0.373	-0.184	HIVEP3	0.353	~p_tau+(1|manifest)+cngeneson+pc_mito+brain_region+Sex	5.369	640.000
ENSG00000010810	0.182	0.095	0.236	-0.047	FYN	0.257	~p_tau+(1|manifest)+cngeneson+pc_mito+brain_region+Sex	6.358	503.000
ENSG00000001629	0.011	0.094	0.223	-0.034	ANKIB1	0.032	~p_tau+(1|manifest)+cngeneson+pc_mito+brain_region+Sex	0.864	2771.000
ENSG00000100201	0.075	0.094	0.175	0.014	DDX17	0.130	~p_tau+(1|manifest)+cngeneson+pc_mito+brain_region+Sex	10.032	259.000
ENSG00000121879	0.085	0.094	0.306	-0.117	PIK3CA	0.144	~p_tau+(1|manifest)+cngeneson+pc_mito+brain_region+Sex	1.182	2504.000
ENSG00000112992	0.002	0.094	0.183	0.005	NNT	0.008	~p_tau+(1|manifest)+cngeneson+pc_mito+brain_region+Sex	1.627	2118.000
ENSG00000196535	0.005	0.094	0.322	-0.134	MYO18A	0.018	~p_tau+(1|manifest)+cngeneson+pc_mito+brain_region+Sex	3.435	1079.000
ENSG00000145675	0.483	0.094	0.432	-0.245	PIK3R1	0.551	~p_tau+(1|manifest)+cngeneson+pc_mito+brain_region+Sex	2.514	1507.000
ENSG00000171612	0.242	0.093	0.382	-0.195	SLC25A33	0.321	~p_tau+(1|manifest)+cngeneson+pc_mito+brain_region+Sex	3.202	1177.000
ENSG00000003756	0.006	0.093	0.228	-0.041	RBM5	0.021	~p_tau+(1|manifest)+cngeneson+pc_mito+brain_region+Sex	2.723	1390.000
ENSG00000025293	0.000	0.093	0.182	0.004	PHF20	0.000	~p_tau+(1|manifest)+cngeneson+pc_mito+brain_region+Sex	0.320	3099.000
ENSG00000072736	0.002	0.093	0.199	-0.013	NFATC3	0.009	~p_tau+(1|manifest)+cngeneson+pc_mito+brain_region+Sex	0.739	2877.000
ENSG00000144857	0.058	0.093	0.312	-0.125	BOC	0.108	~p_tau+(1|manifest)+cngeneson+pc_mito+brain_region+Sex	0.208	3125.000
ENSG00000101004	0.022	0.093	0.296	-0.110	NINL	0.052	~p_tau+(1|manifest)+cngeneson+pc_mito+brain_region+Sex	3.752	990.000
ENSG00000163285	0.741	0.093	0.516	-0.330	GABRG1	0.778	~p_tau+(1|manifest)+cngeneson+pc_mito+brain_region+Sex	8.402	331.000
ENSG00000110344	0.003	0.092	0.192	-0.007	UBE4A	0.012	~p_tau+(1|manifest)+cngeneson+pc_mito+brain_region+Sex	0.810	2823.000
ENSG00000180957	0.000	0.092	0.186	-0.002	PITPNB	0.001	~p_tau+(1|manifest)+cngeneson+pc_mito+brain_region+Sex	0.560	2979.000
ENSG00000158636	0.001	0.092	0.253	-0.068	EMSY	0.008	~p_tau+(1|manifest)+cngeneson+pc_mito+brain_region+Sex	2.050	1802.000
ENSG00000099954	0.423	0.092	0.357	-0.173	CECR2	0.498	~p_tau+(1|manifest)+cngeneson+pc_mito+brain_region+Sex	1.180	2507.000
ENSG00000005379	0.077	0.092	0.515	-0.332	TSPOAP1	0.133	~p_tau+(1|manifest)+cngeneson+pc_mito+brain_region+Sex	7.243	420.000
ENSG00000105856	0.000	0.091	0.237	-0.054	HBP1	0.000	~p_tau+(1|manifest)+cngeneson+pc_mito+brain_region+Sex	0.921	2712.000
ENSG00000168461	0.272	0.091	0.328	-0.146	RAB31	0.353	~p_tau+(1|manifest)+cngeneson+pc_mito+brain_region+Sex	1.769	2002.000
ENSG00000035403	0.386	0.091	0.417	-0.235	VCL	0.463	~p_tau+(1|manifest)+cngeneson+pc_mito+brain_region+Sex	8.501	323.000
ENSG00000011566	0.007	0.091	0.173	0.009	MAP4K3	0.024	~p_tau+(1|manifest)+cngeneson+pc_mito+brain_region+Sex	3.354	1113.000
ENSG00000100354	0.079	0.091	0.168	0.013	TNRC6B	0.137	~p_tau+(1|manifest)+cngeneson+pc_mito+brain_region+Sex	1.616	2129.000
ENSG00000020577	0.856	0.091	0.410	-0.229	SAMD4A	0.876	~p_tau+(1|manifest)+cngeneson+pc_mito+brain_region+Sex	17.771	70.000
ENSG00000172766	0.002	0.091	0.264	-0.083	NAA16	0.009	~p_tau+(1|manifest)+cngeneson+pc_mito+brain_region+Sex	0.377	3076.000
ENSG00000077585	0.012	0.091	0.310	-0.128	GPR137B	0.033	~p_tau+(1|manifest)+cngeneson+pc_mito+brain_region+Sex	0.996	2654.000
ENSG00000186815	0.824	0.091	0.675	-0.493	TPCN1	0.848	~p_tau+(1|manifest)+cngeneson+pc_mito+brain_region+Sex	22.911	37.000
ENSG00000130414	0.001	0.090	0.185	-0.004	NDUFA10	0.004	~p_tau+(1|manifest)+cngeneson+pc_mito+brain_region+Sex	1.161	2522.000
ENSG00000131507	0.005	0.090	0.206	-0.025	NDFIP1	0.017	~p_tau+(1|manifest)+cngeneson+pc_mito+brain_region+Sex	2.007	1834.000
ENSG00000112079	0.002	0.090	0.225	-0.045	STK38	0.009	~p_tau+(1|manifest)+cngeneson+pc_mito+brain_region+Sex	0.893	2741.000
ENSG00000139971	0.099	0.090	0.264	-0.085	ARMH4	0.161	~p_tau+(1|manifest)+cngeneson+pc_mito+brain_region+Sex	3.151	1197.000
ENSG00000124831	0.022	0.090	0.278	-0.099	LRRFIP1	0.052	~p_tau+(1|manifest)+cngeneson+pc_mito+brain_region+Sex	2.118	1761.000
ENSG00000250312	0.022	0.090	0.234	-0.055	ZNF718	0.052	~p_tau+(1|manifest)+cngeneson+pc_mito+brain_region+Sex	2.306	1629.000
ENSG00000019144	0.182	0.089	0.337	-0.158	PHLDB1	0.257	~p_tau+(1|manifest)+cngeneson+pc_mito+brain_region+Sex	8.508	322.000
ENSG00000088367	0.006	0.089	0.322	-0.144	EPB41L1	0.020	~p_tau+(1|manifest)+cngeneson+pc_mito+brain_region+Sex	1.490	2228.000
ENSG00000132676	0.246	0.088	0.415	-0.239	DAP3	0.324	~p_tau+(1|manifest)+cngeneson+pc_mito+brain_region+Sex	7.596	384.000
ENSG00000131508	0.000	0.088	0.229	-0.053	UBE2D2	0.000	~p_tau+(1|manifest)+cngeneson+pc_mito+brain_region+Sex	0.258	3113.000
ENSG00000152818	0.214	0.088	0.216	-0.041	UTRN	0.290	~p_tau+(1|manifest)+cngeneson+pc_mito+brain_region+Sex	3.609	1034.000
ENSG00000171798	0.139	0.087	0.558	-0.383	KNDC1	0.209	~p_tau+(1|manifest)+cngeneson+pc_mito+brain_region+Sex	8.433	328.000
ENSG00000270647	0.096	0.087	0.237	-0.063	TAF15	0.158	~p_tau+(1|manifest)+cngeneson+pc_mito+brain_region+Sex	5.813	566.000
ENSG00000172667	0.303	0.087	0.383	-0.209	ZMAT3	0.384	~p_tau+(1|manifest)+cngeneson+pc_mito+brain_region+Sex	2.473	1535.000
ENSG00000128829	0.001	0.087	0.214	-0.041	EIF2AK4	0.005	~p_tau+(1|manifest)+cngeneson+pc_mito+brain_region+Sex	0.331	3095.000
ENSG00000099204	0.064	0.087	0.156	0.017	ABLIM1	0.116	~p_tau+(1|manifest)+cngeneson+pc_mito+brain_region+Sex	0.358	3084.000
ENSG00000163166	0.002	0.086	0.229	-0.057	IWS1	0.008	~p_tau+(1|manifest)+cngeneson+pc_mito+brain_region+Sex	1.412	2296.000
ENSG00000170310	0.002	0.086	0.189	-0.017	STX8	0.009	~p_tau+(1|manifest)+cngeneson+pc_mito+brain_region+Sex	0.760	2862.000
ENSG00000118689	0.603	0.086	0.282	-0.110	FOXO3	0.658	~p_tau+(1|manifest)+cngeneson+pc_mito+brain_region+Sex	2.293	1640.000
ENSG00000173120	0.003	0.086	0.223	-0.051	KDM2A	0.012	~p_tau+(1|manifest)+cngeneson+pc_mito+brain_region+Sex	1.415	2294.000
ENSG00000129933	0.003	0.086	0.225	-0.054	MAU2	0.012	~p_tau+(1|manifest)+cngeneson+pc_mito+brain_region+Sex	1.956	1874.000
ENSG00000221978	0.173	0.086	0.441	-0.270	CCNL2	0.247	~p_tau+(1|manifest)+cngeneson+pc_mito+brain_region+Sex	8.929	309.000
ENSG00000198431	0.129	0.085	0.324	-0.153	TXNRD1	0.197	~p_tau+(1|manifest)+cngeneson+pc_mito+brain_region+Sex	3.456	1070.000
ENSG00000145016	0.017	0.085	0.214	-0.044	RUBCN	0.042	~p_tau+(1|manifest)+cngeneson+pc_mito+brain_region+Sex	1.408	2299.000
ENSG00000133424	0.027	0.085	0.250	-0.080	LARGE1	0.060	~p_tau+(1|manifest)+cngeneson+pc_mito+brain_region+Sex	0.782	2847.000
ENSG00000107863	0.093	0.085	0.184	-0.015	ARHGAP21	0.153	~p_tau+(1|manifest)+cngeneson+pc_mito+brain_region+Sex	2.517	1498.000
ENSG00000109762	0.003	0.085	0.209	-0.040	SNX25	0.012	~p_tau+(1|manifest)+cngeneson+pc_mito+brain_region+Sex	1.101	2566.000
ENSG00000124789	0.000	0.085	0.206	-0.037	NUP153	0.001	~p_tau+(1|manifest)+cngeneson+pc_mito+brain_region+Sex	1.235	2450.000
ENSG00000008277	0.014	0.084	0.233	-0.065	ADAM22	0.037	~p_tau+(1|manifest)+cngeneson+pc_mito+brain_region+Sex	1.614	2134.000
ENSG00000117139	0.000	0.084	0.182	-0.015	KDM5B	0.002	~p_tau+(1|manifest)+cngeneson+pc_mito+brain_region+Sex	1.509	2209.000
ENSG00000111790	0.002	0.084	0.195	-0.027	FGFR1OP2	0.008	~p_tau+(1|manifest)+cngeneson+pc_mito+brain_region+Sex	0.805	2829.000
ENSG00000114331	0.435	0.084	0.324	-0.157	ACAP2	0.507	~p_tau+(1|manifest)+cngeneson+pc_mito+brain_region+Sex	7.280	412.000
ENSG00000162733	0.525	0.083	0.587	-0.420	DDR2	0.590	~p_tau+(1|manifest)+cngeneson+pc_mito+brain_region+Sex	8.027	354.000
ENSG00000186951	0.177	0.083	0.336	-0.169	PPARA	0.251	~p_tau+(1|manifest)+cngeneson+pc_mito+brain_region+Sex	4.400	820.000
ENSG00000115306	0.227	0.083	0.181	-0.015	SPTBN1	0.303	~p_tau+(1|manifest)+cngeneson+pc_mito+brain_region+Sex	2.560	1478.000
ENSG00000091436	0.127	0.083	0.318	-0.151	MAP3K20	0.194	~p_tau+(1|manifest)+cngeneson+pc_mito+brain_region+Sex	1.977	1861.000
ENSG00000185630	0.716	0.083	0.296	-0.130	PBX1	0.759	~p_tau+(1|manifest)+cngeneson+pc_mito+brain_region+Sex	11.985	175.000
ENSG00000079739	0.026	0.083	0.267	-0.101	PGM1	0.058	~p_tau+(1|manifest)+cngeneson+pc_mito+brain_region+Sex	1.222	2464.000
ENSG00000151693	0.124	0.083	0.248	-0.082	ASAP2	0.191	~p_tau+(1|manifest)+cngeneson+pc_mito+brain_region+Sex	2.304	1632.000
ENSG00000272886	0.000	0.083	0.194	-0.029	DCP1A	0.003	~p_tau+(1|manifest)+cngeneson+pc_mito+brain_region+Sex	1.171	2516.000
ENSG00000107104	0.368	0.083	0.196	-0.031	KANK1	0.445	~p_tau+(1|manifest)+cngeneson+pc_mito+brain_region+Sex	2.567	1474.000
ENSG00000147799	0.093	0.082	0.411	-0.247	ARHGAP39	0.153	~p_tau+(1|manifest)+cngeneson+pc_mito+brain_region+Sex	4.602	769.000
ENSG00000120254	0.198	0.082	0.400	-0.236	MTHFD1L	0.274	~p_tau+(1|manifest)+cngeneson+pc_mito+brain_region+Sex	3.197	1178.000
ENSG00000122786	0.072	0.082	0.468	-0.304	CALD1	0.128	~p_tau+(1|manifest)+cngeneson+pc_mito+brain_region+Sex	0.348	3088.000
ENSG00000070413	0.016	0.082	0.189	-0.026	DGCR2	0.041	~p_tau+(1|manifest)+cngeneson+pc_mito+brain_region+Sex	1.006	2642.000
ENSG00000115839	0.002	0.082	0.226	-0.063	RAB3GAP1	0.008	~p_tau+(1|manifest)+cngeneson+pc_mito+brain_region+Sex	0.363	3079.000
ENSG00000185305	0.206	0.082	0.215	-0.052	ARL15	0.281	~p_tau+(1|manifest)+cngeneson+pc_mito+brain_region+Sex	2.528	1489.000
ENSG00000008086	0.009	0.081	0.307	-0.144	CDKL5	0.028	~p_tau+(1|manifest)+cngeneson+pc_mito+brain_region+Sex	0.591	2964.000
ENSG00000171681	0.018	0.081	0.303	-0.141	ATF7IP	0.045	~p_tau+(1|manifest)+cngeneson+pc_mito+brain_region+Sex	0.359	3083.000
ENSG00000138434	0.140	0.081	0.310	-0.149	ITPRID2	0.209	~p_tau+(1|manifest)+cngeneson+pc_mito+brain_region+Sex	4.440	805.000
ENSG00000170271	0.012	0.080	0.395	-0.234	FAXDC2	0.033	~p_tau+(1|manifest)+cngeneson+pc_mito+brain_region+Sex	3.176	1188.000
ENSG00000175175	0.237	0.080	0.323	-0.163	PPM1E	0.315	~p_tau+(1|manifest)+cngeneson+pc_mito+brain_region+Sex	0.703	2913.000
ENSG00000147421	0.729	0.080	0.379	-0.219	HMBOX1	0.770	~p_tau+(1|manifest)+cngeneson+pc_mito+brain_region+Sex	13.533	132.000
ENSG00000134698	0.000	0.080	0.188	-0.028	AGO4	0.003	~p_tau+(1|manifest)+cngeneson+pc_mito+brain_region+Sex	0.651	2939.000
ENSG00000065883	0.007	0.080	0.161	-0.001	CDK13	0.023	~p_tau+(1|manifest)+cngeneson+pc_mito+brain_region+Sex	1.817	1976.000
ENSG00000109911	0.566	0.080	0.322	-0.162	ELP4	0.626	~p_tau+(1|manifest)+cngeneson+pc_mito+brain_region+Sex	2.719	1392.000
ENSG00000156313	0.000	0.080	0.289	-0.129	RPGR	0.001	~p_tau+(1|manifest)+cngeneson+pc_mito+brain_region+Sex	0.898	2736.000
ENSG00000121742	0.046	0.080	0.403	-0.244	GJB6	0.089	~p_tau+(1|manifest)+cngeneson+pc_mito+brain_region+Sex	2.284	1650.000
ENSG00000136861	0.005	0.079	0.194	-0.036	CDK5RAP2	0.019	~p_tau+(1|manifest)+cngeneson+pc_mito+brain_region+Sex	1.966	1870.000
ENSG00000115170	0.028	0.078	0.235	-0.079	ACVR1	0.062	~p_tau+(1|manifest)+cngeneson+pc_mito+brain_region+Sex	1.896	1917.000
ENSG00000163539	0.482	0.078	0.265	-0.108	CLASP2	0.550	~p_tau+(1|manifest)+cngeneson+pc_mito+brain_region+Sex	7.927	359.000
ENSG00000135144	0.260	0.078	0.371	-0.216	DTX1	0.341	~p_tau+(1|manifest)+cngeneson+pc_mito+brain_region+Sex	4.690	752.000
ENSG00000024048	0.005	0.078	0.181	-0.025	UBR2	0.018	~p_tau+(1|manifest)+cngeneson+pc_mito+brain_region+Sex	1.428	2281.000
ENSG00000179532	0.222	0.078	0.364	-0.209	DNHD1	0.300	~p_tau+(1|manifest)+cngeneson+pc_mito+brain_region+Sex	3.768	984.000
ENSG00000113441	0.004	0.077	0.190	-0.035	LNPEP	0.015	~p_tau+(1|manifest)+cngeneson+pc_mito+brain_region+Sex	1.030	2620.000
ENSG00000057935	0.007	0.077	0.184	-0.029	MTA3	0.023	~p_tau+(1|manifest)+cngeneson+pc_mito+brain_region+Sex	0.655	2934.000
ENSG00000135365	0.419	0.077	0.216	-0.061	PHF21A	0.493	~p_tau+(1|manifest)+cngeneson+pc_mito+brain_region+Sex	1.537	2185.000
ENSG00000162521	0.001	0.077	0.175	-0.021	RBBP4	0.005	~p_tau+(1|manifest)+cngeneson+pc_mito+brain_region+Sex	0.182	3129.000
ENSG00000060762	0.015	0.077	0.250	-0.096	MPC1	0.038	~p_tau+(1|manifest)+cngeneson+pc_mito+brain_region+Sex	1.634	2112.000
ENSG00000064393	0.117	0.077	0.224	-0.070	HIPK2	0.183	~p_tau+(1|manifest)+cngeneson+pc_mito+brain_region+Sex	1.884	1926.000
ENSG00000196428	0.187	0.077	0.222	-0.068	TSC22D2	0.262	~p_tau+(1|manifest)+cngeneson+pc_mito+brain_region+Sex	0.360	3082.000
ENSG00000156170	0.006	0.077	0.237	-0.084	NDUFAF6	0.020	~p_tau+(1|manifest)+cngeneson+pc_mito+brain_region+Sex	1.744	2027.000
ENSG00000101191	0.000	0.077	0.198	-0.045	DIDO1	0.003	~p_tau+(1|manifest)+cngeneson+pc_mito+brain_region+Sex	1.726	2047.000
ENSG00000171843	0.399	0.076	0.320	-0.167	MLLT3	0.475	~p_tau+(1|manifest)+cngeneson+pc_mito+brain_region+Sex	1.837	1958.000
ENSG00000099991	0.049	0.076	0.263	-0.110	CABIN1	0.095	~p_tau+(1|manifest)+cngeneson+pc_mito+brain_region+Sex	3.415	1093.000
ENSG00000115419	0.017	0.076	0.270	-0.117	GLS	0.042	~p_tau+(1|manifest)+cngeneson+pc_mito+brain_region+Sex	2.036	1814.000
ENSG00000100813	0.000	0.076	0.171	-0.019	ACIN1	0.003	~p_tau+(1|manifest)+cngeneson+pc_mito+brain_region+Sex	1.079	2585.000
ENSG00000189350	0.139	0.076	0.509	-0.358	TOGARAM2	0.209	~p_tau+(1|manifest)+cngeneson+pc_mito+brain_region+Sex	6.084	538.000
ENSG00000118322	0.078	0.076	0.270	-0.118	ATP10B	0.135	~p_tau+(1|manifest)+cngeneson+pc_mito+brain_region+Sex	1.736	2038.000
ENSG00000128881	0.004	0.076	0.193	-0.041	TTBK2	0.017	~p_tau+(1|manifest)+cngeneson+pc_mito+brain_region+Sex	1.894	1919.000
ENSG00000110696	0.003	0.076	0.208	-0.057	C11orf58	0.012	~p_tau+(1|manifest)+cngeneson+pc_mito+brain_region+Sex	0.917	2716.000
ENSG00000203667	0.010	0.075	0.235	-0.085	COX20	0.029	~p_tau+(1|manifest)+cngeneson+pc_mito+brain_region+Sex	2.032	1820.000
ENSG00000119938	0.531	0.075	0.341	-0.190	PPP1R3C	0.595	~p_tau+(1|manifest)+cngeneson+pc_mito+brain_region+Sex	1.934	1890.000
ENSG00000143624	0.017	0.075	0.213	-0.063	INTS3	0.043	~p_tau+(1|manifest)+cngeneson+pc_mito+brain_region+Sex	2.963	1280.000
ENSG00000146414	0.015	0.075	0.233	-0.082	SHPRH	0.040	~p_tau+(1|manifest)+cngeneson+pc_mito+brain_region+Sex	0.923	2709.000
ENSG00000150907	0.439	0.075	0.212	-0.062	FOXO1	0.512	~p_tau+(1|manifest)+cngeneson+pc_mito+brain_region+Sex	0.935	2701.000
ENSG00000110844	0.008	0.075	0.197	-0.047	PRPF40B	0.026	~p_tau+(1|manifest)+cngeneson+pc_mito+brain_region+Sex	1.366	2343.000
ENSG00000103740	0.649	0.075	0.372	-0.222	ACSBG1	0.699	~p_tau+(1|manifest)+cngeneson+pc_mito+brain_region+Sex	9.548	282.000
ENSG00000177463	0.001	0.075	0.197	-0.048	NR2C2	0.004	~p_tau+(1|manifest)+cngeneson+pc_mito+brain_region+Sex	0.919	2714.000
ENSG00000102908	0.484	0.074	0.196	-0.047	NFAT5	0.552	~p_tau+(1|manifest)+cngeneson+pc_mito+brain_region+Sex	3.519	1056.000
ENSG00000172728	0.318	0.074	0.347	-0.199	FUT10	0.397	~p_tau+(1|manifest)+cngeneson+pc_mito+brain_region+Sex	2.571	1471.000
ENSG00000140718	0.030	0.073	0.136	0.010	FTO	0.065	~p_tau+(1|manifest)+cngeneson+pc_mito+brain_region+Sex	0.222	3119.000
ENSG00000004864	0.008	0.073	0.182	-0.036	SLC25A13	0.026	~p_tau+(1|manifest)+cngeneson+pc_mito+brain_region+Sex	1.658	2092.000
ENSG00000153814	0.288	0.073	0.315	-0.169	JAZF1	0.369	~p_tau+(1|manifest)+cngeneson+pc_mito+brain_region+Sex	1.509	2208.000
ENSG00000102753	0.002	0.073	0.156	-0.009	KPNA3	0.010	~p_tau+(1|manifest)+cngeneson+pc_mito+brain_region+Sex	1.895	1918.000
ENSG00000172493	0.714	0.073	0.266	-0.120	AFF1	0.757	~p_tau+(1|manifest)+cngeneson+pc_mito+brain_region+Sex	4.092	887.000
ENSG00000018625	0.732	0.073	0.253	-0.107	ATP1A2	0.771	~p_tau+(1|manifest)+cngeneson+pc_mito+brain_region+Sex	17.239	74.000
ENSG00000169439	0.382	0.073	0.280	-0.135	SDC2	0.460	~p_tau+(1|manifest)+cngeneson+pc_mito+brain_region+Sex	0.704	2911.000
ENSG00000164983	0.017	0.073	0.216	-0.070	TMEM65	0.043	~p_tau+(1|manifest)+cngeneson+pc_mito+brain_region+Sex	1.175	2511.000
ENSG00000155111	0.041	0.072	0.199	-0.054	CDK19	0.082	~p_tau+(1|manifest)+cngeneson+pc_mito+brain_region+Sex	0.216	3121.000
ENSG00000172379	0.077	0.072	0.181	-0.037	ARNT2	0.133	~p_tau+(1|manifest)+cngeneson+pc_mito+brain_region+Sex	1.104	2563.000
ENSG00000145349	0.743	0.072	0.331	-0.187	CAMK2D	0.780	~p_tau+(1|manifest)+cngeneson+pc_mito+brain_region+Sex	3.411	1094.000
ENSG00000100154	0.175	0.072	0.146	-0.002	TTC28	0.249	~p_tau+(1|manifest)+cngeneson+pc_mito+brain_region+Sex	2.339	1606.000
ENSG00000119638	0.008	0.072	0.189	-0.045	NEK9	0.026	~p_tau+(1|manifest)+cngeneson+pc_mito+brain_region+Sex	2.010	1831.000
ENSG00000134352	0.227	0.072	0.309	-0.166	IL6ST	0.304	~p_tau+(1|manifest)+cngeneson+pc_mito+brain_region+Sex	1.549	2170.000
ENSG00000124788	0.407	0.071	0.195	-0.052	ATXN1	0.483	~p_tau+(1|manifest)+cngeneson+pc_mito+brain_region+Sex	1.073	2591.000
ENSG00000109452	0.721	0.071	0.440	-0.298	INPP4B	0.763	~p_tau+(1|manifest)+cngeneson+pc_mito+brain_region+Sex	0.781	2848.000
ENSG00000031003	0.002	0.071	0.157	-0.014	FAM13B	0.011	~p_tau+(1|manifest)+cngeneson+pc_mito+brain_region+Sex	1.937	1887.000
ENSG00000126777	0.010	0.071	0.231	-0.088	KTN1	0.029	~p_tau+(1|manifest)+cngeneson+pc_mito+brain_region+Sex	0.547	2989.000
ENSG00000214402	0.635	0.071	0.901	-0.759	LCNL1	0.686	~p_tau+(1|manifest)+cngeneson+pc_mito+brain_region+Sex	9.495	286.000
ENSG00000141252	0.003	0.071	0.255	-0.113	VPS53	0.013	~p_tau+(1|manifest)+cngeneson+pc_mito+brain_region+Sex	4.386	823.000
ENSG00000203666	0.284	0.071	0.355	-0.213	EFCAB2	0.366	~p_tau+(1|manifest)+cngeneson+pc_mito+brain_region+Sex	1.520	2200.000
ENSG00000158321	0.290	0.071	0.175	-0.033	AUTS2	0.371	~p_tau+(1|manifest)+cngeneson+pc_mito+brain_region+Sex	4.045	899.000
ENSG00000109184	0.002	0.070	0.185	-0.044	DCUN1D4	0.010	~p_tau+(1|manifest)+cngeneson+pc_mito+brain_region+Sex	1.636	2109.000
ENSG00000125266	0.019	0.070	0.229	-0.089	EFNB2	0.046	~p_tau+(1|manifest)+cngeneson+pc_mito+brain_region+Sex	1.599	2144.000
ENSG00000100852	0.450	0.070	0.184	-0.043	ARHGAP5	0.522	~p_tau+(1|manifest)+cngeneson+pc_mito+brain_region+Sex	4.276	835.000
ENSG00000155744	0.003	0.069	0.177	-0.038	FAM126B	0.012	~p_tau+(1|manifest)+cngeneson+pc_mito+brain_region+Sex	0.621	2954.000
ENSG00000138594	0.000	0.069	0.133	0.006	TMOD3	0.001	~p_tau+(1|manifest)+cngeneson+pc_mito+brain_region+Sex	0.662	2930.000
ENSG00000147202	0.444	0.069	0.247	-0.108	DIAPH2	0.517	~p_tau+(1|manifest)+cngeneson+pc_mito+brain_region+Sex	0.457	3042.000
ENSG00000196914	0.409	0.069	0.169	-0.031	ARHGEF12	0.485	~p_tau+(1|manifest)+cngeneson+pc_mito+brain_region+Sex	1.985	1851.000
ENSG00000134759	0.006	0.069	0.179	-0.040	ELP2	0.020	~p_tau+(1|manifest)+cngeneson+pc_mito+brain_region+Sex	0.669	2928.000
ENSG00000135951	0.065	0.069	0.214	-0.076	TSGA10	0.117	~p_tau+(1|manifest)+cngeneson+pc_mito+brain_region+Sex	1.821	1971.000
ENSG00000115524	0.025	0.069	0.233	-0.096	SF3B1	0.057	~p_tau+(1|manifest)+cngeneson+pc_mito+brain_region+Sex	3.249	1153.000
ENSG00000158985	0.012	0.069	0.154	-0.017	CDC42SE2	0.033	~p_tau+(1|manifest)+cngeneson+pc_mito+brain_region+Sex	0.818	2814.000
ENSG00000166326	0.013	0.068	0.222	-0.085	TRIM44	0.036	~p_tau+(1|manifest)+cngeneson+pc_mito+brain_region+Sex	0.487	3026.000
ENSG00000146555	0.719	0.068	0.336	-0.199	SDK1	0.761	~p_tau+(1|manifest)+cngeneson+pc_mito+brain_region+Sex	3.112	1213.000
ENSG00000137266	0.091	0.068	0.275	-0.139	SLC22A23	0.151	~p_tau+(1|manifest)+cngeneson+pc_mito+brain_region+Sex	3.358	1112.000
ENSG00000108395	0.001	0.068	0.150	-0.014	TRIM37	0.004	~p_tau+(1|manifest)+cngeneson+pc_mito+brain_region+Sex	0.890	2745.000
ENSG00000112419	0.001	0.068	0.214	-0.078	PHACTR2	0.005	~p_tau+(1|manifest)+cngeneson+pc_mito+brain_region+Sex	0.237	3117.000
ENSG00000142611	0.552	0.068	0.206	-0.070	PRDM16	0.612	~p_tau+(1|manifest)+cngeneson+pc_mito+brain_region+Sex	4.318	833.000
ENSG00000078140	0.007	0.067	0.206	-0.071	UBE2K	0.023	~p_tau+(1|manifest)+cngeneson+pc_mito+brain_region+Sex	1.502	2217.000
ENSG00000089048	0.011	0.067	0.177	-0.043	ESF1	0.031	~p_tau+(1|manifest)+cngeneson+pc_mito+brain_region+Sex	1.267	2435.000
ENSG00000047346	0.160	0.067	0.318	-0.185	FAM214A	0.232	~p_tau+(1|manifest)+cngeneson+pc_mito+brain_region+Sex	1.388	2321.000
ENSG00000109790	0.041	0.067	0.198	-0.065	KLHL5	0.082	~p_tau+(1|manifest)+cngeneson+pc_mito+brain_region+Sex	1.191	2496.000
ENSG00000065534	0.070	0.067	0.339	-0.206	MYLK	0.124	~p_tau+(1|manifest)+cngeneson+pc_mito+brain_region+Sex	2.319	1620.000
ENSG00000102580	0.021	0.067	0.299	-0.166	DNAJC3	0.050	~p_tau+(1|manifest)+cngeneson+pc_mito+brain_region+Sex	2.010	1829.000
ENSG00000151422	0.134	0.067	0.135	-0.002	FER	0.203	~p_tau+(1|manifest)+cngeneson+pc_mito+brain_region+Sex	1.074	2590.000
ENSG00000162415	0.336	0.067	0.248	-0.115	ZSWIM5	0.415	~p_tau+(1|manifest)+cngeneson+pc_mito+brain_region+Sex	2.158	1728.000
ENSG00000102572	0.081	0.066	0.329	-0.196	STK24	0.139	~p_tau+(1|manifest)+cngeneson+pc_mito+brain_region+Sex	2.261	1663.000
ENSG00000170142	0.067	0.066	0.217	-0.084	UBE2E1	0.120	~p_tau+(1|manifest)+cngeneson+pc_mito+brain_region+Sex	1.415	2293.000
ENSG00000166833	0.669	0.066	0.220	-0.088	NAV2	0.715	~p_tau+(1|manifest)+cngeneson+pc_mito+brain_region+Sex	16.616	80.000
ENSG00000116044	0.113	0.066	0.271	-0.140	NFE2L2	0.179	~p_tau+(1|manifest)+cngeneson+pc_mito+brain_region+Sex	1.768	2003.000
ENSG00000185728	0.001	0.065	0.154	-0.023	YTHDF3	0.006	~p_tau+(1|manifest)+cngeneson+pc_mito+brain_region+Sex	0.736	2880.000
ENSG00000180801	0.165	0.065	0.280	-0.149	ARSJ	0.237	~p_tau+(1|manifest)+cngeneson+pc_mito+brain_region+Sex	1.000	2652.000
ENSG00000154127	0.285	0.065	0.412	-0.282	UBASH3B	0.366	~p_tau+(1|manifest)+cngeneson+pc_mito+brain_region+Sex	5.585	606.000
ENSG00000155099	0.001	0.065	0.212	-0.082	PIP4P2	0.006	~p_tau+(1|manifest)+cngeneson+pc_mito+brain_region+Sex	1.205	2479.000
ENSG00000183044	0.117	0.065	0.349	-0.219	ABAT	0.183	~p_tau+(1|manifest)+cngeneson+pc_mito+brain_region+Sex	3.106	1218.000
ENSG00000181090	0.287	0.065	0.269	-0.139	EHMT1	0.368	~p_tau+(1|manifest)+cngeneson+pc_mito+brain_region+Sex	4.979	702.000
ENSG00000011258	0.005	0.065	0.195	-0.066	MBTD1	0.018	~p_tau+(1|manifest)+cngeneson+pc_mito+brain_region+Sex	0.530	2996.000
ENSG00000122376	0.033	0.065	0.147	-0.018	SHLD2	0.070	~p_tau+(1|manifest)+cngeneson+pc_mito+brain_region+Sex	0.334	3094.000
ENSG00000132780	0.061	0.064	0.275	-0.146	NASP	0.112	~p_tau+(1|manifest)+cngeneson+pc_mito+brain_region+Sex	3.456	1069.000
ENSG00000205269	0.093	0.064	0.209	-0.080	TMEM170B	0.154	~p_tau+(1|manifest)+cngeneson+pc_mito+brain_region+Sex	1.957	1873.000
ENSG00000182185	0.713	0.064	0.242	-0.113	RAD51B	0.757	~p_tau+(1|manifest)+cngeneson+pc_mito+brain_region+Sex	0.948	2694.000
ENSG00000077157	0.446	0.064	0.198	-0.070	PPP1R12B	0.519	~p_tau+(1|manifest)+cngeneson+pc_mito+brain_region+Sex	0.720	2896.000
ENSG00000110497	0.005	0.064	0.249	-0.121	AMBRA1	0.018	~p_tau+(1|manifest)+cngeneson+pc_mito+brain_region+Sex	1.669	2088.000
ENSG00000167081	0.723	0.064	0.251	-0.123	PBX3	0.764	~p_tau+(1|manifest)+cngeneson+pc_mito+brain_region+Sex	0.717	2900.000
ENSG00000156535	0.020	0.064	0.351	-0.223	CD109	0.048	~p_tau+(1|manifest)+cngeneson+pc_mito+brain_region+Sex	0.635	2945.000
ENSG00000130449	0.465	0.064	0.184	-0.057	ZSWIM6	0.535	~p_tau+(1|manifest)+cngeneson+pc_mito+brain_region+Sex	4.563	783.000
ENSG00000169398	0.038	0.063	0.113	0.014	PTK2	0.077	~p_tau+(1|manifest)+cngeneson+pc_mito+brain_region+Sex	2.035	1815.000
ENSG00000143437	0.008	0.063	0.176	-0.049	ARNT	0.025	~p_tau+(1|manifest)+cngeneson+pc_mito+brain_region+Sex	0.707	2909.000
ENSG00000091039	0.001	0.063	0.197	-0.071	OSBPL8	0.007	~p_tau+(1|manifest)+cngeneson+pc_mito+brain_region+Sex	1.519	2203.000
ENSG00000099783	0.009	0.063	0.169	-0.044	HNRNPM	0.027	~p_tau+(1|manifest)+cngeneson+pc_mito+brain_region+Sex	2.077	1783.000
ENSG00000075151	0.149	0.063	0.160	-0.034	EIF4G3	0.220	~p_tau+(1|manifest)+cngeneson+pc_mito+brain_region+Sex	3.623	1028.000
ENSG00000133657	0.107	0.063	0.293	-0.167	ATP13A3	0.171	~p_tau+(1|manifest)+cngeneson+pc_mito+brain_region+Sex	4.975	705.000
ENSG00000148700	0.355	0.062	0.150	-0.025	ADD3	0.434	~p_tau+(1|manifest)+cngeneson+pc_mito+brain_region+Sex	3.134	1201.000
ENSG00000108799	0.005	0.062	0.223	-0.099	EZH1	0.018	~p_tau+(1|manifest)+cngeneson+pc_mito+brain_region+Sex	2.080	1782.000
ENSG00000111266	0.002	0.062	0.200	-0.076	DUSP16	0.008	~p_tau+(1|manifest)+cngeneson+pc_mito+brain_region+Sex	0.165	3132.000
ENSG00000170144	0.006	0.062	0.201	-0.077	HNRNPA3	0.021	~p_tau+(1|manifest)+cngeneson+pc_mito+brain_region+Sex	1.268	2434.000
ENSG00000158528	0.588	0.061	0.223	-0.101	PPP1R9A	0.644	~p_tau+(1|manifest)+cngeneson+pc_mito+brain_region+Sex	1.339	2364.000
ENSG00000080503	0.775	0.061	0.250	-0.128	SMARCA2	0.807	~p_tau+(1|manifest)+cngeneson+pc_mito+brain_region+Sex	5.776	575.000
ENSG00000167978	0.431	0.061	0.566	-0.443	SRRM2	0.504	~p_tau+(1|manifest)+cngeneson+pc_mito+brain_region+Sex	12.927	145.000
ENSG00000165091	0.465	0.061	0.199	-0.077	TMC1	0.535	~p_tau+(1|manifest)+cngeneson+pc_mito+brain_region+Sex	0.664	2929.000
ENSG00000106344	0.079	0.061	0.246	-0.125	RBM28	0.136	~p_tau+(1|manifest)+cngeneson+pc_mito+brain_region+Sex	3.000	1264.000
ENSG00000139154	0.265	0.061	0.288	-0.167	AEBP2	0.346	~p_tau+(1|manifest)+cngeneson+pc_mito+brain_region+Sex	3.050	1243.000
ENSG00000173575	0.126	0.061	0.206	-0.085	CHD2	0.194	~p_tau+(1|manifest)+cngeneson+pc_mito+brain_region+Sex	2.743	1384.000
ENSG00000078618	0.001	0.061	0.183	-0.062	NRDC	0.007	~p_tau+(1|manifest)+cngeneson+pc_mito+brain_region+Sex	2.718	1395.000
ENSG00000138069	0.017	0.061	0.263	-0.142	RAB1A	0.042	~p_tau+(1|manifest)+cngeneson+pc_mito+brain_region+Sex	2.663	1423.000
ENSG00000121310	0.322	0.061	0.273	-0.152	ECHDC2	0.402	~p_tau+(1|manifest)+cngeneson+pc_mito+brain_region+Sex	13.768	128.000
ENSG00000100592	0.846	0.061	0.267	-0.145	DAAM1	0.867	~p_tau+(1|manifest)+cngeneson+pc_mito+brain_region+Sex	14.020	123.000
ENSG00000175105	0.000	0.060	0.174	-0.053	ZNF654	0.004	~p_tau+(1|manifest)+cngeneson+pc_mito+brain_region+Sex	1.829	1964.000
ENSG00000124214	0.006	0.060	0.219	-0.099	STAU1	0.021	~p_tau+(1|manifest)+cngeneson+pc_mito+brain_region+Sex	1.869	1940.000
ENSG00000153774	0.001	0.060	0.161	-0.042	CFDP1	0.005	~p_tau+(1|manifest)+cngeneson+pc_mito+brain_region+Sex	0.870	2764.000
ENSG00000119522	0.518	0.060	0.161	-0.042	DENND1A	0.585	~p_tau+(1|manifest)+cngeneson+pc_mito+brain_region+Sex	1.430	2279.000
ENSG00000107263	0.015	0.059	0.244	-0.125	RAPGEF1	0.040	~p_tau+(1|manifest)+cngeneson+pc_mito+brain_region+Sex	1.890	1922.000
ENSG00000157110	0.401	0.059	0.438	-0.319	RBPMS	0.477	~p_tau+(1|manifest)+cngeneson+pc_mito+brain_region+Sex	12.943	142.000
ENSG00000100767	0.523	0.059	0.539	-0.421	PAPLN	0.589	~p_tau+(1|manifest)+cngeneson+pc_mito+brain_region+Sex	14.002	124.000
ENSG00000187605	0.042	0.058	0.202	-0.085	TET3	0.083	~p_tau+(1|manifest)+cngeneson+pc_mito+brain_region+Sex	0.718	2898.000
ENSG00000011275	0.000	0.058	0.195	-0.078	RNF216	0.003	~p_tau+(1|manifest)+cngeneson+pc_mito+brain_region+Sex	0.850	2780.000
ENSG00000135766	0.007	0.058	0.178	-0.062	EGLN1	0.023	~p_tau+(1|manifest)+cngeneson+pc_mito+brain_region+Sex	0.453	3044.000
ENSG00000165959	0.001	0.058	0.302	-0.186	CLMN	0.004	~p_tau+(1|manifest)+cngeneson+pc_mito+brain_region+Sex	1.171	2515.000
ENSG00000084676	0.179	0.058	0.122	-0.006	NCOA1	0.253	~p_tau+(1|manifest)+cngeneson+pc_mito+brain_region+Sex	4.199	858.000
ENSG00000109685	0.001	0.057	0.204	-0.089	NSD2	0.005	~p_tau+(1|manifest)+cngeneson+pc_mito+brain_region+Sex	1.443	2269.000
ENSG00000157426	0.009	0.057	0.242	-0.127	AASDH	0.027	~p_tau+(1|manifest)+cngeneson+pc_mito+brain_region+Sex	2.091	1775.000
ENSG00000140632	0.006	0.057	0.172	-0.059	GLYR1	0.022	~p_tau+(1|manifest)+cngeneson+pc_mito+brain_region+Sex	1.529	2193.000
ENSG00000135540	0.782	0.057	0.232	-0.118	NHSL1	0.813	~p_tau+(1|manifest)+cngeneson+pc_mito+brain_region+Sex	9.702	273.000
ENSG00000196312	0.006	0.056	0.177	-0.064	MFSD14C	0.021	~p_tau+(1|manifest)+cngeneson+pc_mito+brain_region+Sex	0.956	2687.000
ENSG00000198793	0.006	0.056	0.170	-0.058	MTOR	0.020	~p_tau+(1|manifest)+cngeneson+pc_mito+brain_region+Sex	0.956	2686.000
ENSG00000143353	0.006	0.056	0.188	-0.076	LYPLAL1	0.020	~p_tau+(1|manifest)+cngeneson+pc_mito+brain_region+Sex	1.555	2168.000
ENSG00000143164	0.010	0.056	0.259	-0.147	DCAF6	0.029	~p_tau+(1|manifest)+cngeneson+pc_mito+brain_region+Sex	1.628	2115.000
ENSG00000070785	0.001	0.056	0.128	-0.017	EIF2B3	0.006	~p_tau+(1|manifest)+cngeneson+pc_mito+brain_region+Sex	0.385	3071.000
ENSG00000070367	0.000	0.055	0.164	-0.053	EXOC5	0.003	~p_tau+(1|manifest)+cngeneson+pc_mito+brain_region+Sex	0.441	3046.000
ENSG00000198947	0.534	0.055	0.287	-0.176	DMD	0.597	~p_tau+(1|manifest)+cngeneson+pc_mito+brain_region+Sex	2.448	1548.000
ENSG00000111077	0.080	0.055	0.420	-0.310	TNS2	0.138	~p_tau+(1|manifest)+cngeneson+pc_mito+brain_region+Sex	7.262	415.000
ENSG00000134480	0.683	0.055	0.228	-0.118	CCNH	0.729	~p_tau+(1|manifest)+cngeneson+pc_mito+brain_region+Sex	1.755	2016.000
ENSG00000157450	0.004	0.055	0.157	-0.048	RNF111	0.015	~p_tau+(1|manifest)+cngeneson+pc_mito+brain_region+Sex	1.477	2240.000
ENSG00000102910	0.411	0.055	0.176	-0.066	LONP2	0.486	~p_tau+(1|manifest)+cngeneson+pc_mito+brain_region+Sex	4.571	780.000
ENSG00000153187	0.120	0.054	0.287	-0.179	HNRNPU	0.186	~p_tau+(1|manifest)+cngeneson+pc_mito+brain_region+Sex	1.231	2457.000
ENSG00000182670	0.013	0.054	0.197	-0.088	TTC3	0.035	~p_tau+(1|manifest)+cngeneson+pc_mito+brain_region+Sex	2.030	1822.000
ENSG00000119685	0.029	0.054	0.204	-0.095	TTLL5	0.063	~p_tau+(1|manifest)+cngeneson+pc_mito+brain_region+Sex	1.635	2111.000
ENSG00000158470	0.233	0.054	0.312	-0.204	B4GALT5	0.311	~p_tau+(1|manifest)+cngeneson+pc_mito+brain_region+Sex	4.253	841.000
ENSG00000140545	0.219	0.054	0.367	-0.259	MFGE8	0.295	~p_tau+(1|manifest)+cngeneson+pc_mito+brain_region+Sex	3.435	1080.000
ENSG00000173065	0.034	0.054	0.181	-0.073	FAM222B	0.071	~p_tau+(1|manifest)+cngeneson+pc_mito+brain_region+Sex	0.728	2890.000
ENSG00000175806	0.777	0.053	0.341	-0.234	MSRA	0.809	~p_tau+(1|manifest)+cngeneson+pc_mito+brain_region+Sex	7.878	362.000
ENSG00000079691	0.466	0.053	0.143	-0.036	CARMIL1	0.535	~p_tau+(1|manifest)+cngeneson+pc_mito+brain_region+Sex	1.991	1847.000
ENSG00000007168	0.065	0.053	0.220	-0.114	PAFAH1B1	0.117	~p_tau+(1|manifest)+cngeneson+pc_mito+brain_region+Sex	2.469	1537.000
ENSG00000106617	0.060	0.053	0.255	-0.149	PRKAG2	0.110	~p_tau+(1|manifest)+cngeneson+pc_mito+brain_region+Sex	1.733	2041.000
ENSG00000122378	0.044	0.053	0.216	-0.110	PRXL2A	0.087	~p_tau+(1|manifest)+cngeneson+pc_mito+brain_region+Sex	3.093	1223.000
ENSG00000122042	0.099	0.053	0.198	-0.092	UBL3	0.162	~p_tau+(1|manifest)+cngeneson+pc_mito+brain_region+Sex	0.475	3033.000
ENSG00000171735	0.356	0.052	0.123	-0.018	CAMTA1	0.434	~p_tau+(1|manifest)+cngeneson+pc_mito+brain_region+Sex	5.769	578.000
ENSG00000023171	0.155	0.052	0.377	-0.273	GRAMD1B	0.226	~p_tau+(1|manifest)+cngeneson+pc_mito+brain_region+Sex	3.130	1204.000
ENSG00000172086	0.001	0.052	0.133	-0.029	KRCC1	0.004	~p_tau+(1|manifest)+cngeneson+pc_mito+brain_region+Sex	0.652	2937.000
ENSG00000091490	0.065	0.052	0.407	-0.303	SEL1L3	0.118	~p_tau+(1|manifest)+cngeneson+pc_mito+brain_region+Sex	3.837	955.000
ENSG00000186153	0.610	0.052	0.204	-0.100	WWOX	0.664	~p_tau+(1|manifest)+cngeneson+pc_mito+brain_region+Sex	9.312	295.000
ENSG00000101266	0.004	0.052	0.151	-0.048	CSNK2A1	0.017	~p_tau+(1|manifest)+cngeneson+pc_mito+brain_region+Sex	1.862	1944.000
ENSG00000106460	0.029	0.051	0.178	-0.075	TMEM106B	0.063	~p_tau+(1|manifest)+cngeneson+pc_mito+brain_region+Sex	0.791	2840.000
ENSG00000135090	0.414	0.051	0.168	-0.065	TAOK3	0.489	~p_tau+(1|manifest)+cngeneson+pc_mito+brain_region+Sex	2.304	1631.000
ENSG00000064419	0.002	0.051	0.160	-0.058	TNPO3	0.011	~p_tau+(1|manifest)+cngeneson+pc_mito+brain_region+Sex	0.553	2983.000
ENSG00000100345	0.312	0.051	0.351	-0.248	MYH9	0.392	~p_tau+(1|manifest)+cngeneson+pc_mito+brain_region+Sex	6.453	494.000
ENSG00000176783	0.003	0.051	0.239	-0.137	RUFY1	0.012	~p_tau+(1|manifest)+cngeneson+pc_mito+brain_region+Sex	2.712	1400.000
ENSG00000109099	0.180	0.051	0.334	-0.232	PMP22	0.254	~p_tau+(1|manifest)+cngeneson+pc_mito+brain_region+Sex	2.434	1555.000
ENSG00000127481	0.000	0.051	0.181	-0.080	UBR4	0.000	~p_tau+(1|manifest)+cngeneson+pc_mito+brain_region+Sex	1.422	2284.000
ENSG00000113594	0.528	0.051	0.143	-0.042	LIFR	0.592	~p_tau+(1|manifest)+cngeneson+pc_mito+brain_region+Sex	5.159	673.000
ENSG00000173744	0.002	0.051	0.195	-0.094	AGFG1	0.009	~p_tau+(1|manifest)+cngeneson+pc_mito+brain_region+Sex	1.385	2324.000
ENSG00000119004	0.010	0.051	0.194	-0.093	CYP20A1	0.029	~p_tau+(1|manifest)+cngeneson+pc_mito+brain_region+Sex	0.700	2915.000
ENSG00000186001	0.000	0.050	0.154	-0.053	LRCH3	0.000	~p_tau+(1|manifest)+cngeneson+pc_mito+brain_region+Sex	1.311	2389.000
ENSG00000105983	0.000	0.050	0.201	-0.101	LMBR1	0.004	~p_tau+(1|manifest)+cngeneson+pc_mito+brain_region+Sex	2.396	1577.000
ENSG00000261609	0.472	0.050	0.210	-0.110	GAN	0.541	~p_tau+(1|manifest)+cngeneson+pc_mito+brain_region+Sex	2.512	1508.000
ENSG00000104517	0.289	0.050	0.127	-0.026	UBR5	0.370	~p_tau+(1|manifest)+cngeneson+pc_mito+brain_region+Sex	1.736	2036.000
ENSG00000139132	0.693	0.050	0.232	-0.132	FGD4	0.738	~p_tau+(1|manifest)+cngeneson+pc_mito+brain_region+Sex	4.251	844.000
ENSG00000162869	0.015	0.050	0.125	-0.025	PPP1R21	0.039	~p_tau+(1|manifest)+cngeneson+pc_mito+brain_region+Sex	1.472	2245.000
ENSG00000196236	0.012	0.050	0.132	-0.032	XPNPEP3	0.034	~p_tau+(1|manifest)+cngeneson+pc_mito+brain_region+Sex	0.893	2742.000
ENSG00000188596	0.015	0.049	0.447	-0.348	CFAP54	0.040	~p_tau+(1|manifest)+cngeneson+pc_mito+brain_region+Sex	0.579	2969.000
ENSG00000153827	0.032	0.049	0.165	-0.066	TRIP12	0.068	~p_tau+(1|manifest)+cngeneson+pc_mito+brain_region+Sex	1.970	1867.000
ENSG00000188342	0.009	0.049	0.152	-0.053	GTF2F2	0.026	~p_tau+(1|manifest)+cngeneson+pc_mito+brain_region+Sex	0.462	3039.000
ENSG00000157827	0.283	0.049	0.117	-0.019	FMNL2	0.365	~p_tau+(1|manifest)+cngeneson+pc_mito+brain_region+Sex	2.567	1475.000
ENSG00000185483	0.265	0.049	0.506	-0.408	ROR1	0.346	~p_tau+(1|manifest)+cngeneson+pc_mito+brain_region+Sex	7.043	437.000
ENSG00000139842	0.000	0.049	0.235	-0.137	CUL4A	0.004	~p_tau+(1|manifest)+cngeneson+pc_mito+brain_region+Sex	2.613	1448.000
ENSG00000135932	0.000	0.049	0.164	-0.066	CAB39	0.000	~p_tau+(1|manifest)+cngeneson+pc_mito+brain_region+Sex	0.958	2678.000
ENSG00000128585	0.539	0.049	0.139	-0.041	MKLN1	0.601	~p_tau+(1|manifest)+cngeneson+pc_mito+brain_region+Sex	2.479	1528.000
ENSG00000055130	0.001	0.049	0.135	-0.038	CUL1	0.006	~p_tau+(1|manifest)+cngeneson+pc_mito+brain_region+Sex	0.828	2801.000
ENSG00000133789	0.152	0.048	0.210	-0.114	SWAP70	0.224	~p_tau+(1|manifest)+cngeneson+pc_mito+brain_region+Sex	4.225	850.000
ENSG00000135838	0.679	0.048	0.371	-0.274	NPL	0.725	~p_tau+(1|manifest)+cngeneson+pc_mito+brain_region+Sex	10.191	249.000
ENSG00000133943	0.409	0.048	0.305	-0.209	DGLUCY	0.485	~p_tau+(1|manifest)+cngeneson+pc_mito+brain_region+Sex	4.807	726.000
ENSG00000132300	0.011	0.048	0.176	-0.081	PTCD3	0.031	~p_tau+(1|manifest)+cngeneson+pc_mito+brain_region+Sex	1.913	1902.000
ENSG00000118260	0.004	0.048	0.165	-0.069	CREB1	0.015	~p_tau+(1|manifest)+cngeneson+pc_mito+brain_region+Sex	0.902	2734.000
ENSG00000043093	0.280	0.047	0.331	-0.236	DCUN1D1	0.362	~p_tau+(1|manifest)+cngeneson+pc_mito+brain_region+Sex	2.335	1609.000
ENSG00000166532	0.224	0.047	0.239	-0.145	RIMKLB	0.301	~p_tau+(1|manifest)+cngeneson+pc_mito+brain_region+Sex	3.007	1261.000
ENSG00000102606	0.004	0.047	0.160	-0.066	ARHGEF7	0.014	~p_tau+(1|manifest)+cngeneson+pc_mito+brain_region+Sex	0.692	2917.000
ENSG00000100485	0.009	0.047	0.144	-0.050	SOS2	0.028	~p_tau+(1|manifest)+cngeneson+pc_mito+brain_region+Sex	2.005	1837.000
ENSG00000065526	0.071	0.047	0.235	-0.141	SPEN	0.126	~p_tau+(1|manifest)+cngeneson+pc_mito+brain_region+Sex	2.311	1623.000
ENSG00000126070	0.036	0.047	0.184	-0.091	AGO3	0.075	~p_tau+(1|manifest)+cngeneson+pc_mito+brain_region+Sex	2.724	1389.000
ENSG00000165209	0.081	0.046	0.243	-0.150	STRBP	0.139	~p_tau+(1|manifest)+cngeneson+pc_mito+brain_region+Sex	1.545	2174.000
ENSG00000117020	0.630	0.046	0.141	-0.048	AKT3	0.682	~p_tau+(1|manifest)+cngeneson+pc_mito+brain_region+Sex	0.348	3090.000
ENSG00000241962	0.463	0.046	0.246	-0.154	AC079447.1	0.534	~p_tau+(1|manifest)+cngeneson+pc_mito+brain_region+Sex	0.887	2747.000
ENSG00000115525	0.029	0.046	0.187	-0.095	ST3GAL5	0.063	~p_tau+(1|manifest)+cngeneson+pc_mito+brain_region+Sex	2.316	1622.000
ENSG00000070961	0.113	0.046	0.209	-0.118	ATP2B1	0.179	~p_tau+(1|manifest)+cngeneson+pc_mito+brain_region+Sex	1.319	2379.000
ENSG00000168137	0.147	0.046	0.142	-0.051	SETD5	0.217	~p_tau+(1|manifest)+cngeneson+pc_mito+brain_region+Sex	1.475	2242.000
ENSG00000196141	0.426	0.045	0.247	-0.156	SPATS2L	0.500	~p_tau+(1|manifest)+cngeneson+pc_mito+brain_region+Sex	0.812	2819.000
ENSG00000108306	0.000	0.045	0.147	-0.057	FBXL20	0.003	~p_tau+(1|manifest)+cngeneson+pc_mito+brain_region+Sex	0.737	2879.000
ENSG00000102804	0.776	0.045	0.423	-0.333	TSC22D1	0.808	~p_tau+(1|manifest)+cngeneson+pc_mito+brain_region+Sex	2.284	1651.000
ENSG00000198900	0.001	0.045	0.154	-0.064	TOP1	0.004	~p_tau+(1|manifest)+cngeneson+pc_mito+brain_region+Sex	0.511	3009.000
ENSG00000196220	0.876	0.045	0.318	-0.228	SRGAP3	0.894	~p_tau+(1|manifest)+cngeneson+pc_mito+brain_region+Sex	12.614	157.000
ENSG00000153208	0.765	0.045	0.374	-0.284	MERTK	0.799	~p_tau+(1|manifest)+cngeneson+pc_mito+brain_region+Sex	4.657	757.000
ENSG00000185129	0.044	0.045	0.212	-0.123	PURA	0.087	~p_tau+(1|manifest)+cngeneson+pc_mito+brain_region+Sex	1.235	2451.000
ENSG00000080815	0.030	0.044	0.128	-0.039	PSEN1	0.065	~p_tau+(1|manifest)+cngeneson+pc_mito+brain_region+Sex	0.826	2806.000
ENSG00000121774	0.002	0.044	0.148	-0.060	KHDRBS1	0.011	~p_tau+(1|manifest)+cngeneson+pc_mito+brain_region+Sex	0.357	3085.000
ENSG00000165669	0.004	0.044	0.196	-0.107	FAM204A	0.016	~p_tau+(1|manifest)+cngeneson+pc_mito+brain_region+Sex	1.220	2465.000
ENSG00000137261	0.274	0.044	0.331	-0.243	KIAA0319	0.355	~p_tau+(1|manifest)+cngeneson+pc_mito+brain_region+Sex	2.023	1824.000
ENSG00000131941	0.047	0.044	0.255	-0.167	RHPN2	0.092	~p_tau+(1|manifest)+cngeneson+pc_mito+brain_region+Sex	2.980	1273.000
ENSG00000148690	0.002	0.044	0.183	-0.095	FRA10AC1	0.009	~p_tau+(1|manifest)+cngeneson+pc_mito+brain_region+Sex	0.611	2958.000
ENSG00000114030	0.001	0.043	0.181	-0.094	KPNA1	0.005	~p_tau+(1|manifest)+cngeneson+pc_mito+brain_region+Sex	1.698	2067.000
ENSG00000125386	0.011	0.043	0.233	-0.147	FAM193A	0.032	~p_tau+(1|manifest)+cngeneson+pc_mito+brain_region+Sex	1.901	1912.000
ENSG00000166949	0.193	0.043	0.328	-0.242	SMAD3	0.268	~p_tau+(1|manifest)+cngeneson+pc_mito+brain_region+Sex	2.795	1360.000
ENSG00000150627	0.355	0.043	0.380	-0.295	WDR17	0.434	~p_tau+(1|manifest)+cngeneson+pc_mito+brain_region+Sex	2.894	1318.000
ENSG00000168675	0.768	0.043	0.369	-0.284	LDLRAD4	0.802	~p_tau+(1|manifest)+cngeneson+pc_mito+brain_region+Sex	1.880	1931.000
ENSG00000115808	0.001	0.043	0.134	-0.049	STRN	0.005	~p_tau+(1|manifest)+cngeneson+pc_mito+brain_region+Sex	0.943	2695.000
ENSG00000128654	0.059	0.042	0.207	-0.122	MTX2	0.109	~p_tau+(1|manifest)+cngeneson+pc_mito+brain_region+Sex	0.815	2816.000
ENSG00000104133	0.014	0.042	0.142	-0.057	SPG11	0.038	~p_tau+(1|manifest)+cngeneson+pc_mito+brain_region+Sex	2.180	1715.000
ENSG00000173163	0.141	0.042	0.193	-0.109	COMMD1	0.211	~p_tau+(1|manifest)+cngeneson+pc_mito+brain_region+Sex	1.975	1862.000
ENSG00000168917	0.189	0.042	0.436	-0.353	SLC35G2	0.264	~p_tau+(1|manifest)+cngeneson+pc_mito+brain_region+Sex	4.253	842.000
ENSG00000131018	0.857	0.042	0.215	-0.132	SYNE1	0.876	~p_tau+(1|manifest)+cngeneson+pc_mito+brain_region+Sex	7.420	404.000
ENSG00000185278	0.009	0.041	0.152	-0.070	ZBTB37	0.026	~p_tau+(1|manifest)+cngeneson+pc_mito+brain_region+Sex	0.793	2839.000
ENSG00000072310	0.431	0.041	0.562	-0.479	SREBF1	0.504	~p_tau+(1|manifest)+cngeneson+pc_mito+brain_region+Sex	15.085	97.000
ENSG00000182606	0.594	0.041	0.140	-0.058	TRAK1	0.649	~p_tau+(1|manifest)+cngeneson+pc_mito+brain_region+Sex	3.183	1185.000
ENSG00000171791	0.743	0.041	0.157	-0.074	BCL2	0.781	~p_tau+(1|manifest)+cngeneson+pc_mito+brain_region+Sex	2.351	1599.000
ENSG00000187123	0.379	0.041	0.344	-0.262	LYPD6	0.456	~p_tau+(1|manifest)+cngeneson+pc_mito+brain_region+Sex	4.034	904.000
ENSG00000124782	0.006	0.041	0.178	-0.097	RREB1	0.020	~p_tau+(1|manifest)+cngeneson+pc_mito+brain_region+Sex	0.926	2707.000
ENSG00000084710	0.393	0.041	0.294	-0.212	EFR3B	0.470	~p_tau+(1|manifest)+cngeneson+pc_mito+brain_region+Sex	3.774	979.000
ENSG00000178385	0.001	0.041	0.172	-0.091	PLEKHM3	0.007	~p_tau+(1|manifest)+cngeneson+pc_mito+brain_region+Sex	0.366	3077.000
ENSG00000100784	0.621	0.040	0.324	-0.243	RPS6KA5	0.674	~p_tau+(1|manifest)+cngeneson+pc_mito+brain_region+Sex	6.566	479.000
ENSG00000183621	0.351	0.040	0.186	-0.105	ZNF438	0.430	~p_tau+(1|manifest)+cngeneson+pc_mito+brain_region+Sex	0.893	2740.000
ENSG00000107341	0.013	0.040	0.213	-0.133	UBE2R2	0.035	~p_tau+(1|manifest)+cngeneson+pc_mito+brain_region+Sex	1.541	2179.000
ENSG00000149532	0.189	0.040	0.316	-0.235	CPSF7	0.264	~p_tau+(1|manifest)+cngeneson+pc_mito+brain_region+Sex	5.586	604.000
ENSG00000169032	0.018	0.040	0.136	-0.056	MAP2K1	0.045	~p_tau+(1|manifest)+cngeneson+pc_mito+brain_region+Sex	0.707	2910.000
ENSG00000069667	0.754	0.040	0.179	-0.099	RORA	0.790	~p_tau+(1|manifest)+cngeneson+pc_mito+brain_region+Sex	19.954	54.000
ENSG00000096093	0.302	0.040	0.264	-0.185	EFHC1	0.383	~p_tau+(1|manifest)+cngeneson+pc_mito+brain_region+Sex	1.981	1857.000
ENSG00000122085	0.032	0.040	0.221	-0.141	MTERF4	0.068	~p_tau+(1|manifest)+cngeneson+pc_mito+brain_region+Sex	2.540	1483.000
ENSG00000153339	0.000	0.039	0.171	-0.093	TRAPPC8	0.000	~p_tau+(1|manifest)+cngeneson+pc_mito+brain_region+Sex	0.851	2779.000
ENSG00000156026	0.030	0.039	0.129	-0.052	MCU	0.064	~p_tau+(1|manifest)+cngeneson+pc_mito+brain_region+Sex	0.906	2728.000
ENSG00000123384	0.348	0.039	0.222	-0.145	LRP1	0.427	~p_tau+(1|manifest)+cngeneson+pc_mito+brain_region+Sex	3.866	941.000
ENSG00000165006	0.254	0.038	0.269	-0.193	UBAP1	0.333	~p_tau+(1|manifest)+cngeneson+pc_mito+brain_region+Sex	3.550	1047.000
ENSG00000162378	0.001	0.038	0.134	-0.058	ZYG11B	0.008	~p_tau+(1|manifest)+cngeneson+pc_mito+brain_region+Sex	1.059	2604.000
ENSG00000082805	0.288	0.038	0.151	-0.076	ERC1	0.369	~p_tau+(1|manifest)+cngeneson+pc_mito+brain_region+Sex	0.486	3027.000
ENSG00000066027	0.139	0.038	0.228	-0.153	PPP2R5A	0.209	~p_tau+(1|manifest)+cngeneson+pc_mito+brain_region+Sex	0.709	2906.000
ENSG00000138668	0.027	0.037	0.136	-0.061	HNRNPD	0.060	~p_tau+(1|manifest)+cngeneson+pc_mito+brain_region+Sex	2.307	1627.000
ENSG00000140750	0.001	0.037	0.174	-0.100	ARHGAP17	0.004	~p_tau+(1|manifest)+cngeneson+pc_mito+brain_region+Sex	1.611	2136.000
ENSG00000074201	0.044	0.037	0.125	-0.051	CLNS1A	0.087	~p_tau+(1|manifest)+cngeneson+pc_mito+brain_region+Sex	0.365	3078.000
ENSG00000156113	0.728	0.037	0.152	-0.078	KCNMA1	0.769	~p_tau+(1|manifest)+cngeneson+pc_mito+brain_region+Sex	6.259	513.000
ENSG00000114354	0.001	0.037	0.119	-0.044	TFG	0.005	~p_tau+(1|manifest)+cngeneson+pc_mito+brain_region+Sex	0.963	2674.000
ENSG00000136381	0.000	0.036	0.142	-0.069	IREB2	0.001	~p_tau+(1|manifest)+cngeneson+pc_mito+brain_region+Sex	1.826	1966.000
ENSG00000113712	0.010	0.036	0.150	-0.077	CSNK1A1	0.028	~p_tau+(1|manifest)+cngeneson+pc_mito+brain_region+Sex	3.386	1103.000
ENSG00000110367	0.005	0.036	0.131	-0.058	DDX6	0.017	~p_tau+(1|manifest)+cngeneson+pc_mito+brain_region+Sex	0.602	2960.000
ENSG00000152520	0.105	0.036	0.173	-0.100	PAN3	0.169	~p_tau+(1|manifest)+cngeneson+pc_mito+brain_region+Sex	2.097	1773.000
ENSG00000196639	0.140	0.036	0.221	-0.148	HRH1	0.209	~p_tau+(1|manifest)+cngeneson+pc_mito+brain_region+Sex	0.078	3137.000
ENSG00000204272	0.063	0.036	0.276	-0.204	NBDY	0.114	~p_tau+(1|manifest)+cngeneson+pc_mito+brain_region+Sex	0.528	2999.000
ENSG00000121289	0.000	0.036	0.155	-0.083	CEP89	0.003	~p_tau+(1|manifest)+cngeneson+pc_mito+brain_region+Sex	1.004	2647.000
ENSG00000079277	0.028	0.036	0.211	-0.139	MKNK1	0.062	~p_tau+(1|manifest)+cngeneson+pc_mito+brain_region+Sex	2.717	1397.000
ENSG00000198399	0.001	0.036	0.143	-0.071	ITSN2	0.007	~p_tau+(1|manifest)+cngeneson+pc_mito+brain_region+Sex	0.775	2853.000
ENSG00000010017	0.343	0.036	0.247	-0.175	RANBP9	0.422	~p_tau+(1|manifest)+cngeneson+pc_mito+brain_region+Sex	2.788	1367.000
ENSG00000102763	0.523	0.036	0.345	-0.273	VWA8	0.588	~p_tau+(1|manifest)+cngeneson+pc_mito+brain_region+Sex	11.565	191.000
ENSG00000152952	0.758	0.035	0.330	-0.259	PLOD2	0.793	~p_tau+(1|manifest)+cngeneson+pc_mito+brain_region+Sex	0.583	2968.000
ENSG00000279170	0.023	0.035	0.174	-0.103	TSTD3	0.054	~p_tau+(1|manifest)+cngeneson+pc_mito+brain_region+Sex	1.309	2390.000
ENSG00000109654	0.335	0.035	0.199	-0.129	TRIM2	0.414	~p_tau+(1|manifest)+cngeneson+pc_mito+brain_region+Sex	1.870	1938.000
ENSG00000108654	0.474	0.035	0.351	-0.282	DDX5	0.541	~p_tau+(1|manifest)+cngeneson+pc_mito+brain_region+Sex	12.814	147.000
ENSG00000110721	0.721	0.035	0.326	-0.257	CHKA	0.763	~p_tau+(1|manifest)+cngeneson+pc_mito+brain_region+Sex	7.244	418.000
ENSG00000112320	0.382	0.034	0.187	-0.118	SOBP	0.459	~p_tau+(1|manifest)+cngeneson+pc_mito+brain_region+Sex	1.648	2100.000
ENSG00000138443	0.454	0.034	0.193	-0.125	ABI2	0.526	~p_tau+(1|manifest)+cngeneson+pc_mito+brain_region+Sex	1.252	2441.000
ENSG00000205726	0.806	0.034	0.149	-0.080	ITSN1	0.833	~p_tau+(1|manifest)+cngeneson+pc_mito+brain_region+Sex	5.055	692.000
ENSG00000137198	0.332	0.034	0.505	-0.436	GMPR	0.412	~p_tau+(1|manifest)+cngeneson+pc_mito+brain_region+Sex	5.383	637.000
ENSG00000152270	0.813	0.034	0.512	-0.444	PDE3B	0.839	~p_tau+(1|manifest)+cngeneson+pc_mito+brain_region+Sex	6.012	545.000
ENSG00000166033	0.554	0.034	0.386	-0.319	HTRA1	0.614	~p_tau+(1|manifest)+cngeneson+pc_mito+brain_region+Sex	5.259	659.000
ENSG00000272391	0.009	0.034	0.115	-0.048	POM121C	0.028	~p_tau+(1|manifest)+cngeneson+pc_mito+brain_region+Sex	0.506	3014.000
ENSG00000068878	0.163	0.034	0.218	-0.150	PSME4	0.236	~p_tau+(1|manifest)+cngeneson+pc_mito+brain_region+Sex	1.985	1852.000
ENSG00000112769	0.386	0.033	0.457	-0.390	LAMA4	0.463	~p_tau+(1|manifest)+cngeneson+pc_mito+brain_region+Sex	5.037	694.000
ENSG00000129084	0.022	0.033	0.230	-0.163	PSMA1	0.052	~p_tau+(1|manifest)+cngeneson+pc_mito+brain_region+Sex	1.005	2644.000
ENSG00000144369	0.857	0.032	0.271	-0.206	FAM171B	0.876	~p_tau+(1|manifest)+cngeneson+pc_mito+brain_region+Sex	7.428	403.000
ENSG00000067369	0.000	0.032	0.146	-0.081	TP53BP1	0.001	~p_tau+(1|manifest)+cngeneson+pc_mito+brain_region+Sex	1.385	2325.000
ENSG00000132670	0.057	0.032	0.167	-0.103	PTPRA	0.107	~p_tau+(1|manifest)+cngeneson+pc_mito+brain_region+Sex	2.611	1450.000
ENSG00000074054	0.799	0.032	0.126	-0.062	CLASP1	0.828	~p_tau+(1|manifest)+cngeneson+pc_mito+brain_region+Sex	0.828	2803.000
ENSG00000175782	0.050	0.032	0.257	-0.193	SLC35E3	0.096	~p_tau+(1|manifest)+cngeneson+pc_mito+brain_region+Sex	1.518	2204.000
ENSG00000108091	0.105	0.032	0.245	-0.181	CCDC6	0.169	~p_tau+(1|manifest)+cngeneson+pc_mito+brain_region+Sex	1.392	2318.000
ENSG00000008952	0.153	0.032	0.205	-0.141	SEC62	0.224	~p_tau+(1|manifest)+cngeneson+pc_mito+brain_region+Sex	1.066	2600.000
ENSG00000136044	0.145	0.032	0.270	-0.206	APPL2	0.215	~p_tau+(1|manifest)+cngeneson+pc_mito+brain_region+Sex	3.673	1010.000
ENSG00000197081	0.066	0.031	0.225	-0.162	IGF2R	0.118	~p_tau+(1|manifest)+cngeneson+pc_mito+brain_region+Sex	3.337	1120.000
ENSG00000047365	0.541	0.031	0.115	-0.053	ARAP2	0.603	~p_tau+(1|manifest)+cngeneson+pc_mito+brain_region+Sex	4.268	838.000
ENSG00000133026	0.010	0.031	0.130	-0.067	MYH10	0.029	~p_tau+(1|manifest)+cngeneson+pc_mito+brain_region+Sex	1.081	2579.000
ENSG00000165443	0.918	0.031	0.347	-0.285	PHYHIPL	0.931	~p_tau+(1|manifest)+cngeneson+pc_mito+brain_region+Sex	2.679	1415.000
ENSG00000101871	0.428	0.031	0.268	-0.206	MID1	0.502	~p_tau+(1|manifest)+cngeneson+pc_mito+brain_region+Sex	2.354	1597.000
ENSG00000198860	0.098	0.031	0.240	-0.179	TSEN15	0.159	~p_tau+(1|manifest)+cngeneson+pc_mito+brain_region+Sex	0.808	2827.000
ENSG00000163960	0.002	0.030	0.126	-0.065	UBXN7	0.009	~p_tau+(1|manifest)+cngeneson+pc_mito+brain_region+Sex	0.894	2738.000
ENSG00000005436	0.030	0.030	0.136	-0.076	GCFC2	0.065	~p_tau+(1|manifest)+cngeneson+pc_mito+brain_region+Sex	0.996	2656.000
ENSG00000102781	0.008	0.030	0.213	-0.153	KATNAL1	0.026	~p_tau+(1|manifest)+cngeneson+pc_mito+brain_region+Sex	1.070	2593.000
ENSG00000166123	0.651	0.030	0.406	-0.346	GPT2	0.700	~p_tau+(1|manifest)+cngeneson+pc_mito+brain_region+Sex	2.951	1286.000
ENSG00000183722	0.984	0.030	0.349	-0.290	LHFPL6	0.988	~p_tau+(1|manifest)+cngeneson+pc_mito+brain_region+Sex	8.312	341.000
ENSG00000154654	0.550	0.030	0.167	-0.107	NCAM2	0.611	~p_tau+(1|manifest)+cngeneson+pc_mito+brain_region+Sex	8.566	318.000
ENSG00000106554	0.367	0.030	0.217	-0.158	CHCHD3	0.445	~p_tau+(1|manifest)+cngeneson+pc_mito+brain_region+Sex	1.146	2537.000
ENSG00000147588	0.588	0.029	0.441	-0.382	PMP2	0.644	~p_tau+(1|manifest)+cngeneson+pc_mito+brain_region+Sex	7.055	434.000
ENSG00000066084	0.670	0.029	0.221	-0.163	DIP2B	0.716	~p_tau+(1|manifest)+cngeneson+pc_mito+brain_region+Sex	3.405	1099.000
ENSG00000134318	0.645	0.029	0.359	-0.302	ROCK2	0.695	~p_tau+(1|manifest)+cngeneson+pc_mito+brain_region+Sex	7.133	425.000
ENSG00000132471	0.047	0.029	0.234	-0.177	WBP2	0.091	~p_tau+(1|manifest)+cngeneson+pc_mito+brain_region+Sex	3.132	1203.000
ENSG00000181722	0.540	0.029	0.140	-0.083	ZBTB20	0.602	~p_tau+(1|manifest)+cngeneson+pc_mito+brain_region+Sex	14.992	100.000
ENSG00000099139	0.790	0.028	0.172	-0.115	PCSK5	0.820	~p_tau+(1|manifest)+cngeneson+pc_mito+brain_region+Sex	2.629	1441.000
ENSG00000198898	0.024	0.028	0.209	-0.153	CAPZA2	0.055	~p_tau+(1|manifest)+cngeneson+pc_mito+brain_region+Sex	2.465	1540.000
ENSG00000173905	0.601	0.028	0.202	-0.145	GOLIM4	0.656	~p_tau+(1|manifest)+cngeneson+pc_mito+brain_region+Sex	3.419	1087.000
ENSG00000117713	0.320	0.028	0.119	-0.063	ARID1A	0.399	~p_tau+(1|manifest)+cngeneson+pc_mito+brain_region+Sex	2.644	1434.000
ENSG00000044115	0.460	0.028	0.242	-0.186	CTNNA1	0.531	~p_tau+(1|manifest)+cngeneson+pc_mito+brain_region+Sex	1.603	2142.000
ENSG00000136051	0.001	0.028	0.151	-0.095	WASHC4	0.005	~p_tau+(1|manifest)+cngeneson+pc_mito+brain_region+Sex	0.621	2955.000
ENSG00000145982	0.418	0.028	0.219	-0.164	FARS2	0.493	~p_tau+(1|manifest)+cngeneson+pc_mito+brain_region+Sex	0.832	2800.000
ENSG00000063660	0.293	0.028	0.343	-0.288	GPC1	0.374	~p_tau+(1|manifest)+cngeneson+pc_mito+brain_region+Sex	4.038	903.000
ENSG00000146457	0.026	0.027	0.218	-0.164	WTAP	0.058	~p_tau+(1|manifest)+cngeneson+pc_mito+brain_region+Sex	2.623	1444.000
ENSG00000114302	0.030	0.027	0.252	-0.198	PRKAR2A	0.065	~p_tau+(1|manifest)+cngeneson+pc_mito+brain_region+Sex	1.192	2494.000
ENSG00000086666	0.093	0.027	0.152	-0.099	ZFAND6	0.154	~p_tau+(1|manifest)+cngeneson+pc_mito+brain_region+Sex	0.986	2664.000
ENSG00000197694	0.112	0.027	0.192	-0.139	SPTAN1	0.177	~p_tau+(1|manifest)+cngeneson+pc_mito+brain_region+Sex	4.459	801.000
ENSG00000147677	0.004	0.027	0.147	-0.094	EIF3H	0.016	~p_tau+(1|manifest)+cngeneson+pc_mito+brain_region+Sex	0.553	2984.000
ENSG00000187118	0.037	0.026	0.145	-0.092	CMC1	0.076	~p_tau+(1|manifest)+cngeneson+pc_mito+brain_region+Sex	0.533	2994.000
ENSG00000186409	0.047	0.026	0.208	-0.155	CCDC30	0.091	~p_tau+(1|manifest)+cngeneson+pc_mito+brain_region+Sex	1.154	2530.000
ENSG00000227345	0.018	0.026	0.133	-0.081	PARG	0.044	~p_tau+(1|manifest)+cngeneson+pc_mito+brain_region+Sex	1.103	2564.000
ENSG00000127314	0.005	0.026	0.194	-0.141	RAP1B	0.018	~p_tau+(1|manifest)+cngeneson+pc_mito+brain_region+Sex	2.768	1377.000
ENSG00000214113	0.115	0.026	0.141	-0.089	LYRM4	0.180	~p_tau+(1|manifest)+cngeneson+pc_mito+brain_region+Sex	0.591	2963.000
ENSG00000265491	0.014	0.026	0.127	-0.076	RNF115	0.037	~p_tau+(1|manifest)+cngeneson+pc_mito+brain_region+Sex	1.188	2501.000
ENSG00000135250	0.159	0.025	0.114	-0.064	SRPK2	0.231	~p_tau+(1|manifest)+cngeneson+pc_mito+brain_region+Sex	1.992	1845.000
ENSG00000088970	0.002	0.025	0.272	-0.221	KIZ	0.009	~p_tau+(1|manifest)+cngeneson+pc_mito+brain_region+Sex	0.957	2685.000
ENSG00000130066	0.246	0.025	0.398	-0.348	SAT1	0.324	~p_tau+(1|manifest)+cngeneson+pc_mito+brain_region+Sex	6.635	472.000
ENSG00000127947	0.053	0.025	0.222	-0.173	PTPN12	0.101	~p_tau+(1|manifest)+cngeneson+pc_mito+brain_region+Sex	2.391	1580.000
ENSG00000119280	0.291	0.024	0.414	-0.365	C1orf198	0.372	~p_tau+(1|manifest)+cngeneson+pc_mito+brain_region+Sex	6.066	541.000
ENSG00000100934	0.013	0.024	0.121	-0.072	SEC23A	0.035	~p_tau+(1|manifest)+cngeneson+pc_mito+brain_region+Sex	1.379	2330.000
ENSG00000107829	0.038	0.024	0.165	-0.117	FBXW4	0.077	~p_tau+(1|manifest)+cngeneson+pc_mito+brain_region+Sex	0.807	2828.000
ENSG00000139323	0.145	0.024	0.296	-0.248	POC1B	0.215	~p_tau+(1|manifest)+cngeneson+pc_mito+brain_region+Sex	5.068	691.000
ENSG00000169554	0.787	0.024	0.231	-0.184	ZEB2	0.818	~p_tau+(1|manifest)+cngeneson+pc_mito+brain_region+Sex	1.631	2113.000
ENSG00000114948	0.344	0.024	0.327	-0.279	ADAM23	0.422	~p_tau+(1|manifest)+cngeneson+pc_mito+brain_region+Sex	4.224	852.000
ENSG00000113269	0.246	0.024	0.173	-0.126	RNF130	0.325	~p_tau+(1|manifest)+cngeneson+pc_mito+brain_region+Sex	1.290	2414.000
ENSG00000127990	0.130	0.023	0.225	-0.178	SGCE	0.199	~p_tau+(1|manifest)+cngeneson+pc_mito+brain_region+Sex	0.005	3138.000
ENSG00000135040	0.010	0.023	0.147	-0.100	NAA35	0.031	~p_tau+(1|manifest)+cngeneson+pc_mito+brain_region+Sex	1.204	2480.000
ENSG00000115020	0.004	0.023	0.149	-0.102	PIKFYVE	0.017	~p_tau+(1|manifest)+cngeneson+pc_mito+brain_region+Sex	1.418	2288.000
ENSG00000109756	0.604	0.023	0.184	-0.138	RAPGEF2	0.659	~p_tau+(1|manifest)+cngeneson+pc_mito+brain_region+Sex	1.103	2565.000
ENSG00000137871	0.091	0.023	0.267	-0.221	ZNF280D	0.151	~p_tau+(1|manifest)+cngeneson+pc_mito+brain_region+Sex	3.392	1102.000
ENSG00000149294	0.925	0.023	0.140	-0.094	NCAM1	0.938	~p_tau+(1|manifest)+cngeneson+pc_mito+brain_region+Sex	6.886	456.000
ENSG00000278540	0.243	0.023	0.128	-0.083	ACACA	0.321	~p_tau+(1|manifest)+cngeneson+pc_mito+brain_region+Sex	1.299	2406.000
ENSG00000121440	0.962	0.023	0.198	-0.153	PDZRN3	0.969	~p_tau+(1|manifest)+cngeneson+pc_mito+brain_region+Sex	3.207	1172.000
ENSG00000137285	0.397	0.023	0.555	-0.510	TUBB2B	0.474	~p_tau+(1|manifest)+cngeneson+pc_mito+brain_region+Sex	2.949	1289.000
ENSG00000154511	0.651	0.022	0.188	-0.143	DIPK1A	0.700	~p_tau+(1|manifest)+cngeneson+pc_mito+brain_region+Sex	2.445	1551.000
ENSG00000138771	0.954	0.022	0.407	-0.362	SHROOM3	0.963	~p_tau+(1|manifest)+cngeneson+pc_mito+brain_region+Sex	3.049	1244.000
ENSG00000096060	0.730	0.022	0.329	-0.285	FKBP5	0.770	~p_tau+(1|manifest)+cngeneson+pc_mito+brain_region+Sex	3.245	1156.000
ENSG00000127249	0.986	0.022	0.271	-0.227	ATP13A4	0.989	~p_tau+(1|manifest)+cngeneson+pc_mito+brain_region+Sex	13.266	138.000
ENSG00000106536	0.944	0.022	0.484	-0.441	POU6F2	0.953	~p_tau+(1|manifest)+cngeneson+pc_mito+brain_region+Sex	12.658	155.000
ENSG00000115464	0.295	0.022	0.089	-0.046	USP34	0.376	~p_tau+(1|manifest)+cngeneson+pc_mito+brain_region+Sex	4.824	723.000
ENSG00000121691	0.185	0.022	0.220	-0.177	CAT	0.260	~p_tau+(1|manifest)+cngeneson+pc_mito+brain_region+Sex	1.198	2482.000
ENSG00000109046	0.410	0.021	0.232	-0.189	WSB1	0.485	~p_tau+(1|manifest)+cngeneson+pc_mito+brain_region+Sex	14.055	122.000
ENSG00000162688	0.095	0.021	0.216	-0.174	AGL	0.156	~p_tau+(1|manifest)+cngeneson+pc_mito+brain_region+Sex	1.983	1856.000
ENSG00000154229	0.579	0.021	0.140	-0.098	PRKCA	0.637	~p_tau+(1|manifest)+cngeneson+pc_mito+brain_region+Sex	14.881	101.000
ENSG00000163625	0.606	0.021	0.102	-0.060	WDFY3	0.660	~p_tau+(1|manifest)+cngeneson+pc_mito+brain_region+Sex	1.050	2608.000

ENSG00000185920	0.551	0.021	0.375	-0.333	PTCH1	0.612	~p_tau+(1|manifest)+cngeneson+pc_mito+brain_region+Sex	4.529	790.000
ENSG00000181322	0.395	0.021	0.217	-0.175	NME9	0.471	~p_tau+(1|manifest)+cngeneson+pc_mito+brain_region+Sex	2.074	1785.000
ENSG00000159256	0.008	0.021	0.133	-0.092	MORC3	0.026	~p_tau+(1|manifest)+cngeneson+pc_mito+brain_region+Sex	1.027	2623.000
ENSG00000138071	0.011	0.020	0.091	-0.051	ACTR2	0.032	~p_tau+(1|manifest)+cngeneson+pc_mito+brain_region+Sex	0.660	2931.000
ENSG00000176102	0.011	0.020	0.167	-0.126	CSTF3	0.031	~p_tau+(1|manifest)+cngeneson+pc_mito+brain_region+Sex	0.228	3118.000
ENSG00000081320	0.200	0.020	0.289	-0.249	STK17B	0.276	~p_tau+(1|manifest)+cngeneson+pc_mito+brain_region+Sex	1.108	2561.000
ENSG00000153902	0.404	0.020	0.350	-0.310	LGI4	0.481	~p_tau+(1|manifest)+cngeneson+pc_mito+brain_region+Sex	4.532	788.000
ENSG00000114166	0.826	0.020	0.281	-0.241	KAT2B	0.849	~p_tau+(1|manifest)+cngeneson+pc_mito+brain_region+Sex	5.330	646.000
ENSG00000131375	0.002	0.019	0.130	-0.091	CAPN7	0.008	~p_tau+(1|manifest)+cngeneson+pc_mito+brain_region+Sex	2.161	1724.000
ENSG00000283154	0.735	0.019	0.232	-0.193	IQCJ-SCHIP1	0.773	~p_tau+(1|manifest)+cngeneson+pc_mito+brain_region+Sex	2.893	1319.000
ENSG00000273136	0.007	0.019	0.359	-0.321	NBPF26	0.023	~p_tau+(1|manifest)+cngeneson+pc_mito+brain_region+Sex	1.242	2446.000
ENSG00000160145	0.812	0.019	0.229	-0.191	KALRN	0.839	~p_tau+(1|manifest)+cngeneson+pc_mito+brain_region+Sex	10.460	235.000
ENSG00000177200	0.571	0.019	0.141	-0.103	CHD9	0.629	~p_tau+(1|manifest)+cngeneson+pc_mito+brain_region+Sex	5.692	592.000
ENSG00000176407	0.000	0.019	0.141	-0.104	KCMF1	0.004	~p_tau+(1|manifest)+cngeneson+pc_mito+brain_region+Sex	1.306	2394.000
ENSG00000115266	0.650	0.019	0.482	-0.445	APC2	0.700	~p_tau+(1|manifest)+cngeneson+pc_mito+brain_region+Sex	16.927	78.000
ENSG00000172845	0.003	0.018	0.141	-0.104	SP3	0.012	~p_tau+(1|manifest)+cngeneson+pc_mito+brain_region+Sex	0.512	3008.000
ENSG00000131966	0.001	0.018	0.152	-0.115	ACTR10	0.004	~p_tau+(1|manifest)+cngeneson+pc_mito+brain_region+Sex	0.499	3018.000
ENSG00000180537	0.504	0.018	0.274	-0.238	RNF182	0.571	~p_tau+(1|manifest)+cngeneson+pc_mito+brain_region+Sex	3.739	995.000
ENSG00000111011	0.009	0.018	0.155	-0.120	RSRC2	0.027	~p_tau+(1|manifest)+cngeneson+pc_mito+brain_region+Sex	1.938	1886.000
ENSG00000186868	0.386	0.018	0.185	-0.150	MAPT	0.463	~p_tau+(1|manifest)+cngeneson+pc_mito+brain_region+Sex	12.237	169.000
ENSG00000109320	0.087	0.017	0.258	-0.223	NFKB1	0.146	~p_tau+(1|manifest)+cngeneson+pc_mito+brain_region+Sex	2.118	1760.000
ENSG00000123607	0.001	0.017	0.070	-0.036	TTC21B	0.005	~p_tau+(1|manifest)+cngeneson+pc_mito+brain_region+Sex	0.747	2868.000
ENSG00000100722	0.017	0.017	0.122	-0.087	ZC3H14	0.043	~p_tau+(1|manifest)+cngeneson+pc_mito+brain_region+Sex	0.906	2730.000
ENSG00000175582	0.001	0.016	0.121	-0.088	RAB6A	0.007	~p_tau+(1|manifest)+cngeneson+pc_mito+brain_region+Sex	2.289	1643.000
ENSG00000040341	0.202	0.016	0.171	-0.139	STAU2	0.278	~p_tau+(1|manifest)+cngeneson+pc_mito+brain_region+Sex	1.710	2058.000
ENSG00000179715	0.471	0.016	0.434	-0.401	PCED1B	0.540	~p_tau+(1|manifest)+cngeneson+pc_mito+brain_region+Sex	6.120	532.000
ENSG00000009413	0.408	0.016	0.096	-0.063	REV3L	0.484	~p_tau+(1|manifest)+cngeneson+pc_mito+brain_region+Sex	1.104	2562.000
ENSG00000130723	0.275	0.016	0.237	-0.205	PRRC2B	0.357	~p_tau+(1|manifest)+cngeneson+pc_mito+brain_region+Sex	3.623	1029.000
ENSG00000142599	0.833	0.016	0.069	-0.038	RERE	0.856	~p_tau+(1|manifest)+cngeneson+pc_mito+brain_region+Sex	1.912	1904.000
ENSG00000155816	0.756	0.016	0.130	-0.099	FMN2	0.792	~p_tau+(1|manifest)+cngeneson+pc_mito+brain_region+Sex	6.326	509.000
ENSG00000065609	0.235	0.016	0.152	-0.121	SNAP91	0.313	~p_tau+(1|manifest)+cngeneson+pc_mito+brain_region+Sex	0.174	3130.000
ENSG00000124198	0.000	0.015	0.142	-0.112	ARFGEF2	0.000	~p_tau+(1|manifest)+cngeneson+pc_mito+brain_region+Sex	1.728	2044.000
ENSG00000074211	0.451	0.014	0.390	-0.361	PPP2R2C	0.523	~p_tau+(1|manifest)+cngeneson+pc_mito+brain_region+Sex	5.127	679.000
ENSG00000103018	0.823	0.014	0.267	-0.238	CYB5B	0.847	~p_tau+(1|manifest)+cngeneson+pc_mito+brain_region+Sex	7.445	402.000
ENSG00000056972	0.587	0.014	0.233	-0.205	TRAF3IP2	0.644	~p_tau+(1|manifest)+cngeneson+pc_mito+brain_region+Sex	4.916	708.000
ENSG00000137513	0.039	0.014	0.090	-0.062	NARS2	0.079	~p_tau+(1|manifest)+cngeneson+pc_mito+brain_region+Sex	0.717	2899.000
ENSG00000151461	0.044	0.014	0.159	-0.131	UPF2	0.087	~p_tau+(1|manifest)+cngeneson+pc_mito+brain_region+Sex	1.623	2123.000
ENSG00000167601	0.056	0.013	0.220	-0.193	AXL	0.105	~p_tau+(1|manifest)+cngeneson+pc_mito+brain_region+Sex	1.988	1848.000
ENSG00000170743	0.109	0.013	0.230	-0.204	SYT9	0.173	~p_tau+(1|manifest)+cngeneson+pc_mito+brain_region+Sex	0.623	2952.000
ENSG00000148484	0.038	0.013	0.150	-0.125	RSU1	0.078	~p_tau+(1|manifest)+cngeneson+pc_mito+brain_region+Sex	2.307	1626.000
ENSG00000182326	0.374	0.013	0.305	-0.279	C1S	0.451	~p_tau+(1|manifest)+cngeneson+pc_mito+brain_region+Sex	2.951	1287.000
ENSG00000175216	0.001	0.013	0.097	-0.072	CKAP5	0.008	~p_tau+(1|manifest)+cngeneson+pc_mito+brain_region+Sex	1.397	2312.000
ENSG00000095794	0.574	0.012	0.160	-0.135	CREM	0.632	~p_tau+(1|manifest)+cngeneson+pc_mito+brain_region+Sex	0.551	2986.000
ENSG00000112308	0.035	0.012	0.227	-0.202	C6orf62	0.073	~p_tau+(1|manifest)+cngeneson+pc_mito+brain_region+Sex	1.015	2634.000
ENSG00000198265	0.004	0.012	0.097	-0.073	HELZ	0.016	~p_tau+(1|manifest)+cngeneson+pc_mito+brain_region+Sex	1.189	2500.000
ENSG00000122779	0.003	0.012	0.132	-0.108	TRIM24	0.014	~p_tau+(1|manifest)+cngeneson+pc_mito+brain_region+Sex	1.496	2223.000
ENSG00000147687	0.002	0.012	0.148	-0.123	TATDN1	0.009	~p_tau+(1|manifest)+cngeneson+pc_mito+brain_region+Sex	0.683	2922.000
ENSG00000168175	0.004	0.012	0.202	-0.178	MAPK1IP1L	0.015	~p_tau+(1|manifest)+cngeneson+pc_mito+brain_region+Sex	1.687	2077.000
ENSG00000196712	0.545	0.012	0.063	-0.039	NF1	0.607	~p_tau+(1|manifest)+cngeneson+pc_mito+brain_region+Sex	2.052	1798.000
ENSG00000108669	0.034	0.012	0.157	-0.134	CYTH1	0.071	~p_tau+(1|manifest)+cngeneson+pc_mito+brain_region+Sex	1.516	2206.000
ENSG00000102038	0.108	0.012	0.222	-0.198	SMARCA1	0.172	~p_tau+(1|manifest)+cngeneson+pc_mito+brain_region+Sex	2.049	1804.000
ENSG00000008294	0.031	0.012	0.082	-0.058	SPAG9	0.066	~p_tau+(1|manifest)+cngeneson+pc_mito+brain_region+Sex	3.589	1040.000
ENSG00000178691	0.005	0.011	0.084	-0.061	SUZ12	0.018	~p_tau+(1|manifest)+cngeneson+pc_mito+brain_region+Sex	0.744	2871.000
ENSG00000148053	0.990	0.011	0.184	-0.161	NTRK2	0.993	~p_tau+(1|manifest)+cngeneson+pc_mito+brain_region+Sex	19.122	57.000
ENSG00000167491	0.022	0.011	0.167	-0.145	GATAD2A	0.052	~p_tau+(1|manifest)+cngeneson+pc_mito+brain_region+Sex	2.010	1830.000
ENSG00000055917	0.024	0.011	0.151	-0.130	PUM2	0.055	~p_tau+(1|manifest)+cngeneson+pc_mito+brain_region+Sex	1.118	2557.000
ENSG00000142655	0.000	0.011	0.110	-0.089	PEX14	0.003	~p_tau+(1|manifest)+cngeneson+pc_mito+brain_region+Sex	0.808	2826.000
ENSG00000123636	0.573	0.011	0.104	-0.083	BAZ2B	0.631	~p_tau+(1|manifest)+cngeneson+pc_mito+brain_region+Sex	4.129	878.000
ENSG00000153823	0.729	0.011	0.276	-0.255	PID1	0.770	~p_tau+(1|manifest)+cngeneson+pc_mito+brain_region+Sex	3.563	1046.000
ENSG00000048342	0.048	0.011	0.205	-0.183	CC2D2A	0.092	~p_tau+(1|manifest)+cngeneson+pc_mito+brain_region+Sex	1.917	1899.000
ENSG00000104447	0.795	0.011	0.133	-0.112	TRPS1	0.825	~p_tau+(1|manifest)+cngeneson+pc_mito+brain_region+Sex	6.150	527.000
ENSG00000197548	0.856	0.011	0.103	-0.081	ATG7	0.876	~p_tau+(1|manifest)+cngeneson+pc_mito+brain_region+Sex	2.859	1336.000
ENSG00000138777	0.122	0.011	0.187	-0.166	PPA2	0.189	~p_tau+(1|manifest)+cngeneson+pc_mito+brain_region+Sex	0.597	2961.000
ENSG00000115295	0.715	0.011	0.249	-0.228	CLIP4	0.758	~p_tau+(1|manifest)+cngeneson+pc_mito+brain_region+Sex	1.838	1957.000
ENSG00000122218	0.009	0.010	0.124	-0.104	COPA	0.026	~p_tau+(1|manifest)+cngeneson+pc_mito+brain_region+Sex	0.418	3054.000
ENSG00000132849	0.116	0.010	0.159	-0.139	PATJ	0.182	~p_tau+(1|manifest)+cngeneson+pc_mito+brain_region+Sex	7.064	433.000
ENSG00000164190	0.199	0.010	0.104	-0.084	NIPBL	0.274	~p_tau+(1|manifest)+cngeneson+pc_mito+brain_region+Sex	1.535	2187.000
ENSG00000165322	0.107	0.010	0.106	-0.086	ARHGAP12	0.171	~p_tau+(1|manifest)+cngeneson+pc_mito+brain_region+Sex	0.978	2668.000
ENSG00000067182	0.181	0.010	0.285	-0.264	TNFRSF1A	0.255	~p_tau+(1|manifest)+cngeneson+pc_mito+brain_region+Sex	2.858	1337.000
ENSG00000197969	0.000	0.010	0.106	-0.086	VPS13A	0.001	~p_tau+(1|manifest)+cngeneson+pc_mito+brain_region+Sex	1.452	2260.000
ENSG00000107854	0.008	0.010	0.105	-0.085	TNKS2	0.025	~p_tau+(1|manifest)+cngeneson+pc_mito+brain_region+Sex	0.904	2732.000
ENSG00000101966	0.002	0.009	0.120	-0.103	XIAP	0.010	~p_tau+(1|manifest)+cngeneson+pc_mito+brain_region+Sex	1.274	2427.000
ENSG00000164258	0.012	0.008	0.105	-0.089	NDUFS4	0.033	~p_tau+(1|manifest)+cngeneson+pc_mito+brain_region+Sex	1.012	2637.000
ENSG00000059758	0.155	0.008	0.171	-0.155	CDK17	0.226	~p_tau+(1|manifest)+cngeneson+pc_mito+brain_region+Sex	1.065	2601.000
ENSG00000088448	0.116	0.008	0.211	-0.196	ANKRD10	0.181	~p_tau+(1|manifest)+cngeneson+pc_mito+brain_region+Sex	4.490	797.000
ENSG00000113580	0.082	0.008	0.141	-0.125	NR3C1	0.140	~p_tau+(1|manifest)+cngeneson+pc_mito+brain_region+Sex	0.745	2870.000
ENSG00000119844	0.005	0.008	0.115	-0.100	AFTPH	0.018	~p_tau+(1|manifest)+cngeneson+pc_mito+brain_region+Sex	1.416	2292.000
ENSG00000134313	0.224	0.008	0.164	-0.148	KIDINS220	0.301	~p_tau+(1|manifest)+cngeneson+pc_mito+brain_region+Sex	1.707	2061.000
ENSG00000032219	0.001	0.008	0.119	-0.104	ARID4A	0.008	~p_tau+(1|manifest)+cngeneson+pc_mito+brain_region+Sex	0.870	2765.000
ENSG00000198677	0.002	0.008	0.099	-0.084	TTC37	0.008	~p_tau+(1|manifest)+cngeneson+pc_mito+brain_region+Sex	0.796	2838.000
ENSG00000133401	0.579	0.007	0.301	-0.286	PDZD2	0.637	~p_tau+(1|manifest)+cngeneson+pc_mito+brain_region+Sex	2.799	1358.000
ENSG00000144426	0.003	0.007	0.127	-0.112	NBEAL1	0.012	~p_tau+(1|manifest)+cngeneson+pc_mito+brain_region+Sex	2.448	1549.000
ENSG00000011405	0.266	0.007	0.258	-0.243	PIK3C2A	0.347	~p_tau+(1|manifest)+cngeneson+pc_mito+brain_region+Sex	5.253	660.000
ENSG00000130226	1.000	0.007	0.565	-0.551	DPP6	1.000	~p_tau+(1|manifest)+cngeneson+pc_mito+brain_region+Sex	5.003	700.000
ENSG00000120616	0.408	0.007	0.123	-0.109	EPC1	0.484	~p_tau+(1|manifest)+cngeneson+pc_mito+brain_region+Sex	2.706	1402.000
ENSG00000159917	0.069	0.007	0.095	-0.081	ZNF235	0.123	~p_tau+(1|manifest)+cngeneson+pc_mito+brain_region+Sex	0.834	2795.000
ENSG00000107669	0.004	0.007	0.114	-0.101	ATE1	0.017	~p_tau+(1|manifest)+cngeneson+pc_mito+brain_region+Sex	1.088	2577.000
ENSG00000178567	0.031	0.007	0.126	-0.112	EPM2AIP1	0.067	~p_tau+(1|manifest)+cngeneson+pc_mito+brain_region+Sex	1.360	2348.000
ENSG00000115947	0.009	0.007	0.110	-0.097	ORC4	0.028	~p_tau+(1|manifest)+cngeneson+pc_mito+brain_region+Sex	0.493	3023.000
ENSG00000120798	0.003	0.007	0.130	-0.117	NR2C1	0.012	~p_tau+(1|manifest)+cngeneson+pc_mito+brain_region+Sex	1.898	1915.000
ENSG00000168066	0.193	0.007	0.199	-0.185	SF1	0.268	~p_tau+(1|manifest)+cngeneson+pc_mito+brain_region+Sex	3.591	1039.000
ENSG00000134283	0.052	0.006	0.124	-0.111	PPHLN1	0.099	~p_tau+(1|manifest)+cngeneson+pc_mito+brain_region+Sex	0.868	2766.000
ENSG00000077254	0.002	0.006	0.082	-0.070	USP33	0.011	~p_tau+(1|manifest)+cngeneson+pc_mito+brain_region+Sex	1.689	2075.000
ENSG00000139697	0.023	0.006	0.149	-0.138	SBNO1	0.053	~p_tau+(1|manifest)+cngeneson+pc_mito+brain_region+Sex	1.973	1864.000
ENSG00000185818	0.883	0.006	0.570	-0.559	NAT8L	0.901	~p_tau+(1|manifest)+cngeneson+pc_mito+brain_region+Sex	10.773	222.000
ENSG00000204217	0.308	0.006	0.140	-0.129	BMPR2	0.388	~p_tau+(1|manifest)+cngeneson+pc_mito+brain_region+Sex	2.318	1621.000
ENSG00000170832	0.123	0.006	0.186	-0.175	USP32	0.190	~p_tau+(1|manifest)+cngeneson+pc_mito+brain_region+Sex	3.525	1053.000
ENSG00000123106	0.634	0.006	0.229	-0.218	CCDC91	0.685	~p_tau+(1|manifest)+cngeneson+pc_mito+brain_region+Sex	1.195	2489.000
ENSG00000095637	0.990	0.006	0.165	-0.154	SORBS1	0.993	~p_tau+(1|manifest)+cngeneson+pc_mito+brain_region+Sex	26.627	25.000
ENSG00000174306	0.889	0.005	0.130	-0.119	ZHX3	0.906	~p_tau+(1|manifest)+cngeneson+pc_mito+brain_region+Sex	6.144	529.000
ENSG00000180376	0.028	0.005	0.135	-0.125	CCDC66	0.063	~p_tau+(1|manifest)+cngeneson+pc_mito+brain_region+Sex	1.833	1961.000
ENSG00000106261	0.038	0.005	0.268	-0.258	ZKSCAN1	0.078	~p_tau+(1|manifest)+cngeneson+pc_mito+brain_region+Sex	3.843	954.000
ENSG00000131558	0.758	0.005	0.078	-0.069	EXOC4	0.793	~p_tau+(1|manifest)+cngeneson+pc_mito+brain_region+Sex	1.337	2367.000
ENSG00000047188	0.006	0.004	0.147	-0.138	YTHDC2	0.020	~p_tau+(1|manifest)+cngeneson+pc_mito+brain_region+Sex	0.913	2721.000
ENSG00000107242	0.318	0.004	0.163	-0.154	PIP5K1B	0.397	~p_tau+(1|manifest)+cngeneson+pc_mito+brain_region+Sex	2.598	1457.000
ENSG00000109381	0.473	0.004	0.169	-0.161	ELF2	0.541	~p_tau+(1|manifest)+cngeneson+pc_mito+brain_region+Sex	3.372	1106.000
ENSG00000197442	0.988	0.004	0.085	-0.077	MAP3K5	0.991	~p_tau+(1|manifest)+cngeneson+pc_mito+brain_region+Sex	6.698	466.000
ENSG00000258890	0.013	0.004	0.130	-0.122	CEP95	0.036	~p_tau+(1|manifest)+cngeneson+pc_mito+brain_region+Sex	0.517	3006.000
ENSG00000205683	0.765	0.004	0.297	-0.290	DPF3	0.799	~p_tau+(1|manifest)+cngeneson+pc_mito+brain_region+Sex	3.592	1037.000
ENSG00000128872	0.230	0.003	0.256	-0.249	TMOD2	0.308	~p_tau+(1|manifest)+cngeneson+pc_mito+brain_region+Sex	4.242	845.000
ENSG00000230124	0.101	0.003	0.165	-0.158	ACBD6	0.164	~p_tau+(1|manifest)+cngeneson+pc_mito+brain_region+Sex	0.327	3096.000
ENSG00000169967	0.015	0.003	0.128	-0.121	MAP3K2	0.040	~p_tau+(1|manifest)+cngeneson+pc_mito+brain_region+Sex	2.247	1670.000
ENSG00000136527	0.002	0.003	0.088	-0.082	TRA2B	0.009	~p_tau+(1|manifest)+cngeneson+pc_mito+brain_region+Sex	0.494	3022.000
ENSG00000135315	0.023	0.003	0.144	-0.138	CEP162	0.053	~p_tau+(1|manifest)+cngeneson+pc_mito+brain_region+Sex	1.245	2444.000
ENSG00000157181	0.029	0.003	0.110	-0.104	ODR4	0.063	~p_tau+(1|manifest)+cngeneson+pc_mito+brain_region+Sex	1.864	1941.000
ENSG00000156831	0.288	0.003	0.105	-0.100	NSMCE2	0.369	~p_tau+(1|manifest)+cngeneson+pc_mito+brain_region+Sex	0.343	3092.000
ENSG00000186908	0.006	0.003	0.120	-0.115	ZDHHC17	0.021	~p_tau+(1|manifest)+cngeneson+pc_mito+brain_region+Sex	0.822	2809.000
ENSG00000132561	0.914	0.003	0.614	-0.608	MATN2	0.928	~p_tau+(1|manifest)+cngeneson+pc_mito+brain_region+Sex	19.116	58.000
ENSG00000134152	0.003	0.003	0.096	-0.091	KATNBL1	0.012	~p_tau+(1|manifest)+cngeneson+pc_mito+brain_region+Sex	2.386	1582.000
ENSG00000095951	0.125	0.003	0.178	-0.173	HIVEP1	0.192	~p_tau+(1|manifest)+cngeneson+pc_mito+brain_region+Sex	3.242	1158.000
ENSG00000158161	0.018	0.002	0.088	-0.084	EYA3	0.044	~p_tau+(1|manifest)+cngeneson+pc_mito+brain_region+Sex	0.413	3058.000
ENSG00000101144	0.298	0.002	0.318	-0.314	BMP7	0.379	~p_tau+(1|manifest)+cngeneson+pc_mito+brain_region+Sex	3.418	1089.000
ENSG00000180263	0.139	0.002	0.179	-0.175	FGD6	0.209	~p_tau+(1|manifest)+cngeneson+pc_mito+brain_region+Sex	2.449	1547.000
ENSG00000117000	0.026	0.002	0.156	-0.153	RLF	0.059	~p_tau+(1|manifest)+cngeneson+pc_mito+brain_region+Sex	1.548	2171.000
ENSG00000011376	0.021	0.002	0.076	-0.073	LARS2	0.050	~p_tau+(1|manifest)+cngeneson+pc_mito+brain_region+Sex	0.636	2944.000
ENSG00000180370	0.001	0.001	0.095	-0.093	PAK2	0.006	~p_tau+(1|manifest)+cngeneson+pc_mito+brain_region+Sex	0.983	2666.000
ENSG00000168118	0.164	0.001	0.163	-0.161	RAB4A	0.236	~p_tau+(1|manifest)+cngeneson+pc_mito+brain_region+Sex	1.701	2065.000
ENSG00000143442	0.665	0.001	0.092	-0.090	POGZ	0.713	~p_tau+(1|manifest)+cngeneson+pc_mito+brain_region+Sex	5.908	554.000
ENSG00000173473	0.031	0.001	0.205	-0.203	SMARCC1	0.067	~p_tau+(1|manifest)+cngeneson+pc_mito+brain_region+Sex	2.925	1305.000
ENSG00000135829	0.048	0.001	0.114	-0.112	DHX9	0.093	~p_tau+(1|manifest)+cngeneson+pc_mito+brain_region+Sex	1.629	2114.000
ENSG00000144040	0.992	0.001	0.118	-0.116	SFXN5	0.995	~p_tau+(1|manifest)+cngeneson+pc_mito+brain_region+Sex	6.226	519.000
ENSG00000145012	0.998	0.001	0.124	-0.122	LPP	0.998	~p_tau+(1|manifest)+cngeneson+pc_mito+brain_region+Sex	11.586	189.000
ENSG00000153291	0.011	0.001	0.140	-0.139	SLC25A27	0.031	~p_tau+(1|manifest)+cngeneson+pc_mito+brain_region+Sex	1.635	2110.000
ENSG00000156687	0.954	0.001	0.276	-0.275	UNC5D	0.963	~p_tau+(1|manifest)+cngeneson+pc_mito+brain_region+Sex	2.484	1524.000
ENSG00000150760	0.502	0.001	0.110	-0.109	DOCK1	0.569	~p_tau+(1|manifest)+cngeneson+pc_mito+brain_region+Sex	2.485	1522.000
ENSG00000187239	0.885	0.001	0.083	-0.082	FNBP1	0.901	~p_tau+(1|manifest)+cngeneson+pc_mito+brain_region+Sex	10.723	224.000
ENSG00000089472	0.084	0.000	0.191	-0.191	HEPH	0.143	~p_tau+(1|manifest)+cngeneson+pc_mito+brain_region+Sex	1.315	2384.000
ENSG00000137497	0.096	0.000	0.220	-0.219	NUMA1	0.158	~p_tau+(1|manifest)+cngeneson+pc_mito+brain_region+Sex	2.939	1296.000
ENSG00000144893	0.997	0.000	0.160	-0.159	MED12L	0.998	~p_tau+(1|manifest)+cngeneson+pc_mito+brain_region+Sex	10.531	233.000
ENSG00000030066	0.003	0.000	0.091	-0.090	NUP160	0.014	~p_tau+(1|manifest)+cngeneson+pc_mito+brain_region+Sex	1.337	2368.000
ENSG00000169372	0.818	0.000	0.162	-0.161	CRADD	0.843	~p_tau+(1|manifest)+cngeneson+pc_mito+brain_region+Sex	2.385	1583.000
ENSG00000056097	0.000	0.000	0.101	-0.101	ZFR	0.001	~p_tau+(1|manifest)+cngeneson+pc_mito+brain_region+Sex	1.371	2338.000
ENSG00000152409	0.020	0.000	0.118	-0.118	JMY	0.048	~p_tau+(1|manifest)+cngeneson+pc_mito+brain_region+Sex	0.801	2834.000
ENSG00000134644	0.007	0.000	0.118	-0.119	PUM1	0.024	~p_tau+(1|manifest)+cngeneson+pc_mito+brain_region+Sex	1.893	1921.000
ENSG00000261115	0.623	0.000	0.201	-0.202	TMEM178B	0.676	~p_tau+(1|manifest)+cngeneson+pc_mito+brain_region+Sex	1.642	2106.000
ENSG00000101849	0.513	0.000	0.173	-0.173	TBL1X	0.579	~p_tau+(1|manifest)+cngeneson+pc_mito+brain_region+Sex	1.301	2402.000
ENSG00000138246	0.000	0.000	0.121	-0.122	DNAJC13	0.000	~p_tau+(1|manifest)+cngeneson+pc_mito+brain_region+Sex	0.850	2781.000
ENSG00000097007	0.131	0.000	0.127	-0.128	ABL1	0.200	~p_tau+(1|manifest)+cngeneson+pc_mito+brain_region+Sex	1.460	2252.000
ENSG00000182901	0.816	-0.001	0.482	-0.483	RGS7	0.841	~p_tau+(1|manifest)+cngeneson+pc_mito+brain_region+Sex	8.839	312.000
ENSG00000178075	0.458	-0.001	0.236	-0.237	GRAMD1C	0.529	~p_tau+(1|manifest)+cngeneson+pc_mito+brain_region+Sex	4.440	806.000
ENSG00000085832	0.236	-0.002	0.102	-0.105	EPS15	0.314	~p_tau+(1|manifest)+cngeneson+pc_mito+brain_region+Sex	1.836	1959.000
ENSG00000197102	0.025	-0.002	0.165	-0.168	DYNC1H1	0.057	~p_tau+(1|manifest)+cngeneson+pc_mito+brain_region+Sex	3.868	939.000
ENSG00000179152	0.271	-0.002	0.136	-0.140	TCAIM	0.352	~p_tau+(1|manifest)+cngeneson+pc_mito+brain_region+Sex	1.810	1981.000
ENSG00000183878	0.490	-0.002	0.001	-0.004	UTY	0.557	~p_tau+(1|manifest)+cngeneson+pc_mito+brain_region+Sex	49.838	6.000
ENSG00000148358	0.005	-0.002	0.134	-0.138	GPR107	0.020	~p_tau+(1|manifest)+cngeneson+pc_mito+brain_region+Sex	2.142	1742.000
ENSG00000165246	0.835	-0.002	0.006	-0.009	NLGN4Y	0.857	~p_tau+(1|manifest)+cngeneson+pc_mito+brain_region+Sex	29.586	22.000
ENSG00000075711	0.153	-0.002	0.132	-0.136	DLG1	0.224	~p_tau+(1|manifest)+cngeneson+pc_mito+brain_region+Sex	1.229	2460.000
ENSG00000189283	0.975	-0.002	0.095	-0.098	FHIT	0.980	~p_tau+(1|manifest)+cngeneson+pc_mito+brain_region+Sex	1.806	1984.000
ENSG00000117419	0.816	-0.002	0.174	-0.178	ERI3	0.841	~p_tau+(1|manifest)+cngeneson+pc_mito+brain_region+Sex	4.730	741.000
ENSG00000115145	0.002	-0.002	0.111	-0.115	STAM2	0.010	~p_tau+(1|manifest)+cngeneson+pc_mito+brain_region+Sex	0.211	3123.000
ENSG00000141867	0.158	-0.002	0.185	-0.189	BRD4	0.229	~p_tau+(1|manifest)+cngeneson+pc_mito+brain_region+Sex	2.100	1770.000
ENSG00000163785	0.204	-0.002	0.188	-0.192	RYK	0.279	~p_tau+(1|manifest)+cngeneson+pc_mito+brain_region+Sex	2.523	1494.000
ENSG00000071054	0.841	-0.002	0.127	-0.131	MAP4K4	0.862	~p_tau+(1|manifest)+cngeneson+pc_mito+brain_region+Sex	5.099	687.000
ENSG00000186432	0.003	-0.002	0.088	-0.093	KPNA4	0.013	~p_tau+(1|manifest)+cngeneson+pc_mito+brain_region+Sex	1.389	2320.000
ENSG00000132424	0.224	-0.003	0.129	-0.134	PNISR	0.301	~p_tau+(1|manifest)+cngeneson+pc_mito+brain_region+Sex	11.184	203.000
ENSG00000139116	0.271	-0.003	0.146	-0.152	KIF21A	0.352	~p_tau+(1|manifest)+cngeneson+pc_mito+brain_region+Sex	5.810	567.000
ENSG00000148634	0.158	-0.003	0.154	-0.160	HERC4	0.230	~p_tau+(1|manifest)+cngeneson+pc_mito+brain_region+Sex	0.249	3115.000
ENSG00000168214	0.180	-0.003	0.156	-0.162	RBPJ	0.254	~p_tau+(1|manifest)+cngeneson+pc_mito+brain_region+Sex	1.002	2650.000
ENSG00000148541	0.584	-0.003	0.376	-0.382	FAM13C	0.641	~p_tau+(1|manifest)+cngeneson+pc_mito+brain_region+Sex	2.838	1343.000
ENSG00000151532	0.932	-0.003	0.155	-0.162	VTI1A	0.943	~p_tau+(1|manifest)+cngeneson+pc_mito+brain_region+Sex	0.904	2733.000
ENSG00000117528	0.181	-0.004	0.157	-0.164	ABCD3	0.255	~p_tau+(1|manifest)+cngeneson+pc_mito+brain_region+Sex	1.144	2541.000
ENSG00000170525	0.930	-0.004	0.452	-0.460	PFKFB3	0.942	~p_tau+(1|manifest)+cngeneson+pc_mito+brain_region+Sex	20.426	48.000
ENSG00000196628	0.995	-0.004	0.077	-0.085	TCF4	0.996	~p_tau+(1|manifest)+cngeneson+pc_mito+brain_region+Sex	3.733	997.000
ENSG00000048740	0.994	-0.004	0.098	-0.107	CELF2	0.996	~p_tau+(1|manifest)+cngeneson+pc_mito+brain_region+Sex	6.833	457.000
ENSG00000164494	0.240	-0.005	0.133	-0.143	PDSS2	0.319	~p_tau+(1|manifest)+cngeneson+pc_mito+brain_region+Sex	0.721	2894.000
ENSG00000018189	0.825	-0.005	0.084	-0.094	RUFY3	0.849	~p_tau+(1|manifest)+cngeneson+pc_mito+brain_region+Sex	4.885	712.000
ENSG00000112146	0.047	-0.005	0.141	-0.152	FBXO9	0.092	~p_tau+(1|manifest)+cngeneson+pc_mito+brain_region+Sex	1.698	2070.000
ENSG00000005206	0.512	-0.005	0.453	-0.464	SPPL2B	0.579	~p_tau+(1|manifest)+cngeneson+pc_mito+brain_region+Sex	9.973	261.000
ENSG00000083896	0.022	-0.005	0.164	-0.175	YTHDC1	0.051	~p_tau+(1|manifest)+cngeneson+pc_mito+brain_region+Sex	0.360	3081.000
ENSG00000124177	0.076	-0.006	0.071	-0.082	CHD6	0.133	~p_tau+(1|manifest)+cngeneson+pc_mito+brain_region+Sex	1.640	2107.000
ENSG00000165119	0.031	-0.006	0.146	-0.157	HNRNPK	0.066	~p_tau+(1|manifest)+cngeneson+pc_mito+brain_region+Sex	1.455	2255.000
ENSG00000137962	0.525	-0.006	0.339	-0.351	ARHGAP29	0.590	~p_tau+(1|manifest)+cngeneson+pc_mito+brain_region+Sex	4.361	828.000
ENSG00000145833	0.084	-0.006	0.187	-0.199	DDX46	0.143	~p_tau+(1|manifest)+cngeneson+pc_mito+brain_region+Sex	2.239	1677.000
ENSG00000005889	0.008	-0.006	0.097	-0.109	ZFX	0.025	~p_tau+(1|manifest)+cngeneson+pc_mito+brain_region+Sex	1.933	1891.000
ENSG00000069956	0.038	-0.006	0.109	-0.121	MAPK6	0.077	~p_tau+(1|manifest)+cngeneson+pc_mito+brain_region+Sex	0.801	2833.000
ENSG00000015676	0.025	-0.006	0.067	-0.080	NUDCD3	0.056	~p_tau+(1|manifest)+cngeneson+pc_mito+brain_region+Sex	1.213	2471.000
ENSG00000119596	0.120	-0.006	0.129	-0.142	YLPM1	0.186	~p_tau+(1|manifest)+cngeneson+pc_mito+brain_region+Sex	2.301	1635.000
ENSG00000134909	0.528	-0.006	0.099	-0.112	ARHGAP32	0.592	~p_tau+(1|manifest)+cngeneson+pc_mito+brain_region+Sex	1.747	2022.000
ENSG00000138621	0.091	-0.007	0.208	-0.221	PPCDC	0.151	~p_tau+(1|manifest)+cngeneson+pc_mito+brain_region+Sex	1.680	2080.000
ENSG00000084754	0.046	-0.007	0.120	-0.134	HADHA	0.089	~p_tau+(1|manifest)+cngeneson+pc_mito+brain_region+Sex	1.805	1985.000
ENSG00000198646	0.001	-0.007	0.108	-0.122	NCOA6	0.004	~p_tau+(1|manifest)+cngeneson+pc_mito+brain_region+Sex	2.367	1594.000
ENSG00000122507	0.685	-0.007	0.224	-0.238	BBS9	0.731	~p_tau+(1|manifest)+cngeneson+pc_mito+brain_region+Sex	2.288	1644.000
ENSG00000187527	0.174	-0.007	0.280	-0.295	ATP13A5	0.247	~p_tau+(1|manifest)+cngeneson+pc_mito+brain_region+Sex	4.506	794.000
ENSG00000149084	0.063	-0.007	0.127	-0.142	HSD17B12	0.114	~p_tau+(1|manifest)+cngeneson+pc_mito+brain_region+Sex	1.234	2453.000
ENSG00000011243	0.151	-0.008	0.241	-0.256	AKAP8L	0.222	~p_tau+(1|manifest)+cngeneson+pc_mito+brain_region+Sex	3.094	1222.000
ENSG00000114374	0.170	-0.008	0.012	-0.027	USP9Y	0.243	~p_tau+(1|manifest)+cngeneson+pc_mito+brain_region+Sex	33.554	14.000
ENSG00000141314	0.225	-0.008	0.230	-0.245	RHBDL3	0.301	~p_tau+(1|manifest)+cngeneson+pc_mito+brain_region+Sex	7.455	401.000
ENSG00000062716	0.002	-0.008	0.058	-0.074	VMP1	0.011	~p_tau+(1|manifest)+cngeneson+pc_mito+brain_region+Sex	0.783	2846.000
ENSG00000163104	0.012	-0.008	0.121	-0.137	SMARCAD1	0.034	~p_tau+(1|manifest)+cngeneson+pc_mito+brain_region+Sex	0.963	2673.000
ENSG00000146966	0.307	-0.008	0.209	-0.225	DENND2A	0.388	~p_tau+(1|manifest)+cngeneson+pc_mito+brain_region+Sex	2.343	1605.000
ENSG00000090565	0.109	-0.008	0.224	-0.240	RAB11FIP3	0.173	~p_tau+(1|manifest)+cngeneson+pc_mito+brain_region+Sex	3.973	917.000
ENSG00000008083	0.990	-0.008	0.158	-0.174	JARID2	0.993	~p_tau+(1|manifest)+cngeneson+pc_mito+brain_region+Sex	8.431	329.000
ENSG00000065833	0.520	-0.008	0.352	-0.368	ME1	0.586	~p_tau+(1|manifest)+cngeneson+pc_mito+brain_region+Sex	1.169	2519.000
ENSG00000100030	0.057	-0.008	0.095	-0.111	MAPK1	0.106	~p_tau+(1|manifest)+cngeneson+pc_mito+brain_region+Sex	1.217	2468.000
ENSG00000131171	0.335	-0.008	0.196	-0.212	SH3BGRL	0.414	~p_tau+(1|manifest)+cngeneson+pc_mito+brain_region+Sex	1.738	2033.000
ENSG00000122257	0.003	-0.008	0.125	-0.142	RBBP6	0.014	~p_tau+(1|manifest)+cngeneson+pc_mito+brain_region+Sex	1.981	1858.000
ENSG00000148737	0.921	-0.008	0.064	-0.080	TCF7L2	0.935	~p_tau+(1|manifest)+cngeneson+pc_mito+brain_region+Sex	4.797	731.000
ENSG00000185532	0.910	-0.009	0.171	-0.189	PRKG1	0.925	~p_tau+(1|manifest)+cngeneson+pc_mito+brain_region+Sex	10.890	216.000
ENSG00000164134	0.005	-0.009	0.122	-0.140	NAA15	0.019	~p_tau+(1|manifest)+cngeneson+pc_mito+brain_region+Sex	2.148	1733.000
ENSG00000138650	0.197	-0.009	0.218	-0.236	PCDH10	0.273	~p_tau+(1|manifest)+cngeneson+pc_mito+brain_region+Sex	1.175	2510.000
ENSG00000167258	0.000	-0.009	0.088	-0.106	CDK12	0.004	~p_tau+(1|manifest)+cngeneson+pc_mito+brain_region+Sex	1.072	2592.000
ENSG00000138688	0.104	-0.009	0.102	-0.121	KIAA1109	0.168	~p_tau+(1|manifest)+cngeneson+pc_mito+brain_region+Sex	1.664	2090.000
ENSG00000155189	0.010	-0.009	0.094	-0.112	AGPAT5	0.029	~p_tau+(1|manifest)+cngeneson+pc_mito+brain_region+Sex	0.958	2682.000
ENSG00000108424	0.069	-0.009	0.150	-0.169	KPNB1	0.123	~p_tau+(1|manifest)+cngeneson+pc_mito+brain_region+Sex	2.266	1659.000
ENSG00000165629	0.000	-0.010	0.133	-0.152	ATP5F1C	0.001	~p_tau+(1|manifest)+cngeneson+pc_mito+brain_region+Sex	1.332	2369.000
ENSG00000197912	0.102	-0.010	0.166	-0.186	SPG7	0.166	~p_tau+(1|manifest)+cngeneson+pc_mito+brain_region+Sex	3.059	1238.000
ENSG00000116678	0.486	-0.010	0.159	-0.179	LEPR	0.554	~p_tau+(1|manifest)+cngeneson+pc_mito+brain_region+Sex	1.820	1973.000
ENSG00000108582	0.026	-0.010	0.121	-0.141	CPD	0.059	~p_tau+(1|manifest)+cngeneson+pc_mito+brain_region+Sex	1.169	2518.000
ENSG00000118922	0.995	-0.010	0.300	-0.321	KLF12	0.996	~p_tau+(1|manifest)+cngeneson+pc_mito+brain_region+Sex	7.467	399.000
ENSG00000131374	0.745	-0.011	0.068	-0.089	TBC1D5	0.782	~p_tau+(1|manifest)+cngeneson+pc_mito+brain_region+Sex	3.319	1127.000
ENSG00000198408	0.350	-0.011	0.173	-0.194	OGA	0.428	~p_tau+(1|manifest)+cngeneson+pc_mito+brain_region+Sex	1.507	2213.000
ENSG00000134046	0.001	-0.011	0.099	-0.120	MBD2	0.007	~p_tau+(1|manifest)+cngeneson+pc_mito+brain_region+Sex	1.793	1992.000
ENSG00000080298	0.962	-0.011	0.089	-0.111	RFX3	0.969	~p_tau+(1|manifest)+cngeneson+pc_mito+brain_region+Sex	1.522	2198.000
ENSG00000187676	0.582	-0.011	0.287	-0.309	B3GLCT	0.639	~p_tau+(1|manifest)+cngeneson+pc_mito+brain_region+Sex	3.191	1181.000
ENSG00000152601	0.922	-0.011	0.123	-0.146	MBNL1	0.935	~p_tau+(1|manifest)+cngeneson+pc_mito+brain_region+Sex	3.864	943.000
ENSG00000120910	0.063	-0.012	0.165	-0.188	PPP3CC	0.115	~p_tau+(1|manifest)+cngeneson+pc_mito+brain_region+Sex	1.591	2148.000
ENSG00000115993	0.046	-0.012	0.114	-0.137	TRAK2	0.090	~p_tau+(1|manifest)+cngeneson+pc_mito+brain_region+Sex	0.866	2769.000
ENSG00000143324	0.422	-0.012	0.236	-0.260	XPR1	0.497	~p_tau+(1|manifest)+cngeneson+pc_mito+brain_region+Sex	0.624	2951.000
ENSG00000106299	0.008	-0.012	0.080	-0.105	WASL	0.025	~p_tau+(1|manifest)+cngeneson+pc_mito+brain_region+Sex	0.921	2710.000
ENSG00000157837	0.026	-0.012	0.100	-0.124	SPPL3	0.058	~p_tau+(1|manifest)+cngeneson+pc_mito+brain_region+Sex	1.022	2629.000
ENSG00000114742	0.019	-0.012	0.123	-0.148	WDR48	0.047	~p_tau+(1|manifest)+cngeneson+pc_mito+brain_region+Sex	1.676	2082.000
ENSG00000129244	0.891	-0.013	0.223	-0.248	ATP1B2	0.907	~p_tau+(1|manifest)+cngeneson+pc_mito+brain_region+Sex	13.374	136.000
ENSG00000066739	0.001	-0.013	0.070	-0.095	ATG2B	0.006	~p_tau+(1|manifest)+cngeneson+pc_mito+brain_region+Sex	0.854	2775.000
ENSG00000146426	0.638	-0.013	0.305	-0.331	TIAM2	0.689	~p_tau+(1|manifest)+cngeneson+pc_mito+brain_region+Sex	3.124	1206.000
ENSG00000103550	0.671	-0.013	0.552	-0.578	KNOP1	0.717	~p_tau+(1|manifest)+cngeneson+pc_mito+brain_region+Sex	20.057	53.000
ENSG00000078295	0.984	-0.013	0.150	-0.176	ADCY2	0.989	~p_tau+(1|manifest)+cngeneson+pc_mito+brain_region+Sex	2.516	1504.000
ENSG00000136770	0.310	-0.013	0.171	-0.197	DNAJC1	0.390	~p_tau+(1|manifest)+cngeneson+pc_mito+brain_region+Sex	2.296	1638.000
ENSG00000125246	0.382	-0.013	0.366	-0.392	CLYBL	0.459	~p_tau+(1|manifest)+cngeneson+pc_mito+brain_region+Sex	7.545	388.000
ENSG00000165525	0.001	-0.013	0.117	-0.143	NEMF	0.004	~p_tau+(1|manifest)+cngeneson+pc_mito+brain_region+Sex	2.278	1654.000
ENSG00000113360	0.012	-0.013	0.085	-0.112	DROSHA	0.033	~p_tau+(1|manifest)+cngeneson+pc_mito+brain_region+Sex	1.239	2448.000
ENSG00000135899	0.131	-0.013	0.218	-0.245	SP110	0.199	~p_tau+(1|manifest)+cngeneson+pc_mito+brain_region+Sex	2.487	1521.000
ENSG00000145832	0.541	-0.014	0.328	-0.355	SLC25A48	0.603	~p_tau+(1|manifest)+cngeneson+pc_mito+brain_region+Sex	0.522	3003.000
ENSG00000112981	0.009	-0.014	0.204	-0.232	NME5	0.028	~p_tau+(1|manifest)+cngeneson+pc_mito+brain_region+Sex	1.540	2181.000
ENSG00000115233	0.015	-0.014	0.124	-0.152	PSMD14	0.039	~p_tau+(1|manifest)+cngeneson+pc_mito+brain_region+Sex	1.161	2523.000
ENSG00000134532	0.552	-0.014	0.098	-0.126	SOX5	0.612	~p_tau+(1|manifest)+cngeneson+pc_mito+brain_region+Sex	7.775	369.000
ENSG00000132953	0.000	-0.014	0.072	-0.100	XPO4	0.003	~p_tau+(1|manifest)+cngeneson+pc_mito+brain_region+Sex	1.483	2236.000
ENSG00000143878	0.370	-0.014	0.372	-0.401	RHOB	0.447	~p_tau+(1|manifest)+cngeneson+pc_mito+brain_region+Sex	11.716	184.000
ENSG00000198160	0.000	-0.014	0.117	-0.146	MIER1	0.004	~p_tau+(1|manifest)+cngeneson+pc_mito+brain_region+Sex	1.859	1946.000
ENSG00000142949	0.193	-0.015	0.228	-0.257	PTPRF	0.268	~p_tau+(1|manifest)+cngeneson+pc_mito+brain_region+Sex	1.540	2180.000
ENSG00000135870	0.009	-0.015	0.074	-0.103	RC3H1	0.027	~p_tau+(1|manifest)+cngeneson+pc_mito+brain_region+Sex	1.185	2502.000
ENSG00000049618	0.674	-0.015	0.048	-0.077	ARID1B	0.721	~p_tau+(1|manifest)+cngeneson+pc_mito+brain_region+Sex	0.936	2700.000
ENSG00000079482	0.876	-0.015	0.089	-0.119	OPHN1	0.894	~p_tau+(1|manifest)+cngeneson+pc_mito+brain_region+Sex	0.708	2907.000
ENSG00000253729	0.002	-0.015	0.096	-0.126	PRKDC	0.009	~p_tau+(1|manifest)+cngeneson+pc_mito+brain_region+Sex	1.543	2177.000
ENSG00000122707	0.014	-0.015	0.075	-0.105	RECK	0.037	~p_tau+(1|manifest)+cngeneson+pc_mito+brain_region+Sex	0.954	2690.000
ENSG00000186566	0.270	-0.015	0.085	-0.115	GPATCH8	0.352	~p_tau+(1|manifest)+cngeneson+pc_mito+brain_region+Sex	1.402	2309.000
ENSG00000137693	0.839	-0.015	0.144	-0.174	YAP1	0.860	~p_tau+(1|manifest)+cngeneson+pc_mito+brain_region+Sex	3.819	963.000
ENSG00000138756	0.703	-0.015	0.117	-0.148	BMP2K	0.748	~p_tau+(1|manifest)+cngeneson+pc_mito+brain_region+Sex	4.622	764.000
ENSG00000076108	0.055	-0.015	0.099	-0.130	BAZ2A	0.103	~p_tau+(1|manifest)+cngeneson+pc_mito+brain_region+Sex	1.197	2485.000
ENSG00000115761	0.011	-0.015	0.066	-0.097	NOL10	0.032	~p_tau+(1|manifest)+cngeneson+pc_mito+brain_region+Sex	1.317	2381.000
ENSG00000061936	0.017	-0.015	0.119	-0.150	SFSWAP	0.042	~p_tau+(1|manifest)+cngeneson+pc_mito+brain_region+Sex	2.141	1744.000
ENSG00000129003	0.045	-0.016	0.069	-0.101	VPS13C	0.087	~p_tau+(1|manifest)+cngeneson+pc_mito+brain_region+Sex	0.818	2813.000
ENSG00000183530	0.023	-0.016	0.110	-0.142	PRR14L	0.054	~p_tau+(1|manifest)+cngeneson+pc_mito+brain_region+Sex	0.613	2957.000
ENSG00000157077	0.004	-0.016	0.072	-0.105	ZFYVE9	0.016	~p_tau+(1|manifest)+cngeneson+pc_mito+brain_region+Sex	0.628	2950.000
ENSG00000177311	0.004	-0.017	0.123	-0.156	ZBTB38	0.015	~p_tau+(1|manifest)+cngeneson+pc_mito+brain_region+Sex	1.154	2528.000
ENSG00000150093	0.307	-0.017	0.209	-0.242	ITGB1	0.388	~p_tau+(1|manifest)+cngeneson+pc_mito+brain_region+Sex	5.719	584.000
ENSG00000006607	0.015	-0.017	0.092	-0.126	FARP2	0.040	~p_tau+(1|manifest)+cngeneson+pc_mito+brain_region+Sex	1.327	2374.000
ENSG00000156958	0.021	-0.017	0.061	-0.094	GALK2	0.049	~p_tau+(1|manifest)+cngeneson+pc_mito+brain_region+Sex	1.142	2542.000
ENSG00000175354	0.044	-0.017	0.225	-0.259	PTPN2	0.087	~p_tau+(1|manifest)+cngeneson+pc_mito+brain_region+Sex	2.723	1391.000
ENSG00000085382	0.013	-0.017	0.103	-0.137	HACE1	0.035	~p_tau+(1|manifest)+cngeneson+pc_mito+brain_region+Sex	1.145	2539.000
ENSG00000105220	0.339	-0.017	0.315	-0.349	GPI	0.417	~p_tau+(1|manifest)+cngeneson+pc_mito+brain_region+Sex	3.898	930.000
ENSG00000110693	0.924	-0.017	0.069	-0.103	SOX6	0.936	~p_tau+(1|manifest)+cngeneson+pc_mito+brain_region+Sex	1.883	1928.000
ENSG00000144908	0.777	-0.017	0.169	-0.204	ALDH1L1	0.809	~p_tau+(1|manifest)+cngeneson+pc_mito+brain_region+Sex	6.085	537.000
ENSG00000165338	0.114	-0.017	0.133	-0.168	HECTD2	0.180	~p_tau+(1|manifest)+cngeneson+pc_mito+brain_region+Sex	2.743	1385.000
ENSG00000067177	0.329	-0.017	0.320	-0.354	PHKA1	0.408	~p_tau+(1|manifest)+cngeneson+pc_mito+brain_region+Sex	6.909	455.000
ENSG00000243317	0.017	-0.017	0.165	-0.200	STMP1	0.043	~p_tau+(1|manifest)+cngeneson+pc_mito+brain_region+Sex	1.375	2335.000
ENSG00000196975	0.144	-0.018	0.139	-0.174	ANXA4	0.215	~p_tau+(1|manifest)+cngeneson+pc_mito+brain_region+Sex	1.735	2039.000
ENSG00000141376	0.952	-0.018	0.151	-0.186	BCAS3	0.961	~p_tau+(1|manifest)+cngeneson+pc_mito+brain_region+Sex	3.210	1171.000
ENSG00000119698	0.025	-0.018	0.202	-0.238	PPP4R4	0.057	~p_tau+(1|manifest)+cngeneson+pc_mito+brain_region+Sex	1.448	2264.000
ENSG00000119242	0.034	-0.018	0.131	-0.168	CCDC92	0.071	~p_tau+(1|manifest)+cngeneson+pc_mito+brain_region+Sex	1.352	2352.000
ENSG00000009954	0.005	-0.019	0.160	-0.197	BAZ1B	0.020	~p_tau+(1|manifest)+cngeneson+pc_mito+brain_region+Sex	1.693	2073.000
ENSG00000009307	0.004	-0.019	0.073	-0.110	CSDE1	0.016	~p_tau+(1|manifest)+cngeneson+pc_mito+brain_region+Sex	1.100	2568.000
ENSG00000125107	0.002	-0.019	0.101	-0.138	CNOT1	0.009	~p_tau+(1|manifest)+cngeneson+pc_mito+brain_region+Sex	1.732	2043.000
ENSG00000006468	0.278	-0.019	0.293	-0.330	ETV1	0.359	~p_tau+(1|manifest)+cngeneson+pc_mito+brain_region+Sex	3.695	1004.000
ENSG00000107249	0.978	-0.019	0.185	-0.222	GLIS3	0.983	~p_tau+(1|manifest)+cngeneson+pc_mito+brain_region+Sex	25.462	27.000
ENSG00000138674	0.002	-0.019	0.073	-0.111	SEC31A	0.009	~p_tau+(1|manifest)+cngeneson+pc_mito+brain_region+Sex	1.402	2308.000
ENSG00000264522	0.589	-0.019	0.305	-0.343	OTUD7B	0.645	~p_tau+(1|manifest)+cngeneson+pc_mito+brain_region+Sex	3.162	1193.000
ENSG00000123600	0.346	-0.020	0.120	-0.160	METTL8	0.424	~p_tau+(1|manifest)+cngeneson+pc_mito+brain_region+Sex	1.826	1967.000
ENSG00000111785	0.196	-0.020	0.133	-0.172	RIC8B	0.271	~p_tau+(1|manifest)+cngeneson+pc_mito+brain_region+Sex	2.050	1803.000
ENSG00000054118	0.007	-0.020	0.084	-0.123	THRAP3	0.024	~p_tau+(1|manifest)+cngeneson+pc_mito+brain_region+Sex	1.721	2052.000
ENSG00000124151	0.017	-0.020	0.092	-0.131	NCOA3	0.042	~p_tau+(1|manifest)+cngeneson+pc_mito+brain_region+Sex	1.085	2578.000
ENSG00000140836	0.942	-0.020	0.109	-0.149	ZFHX3	0.952	~p_tau+(1|manifest)+cngeneson+pc_mito+brain_region+Sex	2.598	1456.000
ENSG00000270629	0.111	-0.020	0.217	-0.257	NBPF14	0.176	~p_tau+(1|manifest)+cngeneson+pc_mito+brain_region+Sex	3.161	1194.000
ENSG00000198586	0.683	-0.020	0.130	-0.171	TLK1	0.729	~p_tau+(1|manifest)+cngeneson+pc_mito+brain_region+Sex	4.110	884.000
ENSG00000071794	0.086	-0.020	0.124	-0.164	HLTF	0.145	~p_tau+(1|manifest)+cngeneson+pc_mito+brain_region+Sex	1.194	2490.000
ENSG00000169084	0.336	-0.021	0.179	-0.220	DHRSX	0.415	~p_tau+(1|manifest)+cngeneson+pc_mito+brain_region+Sex	4.111	883.000
ENSG00000090975	0.652	-0.021	0.307	-0.349	PITPNM2	0.701	~p_tau+(1|manifest)+cngeneson+pc_mito+brain_region+Sex	7.866	366.000
ENSG00000151320	0.957	-0.021	0.490	-0.531	AKAP6	0.965	~p_tau+(1|manifest)+cngeneson+pc_mito+brain_region+Sex	4.479	800.000
ENSG00000175215	0.059	-0.021	0.123	-0.165	CTDSP2	0.109	~p_tau+(1|manifest)+cngeneson+pc_mito+brain_region+Sex	1.052	2607.000
ENSG00000128923	0.002	-0.021	0.072	-0.114	MINDY2	0.009	~p_tau+(1|manifest)+cngeneson+pc_mito+brain_region+Sex	0.859	2772.000
ENSG00000172461	0.913	-0.021	0.095	-0.137	FUT9	0.928	~p_tau+(1|manifest)+cngeneson+pc_mito+brain_region+Sex	3.666	1013.000
ENSG00000166575	0.725	-0.021	0.148	-0.190	TMEM135	0.766	~p_tau+(1|manifest)+cngeneson+pc_mito+brain_region+Sex	3.772	980.000
ENSG00000162670	0.994	-0.021	0.424	-0.466	BRINP3	0.996	~p_tau+(1|manifest)+cngeneson+pc_mito+brain_region+Sex	8.345	336.000
ENSG00000125633	0.027	-0.022	0.101	-0.144	CCDC93	0.060	~p_tau+(1|manifest)+cngeneson+pc_mito+brain_region+Sex	1.963	1872.000
ENSG00000099968	0.053	-0.022	0.126	-0.169	BCL2L13	0.101	~p_tau+(1|manifest)+cngeneson+pc_mito+brain_region+Sex	1.771	2001.000
ENSG00000204128	0.571	-0.022	0.432	-0.475	C2orf72	0.629	~p_tau+(1|manifest)+cngeneson+pc_mito+brain_region+Sex	14.212	117.000
ENSG00000173757	0.071	-0.022	0.112	-0.156	STAT5B	0.126	~p_tau+(1|manifest)+cngeneson+pc_mito+brain_region+Sex	1.645	2104.000
ENSG00000153944	0.639	-0.022	0.088	-0.133	MSI2	0.689	~p_tau+(1|manifest)+cngeneson+pc_mito+brain_region+Sex	9.482	288.000
ENSG00000151893	0.005	-0.022	0.076	-0.121	CACUL1	0.020	~p_tau+(1|manifest)+cngeneson+pc_mito+brain_region+Sex	1.233	2455.000
ENSG00000088808	0.048	-0.023	0.119	-0.164	PPP1R13B	0.093	~p_tau+(1|manifest)+cngeneson+pc_mito+brain_region+Sex	1.288	2418.000
ENSG00000174197	0.240	-0.023	0.147	-0.192	MGA	0.319	~p_tau+(1|manifest)+cngeneson+pc_mito+brain_region+Sex	4.167	867.000
ENSG00000111913	0.144	-0.023	0.238	-0.283	RIPOR2	0.215	~p_tau+(1|manifest)+cngeneson+pc_mito+brain_region+Sex	3.778	976.000
ENSG00000087266	0.178	-0.023	0.245	-0.290	SH3BP2	0.253	~p_tau+(1|manifest)+cngeneson+pc_mito+brain_region+Sex	3.348	1117.000
ENSG00000171824	0.034	-0.023	0.242	-0.288	EXOSC10	0.071	~p_tau+(1|manifest)+cngeneson+pc_mito+brain_region+Sex	3.094	1221.000
ENSG00000056487	0.595	-0.023	0.145	-0.191	PHF21B	0.651	~p_tau+(1|manifest)+cngeneson+pc_mito+brain_region+Sex	1.525	2196.000
ENSG00000129353	0.129	-0.023	0.216	-0.262	SLC44A2	0.197	~p_tau+(1|manifest)+cngeneson+pc_mito+brain_region+Sex	1.743	2029.000
ENSG00000143772	0.937	-0.023	0.138	-0.184	ITPKB	0.948	~p_tau+(1|manifest)+cngeneson+pc_mito+brain_region+Sex	10.783	221.000
ENSG00000132964	0.113	-0.023	0.114	-0.161	CDK8	0.178	~p_tau+(1|manifest)+cngeneson+pc_mito+brain_region+Sex	1.910	1906.000
ENSG00000137770	0.008	-0.024	0.074	-0.121	CTDSPL2	0.026	~p_tau+(1|manifest)+cngeneson+pc_mito+brain_region+Sex	1.406	2303.000
ENSG00000140199	0.063	-0.024	0.128	-0.175	SLC12A6	0.115	~p_tau+(1|manifest)+cngeneson+pc_mito+brain_region+Sex	1.744	2028.000
ENSG00000130227	0.000	-0.024	0.075	-0.123	XPO7	0.001	~p_tau+(1|manifest)+cngeneson+pc_mito+brain_region+Sex	0.356	3086.000
ENSG00000114098	0.011	-0.024	0.098	-0.145	ARMC8	0.032	~p_tau+(1|manifest)+cngeneson+pc_mito+brain_region+Sex	1.616	2131.000
ENSG00000174437	0.084	-0.024	0.123	-0.170	ATP2A2	0.142	~p_tau+(1|manifest)+cngeneson+pc_mito+brain_region+Sex	1.747	2023.000
ENSG00000113194	0.001	-0.024	0.070	-0.118	FAF2	0.004	~p_tau+(1|manifest)+cngeneson+pc_mito+brain_region+Sex	1.194	2492.000
ENSG00000153015	0.301	-0.024	0.154	-0.202	CWC27	0.382	~p_tau+(1|manifest)+cngeneson+pc_mito+brain_region+Sex	1.038	2614.000
ENSG00000145390	0.401	-0.024	0.185	-0.234	USP53	0.477	~p_tau+(1|manifest)+cngeneson+pc_mito+brain_region+Sex	4.409	812.000
ENSG00000197121	0.461	-0.025	0.198	-0.248	PGAP1	0.532	~p_tau+(1|manifest)+cngeneson+pc_mito+brain_region+Sex	3.742	994.000
ENSG00000038382	0.938	-0.025	0.146	-0.196	TRIO	0.948	~p_tau+(1|manifest)+cngeneson+pc_mito+brain_region+Sex	15.129	96.000
ENSG00000180357	0.740	-0.025	0.160	-0.210	ZNF609	0.778	~p_tau+(1|manifest)+cngeneson+pc_mito+brain_region+Sex	2.690	1409.000
ENSG00000116171	0.044	-0.025	0.118	-0.169	SCP2	0.087	~p_tau+(1|manifest)+cngeneson+pc_mito+brain_region+Sex	1.364	2344.000
ENSG00000163939	0.000	-0.025	0.062	-0.113	PBRM1	0.001	~p_tau+(1|manifest)+cngeneson+pc_mito+brain_region+Sex	1.912	1905.000
ENSG00000168036	0.339	-0.025	0.184	-0.235	CTNNB1	0.417	~p_tau+(1|manifest)+cngeneson+pc_mito+brain_region+Sex	1.508	2211.000
ENSG00000172164	0.956	-0.026	0.411	-0.462	SNTB1	0.964	~p_tau+(1|manifest)+cngeneson+pc_mito+brain_region+Sex	2.447	1550.000
ENSG00000152104	0.083	-0.026	0.074	-0.126	PTPN14	0.142	~p_tau+(1|manifest)+cngeneson+pc_mito+brain_region+Sex	0.788	2841.000
ENSG00000060749	0.005	-0.026	0.057	-0.109	QSER1	0.018	~p_tau+(1|manifest)+cngeneson+pc_mito+brain_region+Sex	0.875	2762.000
ENSG00000162174	0.570	-0.026	0.315	-0.367	ASRGL1	0.629	~p_tau+(1|manifest)+cngeneson+pc_mito+brain_region+Sex	8.024	355.000
ENSG00000113658	0.049	-0.026	0.073	-0.126	SMAD5	0.095	~p_tau+(1|manifest)+cngeneson+pc_mito+brain_region+Sex	0.713	2904.000
ENSG00000144468	0.206	-0.027	0.148	-0.202	RHBDD1	0.282	~p_tau+(1|manifest)+cngeneson+pc_mito+brain_region+Sex	1.134	2548.000
ENSG00000182732	0.967	-0.027	0.308	-0.361	RGS6	0.973	~p_tau+(1|manifest)+cngeneson+pc_mito+brain_region+Sex	6.200	524.000
ENSG00000128607	0.062	-0.027	0.127	-0.181	KLHDC10	0.113	~p_tau+(1|manifest)+cngeneson+pc_mito+brain_region+Sex	1.451	2261.000
ENSG00000129657	0.624	-0.027	0.248	-0.302	SEC14L1	0.677	~p_tau+(1|manifest)+cngeneson+pc_mito+brain_region+Sex	4.607	767.000
ENSG00000197776	0.104	-0.027	0.134	-0.188	KLHDC1	0.167	~p_tau+(1|manifest)+cngeneson+pc_mito+brain_region+Sex	2.369	1592.000
ENSG00000130177	0.009	-0.027	0.095	-0.149	CDC16	0.026	~p_tau+(1|manifest)+cngeneson+pc_mito+brain_region+Sex	1.615	2132.000
ENSG00000163624	0.348	-0.027	0.207	-0.261	CDS1	0.427	~p_tau+(1|manifest)+cngeneson+pc_mito+brain_region+Sex	1.761	2008.000
ENSG00000143776	0.708	-0.028	0.098	-0.153	CDC42BPA	0.752	~p_tau+(1|manifest)+cngeneson+pc_mito+brain_region+Sex	3.117	1211.000
ENSG00000165995	0.970	-0.028	0.225	-0.280	CACNB2	0.976	~p_tau+(1|manifest)+cngeneson+pc_mito+brain_region+Sex	6.660	470.000
ENSG00000166734	0.317	-0.028	0.095	-0.150	CASC4	0.396	~p_tau+(1|manifest)+cngeneson+pc_mito+brain_region+Sex	2.166	1721.000
ENSG00000112200	0.072	-0.028	0.150	-0.206	ZNF451	0.126	~p_tau+(1|manifest)+cngeneson+pc_mito+brain_region+Sex	2.397	1575.000
ENSG00000118420	0.279	-0.028	0.029	-0.086	UBE3D	0.360	~p_tau+(1|manifest)+cngeneson+pc_mito+brain_region+Sex	0.819	2812.000
ENSG00000102893	0.088	-0.028	0.098	-0.155	PHKB	0.148	~p_tau+(1|manifest)+cngeneson+pc_mito+brain_region+Sex	0.849	2782.000
ENSG00000163629	0.954	-0.028	0.169	-0.225	PTPN13	0.963	~p_tau+(1|manifest)+cngeneson+pc_mito+brain_region+Sex	5.395	636.000
ENSG00000121741	0.212	-0.028	0.118	-0.174	ZMYM2	0.289	~p_tau+(1|manifest)+cngeneson+pc_mito+brain_region+Sex	1.476	2241.000
ENSG00000214013	0.089	-0.029	0.128	-0.185	GANC	0.149	~p_tau+(1|manifest)+cngeneson+pc_mito+brain_region+Sex	1.546	2173.000
ENSG00000165868	0.196	-0.029	0.140	-0.197	HSPA12A	0.272	~p_tau+(1|manifest)+cngeneson+pc_mito+brain_region+Sex	0.454	3043.000
ENSG00000067955	0.145	-0.029	0.160	-0.218	CBFB	0.215	~p_tau+(1|manifest)+cngeneson+pc_mito+brain_region+Sex	0.653	2936.000
ENSG00000183066	0.282	-0.029	0.312	-0.370	WBP2NL	0.363	~p_tau+(1|manifest)+cngeneson+pc_mito+brain_region+Sex	5.236	663.000
ENSG00000078018	0.678	-0.029	0.531	-0.589	MAP2	0.723	~p_tau+(1|manifest)+cngeneson+pc_mito+brain_region+Sex	11.120	204.000
ENSG00000178662	0.436	-0.029	0.205	-0.263	CSRNP3	0.508	~p_tau+(1|manifest)+cngeneson+pc_mito+brain_region+Sex	1.506	2214.000
ENSG00000160584	0.568	-0.029	0.032	-0.091	SIK3	0.628	~p_tau+(1|manifest)+cngeneson+pc_mito+brain_region+Sex	2.204	1700.000
ENSG00000067798	0.891	-0.030	0.102	-0.161	NAV3	0.907	~p_tau+(1|manifest)+cngeneson+pc_mito+brain_region+Sex	11.091	206.000
ENSG00000138036	0.027	-0.030	0.141	-0.201	DYNC2LI1	0.060	~p_tau+(1|manifest)+cngeneson+pc_mito+brain_region+Sex	2.327	1616.000
ENSG00000074047	0.585	-0.030	0.293	-0.353	GLI2	0.642	~p_tau+(1|manifest)+cngeneson+pc_mito+brain_region+Sex	1.291	2412.000
ENSG00000196313	0.007	-0.030	0.030	-0.091	POM121	0.022	~p_tau+(1|manifest)+cngeneson+pc_mito+brain_region+Sex	0.306	3103.000
ENSG00000139946	0.667	-0.030	0.332	-0.393	PELI2	0.714	~p_tau+(1|manifest)+cngeneson+pc_mito+brain_region+Sex	4.214	855.000
ENSG00000249240	0.537	-0.031	0.349	-0.410	AC069368.1	0.600	~p_tau+(1|manifest)+cngeneson+pc_mito+brain_region+Sex	2.924	1306.000
ENSG00000033327	0.825	-0.031	0.408	-0.469	GAB2	0.849	~p_tau+(1|manifest)+cngeneson+pc_mito+brain_region+Sex	4.638	762.000
ENSG00000163913	0.025	-0.031	0.105	-0.166	IFT122	0.057	~p_tau+(1|manifest)+cngeneson+pc_mito+brain_region+Sex	0.788	2842.000
ENSG00000173226	0.011	-0.031	0.086	-0.148	IQCB1	0.031	~p_tau+(1|manifest)+cngeneson+pc_mito+brain_region+Sex	1.320	2378.000
ENSG00000104408	0.547	-0.031	0.268	-0.331	EIF3E	0.608	~p_tau+(1|manifest)+cngeneson+pc_mito+brain_region+Sex	3.030	1250.000
ENSG00000116731	0.055	-0.031	0.042	-0.104	PRDM2	0.104	~p_tau+(1|manifest)+cngeneson+pc_mito+brain_region+Sex	0.335	3093.000
ENSG00000149187	0.357	-0.031	0.100	-0.163	CELF1	0.435	~p_tau+(1|manifest)+cngeneson+pc_mito+brain_region+Sex	0.912	2725.000
ENSG00000116497	0.015	-0.032	0.092	-0.155	S100PBP	0.039	~p_tau+(1|manifest)+cngeneson+pc_mito+brain_region+Sex	1.841	1954.000
ENSG00000138760	0.016	-0.032	0.100	-0.163	SCARB2	0.040	~p_tau+(1|manifest)+cngeneson+pc_mito+brain_region+Sex	1.156	2526.000
ENSG00000115966	0.047	-0.032	0.145	-0.209	ATF2	0.091	~p_tau+(1|manifest)+cngeneson+pc_mito+brain_region+Sex	0.481	3031.000
ENSG00000138802	0.000	-0.032	0.064	-0.128	SEC24B	0.000	~p_tau+(1|manifest)+cngeneson+pc_mito+brain_region+Sex	1.258	2437.000
ENSG00000066427	0.038	-0.032	0.198	-0.262	ATXN3	0.078	~p_tau+(1|manifest)+cngeneson+pc_mito+brain_region+Sex	2.386	1581.000
ENSG00000017260	0.814	-0.032	0.393	-0.458	ATP2C1	0.840	~p_tau+(1|manifest)+cngeneson+pc_mito+brain_region+Sex	11.207	202.000
ENSG00000171634	0.490	-0.032	0.070	-0.134	BPTF	0.557	~p_tau+(1|manifest)+cngeneson+pc_mito+brain_region+Sex	4.176	862.000
ENSG00000123124	0.006	-0.033	0.120	-0.185	WWP1	0.022	~p_tau+(1|manifest)+cngeneson+pc_mito+brain_region+Sex	0.384	3072.000
ENSG00000009335	0.002	-0.033	0.114	-0.180	UBE3C	0.009	~p_tau+(1|manifest)+cngeneson+pc_mito+brain_region+Sex	1.345	2360.000
ENSG00000159459	0.015	-0.033	0.059	-0.125	UBR1	0.040	~p_tau+(1|manifest)+cngeneson+pc_mito+brain_region+Sex	1.742	2030.000
ENSG00000171044	0.302	-0.033	0.469	-0.535	XKR6	0.383	~p_tau+(1|manifest)+cngeneson+pc_mito+brain_region+Sex	1.664	2091.000
ENSG00000118762	0.654	-0.033	0.284	-0.350	PKD2	0.702	~p_tau+(1|manifest)+cngeneson+pc_mito+brain_region+Sex	8.588	315.000
ENSG00000134871	0.940	-0.033	0.213	-0.279	COL4A2	0.950	~p_tau+(1|manifest)+cngeneson+pc_mito+brain_region+Sex	1.665	2089.000
ENSG00000171316	0.431	-0.033	0.119	-0.185	CHD7	0.504	~p_tau+(1|manifest)+cngeneson+pc_mito+brain_region+Sex	3.329	1124.000
ENSG00000149218	0.080	-0.033	0.200	-0.267	ENDOD1	0.137	~p_tau+(1|manifest)+cngeneson+pc_mito+brain_region+Sex	5.657	595.000
ENSG00000150893	0.982	-0.033	0.667	-0.734	FREM2	0.987	~p_tau+(1|manifest)+cngeneson+pc_mito+brain_region+Sex	21.256	43.000
ENSG00000033178	0.001	-0.034	0.104	-0.171	UBA6	0.004	~p_tau+(1|manifest)+cngeneson+pc_mito+brain_region+Sex	1.840	1955.000
ENSG00000198752	0.004	-0.034	0.106	-0.173	CDC42BPB	0.014	~p_tau+(1|manifest)+cngeneson+pc_mito+brain_region+Sex	1.541	2178.000
ENSG00000266714	0.225	-0.034	0.300	-0.368	MYO15B	0.302	~p_tau+(1|manifest)+cngeneson+pc_mito+brain_region+Sex	3.897	931.000
ENSG00000070882	0.351	-0.034	0.204	-0.272	OSBPL3	0.430	~p_tau+(1|manifest)+cngeneson+pc_mito+brain_region+Sex	1.888	1925.000
ENSG00000170921	0.504	-0.034	0.158	-0.227	TANC2	0.571	~p_tau+(1|manifest)+cngeneson+pc_mito+brain_region+Sex	2.476	1529.000
ENSG00000072364	0.118	-0.035	0.150	-0.219	AFF4	0.183	~p_tau+(1|manifest)+cngeneson+pc_mito+brain_region+Sex	3.358	1111.000
ENSG00000130741	0.039	-0.035	0.127	-0.197	EIF2S3	0.078	~p_tau+(1|manifest)+cngeneson+pc_mito+brain_region+Sex	2.586	1465.000
ENSG00000170471	0.001	-0.035	0.043	-0.113	RALGAPB	0.006	~p_tau+(1|manifest)+cngeneson+pc_mito+brain_region+Sex	1.194	2491.000
ENSG00000117868	0.296	-0.035	0.136	-0.206	ESYT2	0.378	~p_tau+(1|manifest)+cngeneson+pc_mito+brain_region+Sex	2.689	1411.000
ENSG00000054267	0.020	-0.035	0.068	-0.138	ARID4B	0.047	~p_tau+(1|manifest)+cngeneson+pc_mito+brain_region+Sex	1.588	2152.000
ENSG00000188647	0.793	-0.035	0.295	-0.366	PTAR1	0.823	~p_tau+(1|manifest)+cngeneson+pc_mito+brain_region+Sex	4.805	727.000
ENSG00000156052	0.571	-0.035	0.030	-0.101	GNAQ	0.629	~p_tau+(1|manifest)+cngeneson+pc_mito+brain_region+Sex	2.475	1530.000
ENSG00000213639	0.035	-0.036	0.175	-0.246	PPP1CB	0.073	~p_tau+(1|manifest)+cngeneson+pc_mito+brain_region+Sex	2.226	1685.000
ENSG00000116539	0.722	-0.036	0.052	-0.124	ASH1L	0.764	~p_tau+(1|manifest)+cngeneson+pc_mito+brain_region+Sex	3.009	1258.000
ENSG00000172262	0.078	-0.036	0.107	-0.179	ZNF131	0.135	~p_tau+(1|manifest)+cngeneson+pc_mito+brain_region+Sex	2.105	1767.000
ENSG00000150527	0.007	-0.036	0.087	-0.160	MIA2	0.023	~p_tau+(1|manifest)+cngeneson+pc_mito+brain_region+Sex	1.601	2143.000
ENSG00000132589	0.297	-0.036	0.179	-0.251	FLOT2	0.378	~p_tau+(1|manifest)+cngeneson+pc_mito+brain_region+Sex	1.440	2270.000
ENSG00000003402	0.001	-0.036	0.121	-0.193	CFLAR	0.004	~p_tau+(1|manifest)+cngeneson+pc_mito+brain_region+Sex	0.842	2789.000
ENSG00000277149	0.008	-0.036	0.035	-0.108	TYW1B	0.026	~p_tau+(1|manifest)+cngeneson+pc_mito+brain_region+Sex	0.732	2886.000
ENSG00000107758	0.006	-0.036	0.109	-0.182	PPP3CB	0.020	~p_tau+(1|manifest)+cngeneson+pc_mito+brain_region+Sex	2.195	1706.000
ENSG00000145362	0.805	-0.037	0.085	-0.158	ANK2	0.833	~p_tau+(1|manifest)+cngeneson+pc_mito+brain_region+Sex	7.273	413.000
ENSG00000036530	0.062	-0.037	0.206	-0.280	CYP46A1	0.113	~p_tau+(1|manifest)+cngeneson+pc_mito+brain_region+Sex	2.595	1459.000
ENSG00000154930	0.972	-0.037	0.427	-0.501	ACSS1	0.977	~p_tau+(1|manifest)+cngeneson+pc_mito+brain_region+Sex	12.618	156.000
ENSG00000198420	0.033	-0.037	0.070	-0.143	TCAF1	0.070	~p_tau+(1|manifest)+cngeneson+pc_mito+brain_region+Sex	1.331	2370.000
ENSG00000113645	0.791	-0.037	0.123	-0.197	WWC1	0.821	~p_tau+(1|manifest)+cngeneson+pc_mito+brain_region+Sex	3.916	928.000
ENSG00000140153	0.211	-0.037	0.084	-0.159	WDR20	0.288	~p_tau+(1|manifest)+cngeneson+pc_mito+brain_region+Sex	1.708	2060.000
ENSG00000078070	0.015	-0.037	0.098	-0.173	MCCC1	0.039	~p_tau+(1|manifest)+cngeneson+pc_mito+brain_region+Sex	1.300	2403.000
ENSG00000165671	0.041	-0.038	0.085	-0.160	NSD1	0.082	~p_tau+(1|manifest)+cngeneson+pc_mito+brain_region+Sex	1.624	2122.000
ENSG00000097033	0.085	-0.038	0.125	-0.200	SH3GLB1	0.144	~p_tau+(1|manifest)+cngeneson+pc_mito+brain_region+Sex	2.831	1346.000
ENSG00000165478	0.821	-0.038	0.210	-0.286	HEPACAM	0.845	~p_tau+(1|manifest)+cngeneson+pc_mito+brain_region+Sex	7.846	368.000
ENSG00000163393	0.776	-0.038	0.151	-0.227	SLC22A15	0.808	~p_tau+(1|manifest)+cngeneson+pc_mito+brain_region+Sex	1.783	1997.000
ENSG00000164506	0.703	-0.038	0.112	-0.189	STXBP5	0.748	~p_tau+(1|manifest)+cngeneson+pc_mito+brain_region+Sex	5.955	550.000
ENSG00000159592	0.030	-0.038	0.099	-0.176	GPBP1L1	0.065	~p_tau+(1|manifest)+cngeneson+pc_mito+brain_region+Sex	2.526	1490.000
ENSG00000166979	0.864	-0.038	0.233	-0.310	EVA1C	0.882	~p_tau+(1|manifest)+cngeneson+pc_mito+brain_region+Sex	4.126	880.000
ENSG00000187391	0.806	-0.038	0.077	-0.154	MAGI2	0.833	~p_tau+(1|manifest)+cngeneson+pc_mito+brain_region+Sex	11.895	177.000
ENSG00000078747	0.030	-0.038	0.141	-0.218	ITCH	0.065	~p_tau+(1|manifest)+cngeneson+pc_mito+brain_region+Sex	3.348	1118.000
ENSG00000065615	0.013	-0.038	0.034	-0.111	CYB5R4	0.036	~p_tau+(1|manifest)+cngeneson+pc_mito+brain_region+Sex	0.729	2888.000
ENSG00000140382	0.137	-0.039	0.081	-0.158	HMG20A	0.206	~p_tau+(1|manifest)+cngeneson+pc_mito+brain_region+Sex	1.154	2529.000
ENSG00000204130	0.006	-0.039	0.100	-0.177	RUFY2	0.021	~p_tau+(1|manifest)+cngeneson+pc_mito+brain_region+Sex	0.734	2883.000
ENSG00000149970	0.881	-0.039	0.393	-0.471	CNKSR2	0.899	~p_tau+(1|manifest)+cngeneson+pc_mito+brain_region+Sex	6.580	478.000
ENSG00000146535	0.705	-0.039	0.230	-0.308	GNA12	0.749	~p_tau+(1|manifest)+cngeneson+pc_mito+brain_region+Sex	6.215	521.000
ENSG00000101542	0.794	-0.039	0.106	-0.184	CDH20	0.823	~p_tau+(1|manifest)+cngeneson+pc_mito+brain_region+Sex	12.382	166.000
ENSG00000135931	0.039	-0.039	0.086	-0.164	ARMC9	0.079	~p_tau+(1|manifest)+cngeneson+pc_mito+brain_region+Sex	0.774	2854.000
ENSG00000109670	0.844	-0.040	0.127	-0.206	FBXW7	0.866	~p_tau+(1|manifest)+cngeneson+pc_mito+brain_region+Sex	3.532	1050.000
ENSG00000151835	0.157	-0.040	0.244	-0.323	SACS	0.228	~p_tau+(1|manifest)+cngeneson+pc_mito+brain_region+Sex	3.272	1145.000
ENSG00000005812	0.001	-0.040	0.031	-0.110	FBXL3	0.004	~p_tau+(1|manifest)+cngeneson+pc_mito+brain_region+Sex	0.753	2866.000
ENSG00000198795	0.893	-0.040	0.128	-0.208	ZNF521	0.908	~p_tau+(1|manifest)+cngeneson+pc_mito+brain_region+Sex	2.275	1655.000
ENSG00000172765	0.298	-0.040	0.065	-0.145	TMCC1	0.379	~p_tau+(1|manifest)+cngeneson+pc_mito+brain_region+Sex	2.156	1730.000
ENSG00000189180	0.018	-0.040	0.128	-0.209	ZNF33A	0.045	~p_tau+(1|manifest)+cngeneson+pc_mito+brain_region+Sex	2.006	1836.000
ENSG00000107164	0.006	-0.040	0.055	-0.136	FUBP3	0.020	~p_tau+(1|manifest)+cngeneson+pc_mito+brain_region+Sex	0.876	2760.000
ENSG00000083168	0.360	-0.041	0.129	-0.211	KAT6A	0.437	~p_tau+(1|manifest)+cngeneson+pc_mito+brain_region+Sex	3.451	1076.000
ENSG00000144959	0.203	-0.041	0.092	-0.174	NCEH1	0.279	~p_tau+(1|manifest)+cngeneson+pc_mito+brain_region+Sex	1.155	2527.000
ENSG00000113761	0.031	-0.041	0.158	-0.240	ZNF346	0.066	~p_tau+(1|manifest)+cngeneson+pc_mito+brain_region+Sex	2.111	1764.000
ENSG00000136478	0.388	-0.041	0.224	-0.307	TEX2	0.465	~p_tau+(1|manifest)+cngeneson+pc_mito+brain_region+Sex	4.383	824.000
ENSG00000180881	0.783	-0.041	0.145	-0.227	CAPS2	0.813	~p_tau+(1|manifest)+cngeneson+pc_mito+brain_region+Sex	3.132	1202.000
ENSG00000134138	0.631	-0.041	0.048	-0.130	MEIS2	0.683	~p_tau+(1|manifest)+cngeneson+pc_mito+brain_region+Sex	3.327	1125.000
ENSG00000078142	0.121	-0.042	0.112	-0.195	PIK3C3	0.187	~p_tau+(1|manifest)+cngeneson+pc_mito+brain_region+Sex	1.190	2498.000
ENSG00000144451	0.487	-0.042	0.130	-0.214	SPAG16	0.555	~p_tau+(1|manifest)+cngeneson+pc_mito+brain_region+Sex	1.014	2635.000
ENSG00000033170	0.910	-0.042	0.159	-0.243	FUT8	0.925	~p_tau+(1|manifest)+cngeneson+pc_mito+brain_region+Sex	5.159	671.000
ENSG00000100888	0.017	-0.042	0.040	-0.124	CHD8	0.042	~p_tau+(1|manifest)+cngeneson+pc_mito+brain_region+Sex	1.081	2580.000
ENSG00000132912	0.005	-0.042	0.047	-0.131	DCTN4	0.018	~p_tau+(1|manifest)+cngeneson+pc_mito+brain_region+Sex	1.319	2380.000
ENSG00000183579	0.811	-0.042	0.091	-0.175	ZNRF3	0.838	~p_tau+(1|manifest)+cngeneson+pc_mito+brain_region+Sex	11.269	199.000
ENSG00000100234	0.560	-0.042	0.301	-0.386	TIMP3	0.620	~p_tau+(1|manifest)+cngeneson+pc_mito+brain_region+Sex	5.316	648.000
ENSG00000078124	0.118	-0.042	0.084	-0.169	ACER3	0.183	~p_tau+(1|manifest)+cngeneson+pc_mito+brain_region+Sex	1.753	2019.000
ENSG00000078725	0.913	-0.043	0.156	-0.242	BRINP1	0.927	~p_tau+(1|manifest)+cngeneson+pc_mito+brain_region+Sex	3.241	1159.000
ENSG00000088179	0.438	-0.043	0.155	-0.241	PTPN4	0.511	~p_tau+(1|manifest)+cngeneson+pc_mito+brain_region+Sex	3.873	937.000
ENSG00000141639	0.877	-0.043	0.129	-0.215	MAPK4	0.895	~p_tau+(1|manifest)+cngeneson+pc_mito+brain_region+Sex	3.858	945.000
ENSG00000151338	0.581	-0.043	0.200	-0.285	MIPOL1	0.639	~p_tau+(1|manifest)+cngeneson+pc_mito+brain_region+Sex	0.808	2825.000
ENSG00000169862	0.504	-0.043	0.031	-0.116	CTNND2	0.571	~p_tau+(1|manifest)+cngeneson+pc_mito+brain_region+Sex	2.697	1405.000
ENSG00000112530	0.968	-0.043	0.289	-0.375	PACRG	0.974	~p_tau+(1|manifest)+cngeneson+pc_mito+brain_region+Sex	1.357	2349.000
ENSG00000116141	0.086	-0.044	0.182	-0.269	MARK1	0.145	~p_tau+(1|manifest)+cngeneson+pc_mito+brain_region+Sex	2.102	1768.000
ENSG00000170390	0.588	-0.044	0.078	-0.166	DCLK2	0.644	~p_tau+(1|manifest)+cngeneson+pc_mito+brain_region+Sex	4.734	740.000
ENSG00000103544	0.001	-0.044	0.117	-0.206	VPS35L	0.007	~p_tau+(1|manifest)+cngeneson+pc_mito+brain_region+Sex	1.803	1986.000
ENSG00000104388	0.000	-0.044	0.049	-0.138	RAB2A	0.002	~p_tau+(1|manifest)+cngeneson+pc_mito+brain_region+Sex	0.820	2811.000
ENSG00000090863	0.411	-0.045	0.072	-0.161	GLG1	0.486	~p_tau+(1|manifest)+cngeneson+pc_mito+brain_region+Sex	3.065	1237.000
ENSG00000166747	0.000	-0.045	0.053	-0.142	AP1G1	0.004	~p_tau+(1|manifest)+cngeneson+pc_mito+brain_region+Sex	1.311	2387.000
ENSG00000153914	0.180	-0.045	0.132	-0.222	SREK1	0.254	~p_tau+(1|manifest)+cngeneson+pc_mito+brain_region+Sex	1.625	2120.000
ENSG00000205659	0.051	-0.045	0.089	-0.180	LIN52	0.097	~p_tau+(1|manifest)+cngeneson+pc_mito+brain_region+Sex	0.833	2797.000
ENSG00000085511	0.000	-0.045	0.039	-0.130	MAP3K4	0.003	~p_tau+(1|manifest)+cngeneson+pc_mito+brain_region+Sex	1.340	2363.000
ENSG00000055609	0.029	-0.046	0.010	-0.101	KMT2C	0.063	~p_tau+(1|manifest)+cngeneson+pc_mito+brain_region+Sex	2.676	1417.000
ENSG00000075945	0.001	-0.046	0.090	-0.182	KIFAP3	0.006	~p_tau+(1|manifest)+cngeneson+pc_mito+brain_region+Sex	0.619	2956.000
ENSG00000139737	0.804	-0.046	0.134	-0.226	SLAIN1	0.833	~p_tau+(1|manifest)+cngeneson+pc_mito+brain_region+Sex	6.603	476.000
ENSG00000112531	0.412	-0.046	0.032	-0.124	QKI	0.487	~p_tau+(1|manifest)+cngeneson+pc_mito+brain_region+Sex	2.822	1350.000
ENSG00000154845	0.001	-0.046	0.072	-0.164	PPP4R1	0.006	~p_tau+(1|manifest)+cngeneson+pc_mito+brain_region+Sex	0.552	2985.000

ENSG00000067141	0.772	-0.046	0.082	-0.174	NEO1	0.805	~p_tau+(1|manifest)+cngeneson+pc_mito+brain_region+Sex	9.769	271.000
ENSG00000120029	0.002	-0.046	0.082	-0.174	ARMH3	0.009	~p_tau+(1|manifest)+cngeneson+pc_mito+brain_region+Sex	1.493	2227.000
ENSG00000065559	0.121	-0.047	0.117	-0.211	MAP2K4	0.187	~p_tau+(1|manifest)+cngeneson+pc_mito+brain_region+Sex	1.387	2322.000
ENSG00000137504	0.093	-0.047	0.131	-0.225	CREB3L1	0.154	~p_tau+(1|manifest)+cngeneson+pc_mito+brain_region+Sex	3.037	1249.000
ENSG00000211584	0.077	-0.047	0.184	-0.279	SLC48A1	0.133	~p_tau+(1|manifest)+cngeneson+pc_mito+brain_region+Sex	2.263	1660.000
ENSG00000114439	0.394	-0.047	0.076	-0.171	BBX	0.470	~p_tau+(1|manifest)+cngeneson+pc_mito+brain_region+Sex	1.189	2499.000
ENSG00000136709	0.120	-0.047	0.119	-0.214	WDR33	0.186	~p_tau+(1|manifest)+cngeneson+pc_mito+brain_region+Sex	0.953	2691.000
ENSG00000087206	0.004	-0.048	0.084	-0.179	UIMC1	0.017	~p_tau+(1|manifest)+cngeneson+pc_mito+brain_region+Sex	1.646	2103.000
ENSG00000083312	0.000	-0.048	0.083	-0.179	TNPO1	0.003	~p_tau+(1|manifest)+cngeneson+pc_mito+brain_region+Sex	1.816	1978.000
ENSG00000102230	0.044	-0.048	0.195	-0.290	PCYT1B	0.087	~p_tau+(1|manifest)+cngeneson+pc_mito+brain_region+Sex	2.051	1801.000
ENSG00000128016	0.641	-0.048	0.519	-0.615	ZFP36	0.691	~p_tau+(1|manifest)+cngeneson+pc_mito+brain_region+Sex	4.014	907.000
ENSG00000197386	0.005	-0.048	0.078	-0.174	HTT	0.019	~p_tau+(1|manifest)+cngeneson+pc_mito+brain_region+Sex	2.732	1388.000
ENSG00000076641	0.313	-0.048	0.100	-0.197	PAG1	0.393	~p_tau+(1|manifest)+cngeneson+pc_mito+brain_region+Sex	2.108	1766.000
ENSG00000132470	0.630	-0.049	0.420	-0.517	ITGB4	0.682	~p_tau+(1|manifest)+cngeneson+pc_mito+brain_region+Sex	11.991	174.000
ENSG00000101019	0.007	-0.049	0.031	-0.128	UQCC1	0.024	~p_tau+(1|manifest)+cngeneson+pc_mito+brain_region+Sex	1.014	2636.000
ENSG00000109919	0.075	-0.049	0.151	-0.248	MTCH2	0.131	~p_tau+(1|manifest)+cngeneson+pc_mito+brain_region+Sex	1.470	2246.000
ENSG00000068024	0.224	-0.049	0.112	-0.210	HDAC4	0.301	~p_tau+(1|manifest)+cngeneson+pc_mito+brain_region+Sex	1.611	2135.000
ENSG00000169946	0.831	-0.049	0.118	-0.217	ZFPM2	0.854	~p_tau+(1|manifest)+cngeneson+pc_mito+brain_region+Sex	1.846	1953.000
ENSG00000151240	0.793	-0.049	0.099	-0.198	DIP2C	0.823	~p_tau+(1|manifest)+cngeneson+pc_mito+brain_region+Sex	7.011	443.000
ENSG00000105778	0.660	-0.049	0.112	-0.211	AVL9	0.708	~p_tau+(1|manifest)+cngeneson+pc_mito+brain_region+Sex	4.337	830.000
ENSG00000061676	0.538	-0.050	0.092	-0.192	NCKAP1	0.601	~p_tau+(1|manifest)+cngeneson+pc_mito+brain_region+Sex	3.475	1064.000
ENSG00000164066	0.365	-0.050	0.083	-0.182	INTU	0.443	~p_tau+(1|manifest)+cngeneson+pc_mito+brain_region+Sex	3.270	1146.000
ENSG00000109339	0.425	-0.050	0.024	-0.124	MAPK10	0.499	~p_tau+(1|manifest)+cngeneson+pc_mito+brain_region+Sex	2.301	1633.000
ENSG00000133812	0.292	-0.050	0.021	-0.121	SBF2	0.373	~p_tau+(1|manifest)+cngeneson+pc_mito+brain_region+Sex	4.413	811.000
ENSG00000106028	0.000	-0.050	0.068	-0.168	SSBP1	0.003	~p_tau+(1|manifest)+cngeneson+pc_mito+brain_region+Sex	0.407	3060.000
ENSG00000166405	0.233	-0.050	0.143	-0.243	RIC3	0.310	~p_tau+(1|manifest)+cngeneson+pc_mito+brain_region+Sex	3.679	1008.000
ENSG00000160216	0.026	-0.050	0.135	-0.235	AGPAT3	0.058	~p_tau+(1|manifest)+cngeneson+pc_mito+brain_region+Sex	2.097	1772.000
ENSG00000086015	0.002	-0.050	0.135	-0.235	MAST2	0.011	~p_tau+(1|manifest)+cngeneson+pc_mito+brain_region+Sex	3.112	1214.000
ENSG00000131626	0.828	-0.050	0.132	-0.232	PPFIA1	0.851	~p_tau+(1|manifest)+cngeneson+pc_mito+brain_region+Sex	10.766	223.000
ENSG00000033800	0.561	-0.050	0.094	-0.194	PIAS1	0.620	~p_tau+(1|manifest)+cngeneson+pc_mito+brain_region+Sex	1.765	2004.000
ENSG00000138600	0.613	-0.050	0.211	-0.311	SPPL2A	0.666	~p_tau+(1|manifest)+cngeneson+pc_mito+brain_region+Sex	7.355	406.000
ENSG00000111605	0.015	-0.050	0.108	-0.208	CPSF6	0.038	~p_tau+(1|manifest)+cngeneson+pc_mito+brain_region+Sex	2.533	1488.000
ENSG00000136021	0.003	-0.051	0.078	-0.179	SCYL2	0.014	~p_tau+(1|manifest)+cngeneson+pc_mito+brain_region+Sex	2.121	1755.000
ENSG00000166167	0.474	-0.051	0.170	-0.271	BTRC	0.542	~p_tau+(1|manifest)+cngeneson+pc_mito+brain_region+Sex	1.294	2410.000
ENSG00000099917	0.136	-0.051	0.174	-0.276	MED15	0.205	~p_tau+(1|manifest)+cngeneson+pc_mito+brain_region+Sex	3.809	965.000
ENSG00000137776	0.020	-0.051	0.122	-0.224	SLTM	0.049	~p_tau+(1|manifest)+cngeneson+pc_mito+brain_region+Sex	3.221	1167.000
ENSG00000165699	0.004	-0.051	0.081	-0.184	TSC1	0.017	~p_tau+(1|manifest)+cngeneson+pc_mito+brain_region+Sex	1.196	2488.000
ENSG00000196911	0.020	-0.051	0.047	-0.150	KPNA5	0.049	~p_tau+(1|manifest)+cngeneson+pc_mito+brain_region+Sex	0.880	2755.000
ENSG00000141425	0.003	-0.051	0.050	-0.152	RPRD1A	0.014	~p_tau+(1|manifest)+cngeneson+pc_mito+brain_region+Sex	1.031	2619.000
ENSG00000070018	0.352	-0.052	0.175	-0.278	LRP6	0.431	~p_tau+(1|manifest)+cngeneson+pc_mito+brain_region+Sex	3.016	1254.000
ENSG00000145715	0.103	-0.052	0.125	-0.230	RASA1	0.167	~p_tau+(1|manifest)+cngeneson+pc_mito+brain_region+Sex	1.996	1843.000
ENSG00000138138	0.002	-0.053	0.033	-0.138	ATAD1	0.011	~p_tau+(1|manifest)+cngeneson+pc_mito+brain_region+Sex	1.293	2411.000
ENSG00000152977	0.650	-0.053	0.431	-0.536	ZIC1	0.700	~p_tau+(1|manifest)+cngeneson+pc_mito+brain_region+Sex	14.488	108.000
ENSG00000137817	0.110	-0.053	0.264	-0.370	PARP6	0.175	~p_tau+(1|manifest)+cngeneson+pc_mito+brain_region+Sex	5.000	701.000
ENSG00000170425	0.104	-0.053	0.105	-0.212	ADORA2B	0.168	~p_tau+(1|manifest)+cngeneson+pc_mito+brain_region+Sex	2.158	1729.000
ENSG00000110888	0.063	-0.053	0.132	-0.239	CAPRIN2	0.114	~p_tau+(1|manifest)+cngeneson+pc_mito+brain_region+Sex	2.606	1453.000
ENSG00000051382	0.060	-0.054	0.119	-0.226	PIK3CB	0.111	~p_tau+(1|manifest)+cngeneson+pc_mito+brain_region+Sex	2.572	1470.000
ENSG00000169504	0.966	-0.054	0.596	-0.704	CLIC4	0.972	~p_tau+(1|manifest)+cngeneson+pc_mito+brain_region+Sex	17.237	75.000
ENSG00000271723	0.319	-0.054	0.470	-0.578	[illegible]	0.399	~p_tau+(1|manifest)+cngeneson+pc_mito+brain_region+Sex	8.877	311.000
ENSG00000116754	0.084	-0.054	0.147	-0.255	SRSF11	0.143	~p_tau+(1|manifest)+cngeneson+pc_mito+brain_region+Sex	4.526	791.000
ENSG00000114423	0.044	-0.054	0.069	-0.177	CBLB	0.087	~p_tau+(1|manifest)+cngeneson+pc_mito+brain_region+Sex	0.692	2918.000
ENSG00000007341	0.018	-0.054	0.102	-0.210	ST7L	0.045	~p_tau+(1|manifest)+cngeneson+pc_mito+brain_region+Sex	0.636	2943.000
ENSG00000062725	0.000	-0.054	0.056	-0.165	APPBP2	0.000	~p_tau+(1|manifest)+cngeneson+pc_mito+brain_region+Sex	1.192	2495.000
ENSG00000116704	0.010	-0.054	0.031	-0.140	SLC35D1	0.029	~p_tau+(1|manifest)+cngeneson+pc_mito+brain_region+Sex	0.865	2770.000
ENSG00000140386	0.157	-0.055	0.036	-0.146	SCAPER	0.229	~p_tau+(1|manifest)+cngeneson+pc_mito+brain_region+Sex	2.944	1293.000
ENSG00000135929	0.042	-0.055	0.107	-0.217	CYP27A1	0.084	~p_tau+(1|manifest)+cngeneson+pc_mito+brain_region+Sex	1.533	2189.000
ENSG00000119650	0.067	-0.055	0.113	-0.223	IFT43	0.120	~p_tau+(1|manifest)+cngeneson+pc_mito+brain_region+Sex	2.466	1538.000
ENSG00000135341	0.001	-0.055	0.087	-0.197	MAP3K7	0.007	~p_tau+(1|manifest)+cngeneson+pc_mito+brain_region+Sex	1.737	2034.000
ENSG00000102189	0.224	-0.055	0.144	-0.254	EEA1	0.301	~p_tau+(1|manifest)+cngeneson+pc_mito+brain_region+Sex	3.981	915.000
ENSG00000023516	0.039	-0.055	0.064	-0.174	AKAP11	0.079	~p_tau+(1|manifest)+cngeneson+pc_mito+brain_region+Sex	1.222	2463.000
ENSG00000163110	0.553	-0.055	0.055	-0.166	PDLIM5	0.613	~p_tau+(1|manifest)+cngeneson+pc_mito+brain_region+Sex	1.255	2439.000
ENSG00000118965	0.026	-0.056	0.122	-0.234	WDR35	0.058	~p_tau+(1|manifest)+cngeneson+pc_mito+brain_region+Sex	1.421	2285.000
ENSG00000198825	0.888	-0.056	0.250	-0.362	INPP5F	0.905	~p_tau+(1|manifest)+cngeneson+pc_mito+brain_region+Sex	5.775	576.000
ENSG00000141380	0.007	-0.056	0.064	-0.176	SS18	0.022	~p_tau+(1|manifest)+cngeneson+pc_mito+brain_region+Sex	2.043	1811.000
ENSG00000172939	0.003	-0.056	0.032	-0.145	OXSR1	0.011	~p_tau+(1|manifest)+cngeneson+pc_mito+brain_region+Sex	0.738	2878.000
ENSG00000066777	0.005	-0.057	0.065	-0.178	ARFGEF1	0.019	~p_tau+(1|manifest)+cngeneson+pc_mito+brain_region+Sex	1.947	1883.000
ENSG00000135298	0.811	-0.057	0.120	-0.233	ADGRB3	0.838	~p_tau+(1|manifest)+cngeneson+pc_mito+brain_region+Sex	24.777	31.000
ENSG00000006125	0.035	-0.057	0.073	-0.187	AP2B1	0.072	~p_tau+(1|manifest)+cngeneson+pc_mito+brain_region+Sex	1.508	2210.000
ENSG00000118873	0.002	-0.057	0.071	-0.185	RAB3GAP2	0.009	~p_tau+(1|manifest)+cngeneson+pc_mito+brain_region+Sex	2.331	1613.000
ENSG00000177707	0.079	-0.057	0.105	-0.220	NECTIN3	0.136	~p_tau+(1|manifest)+cngeneson+pc_mito+brain_region+Sex	0.511	3010.000
ENSG00000145375	0.338	-0.057	0.038	-0.153	SPATA5	0.416	~p_tau+(1|manifest)+cngeneson+pc_mito+brain_region+Sex	1.297	2409.000
ENSG00000175265	0.404	-0.057	0.384	-0.499	GOLGA8A	0.480	~p_tau+(1|manifest)+cngeneson+pc_mito+brain_region+Sex	8.389	332.000
ENSG00000164182	0.307	-0.058	0.082	-0.197	NDUFAF2	0.388	~p_tau+(1|manifest)+cngeneson+pc_mito+brain_region+Sex	2.149	1731.000
ENSG00000101557	0.008	-0.058	0.047	-0.162	USP14	0.025	~p_tau+(1|manifest)+cngeneson+pc_mito+brain_region+Sex	1.736	2035.000
ENSG00000173611	0.076	-0.058	0.049	-0.164	SCAI	0.133	~p_tau+(1|manifest)+cngeneson+pc_mito+brain_region+Sex	1.025	2625.000
ENSG00000185104	0.335	-0.058	0.020	-0.136	FAF1	0.414	~p_tau+(1|manifest)+cngeneson+pc_mito+brain_region+Sex	2.606	1454.000
ENSG00000173482	0.739	-0.058	0.091	-0.207	PTPRM	0.777	~p_tau+(1|manifest)+cngeneson+pc_mito+brain_region+Sex	1.395	2314.000
ENSG00000180776	0.004	-0.058	0.068	-0.184	ZDHHC20	0.015	~p_tau+(1|manifest)+cngeneson+pc_mito+brain_region+Sex	1.207	2476.000
ENSG00000112062	0.007	-0.058	0.063	-0.179	MAPK14	0.023	~p_tau+(1|manifest)+cngeneson+pc_mito+brain_region+Sex	2.016	1827.000
ENSG00000168904	0.170	-0.059	0.052	-0.170	LRRC28	0.243	~p_tau+(1|manifest)+cngeneson+pc_mito+brain_region+Sex	1.437	2273.000
ENSG00000197535	0.007	-0.059	0.102	-0.220	MYO5A	0.023	~p_tau+(1|manifest)+cngeneson+pc_mito+brain_region+Sex	0.742	2872.000
ENSG00000137075	0.118	-0.059	0.093	-0.211	RNF38	0.183	~p_tau+(1|manifest)+cngeneson+pc_mito+brain_region+Sex	1.714	2054.000
ENSG00000213047	0.019	-0.059	0.068	-0.186	DENND1B	0.047	~p_tau+(1|manifest)+cngeneson+pc_mito+brain_region+Sex	0.632	2947.000
ENSG00000069275	0.038	-0.059	0.087	-0.205	NUCKS1	0.078	~p_tau+(1|manifest)+cngeneson+pc_mito+brain_region+Sex	2.693	1407.000
ENSG00000112699	0.808	-0.060	0.150	-0.269	GMDS	0.835	~p_tau+(1|manifest)+cngeneson+pc_mito+brain_region+Sex	2.520	1496.000
ENSG00000100796	0.002	-0.060	0.032	-0.152	PPP4R3A	0.008	~p_tau+(1|manifest)+cngeneson+pc_mito+brain_region+Sex	1.159	2525.000
ENSG00000174106	0.019	-0.060	0.035	-0.155	LEMD3	0.047	~p_tau+(1|manifest)+cngeneson+pc_mito+brain_region+Sex	0.708	2908.000
ENSG00000115970	0.190	-0.060	0.053	-0.173	THADA	0.265	~p_tau+(1|manifest)+cngeneson+pc_mito+brain_region+Sex	1.412	2295.000
ENSG00000204406	0.037	-0.060	-0.004	-0.117	MBD5	0.077	~p_tau+(1|manifest)+cngeneson+pc_mito+brain_region+Sex	2.595	1458.000
ENSG00000088930	0.039	-0.060	0.068	-0.189	XRN2	0.080	~p_tau+(1|manifest)+cngeneson+pc_mito+brain_region+Sex	2.535	1486.000
ENSG00000138698	0.533	-0.060	0.158	-0.279	RAP1GDS1	0.596	~p_tau+(1|manifest)+cngeneson+pc_mito+brain_region+Sex	1.080	2584.000
ENSG00000116698	0.150	-0.061	0.134	-0.255	SMG7	0.220	~p_tau+(1|manifest)+cngeneson+pc_mito+brain_region+Sex	2.132	1747.000
ENSG00000136111	0.529	-0.061	0.113	-0.234	TBC1D4	0.593	~p_tau+(1|manifest)+cngeneson+pc_mito+brain_region+Sex	8.317	340.000
ENSG00000109572	0.032	-0.061	0.055	-0.177	CLCN3	0.067	~p_tau+(1|manifest)+cngeneson+pc_mito+brain_region+Sex	0.587	2965.000
ENSG00000157107	0.025	-0.061	0.162	-0.284	FCHO2	0.057	~p_tau+(1|manifest)+cngeneson+pc_mito+brain_region+Sex	0.549	2988.000
ENSG00000140262	0.161	-0.061	0.015	-0.137	TCF12	0.233	~p_tau+(1|manifest)+cngeneson+pc_mito+brain_region+Sex	2.636	1436.000
ENSG00000107862	0.003	-0.061	0.046	-0.168	GBF1	0.013	~p_tau+(1|manifest)+cngeneson+pc_mito+brain_region+Sex	1.852	1949.000
ENSG00000163946	0.004	-0.061	0.036	-0.158	TASOR	0.016	~p_tau+(1|manifest)+cngeneson+pc_mito+brain_region+Sex	0.740	2875.000
ENSG00000178568	0.390	-0.062	0.047	-0.170	ERBB4	0.467	~p_tau+(1|manifest)+cngeneson+pc_mito+brain_region+Sex	9.565	280.000
ENSG00000163527	0.021	-0.062	0.134	-0.258	STT3B	0.051	~p_tau+(1|manifest)+cngeneson+pc_mito+brain_region+Sex	2.473	1533.000
ENSG00000169057	0.026	-0.062	0.073	-0.197	MECP2	0.059	~p_tau+(1|manifest)+cngeneson+pc_mito+brain_region+Sex	1.027	2624.000
ENSG00000120725	0.125	-0.062	0.050	-0.175	SIL1	0.192	~p_tau+(1|manifest)+cngeneson+pc_mito+brain_region+Sex	1.077	2586.000
ENSG00000109606	0.003	-0.063	0.036	-0.161	DHX15	0.013	~p_tau+(1|manifest)+cngeneson+pc_mito+brain_region+Sex	1.049	2609.000
ENSG00000076321	0.013	-0.063	0.049	-0.174	KLHL20	0.036	~p_tau+(1|manifest)+cngeneson+pc_mito+brain_region+Sex	1.352	2354.000
ENSG00000183576	0.025	-0.063	0.045	-0.170	SETD3	0.056	~p_tau+(1|manifest)+cngeneson+pc_mito+brain_region+Sex	1.133	2549.000
ENSG00000156650	0.346	-0.063	0.097	-0.222	KAT6B	0.425	~p_tau+(1|manifest)+cngeneson+pc_mito+brain_region+Sex	2.071	1788.000
ENSG00000082258	0.005	-0.063	0.073	-0.198	CCNT2	0.018	~p_tau+(1|manifest)+cngeneson+pc_mito+brain_region+Sex	1.055	2605.000
ENSG00000164309	0.399	-0.063	0.244	-0.370	CMYA5	0.475	~p_tau+(1|manifest)+cngeneson+pc_mito+brain_region+Sex	3.468	1067.000
ENSG00000185565	0.215	-0.063	0.007	-0.133	LSAMP	0.292	~p_tau+(1|manifest)+cngeneson+pc_mito+brain_region+Sex	12.706	152.000
ENSG00000021574	0.007	-0.063	0.019	-0.145	SPAST	0.022	~p_tau+(1|manifest)+cngeneson+pc_mito+brain_region+Sex	0.778	2851.000
ENSG00000183495	0.009	-0.063	0.069	-0.195	EP400	0.027	~p_tau+(1|manifest)+cngeneson+pc_mito+brain_region+Sex	1.969	1869.000
ENSG00000206560	0.376	-0.063	0.287	-0.414	ANKRD28	0.453	~p_tau+(1|manifest)+cngeneson+pc_mito+brain_region+Sex	5.623	599.000
ENSG00000122008	0.007	-0.063	0.062	-0.188	POLK	0.022	~p_tau+(1|manifest)+cngeneson+pc_mito+brain_region+Sex	0.898	2737.000
ENSG00000198742	0.188	-0.063	0.051	-0.178	SMURF1	0.263	~p_tau+(1|manifest)+cngeneson+pc_mito+brain_region+Sex	1.344	2361.000
ENSG00000135272	0.591	-0.063	0.179	-0.306	MDFIC	0.647	~p_tau+(1|manifest)+cngeneson+pc_mito+brain_region+Sex	0.755	2865.000
ENSG00000137872	0.357	-0.064	0.079	-0.206	SEMA6D	0.435	~p_tau+(1|manifest)+cngeneson+pc_mito+brain_region+Sex	1.494	2226.000
ENSG00000135049	0.057	-0.064	0.079	-0.207	AGTPBP1	0.106	~p_tau+(1|manifest)+cngeneson+pc_mito+brain_region+Sex	1.527	2195.000
ENSG00000114670	0.088	-0.064	0.098	-0.226	NEK11	0.147	~p_tau+(1|manifest)+cngeneson+pc_mito+brain_region+Sex	0.811	2822.000
ENSG00000129158	0.936	-0.064	0.338	-0.466	SERGEF	0.947	~p_tau+(1|manifest)+cngeneson+pc_mito+brain_region+Sex	7.508	393.000
ENSG00000168310	0.096	-0.064	0.075	-0.203	IRF2	0.158	~p_tau+(1|manifest)+cngeneson+pc_mito+brain_region+Sex	1.557	2166.000
ENSG00000100320	0.559	-0.064	0.071	-0.199	RBFOX2	0.620	~p_tau+(1|manifest)+cngeneson+pc_mito+brain_region+Sex	5.462	631.000
ENSG00000080802	0.178	-0.064	0.067	-0.195	CNOT4	0.252	~p_tau+(1|manifest)+cngeneson+pc_mito+brain_region+Sex	2.072	1786.000
ENSG00000008869	0.004	-0.064	0.006	-0.135	HEATR5B	0.017	~p_tau+(1|manifest)+cngeneson+pc_mito+brain_region+Sex	1.146	2536.000
ENSG00000145555	0.770	-0.065	0.283	-0.412	MYO10	0.804	~p_tau+(1|manifest)+cngeneson+pc_mito+brain_region+Sex	2.712	1401.000
ENSG00000162604	0.046	-0.065	0.063	-0.192	TM2D1	0.090	~p_tau+(1|manifest)+cngeneson+pc_mito+brain_region+Sex	1.228	2461.000
ENSG00000082701	0.467	-0.065	0.107	-0.236	GSK3B	0.537	~p_tau+(1|manifest)+cngeneson+pc_mito+brain_region+Sex	4.456	802.000
ENSG00000105176	0.101	-0.065	0.082	-0.212	URI1	0.164	~p_tau+(1|manifest)+cngeneson+pc_mito+brain_region+Sex	2.395	1579.000
ENSG00000123096	0.175	-0.065	0.287	-0.417	SSPN	0.249	~p_tau+(1|manifest)+cngeneson+pc_mito+brain_region+Sex	4.046	898.000
ENSG00000152092	0.801	-0.065	0.127	-0.257	ASTN1	0.829	~p_tau+(1|manifest)+cngeneson+pc_mito+brain_region+Sex	2.509	1511.000
ENSG00000184014	0.167	-0.065	0.084	-0.213	DENND5A	0.239	~p_tau+(1|manifest)+cngeneson+pc_mito+brain_region+Sex	2.373	1589.000
ENSG00000070269	0.012	-0.065	0.036	-0.166	TMEM260	0.034	~p_tau+(1|manifest)+cngeneson+pc_mito+brain_region+Sex	1.067	2598.000
ENSG00000134744	0.089	-0.065	0.049	-0.179	TUT4	0.148	~p_tau+(1|manifest)+cngeneson+pc_mito+brain_region+Sex	1.949	1879.000
ENSG00000204138	0.019	-0.065	0.052	-0.182	PHACTR4	0.046	~p_tau+(1|manifest)+cngeneson+pc_mito+brain_region+Sex	1.883	1929.000
ENSG00000179455	0.731	-0.065	0.158	-0.288	MKRN3	0.771	~p_tau+(1|manifest)+cngeneson+pc_mito+brain_region+Sex	1.906	1907.000
ENSG00000115760	0.172	-0.065	0.012	-0.142	BIRC6	0.246	~p_tau+(1|manifest)+cngeneson+pc_mito+brain_region+Sex	5.818	564.000
ENSG00000272325	0.486	-0.065	0.322	-0.452	NUDT3	0.554	~p_tau+(1|manifest)+cngeneson+pc_mito+brain_region+Sex	3.685	1006.000
ENSG00000117335	0.006	-0.065	0.047	-0.178	CD46	0.021	~p_tau+(1|manifest)+cngeneson+pc_mito+brain_region+Sex	1.971	1865.000
ENSG00000089902	0.012	-0.066	0.034	-0.165	RCOR1	0.034	~p_tau+(1|manifest)+cngeneson+pc_mito+brain_region+Sex	1.289	2415.000
ENSG00000138592	0.083	-0.066	0.071	-0.202	USP8	0.142	~p_tau+(1|manifest)+cngeneson+pc_mito+brain_region+Sex	1.149	2533.000
ENSG00000013619	0.519	-0.066	0.152	-0.283	MAMLD1	0.585	~p_tau+(1|manifest)+cngeneson+pc_mito+brain_region+Sex	2.040	1812.000
ENSG00000103194	0.003	-0.066	0.023	-0.155	USP10	0.013	~p_tau+(1|manifest)+cngeneson+pc_mito+brain_region+Sex	1.010	2640.000
ENSG00000065243	0.002	-0.066	0.050	-0.182	PKN2	0.009	~p_tau+(1|manifest)+cngeneson+pc_mito+brain_region+Sex	1.709	2059.000
ENSG00000171723	0.062	-0.066	0.002	-0.135	GPHN	0.113	~p_tau+(1|manifest)+cngeneson+pc_mito+brain_region+Sex	3.141	1199.000
ENSG00000058063	0.000	-0.067	0.018	-0.152	ATP11B	0.000	~p_tau+(1|manifest)+cngeneson+pc_mito+brain_region+Sex	1.176	2509.000
ENSG00000154553	0.013	-0.067	0.143	-0.276	PDLIM3	0.035	~p_tau+(1|manifest)+cngeneson+pc_mito+brain_region+Sex	2.480	1526.000
ENSG00000112701	0.431	-0.067	0.047	-0.181	SENP6	0.504	~p_tau+(1|manifest)+cngeneson+pc_mito+brain_region+Sex	2.649	1432.000
ENSG00000132718	0.430	-0.067	0.178	-0.312	SYT11	0.503	~p_tau+(1|manifest)+cngeneson+pc_mito+brain_region+Sex	2.280	1653.000
ENSG00000182985	0.582	-0.067	0.063	-0.198	CADM1	0.639	~p_tau+(1|manifest)+cngeneson+pc_mito+brain_region+Sex	13.510	133.000
ENSG00000048707	0.573	-0.068	0.112	-0.247	VPS13D	0.631	~p_tau+(1|manifest)+cngeneson+pc_mito+brain_region+Sex	4.147	874.000
ENSG00000100393	0.101	-0.068	0.063	-0.199	EP300	0.163	~p_tau+(1|manifest)+cngeneson+pc_mito+brain_region+Sex	2.167	1720.000
ENSG00000054611	0.013	-0.068	0.015	-0.151	TBC1D22A	0.036	~p_tau+(1|manifest)+cngeneson+pc_mito+brain_region+Sex	0.843	2788.000
ENSG00000133835	0.006	-0.068	0.030	-0.166	HSD17B4	0.020	~p_tau+(1|manifest)+cngeneson+pc_mito+brain_region+Sex	1.384	2327.000
ENSG00000077147	0.008	-0.068	0.029	-0.165	TM9SF3	0.026	~p_tau+(1|manifest)+cngeneson+pc_mito+brain_region+Sex	1.673	2085.000
ENSG00000122729	0.086	-0.068	0.088	-0.224	ACO1	0.144	~p_tau+(1|manifest)+cngeneson+pc_mito+brain_region+Sex	2.654	1430.000
ENSG00000143641	0.838	-0.068	0.171	-0.307	GALNT2	0.860	~p_tau+(1|manifest)+cngeneson+pc_mito+brain_region+Sex	4.699	748.000
ENSG00000116128	0.008	-0.068	0.052	-0.189	BCL9	0.025	~p_tau+(1|manifest)+cngeneson+pc_mito+brain_region+Sex	1.081	2581.000
ENSG00000064933	0.019	-0.069	0.051	-0.189	PMS1	0.045	~p_tau+(1|manifest)+cngeneson+pc_mito+brain_region+Sex	2.181	1714.000
ENSG00000113448	0.824	-0.069	0.176	-0.314	PDE4D	0.848	~p_tau+(1|manifest)+cngeneson+pc_mito+brain_region+Sex	9.877	266.000
ENSG00000178105	0.021	-0.070	0.014	-0.154	DDX10	0.051	~p_tau+(1|manifest)+cngeneson+pc_mito+brain_region+Sex	1.599	2145.000
ENSG00000273294	0.065	-0.070	0.098	-0.237	[illegible]	0.117	~p_tau+(1|manifest)+cngeneson+pc_mito+brain_region+Sex	0.658	2933.000
ENSG00000153956	0.667	-0.070	0.144	-0.284	CACNA2D1	0.714	~p_tau+(1|manifest)+cngeneson+pc_mito+brain_region+Sex	4.693	750.000
ENSG00000057663	0.074	-0.070	0.023	-0.163	ATG5	0.130	~p_tau+(1|manifest)+cngeneson+pc_mito+brain_region+Sex	0.884	2751.000
ENSG00000048649	0.048	-0.070	0.042	-0.182	RSF1	0.093	~p_tau+(1|manifest)+cngeneson+pc_mito+brain_region+Sex	2.399	1574.000
ENSG00000144724	0.758	-0.070	0.127	-0.268	PTPRG	0.793	~p_tau+(1|manifest)+cngeneson+pc_mito+brain_region+Sex	6.643	471.000
ENSG00000198718	0.003	-0.070	-0.002	-0.139	TOGARAM1	0.012	~p_tau+(1|manifest)+cngeneson+pc_mito+brain_region+Sex	1.407	2300.000
ENSG00000110090	0.116	-0.071	0.092	-0.233	CPT1A	0.181	~p_tau+(1|manifest)+cngeneson+pc_mito+brain_region+Sex	2.051	1800.000
ENSG00000100991	0.041	-0.071	0.085	-0.227	TRPC4AP	0.082	~p_tau+(1|manifest)+cngeneson+pc_mito+brain_region+Sex	2.201	1701.000
ENSG00000171853	0.019	-0.071	0.083	-0.225	TRAPPC12	0.047	~p_tau+(1|manifest)+cngeneson+pc_mito+brain_region+Sex	1.745	2025.000
ENSG00000198625	0.015	-0.071	0.072	-0.213	MDM4	0.040	~p_tau+(1|manifest)+cngeneson+pc_mito+brain_region+Sex	1.855	1947.000
ENSG00000164300	0.394	-0.071	0.093	-0.235	SERINC5	0.470	~p_tau+(1|manifest)+cngeneson+pc_mito+brain_region+Sex	0.917	2715.000
ENSG00000107929	0.034	-0.072	0.064	-0.207	LARP4B	0.071	~p_tau+(1|manifest)+cngeneson+pc_mito+brain_region+Sex	1.988	1849.000
ENSG00000138709	0.479	-0.072	0.187	-0.331	LARP1B	0.548	~p_tau+(1|manifest)+cngeneson+pc_mito+brain_region+Sex	4.705	747.000
ENSG00000151376	0.327	-0.072	0.124	-0.268	ME3	0.405	~p_tau+(1|manifest)+cngeneson+pc_mito+brain_region+Sex	5.100	686.000
ENSG00000151413	0.371	-0.072	0.130	-0.274	NUBPL	0.449	~p_tau+(1|manifest)+cngeneson+pc_mito+brain_region+Sex	0.586	2966.000
ENSG00000160551	0.606	-0.072	0.112	-0.257	TAOK1	0.660	~p_tau+(1|manifest)+cngeneson+pc_mito+brain_region+Sex	5.980	548.000
ENSG00000072274	0.327	-0.072	0.215	-0.360	TFRC	0.405	~p_tau+(1|manifest)+cngeneson+pc_mito+brain_region+Sex	4.696	749.000
ENSG00000111670	0.041	-0.073	0.097	-0.242	GNPTAB	0.082	~p_tau+(1|manifest)+cngeneson+pc_mito+brain_region+Sex	2.365	1595.000
ENSG00000141027	0.282	-0.073	0.043	-0.189	NCOR1	0.363	~p_tau+(1|manifest)+cngeneson+pc_mito+brain_region+Sex	5.026	695.000
ENSG00000018408	0.925	-0.073	0.300	-0.446	WWTR1	0.937	~p_tau+(1|manifest)+cngeneson+pc_mito+brain_region+Sex	12.940	143.000
ENSG00000143458	0.008	-0.073	0.106	-0.252	GABPB2	0.025	~p_tau+(1|manifest)+cngeneson+pc_mito+brain_region+Sex	0.437	3047.000
ENSG00000148429	0.126	-0.073	0.092	-0.239	USP6NL	0.194	~p_tau+(1|manifest)+cngeneson+pc_mito+brain_region+Sex	1.482	2237.000
ENSG00000198911	0.341	-0.073	0.234	-0.380	SREBF2	0.420	~p_tau+(1|manifest)+cngeneson+pc_mito+brain_region+Sex	6.925	454.000
ENSG00000165219	0.001	-0.073	0.011	-0.158	GAPVD1	0.004	~p_tau+(1|manifest)+cngeneson+pc_mito+brain_region+Sex	1.095	2573.000
ENSG00000168300	0.083	-0.074	0.085	-0.232	PCMTD1	0.141	~p_tau+(1|manifest)+cngeneson+pc_mito+brain_region+Sex	1.347	2359.000
ENSG00000082269	0.774	-0.074	0.146	-0.293	FAM135A	0.807	~p_tau+(1|manifest)+cngeneson+pc_mito+brain_region+Sex	5.779	574.000
ENSG00000119471	0.153	-0.074	0.153	-0.301	HSDL2	0.224	~p_tau+(1|manifest)+cngeneson+pc_mito+brain_region+Sex	3.789	970.000
ENSG00000112305	0.056	-0.074	0.105	-0.253	SMAP1	0.105	~p_tau+(1|manifest)+cngeneson+pc_mito+brain_region+Sex	2.320	1619.000
ENSG00000164975	0.000	-0.074	-0.013	-0.135	SNAPC3	0.001	~p_tau+(1|manifest)+cngeneson+pc_mito+brain_region+Sex	0.677	2924.000
ENSG00000104299	0.046	-0.074	0.106	-0.254	INTS9	0.090	~p_tau+(1|manifest)+cngeneson+pc_mito+brain_region+Sex	2.534	1487.000
ENSG00000214517	0.009	-0.074	0.052	-0.201	PPME1	0.027	~p_tau+(1|manifest)+cngeneson+pc_mito+brain_region+Sex	1.884	1927.000
ENSG00000048991	0.117	-0.074	0.026	-0.175	R3HDM1	0.183	~p_tau+(1|manifest)+cngeneson+pc_mito+brain_region+Sex	2.737	1387.000
ENSG00000133048	0.899	-0.074	1.215	-1.364	CHI3L1	0.914	~p_tau+(1|manifest)+cngeneson+pc_mito+brain_region+Sex	48.737	7.000
ENSG00000166503	0.005	-0.075	0.009	-0.158	HDGFL3	0.018	~p_tau+(1|manifest)+cngeneson+pc_mito+brain_region+Sex	0.951	2693.000
ENSG00000198839	0.281	-0.075	0.084	-0.233	ZNF277	0.362	~p_tau+(1|manifest)+cngeneson+pc_mito+brain_region+Sex	1.484	2234.000
ENSG00000111860	0.631	-0.075	0.093	-0.242	CEP85L	0.683	~p_tau+(1|manifest)+cngeneson+pc_mito+brain_region+Sex	3.757	989.000
ENSG00000074755	0.085	-0.075	0.074	-0.224	ZZEF1	0.144	~p_tau+(1|manifest)+cngeneson+pc_mito+brain_region+Sex	2.645	1433.000
ENSG00000115365	0.029	-0.075	0.013	-0.163	LANCL1	0.063	~p_tau+(1|manifest)+cngeneson+pc_mito+brain_region+Sex	1.303	2397.000
ENSG00000113048	0.001	-0.075	0.006	-0.156	MRPS27	0.006	~p_tau+(1|manifest)+cngeneson+pc_mito+brain_region+Sex	0.983	2667.000
ENSG00000135317	0.002	-0.075	0.016	-0.166	SNX14	0.009	~p_tau+(1|manifest)+cngeneson+pc_mito+brain_region+Sex	0.991	2658.000
ENSG00000035681	0.003	-0.075	0.113	-0.264	NSMAF	0.014	~p_tau+(1|manifest)+cngeneson+pc_mito+brain_region+Sex	3.342	1119.000
ENSG00000008394	0.663	-0.076	0.122	-0.273	MGST1	0.711	~p_tau+(1|manifest)+cngeneson+pc_mito+brain_region+Sex	9.814	270.000
ENSG00000131269	0.271	-0.076	0.071	-0.222	ABCB7	0.353	~p_tau+(1|manifest)+cngeneson+pc_mito+brain_region+Sex	1.098	2569.000
ENSG00000196199	0.077	-0.076	0.207	-0.358	MPHOSPH8	0.134	~p_tau+(1|manifest)+cngeneson+pc_mito+brain_region+Sex	4.217	854.000
ENSG00000104324	0.598	-0.076	0.092	-0.244	CPQ	0.654	~p_tau+(1|manifest)+cngeneson+pc_mito+brain_region+Sex	1.861	1945.000
ENSG00000051825	0.054	-0.076	0.130	-0.282	MPHOSPH9	0.102	~p_tau+(1|manifest)+cngeneson+pc_mito+brain_region+Sex	2.308	1625.000
ENSG00000047579	0.199	-0.076	0.085	-0.238	DTNBP1	0.274	~p_tau+(1|manifest)+cngeneson+pc_mito+brain_region+Sex	3.417	1092.000
ENSG00000184005	0.866	-0.076	0.397	-0.549	[illegible]	0.885	~p_tau+(1|manifest)+cngeneson+pc_mito+brain_region+Sex	6.483	492.000
ENSG00000144741	0.217	-0.077	0.078	-0.231	SLC25A26	0.293	~p_tau+(1|manifest)+cngeneson+pc_mito+brain_region+Sex	1.870	1937.000
ENSG00000164054	0.204	-0.077	0.287	-0.441	SHISA5	0.279	~p_tau+(1|manifest)+cngeneson+pc_mito+brain_region+Sex	5.213	666.000
ENSG00000221914	0.023	-0.077	0.075	-0.229	PPP2R2A	0.053	~p_tau+(1|manifest)+cngeneson+pc_mito+brain_region+Sex	1.229	2459.000
ENSG00000164253	0.021	-0.077	0.043	-0.198	WDR41	0.050	~p_tau+(1|manifest)+cngeneson+pc_mito+brain_region+Sex	1.566	2160.000
ENSG00000021776	0.036	-0.077	0.031	-0.185	AQR	0.075	~p_tau+(1|manifest)+cngeneson+pc_mito+brain_region+Sex	0.725	2892.000
ENSG00000204116	0.098	-0.078	0.092	-0.247	CHIC1	0.159	~p_tau+(1|manifest)+cngeneson+pc_mito+brain_region+Sex	1.535	2188.000
ENSG00000142687	0.149	-0.078	0.133	-0.289	KIAA0319L	0.220	~p_tau+(1|manifest)+cngeneson+pc_mito+brain_region+Sex	4.336	831.000
ENSG00000004866	0.097	-0.078	0.034	-0.190	ST7	0.159	~p_tau+(1|manifest)+cngeneson+pc_mito+brain_region+Sex	1.230	2458.000
ENSG00000117859	0.324	-0.078	0.112	-0.269	OSBPL9	0.403	~p_tau+(1|manifest)+cngeneson+pc_mito+brain_region+Sex	3.853	948.000
ENSG00000184408	0.357	-0.079	0.516	-0.673	KCND2	0.435	~p_tau+(1|manifest)+cngeneson+pc_mito+brain_region+Sex	12.448	164.000
ENSG00000068305	0.307	-0.079	0.058	-0.215	MEF2A	0.388	~p_tau+(1|manifest)+cngeneson+pc_mito+brain_region+Sex	2.780	1370.000
ENSG00000147274	0.299	-0.079	0.089	-0.247	RBMX	0.380	~p_tau+(1|manifest)+cngeneson+pc_mito+brain_region+Sex	3.759	988.000
ENSG00000147050	0.498	-0.079	0.066	-0.224	KDM6A	0.566	~p_tau+(1|manifest)+cngeneson+pc_mito+brain_region+Sex	2.331	1612.000
ENSG00000164209	0.021	-0.079	0.014	-0.173	SLC25A46	0.049	~p_tau+(1|manifest)+cngeneson+pc_mito+brain_region+Sex	0.989	2661.000
ENSG00000113916	0.806	-0.079	0.219	-0.378	BCL6	0.833	~p_tau+(1|manifest)+cngeneson+pc_mito+brain_region+Sex	23.741	33.000
ENSG00000146776	0.284	-0.080	0.057	-0.216	ATXN7L1	0.365	~p_tau+(1|manifest)+cngeneson+pc_mito+brain_region+Sex	0.887	2748.000
ENSG00000118690	0.096	-0.080	0.155	-0.314	ARMC2	0.157	~p_tau+(1|manifest)+cngeneson+pc_mito+brain_region+Sex	3.004	1263.000
ENSG00000071189	0.038	-0.080	0.129	-0.289	SNX13	0.078	~p_tau+(1|manifest)+cngeneson+pc_mito+brain_region+Sex	2.019	1826.000
ENSG00000112159	0.018	-0.080	0.069	-0.230	MDN1	0.045	~p_tau+(1|manifest)+cngeneson+pc_mito+brain_region+Sex	1.608	2139.000
ENSG00000100994	0.196	-0.081	0.250	-0.411	PYGB	0.271	~p_tau+(1|manifest)+cngeneson+pc_mito+brain_region+Sex	5.455	632.000
ENSG00000075568	0.532	-0.081	0.103	-0.264	TMEM131	0.595	~p_tau+(1|manifest)+cngeneson+pc_mito+brain_region+Sex	4.239	847.000
ENSG00000092148	0.016	-0.081	0.032	-0.193	HECTD1	0.041	~p_tau+(1|manifest)+cngeneson+pc_mito+brain_region+Sex	2.838	1342.000
ENSG00000133800	0.366	-0.081	0.202	-0.364	LYVE1	0.444	~p_tau+(1|manifest)+cngeneson+pc_mito+brain_region+Sex	3.771	981.000
ENSG00000142875	0.141	-0.081	0.005	-0.168	PRKACB	0.210	~p_tau+(1|manifest)+cngeneson+pc_mito+brain_region+Sex	1.060	2601.000
ENSG00000114480	0.435	-0.081	0.187	-0.350	GBE1	0.508	~p_tau+(1|manifest)+cngeneson+pc_mito+brain_region+Sex	5.248	661.000
ENSG00000143322	0.418	-0.082	0.097	-0.260	ABL2	0.493	~p_tau+(1|manifest)+cngeneson+pc_mito+brain_region+Sex	2.437	1553.000
ENSG00000112624	0.245	-0.082	0.098	-0.261	BICRAL	0.323	~p_tau+(1|manifest)+cngeneson+pc_mito+brain_region+Sex	3.000	1265.000
ENSG00000128915	0.007	-0.082	0.032	-0.196	ICE2	0.022	~p_tau+(1|manifest)+cngeneson+pc_mito+brain_region+Sex	0.506	3013.000
ENSG00000107077	0.769	-0.082	0.161	-0.324	KDM4C	0.803	~p_tau+(1|manifest)+cngeneson+pc_mito+brain_region+Sex	4.166	868.000
ENSG00000116747	0.015	-0.082	0.025	-0.189	RO60	0.038	~p_tau+(1|manifest)+cngeneson+pc_mito+brain_region+Sex	0.939	2697.000
ENSG00000120925	0.029	-0.082	0.087	-0.250	RNF170	0.063	~p_tau+(1|manifest)+cngeneson+pc_mito+brain_region+Sex	2.262	1662.000
ENSG00000135945	0.003	-0.082	0.026	-0.190	REV1	0.012	~p_tau+(1|manifest)+cngeneson+pc_mito+brain_region+Sex	1.298	2408.000
ENSG00000143013	0.357	-0.082	0.151	-0.315	LMO4	0.435	~p_tau+(1|manifest)+cngeneson+pc_mito+brain_region+Sex	2.241	1674.000
ENSG00000078674	0.064	-0.082	0.017	-0.182	PCM1	0.116	~p_tau+(1|manifest)+cngeneson+pc_mito+brain_region+Sex	2.048	1805.000
ENSG00000110274	0.105	-0.082	0.080	-0.245	CEP164	0.168	~p_tau+(1|manifest)+cngeneson+pc_mito+brain_region+Sex	3.125	1205.000
ENSG00000154001	0.016	-0.083	0.024	-0.190	PPP2R5E	0.040	~p_tau+(1|manifest)+cngeneson+pc_mito+brain_region+Sex	1.228	2462.000
ENSG00000182903	0.004	-0.084	0.009	-0.176	ZNF721	0.016	~p_tau+(1|manifest)+cngeneson+pc_mito+brain_region+Sex	0.649	2940.000
ENSG00000118200	0.464	-0.084	0.057	-0.224	CAMSAP2	0.535	~p_tau+(1|manifest)+cngeneson+pc_mito+brain_region+Sex	5.304	649.000
ENSG00000106603	0.055	-0.084	0.010	-0.178	COA1	0.104	~p_tau+(1|manifest)+cngeneson+pc_mito+brain_region+Sex	1.536	2186.000
ENSG00000023318	0.027	-0.084	0.021	-0.189	ERP44	0.060	~p_tau+(1|manifest)+cngeneson+pc_mito+brain_region+Sex	1.611	2137.000
ENSG00000130340	0.010	-0.084	0.081	-0.249	SNX9	0.030	~p_tau+(1|manifest)+cngeneson+pc_mito+brain_region+Sex	1.983	1855.000
ENSG00000121749	0.014	-0.084	0.048	-0.216	TBC1D15	0.037	~p_tau+(1|manifest)+cngeneson+pc_mito+brain_region+Sex	1.219	2466.000
ENSG00000166263	0.034	-0.084	-0.005	-0.163	STXBP4	0.072	~p_tau+(1|manifest)+cngeneson+pc_mito+brain_region+Sex	0.377	3075.000
ENSG00000186716	0.363	-0.084	0.175	-0.344	BCR	0.441	~p_tau+(1|manifest)+cngeneson+pc_mito+brain_region+Sex	4.149	872.000
ENSG00000165714	0.003	-0.084	0.014	-0.183	BORCS5	0.013	~p_tau+(1|manifest)+cngeneson+pc_mito+brain_region+Sex	0.527	3000.000
ENSG00000135919	0.315	-0.085	0.026	-0.196	SERPINE2	0.395	~p_tau+(1|manifest)+cngeneson+pc_mito+brain_region+Sex	0.804	2830.000
ENSG00000109016	0.167	-0.085	0.076	-0.246	DHRS7B	0.239	~p_tau+(1|manifest)+cngeneson+pc_mito+brain_region+Sex	1.790	1994.000
ENSG00000064313	0.001	-0.085	-0.001	-0.169	TAF2	0.007	~p_tau+(1|manifest)+cngeneson+pc_mito+brain_region+Sex	1.456	2254.000
ENSG00000020426	0.053	-0.085	0.042	-0.212	MNAT1	0.101	~p_tau+(1|manifest)+cngeneson+pc_mito+brain_region+Sex	1.497	2222.000
ENSG00000075213	0.289	-0.085	0.224	-0.395	SEMA3A	0.370	~p_tau+(1|manifest)+cngeneson+pc_mito+brain_region+Sex	2.082	1780.000
ENSG00000197601	0.070	-0.086	0.123	-0.294	FAR1	0.124	~p_tau+(1|manifest)+cngeneson+pc_mito+brain_region+Sex	0.989	2660.000
ENSG00000114127	0.018	-0.086	0.029	-0.201	XRN1	0.044	~p_tau+(1|manifest)+cngeneson+pc_mito+brain_region+Sex	1.517	2205.000
ENSG00000117519	0.893	-0.086	0.291	-0.463	CNN3	0.909	~p_tau+(1|manifest)+cngeneson+pc_mito+brain_region+Sex	13.009	140.000
ENSG00000128908	0.002	-0.086	0.035	-0.208	INO80	0.009	~p_tau+(1|manifest)+cngeneson+pc_mito+brain_region+Sex	1.819	1974.000
ENSG00000137145	0.030	-0.086	0.040	-0.212	DENND4C	0.065	~p_tau+(1|manifest)+cngeneson+pc_mito+brain_region+Sex	3.467	1068.000
ENSG00000125703	0.460	-0.087	0.096	-0.270	ATG4C	0.531	~p_tau+(1|manifest)+cngeneson+pc_mito+brain_region+Sex	3.081	1226.000
ENSG00000213160	0.265	-0.087	0.182	-0.357	KLHL23	0.346	~p_tau+(1|manifest)+cngeneson+pc_mito+brain_region+Sex	3.734	996.000
ENSG00000113273	0.634	-0.087	0.097	-0.272	ARSB	0.685	~p_tau+(1|manifest)+cngeneson+pc_mito+brain_region+Sex	2.509	1512.000
ENSG00000074603	0.133	-0.088	0.144	-0.319	DPP8	0.202	~p_tau+(1|manifest)+cngeneson+pc_mito+brain_region+Sex	3.286	1139.000
ENSG00000148925	0.003	-0.088	0.011	-0.187	BTBD10	0.013	~p_tau+(1|manifest)+cngeneson+pc_mito+brain_region+Sex	1.114	2559.000
ENSG00000185737	0.058	-0.088	-0.009	-0.167	NRG3	0.108	~p_tau+(1|manifest)+cngeneson+pc_mito+brain_region+Sex	8.958	307.000
ENSG00000090686	0.000	-0.088	-0.001	-0.175	USP48	0.001	~p_tau+(1|manifest)+cngeneson+pc_mito+brain_region+Sex	0.957	2683.000
ENSG00000110713	0.000	-0.088	0.025	-0.202	NUP98	0.003	~p_tau+(1|manifest)+cngeneson+pc_mito+brain_region+Sex	1.283	2421.000
ENSG00000131981	0.396	-0.088	0.323	-0.500	LGALS3	0.472	~p_tau+(1|manifest)+cngeneson+pc_mito+brain_region+Sex	6.234	518.000
ENSG00000101126	0.213	-0.088	0.035	-0.212	ADNP	0.289	~p_tau+(1|manifest)+cngeneson+pc_mito+brain_region+Sex	2.194	1708.000
ENSG00000163872	0.002	-0.088	0.006	-0.182	YEATS2	0.011	~p_tau+(1|manifest)+cngeneson+pc_mito+brain_region+Sex	1.311	2388.000
ENSG00000178538	0.857	-0.088	0.224	-0.401	CA8	0.876	~p_tau+(1|manifest)+cngeneson+pc_mito+brain_region+Sex	2.773	1372.000
ENSG00000107771	0.437	-0.089	0.076	-0.253	CCSER2	0.510	~p_tau+(1|manifest)+cngeneson+pc_mito+brain_region+Sex	5.082	690.000
ENSG00000144357	0.176	-0.089	0.007	-0.185	UBR3	0.250	~p_tau+(1|manifest)+cngeneson+pc_mito+brain_region+Sex	3.404	1100.000
ENSG00000106278	0.001	-0.089	0.010	-0.189	PTPRZ1	0.007	~p_tau+(1|manifest)+cngeneson+pc_mito+brain_region+Sex	12.944	141.000
ENSG00000136874	0.024	-0.089	0.030	-0.209	STX17	0.056	~p_tau+(1|manifest)+cngeneson+pc_mito+brain_region+Sex	1.093	2574.000
ENSG00000148516	0.325	-0.089	0.032	-0.211	ZEB1	0.404	~p_tau+(1|manifest)+cngeneson+pc_mito+brain_region+Sex	3.218	1169.000
ENSG00000144036	0.108	-0.089	-0.005	-0.174	EXOC6B	0.172	~p_tau+(1|manifest)+cngeneson+pc_mito+brain_region+Sex	2.954	1284.000
ENSG00000172733	0.278	-0.090	0.137	-0.316	PURG	0.360	~p_tau+(1|manifest)+cngeneson+pc_mito+brain_region+Sex	1.643	2105.000
ENSG00000112685	0.008	-0.090	0.079	-0.258	EXOC2	0.026	~p_tau+(1|manifest)+cngeneson+pc_mito+brain_region+Sex	2.001	1839.000
ENSG00000152620	0.310	-0.090	0.074	-0.254	NADK2	0.390	~p_tau+(1|manifest)+cngeneson+pc_mito+brain_region+Sex	3.283	1142.000
ENSG00000138398	0.004	-0.090	0.023	-0.203	PPIG	0.017	~p_tau+(1|manifest)+cngeneson+pc_mito+brain_region+Sex	1.420	2286.000
ENSG00000134982	0.032	-0.090	-0.019	-0.162	APC	0.069	~p_tau+(1|manifest)+cngeneson+pc_mito+brain_region+Sex	5.712	587.000
ENSG00000136631	0.154	-0.090	0.040	-0.220	VPS45	0.225	~p_tau+(1|manifest)+cngeneson+pc_mito+brain_region+Sex	0.838	2792.000
ENSG00000165533	0.034	-0.090	0.031	-0.211	TTC8	0.071	~p_tau+(1|manifest)+cngeneson+pc_mito+brain_region+Sex	0.756	2863.000
ENSG00000143924	0.069	-0.090	0.038	-0.219	EML4	0.123	~p_tau+(1|manifest)+cngeneson+pc_mito+brain_region+Sex	2.114	1762.000
ENSG00000152767	0.615	-0.091	0.105	-0.286	FARP1	0.668	~p_tau+(1|manifest)+cngeneson+pc_mito+brain_region+Sex	9.423	292.000
ENSG00000185658	0.386	-0.091	0.039	-0.220	BRWD1	0.463	~p_tau+(1|manifest)+cngeneson+pc_mito+brain_region+Sex	4.135	876.000
ENSG00000145241	0.003	-0.091	0.029	-0.210	CENPC	0.013	~p_tau+(1|manifest)+cngeneson+pc_mito+brain_region+Sex	0.385	3070.000
ENSG00000065613	0.023	-0.091	0.033	-0.214	SLK	0.053	~p_tau+(1|manifest)+cngeneson+pc_mito+brain_region+Sex	1.834	1960.000
ENSG00000198382	0.000	-0.091	-0.016	-0.165	UVRAG	0.003	~p_tau+(1|manifest)+cngeneson+pc_mito+brain_region+Sex	1.138	2545.000
ENSG00000136848	0.243	-0.091	0.101	-0.283	DAB2IP	0.321	~p_tau+(1|manifest)+cngeneson+pc_mito+brain_region+Sex	2.872	1329.000
ENSG00000125818	0.032	-0.091	0.012	-0.194	PSMF1	0.067	~p_tau+(1|manifest)+cngeneson+pc_mito+brain_region+Sex	1.495	2225.000
ENSG00000134369	0.940	-0.091	0.458	-0.641	NAV1	0.950	~p_tau+(1|manifest)+cngeneson+pc_mito+brain_region+Sex	18.375	62.000
ENSG00000103647	0.231	-0.091	0.119	-0.301	CORO2B	0.308	~p_tau+(1|manifest)+cngeneson+pc_mito+brain_region+Sex	1.172	2513.000
ENSG00000172869	0.166	-0.092	0.056	-0.241	DMXL1	0.239	~p_tau+(1|manifest)+cngeneson+pc_mito+brain_region+Sex	2.653	1431.000
ENSG00000151348	0.012	-0.093	0.043	-0.229	EXT2	0.033	~p_tau+(1|manifest)+cngeneson+pc_mito+brain_region+Sex	1.825	1970.000
ENSG00000123066	0.295	-0.093	0.031	-0.216	MED13L	0.376	~p_tau+(1|manifest)+cngeneson+pc_mito+brain_region+Sex	8.561	319.000
ENSG00000164574	0.764	-0.093	0.158	-0.343	GALNT10	0.798	~p_tau+(1|manifest)+cngeneson+pc_mito+brain_region+Sex	5.790	571.000
ENSG00000152583	0.058	-0.093	0.033	-0.219	SPARCL1	0.107	~p_tau+(1|manifest)+cngeneson+pc_mito+brain_region+Sex	4.437	807.000
ENSG00000092421	0.332	-0.093	0.160	-0.346	SEMA6A	0.411	~p_tau+(1|manifest)+cngeneson+pc_mito+brain_region+Sex	1.168	2520.000
ENSG00000138767	0.295	-0.093	0.104	-0.290	CNOT6L	0.376	~p_tau+(1|manifest)+cngeneson+pc_mito+brain_region+Sex	2.945	1292.000
ENSG00000114120	0.052	-0.093	0.071	-0.257	SLC25A36	0.099	~p_tau+(1|manifest)+cngeneson+pc_mito+brain_region+Sex	2.738	1386.000
ENSG00000153046	0.179	-0.093	0.086	-0.272	CDYL	0.253	~p_tau+(1|manifest)+cngeneson+pc_mito+brain_region+Sex	3.022	1252.000
ENSG00000149289	0.637	-0.093	0.111	-0.298	ZC3H12C	0.687	~p_tau+(1|manifest)+cngeneson+pc_mito+brain_region+Sex	3.070	1233.000
ENSG00000039123	0.009	-0.093	0.033	-0.220	MTREX	0.027	~p_tau+(1|manifest)+cngeneson+pc_mito+brain_region+Sex	1.651	2098.000
ENSG00000096872	0.006	-0.094	0.027	-0.215	IFT74	0.021	~p_tau+(1|manifest)+cngeneson+pc_mito+brain_region+Sex	0.578	2970.000
ENSG00000086102	0.002	-0.094	0.032	-0.220	NFX1	0.010	~p_tau+(1|manifest)+cngeneson+pc_mito+brain_region+Sex	1.746	2024.000
ENSG00000088854	0.230	-0.094	0.014	-0.202	C20orf194	0.308	~p_tau+(1|manifest)+cngeneson+pc_mito+brain_region+Sex	8.426	330.000
ENSG00000146872	0.006	-0.094	0.053	-0.241	TLK2	0.020	~p_tau+(1|manifest)+cngeneson+pc_mito+brain_region+Sex	1.320	2377.000
ENSG00000135679	0.385	-0.094	0.104	-0.292	MDM2	0.462	~p_tau+(1|manifest)+cngeneson+pc_mito+brain_region+Sex	4.242	846.000
ENSG00000150471	0.185	-0.094	0.006	-0.194	ADGRL3	0.259	~p_tau+(1|manifest)+cngeneson+pc_mito+brain_region+Sex	6.442	495.000
ENSG00000171033	0.814	-0.095	0.194	-0.384	PKIA	0.840	~p_tau+(1|manifest)+cngeneson+pc_mito+brain_region+Sex	3.625	1025.000
ENSG00000139163	0.006	-0.095	0.032	-0.222	ETNK1	0.020	~p_tau+(1|manifest)+cngeneson+pc_mito+brain_region+Sex	1.548	2172.000
ENSG00000104412	0.050	-0.095	0.050	-0.241	EMC2	0.095	~p_tau+(1|manifest)+cngeneson+pc_mito+brain_region+Sex	1.464	2250.000
ENSG00000116266	0.010	-0.095	0.041	-0.232	STXBP3	0.030	~p_tau+(1|manifest)+cngeneson+pc_mito+brain_region+Sex	0.576	2972.000
ENSG00000170324	0.271	-0.095	0.294	-0.485	FRMPD2	0.352	~p_tau+(1|manifest)+cngeneson+pc_mito+brain_region+Sex	3.119	1208.000
ENSG00000241058	0.189	-0.095	0.109	-0.300	NSUN6	0.264	~p_tau+(1|manifest)+cngeneson+pc_mito+brain_region+Sex	3.310	1133.000
ENSG00000055208	0.704	-0.096	0.157	-0.348	TAB2	0.748	~p_tau+(1|manifest)+cngeneson+pc_mito+brain_region+Sex	8.067	353.000
ENSG00000141627	0.431	-0.096	0.052	-0.244	DYM	0.505	~p_tau+(1|manifest)+cngeneson+pc_mito+brain_region+Sex	3.881	934.000
ENSG00000106078	0.776	-0.096	0.204	-0.396	COBL	0.808	~p_tau+(1|manifest)+cngeneson+pc_mito+brain_region+Sex	3.909	929.000
ENSG00000262655	0.692	-0.096	0.128	-0.320	SPON1	0.738	~p_tau+(1|manifest)+cngeneson+pc_mito+brain_region+Sex	6.489	488.000
ENSG00000011454	0.241	-0.096	0.026	-0.219	RABGAP1	0.319	~p_tau+(1|manifest)+cngeneson+pc_mito+brain_region+Sex	4.130	877.000
ENSG00000182263	0.731	-0.097	0.150	-0.343	FIGN	0.771	~p_tau+(1|manifest)+cngeneson+pc_mito+brain_region+Sex	5.350	644.000
ENSG00000047410	0.009	-0.097	0.013	-0.206	TPR	0.028	~p_tau+(1|manifest)+cngeneson+pc_mito+brain_region+Sex	0.739	2876.000
ENSG00000157764	0.085	-0.097	-0.007	-0.187	BRAF	0.144	~p_tau+(1|manifest)+cngeneson+pc_mito+brain_region+Sex	2.954	1283.000
ENSG00000170903	0.176	-0.097	0.103	-0.298	MSANTD4	0.250	~p_tau+(1|manifest)+cngeneson+pc_mito+brain_region+Sex	3.166	1190.000
ENSG00000042317	0.021	-0.098	0.090	-0.285	SPATA7	0.050	~p_tau+(1|manifest)+cngeneson+pc_mito+brain_region+Sex	2.280	1652.000
ENSG00000184985	0.569	-0.098	0.089	-0.285	SORCS2	0.628	~p_tau+(1|manifest)+cngeneson+pc_mito+brain_region+Sex	5.088	688.000
ENSG00000118263	0.745	-0.098	0.173	-0.369	KLF7	0.782	~p_tau+(1|manifest)+cngeneson+pc_mito+brain_region+Sex	2.515	1505.000
ENSG00000175387	0.022	-0.098	0.015	-0.211	SMAD2	0.051	~p_tau+(1|manifest)+cngeneson+pc_mito+brain_region+Sex	1.519	2202.000
ENSG00000198722	0.310	-0.098	0.069	-0.266	UNC13B	0.390	~p_tau+(1|manifest)+cngeneson+pc_mito+brain_region+Sex	1.742	2031.000
ENSG00000162836	0.244	-0.099	0.124	-0.321	ACP6	0.322	~p_tau+(1|manifest)+cngeneson+pc_mito+brain_region+Sex	2.918	1311.000
ENSG00000135537	0.124	-0.099	0.018	-0.216	AFG1L	0.192	~p_tau+(1|manifest)+cngeneson+pc_mito+brain_region+Sex	1.418	2291.000
ENSG00000128512	0.165	-0.099	0.002	-0.201	DOCK4	0.237	~p_tau+(1|manifest)+cngeneson+pc_mito+brain_region+Sex	14.797	103.000
ENSG00000119509	0.179	-0.099	0.002	-0.201	INVS	0.253	~p_tau+(1|manifest)+cngeneson+pc_mito+brain_region+Sex	1.570	2158.000
ENSG00000170348	0.450	-0.099	0.175	-0.374	TMED10	0.523	~p_tau+(1|manifest)+cngeneson+pc_mito+brain_region+Sex	3.136	1200.000
ENSG00000157483	0.224	-0.100	0.092	-0.291	MYO1E	0.301	~p_tau+(1|manifest)+cngeneson+pc_mito+brain_region+Sex	1.899	1914.000
ENSG00000198846	0.626	-0.100	0.113	-0.313	TOX	0.679	~p_tau+(1|manifest)+cngeneson+pc_mito+brain_region+Sex	2.255	1665.000
ENSG00000145284	0.161	-0.100	0.005	-0.205	SCD5	0.232	~p_tau+(1|manifest)+cngeneson+pc_mito+brain_region+Sex	0.420	3052.000
ENSG00000152990	0.198	-0.100	0.019	-0.219	ADGRA3	0.274	~p_tau+(1|manifest)+cngeneson+pc_mito+brain_region+Sex	3.743	993.000
ENSG00000122550	0.023	-0.100	0.000	-0.200	KLHL7	0.053	~p_tau+(1|manifest)+cngeneson+pc_mito+brain_region+Sex	1.520	2201.000
ENSG00000148143	0.526	-0.100	0.107	-0.307	ZNF462	0.591	~p_tau+(1|manifest)+cngeneson+pc_mito+brain_region+Sex	2.237	1679.000
ENSG00000170802	0.169	-0.101	0.109	-0.310	FOXN2	0.242	~p_tau+(1|manifest)+cngeneson+pc_mito+brain_region+Sex	3.269	1147.000
ENSG00000170522	0.139	-0.101	0.032	-0.234	ELOVL6	0.208	~p_tau+(1|manifest)+cngeneson+pc_mito+brain_region+Sex	1.405	2305.000
ENSG00000198879	0.154	-0.101	0.000	-0.201	SFMBT2	0.225	~p_tau+(1|manifest)+cngeneson+pc_mito+brain_region+Sex	1.141	2543.000
ENSG00000078687	0.061	-0.101	-0.004	-0.198	TNRC6C	0.111	~p_tau+(1|manifest)+cngeneson+pc_mito+brain_region+Sex	1.307	2392.000
ENSG00000151623	0.450	-0.101	0.103	-0.305	NR3C2	0.522	~p_tau+(1|manifest)+cngeneson+pc_mito+brain_region+Sex	5.708	588.000
ENSG00000186998	0.829	-0.101	0.444	-0.646	EMID1	0.852	~p_tau+(1|manifest)+cngeneson+pc_mito+brain_region+Sex	9.741	272.000
ENSG00000109220	0.011	-0.102	0.028	-0.231	CHIC2	0.032	~p_tau+(1|manifest)+cngeneson+pc_mito+brain_region+Sex	0.432	3048.000
ENSG00000180628	0.603	-0.102	0.108	-0.312	PCGF5	0.658	~p_tau+(1|manifest)+cngeneson+pc_mito+brain_region+Sex	4.394	821.000
ENSG00000205209	0.074	-0.103	0.047	-0.253	SCGB2B2	0.130	~p_tau+(1|manifest)+cngeneson+pc_mito+brain_region+Sex	1.411	2297.000
ENSG00000120860	0.002	-0.103	0.043	-0.249	WASHC3	0.010	~p_tau+(1|manifest)+cngeneson+pc_mito+brain_region+Sex	1.234	2454.000
ENSG00000128536	0.515	-0.103	0.087	-0.292	CDHR3	0.581	~p_tau+(1|manifest)+cngeneson+pc_mito+brain_region+Sex	12.937	144.000
ENSG00000018510	0.001	-0.103	-0.023	-0.183	AGPS	0.007	~p_tau+(1|manifest)+cngeneson+pc_mito+brain_region+Sex	0.875	2761.000
ENSG00000141564	0.012	-0.103	0.041	-0.247	RPTOR	0.034	~p_tau+(1|manifest)+cngeneson+pc_mito+brain_region+Sex	1.306	2393.000
ENSG00000135597	0.047	-0.103	-0.020	-0.187	REPS1	0.092	~p_tau+(1|manifest)+cngeneson+pc_mito+brain_region+Sex	4.168	865.000
ENSG00000108848	0.465	-0.103	0.137	-0.344	LUC7L3	0.535	~p_tau+(1|manifest)+cngeneson+pc_mito+brain_region+Sex	15.828	88.000
ENSG00000129675	0.318	-0.104	0.101	-0.308	ARHGEF6	0.397	~p_tau+(1|manifest)+cngeneson+pc_mito+brain_region+Sex	2.461	1543.000
ENSG00000010818	0.522	-0.104	0.160	-0.368	HIVEP2	0.588	~p_tau+(1|manifest)+cngeneson+pc_mito+brain_region+Sex	2.047	1808.000
ENSG00000135913	0.008	-0.104	0.002	-0.210	USP37	0.026	~p_tau+(1|manifest)+cngeneson+pc_mito+brain_region+Sex	1.426	2282.000
ENSG00000103047	0.002	-0.104	-0.002	-0.206	TANGO6	0.008	~p_tau+(1|manifest)+cngeneson+pc_mito+brain_region+Sex	0.887	2746.000
ENSG00000185736	0.457	-0.104	0.519	-0.727	ADARB2	0.529	~p_tau+(1|manifest)+cngeneson+pc_mito+brain_region+Sex	6.937	452.000
ENSG00000107890	0.212	-0.105	0.096	-0.305	ANKRD26	0.289	~p_tau+(1|manifest)+cngeneson+pc_mito+brain_region+Sex	3.185	1183.000
ENSG00000168216	0.071	-0.105	0.029	-0.238	LMBRD1	0.126	~p_tau+(1|manifest)+cngeneson+pc_mito+brain_region+Sex	1.651	2097.000
ENSG00000106049	0.155	-0.105	0.050	-0.260	HIBADH	0.227	~p_tau+(1|manifest)+cngeneson+pc_mito+brain_region+Sex	0.514	3007.000
ENSG00000196865	0.001	-0.105	-0.022	-0.188	NHLRC2	0.006	~p_tau+(1|manifest)+cngeneson+pc_mito+brain_region+Sex	1.338	2365.000
ENSG00000143919	0.462	-0.105	0.061	-0.272	CAMKMT	0.533	~p_tau+(1|manifest)+cngeneson+pc_mito+brain_region+Sex	1.754	2018.000
ENSG00000115504	0.628	-0.105	0.110	-0.321	EHBP1	0.680	~p_tau+(1|manifest)+cngeneson+pc_mito+brain_region+Sex	25.008	30.000
ENSG00000125676	0.001	-0.105	0.025	-0.236	THOC2	0.006	~p_tau+(1|manifest)+cngeneson+pc_mito+brain_region+Sex	2.494	1517.000
ENSG00000094975	0.013	-0.105	0.008	-0.219	SUCO	0.035	~p_tau+(1|manifest)+cngeneson+pc_mito+brain_region+Sex	1.830	1962.000
ENSG00000106443	0.143	-0.106	0.026	-0.237	PHF14	0.212	~p_tau+(1|manifest)+cngeneson+pc_mito+brain_region+Sex	1.713	2056.000
ENSG00000171988	0.023	-0.106	-0.030	-0.181	JMJD1C	0.053	~p_tau+(1|manifest)+cngeneson+pc_mito+brain_region+Sex	1.944	1884.000
ENSG00000128513	0.034	-0.106	0.005	-0.217	POT1	0.071	~p_tau+(1|manifest)+cngeneson+pc_mito+brain_region+Sex	1.269	2432.000
ENSG00000185942	0.473	-0.106	0.086	-0.298	NKAIN3	0.541	~p_tau+(1|manifest)+cngeneson+pc_mito+brain_region+Sex	14.450	111.000
ENSG00000171862	0.062	-0.106	0.031	-0.243	PTEN	0.114	~p_tau+(1|manifest)+cngeneson+pc_mito+brain_region+Sex	0.853	2776.000
ENSG00000140577	0.007	-0.106	0.033	-0.246	CRTC3	0.023	~p_tau+(1|manifest)+cngeneson+pc_mito+brain_region+Sex	2.112	1763.000
ENSG00000152061	0.318	-0.107	0.039	-0.253	RABGAP1L	0.397	~p_tau+(1|manifest)+cngeneson+pc_mito+brain_region+Sex	4.722	743.000
ENSG00000180488	0.002	-0.107	-0.036	-0.178	MIGA1	0.010	~p_tau+(1|manifest)+cngeneson+pc_mito+brain_region+Sex	1.194	2493.000
ENSG00000076067	0.075	-0.107	0.037	-0.251	RBMS2	0.131	~p_tau+(1|manifest)+cngeneson+pc_mito+brain_region+Sex	1.448	2265.000
ENSG00000197965	0.257	-0.107	0.100	-0.314	MPZL1	0.338	~p_tau+(1|manifest)+cngeneson+pc_mito+brain_region+Sex	4.452	803.000
ENSG00000118007	0.433	-0.107	0.069	-0.283	STAG1	0.506	~p_tau+(1|manifest)+cngeneson+pc_mito+brain_region+Sex	1.401	2310.000
ENSG00000130638	0.188	-0.107	0.016	-0.230	ATXN10	0.263	~p_tau+(1|manifest)+cngeneson+pc_mito+brain_region+Sex	2.920	1309.000
ENSG00000137561	0.173	-0.107	0.165	-0.380	TTPA	0.247	~p_tau+(1|manifest)+cngeneson+pc_mito+brain_region+Sex	2.241	1675.000
ENSG00000131023	0.001	-0.107	0.006	-0.221	LATS1	0.007	~p_tau+(1|manifest)+cngeneson+pc_mito+brain_region+Sex	1.308	2391.000
ENSG00000180530	0.137	-0.107	0.034	-0.249	NRIP1	0.207	~p_tau+(1|manifest)+cngeneson+pc_mito+brain_region+Sex	2.936	1297.000
ENSG00000166272	0.002	-0.108	-0.014	-0.201	WBP1L	0.008	~p_tau+(1|manifest)+cngeneson+pc_mito+brain_region+Sex	0.488	3025.000
ENSG00000188419	0.009	-0.108	0.020	-0.236	CHM	0.028	~p_tau+(1|manifest)+cngeneson+pc_mito+brain_region+Sex	1.751	2021.000
ENSG00000111850	0.126	-0.108	0.131	-0.347	SMIM8	0.194	~p_tau+(1|manifest)+cngeneson+pc_mito+brain_region+Sex	3.290	1137.000
ENSG00000111647	0.091	-0.108	0.083	-0.300	UHRF1BP1L	0.151	~p_tau+(1|manifest)+cngeneson+pc_mito+brain_region+Sex	4.168	866.000
ENSG00000139974	0.017	-0.109	0.024	-0.242	SLC38A6	0.043	~p_tau+(1|manifest)+cngeneson+pc_mito+brain_region+Sex	0.734	2884.000
ENSG00000164327	0.034	-0.109	0.026	-0.244	RICTOR	0.071	~p_tau+(1|manifest)+cngeneson+pc_mito+brain_region+Sex	2.289	1642.000
ENSG00000111731	0.012	-0.109	0.000	-0.217	C2CD5	0.034	~p_tau+(1|manifest)+cngeneson+pc_mito+brain_region+Sex	1.067	2599.000
ENSG00000184194	0.019	-0.109	0.124	-0.343	GPR173	0.046	~p_tau+(1|manifest)+cngeneson+pc_mito+brain_region+Sex	2.472	1536.000
ENSG00000038532	0.551	-0.109	0.106	-0.325	CLEC16A	0.612	~p_tau+(1|manifest)+cngeneson+pc_mito+brain_region+Sex	10.312	243.000
ENSG00000170035	0.608	-0.110	0.107	-0.326	UBE2E3	0.661	~p_tau+(1|manifest)+cngeneson+pc_mito+brain_region+Sex	0.916	2718.000
ENSG00000182923	0.000	-0.110	-0.049	-0.170	CEP63	0.002	~p_tau+(1|manifest)+cngeneson+pc_mito+brain_region+Sex	1.174	2512.000
ENSG00000198964	0.522	-0.110	0.079	-0.298	SGMS1	0.588	~p_tau+(1|manifest)+cngeneson+pc_mito+brain_region+Sex	2.053	1796.000
ENSG00000028203	0.231	-0.110	0.045	-0.264	VEZT	0.308	~p_tau+(1|manifest)+cngeneson+pc_mito+brain_region+Sex	3.095	1220.000
ENSG00000047249	0.224	-0.110	0.054	-0.274	ATP6V1H	0.301	~p_tau+(1|manifest)+cngeneson+pc_mito+brain_region+Sex	0.459	3040.000
ENSG00000130396	0.161	-0.110	0.032	-0.253	AFDN	0.233	~p_tau+(1|manifest)+cngeneson+pc_mito+brain_region+Sex	1.435	2276.000
ENSG00000171055	0.004	-0.111	0.011	-0.232	FEZ2	0.015	~p_tau+(1|manifest)+cngeneson+pc_mito+brain_region+Sex	1.997	1842.000
ENSG00000183580	0.012	-0.111	-0.038	-0.183	FBXL7	0.033	~p_tau+(1|manifest)+cngeneson+pc_mito+brain_region+Sex	3.302	1136.000
ENSG00000082068	0.186	-0.111	0.008	-0.230	WDR70	0.261	~p_tau+(1|manifest)+cngeneson+pc_mito+brain_region+Sex	2.869	1330.000
ENSG00000048471	0.479	-0.111	0.124	-0.346	SNX29	0.547	~p_tau+(1|manifest)+cngeneson+pc_mito+brain_region+Sex	3.940	923.000
ENSG00000164144	0.094	-0.112	0.047	-0.270	ARFIP1	0.155	~p_tau+(1|manifest)+cngeneson+pc_mito+brain_region+Sex	2.925	1304.000
ENSG00000066651	0.333	-0.112	0.079	-0.303	TRMT11	0.412	~p_tau+(1|manifest)+cngeneson+pc_mito+brain_region+Sex	2.051	1799.000
ENSG00000138376	0.322	-0.112	0.110	-0.334	BARD1	0.401	~p_tau+(1|manifest)+cngeneson+pc_mito+brain_region+Sex	4.566	782.000
ENSG00000144847	0.563	-0.112	0.094	-0.319	IGSF11	0.622	~p_tau+(1|manifest)+cngeneson+pc_mito+brain_region+Sex	1.786	1996.000
ENSG00000125462	0.716	-0.113	0.208	-0.433	C1orf61	0.759	~p_tau+(1|manifest)+cngeneson+pc_mito+brain_region+Sex	30.298	20.000
ENSG00000214456	0.407	-0.113	0.319	-0.545	PLIN5	0.483	~p_tau+(1|manifest)+cngeneson+pc_mito+brain_region+Sex	7.243	419.000
ENSG00000175161	0.500	-0.113	0.076	-0.302	CADM2	0.567	~p_tau+(1|manifest)+cngeneson+pc_mito+brain_region+Sex	26.223	26.000
ENSG00000143669	0.546	-0.114	0.160	-0.388	LYST	0.608	~p_tau+(1|manifest)+cngeneson+pc_mito+brain_region+Sex	7.491	395.000
ENSG00000170759	0.017	-0.114	0.015	-0.243	KIF5B	0.043	~p_tau+(1|manifest)+cngeneson+pc_mito+brain_region+Sex	2.059	1795.000
ENSG00000275052	0.004	-0.114	0.027	-0.255	PPP4R3B	0.015	~p_tau+(1|manifest)+cngeneson+pc_mito+brain_region+Sex	2.537	1485.000
ENSG00000119778	0.095	-0.114	0.015	-0.243	ATAD2B	0.157	~p_tau+(1|manifest)+cngeneson+pc_mito+brain_region+Sex	1.850	1951.000
ENSG00000104325	0.425	-0.114	0.164	-0.393	DECR1	0.499	~p_tau+(1|manifest)+cngeneson+pc_mito+brain_region+Sex	3.392	1101.000
ENSG00000154310	0.099	-0.115	-0.012	-0.217	TNIK	0.161	~p_tau+(1|manifest)+cngeneson+pc_mito+brain_region+Sex	6.749	463.000
ENSG00000101868	0.340	-0.115	0.044	-0.274	POLA1	0.418	~p_tau+(1|manifest)+cngeneson+pc_mito+brain_region+Sex	2.035	1818.000
ENSG00000086758	0.039	-0.115	0.008	-0.237	HUWE1	0.079	~p_tau+(1|manifest)+cngeneson+pc_mito+brain_region+Sex	3.203	1174.000
ENSG00000196597	0.048	-0.115	0.000	-0.230	ZNF782	0.093	~p_tau+(1|manifest)+cngeneson+pc_mito+brain_region+Sex	2.173	1718.000
ENSG00000068784	0.063	-0.115	-0.025	-0.206	SRBD1	0.115	~p_tau+(1|manifest)+cngeneson+pc_mito+brain_region+Sex	0.781	2850.000
ENSG00000114933	0.725	-0.115	0.196	-0.427	INO80D	0.766	~p_tau+(1|manifest)+cngeneson+pc_mito+brain_region+Sex	50.019	5.000
ENSG00000106052	0.030	-0.115	0.025	-0.256	TAX1BP1	0.065	~p_tau+(1|manifest)+cngeneson+pc_mito+brain_region+Sex	2.086	1777.000
ENSG00000075292	0.031	-0.116	-0.027	-0.204	ZNF638	0.066	~p_tau+(1|manifest)+cngeneson+pc_mito+brain_region+Sex	2.119	1758.000
ENSG00000164031	0.000	-0.116	-0.041	-0.190	DNAJB14	0.001	~p_tau+(1|manifest)+cngeneson+pc_mito+brain_region+Sex	1.392	2316.000
ENSG00000121542	0.001	-0.116	-0.010	-0.222	SEC22A	0.006	~p_tau+(1|manifest)+cngeneson+pc_mito+brain_region+Sex	0.835	2793.000
ENSG00000135457	0.084	-0.116	0.030	-0.262	TFCP2	0.143	~p_tau+(1|manifest)+cngeneson+pc_mito+brain_region+Sex	1.350	2356.000
ENSG00000004766	0.084	-0.116	0.020	-0.252	VPS50	0.143	~p_tau+(1|manifest)+cngeneson+pc_mito+brain_region+Sex	0.880	2754.000
ENSG00000082293	0.216	-0.116	0.200	-0.432	COL19A1	0.292	~p_tau+(1|manifest)+cngeneson+pc_mito+brain_region+Sex	1.502	2216.000
ENSG00000068650	0.531	-0.116	0.133	-0.365	ATP11A	0.594	~p_tau+(1|manifest)+cngeneson+pc_mito+brain_region+Sex	5.781	573.000
ENSG00000168702	0.343	-0.116	0.036	-0.269	LRP1B	0.421	~p_tau+(1|manifest)+cngeneson+pc_mito+brain_region+Sex	27.162	24.000
ENSG00000005810	0.005	-0.117	-0.037	-0.196	MYCBP2	0.018	~p_tau+(1|manifest)+cngeneson+pc_mito+brain_region+Sex	1.325	2375.000
ENSG00000204120	0.305	-0.117	0.039	-0.272	GIGYF2	0.386	~p_tau+(1|manifest)+cngeneson+pc_mito+brain_region+Sex	4.022	906.000
ENSG00000198561	0.001	-0.117	-0.042	-0.192	CTNND1	0.004	~p_tau+(1|manifest)+cngeneson+pc_mito+brain_region+Sex	1.190	2497.000
ENSG00000116117	0.007	-0.117	-0.047	-0.187	PARD3B	0.024	~p_tau+(1|manifest)+cngeneson+pc_mito+brain_region+Sex	1.122	2552.000
ENSG00000109171	0.004	-0.117	-0.015	-0.219	SLAIN2	0.014	~p_tau+(1|manifest)+cngeneson+pc_mito+brain_region+Sex	0.995	2657.000
ENSG00000131323	0.097	-0.117	0.041	-0.275	TRAF3	0.159	~p_tau+(1|manifest)+cngeneson+pc_mito+brain_region+Sex	1.474	2244.000
ENSG00000133103	0.112	-0.117	0.027	-0.261	COG6	0.177	~p_tau+(1|manifest)+cngeneson+pc_mito+brain_region+Sex	1.733	2042.000
ENSG00000141429	0.060	-0.117	0.012	-0.246	GALNT1	0.111	~p_tau+(1|manifest)+cngeneson+pc_mito+brain_region+Sex	2.147	1736.000
ENSG00000079134	0.009	-0.117	0.010	-0.244	THOC1	0.027	~p_tau+(1|manifest)+cngeneson+pc_mito+brain_region+Sex	1.935	1889.000
ENSG00000091129	0.026	-0.117	0.014	-0.249	NRCAM	0.058	~p_tau+(1|manifest)+cngeneson+pc_mito+brain_region+Sex	21.683	41.000
ENSG00000189221	0.036	-0.117	-0.031	-0.203	MAOA	0.075	~p_tau+(1|manifest)+cngeneson+pc_mito+brain_region+Sex	0.917	2717.000
ENSG00000078328	0.772	-0.118	0.205	-0.440	RBFOX1	0.806	~p_tau+(1|manifest)+cngeneson+pc_mito+brain_region+Sex	1.575	2156.000
ENSG00000143514	0.035	-0.118	0.003	-0.238	TP53BP2	0.073	~p_tau+(1|manifest)+cngeneson+pc_mito+brain_region+Sex	1.912	1903.000
ENSG00000166225	0.238	-0.118	0.054	-0.291	FRS2	0.317	~p_tau+(1|manifest)+cngeneson+pc_mito+brain_region+Sex	2.074	1784.000
ENSG00000163848	0.160	-0.118	0.043	-0.280	ZNF148	0.232	~p_tau+(1|manifest)+cngeneson+pc_mito+brain_region+Sex	1.848	1952.000
ENSG00000176204	0.471	-0.119	0.169	-0.406	LRRTM4	0.540	~p_tau+(1|manifest)+cngeneson+pc_mito+brain_region+Sex	3.144	1198.000
ENSG00000148672	0.302	-0.119	0.032	-0.269	GLUD1	0.383	~p_tau+(1|manifest)+cngeneson+pc_mito+brain_region+Sex	5.344	645.000
ENSG00000138430	0.150	-0.119	0.009	-0.247	OLA1	0.221	~p_tau+(1|manifest)+cngeneson+pc_mito+brain_region+Sex	1.925	1896.000
ENSG00000173889	0.034	-0.119	-0.005	-0.232	PHC3	0.072	~p_tau+(1|manifest)+cngeneson+pc_mito+brain_region+Sex	2.008	1833.000
ENSG00000139746	0.085	-0.119	-0.008	-0.230	RBM26	0.144	~p_tau+(1|manifest)+cngeneson+pc_mito+brain_region+Sex	2.756	1382.000
ENSG00000101901	0.002	-0.119	-0.031	-0.207	ALG13	0.009	~p_tau+(1|manifest)+cngeneson+pc_mito+brain_region+Sex	0.784	2844.000
ENSG00000180773	0.017	-0.119	-0.016	-0.222	SLC36A4	0.042	~p_tau+(1|manifest)+cngeneson+pc_mito+brain_region+Sex	1.069	2594.000
ENSG00000153922	0.037	-0.119	-0.001	-0.238	CHD1	0.076	~p_tau+(1|manifest)+cngeneson+pc_mito+brain_region+Sex	1.384	2326.000
ENSG00000163788	0.467	-0.119	0.105	-0.343	SNRK	0.536	~p_tau+(1|manifest)+cngeneson+pc_mito+brain_region+Sex	2.769	1375.000
ENSG00000118058	0.018	-0.119	0.017	-0.256	KMT2A	0.045	~p_tau+(1|manifest)+cngeneson+pc_mito+brain_region+Sex	1.675	2084.000
ENSG00000111596	0.080	-0.120	0.047	-0.286	CNOT2	0.137	~p_tau+(1|manifest)+cngeneson+pc_mito+brain_region+Sex	0.814	2817.000
ENSG00000198838	0.072	-0.120	-0.013	-0.226	RYR3	0.126	~p_tau+(1|manifest)+cngeneson+pc_mito+brain_region+Sex	8.497	324.000
ENSG00000162695	0.002	-0.120	-0.017	-0.223	SLC30A7	0.009	~p_tau+(1|manifest)+cngeneson+pc_mito+brain_region+Sex	2.090	1776.000
ENSG00000162885	0.007	-0.120	0.001	-0.240	B3GALNT2	0.024	~p_tau+(1|manifest)+cngeneson+pc_mito+brain_region+Sex	1.289	2416.000
ENSG00000112584	0.153	-0.120	0.046	-0.287	FAM120B	0.224	~p_tau+(1|manifest)+cngeneson+pc_mito+brain_region+Sex	2.328	1615.000
ENSG00000138095	0.006	-0.120	0.022	-0.263	LRPPRC	0.021	~p_tau+(1|manifest)+cngeneson+pc_mito+brain_region+Sex	0.700	2914.000
ENSG00000080823	0.061	-0.120	0.039	-0.280	MOK	0.113	~p_tau+(1|manifest)+cngeneson+pc_mito+brain_region+Sex	1.563	2163.000
ENSG00000019991	0.733	-0.121	0.273	-0.514	HGF	0.772	~p_tau+(1|manifest)+cngeneson+pc_mito+brain_region+Sex	12.186	170.000
ENSG00000174839	0.002	-0.121	-0.026	-0.215	DENND6A	0.008	~p_tau+(1|manifest)+cngeneson+pc_mito+brain_region+Sex	1.075	2589.000
ENSG00000196704	0.413	-0.121	0.251	-0.492	AMZ2	0.488	~p_tau+(1|manifest)+cngeneson+pc_mito+brain_region+Sex	4.009	910.000
ENSG00000066032	0.114	-0.121	-0.012	-0.229	CTNNA2	0.180	~p_tau+(1|manifest)+cngeneson+pc_mito+brain_region+Sex	10.150	253.000

ENSG00000101464	0.031	-0.121	0.001	-0.243	PIGU	0.066	~p_tau+(1|manifest)+cngeneson+pc_mito+brain_region+Sex	1.276	2424.000
ENSG00000120071	0.133	-0.122	0.001	-0.245	KANSL1	0.201	~p_tau+(1|manifest)+cngeneson+pc_mito+brain_region+Sex	16.182	83.000
ENSG00000163116	0.464	-0.122	0.103	-0.346	STPG2	0.534	~p_tau+(1|manifest)+cngeneson+pc_mito+brain_region+Sex	0.939	2698.000
ENSG00000146463	0.130	-0.122	0.023	-0.267	ZMYM4	0.198	~p_tau+(1|manifest)+cngeneson+pc_mito+brain_region+Sex	1.299	2407.000
ENSG00000176834	0.139	-0.122	0.130	-0.374	VSIG10	0.209	~p_tau+(1|manifest)+cngeneson+pc_mito+brain_region+Sex	3.470	1065.000
ENSG00000139218	0.044	-0.122	0.006	-0.250	SCAF11	0.086	~p_tau+(1|manifest)+cngeneson+pc_mito+brain_region+Sex	2.988	1270.000
ENSG00000120519	0.078	-0.122	0.016	-0.261	SLC10A7	0.135	~p_tau+(1|manifest)+cngeneson+pc_mito+brain_region+Sex	1.196	2487.000
ENSG00000131089	0.116	-0.122	0.025	-0.270	ARHGEF9	0.182	~p_tau+(1|manifest)+cngeneson+pc_mito+brain_region+Sex	2.149	1732.000
ENSG00000132694	0.296	-0.122	0.059	-0.304	ARHGEF11	0.377	~p_tau+(1|manifest)+cngeneson+pc_mito+brain_region+Sex	4.843	719.000
ENSG00000175054	0.037	-0.123	0.003	-0.249	ATR	0.076	~p_tau+(1|manifest)+cngeneson+pc_mito+brain_region+Sex	2.085	1779.000
ENSG00000168818	0.002	-0.123	-0.006	-0.241	STX18	0.009	~p_tau+(1|manifest)+cngeneson+pc_mito+brain_region+Sex	1.021	2630.000
ENSG00000171444	0.543	-0.124	0.103	-0.350	MCC	0.604	~p_tau+(1|manifest)+cngeneson+pc_mito+brain_region+Sex	2.086	1778.000
ENSG00000136754	0.140	-0.124	-0.001	-0.246	ABI1	0.209	~p_tau+(1|manifest)+cngeneson+pc_mito+brain_region+Sex	1.418	2290.000
ENSG00000135837	0.260	-0.124	0.094	-0.342	CEP350	0.341	~p_tau+(1|manifest)+cngeneson+pc_mito+brain_region+Sex	6.783	461.000
ENSG00000109920	0.334	-0.124	0.138	-0.386	FNBP4	0.413	~p_tau+(1|manifest)+cngeneson+pc_mito+brain_region+Sex	5.526	618.000
ENSG00000165494	0.000	-0.124	-0.042	-0.206	PCF11	0.002	~p_tau+(1|manifest)+cngeneson+pc_mito+brain_region+Sex	0.564	2976.000
ENSG00000198836	0.005	-0.125	0.002	-0.251	OPA1	0.017	~p_tau+(1|manifest)+cngeneson+pc_mito+brain_region+Sex	1.273	2428.000
ENSG00000079246	0.282	-0.125	0.052	-0.302	XRCC5	0.364	~p_tau+(1|manifest)+cngeneson+pc_mito+brain_region+Sex	4.571	779.000
ENSG00000145743	0.135	-0.125	-0.004	-0.246	FBXL17	0.205	~p_tau+(1|manifest)+cngeneson+pc_mito+brain_region+Sex	2.935	1298.000
ENSG00000070366	0.034	-0.125	0.032	-0.283	SMG6	0.071	~p_tau+(1|manifest)+cngeneson+pc_mito+brain_region+Sex	2.147	1735.000
ENSG00000055163	0.361	-0.126	0.101	-0.352	CYFIP2	0.439	~p_tau+(1|manifest)+cngeneson+pc_mito+brain_region+Sex	4.485	799.000
ENSG00000092964	0.161	-0.126	0.004	-0.256	DPYSL2	0.232	~p_tau+(1|manifest)+cngeneson+pc_mito+brain_region+Sex	7.633	381.000
ENSG00000147905	0.378	-0.127	0.050	-0.303	ZCCHC7	0.455	~p_tau+(1|manifest)+cngeneson+pc_mito+brain_region+Sex	2.431	1557.000
ENSG00000162434	0.503	-0.127	0.100	-0.353	JAK1	0.570	~p_tau+(1|manifest)+cngeneson+pc_mito+brain_region+Sex	6.379	501.000
ENSG00000170242	0.178	-0.127	0.008	-0.262	USP47	0.252	~p_tau+(1|manifest)+cngeneson+pc_mito+brain_region+Sex	4.160	870.000
ENSG00000103978	0.062	-0.127	0.039	-0.293	TMEM87A	0.113	~p_tau+(1|manifest)+cngeneson+pc_mito+brain_region+Sex	1.828	1965.000
ENSG00000181555	0.075	-0.127	-0.003	-0.252	SETD2	0.131	~p_tau+(1|manifest)+cngeneson+pc_mito+brain_region+Sex	2.588	1462.000
ENSG00000154240	0.459	-0.127	0.088	-0.342	CEP112	0.530	~p_tau+(1|manifest)+cngeneson+pc_mito+brain_region+Sex	1.759	2012.000
ENSG00000138814	0.175	-0.128	0.011	-0.267	PPP3CA	0.249	~p_tau+(1|manifest)+cngeneson+pc_mito+brain_region+Sex	8.525	321.000
ENSG00000167632	0.134	-0.128	0.025	-0.281	TRAPPC9	0.202	~p_tau+(1|manifest)+cngeneson+pc_mito+brain_region+Sex	3.433	1081.000
ENSG00000162407	0.736	-0.128	0.204	-0.460	PLPP3	0.774	~p_tau+(1|manifest)+cngeneson+pc_mito+brain_region+Sex	6.259	515.000
ENSG00000188994	0.118	-0.128	-0.003	-0.253	ZNF292	0.183	~p_tau+(1|manifest)+cngeneson+pc_mito+brain_region+Sex	3.157	1195.000
ENSG00000152127	0.782	-0.128	0.243	-0.499	MGAT5	0.813	~p_tau+(1|manifest)+cngeneson+pc_mito+brain_region+Sex	14.120	119.000
ENSG00000088812	0.017	-0.128	-0.021	-0.235	ATRN	0.042	~p_tau+(1|manifest)+cngeneson+pc_mito+brain_region+Sex	1.984	1853.000
ENSG00000155313	0.114	-0.128	0.031	-0.288	USP25	0.180	~p_tau+(1|manifest)+cngeneson+pc_mito+brain_region+Sex	2.222	1689.000
ENSG00000163738	0.368	-0.128	0.116	-0.372	MTHFD2L	0.445	~p_tau+(1|manifest)+cngeneson+pc_mito+brain_region+Sex	1.852	1948.000
ENSG00000186073	0.028	-0.128	-0.041	-0.215	C15orf41	0.062	~p_tau+(1|manifest)+cngeneson+pc_mito+brain_region+Sex	1.589	2150.000
ENSG00000160563	0.176	-0.129	0.028	-0.285	MED27	0.250	~p_tau+(1|manifest)+cngeneson+pc_mito+brain_region+Sex	3.246	1155.000
ENSG00000143190	0.066	-0.129	-0.021	-0.236	POU2F1	0.118	~p_tau+(1|manifest)+cngeneson+pc_mito+brain_region+Sex	5.268	656.000
ENSG00000127720	0.226	-0.129	0.023	-0.280	METTL25	0.302	~p_tau+(1|manifest)+cngeneson+pc_mito+brain_region+Sex	1.398	2311.000
ENSG00000165288	0.024	-0.129	0.011	-0.270	BRWD3	0.056	~p_tau+(1|manifest)+cngeneson+pc_mito+brain_region+Sex	2.098	1771.000
ENSG00000145348	0.150	-0.130	0.006	-0.265	TBCK	0.220	~p_tau+(1|manifest)+cngeneson+pc_mito+brain_region+Sex	1.791	1993.000
ENSG00000150625	0.279	-0.130	0.031	-0.290	GPM6A	0.360	~p_tau+(1|manifest)+cngeneson+pc_mito+brain_region+Sex	17.298	73.000
ENSG00000117569	0.075	-0.130	0.010	-0.270	PTBP2	0.131	~p_tau+(1|manifest)+cngeneson+pc_mito+brain_region+Sex	2.047	1807.000
ENSG00000148950	0.112	-0.130	0.035	-0.295	IMMP1L	0.177	~p_tau+(1|manifest)+cngeneson+pc_mito+brain_region+Sex	1.465	2248.000
ENSG00000080345	0.009	-0.130	-0.028	-0.232	RIF1	0.028	~p_tau+(1|manifest)+cngeneson+pc_mito+brain_region+Sex	0.822	2808.000
ENSG00000130856	0.009	-0.130	0.000	-0.260	ZNF236	0.026	~p_tau+(1|manifest)+cngeneson+pc_mito+brain_region+Sex	1.754	2017.000
ENSG00000160746	0.010	-0.131	-0.012	-0.249	ANO10	0.029	~p_tau+(1|manifest)+cngeneson+pc_mito+brain_region+Sex	1.758	2014.000
ENSG00000170017	0.214	-0.131	0.017	-0.278	ALCAM	0.290	~p_tau+(1|manifest)+cngeneson+pc_mito+brain_region+Sex	2.013	1828.000
ENSG00000174373	0.090	-0.131	-0.016	-0.245	RALGAPA1	0.150	~p_tau+(1|manifest)+cngeneson+pc_mito+brain_region+Sex	2.565	1476.000
ENSG00000100580	0.017	-0.131	-0.018	-0.243	TMED8	0.043	~p_tau+(1|manifest)+cngeneson+pc_mito+brain_region+Sex	1.147	2535.000
ENSG00000136895	0.428	-0.131	0.296	-0.558	GARNL3	0.502	~p_tau+(1|manifest)+cngeneson+pc_mito+brain_region+Sex	7.248	416.000
ENSG00000046889	0.018	-0.131	-0.043	-0.219	PREX2	0.044	~p_tau+(1|manifest)+cngeneson+pc_mito+brain_region+Sex	2.424	1561.000
ENSG00000143614	0.074	-0.131	0.039	-0.301	GATAD2B	0.130	~p_tau+(1|manifest)+cngeneson+pc_mito+brain_region+Sex	2.110	1765.000
ENSG00000062650	0.001	-0.131	-0.028	-0.234	WAPL	0.005	~p_tau+(1|manifest)+cngeneson+pc_mito+brain_region+Sex	1.646	2102.000
ENSG00000060339	0.029	-0.131	0.059	-0.321	CCAR1	0.063	~p_tau+(1|manifest)+cngeneson+pc_mito+brain_region+Sex	2.921	1308.000
ENSG00000166233	0.191	-0.132	0.020	-0.284	ARIH1	0.266	~p_tau+(1|manifest)+cngeneson+pc_mito+brain_region+Sex	3.317	1128.000
ENSG00000151065	0.009	-0.132	-0.031	-0.233	DCP1B	0.027	~p_tau+(1|manifest)+cngeneson+pc_mito+brain_region+Sex	0.920	2713.000
ENSG00000103489	0.336	-0.132	0.119	-0.383	XYLT1	0.415	~p_tau+(1|manifest)+cngeneson+pc_mito+brain_region+Sex	0.405	3062.000
ENSG00000166266	0.001	-0.132	-0.028	-0.236	CUL5	0.007	~p_tau+(1|manifest)+cngeneson+pc_mito+brain_region+Sex	1.929	1893.000
ENSG00000183354	0.207	-0.132	0.050	-0.314	KIAA2026	0.283	~p_tau+(1|manifest)+cngeneson+pc_mito+brain_region+Sex	3.076	1230.000
ENSG00000162599	0.044	-0.132	-0.030	-0.235	NFIA	0.087	~p_tau+(1|manifest)+cngeneson+pc_mito+brain_region+Sex	10.312	242.000
ENSG00000186166	0.021	-0.132	0.031	-0.295	CCDC84	0.049	~p_tau+(1|manifest)+cngeneson+pc_mito+brain_region+Sex	2.490	1519.000
ENSG00000186314	0.432	-0.132	0.092	-0.357	PRELID2	0.505	~p_tau+(1|manifest)+cngeneson+pc_mito+brain_region+Sex	0.886	2750.000
ENSG00000171503	0.035	-0.133	0.021	-0.286	ETFDH	0.073	~p_tau+(1|manifest)+cngeneson+pc_mito+brain_region+Sex	2.400	1572.000
ENSG00000168038	0.284	-0.133	0.041	-0.307	ULK4	0.366	~p_tau+(1|manifest)+cngeneson+pc_mito+brain_region+Sex	2.143	1741.000
ENSG00000110066	0.074	-0.134	0.016	-0.283	KMT5B	0.130	~p_tau+(1|manifest)+cngeneson+pc_mito+brain_region+Sex	1.864	1942.000
ENSG00000101654	0.003	-0.134	-0.028	-0.240	RNMT	0.013	~p_tau+(1|manifest)+cngeneson+pc_mito+brain_region+Sex	0.928	2706.000
ENSG00000126785	0.633	-0.134	0.252	-0.519	RHOJ	0.684	~p_tau+(1|manifest)+cngeneson+pc_mito+brain_region+Sex	3.310	1132.000
ENSG00000170248	0.035	-0.135	0.021	-0.291	PDCD6IP	0.073	~p_tau+(1|manifest)+cngeneson+pc_mito+brain_region+Sex	2.522	1495.000
ENSG00000169783	0.624	-0.135	0.233	-0.503	LINGO1	0.677	~p_tau+(1|manifest)+cngeneson+pc_mito+brain_region+Sex	78.340	1.000
ENSG00000102466	0.325	-0.135	0.037	-0.307	FGF14	0.404	~p_tau+(1|manifest)+cngeneson+pc_mito+brain_region+Sex	1.875	1934.000
ENSG00000133687	0.752	-0.135	0.271	-0.542	TMTC1	0.788	~p_tau+(1|manifest)+cngeneson+pc_mito+brain_region+Sex	10.954	212.000
ENSG00000196586	0.130	-0.135	0.003	-0.274	MYO6	0.198	~p_tau+(1|manifest)+cngeneson+pc_mito+brain_region+Sex	4.173	863.000
ENSG00000164197	0.080	-0.136	-0.023	-0.248	RNF180	0.138	~p_tau+(1|manifest)+cngeneson+pc_mito+brain_region+Sex	0.913	2722.000
ENSG00000151292	0.002	-0.136	-0.056	-0.216	CSNK1G3	0.010	~p_tau+(1|manifest)+cngeneson+pc_mito+brain_region+Sex	1.003	2648.000
ENSG00000177453	0.115	-0.136	0.010	-0.281	NIM1K	0.181	~p_tau+(1|manifest)+cngeneson+pc_mito+brain_region+Sex	1.760	2010.000
ENSG00000083290	0.391	-0.136	0.206	-0.478	ULK2	0.468	~p_tau+(1|manifest)+cngeneson+pc_mito+brain_region+Sex	1.591	2147.000
ENSG00000165392	0.353	-0.136	0.071	-0.343	WRN	0.431	~p_tau+(1|manifest)+cngeneson+pc_mito+brain_region+Sex	4.576	776.000
ENSG00000100644	0.421	-0.136	0.116	-0.388	HIF1A	0.496	~p_tau+(1|manifest)+cngeneson+pc_mito+brain_region+Sex	3.782	974.000
ENSG00000100614	0.003	-0.136	-0.051	-0.221	PPM1A	0.012	~p_tau+(1|manifest)+cngeneson+pc_mito+brain_region+Sex	1.461	2251.000
ENSG00000196405	0.069	-0.136	0.039	-0.312	EVL	0.123	~p_tau+(1|manifest)+cngeneson+pc_mito+brain_region+Sex	1.610	2138.000
ENSG00000173852	0.532	-0.136	0.160	-0.433	DPY19L1	0.595	~p_tau+(1|manifest)+cngeneson+pc_mito+brain_region+Sex	7.730	372.000
ENSG00000011201	0.676	-0.136	0.164	-0.437	ANOS1	0.722	~p_tau+(1|manifest)+cngeneson+pc_mito+brain_region+Sex	2.682	1414.000
ENSG00000170776	0.718	-0.136	0.196	-0.469	AKAP13	0.760	~p_tau+(1|manifest)+cngeneson+pc_mito+brain_region+Sex	13.385	135.000
ENSG00000147133	0.001	-0.136	-0.050	-0.223	TAF1	0.007	~p_tau+(1|manifest)+cngeneson+pc_mito+brain_region+Sex	1.012	2638.000
ENSG00000067208	0.246	-0.137	0.022	-0.296	EVI5	0.324	~p_tau+(1|manifest)+cngeneson+pc_mito+brain_region+Sex	2.934	1299.000
ENSG00000113391	0.058	-0.137	-0.026	-0.248	FAM172A	0.108	~p_tau+(1|manifest)+cngeneson+pc_mito+brain_region+Sex	1.314	2385.000
ENSG00000122482	0.270	-0.137	0.026	-0.300	ZNF644	0.352	~p_tau+(1|manifest)+cngeneson+pc_mito+brain_region+Sex	2.920	1310.000
ENSG00000106571	0.277	-0.137	0.031	-0.305	GLI3	0.359	~p_tau+(1|manifest)+cngeneson+pc_mito+brain_region+Sex	2.671	1421.000
ENSG00000010244	0.130	-0.137	0.064	-0.339	ZNF207	0.198	~p_tau+(1|manifest)+cngeneson+pc_mito+brain_region+Sex	2.191	1709.000
ENSG00000213079	0.095	-0.137	0.017	-0.291	SCAF8	0.157	~p_tau+(1|manifest)+cngeneson+pc_mito+brain_region+Sex	2.889	1321.000
ENSG00000114757	0.368	-0.137	0.050	-0.324	PEX5L	0.445	~p_tau+(1|manifest)+cngeneson+pc_mito+brain_region+Sex	0.554	2982.000
ENSG00000103479	0.007	-0.137	0.032	-0.307	RBL2	0.024	~p_tau+(1|manifest)+cngeneson+pc_mito+brain_region+Sex	1.937	1888.000
ENSG00000163590	0.136	-0.138	-0.006	-0.270	PPM1L	0.205	~p_tau+(1|manifest)+cngeneson+pc_mito+brain_region+Sex	1.783	1998.000
ENSG00000167522	0.212	-0.138	0.047	-0.323	ANKRD11	0.288	~p_tau+(1|manifest)+cngeneson+pc_mito+brain_region+Sex	1.817	1977.000
ENSG00000170430	0.168	-0.138	0.039	-0.315	MGMT	0.241	~p_tau+(1|manifest)+cngeneson+pc_mito+brain_region+Sex	0.415	3056.000
ENSG00000164749	0.393	-0.138	0.056	-0.333	HNF4G	0.470	~p_tau+(1|manifest)+cngeneson+pc_mito+brain_region+Sex	1.713	2057.000
ENSG00000164808	0.124	-0.139	0.001	-0.279	SPIDR	0.191	~p_tau+(1|manifest)+cngeneson+pc_mito+brain_region+Sex	2.473	1534.000
ENSG00000054282	0.071	-0.139	-0.023	-0.255	SDCCAG8	0.126	~p_tau+(1|manifest)+cngeneson+pc_mito+brain_region+Sex	2.719	1393.000
ENSG00000103528	0.531	-0.139	0.108	-0.387	SYT17	0.595	~p_tau+(1|manifest)+cngeneson+pc_mito+brain_region+Sex	3.107	1216.000
ENSG00000162601	0.003	-0.139	-0.033	-0.246	MYSM1	0.014	~p_tau+(1|manifest)+cngeneson+pc_mito+brain_region+Sex	2.630	1440.000
ENSG00000090989	0.009	-0.140	0.006	-0.286	EXOC1	0.028	~p_tau+(1|manifest)+cngeneson+pc_mito+brain_region+Sex	1.301	2399.000
ENSG00000091157	0.012	-0.140	-0.039	-0.241	WDR7	0.034	~p_tau+(1|manifest)+cngeneson+pc_mito+brain_region+Sex	1.544	2176.000
ENSG00000115896	0.305	-0.140	0.039	-0.319	PLCL1	0.386	~p_tau+(1|manifest)+cngeneson+pc_mito+brain_region+Sex	1.656	2094.000
ENSG00000137764	0.136	-0.140	-0.007	-0.273	MAP2K5	0.205	~p_tau+(1|manifest)+cngeneson+pc_mito+brain_region+Sex	0.607	2959.000
ENSG00000146350	0.305	-0.140	0.081	-0.361	TBC1D32	0.386	~p_tau+(1|manifest)+cngeneson+pc_mito+brain_region+Sex	0.348	3089.000
ENSG00000131016	0.688	-0.140	0.304	-0.584	AKAP12	0.733	~p_tau+(1|manifest)+cngeneson+pc_mito+brain_region+Sex	1.207	2474.000
ENSG00000073803	0.470	-0.141	0.106	-0.387	MAP3K13	0.540	~p_tau+(1|manifest)+cngeneson+pc_mito+brain_region+Sex	2.178	1716.000
ENSG00000083535	0.033	-0.141	-0.041	-0.242	PIBF1	0.071	~p_tau+(1|manifest)+cngeneson+pc_mito+brain_region+Sex	2.525	1493.000
ENSG00000067900	0.027	-0.141	-0.012	-0.270	ROCK1	0.061	~p_tau+(1|manifest)+cngeneson+pc_mito+brain_region+Sex	1.764	2005.000
ENSG00000198589	0.187	-0.142	0.007	-0.290	LRBA	0.262	~p_tau+(1|manifest)+cngeneson+pc_mito+brain_region+Sex	4.876	713.000
ENSG00000185345	0.021	-0.142	-0.043	-0.241	PRKN	0.050	~p_tau+(1|manifest)+cngeneson+pc_mito+brain_region+Sex	6.428	496.000
ENSG00000154217	0.028	-0.142	-0.036	-0.248	PITPNC1	0.061	~p_tau+(1|manifest)+cngeneson+pc_mito+brain_region+Sex	3.592	1038.000
ENSG00000188580	0.451	-0.142	0.215	-0.500	NKAIN2	0.523	~p_tau+(1|manifest)+cngeneson+pc_mito+brain_region+Sex	3.069	1234.000
ENSG00000015171	0.086	-0.143	0.015	-0.300	ZMYND11	0.144	~p_tau+(1|manifest)+cngeneson+pc_mito+brain_region+Sex	2.377	1588.000
ENSG00000198707	0.008	-0.143	0.053	-0.339	CEP290	0.026	~p_tau+(1|manifest)+cngeneson+pc_mito+brain_region+Sex	0.507	3012.000
ENSG00000124486	0.000	-0.143	-0.021	-0.265	USP9X	0.003	~p_tau+(1|manifest)+cngeneson+pc_mito+brain_region+Sex	2.026	1823.000
ENSG00000119487	0.044	-0.143	-0.001	-0.285	MAPKAP1	0.087	~p_tau+(1|manifest)+cngeneson+pc_mito+brain_region+Sex	1.637	2108.000
ENSG00000152422	0.041	-0.143	-0.041	-0.246	XRCC4	0.082	~p_tau+(1|manifest)+cngeneson+pc_mito+brain_region+Sex	1.180	2506.000
ENSG00000079156	0.572	-0.143	0.133	-0.420	OSBPL6	0.630	~p_tau+(1|manifest)+cngeneson+pc_mito+brain_region+Sex	2.300	1636.000
ENSG00000109586	0.432	-0.144	0.077	-0.364	GALNT7	0.505	~p_tau+(1|manifest)+cngeneson+pc_mito+brain_region+Sex	5.024	696.000
ENSG00000150347	0.356	-0.144	0.130	-0.417	ARID5B	0.435	~p_tau+(1|manifest)+cngeneson+pc_mito+brain_region+Sex	3.846	951.000
ENSG00000198648	0.072	-0.144	0.011	-0.299	STK39	0.128	~p_tau+(1|manifest)+cngeneson+pc_mito+brain_region+Sex	0.500	3016.000
ENSG00000198363	0.018	-0.144	-0.041	-0.247	ASPH	0.045	~p_tau+(1|manifest)+cngeneson+pc_mito+brain_region+Sex	9.209	298.000
ENSG00000052841	0.001	-0.144	-0.078	-0.211	TTC17	0.005	~p_tau+(1|manifest)+cngeneson+pc_mito+brain_region+Sex	2.978	1275.000
ENSG00000106714	0.618	-0.144	0.246	-0.535	CNTNAP3	0.671	~p_tau+(1|manifest)+cngeneson+pc_mito+brain_region+Sex	7.482	396.000
ENSG00000136643	0.218	-0.144	0.013	-0.302	RPS6KC1	0.295	~p_tau+(1|manifest)+cngeneson+pc_mito+brain_region+Sex	2.118	1759.000
ENSG00000146282	0.008	-0.144	-0.005	-0.283	RARS2	0.026	~p_tau+(1|manifest)+cngeneson+pc_mito+brain_region+Sex	0.569	2975.000
ENSG00000121957	0.440	-0.145	0.083	-0.372	GPSM2	0.513	~p_tau+(1|manifest)+cngeneson+pc_mito+brain_region+Sex	5.586	605.000
ENSG00000184588	0.060	-0.145	-0.022	-0.267	PDE4B	0.110	~p_tau+(1|manifest)+cngeneson+pc_mito+brain_region+Sex	10.445	236.000
ENSG00000147548	0.300	-0.145	0.046	-0.336	NSD3	0.381	~p_tau+(1|manifest)+cngeneson+pc_mito+brain_region+Sex	3.828	961.000
ENSG00000166024	0.000	-0.145	-0.070	-0.221	R3HCC1L	0.002	~p_tau+(1|manifest)+cngeneson+pc_mito+brain_region+Sex	1.160	2524.000
ENSG00000131848	0.174	-0.145	0.041	-0.332	ZSCAN5A	0.247	~p_tau+(1|manifest)+cngeneson+pc_mito+brain_region+Sex	1.762	2007.000
ENSG00000134900	0.001	-0.145	-0.047	-0.244	TPP2	0.005	~p_tau+(1|manifest)+cngeneson+pc_mito+brain_region+Sex	1.120	2554.000
ENSG00000116580	0.012	-0.145	0.005	-0.295	GON4L	0.034	~p_tau+(1|manifest)+cngeneson+pc_mito+brain_region+Sex	2.763	1379.000
ENSG00000138496	0.111	-0.145	0.144	-0.435	PARP9	0.176	~p_tau+(1|manifest)+cngeneson+pc_mito+brain_region+Sex	4.590	772.000
ENSG00000082212	0.005	-0.146	-0.051	-0.241	ME2	0.018	~p_tau+(1|manifest)+cngeneson+pc_mito+brain_region+Sex	0.295	3106.000
ENSG00000049167	0.002	-0.146	-0.024	-0.268	ERCC8	0.011	~p_tau+(1|manifest)+cngeneson+pc_mito+brain_region+Sex	1.430	2278.000
ENSG00000182771	0.110	-0.147	-0.012	-0.281	GRID1	0.174	~p_tau+(1|manifest)+cngeneson+pc_mito+brain_region+Sex	3.205	1173.000
ENSG00000044459	0.525	-0.147	0.130	-0.423	CNTLN	0.590	~p_tau+(1|manifest)+cngeneson+pc_mito+brain_region+Sex	1.964	1871.000
ENSG00000198780	0.042	-0.148	-0.003	-0.292	FAM169A	0.084	~p_tau+(1|manifest)+cngeneson+pc_mito+brain_region+Sex	2.126	1751.000
ENSG00000073712	0.093	-0.148	-0.014	-0.281	FERMT2	0.153	~p_tau+(1|manifest)+cngeneson+pc_mito+brain_region+Sex	3.867	940.000
ENSG00000153707	0.451	-0.148	0.128	-0.424	PTPRD	0.523	~p_tau+(1|manifest)+cngeneson+pc_mito+brain_region+Sex	6.080	540.000
ENSG00000116729	0.214	-0.148	0.017	-0.312	WLS	0.290	~p_tau+(1|manifest)+cngeneson+pc_mito+brain_region+Sex	5.998	546.000
ENSG00000145996	0.021	-0.148	-0.047	-0.249	CDKAL1	0.050	~p_tau+(1|manifest)+cngeneson+pc_mito+brain_region+Sex	1.360	2347.000
ENSG00000171100	0.206	-0.148	0.013	-0.310	MTM1	0.282	~p_tau+(1|manifest)+cngeneson+pc_mito+brain_region+Sex	1.655	2095.000
ENSG00000138078	0.050	-0.148	0.013	-0.310	PREPL	0.096	~p_tau+(1|manifest)+cngeneson+pc_mito+brain_region+Sex	2.397	1576.000
ENSG00000157741	0.007	-0.148	-0.035	-0.262	UBN2	0.023	~p_tau+(1|manifest)+cngeneson+pc_mito+brain_region+Sex	1.670	2086.000
ENSG00000169379	0.008	-0.148	-0.034	-0.263	ARL13B	0.025	~p_tau+(1|manifest)+cngeneson+pc_mito+brain_region+Sex	1.723	2051.000
ENSG00000107036	0.142	-0.148	-0.008	-0.289	RIC1	0.212	~p_tau+(1|manifest)+cngeneson+pc_mito+brain_region+Sex	2.771	1373.000
ENSG00000165632	0.039	-0.149	-0.033	-0.264	TAF3	0.079	~p_tau+(1|manifest)+cngeneson+pc_mito+brain_region+Sex	1.419	2287.000
ENSG00000064652	0.026	-0.149	-0.035	-0.262	SNX24	0.058	~p_tau+(1|manifest)+cngeneson+pc_mito+brain_region+Sex	1.587	2153.000
ENSG00000111300	0.020	-0.149	0.037	-0.335	NAA25	0.048	~p_tau+(1|manifest)+cngeneson+pc_mito+brain_region+Sex	3.210	1170.000
ENSG00000137478	0.218	-0.150	0.016	-0.316	FCHSD2	0.295	~p_tau+(1|manifest)+cngeneson+pc_mito+brain_region+Sex	5.045	693.000
ENSG00000132485	0.046	-0.151	0.037	-0.339	ZRANB2	0.089	~p_tau+(1|manifest)+cngeneson+pc_mito+brain_region+Sex	5.628	598.000
ENSG00000135164	0.037	-0.151	0.023	-0.325	DMTF1	0.076	~p_tau+(1|manifest)+cngeneson+pc_mito+brain_region+Sex	0.984	2665.000
ENSG00000133858	0.006	-0.152	-0.033	-0.271	ZFC3H1	0.022	~p_tau+(1|manifest)+cngeneson+pc_mito+brain_region+Sex	1.449	2263.000
ENSG00000085982	0.070	-0.152	0.016	-0.320	USP40	0.124	~p_tau+(1|manifest)+cngeneson+pc_mito+brain_region+Sex	2.372	1591.000
ENSG00000151276	0.646	-0.152	0.187	-0.491	MAGI1	0.695	~p_tau+(1|manifest)+cngeneson+pc_mito+brain_region+Sex	19.549	56.000
ENSG00000144224	0.011	-0.152	0.014	-0.319	UBXN4	0.031	~p_tau+(1|manifest)+cngeneson+pc_mito+brain_region+Sex	2.793	1364.000
ENSG00000175893	0.451	-0.152	0.088	-0.392	ZDHHC21	0.523	~p_tau+(1|manifest)+cngeneson+pc_mito+brain_region+Sex	10.557	231.000
ENSG00000089916	0.002	-0.153	-0.064	-0.241	GPATCH2L	0.009	~p_tau+(1|manifest)+cngeneson+pc_mito+brain_region+Sex	1.140	2544.000
ENSG00000183783	0.702	-0.153	0.205	-0.510	KCTD8	0.747	~p_tau+(1|manifest)+cngeneson+pc_mito+brain_region+Sex	4.146	875.000
ENSG00000171385	0.796	-0.153	0.310	-0.616	KCND3	0.825	~p_tau+(1|manifest)+cngeneson+pc_mito+brain_region+Sex	9.321	294.000
ENSG00000096968	0.020	-0.153	-0.018	-0.289	JAK2	0.047	~p_tau+(1|manifest)+cngeneson+pc_mito+brain_region+Sex	2.373	1590.000
ENSG00000124145	0.749	-0.154	0.271	-0.578	SDC4	0.785	~p_tau+(1|manifest)+cngeneson+pc_mito+brain_region+Sex	12.268	168.000
ENSG00000184863	0.016	-0.154	-0.035	-0.272	RBM33	0.042	~p_tau+(1|manifest)+cngeneson+pc_mito+brain_region+Sex	1.825	1969.000
ENSG00000134121	0.647	-0.154	0.172	-0.479	CHL1	0.697	~p_tau+(1|manifest)+cngeneson+pc_mito+brain_region+Sex	12.919	146.000
ENSG00000127603	0.052	-0.154	-0.023	-0.284	MACF1	0.098	~p_tau+(1|manifest)+cngeneson+pc_mito+brain_region+Sex	28.716	23.000
ENSG00000178234	0.005	-0.154	-0.039	-0.269	GALNT11	0.018	~p_tau+(1|manifest)+cngeneson+pc_mito+brain_region+Sex	1.145	2538.000
ENSG00000184226	0.006	-0.154	-0.067	-0.241	PCDH9	0.021	~p_tau+(1|manifest)+cngeneson+pc_mito+brain_region+Sex	9.276	297.000
ENSG00000103852	0.015	-0.154	-0.025	-0.283	TTC23	0.040	~p_tau+(1|manifest)+cngeneson+pc_mito+brain_region+Sex	2.046	1809.000
ENSG00000080200	0.073	-0.154	-0.011	-0.297	CRYBG3	0.129	~p_tau+(1|manifest)+cngeneson+pc_mito+brain_region+Sex	1.903	1910.000
ENSG00000147408	0.002	-0.154	-0.074	-0.235	[illegible]	0.010	~p_tau+(1|manifest)+cngeneson+pc_mito+brain_region+Sex	4.863	716.000
ENSG00000157106	0.013	-0.155	-0.012	-0.297	SMG1	0.035	~p_tau+(1|manifest)+cngeneson+pc_mito+brain_region+Sex	3.381	1104.000
ENSG00000183826	0.160	-0.155	0.005	-0.315	BTBD9	0.231	~p_tau+(1|manifest)+cngeneson+pc_mito+brain_region+Sex	2.658	1428.000
ENSG00000120594	0.710	-0.155	0.238	-0.548	PLXDC2	0.754	~p_tau+(1|manifest)+cngeneson+pc_mito+brain_region+Sex	2.003	1838.000
ENSG00000092108	0.002	-0.155	-0.053	-0.257	SCFD1	0.009	~p_tau+(1|manifest)+cngeneson+pc_mito+brain_region+Sex	2.035	1816.000
ENSG00000135821	0.471	-0.155	0.106	-0.416	GLUL	0.540	~p_tau+(1|manifest)+cngeneson+pc_mito+brain_region+Sex	5.833	562.000
ENSG00000170634	0.197	-0.156	0.012	-0.324	ACYP2	0.272	~p_tau+(1|manifest)+cngeneson+pc_mito+brain_region+Sex	1.724	2048.000
ENSG00000091656	0.303	-0.156	0.044	-0.356	ZFHX4	0.384	~p_tau+(1|manifest)+cngeneson+pc_mito+brain_region+Sex	9.167	299.000
ENSG00000134371	0.061	-0.156	0.015	-0.327	CDC73	0.113	~p_tau+(1|manifest)+cngeneson+pc_mito+brain_region+Sex	1.775	1999.000
ENSG00000175600	0.287	-0.157	0.087	-0.400	SUGCT	0.369	~p_tau+(1|manifest)+cngeneson+pc_mito+brain_region+Sex	2.661	1424.000
ENSG00000179399	0.017	-0.157	-0.046	-0.268	GPC5	0.042	~p_tau+(1|manifest)+cngeneson+pc_mito+brain_region+Sex	3.353	1115.000
ENSG00000138696	0.003	-0.157	-0.069	-0.245	BMPR1B	0.014	~p_tau+(1|manifest)+cngeneson+pc_mito+brain_region+Sex	2.399	1573.000
ENSG00000173273	0.103	-0.157	-0.014	-0.300	TNKS	0.166	~p_tau+(1|manifest)+cngeneson+pc_mito+brain_region+Sex	1.675	2083.000
ENSG00000132530	0.007	-0.157	0.124	-0.438	XAF1	0.022	~p_tau+(1|manifest)+cngeneson+pc_mito+brain_region+Sex	1.948	1881.000
ENSG00000053524	0.754	-0.157	0.251	-0.566	MCF2L2	0.790	~p_tau+(1|manifest)+cngeneson+pc_mito+brain_region+Sex	1.320	2376.000
ENSG00000113296	0.064	-0.157	0.027	-0.342	THBS4	0.116	~p_tau+(1|manifest)+cngeneson+pc_mito+brain_region+Sex	1.485	2233.000
ENSG00000126773	0.131	-0.158	0.045	-0.361	PCNX4	0.200	~p_tau+(1|manifest)+cngeneson+pc_mito+brain_region+Sex	2.485	1523.000
ENSG00000156475	0.013	-0.158	-0.056	-0.259	PPP2R2B	0.035	~p_tau+(1|manifest)+cngeneson+pc_mito+brain_region+Sex	8.755	313.000
ENSG00000118217	0.108	-0.158	-0.005	-0.311	ATF6	0.172	~p_tau+(1|manifest)+cngeneson+pc_mito+brain_region+Sex	1.498	2221.000
ENSG00000148481	0.001	-0.158	-0.063	-0.252	MINDY3	0.005	~p_tau+(1|manifest)+cngeneson+pc_mito+brain_region+Sex	0.906	2729.000
ENSG00000119927	0.465	-0.158	0.226	-0.542	GPAM	0.535	~p_tau+(1|manifest)+cngeneson+pc_mito+brain_region+Sex	5.020	697.000
ENSG00000153207	0.014	-0.158	0.048	-0.364	AHCTF1	0.037	~p_tau+(1|manifest)+cngeneson+pc_mito+brain_region+Sex	2.324	1618.000
ENSG00000058866	0.309	-0.158	0.047	-0.364	DGKG	0.389	~p_tau+(1|manifest)+cngeneson+pc_mito+brain_region+Sex	11.315	197.000
ENSG00000102531	0.250	-0.158	0.030	-0.347	FNDC3A	0.329	~p_tau+(1|manifest)+cngeneson+pc_mito+brain_region+Sex	5.543	616.000
ENSG00000115840	0.060	-0.159	-0.019	-0.299	SLC25A12	0.110	~p_tau+(1|manifest)+cngeneson+pc_mito+brain_region+Sex	2.197	1703.000
ENSG00000047634	0.312	-0.159	0.107	-0.425	SCML1	0.392	~p_tau+(1|manifest)+cngeneson+pc_mito+brain_region+Sex	6.622	474.000
ENSG00000128656	0.224	-0.159	0.025	-0.343	CHN1	0.301	~p_tau+(1|manifest)+cngeneson+pc_mito+brain_region+Sex	1.304	2395.000
ENSG00000087095	0.025	-0.159	-0.045	-0.273	NLK	0.056	~p_tau+(1|manifest)+cngeneson+pc_mito+brain_region+Sex	2.466	1539.000
ENSG00000164199	0.007	-0.160	-0.063	-0.256	ADGRV1	0.024	~p_tau+(1|manifest)+cngeneson+pc_mito+brain_region+Sex	4.615	766.000
ENSG00000129292	0.001	-0.160	-0.041	-0.278	PHF20L1	0.007	~p_tau+(1|manifest)+cngeneson+pc_mito+brain_region+Sex	1.316	2383.000
ENSG00000089177	0.035	-0.160	-0.008	-0.312	KIF16B	0.073	~p_tau+(1|manifest)+cngeneson+pc_mito+brain_region+Sex	1.757	2015.000
ENSG00000176623	0.000	-0.160	-0.067	-0.253	RMDN1	0.002	~p_tau+(1|manifest)+cngeneson+pc_mito+brain_region+Sex	0.914	2720.000
ENSG00000184719	0.066	-0.160	-0.023	-0.298	RNLS	0.119	~p_tau+(1|manifest)+cngeneson+pc_mito+brain_region+Sex	0.835	2794.000
ENSG00000078246	0.450	-0.160	0.175	-0.495	TULP3	0.523	~p_tau+(1|manifest)+cngeneson+pc_mito+brain_region+Sex	3.123	1207.000
ENSG00000014824	0.013	-0.161	0.006	-0.327	SLC30A9	0.035	~p_tau+(1|manifest)+cngeneson+pc_mito+brain_region+Sex	2.538	1484.000
ENSG00000144535	0.024	-0.161	-0.037	-0.285	DIS3L2	0.056	~p_tau+(1|manifest)+cngeneson+pc_mito+brain_region+Sex	0.854	2774.000
ENSG00000113578	0.409	-0.161	0.078	-0.400	FGF1	0.485	~p_tau+(1|manifest)+cngeneson+pc_mito+brain_region+Sex	2.212	1694.000
ENSG00000127914	0.083	-0.161	0.023	-0.345	AKAP9	0.142	~p_tau+(1|manifest)+cngeneson+pc_mito+brain_region+Sex	3.221	1166.000
ENSG00000140538	0.028	-0.162	-0.030	-0.294	NTRK3	0.063	~p_tau+(1|manifest)+cngeneson+pc_mito+brain_region+Sex	14.435	112.000
ENSG00000144566	0.006	-0.162	-0.021	-0.303	RAB5A	0.022	~p_tau+(1|manifest)+cngeneson+pc_mito+brain_region+Sex	1.992	1846.000
ENSG00000119397	0.009	-0.163	-0.014	-0.311	CNTRL	0.028	~p_tau+(1|manifest)+cngeneson+pc_mito+brain_region+Sex	0.833	2798.000
ENSG00000172954	0.022	-0.163	-0.048	-0.277	LCLAT1	0.052	~p_tau+(1|manifest)+cngeneson+pc_mito+brain_region+Sex	0.740	2874.000
ENSG00000134769	0.078	-0.163	-0.012	-0.314	DTNA	0.135	~p_tau+(1|manifest)+cngeneson+pc_mito+brain_region+Sex	32.894	15.000
ENSG00000196792	0.111	-0.163	-0.016	-0.310	STRN3	0.177	~p_tau+(1|manifest)+cngeneson+pc_mito+brain_region+Sex	2.762	1380.000
ENSG00000114107	0.017	-0.163	-0.004	-0.322	CEP70	0.042	~p_tau+(1|manifest)+cngeneson+pc_mito+brain_region+Sex	1.375	2334.000
ENSG00000147454	0.384	-0.163	0.249	-0.575	SLC25A37	0.462	~p_tau+(1|manifest)+cngeneson+pc_mito+brain_region+Sex	7.597	383.000
ENSG00000134851	0.010	-0.163	-0.020	-0.306	TMEM165	0.028	~p_tau+(1|manifest)+cngeneson+pc_mito+brain_region+Sex	2.064	1791.000
ENSG00000077097	0.002	-0.163	-0.060	-0.267	TOP2B	0.009	~p_tau+(1|manifest)+cngeneson+pc_mito+brain_region+Sex	1.481	2239.000
ENSG00000164329	0.158	-0.163	0.006	-0.333	TENT2	0.229	~p_tau+(1|manifest)+cngeneson+pc_mito+brain_region+Sex	2.248	1668.000
ENSG00000113649	0.003	-0.163	-0.042	-0.285	TCERG1	0.012	~p_tau+(1|manifest)+cngeneson+pc_mito+brain_region+Sex	1.872	1935.000
ENSG00000100505	0.022	-0.164	-0.046	-0.282	TRIM9	0.052	~p_tau+(1|manifest)+cngeneson+pc_mito+brain_region+Sex	4.739	739.000
ENSG00000112137	0.363	-0.164	0.091	-0.418	PHACTR1	0.440	~p_tau+(1|manifest)+cngeneson+pc_mito+brain_region+Sex	3.008	1260.000
ENSG00000198874	0.008	-0.164	-0.044	-0.283	TYW1	0.026	~p_tau+(1|manifest)+cngeneson+pc_mito+brain_region+Sex	1.658	2093.000
ENSG00000149485	0.509	-0.164	0.184	-0.511	FADS1	0.576	~p_tau+(1|manifest)+cngeneson+pc_mito+brain_region+Sex	5.688	593.000
ENSG00000047056	0.245	-0.164	0.030	-0.358	WDR37	0.323	~p_tau+(1|manifest)+cngeneson+pc_mito+brain_region+Sex	3.730	999.000
ENSG00000164074	0.060	-0.164	0.054	-0.383	ABHD18	0.111	~p_tau+(1|manifest)+cngeneson+pc_mito+brain_region+Sex	2.883	1323.000
ENSG00000165495	0.604	-0.164	0.156	-0.485	PKNOX2	0.659	~p_tau+(1|manifest)+cngeneson+pc_mito+brain_region+Sex	10.678	226.000
ENSG00000104112	0.263	-0.165	0.199	-0.529	SCG3	0.344	~p_tau+(1|manifest)+cngeneson+pc_mito+brain_region+Sex	2.848	1339.000
ENSG00000101746	0.230	-0.166	0.048	-0.380	NOL4	0.307	~p_tau+(1|manifest)+cngeneson+pc_mito+brain_region+Sex	2.961	1281.000
ENSG00000068078	0.753	-0.166	0.291	-0.624	FGFR3	0.788	~p_tau+(1|manifest)+cngeneson+pc_mito+brain_region+Sex	22.578	38.000
ENSG00000164597	0.166	-0.166	0.007	-0.340	COG5	0.238	~p_tau+(1|manifest)+cngeneson+pc_mito+brain_region+Sex	5.470	629.000
ENSG00000085224	0.029	-0.167	-0.049	-0.284	ATRX	0.063	~p_tau+(1|manifest)+cngeneson+pc_mito+brain_region+Sex	3.054	1239.000
ENSG00000156011	0.011	-0.167	-0.059	-0.275	PSD3	0.031	~p_tau+(1|manifest)+cngeneson+pc_mito+brain_region+Sex	9.662	275.000
ENSG00000155903	0.036	-0.167	-0.022	-0.313	RASA2	0.074	~p_tau+(1|manifest)+cngeneson+pc_mito+brain_region+Sex	2.541	1482.000
ENSG00000080608	0.044	-0.167	-0.036	-0.299	PUM3	0.087	~p_tau+(1|manifest)+cngeneson+pc_mito+brain_region+Sex	2.245	1673.000
ENSG00000147650	0.041	-0.167	0.017	-0.352	LRP12	0.082	~p_tau+(1|manifest)+cngeneson+pc_mito+brain_region+Sex	2.143	1739.000
ENSG00000181804	0.217	-0.168	0.022	-0.359	SLC9A9	0.293	~p_tau+(1|manifest)+cngeneson+pc_mito+brain_region+Sex	10.275	244.000
ENSG00000169641	0.173	-0.169	0.021	-0.359	LUZP1	0.247	~p_tau+(1|manifest)+cngeneson+pc_mito+brain_region+Sex	3.497	1058.000
ENSG00000143207	0.039	-0.169	-0.044	-0.293	COP1	0.080	~p_tau+(1|manifest)+cngeneson+pc_mito+brain_region+Sex	3.405	1098.000
ENSG00000165115	0.035	-0.169	-0.006	-0.332	KIF27	0.073	~p_tau+(1|manifest)+cngeneson+pc_mito+brain_region+Sex	2.569	1472.000
ENSG00000116350	0.055	-0.169	-0.001	-0.337	SRSF4	0.103	~p_tau+(1|manifest)+cngeneson+pc_mito+brain_region+Sex	2.692	1408.000
ENSG00000166848	0.399	-0.169	0.070	-0.408	TERF2IP	0.475	~p_tau+(1|manifest)+cngeneson+pc_mito+brain_region+Sex	2.980	1274.000
ENSG00000143420	0.658	-0.169	0.193	-0.531	ENSA	0.707	~p_tau+(1|manifest)+cngeneson+pc_mito+brain_region+Sex	5.719	585.000
ENSG00000126653	0.048	-0.169	-0.017	-0.321	NSRP1	0.093	~p_tau+(1|manifest)+cngeneson+pc_mito+brain_region+Sex	2.718	1394.000
ENSG00000159267	0.095	-0.169	-0.009	-0.329	HLCS	0.157	~p_tau+(1|manifest)+cngeneson+pc_mito+brain_region+Sex	1.736	2037.000
ENSG00000171094	0.598	-0.169	0.157	-0.495	ALK	0.653	~p_tau+(1|manifest)+cngeneson+pc_mito+brain_region+Sex	4.785	734.000
ENSG00000154447	0.512	-0.169	0.116	-0.455	SH3RF1	0.579	~p_tau+(1|manifest)+cngeneson+pc_mito+brain_region+Sex	4.069	893.000
ENSG00000158458	0.371	-0.169	0.063	-0.402	NRG2	0.449	~p_tau+(1|manifest)+cngeneson+pc_mito+brain_region+Sex	5.760	580.000
ENSG00000100330	0.088	-0.170	-0.024	-0.315	MTMR3	0.147	~p_tau+(1|manifest)+cngeneson+pc_mito+brain_region+Sex	6.241	517.000
ENSG00000115084	0.012	-0.170	-0.033	-0.308	SLC35F5	0.034	~p_tau+(1|manifest)+cngeneson+pc_mito+brain_region+Sex	1.356	2350.000
ENSG00000092199	0.396	-0.171	0.075	-0.417	HNRNPC	0.472	~p_tau+(1|manifest)+cngeneson+pc_mito+brain_region+Sex	4.211	856.000
ENSG00000169933	0.653	-0.171	0.192	-0.534	FRMPD4	0.702	~p_tau+(1|manifest)+cngeneson+pc_mito+brain_region+Sex	1.876	1933.000
ENSG00000111817	0.335	-0.171	0.051	-0.393	DSE	0.414	~p_tau+(1|manifest)+cngeneson+pc_mito+brain_region+Sex	1.303	2398.000
ENSG00000249242	0.171	-0.171	0.124	-0.467	TMEM150C	0.244	~p_tau+(1|manifest)+cngeneson+pc_mito+brain_region+Sex	0.987	2663.000
ENSG00000138768	0.062	-0.172	-0.001	-0.343	USO1	0.113	~p_tau+(1|manifest)+cngeneson+pc_mito+brain_region+Sex	2.685	1413.000
ENSG00000187231	0.204	-0.172	0.052	-0.395	SESTD1	0.279	~p_tau+(1|manifest)+cngeneson+pc_mito+brain_region+Sex	3.829	960.000
ENSG00000132549	0.040	-0.172	-0.043	-0.300	VPS13B	0.081	~p_tau+(1|manifest)+cngeneson+pc_mito+brain_region+Sex	2.174	1717.000
ENSG00000127995	0.004	-0.172	-0.055	-0.290	CASD1	0.015	~p_tau+(1|manifest)+cngeneson+pc_mito+brain_region+Sex	1.871	1936.000
ENSG00000251247	0.452	-0.173	0.091	-0.437	ZNF345	0.524	~p_tau+(1|manifest)+cngeneson+pc_mito+brain_region+Sex	6.117	533.000
ENSG00000166348	0.409	-0.173	0.109	-0.455	USP54	0.485	~p_tau+(1|manifest)+cngeneson+pc_mito+brain_region+Sex	4.552	784.000
ENSG00000185946	0.187	-0.173	0.054	-0.400	RNPC3	0.262	~p_tau+(1|manifest)+cngeneson+pc_mito+brain_region+Sex	6.825	459.000
ENSG00000164151	0.001	-0.174	-0.062	-0.285	ICE1	0.006	~p_tau+(1|manifest)+cngeneson+pc_mito+brain_region+Sex	1.116	2558.000
ENSG00000109680	0.016	-0.175	0.010	-0.359	TBC1D19	0.041	~p_tau+(1|manifest)+cngeneson+pc_mito+brain_region+Sex	1.052	2606.000
ENSG00000123200	0.001	-0.175	-0.069	-0.280	ZC3H13	0.006	~p_tau+(1|manifest)+cngeneson+pc_mito+brain_region+Sex	1.110	2560.000
ENSG00000151612	0.109	-0.176	-0.018	-0.333	ZNF827	0.174	~p_tau+(1|manifest)+cngeneson+pc_mito+brain_region+Sex	3.108	1215.000
ENSG00000108061	0.051	-0.176	-0.010	-0.342	SHOC2	0.097	~p_tau+(1|manifest)+cngeneson+pc_mito+brain_region+Sex	2.669	1422.000
ENSG00000145730	0.695	-0.176	0.248	-0.599	PAM	0.740	~p_tau+(1|manifest)+cngeneson+pc_mito+brain_region+Sex	10.815	218.000
ENSG00000072803	0.038	-0.176	-0.043	-0.309	FBXW11	0.077	~p_tau+(1|manifest)+cngeneson+pc_mito+brain_region+Sex	2.123	1754.000
ENSG00000136560	0.312	-0.176	0.062	-0.414	TANK	0.392	~p_tau+(1|manifest)+cngeneson+pc_mito+brain_region+Sex	2.903	1317.000
ENSG00000151779	0.031	-0.176	-0.049	-0.303	NBAS	0.066	~p_tau+(1|manifest)+cngeneson+pc_mito+brain_region+Sex	2.124	1752.000
ENSG00000196511	0.217	-0.176	0.022	-0.373	TPK1	0.293	~p_tau+(1|manifest)+cngeneson+pc_mito+brain_region+Sex	2.208	1696.000
ENSG00000100503	0.147	-0.176	0.016	-0.368	NIN	0.217	~p_tau+(1|manifest)+cngeneson+pc_mito+brain_region+Sex	2.914	1312.000
ENSG00000120733	0.003	-0.176	-0.060	-0.292	KDM3B	0.012	~p_tau+(1|manifest)+cngeneson+pc_mito+brain_region+Sex	1.214	2470.000
ENSG00000249853	0.451	-0.177	0.147	-0.500	HS3ST5	0.523	~p_tau+(1|manifest)+cngeneson+pc_mito+brain_region+Sex	2.526	1492.000
ENSG00000173588	0.002	-0.177	-0.058	-0.296	CEP83	0.008	~p_tau+(1|manifest)+cngeneson+pc_mito+brain_region+Sex	1.649	2099.000
ENSG00000139220	0.316	-0.177	0.102	-0.456	PPFIA2	0.396	~p_tau+(1|manifest)+cngeneson+pc_mito+brain_region+Sex	3.367	1109.000
ENSG00000138593	0.010	-0.177	-0.043	-0.311	SECISBP2L	0.030	~p_tau+(1|manifest)+cngeneson+pc_mito+brain_region+Sex	2.349	1600.000
ENSG00000137601	0.086	-0.177	0.007	-0.361	NEK1	0.144	~p_tau+(1|manifest)+cngeneson+pc_mito+brain_region+Sex	3.788	971.000
ENSG00000188352	0.210	-0.177	0.023	-0.378	FOCAD	0.287	~p_tau+(1|manifest)+cngeneson+pc_mito+brain_region+Sex	5.175	668.000
ENSG00000087470	0.183	-0.178	0.025	-0.381	DNM1L	0.257	~p_tau+(1|manifest)+cngeneson+pc_mito+brain_region+Sex	4.007	911.000
ENSG00000126883	0.001	-0.179	-0.083	-0.274	NUP214	0.007	~p_tau+(1|manifest)+cngeneson+pc_mito+brain_region+Sex	1.287	2419.000
ENSG00000163659	0.324	-0.179	0.053	-0.412	TIPARP	0.403	~p_tau+(1|manifest)+cngeneson+pc_mito+brain_region+Sex	4.002	912.000
ENSG00000156049	0.520	-0.179	0.145	-0.504	GNA14	0.587	~p_tau+(1|manifest)+cngeneson+pc_mito+brain_region+Sex	8.990	305.000
ENSG00000138190	0.008	-0.179	-0.061	-0.298	EXOC6	0.025	~p_tau+(1|manifest)+cngeneson+pc_mito+brain_region+Sex	2.127	1750.000
ENSG00000079819	0.230	-0.180	0.032	-0.391	EPB41L2	0.308	~p_tau+(1|manifest)+cngeneson+pc_mito+brain_region+Sex	3.053	1241.000
ENSG00000198961	0.043	-0.180	-0.004	-0.355	PJA2	0.086	~p_tau+(1|manifest)+cngeneson+pc_mito+brain_region+Sex	3.451	1073.000
ENSG00000155621	0.000	-0.180	-0.095	-0.264	C9orf85	0.002	~p_tau+(1|manifest)+cngeneson+pc_mito+brain_region+Sex	0.876	2759.000
ENSG00000109771	0.302	-0.180	0.096	-0.457	LRP2BP	0.383	~p_tau+(1|manifest)+cngeneson+pc_mito+brain_region+Sex	1.893	1920.000
ENSG00000169855	0.090	-0.180	-0.023	-0.338	ROBO1	0.150	~p_tau+(1|manifest)+cngeneson+pc_mito+brain_region+Sex	1.971	1866.000
ENSG00000198887	0.505	-0.180	0.150	-0.510	SMC5	0.572	~p_tau+(1|manifest)+cngeneson+pc_mito+brain_region+Sex	8.350	335.000
ENSG00000121067	0.275	-0.180	0.064	-0.425	SPOP	0.357	~p_tau+(1|manifest)+cngeneson+pc_mito+brain_region+Sex	3.574	1042.000
ENSG00000135926	0.749	-0.181	0.369	-0.730	TMBIM1	0.785	~p_tau+(1|manifest)+cngeneson+pc_mito+brain_region+Sex	7.461	400.000
ENSG00000175575	0.137	-0.181	0.142	-0.504	PAAF1	0.207	~p_tau+(1|manifest)+cngeneson+pc_mito+brain_region+Sex	4.658	756.000
ENSG00000139496	0.004	-0.181	-0.045	-0.317	NUP58	0.015	~p_tau+(1|manifest)+cngeneson+pc_mito+brain_region+Sex	1.234	2452.000
ENSG00000135655	0.006	-0.181	-0.066	-0.296	USP15	0.021	~p_tau+(1|manifest)+cngeneson+pc_mito+brain_region+Sex	2.407	1568.000
ENSG00000108599	0.489	-0.182	0.151	-0.515	AKAP10	0.556	~p_tau+(1|manifest)+cngeneson+pc_mito+brain_region+Sex	4.405	816.000
ENSG00000183166	0.164	-0.182	0.034	-0.398	CALN1	0.236	~p_tau+(1|manifest)+cngeneson+pc_mito+brain_region+Sex	14.802	102.000
ENSG00000153904	0.016	-0.182	-0.046	-0.318	DDAH1	0.042	~p_tau+(1|manifest)+cngeneson+pc_mito+brain_region+Sex	1.727	2046.000
ENSG00000151322	0.000	-0.182	-0.112	-0.253	NPAS3	0.001	~p_tau+(1|manifest)+cngeneson+pc_mito+brain_region+Sex	11.071	207.000
ENSG00000007392	0.210	-0.183	0.134	-0.500	LUC7L	0.286	~p_tau+(1|manifest)+cngeneson+pc_mito+brain_region+Sex	3.832	957.000
ENSG00000171714	0.018	-0.183	0.157	-0.522	ANO5	0.045	~p_tau+(1|manifest)+cngeneson+pc_mito+brain_region+Sex	10.802	219.000
ENSG00000079308	0.459	-0.183	0.147	-0.514	TNS1	0.530	~p_tau+(1|manifest)+cngeneson+pc_mito+brain_region+Sex	5.719	586.000
ENSG00000079102	0.149	-0.184	0.013	-0.381	RUNX1T1	0.220	~p_tau+(1|manifest)+cngeneson+pc_mito+brain_region+Sex	1.565	2161.000
ENSG00000181827	0.030	-0.184	-0.052	-0.315	RFX7	0.064	~p_tau+(1|manifest)+cngeneson+pc_mito+brain_region+Sex	2.908	1314.000
ENSG00000168710	0.132	-0.184	0.018	-0.385	AHCYL1	0.201	~p_tau+(1|manifest)+cngeneson+pc_mito+brain_region+Sex	25.011	29.000
ENSG00000135336	0.012	-0.184	-0.008	-0.361	ORC3	0.033	~p_tau+(1|manifest)+cngeneson+pc_mito+brain_region+Sex	0.578	2971.000
ENSG00000110435	0.008	-0.185	-0.038	-0.331	PDHX	0.025	~p_tau+(1|manifest)+cngeneson+pc_mito+brain_region+Sex	2.875	1326.000
ENSG00000131051	0.118	-0.185	-0.014	-0.356	RBM39	0.183	~p_tau+(1|manifest)+cngeneson+pc_mito+brain_region+Sex	4.255	840.000
ENSG00000073711	0.310	-0.185	0.047	-0.418	PPP2R3A	0.390	~p_tau+(1|manifest)+cngeneson+pc_mito+brain_region+Sex	4.084	889.000
ENSG00000123104	0.026	-0.186	-0.016	-0.355	ITPR2	0.058	~p_tau+(1|manifest)+cngeneson+pc_mito+brain_region+Sex	32.717	16.000
ENSG00000081803	0.470	-0.186	0.126	-0.498	CADPS2	0.540	~p_tau+(1|manifest)+cngeneson+pc_mito+brain_region+Sex	0.921	2711.000
ENSG00000138685	0.107	-0.186	-0.016	-0.357	FGF2	0.171	~p_tau+(1|manifest)+cngeneson+pc_mito+brain_region+Sex	4.372	826.000
ENSG00000041982	0.446	-0.186	0.397	-0.770	TNC	0.519	~p_tau+(1|manifest)+cngeneson+pc_mito+brain_region+Sex	7.546	387.000
ENSG00000196284	0.006	-0.186	-0.085	-0.288	SUPT3H	0.021	~p_tau+(1|manifest)+cngeneson+pc_mito+brain_region+Sex	1.799	1988.000
ENSG00000112242	0.103	-0.187	0.043	-0.416	E2F3	0.167	~p_tau+(1|manifest)+cngeneson+pc_mito+brain_region+Sex	3.042	1246.000
ENSG00000049323	0.606	-0.189	0.201	-0.579	LTBP1	0.660	~p_tau+(1|manifest)+cngeneson+pc_mito+brain_region+Sex	8.002	357.000
ENSG00000075539	0.024	-0.190	-0.062	-0.317	FRYL	0.054	~p_tau+(1|manifest)+cngeneson+pc_mito+brain_region+Sex	3.277	1144.000
ENSG00000149256	0.721	-0.190	0.348	-0.727	TENM4	0.763	~p_tau+(1|manifest)+cngeneson+pc_mito+brain_region+Sex	21.967	39.000
ENSG00000078304	0.010	-0.190	-0.076	-0.303	PPP2R5C	0.029	~p_tau+(1|manifest)+cngeneson+pc_mito+brain_region+Sex	1.507	2212.000
ENSG00000083097	0.012	-0.190	-0.062	-0.318	DOP1A	0.034	~p_tau+(1|manifest)+cngeneson+pc_mito+brain_region+Sex	1.900	1913.000
ENSG00000198185	0.016	-0.191	0.040	-0.422	ZNF334	0.040	~p_tau+(1|manifest)+cngeneson+pc_mito+brain_region+Sex	0.802	2831.000
ENSG00000136813	0.018	-0.191	-0.044	-0.337	ECPAS	0.044	~p_tau+(1|manifest)+cngeneson+pc_mito+brain_region+Sex	3.371	1107.000
ENSG00000111880	0.114	-0.191	-0.010	-0.371	RNGTT	0.180	~p_tau+(1|manifest)+cngeneson+pc_mito+brain_region+Sex	3.625	1026.000
ENSG00000232593	0.268	-0.191	0.044	-0.426	KANTR	0.349	~p_tau+(1|manifest)+cngeneson+pc_mito+brain_region+Sex	6.686	467.000
ENSG00000081026	0.103	-0.191	0.001	-0.382	MAGI3	0.167	~p_tau+(1|manifest)+cngeneson+pc_mito+brain_region+Sex	3.436	1078.000
ENSG00000083123	0.054	-0.191	-0.033	-0.349	BCKDHB	0.102	~p_tau+(1|manifest)+cngeneson+pc_mito+brain_region+Sex	4.709	746.000
ENSG00000181744	0.073	-0.191	-0.016	-0.367	DIPK2A	0.129	~p_tau+(1|manifest)+cngeneson+pc_mito+brain_region+Sex	1.926	1895.000
ENSG00000062598	0.034	-0.191	-0.025	-0.357	ELMO2	0.071	~p_tau+(1|manifest)+cngeneson+pc_mito+brain_region+Sex	2.288	1645.000
ENSG00000136267	0.703	-0.192	0.255	-0.640	DGKB	0.748	~p_tau+(1|manifest)+cngeneson+pc_mito+brain_region+Sex	3.420	1086.000
ENSG00000162664	0.008	-0.193	-0.051	-0.334	ZNF326	0.024	~p_tau+(1|manifest)+cngeneson+pc_mito+brain_region+Sex	2.070	1789.000
ENSG00000197323	0.010	-0.193	-0.054	-0.331	TRIM33	0.028	~p_tau+(1|manifest)+cngeneson+pc_mito+brain_region+Sex	2.230	1683.000
ENSG00000158966	0.007	-0.193	-0.074	-0.311	CACHD1	0.023	~p_tau+(1|manifest)+cngeneson+pc_mito+brain_region+Sex	4.914	709.000
ENSG00000189195	0.122	-0.193	0.171	-0.557	BTBD8	0.188	~p_tau+(1|manifest)+cngeneson+pc_mito+brain_region+Sex	1.761	2009.000
ENSG00000166147	0.183	-0.193	0.117	-0.503	FBN1	0.257	~p_tau+(1|manifest)+cngeneson+pc_mito+brain_region+Sex	2.931	1302.000
ENSG00000153113	0.376	-0.193	0.096	-0.482	CAST	0.453	~p_tau+(1|manifest)+cngeneson+pc_mito+brain_region+Sex	4.642	760.000
ENSG00000143952	0.008	-0.193	-0.069	-0.318	VPS54	0.026	~p_tau+(1|manifest)+cngeneson+pc_mito+brain_region+Sex	2.224	1688.000
ENSG00000143786	0.487	-0.194	0.122	-0.510	CNIH3	0.555	~p_tau+(1|manifest)+cngeneson+pc_mito+brain_region+Sex	4.275	836.000
ENSG00000163430	0.215	-0.194	0.021	-0.408	FSTL1	0.292	~p_tau+(1|manifest)+cngeneson+pc_mito+brain_region+Sex	3.574	1041.000
ENSG00000135541	0.174	-0.194	0.010	-0.398	AHI1	0.247	~p_tau+(1|manifest)+cngeneson+pc_mito+brain_region+Sex	7.671	376.000
ENSG00000158195	0.089	-0.194	-0.025	-0.363	WASF2	0.148	~p_tau+(1|manifest)+cngeneson+pc_mito+brain_region+Sex	3.496	1059.000
ENSG00000173208	0.460	-0.195	0.200	-0.590	ABCD2	0.531	~p_tau+(1|manifest)+cngeneson+pc_mito+brain_region+Sex	7.873	364.000
ENSG00000163328	0.157	-0.195	0.088	-0.477	GPR155	0.229	~p_tau+(1|manifest)+cngeneson+pc_mito+brain_region+Sex	4.867	715.000
ENSG00000163947	0.930	-0.195	0.862	-1.252	ARHGEF3	0.942	~p_tau+(1|manifest)+cngeneson+pc_mito+brain_region+Sex	32.093	19.000
ENSG00000144909	0.564	-0.195	0.170	-0.560	OSBPL11	0.623	~p_tau+(1|manifest)+cngeneson+pc_mito+brain_region+Sex	6.961	448.000
ENSG00000141510	0.264	-0.195	0.041	-0.431	TP53	0.346	~p_tau+(1|manifest)+cngeneson+pc_mito+brain_region+Sex	2.768	1376.000
ENSG00000153936	0.155	-0.195	-0.004	-0.387	HS2ST1	0.226	~p_tau+(1|manifest)+cngeneson+pc_mito+brain_region+Sex	3.480	1062.000
ENSG00000152404	0.000	-0.195	-0.090	-0.301	CWF19L2	0.001	~p_tau+(1|manifest)+cngeneson+pc_mito+brain_region+Sex	0.801	2832.000
ENSG00000005700	0.126	-0.196	0.027	-0.418	IBTK	0.194	~p_tau+(1|manifest)+cngeneson+pc_mito+brain_region+Sex	4.854	718.000
ENSG00000198162	0.002	-0.196	-0.093	-0.298	MAN1A2	0.011	~p_tau+(1|manifest)+cngeneson+pc_mito+brain_region+Sex	2.274	1656.000
ENSG00000144749	0.073	-0.196	-0.025	-0.367	LRIG1	0.128	~p_tau+(1|manifest)+cngeneson+pc_mito+brain_region+Sex	20.846	46.000
ENSG00000078043	0.000	-0.196	-0.106	-0.285	PIAS2	0.001	~p_tau+(1|manifest)+cngeneson+pc_mito+brain_region+Sex	1.879	1932.000
ENSG00000172270	0.469	-0.196	0.157	-0.549	BSG	0.539	~p_tau+(1|manifest)+cngeneson+pc_mito+brain_region+Sex	4.680	754.000
ENSG00000089123	0.021	-0.196	-0.035	-0.357	TASP1	0.050	~p_tau+(1|manifest)+cngeneson+pc_mito+brain_region+Sex	1.452	2259.000
ENSG00000119684	0.009	-0.196	-0.056	-0.336	MLH3	0.028	~p_tau+(1|manifest)+cngeneson+pc_mito+brain_region+Sex	1.800	1987.000
ENSG00000118454	0.014	-0.197	-0.047	-0.347	ANKRD13C	0.038	~p_tau+(1|manifest)+cngeneson+pc_mito+brain_region+Sex	2.308	1624.000
ENSG00000157087	0.458	-0.197	0.111	-0.505	ATP2B2	0.530	~p_tau+(1|manifest)+cngeneson+pc_mito+brain_region+Sex	7.353	407.000
ENSG00000169604	0.504	-0.197	0.148	-0.542	ANTXR1	0.571	~p_tau+(1|manifest)+cngeneson+pc_mito+brain_region+Sex	5.659	594.000
ENSG00000196663	0.127	-0.198	0.017	-0.413	TECPR2	0.195	~p_tau+(1|manifest)+cngeneson+pc_mito+brain_region+Sex	2.245	1671.000
ENSG00000161040	0.205	-0.198	0.018	-0.414	FBXL13	0.280	~p_tau+(1|manifest)+cngeneson+pc_mito+brain_region+Sex	3.407	1097.000
ENSG00000116406	0.094	-0.198	-0.021	-0.376	EDEM3	0.155	~p_tau+(1|manifest)+cngeneson+pc_mito+brain_region+Sex	2.968	1279.000
ENSG00000197360	0.884	-0.199	0.674	-1.072	ZNF98	0.901	~p_tau+(1|manifest)+cngeneson+pc_mito+brain_region+Sex	15.076	98.000
ENSG00000048540	0.585	-0.199	0.226	-0.624	LMO3	0.642	~p_tau+(1|manifest)+cngeneson+pc_mito+brain_region+Sex	15.167	94.000
ENSG00000121390	0.147	-0.200	-0.004	-0.395	PSPC1	0.217	~p_tau+(1|manifest)+cngeneson+pc_mito+brain_region+Sex	2.231	1682.000
ENSG00000128563	0.047	-0.200	0.004	-0.403	PRKRIP1	0.091	~p_tau+(1|manifest)+cngeneson+pc_mito+brain_region+Sex	2.480	1527.000
ENSG00000146409	0.374	-0.200	0.108	-0.508	SLC18B1	0.451	~p_tau+(1|manifest)+cngeneson+pc_mito+brain_region+Sex	5.525	619.000
ENSG00000118564	0.025	-0.200	-0.047	-0.353	FBXL5	0.057	~p_tau+(1|manifest)+cngeneson+pc_mito+brain_region+Sex	3.689	1005.000
ENSG00000175198	0.017	-0.201	-0.069	-0.332	PCCA	0.043	~p_tau+(1|manifest)+cngeneson+pc_mito+brain_region+Sex	3.610	1033.000
ENSG00000089280	0.148	-0.201	0.052	-0.454	FUS	0.218	~p_tau+(1|manifest)+cngeneson+pc_mito+brain_region+Sex	3.083	1224.000
ENSG00000182197	0.049	-0.201	-0.049	-0.353	EXT1	0.095	~p_tau+(1|manifest)+cngeneson+pc_mito+brain_region+Sex	1.317	2382.000
ENSG00000111728	0.690	-0.201	0.281	-0.683	ST8SIA1	0.736	~p_tau+(1|manifest)+cngeneson+pc_mito+brain_region+Sex	1.475	2243.000
ENSG00000169122	0.003	-0.201	-0.088	-0.315	FAM110B	0.014	~p_tau+(1|manifest)+cngeneson+pc_mito+brain_region+Sex	0.721	2895.000
ENSG00000163617	0.012	-0.203	0.011	-0.416	CCDC191	0.035	~p_tau+(1|manifest)+cngeneson+pc_mito+brain_region+Sex	1.598	2146.000
ENSG00000196376	0.301	-0.203	0.068	-0.474	SLC35F1	0.382	~p_tau+(1|manifest)+cngeneson+pc_mito+brain_region+Sex	7.054	435.000
ENSG00000146938	0.360	-0.204	0.074	-0.481	NLGN4X	0.437	~p_tau+(1|manifest)+cngeneson+pc_mito+brain_region+Sex	2.032	1821.000
ENSG00000117625	0.145	-0.204	-0.007	-0.400	RCOR3	0.215	~p_tau+(1|manifest)+cngeneson+pc_mito+brain_region+Sex	3.283	1141.000
ENSG00000101639	0.027	-0.204	-0.048	-0.360	CEP192	0.060	~p_tau+(1|manifest)+cngeneson+pc_mito+brain_region+Sex	2.428	1559.000
ENSG00000116489	0.027	-0.204	-0.063	-0.345	CAPZA1	0.060	~p_tau+(1|manifest)+cngeneson+pc_mito+brain_region+Sex	2.335	1608.000
ENSG00000137821	0.027	-0.204	-0.057	-0.352	LRRC49	0.060	~p_tau+(1|manifest)+cngeneson+pc_mito+brain_region+Sex	1.238	2449.000
ENSG00000138670	0.529	-0.205	0.179	-0.588	RASGEF1B	0.593	~p_tau+(1|manifest)+cngeneson+pc_mito+brain_region+Sex	67.371	3.000
ENSG00000162669	0.008	-0.205	-0.034	-0.375	HFM1	0.026	~p_tau+(1|manifest)+cngeneson+pc_mito+brain_region+Sex	0.868	2767.000
ENSG00000136758	0.006	-0.205	-0.021	-0.389	YME1L1	0.021	~p_tau+(1|manifest)+cngeneson+pc_mito+brain_region+Sex	2.951	1288.000
ENSG00000143995	0.074	-0.206	-0.038	-0.374	MEIS1	0.130	~p_tau+(1|manifest)+cngeneson+pc_mito+brain_region+Sex	2.764	1378.000
ENSG00000132294	0.143	-0.207	-0.007	-0.407	EFR3A	0.213	~p_tau+(1|manifest)+cngeneson+pc_mito+brain_region+Sex	2.606	1452.000
ENSG00000182667	0.001	-0.207	-0.098	-0.316	NTM	0.004	~p_tau+(1|manifest)+cngeneson+pc_mito+brain_region+Sex	10.051	257.000
ENSG00000163288	0.004	-0.207	-0.092	-0.323	GABRB1	0.015	~p_tau+(1|manifest)+cngeneson+pc_mito+brain_region+Sex	3.769	982.000
ENSG00000122484	0.037	-0.208	-0.050	-0.365	RPAP2	0.076	~p_tau+(1|manifest)+cngeneson+pc_mito+brain_region+Sex	2.625	1443.000
ENSG00000198963	0.147	-0.208	0.029	-0.445	RORB	0.218	~p_tau+(1|manifest)+cngeneson+pc_mito+brain_region+Sex	4.038	902.000
ENSG00000134444	0.014	-0.208	-0.066	-0.350	RELCH	0.037	~p_tau+(1|manifest)+cngeneson+pc_mito+brain_region+Sex	1.069	2595.000
ENSG00000147642	0.225	-0.208	0.028	-0.445	SYBU	0.302	~p_tau+(1|manifest)+cngeneson+pc_mito+brain_region+Sex	2.225	1686.000
ENSG00000151914	0.000	-0.209	-0.043	-0.375	DST	0.001	~p_tau+(1|manifest)+cngeneson+pc_mito+brain_region+Sex	29.709	21.000
ENSG00000104419	0.627	-0.209	0.217	-0.636	NDRG1	0.679	~p_tau+(1|manifest)+cngeneson+pc_mito+brain_region+Sex	2.236	1681.000
ENSG00000162613	0.001	-0.209	-0.090	-0.329	FUBP1	0.007	~p_tau+(1|manifest)+cngeneson+pc_mito+brain_region+Sex	1.539	2182.000
ENSG00000151892	0.222	-0.210	0.023	-0.442	GFRA1	0.300	~p_tau+(1|manifest)+cngeneson+pc_mito+brain_region+Sex	3.190	1182.000
ENSG00000149346	0.075	-0.210	0.044	-0.464	SLX4IP	0.131	~p_tau+(1|manifest)+cngeneson+pc_mito+brain_region+Sex	1.080	2583.000
ENSG00000050030	0.085	-0.210	-0.033	-0.387	NEXMIF	0.144	~p_tau+(1|manifest)+cngeneson+pc_mito+brain_region+Sex	1.439	2271.000
ENSG00000066933	0.146	-0.210	0.003	-0.424	MYO9A	0.216	~p_tau+(1|manifest)+cngeneson+pc_mito+brain_region+Sex	9.521	284.000
ENSG00000139173	0.146	-0.212	-0.006	-0.418	TMEM117	0.216	~p_tau+(1|manifest)+cngeneson+pc_mito+brain_region+Sex	2.592	1460.000
ENSG00000187957	0.560	-0.212	0.179	-0.603	DNER	0.620	~p_tau+(1|manifest)+cngeneson+pc_mito+brain_region+Sex	10.614	229.000
ENSG00000124496	0.448	-0.213	0.136	-0.561	TRERF1	0.521	~p_tau+(1|manifest)+cngeneson+pc_mito+brain_region+Sex	7.265	414.000
ENSG00000112379	0.030	-0.213	-0.043	-0.384	ARFGEF3	0.065	~p_tau+(1|manifest)+cngeneson+pc_mito+brain_region+Sex	1.255	2438.000
ENSG00000186448	0.002	-0.214	-0.114	-0.314	ZNF197	0.008	~p_tau+(1|manifest)+cngeneson+pc_mito+brain_region+Sex	1.496	2224.000
ENSG00000151414	0.087	-0.214	-0.033	-0.395	NEK7	0.146	~p_tau+(1|manifest)+cngeneson+pc_mito+brain_region+Sex	1.377	2331.000
ENSG00000185033	0.070	-0.214	-0.006	-0.423	SEMA4B	0.124	~p_tau+(1|manifest)+cngeneson+pc_mito+brain_region+Sex	4.097	885.000
ENSG00000182054	0.207	-0.215	0.181	-0.610	IDH2	0.282	~p_tau+(1|manifest)+cngeneson+pc_mito+brain_region+Sex	6.955	449.000
ENSG00000119408	0.184	-0.215	0.069	-0.499	NEK6	0.258	~p_tau+(1|manifest)+cngeneson+pc_mito+brain_region+Sex	2.696	1406.000
ENSG00000170624	0.274	-0.215	0.050	-0.480	SGCD	0.355	~p_tau+(1|manifest)+cngeneson+pc_mito+brain_region+Sex	4.827	722.000
ENSG00000172915	0.202	-0.215	0.016	-0.446	NBEA	0.278	~p_tau+(1|manifest)+cngeneson+pc_mito+brain_region+Sex	0.798	2835.000
ENSG00000104313	0.570	-0.215	0.187	-0.617	EYA1	0.629	~p_tau+(1|manifest)+cngeneson+pc_mito+brain_region+Sex	5.882	559.000
ENSG00000107643	0.100	-0.215	-0.013	-0.418	MAPK8	0.163	~p_tau+(1|manifest)+cngeneson+pc_mito+brain_region+Sex	3.981	916.000
ENSG00000100629	0.057	-0.216	-0.047	-0.386	CEP128	0.107	~p_tau+(1|manifest)+cngeneson+pc_mito+brain_region+Sex	1.314	2386.000
ENSG00000185261	0.182	-0.216	0.017	-0.450	KIAA0825	0.257	~p_tau+(1|manifest)+cngeneson+pc_mito+brain_region+Sex	1.384	2328.000
ENSG00000196358	0.218	-0.217	0.159	-0.593	NTNG2	0.294	~p_tau+(1|manifest)+cngeneson+pc_mito+brain_region+Sex	5.524	620.000
ENSG00000135063	0.097	-0.217	-0.021	-0.414	FAM189A2	0.159	~p_tau+(1|manifest)+cngeneson+pc_mito+brain_region+Sex	7.538	390.000
ENSG00000168172	0.001	-0.217	-0.095	-0.340	HOOK3	0.004	~p_tau+(1|manifest)+cngeneson+pc_mito+brain_region+Sex	2.334	1610.000
ENSG00000103657	0.007	-0.218	-0.085	-0.351	HERC1	0.024	~p_tau+(1|manifest)+cngeneson+pc_mito+brain_region+Sex	3.432	1082.000
ENSG00000168077	0.589	-0.219	0.207	-0.644	SCARA3	0.645	~p_tau+(1|manifest)+cngeneson+pc_mito+brain_region+Sex	10.036	258.000
ENSG00000133226	0.193	-0.219	0.115	-0.553	SRRM1	0.268	~p_tau+(1|manifest)+cngeneson+pc_mito+brain_region+Sex	4.073	892.000
ENSG00000198815	0.154	-0.219	-0.002	-0.436	FOXJ3	0.225	~p_tau+(1|manifest)+cngeneson+pc_mito+brain_region+Sex	6.485	491.000
ENSG00000151687	0.090	-0.220	-0.012	-0.428	ANKAR	0.150	~p_tau+(1|manifest)+cngeneson+pc_mito+brain_region+Sex	0.729	2889.000
ENSG00000074657	0.094	-0.220	-0.027	-0.413	ZNF532	0.156	~p_tau+(1|manifest)+cngeneson+pc_mito+brain_region+Sex	1.882	1930.000
ENSG00000126870	0.424	-0.220	0.118	-0.559	WDR60	0.498	~p_tau+(1|manifest)+cngeneson+pc_mito+brain_region+Sex	5.405	635.000
ENSG00000143156	0.005	-0.221	-0.097	-0.344	NME7	0.020	~p_tau+(1|manifest)+cngeneson+pc_mito+brain_region+Sex	0.870	2763.000
ENSG00000125124	0.205	-0.222	0.022	-0.465	BBS2	0.280	~p_tau+(1|manifest)+cngeneson+pc_mito+brain_region+Sex	4.120	882.000
ENSG00000014123	0.010	-0.222	-0.029	-0.415	UFL1	0.030	~p_tau+(1|manifest)+cngeneson+pc_mito+brain_region+Sex	1.925	1897.000
ENSG00000183762	0.387	-0.222	0.092	-0.537	KREMEN1	0.464	~p_tau+(1|manifest)+cngeneson+pc_mito+brain_region+Sex	3.118	1209.000
ENSG00000038219	0.057	-0.222	0.010	-0.455	BOD1L1	0.107	~p_tau+(1|manifest)+cngeneson+pc_mito+brain_region+Sex	0.761	2860.000
ENSG00000163138	0.000	-0.223	-0.127	-0.318	PACRGL	0.001	~p_tau+(1|manifest)+cngeneson+pc_mito+brain_region+Sex	1.338	2366.000
ENSG00000110436	0.023	-0.223	-0.076	-0.370	SLC1A2	0.053	~p_tau+(1|manifest)+cngeneson+pc_mito+brain_region+Sex	5.377	639.000
ENSG00000118961	0.027	-0.223	-0.039	-0.408	LDAH	0.060	~p_tau+(1|manifest)+cngeneson+pc_mito+brain_region+Sex	0.821	2810.000
ENSG00000165487	0.049	-0.224	-0.057	-0.391	MICU2	0.095	~p_tau+(1|manifest)+cngeneson+pc_mito+brain_region+Sex	2.705	1403.000
ENSG00000134874	0.033	-0.224	-0.022	-0.427	DZIP1	0.070	~p_tau+(1|manifest)+cngeneson+pc_mito+brain_region+Sex	2.139	1745.000
ENSG00000151466	0.105	-0.224	-0.021	-0.428	SCLT1	0.169	~p_tau+(1|manifest)+cngeneson+pc_mito+brain_region+Sex	2.365	1596.000
ENSG00000184220	0.108	-0.225	-0.023	-0.427	CMSS1	0.172	~p_tau+(1|manifest)+cngeneson+pc_mito+brain_region+Sex	3.040	1247.000
ENSG00000154274	0.510	-0.225	0.150	-0.600	C4orf19	0.577	~p_tau+(1|manifest)+cngeneson+pc_mito+brain_region+Sex	11.279	198.000
ENSG00000174776	0.669	-0.226	0.297	-0.748	WDR49	0.715	~p_tau+(1|manifest)+cngeneson+pc_mito+brain_region+Sex	1.862	1943.000
ENSG00000181826	0.187	-0.226	0.007	-0.459	RELL1	0.262	~p_tau+(1|manifest)+cngeneson+pc_mito+brain_region+Sex	10.468	234.000
ENSG00000171408	0.133	-0.227	0.015	-0.468	PDE7B	0.201	~p_tau+(1|manifest)+cngeneson+pc_mito+brain_region+Sex	8.093	351.000
ENSG00000163596	0.076	-0.227	-0.021	-0.433	ICA1L	0.133	~p_tau+(1|manifest)+cngeneson+pc_mito+brain_region+Sex	3.077	1229.000
ENSG00000138780	0.014	-0.227	-0.087	-0.367	GSTCD	0.038	~p_tau+(1|manifest)+cngeneson+pc_mito+brain_region+Sex	1.994	1844.000
ENSG00000102452	0.397	-0.227	0.122	-0.577	NALCN	0.474	~p_tau+(1|manifest)+cngeneson+pc_mito+brain_region+Sex	0.954	2689.000
ENSG00000115355	0.019	-0.228	-0.073	-0.383	CCDC88A	0.046	~p_tau+(1|manifest)+cngeneson+pc_mito+brain_region+Sex	6.392	500.000
ENSG00000091138	0.579	-0.228	0.243	-0.699	SLC26A3	0.637	~p_tau+(1|manifest)+cngeneson+pc_mito+brain_region+Sex	71.087	2.000
ENSG00000138336	0.063	-0.228	-0.010	-0.446	TET1	0.114	~p_tau+(1|manifest)+cngeneson+pc_mito+brain_region+Sex	2.970	1277.000
ENSG00000143033	0.027	-0.228	-0.025	-0.432	MTF2	0.061	~p_tau+(1|manifest)+cngeneson+pc_mito+brain_region+Sex	2.554	1479.000
ENSG00000184156	0.778	-0.228	0.420	-0.876	KCNQ3	0.810	~p_tau+(1|manifest)+cngeneson+pc_mito+brain_region+Sex	7.035	440.000
ENSG00000108587	0.008	-0.229	-0.076	-0.382	GOSR1	0.026	~p_tau+(1|manifest)+cngeneson+pc_mito+brain_region+Sex	2.635	1438.000
ENSG00000100815	0.001	-0.229	-0.125	-0.333	TRIP11	0.005	~p_tau+(1|manifest)+cngeneson+pc_mito+brain_region+Sex	1.022	2628.000
ENSG00000197157	0.156	-0.229	0.000	-0.458	SND1	0.227	~p_tau+(1|manifest)+cngeneson+pc_mito+brain_region+Sex	4.549	786.000
ENSG00000092969	0.250	-0.229	0.045	-0.504	TGFB2	0.328	~p_tau+(1|manifest)+cngeneson+pc_mito+brain_region+Sex	7.469	398.000
ENSG00000169410	0.568	-0.230	0.193	-0.653	PTPN9	0.628	~p_tau+(1|manifest)+cngeneson+pc_mito+brain_region+Sex	14.419	113.000
ENSG00000154493	0.116	-0.230	0.004	-0.463	C10orf90	0.182	~p_tau+(1|manifest)+cngeneson+pc_mito+brain_region+Sex	2.912	1313.000
ENSG00000154743	0.001	-0.230	-0.122	-0.338	TSEN2	0.004	~p_tau+(1|manifest)+cngeneson+pc_mito+brain_region+Sex	0.827	2805.000
ENSG00000196782	0.075	-0.230	-0.033	-0.427	MAML3	0.131	~p_tau+(1|manifest)+cngeneson+pc_mito+brain_region+Sex	1.724	2050.000
ENSG00000142856	0.142	-0.231	0.010	-0.471	ITGB3BP	0.212	~p_tau+(1|manifest)+cngeneson+pc_mito+brain_region+Sex	3.665	1014.000
ENSG00000169764	0.112	-0.232	-0.021	-0.443	UGP2	0.177	~p_tau+(1|manifest)+cngeneson+pc_mito+brain_region+Sex	1.951	1878.000
ENSG00000116984	0.022	-0.233	-0.060	-0.407	MTR	0.051	~p_tau+(1|manifest)+cngeneson+pc_mito+brain_region+Sex	2.474	1531.000
ENSG00000169062	0.521	-0.235	0.165	-0.634	UPF3A	0.588	~p_tau+(1|manifest)+cngeneson+pc_mito+brain_region+Sex	10.844	217.000
ENSG00000114698	0.106	-0.236	-0.016	-0.455	PLSCR4	0.170	~p_tau+(1|manifest)+cngeneson+pc_mito+brain_region+Sex	5.380	638.000
ENSG00000081377	0.137	-0.236	-0.008	-0.464	CDC14B	0.207	~p_tau+(1|manifest)+cngeneson+pc_mito+brain_region+Sex	2.818	1352.000
ENSG00000166435	0.179	-0.236	0.081	-0.553	XRRA1	0.253	~p_tau+(1|manifest)+cngeneson+pc_mito+brain_region+Sex	3.765	987.000
ENSG00000133958	0.432	-0.236	0.114	-0.587	UNC79	0.505	~p_tau+(1|manifest)+cngeneson+pc_mito+brain_region+Sex	5.360	641.000
ENSG00000196482	0.635	-0.237	0.285	-0.760	ESRRG	0.686	~p_tau+(1|manifest)+cngeneson+pc_mito+brain_region+Sex	15.706	92.000
ENSG00000134716	0.088	-0.237	-0.027	-0.448	CYP2J2	0.148	~p_tau+(1|manifest)+cngeneson+pc_mito+brain_region+Sex	0.935	2702.000
ENSG00000100731	0.003	-0.239	-0.110	-0.368	PCNX1	0.014	~p_tau+(1|manifest)+cngeneson+pc_mito+brain_region+Sex	3.306	1135.000
ENSG00000135976	0.071	-0.239	-0.017	-0.461	ANKRD36	0.126	~p_tau+(1|manifest)+cngeneson+pc_mito+brain_region+Sex	1.621	2126.000
ENSG00000166436	0.189	-0.239	0.102	-0.581	TRIM66	0.264	~p_tau+(1|manifest)+cngeneson+pc_mito+brain_region+Sex	7.178	423.000

ENSG00000162402	0.028	-0.240	-0.076	-0.403	USP24	0.061	~p_tau+(1|manifest)+cngeneson+pc_mito+brain_region+Sex	3.613	1032.000
ENSG00000185046	0.007	-0.241	-0.070	-0.411	ANKS1B	0.023	~p_tau+(1|manifest)+cngeneson+pc_mito+brain_region+Sex	0.844	2785.000
ENSG00000169756	0.309	-0.241	0.066	-0.548	LIMS1	0.389	~p_tau+(1|manifest)+cngeneson+pc_mito+brain_region+Sex	10.894	215.000
ENSG00000160712	0.047	-0.241	-0.048	-0.434	IL6R	0.091	~p_tau+(1|manifest)+cngeneson+pc_mito+brain_region+Sex	2.285	1649.000
ENSG00000135241	0.337	-0.241	0.100	-0.582	PNPLA8	0.415	~p_tau+(1|manifest)+cngeneson+pc_mito+brain_region+Sex	10.121	255.000
ENSG00000166068	0.175	-0.242	0.007	-0.490	SPRED1	0.249	~p_tau+(1|manifest)+cngeneson+pc_mito+brain_region+Sex	3.455	1071.000
ENSG00000166839	0.662	-0.242	0.301	-0.785	ANKDD1A	0.710	~p_tau+(1|manifest)+cngeneson+pc_mito+brain_region+Sex	21.029	45.000
ENSG00000143398	0.118	-0.243	-0.002	-0.484	PIP5K1A	0.183	~p_tau+(1|manifest)+cngeneson+pc_mito+brain_region+Sex	3.924	926.000
ENSG00000172264	0.481	-0.243	0.187	-0.674	MACROD2	0.549	~p_tau+(1|manifest)+cngeneson+pc_mito+brain_region+Sex	6.400	498.000
ENSG00000128000	0.031	-0.243	-0.017	-0.470	ZNF780B	0.067	~p_tau+(1|manifest)+cngeneson+pc_mito+brain_region+Sex	1.813	1979.000
ENSG00000176809	0.336	-0.244	0.102	-0.589	LRRC37A3	0.414	~p_tau+(1|manifest)+cngeneson+pc_mito+brain_region+Sex	12.803	148.000
ENSG00000153815	0.189	-0.244	0.015	-0.503	CMIP	0.264	~p_tau+(1|manifest)+cngeneson+pc_mito+brain_region+Sex	7.729	373.000
ENSG00000113318	0.083	-0.244	-0.038	-0.450	MSH3	0.141	~p_tau+(1|manifest)+cngeneson+pc_mito+brain_region+Sex	3.008	1259.000
ENSG00000197563	0.015	-0.246	-0.087	-0.404	PIGN	0.040	~p_tau+(1|manifest)+cngeneson+pc_mito+brain_region+Sex	1.506	2215.000
ENSG00000227124	0.115	-0.246	-0.023	-0.469	ZNF717	0.180	~p_tau+(1|manifest)+cngeneson+pc_mito+brain_region+Sex	3.451	1074.000
ENSG00000115109	0.020	-0.247	-0.089	-0.404	EPB41L5	0.048	~p_tau+(1|manifest)+cngeneson+pc_mito+brain_region+Sex	3.284	1140.000
ENSG00000112234	0.006	-0.247	-0.086	-0.408	FBXL4	0.021	~p_tau+(1|manifest)+cngeneson+pc_mito+brain_region+Sex	1.499	2218.000
ENSG00000117616	0.266	-0.248	0.055	-0.551	RSRP1	0.347	~p_tau+(1|manifest)+cngeneson+pc_mito+brain_region+Sex	11.096	205.000
ENSG00000100697	0.020	-0.250	-0.086	-0.414	DICER1	0.048	~p_tau+(1|manifest)+cngeneson+pc_mito+brain_region+Sex	2.659	1427.000
ENSG00000140374	0.068	-0.251	-0.031	-0.472	ETFA	0.122	~p_tau+(1|manifest)+cngeneson+pc_mito+brain_region+Sex	2.269	1658.000
ENSG00000160271	0.490	-0.253	0.190	-0.695	RALGDS	0.557	~p_tau+(1|manifest)+cngeneson+pc_mito+brain_region+Sex	9.070	303.000
ENSG00000111879	0.030	-0.253	-0.072	-0.434	FAM184A	0.065	~p_tau+(1|manifest)+cngeneson+pc_mito+brain_region+Sex	2.080	1781.000
ENSG00000145725	0.002	-0.253	-0.110	-0.396	PPIP5K2	0.010	~p_tau+(1|manifest)+cngeneson+pc_mito+brain_region+Sex	2.238	1678.000
ENSG00000086544	0.455	-0.253	0.139	-0.645	ITPKC	0.526	~p_tau+(1|manifest)+cngeneson+pc_mito+brain_region+Sex	9.646	276.000
ENSG00000139597	0.140	-0.254	0.030	-0.538	N4BP2L1	0.209	~p_tau+(1|manifest)+cngeneson+pc_mito+brain_region+Sex	4.061	894.000
ENSG00000131873	0.025	-0.254	-0.081	-0.427	CHSY1	0.057	~p_tau+(1|manifest)+cngeneson+pc_mito+brain_region+Sex	0.958	2679.000
ENSG00000151474	0.609	-0.254	0.273	-0.782	FRMD4A	0.662	~p_tau+(1|manifest)+cngeneson+pc_mito+brain_region+Sex	6.943	450.000
ENSG00000104177	0.015	-0.255	-0.032	-0.477	MYEF2	0.039	~p_tau+(1|manifest)+cngeneson+pc_mito+brain_region+Sex	3.227	1163.000
ENSG00000122557	0.027	-0.255	-0.080	-0.430	HERPUD2	0.060	~p_tau+(1|manifest)+cngeneson+pc_mito+brain_region+Sex	3.335	1123.000
ENSG00000166012	0.063	-0.255	0.006	-0.517	TAF1D	0.114	~p_tau+(1|manifest)+cngeneson+pc_mito+brain_region+Sex	5.277	653.000
ENSG00000110422	0.348	-0.256	0.085	-0.597	HIPK3	0.426	~p_tau+(1|manifest)+cngeneson+pc_mito+brain_region+Sex	7.306	409.000
ENSG00000106415	0.262	-0.256	0.062	-0.573	GLCCI1	0.343	~p_tau+(1|manifest)+cngeneson+pc_mito+brain_region+Sex	5.642	596.000
ENSG00000148655	0.667	-0.256	0.316	-0.828	LRMDA	0.714	~p_tau+(1|manifest)+cngeneson+pc_mito+brain_region+Sex	12.706	151.000
ENSG00000115935	0.012	-0.257	-0.101	-0.413	WIPF1	0.034	~p_tau+(1|manifest)+cngeneson+pc_mito+brain_region+Sex	2.048	1806.000
ENSG00000175455	0.158	-0.257	0.020	-0.535	CCDC14	0.229	~p_tau+(1|manifest)+cngeneson+pc_mito+brain_region+Sex	2.474	1532.000
ENSG00000099256	0.152	-0.258	-0.007	-0.509	PRTFDC1	0.223	~p_tau+(1|manifest)+cngeneson+pc_mito+brain_region+Sex	2.294	1639.000
ENSG00000143951	0.012	-0.258	-0.092	-0.424	WDPCP	0.033	~p_tau+(1|manifest)+cngeneson+pc_mito+brain_region+Sex	1.453	2256.000
ENSG00000006062	0.552	-0.258	0.223	-0.740	MAP3K14	0.612	~p_tau+(1|manifest)+cngeneson+pc_mito+brain_region+Sex	9.964	264.000
ENSG00000117069	0.013	-0.259	-0.021	-0.496	[illegible]	0.036	~p_tau+(1|manifest)+cngeneson+pc_mito+brain_region+Sex	2.332	1611.000
ENSG00000182050	0.032	-0.260	-0.056	-0.464	MGAT4C	0.067	~p_tau+(1|manifest)+cngeneson+pc_mito+brain_region+Sex	5.149	675.000
ENSG00000163714	0.001	-0.260	-0.132	-0.388	U2SURP	0.004	~p_tau+(1|manifest)+cngeneson+pc_mito+brain_region+Sex	2.429	1558.000
ENSG00000158270	0.492	-0.260	0.214	-0.735	COLEC12	0.559	~p_tau+(1|manifest)+cngeneson+pc_mito+brain_region+Sex	11.888	178.000
ENSG00000140937	0.020	-0.261	-0.087	-0.435	CDH11	0.049	~p_tau+(1|manifest)+cngeneson+pc_mito+brain_region+Sex	1.986	1850.000
ENSG00000117523	0.094	-0.261	-0.034	-0.489	PRRC2C	0.156	~p_tau+(1|manifest)+cngeneson+pc_mito+brain_region+Sex	3.288	1138.000
ENSG00000180822	0.094	-0.262	-0.035	-0.488	PSMG4	0.155	~p_tau+(1|manifest)+cngeneson+pc_mito+brain_region+Sex	3.793	969.000
ENSG00000188158	0.170	-0.262	0.005	-0.529	NHS	0.243	~p_tau+(1|manifest)+cngeneson+pc_mito+brain_region+Sex	7.646	380.000
ENSG00000143036	0.527	-0.262	0.192	-0.716	SLC44A3	0.592	~p_tau+(1|manifest)+cngeneson+pc_mito+brain_region+Sex	8.317	339.000
ENSG00000136297	0.626	-0.262	0.305	-0.830	MMD2	0.679	~p_tau+(1|manifest)+cngeneson+pc_mito+brain_region+Sex	6.748	464.000
ENSG00000186952	0.223	-0.263	0.029	-0.556	TMEM232	0.301	~p_tau+(1|manifest)+cngeneson+pc_mito+brain_region+Sex	3.222	1165.000
ENSG00000157869	0.014	-0.264	-0.101	-0.427	RAB28	0.037	~p_tau+(1|manifest)+cngeneson+pc_mito+brain_region+Sex	3.336	1121.000
ENSG00000138448	0.065	-0.265	-0.047	-0.484	ITGAV	0.118	~p_tau+(1|manifest)+cngeneson+pc_mito+brain_region+Sex	8.074	352.000
ENSG00000168785	0.001	-0.265	-0.130	-0.401	TSPAN5	0.007	~p_tau+(1|manifest)+cngeneson+pc_mito+brain_region+Sex	1.975	1863.000
ENSG00000134508	0.539	-0.267	0.261	-0.795	CABLES1	0.601	~p_tau+(1|manifest)+cngeneson+pc_mito+brain_region+Sex	7.472	397.000
ENSG00000101596	0.003	-0.267	-0.124	-0.410	SMCHD1	0.014	~p_tau+(1|manifest)+cngeneson+pc_mito+brain_region+Sex	2.659	1426.000
ENSG00000128849	0.133	-0.268	-0.013	-0.524	CGNL1	0.201	~p_tau+(1|manifest)+cngeneson+pc_mito+brain_region+Sex	2.941	1294.000
ENSG00000146802	0.001	-0.268	-0.140	-0.397	TMEM168	0.005	~p_tau+(1|manifest)+cngeneson+pc_mito+brain_region+Sex	2.094	1774.000
ENSG00000100578	0.043	-0.269	-0.068	-0.469	KIAA0586	0.085	~p_tau+(1|manifest)+cngeneson+pc_mito+brain_region+Sex	5.277	654.000
ENSG00000102595	0.005	-0.270	-0.115	-0.425	UGGT2	0.018	~p_tau+(1|manifest)+cngeneson+pc_mito+brain_region+Sex	2.433	1556.000
ENSG00000088280	0.312	-0.270	0.071	-0.611	ASAP3	0.392	~p_tau+(1|manifest)+cngeneson+pc_mito+brain_region+Sex	6.969	447.000
ENSG00000139364	0.577	-0.270	0.242	-0.782	TMEM132B	0.635	~p_tau+(1|manifest)+cngeneson+pc_mito+brain_region+Sex	7.097	431.000
ENSG00000138468	0.022	-0.271	-0.083	-0.458	SENP7	0.052	~p_tau+(1|manifest)+cngeneson+pc_mito+brain_region+Sex	2.382	1587.000
ENSG00000144674	0.038	-0.271	-0.071	-0.470	GOLGA4	0.078	~p_tau+(1|manifest)+cngeneson+pc_mito+brain_region+Sex	5.289	652.000
ENSG00000186231	0.458	-0.272	0.162	-0.706	KLHL32	0.530	~p_tau+(1|manifest)+cngeneson+pc_mito+brain_region+Sex	5.516	621.000
ENSG00000159733	0.539	-0.272	0.275	-0.820	ZFYVE28	0.601	~p_tau+(1|manifest)+cngeneson+pc_mito+brain_region+Sex	16.029	85.000
ENSG00000066382	0.345	-0.274	0.147	-0.694	MPPED2	0.424	~p_tau+(1|manifest)+cngeneson+pc_mito+brain_region+Sex	6.107	535.000
ENSG00000119231	0.055	-0.274	-0.063	-0.485	SENP5	0.104	~p_tau+(1|manifest)+cngeneson+pc_mito+brain_region+Sex	3.826	962.000
ENSG00000129151	0.074	-0.274	-0.047	-0.502	BBOX1	0.130	~p_tau+(1|manifest)+cngeneson+pc_mito+brain_region+Sex	2.384	1584.000
ENSG00000181450	0.022	-0.276	-0.093	-0.459	ZNF678	0.051	~p_tau+(1|manifest)+cngeneson+pc_mito+brain_region+Sex	5.893	555.000
ENSG00000100523	0.118	-0.276	-0.027	-0.525	DDHD1	0.183	~p_tau+(1|manifest)+cngeneson+pc_mito+brain_region+Sex	5.579	610.000
ENSG00000114062	0.013	-0.277	-0.102	-0.452	UBE3A	0.035	~p_tau+(1|manifest)+cngeneson+pc_mito+brain_region+Sex	4.011	908.000
ENSG00000150867	0.286	-0.277	0.086	-0.641	PIP4K2A	0.368	~p_tau+(1|manifest)+cngeneson+pc_mito+brain_region+Sex	6.519	485.000
ENSG00000146151	0.049	-0.278	0.044	-0.599	HMGCLL1	0.093	~p_tau+(1|manifest)+cngeneson+pc_mito+brain_region+Sex	2.867	1332.000
ENSG00000135968	0.007	-0.278	-0.103	-0.453	GCC2	0.024	~p_tau+(1|manifest)+cngeneson+pc_mito+brain_region+Sex	1.605	2141.000
ENSG00000005108	0.000	-0.279	-0.165	-0.393	THSD7A	0.000	~p_tau+(1|manifest)+cngeneson+pc_mito+brain_region+Sex	2.753	1383.000
ENSG00000112182	0.239	-0.279	0.064	-0.622	BACH2	0.317	~p_tau+(1|manifest)+cngeneson+pc_mito+brain_region+Sex	2.659	1425.000
ENSG00000186472	0.288	-0.280	0.172	-0.731	PCLO	0.369	~p_tau+(1|manifest)+cngeneson+pc_mito+brain_region+Sex	5.014	698.000
ENSG00000166323	0.016	-0.280	-0.101	-0.460	C11orf65	0.041	~p_tau+(1|manifest)+cngeneson+pc_mito+brain_region+Sex	1.034	2618.000
ENSG00000036549	0.003	-0.281	-0.139	-0.422	AC118549.1	0.013	~p_tau+(1|manifest)+cngeneson+pc_mito+brain_region+Sex	2.293	1641.000
ENSG00000197603	0.009	-0.281	-0.119	-0.442	CPLANE1	0.027	~p_tau+(1|manifest)+cngeneson+pc_mito+brain_region+Sex	3.163	1192.000
ENSG00000140470	0.241	-0.281	0.047	-0.608	ADAMTS17	0.320	~p_tau+(1|manifest)+cngeneson+pc_mito+brain_region+Sex	4.680	753.000
ENSG00000187240	0.131	-0.281	-0.015	-0.547	DYNC2H1	0.199	~p_tau+(1|manifest)+cngeneson+pc_mito+brain_region+Sex	1.300	2404.000
ENSG00000108474	0.086	-0.282	-0.041	-0.523	PIGL	0.144	~p_tau+(1|manifest)+cngeneson+pc_mito+brain_region+Sex	2.783	1369.000
ENSG00000150938	0.109	-0.283	-0.022	-0.543	CRIM1	0.173	~p_tau+(1|manifest)+cngeneson+pc_mito+brain_region+Sex	5.698	590.000
ENSG00000023287	0.003	-0.283	-0.137	-0.429	RB1CC1	0.013	~p_tau+(1|manifest)+cngeneson+pc_mito+brain_region+Sex	1.574	2157.000
ENSG00000219626	0.145	-0.283	-0.011	-0.555	FAM228B	0.215	~p_tau+(1|manifest)+cngeneson+pc_mito+brain_region+Sex	2.688	1412.000
ENSG00000146416	0.061	-0.283	-0.055	-0.512	AIG1	0.112	~p_tau+(1|manifest)+cngeneson+pc_mito+brain_region+Sex	1.122	2553.000
ENSG00000165323	0.084	-0.283	0.033	-0.599	FAT3	0.142	~p_tau+(1|manifest)+cngeneson+pc_mito+brain_region+Sex	1.028	2621.000
ENSG00000059588	0.060	-0.284	0.029	-0.598	TARBP1	0.110	~p_tau+(1|manifest)+cngeneson+pc_mito+brain_region+Sex	3.315	1130.000
ENSG00000144406	0.201	-0.285	0.133	-0.704	UNC80	0.277	~p_tau+(1|manifest)+cngeneson+pc_mito+brain_region+Sex	1.429	2280.000
ENSG00000104093	0.008	-0.285	-0.117	-0.454	DMXL2	0.025	~p_tau+(1|manifest)+cngeneson+pc_mito+brain_region+Sex	1.903	1909.000
ENSG00000205213	0.012	-0.285	-0.093	-0.478	LGR4	0.033	~p_tau+(1|manifest)+cngeneson+pc_mito+brain_region+Sex	6.216	520.000
ENSG00000073614	0.000	-0.286	-0.166	-0.406	KDM5A	0.003	~p_tau+(1|manifest)+cngeneson+pc_mito+brain_region+Sex	1.559	2165.000
ENSG00000132837	0.193	-0.286	0.020	-0.593	DMGDH	0.268	~p_tau+(1|manifest)+cngeneson+pc_mito+brain_region+Sex	3.417	1091.000
ENSG00000010803	0.045	-0.286	-0.052	-0.521	SCMH1	0.089	~p_tau+(1|manifest)+cngeneson+pc_mito+brain_region+Sex	7.766	370.000
ENSG00000177706	0.604	-0.287	0.275	-0.848	FAM20C	0.659	~p_tau+(1|manifest)+cngeneson+pc_mito+brain_region+Sex	12.522	161.000
ENSG00000100941	0.168	-0.287	0.050	-0.624	PNN	0.241	~p_tau+(1|manifest)+cngeneson+pc_mito+brain_region+Sex	5.135	677.000
ENSG00000167470	0.195	-0.288	0.022	-0.598	MIDN	0.271	~p_tau+(1|manifest)+cngeneson+pc_mito+brain_region+Sex	5.014	699.000
ENSG00000119707	0.051	-0.288	-0.067	-0.510	RBM25	0.097	~p_tau+(1|manifest)+cngeneson+pc_mito+brain_region+Sex	3.964	919.000
ENSG00000107560	0.014	-0.289	-0.105	-0.472	RAB11FIP2	0.038	~p_tau+(1|manifest)+cngeneson+pc_mito+brain_region+Sex	2.576	1469.000
ENSG00000197818	0.025	-0.290	-0.054	-0.525	SLC9A8	0.056	~p_tau+(1|manifest)+cngeneson+pc_mito+brain_region+Sex	3.618	1030.000
ENSG00000111058	0.061	-0.290	-0.046	-0.533	ACSS3	0.112	~p_tau+(1|manifest)+cngeneson+pc_mito+brain_region+Sex	9.967	262.000
ENSG00000150672	0.001	-0.290	-0.146	-0.433	DLG2	0.004	~p_tau+(1|manifest)+cngeneson+pc_mito+brain_region+Sex	6.487	490.000
ENSG00000107290	0.028	-0.291	-0.092	-0.489	SETX	0.063	~p_tau+(1|manifest)+cngeneson+pc_mito+brain_region+Sex	3.665	1015.000
ENSG00000179104	0.006	-0.291	-0.116	-0.466	TMTC2	0.021	~p_tau+(1|manifest)+cngeneson+pc_mito+brain_region+Sex	9.124	301.000
ENSG00000119787	0.136	-0.291	-0.013	-0.570	ATL2	0.205	~p_tau+(1|manifest)+cngeneson+pc_mito+brain_region+Sex	4.436	808.000
ENSG00000148090	0.128	-0.293	-0.016	-0.571	AUH	0.196	~p_tau+(1|manifest)+cngeneson+pc_mito+brain_region+Sex	8.388	334.000
ENSG00000188559	0.040	-0.294	-0.062	-0.525	RALGAPA2	0.080	~p_tau+(1|manifest)+cngeneson+pc_mito+brain_region+Sex	11.810	181.000
ENSG00000119906	0.006	-0.295	-0.132	-0.458	SLF2	0.022	~p_tau+(1|manifest)+cngeneson+pc_mito+brain_region+Sex	2.330	1614.000
ENSG00000169918	0.066	-0.295	-0.035	-0.556	OTUD7A	0.119	~p_tau+(1|manifest)+cngeneson+pc_mito+brain_region+Sex	9.639	278.000
ENSG00000188177	0.039	-0.296	-0.083	-0.509	ZC3H6	0.079	~p_tau+(1|manifest)+cngeneson+pc_mito+brain_region+Sex	2.422	1564.000
ENSG00000146729	0.273	-0.296	0.058	-0.651	NIPSNAP2	0.354	~p_tau+(1|manifest)+cngeneson+pc_mito+brain_region+Sex	7.247	417.000
ENSG00000025796	0.002	-0.296	-0.149	-0.444	SEC63	0.008	~p_tau+(1|manifest)+cngeneson+pc_mito+brain_region+Sex	2.343	1603.000
ENSG00000198948	0.115	-0.297	-0.008	-0.585	MFAP3L	0.181	~p_tau+(1|manifest)+cngeneson+pc_mito+brain_region+Sex	4.954	707.000
ENSG00000174891	0.005	-0.297	-0.135	-0.460	RSRC1	0.019	~p_tau+(1|manifest)+cngeneson+pc_mito+brain_region+Sex	1.406	2302.000
ENSG00000182150	0.005	-0.298	-0.131	-0.465	ERCC6L2	0.018	~p_tau+(1|manifest)+cngeneson+pc_mito+brain_region+Sex	3.921	927.000
ENSG00000069020	0.000	-0.298	-0.188	-0.408	MAST4	0.000	~p_tau+(1|manifest)+cngeneson+pc_mito+brain_region+Sex	3.164	1191.000
ENSG00000140022	0.002	-0.299	-0.133	-0.465	STON2	0.011	~p_tau+(1|manifest)+cngeneson+pc_mito+brain_region+Sex	13.809	126.000
ENSG00000139083	0.424	-0.299	0.183	-0.782	ETV6	0.498	~p_tau+(1|manifest)+cngeneson+pc_mito+brain_region+Sex	16.410	81.000
ENSG00000112249	0.001	-0.299	-0.148	-0.451	ASCC3	0.007	~p_tau+(1|manifest)+cngeneson+pc_mito+brain_region+Sex	2.425	1560.000
ENSG00000118482	0.043	-0.300	-0.076	-0.523	PHF3	0.086	~p_tau+(1|manifest)+cngeneson+pc_mito+brain_region+Sex	4.010	909.000
ENSG00000151834	0.000	-0.300	-0.159	-0.441	GABRA2	0.003	~p_tau+(1|manifest)+cngeneson+pc_mito+brain_region+Sex	5.154	674.000
ENSG00000114857	0.005	-0.301	-0.125	-0.477	NKTR	0.018	~p_tau+(1|manifest)+cngeneson+pc_mito+brain_region+Sex	6.137	530.000
ENSG00000104375	0.038	-0.301	-0.054	-0.547	STK3	0.078	~p_tau+(1|manifest)+cngeneson+pc_mito+brain_region+Sex	11.331	196.000
ENSG00000115159	0.051	-0.302	-0.070	-0.533	GPD2	0.097	~p_tau+(1|manifest)+cngeneson+pc_mito+brain_region+Sex	5.914	553.000
ENSG00000139117	0.428	-0.302	0.271	-0.874	CPNE8	0.502	~p_tau+(1|manifest)+cngeneson+pc_mito+brain_region+Sex	15.746	91.000
ENSG00000147324	0.045	-0.303	-0.055	-0.552	MFHAS1	0.088	~p_tau+(1|manifest)+cngeneson+pc_mito+brain_region+Sex	8.168	349.000
ENSG00000197857	0.014	-0.304	-0.119	-0.489	ZNF44	0.037	~p_tau+(1|manifest)+cngeneson+pc_mito+brain_region+Sex	2.162	1723.000
ENSG00000156076	0.551	-0.304	0.308	-0.917	WIF1	0.612	~p_tau+(1|manifest)+cngeneson+pc_mito+brain_region+Sex	9.494	287.000
ENSG00000165914	0.013	-0.305	-0.114	-0.495	TTC7B	0.036	~p_tau+(1|manifest)+cngeneson+pc_mito+brain_region+Sex	2.823	1349.000
ENSG00000066468	0.024	-0.305	-0.067	-0.543	FGFR2	0.055	~p_tau+(1|manifest)+cngeneson+pc_mito+brain_region+Sex	6.335	507.000
ENSG00000141469	0.367	-0.305	0.142	-0.752	SLC14A1	0.445	~p_tau+(1|manifest)+cngeneson+pc_mito+brain_region+Sex	10.161	251.000
ENSG00000069431	0.382	-0.306	0.138	-0.749	ABCC9	0.460	~p_tau+(1|manifest)+cngeneson+pc_mito+brain_region+Sex	12.739	150.000
ENSG00000080493	0.000	-0.306	-0.157	-0.455	SLC4A4	0.003	~p_tau+(1|manifest)+cngeneson+pc_mito+brain_region+Sex	9.558	281.000
ENSG00000174485	0.076	-0.307	-0.032	-0.581	DENND4A	0.132	~p_tau+(1|manifest)+cngeneson+pc_mito+brain_region+Sex	11.487	192.000
ENSG00000107551	0.313	-0.307	0.079	-0.692	RASSF4	0.393	~p_tau+(1|manifest)+cngeneson+pc_mito+brain_region+Sex	7.897	361.000
ENSG00000152377	0.317	-0.307	0.117	-0.730	SPOCK1	0.396	~p_tau+(1|manifest)+cngeneson+pc_mito+brain_region+Sex	12.546	160.000
ENSG00000156110	0.005	-0.307	-0.124	-0.491	ADK	0.020	~p_tau+(1|manifest)+cngeneson+pc_mito+brain_region+Sex	4.594	770.000
ENSG00000156042	0.285	-0.308	0.067	-0.682	CFAP70	0.366	~p_tau+(1|manifest)+cngeneson+pc_mito+brain_region+Sex	5.801	569.000
ENSG00000155970	0.000	-0.308	-0.186	-0.431	MICU3	0.003	~p_tau+(1|manifest)+cngeneson+pc_mito+brain_region+Sex	2.435	1554.000
ENSG00000135108	0.008	-0.310	-0.125	-0.495	FBXO21	0.026	~p_tau+(1|manifest)+cngeneson+pc_mito+brain_region+Sex	1.888	1924.000
ENSG00000116560	0.000	-0.311	-0.180	-0.441	SFPQ	0.002	~p_tau+(1|manifest)+cngeneson+pc_mito+brain_region+Sex	4.096	886.000
ENSG00000182621	0.015	-0.311	-0.019	-0.602	PLCB1	0.040	~p_tau+(1|manifest)+cngeneson+pc_mito+brain_region+Sex	4.804	729.000
ENSG00000060138	0.471	-0.311	0.179	-0.801	YBX3	0.540	~p_tau+(1|manifest)+cngeneson+pc_mito+brain_region+Sex	10.260	245.000
ENSG00000205336	0.431	-0.313	0.172	-0.798	ADGRG1	0.504	~p_tau+(1|manifest)+cngeneson+pc_mito+brain_region+Sex	8.466	325.000
ENSG00000168575	0.161	-0.314	0.007	-0.634	SLC20A2	0.233	~p_tau+(1|manifest)+cngeneson+pc_mito+brain_region+Sex	6.169	525.000
ENSG00000104613	0.194	-0.314	0.017	-0.645	INTS10	0.269	~p_tau+(1|manifest)+cngeneson+pc_mito+brain_region+Sex	7.869	365.000
ENSG00000121988	0.035	-0.315	-0.087	-0.544	ZRANB3	0.072	~p_tau+(1|manifest)+cngeneson+pc_mito+brain_region+Sex	2.619	1446.000
ENSG00000169519	0.000	-0.317	-0.170	-0.465	METTL15	0.003	~p_tau+(1|manifest)+cngeneson+pc_mito+brain_region+Sex	3.428	1083.000
ENSG00000129636	0.000	-0.317	-0.189	-0.446	ITFG1	0.000	~p_tau+(1|manifest)+cngeneson+pc_mito+brain_region+Sex	3.069	1235.000
ENSG00000176771	0.345	-0.318	0.133	-0.769	NCKAP5	0.424	~p_tau+(1|manifest)+cngeneson+pc_mito+brain_region+Sex	18.548	61.000
ENSG00000184500	0.142	-0.318	0.002	-0.638	PROS1	0.211	~p_tau+(1|manifest)+cngeneson+pc_mito+brain_region+Sex	10.577	230.000
ENSG00000064692	0.010	-0.318	-0.121	-0.515	SNCAIP	0.029	~p_tau+(1|manifest)+cngeneson+pc_mito+brain_region+Sex	2.138	1746.000
ENSG00000145431	0.006	-0.319	-0.120	-0.517	PDGFC	0.022	~p_tau+(1|manifest)+cngeneson+pc_mito+brain_region+Sex	3.373	1105.000
ENSG00000182541	0.391	-0.319	0.180	-0.818	LIMK2	0.468	~p_tau+(1|manifest)+cngeneson+pc_mito+brain_region+Sex	8.099	350.000
ENSG00000166669	0.010	-0.320	-0.131	-0.509	ATF7IP2	0.029	~p_tau+(1|manifest)+cngeneson+pc_mito+brain_region+Sex	1.436	2274.000
ENSG00000163125	0.001	-0.321	-0.171	-0.470	RPRD2	0.005	~p_tau+(1|manifest)+cngeneson+pc_mito+brain_region+Sex	2.863	1335.000
ENSG00000061987	0.000	-0.321	-0.181	-0.460	MON2	0.003	~p_tau+(1|manifest)+cngeneson+pc_mito+brain_region+Sex	4.789	733.000
ENSG00000127989	0.104	-0.321	0.030	-0.672	MTERF1	0.167	~p_tau+(1|manifest)+cngeneson+pc_mito+brain_region+Sex	5.319	647.000
ENSG00000151575	0.146	-0.321	-0.010	-0.632	TEX9	0.216	~p_tau+(1|manifest)+cngeneson+pc_mito+brain_region+Sex	1.171	2514.000
ENSG00000040731	0.278	-0.322	0.075	-0.720	CDH10	0.360	~p_tau+(1|manifest)+cngeneson+pc_mito+brain_region+Sex	5.510	622.000
ENSG00000198520	0.562	-0.324	0.261	-0.909	ARMH1	0.622	~p_tau+(1|manifest)+cngeneson+pc_mito+brain_region+Sex	9.505	285.000
ENSG00000176049	0.000	-0.324	-0.164	-0.483	JAKMIP2	0.003	~p_tau+(1|manifest)+cngeneson+pc_mito+brain_region+Sex	1.288	2417.000
ENSG00000236383	0.427	-0.324	0.162	-0.810	CCDC200	0.501	~p_tau+(1|manifest)+cngeneson+pc_mito+brain_region+Sex	14.272	115.000
ENSG00000187715	0.021	-0.324	-0.108	-0.540	KBTBD12	0.049	~p_tau+(1|manifest)+cngeneson+pc_mito+brain_region+Sex	3.226	1164.000
ENSG00000149090	0.052	-0.324	-0.045	-0.603	PAMR1	0.099	~p_tau+(1|manifest)+cngeneson+pc_mito+brain_region+Sex	11.570	190.000
ENSG00000029363	0.020	-0.324	-0.117	-0.531	BCLAF1	0.047	~p_tau+(1|manifest)+cngeneson+pc_mito+brain_region+Sex	3.603	1035.000
ENSG00000138738	0.001	-0.326	-0.180	-0.473	PRDM5	0.005	~p_tau+(1|manifest)+cngeneson+pc_mito+brain_region+Sex	1.606	2140.000
ENSG00000165730	0.007	-0.327	-0.130	-0.524	STOX1	0.022	~p_tau+(1|manifest)+cngeneson+pc_mito+brain_region+Sex	0.909	2726.000
ENSG00000165102	0.081	-0.327	0.016	-0.670	HGSNAT	0.139	~p_tau+(1|manifest)+cngeneson+pc_mito+brain_region+Sex	5.587	603.000
ENSG00000205464	0.216	-0.327	0.039	-0.693	ATP6AP1L	0.292	~p_tau+(1|manifest)+cngeneson+pc_mito+brain_region+Sex	4.976	704.000
ENSG00000122870	0.031	-0.332	-0.075	-0.590	BICC1	0.067	~p_tau+(1|manifest)+cngeneson+pc_mito+brain_region+Sex	6.677	468.000
ENSG00000163728	0.003	-0.333	-0.157	-0.509	TTC14	0.012	~p_tau+(1|manifest)+cngeneson+pc_mito+brain_region+Sex	4.514	793.000
ENSG00000121671	0.105	-0.334	-0.027	-0.642	CRY2	0.169	~p_tau+(1|manifest)+cngeneson+pc_mito+brain_region+Sex	4.038	901.000
ENSG00000179915	0.043	-0.335	-0.062	-0.608	NRXN1	0.085	~p_tau+(1|manifest)+cngeneson+pc_mito+brain_region+Sex	12.763	149.000
ENSG00000104635	0.511	-0.336	0.255	-0.927	SLC39A14	0.578	~p_tau+(1|manifest)+cngeneson+pc_mito+brain_region+Sex	10.407	239.000
ENSG00000079215	0.000	-0.337	-0.186	-0.488	SLC1A3	0.002	~p_tau+(1|manifest)+cngeneson+pc_mito+brain_region+Sex	17.971	69.000
ENSG00000104381	0.222	-0.337	0.034	-0.708	GDAP1	0.300	~p_tau+(1|manifest)+cngeneson+pc_mito+brain_region+Sex	2.718	1396.000
ENSG00000095564	0.003	-0.338	-0.165	-0.511	BTAF1	0.012	~p_tau+(1|manifest)+cngeneson+pc_mito+brain_region+Sex	2.891	1320.000
ENSG00000168916	0.085	-0.338	-0.019	-0.658	ZNF608	0.144	~p_tau+(1|manifest)+cngeneson+pc_mito+brain_region+Sex	6.832	458.000
ENSG00000004468	0.051	-0.339	-0.041	-0.637	CD38	0.097	~p_tau+(1|manifest)+cngeneson+pc_mito+brain_region+Sex	4.575	778.000
ENSG00000166262	0.089	-0.340	-0.049	-0.630	FAM227B	0.148	~p_tau+(1|manifest)+cngeneson+pc_mito+brain_region+Sex	3.245	1157.000
ENSG00000134824	0.321	-0.340	0.107	-0.787	FADS2	0.400	~p_tau+(1|manifest)+cngeneson+pc_mito+brain_region+Sex	11.333	195.000
ENSG00000215421	0.001	-0.340	-0.174	-0.507	ZNF407	0.004	~p_tau+(1|manifest)+cngeneson+pc_mito+brain_region+Sex	3.248	1154.000
ENSG00000030419	0.018	-0.341	-0.116	-0.566	IKZF2	0.045	~p_tau+(1|manifest)+cngeneson+pc_mito+brain_region+Sex	4.450	804.000
ENSG00000058091	0.000	-0.342	-0.184	-0.500	CDK14	0.003	~p_tau+(1|manifest)+cngeneson+pc_mito+brain_region+Sex	3.072	1232.000
ENSG00000135999	0.011	-0.342	-0.130	-0.554	EPC2	0.031	~p_tau+(1|manifest)+cngeneson+pc_mito+brain_region+Sex	4.371	827.000
ENSG00000120278	0.014	-0.342	-0.121	-0.564	PLEKHG1	0.038	~p_tau+(1|manifest)+cngeneson+pc_mito+brain_region+Sex	1.184	2503.000
ENSG00000092978	0.000	-0.343	-0.208	-0.478	GPATCH2	0.002	~p_tau+(1|manifest)+cngeneson+pc_mito+brain_region+Sex	2.551	1480.000
ENSG00000174501	0.009	-0.343	-0.133	-0.553	ANKRD36C	0.028	~p_tau+(1|manifest)+cngeneson+pc_mito+brain_region+Sex	1.538	2183.000
ENSG00000173681	0.000	-0.345	-0.206	-0.483	BCLAF3	0.001	~p_tau+(1|manifest)+cngeneson+pc_mito+brain_region+Sex	1.275	2425.000
ENSG00000104427	0.202	-0.346	0.037	-0.729	ZC2HC1A	0.277	~p_tau+(1|manifest)+cngeneson+pc_mito+brain_region+Sex	1.456	2253.000
ENSG00000182141	0.140	-0.349	-0.019	-0.678	ZNF708	0.209	~p_tau+(1|manifest)+cngeneson+pc_mito+brain_region+Sex	10.155	252.000
ENSG00000069535	0.315	-0.350	0.121	-0.821	MAOB	0.395	~p_tau+(1|manifest)+cngeneson+pc_mito+brain_region+Sex	17.483	72.000
ENSG00000101745	0.032	-0.352	-0.104	-0.600	ANKRD12	0.067	~p_tau+(1|manifest)+cngeneson+pc_mito+brain_region+Sex	5.159	672.000
ENSG00000168769	0.005	-0.352	-0.163	-0.541	TET2	0.019	~p_tau+(1|manifest)+cngeneson+pc_mito+brain_region+Sex	2.517	1501.000
ENSG00000083544	0.000	-0.353	-0.180	-0.525	TDRD3	0.003	~p_tau+(1|manifest)+cngeneson+pc_mito+brain_region+Sex	6.347	506.000
ENSG00000187068	0.243	-0.354	0.063	-0.770	C3orf70	0.321	~p_tau+(1|manifest)+cngeneson+pc_mito+brain_region+Sex	9.844	268.000
ENSG00000165424	0.159	-0.354	-0.006	-0.702	ZCCHC24	0.231	~p_tau+(1|manifest)+cngeneson+pc_mito+brain_region+Sex	5.301	651.000
ENSG00000171476	0.184	-0.354	0.014	-0.722	HOPX	0.258	~p_tau+(1|manifest)+cngeneson+pc_mito+brain_region+Sex	9.341	293.000
ENSG00000198919	0.017	-0.355	-0.129	-0.581	DZIP3	0.042	~p_tau+(1|manifest)+cngeneson+pc_mito+brain_region+Sex	1.821	1972.000
ENSG00000186976	0.009	-0.355	-0.091	-0.619	EFCAB6	0.028	~p_tau+(1|manifest)+cngeneson+pc_mito+brain_region+Sex	1.591	2149.000
ENSG00000132842	0.000	-0.355	-0.190	-0.520	AP3B1	0.003	~p_tau+(1|manifest)+cngeneson+pc_mito+brain_region+Sex	2.441	1552.000
ENSG00000039319	0.006	-0.356	-0.153	-0.559	ZFYVE16	0.020	~p_tau+(1|manifest)+cngeneson+pc_mito+brain_region+Sex	5.585	607.000
ENSG00000112425	0.081	-0.357	-0.049	-0.665	EPM2A	0.139	~p_tau+(1|manifest)+cngeneson+pc_mito+brain_region+Sex	3.410	1095.000
ENSG00000152495	0.135	-0.357	-0.009	-0.706	CAMK4	0.204	~p_tau+(1|manifest)+cngeneson+pc_mito+brain_region+Sex	6.516	486.000
ENSG00000150636	0.022	-0.358	-0.050	-0.667	CCDC102B	0.052	~p_tau+(1|manifest)+cngeneson+pc_mito+brain_region+Sex	3.409	1096.000
ENSG00000143344	0.003	-0.359	-0.150	-0.568	RGL1	0.013	~p_tau+(1|manifest)+cngeneson+pc_mito+brain_region+Sex	10.211	247.000
ENSG00000181234	0.434	-0.361	0.240	-0.962	TMEM132C	0.506	~p_tau+(1|manifest)+cngeneson+pc_mito+brain_region+Sex	3.848	950.000
ENSG00000184178	0.000	-0.363	-0.194	-0.531	SCFD2	0.002	~p_tau+(1|manifest)+cngeneson+pc_mito+brain_region+Sex	4.486	798.000
ENSG00000118507	0.001	-0.364	-0.174	-0.554	AKAP7	0.008	~p_tau+(1|manifest)+cngeneson+pc_mito+brain_region+Sex	2.159	1727.000
ENSG00000146247	0.000	-0.365	-0.230	-0.499	PHIP	0.000	~p_tau+(1|manifest)+cngeneson+pc_mito+brain_region+Sex	3.685	1007.000
ENSG00000132199	0.053	-0.367	-0.071	-0.664	ENOSF1	0.100	~p_tau+(1|manifest)+cngeneson+pc_mito+brain_region+Sex	1.445	2268.000
ENSG00000173230	0.041	-0.368	-0.095	-0.641	GOLGB1	0.082	~p_tau+(1|manifest)+cngeneson+pc_mito+brain_region+Sex	3.958	920.000
ENSG00000157103	0.314	-0.369	0.115	-0.853	SLC6A1	0.394	~p_tau+(1|manifest)+cngeneson+pc_mito+brain_region+Sex	12.327	167.000
ENSG00000196776	0.103	-0.370	-0.042	-0.698	CD47	0.167	~p_tau+(1|manifest)+cngeneson+pc_mito+brain_region+Sex	5.571	611.000
ENSG00000197977	0.012	-0.371	-0.144	-0.597	ELOVL2	0.034	~p_tau+(1|manifest)+cngeneson+pc_mito+brain_region+Sex	4.253	843.000
ENSG00000132321	0.059	-0.371	-0.027	-0.716	IQCA1	0.109	~p_tau+(1|manifest)+cngeneson+pc_mito+brain_region+Sex	10.214	246.000
ENSG00000148948	0.041	-0.372	-0.060	-0.684	LRRC4C	0.082	~p_tau+(1|manifest)+cngeneson+pc_mito+brain_region+Sex	1.351	2355.000
ENSG00000080224	0.121	-0.375	0.006	-0.756	EPHA6	0.187	~p_tau+(1|manifest)+cngeneson+pc_mito+brain_region+Sex	3.657	1017.000
ENSG00000111962	0.084	-0.375	-0.057	-0.693	UST	0.142	~p_tau+(1|manifest)+cngeneson+pc_mito+brain_region+Sex	2.577	1468.000
ENSG00000136636	0.113	-0.379	-0.039	-0.718	KCTD3	0.179	~p_tau+(1|manifest)+cngeneson+pc_mito+brain_region+Sex	4.501	796.000
ENSG00000196660	0.118	-0.379	-0.032	-0.726	SLC30A10	0.183	~p_tau+(1|manifest)+cngeneson+pc_mito+brain_region+Sex	4.859	717.000
ENSG00000145781	0.000	-0.379	-0.250	-0.509	COMMD10	0.000	~p_tau+(1|manifest)+cngeneson+pc_mito+brain_region+Sex	3.258	1148.000
ENSG00000130779	0.014	-0.381	-0.119	-0.644	CLIP1	0.038	~p_tau+(1|manifest)+cngeneson+pc_mito+brain_region+Sex	10.193	248.000
ENSG00000121964	0.001	-0.382	-0.203	-0.561	GTDC1	0.004	~p_tau+(1|manifest)+cngeneson+pc_mito+brain_region+Sex	1.019	2631.000
ENSG00000167232	0.007	-0.382	-0.165	-0.600	ZNF91	0.024	~p_tau+(1|manifest)+cngeneson+pc_mito+brain_region+Sex	3.845	952.000
ENSG00000060718	0.132	-0.384	-0.007	-0.761	COL11A1	0.200	~p_tau+(1|manifest)+cngeneson+pc_mito+brain_region+Sex	3.236	1161.000
ENSG00000198105	0.068	-0.389	-0.027	-0.752	ZNF248	0.122	~p_tau+(1|manifest)+cngeneson+pc_mito+brain_region+Sex	6.309	510.000
ENSG00000149089	0.202	-0.391	0.028	-0.810	APIP	0.278	~p_tau+(1|manifest)+cngeneson+pc_mito+brain_region+Sex	11.269	200.000
ENSG00000124406	0.030	-0.392	-0.073	-0.711	ATP8A1	0.065	~p_tau+(1|manifest)+cngeneson+pc_mito+brain_region+Sex	4.870	714.000
ENSG00000134376	0.000	-0.395	-0.187	-0.603	CRB1	0.004	~p_tau+(1|manifest)+cngeneson+pc_mito+brain_region+Sex	8.181	348.000
ENSG00000164989	0.029	-0.395	-0.105	-0.686	CCDC171	0.064	~p_tau+(1|manifest)+cngeneson+pc_mito+brain_region+Sex	2.838	1341.000
ENSG00000134882	0.057	-0.398	-0.080	-0.715	UBAC2	0.107	~p_tau+(1|manifest)+cngeneson+pc_mito+brain_region+Sex	13.068	139.000
ENSG00000159140	0.013	-0.398	-0.128	-0.668	SON	0.036	~p_tau+(1|manifest)+cngeneson+pc_mito+brain_region+Sex	11.679	186.000
ENSG00000144455	0.005	-0.401	-0.108	-0.693	SUMF1	0.018	~p_tau+(1|manifest)+cngeneson+pc_mito+brain_region+Sex	4.583	775.000
ENSG00000134533	0.029	-0.402	-0.034	-0.771	RERG	0.063	~p_tau+(1|manifest)+cngeneson+pc_mito+brain_region+Sex	3.095	1219.000
ENSG00000283782	0.189	-0.403	0.039	-0.845	AC116366.3	0.264	~p_tau+(1|manifest)+cngeneson+pc_mito+brain_region+Sex	4.592	771.000
ENSG00000211448	0.093	-0.404	-0.051	-0.757	DIO2	0.154	~p_tau+(1|manifest)+cngeneson+pc_mito+brain_region+Sex	5.554	614.000
ENSG00000144642	0.014	-0.404	-0.091	-0.718	RBMS3	0.037	~p_tau+(1|manifest)+cngeneson+pc_mito+brain_region+Sex	0.420	3051.000
ENSG00000271447	0.146	-0.408	0.003	-0.819	MMP28	0.216	~p_tau+(1|manifest)+cngeneson+pc_mito+brain_region+Sex	4.720	744.000
ENSG00000186260	0.242	-0.410	0.082	-0.902	MRTFB	0.321	~p_tau+(1|manifest)+cngeneson+pc_mito+brain_region+Sex	17.195	76.000
ENSG00000204740	0.011	-0.411	-0.160	-0.662	MALRD1	0.031	~p_tau+(1|manifest)+cngeneson+pc_mito+brain_region+Sex	0.426	3049.000
ENSG00000135749	0.051	-0.411	-0.028	-0.794	PCNX2	0.098	~p_tau+(1|manifest)+cngeneson+pc_mito+brain_region+Sex	1.025	2626.000
ENSG00000100033	0.418	-0.411	0.301	-1.124	PRODH	0.493	~p_tau+(1|manifest)+cngeneson+pc_mito+brain_region+Sex	21.057	44.000
ENSG00000164929	0.072	-0.412	-0.016	-0.807	BAALC	0.127	~p_tau+(1|manifest)+cngeneson+pc_mito+brain_region+Sex	13.295	137.000
ENSG00000184903	0.000	-0.412	-0.192	-0.632	IMMP2L	0.003	~p_tau+(1|manifest)+cngeneson+pc_mito+brain_region+Sex	5.569	612.000
ENSG00000158019	0.000	-0.412	-0.218	-0.607	BABAM2	0.001	~p_tau+(1|manifest)+cngeneson+pc_mito+brain_region+Sex	3.053	1240.000
ENSG00000008513	0.014	-0.413	-0.158	-0.667	ST3GAL1	0.037	~p_tau+(1|manifest)+cngeneson+pc_mito+brain_region+Sex	3.523	1054.000
ENSG00000178425	0.007	-0.417	-0.181	-0.653	NT5DC1	0.023	~p_tau+(1|manifest)+cngeneson+pc_mito+brain_region+Sex	1.760	2011.000
ENSG00000196440	0.045	-0.418	-0.105	-0.732	ARMCX4	0.089	~p_tau+(1|manifest)+cngeneson+pc_mito+brain_region+Sex	4.314	834.000
ENSG00000182168	0.057	-0.420	0.010	-0.850	UNC5C	0.106	~p_tau+(1|manifest)+cngeneson+pc_mito+brain_region+Sex	11.662	187.000
ENSG00000172817	0.022	-0.421	-0.136	-0.706	CYP7B1	0.051	~p_tau+(1|manifest)+cngeneson+pc_mito+brain_region+Sex	2.262	1661.000
ENSG00000198822	0.205	-0.423	0.056	-0.902	GRM3	0.280	~p_tau+(1|manifest)+cngeneson+pc_mito+brain_region+Sex	3.767	986.000
ENSG00000270099	0.009	-0.423	-0.135	-0.711	AL365273.2	0.027	~p_tau+(1|manifest)+cngeneson+pc_mito+brain_region+Sex	3.449	1077.000
ENSG00000177853	0.005	-0.430	-0.180	-0.681	ZNF518A	0.019	~p_tau+(1|manifest)+cngeneson+pc_mito+brain_region+Sex	3.039	1248.000
ENSG00000169251	0.020	-0.433	-0.117	-0.748	NMD3	0.048	~p_tau+(1|manifest)+cngeneson+pc_mito+brain_region+Sex	2.867	1333.000
ENSG00000175497	0.373	-0.435	0.209	-1.078	DPP10	0.450	~p_tau+(1|manifest)+cngeneson+pc_mito+brain_region+Sex	12.135	171.000
ENSG00000172572	0.099	-0.435	0.045	-0.915	PDE3A	0.162	~p_tau+(1|manifest)+cngeneson+pc_mito+brain_region+Sex	7.096	432.000
ENSG00000153930	0.072	-0.436	-0.067	-0.805	ANKFN1	0.128	~p_tau+(1|manifest)+cngeneson+pc_mito+brain_region+Sex	2.407	1569.000
ENSG00000188313	0.204	-0.438	0.076	-0.952	PLSCR1	0.279	~p_tau+(1|manifest)+cngeneson+pc_mito+brain_region+Sex	14.108	121.000
ENSG00000118985	0.148	-0.438	0.079	-0.955	ELL2	0.219	~p_tau+(1|manifest)+cngeneson+pc_mito+brain_region+Sex	13.634	130.000
ENSG00000130413	0.004	-0.439	-0.178	-0.699	STK33	0.017	~p_tau+(1|manifest)+cngeneson+pc_mito+brain_region+Sex	4.606	768.000
ENSG00000132122	0.015	-0.441	-0.141	-0.742	SPATA6	0.039	~p_tau+(1|manifest)+cngeneson+pc_mito+brain_region+Sex	8.333	338.000
ENSG00000112893	0.021	-0.443	-0.113	-0.772	MAN2A1	0.050	~p_tau+(1|manifest)+cngeneson+pc_mito+brain_region+Sex	11.815	179.000
ENSG00000178904	0.049	-0.444	-0.106	-0.783	DPY19L3	0.094	~p_tau+(1|manifest)+cngeneson+pc_mito+brain_region+Sex	8.932	308.000
ENSG00000198797	0.173	-0.446	0.069	-0.961	BRINP2	0.247	~p_tau+(1|manifest)+cngeneson+pc_mito+brain_region+Sex	5.242	662.000
ENSG00000172340	0.002	-0.447	-0.216	-0.677	SUCLG2	0.009	~p_tau+(1|manifest)+cngeneson+pc_mito+brain_region+Sex	3.830	959.000
ENSG00000152348	0.006	-0.447	-0.187	-0.707	ATG10	0.020	~p_tau+(1|manifest)+cngeneson+pc_mito+brain_region+Sex	1.978	1859.000
ENSG00000116127	0.004	-0.447	-0.185	-0.709	ALMS1	0.015	~p_tau+(1|manifest)+cngeneson+pc_mito+brain_region+Sex	4.651	758.000
ENSG00000165475	0.097	-0.448	-0.055	-0.841	CRYL1	0.158	~p_tau+(1|manifest)+cngeneson+pc_mito+brain_region+Sex	4.256	839.000
ENSG00000157600	0.031	-0.448	-0.110	-0.787	TMEM164	0.066	~p_tau+(1|manifest)+cngeneson+pc_mito+brain_region+Sex	11.363	193.000
ENSG00000108854	0.001	-0.454	-0.249	-0.659	SMURF2	0.004	~p_tau+(1|manifest)+cngeneson+pc_mito+brain_region+Sex	6.117	534.000
ENSG00000166974	0.222	-0.456	0.102	-1.015	MAPRE2	0.300	~p_tau+(1|manifest)+cngeneson+pc_mito+brain_region+Sex	18.371	63.000
ENSG00000168952	0.018	-0.459	-0.083	-0.834	STXBP6	0.044	~p_tau+(1|manifest)+cngeneson+pc_mito+brain_region+Sex	9.999	260.000
ENSG00000172260	0.012	-0.466	-0.133	-0.800	NEGR1	0.033	~p_tau+(1|manifest)+cngeneson+pc_mito+brain_region+Sex	3.487	1061.000
ENSG00000226124	0.035	-0.467	-0.137	-0.796	FTCDNL1	0.072	~p_tau+(1|manifest)+cngeneson+pc_mito+brain_region+Sex	2.563	1477.000
ENSG00000112739	0.043	-0.470	-0.125	-0.816	PRPF4B	0.085	~p_tau+(1|manifest)+cngeneson+pc_mito+brain_region+Sex	7.107	429.000
ENSG00000138639	0.039	-0.474	-0.024	-0.923	ARHGAP24	0.080	~p_tau+(1|manifest)+cngeneson+pc_mito+brain_region+Sex	5.504	624.000
ENSG00000170153	0.016	-0.482	-0.150	-0.815	RNF150	0.042	~p_tau+(1|manifest)+cngeneson+pc_mito+brain_region+Sex	5.267	657.000
ENSG00000147509	0.012	-0.486	-0.153	-0.819	RGS20	0.033	~p_tau+(1|manifest)+cngeneson+pc_mito+brain_region+Sex	12.676	153.000
ENSG00000154529	0.159	-0.492	0.058	-1.043	CNTNAP3B	0.230	~p_tau+(1|manifest)+cngeneson+pc_mito+brain_region+Sex	17.687	71.000
ENSG00000125772	0.004	-0.493	-0.222	-0.763	GPCPD1	0.015	~p_tau+(1|manifest)+cngeneson+pc_mito+brain_region+Sex	2.674	1420.000
ENSG00000145623	0.235	-0.494	0.172	-1.161	OSMR	0.313	~p_tau+(1|manifest)+cngeneson+pc_mito+brain_region+Sex	21.865	40.000
ENSG00000144935	0.001	-0.498	-0.235	-0.760	TRPC1	0.007	~p_tau+(1|manifest)+cngeneson+pc_mito+brain_region+Sex	4.743	738.000
ENSG00000145934	0.237	-0.501	0.171	-1.173	TENM2	0.315	~p_tau+(1|manifest)+cngeneson+pc_mito+brain_region+Sex	20.215	51.000
ENSG00000115380	0.003	-0.507	-0.158	-0.855	EFEMP1	0.013	~p_tau+(1|manifest)+cngeneson+pc_mito+brain_region+Sex	1.537	2184.000
ENSG00000216937	0.003	-0.519	-0.221	-0.818	CCDC7	0.014	~p_tau+(1|manifest)+cngeneson+pc_mito+brain_region+Sex	2.969	1278.000
ENSG00000144868	0.013	-0.520	-0.143	-0.896	TMEM108	0.036	~p_tau+(1|manifest)+cngeneson+pc_mito+brain_region+Sex	4.380	825.000
ENSG00000204442	0.007	-0.522	-0.100	-0.944	FAM155A	0.023	~p_tau+(1|manifest)+cngeneson+pc_mito+brain_region+Sex	14.706	105.000
ENSG00000152217	0.000	-0.534	-0.333	-0.735	SETBP1	0.000	~p_tau+(1|manifest)+cngeneson+pc_mito+brain_region+Sex	0.756	2864.000
ENSG00000032742	0.003	-0.537	-0.254	-0.820	IFT88	0.012	~p_tau+(1|manifest)+cngeneson+pc_mito+brain_region+Sex	2.481	1525.000
ENSG00000138185	0.000	-0.541	-0.338	-0.744	ENTPD1	0.000	~p_tau+(1|manifest)+cngeneson+pc_mito+brain_region+Sex	4.165	869.000
ENSG00000197410	0.067	-0.542	-0.096	-0.988	DCHS2	0.120	~p_tau+(1|manifest)+cngeneson+pc_mito+brain_region+Sex	3.079	1227.000
ENSG00000007001	0.084	-0.547	-0.041	-1.053	UPP2	0.142	~p_tau+(1|manifest)+cngeneson+pc_mito+brain_region+Sex	6.532	482.000
ENSG00000188649	0.003	-0.555	-0.176	-0.934	CC2D2B	0.014	~p_tau+(1|manifest)+cngeneson+pc_mito+brain_region+Sex	3.418	1090.000
ENSG00000105894	0.025	-0.556	-0.149	-0.963	PTN	0.058	~p_tau+(1|manifest)+cngeneson+pc_mito+brain_region+Sex	7.315	408.000
ENSG00000172456	0.000	-0.565	-0.334	-0.795	FGGY	0.000	~p_tau+(1|manifest)+cngeneson+pc_mito+brain_region+Sex	1.698	2069.000
ENSG00000158296	0.058	-0.566	-0.049	-1.083	SLC13A3	0.107	~p_tau+(1|manifest)+cngeneson+pc_mito+brain_region+Sex	7.222	421.000
ENSG00000215252	0.199	-0.570	0.028	-1.168	GOLGA8B	0.274	~p_tau+(1|manifest)+cngeneson+pc_mito+brain_region+Sex	13.835	125.000
ENSG00000169902	0.320	-0.577	0.275	-1.428	TPST1	0.399	~p_tau+(1|manifest)+cngeneson+pc_mito+brain_region+Sex	36.135	10.000
ENSG00000137033	0.008	-0.579	-0.236	-0.922	IL33	0.025	~p_tau+(1|manifest)+cngeneson+pc_mito+brain_region+Sex	3.570	1044.000
ENSG00000163618	0.157	-0.580	0.052	-1.212	CADPS	0.229	~p_tau+(1|manifest)+cngeneson+pc_mito+brain_region+Sex	33.842	12.000
ENSG00000188803	0.037	-0.580	-0.139	-1.021	SHISA6	0.076	~p_tau+(1|manifest)+cngeneson+pc_mito+brain_region+Sex	5.770	577.000
ENSG00000177694	0.001	-0.584	-0.217	-0.951	NAALADL2	0.006	~p_tau+(1|manifest)+cngeneson+pc_mito+brain_region+Sex	11.004	208.000
ENSG00000182132	0.010	-0.611	-0.178	-1.043	KCNIP1	0.030	~p_tau+(1|manifest)+cngeneson+pc_mito+brain_region+Sex	14.286	114.000
ENSG00000072952	0.003	-0.620	-0.239	-1.001	MRVI1	0.012	~p_tau+(1|manifest)+cngeneson+pc_mito+brain_region+Sex	11.748	183.000
ENSG00000150477	0.000	-0.620	-0.347	-0.894	KIAA1328	0.001	~p_tau+(1|manifest)+cngeneson+pc_mito+brain_region+Sex	3.767	985.000
ENSG00000008311	0.000	-0.633	-0.288	-0.977	AASS	0.003	~p_tau+(1|manifest)+cngeneson+pc_mito+brain_region+Sex	8.388	333.000
ENSG00000132164	0.057	-0.635	-0.115	-1.155	SLC6A11	0.107	~p_tau+(1|manifest)+cngeneson+pc_mito+brain_region+Sex	11.714	185.000
ENSG00000167772	0.178	-0.651	0.065	-1.367	ANGPTL4	0.253	~p_tau+(1|manifest)+cngeneson+pc_mito+brain_region+Sex	14.527	107.000
ENSG00000120251	0.047	-0.652	-0.117	-1.187	GRIA2	0.091	~p_tau+(1|manifest)+cngeneson+pc_mito+brain_region+Sex	13.638	129.000
ENSG00000063601	0.000	-0.659	-0.330	-0.989	MTMR1	0.004	~p_tau+(1|manifest)+cngeneson+pc_mito+brain_region+Sex	5.842	561.000
ENSG00000120658	0.000	-0.677	-0.343	-1.011	ENOX1	0.001	~p_tau+(1|manifest)+cngeneson+pc_mito+brain_region+Sex	4.407	813.000
ENSG00000179796	0.000	-0.680	-0.331	-1.030	LRRC3B	0.001	~p_tau+(1|manifest)+cngeneson+pc_mito+brain_region+Sex	5.110	684.000
ENSG00000139155	0.001	-0.714	-0.342	-1.087	SLCO1C1	0.005	~p_tau+(1|manifest)+cngeneson+pc_mito+brain_region+Sex	12.047	172.000
ENSG00000265972	0.018	-0.727	-0.241	-1.212	TXNIP	0.044	~p_tau+(1|manifest)+cngeneson+pc_mito+brain_region+Sex	0.929	2704.000
ENSG00000186094	0.003	-0.773	-0.224	-1.322	AGBL4	0.014	~p_tau+(1|manifest)+cngeneson+pc_mito+brain_region+Sex	5.265	658.000
ENSG00000099250	0.008	-0.795	-0.291	-1.299	NRP1	0.026	~p_tau+(1|manifest)+cngeneson+pc_mito+brain_region+Sex	20.108	52.000
ENSG00000165092	0.002	-0.807	-0.130	-1.483	ALDH1A1	0.011	~p_tau+(1|manifest)+cngeneson+pc_mito+brain_region+Sex	12.568	159.000
ENSG00000099622	0.080	-0.872	-0.090	-1.654	CIRBP	0.138	~p_tau+(1|manifest)+cngeneson+pc_mito+brain_region+Sex	23.376	35.000
ENSG00000117525	0.030	-0.910	-0.164	-1.655	F3	0.065	~p_tau+(1|manifest)+cngeneson+pc_mito+brain_region+Sex	35.897	11.000
ENSG00000166432	0.000	-0.914	-0.422	-1.405	ZMAT1	0.004	~p_tau+(1|manifest)+cngeneson+pc_mito+brain_region+Sex	7.035	439.000
ENSG00000134146	0.000	-1.106	-0.578	-1.634	DPH6	0.001	~p_tau+(1|manifest)+cngeneson+pc_mito+brain_region+Sex	7.284	411.000
[illegible]	[illegible]	[illegible]	[illegible]	[illegible]	[illegible]	[illegible]	[illegible]	[illegible]	[illegible]

Supplementary Table 6_Astrocyte DGE
Astrocytes beta amyloid

DGE analysis in Total Astrocytes using MAST and beta_amyloid as contrast

ensembl_gene_id	pval	logFC	ci.hi	ci.lo	gene	padj	model	variance	variance_rank
ENSG00000109846	0.000	1.869	2.759	0.979	CRYAB	0.002	~amyloid_beta+(1|manifest)+cngeneson+pc_mito+brain_region+Sex	17.300	77.000
ENSG00000106211	0.000	1.573	2.316	0.831	HSPB1	0.002	~amyloid_beta+(1|manifest)+cngeneson+pc_mito+brain_region+Sex	18.664	59.000
ENSG00000116661	0.000	1.419	1.922	0.916	FBXO2	0.000	~amyloid_beta+(1|manifest)+cngeneson+pc_mito+brain_region+Sex	2.433	1954.000
ENSG00000176046	0.000	1.398	2.127	0.668	NUPR1	0.000	~amyloid_beta+(1|manifest)+cngeneson+pc_mito+brain_region+Sex	3.399	1317.000
ENSG00000183963	0.000	1.331	2.014	0.648	SMTN	0.001	~amyloid_beta+(1|manifest)+cngeneson+pc_mito+brain_region+Sex	3.398	1318.000
ENSG00000120694	0.007	1.331	2.503	0.159	HSPH1	0.033	~amyloid_beta+(1|manifest)+cngeneson+pc_mito+brain_region+Sex	32.638	17.000
ENSG00000134294	0.000	1.298	2.009	0.586	SLC38A2	0.001	~amyloid_beta+(1|manifest)+cngeneson+pc_mito+brain_region+Sex	1.136	3848.000
ENSG00000125144	0.000	1.222	1.953	0.490	MT1G	0.001	~amyloid_beta+(1|manifest)+cngeneson+pc_mito+brain_region+Sex	6.345	545.000
ENSG00000080824	0.000	1.211	1.755	0.667	HSP90AA1	0.002	~amyloid_beta+(1|manifest)+cngeneson+pc_mito+brain_region+Sex	16.485	80.000
ENSG00000116132	0.005	1.198	2.001	0.394	PRRX1	0.025	~amyloid_beta+(1|manifest)+cngeneson+pc_mito+brain_region+Sex	10.324	255.000
ENSG00000110958	0.003	1.193	1.984	0.403	PTGES3	0.021	~amyloid_beta+(1|manifest)+cngeneson+pc_mito+brain_region+Sex	23.650	34.000
ENSG00000007516	0.000	1.118	1.869	0.367	BAIAP3	0.000	~amyloid_beta+(1|manifest)+cngeneson+pc_mito+brain_region+Sex	2.065	2352.000
ENSG00000155368	0.000	1.071	1.740	0.401	DBI	0.003	~amyloid_beta+(1|manifest)+cngeneson+pc_mito+brain_region+Sex	4.024	1032.000
ENSG00000006652	0.000	1.040	1.539	0.540	IFRD1	0.001	~amyloid_beta+(1|manifest)+cngeneson+pc_mito+brain_region+Sex	2.067	2350.000
ENSG00000102362	0.000	1.034	1.648	0.420	SYTL4	0.003	~amyloid_beta+(1|manifest)+cngeneson+pc_mito+brain_region+Sex	7.379	428.000
ENSG00000106665	0.000	1.030	1.463	0.597	CLIP2	0.000	~amyloid_beta+(1|manifest)+cngeneson+pc_mito+brain_region+Sex	0.932	4298.000
ENSG00000140044	0.000	0.979	1.575	0.384	JDP2	0.004	~amyloid_beta+(1|manifest)+cngeneson+pc_mito+brain_region+Sex	8.633	336.000
ENSG00000198417	0.000	0.974	1.805	0.144	MT1F	0.006	~amyloid_beta+(1|manifest)+cngeneson+pc_mito+brain_region+Sex	6.809	490.000
ENSG00000124172	0.003	0.966	1.683	0.249	ATP5F1E	0.019	~amyloid_beta+(1|manifest)+cngeneson+pc_mito+brain_region+Sex	4.286	952.000
ENSG00000088986	0.006	0.964	1.693	0.235	DYNLL1	0.029	~amyloid_beta+(1|manifest)+cngeneson+pc_mito+brain_region+Sex	18.224	68.000
ENSG00000132002	0.009	0.953	1.759	0.146	DNAJB1	0.038	~amyloid_beta+(1|manifest)+cngeneson+pc_mito+brain_region+Sex	16.175	85.000
ENSG00000172428	0.001	0.934	1.581	0.288	COPS9	0.010	~amyloid_beta+(1|manifest)+cngeneson+pc_mito+brain_region+Sex	3.320	1357.000
ENSG00000178980	0.000	0.933	1.420	0.446	SELENOW	0.002	~amyloid_beta+(1|manifest)+cngeneson+pc_mito+brain_region+Sex	3.053	1506.000
ENSG00000096384	0.001	0.928	1.568	0.288	HSP90AB1	0.008	~amyloid_beta+(1|manifest)+cngeneson+pc_mito+brain_region+Sex	4.885	802.000
ENSG00000169715	0.003	0.928	1.613	0.242	MT1E	0.021	~amyloid_beta+(1|manifest)+cngeneson+pc_mito+brain_region+Sex	14.800	104.000
ENSG00000158106	0.000	0.917	1.427	0.407	RHPN1	0.003	~amyloid_beta+(1|manifest)+cngeneson+pc_mito+brain_region+Sex	4.242	967.000
ENSG00000087460	0.000	0.912	1.169	0.656	GNAS	0.000	~amyloid_beta+(1|manifest)+cngeneson+pc_mito+brain_region+Sex	1.813	2678.000
ENSG00000196739	0.000	0.907	1.223	0.590	COL27A1	0.000	~amyloid_beta+(1|manifest)+cngeneson+pc_mito+brain_region+Sex	2.534	1872.000
ENSG00000131748	0.000	0.894	1.248	0.541	STARD3	0.000	~amyloid_beta+(1|manifest)+cngeneson+pc_mito+brain_region+Sex	4.252	960.000
ENSG00000161011	0.000	0.880	1.334	0.426	SQSTM1	0.001	~amyloid_beta+(1|manifest)+cngeneson+pc_mito+brain_region+Sex	0.928	4309.000
ENSG00000170011	0.000	0.880	1.406	0.353	MYRIP	0.000	~amyloid_beta+(1|manifest)+cngeneson+pc_mito+brain_region+Sex	3.213	1417.000
ENSG00000105993	0.000	0.863	1.410	0.317	DNAJB6	0.006	~amyloid_beta+(1|manifest)+cngeneson+pc_mito+brain_region+Sex	5.291	711.000
ENSG00000032444	0.000	0.856	1.342	0.371	PNPLA6	0.001	~amyloid_beta+(1|manifest)+cngeneson+pc_mito+brain_region+Sex	1.372	3375.000
ENSG00000158186	0.000	0.849	1.176	0.522	MRAS	0.001	~amyloid_beta+(1|manifest)+cngeneson+pc_mito+brain_region+Sex	3.316	1360.000
ENSG00000112186	0.000	0.844	1.565	0.124	CAP2	0.002	~amyloid_beta+(1|manifest)+cngeneson+pc_mito+brain_region+Sex	10.952	225.000
ENSG00000079841	0.004	0.838	1.542	0.133	RIMS1	0.024	~amyloid_beta+(1|manifest)+cngeneson+pc_mito+brain_region+Sex	11.878	181.000
ENSG00000152137	0.000	0.816	1.117	0.516	HSPB8	0.000	~amyloid_beta+(1|manifest)+cngeneson+pc_mito+brain_region+Sex	2.503	1900.000
ENSG00000100490	0.000	0.794	1.160	0.428	CDKL1	0.000	~amyloid_beta+(1|manifest)+cngeneson+pc_mito+brain_region+Sex	1.091	3948.000
ENSG00000150991	0.003	0.793	1.352	0.235	UBC	0.021	~amyloid_beta+(1|manifest)+cngeneson+pc_mito+brain_region+Sex	3.959	1059.000
ENSG00000178498	0.000	0.792	1.180	0.404	DTX3	0.000	~amyloid_beta+(1|manifest)+cngeneson+pc_mito+brain_region+Sex	2.700	1728.000
ENSG00000086061	0.001	0.791	1.517	0.065	DNAJA1	0.011	~amyloid_beta+(1|manifest)+cngeneson+pc_mito+brain_region+Sex	6.988	474.000
ENSG00000157617	0.000	0.780	1.165	0.395	C2CD2	0.000	~amyloid_beta+(1|manifest)+cngeneson+pc_mito+brain_region+Sex	0.831	4541.000
ENSG00000160678	0.001	0.778	1.412	0.145	S100A1	0.010	~amyloid_beta+(1|manifest)+cngeneson+pc_mito+brain_region+Sex	2.575	1831.000
ENSG00000003989	0.000	0.770	1.167	0.374	SLC7A2	0.001	~amyloid_beta+(1|manifest)+cngeneson+pc_mito+brain_region+Sex	8.494	346.000
ENSG00000125148	0.003	0.768	1.184	0.353	MT2A	0.019	~amyloid_beta+(1|manifest)+cngeneson+pc_mito+brain_region+Sex	13.437	140.000
ENSG00000110172	0.004	0.764	1.534	-0.006	CHORDC1	0.025	~amyloid_beta+(1|manifest)+cngeneson+pc_mito+brain_region+Sex	12.961	151.000
ENSG00000119729	0.000	0.763	1.106	0.421	RHOQ	0.000	~amyloid_beta+(1|manifest)+cngeneson+pc_mito+brain_region+Sex	0.995	4149.000
ENSG00000127483	0.000	0.763	1.012	0.514	HP1BP3	0.000	~amyloid_beta+(1|manifest)+cngeneson+pc_mito+brain_region+Sex	0.896	4383.000
ENSG00000067057	0.001	0.759	1.291	0.227	PFKP	0.010	~amyloid_beta+(1|manifest)+cngeneson+pc_mito+brain_region+Sex	3.710	1158.000
ENSG00000069849	0.000	0.755	1.236	0.275	ATP1B3	0.002	~amyloid_beta+(1|manifest)+cngeneson+pc_mito+brain_region+Sex	3.149	1453.000
ENSG00000163931	0.000	0.749	1.147	0.351	TKT	0.001	~amyloid_beta+(1|manifest)+cngeneson+pc_mito+brain_region+Sex	1.805	2688.000
ENSG00000140264	0.000	0.743	1.228	0.258	SERF2	0.003	~amyloid_beta+(1|manifest)+cngeneson+pc_mito+brain_region+Sex	0.826	4552.000
ENSG00000182272	0.005	0.743	1.287	0.199	B4GALNT4	0.026	~amyloid_beta+(1|manifest)+cngeneson+pc_mito+brain_region+Sex	15.926	88.000
ENSG00000026508	0.292	0.743	1.813	-0.327	CD44	0.345	~amyloid_beta+(1|manifest)+cngeneson+pc_mito+brain_region+Sex	20.611	46.000
ENSG00000166295	0.000	0.739	1.032	0.447	ANAPC16	0.000	~amyloid_beta+(1|manifest)+cngeneson+pc_mito+brain_region+Sex	2.954	1569.000
ENSG00000154723	0.002	0.737	1.352	0.122	ATP5PF	0.014	~amyloid_beta+(1|manifest)+cngeneson+pc_mito+brain_region+Sex	2.899	1603.000
ENSG00000170485	0.000	0.732	1.056	0.409	NPAS2	0.004	~amyloid_beta+(1|manifest)+cngeneson+pc_mito+brain_region+Sex	1.129	3864.000
ENSG00000108773	0.000	0.731	1.163	0.299	KAT2A	0.005	~amyloid_beta+(1|manifest)+cngeneson+pc_mito+brain_region+Sex	6.400	536.000
ENSG00000168439	0.000	0.730	1.226	0.235	STIP1	0.001	~amyloid_beta+(1|manifest)+cngeneson+pc_mito+brain_region+Sex	2.155	2254.000
ENSG00000102547	0.000	0.729	1.001	0.457	CAB39L	0.000	~amyloid_beta+(1|manifest)+cngeneson+pc_mito+brain_region+Sex	1.111	3907.000
ENSG00000185955	0.000	0.727	1.239	0.215	C7orf61	0.005	~amyloid_beta+(1|manifest)+cngeneson+pc_mito+brain_region+Sex	2.698	1731.000
ENSG00000103253	0.001	0.723	1.316	0.130	HAGHL	0.013	~amyloid_beta+(1|manifest)+cngeneson+pc_mito+brain_region+Sex	3.936	1071.000
ENSG00000125971	0.000	0.720	1.072	0.368	DYNLRB1	0.000	~amyloid_beta+(1|manifest)+cngeneson+pc_mito+brain_region+Sex	0.674	4952.000
ENSG00000100364	0.000	0.716	0.999	0.434	KIAA0930	0.001	~amyloid_beta+(1|manifest)+cngeneson+pc_mito+brain_region+Sex	15.423	96.000
ENSG00000074964	0.000	0.715	0.950	0.479	[illegible]	0.000	~amyloid_beta+(1|manifest)+cngeneson+pc_mito+brain_region+Sex	3.491	1254.000
ENSG00000187514	0.001	0.713	1.223	0.202	PTMA	0.008	~amyloid_beta+(1|manifest)+cngeneson+pc_mito+brain_region+Sex	2.509	1888.000
ENSG00000127184	0.003	0.706	1.336	0.075	COX7C	0.018	~amyloid_beta+(1|manifest)+cngeneson+pc_mito+brain_region+Sex	3.208	1420.000
ENSG00000056736	0.000	0.704	1.046	0.362	IL17RB	0.001	~amyloid_beta+(1|manifest)+cngeneson+pc_mito+brain_region+Sex	1.746	2753.000
ENSG00000197063	0.000	0.701	1.016	0.386	MAFG	0.000	~amyloid_beta+(1|manifest)+cngeneson+pc_mito+brain_region+Sex	1.207	3689.000
ENSG00000107736	0.000	0.694	1.104	0.284	CDH23	0.002	~amyloid_beta+(1|manifest)+cngeneson+pc_mito+brain_region+Sex	3.706	1160.000
ENSG00000177600	0.000	0.691	1.103	0.278	RPLP2	0.001	~amyloid_beta+(1|manifest)+cngeneson+pc_mito+brain_region+Sex	4.908	794.000
ENSG00000182253	0.000	0.685	1.101	0.269	SYNM	0.000	~amyloid_beta+(1|manifest)+cngeneson+pc_mito+brain_region+Sex	3.987	1045.000
ENSG00000111328	0.000	0.684	1.037	0.331	CDK2AP1	0.000	~amyloid_beta+(1|manifest)+cngeneson+pc_mito+brain_region+Sex	0.697	4904.000
ENSG00000127922	0.011	0.678	1.243	0.112	SEM1	0.042	~amyloid_beta+(1|manifest)+cngeneson+pc_mito+brain_region+Sex	7.900	384.000
ENSG00000137818	0.000	0.672	1.175	0.169	RPLP1	0.007	~amyloid_beta+(1|manifest)+cngeneson+pc_mito+brain_region+Sex	4.679	845.000
ENSG00000147724	0.000	0.672	1.046	0.298	FAM135B	0.004	~amyloid_beta+(1|manifest)+cngeneson+pc_mito+brain_region+Sex	1.306	3488.000
ENSG00000130522	0.000	0.670	1.087	0.253	JUND	0.006	~amyloid_beta+(1|manifest)+cngeneson+pc_mito+brain_region+Sex	1.987	2450.000
ENSG00000133112	0.001	0.669	1.127	0.211	TPT1	0.012	~amyloid_beta+(1|manifest)+cngeneson+pc_mito+brain_region+Sex	3.904	1087.000
ENSG00000196924	0.003	0.667	1.199	0.135	FLNA	0.022	~amyloid_beta+(1|manifest)+cngeneson+pc_mito+brain_region+Sex	4.905	796.000
ENSG00000133195	0.000	0.664	0.930	0.399	SLC39A11	0.000	~amyloid_beta+(1|manifest)+cngeneson+pc_mito+brain_region+Sex	15.284	98.000
ENSG00000158201	0.002	0.662	1.189	0.134	ABHD3	0.014	~amyloid_beta+(1|manifest)+cngeneson+pc_mito+brain_region+Sex	6.007	590.000
ENSG00000165238	0.000	0.659	1.022	0.296	WNK2	0.001	~amyloid_beta+(1|manifest)+cngeneson+pc_mito+brain_region+Sex	3.010	1536.000
ENSG00000142168	0.000	0.657	1.212	0.102	SOD1	0.006	~amyloid_beta+(1|manifest)+cngeneson+pc_mito+brain_region+Sex	2.698	1732.000
ENSG00000146278	0.000	0.654	0.979	0.329	PNRC1	0.001	~amyloid_beta+(1|manifest)+cngeneson+pc_mito+brain_region+Sex	0.672	4961.000
ENSG00000178209	0.000	0.650	0.931	0.369	PLEC	0.001	~amyloid_beta+(1|manifest)+cngeneson+pc_mito+brain_region+Sex	9.567	291.000
ENSG00000181019	0.000	0.643	1.215	0.070	NQO1	0.001	~amyloid_beta+(1|manifest)+cngeneson+pc_mito+brain_region+Sex	5.619	662.000
ENSG00000176533	0.000	0.640	0.959	0.322	GNG7	0.000	~amyloid_beta+(1|manifest)+cngeneson+pc_mito+brain_region+Sex	2.304	2100.000
ENSG00000185829	0.000	0.640	1.095	0.186	ARL17A	0.004	~amyloid_beta+(1|manifest)+cngeneson+pc_mito+brain_region+Sex	1.536	3075.000
ENSG00000079459	0.000	0.638	0.895	0.380	FDFT1	0.000	~amyloid_beta+(1|manifest)+cngeneson+pc_mito+brain_region+Sex	0.885	4413.000
ENSG00000185950	0.001	0.637	0.996	0.277	IRS2	0.013	~amyloid_beta+(1|manifest)+cngeneson+pc_mito+brain_region+Sex	4.229	972.000
ENSG00000112335	0.000	0.632	1.084	0.180	SNX3	0.005	~amyloid_beta+(1|manifest)+cngeneson+pc_mito+brain_region+Sex	2.688	1737.000
ENSG00000117450	0.003	0.629	1.203	0.054	PRDX1	0.018	~amyloid_beta+(1|manifest)+cngeneson+pc_mito+brain_region+Sex	3.170	1442.000
ENSG00000087086	0.013	0.625	1.085	0.165	FTL	0.047	~amyloid_beta+(1|manifest)+cngeneson+pc_mito+brain_region+Sex	5.951	598.000
ENSG00000130304	0.000	0.625	0.936	0.314	SLC27A1	0.002	~amyloid_beta+(1|manifest)+cngeneson+pc_mito+brain_region+Sex	2.670	1753.000
ENSG00000198792	0.008	0.623	1.087	0.160	[illegible]	0.034	~amyloid_beta+(1|manifest)+cngeneson+pc_mito+brain_region+Sex	6.741	500.000
ENSG00000186635	0.000	0.622	0.923	0.322	ARAP1	0.000	~amyloid_beta+(1|manifest)+cngeneson+pc_mito+brain_region+Sex	1.469	3188.000
ENSG00000144381	0.002	0.619	1.149	0.089	HSPD1	0.016	~amyloid_beta+(1|manifest)+cngeneson+pc_mito+brain_region+Sex	3.416	1304.000
ENSG00000148411	0.001	0.619	1.001	0.237	NACC2	0.008	~amyloid_beta+(1|manifest)+cngeneson+pc_mito+brain_region+Sex	7.509	415.000
ENSG00000089220	0.000	0.616	0.872	0.361	PEBP1	0.000	~amyloid_beta+(1|manifest)+cngeneson+pc_mito+brain_region+Sex	2.640	1778.000
ENSG00000080546	0.002	0.615	0.992	0.239	SESN1	0.014	~amyloid_beta+(1|manifest)+cngeneson+pc_mito+brain_region+Sex	2.243	2162.000
ENSG00000173406	0.005	0.613	1.166	0.060	DAB1	0.026	~amyloid_beta+(1|manifest)+cngeneson+pc_mito+brain_region+Sex	3.618	1198.000
ENSG00000138193	0.019	0.611	1.342	-0.119	PLCE1	0.057	~amyloid_beta+(1|manifest)+cngeneson+pc_mito+brain_region+Sex	10.428	250.000
ENSG00000173812	0.001	0.611	1.108	0.114	EIF1	0.013	~amyloid_beta+(1|manifest)+cngeneson+pc_mito+brain_region+Sex	3.388	1324.000
ENSG00000101752	0.005	0.611	1.019	0.202	MIB1	0.026	~amyloid_beta+(1|manifest)+cngeneson+pc_mito+brain_region+Sex	7.856	386.000
ENSG00000164949	0.005	0.607	1.074	0.139	GEM	0.027	~amyloid_beta+(1|manifest)+cngeneson+pc_mito+brain_region+Sex	2.945	1578.000
ENSG00000162545	0.000	0.606	0.916	0.297	CAMK2N1	0.000	~amyloid_beta+(1|manifest)+cngeneson+pc_mito+brain_region+Sex	2.384	2010.000
ENSG00000169733	0.000	0.606	0.959	0.253	RFNG	0.002	~amyloid_beta+(1|manifest)+cngeneson+pc_mito+brain_region+Sex	3.987	1046.000
ENSG00000120693	0.000	0.603	1.121	0.086	SMAD9	0.005	~amyloid_beta+(1|manifest)+cngeneson+pc_mito+brain_region+Sex	0.519	5306.000
ENSG00000159176	0.001	0.601	0.922	0.279	CSRP1	0.007	~amyloid_beta+(1|manifest)+cngeneson+pc_mito+brain_region+Sex	3.421	1300.000
ENSG00000103260	0.036	0.599	1.198	0.000	METRN	0.082	~amyloid_beta+(1|manifest)+cngeneson+pc_mito+brain_region+Sex	9.554	294.000
ENSG00000205364	0.014	0.599	1.251	-0.054	MT1M	0.047	~amyloid_beta+(1|manifest)+cngeneson+pc_mito+brain_region+Sex	5.046	763.000
ENSG00000135862	0.000	0.597	0.984	0.210	LAMC1	0.001	~amyloid_beta+(1|manifest)+cngeneson+pc_mito+brain_region+Sex	2.508	1891.000
ENSG00000119900	0.000	0.596	0.896	0.297	OGFRL1	0.005	~amyloid_beta+(1|manifest)+cngeneson+pc_mito+brain_region+Sex	6.698	508.000
ENSG00000149177	0.000	0.596	0.973	0.220	PTPRJ	0.000	~amyloid_beta+(1|manifest)+cngeneson+pc_mito+brain_region+Sex	1.798	2702.000
ENSG00000175866	0.000	0.595	0.939	0.252	BAIAP2	0.001	~amyloid_beta+(1|manifest)+cngeneson+pc_mito+brain_region+Sex	2.195	2208.000
ENSG00000049759	0.019	0.595	1.050	0.139	NEDD4L	0.057	~amyloid_beta+(1|manifest)+cngeneson+pc_mito+brain_region+Sex	7.086	460.000
ENSG00000178573	0.000	0.591	0.974	0.208	MAF	0.003	~amyloid_beta+(1|manifest)+cngeneson+pc_mito+brain_region+Sex	1.961	2478.000
ENSG00000184371	0.000	0.591	0.935	0.246	CSF1	0.001	~amyloid_beta+(1|manifest)+cngeneson+pc_mito+brain_region+Sex	0.842	4519.000
ENSG00000173915	0.009	0.587	1.179	-0.005	ATP5MD	0.037	~amyloid_beta+(1|manifest)+cngeneson+pc_mito+brain_region+Sex	3.870	1097.000
ENSG00000157216	0.000	0.584	0.855	0.314	SSBP3	0.001	~amyloid_beta+(1|manifest)+cngeneson+pc_mito+brain_region+Sex	2.283	2120.000
ENSG00000162734	0.000	0.583	0.926	0.241	PEA15	0.001	~amyloid_beta+(1|manifest)+cngeneson+pc_mito+brain_region+Sex	3.460	1271.000
ENSG00000132329	0.000	0.582	0.813	0.351	RAMP1	0.000	~amyloid_beta+(1|manifest)+cngeneson+pc_mito+brain_region+Sex	0.850	4502.000
ENSG00000162413	0.000	0.580	0.887	0.273	KLHL21	0.001	~amyloid_beta+(1|manifest)+cngeneson+pc_mito+brain_region+Sex	1.695	2829.000
ENSG00000132024	0.000	0.579	0.820	0.339	CC2D1A	0.000	~amyloid_beta+(1|manifest)+cngeneson+pc_mito+brain_region+Sex	1.890	2576.000
ENSG00000135404	0.001	0.579	1.041	0.117	CD63	0.010	~amyloid_beta+(1|manifest)+cngeneson+pc_mito+brain_region+Sex	3.162	1446.000
ENSG00000178104	0.001	0.573	0.870	0.277	PDE4DIP	0.011	~amyloid_beta+(1|manifest)+cngeneson+pc_mito+brain_region+Sex	3.000	1544.000
ENSG00000170315	0.001	0.573	1.158	-0.013	UBB	0.011	~amyloid_beta+(1|manifest)+cngeneson+pc_mito+brain_region+Sex	3.583	1215.000
ENSG00000143819	0.003	0.571	0.978	0.164	EPHX1	0.019	~amyloid_beta+(1|manifest)+cngeneson+pc_mito+brain_region+Sex	3.071	1497.000
ENSG00000189403	0.151	0.568	1.136	-0.001	HMGB1	0.207	~amyloid_beta+(1|manifest)+cngeneson+pc_mito+brain_region+Sex	39.484	8.000
ENSG00000129422	0.000	0.566	0.782	0.350	MTUS1	0.001	~amyloid_beta+(1|manifest)+cngeneson+pc_mito+brain_region+Sex	2.169	2238.000
ENSG00000156299	0.000	0.565	0.923	0.206	TIAM1	0.005	~amyloid_beta+(1|manifest)+cngeneson+pc_mito+brain_region+Sex	2.200	2202.000
ENSG00000172987	0.089	0.563	1.440	-0.314	HPSE2	0.144	~amyloid_beta+(1|manifest)+cngeneson+pc_mito+brain_region+Sex	11.069	217.000
ENSG00000131584	0.000	0.562	0.918	0.206	ACAP3	0.004	~amyloid_beta+(1|manifest)+cngeneson+pc_mito+brain_region+Sex	2.676	1749.000
ENSG00000170385	0.000	0.562	0.984	0.139	SLC30A1	0.004	~amyloid_beta+(1|manifest)+cngeneson+pc_mito+brain_region+Sex	1.399	3323.000
ENSG00000172809	0.001	0.558	1.069	0.048	RPL38	0.013	~amyloid_beta+(1|manifest)+cngeneson+pc_mito+brain_region+Sex	4.329	940.000
ENSG00000101236	0.010	0.558	1.040	0.076	RNF24	0.039	~amyloid_beta+(1|manifest)+cngeneson+pc_mito+brain_region+Sex	2.719	1720.000
ENSG00000172216	0.002	0.557	1.000	0.115	CEBPB	0.016	~amyloid_beta+(1|manifest)+cngeneson+pc_mito+brain_region+Sex	2.074	2342.000
ENSG00000124749	0.112	0.557	1.289	-0.174	COL21A1	0.168	~amyloid_beta+(1|manifest)+cngeneson+pc_mito+brain_region+Sex	5.220	724.000
ENSG00000188385	0.000	0.555	0.848	0.262	JAKMIP3	0.000	~amyloid_beta+(1|manifest)+cngeneson+pc_mito+brain_region+Sex	0.603	5120.000
ENSG00000140319	0.007	0.551	1.136	-0.033	SRP14	0.032	~amyloid_beta+(1|manifest)+cngeneson+pc_mito+brain_region+Sex	2.077	2336.000
ENSG00000189043	0.002	0.546	1.039	0.052	NDUFA4	0.015	~amyloid_beta+(1|manifest)+cngeneson+pc_mito+brain_region+Sex	2.516	1882.000
ENSG00000120306	0.001	0.545	0.899	0.192	CYSTM1	0.014	~amyloid_beta+(1|manifest)+cngeneson+pc_mito+brain_region+Sex	2.128	2281.000
ENSG00000124942	0.000	0.545	0.726	0.365	AHNAK	0.000	~amyloid_beta+(1|manifest)+cngeneson+pc_mito+brain_region+Sex	2.385	2008.000
ENSG00000154721	0.000	0.543	0.896	0.191	JAM2	0.004	~amyloid_beta+(1|manifest)+cngeneson+pc_mito+brain_region+Sex	1.469	3187.000
ENSG00000156482	0.006	0.543	1.052	0.034	RPL30	0.028	~amyloid_beta+(1|manifest)+cngeneson+pc_mito+brain_region+Sex	4.640	858.000
ENSG00000104960	0.000	0.543	0.870	0.216	PTOV1	0.002	~amyloid_beta+(1|manifest)+cngeneson+pc_mito+brain_region+Sex	2.375	2024.000
ENSG00000142676	0.004	0.542	1.047	0.038	RPL11	0.025	~amyloid_beta+(1|manifest)+cngeneson+pc_mito+brain_region+Sex	6.059	584.000
ENSG00000164919	0.000	0.542	0.948	0.135	COX6C	0.007	~amyloid_beta+(1|manifest)+cngeneson+pc_mito+brain_region+Sex	0.704	4869.000
ENSG00000111885	0.000	0.541	0.804	0.279	MAN1A1	0.000	~amyloid_beta+(1|manifest)+cngeneson+pc_mito+brain_region+Sex	0.521	5304.000
ENSG00000137509	0.000	0.539	0.814	0.265	PRCP	0.002	~amyloid_beta+(1|manifest)+cngeneson+pc_mito+brain_region+Sex	1.674	2865.000
ENSG00000240583	0.217	0.537	1.185	-0.111	AQP1	0.271	~amyloid_beta+(1|manifest)+cngeneson+pc_mito+brain_region+Sex	11.404	203.000
ENSG00000170606	0.000	0.536	0.917	0.154	HSPA4	0.003	~amyloid_beta+(1|manifest)+cngeneson+pc_mito+brain_region+Sex	1.799	2700.000
ENSG00000154380	0.000	0.535	0.801	0.269	ENAH	0.004	~amyloid_beta+(1|manifest)+cngeneson+pc_mito+brain_region+Sex	3.332	1352.000
ENSG00000177807	0.019	0.535	1.026	0.043	KCNJ10	0.057	~amyloid_beta+(1|manifest)+cngeneson+pc_mito+brain_region+Sex	3.627	1191.000
ENSG00000197756	0.001	0.535	0.888	0.182	RPL37A	0.010	~amyloid_beta+(1|manifest)+cngeneson+pc_mito+brain_region+Sex	2.876	1619.000
ENSG00000188039	0.043	0.534	1.195	-0.128	NWD1	0.091	~amyloid_beta+(1|manifest)+cngeneson+pc_mito+brain_region+Sex	8.825	324.000
ENSG00000131143	0.000	0.532	0.862	0.201	COX4I1	0.002	~amyloid_beta+(1|manifest)+cngeneson+pc_mito+brain_region+Sex	1.819	2668.000
ENSG00000079337	0.005	0.530	0.944	0.116	RAPGEF3	0.028	~amyloid_beta+(1|manifest)+cngeneson+pc_mito+brain_region+Sex	6.309	548.000
ENSG00000178951	0.000	0.529	0.734	0.324	ZBTB7A	0.000	~amyloid_beta+(1|manifest)+cngeneson+pc_mito+brain_region+Sex	1.706	2816.000
ENSG00000099875	0.001	0.528	0.933	0.123	MKNK2	0.010	~amyloid_beta+(1|manifest)+cngeneson+pc_mito+brain_region+Sex	5.901	607.000
ENSG00000109906	0.000	0.527	0.702	0.351	ZBTB16	0.000	~amyloid_beta+(1|manifest)+cngeneson+pc_mito+brain_region+Sex	0.914	4344.000
ENSG00000149403	0.000	0.525	0.861	0.190	GRIK4	0.006	~amyloid_beta+(1|manifest)+cngeneson+pc_mito+brain_region+Sex	1.498	3139.000
ENSG00000164070	0.023	0.525	1.161	-0.111	HSPA4L	0.064	~amyloid_beta+(1|manifest)+cngeneson+pc_mito+brain_region+Sex	13.861	126.000
ENSG00000163703	0.001	0.524	0.851	0.197	CRELD1	0.013	~amyloid_beta+(1|manifest)+cngeneson+pc_mito+brain_region+Sex	4.251	961.000
ENSG00000100380	0.006	0.524	1.047	0.000	ST13	0.028	~amyloid_beta+(1|manifest)+cngeneson+pc_mito+brain_region+Sex	3.550	1226.000
ENSG00000157445	0.036	0.520	1.101	-0.060	CACNA2D3	0.082	~amyloid_beta+(1|manifest)+cngeneson+pc_mito+brain_region+Sex	9.546	295.000
ENSG00000008710	0.003	0.517	0.880	0.154	PKD1	0.018	~amyloid_beta+(1|manifest)+cngeneson+pc_mito+brain_region+Sex	6.431	533.000
ENSG00000133794	0.000	0.517	0.922	0.111	ARNTL	0.004	~amyloid_beta+(1|manifest)+cngeneson+pc_mito+brain_region+Sex	0.649	5000.000
ENSG00000151468	0.000	0.515	0.849	0.182	CCDC3	0.002	~amyloid_beta+(1|manifest)+cngeneson+pc_mito+brain_region+Sex	1.592	2995.000
ENSG00000090530	0.000	0.515	0.891	0.138	P3H2	0.003	~amyloid_beta+(1|manifest)+cngeneson+pc_mito+brain_region+Sex	0.924	4321.000
ENSG00000170579	0.019	0.515	0.953	0.076	DLGAP1	0.057	~amyloid_beta+(1|manifest)+cngeneson+pc_mito+brain_region+Sex	9.650	285.000
ENSG00000130997	0.000	0.513	0.716	0.311	POLN	0.000	~amyloid_beta+(1|manifest)+cngeneson+pc_mito+brain_region+Sex	0.572	5182.000
ENSG00000172530	0.000	0.513	0.823	0.203	BANP	0.006	~amyloid_beta+(1|manifest)+cngeneson+pc_mito+brain_region+Sex	2.412	1971.000
ENSG00000141504	0.005	0.511	0.969	0.054	SAT2	0.028	~amyloid_beta+(1|manifest)+cngeneson+pc_mito+brain_region+Sex	5.761	633.000
ENSG00000168056	0.004	0.510	0.915	0.105	LTBP3	0.023	~amyloid_beta+(1|manifest)+cngeneson+pc_mito+brain_region+Sex	7.509	417.000
ENSG00000160213	0.016	0.510	1.166	-0.147	CSTB	0.052	~amyloid_beta+(1|manifest)+cngeneson+pc_mito+brain_region+Sex	3.511	1245.000
ENSG00000087903	0.002	0.510	0.792	0.227	RFX2	0.015	~amyloid_beta+(1|manifest)+cngeneson+pc_mito+brain_region+Sex	4.678	846.000
ENSG00000173599	0.000	0.509	0.696	0.322	PC	0.000	~amyloid_beta+(1|manifest)+cngeneson+pc_mito+brain_region+Sex	0.983	4182.000
ENSG00000196683	0.001	0.508	0.968	0.048	TOMM7	0.010	~amyloid_beta+(1|manifest)+cngeneson+pc_mito+brain_region+Sex	4.388	920.000
ENSG00000092931	0.000	0.507	0.733	0.282	MFSD11	0.000	~amyloid_beta+(1|manifest)+cngeneson+pc_mito+brain_region+Sex	1.937	2512.000
ENSG00000160789	0.000	0.507	0.797	0.217	LMNA	0.002	~amyloid_beta+(1|manifest)+cngeneson+pc_mito+brain_region+Sex	2.514	1884.000
ENSG00000122756	0.002	0.505	0.935	0.076	CNTFR	0.017	~amyloid_beta+(1|manifest)+cngeneson+pc_mito+brain_region+Sex	5.436	686.000
ENSG00000164970	0.000	0.504	0.752	0.256	FAM219A	0.000	~amyloid_beta+(1|manifest)+cngeneson+pc_mito+brain_region+Sex	1.236	3622.000
ENSG00000171206	0.000	0.503	0.821	0.185	TRIM8	0.006	~amyloid_beta+(1|manifest)+cngeneson+pc_mito+brain_region+Sex	3.456	1273.000
ENSG00000135924	0.003	0.502	0.918	0.086	DNAJB2	0.021	~amyloid_beta+(1|manifest)+cngeneson+pc_mito+brain_region+Sex	6.379	539.000
ENSG00000073849	0.002	0.500	1.133	-0.132	ST6GAL1	0.016	~amyloid_beta+(1|manifest)+cngeneson+pc_mito+brain_region+Sex	4.721	836.000
ENSG00000158864	0.001	0.499	0.884	0.113	NDUFS2	0.012	~amyloid_beta+(1|manifest)+cngeneson+pc_mito+brain_region+Sex	1.891	2572.000
ENSG00000132334	0.001	0.499	0.834	0.163	PTPRE	0.011	~amyloid_beta+(1|manifest)+cngeneson+pc_mito+brain_region+Sex	2.538	1869.000
ENSG00000232112	0.013	0.498	1.050	-0.054	TMA7	0.047	~amyloid_beta+(1|manifest)+cngeneson+pc_mito+brain_region+Sex	3.498	1251.000
ENSG00000165795	0.027	0.496	0.839	0.154	NDRG2	0.070	~amyloid_beta+(1|manifest)+cngeneson+pc_mito+brain_region+Sex	22.859	35.000
ENSG00000163637	0.008	0.496	0.861	0.131	PRICKLE2	0.034	~amyloid_beta+(1|manifest)+cngeneson+pc_mito+brain_region+Sex	6.061	583.000
ENSG00000165416	0.005	0.494	0.974	0.013	SUGT1	0.027	~amyloid_beta+(1|manifest)+cngeneson+pc_mito+brain_region+Sex	3.999	1040.000
ENSG00000092096	0.007	0.493	0.973	0.014	SLC22A17	0.033	~amyloid_beta+(1|manifest)+cngeneson+pc_mito+brain_region+Sex	3.524	1237.000
ENSG00000166925	0.005	0.493	0.864	0.121	TSC22D4	0.028	~amyloid_beta+(1|manifest)+cngeneson+pc_mito+brain_region+Sex	7.504	418.000
ENSG00000133083	0.124	0.493	1.187	-0.202	DCLK1	0.180	~amyloid_beta+(1|manifest)+cngeneson+pc_mito+brain_region+Sex	4.110	1010.000
ENSG00000139793	0.004	0.492	0.769	0.215	MBNL2	0.025	~amyloid_beta+(1|manifest)+cngeneson+pc_mito+brain_region+Sex	11.305	206.000
ENSG00000147162	0.000	0.490	0.744	0.236	OGT	0.001	~amyloid_beta+(1|manifest)+cngeneson+pc_mito+brain_region+Sex	0.898	4377.000
ENSG00000123094	0.015	0.490	0.816	0.163	RASSF8	0.051	~amyloid_beta+(1|manifest)+cngeneson+pc_mito+brain_region+Sex	2.507	1893.000
ENSG00000156508	0.003	0.487	0.946	0.029	EEF1A1	0.021	~amyloid_beta+(1|manifest)+cngeneson+pc_mito+brain_region+Sex	4.278	953.000
ENSG00000105855	0.000	0.485	0.664	0.305	ITGB8	0.001	~amyloid_beta+(1|manifest)+cngeneson+pc_mito+brain_region+Sex	0.444	5472.000
ENSG00000198668	0.003	0.485	0.789	0.180	CALM1	0.019	~amyloid_beta+(1|manifest)+cngeneson+pc_mito+brain_region+Sex	2.930	1588.000
ENSG00000087250	0.024	0.481	0.805	0.156	MT3	0.065	~amyloid_beta+(1|manifest)+cngeneson+pc_mito+brain_region+Sex	14.685	107.000
ENSG00000170365	0.027	0.478	0.936	0.021	SMAD1	0.070	~amyloid_beta+(1|manifest)+cngeneson+pc_mito+brain_region+Sex	5.820	622.000
ENSG00000126858	0.001	0.478	0.732	0.224	RHOT1	0.008	~amyloid_beta+(1|manifest)+cngeneson+pc_mito+brain_region+Sex	2.147	2265.000
ENSG00000106397	0.002	0.478	0.925	0.031	PLOD3	0.017	~amyloid_beta+(1|manifest)+cngeneson+pc_mito+brain_region+Sex	3.062	1503.000
ENSG00000067113	0.004	0.478	0.759	0.196	PLPP1	0.024	~amyloid_beta+(1|manifest)+cngeneson+pc_mito+brain_region+Sex	3.747	1145.000
ENSG00000139970	0.000	0.472	0.791	0.154	RTN1	0.002	~amyloid_beta+(1|manifest)+cngeneson+pc_mito+brain_region+Sex	1.613	2962.000
ENSG00000103249	0.001	0.472	0.802	0.142	CLCN7	0.010	~amyloid_beta+(1|manifest)+cngeneson+pc_mito+brain_region+Sex	1.724	2792.000
ENSG00000170638	0.000	0.472	0.756	0.188	TRABD	0.003	~amyloid_beta+(1|manifest)+cngeneson+pc_mito+brain_region+Sex	1.733	2775.000
ENSG00000196233	0.001	0.472	0.743	0.201	LCOR	0.010	~amyloid_beta+(1|manifest)+cngeneson+pc_mito+brain_region+Sex	1.741	2763.000
ENSG00000100591	0.000	0.472	0.778	0.166	AHSA1	0.001	~amyloid_beta+(1|manifest)+cngeneson+pc_mito+brain_region+Sex	0.852	4495.000
ENSG00000142192	0.011	0.471	0.937	0.005	APP	0.042	~amyloid_beta+(1|manifest)+cngeneson+pc_mito+brain_region+Sex	4.013	1037.000
ENSG00000183723	0.000	0.471	0.686	0.256	CMTM4	0.000	~amyloid_beta+(1|manifest)+cngeneson+pc_mito+brain_region+Sex	0.211	5860.000
ENSG00000130511	0.000	0.471	0.756	0.186	SSBP4	0.004	~amyloid_beta+(1|manifest)+cngeneson+pc_mito+brain_region+Sex	2.151	2260.000
ENSG00000162733	0.068	0.470	1.017	-0.076	DDR2	0.122	~amyloid_beta+(1|manifest)+cngeneson+pc_mito+brain_region+Sex	8.304	358.000
ENSG00000084090	0.000	0.468	0.697	0.238	STARD7	0.000	~amyloid_beta+(1|manifest)+cngeneson+pc_mito+brain_region+Sex	0.845	4511.000
ENSG00000177030	0.000	0.467	0.762	0.172	DEAF1	0.004	~amyloid_beta+(1|manifest)+cngeneson+pc_mito+brain_region+Sex	1.263	3575.000
ENSG00000275023	0.001	0.466	0.764	0.167	MLLT6	0.010	~amyloid_beta+(1|manifest)+cngeneson+pc_mito+brain_region+Sex	4.698	840.000
ENSG00000171604	0.000	0.463	0.610	0.316	CXXC5	0.000	~amyloid_beta+(1|manifest)+cngeneson+pc_mito+brain_region+Sex	0.269	5783.000
ENSG00000117115	0.007	0.461	1.030	-0.107	PADI2	0.032	~amyloid_beta+(1|manifest)+cngeneson+pc_mito+brain_region+Sex	4.549	880.000
ENSG00000119899	0.000	0.461	0.643	0.280	SLC17A5	0.000	~amyloid_beta+(1|manifest)+cngeneson+pc_mito+brain_region+Sex	0.788	4656.000
ENSG00000176463	0.001	0.461	0.778	0.144	SLCO3A1	0.012	~amyloid_beta+(1|manifest)+cngeneson+pc_mito+brain_region+Sex	0.844	4512.000
ENSG00000131459	0.000	0.458	0.738	0.179	GFPT2	0.003	~amyloid_beta+(1|manifest)+cngeneson+pc_mito+brain_region+Sex	1.590	2999.000
ENSG00000173598	0.002	0.458	0.862	0.053	NUDT4	0.015	~amyloid_beta+(1|manifest)+cngeneson+pc_mito+brain_region+Sex	5.139	742.000
ENSG00000099624	0.000	0.458	0.802	0.113	ATP5F1D	0.003	~amyloid_beta+(1|manifest)+cngeneson+pc_mito+brain_region+Sex	1.699	2821.000
ENSG00000130254	0.001	0.457	0.772	0.141	SAFB2	0.013	~amyloid_beta+(1|manifest)+cngeneson+pc_mito+brain_region+Sex	5.072	761.000
ENSG00000154655	0.003	0.455	1.050	-0.139	L3MBTL4	0.022	~amyloid_beta+(1|manifest)+cngeneson+pc_mito+brain_region+Sex	5.314	706.000
ENSG00000174943	0.001	0.455	0.803	0.106	KCTD13	0.008	~amyloid_beta+(1|manifest)+cngeneson+pc_mito+brain_region+Sex	2.057	2359.000
ENSG00000163453	0.029	0.454	0.801	0.107	IGFBP7	0.072	~amyloid_beta+(1|manifest)+cngeneson+pc_mito+brain_region+Sex	14.565	110.000
ENSG00000163531	0.004	0.454	0.941	-0.033	NFASC	0.024	~amyloid_beta+(1|manifest)+cngeneson+pc_mito+brain_region+Sex	3.537	1230.000
ENSG00000123933	0.006	0.454	0.819	0.088	MXD4	0.029	~amyloid_beta+(1|manifest)+cngeneson+pc_mito+brain_region+Sex	4.042	1030.000
ENSG00000168397	0.002	0.452	0.812	0.093	ATG4B	0.017	~amyloid_beta+(1|manifest)+cngeneson+pc_mito+brain_region+Sex	3.798	1123.000
ENSG00000130203	0.130	0.451	0.872	0.030	APOE	0.187	~amyloid_beta+(1|manifest)+cngeneson+pc_mito+brain_region+Sex	20.318	51.000
ENSG00000087365	0.000	0.450	0.788	0.112	SF3B2	0.006	~amyloid_beta+(1|manifest)+cngeneson+pc_mito+brain_region+Sex	0.995	4150.000
ENSG00000109390	0.000	0.450	0.761	0.138	NDUFC1	0.004	~amyloid_beta+(1|manifest)+cngeneson+pc_mito+brain_region+Sex	0.873	4430.000
ENSG00000159069	0.005	0.448	0.864	0.033	FBXW5	0.026	~amyloid_beta+(1|manifest)+cngeneson+pc_mito+brain_region+Sex	3.056	1505.000
ENSG00000123836	0.005	0.444	0.900	-0.012	PFKFB2	0.026	~amyloid_beta+(1|manifest)+cngeneson+pc_mito+brain_region+Sex	4.822	817.000
ENSG00000112309	0.020	0.443	0.890	-0.004	B3GAT2	0.059	~amyloid_beta+(1|manifest)+cngeneson+pc_mito+brain_region+Sex	1.054	4015.000
ENSG00000100906	0.000	0.442	0.804	0.080	NFKBIA	0.004	~amyloid_beta+(1|manifest)+cngeneson+pc_mito+brain_region+Sex	1.574	3017.000
ENSG00000164050	0.013	0.441	0.762	0.119	PLXNB1	0.047	~amyloid_beta+(1|manifest)+cngeneson+pc_mito+brain_region+Sex	9.815	279.000
ENSG00000160445	0.000	0.440	0.614	0.267	ZER1	0.000	~amyloid_beta+(1|manifest)+cngeneson+pc_mito+brain_region+Sex	2.358	2041.000
ENSG00000095319	0.000	0.440	0.690	0.191	NUP188	0.001	~amyloid_beta+(1|manifest)+cngeneson+pc_mito+brain_region+Sex	1.375	3367.000
ENSG00000156467	0.028	0.439	0.921	-0.043	UQCRB	0.072	~amyloid_beta+(1|manifest)+cngeneson+pc_mito+brain_region+Sex	3.850	1105.000
ENSG00000174705	0.001	0.437	0.654	0.220	SH3PXD2B	0.008	~amyloid_beta+(1|manifest)+cngeneson+pc_mito+brain_region+Sex	2.599	1814.000
ENSG00000184752	0.002	0.437	0.793	0.080	NDUFA12	0.014	~amyloid_beta+(1|manifest)+cngeneson+pc_mito+brain_region+Sex	0.889	4401.000
ENSG00000072954	0.000	0.437	0.734	0.139	TMEM38A	0.000	~amyloid_beta+(1|manifest)+cngeneson+pc_mito+brain_region+Sex	2.150	2261.000
ENSG00000241127	0.000	0.436	0.676	0.197	YAE1	0.002	~amyloid_beta+(1|manifest)+cngeneson+pc_mito+brain_region+Sex	1.485	3169.000
ENSG00000173660	0.000	0.436	0.706	0.166	UQCRH	0.002	~amyloid_beta+(1|manifest)+cngeneson+pc_mito+brain_region+Sex	1.045	4035.000
ENSG00000114416	0.021	0.435	0.832	0.038	FXR1	0.061	~amyloid_beta+(1|manifest)+cngeneson+pc_mito+brain_region+Sex	3.211	1419.000
ENSG00000130402	0.000	0.435	0.596	0.274	ACTN4	0.000	~amyloid_beta+(1|manifest)+cngeneson+pc_mito+brain_region+Sex	0.698	4898.000
ENSG00000170266	0.000	0.433	0.579	0.287	GLB1	0.000	~amyloid_beta+(1|manifest)+cngeneson+pc_mito+brain_region+Sex	0.453	5449.000
ENSG00000188343	0.000	0.433	0.587	0.278	FAM92A	0.000	~amyloid_beta+(1|manifest)+cngeneson+pc_mito+brain_region+Sex	0.601	5126.000
ENSG00000154122	0.000	0.433	0.621	0.245	ANKH	0.000	~amyloid_beta+(1|manifest)+cngeneson+pc_mito+brain_region+Sex	0.476	5408.000
ENSG00000165566	0.014	0.432	0.910	-0.045	AMER2	0.049	~amyloid_beta+(1|manifest)+cngeneson+pc_mito+brain_region+Sex	4.449	905.000
ENSG00000126804	0.000	0.432	0.639	0.225	ZBTB1	0.001	~amyloid_beta+(1|manifest)+cngeneson+pc_mito+brain_region+Sex	2.045	2375.000
ENSG00000173715	0.000	0.432	0.724	0.139	C11orf80	0.001	~amyloid_beta+(1|manifest)+cngeneson+pc_mito+brain_region+Sex	0.908	4360.000
ENSG00000144476	0.001	0.431	0.751	0.112	ACKR3	0.013	~amyloid_beta+(1|manifest)+cngeneson+pc_mito+brain_region+Sex	2.195	2207.000
ENSG00000115484	0.001	0.431	0.805	0.057	CCT4	0.008	~amyloid_beta+(1|manifest)+cngeneson+pc_mito+brain_region+Sex	2.558	1849.000
ENSG00000130287	0.028	0.430	0.928	-0.067	NCAN	0.072	~amyloid_beta+(1|manifest)+cngeneson+pc_mito+brain_region+Sex	5.804	624.000
ENSG00000052126	0.379	0.429	1.074	-0.216	PLEKHA5	0.428	~amyloid_beta+(1|manifest)+cngeneson+pc_mito+brain_region+Sex	32.433	19.000
ENSG00000166441	0.000	0.429	0.761	0.098	RPL27A	0.006	~amyloid_beta+(1|manifest)+cngeneson+pc_mito+brain_region+Sex	3.287	1381.000
ENSG00000113013	0.001	0.429	0.725	0.133	HSPA9	0.010	~amyloid_beta+(1|manifest)+cngeneson+pc_mito+brain_region+Sex	0.862	4460.000
ENSG00000116288	0.000	0.428	0.659	0.197	PARK7	0.001	~amyloid_beta+(1|manifest)+cngeneson+pc_mito+brain_region+Sex	0.427	5514.000
ENSG00000031081	0.000	0.428	0.642	0.215	ARHGAP31	0.003	~amyloid_beta+(1|manifest)+cngeneson+pc_mito+brain_region+Sex	1.814	2677.000
ENSG00000171310	0.041	0.428	0.811	0.045	CHST11	0.089	~amyloid_beta+(1|manifest)+cngeneson+pc_mito+brain_region+Sex	1.851	2623.000
ENSG00000071859	0.001	0.427	0.745	0.110	FAM50A	0.008	~amyloid_beta+(1|manifest)+cngeneson+pc_mito+brain_region+Sex	2.629	1789.000
ENSG00000106351	0.001	0.427	0.778	0.077	AGFG2	0.008	~amyloid_beta+(1|manifest)+cngeneson+pc_mito+brain_region+Sex	2.068	2349.000
ENSG00000164188	0.025	0.427	0.727	0.127	RANBP3L	0.067	~amyloid_beta+(1|manifest)+cngeneson+pc_mito+brain_region+Sex	8.712	329.000
ENSG00000188483	0.022	0.424	0.938	-0.089	IER5L	0.062	~amyloid_beta+(1|manifest)+cngeneson+pc_mito+brain_region+Sex	3.662	1182.000
ENSG00000135473	0.000	0.424	0.638	0.210	PAN2	0.001	~amyloid_beta+(1|manifest)+cngeneson+pc_mito+brain_region+Sex	1.718	2798.000
ENSG00000169020	0.038	0.424	0.931	-0.082	ATP5ME	0.085	~amyloid_beta+(1|manifest)+cngeneson+pc_mito+brain_region+Sex	2.064	2353.000
ENSG00000196531	0.010	0.423	0.839	0.006	NACA	0.039	~amyloid_beta+(1|manifest)+cngeneson+pc_mito+brain_region+Sex	3.105	1479.000
ENSG00000102760	0.008	0.422	0.947	-0.103	RGCC	0.036	~amyloid_beta+(1|manifest)+cngeneson+pc_mito+brain_region+Sex	4.131	1004.000
ENSG00000186187	0.000	0.422	0.687	0.156	ZNRF1	0.001	~amyloid_beta+(1|manifest)+cngeneson+pc_mito+brain_region+Sex	0.449	5457.000
ENSG00000104695	0.000	0.421	0.624	0.219	PPP2CB	0.003	~amyloid_beta+(1|manifest)+cngeneson+pc_mito+brain_region+Sex	2.472	1921.000
ENSG00000111371	0.237	0.420	0.972	-0.132	SLC38A1	0.291	~amyloid_beta+(1|manifest)+cngeneson+pc_mito+brain_region+Sex	1.420	3292.000
ENSG00000187954	0.018	0.420	0.855	-0.015	CYHR1	0.056	~amyloid_beta+(1|manifest)+cngeneson+pc_mito+brain_region+Sex	4.346	935.000
ENSG00000008988	0.000	0.420	0.706	0.133	RPS20	0.004	~amyloid_beta+(1|manifest)+cngeneson+pc_mito+brain_region+Sex	2.631	1787.000
ENSG00000175029	0.001	0.419	0.671	0.168	CTBP2	0.011	~amyloid_beta+(1|manifest)+cngeneson+pc_mito+brain_region+Sex	1.468	3189.000
ENSG00000144677	0.014	0.419	0.685	0.153	CTDSPL	0.048	~amyloid_beta+(1|manifest)+cngeneson+pc_mito+brain_region+Sex	7.785	392.000
ENSG00000197982	0.005	0.419	0.802	0.036	C1orf122	0.027	~amyloid_beta+(1|manifest)+cngeneson+pc_mito+brain_region+Sex	2.296	2112.000
ENSG00000167702	0.002	0.419	0.769	0.068	KIFC2	0.017	~amyloid_beta+(1|manifest)+cngeneson+pc_mito+brain_region+Sex	3.721	1155.000
ENSG00000185088	0.001	0.419	0.791	0.046	RPS27L	0.014	~amyloid_beta+(1|manifest)+cngeneson+pc_mito+brain_region+Sex	1.210	3682.000
ENSG00000182310	0.001	0.418	0.788	0.047	SPACA6	0.013	~amyloid_beta+(1|manifest)+cngeneson+pc_mito+brain_region+Sex	1.519	3107.000
ENSG00000148848	0.219	0.418	1.021	-0.185	ADAM12	0.274	~amyloid_beta+(1|manifest)+cngeneson+pc_mito+brain_region+Sex	12.878	153.000
ENSG00000064655	0.016	0.417	0.705	0.129	EYA2	0.053	~amyloid_beta+(1|manifest)+cngeneson+pc_mito+brain_region+Sex	8.274	359.000
ENSG00000182348	0.000	0.417	0.720	0.113	ZNF804B	0.005	~amyloid_beta+(1|manifest)+cngeneson+pc_mito+brain_region+Sex	6.148	572.000
ENSG00000095066	0.002	0.416	0.755	0.077	HOOK2	0.017	~amyloid_beta+(1|manifest)+cngeneson+pc_mito+brain_region+Sex	3.920	1079.000
ENSG00000075420	0.022	0.415	0.745	0.086	FNDC3B	0.062	~amyloid_beta+(1|manifest)+cngeneson+pc_mito+brain_region+Sex	5.697	650.000
ENSG00000175048	0.000	0.415	0.608	0.222	ZDHHC14	0.000	~amyloid_beta+(1|manifest)+cngeneson+pc_mito+brain_region+Sex	0.814	4588.000
ENSG00000151929	0.209	0.412	1.212	-0.387	BAG3	0.264	~amyloid_beta+(1|manifest)+cngeneson+pc_mito+brain_region+Sex	16.204	84.000
ENSG00000137802	0.000	0.412	0.648	0.175	MAPKBP1	0.004	~amyloid_beta+(1|manifest)+cngeneson+pc_mito+brain_region+Sex	1.645	2913.000
ENSG00000006118	0.022	0.411	0.833	-0.011	[illegible]	0.063	~amyloid_beta+(1|manifest)+cngeneson+pc_mito+brain_region+Sex	1.776	2721.000
ENSG00000106624	0.006	0.410	1.112	-0.292	AEBP1	0.029	~amyloid_beta+(1|manifest)+cngeneson+pc_mito+brain_region+Sex	3.280	1383.000
ENSG00000129521	0.177	0.409	1.096	-0.278	EGLN3	0.232	~amyloid_beta+(1|manifest)+cngeneson+pc_mito+brain_region+Sex	14.477	114.000
ENSG00000109971	0.018	0.407	0.949	-0.135	HSPA8	0.056	~amyloid_beta+(1|manifest)+cngeneson+pc_mito+brain_region+Sex	3.437	1285.000
ENSG00000138002	0.000	0.407	0.607	0.207	IFT172	0.001	~amyloid_beta+(1|manifest)+cngeneson+pc_mito+brain_region+Sex	1.797	2704.000
ENSG00000206199	0.000	0.405	0.675	0.136	ANKUB1	0.000	~amyloid_beta+(1|manifest)+cngeneson+pc_mito+brain_region+Sex	1.183	3738.000
ENSG00000090266	0.001	0.405	0.727	0.084	NDUFB2	0.010	~amyloid_beta+(1|manifest)+cngeneson+pc_mito+brain_region+Sex	0.573	5179.000
ENSG00000182899	0.010	0.405	0.874	-0.064	RPL35A	0.040	~amyloid_beta+(1|manifest)+cngeneson+pc_mito+brain_region+Sex	3.805	1118.000
ENSG00000126267	0.001	0.405	0.728	0.081	COX6B1	0.012	~amyloid_beta+(1|manifest)+cngeneson+pc_mito+brain_region+Sex	0.933	4295.000
ENSG00000186350	0.007	0.404	0.793	0.016	RXRA	0.031	~amyloid_beta+(1|manifest)+cngeneson+pc_mito+brain_region+Sex	3.482	1255.000
ENSG00000256463	0.016	0.404	0.792	0.016	SALL3	0.053	~amyloid_beta+(1|manifest)+cngeneson+pc_mito+brain_region+Sex	4.565	872.000
ENSG00000109458	0.013	0.404	0.712	0.096	GAB1	0.047	~amyloid_beta+(1|manifest)+cngeneson+pc_mito+brain_region+Sex	2.948	1576.000
ENSG00000163884	0.002	0.402	0.745	0.060	KLF15	0.015	~amyloid_beta+(1|manifest)+cngeneson+pc_mito+brain_region+Sex	3.289	1378.000
ENSG00000107186	0.001	0.402	0.640	0.164	MPDZ	0.011	~amyloid_beta+(1|manifest)+cngeneson+pc_mito+brain_region+Sex	0.306	5723.000
ENSG00000147853	0.002	0.402	0.619	0.184	AK3	0.015	~amyloid_beta+(1|manifest)+cngeneson+pc_mito+brain_region+Sex	2.052	2364.000
ENSG00000160307	0.031	0.401	0.953	-0.152	S100B	0.076	~amyloid_beta+(1|manifest)+cngeneson+pc_mito+brain_region+Sex	4.593	865.000
ENSG00000148175	0.005	0.398	0.737	0.059	STOM	0.028	~amyloid_beta+(1|manifest)+cngeneson+pc_mito+brain_region+Sex	2.271	2130.000
ENSG00000163468	0.001	0.398	0.750	0.046	CCT3	0.010	~amyloid_beta+(1|manifest)+cngeneson+pc_mito+brain_region+Sex	1.357	3399.000
ENSG00000173020	0.003	0.398	0.732	0.063	GRK2	0.018	~amyloid_beta+(1|manifest)+cngeneson+pc_mito+brain_region+Sex	2.723	1717.000
ENSG00000176978	0.019	0.398	0.837	-0.042	DPP7	0.057	~amyloid_beta+(1|manifest)+cngeneson+pc_mito+brain_region+Sex	6.354	544.000
ENSG00000115286	0.002	0.397	0.736	0.059	NDUFS7	0.018	~amyloid_beta+(1|manifest)+cngeneson+pc_mito+brain_region+Sex	2.334	2064.000
ENSG00000105229	0.000	0.397	0.608	0.186	PIAS4	0.001	~amyloid_beta+(1|manifest)+cngeneson+pc_mito+brain_region+Sex	1.010	4117.000
ENSG00000196218	0.031	0.397	0.967	-0.172	RYR1	0.075	~amyloid_beta+(1|manifest)+cngeneson+pc_mito+brain_region+Sex	7.698	400.000
ENSG00000183864	0.035	0.397	0.824	-0.030	TOB2	0.080	~amyloid_beta+(1|manifest)+cngeneson+pc_mito+brain_region+Sex	5.532	675.000
ENSG00000177119	0.159	0.397	0.851	-0.058	ANO6	0.214	~amyloid_beta+(1|manifest)+cngeneson+pc_mito+brain_region+Sex	11.597	195.000
ENSG00000146122	0.036	0.396	0.806	-0.014	DAAM2	0.082	~amyloid_beta+(1|manifest)+cngeneson+pc_mito+brain_region+Sex	6.264	555.000
ENSG00000153048	0.000	0.396	0.665	0.126	CARHSP1	0.006	~amyloid_beta+(1|manifest)+cngeneson+pc_mito+brain_region+Sex	1.818	2672.000
ENSG00000110237	0.000	0.395	0.650	0.140	ARHGEF17	0.006	~amyloid_beta+(1|manifest)+cngeneson+pc_mito+brain_region+Sex	3.765	1136.000
ENSG00000185432	0.002	0.395	0.642	0.149	METTL7A	0.015	~amyloid_beta+(1|manifest)+cngeneson+pc_mito+brain_region+Sex	2.251	2154.000
ENSG00000092841	0.001	0.394	0.720	0.069	MYL6	0.008	~amyloid_beta+(1|manifest)+cngeneson+pc_mito+brain_region+Sex	2.640	1779.000
ENSG00000134324	0.000	0.393	0.523	0.262	LPIN1	0.000	~amyloid_beta+(1|manifest)+cngeneson+pc_mito+brain_region+Sex	2.282	2125.000
ENSG00000111906	0.000	0.392	0.642	0.142	HDDC2	0.006	~amyloid_beta+(1|manifest)+cngeneson+pc_mito+brain_region+Sex	0.851	4498.000
ENSG00000168309	0.001	0.391	0.571	0.212	FAM107A	0.008	~amyloid_beta+(1|manifest)+cngeneson+pc_mito+brain_region+Sex	2.565	1842.000
ENSG00000141458	0.000	0.390	0.629	0.152	NPC1	0.001	~amyloid_beta+(1|manifest)+cngeneson+pc_mito+brain_region+Sex	3.057	1504.000
ENSG00000138326	0.010	0.390	0.757	0.023	RPS24	0.039	~amyloid_beta+(1|manifest)+cngeneson+pc_mito+brain_region+Sex	3.881	1094.000
ENSG00000172292	0.098	0.390	0.957	-0.177	CERS6	0.153	~amyloid_beta+(1|manifest)+cngeneson+pc_mito+brain_region+Sex	1.762	2734.000
ENSG00000148219	0.006	0.390	0.609	0.171	ASTN2	0.029	~amyloid_beta+(1|manifest)+cngeneson+pc_mito+brain_region+Sex	2.216	2182.000
ENSG00000127946	0.000	0.390	0.595	0.184	HIP1	0.000	~amyloid_beta+(1|manifest)+cngeneson+pc_mito+brain_region+Sex	1.388	3342.000
ENSG00000151090	0.012	0.389	0.671	0.108	THRB	0.045	~amyloid_beta+(1|manifest)+cngeneson+pc_mito+brain_region+Sex	2.794	1672.000
ENSG00000104765	0.000	0.389	0.658	0.120	BNIP3L	0.003	~amyloid_beta+(1|manifest)+cngeneson+pc_mito+brain_region+Sex	0.912	4348.000
ENSG00000134265	0.000	0.386	0.571	0.202	NAPG	0.001	~amyloid_beta+(1|manifest)+cngeneson+pc_mito+brain_region+Sex	0.504	5341.000
ENSG00000111674	0.011	0.385	0.800	-0.030	ENO2	0.043	~amyloid_beta+(1|manifest)+cngeneson+pc_mito+brain_region+Sex	3.167	1444.000
ENSG00000111907	0.000	0.385	0.542	0.227	TPD52L1	0.001	~amyloid_beta+(1|manifest)+cngeneson+pc_mito+brain_region+Sex	0.863	4457.000
ENSG00000165458	0.011	0.385	0.748	0.022	INPPL1	0.042	~amyloid_beta+(1|manifest)+cngeneson+pc_mito+brain_region+Sex	4.073	1023.000
ENSG00000083857	0.000	0.383	0.594	0.173	FAT1	0.001	~amyloid_beta+(1|manifest)+cngeneson+pc_mito+brain_region+Sex	1.421	3291.000
ENSG00000174628	0.002	0.382	0.614	0.151	IQCK	0.017	~amyloid_beta+(1|manifest)+cngeneson+pc_mito+brain_region+Sex	3.080	1493.000
ENSG00000148180	0.011	0.382	0.686	0.077	GSN	0.042	~amyloid_beta+(1|manifest)+cngeneson+pc_mito+brain_region+Sex	6.721	505.000
ENSG00000043355	0.001	0.381	0.658	0.105	ZIC2	0.009	~amyloid_beta+(1|manifest)+cngeneson+pc_mito+brain_region+Sex	0.922	4323.000
ENSG00000096746	0.000	0.381	0.561	0.202	HNRNPH3	0.001	~amyloid_beta+(1|manifest)+cngeneson+pc_mito+brain_region+Sex	0.381	5597.000
ENSG00000004399	0.000	0.379	0.625	0.134	PLXND1	0.001	~amyloid_beta+(1|manifest)+cngeneson+pc_mito+brain_region+Sex	1.038	4048.000
ENSG00000106689	0.009	0.379	0.734	0.024	LHX2	0.039	~amyloid_beta+(1|manifest)+cngeneson+pc_mito+brain_region+Sex	1.665	2881.000
ENSG00000120963	0.000	0.379	0.638	0.119	ZNF706	0.006	~amyloid_beta+(1|manifest)+cngeneson+pc_mito+brain_region+Sex	0.367	5623.000
ENSG00000182175	0.072	0.377	0.808	-0.053	RGMA	0.126	~amyloid_beta+(1|manifest)+cngeneson+pc_mito+brain_region+Sex	6.369	541.000
ENSG00000177301	0.011	0.377	0.779	-0.025	KCNA2	0.043	~amyloid_beta+(1|manifest)+cngeneson+pc_mito+brain_region+Sex	3.214	1416.000
ENSG00000154188	0.391	0.376	1.028	-0.276	ANGPT1	0.440	~amyloid_beta+(1|manifest)+cngeneson+pc_mito+brain_region+Sex	2.881	1615.000
ENSG00000187753	0.000	0.375	0.697	0.054	C9orf153	0.003	~amyloid_beta+(1|manifest)+cngeneson+pc_mito+brain_region+Sex	2.412	1970.000
ENSG00000074800	0.004	0.374	0.650	0.098	ENO1	0.024	~amyloid_beta+(1|manifest)+cngeneson+pc_mito+brain_region+Sex	1.293	3521.000
ENSG00000182809	0.001	0.374	0.633	0.115	CRIP2	0.010	~amyloid_beta+(1|manifest)+cngeneson+pc_mito+brain_region+Sex	2.071	2343.000
ENSG00000122034	0.000	0.373	0.519	0.228	GTF3A	0.000	~amyloid_beta+(1|manifest)+cngeneson+pc_mito+brain_region+Sex	0.474	5412.000
ENSG00000116675	0.005	0.373	0.702	0.044	DNAJC6	0.027	~amyloid_beta+(1|manifest)+cngeneson+pc_mito+brain_region+Sex	7.694	401.000
ENSG00000100376	0.000	0.373	0.631	0.116	FAM118A	0.001	~amyloid_beta+(1|manifest)+cngeneson+pc_mito+brain_region+Sex	3.797	1124.000
ENSG00000105771	0.000	0.371	0.612	0.131	SMG9	0.003	~amyloid_beta+(1|manifest)+cngeneson+pc_mito+brain_region+Sex	0.297	5739.000
ENSG00000177595	0.010	0.371	0.765	-0.024	PIDD1	0.040	~amyloid_beta+(1|manifest)+cngeneson+pc_mito+brain_region+Sex	4.380	922.000
ENSG00000054965	0.000	0.370	0.511	0.230	FAM168A	0.001	~amyloid_beta+(1|manifest)+cngeneson+pc_mito+brain_region+Sex	3.064	1501.000
ENSG00000198756	0.004	0.369	0.696	0.042	COLGALT2	0.022	~amyloid_beta+(1|manifest)+cngeneson+pc_mito+brain_region+Sex	2.203	2200.000
ENSG00000069702	0.004	0.369	0.653	0.085	TGFBR3	0.023	~amyloid_beta+(1|manifest)+cngeneson+pc_mito+brain_region+Sex	4.198	985.000
ENSG00000120063	0.001	0.367	0.604	0.130	GNA13	0.013	~amyloid_beta+(1|manifest)+cngeneson+pc_mito+brain_region+Sex	0.415	5536.000
ENSG00000163346	0.017	0.367	0.715	0.019	PBXIP1	0.054	~amyloid_beta+(1|manifest)+cngeneson+pc_mito+brain_region+Sex	3.466	1268.000
ENSG00000196091	0.042	0.366	0.887	-0.155	MYBPC1	0.089	~amyloid_beta+(1|manifest)+cngeneson+pc_mito+brain_region+Sex	5.951	599.000
ENSG00000068323	0.000	0.366	0.537	0.195	TFE3	0.000	~amyloid_beta+(1|manifest)+cngeneson+pc_mito+brain_region+Sex	0.167	5908.000
ENSG00000114126	0.000	0.365	0.561	0.168	TFDP2	0.001	~amyloid_beta+(1|manifest)+cngeneson+pc_mito+brain_region+Sex	2.696	1733.000
ENSG00000108256	0.001	0.365	0.638	0.092	NUFIP2	0.012	~amyloid_beta+(1|manifest)+cngeneson+pc_mito+brain_region+Sex	2.942	1582.000
ENSG00000101040	0.006	0.364	0.642	0.085	ZMYND8	0.030	~amyloid_beta+(1|manifest)+cngeneson+pc_mito+brain_region+Sex	2.408	1977.000
ENSG00000123095	0.005	0.363	0.708	0.019	BHLHE41	0.027	~amyloid_beta+(1|manifest)+cngeneson+pc_mito+brain_region+Sex	2.069	2346.000
ENSG00000012660	0.003	0.363	0.637	0.089	ELOVL5	0.021	~amyloid_beta+(1|manifest)+cngeneson+pc_mito+brain_region+Sex	1.278	3550.000
ENSG00000168906	0.030	0.363	0.692	0.034	MAT2A	0.074	~amyloid_beta+(1|manifest)+cngeneson+pc_mito+brain_region+Sex	6.049	587.000
ENSG00000115756	0.000	0.363	0.597	0.128	HPCAL1	0.002	~amyloid_beta+(1|manifest)+cngeneson+pc_mito+brain_region+Sex	0.606	5116.000
ENSG00000173805	0.041	0.362	0.940	-0.217	HAP1	0.089	~amyloid_beta+(1|manifest)+cngeneson+pc_mito+brain_region+Sex	11.188	213.000
ENSG00000077942	0.006	0.361	0.733	-0.011	FBLN1	0.029	~amyloid_beta+(1|manifest)+cngeneson+pc_mito+brain_region+Sex	2.844	1641.000
ENSG00000156381	0.049	0.360	0.882	-0.162	ANKRD9	0.099	~amyloid_beta+(1|manifest)+cngeneson+pc_mito+brain_region+Sex	4.474	897.000
ENSG00000175745	0.031	0.360	0.723	-0.004	NR2F1	0.075	~amyloid_beta+(1|manifest)+cngeneson+pc_mito+brain_region+Sex	3.961	1058.000
ENSG00000248643	0.000	0.360	0.571	0.149	[illegible]	0.002	~amyloid_beta+(1|manifest)+cngeneson+pc_mito+brain_region+Sex	0.254	5802.000
ENSG00000175115	0.001	0.360	0.537	0.183	PACS1	0.007	~amyloid_beta+(1|manifest)+cngeneson+pc_mito+brain_region+Sex	2.173	2232.000
ENSG00000130584	0.000	0.360	0.597	0.122	ZBTB46	0.003	~amyloid_beta+(1|manifest)+cngeneson+pc_mito+brain_region+Sex	1.352	3406.000
ENSG00000205517	0.002	0.358	0.677	0.040	RGL3	0.016	~amyloid_beta+(1|manifest)+cngeneson+pc_mito+brain_region+Sex	3.102	1480.000
ENSG00000058668	0.018	0.358	0.604	0.113	ATP2B4	0.056	~amyloid_beta+(1|manifest)+cngeneson+pc_mito+brain_region+Sex	3.290	1376.000
ENSG00000189171	0.010	0.358	0.719	-0.002	S100A13	0.040	~amyloid_beta+(1|manifest)+cngeneson+pc_mito+brain_region+Sex	2.472	1920.000
ENSG00000125257	0.001	0.358	0.621	0.094	ABCC4	0.010	~amyloid_beta+(1|manifest)+cngeneson+pc_mito+brain_region+Sex	1.199	3706.000
ENSG00000158169	0.000	0.357	0.664	0.050	FANCC	0.003	~amyloid_beta+(1|manifest)+cngeneson+pc_mito+brain_region+Sex	0.273	5777.000
ENSG00000139687	0.000	0.356	0.522	0.191	RB1	0.000	~amyloid_beta+(1|manifest)+cngeneson+pc_mito+brain_region+Sex	0.702	4879.000
ENSG00000099797	0.006	0.356	0.708	0.005	TECR	0.030	~amyloid_beta+(1|manifest)+cngeneson+pc_mito+brain_region+Sex	2.771	1690.000
ENSG00000171132	0.000	0.356	0.520	0.191	PRKCE	0.004	~amyloid_beta+(1|manifest)+cngeneson+pc_mito+brain_region+Sex	0.868	4446.000
ENSG00000072110	0.096	0.354	0.829	-0.121	ACTN1	0.151	~amyloid_beta+(1|manifest)+cngeneson+pc_mito+brain_region+Sex	8.387	354.000
ENSG00000175287	0.000	0.353	0.657	0.049	PHYHD1	0.004	~amyloid_beta+(1|manifest)+cngeneson+pc_mito+brain_region+Sex	3.149	1454.000
ENSG00000100263	0.000	0.353	0.554	0.151	RHBDD3	0.003	~amyloid_beta+(1|manifest)+cngeneson+pc_mito+brain_region+Sex	1.349	3416.000
ENSG00000122986	0.000	0.352	0.524	0.179	HVCN1	0.000	~amyloid_beta+(1|manifest)+cngeneson+pc_mito+brain_region+Sex	0.350	5653.000
ENSG00000185340	0.002	0.352	0.633	0.070	GAS2L1	0.015	~amyloid_beta+(1|manifest)+cngeneson+pc_mito+brain_region+Sex	1.929	2523.000
ENSG00000109475	0.007	0.351	0.701	0.001	RPL34	0.033	~amyloid_beta+(1|manifest)+cngeneson+pc_mito+brain_region+Sex	3.051	1509.000
ENSG00000172037	0.001	0.350	0.624	0.077	LAMB2	0.013	~amyloid_beta+(1|manifest)+cngeneson+pc_mito+brain_region+Sex	2.005	2421.000
ENSG00000120709	0.000	0.350	0.529	0.172	FAM53C	0.002	~amyloid_beta+(1|manifest)+cngeneson+pc_mito+brain_region+Sex	1.207	3688.000
ENSG00000123562	0.000	0.350	0.572	0.127	MORF4L2	0.005	~amyloid_beta+(1|manifest)+cngeneson+pc_mito+brain_region+Sex	0.174	5904.000
ENSG00000115183	0.013	0.349	0.707	-0.009	TANC1	0.046	~amyloid_beta+(1|manifest)+cngeneson+pc_mito+brain_region+Sex	5.629	660.000
ENSG00000130985	0.002	0.349	0.616	0.081	UBA1	0.016	~amyloid_beta+(1|manifest)+cngeneson+pc_mito+brain_region+Sex	2.905	1600.000
ENSG00000114796	0.001	0.348	0.544	0.153	KLHL24	0.010	~amyloid_beta+(1|manifest)+cngeneson+pc_mito+brain_region+Sex	1.948	2497.000
ENSG00000077782	0.002	0.348	0.607	0.090	FGFR1	0.015	~amyloid_beta+(1|manifest)+cngeneson+pc_mito+brain_region+Sex	0.703	4875.000
ENSG00000250479	0.001	0.347	0.614	0.081	CHCHD10	0.012	~amyloid_beta+(1|manifest)+cngeneson+pc_mito+brain_region+Sex	1.592	2994.000
ENSG00000142534	0.016	0.347	0.767	-0.074	RPS11	0.053	~amyloid_beta+(1|manifest)+cngeneson+pc_mito+brain_region+Sex	7.322	438.000
ENSG00000099331	0.000	0.346	0.510	0.182	MYO9B	0.002	~amyloid_beta+(1|manifest)+cngeneson+pc_mito+brain_region+Sex	1.381	3356.000
ENSG00000151746	0.004	0.346	0.545	0.147	BICD1	0.023	~amyloid_beta+(1|manifest)+cngeneson+pc_mito+brain_region+Sex	7.029	466.000
ENSG00000168264	0.006	0.346	0.652	0.040	IRF2BP2	0.028	~amyloid_beta+(1|manifest)+cngeneson+pc_mito+brain_region+Sex	2.052	2367.000
ENSG00000011295	0.002	0.346	0.598	0.094	TTC19	0.017	~amyloid_beta+(1|manifest)+cngeneson+pc_mito+brain_region+Sex	3.959	1060.000
ENSG00000136002	0.000	0.345	0.505	0.185	ARHGEF4	0.007	~amyloid_beta+(1|manifest)+cngeneson+pc_mito+brain_region+Sex	5.841	618.000
ENSG00000139668	0.000	0.345	0.481	0.209	WDFY2	0.002	~amyloid_beta+(1|manifest)+cngeneson+pc_mito+brain_region+Sex	0.274	5775.000
ENSG00000106780	0.000	0.345	0.519	0.170	MEGF9	0.005	~amyloid_beta+(1|manifest)+cngeneson+pc_mito+brain_region+Sex	1.551	3049.000
ENSG00000177169	0.000	0.345	0.528	0.161	ULK1	0.001	~amyloid_beta+(1|manifest)+cngeneson+pc_mito+brain_region+Sex	1.609	2967.000
ENSG00000169826	0.000	0.343	0.557	0.130	[illegible]	0.002	~amyloid_beta+(1|manifest)+cngeneson+pc_mito+brain_region+Sex	2.248	2158.000
ENSG00000107521	0.000	0.343	0.533	0.153	HPS1	0.001	~amyloid_beta+(1|manifest)+cngeneson+pc_mito+brain_region+Sex	0.946	4262.000
ENSG00000126368	0.007	0.342	0.692	-0.008	NR1D1	0.033	~amyloid_beta+(1|manifest)+cngeneson+pc_mito+brain_region+Sex	2.197	2206.000
ENSG00000144713	0.012	0.342	0.781	-0.097	RPL32	0.044	~amyloid_beta+(1|manifest)+cngeneson+pc_mito+brain_region+Sex	4.956	786.000
ENSG00000072778	0.067	0.341	0.767	-0.085	ACADVL	0.120	~amyloid_beta+(1|manifest)+cngeneson+pc_mito+brain_region+Sex	18.405	64.000
ENSG00000173542	0.002	0.341	0.614	0.068	MOB1B	0.017	~amyloid_beta+(1|manifest)+cngeneson+pc_mito+brain_region+Sex	0.359	5636.000
ENSG00000080573	0.150	0.339	0.719	-0.041	COL5A3	0.205	~amyloid_beta+(1|manifest)+cngeneson+pc_mito+brain_region+Sex	5.900	609.000
ENSG00000101400	0.148	0.338	0.894	-0.217	SNTA1	0.204	~amyloid_beta+(1|manifest)+cngeneson+pc_mito+brain_region+Sex	9.523	297.000
ENSG00000140090	0.052	0.338	0.769	-0.094	SLC24A4	0.102	~amyloid_beta+(1|manifest)+cngeneson+pc_mito+brain_region+Sex	5.454	684.000
ENSG00000196730	0.022	0.337	0.597	0.077	DAPK1	0.063	~amyloid_beta+(1|manifest)+cngeneson+pc_mito+brain_region+Sex	2.402	1979.000
ENSG00000107957	0.000	0.337	0.596	0.078	SH3PXD2A	0.001	~amyloid_beta+(1|manifest)+cngeneson+pc_mito+brain_region+Sex	1.217	3669.000
ENSG00000130175	0.000	0.337	0.576	0.098	PRKCSH	0.006	~amyloid_beta+(1|manifest)+cngeneson+pc_mito+brain_region+Sex	1.152	3809.000
ENSG00000117425	0.006	0.336	0.676	-0.004	PTCH2	0.029	~amyloid_beta+(1|manifest)+cngeneson+pc_mito+brain_region+Sex	3.521	1239.000
ENSG00000104450	0.001	0.336	0.616	0.057	SPAG1	0.007	~amyloid_beta+(1|manifest)+cngeneson+pc_mito+brain_region+Sex	1.004	4130.000
ENSG00000090060	0.032	0.336	0.732	-0.060	PAPOLA	0.077	~amyloid_beta+(1|manifest)+cngeneson+pc_mito+brain_region+Sex	2.450	1936.000
ENSG00000184207	0.004	0.336	0.663	0.009	PGP	0.024	~amyloid_beta+(1|manifest)+cngeneson+pc_mito+brain_region+Sex	3.391	1322.000
ENSG00000113721	0.070	0.336	0.815	-0.143	PDGFRB	0.123	~amyloid_beta+(1|manifest)+cngeneson+pc_mito+brain_region+Sex	10.072	267.000
ENSG00000187531	0.000	0.336	0.515	0.156	SIRT7	0.002	~amyloid_beta+(1|manifest)+cngeneson+pc_mito+brain_region+Sex	1.651	2903.000
ENSG00000050767	0.001	0.335	0.636	0.034	COL23A1	0.010	~amyloid_beta+(1|manifest)+cngeneson+pc_mito+brain_region+Sex	1.826	2658.000
ENSG00000142657	0.000	0.335	0.475	0.196	PGD	0.000	~amyloid_beta+(1|manifest)+cngeneson+pc_mito+brain_region+Sex	0.638	5034.000
ENSG00000133612	0.000	0.335	0.512	0.158	AGAP3	0.004	~amyloid_beta+(1|manifest)+cngeneson+pc_mito+brain_region+Sex	1.286	3532.000
ENSG00000104859	0.000	0.335	0.459	0.211	CLASRP	0.000	~amyloid_beta+(1|manifest)+cngeneson+pc_mito+brain_region+Sex	1.269	3564.000
ENSG00000100979	0.026	0.335	0.710	-0.041	PLTP	0.068	~amyloid_beta+(1|manifest)+cngeneson+pc_mito+brain_region+Sex	1.483	3171.000
ENSG00000188243	0.003	0.334	0.696	-0.028	COMMD6	0.020	~amyloid_beta+(1|manifest)+cngeneson+pc_mito+brain_region+Sex	1.526	3093.000
ENSG00000110328	0.016	0.334	0.623	0.045	GALNT18	0.052	~amyloid_beta+(1|manifest)+cngeneson+pc_mito+brain_region+Sex	2.435	1949.000
ENSG00000198171	0.019	0.333	0.756	-0.089	DDRGK1	0.057	~amyloid_beta+(1|manifest)+cngeneson+pc_mito+brain_region+Sex	3.472	1262.000
ENSG00000164823	0.001	0.333	0.561	0.104	OSGIN2	0.008	~amyloid_beta+(1|manifest)+cngeneson+pc_mito+brain_region+Sex	1.547	3057.000
ENSG00000168003	0.085	0.332	0.782	-0.117	SLC3A2	0.140	~amyloid_beta+(1|manifest)+cngeneson+pc_mito+brain_region+Sex	5.734	640.000
ENSG00000114805	0.009	0.332	0.600	0.065	PLCH1	0.037	~amyloid_beta+(1|manifest)+cngeneson+pc_mito+brain_region+Sex	0.904	4369.000
ENSG00000164880	0.004	0.331	0.616	0.046	INTS1	0.023	~amyloid_beta+(1|manifest)+cngeneson+pc_mito+brain_region+Sex	3.375	1333.000
ENSG00000118046	0.000	0.331	0.522	0.139	STK11	0.002	~amyloid_beta+(1|manifest)+cngeneson+pc_mito+brain_region+Sex	1.567	3028.000
ENSG00000130294	0.003	0.331	0.583	0.078	KIF1A	0.021	~amyloid_beta+(1|manifest)+cngeneson+pc_mito+brain_region+Sex	1.817	2674.000
ENSG00000151012	0.087	0.330	0.624	0.037	SLC7A11	0.142	~amyloid_beta+(1|manifest)+cngeneson+pc_mito+brain_region+Sex	18.579	60.000
ENSG00000082014	0.008	0.330	0.674	-0.014	SMARCD3	0.036	~amyloid_beta+(1|manifest)+cngeneson+pc_mito+brain_region+Sex	3.678	1174.000
ENSG00000090273	0.000	0.330	0.533	0.126	NUDC	0.001	~amyloid_beta+(1|manifest)+cngeneson+pc_mito+brain_region+Sex	0.315	5703.000
ENSG00000198039	0.001	0.329	0.621	0.036	ZNF273	0.013	~amyloid_beta+(1|manifest)+cngeneson+pc_mito+brain_region+Sex	2.331	2070.000
ENSG00000145819	0.000	0.328	0.479	0.178	ARHGAP26	0.007	~amyloid_beta+(1|manifest)+cngeneson+pc_mito+brain_region+Sex	7.509	416.000
ENSG00000088881	0.000	0.328	0.547	0.109	EBF4	0.005	~amyloid_beta+(1|manifest)+cngeneson+pc_mito+brain_region+Sex	0.724	4811.000
ENSG00000118181	0.040	0.328	0.883	-0.227	RPS25	0.088	~amyloid_beta+(1|manifest)+cngeneson+pc_mito+brain_region+Sex	4.578	870.000
ENSG00000105698	0.002	0.328	0.556	0.100	USF2	0.016	~amyloid_beta+(1|manifest)+cngeneson+pc_mito+brain_region+Sex	1.908	2545.000
ENSG00000163644	0.027	0.328	0.681	-0.025	PPM1K	0.069	~amyloid_beta+(1|manifest)+cngeneson+pc_mito+brain_region+Sex	2.587	1822.000
ENSG00000144711	0.001	0.327	0.580	0.074	IQSEC1	0.013	~amyloid_beta+(1|manifest)+cngeneson+pc_mito+brain_region+Sex	1.743	2761.000
ENSG00000102786	0.000	0.327	0.504	0.149	INTS6	0.002	~amyloid_beta+(1|manifest)+cngeneson+pc_mito+brain_region+Sex	0.888	4403.000
ENSG00000156642	0.009	0.326	0.607	0.044	NPTN	0.037	~amyloid_beta+(1|manifest)+cngeneson+pc_mito+brain_region+Sex	0.969	4207.000
ENSG00000160007	0.001	0.323	0.557	0.089	ARHGAP35	0.007	~amyloid_beta+(1|manifest)+cngeneson+pc_mito+brain_region+Sex	0.514	5321.000
ENSG00000168209	0.477	0.322	0.990	-0.345	DDIT4	0.522	~amyloid_beta+(1|manifest)+cngeneson+pc_mito+brain_region+Sex	10.571	244.000
ENSG00000156973	0.000	0.322	0.481	0.164	PDE6D	0.001	~amyloid_beta+(1|manifest)+cngeneson+pc_mito+brain_region+Sex	0.462	5436.000
ENSG00000174720	0.000	0.322	0.494	0.151	LARP7	0.002	~amyloid_beta+(1|manifest)+cngeneson+pc_mito+brain_region+Sex	0.437	5490.000
ENSG00000182095	0.000	0.321	0.488	0.153	TNRC18	0.002	~amyloid_beta+(1|manifest)+cngeneson+pc_mito+brain_region+Sex	1.194	3720.000
ENSG00000141756	0.010	0.319	0.662	-0.023	FKBP10	0.039	~amyloid_beta+(1|manifest)+cngeneson+pc_mito+brain_region+Sex	3.941	1066.000
ENSG00000168874	0.000	0.319	0.545	0.094	ATOH8	0.001	~amyloid_beta+(1|manifest)+cngeneson+pc_mito+brain_region+Sex	0.968	4210.000
ENSG00000163320	0.008	0.319	0.582	0.056	CGGBP1	0.034	~amyloid_beta+(1|manifest)+cngeneson+pc_mito+brain_region+Sex	0.583	5164.000
ENSG00000107864	0.018	0.319	0.557	0.081	CPEB3	0.055	~amyloid_beta+(1|manifest)+cngeneson+pc_mito+brain_region+Sex	3.461	1270.000
ENSG00000092820	0.020	0.319	0.601	0.037	EZR	0.060	~amyloid_beta+(1|manifest)+cngeneson+pc_mito+brain_region+Sex	1.251	3599.000
ENSG00000156411	0.004	0.319	0.601	0.038	ATP5MPL	0.022	~amyloid_beta+(1|manifest)+cngeneson+pc_mito+brain_region+Sex	1.743	2760.000
ENSG00000161203	0.003	0.319	0.606	0.032	AP2M1	0.021	~amyloid_beta+(1|manifest)+cngeneson+pc_mito+brain_region+Sex	0.724	4809.000
ENSG00000149091	0.100	0.318	0.763	-0.126	DGKZ	0.155	~amyloid_beta+(1|manifest)+cngeneson+pc_mito+brain_region+Sex	4.429	907.000

ENSG00000165895	0.024	0.317	0.634	0.000	ARHGAP42	0.065	~amyloid_beta+(1|manifest)+cngeneson+pc_mito+brain_region+Sex	1.585	3004.000
ENSG00000168610	0.011	0.317	0.604	0.030	STAT3	0.043	~amyloid_beta+(1|manifest)+cngeneson+pc_mito+brain_region+Sex	4.538	885.000
ENSG00000113719	0.000	0.316	0.447	0.185	ERGIC1	0.000	~amyloid_beta+(1|manifest)+cngeneson+pc_mito+brain_region+Sex	0.822	4563.000
ENSG00000182103	0.023	0.316	0.686	-0.053	FAM181B	0.064	~amyloid_beta+(1|manifest)+cngeneson+pc_mito+brain_region+Sex	2.880	1616.000
ENSG00000181619	0.000	0.316	0.506	0.127	GPR135	0.002	~amyloid_beta+(1|manifest)+cngeneson+pc_mito+brain_region+Sex	0.649	5001.000
ENSG00000135916	0.291	0.316	0.824	-0.192	ITM2C	0.343	~amyloid_beta+(1|manifest)+cngeneson+pc_mito+brain_region+Sex	12.018	174.000
ENSG00000168301	0.001	0.315	0.533	0.098	KCTD6	0.009	~amyloid_beta+(1|manifest)+cngeneson+pc_mito+brain_region+Sex	0.555	5222.000
ENSG00000187266	0.005	0.315	0.661	-0.030	EPOR	0.027	~amyloid_beta+(1|manifest)+cngeneson+pc_mito+brain_region+Sex	2.747	1708.000
ENSG00000214655	0.019	0.315	0.631	-0.001	ZSWIM8	0.058	~amyloid_beta+(1|manifest)+cngeneson+pc_mito+brain_region+Sex	4.530	887.000
ENSG00000103024	0.188	0.315	0.882	-0.253	NME3	0.243	~amyloid_beta+(1|manifest)+cngeneson+pc_mito+brain_region+Sex	5.790	626.000
ENSG00000130821	0.000	0.315	0.480	0.149	SLC6A8	0.002	~amyloid_beta+(1|manifest)+cngeneson+pc_mito+brain_region+Sex	1.352	3409.000
ENSG00000162944	0.084	0.314	0.650	-0.022	RFTN2	0.138	~amyloid_beta+(1|manifest)+cngeneson+pc_mito+brain_region+Sex	7.708	399.000
ENSG00000007372	0.004	0.314	0.554	0.074	PAX6	0.022	~amyloid_beta+(1|manifest)+cngeneson+pc_mito+brain_region+Sex	1.691	2839.000
ENSG00000111961	0.000	0.313	0.470	0.157	SASH1	0.003	~amyloid_beta+(1|manifest)+cngeneson+pc_mito+brain_region+Sex	5.238	717.000
ENSG00000107779	0.004	0.313	0.478	0.148	BMPR1A	0.023	~amyloid_beta+(1|manifest)+cngeneson+pc_mito+brain_region+Sex	8.484	349.000
ENSG00000134569	0.094	0.313	0.615	0.011	LRP4	0.149	~amyloid_beta+(1|manifest)+cngeneson+pc_mito+brain_region+Sex	11.449	202.000
ENSG00000183943	0.001	0.313	0.591	0.035	PRKX	0.009	~amyloid_beta+(1|manifest)+cngeneson+pc_mito+brain_region+Sex	1.358	3396.000
ENSG00000170234	0.000	0.313	0.493	0.132	PWWP2A	0.003	~amyloid_beta+(1|manifest)+cngeneson+pc_mito+brain_region+Sex	0.527	5292.000
ENSG00000115904	0.003	0.312	0.507	0.116	SOS1	0.019	~amyloid_beta+(1|manifest)+cngeneson+pc_mito+brain_region+Sex	0.392	5576.000
ENSG00000104218	0.000	0.311	0.448	0.175	CSPP1	0.000	~amyloid_beta+(1|manifest)+cngeneson+pc_mito+brain_region+Sex	1.043	4044.000
ENSG00000103365	0.000	0.311	0.491	0.132	GGA2	0.005	~amyloid_beta+(1|manifest)+cngeneson+pc_mito+brain_region+Sex	0.569	5187.000
ENSG00000167972	0.001	0.311	0.634	-0.011	ABCA3	0.013	~amyloid_beta+(1|manifest)+cngeneson+pc_mito+brain_region+Sex	2.452	1935.000
ENSG00000168273	0.000	0.311	0.493	0.130	SMIM4	0.004	~amyloid_beta+(1|manifest)+cngeneson+pc_mito+brain_region+Sex	0.903	4371.000
ENSG00000131711	0.152	0.311	0.808	-0.186	MAP1B	0.207	~amyloid_beta+(1|manifest)+cngeneson+pc_mito+brain_region+Sex	5.167	736.000
ENSG00000083642	0.002	0.311	0.530	0.091	PDS5B	0.015	~amyloid_beta+(1|manifest)+cngeneson+pc_mito+brain_region+Sex	0.566	5196.000
ENSG00000065518	0.024	0.311	0.731	-0.110	NDUFB4	0.066	~amyloid_beta+(1|manifest)+cngeneson+pc_mito+brain_region+Sex	1.780	2716.000
ENSG00000035115	0.000	0.310	0.478	0.141	SH3YL1	0.001	~amyloid_beta+(1|manifest)+cngeneson+pc_mito+brain_region+Sex	1.026	4086.000
ENSG00000167553	0.001	0.309	0.552	0.066	TUBA1C	0.010	~amyloid_beta+(1|manifest)+cngeneson+pc_mito+brain_region+Sex	1.530	3086.000
ENSG00000111275	0.019	0.308	0.652	-0.036	ALDH2	0.058	~amyloid_beta+(1|manifest)+cngeneson+pc_mito+brain_region+Sex	3.393	1321.000
ENSG00000143157	0.000	0.308	0.468	0.147	POGK	0.001	~amyloid_beta+(1|manifest)+cngeneson+pc_mito+brain_region+Sex	0.131	5932.000
ENSG00000164106	0.022	0.308	0.711	-0.095	SCRG1	0.063	~amyloid_beta+(1|manifest)+cngeneson+pc_mito+brain_region+Sex	2.543	1864.000
ENSG00000180011	0.000	0.308	0.447	0.168	ZADH2	0.000	~amyloid_beta+(1|manifest)+cngeneson+pc_mito+brain_region+Sex	0.628	5065.000
ENSG00000118515	0.091	0.307	0.776	-0.162	SGK1	0.146	~amyloid_beta+(1|manifest)+cngeneson+pc_mito+brain_region+Sex	4.674	848.000
ENSG00000140526	0.015	0.307	0.591	0.023	ABHD2	0.050	~amyloid_beta+(1|manifest)+cngeneson+pc_mito+brain_region+Sex	0.799	4635.000
ENSG00000182154	0.080	0.306	0.829	-0.216	MRPL41	0.135	~amyloid_beta+(1|manifest)+cngeneson+pc_mito+brain_region+Sex	15.840	91.000
ENSG00000163956	0.001	0.306	0.551	0.061	LRPAP1	0.011	~amyloid_beta+(1|manifest)+cngeneson+pc_mito+brain_region+Sex	0.781	4663.000
ENSG00000162804	0.074	0.306	0.915	-0.303	SNED1	0.128	~amyloid_beta+(1|manifest)+cngeneson+pc_mito+brain_region+Sex	7.218	450.000
ENSG00000049540	0.024	0.306	0.593	0.019	ELN	0.066	~amyloid_beta+(1|manifest)+cngeneson+pc_mito+brain_region+Sex	1.801	2695.000
ENSG00000187105	0.001	0.305	0.574	0.036	HEATR4	0.011	~amyloid_beta+(1|manifest)+cngeneson+pc_mito+brain_region+Sex	3.017	1532.000
ENSG00000119705	0.000	0.304	0.491	0.118	SLIRP	0.006	~amyloid_beta+(1|manifest)+cngeneson+pc_mito+brain_region+Sex	0.712	4838.000
ENSG00000204634	0.000	0.304	0.451	0.158	TBC1D8	0.000	~amyloid_beta+(1|manifest)+cngeneson+pc_mito+brain_region+Sex	0.603	5121.000
ENSG00000167191	0.006	0.304	0.476	0.132	GPRC5B	0.030	~amyloid_beta+(1|manifest)+cngeneson+pc_mito+brain_region+Sex	5.832	621.000
ENSG00000090615	0.000	0.304	0.446	0.162	GOLGA3	0.001	~amyloid_beta+(1|manifest)+cngeneson+pc_mito+brain_region+Sex	0.376	5604.000
ENSG00000196576	0.016	0.304	0.589	0.019	PLXNB2	0.053	~amyloid_beta+(1|manifest)+cngeneson+pc_mito+brain_region+Sex	1.801	2694.000
ENSG00000167193	0.000	0.302	0.406	0.198	CRK	0.000	~amyloid_beta+(1|manifest)+cngeneson+pc_mito+brain_region+Sex	0.252	5806.000
ENSG00000034677	0.107	0.302	0.597	0.006	RNF19A	0.163	~amyloid_beta+(1|manifest)+cngeneson+pc_mito+brain_region+Sex	6.480	528.000
ENSG00000146648	0.031	0.302	0.535	0.068	EGFR	0.076	~amyloid_beta+(1|manifest)+cngeneson+pc_mito+brain_region+Sex	2.647	1773.000
ENSG00000143198	0.000	0.302	0.409	0.194	MGST3	0.000	~amyloid_beta+(1|manifest)+cngeneson+pc_mito+brain_region+Sex	0.730	4794.000
ENSG00000123349	0.008	0.301	0.606	-0.003	PFDN5	0.034	~amyloid_beta+(1|manifest)+cngeneson+pc_mito+brain_region+Sex	4.303	949.000
ENSG00000088387	0.000	0.301	0.495	0.108	DOCK9	0.001	~amyloid_beta+(1|manifest)+cngeneson+pc_mito+brain_region+Sex	1.553	3044.000
ENSG00000125691	0.002	0.301	0.546	0.056	RPL23	0.016	~amyloid_beta+(1|manifest)+cngeneson+pc_mito+brain_region+Sex	2.394	1993.000
ENSG00000164877	0.082	0.301	0.661	-0.060	MICALL2	0.137	~amyloid_beta+(1|manifest)+cngeneson+pc_mito+brain_region+Sex	10.520	246.000
ENSG00000056998	0.023	0.300	0.729	-0.129	GYG2	0.063	~amyloid_beta+(1|manifest)+cngeneson+pc_mito+brain_region+Sex	4.471	899.000
ENSG00000107738	0.031	0.299	0.691	-0.092	VSIR	0.076	~amyloid_beta+(1|manifest)+cngeneson+pc_mito+brain_region+Sex	2.855	1631.000
ENSG00000100664	0.006	0.299	0.544	0.054	EIF5	0.029	~amyloid_beta+(1|manifest)+cngeneson+pc_mito+brain_region+Sex	0.417	5533.000
ENSG00000099864	0.038	0.299	0.658	-0.060	PALM	0.086	~amyloid_beta+(1|manifest)+cngeneson+pc_mito+brain_region+Sex	9.587	289.000
ENSG00000184076	0.021	0.299	0.718	-0.121	UQCR10	0.062	~amyloid_beta+(1|manifest)+cngeneson+pc_mito+brain_region+Sex	1.183	3740.000
ENSG00000068903	0.003	0.298	0.529	0.068	SIRT2	0.021	~amyloid_beta+(1|manifest)+cngeneson+pc_mito+brain_region+Sex	2.764	1695.000
ENSG00000103769	0.026	0.298	0.616	-0.020	RAB11A	0.068	~amyloid_beta+(1|manifest)+cngeneson+pc_mito+brain_region+Sex	3.252	1394.000
ENSG00000005893	0.003	0.298	0.532	0.064	LAMP2	0.020	~amyloid_beta+(1|manifest)+cngeneson+pc_mito+brain_region+Sex	1.690	2844.000
ENSG00000157985	0.001	0.297	0.470	0.124	AGAP1	0.010	~amyloid_beta+(1|manifest)+cngeneson+pc_mito+brain_region+Sex	0.376	5605.000
ENSG00000074696	0.002	0.297	0.471	0.123	HACD3	0.014	~amyloid_beta+(1|manifest)+cngeneson+pc_mito+brain_region+Sex	1.192	3722.000
ENSG00000100401	0.000	0.297	0.497	0.097	RANGAP1	0.003	~amyloid_beta+(1|manifest)+cngeneson+pc_mito+brain_region+Sex	0.839	4520.000
ENSG00000113732	0.000	0.296	0.453	0.140	ATP6V0E1	0.000	~amyloid_beta+(1|manifest)+cngeneson+pc_mito+brain_region+Sex	0.368	5622.000
ENSG00000096060	0.072	0.296	0.609	-0.017	FKBP5	0.126	~amyloid_beta+(1|manifest)+cngeneson+pc_mito+brain_region+Sex	2.936	1584.000
ENSG00000145088	0.177	0.295	0.723	-0.132	EAF2	0.231	~amyloid_beta+(1|manifest)+cngeneson+pc_mito+brain_region+Sex	5.943	600.000
ENSG00000120451	0.001	0.295	0.654	-0.063	SNX19	0.007	~amyloid_beta+(1|manifest)+cngeneson+pc_mito+brain_region+Sex	4.898	798.000
ENSG00000174944	0.022	0.295	0.700	-0.109	P2RY14	0.063	~amyloid_beta+(1|manifest)+cngeneson+pc_mito+brain_region+Sex	3.101	1481.000
ENSG00000172757	0.000	0.295	0.481	0.109	CFL1	0.002	~amyloid_beta+(1|manifest)+cngeneson+pc_mito+brain_region+Sex	0.935	4288.000
ENSG00000103319	0.038	0.294	0.663	-0.074	EEF2K	0.085	~amyloid_beta+(1|manifest)+cngeneson+pc_mito+brain_region+Sex	7.286	443.000
ENSG00000170871	0.001	0.294	0.509	0.078	KIAA0232	0.008	~amyloid_beta+(1|manifest)+cngeneson+pc_mito+brain_region+Sex	1.304	3499.000
ENSG00000126934	0.001	0.293	0.484	0.102	MAP2K2	0.008	~amyloid_beta+(1|manifest)+cngeneson+pc_mito+brain_region+Sex	1.419	3295.000
ENSG00000162244	0.021	0.293	0.698	-0.112	RPL29	0.061	~amyloid_beta+(1|manifest)+cngeneson+pc_mito+brain_region+Sex	4.794	826.000
ENSG00000196262	0.000	0.293	0.481	0.104	PPIA	0.002	~amyloid_beta+(1|manifest)+cngeneson+pc_mito+brain_region+Sex	0.737	4779.000
ENSG00000107223	0.001	0.292	0.542	0.043	EDF1	0.010	~amyloid_beta+(1|manifest)+cngeneson+pc_mito+brain_region+Sex	1.638	2925.000
ENSG00000131174	0.007	0.292	0.655	-0.071	COX7B	0.032	~amyloid_beta+(1|manifest)+cngeneson+pc_mito+brain_region+Sex	0.994	4155.000
ENSG00000100207	0.000	0.292	0.428	0.156	TCF20	0.000	~amyloid_beta+(1|manifest)+cngeneson+pc_mito+brain_region+Sex	0.190	5888.000
ENSG00000117643	0.524	0.292	1.020	-0.436	MAN1C1	0.566	~amyloid_beta+(1|manifest)+cngeneson+pc_mito+brain_region+Sex	24.372	32.000
ENSG00000173517	0.006	0.292	0.495	0.088	PEAK1	0.029	~amyloid_beta+(1|manifest)+cngeneson+pc_mito+brain_region+Sex	3.969	1052.000
ENSG00000141576	0.000	0.291	0.465	0.118	RNF157	0.003	~amyloid_beta+(1|manifest)+cngeneson+pc_mito+brain_region+Sex	0.435	5498.000
ENSG00000101079	0.000	0.291	0.420	0.162	NDRG3	0.000	~amyloid_beta+(1|manifest)+cngeneson+pc_mito+brain_region+Sex	0.439	5487.000
ENSG00000141519	0.000	0.291	0.474	0.108	CCDC40	0.004	~amyloid_beta+(1|manifest)+cngeneson+pc_mito+brain_region+Sex	1.328	3448.000
ENSG00000099910	0.000	0.289	0.474	0.104	KLHL22	0.004	~amyloid_beta+(1|manifest)+cngeneson+pc_mito+brain_region+Sex	0.854	4489.000
ENSG00000169564	0.005	0.288	0.571	0.005	PCBP1	0.025	~amyloid_beta+(1|manifest)+cngeneson+pc_mito+brain_region+Sex	1.843	2637.000
ENSG00000074416	0.114	0.288	0.592	-0.016	MGLL	0.170	~amyloid_beta+(1|manifest)+cngeneson+pc_mito+brain_region+Sex	1.954	2488.000
ENSG00000170345	0.336	0.288	1.197	-0.621	FOS	0.387	~amyloid_beta+(1|manifest)+cngeneson+pc_mito+brain_region+Sex	5.024	770.000
ENSG00000197530	0.018	0.288	0.635	-0.060	MIB2	0.055	~amyloid_beta+(1|manifest)+cngeneson+pc_mito+brain_region+Sex	3.931	1076.000
ENSG00000167216	0.012	0.288	0.544	0.031	KATNAL2	0.044	~amyloid_beta+(1|manifest)+cngeneson+pc_mito+brain_region+Sex	1.485	3168.000
ENSG00000198837	0.000	0.286	0.463	0.109	DENND4B	0.004	~amyloid_beta+(1|manifest)+cngeneson+pc_mito+brain_region+Sex	1.048	4031.000
ENSG00000239521	0.023	0.286	0.683	-0.112	CASTOR3	0.065	~amyloid_beta+(1|manifest)+cngeneson+pc_mito+brain_region+Sex	2.161	2248.000
ENSG00000120896	0.014	0.286	0.582	-0.011	SORBS3	0.048	~amyloid_beta+(1|manifest)+cngeneson+pc_mito+brain_region+Sex	2.418	1963.000
ENSG00000145687	0.009	0.285	0.456	0.115	SSBP2	0.038	~amyloid_beta+(1|manifest)+cngeneson+pc_mito+brain_region+Sex	1.796	2707.000
ENSG00000130827	0.003	0.284	0.542	0.025	PLXNA3	0.021	~amyloid_beta+(1|manifest)+cngeneson+pc_mito+brain_region+Sex	1.755	2743.000
ENSG00000179981	0.041	0.284	0.679	-0.112	TSHZ1	0.089	~amyloid_beta+(1|manifest)+cngeneson+pc_mito+brain_region+Sex	3.970	1051.000
ENSG00000126351	0.001	0.283	0.484	0.082	THRA	0.007	~amyloid_beta+(1|manifest)+cngeneson+pc_mito+brain_region+Sex	2.033	2391.000
ENSG00000106153	0.005	0.283	0.521	0.046	CHCHD2	0.028	~amyloid_beta+(1|manifest)+cngeneson+pc_mito+brain_region+Sex	2.796	1669.000
ENSG00000012822	0.001	0.283	0.468	0.098	CALCOCO1	0.010	~amyloid_beta+(1|manifest)+cngeneson+pc_mito+brain_region+Sex	1.656	2895.000
ENSG00000137171	0.000	0.283	0.447	0.118	KLC4	0.003	~amyloid_beta+(1|manifest)+cngeneson+pc_mito+brain_region+Sex	1.524	3097.000
ENSG00000169504	0.704	0.283	1.022	-0.457	CLIC4	0.733	~amyloid_beta+(1|manifest)+cngeneson+pc_mito+brain_region+Sex	17.559	74.000
ENSG00000150764	0.000	0.282	0.493	0.071	DIXDC1	0.001	~amyloid_beta+(1|manifest)+cngeneson+pc_mito+brain_region+Sex	1.086	3957.000
ENSG00000104290	0.005	0.282	0.545	0.019	FZD3	0.028	~amyloid_beta+(1|manifest)+cngeneson+pc_mito+brain_region+Sex	1.503	3134.000
ENSG00000172932	0.001	0.282	0.453	0.111	ANKRD13D	0.011	~amyloid_beta+(1|manifest)+cngeneson+pc_mito+brain_region+Sex	1.974	2463.000
ENSG00000105618	0.001	0.282	0.480	0.084	PRPF31	0.009	~amyloid_beta+(1|manifest)+cngeneson+pc_mito+brain_region+Sex	1.548	3052.000
ENSG00000117174	0.000	0.282	0.439	0.124	ZNHIT6	0.003	~amyloid_beta+(1|manifest)+cngeneson+pc_mito+brain_region+Sex	0.234	5832.000
ENSG00000149557	0.000	0.281	0.427	0.136	FEZ1	0.001	~amyloid_beta+(1|manifest)+cngeneson+pc_mito+brain_region+Sex	0.699	4891.000
ENSG00000141556	0.000	0.281	0.496	0.066	TBCD	0.004	~amyloid_beta+(1|manifest)+cngeneson+pc_mito+brain_region+Sex	0.332	5669.000
ENSG00000084112	0.000	0.281	0.427	0.134	SSH1	0.001	~amyloid_beta+(1|manifest)+cngeneson+pc_mito+brain_region+Sex	0.709	4852.000
ENSG00000038427	0.148	0.281	0.937	-0.375	VCAN	0.203	~amyloid_beta+(1|manifest)+cngeneson+pc_mito+brain_region+Sex	6.937	475.000
ENSG00000189079	0.000	0.280	0.412	0.149	ARID2	0.001	~amyloid_beta+(1|manifest)+cngeneson+pc_mito+brain_region+Sex	1.601	2983.000
ENSG00000164543	0.000	0.280	0.467	0.093	STK17A	0.003	~amyloid_beta+(1|manifest)+cngeneson+pc_mito+brain_region+Sex	1.528	3090.000
ENSG00000020577	0.282	0.280	0.614	-0.055	SAMD4A	0.335	~amyloid_beta+(1|manifest)+cngeneson+pc_mito+brain_region+Sex	18.149	69.000
ENSG00000175928	0.015	0.279	0.577	-0.018	LRRN1	0.051	~amyloid_beta+(1|manifest)+cngeneson+pc_mito+brain_region+Sex	1.014	4107.000
ENSG00000120162	0.030	0.278	0.656	-0.100	MOB3B	0.075	~amyloid_beta+(1|manifest)+cngeneson+pc_mito+brain_region+Sex	1.692	2833.000
ENSG00000240230	0.001	0.278	0.480	0.076	COX19	0.008	~amyloid_beta+(1|manifest)+cngeneson+pc_mito+brain_region+Sex	0.652	4994.000
ENSG00000162390	0.046	0.278	0.516	0.040	ACOT11	0.095	~amyloid_beta+(1|manifest)+cngeneson+pc_mito+brain_region+Sex	14.460	115.000
ENSG00000075399	0.001	0.278	0.496	0.059	VPS9D1	0.008	~amyloid_beta+(1|manifest)+cngeneson+pc_mito+brain_region+Sex	2.076	2337.000
ENSG00000125755	0.017	0.277	0.595	-0.040	SYMPK	0.054	~amyloid_beta+(1|manifest)+cngeneson+pc_mito+brain_region+Sex	2.673	1751.000
ENSG00000085733	0.017	0.277	0.584	-0.030	CTTN	0.053	~amyloid_beta+(1|manifest)+cngeneson+pc_mito+brain_region+Sex	5.568	667.000
ENSG00000166689	0.057	0.277	0.547	0.007	PLEKHA7	0.108	~amyloid_beta+(1|manifest)+cngeneson+pc_mito+brain_region+Sex	3.509	1248.000
ENSG00000100321	0.001	0.276	0.462	0.090	SYNGR1	0.010	~amyloid_beta+(1|manifest)+cngeneson+pc_mito+brain_region+Sex	1.008	4123.000
ENSG00000103335	0.006	0.276	0.563	-0.010	PIEZO1	0.028	~amyloid_beta+(1|manifest)+cngeneson+pc_mito+brain_region+Sex	2.530	1873.000
ENSG00000152894	0.046	0.276	0.511	0.041	PTPRK	0.095	~amyloid_beta+(1|manifest)+cngeneson+pc_mito+brain_region+Sex	0.605	5118.000
ENSG00000145907	0.000	0.276	0.495	0.057	G3BP1	0.007	~amyloid_beta+(1|manifest)+cngeneson+pc_mito+brain_region+Sex	0.725	4806.000
ENSG00000108298	0.009	0.276	0.694	-0.142	RPL19	0.037	~amyloid_beta+(1|manifest)+cngeneson+pc_mito+brain_region+Sex	5.405	693.000
ENSG00000034713	0.002	0.276	0.526	0.025	[illegible]	0.017	~amyloid_beta+(1|manifest)+cngeneson+pc_mito+brain_region+Sex	1.300	3507.000
ENSG00000162104	0.001	0.276	0.522	0.030	ADCY9	0.008	~amyloid_beta+(1|manifest)+cngeneson+pc_mito+brain_region+Sex	0.697	4901.000
ENSG00000173511	0.010	0.275	0.546	0.005	VEGFB	0.040	~amyloid_beta+(1|manifest)+cngeneson+pc_mito+brain_region+Sex	3.604	1205.000
ENSG00000184828	0.024	0.275	0.611	-0.061	ZBTB7C	0.066	~amyloid_beta+(1|manifest)+cngeneson+pc_mito+brain_region+Sex	3.334	1350.000
ENSG00000108840	0.000	0.275	0.425	0.125	HDAC5	0.002	~amyloid_beta+(1|manifest)+cngeneson+pc_mito+brain_region+Sex	1.109	3910.000
ENSG00000115307	0.003	0.274	0.507	0.042	AUP1	0.021	~amyloid_beta+(1|manifest)+cngeneson+pc_mito+brain_region+Sex	2.191	2215.000
ENSG00000167969	0.000	0.274	0.447	0.101	ECI1	0.004	~amyloid_beta+(1|manifest)+cngeneson+pc_mito+brain_region+Sex	0.811	4596.000
ENSG00000136802	0.015	0.274	0.550	-0.002	LRRC8A	0.051	~amyloid_beta+(1|manifest)+cngeneson+pc_mito+brain_region+Sex	1.795	2709.000
ENSG00000100413	0.002	0.274	0.499	0.049	POLR3H	0.014	~amyloid_beta+(1|manifest)+cngeneson+pc_mito+brain_region+Sex	1.363	3385.000
ENSG00000111752	0.006	0.274	0.523	0.025	PHC1	0.030	~amyloid_beta+(1|manifest)+cngeneson+pc_mito+brain_region+Sex	3.248	1397.000
ENSG00000161542	0.002	0.274	0.467	0.080	PRPSAP1	0.017	~amyloid_beta+(1|manifest)+cngeneson+pc_mito+brain_region+Sex	0.677	4944.000
ENSG00000023909	0.001	0.274	0.582	-0.035	GCLM	0.008	~amyloid_beta+(1|manifest)+cngeneson+pc_mito+brain_region+Sex	1.118	3889.000
ENSG00000213676	0.003	0.272	0.533	0.011	ATF6B	0.021	~amyloid_beta+(1|manifest)+cngeneson+pc_mito+brain_region+Sex	2.052	2365.000
ENSG00000196504	0.001	0.271	0.491	0.052	PRPF40A	0.011	~amyloid_beta+(1|manifest)+cngeneson+pc_mito+brain_region+Sex	0.944	4269.000
ENSG00000139926	0.007	0.270	0.523	0.018	FRMD6	0.032	~amyloid_beta+(1|manifest)+cngeneson+pc_mito+brain_region+Sex	0.821	4569.000
ENSG00000155506	0.001	0.270	0.440	0.101	LARP1	0.010	~amyloid_beta+(1|manifest)+cngeneson+pc_mito+brain_region+Sex	1.087	3956.000
ENSG00000132669	0.027	0.270	0.463	0.077	RIN2	0.070	~amyloid_beta+(1|manifest)+cngeneson+pc_mito+brain_region+Sex	5.420	690.000
ENSG00000183049	0.014	0.270	0.568	-0.028	CAMK1D	0.049	~amyloid_beta+(1|manifest)+cngeneson+pc_mito+brain_region+Sex	1.686	2850.000
ENSG00000104936	0.009	0.270	0.565	-0.025	DMPK	0.037	~amyloid_beta+(1|manifest)+cngeneson+pc_mito+brain_region+Sex	3.769	1133.000
ENSG00000134278	0.005	0.270	0.424	0.115	SPIRE1	0.026	~amyloid_beta+(1|manifest)+cngeneson+pc_mito+brain_region+Sex	0.547	5235.000
ENSG00000137713	0.006	0.269	0.559	-0.021	PPP2R1B	0.030	~amyloid_beta+(1|manifest)+cngeneson+pc_mito+brain_region+Sex	2.806	1660.000
ENSG00000126016	0.001	0.268	0.460	0.076	AMOT	0.010	~amyloid_beta+(1|manifest)+cngeneson+pc_mito+brain_region+Sex	0.708	4855.000
ENSG00000016391	0.016	0.267	0.542	-0.008	CHDH	0.052	~amyloid_beta+(1|manifest)+cngeneson+pc_mito+brain_region+Sex	1.233	3627.000
ENSG00000059728	0.000	0.267	0.471	0.064	MXD1	0.007	~amyloid_beta+(1|manifest)+cngeneson+pc_mito+brain_region+Sex	0.382	5594.000
ENSG00000157110	0.113	0.267	0.728	-0.194	RBPMS	0.168	~amyloid_beta+(1|manifest)+cngeneson+pc_mito+brain_region+Sex	13.103	146.000
ENSG00000100077	0.082	0.267	0.667	-0.134	GRK3	0.137	~amyloid_beta+(1|manifest)+cngeneson+pc_mito+brain_region+Sex	4.250	962.000
ENSG00000143515	0.001	0.266	0.504	0.028	ATP8B2	0.007	~amyloid_beta+(1|manifest)+cngeneson+pc_mito+brain_region+Sex	1.326	3452.000
ENSG00000131149	0.014	0.266	0.603	-0.072	GSE1	0.047	~amyloid_beta+(1|manifest)+cngeneson+pc_mito+brain_region+Sex	2.508	1889.000
ENSG00000144668	0.030	0.266	0.506	0.025	ITGA9	0.074	~amyloid_beta+(1|manifest)+cngeneson+pc_mito+brain_region+Sex	0.956	4236.000
ENSG00000122547	0.016	0.265	0.574	-0.043	EEPD1	0.052	~amyloid_beta+(1|manifest)+cngeneson+pc_mito+brain_region+Sex	1.585	3006.000
ENSG00000164713	0.000	0.265	0.415	0.114	BRI3	0.003	~amyloid_beta+(1|manifest)+cngeneson+pc_mito+brain_region+Sex	1.441	3244.000
ENSG00000083099	0.000	0.265	0.390	0.140	LYRM2	0.001	~amyloid_beta+(1|manifest)+cngeneson+pc_mito+brain_region+Sex	0.469	5420.000
ENSG00000034053	0.012	0.265	0.633	-0.104	APBA2	0.044	~amyloid_beta+(1|manifest)+cngeneson+pc_mito+brain_region+Sex	4.826	814.000
ENSG00000136156	0.032	0.264	0.519	0.009	ITM2B	0.077	~amyloid_beta+(1|manifest)+cngeneson+pc_mito+brain_region+Sex	2.453	1933.000
ENSG00000006704	0.001	0.264	0.528	0.000	GTF2IRD1	0.013	~amyloid_beta+(1|manifest)+cngeneson+pc_mito+brain_region+Sex	2.852	1633.000
ENSG00000187147	0.159	0.264	0.678	-0.150	RNF220	0.214	~amyloid_beta+(1|manifest)+cngeneson+pc_mito+brain_region+Sex	4.020	1035.000
ENSG00000177570	0.014	0.263	0.640	-0.113	SAMD12	0.048	~amyloid_beta+(1|manifest)+cngeneson+pc_mito+brain_region+Sex	3.402	1315.000
ENSG00000176871	0.000	0.263	0.375	0.151	WSB2	0.000	~amyloid_beta+(1|manifest)+cngeneson+pc_mito+brain_region+Sex	0.283	5757.000
ENSG00000172613	0.002	0.262	0.462	0.063	RAD9A	0.016	~amyloid_beta+(1|manifest)+cngeneson+pc_mito+brain_region+Sex	2.002	2427.000
ENSG00000143850	0.471	0.262	0.938	-0.414	PLEKHA6	0.516	~amyloid_beta+(1|manifest)+cngeneson+pc_mito+brain_region+Sex	11.083	216.000
ENSG00000144445	0.025	0.261	0.463	0.059	KANSL1L	0.067	~amyloid_beta+(1|manifest)+cngeneson+pc_mito+brain_region+Sex	1.133	3858.000
ENSG00000161813	0.005	0.261	0.547	-0.025	LARP4	0.027	~amyloid_beta+(1|manifest)+cngeneson+pc_mito+brain_region+Sex	1.767	2726.000
ENSG00000164916	0.000	0.260	0.493	0.027	FOXK1	0.004	~amyloid_beta+(1|manifest)+cngeneson+pc_mito+brain_region+Sex	1.718	2797.000
ENSG00000105223	0.003	0.260	0.509	0.012	PLD3	0.018	~amyloid_beta+(1|manifest)+cngeneson+pc_mito+brain_region+Sex	2.969	1559.000
ENSG00000026025	0.188	0.260	0.893	-0.374	VIM	0.243	~amyloid_beta+(1|manifest)+cngeneson+pc_mito+brain_region+Sex	10.942	226.000
ENSG00000131730	0.009	0.260	0.543	-0.024	CKMT2	0.038	~amyloid_beta+(1|manifest)+cngeneson+pc_mito+brain_region+Sex	3.341	1345.000
ENSG00000141905	0.001	0.259	0.469	0.049	NFIC	0.010	~amyloid_beta+(1|manifest)+cngeneson+pc_mito+brain_region+Sex	2.644	1775.000
ENSG00000100345	0.048	0.259	0.614	-0.096	MYH9	0.097	~amyloid_beta+(1|manifest)+cngeneson+pc_mito+brain_region+Sex	6.571	518.000
ENSG00000092531	0.003	0.259	0.462	0.055	SNAP23	0.020	~amyloid_beta+(1|manifest)+cngeneson+pc_mito+brain_region+Sex	0.451	5452.000
ENSG00000128602	0.001	0.258	0.464	0.053	SMO	0.013	~amyloid_beta+(1|manifest)+cngeneson+pc_mito+brain_region+Sex	2.035	2388.000
ENSG00000079805	0.000	0.258	0.456	0.061	DNM2	0.002	~amyloid_beta+(1|manifest)+cngeneson+pc_mito+brain_region+Sex	1.648	2908.000
ENSG00000179241	0.002	0.258	0.483	0.034	LDLRAD3	0.017	~amyloid_beta+(1|manifest)+cngeneson+pc_mito+brain_region+Sex	1.674	2862.000
ENSG00000144029	0.002	0.257	0.433	0.081	MRPS5	0.015	~amyloid_beta+(1|manifest)+cngeneson+pc_mito+brain_region+Sex	1.351	3411.000
ENSG00000107331	0.064	0.257	0.659	-0.145	ABCA2	0.116	~amyloid_beta+(1|manifest)+cngeneson+pc_mito+brain_region+Sex	4.902	797.000
ENSG00000121892	0.001	0.257	0.458	0.056	PDS5A	0.012	~amyloid_beta+(1|manifest)+cngeneson+pc_mito+brain_region+Sex	0.212	5858.000
ENSG00000133318	0.003	0.256	0.434	0.078	RTN3	0.021	~amyloid_beta+(1|manifest)+cngeneson+pc_mito+brain_region+Sex	0.637	5039.000
ENSG00000075785	0.001	0.256	0.429	0.082	RAB7A	0.009	~amyloid_beta+(1|manifest)+cngeneson+pc_mito+brain_region+Sex	2.829	1647.000
ENSG00000182247	0.224	0.256	0.572	-0.061	UBE2E2	0.279	~amyloid_beta+(1|manifest)+cngeneson+pc_mito+brain_region+Sex	3.915	1082.000
ENSG00000184349	0.216	0.255	0.566	-0.056	EFNA5	0.271	~amyloid_beta+(1|manifest)+cngeneson+pc_mito+brain_region+Sex	3.177	1438.000
ENSG00000139990	0.000	0.255	0.420	0.090	DCAF5	0.004	~amyloid_beta+(1|manifest)+cngeneson+pc_mito+brain_region+Sex	0.993	4159.000
ENSG00000157193	0.004	0.255	0.553	-0.043	LRP8	0.024	~amyloid_beta+(1|manifest)+cngeneson+pc_mito+brain_region+Sex	0.937	4284.000
ENSG00000165280	0.000	0.255	0.392	0.118	VCP	0.003	~amyloid_beta+(1|manifest)+cngeneson+pc_mito+brain_region+Sex	0.802	4624.000
ENSG00000002834	0.000	0.255	0.411	0.098	LASP1	0.003	~amyloid_beta+(1|manifest)+cngeneson+pc_mito+brain_region+Sex	0.507	5335.000
ENSG00000165637	0.000	0.255	0.388	0.121	VDAC2	0.002	~amyloid_beta+(1|manifest)+cngeneson+pc_mito+brain_region+Sex	0.313	5707.000
ENSG00000174080	0.058	0.254	0.639	-0.130	CTSF	0.109	~amyloid_beta+(1|manifest)+cngeneson+pc_mito+brain_region+Sex	3.520	1240.000
ENSG00000165802	0.267	0.254	0.767	-0.258	NSMF	0.321	~amyloid_beta+(1|manifest)+cngeneson+pc_mito+brain_region+Sex	9.292	307.000
ENSG00000176165	0.009	0.254	0.538	-0.029	FOXG1	0.037	~amyloid_beta+(1|manifest)+cngeneson+pc_mito+brain_region+Sex	3.625	1193.000
ENSG00000125970	0.000	0.254	0.391	0.116	RALY	0.001	~amyloid_beta+(1|manifest)+cngeneson+pc_mito+brain_region+Sex	0.393	5574.000
ENSG00000198429	0.000	0.254	0.410	0.097	ZNF69	0.000	~amyloid_beta+(1|manifest)+cngeneson+pc_mito+brain_region+Sex	0.662	4973.000
ENSG00000120907	0.072	0.254	0.592	-0.085	ADRA1A	0.126	~amyloid_beta+(1|manifest)+cngeneson+pc_mito+brain_region+Sex	3.584	1213.000
ENSG00000103994	0.001	0.254	0.417	0.090	ZNF106	0.008	~amyloid_beta+(1|manifest)+cngeneson+pc_mito+brain_region+Sex	0.148	5925.000
ENSG00000137486	0.252	0.254	0.720	-0.213	ARRB1	0.306	~amyloid_beta+(1|manifest)+cngeneson+pc_mito+brain_region+Sex	9.316	304.000
ENSG00000100711	0.182	0.253	0.747	-0.240	ZFYVE21	0.236	~amyloid_beta+(1|manifest)+cngeneson+pc_mito+brain_region+Sex	7.313	439.000
ENSG00000213918	0.000	0.253	0.408	0.098	DNASE1	0.007	~amyloid_beta+(1|manifest)+cngeneson+pc_mito+brain_region+Sex	1.252	3595.000
ENSG00000187764	0.026	0.253	0.589	-0.083	SEMA4D	0.068	~amyloid_beta+(1|manifest)+cngeneson+pc_mito+brain_region+Sex	3.462	1269.000
ENSG00000169714	0.001	0.253	0.430	0.076	CNBP	0.010	~amyloid_beta+(1|manifest)+cngeneson+pc_mito+brain_region+Sex	0.250	5809.000
ENSG00000138640	0.069	0.253	0.637	-0.131	FAM13A	0.122	~amyloid_beta+(1|manifest)+cngeneson+pc_mito+brain_region+Sex	3.381	1329.000
ENSG00000053254	0.039	0.252	0.473	0.031	FOXN3	0.086	~amyloid_beta+(1|manifest)+cngeneson+pc_mito+brain_region+Sex	3.442	1281.000
ENSG00000187079	0.012	0.252	0.406	0.097	TEAD1	0.043	~amyloid_beta+(1|manifest)+cngeneson+pc_mito+brain_region+Sex	4.665	853.000
ENSG00000169045	0.013	0.252	0.525	-0.022	HNRNPH1	0.046	~amyloid_beta+(1|manifest)+cngeneson+pc_mito+brain_region+Sex	2.119	2289.000
ENSG00000182087	0.183	0.251	0.778	-0.276	TMEM259	0.237	~amyloid_beta+(1|manifest)+cngeneson+pc_mito+brain_region+Sex	8.939	318.000
ENSG00000015153	0.001	0.250	0.398	0.101	YAF2	0.007	~amyloid_beta+(1|manifest)+cngeneson+pc_mito+brain_region+Sex	0.562	5202.000
ENSG00000103148	0.000	0.250	0.419	0.080	NPRL3	0.007	~amyloid_beta+(1|manifest)+cngeneson+pc_mito+brain_region+Sex	1.525	3096.000
ENSG00000203880	0.002	0.249	0.490	0.008	PCMTD2	0.016	~amyloid_beta+(1|manifest)+cngeneson+pc_mito+brain_region+Sex	1.192	3721.000
ENSG00000101493	0.163	0.249	0.597	-0.099	ZNF516	0.218	~amyloid_beta+(1|manifest)+cngeneson+pc_mito+brain_region+Sex	3.202	1424.000
ENSG00000160199	0.000	0.249	0.397	0.101	PKNOX1	0.003	~amyloid_beta+(1|manifest)+cngeneson+pc_mito+brain_region+Sex	0.691	4917.000
ENSG00000137074	0.007	0.249	0.455	0.043	APTX	0.032	~amyloid_beta+(1|manifest)+cngeneson+pc_mito+brain_region+Sex	0.887	4405.000
ENSG00000122884	0.349	0.248	0.636	-0.139	P4HA1	0.399	~amyloid_beta+(1|manifest)+cngeneson+pc_mito+brain_region+Sex	3.444	1279.000
ENSG00000090238	0.004	0.248	0.536	-0.040	YPEL3	0.024	~amyloid_beta+(1|manifest)+cngeneson+pc_mito+brain_region+Sex	3.429	1293.000
ENSG00000165164	0.013	0.248	0.663	-0.167	CFAP47	0.045	~amyloid_beta+(1|manifest)+cngeneson+pc_mito+brain_region+Sex	2.075	2340.000
ENSG00000112306	0.003	0.248	0.451	0.045	RPS12	0.018	~amyloid_beta+(1|manifest)+cngeneson+pc_mito+brain_region+Sex	2.324	2076.000
ENSG00000161714	0.000	0.248	0.396	0.099	PLCD3	0.002	~amyloid_beta+(1|manifest)+cngeneson+pc_mito+brain_region+Sex	1.216	3670.000
ENSG00000255302	0.010	0.248	0.499	-0.004	EID1	0.039	~amyloid_beta+(1|manifest)+cngeneson+pc_mito+brain_region+Sex	1.692	2835.000
ENSG00000105426	0.011	0.247	0.466	0.029	PTPRS	0.043	~amyloid_beta+(1|manifest)+cngeneson+pc_mito+brain_region+Sex	3.511	1246.000
ENSG00000088682	0.002	0.247	0.448	0.046	COQ9	0.015	~amyloid_beta+(1|manifest)+cngeneson+pc_mito+brain_region+Sex	1.619	2954.000
ENSG00000147799	0.014	0.247	0.636	-0.143	ARHGAP39	0.048	~amyloid_beta+(1|manifest)+cngeneson+pc_mito+brain_region+Sex	4.696	841.000
ENSG00000113558	0.002	0.247	0.459	0.035	SKP1	0.017	~amyloid_beta+(1|manifest)+cngeneson+pc_mito+brain_region+Sex	0.469	5422.000
ENSG00000140474	0.019	0.247	0.511	-0.018	ULK3	0.057	~amyloid_beta+(1|manifest)+cngeneson+pc_mito+brain_region+Sex	1.940	2507.000
ENSG00000133065	0.001	0.246	0.403	0.090	SLC41A1	0.008	~amyloid_beta+(1|manifest)+cngeneson+pc_mito+brain_region+Sex	0.878	4420.000
ENSG00000115073	0.004	0.246	0.476	0.017	ACTR1B	0.023	~amyloid_beta+(1|manifest)+cngeneson+pc_mito+brain_region+Sex	1.927	2527.000
ENSG00000063176	0.000	0.246	0.411	0.082	SPHK2	0.006	~amyloid_beta+(1|manifest)+cngeneson+pc_mito+brain_region+Sex	0.672	4959.000
ENSG00000197077	0.013	0.246	0.441	0.052	KIAA1671	0.046	~amyloid_beta+(1|manifest)+cngeneson+pc_mito+brain_region+Sex	2.700	1729.000
ENSG00000174611	0.017	0.245	0.606	-0.115	KY	0.053	~amyloid_beta+(1|manifest)+cngeneson+pc_mito+brain_region+Sex	2.931	1587.000
ENSG00000105552	0.125	0.245	0.606	-0.115	BCAT2	0.181	~amyloid_beta+(1|manifest)+cngeneson+pc_mito+brain_region+Sex	6.715	506.000
ENSG00000067992	0.000	0.245	0.454	0.036	PDK3	0.004	~amyloid_beta+(1|manifest)+cngeneson+pc_mito+brain_region+Sex	1.027	4084.000
ENSG00000063854	0.001	0.245	0.425	0.066	HAGH	0.013	~amyloid_beta+(1|manifest)+cngeneson+pc_mito+brain_region+Sex	1.860	2616.000
ENSG00000153561	0.000	0.245	0.423	0.066	RMND5A	0.004	~amyloid_beta+(1|manifest)+cngeneson+pc_mito+brain_region+Sex	0.417	5532.000
ENSG00000132963	0.011	0.244	0.529	-0.042	POMP	0.043	~amyloid_beta+(1|manifest)+cngeneson+pc_mito+brain_region+Sex	0.611	5105.000
ENSG00000147679	0.033	0.244	0.549	-0.061	UTP23	0.079	~amyloid_beta+(1|manifest)+cngeneson+pc_mito+brain_region+Sex	1.480	3175.000
ENSG00000109736	0.015	0.244	0.529	-0.042	MFSD10	0.050	~amyloid_beta+(1|manifest)+cngeneson+pc_mito+brain_region+Sex	1.906	2547.000
ENSG00000067560	0.000	0.243	0.391	0.095	RHOA	0.005	~amyloid_beta+(1|manifest)+cngeneson+pc_mito+brain_region+Sex	0.269	5784.000
ENSG00000100242	0.024	0.243	0.547	-0.060	SUN2	0.066	~amyloid_beta+(1|manifest)+cngeneson+pc_mito+brain_region+Sex	2.830	1645.000
ENSG00000136158	0.019	0.243	0.508	-0.022	SPRY2	0.057	~amyloid_beta+(1|manifest)+cngeneson+pc_mito+brain_region+Sex	3.035	1522.000
ENSG00000134504	0.006	0.243	0.426	0.059	KCTD1	0.029	~amyloid_beta+(1|manifest)+cngeneson+pc_mito+brain_region+Sex	0.856	4474.000
ENSG00000155085	0.001	0.243	0.412	0.073	AK9	0.013	~amyloid_beta+(1|manifest)+cngeneson+pc_mito+brain_region+Sex	0.934	4291.000
ENSG00000138031	0.013	0.243	0.592	-0.107	ADCY3	0.047	~amyloid_beta+(1|manifest)+cngeneson+pc_mito+brain_region+Sex	3.015	1534.000
ENSG00000123595	0.000	0.242	0.363	0.122	RAB9A	0.002	~amyloid_beta+(1|manifest)+cngeneson+pc_mito+brain_region+Sex	0.366	5625.000
ENSG00000185650	0.110	0.242	0.477	0.007	ZFP36L1	0.166	~amyloid_beta+(1|manifest)+cngeneson+pc_mito+brain_region+Sex	2.351	2046.000
ENSG00000050165	0.143	0.242	0.517	-0.034	DKK3	0.199	~amyloid_beta+(1|manifest)+cngeneson+pc_mito+brain_region+Sex	7.402	425.000
ENSG00000180098	0.002	0.241	0.446	0.036	TRNAU1AP	0.015	~amyloid_beta+(1|manifest)+cngeneson+pc_mito+brain_region+Sex	1.123	3877.000
ENSG00000154127	0.025	0.241	0.683	-0.201	UBASH3B	0.067	~amyloid_beta+(1|manifest)+cngeneson+pc_mito+brain_region+Sex	5.685	651.000
ENSG00000143774	0.010	0.241	0.472	0.011	GUK1	0.040	~amyloid_beta+(1|manifest)+cngeneson+pc_mito+brain_region+Sex	2.751	1703.000
ENSG00000125977	0.000	0.241	0.405	0.077	EIF2S2	0.002	~amyloid_beta+(1|manifest)+cngeneson+pc_mito+brain_region+Sex	0.124	5938.000
ENSG00000104365	0.000	0.241	0.358	0.124	IKBKB	0.001	~amyloid_beta+(1|manifest)+cngeneson+pc_mito+brain_region+Sex	0.692	4912.000
ENSG00000170370	0.276	0.241	0.666	-0.184	EMX2	0.329	~amyloid_beta+(1|manifest)+cngeneson+pc_mito+brain_region+Sex	2.231	2172.000
ENSG00000136213	0.004	0.241	0.529	-0.048	CHST12	0.024	~amyloid_beta+(1|manifest)+cngeneson+pc_mito+brain_region+Sex	1.880	2591.000
ENSG00000134775	0.029	0.241	0.468	0.013	FHOD3	0.072	~amyloid_beta+(1|manifest)+cngeneson+pc_mito+brain_region+Sex	1.306	3491.000
ENSG00000187535	0.000	0.241	0.357	0.124	IFT140	0.001	~amyloid_beta+(1|manifest)+cngeneson+pc_mito+brain_region+Sex	0.830	4542.000
ENSG00000105738	0.001	0.240	0.420	0.061	SIPA1L3	0.008	~amyloid_beta+(1|manifest)+cngeneson+pc_mito+brain_region+Sex	0.625	5072.000
ENSG00000108107	0.009	0.240	0.488	-0.008	RPL28	0.037	~amyloid_beta+(1|manifest)+cngeneson+pc_mito+brain_region+Sex	1.513	3116.000
ENSG00000225921	0.001	0.240	0.434	0.045	NOL7	0.013	~amyloid_beta+(1|manifest)+cngeneson+pc_mito+brain_region+Sex	0.356	5642.000
ENSG00000117298	0.025	0.239	0.539	-0.061	ECE1	0.067	~amyloid_beta+(1|manifest)+cngeneson+pc_mito+brain_region+Sex	3.616	1199.000
ENSG00000131067	0.002	0.239	0.403	0.075	GGT7	0.015	~amyloid_beta+(1|manifest)+cngeneson+pc_mito+brain_region+Sex	1.512	3117.000
ENSG00000071626	0.000	0.239	0.369	0.109	DAZAP1	0.005	~amyloid_beta+(1|manifest)+cngeneson+pc_mito+brain_region+Sex	1.492	3152.000
ENSG00000067082	0.020	0.238	0.563	-0.087	KLF6	0.059	~amyloid_beta+(1|manifest)+cngeneson+pc_mito+brain_region+Sex	2.105	2307.000
ENSG00000196588	0.127	0.238	0.545	-0.069	MRTFA	0.183	~amyloid_beta+(1|manifest)+cngeneson+pc_mito+brain_region+Sex	4.986	778.000
ENSG00000093010	0.004	0.238	0.410	0.067	COMT	0.022	~amyloid_beta+(1|manifest)+cngeneson+pc_mito+brain_region+Sex	0.680	4940.000
ENSG00000167971	0.223	0.238	0.730	-0.253	CASKIN1	0.278	~amyloid_beta+(1|manifest)+cngeneson+pc_mito+brain_region+Sex	6.178	568.000
ENSG00000109332	0.012	0.238	0.445	0.031	UBE2D3	0.044	~amyloid_beta+(1|manifest)+cngeneson+pc_mito+brain_region+Sex	0.673	4958.000
ENSG00000218336	0.256	0.238	0.700	-0.224	TENM3	0.310	~amyloid_beta+(1|manifest)+cngeneson+pc_mito+brain_region+Sex	8.715	328.000
ENSG00000148204	0.172	0.237	0.630	-0.156	CRB2	0.227	~amyloid_beta+(1|manifest)+cngeneson+pc_mito+brain_region+Sex	2.704	1723.000
ENSG00000140575	0.005	0.237	0.599	-0.125	IQGAP1	0.026	~amyloid_beta+(1|manifest)+cngeneson+pc_mito+brain_region+Sex	2.957	1565.000
ENSG00000179242	0.054	0.237	0.457	0.016	CDH4	0.104	~amyloid_beta+(1|manifest)+cngeneson+pc_mito+brain_region+Sex	2.362	2037.000
ENSG00000019995	0.000	0.236	0.376	0.096	ZRANB1	0.003	~amyloid_beta+(1|manifest)+cngeneson+pc_mito+brain_region+Sex	1.154	3804.000
ENSG00000133121	0.098	0.236	0.441	0.030	STARD13	0.153	~amyloid_beta+(1|manifest)+cngeneson+pc_mito+brain_region+Sex	4.374	924.000
ENSG00000179456	0.002	0.236	0.448	0.023	ZBTB18	0.017	~amyloid_beta+(1|manifest)+cngeneson+pc_mito+brain_region+Sex	0.918	4332.000
ENSG00000168488	0.025	0.235	0.535	-0.064	ATXN2L	0.067	~amyloid_beta+(1|manifest)+cngeneson+pc_mito+brain_region+Sex	3.843	1107.000
ENSG00000107882	0.002	0.235	0.416	0.054	SUFU	0.018	~amyloid_beta+(1|manifest)+cngeneson+pc_mito+brain_region+Sex	2.036	2387.000
ENSG00000110651	0.198	0.235	0.577	-0.107	CD81	0.253	~amyloid_beta+(1|manifest)+cngeneson+pc_mito+brain_region+Sex	4.043	1029.000
ENSG00000141441	0.058	0.235	0.415	0.055	GAREM1	0.109	~amyloid_beta+(1|manifest)+cngeneson+pc_mito+brain_region+Sex	6.766	497.000
ENSG00000257923	0.000	0.235	0.370	0.099	CUX1	0.002	~amyloid_beta+(1|manifest)+cngeneson+pc_mito+brain_region+Sex	0.927	4314.000
ENSG00000175182	0.001	0.235	0.402	0.067	FAM131A	0.009	~amyloid_beta+(1|manifest)+cngeneson+pc_mito+brain_region+Sex	1.712	2806.000
ENSG00000196781	0.226	0.235	0.566	-0.097	TLE1	0.280	~amyloid_beta+(1|manifest)+cngeneson+pc_mito+brain_region+Sex	3.866	1099.000
ENSG00000196182	0.035	0.234	0.504	-0.035	STK40	0.081	~amyloid_beta+(1|manifest)+cngeneson+pc_mito+brain_region+Sex	4.105	1013.000
ENSG00000197746	0.120	0.234	0.530	-0.062	PSAP	0.176	~amyloid_beta+(1|manifest)+cngeneson+pc_mito+brain_region+Sex	3.298	1369.000
ENSG00000183283	0.002	0.234	0.418	0.050	DAZAP2	0.014	~amyloid_beta+(1|manifest)+cngeneson+pc_mito+brain_region+Sex	1.532	3084.000
ENSG00000186298	0.002	0.234	0.426	0.042	PPP1CC	0.017	~amyloid_beta+(1|manifest)+cngeneson+pc_mito+brain_region+Sex	0.186	5892.000
ENSG00000172380	0.003	0.234	0.442	0.026	GNG12	0.021	~amyloid_beta+(1|manifest)+cngeneson+pc_mito+brain_region+Sex	0.382	5596.000
ENSG00000133872	0.088	0.233	0.551	-0.084	SARAF	0.143	~amyloid_beta+(1|manifest)+cngeneson+pc_mito+brain_region+Sex	1.699	2824.000
ENSG00000172201	0.187	0.233	0.567	-0.100	ID4	0.241	~amyloid_beta+(1|manifest)+cngeneson+pc_mito+brain_region+Sex	5.229	720.000
ENSG00000119048	0.135	0.233	0.743	-0.276	UBE2B	0.191	~amyloid_beta+(1|manifest)+cngeneson+pc_mito+brain_region+Sex	2.253	2151.000
ENSG00000133104	0.008	0.233	0.437	0.029	SPART	0.035	~amyloid_beta+(1|manifest)+cngeneson+pc_mito+brain_region+Sex	0.308	5713.000
ENSG00000081189	0.007	0.233	0.450	0.016	MEF2C	0.033	~amyloid_beta+(1|manifest)+cngeneson+pc_mito+brain_region+Sex	1.339	3430.000
ENSG00000169851	0.300	0.233	0.530	-0.065	PCDH7	0.353	~amyloid_beta+(1|manifest)+cngeneson+pc_mito+brain_region+Sex	5.077	760.000
ENSG00000137177	0.011	0.233	0.407	0.058	KIF13A	0.042	~amyloid_beta+(1|manifest)+cngeneson+pc_mito+brain_region+Sex	2.026	2401.000
ENSG00000189077	0.000	0.232	0.381	0.084	[illegible]	0.006	~amyloid_beta+(1|manifest)+cngeneson+pc_mito+brain_region+Sex	0.595	5143.000
ENSG00000160972	0.027	0.232	0.541	-0.077	PPP1R16A	0.070	~amyloid_beta+(1|manifest)+cngeneson+pc_mito+brain_region+Sex	2.564	1844.000
ENSG00000168653	0.012	0.232	0.529	-0.065	NDUFS5	0.044	~amyloid_beta+(1|manifest)+cngeneson+pc_mito+brain_region+Sex	1.652	2900.000
ENSG00000185359	0.000	0.232	0.373	0.092	HGS	0.006	~amyloid_beta+(1|manifest)+cngeneson+pc_mito+brain_region+Sex	1.425	3276.000
ENSG00000119950	0.082	0.232	0.457	0.007	MXI1	0.137	~amyloid_beta+(1|manifest)+cngeneson+pc_mito+brain_region+Sex	3.831	1111.000
ENSG00000171552	0.000	0.232	0.391	0.073	BCL2L1	0.005	~amyloid_beta+(1|manifest)+cngeneson+pc_mito+brain_region+Sex	0.921	4326.000
ENSG00000146830	0.004	0.231	0.443	0.019	GIGYF1	0.025	~amyloid_beta+(1|manifest)+cngeneson+pc_mito+brain_region+Sex	2.656	1764.000
ENSG00000140443	0.200	0.231	0.491	-0.028	IGF1R	0.255	~amyloid_beta+(1|manifest)+cngeneson+pc_mito+brain_region+Sex	5.973	595.000
ENSG00000148498	0.000	0.231	0.386	0.077	PARD3	0.005	~amyloid_beta+(1|manifest)+cngeneson+pc_mito+brain_region+Sex	9.837	278.000
ENSG00000112576	0.163	0.231	0.884	-0.422	CCND3	0.218	~amyloid_beta+(1|manifest)+cngeneson+pc_mito+brain_region+Sex	8.486	348.000
ENSG00000183722	0.365	0.230	0.546	-0.086	LHFPL6	0.416	~amyloid_beta+(1|manifest)+cngeneson+pc_mito+brain_region+Sex	8.497	345.000
ENSG00000163882	0.007	0.230	0.450	0.010	POLR2H	0.031	~amyloid_beta+(1|manifest)+cngeneson+pc_mito+brain_region+Sex	1.608	2970.000
ENSG00000203780	0.002	0.230	0.467	-0.007	FANK1	0.014	~amyloid_beta+(1|manifest)+cngeneson+pc_mito+brain_region+Sex	0.962	4222.000
ENSG00000110723	0.067	0.229	0.531	-0.073	EXPH5	0.121	~amyloid_beta+(1|manifest)+cngeneson+pc_mito+brain_region+Sex	2.081	2335.000
ENSG00000136444	0.001	0.229	0.396	0.063	RSAD1	0.011	~amyloid_beta+(1|manifest)+cngeneson+pc_mito+brain_region+Sex	1.191	3725.000
ENSG00000162433	0.095	0.228	0.570	-0.114	AK4	0.150	~amyloid_beta+(1|manifest)+cngeneson+pc_mito+brain_region+Sex	1.704	2817.000
ENSG00000141664	0.002	0.228	0.402	0.054	ZCCHC2	0.015	~amyloid_beta+(1|manifest)+cngeneson+pc_mito+brain_region+Sex	0.238	5827.000
ENSG00000188529	0.001	0.228	0.391	0.064	SRSF10	0.013	~amyloid_beta+(1|manifest)+cngeneson+pc_mito+brain_region+Sex	0.807	4609.000
ENSG00000068976	0.024	0.227	0.582	-0.128	PYGM	0.066	~amyloid_beta+(1|manifest)+cngeneson+pc_mito+brain_region+Sex	3.236	1407.000
ENSG00000132879	0.016	0.227	0.501	-0.047	FBXO44	0.052	~amyloid_beta+(1|manifest)+cngeneson+pc_mito+brain_region+Sex	1.920	2537.000
ENSG00000171163	0.006	0.227	0.463	-0.009	ZNF692	0.030	~amyloid_beta+(1|manifest)+cngeneson+pc_mito+brain_region+Sex	2.130	2278.000
ENSG00000128272	0.001	0.227	0.416	0.037	ATF4	0.009	~amyloid_beta+(1|manifest)+cngeneson+pc_mito+brain_region+Sex	1.023	4093.000
ENSG00000073060	0.001	0.227	0.412	0.042	SCARB1	0.007	~amyloid_beta+(1|manifest)+cngeneson+pc_mito+brain_region+Sex	0.805	4616.000
ENSG00000037637	0.004	0.227	0.409	0.045	FBXO42	0.022	~amyloid_beta+(1|manifest)+cngeneson+pc_mito+brain_region+Sex	0.394	5572.000
ENSG00000089063	0.000	0.227	0.303	0.150	TMEM230	0.000	~amyloid_beta+(1|manifest)+cngeneson+pc_mito+brain_region+Sex	0.377	5601.000
ENSG00000205531	0.009	0.226	0.464	-0.012	NAP1L4	0.036	~amyloid_beta+(1|manifest)+cngeneson+pc_mito+brain_region+Sex	1.512	3121.000
ENSG00000169710	0.038	0.226	0.535	-0.083	FASN	0.085	~amyloid_beta+(1|manifest)+cngeneson+pc_mito+brain_region+Sex	2.522	1878.000
ENSG00000147862	0.008	0.226	0.391	0.061	NFIB	0.035	~amyloid_beta+(1|manifest)+cngeneson+pc_mito+brain_region+Sex	5.006	773.000
ENSG00000092621	0.011	0.226	0.518	-0.067	PHGDH	0.042	~amyloid_beta+(1|manifest)+cngeneson+pc_mito+brain_region+Sex	1.895	2557.000
ENSG00000126602	0.000	0.225	0.314	0.137	TRAP1	0.000	~amyloid_beta+(1|manifest)+cngeneson+pc_mito+brain_region+Sex	0.311	5710.000
ENSG00000160469	0.000	0.225	0.375	0.076	BRSK1	0.007	~amyloid_beta+(1|manifest)+cngeneson+pc_mito+brain_region+Sex	1.384	3349.000
ENSG00000171314	0.011	0.225	0.454	-0.003	PGAM1	0.041	~amyloid_beta+(1|manifest)+cngeneson+pc_mito+brain_region+Sex	1.162	3784.000
ENSG00000178605	0.001	0.225	0.381	0.069	GTPBP6	0.013	~amyloid_beta+(1|manifest)+cngeneson+pc_mito+brain_region+Sex	0.857	4472.000
ENSG00000140995	0.004	0.225	0.439	0.011	DEF8	0.024	~amyloid_beta+(1|manifest)+cngeneson+pc_mito+brain_region+Sex	1.823	2663.000
ENSG00000197043	0.000	0.225	0.328	0.122	ANXA6	0.000	~amyloid_beta+(1|manifest)+cngeneson+pc_mito+brain_region+Sex	0.306	5722.000
ENSG00000103449	0.002	0.225	0.410	0.040	SALL1	0.014	~amyloid_beta+(1|manifest)+cngeneson+pc_mito+brain_region+Sex	0.760	4715.000
ENSG00000105819	0.006	0.225	0.421	0.028	PMPCB	0.029	~amyloid_beta+(1|manifest)+cngeneson+pc_mito+brain_region+Sex	0.616	5097.000
ENSG00000147421	0.257	0.225	0.537	-0.087	HMBOX1	0.311	~amyloid_beta+(1|manifest)+cngeneson+pc_mito+brain_region+Sex	13.853	127.000
ENSG00000155897	0.027	0.224	0.459	-0.010	ADCY8	0.070	~amyloid_beta+(1|manifest)+cngeneson+pc_mito+brain_region+Sex	4.549	881.000
ENSG00000143612	0.000	0.224	0.339	0.110	C1orf43	0.001	~amyloid_beta+(1|manifest)+cngeneson+pc_mito+brain_region+Sex	0.725	4807.000
ENSG00000189337	0.166	0.224	0.544	-0.095	KAZN	0.221	~amyloid_beta+(1|manifest)+cngeneson+pc_mito+brain_region+Sex	1.606	2975.000
ENSG00000137575	0.000	0.224	0.344	0.104	SDCBP	0.001	~amyloid_beta+(1|manifest)+cngeneson+pc_mito+brain_region+Sex	0.383	5590.000
ENSG00000077092	0.013	0.224	0.485	-0.036	RARB	0.047	~amyloid_beta+(1|manifest)+cngeneson+pc_mito+brain_region+Sex	1.733	2777.000
ENSG00000007047	0.000	0.224	0.382	0.066	MARK4	0.004	~amyloid_beta+(1|manifest)+cngeneson+pc_mito+brain_region+Sex	0.738	4776.000
ENSG00000143970	0.002	0.224	0.383	0.065	ASXL2	0.017	~amyloid_beta+(1|manifest)+cngeneson+pc_mito+brain_region+Sex	1.212	3676.000
ENSG00000137449	0.156	0.224	0.626	-0.178	CPEB2	0.212	~amyloid_beta+(1|manifest)+cngeneson+pc_mito+brain_region+Sex	2.465	1926.000
ENSG00000113312	0.000	0.224	0.360	0.088	TTC1	0.003	~amyloid_beta+(1|manifest)+cngeneson+pc_mito+brain_region+Sex	0.762	4710.000
ENSG00000136193	0.021	0.224	0.472	-0.024	SCRN1	0.061	~amyloid_beta+(1|manifest)+cngeneson+pc_mito+brain_region+Sex	0.643	5022.000
ENSG00000147403	0.051	0.224	0.727	-0.280	RPL10	0.101	~amyloid_beta+(1|manifest)+cngeneson+pc_mito+brain_region+Sex	7.666	404.000
ENSG00000213339	0.156	0.224	0.609	-0.161	QTRT1	0.211	~amyloid_beta+(1|manifest)+cngeneson+pc_mito+brain_region+Sex	6.049	586.000
ENSG00000197119	0.007	0.223	0.624	-0.178	SLC25A29	0.031	~amyloid_beta+(1|manifest)+cngeneson+pc_mito+brain_region+Sex	4.099	1016.000
ENSG00000137106	0.000	0.223	0.369	0.078	GRHPR	0.005	~amyloid_beta+(1|manifest)+cngeneson+pc_mito+brain_region+Sex	1.034	4062.000
ENSG00000127948	0.006	0.223	0.419	0.028	POR	0.028	~amyloid_beta+(1|manifest)+cngeneson+pc_mito+brain_region+Sex	0.696	4905.000
ENSG00000160752	0.001	0.223	0.423	0.023	FDPS	0.007	~amyloid_beta+(1|manifest)+cngeneson+pc_mito+brain_region+Sex	2.305	2097.000
ENSG00000107816	0.002	0.223	0.393	0.053	LZTS2	0.015	~amyloid_beta+(1|manifest)+cngeneson+pc_mito+brain_region+Sex	1.049	4027.000
ENSG00000124126	0.053	0.223	0.472	-0.026	PREX1	0.103	~amyloid_beta+(1|manifest)+cngeneson+pc_mito+brain_region+Sex	3.988	1044.000
ENSG00000176390	0.009	0.222	0.421	0.023	CRLF3	0.037	~amyloid_beta+(1|manifest)+cngeneson+pc_mito+brain_region+Sex	2.438	1944.000
ENSG00000134308	0.000	0.222	0.372	0.072	YWHAQ	0.003	~amyloid_beta+(1|manifest)+cngeneson+pc_mito+brain_region+Sex	0.345	5660.000
ENSG00000144485	0.093	0.222	0.590	-0.146	HES6	0.149	~amyloid_beta+(1|manifest)+cngeneson+pc_mito+brain_region+Sex	4.121	1008.000
ENSG00000165186	0.224	0.222	0.639	-0.195	PTCHD1	0.278	~amyloid_beta+(1|manifest)+cngeneson+pc_mito+brain_region+Sex	7.220	449.000
ENSG00000179832	0.000	0.222	0.378	0.066	MROH1	0.003	~amyloid_beta+(1|manifest)+cngeneson+pc_mito+brain_region+Sex	1.352	3407.000
ENSG00000121274	0.003	0.222	0.448	-0.004	TENT4B	0.020	~amyloid_beta+(1|manifest)+cngeneson+pc_mito+brain_region+Sex	1.118	3890.000
ENSG00000114353	0.005	0.222	0.401	0.042	GNAI2	0.027	~amyloid_beta+(1|manifest)+cngeneson+pc_mito+brain_region+Sex	1.688	2849.000
ENSG00000114646	0.179	0.221	0.786	-0.343	CSPG5	0.233	~amyloid_beta+(1|manifest)+cngeneson+pc_mito+brain_region+Sex	2.928	1592.000
ENSG00000023228	0.000	0.221	0.344	0.098	NDUFS1	0.004	~amyloid_beta+(1|manifest)+cngeneson+pc_mito+brain_region+Sex	1.000	4139.000
ENSG00000126705	0.001	0.221	0.460	-0.018	AHDC1	0.008	~amyloid_beta+(1|manifest)+cngeneson+pc_mito+brain_region+Sex	2.146	2266.000
ENSG00000173040	0.016	0.221	0.509	-0.067	EVC2	0.053	~amyloid_beta+(1|manifest)+cngeneson+pc_mito+brain_region+Sex	1.764	2730.000
ENSG00000100083	0.017	0.221	0.430	0.011	GGA1	0.054	~amyloid_beta+(1|manifest)+cngeneson+pc_mito+brain_region+Sex	3.726	1149.000
ENSG00000157833	0.080	0.220	0.619	-0.178	GAREM2	0.134	~amyloid_beta+(1|manifest)+cngeneson+pc_mito+brain_region+Sex	3.039	1521.000
ENSG00000183098	0.273	0.220	0.700	-0.260	GPC6	0.326	~amyloid_beta+(1|manifest)+cngeneson+pc_mito+brain_region+Sex	2.535	1871.000
ENSG00000171720	0.003	0.219	0.394	0.044	HDAC3	0.019	~amyloid_beta+(1|manifest)+cngeneson+pc_mito+brain_region+Sex	0.304	5726.000
ENSG00000160360	0.003	0.219	0.410	0.028	GPSM1	0.019	~amyloid_beta+(1|manifest)+cngeneson+pc_mito+brain_region+Sex	2.640	1781.000
ENSG00000110880	0.000	0.219	0.364	0.074	CORO1C	0.001	~amyloid_beta+(1|manifest)+cngeneson+pc_mito+brain_region+Sex	1.143	3832.000
ENSG00000122359	0.002	0.218	0.454	-0.018	ANXA11	0.015	~amyloid_beta+(1|manifest)+cngeneson+pc_mito+brain_region+Sex	2.157	2253.000
ENSG00000071051	0.001	0.218	0.362	0.075	NCK2	0.008	~amyloid_beta+(1|manifest)+cngeneson+pc_mito+brain_region+Sex	0.706	4860.000
ENSG00000122912	0.000	0.218	0.338	0.098	SLC25A16	0.002	~amyloid_beta+(1|manifest)+cngeneson+pc_mito+brain_region+Sex	0.428	5512.000
ENSG00000185070	0.391	0.218	0.698	-0.262	FLRT2	0.440	~amyloid_beta+(1|manifest)+cngeneson+pc_mito+brain_region+Sex	17.396	76.000
ENSG00000185722	0.000	0.218	0.349	0.087	ANKFY1	0.002	~amyloid_beta+(1|manifest)+cngeneson+pc_mito+brain_region+Sex	1.154	3805.000
ENSG00000125648	0.000	0.218	0.302	0.133	SLC25A23	0.000	~amyloid_beta+(1|manifest)+cngeneson+pc_mito+brain_region+Sex	0.560	5212.000
ENSG00000125868	0.000	0.217	0.347	0.088	DSTN	0.003	~amyloid_beta+(1|manifest)+cngeneson+pc_mito+brain_region+Sex	0.493	5381.000
ENSG00000102935	0.174	0.217	0.623	-0.188	ZNF423	0.229	~amyloid_beta+(1|manifest)+cngeneson+pc_mito+brain_region+Sex	6.816	488.000
ENSG00000129116	0.225	0.217	0.455	-0.021	PALLD	0.280	~amyloid_beta+(1|manifest)+cngeneson+pc_mito+brain_region+Sex	6.472	531.000
ENSG00000132361	0.019	0.217	0.456	-0.022	CLUH	0.057	~amyloid_beta+(1|manifest)+cngeneson+pc_mito+brain_region+Sex	1.455	3216.000
ENSG00000184619	0.000	0.217	0.356	0.078	KRBA2	0.003	~amyloid_beta+(1|manifest)+cngeneson+pc_mito+brain_region+Sex	1.117	3891.000
ENSG00000169992	0.037	0.217	0.525	-0.092	NLGN2	0.084	~amyloid_beta+(1|manifest)+cngeneson+pc_mito+brain_region+Sex	2.876	1622.000
ENSG00000088899	0.002	0.216	0.377	0.055	LZTS3	0.016	~amyloid_beta+(1|manifest)+cngeneson+pc_mito+brain_region+Sex	1.122	3884.000
ENSG00000159352	0.000	0.216	0.326	0.107	PSMD4	0.001	~amyloid_beta+(1|manifest)+cngeneson+pc_mito+brain_region+Sex	0.514	5318.000
ENSG00000113384	0.001	0.216	0.402	0.031	GOLPH3	0.011	~amyloid_beta+(1|manifest)+cngeneson+pc_mito+brain_region+Sex	0.494	5376.000
ENSG00000136448	0.019	0.216	0.480	-0.048	NMT1	0.058	~amyloid_beta+(1|manifest)+cngeneson+pc_mito+brain_region+Sex	3.396	1320.000
ENSG00000077549	0.001	0.215	0.358	0.073	CAPZB	0.010	~amyloid_beta+(1|manifest)+cngeneson+pc_mito+brain_region+Sex	1.948	2498.000
ENSG00000175806	0.270	0.215	0.510	-0.079	MSRA	0.324	~amyloid_beta+(1|manifest)+cngeneson+pc_mito+brain_region+Sex	8.150	365.000
ENSG00000114023	0.039	0.215	0.439	-0.009	FAM162A	0.086	~amyloid_beta+(1|manifest)+cngeneson+pc_mito+brain_region+Sex	1.105	3914.000
ENSG00000178074	0.000	0.215	0.345	0.085	C2orf69	0.003	~amyloid_beta+(1|manifest)+cngeneson+pc_mito+brain_region+Sex	0.098	5955.000
ENSG00000130158	0.001	0.215	0.392	0.038	DOCK6	0.013	~amyloid_beta+(1|manifest)+cngeneson+pc_mito+brain_region+Sex	1.607	2972.000
ENSG00000183255	0.030	0.214	0.519	-0.090	PTTG1IP	0.074	~amyloid_beta+(1|manifest)+cngeneson+pc_mito+brain_region+Sex	2.254	2148.000
ENSG00000174738	0.029	0.214	0.505	-0.077	NR1D2	0.072	~amyloid_beta+(1|manifest)+cngeneson+pc_mito+brain_region+Sex	1.889	2578.000
ENSG00000136451	0.004	0.214	0.393	0.035	VEZF1	0.023	~amyloid_beta+(1|manifest)+cngeneson+pc_mito+brain_region+Sex	0.218	5848.000
ENSG00000136238	0.001	0.214	0.342	0.086	RAC1	0.008	~amyloid_beta+(1|manifest)+cngeneson+pc_mito+brain_region+Sex	0.592	5148.000
ENSG00000148468	0.211	0.213	0.450	-0.023	[illegible]	0.266	~amyloid_beta+(1|manifest)+cngeneson+pc_mito+brain_region+Sex	7.128	456.000
ENSG00000156273	0.009	0.213	0.451	-0.025	BACH1	0.037	~amyloid_beta+(1|manifest)+cngeneson+pc_mito+brain_region+Sex	2.256	2147.000
ENSG00000169696	0.008	0.213	0.443	-0.018	ASPSCR1	0.035	~amyloid_beta+(1|manifest)+cngeneson+pc_mito+brain_region+Sex	2.919	1596.000
ENSG00000165516	0.000	0.212	0.323	0.102	KLHDC2	0.002	~amyloid_beta+(1|manifest)+cngeneson+pc_mito+brain_region+Sex	1.179	3746.000
ENSG00000102572	0.013	0.212	0.516	-0.093	STK24	0.047	~amyloid_beta+(1|manifest)+cngeneson+pc_mito+brain_region+Sex	2.327	2072.000
ENSG00000173960	0.000	0.212	0.310	0.114	UBXN2A	0.001	~amyloid_beta+(1|manifest)+cngeneson+pc_mito+brain_region+Sex	0.662	4975.000
ENSG00000006831	0.000	0.212	0.359	0.065	ADIPOR2	0.004	~amyloid_beta+(1|manifest)+cngeneson+pc_mito+brain_region+Sex	1.078	3969.000
ENSG00000137266	0.009	0.212	0.448	-0.025	SLC22A23	0.037	~amyloid_beta+(1|manifest)+cngeneson+pc_mito+brain_region+Sex	3.510	1247.000
ENSG00000178719	0.109	0.212	0.589	-0.165	GRINA	0.165	~amyloid_beta+(1|manifest)+cngeneson+pc_mito+brain_region+Sex	2.375	2023.000
ENSG00000198853	0.000	0.211	0.325	0.098	RUSC2	0.001	~amyloid_beta+(1|manifest)+cngeneson+pc_mito+brain_region+Sex	0.717	4828.000
ENSG00000139800	0.003	0.211	0.381	0.042	ZIC5	0.018	~amyloid_beta+(1|manifest)+cngeneson+pc_mito+brain_region+Sex	0.819	4573.000
ENSG00000140941	0.001	0.211	0.350	0.072	MAP1LC3B	0.010	~amyloid_beta+(1|manifest)+cngeneson+pc_mito+brain_region+Sex	0.406	5555.000
ENSG00000120727	0.000	0.211	0.340	0.082	PAIP2	0.006	~amyloid_beta+(1|manifest)+cngeneson+pc_mito+brain_region+Sex	0.709	4850.000
ENSG00000160190	0.001	0.211	0.376	0.046	SLC37A1	0.009	~amyloid_beta+(1|manifest)+cngeneson+pc_mito+brain_region+Sex	1.025	4090.000
ENSG00000203879	0.006	0.211	0.409	0.012	GDI1	0.029	~amyloid_beta+(1|manifest)+cngeneson+pc_mito+brain_region+Sex	0.988	4170.000
ENSG00000102858	0.000	0.211	0.346	0.075	MGRN1	0.004	~amyloid_beta+(1|manifest)+cngeneson+pc_mito+brain_region+Sex	1.248	3604.000
ENSG00000156639	0.005	0.210	0.332	0.089	ZFAND3	0.028	~amyloid_beta+(1|manifest)+cngeneson+pc_mito+brain_region+Sex	2.889	1612.000
ENSG00000162496	0.016	0.210	0.516	-0.095	DHRS3	0.052	~amyloid_beta+(1|manifest)+cngeneson+pc_mito+brain_region+Sex	2.801	1666.000
ENSG00000089486	0.078	0.210	0.532	-0.111	CDIP1	0.132	~amyloid_beta+(1|manifest)+cngeneson+pc_mito+brain_region+Sex	2.379	2018.000
ENSG00000087842	0.102	0.209	0.516	-0.097	PIR	0.157	~amyloid_beta+(1|manifest)+cngeneson+pc_mito+brain_region+Sex	1.133	3854.000
ENSG00000105701	0.003	0.209	0.396	0.022	FKBP8	0.021	~amyloid_beta+(1|manifest)+cngeneson+pc_mito+brain_region+Sex	1.990	2447.000
ENSG00000138834	0.027	0.209	0.472	-0.055	MAPK8IP3	0.070	~amyloid_beta+(1|manifest)+cngeneson+pc_mito+brain_region+Sex	4.551	877.000
ENSG00000001561	0.001	0.208	0.417	0.000	ENPP4	0.009	~amyloid_beta+(1|manifest)+cngeneson+pc_mito+brain_region+Sex	0.620	5084.000
ENSG00000154832	0.012	0.208	0.474	-0.057	CXXC1	0.044	~amyloid_beta+(1|manifest)+cngeneson+pc_mito+brain_region+Sex	3.148	1455.000
ENSG00000108100	0.048	0.208	0.420	-0.004	CCNY	0.097	~amyloid_beta+(1|manifest)+cngeneson+pc_mito+brain_region+Sex	1.443	3242.000
ENSG00000074855	0.260	0.207	0.714	-0.300	ANO8	0.314	~amyloid_beta+(1|manifest)+cngeneson+pc_mito+brain_region+Sex	10.027	268.000
ENSG00000143842	0.006	0.207	0.406	0.008	SOX13	0.029	~amyloid_beta+(1|manifest)+cngeneson+pc_mito+brain_region+Sex	2.157	2252.000
ENSG00000029725	0.002	0.206	0.371	0.041	RABEP1	0.015	~amyloid_beta+(1|manifest)+cngeneson+pc_mito+brain_region+Sex	1.849	2628.000
ENSG00000008838	0.001	0.206	0.354	0.058	MED24	0.013	~amyloid_beta+(1|manifest)+cngeneson+pc_mito+brain_region+Sex	1.265	3568.000
ENSG00000105520	0.006	0.206	0.396	0.016	PLPPR2	0.029	~amyloid_beta+(1|manifest)+cngeneson+pc_mito+brain_region+Sex	1.230	3642.000
ENSG00000076555	0.306	0.206	0.482	-0.070	ACACB	0.359	~amyloid_beta+(1|manifest)+cngeneson+pc_mito+brain_region+Sex	14.062	122.000
ENSG00000166949	0.042	0.206	0.532	-0.120	SMAD3	0.089	~amyloid_beta+(1|manifest)+cngeneson+pc_mito+brain_region+Sex	2.877	1618.000
ENSG00000163902	0.002	0.206	0.456	-0.045	RPN1	0.017	~amyloid_beta+(1|manifest)+cngeneson+pc_mito+brain_region+Sex	2.389	2001.000
ENSG00000147526	0.001	0.206	0.366	0.045	TACC1	0.013	~amyloid_beta+(1|manifest)+cngeneson+pc_mito+brain_region+Sex	1.733	2776.000
ENSG00000185305	0.002	0.206	0.357	0.054	ARL15	0.017	~amyloid_beta+(1|manifest)+cngeneson+pc_mito+brain_region+Sex	2.584	1824.000
ENSG00000101558	0.009	0.205	0.441	-0.030	VAPA	0.037	~amyloid_beta+(1|manifest)+cngeneson+pc_mito+brain_region+Sex	0.324	5687.000
ENSG00000167526	0.005	0.205	0.449	-0.038	RPL13	0.028	~amyloid_beta+(1|manifest)+cngeneson+pc_mito+brain_region+Sex	2.414	1967.000
ENSG00000058453	0.023	0.205	0.429	-0.019	CROCC	0.063	~amyloid_beta+(1|manifest)+cngeneson+pc_mito+brain_region+Sex	1.418	3296.000
ENSG00000117592	0.229	0.205	0.624	-0.214	PRDX6	0.283	~amyloid_beta+(1|manifest)+cngeneson+pc_mito+brain_region+Sex	4.587	866.000
ENSG00000134531	0.775	0.205	0.810	-0.401	EMP1	0.797	~amyloid_beta+(1|manifest)+cngeneson+pc_mito+brain_region+Sex	18.308	65.000
ENSG00000143742	0.010	0.204	0.425	-0.017	SRP9	0.040	~amyloid_beta+(1|manifest)+cngeneson+pc_mito+brain_region+Sex	1.168	3770.000
ENSG00000173041	0.091	0.204	0.525	-0.117	ZNF680	0.147	~amyloid_beta+(1|manifest)+cngeneson+pc_mito+brain_region+Sex	1.284	3536.000
ENSG00000141503	0.039	0.204	0.493	-0.086	MINK1	0.086	~amyloid_beta+(1|manifest)+cngeneson+pc_mito+brain_region+Sex	5.031	766.000
ENSG00000100142	0.000	0.204	0.326	0.082	POLR2F	0.006	~amyloid_beta+(1|manifest)+cngeneson+pc_mito+brain_region+Sex	0.928	4311.000
ENSG00000179364	0.002	0.203	0.356	0.051	PACS2	0.017	~amyloid_beta+(1|manifest)+cngeneson+pc_mito+brain_region+Sex	2.415	1965.000
ENSG00000100968	0.144	0.203	0.590	-0.184	NFATC4	0.200	~amyloid_beta+(1|manifest)+cngeneson+pc_mito+brain_region+Sex	3.814	1115.000
ENSG00000156931	0.001	0.203	0.339	0.067	VPS8	0.013	~amyloid_beta+(1|manifest)+cngeneson+pc_mito+brain_region+Sex	1.643	2918.000
ENSG00000138835	0.003	0.203	0.423	-0.018	RGS3	0.021	~amyloid_beta+(1|manifest)+cngeneson+pc_mito+brain_region+Sex	2.022	2408.000
ENSG00000186591	0.173	0.203	0.514	-0.109	UBE2H	0.228	~amyloid_beta+(1|manifest)+cngeneson+pc_mito+brain_region+Sex	3.823	1113.000
ENSG00000126005	0.005	0.203	0.412	-0.007	[illegible]	0.027	~amyloid_beta+(1|manifest)+cngeneson+pc_mito+brain_region+Sex	2.399	1986.000
ENSG00000015475	0.000	0.202	0.351	0.053	BID	0.003	~amyloid_beta+(1|manifest)+cngeneson+pc_mito+brain_region+Sex	0.307	5719.000
ENSG00000129158	0.482	0.202	0.661	-0.257	SERGEF	0.526	~amyloid_beta+(1|manifest)+cngeneson+pc_mito+brain_region+Sex	7.721	396.000
ENSG00000124422	0.001	0.202	0.340	0.064	USP22	0.010	~amyloid_beta+(1|manifest)+cngeneson+pc_mito+brain_region+Sex	0.953	4246.000
ENSG00000157350	0.002	0.202	0.364	0.040	ST3GAL2	0.015	~amyloid_beta+(1|manifest)+cngeneson+pc_mito+brain_region+Sex	0.797	4639.000
ENSG00000131095	0.586	0.201	0.647	-0.244	GFAP	0.623	~amyloid_beta+(1|manifest)+cngeneson+pc_mito+brain_region+Sex	33.472	15.000
ENSG00000135390	0.024	0.201	0.483	-0.082	ATP5MC2	0.066	~amyloid_beta+(1|manifest)+cngeneson+pc_mito+brain_region+Sex	5.632	659.000
ENSG00000134548	0.003	0.201	0.462	-0.060	SPX	0.019	~amyloid_beta+(1|manifest)+cngeneson+pc_mito+brain_region+Sex	1.516	3110.000
ENSG00000084652	0.000	0.201	0.306	0.096	TXLNA	0.003	~amyloid_beta+(1|manifest)+cngeneson+pc_mito+brain_region+Sex	0.489	5385.000
ENSG00000213741	0.003	0.201	0.355	0.046	RPS29	0.020	~amyloid_beta+(1|manifest)+cngeneson+pc_mito+brain_region+Sex	0.430	5509.000
ENSG00000128487	0.137	0.200	0.558	-0.158	SPECC1	0.194	~amyloid_beta+(1|manifest)+cngeneson+pc_mito+brain_region+Sex	2.882	1614.000
ENSG00000104760	0.100	0.200	0.566	-0.165	FGL1	0.156	~amyloid_beta+(1|manifest)+cngeneson+pc_mito+brain_region+Sex	4.263	958.000
ENSG00000146373	0.004	0.200	0.421	-0.020	RNF217	0.024	~amyloid_beta+(1|manifest)+cngeneson+pc_mito+brain_region+Sex	1.177	3754.000
ENSG00000076356	0.205	0.200	0.558	-0.158	PLXNA2	0.260	~amyloid_beta+(1|manifest)+cngeneson+pc_mito+brain_region+Sex	5.465	681.000
ENSG00000255690	0.260	0.200	0.721	-0.320	TRIL	0.314	~amyloid_beta+(1|manifest)+cngeneson+pc_mito+brain_region+Sex	7.369	430.000
ENSG00000163485	0.060	0.200	0.514	-0.115	ADORA1	0.112	~amyloid_beta+(1|manifest)+cngeneson+pc_mito+brain_region+Sex	1.856	2619.000
ENSG00000183648	0.014	0.199	0.459	-0.060	NDUFB1	0.048	~amyloid_beta+(1|manifest)+cngeneson+pc_mito+brain_region+Sex	0.824	4556.000
ENSG00000115548	0.040	0.199	0.406	-0.007	KDM3A	0.087	~amyloid_beta+(1|manifest)+cngeneson+pc_mito+brain_region+Sex	1.522	3103.000
ENSG00000129946	0.046	0.199	0.496	-0.097	SHC2	0.095	~amyloid_beta+(1|manifest)+cngeneson+pc_mito+brain_region+Sex	5.010	772.000
ENSG00000124226	0.001	0.199	0.321	0.077	RNF114	0.008	~amyloid_beta+(1|manifest)+cngeneson+pc_mito+brain_region+Sex	0.762	4712.000
ENSG00000164038	0.002	0.199	0.464	-0.066	SLC9B2	0.016	~amyloid_beta+(1|manifest)+cngeneson+pc_mito+brain_region+Sex	3.607	1203.000
ENSG00000072682	0.000	0.199	0.351	0.047	P4HA2	0.003	~amyloid_beta+(1|manifest)+cngeneson+pc_mito+brain_region+Sex	0.150	5922.000
ENSG00000160310	0.006	0.199	0.425	-0.027	PRMT2	0.030	~amyloid_beta+(1|manifest)+cngeneson+pc_mito+brain_region+Sex	1.494	3146.000
ENSG00000184384	0.004	0.199	0.351	0.046	MAML2	0.025	~amyloid_beta+(1|manifest)+cngeneson+pc_mito+brain_region+Sex	4.307	948.000
ENSG00000104973	0.002	0.199	0.388	0.010	MED25	0.016	~amyloid_beta+(1|manifest)+cngeneson+pc_mito+brain_region+Sex	0.731	4791.000
ENSG00000166046	0.000	0.199	0.325	0.073	TCP11L2	0.001	~amyloid_beta+(1|manifest)+cngeneson+pc_mito+brain_region+Sex	0.446	5462.000
ENSG00000141568	0.002	0.199	0.361	0.037	FOXK2	0.015	~amyloid_beta+(1|manifest)+cngeneson+pc_mito+brain_region+Sex	1.389	3339.000
ENSG00000060237	0.012	0.199	0.394	0.003	WNK1	0.044	~amyloid_beta+(1|manifest)+cngeneson+pc_mito+brain_region+Sex	2.381	2014.000
ENSG00000111640	0.328	0.198	0.594	-0.198	GAPDH	0.380	~amyloid_beta+(1|manifest)+cngeneson+pc_mito+brain_region+Sex	6.178	566.000
ENSG00000205268	0.006	0.198	0.434	-0.038	PDE7A	0.030	~amyloid_beta+(1|manifest)+cngeneson+pc_mito+brain_region+Sex	2.117	2292.000
ENSG00000170145	0.053	0.197	0.379	0.016	SIK2	0.103	~amyloid_beta+(1|manifest)+cngeneson+pc_mito+brain_region+Sex	3.256	1391.000
ENSG00000139684	0.003	0.197	0.386	0.008	ESD	0.021	~amyloid_beta+(1|manifest)+cngeneson+pc_mito+brain_region+Sex	0.255	5800.000
ENSG00000168913	0.117	0.197	0.643	-0.250	ENHO	0.174	~amyloid_beta+(1|manifest)+cngeneson+pc_mito+brain_region+Sex	3.340	1347.000
ENSG00000082641	0.004	0.197	0.384	0.009	NFE2L1	0.025	~amyloid_beta+(1|manifest)+cngeneson+pc_mito+brain_region+Sex	0.772	4686.000

ENSG00000166165	0.540	0.196	0.578	-0.185	CKB	0.581	~amyloid_beta+(1|manifest)+cngeneson+pc_mito+brain_region+Sex	19.743	54.000
ENSG00000100422	0.000	0.196	0.335	0.057	CERK	0.007	~amyloid_beta+(1|manifest)+cngeneson+pc_mito+brain_region+Sex	0.487	5390.000
ENSG00000134318	0.264	0.196	0.555	-0.162	ROCK2	0.318	~amyloid_beta+(1|manifest)+cngeneson+pc_mito+brain_region+Sex	7.392	426.000
ENSG00000198130	0.237	0.196	0.445	-0.052	HIBCH	0.291	~amyloid_beta+(1|manifest)+cngeneson+pc_mito+brain_region+Sex	4.170	993.000
ENSG00000119559	0.005	0.196	0.361	0.030	C19orf25	0.027	~amyloid_beta+(1|manifest)+cngeneson+pc_mito+brain_region+Sex	2.106	2304.000
ENSG00000075624	0.205	0.196	0.681	-0.289	ACTB	0.260	~amyloid_beta+(1|manifest)+cngeneson+pc_mito+brain_region+Sex	6.869	481.000
ENSG00000156515	0.001	0.196	0.438	-0.047	HK1	0.013	~amyloid_beta+(1|manifest)+cngeneson+pc_mito+brain_region+Sex	2.312	2088.000
ENSG00000115738	0.579	0.195	0.568	-0.177	ID2	0.617	~amyloid_beta+(1|manifest)+cngeneson+pc_mito+brain_region+Sex	4.018	1036.000
ENSG00000129595	0.073	0.195	0.493	-0.102	EPB41L4A	0.126	~amyloid_beta+(1|manifest)+cngeneson+pc_mito+brain_region+Sex	0.692	4913.000
ENSG00000132388	0.000	0.195	0.344	0.046	UBE2G1	0.004	~amyloid_beta+(1|manifest)+cngeneson+pc_mito+brain_region+Sex	0.867	4452.000
ENSG00000186654	0.002	0.195	0.402	-0.012	PRR5	0.016	~amyloid_beta+(1|manifest)+cngeneson+pc_mito+brain_region+Sex	1.162	3786.000
ENSG00000119943	0.001	0.195	0.345	0.044	PYROXD2	0.013	~amyloid_beta+(1|manifest)+cngeneson+pc_mito+brain_region+Sex	1.133	3855.000
ENSG00000123178	0.002	0.194	0.357	0.032	SPRYD7	0.016	~amyloid_beta+(1|manifest)+cngeneson+pc_mito+brain_region+Sex	0.209	5862.000
ENSG00000163605	0.004	0.194	0.389	-0.001	PPP4R2	0.023	~amyloid_beta+(1|manifest)+cngeneson+pc_mito+brain_region+Sex	0.955	4240.000
ENSG00000103995	0.000	0.194	0.383	0.005	CEP152	0.006	~amyloid_beta+(1|manifest)+cngeneson+pc_mito+brain_region+Sex	1.629	2940.000
ENSG00000143376	0.001	0.194	0.342	0.046	SNX27	0.009	~amyloid_beta+(1|manifest)+cngeneson+pc_mito+brain_region+Sex	0.644	5021.000
ENSG00000089335	0.008	0.194	0.392	-0.004	ZNF302	0.035	~amyloid_beta+(1|manifest)+cngeneson+pc_mito+brain_region+Sex	2.083	2332.000
ENSG00000187098	0.002	0.194	0.352	0.036	MITF	0.015	~amyloid_beta+(1|manifest)+cngeneson+pc_mito+brain_region+Sex	1.050	4024.000
ENSG00000196642	0.014	0.194	0.428	-0.041	RABL6	0.048	~amyloid_beta+(1|manifest)+cngeneson+pc_mito+brain_region+Sex	2.448	1937.000
ENSG00000187109	0.017	0.194	0.437	-0.049	NAP1L1	0.054	~amyloid_beta+(1|manifest)+cngeneson+pc_mito+brain_region+Sex	3.188	1432.000
ENSG00000140416	0.009	0.194	0.482	-0.095	TPM1	0.038	~amyloid_beta+(1|manifest)+cngeneson+pc_mito+brain_region+Sex	1.160	3789.000
ENSG00000108557	0.005	0.193	0.377	0.010	RAI1	0.028	~amyloid_beta+(1|manifest)+cngeneson+pc_mito+brain_region+Sex	0.504	5343.000
ENSG00000095397	0.013	0.193	0.498	-0.111	WHRN	0.046	~amyloid_beta+(1|manifest)+cngeneson+pc_mito+brain_region+Sex	1.393	3334.000
ENSG00000116667	0.026	0.193	0.329	0.057	C1orf21	0.068	~amyloid_beta+(1|manifest)+cngeneson+pc_mito+brain_region+Sex	1.074	3979.000
ENSG00000165801	0.022	0.193	0.418	-0.032	ARHGEF40	0.063	~amyloid_beta+(1|manifest)+cngeneson+pc_mito+brain_region+Sex	1.070	3987.000
ENSG00000008130	0.002	0.193	0.370	0.016	NADK	0.017	~amyloid_beta+(1|manifest)+cngeneson+pc_mito+brain_region+Sex	0.308	5712.000
ENSG00000181790	0.121	0.193	0.725	-0.339	ADGRB1	0.178	~amyloid_beta+(1|manifest)+cngeneson+pc_mito+brain_region+Sex	5.711	647.000
ENSG00000107745	0.006	0.193	0.392	-0.007	MICU1	0.028	~amyloid_beta+(1|manifest)+cngeneson+pc_mito+brain_region+Sex	0.319	5692.000
ENSG00000104969	0.006	0.193	0.375	0.010	SGTA	0.030	~amyloid_beta+(1|manifest)+cngeneson+pc_mito+brain_region+Sex	1.726	2787.000
ENSG00000167552	0.038	0.193	0.566	-0.181	TUBA1A	0.086	~amyloid_beta+(1|manifest)+cngeneson+pc_mito+brain_region+Sex	1.529	3088.000
ENSG00000109929	0.000	0.192	0.317	0.067	SC5D	0.004	~amyloid_beta+(1|manifest)+cngeneson+pc_mito+brain_region+Sex	0.647	5011.000
ENSG00000198089	0.000	0.192	0.317	0.067	SFI1	0.003	~amyloid_beta+(1|manifest)+cngeneson+pc_mito+brain_region+Sex	1.037	4053.000
ENSG00000120885	0.265	0.191	0.442	-0.059	CLU	0.318	~amyloid_beta+(1|manifest)+cngeneson+pc_mito+brain_region+Sex	13.322	143.000
ENSG00000141298	0.003	0.191	0.348	0.034	SSH2	0.019	~amyloid_beta+(1|manifest)+cngeneson+pc_mito+brain_region+Sex	1.308	3485.000
ENSG00000185716	0.003	0.191	0.373	0.009	MOSMO	0.021	~amyloid_beta+(1|manifest)+cngeneson+pc_mito+brain_region+Sex	0.796	4642.000
ENSG00000147316	0.156	0.191	0.562	-0.181	MCPH1	0.211	~amyloid_beta+(1|manifest)+cngeneson+pc_mito+brain_region+Sex	1.110	3909.000
ENSG00000138735	0.140	0.191	0.626	-0.244	PDE5A	0.197	~amyloid_beta+(1|manifest)+cngeneson+pc_mito+brain_region+Sex	2.377	2022.000
ENSG00000173153	0.005	0.191	0.382	-0.001	ESRRA	0.027	~amyloid_beta+(1|manifest)+cngeneson+pc_mito+brain_region+Sex	0.746	4753.000
ENSG00000137409	0.176	0.190	0.589	-0.208	MTCH1	0.231	~amyloid_beta+(1|manifest)+cngeneson+pc_mito+brain_region+Sex	4.546	884.000
ENSG00000162032	0.066	0.190	0.523	-0.143	SPSB3	0.119	~amyloid_beta+(1|manifest)+cngeneson+pc_mito+brain_region+Sex	4.403	915.000
ENSG00000141542	0.008	0.190	0.387	-0.007	RAB40B	0.035	~amyloid_beta+(1|manifest)+cngeneson+pc_mito+brain_region+Sex	0.440	5485.000
ENSG00000148200	0.110	0.190	0.404	-0.025	NR6A1	0.166	~amyloid_beta+(1|manifest)+cngeneson+pc_mito+brain_region+Sex	0.125	5935.000
ENSG00000176261	0.005	0.189	0.366	0.013	ZBTB8OS	0.025	~amyloid_beta+(1|manifest)+cngeneson+pc_mito+brain_region+Sex	1.225	3655.000
ENSG00000070759	0.152	0.189	0.546	-0.167	TESK2	0.207	~amyloid_beta+(1|manifest)+cngeneson+pc_mito+brain_region+Sex	1.959	2480.000
ENSG00000138801	0.008	0.189	0.404	-0.025	PAPSS1	0.034	~amyloid_beta+(1|manifest)+cngeneson+pc_mito+brain_region+Sex	0.445	5463.000
ENSG00000184792	0.009	0.189	0.360	0.018	OSBP2	0.038	~amyloid_beta+(1|manifest)+cngeneson+pc_mito+brain_region+Sex	0.958	4228.000
ENSG00000104267	0.001	0.189	0.315	0.063	CA2	0.010	~amyloid_beta+(1|manifest)+cngeneson+pc_mito+brain_region+Sex	0.545	5245.000
ENSG00000100266	0.001	0.189	0.351	0.027	PACSIN2	0.009	~amyloid_beta+(1|manifest)+cngeneson+pc_mito+brain_region+Sex	1.809	2681.000
ENSG00000139971	0.025	0.189	0.376	0.002	ARMH4	0.067	~amyloid_beta+(1|manifest)+cngeneson+pc_mito+brain_region+Sex	3.181	1436.000
ENSG00000120053	0.000	0.189	0.310	0.068	GOT1	0.005	~amyloid_beta+(1|manifest)+cngeneson+pc_mito+brain_region+Sex	0.869	4442.000
ENSG00000169762	0.000	0.189	0.333	0.044	TAPT1	0.005	~amyloid_beta+(1|manifest)+cngeneson+pc_mito+brain_region+Sex	1.505	3131.000
ENSG00000135069	0.082	0.188	0.553	-0.176	PSAT1	0.137	~amyloid_beta+(1|manifest)+cngeneson+pc_mito+brain_region+Sex	2.434	1953.000
ENSG00000229117	0.084	0.188	0.584	-0.208	RPL41	0.139	~amyloid_beta+(1|manifest)+cngeneson+pc_mito+brain_region+Sex	2.768	1693.000
ENSG00000110321	0.006	0.188	0.356	0.020	EIF4G2	0.029	~amyloid_beta+(1|manifest)+cngeneson+pc_mito+brain_region+Sex	0.496	5372.000
ENSG00000135097	0.015	0.187	0.409	-0.034	MSI1	0.049	~amyloid_beta+(1|manifest)+cngeneson+pc_mito+brain_region+Sex	1.978	2459.000
ENSG00000196208	0.085	0.187	0.482	-0.108	GREB1	0.140	~amyloid_beta+(1|manifest)+cngeneson+pc_mito+brain_region+Sex	3.892	1092.000
ENSG00000138641	0.002	0.187	0.356	0.019	HERC3	0.017	~amyloid_beta+(1|manifest)+cngeneson+pc_mito+brain_region+Sex	0.618	5090.000
ENSG00000140332	0.000	0.187	0.324	0.050	TLE3	0.007	~amyloid_beta+(1|manifest)+cngeneson+pc_mito+brain_region+Sex	0.053	5974.000
ENSG00000128951	0.009	0.187	0.363	0.011	DUT	0.037	~amyloid_beta+(1|manifest)+cngeneson+pc_mito+brain_region+Sex	1.206	3690.000
ENSG00000130702	0.052	0.187	0.519	-0.145	LAMA5	0.102	~amyloid_beta+(1|manifest)+cngeneson+pc_mito+brain_region+Sex	3.436	1288.000
ENSG00000181852	0.002	0.186	0.338	0.035	RNF41	0.015	~amyloid_beta+(1|manifest)+cngeneson+pc_mito+brain_region+Sex	0.705	4863.000
ENSG00000090661	0.005	0.186	0.392	-0.019	CERS4	0.028	~amyloid_beta+(1|manifest)+cngeneson+pc_mito+brain_region+Sex	1.806	2685.000
ENSG00000157933	0.009	0.186	0.385	-0.012	SKI	0.037	~amyloid_beta+(1|manifest)+cngeneson+pc_mito+brain_region+Sex	0.410	5548.000
ENSG00000008083	0.094	0.186	0.357	0.015	JARID2	0.149	~amyloid_beta+(1|manifest)+cngeneson+pc_mito+brain_region+Sex	8.383	355.000
ENSG00000166681	0.040	0.186	0.455	-0.083	BEX3	0.087	~amyloid_beta+(1|manifest)+cngeneson+pc_mito+brain_region+Sex	1.777	2718.000
ENSG00000158411	0.000	0.186	0.311	0.061	MITD1	0.006	~amyloid_beta+(1|manifest)+cngeneson+pc_mito+brain_region+Sex	0.820	4570.000
ENSG00000107130	0.018	0.186	0.422	-0.050	NCS1	0.056	~amyloid_beta+(1|manifest)+cngeneson+pc_mito+brain_region+Sex	1.642	2920.000
ENSG00000138385	0.001	0.186	0.301	0.070	SSB	0.009	~amyloid_beta+(1|manifest)+cngeneson+pc_mito+brain_region+Sex	0.392	5575.000
ENSG00000137269	0.019	0.185	0.422	-0.051	LRRC1	0.057	~amyloid_beta+(1|manifest)+cngeneson+pc_mito+brain_region+Sex	1.448	3233.000
ENSG00000160801	0.024	0.185	0.450	-0.079	PTH1R	0.066	~amyloid_beta+(1|manifest)+cngeneson+pc_mito+brain_region+Sex	3.143	1457.000
ENSG00000119772	0.009	0.185	0.408	-0.038	DNMT3A	0.038	~amyloid_beta+(1|manifest)+cngeneson+pc_mito+brain_region+Sex	1.323	3457.000
ENSG00000126214	0.012	0.185	0.325	0.044	KLC1	0.043	~amyloid_beta+(1|manifest)+cngeneson+pc_mito+brain_region+Sex	2.090	2325.000
ENSG00000001084	0.072	0.185	0.444	-0.074	GCLC	0.126	~amyloid_beta+(1|manifest)+cngeneson+pc_mito+brain_region+Sex	0.795	4644.000
ENSG00000134215	0.276	0.184	0.821	-0.452	VAV3	0.329	~amyloid_beta+(1|manifest)+cngeneson+pc_mito+brain_region+Sex	5.842	617.000
ENSG00000184304	0.141	0.184	0.359	0.009	PRKD1	0.197	~amyloid_beta+(1|manifest)+cngeneson+pc_mito+brain_region+Sex	4.456	904.000
ENSG00000087053	0.000	0.184	0.391	-0.023	MTMR2	0.004	~amyloid_beta+(1|manifest)+cngeneson+pc_mito+brain_region+Sex	1.422	3287.000
ENSG00000111666	0.026	0.184	0.310	0.058	CHPT1	0.069	~amyloid_beta+(1|manifest)+cngeneson+pc_mito+brain_region+Sex	3.238	1403.000
ENSG00000118816	0.002	0.184	0.317	0.051	CCNI	0.018	~amyloid_beta+(1|manifest)+cngeneson+pc_mito+brain_region+Sex	0.818	4577.000
ENSG00000168843	0.331	0.184	0.786	-0.419	FSTL5	0.383	~amyloid_beta+(1|manifest)+cngeneson+pc_mito+brain_region+Sex	5.880	610.000
ENSG00000101096	0.003	0.184	0.401	-0.033	NFATC2	0.019	~amyloid_beta+(1|manifest)+cngeneson+pc_mito+brain_region+Sex	0.614	5102.000
ENSG00000148482	0.091	0.184	0.414	-0.046	SLC39A12	0.147	~amyloid_beta+(1|manifest)+cngeneson+pc_mito+brain_region+Sex	1.939	2509.000
ENSG00000141959	0.099	0.184	0.563	-0.196	PFKL	0.155	~amyloid_beta+(1|manifest)+cngeneson+pc_mito+brain_region+Sex	8.648	333.000
ENSG00000131771	0.191	0.184	0.617	-0.249	PPP1R1B	0.246	~amyloid_beta+(1|manifest)+cngeneson+pc_mito+brain_region+Sex	5.304	710.000
ENSG00000145439	0.003	0.183	0.326	0.041	CBR4	0.020	~amyloid_beta+(1|manifest)+cngeneson+pc_mito+brain_region+Sex	3.130	1464.000
ENSG00000114770	0.009	0.183	0.344	0.023	ABCC5	0.037	~amyloid_beta+(1|manifest)+cngeneson+pc_mito+brain_region+Sex	0.546	5238.000
ENSG00000178764	0.291	0.183	0.411	-0.045	ZHX2	0.343	~amyloid_beta+(1|manifest)+cngeneson+pc_mito+brain_region+Sex	9.396	303.000
ENSG00000072210	0.000	0.183	0.330	0.036	ALDH3A2	0.006	~amyloid_beta+(1|manifest)+cngeneson+pc_mito+brain_region+Sex	0.070	5967.000
ENSG00000120690	0.004	0.183	0.389	-0.024	ELF1	0.025	~amyloid_beta+(1|manifest)+cngeneson+pc_mito+brain_region+Sex	2.762	1696.000
ENSG00000075413	0.000	0.183	0.265	0.101	MARK3	0.007	~amyloid_beta+(1|manifest)+cngeneson+pc_mito+brain_region+Sex	2.020	2411.000
ENSG00000155974	0.136	0.183	0.551	-0.186	GRIP1	0.192	~amyloid_beta+(1|manifest)+cngeneson+pc_mito+brain_region+Sex	3.289	1379.000
ENSG00000040933	0.002	0.182	0.388	-0.023	INPP4A	0.016	~amyloid_beta+(1|manifest)+cngeneson+pc_mito+brain_region+Sex	0.769	4693.000
ENSG00000196850	0.000	0.182	0.354	0.010	PPTC7	0.007	~amyloid_beta+(1|manifest)+cngeneson+pc_mito+brain_region+Sex	1.074	3982.000
ENSG00000083067	0.270	0.182	0.432	-0.068	TRPM3	0.324	~amyloid_beta+(1|manifest)+cngeneson+pc_mito+brain_region+Sex	24.787	30.000
ENSG00000128989	0.004	0.182	0.334	0.031	ARPP19	0.024	~amyloid_beta+(1|manifest)+cngeneson+pc_mito+brain_region+Sex	0.801	4627.000
ENSG00000141279	0.012	0.182	0.354	0.010	NPEPPS	0.045	~amyloid_beta+(1|manifest)+cngeneson+pc_mito+brain_region+Sex	4.210	979.000
ENSG00000008018	0.000	0.182	0.274	0.090	PSMB1	0.001	~amyloid_beta+(1|manifest)+cngeneson+pc_mito+brain_region+Sex	0.073	5966.000
ENSG00000052802	0.001	0.181	0.392	-0.029	MSMO1	0.012	~amyloid_beta+(1|manifest)+cngeneson+pc_mito+brain_region+Sex	2.525	1875.000
ENSG00000160305	0.006	0.181	0.379	-0.017	DIP2A	0.030	~amyloid_beta+(1|manifest)+cngeneson+pc_mito+brain_region+Sex	0.648	5010.000
ENSG00000104980	0.008	0.181	0.350	0.012	TIMM44	0.034	~amyloid_beta+(1|manifest)+cngeneson+pc_mito+brain_region+Sex	0.825	4553.000
ENSG00000164684	0.003	0.181	0.425	-0.063	ZNF704	0.021	~amyloid_beta+(1|manifest)+cngeneson+pc_mito+brain_region+Sex	0.906	4366.000
ENSG00000122741	0.001	0.181	0.285	0.077	DCAF10	0.008	~amyloid_beta+(1|manifest)+cngeneson+pc_mito+brain_region+Sex	0.266	5787.000
ENSG00000118997	0.396	0.181	0.627	-0.266	DNAH7	0.444	~amyloid_beta+(1|manifest)+cngeneson+pc_mito+brain_region+Sex	6.213	561.000
ENSG00000185787	0.001	0.181	0.332	0.029	MORF4L1	0.012	~amyloid_beta+(1|manifest)+cngeneson+pc_mito+brain_region+Sex	1.249	3601.000
ENSG00000101577	0.112	0.181	0.502	-0.140	LPIN2	0.168	~amyloid_beta+(1|manifest)+cngeneson+pc_mito+brain_region+Sex	1.200	3705.000
ENSG00000102878	0.087	0.181	0.559	-0.198	HSF4	0.142	~amyloid_beta+(1|manifest)+cngeneson+pc_mito+brain_region+Sex	5.746	637.000
ENSG00000184787	0.012	0.181	0.329	0.032	UBE2G2	0.043	~amyloid_beta+(1|manifest)+cngeneson+pc_mito+brain_region+Sex	2.666	1757.000
ENSG00000153786	0.000	0.180	0.277	0.084	ZDHHC7	0.002	~amyloid_beta+(1|manifest)+cngeneson+pc_mito+brain_region+Sex	0.032	5977.000
ENSG00000173064	0.001	0.180	0.299	0.061	HECTD4	0.007	~amyloid_beta+(1|manifest)+cngeneson+pc_mito+brain_region+Sex	1.725	2788.000
ENSG00000136720	0.002	0.180	0.306	0.055	HS6ST1	0.017	~amyloid_beta+(1|manifest)+cngeneson+pc_mito+brain_region+Sex	0.824	4557.000
ENSG00000174695	0.001	0.180	0.306	0.055	[illegible]	0.008	~amyloid_beta+(1|manifest)+cngeneson+pc_mito+brain_region+Sex	0.483	5396.000
ENSG00000144744	0.000	0.180	0.259	0.101	UBA3	0.001	~amyloid_beta+(1|manifest)+cngeneson+pc_mito+brain_region+Sex	0.779	4670.000
ENSG00000257103	0.000	0.180	0.302	0.057	LSM14A	0.005	~amyloid_beta+(1|manifest)+cngeneson+pc_mito+brain_region+Sex	1.313	3476.000
ENSG00000166833	0.030	0.179	0.345	0.013	NAV2	0.074	~amyloid_beta+(1|manifest)+cngeneson+pc_mito+brain_region+Sex	16.164	86.000
ENSG00000075239	0.000	0.179	0.271	0.087	ACAT1	0.002	~amyloid_beta+(1|manifest)+cngeneson+pc_mito+brain_region+Sex	0.657	4983.000
ENSG00000136160	0.345	0.179	0.582	-0.224	EDNRB	0.396	~amyloid_beta+(1|manifest)+cngeneson+pc_mito+brain_region+Sex	5.175	734.000
ENSG00000104219	0.016	0.179	0.430	-0.072	ZDHHC2	0.053	~amyloid_beta+(1|manifest)+cngeneson+pc_mito+brain_region+Sex	3.051	1510.000
ENSG00000189339	0.002	0.179	0.308	0.050	SLC35E2B	0.017	~amyloid_beta+(1|manifest)+cngeneson+pc_mito+brain_region+Sex	1.470	3184.000
ENSG00000100811	0.001	0.179	0.305	0.052	YY1	0.013	~amyloid_beta+(1|manifest)+cngeneson+pc_mito+brain_region+Sex	0.313	5708.000
ENSG00000080845	0.008	0.179	0.397	-0.039	DLGAP4	0.035	~amyloid_beta+(1|manifest)+cngeneson+pc_mito+brain_region+Sex	1.344	3419.000
ENSG00000006715	0.000	0.179	0.300	0.058	VPS41	0.006	~amyloid_beta+(1|manifest)+cngeneson+pc_mito+brain_region+Sex	0.542	5253.000
ENSG00000115170	0.003	0.179	0.361	-0.004	ACVR1	0.019	~amyloid_beta+(1|manifest)+cngeneson+pc_mito+brain_region+Sex	1.964	2470.000
ENSG00000090097	0.080	0.179	0.445	-0.087	PCBP4	0.135	~amyloid_beta+(1|manifest)+cngeneson+pc_mito+brain_region+Sex	2.751	1704.000
ENSG00000118689	0.190	0.178	0.388	-0.032	FOXO3	0.244	~amyloid_beta+(1|manifest)+cngeneson+pc_mito+brain_region+Sex	2.425	1960.000
ENSG00000077044	0.225	0.178	0.495	-0.138	DGKD	0.280	~amyloid_beta+(1|manifest)+cngeneson+pc_mito+brain_region+Sex	5.901	608.000
ENSG00000182944	0.007	0.178	0.322	0.034	EWSR1	0.032	~amyloid_beta+(1|manifest)+cngeneson+pc_mito+brain_region+Sex	0.850	4500.000
ENSG00000274070	0.000	0.178	0.336	0.020	CASTOR2	0.002	~amyloid_beta+(1|manifest)+cngeneson+pc_mito+brain_region+Sex	0.281	5763.000
ENSG00000141068	0.015	0.178	0.365	-0.009	KSR1	0.050	~amyloid_beta+(1|manifest)+cngeneson+pc_mito+brain_region+Sex	0.428	5510.000
ENSG00000198146	0.006	0.178	0.328	0.027	ZNF770	0.028	~amyloid_beta+(1|manifest)+cngeneson+pc_mito+brain_region+Sex	0.251	5808.000
ENSG00000277443	0.046	0.178	0.438	-0.083	MARCKS	0.095	~amyloid_beta+(1|manifest)+cngeneson+pc_mito+brain_region+Sex	1.595	2991.000
ENSG00000062194	0.094	0.177	0.398	-0.043	GPBP1	0.149	~amyloid_beta+(1|manifest)+cngeneson+pc_mito+brain_region+Sex	0.349	5654.000
ENSG00000196935	0.099	0.177	0.330	0.024	SRGAP1	0.155	~amyloid_beta+(1|manifest)+cngeneson+pc_mito+brain_region+Sex	4.020	1034.000
ENSG00000146828	0.002	0.177	0.329	0.025	SLC12A9	0.016	~amyloid_beta+(1|manifest)+cngeneson+pc_mito+brain_region+Sex	0.878	4421.000
ENSG00000087495	0.289	0.176	0.385	-0.033	PHACTR3	0.341	~amyloid_beta+(1|manifest)+cngeneson+pc_mito+brain_region+Sex	14.372	120.000
ENSG00000196998	0.003	0.176	0.328	0.025	WDR45	0.020	~amyloid_beta+(1|manifest)+cngeneson+pc_mito+brain_region+Sex	0.509	5330.000
ENSG00000084733	0.000	0.176	0.292	0.059	RAB10	0.004	~amyloid_beta+(1|manifest)+cngeneson+pc_mito+brain_region+Sex	0.433	5499.000
ENSG00000094916	0.007	0.176	0.360	-0.009	CBX5	0.031	~amyloid_beta+(1|manifest)+cngeneson+pc_mito+brain_region+Sex	1.212	3677.000
ENSG00000159023	0.002	0.176	0.413	-0.061	EPB41	0.017	~amyloid_beta+(1|manifest)+cngeneson+pc_mito+brain_region+Sex	1.634	2932.000
ENSG00000115310	0.266	0.176	0.450	-0.099	RTN4	0.319	~amyloid_beta+(1|manifest)+cngeneson+pc_mito+brain_region+Sex	6.793	492.000
ENSG00000168280	0.018	0.175	0.390	-0.039	KIF5C	0.055	~amyloid_beta+(1|manifest)+cngeneson+pc_mito+brain_region+Sex	1.241	3616.000
ENSG00000120254	0.098	0.175	0.530	-0.179	MTHFD1L	0.153	~amyloid_beta+(1|manifest)+cngeneson+pc_mito+brain_region+Sex	3.326	1354.000
ENSG00000179222	0.073	0.175	0.490	-0.140	MAGED1	0.127	~amyloid_beta+(1|manifest)+cngeneson+pc_mito+brain_region+Sex	2.399	1985.000
ENSG00000082781	0.019	0.175	0.395	-0.046	ITGB5	0.057	~amyloid_beta+(1|manifest)+cngeneson+pc_mito+brain_region+Sex	1.050	4022.000
ENSG00000064393	0.019	0.175	0.334	0.015	HIPK2	0.058	~amyloid_beta+(1|manifest)+cngeneson+pc_mito+brain_region+Sex	1.958	2483.000
ENSG00000123352	0.000	0.175	0.293	0.056	SPATS2	0.006	~amyloid_beta+(1|manifest)+cngeneson+pc_mito+brain_region+Sex	0.867	4448.000
ENSG00000101680	0.158	0.174	0.552	-0.203	LAMA1	0.213	~amyloid_beta+(1|manifest)+cngeneson+pc_mito+brain_region+Sex	4.558	874.000
ENSG00000166598	0.145	0.174	0.580	-0.231	HSP90B1	0.201	~amyloid_beta+(1|manifest)+cngeneson+pc_mito+brain_region+Sex	5.196	729.000
ENSG00000148343	0.008	0.174	0.328	0.020	MIGA2	0.034	~amyloid_beta+(1|manifest)+cngeneson+pc_mito+brain_region+Sex	1.126	3871.000
ENSG00000186812	0.001	0.174	0.288	0.061	ZNF397	0.010	~amyloid_beta+(1|manifest)+cngeneson+pc_mito+brain_region+Sex	0.803	4622.000
ENSG00000066855	0.000	0.174	0.295	0.053	MTFR1	0.005	~amyloid_beta+(1|manifest)+cngeneson+pc_mito+brain_region+Sex	0.110	5947.000
ENSG00000162909	0.177	0.174	0.468	-0.121	CAPN2	0.231	~amyloid_beta+(1|manifest)+cngeneson+pc_mito+brain_region+Sex	5.984	594.000
ENSG00000111669	0.019	0.173	0.458	-0.111	TPI1	0.058	~amyloid_beta+(1|manifest)+cngeneson+pc_mito+brain_region+Sex	1.282	3539.000
ENSG00000108231	0.345	0.173	0.478	-0.131	LGI1	0.396	~amyloid_beta+(1|manifest)+cngeneson+pc_mito+brain_region+Sex	3.949	1064.000
ENSG00000070950	0.004	0.173	0.299	0.047	RAD18	0.022	~amyloid_beta+(1|manifest)+cngeneson+pc_mito+brain_region+Sex	2.075	2339.000
ENSG00000172667	0.207	0.173	0.500	-0.155	ZMAT3	0.262	~amyloid_beta+(1|manifest)+cngeneson+pc_mito+brain_region+Sex	2.523	1877.000
ENSG00000125388	0.004	0.173	0.313	0.033	GRK4	0.023	~amyloid_beta+(1|manifest)+cngeneson+pc_mito+brain_region+Sex	1.318	3466.000
ENSG00000205302	0.007	0.172	0.326	0.019	SNX2	0.032	~amyloid_beta+(1|manifest)+cngeneson+pc_mito+brain_region+Sex	0.478	5405.000
ENSG00000119537	0.000	0.172	0.279	0.065	KDSR	0.006	~amyloid_beta+(1|manifest)+cngeneson+pc_mito+brain_region+Sex	0.772	4687.000
ENSG00000188026	0.007	0.172	0.399	-0.054	RILPL1	0.032	~amyloid_beta+(1|manifest)+cngeneson+pc_mito+brain_region+Sex	2.251	2155.000
ENSG00000165476	0.000	0.172	0.259	0.086	REEP3	0.000	~amyloid_beta+(1|manifest)+cngeneson+pc_mito+brain_region+Sex	0.636	5042.000
ENSG00000088538	0.016	0.172	0.279	0.064	DOCK3	0.051	~amyloid_beta+(1|manifest)+cngeneson+pc_mito+brain_region+Sex	1.610	2966.000
ENSG00000103550	0.345	0.171	0.835	-0.493	KNOP1	0.396	~amyloid_beta+(1|manifest)+cngeneson+pc_mito+brain_region+Sex	20.407	49.000
ENSG00000106479	0.018	0.171	0.333	0.009	ZNF862	0.056	~amyloid_beta+(1|manifest)+cngeneson+pc_mito+brain_region+Sex	1.986	2452.000
ENSG00000124181	0.287	0.170	0.536	-0.195	PLCG1	0.340	~amyloid_beta+(1|manifest)+cngeneson+pc_mito+brain_region+Sex	5.908	605.000
ENSG00000060971	0.015	0.170	0.427	-0.086	ACAA1	0.050	~amyloid_beta+(1|manifest)+cngeneson+pc_mito+brain_region+Sex	2.948	1575.000
ENSG00000105605	0.002	0.170	0.310	0.030	CACNG7	0.017	~amyloid_beta+(1|manifest)+cngeneson+pc_mito+brain_region+Sex	0.711	4844.000
ENSG00000182180	0.002	0.170	0.291	0.049	MRPS16	0.015	~amyloid_beta+(1|manifest)+cngeneson+pc_mito+brain_region+Sex	0.541	5254.000
ENSG00000100284	0.001	0.170	0.290	0.049	TOM1	0.010	~amyloid_beta+(1|manifest)+cngeneson+pc_mito+brain_region+Sex	0.618	5091.000
ENSG00000101152	0.001	0.169	0.298	0.041	DNAJC5	0.011	~amyloid_beta+(1|manifest)+cngeneson+pc_mito+brain_region+Sex	0.124	5937.000
ENSG00000138101	0.000	0.168	0.303	0.034	DTNB	0.005	~amyloid_beta+(1|manifest)+cngeneson+pc_mito+brain_region+Sex	1.168	3772.000
ENSG00000101134	0.192	0.168	0.415	-0.079	DOK5	0.246	~amyloid_beta+(1|manifest)+cngeneson+pc_mito+brain_region+Sex	1.836	2648.000
ENSG00000067064	0.007	0.168	0.327	0.009	IDI1	0.032	~amyloid_beta+(1|manifest)+cngeneson+pc_mito+brain_region+Sex	1.499	3138.000
ENSG00000108312	0.003	0.168	0.308	0.027	UBTF	0.021	~amyloid_beta+(1|manifest)+cngeneson+pc_mito+brain_region+Sex	0.375	5607.000
ENSG00000175764	0.001	0.168	0.280	0.055	TTLL11	0.009	~amyloid_beta+(1|manifest)+cngeneson+pc_mito+brain_region+Sex	0.343	5661.000
ENSG00000127663	0.003	0.167	0.300	0.035	KDM4B	0.020	~amyloid_beta+(1|manifest)+cngeneson+pc_mito+brain_region+Sex	1.366	3380.000
ENSG00000109819	0.402	0.167	0.601	-0.266	PPARGC1A	0.449	~amyloid_beta+(1|manifest)+cngeneson+pc_mito+brain_region+Sex	3.065	1499.000
ENSG00000179604	0.233	0.167	0.477	-0.143	CDC42EP4	0.287	~amyloid_beta+(1|manifest)+cngeneson+pc_mito+brain_region+Sex	3.134	1462.000
ENSG00000164751	0.004	0.167	0.331	0.003	PEX2	0.025	~amyloid_beta+(1|manifest)+cngeneson+pc_mito+brain_region+Sex	0.492	5383.000
ENSG00000166439	0.018	0.167	0.425	-0.092	RNF169	0.056	~amyloid_beta+(1|manifest)+cngeneson+pc_mito+brain_region+Sex	0.745	4757.000
ENSG00000061273	0.317	0.166	0.616	-0.283	HDAC7	0.369	~amyloid_beta+(1|manifest)+cngeneson+pc_mito+brain_region+Sex	7.429	423.000
ENSG00000103187	0.028	0.166	0.325	0.007	COTL1	0.072	~amyloid_beta+(1|manifest)+cngeneson+pc_mito+brain_region+Sex	1.538	3071.000
ENSG00000137216	0.001	0.166	0.273	0.058	TMEM63B	0.012	~amyloid_beta+(1|manifest)+cngeneson+pc_mito+brain_region+Sex	0.464	5428.000
ENSG00000121879	0.049	0.166	0.404	-0.073	PIK3CA	0.098	~amyloid_beta+(1|manifest)+cngeneson+pc_mito+brain_region+Sex	1.282	3540.000
ENSG00000160049	0.000	0.166	0.226	0.105	DFFA	0.000	~amyloid_beta+(1|manifest)+cngeneson+pc_mito+brain_region+Sex	0.193	5879.000
ENSG00000082898	0.264	0.165	0.386	-0.055	XPO1	0.318	~amyloid_beta+(1|manifest)+cngeneson+pc_mito+brain_region+Sex	2.167	2243.000
ENSG00000105953	0.003	0.165	0.382	-0.051	OGDH	0.019	~amyloid_beta+(1|manifest)+cngeneson+pc_mito+brain_region+Sex	1.961	2477.000
ENSG00000124440	0.364	0.165	0.397	-0.067	HIF3A	0.415	~amyloid_beta+(1|manifest)+cngeneson+pc_mito+brain_region+Sex	35.741	11.000
ENSG00000198034	0.045	0.165	0.550	-0.220	RPS4X	0.094	~amyloid_beta+(1|manifest)+cngeneson+pc_mito+brain_region+Sex	7.265	446.000
ENSG00000167065	0.001	0.165	0.276	0.054	DUSP18	0.013	~amyloid_beta+(1|manifest)+cngeneson+pc_mito+brain_region+Sex	0.192	5880.000
ENSG00000120265	0.000	0.165	0.276	0.054	PCMT1	0.007	~amyloid_beta+(1|manifest)+cngeneson+pc_mito+brain_region+Sex	0.138	5930.000
ENSG00000122566	0.081	0.165	0.330	-0.001	[illegible]	0.135	~amyloid_beta+(1|manifest)+cngeneson+pc_mito+brain_region+Sex	2.270	2132.000
ENSG00000163428	0.006	0.165	0.296	0.033	LRRC58	0.030	~amyloid_beta+(1|manifest)+cngeneson+pc_mito+brain_region+Sex	0.306	5721.000
ENSG00000167106	0.030	0.164	0.359	-0.030	FAM102A	0.074	~amyloid_beta+(1|manifest)+cngeneson+pc_mito+brain_region+Sex	1.025	4088.000
ENSG00000178685	0.129	0.164	0.453	-0.124	PARP10	0.185	~amyloid_beta+(1|manifest)+cngeneson+pc_mito+brain_region+Sex	3.307	1367.000
ENSG00000103168	0.067	0.164	0.452	-0.124	TAF1C	0.121	~amyloid_beta+(1|manifest)+cngeneson+pc_mito+brain_region+Sex	2.328	2071.000
ENSG00000100412	0.065	0.164	0.403	-0.075	ACO2	0.117	~amyloid_beta+(1|manifest)+cngeneson+pc_mito+brain_region+Sex	2.395	1991.000
ENSG00000123268	0.000	0.164	0.305	0.022	ATF1	0.007	~amyloid_beta+(1|manifest)+cngeneson+pc_mito+brain_region+Sex	0.805	4618.000
ENSG00000198952	0.002	0.164	0.283	0.044	SMG5	0.017	~amyloid_beta+(1|manifest)+cngeneson+pc_mito+brain_region+Sex	0.723	4816.000
ENSG00000242485	0.005	0.164	0.317	0.010	MRPL20	0.026	~amyloid_beta+(1|manifest)+cngeneson+pc_mito+brain_region+Sex	1.224	3657.000
ENSG00000151067	0.036	0.163	0.418	-0.091	CACNA1C	0.083	~amyloid_beta+(1|manifest)+cngeneson+pc_mito+brain_region+Sex	3.412	1308.000
ENSG00000065882	0.033	0.163	0.443	-0.116	TBC1D1	0.078	~amyloid_beta+(1|manifest)+cngeneson+pc_mito+brain_region+Sex	2.094	2321.000
ENSG00000124193	0.115	0.163	0.496	-0.170	SRSF6	0.171	~amyloid_beta+(1|manifest)+cngeneson+pc_mito+brain_region+Sex	4.046	1026.000
ENSG00000087274	0.026	0.163	0.295	0.031	ADD1	0.069	~amyloid_beta+(1|manifest)+cngeneson+pc_mito+brain_region+Sex	2.383	2011.000
ENSG00000137714	0.001	0.163	0.270	0.056	FDX1	0.012	~amyloid_beta+(1|manifest)+cngeneson+pc_mito+brain_region+Sex	0.052	5975.000
ENSG00000140396	0.075	0.163	0.297	0.029	NCOA2	0.129	~amyloid_beta+(1|manifest)+cngeneson+pc_mito+brain_region+Sex	0.618	5088.000
ENSG00000108262	0.069	0.163	0.397	-0.071	GIT1	0.122	~amyloid_beta+(1|manifest)+cngeneson+pc_mito+brain_region+Sex	1.414	3301.000
ENSG00000101290	0.002	0.163	0.274	0.051	CDS2	0.018	~amyloid_beta+(1|manifest)+cngeneson+pc_mito+brain_region+Sex	1.214	3674.000
ENSG00000147044	0.022	0.163	0.335	-0.010	CASK	0.063	~amyloid_beta+(1|manifest)+cngeneson+pc_mito+brain_region+Sex	1.263	3574.000
ENSG00000012983	0.043	0.163	0.301	0.025	MAP4K5	0.091	~amyloid_beta+(1|manifest)+cngeneson+pc_mito+brain_region+Sex	1.293	3519.000
ENSG00000150756	0.007	0.163	0.320	0.005	[illegible]	0.031	~amyloid_beta+(1|manifest)+cngeneson+pc_mito+brain_region+Sex	0.277	5770.000
ENSG00000153317	0.026	0.163	0.273	0.052	ASAP1	0.069	~amyloid_beta+(1|manifest)+cngeneson+pc_mito+brain_region+Sex	2.699	1730.000
ENSG00000171298	0.077	0.162	0.415	-0.090	GAA	0.132	~amyloid_beta+(1|manifest)+cngeneson+pc_mito+brain_region+Sex	3.533	1231.000
ENSG00000165861	0.000	0.162	0.288	0.037	ZFYVE1	0.005	~amyloid_beta+(1|manifest)+cngeneson+pc_mito+brain_region+Sex	0.811	4597.000
ENSG00000171877	0.506	0.162	0.469	-0.145	FRMD5	0.549	~amyloid_beta+(1|manifest)+cngeneson+pc_mito+brain_region+Sex	2.938	1583.000
ENSG00000153558	0.007	0.162	0.457	-0.134	FBXL2	0.032	~amyloid_beta+(1|manifest)+cngeneson+pc_mito+brain_region+Sex	1.461	3204.000
ENSG00000070010	0.002	0.161	0.307	0.016	UFD1	0.017	~amyloid_beta+(1|manifest)+cngeneson+pc_mito+brain_region+Sex	0.793	4647.000
ENSG00000109762	0.001	0.161	0.292	0.030	SNX25	0.010	~amyloid_beta+(1|manifest)+cngeneson+pc_mito+brain_region+Sex	1.075	3976.000
ENSG00000182872	0.003	0.161	0.283	0.039	RBM10	0.021	~amyloid_beta+(1|manifest)+cngeneson+pc_mito+brain_region+Sex	0.806	4614.000
ENSG00000138434	0.072	0.161	0.420	-0.098	ITPRID2	0.126	~amyloid_beta+(1|manifest)+cngeneson+pc_mito+brain_region+Sex	4.549	879.000
ENSG00000196187	0.006	0.161	0.313	0.008	TMEM63A	0.030	~amyloid_beta+(1|manifest)+cngeneson+pc_mito+brain_region+Sex	1.336	3438.000
ENSG00000211455	0.001	0.161	0.287	0.034	STK38L	0.010	~amyloid_beta+(1|manifest)+cngeneson+pc_mito+brain_region+Sex	1.146	3824.000
ENSG00000108175	0.063	0.160	0.401	-0.080	ZMIZ1	0.115	~amyloid_beta+(1|manifest)+cngeneson+pc_mito+brain_region+Sex	2.202	2201.000
ENSG00000066044	0.001	0.160	0.261	0.060	ELAVL1	0.009	~amyloid_beta+(1|manifest)+cngeneson+pc_mito+brain_region+Sex	0.947	4260.000
ENSG00000070214	0.123	0.160	0.410	-0.089	SLC44A1	0.180	~amyloid_beta+(1|manifest)+cngeneson+pc_mito+brain_region+Sex	2.265	2139.000
ENSG00000143702	0.016	0.160	0.348	-0.028	CEP170	0.053	~amyloid_beta+(1|manifest)+cngeneson+pc_mito+brain_region+Sex	2.034	2389.000
ENSG00000037042	0.009	0.160	0.344	-0.023	TUBG2	0.036	~amyloid_beta+(1|manifest)+cngeneson+pc_mito+brain_region+Sex	2.523	1876.000
ENSG00000138081	0.004	0.160	0.283	0.038	FBXO11	0.024	~amyloid_beta+(1|manifest)+cngeneson+pc_mito+brain_region+Sex	0.680	4938.000
ENSG00000152465	0.001	0.160	0.277	0.043	NMT2	0.009	~amyloid_beta+(1|manifest)+cngeneson+pc_mito+brain_region+Sex	0.418	5528.000
ENSG00000172081	0.000	0.160	0.231	0.089	MOB3A	0.001	~amyloid_beta+(1|manifest)+cngeneson+pc_mito+brain_region+Sex	0.462	5439.000
ENSG00000072134	0.119	0.160	0.378	-0.059	EPN2	0.175	~amyloid_beta+(1|manifest)+cngeneson+pc_mito+brain_region+Sex	2.950	1574.000
ENSG00000092439	0.000	0.160	0.252	0.067	TRPM7	0.002	~amyloid_beta+(1|manifest)+cngeneson+pc_mito+brain_region+Sex	1.158	3795.000
ENSG00000135018	0.002	0.159	0.294	0.025	UBQLN1	0.014	~amyloid_beta+(1|manifest)+cngeneson+pc_mito+brain_region+Sex	0.231	5834.000
ENSG00000148337	0.002	0.159	0.280	0.039	CIZ1	0.017	~amyloid_beta+(1|manifest)+cngeneson+pc_mito+brain_region+Sex	1.581	3011.000
ENSG00000124587	0.002	0.159	0.276	0.042	PEX6	0.018	~amyloid_beta+(1|manifest)+cngeneson+pc_mito+brain_region+Sex	1.572	3020.000
ENSG00000133961	0.075	0.159	0.355	-0.036	NUMB	0.129	~amyloid_beta+(1|manifest)+cngeneson+pc_mito+brain_region+Sex	1.959	2481.000
ENSG00000134243	0.007	0.159	0.284	0.034	SORT1	0.031	~amyloid_beta+(1|manifest)+cngeneson+pc_mito+brain_region+Sex	0.230	5835.000
ENSG00000070476	0.093	0.159	0.366	-0.049	ZXDC	0.149	~amyloid_beta+(1|manifest)+cngeneson+pc_mito+brain_region+Sex	3.266	1387.000
ENSG00000130164	0.301	0.159	0.531	-0.213	LDLR	0.354	~amyloid_beta+(1|manifest)+cngeneson+pc_mito+brain_region+Sex	10.085	266.000
ENSG00000165966	0.138	0.159	0.603	-0.286	PDZRN4	0.195	~amyloid_beta+(1|manifest)+cngeneson+pc_mito+brain_region+Sex	6.380	538.000
ENSG00000179912	0.039	0.158	0.381	-0.065	R3HDM2	0.087	~amyloid_beta+(1|manifest)+cngeneson+pc_mito+brain_region+Sex	0.405	5556.000
ENSG00000148341	0.097	0.158	0.465	-0.148	SH3GLB2	0.152	~amyloid_beta+(1|manifest)+cngeneson+pc_mito+brain_region+Sex	6.244	557.000
ENSG00000105662	0.105	0.158	0.430	-0.114	CRTC1	0.161	~amyloid_beta+(1|manifest)+cngeneson+pc_mito+brain_region+Sex	3.432	1290.000
ENSG00000249209	0.087	0.158	0.469	-0.153	[illegible]	0.142	~amyloid_beta+(1|manifest)+cngeneson+pc_mito+brain_region+Sex	4.028	1031.000
ENSG00000165113	0.014	0.158	0.348	-0.032	GKAP1	0.048	~amyloid_beta+(1|manifest)+cngeneson+pc_mito+brain_region+Sex	0.264	5792.000
ENSG00000151694	0.006	0.158	0.385	-0.070	ADAM17	0.030	~amyloid_beta+(1|manifest)+cngeneson+pc_mito+brain_region+Sex	1.982	2456.000
ENSG00000140983	0.101	0.158	0.498	-0.183	RHOT2	0.156	~amyloid_beta+(1|manifest)+cngeneson+pc_mito+brain_region+Sex	3.423	1298.000
ENSG00000165264	0.001	0.158	0.265	0.050	NDUFB6	0.008	~amyloid_beta+(1|manifest)+cngeneson+pc_mito+brain_region+Sex	0.091	5961.000
ENSG00000132676	0.260	0.158	0.530	-0.215	DAP3	0.314	~amyloid_beta+(1|manifest)+cngeneson+pc_mito+brain_region+Sex	7.768	393.000
ENSG00000010626	0.003	0.157	0.291	0.023	LRRC23	0.022	~amyloid_beta+(1|manifest)+cngeneson+pc_mito+brain_region+Sex	0.843	4514.000
ENSG00000160460	0.029	0.157	0.412	-0.098	SPTBN4	0.073	~amyloid_beta+(1|manifest)+cngeneson+pc_mito+brain_region+Sex	3.290	1375.000
ENSG00000170382	0.023	0.157	0.474	-0.161	LRRN2	0.064	~amyloid_beta+(1|manifest)+cngeneson+pc_mito+brain_region+Sex	2.681	1744.000
ENSG00000175854	0.014	0.156	0.309	0.004	SWI5	0.048	~amyloid_beta+(1|manifest)+cngeneson+pc_mito+brain_region+Sex	1.682	2855.000
ENSG00000108510	0.010	0.156	0.302	0.011	MED13	0.040	~amyloid_beta+(1|manifest)+cngeneson+pc_mito+brain_region+Sex	1.653	2899.000
ENSG00000146433	0.001	0.156	0.279	0.033	TMEM181	0.008	~amyloid_beta+(1|manifest)+cngeneson+pc_mito+brain_region+Sex	0.545	5241.000
ENSG00000175662	0.162	0.156	0.336	-0.024	TOM1L2	0.217	~amyloid_beta+(1|manifest)+cngeneson+pc_mito+brain_region+Sex	6.541	522.000
ENSG00000164403	0.017	0.156	0.347	-0.035	SHROOM1	0.055	~amyloid_beta+(1|manifest)+cngeneson+pc_mito+brain_region+Sex	1.995	2440.000
ENSG00000052749	0.000	0.156	0.231	0.081	RRP12	0.002	~amyloid_beta+(1|manifest)+cngeneson+pc_mito+brain_region+Sex	0.377	5600.000
ENSG00000115414	0.315	0.155	0.394	-0.083	FN1	0.367	~amyloid_beta+(1|manifest)+cngeneson+pc_mito+brain_region+Sex	2.674	1750.000
ENSG00000065060	0.013	0.155	0.339	-0.029	UHRF1BP1	0.046	~amyloid_beta+(1|manifest)+cngeneson+pc_mito+brain_region+Sex	0.395	5571.000
ENSG00000121406	0.000	0.155	0.263	0.047	ZNF549	0.007	~amyloid_beta+(1|manifest)+cngeneson+pc_mito+brain_region+Sex	0.303	5728.000
ENSG00000213551	0.000	0.155	0.244	0.065	DNAJC9	0.005	~amyloid_beta+(1|manifest)+cngeneson+pc_mito+brain_region+Sex	0.493	5378.000
ENSG00000196367	0.003	0.155	0.310	-0.001	TRRAP	0.020	~amyloid_beta+(1|manifest)+cngeneson+pc_mito+brain_region+Sex	1.030	4079.000
ENSG00000132639	0.016	0.154	0.425	-0.116	SNAP25	0.053	~amyloid_beta+(1|manifest)+cngeneson+pc_mito+brain_region+Sex	2.191	2213.000
ENSG00000110318	0.001	0.154	0.452	-0.143	CEP126	0.014	~amyloid_beta+(1|manifest)+cngeneson+pc_mito+brain_region+Sex	0.781	4665.000
ENSG00000196361	0.020	0.154	0.340	-0.032	ELAVL3	0.059	~amyloid_beta+(1|manifest)+cngeneson+pc_mito+brain_region+Sex	1.147	3820.000
ENSG00000142156	0.022	0.154	0.360	-0.052	COL6A1	0.063	~amyloid_beta+(1|manifest)+cngeneson+pc_mito+brain_region+Sex	0.641	5028.000
ENSG00000174915	0.012	0.154	0.316	-0.008	PTDSS2	0.044	~amyloid_beta+(1|manifest)+cngeneson+pc_mito+brain_region+Sex	0.550	5230.000
ENSG00000068137	0.064	0.154	0.354	-0.046	PLEKHH3	0.117	~amyloid_beta+(1|manifest)+cngeneson+pc_mito+brain_region+Sex	1.938	2510.000
ENSG00000151176	0.001	0.154	0.284	0.024	PLBD2	0.009	~amyloid_beta+(1|manifest)+cngeneson+pc_mito+brain_region+Sex	0.192	5883.000
ENSG00000198242	0.118	0.154	0.490	-0.182	RPL23A	0.175	~amyloid_beta+(1|manifest)+cngeneson+pc_mito+brain_region+Sex	3.714	1157.000
ENSG00000131044	0.017	0.154	0.342	-0.034	TTLL9	0.054	~amyloid_beta+(1|manifest)+cngeneson+pc_mito+brain_region+Sex	1.569	3024.000
ENSG00000081923	0.008	0.154	0.330	-0.023	ATP8B1	0.036	~amyloid_beta+(1|manifest)+cngeneson+pc_mito+brain_region+Sex	1.088	3951.000
ENSG00000159210	0.000	0.153	0.238	0.069	SNF8	0.006	~amyloid_beta+(1|manifest)+cngeneson+pc_mito+brain_region+Sex	0.407	5553.000
ENSG00000006468	0.107	0.153	0.499	-0.193	ETV1	0.163	~amyloid_beta+(1|manifest)+cngeneson+pc_mito+brain_region+Sex	3.779	1130.000
ENSG00000049246	0.228	0.153	0.586	-0.280	PER3	0.282	~amyloid_beta+(1|manifest)+cngeneson+pc_mito+brain_region+Sex	4.809	821.000
ENSG00000171365	0.001	0.153	0.263	0.042	CLCN5	0.012	~amyloid_beta+(1|manifest)+cngeneson+pc_mito+brain_region+Sex	0.245	5814.000
ENSG00000099194	0.052	0.152	0.431	-0.126	SCD	0.102	~amyloid_beta+(1|manifest)+cngeneson+pc_mito+brain_region+Sex	3.308	1364.000
ENSG00000104312	0.019	0.152	0.324	-0.020	RIPK2	0.057	~amyloid_beta+(1|manifest)+cngeneson+pc_mito+brain_region+Sex	1.007	4124.000
ENSG00000166446	0.033	0.152	0.325	-0.021	CDYL2	0.078	~amyloid_beta+(1|manifest)+cngeneson+pc_mito+brain_region+Sex	0.404	5559.000
ENSG00000075391	0.486	0.152	0.447	-0.143	RASAL2	0.530	~amyloid_beta+(1|manifest)+cngeneson+pc_mito+brain_region+Sex	6.481	527.000
ENSG00000140350	0.015	0.152	0.303	0.000	ANP32A	0.051	~amyloid_beta+(1|manifest)+cngeneson+pc_mito+brain_region+Sex	0.425	5517.000
ENSG00000065413	0.001	0.152	0.322	-0.019	ANKRD44	0.013	~amyloid_beta+(1|manifest)+cngeneson+pc_mito+brain_region+Sex	0.548	5233.000
ENSG00000152818	0.072	0.152	0.286	0.017	UTRN	0.126	~amyloid_beta+(1|manifest)+cngeneson+pc_mito+brain_region+Sex	3.570	1220.000
ENSG00000028528	0.000	0.151	0.223	0.079	SNX1	0.002	~amyloid_beta+(1|manifest)+cngeneson+pc_mito+brain_region+Sex	0.851	4497.000
ENSG00000139921	0.002	0.151	0.253	0.050	TMX1	0.017	~amyloid_beta+(1|manifest)+cngeneson+pc_mito+brain_region+Sex	0.131	5933.000
ENSG00000174099	0.049	0.151	0.382	-0.080	MSRB3	0.098	~amyloid_beta+(1|manifest)+cngeneson+pc_mito+brain_region+Sex	1.416	3299.000
ENSG00000078699	0.007	0.151	0.243	0.059	CBFA2T2	0.031	~amyloid_beta+(1|manifest)+cngeneson+pc_mito+brain_region+Sex	1.096	3936.000
ENSG00000130653	0.228	0.151	0.605	-0.304	PNPLA7	0.282	~amyloid_beta+(1|manifest)+cngeneson+pc_mito+brain_region+Sex	6.404	535.000
ENSG00000116044	0.100	0.151	0.378	-0.077	NFE2L2	0.155	~amyloid_beta+(1|manifest)+cngeneson+pc_mito+brain_region+Sex	1.956	2487.000
ENSG00000073910	0.026	0.150	0.399	-0.099	FRY	0.068	~amyloid_beta+(1|manifest)+cngeneson+pc_mito+brain_region+Sex	0.569	5186.000
ENSG00000146414	0.016	0.150	0.314	-0.015	SHPRH	0.053	~amyloid_beta+(1|manifest)+cngeneson+pc_mito+brain_region+Sex	1.019	4097.000
ENSG00000135387	0.006	0.150	0.290	0.009	CAPRIN1	0.028	~amyloid_beta+(1|manifest)+cngeneson+pc_mito+brain_region+Sex	0.582	5167.000
ENSG00000176293	0.052	0.150	0.385	-0.086	ZNF135	0.102	~amyloid_beta+(1|manifest)+cngeneson+pc_mito+brain_region+Sex	2.394	1992.000
ENSG00000144560	0.070	0.150	0.292	0.007	VGLL4	0.124	~amyloid_beta+(1|manifest)+cngeneson+pc_mito+brain_region+Sex	0.392	5577.000
ENSG00000102580	0.062	0.150	0.400	-0.101	DNAJC3	0.114	~amyloid_beta+(1|manifest)+cngeneson+pc_mito+brain_region+Sex	2.108	2302.000
ENSG00000129625	0.007	0.150	0.293	0.006	REEP5	0.031	~amyloid_beta+(1|manifest)+cngeneson+pc_mito+brain_region+Sex	0.629	5061.000
ENSG00000272333	0.015	0.150	0.317	-0.018	KMT2B	0.049	~amyloid_beta+(1|manifest)+cngeneson+pc_mito+brain_region+Sex	1.841	2639.000
ENSG00000066136	0.039	0.149	0.355	-0.056	NFYC	0.086	~amyloid_beta+(1|manifest)+cngeneson+pc_mito+brain_region+Sex	1.449	3232.000
ENSG00000108604	0.030	0.149	0.430	-0.131	SMARCD2	0.075	~amyloid_beta+(1|manifest)+cngeneson+pc_mito+brain_region+Sex	2.296	2113.000
ENSG00000077713	0.008	0.149	0.392	-0.094	SLC25A43	0.036	~amyloid_beta+(1|manifest)+cngeneson+pc_mito+brain_region+Sex	0.626	5069.000
ENSG00000110075	0.027	0.148	0.264	0.032	PPP6R3	0.069	~amyloid_beta+(1|manifest)+cngeneson+pc_mito+brain_region+Sex	1.176	3757.000
ENSG00000087884	0.003	0.148	0.266	0.031	AAMDC	0.020	~amyloid_beta+(1|manifest)+cngeneson+pc_mito+brain_region+Sex	0.294	5741.000
ENSG00000115468	0.018	0.148	0.375	-0.079	EFHD1	0.056	~amyloid_beta+(1|manifest)+cngeneson+pc_mito+brain_region+Sex	1.190	3726.000
ENSG00000113387	0.005	0.148	0.302	-0.005	SUB1	0.027	~amyloid_beta+(1|manifest)+cngeneson+pc_mito+brain_region+Sex	0.155	5919.000
ENSG00000237765	0.005	0.148	0.283	0.014	FAM200B	0.026	~amyloid_beta+(1|manifest)+cngeneson+pc_mito+brain_region+Sex	0.254	5803.000
ENSG00000134030	0.144	0.148	0.400	-0.104	CTIF	0.200	~amyloid_beta+(1|manifest)+cngeneson+pc_mito+brain_region+Sex	3.291	1374.000
ENSG00000083720	0.002	0.148	0.301	-0.006	OXCT1	0.015	~amyloid_beta+(1|manifest)+cngeneson+pc_mito+brain_region+Sex	1.005	4128.000
ENSG00000154153	0.036	0.148	0.504	-0.209	RETREG1	0.082	~amyloid_beta+(1|manifest)+cngeneson+pc_mito+brain_region+Sex	2.438	1945.000
ENSG00000149273	0.003	0.148	0.290	0.005	RPS3	0.022	~amyloid_beta+(1|manifest)+cngeneson+pc_mito+brain_region+Sex	1.125	3873.000
ENSG00000213096	0.002	0.147	0.319	-0.025	ZNF254	0.016	~amyloid_beta+(1|manifest)+cngeneson+pc_mito+brain_region+Sex	0.745	4755.000
ENSG00000047346	0.072	0.147	0.437	-0.142	FAM214A	0.126	~amyloid_beta+(1|manifest)+cngeneson+pc_mito+brain_region+Sex	1.438	3249.000
ENSG00000168461	0.147	0.147	0.403	-0.109	RAB31	0.203	~amyloid_beta+(1|manifest)+cngeneson+pc_mito+brain_region+Sex	1.954	2490.000
ENSG00000146733	0.002	0.147	0.258	0.036	PSPH	0.016	~amyloid_beta+(1|manifest)+cngeneson+pc_mito+brain_region+Sex	0.284	5754.000
ENSG00000172803	0.007	0.147	0.368	-0.074	SNX32	0.032	~amyloid_beta+(1|manifest)+cngeneson+pc_mito+brain_region+Sex	1.922	2535.000
ENSG00000107372	0.091	0.147	0.389	-0.095	ZFAND5	0.146	~amyloid_beta+(1|manifest)+cngeneson+pc_mito+brain_region+Sex	1.886	2582.000
ENSG00000072518	0.001	0.147	0.242	0.051	MARK2	0.009	~amyloid_beta+(1|manifest)+cngeneson+pc_mito+brain_region+Sex	0.201	5871.000
ENSG00000125952	0.011	0.147	0.302	-0.008	MAX	0.043	~amyloid_beta+(1|manifest)+cngeneson+pc_mito+brain_region+Sex	2.153	2256.000
ENSG00000153201	0.002	0.147	0.286	0.007	RANBP2	0.016	~amyloid_beta+(1|manifest)+cngeneson+pc_mito+brain_region+Sex	0.275	5774.000
ENSG00000198700	0.011	0.147	0.331	-0.038	IPO9	0.043	~amyloid_beta+(1|manifest)+cngeneson+pc_mito+brain_region+Sex	1.606	2977.000
ENSG00000076685	0.073	0.146	0.276	0.017	NT5C2	0.126	~amyloid_beta+(1|manifest)+cngeneson+pc_mito+brain_region+Sex	1.390	3338.000
ENSG00000182287	0.011	0.146	0.425	-0.133	AP1S2	0.041	~amyloid_beta+(1|manifest)+cngeneson+pc_mito+brain_region+Sex	2.261	2143.000
ENSG00000266472	0.023	0.146	0.336	-0.043	MRPS21	0.063	~amyloid_beta+(1|manifest)+cngeneson+pc_mito+brain_region+Sex	0.704	4872.000
ENSG00000012232	0.003	0.146	0.251	0.042	EXTL3	0.019	~amyloid_beta+(1|manifest)+cngeneson+pc_mito+brain_region+Sex	0.739	4771.000
ENSG00000135720	0.003	0.146	0.276	0.016	DYNC1LI2	0.018	~amyloid_beta+(1|manifest)+cngeneson+pc_mito+brain_region+Sex	1.249	3603.000
ENSG00000164466	0.004	0.146	0.283	0.009	SFXN1	0.025	~amyloid_beta+(1|manifest)+cngeneson+pc_mito+brain_region+Sex	1.520	3106.000
ENSG00000145349	0.424	0.146	0.429	-0.137	CAMK2D	0.470	~amyloid_beta+(1|manifest)+cngeneson+pc_mito+brain_region+Sex	3.662	1181.000
ENSG00000170085	0.002	0.146	0.307	-0.015	SIMC1	0.017	~amyloid_beta+(1|manifest)+cngeneson+pc_mito+brain_region+Sex	0.449	5456.000
ENSG00000186951	0.135	0.146	0.429	-0.138	PPARA	0.192	~amyloid_beta+(1|manifest)+cngeneson+pc_mito+brain_region+Sex	4.197	986.000
ENSG00000083828	0.001	0.146	0.308	-0.017	ZNF586	0.008	~amyloid_beta+(1|manifest)+cngeneson+pc_mito+brain_region+Sex	1.481	3172.000
ENSG00000072609	0.002	0.146	0.288	0.003	CHFR	0.015	~amyloid_beta+(1|manifest)+cngeneson+pc_mito+brain_region+Sex	0.999	4145.000
ENSG00000154978	0.020	0.145	0.312	-0.022	VOPP1	0.059	~amyloid_beta+(1|manifest)+cngeneson+pc_mito+brain_region+Sex	0.796	4640.000
ENSG00000145832	0.238	0.145	0.513	-0.223	SLC25A48	0.292	~amyloid_beta+(1|manifest)+cngeneson+pc_mito+brain_region+Sex	0.533	5274.000
ENSG00000135472	0.097	0.145	0.423	-0.133	FAIM2	0.152	~amyloid_beta+(1|manifest)+cngeneson+pc_mito+brain_region+Sex	2.686	1740.000
ENSG00000151692	0.001	0.145	0.380	-0.091	RNF144A	0.008	~amyloid_beta+(1|manifest)+cngeneson+pc_mito+brain_region+Sex	0.699	4892.000
ENSG00000106086	0.002	0.145	0.315	-0.026	PLEKHA8	0.015	~amyloid_beta+(1|manifest)+cngeneson+pc_mito+brain_region+Sex	0.504	5342.000
ENSG00000112787	0.009	0.144	0.281	0.008	FBRSL1	0.037	~amyloid_beta+(1|manifest)+cngeneson+pc_mito+brain_region+Sex	2.001	2430.000
ENSG00000175792	0.001	0.144	0.230	0.059	RUVBL1	0.007	~amyloid_beta+(1|manifest)+cngeneson+pc_mito+brain_region+Sex	0.331	5671.000
ENSG00000164904	0.131	0.144	0.376	-0.087	ALDH7A1	0.187	~amyloid_beta+(1|manifest)+cngeneson+pc_mito+brain_region+Sex	4.486	893.000
ENSG00000071242	0.074	0.144	0.311	-0.022	RPS6KA2	0.128	~amyloid_beta+(1|manifest)+cngeneson+pc_mito+brain_region+Sex	14.830	103.000
ENSG00000138303	0.004	0.144	0.283	0.005	ASCC1	0.024	~amyloid_beta+(1|manifest)+cngeneson+pc_mito+brain_region+Sex	0.742	4763.000
ENSG00000172766	0.011	0.144	0.336	-0.047	NAA16	0.043	~amyloid_beta+(1|manifest)+cngeneson+pc_mito+brain_region+Sex	0.441	5482.000
ENSG00000172493	0.388	0.144	0.350	-0.062	AFF1	0.437	~amyloid_beta+(1|manifest)+cngeneson+pc_mito+brain_region+Sex	4.423	909.000
ENSG00000123411	0.013	0.144	0.275	0.012	IKZF4	0.047	~amyloid_beta+(1|manifest)+cngeneson+pc_mito+brain_region+Sex	1.120	3888.000
ENSG00000119318	0.000	0.144	0.252	0.036	RAD23B	0.004	~amyloid_beta+(1|manifest)+cngeneson+pc_mito+brain_region+Sex	0.653	4991.000
ENSG00000184009	0.341	0.144	0.623	-0.335	ACTG1	0.392	~amyloid_beta+(1|manifest)+cngeneson+pc_mito+brain_region+Sex	5.092	755.000
ENSG00000105088	0.443	0.143	0.582	-0.295	OLFM2	0.488	~amyloid_beta+(1|manifest)+cngeneson+pc_mito+brain_region+Sex	5.119	748.000
ENSG00000135525	0.248	0.143	0.495	-0.209	MAP7	0.302	~amyloid_beta+(1|manifest)+cngeneson+pc_mito+brain_region+Sex	3.796	1125.000
ENSG00000088826	0.131	0.143	0.474	-0.188	SMOX	0.188	~amyloid_beta+(1|manifest)+cngeneson+pc_mito+brain_region+Sex	4.168	994.000
ENSG00000124570	0.006	0.143	0.251	0.034	SERPINB6	0.029	~amyloid_beta+(1|manifest)+cngeneson+pc_mito+brain_region+Sex	1.707	2814.000
ENSG00000135365	0.149	0.143	0.286	-0.001	PHF21A	0.205	~amyloid_beta+(1|manifest)+cngeneson+pc_mito+brain_region+Sex	1.755	2746.000
ENSG00000111832	0.003	0.143	0.269	0.017	RWDD1	0.020	~amyloid_beta+(1|manifest)+cngeneson+pc_mito+brain_region+Sex	0.240	5823.000
ENSG00000131196	0.006	0.142	0.368	-0.083	NFATC1	0.028	~amyloid_beta+(1|manifest)+cngeneson+pc_mito+brain_region+Sex	0.872	4434.000
ENSG00000169738	0.091	0.142	0.449	-0.165	DCXR	0.147	~amyloid_beta+(1|manifest)+cngeneson+pc_mito+brain_region+Sex	3.723	1152.000
ENSG00000112796	0.063	0.142	0.416	-0.132	ENPP5	0.116	~amyloid_beta+(1|manifest)+cngeneson+pc_mito+brain_region+Sex	3.379	1331.000
ENSG00000135678	0.135	0.142	0.465	-0.181	CPM	0.191	~amyloid_beta+(1|manifest)+cngeneson+pc_mito+brain_region+Sex	2.032	2393.000
ENSG00000152661	0.528	0.142	0.384	-0.100	GJA1	0.571	~amyloid_beta+(1|manifest)+cngeneson+pc_mito+brain_region+Sex	7.819	387.000
ENSG00000092445	0.032	0.142	0.424	-0.140	TYRO3	0.076	~amyloid_beta+(1|manifest)+cngeneson+pc_mito+brain_region+Sex	1.342	3423.000
ENSG00000126970	0.082	0.141	0.334	-0.051	ZC4H2	0.137	~amyloid_beta+(1|manifest)+cngeneson+pc_mito+brain_region+Sex	1.418	3297.000
ENSG00000182902	0.735	0.141	0.538	-0.255	SLC25A18	0.761	~amyloid_beta+(1|manifest)+cngeneson+pc_mito+brain_region+Sex	11.866	182.000
ENSG00000196235	0.026	0.141	0.322	-0.040	SUPT5H	0.068	~amyloid_beta+(1|manifest)+cngeneson+pc_mito+brain_region+Sex	3.665	1178.000
ENSG00000171045	0.004	0.141	0.305	-0.023	TSNARE1	0.023	~amyloid_beta+(1|manifest)+cngeneson+pc_mito+brain_region+Sex	0.698	4896.000
ENSG00000031823	0.000	0.141	0.228	0.053	RANBP3	0.005	~amyloid_beta+(1|manifest)+cngeneson+pc_mito+brain_region+Sex	0.120	5943.000
ENSG00000091127	0.003	0.141	0.273	0.009	PUS7	0.019	~amyloid_beta+(1|manifest)+cngeneson+pc_mito+brain_region+Sex	0.561	5204.000
ENSG00000132466	0.101	0.140	0.273	0.008	ANKRD17	0.157	~amyloid_beta+(1|manifest)+cngeneson+pc_mito+brain_region+Sex	0.965	4214.000
ENSG00000151718	0.000	0.140	0.242	0.039	WWC2	0.000	~amyloid_beta+(1|manifest)+cngeneson+pc_mito+brain_region+Sex	0.374	5609.000
ENSG00000173992	0.098	0.140	0.465	-0.184	CCS	0.153	~amyloid_beta+(1|manifest)+cngeneson+pc_mito+brain_region+Sex	5.217	725.000
ENSG00000131386	0.132	0.140	0.631	-0.351	GALNT15	0.188	~amyloid_beta+(1|manifest)+cngeneson+pc_mito+brain_region+Sex	2.971	1558.000
ENSG00000175175	0.149	0.140	0.430	-0.149	PPM1E	0.204	~amyloid_beta+(1|manifest)+cngeneson+pc_mito+brain_region+Sex	0.766	4702.000
ENSG00000197702	0.047	0.140	0.392	-0.112	PARVA	0.095	~amyloid_beta+(1|manifest)+cngeneson+pc_mito+brain_region+Sex	1.196	3714.000
ENSG00000011114	0.006	0.140	0.286	-0.007	BTBD7	0.028	~amyloid_beta+(1|manifest)+cngeneson+pc_mito+brain_region+Sex	2.010	2417.000
ENSG00000137288	0.005	0.140	0.259	0.020	UQCC2	0.026	~amyloid_beta+(1|manifest)+cngeneson+pc_mito+brain_region+Sex	0.924	4319.000
ENSG00000278259	0.001	0.140	0.233	0.047	MYO19	0.010	~amyloid_beta+(1|manifest)+cngeneson+pc_mito+brain_region+Sex	0.544	5247.000
ENSG00000162923	0.003	0.140	0.273	0.006	WDR26	0.022	~amyloid_beta+(1|manifest)+cngeneson+pc_mito+brain_region+Sex	0.521	5305.000
ENSG00000111783	0.005	0.139	0.222	0.057	RFX4	0.025	~amyloid_beta+(1|manifest)+cngeneson+pc_mito+brain_region+Sex	5.309	708.000
ENSG00000113742	0.165	0.139	0.278	0.001	CPEB4	0.220	~amyloid_beta+(1|manifest)+cngeneson+pc_mito+brain_region+Sex	4.671	851.000
ENSG00000153982	0.007	0.139	0.279	0.000	GDPD1	0.033	~amyloid_beta+(1|manifest)+cngeneson+pc_mito+brain_region+Sex	0.710	4847.000
ENSG00000013441	0.086	0.139	0.444	-0.166	CLK1	0.141	~amyloid_beta+(1|manifest)+cngeneson+pc_mito+brain_region+Sex	4.897	799.000
ENSG00000119640	0.001	0.139	0.235	0.043	ACYP1	0.010	~amyloid_beta+(1|manifest)+cngeneson+pc_mito+brain_region+Sex	0.805	4619.000
ENSG00000116473	0.003	0.139	0.304	-0.026	RAP1A	0.019	~amyloid_beta+(1|manifest)+cngeneson+pc_mito+brain_region+Sex	0.791	4652.000
ENSG00000123143	0.003	0.139	0.257	0.021	PKN1	0.020	~amyloid_beta+(1|manifest)+cngeneson+pc_mito+brain_region+Sex	1.233	3631.000
ENSG00000148842	0.039	0.139	0.350	-0.072	CNNM2	0.086	~amyloid_beta+(1|manifest)+cngeneson+pc_mito+brain_region+Sex	1.441	3246.000
ENSG00000106829	0.039	0.139	0.358	-0.080	TLE4	0.086	~amyloid_beta+(1|manifest)+cngeneson+pc_mito+brain_region+Sex	0.595	5140.000
ENSG00000178177	0.088	0.139	0.406	-0.129	LCORL	0.142	~amyloid_beta+(1|manifest)+cngeneson+pc_mito+brain_region+Sex	1.422	3286.000
ENSG00000171681	0.088	0.139	0.388	-0.110	ATF7IP	0.143	~amyloid_beta+(1|manifest)+cngeneson+pc_mito+brain_region+Sex	0.416	5534.000
ENSG00000250305	0.096	0.139	0.458	-0.181	TRMT9B	0.151	~amyloid_beta+(1|manifest)+cngeneson+pc_mito+brain_region+Sex	8.198	362.000
ENSG00000134480	0.134	0.139	0.316	-0.039	CCNH	0.190	~amyloid_beta+(1|manifest)+cngeneson+pc_mito+brain_region+Sex	1.823	2662.000
ENSG00000168807	0.001	0.139	0.325	-0.048	SNTB2	0.010	~amyloid_beta+(1|manifest)+cngeneson+pc_mito+brain_region+Sex	1.349	3415.000
ENSG00000177189	0.052	0.138	0.378	-0.101	RPS6KA3	0.102	~amyloid_beta+(1|manifest)+cngeneson+pc_mito+brain_region+Sex	1.306	3494.000
ENSG00000196365	0.038	0.138	0.307	-0.030	LONP1	0.085	~amyloid_beta+(1|manifest)+cngeneson+pc_mito+brain_region+Sex	2.781	1680.000
ENSG00000145794	0.435	0.138	0.392	-0.115	MEGF10	0.481	~amyloid_beta+(1|manifest)+cngeneson+pc_mito+brain_region+Sex	1.806	2686.000
ENSG00000125398	0.310	0.138	0.408	-0.131	SOX9	0.363	~amyloid_beta+(1|manifest)+cngeneson+pc_mito+brain_region+Sex	3.253	1393.000
ENSG00000103043	0.002	0.138	0.244	0.032	VAC14	0.017	~amyloid_beta+(1|manifest)+cngeneson+pc_mito+brain_region+Sex	0.737	4777.000
ENSG00000069188	0.002	0.138	0.286	-0.010	SDK2	0.016	~amyloid_beta+(1|manifest)+cngeneson+pc_mito+brain_region+Sex	0.508	5331.000
ENSG00000168434	0.006	0.138	0.263	0.014	COG7	0.029	~amyloid_beta+(1|manifest)+cngeneson+pc_mito+brain_region+Sex	0.881	4416.000
ENSG00000125779	0.011	0.138	0.303	-0.026	PANK2	0.041	~amyloid_beta+(1|manifest)+cngeneson+pc_mito+brain_region+Sex	0.764	4707.000
ENSG00000148356	0.025	0.138	0.363	-0.087	LRSAM1	0.067	~amyloid_beta+(1|manifest)+cngeneson+pc_mito+brain_region+Sex	2.106	2306.000
ENSG00000149633	0.066	0.138	0.470	-0.194	KIAA1755	0.120	~amyloid_beta+(1|manifest)+cngeneson+pc_mito+brain_region+Sex	6.357	543.000
ENSG00000109189	0.007	0.138	0.297	-0.022	USP46	0.032	~amyloid_beta+(1|manifest)+cngeneson+pc_mito+brain_region+Sex	1.134	3852.000
ENSG00000110514	0.006	0.138	0.267	0.008	MADD	0.030	~amyloid_beta+(1|manifest)+cngeneson+pc_mito+brain_region+Sex	0.838	4522.000
ENSG00000112561	0.006	0.138	0.248	0.027	TFEB	0.028	~amyloid_beta+(1|manifest)+cngeneson+pc_mito+brain_region+Sex	0.258	5797.000
ENSG00000163126	0.030	0.137	0.281	-0.006	ANKRD23	0.075	~amyloid_beta+(1|manifest)+cngeneson+pc_mito+brain_region+Sex	1.387	3344.000
ENSG00000071082	0.017	0.137	0.328	-0.055	RPL31	0.053	~amyloid_beta+(1|manifest)+cngeneson+pc_mito+brain_region+Sex	1.001	4135.000
ENSG00000105821	0.000	0.136	0.216	0.057	DNAJC2	0.004	~amyloid_beta+(1|manifest)+cngeneson+pc_mito+brain_region+Sex	0.223	5845.000
ENSG00000119185	0.006	0.136	0.332	-0.059	ITGB1BP1	0.029	~amyloid_beta+(1|manifest)+cngeneson+pc_mito+brain_region+Sex	0.245	5815.000
ENSG00000132563	0.010	0.136	0.278	-0.006	REEP2	0.040	~amyloid_beta+(1|manifest)+cngeneson+pc_mito+brain_region+Sex	0.842	4518.000
ENSG00000070610	0.033	0.136	0.299	-0.027	GBA2	0.078	~amyloid_beta+(1|manifest)+cngeneson+pc_mito+brain_region+Sex	2.117	2293.000
ENSG00000176595	0.008	0.136	0.303	-0.031	KBTBD11	0.034	~amyloid_beta+(1|manifest)+cngeneson+pc_mito+brain_region+Sex	0.750	4742.000
ENSG00000137845	0.005	0.136	0.292	-0.020	ADAM10	0.027	~amyloid_beta+(1|manifest)+cngeneson+pc_mito+brain_region+Sex	2.053	2363.000
ENSG00000132323	0.008	0.136	0.264	0.008	ILKAP	0.035	~amyloid_beta+(1|manifest)+cngeneson+pc_mito+brain_region+Sex	1.098	3934.000
ENSG00000116641	0.045	0.136	0.250	0.021	DOCK7	0.094	~amyloid_beta+(1|manifest)+cngeneson+pc_mito+brain_region+Sex	2.159	2250.000
ENSG00000162341	0.004	0.136	0.356	-0.085	TPCN2	0.023	~amyloid_beta+(1|manifest)+cngeneson+pc_mito+brain_region+Sex	2.041	2380.000
ENSG00000115677	0.009	0.136	0.295	-0.024	HDLBP	0.038	~amyloid_beta+(1|manifest)+cngeneson+pc_mito+brain_region+Sex	0.808	4607.000
ENSG00000105696	0.124	0.135	0.432	-0.162	TMEM59L	0.180	~amyloid_beta+(1|manifest)+cngeneson+pc_mito+brain_region+Sex	2.426	1959.000
ENSG00000172985	0.205	0.135	0.499	-0.229	SH3RF3	0.260	~amyloid_beta+(1|manifest)+cngeneson+pc_mito+brain_region+Sex	4.322	942.000
ENSG00000116106	0.220	0.135	0.512	-0.241	EPHA4	0.275	~amyloid_beta+(1|manifest)+cngeneson+pc_mito+brain_region+Sex	3.861	1100.000
ENSG00000010256	0.009	0.135	0.274	-0.004	UQCRC1	0.036	~amyloid_beta+(1|manifest)+cngeneson+pc_mito+brain_region+Sex	1.095	3939.000
ENSG00000196535	0.005	0.135	0.408	-0.138	MYO18A	0.028	~amyloid_beta+(1|manifest)+cngeneson+pc_mito+brain_region+Sex	3.433	1289.000
ENSG00000198380	0.000	0.135	0.206	0.064	GFPT1	0.002	~amyloid_beta+(1|manifest)+cngeneson+pc_mito+brain_region+Sex	0.059	5973.000
ENSG00000172458	0.148	0.135	0.404	-0.134	IL17D	0.204	~amyloid_beta+(1|manifest)+cngeneson+pc_mito+brain_region+Sex	1.746	2754.000
ENSG00000198556	0.000	0.135	0.223	0.047	ZNF789	0.007	~amyloid_beta+(1|manifest)+cngeneson+pc_mito+brain_region+Sex	0.645	5016.000
ENSG00000012171	0.016	0.135	0.526	-0.256	SEMA3B	0.053	~amyloid_beta+(1|manifest)+cngeneson+pc_mito+brain_region+Sex	3.307	1366.000
ENSG00000173692	0.018	0.135	0.290	-0.021	PSMD1	0.056	~amyloid_beta+(1|manifest)+cngeneson+pc_mito+brain_region+Sex	1.083	3963.000
ENSG00000091542	0.022	0.135	0.295	-0.026	ALKBH5	0.063	~amyloid_beta+(1|manifest)+cngeneson+pc_mito+brain_region+Sex	0.795	4643.000
ENSG00000152763	0.021	0.135	0.269	0.001	WDR78	0.062	~amyloid_beta+(1|manifest)+cngeneson+pc_mito+brain_region+Sex	0.412	5542.000
ENSG00000266074	0.024	0.135	0.297	-0.028	BAHCC1	0.066	~amyloid_beta+(1|manifest)+cngeneson+pc_mito+brain_region+Sex	0.526	5296.000
ENSG00000185963	0.017	0.135	0.280	-0.010	BICD2	0.055	~amyloid_beta+(1|manifest)+cngeneson+pc_mito+brain_region+Sex	0.347	5657.000
ENSG00000147121	0.015	0.134	0.276	-0.008	KRBOX4	0.050	~amyloid_beta+(1|manifest)+cngeneson+pc_mito+brain_region+Sex	0.759	4721.000
ENSG00000008086	0.026	0.134	0.390	-0.122	CDKL5	0.069	~amyloid_beta+(1|manifest)+cngeneson+pc_mito+brain_region+Sex	0.649	5004.000
ENSG00000124215	0.068	0.134	0.389	-0.120	CDH26	0.121	~amyloid_beta+(1|manifest)+cngeneson+pc_mito+brain_region+Sex	1.220	3664.000
ENSG00000167792	0.122	0.134	0.427	-0.160	NDUFV1	0.178	~amyloid_beta+(1|manifest)+cngeneson+pc_mito+brain_region+Sex	5.183	731.000
ENSG00000112081	0.026	0.134	0.285	-0.018	SRSF3	0.068	~amyloid_beta+(1|manifest)+cngeneson+pc_mito+brain_region+Sex	1.146	3823.000
ENSG00000214941	0.013	0.134	0.270	-0.002	ZSWIM7	0.047	~amyloid_beta+(1|manifest)+cngeneson+pc_mito+brain_region+Sex	1.208	3687.000
ENSG00000204954	0.000	0.134	0.191	0.077	C12orf73	0.004	~amyloid_beta+(1|manifest)+cngeneson+pc_mito+brain_region+Sex	0.710	4846.000
ENSG00000064999	0.004	0.134	0.230	0.037	ANKS1A	0.022	~amyloid_beta+(1|manifest)+cngeneson+pc_mito+brain_region+Sex	0.443	5476.000
ENSG00000170500	0.033	0.133	0.355	-0.088	LONRF2	0.078	~amyloid_beta+(1|manifest)+cngeneson+pc_mito+brain_region+Sex	1.291	3525.000
ENSG00000158636	0.026	0.133	0.311	-0.045	EMSY	0.068	~amyloid_beta+(1|manifest)+cngeneson+pc_mito+brain_region+Sex	2.122	2286.000
ENSG00000136603	0.002	0.133	0.243	0.023	SKIL	0.018	~amyloid_beta+(1|manifest)+cngeneson+pc_mito+brain_region+Sex	0.745	4756.000
ENSG00000160613	0.033	0.133	0.315	-0.050	PCSK7	0.077	~amyloid_beta+(1|manifest)+cngeneson+pc_mito+brain_region+Sex	1.966	2469.000
ENSG00000169813	0.006	0.133	0.266	0.000	HNRNPF	0.029	~amyloid_beta+(1|manifest)+cngeneson+pc_mito+brain_region+Sex	0.707	4859.000
ENSG00000127616	0.015	0.133	0.348	-0.082	SMARCA4	0.050	~amyloid_beta+(1|manifest)+cngeneson+pc_mito+brain_region+Sex	1.995	2438.000
ENSG00000009830	0.002	0.133	0.223	0.042	POMT2	0.015	~amyloid_beta+(1|manifest)+cngeneson+pc_mito+brain_region+Sex	0.513	5322.000
ENSG00000164896	0.050	0.133	0.357	-0.092	FASTK	0.099	~amyloid_beta+(1|manifest)+cngeneson+pc_mito+brain_region+Sex	2.030	2395.000
ENSG00000050393	0.006	0.132	0.250	0.014	MCUR1	0.028	~amyloid_beta+(1|manifest)+cngeneson+pc_mito+brain_region+Sex	1.415	3300.000
ENSG00000133243	0.001	0.132	0.218	0.046	BTBD2	0.012	~amyloid_beta+(1|manifest)+cngeneson+pc_mito+brain_region+Sex	0.760	4717.000
ENSG00000177606	0.520	0.132	0.726	-0.462	JUN	0.563	~amyloid_beta+(1|manifest)+cngeneson+pc_mito+brain_region+Sex	3.387	1325.000
ENSG00000235194	0.034	0.132	0.333	-0.069	PPP1R3E	0.080	~amyloid_beta+(1|manifest)+cngeneson+pc_mito+brain_region+Sex	1.974	2462.000
ENSG00000130876	0.047	0.132	0.436	-0.172	SLC7A10	0.096	~amyloid_beta+(1|manifest)+cngeneson+pc_mito+brain_region+Sex	3.656	1183.000
ENSG00000100209	0.024	0.132	0.298	-0.035	HSCB	0.066	~amyloid_beta+(1|manifest)+cngeneson+pc_mito+brain_region+Sex	1.489	3160.000
ENSG00000014641	0.017	0.132	0.275	-0.011	MDH1	0.055	~amyloid_beta+(1|manifest)+cngeneson+pc_mito+brain_region+Sex	0.676	4946.000
ENSG00000108021	0.005	0.132	0.277	-0.014	TASOR2	0.027	~amyloid_beta+(1|manifest)+cngeneson+pc_mito+brain_region+Sex	0.809	4604.000
ENSG00000140398	0.014	0.132	0.271	-0.007	NEIL1	0.047	~amyloid_beta+(1|manifest)+cngeneson+pc_mito+brain_region+Sex	1.388	3341.000
ENSG00000185324	0.093	0.132	0.373	-0.109	CDK10	0.148	~amyloid_beta+(1|manifest)+cngeneson+pc_mito+brain_region+Sex	2.233	2169.000
ENSG00000198053	0.298	0.132	0.360	-0.096	SIRPA	0.351	~amyloid_beta+(1|manifest)+cngeneson+pc_mito+brain_region+Sex	3.357	1338.000
ENSG00000165185	0.234	0.132	0.302	-0.039	KIAA1958	0.288	~amyloid_beta+(1|manifest)+cngeneson+pc_mito+brain_region+Sex	2.780	1682.000
ENSG00000221869	0.244	0.131	0.642	-0.379	CEBPD	0.298	~amyloid_beta+(1|manifest)+cngeneson+pc_mito+brain_region+Sex	5.767	632.000
ENSG00000004897	0.001	0.131	0.235	0.027	CDC27	0.012	~amyloid_beta+(1|manifest)+cngeneson+pc_mito+brain_region+Sex	1.123	3881.000
ENSG00000173402	0.026	0.131	0.308	-0.046	DAG1	0.069	~amyloid_beta+(1|manifest)+cngeneson+pc_mito+brain_region+Sex	0.512	5325.000
ENSG00000204084	0.001	0.131	0.268	-0.006	INPP5B	0.013	~amyloid_beta+(1|manifest)+cngeneson+pc_mito+brain_region+Sex	0.619	5086.000
ENSG00000197555	0.215	0.131	0.275	-0.013	SIPA1L1	0.269	~amyloid_beta+(1|manifest)+cngeneson+pc_mito+brain_region+Sex	3.851	1104.000
ENSG00000105737	0.000	0.131	0.195	0.067	GRIK5	0.001	~amyloid_beta+(1|manifest)+cngeneson+pc_mito+brain_region+Sex	0.241	5819.000
ENSG00000184677	0.002	0.131	0.254	0.008	ZBTB40	0.018	~amyloid_beta+(1|manifest)+cngeneson+pc_mito+brain_region+Sex	0.514	5320.000
ENSG00000130939	0.001	0.131	0.278	-0.016	UBE4B	0.010	~amyloid_beta+(1|manifest)+cngeneson+pc_mito+brain_region+Sex	1.209	3684.000
ENSG00000106615	0.147	0.131	0.356	-0.095	RHEB	0.203	~amyloid_beta+(1|manifest)+cngeneson+pc_mito+brain_region+Sex	1.728	2784.000
ENSG00000159228	0.005	0.131	0.337	-0.076	CBR1	0.026	~amyloid_beta+(1|manifest)+cngeneson+pc_mito+brain_region+Sex	0.993	4157.000
ENSG00000099246	0.009	0.131	0.258	0.003	RAB18	0.037	~amyloid_beta+(1|manifest)+cngeneson+pc_mito+brain_region+Sex	0.774	4678.000
ENSG00000058262	0.045	0.130	0.386	-0.126	SEC61A1	0.094	~amyloid_beta+(1|manifest)+cngeneson+pc_mito+brain_region+Sex	0.609	5111.000
ENSG00000122565	0.001	0.130	0.212	0.048	CBX3	0.010	~amyloid_beta+(1|manifest)+cngeneson+pc_mito+brain_region+Sex	0.173	5907.000
ENSG00000114948	0.148	0.130	0.491	-0.231	ADAM23	0.204	~amyloid_beta+(1|manifest)+cngeneson+pc_mito+brain_region+Sex	4.374	925.000
ENSG00000183431	0.024	0.130	0.267	-0.007	SF3A3	0.066	~amyloid_beta+(1|manifest)+cngeneson+pc_mito+brain_region+Sex	0.552	5227.000
ENSG00000164463	0.005	0.130	0.285	-0.025	CREBRF	0.027	~amyloid_beta+(1|manifest)+cngeneson+pc_mito+brain_region+Sex	0.215	5853.000
ENSG00000150457	0.003	0.130	0.233	0.026	LATS2	0.020	~amyloid_beta+(1|manifest)+cngeneson+pc_mito+brain_region+Sex	0.196	5875.000

ENSG00000186184	0.000	0.130	0.186	0.073	POLR1D	0.001	~amyloid_beta+(1|manifest)+cngeneson+pc_mito+brain_region+Sex	0.498	5367.000
ENSG00000104852	0.588	0.129	0.607	-0.349	SNRNP70	0.625	~amyloid_beta+(1|manifest)+cngeneson+pc_mito+brain_region+Sex	17.616	72.000
ENSG00000250312	0.024	0.129	0.293	-0.034	ZNF718	0.066	~amyloid_beta+(1|manifest)+cngeneson+pc_mito+brain_region+Sex	2.288	2118.000
ENSG00000188153	0.040	0.129	0.455	-0.197	COL4A5	0.088	~amyloid_beta+(1|manifest)+cngeneson+pc_mito+brain_region+Sex	0.510	5328.000
ENSG00000123983	0.063	0.129	0.282	-0.024	ACSL3	0.116	~amyloid_beta+(1|manifest)+cngeneson+pc_mito+brain_region+Sex	0.853	4490.000
ENSG00000167996	0.834	0.129	0.606	-0.349	FTH1	0.851	~amyloid_beta+(1|manifest)+cngeneson+pc_mito+brain_region+Sex	8.332	356.000
ENSG00000019144	0.162	0.129	0.416	-0.158	PHLDB1	0.217	~amyloid_beta+(1|manifest)+cngeneson+pc_mito+brain_region+Sex	8.489	347.000
ENSG00000103091	0.002	0.129	0.304	-0.047	WDR59	0.016	~amyloid_beta+(1|manifest)+cngeneson+pc_mito+brain_region+Sex	2.141	2272.000
ENSG00000005339	0.003	0.129	0.230	0.027	CREBBP	0.019	~amyloid_beta+(1|manifest)+cngeneson+pc_mito+brain_region+Sex	0.858	4469.000
ENSG00000162174	0.178	0.129	0.532	-0.275	ASRGL1	0.233	~amyloid_beta+(1|manifest)+cngeneson+pc_mito+brain_region+Sex	7.981	377.000
ENSG00000142089	0.122	0.129	0.872	-0.615	IFITM3	0.179	~amyloid_beta+(1|manifest)+cngeneson+pc_mito+brain_region+Sex	5.685	652.000
ENSG00000111837	0.031	0.129	0.353	-0.096	MAK	0.075	~amyloid_beta+(1|manifest)+cngeneson+pc_mito+brain_region+Sex	3.436	1287.000
ENSG00000053770	0.010	0.128	0.261	-0.005	AP5M1	0.039	~amyloid_beta+(1|manifest)+cngeneson+pc_mito+brain_region+Sex	0.146	5929.000
ENSG00000110955	0.052	0.128	0.344	-0.088	ATP5F1B	0.102	~amyloid_beta+(1|manifest)+cngeneson+pc_mito+brain_region+Sex	0.951	4253.000
ENSG00000113657	0.144	0.128	0.516	-0.260	DPYSL3	0.200	~amyloid_beta+(1|manifest)+cngeneson+pc_mito+brain_region+Sex	5.120	746.000
ENSG00000165792	0.070	0.128	0.336	-0.080	METTL17	0.124	~amyloid_beta+(1|manifest)+cngeneson+pc_mito+brain_region+Sex	2.781	1681.000
ENSG00000163516	0.018	0.128	0.306	-0.050	ANKZF1	0.056	~amyloid_beta+(1|manifest)+cngeneson+pc_mito+brain_region+Sex	2.540	1868.000
ENSG00000106536	0.650	0.127	0.661	-0.407	POU6F2	0.683	~amyloid_beta+(1|manifest)+cngeneson+pc_mito+brain_region+Sex	12.718	160.000
ENSG00000206418	0.002	0.127	0.219	0.036	RAB12	0.017	~amyloid_beta+(1|manifest)+cngeneson+pc_mito+brain_region+Sex	0.196	5874.000
ENSG00000165626	0.012	0.127	0.315	-0.061	BEND7	0.043	~amyloid_beta+(1|manifest)+cngeneson+pc_mito+brain_region+Sex	0.191	5884.000
ENSG00000154814	0.004	0.127	0.231	0.023	OXNAD1	0.024	~amyloid_beta+(1|manifest)+cngeneson+pc_mito+brain_region+Sex	0.443	5475.000
ENSG00000109689	0.317	0.127	0.418	-0.164	STIM2	0.369	~amyloid_beta+(1|manifest)+cngeneson+pc_mito+brain_region+Sex	2.921	1593.000
ENSG00000157927	0.005	0.127	0.401	-0.147	RADIL	0.027	~amyloid_beta+(1|manifest)+cngeneson+pc_mito+brain_region+Sex	2.438	1943.000
ENSG00000100403	0.005	0.127	0.234	0.020	ZC3H7B	0.028	~amyloid_beta+(1|manifest)+cngeneson+pc_mito+brain_region+Sex	0.751	4740.000
ENSG00000100243	0.014	0.127	0.287	-0.033	CYB5R3	0.049	~amyloid_beta+(1|manifest)+cngeneson+pc_mito+brain_region+Sex	0.860	4465.000
ENSG00000119041	0.004	0.127	0.245	0.009	GTF3C3	0.022	~amyloid_beta+(1|manifest)+cngeneson+pc_mito+brain_region+Sex	0.217	5849.000
ENSG00000132155	0.009	0.127	0.245	0.008	RAF1	0.037	~amyloid_beta+(1|manifest)+cngeneson+pc_mito+brain_region+Sex	1.896	2556.000
ENSG00000104885	0.020	0.127	0.372	-0.119	DOT1L	0.059	~amyloid_beta+(1|manifest)+cngeneson+pc_mito+brain_region+Sex	2.399	1983.000
ENSG00000180891	0.013	0.126	0.339	-0.086	CUEDC1	0.046	~amyloid_beta+(1|manifest)+cngeneson+pc_mito+brain_region+Sex	1.661	2884.000
ENSG00000177082	0.006	0.126	0.290	-0.037	WDR73	0.028	~amyloid_beta+(1|manifest)+cngeneson+pc_mito+brain_region+Sex	1.830	2654.000
ENSG00000109501	0.227	0.126	0.497	-0.245	WFS1	0.281	~amyloid_beta+(1|manifest)+cngeneson+pc_mito+brain_region+Sex	2.619	1800.000
ENSG00000162889	0.014	0.126	0.332	-0.080	[illegible]	0.048	~amyloid_beta+(1|manifest)+cngeneson+pc_mito+brain_region+Sex	2.752	1701.000
ENSG00000168014	0.004	0.126	0.237	0.015	C2CD3	0.024	~amyloid_beta+(1|manifest)+cngeneson+pc_mito+brain_region+Sex	1.030	4077.000
ENSG00000011566	0.006	0.126	0.217	0.035	MAP4K3	0.029	~amyloid_beta+(1|manifest)+cngeneson+pc_mito+brain_region+Sex	3.317	1359.000
ENSG00000115977	0.018	0.126	0.258	-0.007	AAK1	0.055	~amyloid_beta+(1|manifest)+cngeneson+pc_mito+brain_region+Sex	1.208	3686.000
ENSG00000072071	0.053	0.126	0.328	-0.076	ADGRL1	0.103	~amyloid_beta+(1|manifest)+cngeneson+pc_mito+brain_region+Sex	1.723	2793.000
ENSG00000263001	0.011	0.126	0.262	-0.010	GTF2I	0.041	~amyloid_beta+(1|manifest)+cngeneson+pc_mito+brain_region+Sex	1.891	2573.000
ENSG00000107104	0.151	0.126	0.247	0.004	KANK1	0.206	~amyloid_beta+(1|manifest)+cngeneson+pc_mito+brain_region+Sex	2.583	1825.000
ENSG00000159363	0.033	0.125	0.273	-0.022	ATP13A2	0.078	~amyloid_beta+(1|manifest)+cngeneson+pc_mito+brain_region+Sex	1.275	3556.000
ENSG00000102144	0.257	0.125	0.455	-0.204	PGK1	0.311	~amyloid_beta+(1|manifest)+cngeneson+pc_mito+brain_region+Sex	0.890	4399.000
ENSG00000134352	0.170	0.125	0.390	-0.140	IL6ST	0.225	~amyloid_beta+(1|manifest)+cngeneson+pc_mito+brain_region+Sex	1.624	2947.000
ENSG00000170542	0.038	0.125	0.325	-0.075	SERPINB9	0.085	~amyloid_beta+(1|manifest)+cngeneson+pc_mito+brain_region+Sex	1.457	3210.000
ENSG00000116030	0.022	0.125	0.283	-0.033	SUMO1	0.063	~amyloid_beta+(1|manifest)+cngeneson+pc_mito+brain_region+Sex	0.272	5778.000
ENSG00000158470	0.158	0.125	0.419	-0.169	B4GALT5	0.213	~amyloid_beta+(1|manifest)+cngeneson+pc_mito+brain_region+Sex	4.415	912.000
ENSG00000128789	0.107	0.125	0.426	-0.177	PSMG2	0.163	~amyloid_beta+(1|manifest)+cngeneson+pc_mito+brain_region+Sex	2.387	2006.000
ENSG00000149311	0.041	0.124	0.273	-0.025	ATM	0.088	~amyloid_beta+(1|manifest)+cngeneson+pc_mito+brain_region+Sex	1.294	3517.000
ENSG00000132196	0.003	0.124	0.314	-0.066	HSD17B7	0.018	~amyloid_beta+(1|manifest)+cngeneson+pc_mito+brain_region+Sex	2.955	1568.000
ENSG00000197024	0.003	0.124	0.237	0.011	ZNF398	0.021	~amyloid_beta+(1|manifest)+cngeneson+pc_mito+brain_region+Sex	0.758	4722.000
ENSG00000076984	0.004	0.124	0.219	0.029	MAP2K7	0.024	~amyloid_beta+(1|manifest)+cngeneson+pc_mito+brain_region+Sex	1.303	3501.000
ENSG00000078114	0.030	0.124	0.256	-0.008	NEBL	0.074	~amyloid_beta+(1|manifest)+cngeneson+pc_mito+brain_region+Sex	6.772	496.000
ENSG00000177565	0.336	0.124	0.388	-0.141	TBL1XR1	0.388	~amyloid_beta+(1|manifest)+cngeneson+pc_mito+brain_region+Sex	1.091	3947.000
ENSG00000233757	0.003	0.124	0.242	0.006	[illegible]	0.018	~amyloid_beta+(1|manifest)+cngeneson+pc_mito+brain_region+Sex	1.353	3404.000
ENSG00000039523	0.015	0.124	0.264	-0.017	RIPOR1	0.049	~amyloid_beta+(1|manifest)+cngeneson+pc_mito+brain_region+Sex	0.756	4724.000
ENSG00000141314	0.145	0.124	0.419	-0.171	RHBDL3	0.201	~amyloid_beta+(1|manifest)+cngeneson+pc_mito+brain_region+Sex	7.437	422.000
ENSG00000076716	0.026	0.124	0.405	-0.158	GPC4	0.069	~amyloid_beta+(1|manifest)+cngeneson+pc_mito+brain_region+Sex	0.316	5701.000
ENSG00000164983	0.002	0.123	0.285	-0.039	TMEM65	0.015	~amyloid_beta+(1|manifest)+cngeneson+pc_mito+brain_region+Sex	1.174	3761.000
ENSG00000112851	0.280	0.123	0.283	-0.036	ERBIN	0.333	~amyloid_beta+(1|manifest)+cngeneson+pc_mito+brain_region+Sex	7.322	437.000
ENSG00000132692	0.412	0.123	0.687	-0.441	BCAN	0.459	~amyloid_beta+(1|manifest)+cngeneson+pc_mito+brain_region+Sex	6.852	486.000
ENSG00000135205	0.007	0.123	0.247	-0.001	CCDC146	0.032	~amyloid_beta+(1|manifest)+cngeneson+pc_mito+brain_region+Sex	0.504	5345.000
ENSG00000125037	0.008	0.123	0.288	-0.042	EMC3	0.035	~amyloid_beta+(1|manifest)+cngeneson+pc_mito+brain_region+Sex	1.949	2494.000
ENSG00000106009	0.048	0.123	0.308	-0.061	BRAT1	0.097	~amyloid_beta+(1|manifest)+cngeneson+pc_mito+brain_region+Sex	2.377	2021.000
ENSG00000133424	0.050	0.123	0.299	-0.053	LARGE1	0.099	~amyloid_beta+(1|manifest)+cngeneson+pc_mito+brain_region+Sex	0.789	4654.000
ENSG00000138107	0.003	0.123	0.226	0.020	ACTR1A	0.021	~amyloid_beta+(1|manifest)+cngeneson+pc_mito+brain_region+Sex	0.674	4953.000
ENSG00000100442	0.006	0.123	0.242	0.004	FKBP3	0.029	~amyloid_beta+(1|manifest)+cngeneson+pc_mito+brain_region+Sex	0.973	4200.000
ENSG00000176171	0.277	0.123	0.409	-0.163	BNIP3	0.330	~amyloid_beta+(1|manifest)+cngeneson+pc_mito+brain_region+Sex	2.034	2390.000
ENSG00000181045	0.022	0.123	0.308	-0.063	SLC26A11	0.063	~amyloid_beta+(1|manifest)+cngeneson+pc_mito+brain_region+Sex	1.765	2727.000
ENSG00000099942	0.002	0.122	0.211	0.033	CRKL	0.015	~amyloid_beta+(1|manifest)+cngeneson+pc_mito+brain_region+Sex	0.242	5816.000
ENSG00000167291	0.002	0.122	0.211	0.034	TBC1D16	0.015	~amyloid_beta+(1|manifest)+cngeneson+pc_mito+brain_region+Sex	0.531	5284.000
ENSG00000159593	0.001	0.122	0.216	0.028	NAE1	0.013	~amyloid_beta+(1|manifest)+cngeneson+pc_mito+brain_region+Sex	0.818	4579.000
ENSG00000168026	0.010	0.122	0.315	-0.070	TTC21A	0.039	~amyloid_beta+(1|manifest)+cngeneson+pc_mito+brain_region+Sex	1.760	2738.000
ENSG00000170558	0.424	0.122	0.307	-0.063	CDH2	0.470	~amyloid_beta+(1|manifest)+cngeneson+pc_mito+brain_region+Sex	3.184	1434.000
ENSG00000161509	0.268	0.122	0.527	-0.283	GRIN2C	0.322	~amyloid_beta+(1|manifest)+cngeneson+pc_mito+brain_region+Sex	6.311	547.000
ENSG00000174672	0.040	0.122	0.355	-0.112	BRSK2	0.088	~amyloid_beta+(1|manifest)+cngeneson+pc_mito+brain_region+Sex	3.418	1302.000
ENSG00000171475	0.178	0.122	0.319	-0.075	WIPF2	0.233	~amyloid_beta+(1|manifest)+cngeneson+pc_mito+brain_region+Sex	2.069	2348.000
ENSG00000173120	0.027	0.122	0.268	-0.025	KDM2A	0.070	~amyloid_beta+(1|manifest)+cngeneson+pc_mito+brain_region+Sex	1.338	3433.000
ENSG00000171867	0.295	0.121	0.396	-0.154	PRNP	0.347	~amyloid_beta+(1|manifest)+cngeneson+pc_mito+brain_region+Sex	1.326	3450.000
ENSG00000203666	0.438	0.121	0.425	-0.183	EFCAB2	0.484	~amyloid_beta+(1|manifest)+cngeneson+pc_mito+brain_region+Sex	1.410	3308.000
ENSG00000135299	0.032	0.121	0.503	-0.261	ANKRD6	0.077	~amyloid_beta+(1|manifest)+cngeneson+pc_mito+brain_region+Sex	0.421	5524.000
ENSG00000180801	0.126	0.121	0.360	-0.118	ARSJ	0.182	~amyloid_beta+(1|manifest)+cngeneson+pc_mito+brain_region+Sex	1.019	4098.000
ENSG00000163349	0.003	0.121	0.266	-0.024	HIPK1	0.019	~amyloid_beta+(1|manifest)+cngeneson+pc_mito+brain_region+Sex	0.754	4729.000
ENSG00000160710	0.014	0.121	0.286	-0.045	ADAR	0.047	~amyloid_beta+(1|manifest)+cngeneson+pc_mito+brain_region+Sex	1.699	2822.000
ENSG00000187699	0.037	0.121	0.278	-0.036	C2orf88	0.083	~amyloid_beta+(1|manifest)+cngeneson+pc_mito+brain_region+Sex	0.978	4188.000
ENSG00000100461	0.017	0.121	0.239	0.002	RBM23	0.054	~amyloid_beta+(1|manifest)+cngeneson+pc_mito+brain_region+Sex	0.915	4342.000
ENSG00000138363	0.003	0.121	0.219	0.022	ATIC	0.021	~amyloid_beta+(1|manifest)+cngeneson+pc_mito+brain_region+Sex	0.387	5585.000
ENSG00000072736	0.004	0.121	0.232	0.009	NFATC3	0.022	~amyloid_beta+(1|manifest)+cngeneson+pc_mito+brain_region+Sex	0.687	4924.000
ENSG00000012048	0.003	0.120	0.215	0.026	BRCA1	0.019	~amyloid_beta+(1|manifest)+cngeneson+pc_mito+brain_region+Sex	0.602	5123.000
ENSG00000276293	0.003	0.120	0.213	0.028	PIP4K2B	0.018	~amyloid_beta+(1|manifest)+cngeneson+pc_mito+brain_region+Sex	0.891	4393.000
ENSG00000185238	0.061	0.120	0.347	-0.107	PRMT3	0.113	~amyloid_beta+(1|manifest)+cngeneson+pc_mito+brain_region+Sex	0.470	5418.000
ENSG00000072958	0.001	0.120	0.206	0.034	AP1M1	0.011	~amyloid_beta+(1|manifest)+cngeneson+pc_mito+brain_region+Sex	0.100	5954.000
ENSG00000183741	0.004	0.120	0.218	0.022	CBX6	0.023	~amyloid_beta+(1|manifest)+cngeneson+pc_mito+brain_region+Sex	0.195	5876.000
ENSG00000168883	0.087	0.120	0.334	-0.095	USP39	0.142	~amyloid_beta+(1|manifest)+cngeneson+pc_mito+brain_region+Sex	1.692	2836.000
ENSG00000285082	0.165	0.120	0.545	-0.306	[illegible]	0.220	~amyloid_beta+(1|manifest)+cngeneson+pc_mito+brain_region+Sex	5.088	756.000
ENSG00000152556	0.020	0.120	0.263	-0.024	PFKM	0.060	~amyloid_beta+(1|manifest)+cngeneson+pc_mito+brain_region+Sex	0.831	4540.000
ENSG00000149930	0.025	0.120	0.293	-0.054	TAOK2	0.068	~amyloid_beta+(1|manifest)+cngeneson+pc_mito+brain_region+Sex	1.781	2714.000
ENSG00000269743	0.007	0.120	0.223	0.016	SLC25A53	0.033	~amyloid_beta+(1|manifest)+cngeneson+pc_mito+brain_region+Sex	0.291	5745.000
ENSG00000158863	0.017	0.119	0.280	-0.041	[illegible]	0.054	~amyloid_beta+(1|manifest)+cngeneson+pc_mito+brain_region+Sex	1.945	2502.000
ENSG00000167107	0.020	0.119	0.311	-0.072	ACSF2	0.060	~amyloid_beta+(1|manifest)+cngeneson+pc_mito+brain_region+Sex	0.952	4247.000
ENSG00000196470	0.008	0.119	0.218	0.020	SIAH1	0.035	~amyloid_beta+(1|manifest)+cngeneson+pc_mito+brain_region+Sex	0.190	5887.000
ENSG00000103275	0.010	0.119	0.235	0.002	UBE2I	0.039	~amyloid_beta+(1|manifest)+cngeneson+pc_mito+brain_region+Sex	0.695	4907.000
ENSG00000036257	0.010	0.119	0.296	-0.058	CUL3	0.041	~amyloid_beta+(1|manifest)+cngeneson+pc_mito+brain_region+Sex	3.384	1327.000
ENSG00000119685	0.007	0.119	0.294	-0.057	TTLL5	0.033	~amyloid_beta+(1|manifest)+cngeneson+pc_mito+brain_region+Sex	1.713	2804.000
ENSG00000168297	0.001	0.118	0.257	-0.020	PXK	0.013	~amyloid_beta+(1|manifest)+cngeneson+pc_mito+brain_region+Sex	0.101	5952.000
ENSG00000122299	0.004	0.118	0.223	0.014	ZC3H7A	0.024	~amyloid_beta+(1|manifest)+cngeneson+pc_mito+brain_region+Sex	0.768	4695.000
ENSG00000164048	0.030	0.118	0.359	-0.122	ZNF589	0.074	~amyloid_beta+(1|manifest)+cngeneson+pc_mito+brain_region+Sex	2.036	2386.000
ENSG00000101439	0.756	0.118	0.507	-0.271	CST3	0.780	~amyloid_beta+(1|manifest)+cngeneson+pc_mito+brain_region+Sex	9.861	276.000
ENSG00000103197	0.164	0.118	0.431	-0.196	TSC2	0.219	~amyloid_beta+(1|manifest)+cngeneson+pc_mito+brain_region+Sex	7.290	442.000
ENSG00000122435	0.020	0.118	0.229	0.007	TRMT13	0.059	~amyloid_beta+(1|manifest)+cngeneson+pc_mito+brain_region+Sex	0.498	5365.000
ENSG00000206527	0.057	0.118	0.275	-0.039	HACD2	0.109	~amyloid_beta+(1|manifest)+cngeneson+pc_mito+brain_region+Sex	0.558	5218.000
ENSG00000214413	0.010	0.118	0.233	0.003	BBIP1	0.039	~amyloid_beta+(1|manifest)+cngeneson+pc_mito+brain_region+Sex	0.216	5851.000
ENSG00000106682	0.005	0.118	0.220	0.016	EIF4H	0.026	~amyloid_beta+(1|manifest)+cngeneson+pc_mito+brain_region+Sex	0.373	5611.000
ENSG00000116574	0.006	0.118	0.270	-0.035	RHOU	0.030	~amyloid_beta+(1|manifest)+cngeneson+pc_mito+brain_region+Sex	1.018	4100.000
ENSG00000114204	0.003	0.117	0.574	-0.339	SERPINI2	0.018	~amyloid_beta+(1|manifest)+cngeneson+pc_mito+brain_region+Sex	3.690	1167.000
ENSG00000111676	0.290	0.117	0.397	-0.162	ATN1	0.343	~amyloid_beta+(1|manifest)+cngeneson+pc_mito+brain_region+Sex	5.238	718.000
ENSG00000139437	0.091	0.117	0.292	-0.057	TCHP	0.146	~amyloid_beta+(1|manifest)+cngeneson+pc_mito+brain_region+Sex	1.748	2750.000
ENSG00000127527	0.193	0.117	0.341	-0.107	EPS15L1	0.247	~amyloid_beta+(1|manifest)+cngeneson+pc_mito+brain_region+Sex	3.722	1154.000
ENSG00000121440	0.383	0.117	0.302	-0.069	PDZRN3	0.433	~amyloid_beta+(1|manifest)+cngeneson+pc_mito+brain_region+Sex	3.479	1256.000
ENSG00000131409	0.198	0.117	0.429	-0.195	LRRC4B	0.252	~amyloid_beta+(1|manifest)+cngeneson+pc_mito+brain_region+Sex	2.641	1777.000
ENSG00000182263	0.653	0.117	0.390	-0.157	FIGN	0.685	~amyloid_beta+(1|manifest)+cngeneson+pc_mito+brain_region+Sex	5.669	653.000
ENSG00000157540	0.247	0.117	0.304	-0.071	DYRK1A	0.301	~amyloid_beta+(1|manifest)+cngeneson+pc_mito+brain_region+Sex	1.608	2971.000
ENSG00000058404	0.245	0.117	0.667	-0.433	CAMK2B	0.299	~amyloid_beta+(1|manifest)+cngeneson+pc_mito+brain_region+Sex	8.853	322.000
ENSG00000173914	0.017	0.117	0.230	0.003	RBM4B	0.053	~amyloid_beta+(1|manifest)+cngeneson+pc_mito+brain_region+Sex	1.243	3614.000
ENSG00000137154	0.073	0.116	0.368	-0.135	RPS6	0.127	~amyloid_beta+(1|manifest)+cngeneson+pc_mito+brain_region+Sex	3.415	1306.000
ENSG00000221926	0.000	0.116	0.195	0.038	TRIM16	0.005	~amyloid_beta+(1|manifest)+cngeneson+pc_mito+brain_region+Sex	0.675	4950.000
ENSG00000187091	0.056	0.116	0.329	-0.097	PLCD1	0.106	~amyloid_beta+(1|manifest)+cngeneson+pc_mito+brain_region+Sex	1.561	3038.000
ENSG00000068796	0.053	0.116	0.263	-0.031	KIF2A	0.103	~amyloid_beta+(1|manifest)+cngeneson+pc_mito+brain_region+Sex	1.196	3713.000
ENSG00000173166	0.150	0.116	0.329	-0.098	RAPH1	0.205	~amyloid_beta+(1|manifest)+cngeneson+pc_mito+brain_region+Sex	1.840	2643.000
ENSG00000066084	0.262	0.116	0.340	-0.109	DIP2B	0.316	~amyloid_beta+(1|manifest)+cngeneson+pc_mito+brain_region+Sex	3.525	1236.000
ENSG00000170836	0.006	0.116	0.245	-0.014	PPM1D	0.029	~amyloid_beta+(1|manifest)+cngeneson+pc_mito+brain_region+Sex	0.645	5015.000
ENSG00000158467	0.287	0.116	0.261	-0.030	AHCYL2	0.340	~amyloid_beta+(1|manifest)+cngeneson+pc_mito+brain_region+Sex	4.081	1019.000
ENSG00000116285	0.010	0.115	0.246	-0.015	ERRFI1	0.039	~amyloid_beta+(1|manifest)+cngeneson+pc_mito+brain_region+Sex	0.184	5895.000
ENSG00000115825	0.002	0.115	0.225	0.006	PRKD3	0.017	~amyloid_beta+(1|manifest)+cngeneson+pc_mito+brain_region+Sex	0.941	4277.000
ENSG00000130024	0.005	0.115	0.227	0.004	PHF10	0.027	~amyloid_beta+(1|manifest)+cngeneson+pc_mito+brain_region+Sex	0.282	5760.000
ENSG00000205476	0.032	0.115	0.341	-0.110	CCDC85C	0.077	~amyloid_beta+(1|manifest)+cngeneson+pc_mito+brain_region+Sex	1.647	2911.000
ENSG00000104946	0.096	0.115	0.360	-0.130	TBC1D17	0.152	~amyloid_beta+(1|manifest)+cngeneson+pc_mito+brain_region+Sex	3.292	1373.000
ENSG00000173875	0.002	0.115	0.200	0.029	ZNF791	0.017	~amyloid_beta+(1|manifest)+cngeneson+pc_mito+brain_region+Sex	0.784	4661.000
ENSG00000197111	0.141	0.115	0.312	-0.083	PCBP2	0.198	~amyloid_beta+(1|manifest)+cngeneson+pc_mito+brain_region+Sex	1.991	2445.000
ENSG00000108946	0.003	0.114	0.200	0.029	PRKAR1A	0.018	~amyloid_beta+(1|manifest)+cngeneson+pc_mito+brain_region+Sex	0.409	5549.000
ENSG00000134852	0.069	0.114	0.339	-0.111	CLOCK	0.122	~amyloid_beta+(1|manifest)+cngeneson+pc_mito+brain_region+Sex	2.582	1827.000
ENSG00000159692	0.011	0.114	0.221	0.008	CTBP1	0.042	~amyloid_beta+(1|manifest)+cngeneson+pc_mito+brain_region+Sex	1.204	3697.000
ENSG00000185158	0.045	0.114	0.321	-0.093	LRRC37B	0.093	~amyloid_beta+(1|manifest)+cngeneson+pc_mito+brain_region+Sex	0.739	4772.000
ENSG00000144401	0.021	0.114	0.285	-0.056	METTL21A	0.061	~amyloid_beta+(1|manifest)+cngeneson+pc_mito+brain_region+Sex	0.920	4330.000
ENSG00000162961	0.007	0.114	0.221	0.007	DPY30	0.031	~amyloid_beta+(1|manifest)+cngeneson+pc_mito+brain_region+Sex	0.062	5971.000
ENSG00000124299	0.002	0.114	0.187	0.041	PEPD	0.015	~amyloid_beta+(1|manifest)+cngeneson+pc_mito+brain_region+Sex	0.147	5927.000
ENSG00000145592	0.040	0.114	0.300	-0.072	RPL37	0.088	~amyloid_beta+(1|manifest)+cngeneson+pc_mito+brain_region+Sex	1.060	4005.000
ENSG00000102606	0.002	0.113	0.242	-0.015	ARHGEF7	0.015	~amyloid_beta+(1|manifest)+cngeneson+pc_mito+brain_region+Sex	0.676	4945.000
ENSG00000136367	0.029	0.113	0.250	-0.024	ZFHX2	0.073	~amyloid_beta+(1|manifest)+cngeneson+pc_mito+brain_region+Sex	1.110	3908.000
ENSG00000112079	0.045	0.113	0.276	-0.049	STK38	0.094	~amyloid_beta+(1|manifest)+cngeneson+pc_mito+brain_region+Sex	0.878	4422.000
ENSG00000185291	0.159	0.113	0.437	-0.211	IL3RA	0.214	~amyloid_beta+(1|manifest)+cngeneson+pc_mito+brain_region+Sex	5.050	762.000
ENSG00000136854	0.005	0.113	0.315	-0.089	STXBP1	0.026	~amyloid_beta+(1|manifest)+cngeneson+pc_mito+brain_region+Sex	0.660	4979.000
ENSG00000131504	0.003	0.113	0.244	-0.018	DIAPH1	0.019	~amyloid_beta+(1|manifest)+cngeneson+pc_mito+brain_region+Sex	0.897	4380.000
ENSG00000143891	0.005	0.113	0.207	0.019	GALM	0.027	~amyloid_beta+(1|manifest)+cngeneson+pc_mito+brain_region+Sex	0.611	5108.000
ENSG00000198791	0.001	0.113	0.205	0.020	CNOT7	0.014	~amyloid_beta+(1|manifest)+cngeneson+pc_mito+brain_region+Sex	0.251	5807.000
ENSG00000116604	0.012	0.113	0.211	0.014	MEF2D	0.043	~amyloid_beta+(1|manifest)+cngeneson+pc_mito+brain_region+Sex	0.992	4161.000
ENSG00000214290	0.024	0.113	0.324	-0.099	COLCA2	0.066	~amyloid_beta+(1|manifest)+cngeneson+pc_mito+brain_region+Sex	2.622	1796.000
ENSG00000054793	0.027	0.112	0.434	-0.210	ATP9A	0.069	~amyloid_beta+(1|manifest)+cngeneson+pc_mito+brain_region+Sex	2.567	1840.000
ENSG00000106608	0.000	0.112	0.179	0.045	URGCP	0.005	~amyloid_beta+(1|manifest)+cngeneson+pc_mito+brain_region+Sex	0.417	5531.000
ENSG00000130159	0.003	0.112	0.214	0.010	ECSIT	0.021	~amyloid_beta+(1|manifest)+cngeneson+pc_mito+brain_region+Sex	0.683	4931.000
ENSG00000173221	0.074	0.112	0.332	-0.108	GLRX	0.128	~amyloid_beta+(1|manifest)+cngeneson+pc_mito+brain_region+Sex	2.680	1745.000
ENSG00000114904	0.001	0.112	0.185	0.039	NEK4	0.012	~amyloid_beta+(1|manifest)+cngeneson+pc_mito+brain_region+Sex	0.207	5863.000
ENSG00000146243	0.044	0.112	0.376	-0.153	IRAK1BP1	0.093	~amyloid_beta+(1|manifest)+cngeneson+pc_mito+brain_region+Sex	1.456	3212.000
ENSG00000163719	0.003	0.112	0.193	0.030	MTMR14	0.019	~amyloid_beta+(1|manifest)+cngeneson+pc_mito+brain_region+Sex	0.743	4761.000
ENSG00000114978	0.002	0.112	0.189	0.034	MOB1A	0.016	~amyloid_beta+(1|manifest)+cngeneson+pc_mito+brain_region+Sex	0.095	5957.000
ENSG00000104853	0.083	0.111	0.327	-0.104	CLPTM1	0.138	~amyloid_beta+(1|manifest)+cngeneson+pc_mito+brain_region+Sex	2.145	2267.000
ENSG00000144567	0.046	0.111	0.274	-0.052	RETREG2	0.095	~amyloid_beta+(1|manifest)+cngeneson+pc_mito+brain_region+Sex	0.988	4169.000
ENSG00000167110	0.008	0.111	0.237	-0.014	GOLGA2	0.035	~amyloid_beta+(1|manifest)+cngeneson+pc_mito+brain_region+Sex	1.298	3509.000
ENSG00000047578	0.005	0.111	0.230	-0.008	KIAA0556	0.027	~amyloid_beta+(1|manifest)+cngeneson+pc_mito+brain_region+Sex	0.867	4450.000
ENSG00000152291	0.033	0.111	0.239	-0.017	TGOLN2	0.078	~amyloid_beta+(1|manifest)+cngeneson+pc_mito+brain_region+Sex	0.792	4651.000
ENSG00000115685	0.001	0.111	0.181	0.041	PPP1R7	0.008	~amyloid_beta+(1|manifest)+cngeneson+pc_mito+brain_region+Sex	0.683	4932.000
ENSG00000010810	0.147	0.111	0.275	-0.053	FYN	0.202	~amyloid_beta+(1|manifest)+cngeneson+pc_mito+brain_region+Sex	6.993	473.000
ENSG00000124214	0.015	0.111	0.297	-0.075	STAU1	0.050	~amyloid_beta+(1|manifest)+cngeneson+pc_mito+brain_region+Sex	1.924	2532.000
ENSG00000091490	0.051	0.111	0.520	-0.298	SEL1L3	0.101	~amyloid_beta+(1|manifest)+cngeneson+pc_mito+brain_region+Sex	3.966	1054.000
ENSG00000213585	0.010	0.111	0.250	-0.029	VDAC1	0.040	~amyloid_beta+(1|manifest)+cngeneson+pc_mito+brain_region+Sex	0.239	5825.000
ENSG00000154727	0.004	0.111	0.261	-0.039	GABPA	0.024	~amyloid_beta+(1|manifest)+cngeneson+pc_mito+brain_region+Sex	0.093	5959.000
ENSG00000196323	0.010	0.111	0.225	-0.003	ZBTB44	0.040	~amyloid_beta+(1|manifest)+cngeneson+pc_mito+brain_region+Sex	0.551	5229.000
ENSG00000144893	0.429	0.111	0.287	-0.065	MED12L	0.475	~amyloid_beta+(1|manifest)+cngeneson+pc_mito+brain_region+Sex	11.025	218.000
ENSG00000156313	0.010	0.111	0.353	-0.132	RPGR	0.040	~amyloid_beta+(1|manifest)+cngeneson+pc_mito+brain_region+Sex	0.988	4168.000
ENSG00000283154	0.658	0.111	0.353	-0.131	[illegible]	0.691	~amyloid_beta+(1|manifest)+cngeneson+pc_mito+brain_region+Sex	2.951	1573.000
ENSG00000178952	0.078	0.111	0.307	-0.086	TUFM	0.133	~amyloid_beta+(1|manifest)+cngeneson+pc_mito+brain_region+Sex	1.009	4121.000
ENSG00000125844	0.006	0.110	0.301	-0.080	RRBP1	0.030	~amyloid_beta+(1|manifest)+cngeneson+pc_mito+brain_region+Sex	1.026	4085.000
ENSG00000185896	0.059	0.110	0.346	-0.125	LAMP1	0.110	~amyloid_beta+(1|manifest)+cngeneson+pc_mito+brain_region+Sex	0.748	4744.000
ENSG00000175414	0.009	0.110	0.228	-0.008	ARL10	0.037	~amyloid_beta+(1|manifest)+cngeneson+pc_mito+brain_region+Sex	1.157	3796.000
ENSG00000152102	0.004	0.110	0.269	-0.049	FAM168B	0.022	~amyloid_beta+(1|manifest)+cngeneson+pc_mito+brain_region+Sex	0.458	5444.000
ENSG00000089876	0.006	0.110	0.309	-0.089	DHX32	0.030	~amyloid_beta+(1|manifest)+cngeneson+pc_mito+brain_region+Sex	2.084	2330.000
ENSG00000110395	0.019	0.110	0.254	-0.034	CBL	0.057	~amyloid_beta+(1|manifest)+cngeneson+pc_mito+brain_region+Sex	1.047	4033.000
ENSG00000114745	0.031	0.110	0.269	-0.049	GORASP1	0.076	~amyloid_beta+(1|manifest)+cngeneson+pc_mito+brain_region+Sex	1.460	3207.000
ENSG00000108370	0.047	0.110	0.434	-0.214	RGS9	0.096	~amyloid_beta+(1|manifest)+cngeneson+pc_mito+brain_region+Sex	2.507	1892.000
ENSG00000123240	0.001	0.110	0.250	-0.031	OPTN	0.014	~amyloid_beta+(1|manifest)+cngeneson+pc_mito+brain_region+Sex	0.908	4359.000
ENSG00000068400	0.006	0.110	0.219	0.000	GRIPAP1	0.029	~amyloid_beta+(1|manifest)+cngeneson+pc_mito+brain_region+Sex	1.252	3594.000
ENSG00000002586	0.026	0.110	0.351	-0.132	CD99	0.068	~amyloid_beta+(1|manifest)+cngeneson+pc_mito+brain_region+Sex	4.390	919.000
ENSG00000095787	0.020	0.110	0.237	-0.018	WAC	0.059	~amyloid_beta+(1|manifest)+cngeneson+pc_mito+brain_region+Sex	2.150	2262.000
ENSG00000124831	0.049	0.109	0.325	-0.107	LRRFIP1	0.098	~amyloid_beta+(1|manifest)+cngeneson+pc_mito+brain_region+Sex	2.193	2210.000
ENSG00000135535	0.034	0.109	0.237	-0.019	CD164	0.080	~amyloid_beta+(1|manifest)+cngeneson+pc_mito+brain_region+Sex	0.083	5965.000
ENSG00000143756	0.008	0.109	0.225	-0.006	FBXO28	0.034	~amyloid_beta+(1|manifest)+cngeneson+pc_mito+brain_region+Sex	0.770	4692.000
ENSG00000164111	0.048	0.109	0.269	-0.051	ANXA5	0.097	~amyloid_beta+(1|manifest)+cngeneson+pc_mito+brain_region+Sex	1.744	2757.000
ENSG00000144857	0.055	0.109	0.358	-0.140	BOC	0.105	~amyloid_beta+(1|manifest)+cngeneson+pc_mito+brain_region+Sex	0.212	5857.000
ENSG00000085377	0.067	0.109	0.301	-0.084	PREP	0.121	~amyloid_beta+(1|manifest)+cngeneson+pc_mito+brain_region+Sex	0.753	4735.000
ENSG00000126777	0.032	0.109	0.274	-0.057	KTN1	0.076	~amyloid_beta+(1|manifest)+cngeneson+pc_mito+brain_region+Sex	0.617	5096.000
ENSG00000068912	0.018	0.109	0.249	-0.032	ERLEC1	0.056	~amyloid_beta+(1|manifest)+cngeneson+pc_mito+brain_region+Sex	0.874	4427.000
ENSG00000186111	0.020	0.109	0.245	-0.028	PIP5K1C	0.060	~amyloid_beta+(1|manifest)+cngeneson+pc_mito+brain_region+Sex	0.916	4338.000
ENSG00000170852	0.001	0.108	0.179	0.038	KBTBD2	0.013	~amyloid_beta+(1|manifest)+cngeneson+pc_mito+brain_region+Sex	0.433	5501.000
ENSG00000081913	0.491	0.108	0.282	-0.065	PHLPP1	0.535	~amyloid_beta+(1|manifest)+cngeneson+pc_mito+brain_region+Sex	5.662	654.000
ENSG00000250722	0.025	0.108	0.344	-0.128	SELENOP	0.067	~amyloid_beta+(1|manifest)+cngeneson+pc_mito+brain_region+Sex	0.907	4361.000
ENSG00000089057	0.007	0.108	0.264	-0.048	SLC23A2	0.033	~amyloid_beta+(1|manifest)+cngeneson+pc_mito+brain_region+Sex	1.546	3058.000
ENSG00000146555	0.531	0.108	0.412	-0.195	SDK1	0.573	~amyloid_beta+(1|manifest)+cngeneson+pc_mito+brain_region+Sex	3.225	1414.000
ENSG00000122042	0.046	0.108	0.259	-0.043	UBL3	0.095	~amyloid_beta+(1|manifest)+cngeneson+pc_mito+brain_region+Sex	0.532	5280.000
ENSG00000196428	0.178	0.108	0.262	-0.046	TSC22D2	0.233	~amyloid_beta+(1|manifest)+cngeneson+pc_mito+brain_region+Sex	0.351	5651.000
ENSG00000112972	0.003	0.108	0.285	-0.069	HMGCS1	0.018	~amyloid_beta+(1|manifest)+cngeneson+pc_mito+brain_region+Sex	2.066	2351.000
ENSG00000127838	0.071	0.108	0.280	-0.064	PNKD	0.124	~amyloid_beta+(1|manifest)+cngeneson+pc_mito+brain_region+Sex	2.037	2384.000
ENSG00000243156	0.066	0.107	0.374	-0.160	MICAL3	0.120	~amyloid_beta+(1|manifest)+cngeneson+pc_mito+brain_region+Sex	2.321	2080.000
ENSG00000089159	0.014	0.107	0.247	-0.033	PXN	0.049	~amyloid_beta+(1|manifest)+cngeneson+pc_mito+brain_region+Sex	1.294	3516.000
ENSG00000119688	0.087	0.107	0.384	-0.171	ABCD4	0.142	~amyloid_beta+(1|manifest)+cngeneson+pc_mito+brain_region+Sex	3.048	1511.000
ENSG00000129657	0.451	0.107	0.421	-0.208	SEC14L1	0.496	~amyloid_beta+(1|manifest)+cngeneson+pc_mito+brain_region+Sex	4.605	862.000
ENSG00000185651	0.011	0.107	0.206	0.007	UBE2L3	0.043	~amyloid_beta+(1|manifest)+cngeneson+pc_mito+brain_region+Sex	0.639	5032.000
ENSG00000100154	0.046	0.107	0.187	0.026	TTC28	0.095	~amyloid_beta+(1|manifest)+cngeneson+pc_mito+brain_region+Sex	2.465	1927.000
ENSG00000115459	0.002	0.107	0.194	0.020	ELMOD3	0.017	~amyloid_beta+(1|manifest)+cngeneson+pc_mito+brain_region+Sex	1.169	3766.000
ENSG00000100027	0.022	0.107	0.299	-0.086	YPEL1	0.062	~amyloid_beta+(1|manifest)+cngeneson+pc_mito+brain_region+Sex	1.387	3345.000
ENSG00000171311	0.002	0.107	0.205	0.008	EXOSC1	0.018	~amyloid_beta+(1|manifest)+cngeneson+pc_mito+brain_region+Sex	1.088	3952.000
ENSG00000101337	0.006	0.106	0.230	-0.018	TM9SF4	0.029	~amyloid_beta+(1|manifest)+cngeneson+pc_mito+brain_region+Sex	1.375	3368.000
ENSG00000167081	0.420	0.106	0.323	-0.111	PBX3	0.467	~amyloid_beta+(1|manifest)+cngeneson+pc_mito+brain_region+Sex	0.754	4730.000
ENSG00000142611	0.328	0.106	0.258	-0.046	PRDM16	0.380	~amyloid_beta+(1|manifest)+cngeneson+pc_mito+brain_region+Sex	4.189	990.000
ENSG00000175203	0.008	0.106	0.195	0.017	DCTN2	0.035	~amyloid_beta+(1|manifest)+cngeneson+pc_mito+brain_region+Sex	0.291	5744.000
ENSG00000075142	0.122	0.106	0.372	-0.160	SRI	0.178	~amyloid_beta+(1|manifest)+cngeneson+pc_mito+brain_region+Sex	0.897	4379.000
ENSG00000090905	0.178	0.106	0.245	-0.034	TNRC6A	0.233	~amyloid_beta+(1|manifest)+cngeneson+pc_mito+brain_region+Sex	9.190	311.000
ENSG00000113269	0.058	0.105	0.279	-0.068	RNF130	0.109	~amyloid_beta+(1|manifest)+cngeneson+pc_mito+brain_region+Sex	1.356	3400.000
ENSG00000143443	0.150	0.105	0.351	-0.141	C1orf56	0.205	~amyloid_beta+(1|manifest)+cngeneson+pc_mito+brain_region+Sex	2.488	1911.000
ENSG00000181090	0.193	0.105	0.335	-0.125	EHMT1	0.248	~amyloid_beta+(1|manifest)+cngeneson+pc_mito+brain_region+Sex	4.986	779.000
ENSG00000182732	0.568	0.105	0.522	-0.312	RGS6	0.607	~amyloid_beta+(1|manifest)+cngeneson+pc_mito+brain_region+Sex	6.208	563.000
ENSG00000152952	0.619	0.105	0.423	-0.214	PLOD2	0.655	~amyloid_beta+(1|manifest)+cngeneson+pc_mito+brain_region+Sex	0.697	4899.000
ENSG00000213923	0.103	0.105	0.334	-0.124	CSNK1E	0.159	~amyloid_beta+(1|manifest)+cngeneson+pc_mito+brain_region+Sex	1.774	2722.000
ENSG00000198604	0.074	0.105	0.319	-0.110	BAZ1A	0.128	~amyloid_beta+(1|manifest)+cngeneson+pc_mito+brain_region+Sex	2.332	2068.000
ENSG00000134871	0.635	0.105	0.396	-0.186	COL4A2	0.669	~amyloid_beta+(1|manifest)+cngeneson+pc_mito+brain_region+Sex	1.653	2898.000
ENSG00000110497	0.015	0.105	0.308	-0.099	AMBRA1	0.050	~amyloid_beta+(1|manifest)+cngeneson+pc_mito+brain_region+Sex	1.709	2811.000
ENSG00000187123	0.284	0.104	0.452	-0.243	LYPD6	0.337	~amyloid_beta+(1|manifest)+cngeneson+pc_mito+brain_region+Sex	4.139	1002.000
ENSG00000058272	0.086	0.104	0.231	-0.022	PPP1R12A	0.141	~amyloid_beta+(1|manifest)+cngeneson+pc_mito+brain_region+Sex	2.956	1566.000
ENSG00000171456	0.034	0.104	0.292	-0.084	ASXL1	0.079	~amyloid_beta+(1|manifest)+cngeneson+pc_mito+brain_region+Sex	1.498	3140.000
ENSG00000178982	0.016	0.104	0.244	-0.036	EIF3K	0.053	~amyloid_beta+(1|manifest)+cngeneson+pc_mito+brain_region+Sex	1.182	3741.000
ENSG00000106771	0.006	0.104	0.205	0.003	TMEM245	0.030	~amyloid_beta+(1|manifest)+cngeneson+pc_mito+brain_region+Sex	0.708	4853.000
ENSG00000164603	0.062	0.104	0.350	-0.142	BMT2	0.115	~amyloid_beta+(1|manifest)+cngeneson+pc_mito+brain_region+Sex	0.960	4225.000
ENSG00000160948	0.145	0.104	0.328	-0.120	VPS28	0.201	~amyloid_beta+(1|manifest)+cngeneson+pc_mito+brain_region+Sex	2.130	2280.000
ENSG00000109911	0.466	0.104	0.366	-0.159	ELP4	0.511	~amyloid_beta+(1|manifest)+cngeneson+pc_mito+brain_region+Sex	2.851	1635.000
ENSG00000171530	0.017	0.103	0.304	-0.097	TBCA	0.054	~amyloid_beta+(1|manifest)+cngeneson+pc_mito+brain_region+Sex	0.722	4817.000
ENSG00000197771	0.000	0.103	0.155	0.052	MCMBP	0.004	~amyloid_beta+(1|manifest)+cngeneson+pc_mito+brain_region+Sex	0.413	5538.000
ENSG00000144746	0.063	0.103	0.315	-0.109	ARL6IP5	0.115	~amyloid_beta+(1|manifest)+cngeneson+pc_mito+brain_region+Sex	1.525	3095.000
ENSG00000168137	0.053	0.103	0.207	0.000	SETD5	0.103	~amyloid_beta+(1|manifest)+cngeneson+pc_mito+brain_region+Sex	1.413	3302.000
ENSG00000168090	0.041	0.103	0.259	-0.053	COPS6	0.088	~amyloid_beta+(1|manifest)+cngeneson+pc_mito+brain_region+Sex	1.402	3316.000
ENSG00000166562	0.007	0.103	0.437	-0.231	SEC11C	0.031	~amyloid_beta+(1|manifest)+cngeneson+pc_mito+brain_region+Sex	1.812	2679.000
ENSG00000144791	0.034	0.103	0.230	-0.024	LIMD1	0.080	~amyloid_beta+(1|manifest)+cngeneson+pc_mito+brain_region+Sex	0.444	5469.000
ENSG00000196914	0.199	0.103	0.213	-0.007	ARHGEF12	0.253	~amyloid_beta+(1|manifest)+cngeneson+pc_mito+brain_region+Sex	2.096	2319.000
ENSG00000155324	0.234	0.103	0.227	-0.022	GRAMD2B	0.288	~amyloid_beta+(1|manifest)+cngeneson+pc_mito+brain_region+Sex	5.546	672.000
ENSG00000079974	0.025	0.103	0.231	-0.026	RABL2B	0.067	~amyloid_beta+(1|manifest)+cngeneson+pc_mito+brain_region+Sex	1.360	3390.000
ENSG00000178974	0.007	0.103	0.212	-0.006	FBXO34	0.031	~amyloid_beta+(1|manifest)+cngeneson+pc_mito+brain_region+Sex	0.322	5689.000
ENSG00000150907	0.408	0.102	0.246	-0.042	FOXO1	0.455	~amyloid_beta+(1|manifest)+cngeneson+pc_mito+brain_region+Sex	1.077	3970.000
ENSG00000174574	0.021	0.102	0.223	-0.019	AKIRIN1	0.061	~amyloid_beta+(1|manifest)+cngeneson+pc_mito+brain_region+Sex	1.230	3640.000
ENSG00000160299	0.013	0.102	0.265	-0.060	PCNT	0.047	~amyloid_beta+(1|manifest)+cngeneson+pc_mito+brain_region+Sex	1.169	3769.000
ENSG00000166016	0.213	0.102	0.243	-0.039	ABTB2	0.268	~amyloid_beta+(1|manifest)+cngeneson+pc_mito+brain_region+Sex	1.144	3826.000
ENSG00000120742	0.201	0.102	0.335	-0.131	SERP1	0.256	~amyloid_beta+(1|manifest)+cngeneson+pc_mito+brain_region+Sex	1.334	3441.000
ENSG00000166313	0.028	0.102	0.224	-0.020	APBB1	0.072	~amyloid_beta+(1|manifest)+cngeneson+pc_mito+brain_region+Sex	0.850	4501.000
ENSG00000181322	0.221	0.102	0.322	-0.119	NME9	0.276	~amyloid_beta+(1|manifest)+cngeneson+pc_mito+brain_region+Sex	2.049	2370.000
ENSG00000153885	0.124	0.102	0.419	-0.215	KCTD15	0.180	~amyloid_beta+(1|manifest)+cngeneson+pc_mito+brain_region+Sex	5.962	596.000
ENSG00000264522	0.451	0.102	0.480	-0.277	OTUD7B	0.496	~amyloid_beta+(1|manifest)+cngeneson+pc_mito+brain_region+Sex	3.347	1341.000
ENSG00000179833	0.020	0.101	0.271	-0.069	SERTAD2	0.058	~amyloid_beta+(1|manifest)+cngeneson+pc_mito+brain_region+Sex	4.249	963.000
ENSG00000204272	0.141	0.101	0.373	-0.171	NBDY	0.198	~amyloid_beta+(1|manifest)+cngeneson+pc_mito+brain_region+Sex	0.485	5394.000
ENSG00000106853	0.000	0.101	0.205	-0.002	PTGR1	0.006	~amyloid_beta+(1|manifest)+cngeneson+pc_mito+brain_region+Sex	0.289	5746.000
ENSG00000079950	0.023	0.101	0.219	-0.017	STX7	0.064	~amyloid_beta+(1|manifest)+cngeneson+pc_mito+brain_region+Sex	0.448	5460.000
ENSG00000105357	0.016	0.101	0.261	-0.059	MYH14	0.052	~amyloid_beta+(1|manifest)+cngeneson+pc_mito+brain_region+Sex	0.906	4364.000
ENSG00000088833	0.003	0.101	0.176	0.025	NSFL1C	0.018	~amyloid_beta+(1|manifest)+cngeneson+pc_mito+brain_region+Sex	0.793	4646.000
ENSG00000068724	0.014	0.101	0.265	-0.063	TTC7A	0.048	~amyloid_beta+(1|manifest)+cngeneson+pc_mito+brain_region+Sex	1.765	2729.000
ENSG00000163166	0.012	0.101	0.257	-0.055	IWS1	0.043	~amyloid_beta+(1|manifest)+cngeneson+pc_mito+brain_region+Sex	1.455	3215.000
ENSG00000145675	0.519	0.101	0.468	-0.267	PIK3R1	0.562	~amyloid_beta+(1|manifest)+cngeneson+pc_mito+brain_region+Sex	2.429	1958.000
ENSG00000153814	0.268	0.101	0.383	-0.181	JAZF1	0.322	~amyloid_beta+(1|manifest)+cngeneson+pc_mito+brain_region+Sex	1.630	2938.000
ENSG00000003056	0.015	0.101	0.200	0.001	M6PR	0.049	~amyloid_beta+(1|manifest)+cngeneson+pc_mito+brain_region+Sex	0.574	5175.000
ENSG00000147140	0.013	0.101	0.255	-0.054	NONO	0.046	~amyloid_beta+(1|manifest)+cngeneson+pc_mito+brain_region+Sex	0.698	4895.000
ENSG00000175390	0.001	0.100	0.174	0.027	EIF3F	0.011	~amyloid_beta+(1|manifest)+cngeneson+pc_mito+brain_region+Sex	0.173	5905.000
ENSG00000164615	0.004	0.100	0.191	0.009	CAMLG	0.023	~amyloid_beta+(1|manifest)+cngeneson+pc_mito+brain_region+Sex	0.697	4902.000
ENSG00000268043	0.081	0.100	0.335	-0.135	NBPF12	0.136	~amyloid_beta+(1|manifest)+cngeneson+pc_mito+brain_region+Sex	2.401	1981.000
ENSG00000166913	0.002	0.100	0.180	0.020	YWHAB	0.017	~amyloid_beta+(1|manifest)+cngeneson+pc_mito+brain_region+Sex	0.503	5350.000
ENSG00000183735	0.016	0.100	0.226	-0.026	TBK1	0.052	~amyloid_beta+(1|manifest)+cngeneson+pc_mito+brain_region+Sex	1.229	3649.000
ENSG00000132128	0.013	0.100	0.196	0.003	LRRC41	0.047	~amyloid_beta+(1|manifest)+cngeneson+pc_mito+brain_region+Sex	0.481	5400.000
ENSG00000197937	0.000	0.099	0.171	0.028	ZNF347	0.005	~amyloid_beta+(1|manifest)+cngeneson+pc_mito+brain_region+Sex	0.741	4766.000
ENSG00000175104	0.004	0.099	0.268	-0.069	TRAF6	0.022	~amyloid_beta+(1|manifest)+cngeneson+pc_mito+brain_region+Sex	1.233	3629.000
ENSG00000132463	0.009	0.099	0.193	0.005	GRSF1	0.038	~amyloid_beta+(1|manifest)+cngeneson+pc_mito+brain_region+Sex	0.134	5931.000
ENSG00000068878	0.148	0.099	0.304	-0.105	PSME4	0.204	~amyloid_beta+(1|manifest)+cngeneson+pc_mito+brain_region+Sex	2.071	2344.000
ENSG00000171603	0.096	0.099	0.289	-0.090	CLSTN1	0.152	~amyloid_beta+(1|manifest)+cngeneson+pc_mito+brain_region+Sex	0.462	5437.000
ENSG00000163634	0.008	0.099	0.206	-0.007	THOC7	0.034	~amyloid_beta+(1|manifest)+cngeneson+pc_mito+brain_region+Sex	0.159	5913.000
ENSG00000165609	0.008	0.099	0.194	0.004	NUDT5	0.034	~amyloid_beta+(1|manifest)+cngeneson+pc_mito+brain_region+Sex	0.600	5128.000
ENSG00000153993	0.153	0.099	0.557	-0.359	SEMA3D	0.208	~amyloid_beta+(1|manifest)+cngeneson+pc_mito+brain_region+Sex	5.038	765.000
ENSG00000139168	0.011	0.099	0.195	0.003	ZCRB1	0.042	~amyloid_beta+(1|manifest)+cngeneson+pc_mito+brain_region+Sex	0.397	5566.000
ENSG00000091409	0.113	0.099	0.352	-0.154	ITGA6	0.169	~amyloid_beta+(1|manifest)+cngeneson+pc_mito+brain_region+Sex	5.562	669.000
ENSG00000213398	0.209	0.098	0.366	-0.169	LCAT	0.264	~amyloid_beta+(1|manifest)+cngeneson+pc_mito+brain_region+Sex	2.846	1640.000
ENSG00000171914	0.295	0.098	0.421	-0.224	TLN2	0.347	~amyloid_beta+(1|manifest)+cngeneson+pc_mito+brain_region+Sex	3.699	1162.000
ENSG00000180537	0.284	0.098	0.390	-0.194	RNF182	0.337	~amyloid_beta+(1|manifest)+cngeneson+pc_mito+brain_region+Sex	3.858	1102.000
ENSG00000244754	0.133	0.098	0.191	0.005	N4BP2L2	0.190	~amyloid_beta+(1|manifest)+cngeneson+pc_mito+brain_region+Sex	1.072	3986.000
ENSG00000164040	0.003	0.098	0.175	0.021	PGRMC2	0.019	~amyloid_beta+(1|manifest)+cngeneson+pc_mito+brain_region+Sex	0.329	5675.000
ENSG00000075240	0.072	0.098	0.327	-0.131	GRAMD4	0.126	~amyloid_beta+(1|manifest)+cngeneson+pc_mito+brain_region+Sex	2.375	2025.000
ENSG00000109472	0.298	0.098	0.243	-0.047	CPE	0.351	~amyloid_beta+(1|manifest)+cngeneson+pc_mito+brain_region+Sex	6.736	502.000
ENSG00000142303	0.035	0.098	0.400	-0.204	[illegible]	0.081	~amyloid_beta+(1|manifest)+cngeneson+pc_mito+brain_region+Sex	3.915	1083.000
ENSG00000116793	0.003	0.098	0.179	0.017	PHTF1	0.021	~amyloid_beta+(1|manifest)+cngeneson+pc_mito+brain_region+Sex	0.994	4153.000
ENSG00000105576	0.126	0.098	0.295	-0.099	TNPO2	0.182	~amyloid_beta+(1|manifest)+cngeneson+pc_mito+brain_region+Sex	2.208	2195.000
ENSG00000150995	0.015	0.098	0.223	-0.027	ITPR1	0.051	~amyloid_beta+(1|manifest)+cngeneson+pc_mito+brain_region+Sex	1.627	2942.000
ENSG00000179134	0.053	0.098	0.232	-0.037	SAMD4B	0.103	~amyloid_beta+(1|manifest)+cngeneson+pc_mito+brain_region+Sex	1.312	3477.000
ENSG00000152234	0.046	0.098	0.245	-0.050	ATP5F1A	0.095	~amyloid_beta+(1|manifest)+cngeneson+pc_mito+brain_region+Sex	0.806	4615.000
ENSG00000109381	0.193	0.098	0.289	-0.094	ELF2	0.247	~amyloid_beta+(1|manifest)+cngeneson+pc_mito+brain_region+Sex	3.372	1334.000
ENSG00000165233	0.036	0.097	0.289	-0.094	CARD19	0.082	~amyloid_beta+(1|manifest)+cngeneson+pc_mito+brain_region+Sex	1.304	3500.000
ENSG00000157637	0.082	0.097	0.276	-0.082	SLC38A10	0.137	~amyloid_beta+(1|manifest)+cngeneson+pc_mito+brain_region+Sex	2.128	2282.000
ENSG00000070614	0.035	0.097	0.261	-0.066	NDST1	0.081	~amyloid_beta+(1|manifest)+cngeneson+pc_mito+brain_region+Sex	1.428	3268.000
ENSG00000155099	0.005	0.097	0.262	-0.068	PIP4P2	0.026	~amyloid_beta+(1|manifest)+cngeneson+pc_mito+brain_region+Sex	1.292	3522.000
ENSG00000135414	0.085	0.097	0.312	-0.119	GDF11	0.140	~amyloid_beta+(1|manifest)+cngeneson+pc_mito+brain_region+Sex	1.651	2905.000
ENSG00000134108	0.022	0.097	0.267	-0.073	ARL8B	0.062	~amyloid_beta+(1|manifest)+cngeneson+pc_mito+brain_region+Sex	1.029	4081.000
ENSG00000102125	0.066	0.097	0.309	-0.115	TAZ	0.119	~amyloid_beta+(1|manifest)+cngeneson+pc_mito+brain_region+Sex	3.098	1483.000
ENSG00000084234	0.015	0.097	0.218	-0.024	APLP2	0.050	~amyloid_beta+(1|manifest)+cngeneson+pc_mito+brain_region+Sex	0.588	5157.000
ENSG00000173068	0.104	0.097	0.315	-0.122	BNC2	0.160	~amyloid_beta+(1|manifest)+cngeneson+pc_mito+brain_region+Sex	2.366	2032.000
ENSG00000084636	0.396	0.097	0.604	-0.410	COL16A1	0.444	~amyloid_beta+(1|manifest)+cngeneson+pc_mito+brain_region+Sex	10.145	263.000
ENSG00000170027	0.006	0.097	0.214	-0.021	YWHAG	0.030	~amyloid_beta+(1|manifest)+cngeneson+pc_mito+brain_region+Sex	0.239	5824.000
ENSG00000197360	0.920	0.097	1.100	-0.907	ZNF98	0.929	~amyloid_beta+(1|manifest)+cngeneson+pc_mito+brain_region+Sex	15.202	100.000
ENSG00000142002	0.029	0.097	0.237	-0.044	DPP9	0.073	~amyloid_beta+(1|manifest)+cngeneson+pc_mito+brain_region+Sex	1.588	3003.000
ENSG00000166535	0.109	0.096	0.428	-0.236	A2ML1	0.165	~amyloid_beta+(1|manifest)+cngeneson+pc_mito+brain_region+Sex	2.570	1838.000
ENSG00000157796	0.009	0.096	0.209	-0.017	WDR19	0.037	~amyloid_beta+(1|manifest)+cngeneson+pc_mito+brain_region+Sex	0.342	5662.000
ENSG00000102738	0.028	0.096	0.248	-0.056	MRPS31	0.072	~amyloid_beta+(1|manifest)+cngeneson+pc_mito+brain_region+Sex	0.460	5440.000
ENSG00000198369	0.387	0.096	0.403	-0.212	SPRED2	0.436	~amyloid_beta+(1|manifest)+cngeneson+pc_mito+brain_region+Sex	1.076	3975.000
ENSG00000125386	0.022	0.096	0.314	-0.122	FAM193A	0.063	~amyloid_beta+(1|manifest)+cngeneson+pc_mito+brain_region+Sex	1.904	2548.000
ENSG00000164924	0.030	0.096	0.255	-0.064	YWHAZ	0.074	~amyloid_beta+(1|manifest)+cngeneson+pc_mito+brain_region+Sex	0.765	4706.000
ENSG00000204624	0.013	0.096	0.241	-0.050	DISP3	0.047	~amyloid_beta+(1|manifest)+cngeneson+pc_mito+brain_region+Sex	1.376	3365.000
ENSG00000158321	0.108	0.095	0.215	-0.024	AUTS2	0.164	~amyloid_beta+(1|manifest)+cngeneson+pc_mito+brain_region+Sex	4.211	977.000
ENSG00000139154	0.228	0.095	0.349	-0.159	AEBP2	0.282	~amyloid_beta+(1|manifest)+cngeneson+pc_mito+brain_region+Sex	3.124	1468.000
ENSG00000074266	0.028	0.095	0.217	-0.026	EED	0.071	~amyloid_beta+(1|manifest)+cngeneson+pc_mito+brain_region+Sex	0.700	4889.000
ENSG00000167566	0.067	0.095	0.260	-0.070	NCKAP5L	0.120	~amyloid_beta+(1|manifest)+cngeneson+pc_mito+brain_region+Sex	1.800	2696.000
ENSG00000174282	0.069	0.095	0.281	-0.091	ZBTB4	0.123	~amyloid_beta+(1|manifest)+cngeneson+pc_mito+brain_region+Sex	2.614	1804.000
ENSG00000184381	0.134	0.095	0.318	-0.128	PLA2G6	0.190	~amyloid_beta+(1|manifest)+cngeneson+pc_mito+brain_region+Sex	3.064	1500.000
ENSG00000204842	0.126	0.095	0.185	0.005	ATXN2	0.182	~amyloid_beta+(1|manifest)+cngeneson+pc_mito+brain_region+Sex	1.105	3913.000
ENSG00000162521	0.007	0.095	0.185	0.004	RBBP4	0.031	~amyloid_beta+(1|manifest)+cngeneson+pc_mito+brain_region+Sex	0.159	5912.000
ENSG00000183087	0.119	0.095	0.340	-0.150	GAS6	0.175	~amyloid_beta+(1|manifest)+cngeneson+pc_mito+brain_region+Sex	1.901	2552.000
ENSG00000109956	0.047	0.094	0.247	-0.058	B3GAT1	0.095	~amyloid_beta+(1|manifest)+cngeneson+pc_mito+brain_region+Sex	0.826	4549.000
ENSG00000138621	0.088	0.094	0.341	-0.152	PPCDC	0.143	~amyloid_beta+(1|manifest)+cngeneson+pc_mito+brain_region+Sex	1.620	2952.000
ENSG00000148450	0.003	0.094	0.163	0.026	MSRB2	0.022	~amyloid_beta+(1|manifest)+cngeneson+pc_mito+brain_region+Sex	0.560	5211.000
ENSG00000140391	0.105	0.094	0.315	-0.127	TSPAN3	0.161	~amyloid_beta+(1|manifest)+cngeneson+pc_mito+brain_region+Sex	1.756	2742.000
ENSG00000131069	0.002	0.094	0.216	-0.028	ACSS2	0.017	~amyloid_beta+(1|manifest)+cngeneson+pc_mito+brain_region+Sex	0.431	5506.000
ENSG00000089351	0.048	0.094	0.274	-0.086	GRAMD1A	0.097	~amyloid_beta+(1|manifest)+cngeneson+pc_mito+brain_region+Sex	2.605	1809.000
ENSG00000167114	0.004	0.094	0.186	0.001	SLC27A4	0.025	~amyloid_beta+(1|manifest)+cngeneson+pc_mito+brain_region+Sex	0.253	5805.000
ENSG00000107263	0.039	0.094	0.305	-0.117	RAPGEF1	0.086	~amyloid_beta+(1|manifest)+cngeneson+pc_mito+brain_region+Sex	1.926	2530.000
ENSG00000125954	0.032	0.094	0.243	-0.056	[illegible]	0.076	~amyloid_beta+(1|manifest)+cngeneson+pc_mito+brain_region+Sex	1.533	3079.000
ENSG00000177888	0.007	0.094	0.227	-0.040	ZBTB41	0.031	~amyloid_beta+(1|manifest)+cngeneson+pc_mito+brain_region+Sex	0.426	5515.000
ENSG00000149179	0.239	0.094	0.247	-0.060	C11orf49	0.292	~amyloid_beta+(1|manifest)+cngeneson+pc_mito+brain_region+Sex	0.682	4934.000
ENSG00000127022	0.125	0.093	0.321	-0.134	CANX	0.182	~amyloid_beta+(1|manifest)+cngeneson+pc_mito+brain_region+Sex	1.031	4073.000
ENSG00000091317	0.009	0.093	0.227	-0.040	CMTM6	0.038	~amyloid_beta+(1|manifest)+cngeneson+pc_mito+brain_region+Sex	0.117	5945.000
ENSG00000062485	0.037	0.093	0.248	-0.061	CS	0.084	~amyloid_beta+(1|manifest)+cngeneson+pc_mito+brain_region+Sex	1.225	3656.000
ENSG00000185250	0.019	0.093	0.203	-0.016	PPIL6	0.057	~amyloid_beta+(1|manifest)+cngeneson+pc_mito+brain_region+Sex	0.092	5960.000
ENSG00000151332	0.021	0.093	0.183	0.004	MBIP	0.061	~amyloid_beta+(1|manifest)+cngeneson+pc_mito+brain_region+Sex	0.062	5972.000
ENSG00000236287	0.008	0.093	0.288	-0.102	ZBED5	0.035	~amyloid_beta+(1|manifest)+cngeneson+pc_mito+brain_region+Sex	1.168	3771.000
ENSG00000141367	0.009	0.093	0.196	-0.010	CLTC	0.038	~amyloid_beta+(1|manifest)+cngeneson+pc_mito+brain_region+Sex	0.973	4199.000
ENSG00000140395	0.013	0.093	0.208	-0.022	WDR61	0.047	~amyloid_beta+(1|manifest)+cngeneson+pc_mito+brain_region+Sex	0.980	4187.000
ENSG00000142453	0.011	0.093	0.291	-0.105	CARM1	0.042	~amyloid_beta+(1|manifest)+cngeneson+pc_mito+brain_region+Sex	1.744	2759.000
ENSG00000122705	0.010	0.093	0.192	-0.007	CLTA	0.039	~amyloid_beta+(1|manifest)+cngeneson+pc_mito+brain_region+Sex	0.767	4700.000
ENSG00000137198	0.187	0.093	0.636	-0.451	GMPR	0.242	~amyloid_beta+(1|manifest)+cngeneson+pc_mito+brain_region+Sex	5.152	738.000
ENSG00000099954	0.381	0.093	0.390	-0.205	CECR2	0.431	~amyloid_beta+(1|manifest)+cngeneson+pc_mito+brain_region+Sex	1.093	3943.000
ENSG00000109452	0.534	0.092	0.501	-0.316	INPP4B	0.575	~amyloid_beta+(1|manifest)+cngeneson+pc_mito+brain_region+Sex	0.819	4574.000
ENSG00000116016	0.050	0.092	0.339	-0.154	EPAS1	0.100	~amyloid_beta+(1|manifest)+cngeneson+pc_mito+brain_region+Sex	2.350	2047.000
ENSG00000122873	0.006	0.092	0.199	-0.015	CISD1	0.029	~amyloid_beta+(1|manifest)+cngeneson+pc_mito+brain_region+Sex	0.255	5799.000
ENSG00000105323	0.006	0.092	0.163	0.021	HNRNPUL1	0.029	~amyloid_beta+(1|manifest)+cngeneson+pc_mito+brain_region+Sex	0.534	5272.000
ENSG00000135838	0.578	0.092	0.457	-0.273	NPL	0.615	~amyloid_beta+(1|manifest)+cngeneson+pc_mito+brain_region+Sex	9.638	286.000
ENSG00000101972	0.044	0.092	0.260	-0.075	STAG2	0.093	~amyloid_beta+(1|manifest)+cngeneson+pc_mito+brain_region+Sex	0.856	4480.000
ENSG00000068383	0.013	0.092	0.229	-0.045	INPP5A	0.047	~amyloid_beta+(1|manifest)+cngeneson+pc_mito+brain_region+Sex	0.288	5747.000
ENSG00000106714	0.270	0.092	0.554	-0.370	CNTNAP3	0.324	~amyloid_beta+(1|manifest)+cngeneson+pc_mito+brain_region+Sex	7.682	402.000
ENSG00000165806	0.048	0.092	0.359	-0.176	CASP7	0.097	~amyloid_beta+(1|manifest)+cngeneson+pc_mito+brain_region+Sex	1.094	3941.000
ENSG00000154473	0.021	0.092	0.193	-0.009	BUB3	0.061	~amyloid_beta+(1|manifest)+cngeneson+pc_mito+brain_region+Sex	0.637	5038.000
ENSG00000221968	0.380	0.092	0.535	-0.352	FADS3	0.429	~amyloid_beta+(1|manifest)+cngeneson+pc_mito+brain_region+Sex	5.846	615.000
ENSG00000163389	0.046	0.092	0.201	-0.018	POGLUT1	0.095	~amyloid_beta+(1|manifest)+cngeneson+pc_mito+brain_region+Sex	0.617	5095.000
ENSG00000001629	0.067	0.091	0.247	-0.064	ANKIB1	0.120	~amyloid_beta+(1|manifest)+cngeneson+pc_mito+brain_region+Sex	0.891	4394.000
ENSG00000146676	0.047	0.091	0.231	-0.049	PURB	0.096	~amyloid_beta+(1|manifest)+cngeneson+pc_mito+brain_region+Sex	0.739	4774.000
ENSG00000128585	0.086	0.091	0.194	-0.011	MKLN1	0.141	~amyloid_beta+(1|manifest)+cngeneson+pc_mito+brain_region+Sex	2.308	2094.000
ENSG00000130787	0.712	0.091	0.717	-0.534	HIP1R	0.740	~amyloid_beta+(1|manifest)+cngeneson+pc_mito+brain_region+Sex	18.426	63.000
ENSG00000140400	0.134	0.091	0.338	-0.156	MAN2C1	0.191	~amyloid_beta+(1|manifest)+cngeneson+pc_mito+brain_region+Sex	4.065	1024.000
ENSG00000114302	0.026	0.091	0.340	-0.158	PRKAR2A	0.068	~amyloid_beta+(1|manifest)+cngeneson+pc_mito+brain_region+Sex	1.143	3830.000
ENSG00000266412	0.004	0.091	0.161	0.021	NCOA4	0.024	~amyloid_beta+(1|manifest)+cngeneson+pc_mito+brain_region+Sex	0.271	5781.000
ENSG00000008256	0.001	0.091	0.155	0.027	CYTH3	0.010	~amyloid_beta+(1|manifest)+cngeneson+pc_mito+brain_region+Sex	0.515	5316.000
ENSG00000182446	0.013	0.091	0.220	-0.038	NPLOC4	0.047	~amyloid_beta+(1|manifest)+cngeneson+pc_mito+brain_region+Sex	1.042	4045.000
ENSG00000121579	0.012	0.091	0.241	-0.059	NAA50	0.043	~amyloid_beta+(1|manifest)+cngeneson+pc_mito+brain_region+Sex	0.720	4823.000
ENSG00000171453	0.028	0.091	0.181	0.001	POLR1C	0.071	~amyloid_beta+(1|manifest)+cngeneson+pc_mito+brain_region+Sex	0.493	5379.000
ENSG00000144908	0.445	0.091	0.305	-0.123	ALDH1L1	0.491	~amyloid_beta+(1|manifest)+cngeneson+pc_mito+brain_region+Sex	5.953	597.000
ENSG00000112419	0.002	0.091	0.259	-0.078	PHACTR2	0.016	~amyloid_beta+(1|manifest)+cngeneson+pc_mito+brain_region+Sex	0.264	5790.000
ENSG00000047849	0.354	0.091	0.254	-0.072	MAP4	0.405	~amyloid_beta+(1|manifest)+cngeneson+pc_mito+brain_region+Sex	3.009	1538.000
ENSG00000111450	0.010	0.090	0.183	-0.002	STX2	0.039	~amyloid_beta+(1|manifest)+cngeneson+pc_mito+brain_region+Sex	0.705	4864.000
ENSG00000139644	0.345	0.090	0.411	-0.231	TMBIM6	0.396	~amyloid_beta+(1|manifest)+cngeneson+pc_mito+brain_region+Sex	3.460	1272.000
ENSG00000142784	0.005	0.090	0.171	0.009	WDTC1	0.027	~amyloid_beta+(1|manifest)+cngeneson+pc_mito+brain_region+Sex	0.584	5159.000
ENSG00000084731	0.005	0.090	0.203	-0.023	KIF3C	0.026	~amyloid_beta+(1|manifest)+cngeneson+pc_mito+brain_region+Sex	0.178	5902.000
ENSG00000198719	0.029	0.090	0.268	-0.088	DLL1	0.073	~amyloid_beta+(1|manifest)+cngeneson+pc_mito+brain_region+Sex	1.765	2728.000
ENSG00000151502	0.004	0.090	0.164	0.016	VPS26B	0.022	~amyloid_beta+(1|manifest)+cngeneson+pc_mito+brain_region+Sex	0.103	5951.000
ENSG00000214402	0.757	0.090	1.024	-0.845	LCNL1	0.781	~amyloid_beta+(1|manifest)+cngeneson+pc_mito+brain_region+Sex	9.244	310.000
ENSG00000198740	0.011	0.090	0.205	-0.025	ZNF652	0.041	~amyloid_beta+(1|manifest)+cngeneson+pc_mito+brain_region+Sex	0.182	5897.000
ENSG00000104205	0.254	0.090	0.233	-0.054	SGK3	0.308	~amyloid_beta+(1|manifest)+cngeneson+pc_mito+brain_region+Sex	2.458	1930.000
ENSG00000171885	0.569	0.090	0.259	-0.080	AQP4	0.608	~amyloid_beta+(1|manifest)+cngeneson+pc_mito+brain_region+Sex	10.607	241.000
ENSG00000130560	0.058	0.089	0.208	-0.030	UBAC1	0.109	~amyloid_beta+(1|manifest)+cngeneson+pc_mito+brain_region+Sex	1.178	3751.000
ENSG00000153187	0.174	0.089	0.340	-0.161	HNRNPU	0.229	~amyloid_beta+(1|manifest)+cngeneson+pc_mito+brain_region+Sex	1.210	3683.000
ENSG00000132952	0.014	0.089	0.197	-0.018	USPL1	0.049	~amyloid_beta+(1|manifest)+cngeneson+pc_mito+brain_region+Sex	0.207	5864.000
ENSG00000176155	0.029	0.089	0.238	-0.060	CCDC57	0.073	~amyloid_beta+(1|manifest)+cngeneson+pc_mito+brain_region+Sex	1.291	3527.000
ENSG00000146457	0.051	0.089	0.306	-0.128	WTAP	0.101	~amyloid_beta+(1|manifest)+cngeneson+pc_mito+brain_region+Sex	2.632	1786.000
ENSG00000007174	0.046	0.089	0.255	-0.077	DNAH9	0.093	~amyloid_beta+(1|manifest)+cngeneson+pc_mito+brain_region+Sex	0.373	5613.000
ENSG00000133773	0.318	0.089	0.314	-0.135	CCDC59	0.371	~amyloid_beta+(1|manifest)+cngeneson+pc_mito+brain_region+Sex	1.455	3213.000
ENSG00000004534	0.060	0.089	0.171	0.007	RBM6	0.111	~amyloid_beta+(1|manifest)+cngeneson+pc_mito+brain_region+Sex	2.278	2127.000
ENSG00000197779	0.104	0.089	0.258	-0.080	ZNF81	0.160	~amyloid_beta+(1|manifest)+cngeneson+pc_mito+brain_region+Sex	1.115	3897.000
ENSG00000119912	0.001	0.089	0.187	-0.010	IDE	0.009	~amyloid_beta+(1|manifest)+cngeneson+pc_mito+brain_region+Sex	0.625	5071.000
ENSG00000100644	0.199	0.089	0.389	-0.212	HIF1A	0.253	~amyloid_beta+(1|manifest)+cngeneson+pc_mito+brain_region+Sex	3.735	1147.000
ENSG00000168675	0.562	0.089	0.461	-0.284	LDLRAD4	0.601	~amyloid_beta+(1|manifest)+cngeneson+pc_mito+brain_region+Sex	1.932	2520.000
ENSG00000131018	0.601	0.088	0.280	-0.103	SYNE1	0.638	~amyloid_beta+(1|manifest)+cngeneson+pc_mito+brain_region+Sex	7.077	461.000
ENSG00000143797	0.146	0.088	0.297	-0.120	MBOAT2	0.202	~amyloid_beta+(1|manifest)+cngeneson+pc_mito+brain_region+Sex	1.751	2748.000
ENSG00000044574	0.298	0.088	0.528	-0.352	HSPA5	0.351	~amyloid_beta+(1|manifest)+cngeneson+pc_mito+brain_region+Sex	4.963	784.000
ENSG00000205542	0.035	0.088	0.403	-0.227	TMSB4X	0.081	~amyloid_beta+(1|manifest)+cngeneson+pc_mito+brain_region+Sex	2.302	2103.000
ENSG00000169193	0.002	0.088	0.180	-0.003	CCDC126	0.017	~amyloid_beta+(1|manifest)+cngeneson+pc_mito+brain_region+Sex	0.352	5647.000
ENSG00000160194	0.015	0.088	0.189	-0.013	NDUFV3	0.051	~amyloid_beta+(1|manifest)+cngeneson+pc_mito+brain_region+Sex	0.500	5360.000
ENSG00000184486	0.392	0.088	0.458	-0.283	POU3F2	0.441	~amyloid_beta+(1|manifest)+cngeneson+pc_mito+brain_region+Sex	3.632	1189.000
ENSG00000033327	0.765	0.088	0.584	-0.409	GAB2	0.788	~amyloid_beta+(1|manifest)+cngeneson+pc_mito+brain_region+Sex	4.616	859.000
ENSG00000148660	0.850	0.088	0.420	-0.244	CAMK2G	0.865	~amyloid_beta+(1|manifest)+cngeneson+pc_mito+brain_region+Sex	20.798	44.000
ENSG00000172005	0.059	0.088	0.302	-0.126	MAL	0.110	~amyloid_beta+(1|manifest)+cngeneson+pc_mito+brain_region+Sex	1.347	3417.000
ENSG00000065000	0.064	0.088	0.255	-0.079	AP3D1	0.116	~amyloid_beta+(1|manifest)+cngeneson+pc_mito+brain_region+Sex	1.824	2660.000
ENSG00000114268	0.018	0.088	0.187	-0.012	PFKFB4	0.055	~amyloid_beta+(1|manifest)+cngeneson+pc_mito+brain_region+Sex	0.961	4224.000
ENSG00000187257	0.008	0.088	0.158	0.017	RSBN1L	0.034	~amyloid_beta+(1|manifest)+cngeneson+pc_mito+brain_region+Sex	0.370	5616.000
ENSG00000166762	0.028	0.087	0.200	-0.025	CATSPER2	0.071	~amyloid_beta+(1|manifest)+cngeneson+pc_mito+brain_region+Sex	0.722	4820.000
ENSG00000196151	0.016	0.087	0.312	-0.137	WDSUB1	0.053	~amyloid_beta+(1|manifest)+cngeneson+pc_mito+brain_region+Sex	0.643	5023.000
ENSG00000170144	0.045	0.087	0.232	-0.058	HNRNPA3	0.094	~amyloid_beta+(1|manifest)+cngeneson+pc_mito+brain_region+Sex	1.127	3866.000
ENSG00000196220	0.788	0.087	0.386	-0.212	SRGAP3	0.808	~amyloid_beta+(1|manifest)+cngeneson+pc_mito+brain_region+Sex	12.268	167.000
ENSG00000214954	0.053	0.087	0.217	-0.043	LRRC69	0.103	~amyloid_beta+(1|manifest)+cngeneson+pc_mito+brain_region+Sex	0.468	5426.000
ENSG00000174684	0.029	0.087	0.272	-0.098	B4GAT1	0.073	~amyloid_beta+(1|manifest)+cngeneson+pc_mito+brain_region+Sex	0.341	5663.000
ENSG00000070756	0.106	0.087	0.316	-0.143	PABPC1	0.162	~amyloid_beta+(1|manifest)+cngeneson+pc_mito+brain_region+Sex	1.410	3306.000
ENSG00000108091	0.044	0.087	0.337	-0.164	CCDC6	0.092	~amyloid_beta+(1|manifest)+cngeneson+pc_mito+brain_region+Sex	1.486	3166.000
ENSG00000163162	0.027	0.087	0.276	-0.103	RNF149	0.070	~amyloid_beta+(1|manifest)+cngeneson+pc_mito+brain_region+Sex	2.728	1715.000
ENSG00000162627	0.022	0.086	0.244	-0.071	SNX7	0.062	~amyloid_beta+(1|manifest)+cngeneson+pc_mito+brain_region+Sex	0.307	5718.000
ENSG00000130558	0.005	0.086	0.208	-0.035	OLFM1	0.026	~amyloid_beta+(1|manifest)+cngeneson+pc_mito+brain_region+Sex	1.272	3562.000
ENSG00000125875	0.018	0.086	0.185	-0.013	TBC1D20	0.056	~amyloid_beta+(1|manifest)+cngeneson+pc_mito+brain_region+Sex	0.547	5237.000
ENSG00000164828	0.095	0.086	0.311	-0.139	SUN1	0.150	~amyloid_beta+(1|manifest)+cngeneson+pc_mito+brain_region+Sex	5.808	623.000
ENSG00000155816	0.067	0.086	0.215	-0.043	FMN2	0.120	~amyloid_beta+(1|manifest)+cngeneson+pc_mito+brain_region+Sex	7.012	469.000
ENSG00000011021	0.037	0.086	0.212	-0.041	CLCN6	0.083	~amyloid_beta+(1|manifest)+cngeneson+pc_mito+brain_region+Sex	0.854	4487.000
ENSG00000159202	0.013	0.086	0.194	-0.022	UBE2Z	0.045	~amyloid_beta+(1|manifest)+cngeneson+pc_mito+brain_region+Sex	0.204	5868.000
ENSG00000169609	0.042	0.085	0.215	-0.044	C15orf40	0.089	~amyloid_beta+(1|manifest)+cngeneson+pc_mito+brain_region+Sex	0.823	4560.000
ENSG00000131508	0.009	0.085	0.248	-0.077	UBE2D2	0.036	~amyloid_beta+(1|manifest)+cngeneson+pc_mito+brain_region+Sex	0.277	5768.000
ENSG00000171316	0.293	0.085	0.257	-0.086	CHD7	0.346	~amyloid_beta+(1|manifest)+cngeneson+pc_mito+brain_region+Sex	3.505	1249.000
ENSG00000141404	0.147	0.085	0.261	-0.090	GNAL	0.203	~amyloid_beta+(1|manifest)+cngeneson+pc_mito+brain_region+Sex	1.231	3637.000
ENSG00000149269	0.053	0.085	0.234	-0.063	PAK1	0.103	~amyloid_beta+(1|manifest)+cngeneson+pc_mito+brain_region+Sex	0.759	4719.000
ENSG00000273540	0.259	0.085	0.323	-0.153	AGBL1	0.313	~amyloid_beta+(1|manifest)+cngeneson+pc_mito+brain_region+Sex	2.422	1961.000
ENSG00000078549	0.221	0.085	0.376	-0.206	[illegible]	0.276	~amyloid_beta+(1|manifest)+cngeneson+pc_mito+brain_region+Sex	3.172	1440.000
ENSG00000164164	0.003	0.085	0.180	-0.010	OTUD4	0.020	~amyloid_beta+(1|manifest)+cngeneson+pc_mito+brain_region+Sex	0.545	5243.000
ENSG00000106459	0.148	0.085	0.242	-0.073	NRF1	0.204	~amyloid_beta+(1|manifest)+cngeneson+pc_mito+brain_region+Sex	1.629	2941.000
ENSG00000163743	0.006	0.084	0.163	0.005	RCHY1	0.029	~amyloid_beta+(1|manifest)+cngeneson+pc_mito+brain_region+Sex	0.541	5256.000
ENSG00000204977	0.039	0.084	0.196	-0.027	TRIM13	0.087	~amyloid_beta+(1|manifest)+cngeneson+pc_mito+brain_region+Sex	0.921	4325.000
ENSG00000104763	0.049	0.084	0.271	-0.102	ASAH1	0.098	~amyloid_beta+(1|manifest)+cngeneson+pc_mito+brain_region+Sex	1.847	2632.000
ENSG00000182606	0.287	0.084	0.192	-0.023	TRAK1	0.340	~amyloid_beta+(1|manifest)+cngeneson+pc_mito+brain_region+Sex	3.147	1456.000
ENSG00000132561	0.839	0.084	0.801	-0.632	MATN2	0.855	~amyloid_beta+(1|manifest)+cngeneson+pc_mito+brain_region+Sex	19.472	56.000
ENSG00000116005	0.073	0.084	0.206	-0.037	PCYOX1	0.127	~amyloid_beta+(1|manifest)+cngeneson+pc_mito+brain_region+Sex	0.527	5291.000
ENSG00000132604	0.006	0.084	0.171	-0.002	TERF2	0.030	~amyloid_beta+(1|manifest)+cngeneson+pc_mito+brain_region+Sex	0.299	5736.000
ENSG00000149182	0.078	0.084	0.264	-0.096	ARFGAP2	0.133	~amyloid_beta+(1|manifest)+cngeneson+pc_mito+brain_region+Sex	1.692	2837.000
ENSG00000173575	0.136	0.084	0.259	-0.091	CHD2	0.193	~amyloid_beta+(1|manifest)+cngeneson+pc_mito+brain_region+Sex	2.748	1706.000
ENSG00000100099	0.013	0.084	0.226	-0.058	HPS4	0.047	~amyloid_beta+(1|manifest)+cngeneson+pc_mito+brain_region+Sex	0.540	5260.000
ENSG00000119396	0.013	0.084	0.160	0.008	RAB14	0.047	~amyloid_beta+(1|manifest)+cngeneson+pc_mito+brain_region+Sex	0.249	5810.000
ENSG00000076928	0.193	0.084	0.355	-0.187	ARHGEF1	0.247	~amyloid_beta+(1|manifest)+cngeneson+pc_mito+brain_region+Sex	4.075	1022.000
ENSG00000186577	0.125	0.084	0.343	-0.175	SMIM29	0.181	~amyloid_beta+(1|manifest)+cngeneson+pc_mito+brain_region+Sex	2.022	2409.000
ENSG00000133030	0.115	0.084	0.219	-0.052	MPRIP	0.171	~amyloid_beta+(1|manifest)+cngeneson+pc_mito+brain_region+Sex	1.763	2731.000
ENSG00000137942	0.159	0.083	0.260	-0.093	FNBP1L	0.214	~amyloid_beta+(1|manifest)+cngeneson+pc_mito+brain_region+Sex	0.796	4641.000
ENSG00000130822	0.099	0.083	0.263	-0.097	PNCK	0.154	~amyloid_beta+(1|manifest)+cngeneson+pc_mito+brain_region+Sex	2.504	1899.000
ENSG00000149313	0.006	0.083	0.145	0.022	AASDHPPT	0.030	~amyloid_beta+(1|manifest)+cngeneson+pc_mito+brain_region+Sex	0.122	5942.000
ENSG00000164292	0.356	0.083	0.195	-0.028	RHOBTB3	0.407	~amyloid_beta+(1|manifest)+cngeneson+pc_mito+brain_region+Sex	3.619	1197.000
ENSG00000166326	0.083	0.083	0.248	-0.081	TRIM44	0.138	~amyloid_beta+(1|manifest)+cngeneson+pc_mito+brain_region+Sex	0.559	5214.000
ENSG00000182259	0.010	0.083	0.183	-0.016	DNAJC7	0.039	~amyloid_beta+(1|manifest)+cngeneson+pc_mito+brain_region+Sex	0.319	5693.000
ENSG00000100997	0.003	0.083	0.218	-0.052	ABHD12	0.022	~amyloid_beta+(1|manifest)+cngeneson+pc_mito+brain_region+Sex	1.018	4101.000
ENSG00000138386	0.002	0.083	0.167	-0.001	NAB1	0.014	~amyloid_beta+(1|manifest)+cngeneson+pc_mito+brain_region+Sex	0.548	5234.000
ENSG00000014216	0.110	0.083	0.227	-0.061	CAPN1	0.166	~amyloid_beta+(1|manifest)+cngeneson+pc_mito+brain_region+Sex	1.308	3486.000
ENSG00000095951	0.067	0.083	0.289	-0.123	HIVEP1	0.121	~amyloid_beta+(1|manifest)+cngeneson+pc_mito+brain_region+Sex	3.429	1292.000
ENSG00000052795	0.003	0.083	0.166	0.000	FNIP2	0.022	~amyloid_beta+(1|manifest)+cngeneson+pc_mito+brain_region+Sex	0.559	5215.000
ENSG00000218739	0.010	0.083	0.168	-0.003	CEBPZOS	0.039	~amyloid_beta+(1|manifest)+cngeneson+pc_mito+brain_region+Sex	0.872	4432.000
ENSG00000177463	0.036	0.083	0.208	-0.043	NR2C2	0.082	~amyloid_beta+(1|manifest)+cngeneson+pc_mito+brain_region+Sex	0.891	4395.000
ENSG00000197006	0.011	0.083	0.197	-0.031	METTL9	0.041	~amyloid_beta+(1|manifest)+cngeneson+pc_mito+brain_region+Sex	0.219	5847.000
ENSG00000103342	0.010	0.083	0.190	-0.025	GSPT1	0.039	~amyloid_beta+(1|manifest)+cngeneson+pc_mito+brain_region+Sex	0.618	5092.000
ENSG00000164548	0.109	0.082	0.268	-0.103	TRA2A	0.165	~amyloid_beta+(1|manifest)+cngeneson+pc_mito+brain_region+Sex	2.920	1595.000

ENSG00000071553	0.054	0.082	0.241	-0.076	ATP6AP1	0.104	~amyloid_beta+(1|manifest)+cngeneson+pc_mito+brain_region+Sex	0.792	4649.000
ENSG00000160209	0.057	0.082	0.293	-0.129	PDXK	0.108	~amyloid_beta+(1|manifest)+cngeneson+pc_mito+brain_region+Sex	1.923	2533.000
ENSG00000114573	0.058	0.082	0.202	-0.037	ATP6V1A	0.109	~amyloid_beta+(1|manifest)+cngeneson+pc_mito+brain_region+Sex	0.855	4485.000
ENSG00000005483	0.064	0.082	0.229	-0.065	KMT2E	0.116	~amyloid_beta+(1|manifest)+cngeneson+pc_mito+brain_region+Sex	0.688	4922.000
ENSG00000165959	0.002	0.082	0.371	-0.207	CLMN	0.016	~amyloid_beta+(1|manifest)+cngeneson+pc_mito+brain_region+Sex	1.211	3680.000
ENSG00000102984	0.012	0.082	0.175	-0.011	ZNF821	0.045	~amyloid_beta+(1|manifest)+cngeneson+pc_mito+brain_region+Sex	0.930	4302.000
ENSG00000169372	0.496	0.082	0.260	-0.096	CRADD	0.540	~amyloid_beta+(1|manifest)+cngeneson+pc_mito+brain_region+Sex	2.391	1998.000
ENSG00000182149	0.001	0.082	0.154	0.009	IST1	0.011	~amyloid_beta+(1|manifest)+cngeneson+pc_mito+brain_region+Sex	0.751	4741.000
ENSG00000198513	0.004	0.082	0.240	-0.077	ATL1	0.025	~amyloid_beta+(1|manifest)+cngeneson+pc_mito+brain_region+Sex	0.555	5223.000
ENSG00000269713	0.051	0.081	0.269	-0.107	NBPF9	0.101	~amyloid_beta+(1|manifest)+cngeneson+pc_mito+brain_region+Sex	0.822	4566.000
ENSG00000164985	0.009	0.081	0.158	0.004	PSIP1	0.037	~amyloid_beta+(1|manifest)+cngeneson+pc_mito+brain_region+Sex	0.238	5828.000
ENSG00000156170	0.031	0.081	0.265	-0.103	NDUFAF6	0.075	~amyloid_beta+(1|manifest)+cngeneson+pc_mito+brain_region+Sex	1.668	2874.000
ENSG00000163297	0.111	0.081	0.295	-0.133	ANTXR2	0.167	~amyloid_beta+(1|manifest)+cngeneson+pc_mito+brain_region+Sex	0.507	5333.000
ENSG00000108551	0.833	0.081	0.513	-0.352	RASD1	0.851	~amyloid_beta+(1|manifest)+cngeneson+pc_mito+brain_region+Sex	4.245	966.000
ENSG00000110076	0.045	0.081	0.196	-0.034	NRXN2	0.094	~amyloid_beta+(1|manifest)+cngeneson+pc_mito+brain_region+Sex	0.566	5194.000
ENSG00000174238	0.006	0.081	0.199	-0.038	PITPNA	0.030	~amyloid_beta+(1|manifest)+cngeneson+pc_mito+brain_region+Sex	0.827	4546.000
ENSG00000147459	0.371	0.081	0.428	-0.266	DOCK5	0.421	~amyloid_beta+(1|manifest)+cngeneson+pc_mito+brain_region+Sex	2.619	1802.000
ENSG00000129493	0.017	0.081	0.186	-0.025	HEATR5A	0.053	~amyloid_beta+(1|manifest)+cngeneson+pc_mito+brain_region+Sex	0.775	4676.000
ENSG00000141867	0.075	0.081	0.297	-0.136	BRD4	0.128	~amyloid_beta+(1|manifest)+cngeneson+pc_mito+brain_region+Sex	2.141	2269.000
ENSG00000100426	0.002	0.081	0.141	0.020	ZBED4	0.016	~amyloid_beta+(1|manifest)+cngeneson+pc_mito+brain_region+Sex	0.361	5632.000
ENSG00000065526	0.121	0.081	0.287	-0.126	SPEN	0.178	~amyloid_beta+(1|manifest)+cngeneson+pc_mito+brain_region+Sex	2.230	2173.000
ENSG00000075151	0.186	0.080	0.187	-0.026	EIF4G3	0.240	~amyloid_beta+(1|manifest)+cngeneson+pc_mito+brain_region+Sex	3.726	1150.000
ENSG00000078369	0.042	0.080	0.251	-0.090	GNB1	0.089	~amyloid_beta+(1|manifest)+cngeneson+pc_mito+brain_region+Sex	1.296	3513.000
ENSG00000127947	0.046	0.080	0.318	-0.157	PTPN12	0.095	~amyloid_beta+(1|manifest)+cngeneson+pc_mito+brain_region+Sex	2.418	1962.000
ENSG00000135677	0.021	0.080	0.191	-0.031	GNS	0.061	~amyloid_beta+(1|manifest)+cngeneson+pc_mito+brain_region+Sex	0.722	4818.000
ENSG00000100201	0.254	0.080	0.173	-0.012	DDX17	0.308	~amyloid_beta+(1|manifest)+cngeneson+pc_mito+brain_region+Sex	9.166	312.000
ENSG00000173065	0.028	0.080	0.223	-0.064	FAM222B	0.072	~amyloid_beta+(1|manifest)+cngeneson+pc_mito+brain_region+Sex	0.706	4861.000
ENSG00000064601	0.108	0.080	0.258	-0.099	CTSA	0.164	~amyloid_beta+(1|manifest)+cngeneson+pc_mito+brain_region+Sex	0.930	4301.000
ENSG00000205356	0.067	0.080	0.241	-0.082	TECPR1	0.121	~amyloid_beta+(1|manifest)+cngeneson+pc_mito+brain_region+Sex	1.580	3012.000
ENSG00000133069	0.204	0.079	0.377	-0.218	TMCC2	0.259	~amyloid_beta+(1|manifest)+cngeneson+pc_mito+brain_region+Sex	4.330	939.000
ENSG00000183530	0.009	0.079	0.228	-0.070	PRR14L	0.037	~amyloid_beta+(1|manifest)+cngeneson+pc_mito+brain_region+Sex	0.631	5055.000
ENSG00000028310	0.021	0.079	0.239	-0.080	BRD9	0.062	~amyloid_beta+(1|manifest)+cngeneson+pc_mito+brain_region+Sex	1.227	3652.000
ENSG00000141252	0.033	0.079	0.291	-0.133	VPS53	0.078	~amyloid_beta+(1|manifest)+cngeneson+pc_mito+brain_region+Sex	4.396	917.000
ENSG00000124160	0.126	0.079	0.233	-0.075	NCOA5	0.182	~amyloid_beta+(1|manifest)+cngeneson+pc_mito+brain_region+Sex	1.099	3930.000
ENSG00000130382	0.009	0.079	0.172	-0.015	MLLT1	0.037	~amyloid_beta+(1|manifest)+cngeneson+pc_mito+brain_region+Sex	0.420	5526.000
ENSG00000121897	0.021	0.079	0.191	-0.034	LIAS	0.061	~amyloid_beta+(1|manifest)+cngeneson+pc_mito+brain_region+Sex	0.641	5030.000
ENSG00000084764	0.052	0.079	0.216	-0.058	MAPRE3	0.102	~amyloid_beta+(1|manifest)+cngeneson+pc_mito+brain_region+Sex	1.962	2476.000
ENSG00000115419	0.100	0.079	0.288	-0.131	GLS	0.156	~amyloid_beta+(1|manifest)+cngeneson+pc_mito+brain_region+Sex	2.099	2316.000
ENSG00000168803	0.044	0.079	0.219	-0.062	ADAL	0.093	~amyloid_beta+(1|manifest)+cngeneson+pc_mito+brain_region+Sex	1.653	2897.000
ENSG00000010295	0.094	0.078	0.236	-0.080	IFFO1	0.150	~amyloid_beta+(1|manifest)+cngeneson+pc_mito+brain_region+Sex	1.666	2878.000
ENSG00000101974	0.085	0.078	0.248	-0.092	ATP11C	0.140	~amyloid_beta+(1|manifest)+cngeneson+pc_mito+brain_region+Sex	2.282	2123.000
ENSG00000227051	0.013	0.078	0.197	-0.041	C14orf132	0.047	~amyloid_beta+(1|manifest)+cngeneson+pc_mito+brain_region+Sex	0.906	4365.000
ENSG00000047644	0.002	0.078	0.149	0.006	WWC3	0.018	~amyloid_beta+(1|manifest)+cngeneson+pc_mito+brain_region+Sex	0.299	5737.000
ENSG00000116668	0.003	0.078	0.193	-0.037	SWT1	0.019	~amyloid_beta+(1|manifest)+cngeneson+pc_mito+brain_region+Sex	0.185	5893.000
ENSG00000142459	0.012	0.078	0.185	-0.029	EVI5L	0.043	~amyloid_beta+(1|manifest)+cngeneson+pc_mito+brain_region+Sex	0.967	4211.000
ENSG00000111790	0.035	0.078	0.210	-0.055	FGFR1OP2	0.081	~amyloid_beta+(1|manifest)+cngeneson+pc_mito+brain_region+Sex	0.799	4636.000
ENSG00000152284	0.391	0.078	0.306	-0.151	TCF7L1	0.440	~amyloid_beta+(1|manifest)+cngeneson+pc_mito+brain_region+Sex	2.308	2093.000
ENSG00000119431	0.088	0.077	0.292	-0.138	HDHD3	0.143	~amyloid_beta+(1|manifest)+cngeneson+pc_mito+brain_region+Sex	3.430	1291.000
ENSG00000121753	0.129	0.077	0.305	-0.150	ADGRB2	0.185	~amyloid_beta+(1|manifest)+cngeneson+pc_mito+brain_region+Sex	1.985	2453.000
ENSG00000146067	0.059	0.077	0.250	-0.096	FAM193B	0.110	~amyloid_beta+(1|manifest)+cngeneson+pc_mito+brain_region+Sex	2.481	1914.000
ENSG00000101391	0.095	0.077	0.192	-0.037	CDK5RAP1	0.150	~amyloid_beta+(1|manifest)+cngeneson+pc_mito+brain_region+Sex	0.820	4572.000
ENSG00000107679	0.005	0.077	0.204	-0.050	PLEKHA1	0.027	~amyloid_beta+(1|manifest)+cngeneson+pc_mito+brain_region+Sex	0.710	4848.000
ENSG00000115204	0.007	0.077	0.163	-0.008	MPV17	0.033	~amyloid_beta+(1|manifest)+cngeneson+pc_mito+brain_region+Sex	0.704	4871.000
ENSG00000152377	0.645	0.077	0.587	-0.433	SPOCK1	0.678	~amyloid_beta+(1|manifest)+cngeneson+pc_mito+brain_region+Sex	12.805	155.000
ENSG00000103264	0.007	0.077	0.151	0.003	FBXO31	0.032	~amyloid_beta+(1|manifest)+cngeneson+pc_mito+brain_region+Sex	0.435	5494.000
ENSG00000185129	0.107	0.077	0.259	-0.105	PURA	0.163	~amyloid_beta+(1|manifest)+cngeneson+pc_mito+brain_region+Sex	1.222	3659.000
ENSG00000150768	0.004	0.077	0.160	-0.006	DLAT	0.023	~amyloid_beta+(1|manifest)+cngeneson+pc_mito+brain_region+Sex	0.529	5288.000
ENSG00000135362	0.029	0.077	0.178	-0.024	PRR5L	0.072	~amyloid_beta+(1|manifest)+cngeneson+pc_mito+brain_region+Sex	0.370	5617.000
ENSG00000140497	0.043	0.077	0.168	-0.015	SCAMP2	0.091	~amyloid_beta+(1|manifest)+cngeneson+pc_mito+brain_region+Sex	0.401	5563.000
ENSG00000132970	0.678	0.077	0.465	-0.311	WASF3	0.709	~amyloid_beta+(1|manifest)+cngeneson+pc_mito+brain_region+Sex	6.210	562.000
ENSG00000011275	0.031	0.077	0.229	-0.075	RNF216	0.075	~amyloid_beta+(1|manifest)+cngeneson+pc_mito+brain_region+Sex	0.881	4417.000
ENSG00000106554	0.322	0.077	0.276	-0.123	CHCHD3	0.375	~amyloid_beta+(1|manifest)+cngeneson+pc_mito+brain_region+Sex	1.260	3583.000
ENSG00000138796	0.000	0.076	0.128	0.025	HADH	0.006	~amyloid_beta+(1|manifest)+cngeneson+pc_mito+brain_region+Sex	0.031	5978.000
ENSG00000096093	0.267	0.076	0.327	-0.174	EFHC1	0.320	~amyloid_beta+(1|manifest)+cngeneson+pc_mito+brain_region+Sex	1.910	2544.000
ENSG00000164715	0.002	0.076	0.123	0.030	LMTK2	0.016	~amyloid_beta+(1|manifest)+cngeneson+pc_mito+brain_region+Sex	0.431	5505.000
ENSG00000102908	0.575	0.076	0.217	-0.064	NFAT5	0.614	~amyloid_beta+(1|manifest)+cngeneson+pc_mito+brain_region+Sex	3.933	1074.000
ENSG00000183605	0.088	0.076	0.232	-0.079	SFXN4	0.143	~amyloid_beta+(1|manifest)+cngeneson+pc_mito+brain_region+Sex	1.808	2683.000
ENSG00000174844	0.048	0.076	0.211	-0.059	DNAH12	0.097	~amyloid_beta+(1|manifest)+cngeneson+pc_mito+brain_region+Sex	0.886	4407.000
ENSG00000101158	0.062	0.076	0.233	-0.080	NELFCD	0.114	~amyloid_beta+(1|manifest)+cngeneson+pc_mito+brain_region+Sex	1.572	3021.000
ENSG00000136878	0.028	0.076	0.173	-0.021	USP20	0.071	~amyloid_beta+(1|manifest)+cngeneson+pc_mito+brain_region+Sex	1.132	3860.000
ENSG00000065978	0.011	0.076	0.187	-0.035	YBX1	0.043	~amyloid_beta+(1|manifest)+cngeneson+pc_mito+brain_region+Sex	0.704	4868.000
ENSG00000172661	0.012	0.076	0.158	-0.005	WASHC2C	0.044	~amyloid_beta+(1|manifest)+cngeneson+pc_mito+brain_region+Sex	0.865	4453.000
ENSG00000141639	0.718	0.076	0.262	-0.109	MAPK4	0.745	~amyloid_beta+(1|manifest)+cngeneson+pc_mito+brain_region+Sex	3.871	1096.000
ENSG00000119760	0.053	0.076	0.189	-0.037	SUPT7L	0.103	~amyloid_beta+(1|manifest)+cngeneson+pc_mito+brain_region+Sex	1.177	3753.000
ENSG00000123908	0.022	0.076	0.302	-0.150	AGO2	0.063	~amyloid_beta+(1|manifest)+cngeneson+pc_mito+brain_region+Sex	1.710	2809.000
ENSG00000156928	0.022	0.076	0.157	-0.006	MALSU1	0.062	~amyloid_beta+(1|manifest)+cngeneson+pc_mito+brain_region+Sex	0.233	5833.000
ENSG00000075415	0.069	0.076	0.196	-0.044	SLC25A3	0.122	~amyloid_beta+(1|manifest)+cngeneson+pc_mito+brain_region+Sex	0.702	4881.000
ENSG00000156599	0.006	0.076	0.162	-0.011	ZDHHC5	0.030	~amyloid_beta+(1|manifest)+cngeneson+pc_mito+brain_region+Sex	0.500	5359.000
ENSG00000106686	0.007	0.075	0.270	-0.119	SPATA6L	0.033	~amyloid_beta+(1|manifest)+cngeneson+pc_mito+brain_region+Sex	1.829	2656.000
ENSG00000078808	0.217	0.075	0.280	-0.129	SDF4	0.272	~amyloid_beta+(1|manifest)+cngeneson+pc_mito+brain_region+Sex	2.179	2225.000
ENSG00000135040	0.024	0.075	0.209	-0.058	NAA35	0.065	~amyloid_beta+(1|manifest)+cngeneson+pc_mito+brain_region+Sex	1.229	3645.000
ENSG00000147439	0.011	0.075	0.186	-0.035	BIN3	0.043	~amyloid_beta+(1|manifest)+cngeneson+pc_mito+brain_region+Sex	0.542	5252.000
ENSG00000156976	0.173	0.075	0.266	-0.116	EIF4A2	0.228	~amyloid_beta+(1|manifest)+cngeneson+pc_mito+brain_region+Sex	1.076	3973.000
ENSG00000148730	0.059	0.075	0.188	-0.039	EIF4EBP2	0.110	~amyloid_beta+(1|manifest)+cngeneson+pc_mito+brain_region+Sex	0.283	5758.000
ENSG00000185049	0.043	0.075	0.207	-0.057	NELFA	0.091	~amyloid_beta+(1|manifest)+cngeneson+pc_mito+brain_region+Sex	0.536	5266.000
ENSG00000105854	0.585	0.075	0.230	-0.081	PON2	0.622	~amyloid_beta+(1|manifest)+cngeneson+pc_mito+brain_region+Sex	5.276	714.000
ENSG00000107863	0.269	0.075	0.184	-0.035	ARHGAP21	0.323	~amyloid_beta+(1|manifest)+cngeneson+pc_mito+brain_region+Sex	2.496	1908.000
ENSG00000114026	0.026	0.074	0.163	-0.014	OGG1	0.069	~amyloid_beta+(1|manifest)+cngeneson+pc_mito+brain_region+Sex	0.842	4517.000
ENSG00000205269	0.175	0.074	0.231	-0.082	[illegible]	0.230	~amyloid_beta+(1|manifest)+cngeneson+pc_mito+brain_region+Sex	2.027	2400.000
ENSG00000185532	0.291	0.074	0.276	-0.128	PRKG1	0.343	~amyloid_beta+(1|manifest)+cngeneson+pc_mito+brain_region+Sex	11.634	193.000
ENSG00000168615	0.047	0.074	0.239	-0.091	ADAM9	0.096	~amyloid_beta+(1|manifest)+cngeneson+pc_mito+brain_region+Sex	0.396	5569.000
ENSG00000100372	0.018	0.074	0.181	-0.033	SLC25A17	0.056	~amyloid_beta+(1|manifest)+cngeneson+pc_mito+brain_region+Sex	0.870	4437.000
ENSG00000258890	0.022	0.074	0.211	-0.063	CEP95	0.062	~amyloid_beta+(1|manifest)+cngeneson+pc_mito+brain_region+Sex	0.523	5299.000
ENSG00000101294	0.006	0.074	0.222	-0.074	HM13	0.028	~amyloid_beta+(1|manifest)+cngeneson+pc_mito+brain_region+Sex	1.216	3671.000
ENSG00000116478	0.035	0.074	0.187	-0.040	HDAC1	0.081	~amyloid_beta+(1|manifest)+cngeneson+pc_mito+brain_region+Sex	1.434	3256.000
ENSG00000138942	0.002	0.074	0.128	0.020	RNF185	0.015	~amyloid_beta+(1|manifest)+cngeneson+pc_mito+brain_region+Sex	0.211	5859.000
ENSG00000109756	0.423	0.073	0.261	-0.114	RAPGEF2	0.469	~amyloid_beta+(1|manifest)+cngeneson+pc_mito+brain_region+Sex	1.212	3678.000
ENSG00000185728	0.028	0.073	0.166	-0.020	YTHDF3	0.072	~amyloid_beta+(1|manifest)+cngeneson+pc_mito+brain_region+Sex	0.754	4732.000
ENSG00000011485	0.098	0.073	0.249	-0.103	PPP5C	0.153	~amyloid_beta+(1|manifest)+cngeneson+pc_mito+brain_region+Sex	1.135	3850.000
ENSG00000181035	0.025	0.073	0.212	-0.066	SLC25A42	0.068	~amyloid_beta+(1|manifest)+cngeneson+pc_mito+brain_region+Sex	1.324	3455.000
ENSG00000078403	0.330	0.073	0.197	-0.051	MLLT10	0.382	~amyloid_beta+(1|manifest)+cngeneson+pc_mito+brain_region+Sex	1.747	2752.000
ENSG00000100324	0.093	0.073	0.266	-0.120	TAB1	0.148	~amyloid_beta+(1|manifest)+cngeneson+pc_mito+brain_region+Sex	2.521	1881.000
ENSG00000173706	0.040	0.073	0.224	-0.078	HEG1	0.087	~amyloid_beta+(1|manifest)+cngeneson+pc_mito+brain_region+Sex	0.869	4440.000
ENSG00000087076	0.035	0.073	0.291	-0.145	HSD17B14	0.081	~amyloid_beta+(1|manifest)+cngeneson+pc_mito+brain_region+Sex	2.653	1767.000
ENSG00000105397	0.023	0.073	0.158	-0.013	TYK2	0.064	~amyloid_beta+(1|manifest)+cngeneson+pc_mito+brain_region+Sex	0.634	5048.000
ENSG00000125826	0.144	0.073	0.320	-0.174	RBCK1	0.200	~amyloid_beta+(1|manifest)+cngeneson+pc_mito+brain_region+Sex	3.312	1362.000
ENSG00000214029	0.004	0.073	0.151	-0.006	ZNF891	0.023	~amyloid_beta+(1|manifest)+cngeneson+pc_mito+brain_region+Sex	0.486	5393.000
ENSG00000122965	0.013	0.073	0.157	-0.012	RBM19	0.047	~amyloid_beta+(1|manifest)+cngeneson+pc_mito+brain_region+Sex	0.573	5178.000
ENSG00000171791	0.550	0.072	0.198	-0.053	BCL2	0.591	~amyloid_beta+(1|manifest)+cngeneson+pc_mito+brain_region+Sex	2.452	1934.000
ENSG00000073169	0.035	0.072	0.211	-0.066	SELENOO	0.081	~amyloid_beta+(1|manifest)+cngeneson+pc_mito+brain_region+Sex	1.505	3130.000
ENSG00000040341	0.179	0.072	0.253	-0.108	STAU2	0.234	~amyloid_beta+(1|manifest)+cngeneson+pc_mito+brain_region+Sex	1.737	2767.000
ENSG00000163291	0.006	0.072	0.213	-0.068	PAQR3	0.030	~amyloid_beta+(1|manifest)+cngeneson+pc_mito+brain_region+Sex	1.007	4126.000
ENSG00000198453	0.001	0.072	0.167	-0.022	ZNF568	0.014	~amyloid_beta+(1|manifest)+cngeneson+pc_mito+brain_region+Sex	1.031	4069.000
ENSG00000123552	0.012	0.072	0.164	-0.020	USP45	0.043	~amyloid_beta+(1|manifest)+cngeneson+pc_mito+brain_region+Sex	0.906	4368.000
ENSG00000138413	0.035	0.072	0.274	-0.130	IDH1	0.081	~amyloid_beta+(1|manifest)+cngeneson+pc_mito+brain_region+Sex	0.786	4660.000
ENSG00000173418	0.004	0.072	0.137	0.006	NAA20	0.023	~amyloid_beta+(1|manifest)+cngeneson+pc_mito+brain_region+Sex	0.123	5941.000
ENSG00000155097	0.001	0.072	0.168	-0.024	ATP6V1C1	0.014	~amyloid_beta+(1|manifest)+cngeneson+pc_mito+brain_region+Sex	0.157	5917.000
ENSG00000161010	0.100	0.072	0.189	-0.046	MRNIP	0.155	~amyloid_beta+(1|manifest)+cngeneson+pc_mito+brain_region+Sex	1.282	3541.000
ENSG00000169727	0.174	0.072	0.346	-0.203	GPS1	0.229	~amyloid_beta+(1|manifest)+cngeneson+pc_mito+brain_region+Sex	3.747	1144.000
ENSG00000137500	0.028	0.072	0.183	-0.040	CCDC90B	0.072	~amyloid_beta+(1|manifest)+cngeneson+pc_mito+brain_region+Sex	0.146	5928.000
ENSG00000101407	0.022	0.071	0.175	-0.032	TTI1	0.062	~amyloid_beta+(1|manifest)+cngeneson+pc_mito+brain_region+Sex	0.321	5691.000
ENSG00000149256	0.938	0.071	0.677	-0.534	TENM4	0.945	~amyloid_beta+(1|manifest)+cngeneson+pc_mito+brain_region+Sex	22.476	37.000
ENSG00000116209	0.070	0.071	0.249	-0.106	TMEM59	0.123	~amyloid_beta+(1|manifest)+cngeneson+pc_mito+brain_region+Sex	1.088	3953.000
ENSG00000171204	0.029	0.071	0.205	-0.063	[illegible]	0.073	~amyloid_beta+(1|manifest)+cngeneson+pc_mito+brain_region+Sex	0.248	5811.000
ENSG00000112659	0.019	0.071	0.164	-0.022	CUL9	0.057	~amyloid_beta+(1|manifest)+cngeneson+pc_mito+brain_region+Sex	0.892	4391.000
ENSG00000138594	0.004	0.071	0.143	-0.001	TMOD3	0.025	~amyloid_beta+(1|manifest)+cngeneson+pc_mito+brain_region+Sex	0.630	5058.000
ENSG00000174197	0.068	0.071	0.272	-0.130	MGA	0.121	~amyloid_beta+(1|manifest)+cngeneson+pc_mito+brain_region+Sex	4.213	975.000
ENSG00000148408	0.093	0.071	0.462	-0.321	CACNA1B	0.149	~amyloid_beta+(1|manifest)+cngeneson+pc_mito+brain_region+Sex	2.752	1702.000
ENSG00000149823	0.031	0.071	0.221	-0.079	VPS51	0.075	~amyloid_beta+(1|manifest)+cngeneson+pc_mito+brain_region+Sex	1.335	3439.000
ENSG00000103222	0.112	0.071	0.266	-0.125	ABCC1	0.168	~amyloid_beta+(1|manifest)+cngeneson+pc_mito+brain_region+Sex	3.273	1386.000
ENSG00000143393	0.067	0.071	0.214	-0.073	PI4KB	0.120	~amyloid_beta+(1|manifest)+cngeneson+pc_mito+brain_region+Sex	1.138	3842.000
ENSG00000196227	0.233	0.071	0.284	-0.143	FAM217B	0.287	~amyloid_beta+(1|manifest)+cngeneson+pc_mito+brain_region+Sex	0.899	4376.000
ENSG00000104131	0.002	0.070	0.135	0.006	EIF3J	0.018	~amyloid_beta+(1|manifest)+cngeneson+pc_mito+brain_region+Sex	0.322	5688.000
ENSG00000152457	0.100	0.070	0.236	-0.095	DCLRE1C	0.155	~amyloid_beta+(1|manifest)+cngeneson+pc_mito+brain_region+Sex	1.202	3700.000
ENSG00000205629	0.010	0.070	0.162	-0.021	LCMT1	0.039	~amyloid_beta+(1|manifest)+cngeneson+pc_mito+brain_region+Sex	0.474	5413.000
ENSG00000067596	0.070	0.070	0.230	-0.089	DHX8	0.124	~amyloid_beta+(1|manifest)+cngeneson+pc_mito+brain_region+Sex	1.461	3206.000
ENSG00000186480	0.023	0.070	0.254	-0.113	INSIG1	0.065	~amyloid_beta+(1|manifest)+cngeneson+pc_mito+brain_region+Sex	1.449	3231.000
ENSG00000140943	0.045	0.070	0.181	-0.040	MBTPS1	0.093	~amyloid_beta+(1|manifest)+cngeneson+pc_mito+brain_region+Sex	1.060	4004.000
ENSG00000104529	0.062	0.070	0.298	-0.157	EEF1D	0.114	~amyloid_beta+(1|manifest)+cngeneson+pc_mito+brain_region+Sex	2.857	1629.000
ENSG00000110756	0.012	0.070	0.142	-0.002	HPS5	0.043	~amyloid_beta+(1|manifest)+cngeneson+pc_mito+brain_region+Sex	0.599	5134.000
ENSG00000124574	0.037	0.070	0.162	-0.022	ABCC10	0.083	~amyloid_beta+(1|manifest)+cngeneson+pc_mito+brain_region+Sex	0.753	4734.000
ENSG00000103671	0.002	0.070	0.140	0.001	TRIP4	0.015	~amyloid_beta+(1|manifest)+cngeneson+pc_mito+brain_region+Sex	0.220	5846.000
ENSG00000196357	0.051	0.070	0.236	-0.096	ZNF565	0.101	~amyloid_beta+(1|manifest)+cngeneson+pc_mito+brain_region+Sex	2.247	2159.000
ENSG00000021300	0.313	0.070	0.376	-0.237	PLEKHB1	0.366	~amyloid_beta+(1|manifest)+cngeneson+pc_mito+brain_region+Sex	3.474	1259.000
ENSG00000171148	0.031	0.070	0.216	-0.077	TADA3	0.076	~amyloid_beta+(1|manifest)+cngeneson+pc_mito+brain_region+Sex	0.933	4294.000
ENSG00000185278	0.044	0.070	0.194	-0.055	ZBTB37	0.093	~amyloid_beta+(1|manifest)+cngeneson+pc_mito+brain_region+Sex	0.826	4551.000
ENSG00000129515	0.024	0.070	0.198	-0.058	SNX6	0.066	~amyloid_beta+(1|manifest)+cngeneson+pc_mito+brain_region+Sex	0.924	4320.000
ENSG00000134202	0.168	0.070	0.300	-0.161	GSTM3	0.224	~amyloid_beta+(1|manifest)+cngeneson+pc_mito+brain_region+Sex	1.280	3546.000
ENSG00000172354	0.119	0.070	0.261	-0.122	GNB2	0.175	~amyloid_beta+(1|manifest)+cngeneson+pc_mito+brain_region+Sex	1.086	3958.000
ENSG00000099204	0.187	0.070	0.153	-0.014	ABLIM1	0.242	~amyloid_beta+(1|manifest)+cngeneson+pc_mito+brain_region+Sex	0.516	5311.000
ENSG00000095794	0.115	0.070	0.235	-0.096	CREM	0.171	~amyloid_beta+(1|manifest)+cngeneson+pc_mito+brain_region+Sex	0.561	5206.000
ENSG00000073921	0.277	0.069	0.215	-0.076	PICALM	0.330	~amyloid_beta+(1|manifest)+cngeneson+pc_mito+brain_region+Sex	1.919	2538.000
ENSG00000111237	0.020	0.069	0.234	-0.095	VPS29	0.059	~amyloid_beta+(1|manifest)+cngeneson+pc_mito+brain_region+Sex	0.358	5638.000
ENSG00000151532	0.623	0.069	0.244	-0.105	VTI1A	0.658	~amyloid_beta+(1|manifest)+cngeneson+pc_mito+brain_region+Sex	1.066	3997.000
ENSG00000164976	0.277	0.069	0.286	-0.147	MYORG	0.330	~amyloid_beta+(1|manifest)+cngeneson+pc_mito+brain_region+Sex	1.030	4075.000
ENSG00000198911	0.069	0.069	0.423	-0.284	SREBF2	0.123	~amyloid_beta+(1|manifest)+cngeneson+pc_mito+brain_region+Sex	6.671	510.000
ENSG00000198399	0.001	0.069	0.202	-0.064	ITSN2	0.010	~amyloid_beta+(1|manifest)+cngeneson+pc_mito+brain_region+Sex	0.773	4680.000
ENSG00000160953	0.273	0.069	0.368	-0.230	PWWP3A	0.326	~amyloid_beta+(1|manifest)+cngeneson+pc_mito+brain_region+Sex	5.422	689.000
ENSG00000184949	0.041	0.069	0.257	-0.119	FAM227A	0.089	~amyloid_beta+(1|manifest)+cngeneson+pc_mito+brain_region+Sex	1.239	3618.000
ENSG00000184277	0.138	0.069	0.197	-0.059	TM2D3	0.195	~amyloid_beta+(1|manifest)+cngeneson+pc_mito+brain_region+Sex	0.423	5520.000
ENSG00000225190	0.005	0.069	0.188	-0.051	PLEKHM1	0.026	~amyloid_beta+(1|manifest)+cngeneson+pc_mito+brain_region+Sex	1.062	4003.000
ENSG00000227500	0.053	0.069	0.210	-0.072	SCAMP4	0.103	~amyloid_beta+(1|manifest)+cngeneson+pc_mito+brain_region+Sex	0.558	5217.000
ENSG00000117394	0.073	0.069	0.236	-0.098	SLC2A1	0.127	~amyloid_beta+(1|manifest)+cngeneson+pc_mito+brain_region+Sex	0.805	4617.000
ENSG00000025293	0.029	0.069	0.169	-0.031	PHF20	0.072	~amyloid_beta+(1|manifest)+cngeneson+pc_mito+brain_region+Sex	0.302	5732.000
ENSG00000105429	0.026	0.069	0.168	-0.030	MEGF8	0.068	~amyloid_beta+(1|manifest)+cngeneson+pc_mito+brain_region+Sex	1.138	3841.000
ENSG00000126226	0.020	0.068	0.163	-0.026	PCID2	0.059	~amyloid_beta+(1|manifest)+cngeneson+pc_mito+brain_region+Sex	0.164	5911.000
ENSG00000054523	0.303	0.068	0.168	-0.031	KIF1B	0.355	~amyloid_beta+(1|manifest)+cngeneson+pc_mito+brain_region+Sex	7.291	440.000
ENSG00000198431	0.409	0.068	0.348	-0.211	TXNRD1	0.456	~amyloid_beta+(1|manifest)+cngeneson+pc_mito+brain_region+Sex	3.580	1217.000
ENSG00000166164	0.021	0.068	0.133	0.003	BRD7	0.061	~amyloid_beta+(1|manifest)+cngeneson+pc_mito+brain_region+Sex	0.300	5734.000
ENSG00000180398	0.053	0.068	0.192	-0.055	MCFD2	0.103	~amyloid_beta+(1|manifest)+cngeneson+pc_mito+brain_region+Sex	1.113	3902.000
ENSG00000115839	0.060	0.068	0.232	-0.096	RAB3GAP1	0.111	~amyloid_beta+(1|manifest)+cngeneson+pc_mito+brain_region+Sex	0.422	5522.000
ENSG00000100784	0.370	0.068	0.400	-0.263	RPS6KA5	0.420	~amyloid_beta+(1|manifest)+cngeneson+pc_mito+brain_region+Sex	6.468	532.000
ENSG00000119004	0.028	0.068	0.221	-0.085	CYP20A1	0.072	~amyloid_beta+(1|manifest)+cngeneson+pc_mito+brain_region+Sex	0.685	4928.000
ENSG00000113594	0.435	0.068	0.169	-0.033	LIFR	0.481	~amyloid_beta+(1|manifest)+cngeneson+pc_mito+brain_region+Sex	5.081	759.000
ENSG00000145860	0.058	0.068	0.174	-0.038	RNF145	0.109	~amyloid_beta+(1|manifest)+cngeneson+pc_mito+brain_region+Sex	0.732	4788.000
ENSG00000142230	0.013	0.068	0.140	-0.004	SAE1	0.047	~amyloid_beta+(1|manifest)+cngeneson+pc_mito+brain_region+Sex	0.096	5956.000
ENSG00000100554	0.037	0.068	0.155	-0.019	ATP6V1D	0.083	~amyloid_beta+(1|manifest)+cngeneson+pc_mito+brain_region+Sex	0.655	4987.000
ENSG00000143486	0.015	0.068	0.139	-0.003	EIF2D	0.051	~amyloid_beta+(1|manifest)+cngeneson+pc_mito+brain_region+Sex	1.144	3828.000
ENSG00000067955	0.077	0.068	0.287	-0.151	CBFB	0.132	~amyloid_beta+(1|manifest)+cngeneson+pc_mito+brain_region+Sex	0.691	4914.000
ENSG00000198900	0.017	0.068	0.189	-0.053	TOP1	0.053	~amyloid_beta+(1|manifest)+cngeneson+pc_mito+brain_region+Sex	0.541	5257.000
ENSG00000109787	0.003	0.067	0.110	0.025	KLF3	0.019	~amyloid_beta+(1|manifest)+cngeneson+pc_mito+brain_region+Sex	0.271	5780.000
ENSG00000100220	0.005	0.067	0.121	0.014	RTCB	0.027	~amyloid_beta+(1|manifest)+cngeneson+pc_mito+brain_region+Sex	0.559	5216.000
ENSG00000109320	0.064	0.067	0.329	-0.195	NFKB1	0.116	~amyloid_beta+(1|manifest)+cngeneson+pc_mito+brain_region+Sex	2.193	2209.000
ENSG00000118260	0.040	0.067	0.198	-0.064	CREB1	0.087	~amyloid_beta+(1|manifest)+cngeneson+pc_mito+brain_region+Sex	0.892	4392.000
ENSG00000152332	0.051	0.067	0.166	-0.032	UHMK1	0.101	~amyloid_beta+(1|manifest)+cngeneson+pc_mito+brain_region+Sex	0.533	5273.000
ENSG00000130414	0.027	0.067	0.168	-0.034	NDUFA10	0.069	~amyloid_beta+(1|manifest)+cngeneson+pc_mito+brain_region+Sex	1.140	3836.000
ENSG00000138764	0.007	0.067	0.172	-0.038	CCNG2	0.032	~amyloid_beta+(1|manifest)+cngeneson+pc_mito+brain_region+Sex	0.699	4890.000
ENSG00000140718	0.142	0.067	0.140	-0.006	FTO	0.199	~amyloid_beta+(1|manifest)+cngeneson+pc_mito+brain_region+Sex	0.239	5826.000
ENSG00000067225	0.209	0.067	0.335	-0.201	PKM	0.264	~amyloid_beta+(1|manifest)+cngeneson+pc_mito+brain_region+Sex	3.042	1519.000
ENSG00000126070	0.057	0.067	0.213	-0.079	AGO3	0.108	~amyloid_beta+(1|manifest)+cngeneson+pc_mito+brain_region+Sex	2.605	1808.000
ENSG00000100567	0.053	0.067	0.187	-0.054	PSMA3	0.103	~amyloid_beta+(1|manifest)+cngeneson+pc_mito+brain_region+Sex	1.229	3644.000
ENSG00000008405	0.098	0.067	0.332	-0.199	CRY1	0.153	~amyloid_beta+(1|manifest)+cngeneson+pc_mito+brain_region+Sex	2.660	1762.000
ENSG00000065883	0.054	0.067	0.163	-0.030	CDK13	0.105	~amyloid_beta+(1|manifest)+cngeneson+pc_mito+brain_region+Sex	1.758	2740.000
ENSG00000002016	0.069	0.067	0.167	-0.034	RAD52	0.123	~amyloid_beta+(1|manifest)+cngeneson+pc_mito+brain_region+Sex	1.377	3363.000
ENSG00000152601	0.670	0.066	0.216	-0.083	MBNL1	0.701	~amyloid_beta+(1|manifest)+cngeneson+pc_mito+brain_region+Sex	4.082	1018.000
ENSG00000151422	0.158	0.066	0.146	-0.013	FER	0.213	~amyloid_beta+(1|manifest)+cngeneson+pc_mito+brain_region+Sex	1.114	3901.000
ENSG00000275832	0.076	0.066	0.261	-0.128	ARHGAP23	0.130	~amyloid_beta+(1|manifest)+cngeneson+pc_mito+brain_region+Sex	1.228	3651.000
ENSG00000175782	0.020	0.066	0.325	-0.192	SLC35E3	0.059	~amyloid_beta+(1|manifest)+cngeneson+pc_mito+brain_region+Sex	1.496	3142.000
ENSG00000133731	0.014	0.066	0.185	-0.053	IMPA1	0.047	~amyloid_beta+(1|manifest)+cngeneson+pc_mito+brain_region+Sex	0.973	4196.000
ENSG00000198000	0.007	0.066	0.140	-0.008	NOL8	0.032	~amyloid_beta+(1|manifest)+cngeneson+pc_mito+brain_region+Sex	0.886	4412.000
ENSG00000133818	0.020	0.066	0.186	-0.054	RRAS2	0.059	~amyloid_beta+(1|manifest)+cngeneson+pc_mito+brain_region+Sex	0.982	4183.000
ENSG00000102804	0.909	0.066	0.497	-0.365	TSC22D1	0.919	~amyloid_beta+(1|manifest)+cngeneson+pc_mito+brain_region+Sex	2.342	2059.000
ENSG00000089048	0.068	0.066	0.190	-0.058	ESF1	0.121	~amyloid_beta+(1|manifest)+cngeneson+pc_mito+brain_region+Sex	1.305	3498.000
ENSG00000175220	0.085	0.066	0.266	-0.135	ARHGAP1	0.140	~amyloid_beta+(1|manifest)+cngeneson+pc_mito+brain_region+Sex	3.024	1528.000
ENSG00000077235	0.048	0.066	0.198	-0.067	GTF3C1	0.097	~amyloid_beta+(1|manifest)+cngeneson+pc_mito+brain_region+Sex	1.268	3565.000
ENSG00000176783	0.022	0.065	0.277	-0.146	RUFY1	0.063	~amyloid_beta+(1|manifest)+cngeneson+pc_mito+brain_region+Sex	2.662	1761.000
ENSG00000118496	0.001	0.065	0.126	0.004	FBXO30	0.012	~amyloid_beta+(1|manifest)+cngeneson+pc_mito+brain_region+Sex	0.285	5753.000
ENSG00000166963	0.015	0.065	0.225	-0.094	MAP1A	0.051	~amyloid_beta+(1|manifest)+cngeneson+pc_mito+brain_region+Sex	1.396	3329.000
ENSG00000179151	0.025	0.065	0.146	-0.016	EDC3	0.067	~amyloid_beta+(1|manifest)+cngeneson+pc_mito+brain_region+Sex	0.545	5246.000
ENSG00000130202	0.024	0.065	0.171	-0.041	NECTIN2	0.066	~amyloid_beta+(1|manifest)+cngeneson+pc_mito+brain_region+Sex	0.747	4748.000
ENSG00000168092	0.050	0.065	0.159	-0.029	PAFAH1B2	0.100	~amyloid_beta+(1|manifest)+cngeneson+pc_mito+brain_region+Sex	0.847	4507.000
ENSG00000135951	0.138	0.065	0.227	-0.098	TSGA10	0.194	~amyloid_beta+(1|manifest)+cngeneson+pc_mito+brain_region+Sex	1.860	2615.000
ENSG00000128159	0.137	0.065	0.337	-0.207	TUBGCP6	0.194	~amyloid_beta+(1|manifest)+cngeneson+pc_mito+brain_region+Sex	3.937	1070.000
ENSG00000165995	0.772	0.065	0.361	-0.232	CACNB2	0.794	~amyloid_beta+(1|manifest)+cngeneson+pc_mito+brain_region+Sex	6.863	484.000
ENSG00000242247	0.011	0.065	0.208	-0.078	ARFGAP3	0.042	~amyloid_beta+(1|manifest)+cngeneson+pc_mito+brain_region+Sex	1.324	3454.000
ENSG00000133422	0.009	0.065	0.137	-0.007	MORC2	0.037	~amyloid_beta+(1|manifest)+cngeneson+pc_mito+brain_region+Sex	0.457	5445.000
ENSG00000176986	0.046	0.064	0.152	-0.023	SEC24C	0.095	~amyloid_beta+(1|manifest)+cngeneson+pc_mito+brain_region+Sex	0.941	4274.000
ENSG00000170502	0.005	0.064	0.165	-0.036	NUDT9	0.027	~amyloid_beta+(1|manifest)+cngeneson+pc_mito+brain_region+Sex	0.033	5976.000
ENSG00000092020	0.077	0.064	0.266	-0.137	PPP2R3C	0.132	~amyloid_beta+(1|manifest)+cngeneson+pc_mito+brain_region+Sex	2.513	1885.000
ENSG00000160208	0.017	0.064	0.135	-0.006	RRP1B	0.053	~amyloid_beta+(1|manifest)+cngeneson+pc_mito+brain_region+Sex	0.598	5136.000
ENSG00000170456	0.048	0.064	0.179	-0.051	DENND5B	0.098	~amyloid_beta+(1|manifest)+cngeneson+pc_mito+brain_region+Sex	1.171	3764.000
ENSG00000111540	0.027	0.064	0.133	-0.005	RAB5B	0.069	~amyloid_beta+(1|manifest)+cngeneson+pc_mito+brain_region+Sex	0.439	5488.000
ENSG00000122068	0.096	0.064	0.170	-0.042	FYTTD1	0.151	~amyloid_beta+(1|manifest)+cngeneson+pc_mito+brain_region+Sex	0.450	5455.000
ENSG00000118322	0.136	0.064	0.286	-0.158	ATP10B	0.192	~amyloid_beta+(1|manifest)+cngeneson+pc_mito+brain_region+Sex	1.607	2974.000
ENSG00000112992	0.019	0.064	0.163	-0.036	NNT	0.057	~amyloid_beta+(1|manifest)+cngeneson+pc_mito+brain_region+Sex	1.646	2912.000
ENSG00000162631	0.409	0.064	0.401	-0.273	NTNG1	0.456	~amyloid_beta+(1|manifest)+cngeneson+pc_mito+brain_region+Sex	1.488	3161.000
ENSG00000102054	0.026	0.064	0.173	-0.045	RBBP7	0.068	~amyloid_beta+(1|manifest)+cngeneson+pc_mito+brain_region+Sex	0.285	5752.000
ENSG00000132535	0.018	0.064	0.131	-0.004	DLG4	0.055	~amyloid_beta+(1|manifest)+cngeneson+pc_mito+brain_region+Sex	0.801	4629.000
ENSG00000163681	0.197	0.064	0.212	-0.084	SLMAP	0.251	~amyloid_beta+(1|manifest)+cngeneson+pc_mito+brain_region+Sex	2.477	1917.000
ENSG00000241878	0.033	0.064	0.152	-0.025	PISD	0.078	~amyloid_beta+(1|manifest)+cngeneson+pc_mito+brain_region+Sex	0.449	5458.000
ENSG00000119689	0.019	0.064	0.134	-0.006	DLST	0.057	~amyloid_beta+(1|manifest)+cngeneson+pc_mito+brain_region+Sex	0.541	5258.000
ENSG00000198860	0.169	0.064	0.296	-0.169	TSEN15	0.224	~amyloid_beta+(1|manifest)+cngeneson+pc_mito+brain_region+Sex	0.863	4456.000
ENSG00000140553	0.032	0.064	0.221	-0.093	UNC45A	0.077	~amyloid_beta+(1|manifest)+cngeneson+pc_mito+brain_region+Sex	1.727	2786.000
ENSG00000101940	0.141	0.064	0.247	-0.120	WDR13	0.198	~amyloid_beta+(1|manifest)+cngeneson+pc_mito+brain_region+Sex	1.898	2554.000
ENSG00000155744	0.024	0.064	0.181	-0.054	FAM126B	0.066	~amyloid_beta+(1|manifest)+cngeneson+pc_mito+brain_region+Sex	0.623	5076.000
ENSG00000160633	0.043	0.064	0.187	-0.060	SAFB	0.091	~amyloid_beta+(1|manifest)+cngeneson+pc_mito+brain_region+Sex	1.579	3013.000
ENSG00000008277	0.013	0.064	0.234	-0.107	ADAM22	0.046	~amyloid_beta+(1|manifest)+cngeneson+pc_mito+brain_region+Sex	1.589	3001.000
ENSG00000069667	0.341	0.064	0.221	-0.094	RORA	0.392	~amyloid_beta+(1|manifest)+cngeneson+pc_mito+brain_region+Sex	19.616	55.000
ENSG00000163947	0.983	0.064	1.260	-1.133	ARHGEF3	0.984	~amyloid_beta+(1|manifest)+cngeneson+pc_mito+brain_region+Sex	32.542	18.000
ENSG00000032219	0.027	0.063	0.204	-0.077	ARID4A	0.070	~amyloid_beta+(1|manifest)+cngeneson+pc_mito+brain_region+Sex	0.885	4414.000
ENSG00000171466	0.014	0.063	0.143	-0.017	ZNF562	0.048	~amyloid_beta+(1|manifest)+cngeneson+pc_mito+brain_region+Sex	0.742	4765.000
ENSG00000154144	0.043	0.063	0.190	-0.064	TBRG1	0.091	~amyloid_beta+(1|manifest)+cngeneson+pc_mito+brain_region+Sex	1.087	3954.000
ENSG00000068697	0.261	0.063	0.296	-0.170	LAPTM4A	0.315	~amyloid_beta+(1|manifest)+cngeneson+pc_mito+brain_region+Sex	1.412	3305.000
ENSG00000186409	0.104	0.063	0.265	-0.139	CCDC30	0.160	~amyloid_beta+(1|manifest)+cngeneson+pc_mito+brain_region+Sex	1.121	3886.000
ENSG00000149196	0.036	0.063	0.196	-0.070	HIKESHI	0.082	~amyloid_beta+(1|manifest)+cngeneson+pc_mito+brain_region+Sex	0.191	5885.000
ENSG00000163629	0.852	0.063	0.287	-0.161	PTPN13	0.867	~amyloid_beta+(1|manifest)+cngeneson+pc_mito+brain_region+Sex	5.785	627.000
ENSG00000105053	0.017	0.063	0.140	-0.014	VRK3	0.054	~amyloid_beta+(1|manifest)+cngeneson+pc_mito+brain_region+Sex	0.699	4894.000
ENSG00000166333	0.132	0.063	0.305	-0.180	ILK	0.188	~amyloid_beta+(1|manifest)+cngeneson+pc_mito+brain_region+Sex	1.847	2629.000
ENSG00000084676	0.178	0.063	0.138	-0.013	NCOA1	0.233	~amyloid_beta+(1|manifest)+cngeneson+pc_mito+brain_region+Sex	4.524	888.000
ENSG00000197020	0.058	0.063	0.249	-0.124	ZNF100	0.109	~amyloid_beta+(1|manifest)+cngeneson+pc_mito+brain_region+Sex	2.006	2420.000
ENSG00000205726	0.648	0.063	0.193	-0.067	ITSN1	0.681	~amyloid_beta+(1|manifest)+cngeneson+pc_mito+brain_region+Sex	5.119	747.000
ENSG00000160058	0.062	0.062	0.209	-0.084	BSDC1	0.115	~amyloid_beta+(1|manifest)+cngeneson+pc_mito+brain_region+Sex	1.732	2778.000
ENSG00000106524	0.002	0.062	0.120	0.005	ANKMY2	0.017	~amyloid_beta+(1|manifest)+cngeneson+pc_mito+brain_region+Sex	0.214	5855.000
ENSG00000105186	0.025	0.062	0.182	-0.058	ANKRD27	0.067	~amyloid_beta+(1|manifest)+cngeneson+pc_mito+brain_region+Sex	0.857	4473.000
ENSG00000154229	0.015	0.062	0.198	-0.074	PRKCA	0.051	~amyloid_beta+(1|manifest)+cngeneson+pc_mito+brain_region+Sex	15.487	94.000
ENSG00000109066	0.041	0.062	0.133	-0.009	TMEM104	0.088	~amyloid_beta+(1|manifest)+cngeneson+pc_mito+brain_region+Sex	0.602	5122.000
ENSG00000116459	0.038	0.062	0.141	-0.017	ATP5PB	0.085	~amyloid_beta+(1|manifest)+cngeneson+pc_mito+brain_region+Sex	0.327	5682.000
ENSG00000110344	0.029	0.062	0.168	-0.044	UBE4A	0.072	~amyloid_beta+(1|manifest)+cngeneson+pc_mito+brain_region+Sex	0.746	4751.000
ENSG00000151693	0.381	0.062	0.252	-0.129	ASAP2	0.430	~amyloid_beta+(1|manifest)+cngeneson+pc_mito+brain_region+Sex	2.305	2098.000
ENSG00000234545	0.024	0.062	0.133	-0.009	FAM133B	0.066	~amyloid_beta+(1|manifest)+cngeneson+pc_mito+brain_region+Sex	0.611	5106.000
ENSG00000019991	0.311	0.062	0.528	-0.405	HGF	0.364	~amyloid_beta+(1|manifest)+cngeneson+pc_mito+brain_region+Sex	12.352	165.000
ENSG00000126261	0.011	0.062	0.195	-0.072	UBA2	0.042	~amyloid_beta+(1|manifest)+cngeneson+pc_mito+brain_region+Sex	1.176	3756.000
ENSG00000133789	0.137	0.061	0.255	-0.133	SWAP70	0.193	~amyloid_beta+(1|manifest)+cngeneson+pc_mito+brain_region+Sex	4.336	936.000
ENSG00000048828	0.198	0.061	0.207	-0.085	FAM120A	0.252	~amyloid_beta+(1|manifest)+cngeneson+pc_mito+brain_region+Sex	2.381	2013.000
ENSG00000144815	0.003	0.061	0.124	-0.001	NXPE3	0.019	~amyloid_beta+(1|manifest)+cngeneson+pc_mito+brain_region+Sex	0.095	5958.000
ENSG00000088888	0.030	0.061	0.205	-0.082	MAVS	0.074	~amyloid_beta+(1|manifest)+cngeneson+pc_mito+brain_region+Sex	1.202	3699.000
ENSG00000167685	0.045	0.061	0.217	-0.094	ZNF444	0.094	~amyloid_beta+(1|manifest)+cngeneson+pc_mito+brain_region+Sex	1.949	2495.000
ENSG00000022267	0.051	0.061	0.237	-0.115	FHL1	0.101	~amyloid_beta+(1|manifest)+cngeneson+pc_mito+brain_region+Sex	1.010	4118.000
ENSG00000166398	0.046	0.061	0.174	-0.052	KIAA0355	0.095	~amyloid_beta+(1|manifest)+cngeneson+pc_mito+brain_region+Sex	1.151	3812.000
ENSG00000172732	0.057	0.061	0.227	-0.105	MUS81	0.108	~amyloid_beta+(1|manifest)+cngeneson+pc_mito+brain_region+Sex	1.161	3787.000
ENSG00000004799	0.854	0.061	0.480	-0.358	PDK4	0.868	~amyloid_beta+(1|manifest)+cngeneson+pc_mito+brain_region+Sex	4.971	783.000
ENSG00000139910	0.426	0.061	0.246	-0.124	NOVA1	0.472	~amyloid_beta+(1|manifest)+cngeneson+pc_mito+brain_region+Sex	3.127	1465.000
ENSG00000104731	0.012	0.061	0.192	-0.071	KLHDC4	0.044	~amyloid_beta+(1|manifest)+cngeneson+pc_mito+brain_region+Sex	1.336	3437.000
ENSG00000104320	0.024	0.060	0.176	-0.056	NBN	0.066	~amyloid_beta+(1|manifest)+cngeneson+pc_mito+brain_region+Sex	0.954	4243.000
ENSG00000136828	0.126	0.060	0.251	-0.131	RALGPS1	0.182	~amyloid_beta+(1|manifest)+cngeneson+pc_mito+brain_region+Sex	1.283	3538.000
ENSG00000146426	0.578	0.060	0.414	-0.294	TIAM2	0.615	~amyloid_beta+(1|manifest)+cngeneson+pc_mito+brain_region+Sex	3.335	1349.000
ENSG00000118473	0.717	0.060	0.215	-0.095	SGIP1	0.744	~amyloid_beta+(1|manifest)+cngeneson+pc_mito+brain_region+Sex	3.442	1280.000
ENSG00000135766	0.037	0.060	0.188	-0.069	EGLN1	0.084	~amyloid_beta+(1|manifest)+cngeneson+pc_mito+brain_region+Sex	0.432	5502.000
ENSG00000213639	0.039	0.060	0.309	-0.190	PPP1CB	0.087	~amyloid_beta+(1|manifest)+cngeneson+pc_mito+brain_region+Sex	2.340	2061.000
ENSG00000119929	0.003	0.060	0.111	0.009	CUTC	0.021	~amyloid_beta+(1|manifest)+cngeneson+pc_mito+brain_region+Sex	0.487	5391.000
ENSG00000130703	0.025	0.060	0.169	-0.050	OSBPL2	0.067	~amyloid_beta+(1|manifest)+cngeneson+pc_mito+brain_region+Sex	1.065	3999.000
ENSG00000008441	0.438	0.060	0.363	-0.243	NFIX	0.484	~amyloid_beta+(1|manifest)+cngeneson+pc_mito+brain_region+Sex	4.396	918.000
ENSG00000278540	0.098	0.060	0.181	-0.062	ACACA	0.153	~amyloid_beta+(1|manifest)+cngeneson+pc_mito+brain_region+Sex	1.279	3548.000
ENSG00000181722	0.146	0.060	0.188	-0.068	ZBTB20	0.202	~amyloid_beta+(1|manifest)+cngeneson+pc_mito+brain_region+Sex	15.843	90.000
ENSG00000196961	0.074	0.059	0.173	-0.054	AP2A1	0.127	~amyloid_beta+(1|manifest)+cngeneson+pc_mito+brain_region+Sex	1.075	3977.000
ENSG00000160323	0.105	0.059	0.184	-0.065	[illegible]	0.161	~amyloid_beta+(1|manifest)+cngeneson+pc_mito+brain_region+Sex	2.075	2341.000
ENSG00000143799	0.035	0.059	0.157	-0.039	PARP1	0.081	~amyloid_beta+(1|manifest)+cngeneson+pc_mito+brain_region+Sex	0.773	4679.000
ENSG00000124532	0.002	0.059	0.116	0.002	MRS2	0.015	~amyloid_beta+(1|manifest)+cngeneson+pc_mito+brain_region+Sex	0.674	4957.000
ENSG00000135338	0.036	0.059	0.204	-0.086	LCA5	0.082	~amyloid_beta+(1|manifest)+cngeneson+pc_mito+brain_region+Sex	1.673	2866.000
ENSG00000136436	0.014	0.059	0.220	-0.101	CALCOCO2	0.048	~amyloid_beta+(1|manifest)+cngeneson+pc_mito+brain_region+Sex	0.618	5093.000
ENSG00000088247	0.079	0.059	0.187	-0.069	KHSRP	0.133	~amyloid_beta+(1|manifest)+cngeneson+pc_mito+brain_region+Sex	1.077	3971.000
ENSG00000112304	0.004	0.059	0.112	0.006	ACOT13	0.023	~amyloid_beta+(1|manifest)+cngeneson+pc_mito+brain_region+Sex	0.413	5539.000
ENSG00000104517	0.392	0.059	0.146	-0.029	UBR5	0.441	~amyloid_beta+(1|manifest)+cngeneson+pc_mito+brain_region+Sex	1.877	2593.000
ENSG00000128016	0.817	0.059	0.719	-0.601	ZFP36	0.835	~amyloid_beta+(1|manifest)+cngeneson+pc_mito+brain_region+Sex	3.993	1042.000
ENSG00000134453	0.055	0.059	0.163	-0.046	RBM17	0.106	~amyloid_beta+(1|manifest)+cngeneson+pc_mito+brain_region+Sex	0.303	5727.000
ENSG00000108799	0.021	0.059	0.241	-0.124	EZH1	0.061	~amyloid_beta+(1|manifest)+cngeneson+pc_mito+brain_region+Sex	1.970	2468.000
ENSG00000164830	0.274	0.059	0.278	-0.161	OXR1	0.327	~amyloid_beta+(1|manifest)+cngeneson+pc_mito+brain_region+Sex	2.821	1652.000
ENSG00000070831	0.039	0.058	0.164	-0.047	CDC42	0.086	~amyloid_beta+(1|manifest)+cngeneson+pc_mito+brain_region+Sex	0.532	5278.000
ENSG00000070785	0.011	0.058	0.141	-0.024	EIF2B3	0.043	~amyloid_beta+(1|manifest)+cngeneson+pc_mito+brain_region+Sex	0.382	5595.000
ENSG00000035403	0.566	0.058	0.431	-0.315	VCL	0.605	~amyloid_beta+(1|manifest)+cngeneson+pc_mito+brain_region+Sex	8.706	330.000
ENSG00000127220	0.193	0.058	0.304	-0.189	ABHD8	0.248	~amyloid_beta+(1|manifest)+cngeneson+pc_mito+brain_region+Sex	3.734	1148.000
ENSG00000197562	0.015	0.058	0.209	-0.094	RAB40C	0.051	~amyloid_beta+(1|manifest)+cngeneson+pc_mito+brain_region+Sex	1.230	3639.000
ENSG00000155111	0.059	0.057	0.215	-0.100	CDK19	0.111	~amyloid_beta+(1|manifest)+cngeneson+pc_mito+brain_region+Sex	0.225	5839.000
ENSG00000144134	0.033	0.057	0.254	-0.140	RABL2A	0.078	~amyloid_beta+(1|manifest)+cngeneson+pc_mito+brain_region+Sex	3.999	1041.000
ENSG00000117419	0.554	0.057	0.260	-0.146	ERI3	0.594	~amyloid_beta+(1|manifest)+cngeneson+pc_mito+brain_region+Sex	4.794	827.000
ENSG00000128294	0.115	0.057	0.166	-0.052	TPST2	0.171	~amyloid_beta+(1|manifest)+cngeneson+pc_mito+brain_region+Sex	0.731	4792.000
ENSG00000022840	0.021	0.057	0.146	-0.032	RNF10	0.061	~amyloid_beta+(1|manifest)+cngeneson+pc_mito+brain_region+Sex	0.626	5070.000
ENSG00000166224	0.153	0.057	0.200	-0.086	SGPL1	0.208	~amyloid_beta+(1|manifest)+cngeneson+pc_mito+brain_region+Sex	0.526	5295.000
ENSG00000124767	0.033	0.057	0.168	-0.054	GLO1	0.079	~amyloid_beta+(1|manifest)+cngeneson+pc_mito+brain_region+Sex	0.825	4554.000
ENSG00000171105	0.047	0.057	0.326	-0.212	INSR	0.096	~amyloid_beta+(1|manifest)+cngeneson+pc_mito+brain_region+Sex	0.861	4464.000
ENSG00000010017	0.315	0.057	0.308	-0.194	RANBP9	0.367	~amyloid_beta+(1|manifest)+cngeneson+pc_mito+brain_region+Sex	2.894	1608.000
ENSG00000096063	0.009	0.057	0.138	-0.025	SRPK1	0.038	~amyloid_beta+(1|manifest)+cngeneson+pc_mito+brain_region+Sex	0.612	5104.000
ENSG00000169499	0.043	0.057	0.143	-0.030	PLEKHA2	0.091	~amyloid_beta+(1|manifest)+cngeneson+pc_mito+brain_region+Sex	0.432	5503.000
ENSG00000185122	0.033	0.057	0.143	-0.030	HSF1	0.078	~amyloid_beta+(1|manifest)+cngeneson+pc_mito+brain_region+Sex	0.646	5012.000
ENSG00000114316	0.012	0.057	0.203	-0.090	USP4	0.043	~amyloid_beta+(1|manifest)+cngeneson+pc_mito+brain_region+Sex	1.310	3482.000
ENSG00000131236	0.044	0.057	0.143	-0.029	CAP1	0.092	~amyloid_beta+(1|manifest)+cngeneson+pc_mito+brain_region+Sex	0.464	5430.000
ENSG00000144283	0.030	0.056	0.202	-0.089	PKP4	0.075	~amyloid_beta+(1|manifest)+cngeneson+pc_mito+brain_region+Sex	1.235	3623.000
ENSG00000137842	0.012	0.056	0.196	-0.083	TMEM62	0.044	~amyloid_beta+(1|manifest)+cngeneson+pc_mito+brain_region+Sex	1.191	3721.000
ENSG00000143889	0.019	0.056	0.144	-0.032	HNRNPLL	0.058	~amyloid_beta+(1|manifest)+cngeneson+pc_mito+brain_region+Sex	0.495	5375.000
ENSG00000158985	0.027	0.056	0.151	-0.039	CDC42SE2	0.069	~amyloid_beta+(1|manifest)+cngeneson+pc_mito+brain_region+Sex	0.817	4580.000
ENSG00000077684	0.076	0.056	0.239	-0.127	JADE1	0.131	~amyloid_beta+(1|manifest)+cngeneson+pc_mito+brain_region+Sex	1.015	4106.000
ENSG00000168522	0.017	0.056	0.128	-0.016	FNTA	0.053	~amyloid_beta+(1|manifest)+cngeneson+pc_mito+brain_region+Sex	0.294	5740.000
ENSG00000117020	0.530	0.056	0.167	-0.055	AKT3	0.572	~amyloid_beta+(1|manifest)+cngeneson+pc_mito+brain_region+Sex	0.540	5259.000
ENSG00000018236	0.730	0.056	0.242	-0.130	CNTN1	0.757	~amyloid_beta+(1|manifest)+cngeneson+pc_mito+brain_region+Sex	11.834	184.000
ENSG00000113161	0.024	0.056	0.157	-0.045	HMGCR	0.066	~amyloid_beta+(1|manifest)+cngeneson+pc_mito+brain_region+Sex	1.231	3636.000
ENSG00000099139	0.751	0.056	0.213	-0.101	PCSK5	0.776	~amyloid_beta+(1|manifest)+cngeneson+pc_mito+brain_region+Sex	2.798	1668.000
ENSG00000003393	0.013	0.056	0.142	-0.030	ALS2	0.047	~amyloid_beta+(1|manifest)+cngeneson+pc_mito+brain_region+Sex	1.056	4010.000
ENSG00000117758	0.080	0.056	0.145	-0.033	STX12	0.135	~amyloid_beta+(1|manifest)+cngeneson+pc_mito+brain_region+Sex	0.912	4345.000
ENSG00000185721	0.010	0.056	0.114	-0.002	DRG1	0.040	~amyloid_beta+(1|manifest)+cngeneson+pc_mito+brain_region+Sex	0.701	4882.000
ENSG00000182220	0.122	0.056	0.220	-0.109	ATP6AP2	0.179	~amyloid_beta+(1|manifest)+cngeneson+pc_mito+brain_region+Sex	0.561	5207.000
ENSG00000177200	0.657	0.055	0.191	-0.081	CHD9	0.689	~amyloid_beta+(1|manifest)+cngeneson+pc_mito+brain_region+Sex	5.649	656.000
ENSG00000143761	0.010	0.055	0.152	-0.041	ARF1	0.040	~amyloid_beta+(1|manifest)+cngeneson+pc_mito+brain_region+Sex	0.503	5348.000
ENSG00000124535	0.008	0.055	0.119	-0.009	WRNIP1	0.035	~amyloid_beta+(1|manifest)+cngeneson+pc_mito+brain_region+Sex	0.656	4985.000
ENSG00000105856	0.039	0.055	0.219	-0.109	HBP1	0.086	~amyloid_beta+(1|manifest)+cngeneson+pc_mito+brain_region+Sex	0.912	4347.000
ENSG00000070540	0.023	0.055	0.162	-0.053	WIPI1	0.064	~amyloid_beta+(1|manifest)+cngeneson+pc_mito+brain_region+Sex	0.957	4232.000
ENSG00000170425	0.034	0.055	0.248	-0.138	ADORA2B	0.080	~amyloid_beta+(1|manifest)+cngeneson+pc_mito+brain_region+Sex	2.177	2227.000
ENSG00000140992	0.056	0.055	0.196	-0.086	PDPK1	0.106	~amyloid_beta+(1|manifest)+cngeneson+pc_mito+brain_region+Sex	1.880	2590.000
ENSG00000272886	0.008	0.055	0.185	-0.075	DCP1A	0.036	~amyloid_beta+(1|manifest)+cngeneson+pc_mito+brain_region+Sex	1.136	3846.000
ENSG00000084073	0.009	0.055	0.112	-0.002	ZMPSTE24	0.039	~amyloid_beta+(1|manifest)+cngeneson+pc_mito+brain_region+Sex	0.284	5756.000
ENSG00000165752	0.035	0.055	0.157	-0.047	STK32C	0.080	~amyloid_beta+(1|manifest)+cngeneson+pc_mito+brain_region+Sex	0.876	4425.000
ENSG00000180370	0.003	0.055	0.167	-0.058	PAK2	0.018	~amyloid_beta+(1|manifest)+cngeneson+pc_mito+brain_region+Sex	1.000	4141.000
ENSG00000143164	0.132	0.054	0.296	-0.187	DCAF6	0.188	~amyloid_beta+(1|manifest)+cngeneson+pc_mito+brain_region+Sex	1.776	2720.000
ENSG00000120992	0.003	0.054	0.097	0.012	LYPLA1	0.019	~amyloid_beta+(1|manifest)+cngeneson+pc_mito+brain_region+Sex	0.433	5500.000
ENSG00000171766	0.166	0.054	0.347	-0.238	GATM	0.222	~amyloid_beta+(1|manifest)+cngeneson+pc_mito+brain_region+Sex	1.126	3870.000
ENSG00000164754	0.036	0.054	0.156	-0.047	RAD21	0.082	~amyloid_beta+(1|manifest)+cngeneson+pc_mito+brain_region+Sex	0.182	5898.000
ENSG00000124795	0.028	0.054	0.178	-0.069	DEK	0.072	~amyloid_beta+(1|manifest)+cngeneson+pc_mito+brain_region+Sex	0.383	5592.000
ENSG00000170142	0.172	0.054	0.228	-0.120	UBE2E1	0.227	~amyloid_beta+(1|manifest)+cngeneson+pc_mito+brain_region+Sex	1.509	3127.000
ENSG00000137824	0.029	0.054	0.162	-0.054	RMDN3	0.072	~amyloid_beta+(1|manifest)+cngeneson+pc_mito+brain_region+Sex	1.031	4071.000
ENSG00000166025	0.047	0.054	0.152	-0.044	AMOTL1	0.096	~amyloid_beta+(1|manifest)+cngeneson+pc_mito+brain_region+Sex	0.494	5377.000
ENSG00000128050	0.051	0.054	0.139	-0.031	PAICS	0.101	~amyloid_beta+(1|manifest)+cngeneson+pc_mito+brain_region+Sex	0.595	5142.000
ENSG00000141447	0.485	0.054	0.327	-0.219	OSBPL1A	0.529	~amyloid_beta+(1|manifest)+cngeneson+pc_mito+brain_region+Sex	1.853	2620.000
ENSG00000163755	0.030	0.054	0.198	-0.090	HPS3	0.074	~amyloid_beta+(1|manifest)+cngeneson+pc_mito+brain_region+Sex	0.087	5963.000
ENSG00000049323	0.395	0.054	0.505	-0.398	LTBP1	0.444	~amyloid_beta+(1|manifest)+cngeneson+pc_mito+brain_region+Sex	8.089	373.000
ENSG00000177614	0.102	0.054	0.180	-0.073	PGBD5	0.157	~amyloid_beta+(1|manifest)+cngeneson+pc_mito+brain_region+Sex	1.133	3857.000
ENSG00000128534	0.019	0.053	0.105	0.002	LSM8	0.058	~amyloid_beta+(1|manifest)+cngeneson+pc_mito+brain_region+Sex	0.325	5686.000
ENSG00000100307	0.037	0.053	0.171	-0.064	CBX7	0.083	~amyloid_beta+(1|manifest)+cngeneson+pc_mito+brain_region+Sex	0.827	4545.000
ENSG00000133059	0.048	0.053	0.167	-0.060	DSTYK	0.098	~amyloid_beta+(1|manifest)+cngeneson+pc_mito+brain_region+Sex	1.030	4078.000
ENSG00000113851	0.086	0.053	0.137	-0.030	CRBN	0.141	~amyloid_beta+(1|manifest)+cngeneson+pc_mito+brain_region+Sex	0.581	5168.000
ENSG00000125503	0.049	0.053	0.194	-0.087	PPP1R12C	0.099	~amyloid_beta+(1|manifest)+cngeneson+pc_mito+brain_region+Sex	1.724	2790.000
ENSG00000115306	0.656	0.053	0.166	-0.060	SPTBN1	0.688	~amyloid_beta+(1|manifest)+cngeneson+pc_mito+brain_region+Sex	2.506	1894.000
ENSG00000124201	0.106	0.053	0.204	-0.098	ZNFX1	0.162	~amyloid_beta+(1|manifest)+cngeneson+pc_mito+brain_region+Sex	1.798	2701.000
ENSG00000163964	0.005	0.053	0.109	-0.003	PIGX	0.027	~amyloid_beta+(1|manifest)+cngeneson+pc_mito+brain_region+Sex	0.436	5492.000
ENSG00000205339	0.021	0.053	0.219	-0.114	IPO7	0.061	~amyloid_beta+(1|manifest)+cngeneson+pc_mito+brain_region+Sex	2.408	1976.000
ENSG00000091436	0.108	0.053	0.325	-0.220	MAP3K20	0.163	~amyloid_beta+(1|manifest)+cngeneson+pc_mito+brain_region+Sex	2.032	2392.000
ENSG00000168096	0.093	0.053	0.157	-0.052	ANKS3	0.148	~amyloid_beta+(1|manifest)+cngeneson+pc_mito+brain_region+Sex	1.183	3739.000
ENSG00000165819	0.087	0.052	0.172	-0.067	METTL3	0.142	~amyloid_beta+(1|manifest)+cngeneson+pc_mito+brain_region+Sex	1.539	3068.000
ENSG00000124104	0.042	0.052	0.233	-0.129	SNX21	0.089	~amyloid_beta+(1|manifest)+cngeneson+pc_mito+brain_region+Sex	2.161	2247.000
ENSG00000108559	0.027	0.052	0.109	-0.005	NUP88	0.069	~amyloid_beta+(1|manifest)+cngeneson+pc_mito+brain_region+Sex	0.505	5337.000
ENSG00000177479	0.158	0.052	0.153	-0.048	ARIH2	0.213	~amyloid_beta+(1|manifest)+cngeneson+pc_mito+brain_region+Sex	1.551	3048.000
ENSG00000149932	0.080	0.052	0.220	-0.115	TMEM219	0.134	~amyloid_beta+(1|manifest)+cngeneson+pc_mito+brain_region+Sex	1.563	3034.000
ENSG00000178567	0.101	0.052	0.185	-0.080	EPM2AIP1	0.156	~amyloid_beta+(1|manifest)+cngeneson+pc_mito+brain_region+Sex	1.272	3560.000
ENSG00000129518	0.030	0.052	0.145	-0.041	EAPP	0.075	~amyloid_beta+(1|manifest)+cngeneson+pc_mito+brain_region+Sex	0.568	5188.000
ENSG00000079739	0.071	0.052	0.251	-0.147	PGM1	0.125	~amyloid_beta+(1|manifest)+cngeneson+pc_mito+brain_region+Sex	1.202	3701.000
ENSG00000137710	0.289	0.052	0.344	-0.240	RDX	0.341	~amyloid_beta+(1|manifest)+cngeneson+pc_mito+brain_region+Sex	4.564	873.000
ENSG00000154134	0.493	0.052	0.545	-0.442	ROBO3	0.537	~amyloid_beta+(1|manifest)+cngeneson+pc_mito+brain_region+Sex	7.988	376.000
ENSG00000170310	0.032	0.052	0.181	-0.078	STX8	0.076	~amyloid_beta+(1|manifest)+cngeneson+pc_mito+brain_region+Sex	0.767	4699.000
ENSG00000139842	0.017	0.052	0.269	-0.166	CUL4A	0.054	~amyloid_beta+(1|manifest)+cngeneson+pc_mito+brain_region+Sex	2.559	1848.000
ENSG00000261609	0.432	0.052	0.236	-0.133	GAN	0.478	~amyloid_beta+(1|manifest)+cngeneson+pc_mito+brain_region+Sex	2.589	1820.000
ENSG00000169155	0.156	0.051	0.189	-0.086	ZBTB43	0.211	~amyloid_beta+(1|manifest)+cngeneson+pc_mito+brain_region+Sex	1.454	3218.000
ENSG00000104756	0.016	0.051	0.146	-0.043	KCTD9	0.052	~amyloid_beta+(1|manifest)+cngeneson+pc_mito+brain_region+Sex	0.800	4632.000
ENSG00000118579	0.021	0.051	0.145	-0.042	MED28	0.061	~amyloid_beta+(1|manifest)+cngeneson+pc_mito+brain_region+Sex	0.319	5695.000
ENSG00000140740	0.187	0.051	0.273	-0.171	UQCRC2	0.241	~amyloid_beta+(1|manifest)+cngeneson+pc_mito+brain_region+Sex	2.823	1650.000
ENSG00000198301	0.006	0.051	0.101	0.002	SDAD1	0.030	~amyloid_beta+(1|manifest)+cngeneson+pc_mito+brain_region+Sex	0.408	5551.000
ENSG00000154174	0.002	0.051	0.090	0.012	TOMM70	0.017	~amyloid_beta+(1|manifest)+cngeneson+pc_mito+brain_region+Sex	0.308	5717.000
ENSG00000162236	0.040	0.051	0.142	-0.040	STX5	0.088	~amyloid_beta+(1|manifest)+cngeneson+pc_mito+brain_region+Sex	0.443	5477.000
ENSG00000102024	0.054	0.051	0.180	-0.078	PLS3	0.105	~amyloid_beta+(1|manifest)+cngeneson+pc_mito+brain_region+Sex	0.978	4189.000
ENSG00000214113	0.177	0.051	0.175	-0.072	LYRM4	0.231	~amyloid_beta+(1|manifest)+cngeneson+pc_mito+brain_region+Sex	0.662	4974.000
ENSG00000243147	0.157	0.051	0.161	-0.059	MRPL33	0.212	~amyloid_beta+(1|manifest)+cngeneson+pc_mito+brain_region+Sex	0.472	5415.000
ENSG00000114867	0.035	0.051	0.219	-0.117	EIF4G1	0.081	~amyloid_beta+(1|manifest)+cngeneson+pc_mito+brain_region+Sex	1.624	2948.000
ENSG00000172339	0.117	0.051	0.278	-0.177	ALG14	0.173	~amyloid_beta+(1|manifest)+cngeneson+pc_mito+brain_region+Sex	3.013	1535.000
ENSG00000140853	0.083	0.051	0.209	-0.107	NLRC5	0.138	~amyloid_beta+(1|manifest)+cngeneson+pc_mito+brain_region+Sex	1.305	3495.000
ENSG00000055130	0.020	0.051	0.144	-0.043	CUL1	0.059	~amyloid_beta+(1|manifest)+cngeneson+pc_mito+brain_region+Sex	0.829	4543.000
ENSG00000147027	0.436	0.051	0.413	-0.311	TMEM47	0.482	~amyloid_beta+(1|manifest)+cngeneson+pc_mito+brain_region+Sex	2.445	1938.000
ENSG00000172086	0.005	0.051	0.154	-0.053	KRCC1	0.025	~amyloid_beta+(1|manifest)+cngeneson+pc_mito+brain_region+Sex	0.650	4999.000
ENSG00000133884	0.033	0.050	0.124	-0.023	DPF2	0.078	~amyloid_beta+(1|manifest)+cngeneson+pc_mito+brain_region+Sex	0.772	4688.000
ENSG00000145685	0.161	0.050	0.162	-0.061	LHFPL2	0.216	~amyloid_beta+(1|manifest)+cngeneson+pc_mito+brain_region+Sex	0.651	4996.000
ENSG00000007168	0.121	0.050	0.233	-0.132	PAFAH1B1	0.178	~amyloid_beta+(1|manifest)+cngeneson+pc_mito+brain_region+Sex	2.415	1966.000
ENSG00000143195	0.481	0.050	0.356	-0.255	ILDR2	0.525	~amyloid_beta+(1|manifest)+cngeneson+pc_mito+brain_region+Sex	5.365	697.000
ENSG00000100354	0.556	0.050	0.140	-0.039	TNRC6B	0.596	~amyloid_beta+(1|manifest)+cngeneson+pc_mito+brain_region+Sex	1.428	3269.000
ENSG00000082701	0.401	0.050	0.255	-0.154	GSK3B	0.449	~amyloid_beta+(1|manifest)+cngeneson+pc_mito+brain_region+Sex	4.662	855.000
ENSG00000155096	0.112	0.050	0.171	-0.070	AZIN1	0.168	~amyloid_beta+(1|manifest)+cngeneson+pc_mito+brain_region+Sex	1.112	3904.000
ENSG00000148400	0.191	0.050	0.344	-0.243	NOTCH1	0.246	~amyloid_beta+(1|manifest)+cngeneson+pc_mito+brain_region+Sex	3.451	1275.000
ENSG00000168175	0.030	0.050	0.266	-0.166	[illegible]	0.074	~amyloid_beta+(1|manifest)+cngeneson+pc_mito+brain_region+Sex	1.718	2799.000
ENSG00000185591	0.026	0.050	0.220	-0.120	SP1	0.069	~amyloid_beta+(1|manifest)+cngeneson+pc_mito+brain_region+Sex	1.261	3581.000
ENSG00000124782	0.154	0.050	0.210	-0.110	RREB1	0.210	~amyloid_beta+(1|manifest)+cngeneson+pc_mito+brain_region+Sex	0.971	4202.000
ENSG00000186625	0.026	0.050	0.096	0.004	KATNA1	0.069	~amyloid_beta+(1|manifest)+cngeneson+pc_mito+brain_region+Sex	0.517	5310.000
ENSG00000107341	0.034	0.050	0.249	-0.149	UBE2R2	0.079	~amyloid_beta+(1|manifest)+cngeneson+pc_mito+brain_region+Sex	1.612	2963.000
ENSG00000065491	0.019	0.050	0.183	-0.084	TBC1D22B	0.057	~amyloid_beta+(1|manifest)+cngeneson+pc_mito+brain_region+Sex	0.836	4527.000
ENSG00000087008	0.023	0.050	0.110	-0.011	ACOX3	0.064	~amyloid_beta+(1|manifest)+cngeneson+pc_mito+brain_region+Sex	0.303	5729.000
ENSG00000143815	0.016	0.049	0.148	-0.049	LBR	0.053	~amyloid_beta+(1|manifest)+cngeneson+pc_mito+brain_region+Sex	0.153	5920.000
ENSG00000073417	0.664	0.049	0.371	-0.272	PDE8A	0.695	~amyloid_beta+(1|manifest)+cngeneson+pc_mito+brain_region+Sex	8.686	331.000
ENSG00000078618	0.015	0.049	0.185	-0.086	NRDC	0.050	~amyloid_beta+(1|manifest)+cngeneson+pc_mito+brain_region+Sex	2.659	1763.000
ENSG00000100106	0.048	0.049	0.183	-0.084	TRIOBP	0.097	~amyloid_beta+(1|manifest)+cngeneson+pc_mito+brain_region+Sex	1.598	2987.000
ENSG00000183718	0.070	0.049	0.133	-0.034	TRIM52	0.124	~amyloid_beta+(1|manifest)+cngeneson+pc_mito+brain_region+Sex	0.723	4815.000
ENSG00000183597	0.062	0.049	0.163	-0.065	TANGO2	0.113	~amyloid_beta+(1|manifest)+cngeneson+pc_mito+brain_region+Sex	0.970	4203.000
ENSG00000130723	0.213	0.049	0.306	-0.208	PRRC2B	0.267	~amyloid_beta+(1|manifest)+cngeneson+pc_mito+brain_region+Sex	3.681	1173.000
ENSG00000110917	0.085	0.049	0.204	-0.106	MLEC	0.140	~amyloid_beta+(1|manifest)+cngeneson+pc_mito+brain_region+Sex	0.476	5407.000
ENSG00000134748	0.029	0.049	0.106	-0.008	PRPF38A	0.073	~amyloid_beta+(1|manifest)+cngeneson+pc_mito+brain_region+Sex	0.366	5626.000
ENSG00000138035	0.053	0.049	0.142	-0.045	PNPT1	0.103	~amyloid_beta+(1|manifest)+cngeneson+pc_mito+brain_region+Sex	0.920	4328.000
ENSG00000127054	0.172	0.049	0.263	-0.165	INTS11	0.227	~amyloid_beta+(1|manifest)+cngeneson+pc_mito+brain_region+Sex	2.225	2175.000
ENSG00000102763	0.591	0.049	0.408	-0.311	VWA8	0.627	~amyloid_beta+(1|manifest)+cngeneson+pc_mito+brain_region+Sex	11.980	178.000
ENSG00000101247	0.028	0.048	0.142	-0.045	NDUFAF5	0.072	~amyloid_beta+(1|manifest)+cngeneson+pc_mito+brain_region+Sex	0.627	5066.000
ENSG00000131437	0.055	0.048	0.190	-0.093	KIF3A	0.106	~amyloid_beta+(1|manifest)+cngeneson+pc_mito+brain_region+Sex	0.812	4593.000
ENSG00000151689	0.183	0.048	0.337	-0.241	INPP1	0.237	~amyloid_beta+(1|manifest)+cngeneson+pc_mito+brain_region+Sex	3.598	1208.000
ENSG00000111266	0.072	0.048	0.206	-0.111	DUSP16	0.126	~amyloid_beta+(1|manifest)+cngeneson+pc_mito+brain_region+Sex	0.197	5873.000
ENSG00000153827	0.191	0.048	0.179	-0.084	TRIP12	0.246	~amyloid_beta+(1|manifest)+cngeneson+pc_mito+brain_region+Sex	2.025	2402.000
ENSG00000184575	0.041	0.047	0.223	-0.128	XPOT	0.089	~amyloid_beta+(1|manifest)+cngeneson+pc_mito+brain_region+Sex	0.435	5497.000
ENSG00000143093	0.042	0.047	0.148	-0.053	STRIP1	0.089	~amyloid_beta+(1|manifest)+cngeneson+pc_mito+brain_region+Sex	1.264	3571.000
ENSG00000145990	0.230	0.047	0.264	-0.169	GFOD1	0.284	~amyloid_beta+(1|manifest)+cngeneson+pc_mito+brain_region+Sex	1.667	2877.000
ENSG00000138439	0.006	0.047	0.164	-0.069	FAM117B	0.030	~amyloid_beta+(1|manifest)+cngeneson+pc_mito+brain_region+Sex	0.704	4870.000
ENSG00000167528	0.152	0.047	0.196	-0.101	ZNF641	0.208	~amyloid_beta+(1|manifest)+cngeneson+pc_mito+brain_region+Sex	1.199	3708.000
ENSG00000171843	0.533	0.047	0.317	-0.222	MLLT3	0.575	~amyloid_beta+(1|manifest)+cngeneson+pc_mito+brain_region+Sex	1.898	2553.000
ENSG00000075303	0.006	0.047	0.127	-0.033	SLC25A40	0.030	~amyloid_beta+(1|manifest)+cngeneson+pc_mito+brain_region+Sex	0.648	5008.000
ENSG00000198825	0.806	0.047	0.405	-0.311	INPP5F	0.826	~amyloid_beta+(1|manifest)+cngeneson+pc_mito+brain_region+Sex	5.932	601.000
ENSG00000070882	0.343	0.047	0.322	-0.228	OSBPL3	0.394	~amyloid_beta+(1|manifest)+cngeneson+pc_mito+brain_region+Sex	1.999	2432.000
ENSG00000163945	0.097	0.047	0.327	-0.233	UVSSA	0.152	~amyloid_beta+(1|manifest)+cngeneson+pc_mito+brain_region+Sex	2.569	1839.000
ENSG00000091039	0.009	0.047	0.207	-0.114	OSBPL8	0.038	~amyloid_beta+(1|manifest)+cngeneson+pc_mito+brain_region+Sex	1.602	2982.000
ENSG00000198793	0.067	0.047	0.169	-0.075	MTOR	0.120	~amyloid_beta+(1|manifest)+cngeneson+pc_mito+brain_region+Sex	0.940	4279.000
ENSG00000117500	0.116	0.047	0.172	-0.078	TMED5	0.172	~amyloid_beta+(1|manifest)+cngeneson+pc_mito+brain_region+Sex	0.770	4691.000
ENSG00000164961	0.021	0.047	0.120	-0.027	WASHC5	0.061	~amyloid_beta+(1|manifest)+cngeneson+pc_mito+brain_region+Sex	0.456	5446.000
ENSG00000132780	0.271	0.047	0.278	-0.185	NASP	0.324	~amyloid_beta+(1|manifest)+cngeneson+pc_mito+brain_region+Sex	3.415	1305.000
ENSG00000065135	0.015	0.046	0.149	-0.056	GNAI3	0.050	~amyloid_beta+(1|manifest)+cngeneson+pc_mito+brain_region+Sex	0.662	4972.000
ENSG00000160087	0.103	0.046	0.145	-0.053	UBE2J2	0.158	~amyloid_beta+(1|manifest)+cngeneson+pc_mito+brain_region+Sex	1.251	3597.000
ENSG00000109184	0.052	0.046	0.163	-0.071	DCUN1D4	0.102	~amyloid_beta+(1|manifest)+cngeneson+pc_mito+brain_region+Sex	1.588	3002.000
ENSG00000137413	0.043	0.046	0.163	-0.071	TAF8	0.091	~amyloid_beta+(1|manifest)+cngeneson+pc_mito+brain_region+Sex	0.991	4162.000
ENSG00000145016	0.099	0.046	0.188	-0.097	RUBCN	0.155	~amyloid_beta+(1|manifest)+cngeneson+pc_mito+brain_region+Sex	1.353	3405.000
ENSG00000126464	0.028	0.046	0.149	-0.057	PRR12	0.072	~amyloid_beta+(1|manifest)+cngeneson+pc_mito+brain_region+Sex	0.737	4778.000
ENSG00000152527	0.064	0.046	0.225	-0.134	PLEKHH2	0.117	~amyloid_beta+(1|manifest)+cngeneson+pc_mito+brain_region+Sex	2.087	2326.000
ENSG00000138029	0.034	0.046	0.213	-0.121	HADHB	0.079	~amyloid_beta+(1|manifest)+cngeneson+pc_mito+brain_region+Sex	2.069	2347.000
ENSG00000152518	0.919	0.046	0.340	-0.249	ZFP36L2	0.929	~amyloid_beta+(1|manifest)+cngeneson+pc_mito+brain_region+Sex	1.554	3042.000
ENSG00000136861	0.067	0.046	0.189	-0.098	CDK5RAP2	0.120	~amyloid_beta+(1|manifest)+cngeneson+pc_mito+brain_region+Sex	2.019	2412.000
ENSG00000119522	0.698	0.045	0.160	-0.069	DENND1A	0.728	~amyloid_beta+(1|manifest)+cngeneson+pc_mito+brain_region+Sex	1.640	2924.000
ENSG00000169439	0.555	0.045	0.258	-0.167	SDC2	0.594	~amyloid_beta+(1|manifest)+cngeneson+pc_mito+brain_region+Sex	0.718	4827.000
ENSG00000165138	0.042	0.045	0.151	-0.060	ANKS6	0.090	~amyloid_beta+(1|manifest)+cngeneson+pc_mito+brain_region+Sex	0.373	5612.000
ENSG00000179218	0.142	0.045	0.364	-0.274	CALR	0.198	~amyloid_beta+(1|manifest)+cngeneson+pc_mito+brain_region+Sex	1.446	3236.000
ENSG00000173905	0.521	0.045	0.249	-0.159	GOLIM4	0.563	~amyloid_beta+(1|manifest)+cngeneson+pc_mito+brain_region+Sex	3.581	1216.000
ENSG00000011243	0.107	0.045	0.328	-0.238	AKAP8L	0.163	~amyloid_beta+(1|manifest)+cngeneson+pc_mito+brain_region+Sex	2.892	1611.000
ENSG00000170464	0.016	0.045	0.145	-0.055	DNAJC18	0.052	~amyloid_beta+(1|manifest)+cngeneson+pc_mito+brain_region+Sex	0.505	5336.000
ENSG00000139436	0.036	0.045	0.126	-0.037	GIT2	0.082	~amyloid_beta+(1|manifest)+cngeneson+pc_mito+brain_region+Sex	0.561	5209.000
ENSG00000101181	0.133	0.045	0.220	-0.130	MTG2	0.190	~amyloid_beta+(1|manifest)+cngeneson+pc_mito+brain_region+Sex	1.895	2558.000
ENSG00000180817	0.040	0.045	0.147	-0.057	PPA1	0.087	~amyloid_beta+(1|manifest)+cngeneson+pc_mito+brain_region+Sex	0.512	5326.000
ENSG00000101109	0.037	0.045	0.091	-0.002	STK4	0.083	~amyloid_beta+(1|manifest)+cngeneson+pc_mito+brain_region+Sex	0.686	4927.000
ENSG00000141198	0.238	0.045	0.211	-0.122	TOM1L1	0.292	~amyloid_beta+(1|manifest)+cngeneson+pc_mito+brain_region+Sex	2.025	2404.000
ENSG00000148218	0.094	0.045	0.216	-0.126	ALAD	0.150	~amyloid_beta+(1|manifest)+cngeneson+pc_mito+brain_region+Sex	1.698	2825.000
ENSG00000108883	0.013	0.045	0.159	-0.070	EFTUD2	0.047	~amyloid_beta+(1|manifest)+cngeneson+pc_mito+brain_region+Sex	1.155	3801.000
ENSG00000033011	0.059	0.044	0.139	-0.050	ALG1	0.110	~amyloid_beta+(1|manifest)+cngeneson+pc_mito+brain_region+Sex	0.716	4830.000
ENSG00000132478	0.051	0.044	0.188	-0.100	UNK	0.101	~amyloid_beta+(1|manifest)+cngeneson+pc_mito+brain_region+Sex	0.708	4856.000
ENSG00000069869	0.038	0.044	0.266	-0.177	NEDD4	0.085	~amyloid_beta+(1|manifest)+cngeneson+pc_mito+brain_region+Sex	0.775	4675.000
ENSG00000071967	0.083	0.044	0.291	-0.203	CYBRD1	0.138	~amyloid_beta+(1|manifest)+cngeneson+pc_mito+brain_region+Sex	0.441	5479.000
ENSG00000165338	0.250	0.044	0.214	-0.126	HECTD2	0.304	~amyloid_beta+(1|manifest)+cngeneson+pc_mito+brain_region+Sex	2.859	1628.000

ENSG00000171735	0.516	0.044	0.121	-0.033	CAMTA1	0.559	~amyloid_beta+(1|manifest)+cngeneson+pc_mito+brain_region+Sex	5.566	668.000
ENSG00000128881	0.013	0.044	0.183	-0.095	TTBK2	0.047	~amyloid_beta+(1|manifest)+cngeneson+pc_mito+brain_region+Sex	1.921	2536.000
ENSG00000113441	0.180	0.044	0.171	-0.084	LNPEP	0.234	~amyloid_beta+(1|manifest)+cngeneson+pc_mito+brain_region+Sex	1.115	3898.000
ENSG00000018408	0.979	0.043	0.467	-0.380	WWTR1	0.981	~amyloid_beta+(1|manifest)+cngeneson+pc_mito+brain_region+Sex	13.158	145.000
ENSG00000153339	0.008	0.043	0.199	-0.113	TRAPPC8	0.035	~amyloid_beta+(1|manifest)+cngeneson+pc_mito+brain_region+Sex	0.928	4305.000
ENSG00000135052	0.037	0.043	0.195	-0.109	GOLM1	0.084	~amyloid_beta+(1|manifest)+cngeneson+pc_mito+brain_region+Sex	0.938	4283.000
ENSG00000080503	0.907	0.043	0.254	-0.168	SMARCA2	0.918	~amyloid_beta+(1|manifest)+cngeneson+pc_mito+brain_region+Sex	5.713	645.000
ENSG00000007202	0.101	0.043	0.164	-0.078	KIAA0100	0.156	~amyloid_beta+(1|manifest)+cngeneson+pc_mito+brain_region+Sex	1.540	3064.000
ENSG00000136478	0.126	0.043	0.355	-0.270	TEX2	0.182	~amyloid_beta+(1|manifest)+cngeneson+pc_mito+brain_region+Sex	4.406	914.000
ENSG00000124789	0.011	0.043	0.186	-0.101	NUP153	0.041	~amyloid_beta+(1|manifest)+cngeneson+pc_mito+brain_region+Sex	1.289	3530.000
ENSG00000129128	0.058	0.043	0.135	-0.050	SPCS3	0.109	~amyloid_beta+(1|manifest)+cngeneson+pc_mito+brain_region+Sex	0.760	4716.000
ENSG00000099956	0.014	0.043	0.124	-0.038	SMARCB1	0.048	~amyloid_beta+(1|manifest)+cngeneson+pc_mito+brain_region+Sex	0.525	5297.000
ENSG00000077157	0.733	0.043	0.198	-0.113	PPP1R12B	0.760	~amyloid_beta+(1|manifest)+cngeneson+pc_mito+brain_region+Sex	0.683	4930.000
ENSG00000188735	0.083	0.043	0.151	-0.066	[illegible]	0.138	~amyloid_beta+(1|manifest)+cngeneson+pc_mito+brain_region+Sex	1.076	3974.000
ENSG00000109685	0.040	0.042	0.213	-0.128	NSD2	0.087	~amyloid_beta+(1|manifest)+cngeneson+pc_mito+brain_region+Sex	1.427	3271.000
ENSG00000100485	0.098	0.042	0.147	-0.062	SOS2	0.153	~amyloid_beta+(1|manifest)+cngeneson+pc_mito+brain_region+Sex	1.971	2467.000
ENSG00000149716	0.149	0.042	0.222	-0.138	LTO1	0.204	~amyloid_beta+(1|manifest)+cngeneson+pc_mito+brain_region+Sex	1.779	2717.000
ENSG00000186106	0.036	0.042	0.131	-0.047	ANKRD46	0.082	~amyloid_beta+(1|manifest)+cngeneson+pc_mito+brain_region+Sex	0.203	5869.000
ENSG00000187446	0.040	0.042	0.130	-0.047	CHP1	0.088	~amyloid_beta+(1|manifest)+cngeneson+pc_mito+brain_region+Sex	0.721	4822.000
ENSG00000186020	0.027	0.042	0.121	-0.038	ZNF529	0.070	~amyloid_beta+(1|manifest)+cngeneson+pc_mito+brain_region+Sex	1.251	3598.000
ENSG00000214078	0.080	0.042	0.239	-0.156	CPNE1	0.135	~amyloid_beta+(1|manifest)+cngeneson+pc_mito+brain_region+Sex	2.298	2110.000
ENSG00000147649	0.043	0.042	0.165	-0.081	MTDH	0.091	~amyloid_beta+(1|manifest)+cngeneson+pc_mito+brain_region+Sex	1.297	3512.000
ENSG00000196498	0.238	0.041	0.315	-0.232	NCOR2	0.292	~amyloid_beta+(1|manifest)+cngeneson+pc_mito+brain_region+Sex	2.847	1637.000
ENSG00000089154	0.050	0.041	0.101	-0.019	GCN1	0.099	~amyloid_beta+(1|manifest)+cngeneson+pc_mito+brain_region+Sex	0.698	4897.000
ENSG00000102753	0.044	0.041	0.142	-0.060	KPNA3	0.093	~amyloid_beta+(1|manifest)+cngeneson+pc_mito+brain_region+Sex	1.928	2524.000
ENSG00000130299	0.168	0.041	0.188	-0.106	GTPBP3	0.223	~amyloid_beta+(1|manifest)+cngeneson+pc_mito+brain_region+Sex	2.366	2033.000
ENSG00000130844	0.229	0.041	0.128	-0.046	ZNF331	0.283	~amyloid_beta+(1|manifest)+cngeneson+pc_mito+brain_region+Sex	0.990	4163.000
ENSG00000169087	0.032	0.041	0.101	-0.019	HSPBAP1	0.076	~amyloid_beta+(1|manifest)+cngeneson+pc_mito+brain_region+Sex	0.747	4749.000
ENSG00000152464	0.055	0.041	0.183	-0.101	RPP38	0.105	~amyloid_beta+(1|manifest)+cngeneson+pc_mito+brain_region+Sex	0.864	4455.000
ENSG00000120137	0.016	0.041	0.155	-0.074	PANK3	0.053	~amyloid_beta+(1|manifest)+cngeneson+pc_mito+brain_region+Sex	1.265	3569.000
ENSG00000141026	0.010	0.041	0.106	-0.025	MED9	0.041	~amyloid_beta+(1|manifest)+cngeneson+pc_mito+brain_region+Sex	0.487	5392.000
ENSG00000064651	0.030	0.041	0.169	-0.088	SLC12A2	0.074	~amyloid_beta+(1|manifest)+cngeneson+pc_mito+brain_region+Sex	1.513	3114.000
ENSG00000137055	0.026	0.041	0.101	-0.020	PLAA	0.068	~amyloid_beta+(1|manifest)+cngeneson+pc_mito+brain_region+Sex	0.535	5268.000
ENSG00000087448	0.137	0.040	0.215	-0.134	KLHL42	0.194	~amyloid_beta+(1|manifest)+cngeneson+pc_mito+brain_region+Sex	0.831	4539.000
ENSG00000160799	0.095	0.040	0.117	-0.037	CCDC12	0.150	~amyloid_beta+(1|manifest)+cngeneson+pc_mito+brain_region+Sex	0.761	4713.000
ENSG00000197375	0.009	0.040	0.220	-0.140	SLC22A5	0.036	~amyloid_beta+(1|manifest)+cngeneson+pc_mito+brain_region+Sex	1.912	2540.000
ENSG00000011105	0.080	0.040	0.145	-0.065	TSPAN9	0.134	~amyloid_beta+(1|manifest)+cngeneson+pc_mito+brain_region+Sex	0.516	5314.000
ENSG00000151304	0.044	0.040	0.127	-0.047	SRFBP1	0.093	~amyloid_beta+(1|manifest)+cngeneson+pc_mito+brain_region+Sex	0.641	5029.000
ENSG00000186575	0.086	0.040	0.124	-0.044	NF2	0.141	~amyloid_beta+(1|manifest)+cngeneson+pc_mito+brain_region+Sex	0.581	5169.000
ENSG00000076513	0.046	0.040	0.135	-0.055	ANKRD13A	0.095	~amyloid_beta+(1|manifest)+cngeneson+pc_mito+brain_region+Sex	0.946	4261.000
ENSG00000112983	0.040	0.040	0.162	-0.082	BRD8	0.087	~amyloid_beta+(1|manifest)+cngeneson+pc_mito+brain_region+Sex	1.464	3199.000
ENSG00000183955	0.190	0.040	0.188	-0.108	KMT5A	0.245	~amyloid_beta+(1|manifest)+cngeneson+pc_mito+brain_region+Sex	1.451	3224.000
ENSG00000046653	0.858	0.040	0.212	-0.132	GPM6B	0.872	~amyloid_beta+(1|manifest)+cngeneson+pc_mito+brain_region+Sex	7.949	379.000
ENSG00000130699	0.053	0.040	0.107	-0.028	TAF4	0.104	~amyloid_beta+(1|manifest)+cngeneson+pc_mito+brain_region+Sex	0.531	5283.000
ENSG00000166411	0.046	0.040	0.141	-0.062	IDH3A	0.095	~amyloid_beta+(1|manifest)+cngeneson+pc_mito+brain_region+Sex	0.959	4227.000
ENSG00000151923	0.065	0.040	0.175	-0.096	TIAL1	0.117	~amyloid_beta+(1|manifest)+cngeneson+pc_mito+brain_region+Sex	1.529	3087.000
ENSG00000114648	0.047	0.040	0.100	-0.021	KLHL18	0.096	~amyloid_beta+(1|manifest)+cngeneson+pc_mito+brain_region+Sex	0.497	5369.000
ENSG00000137962	0.506	0.040	0.437	-0.358	ARHGAP29	0.549	~amyloid_beta+(1|manifest)+cngeneson+pc_mito+brain_region+Sex	4.486	892.000
ENSG00000157087	0.618	0.040	0.406	-0.327	ATP2B2	0.654	~amyloid_beta+(1|manifest)+cngeneson+pc_mito+brain_region+Sex	7.336	433.000
ENSG00000108797	0.181	0.039	0.329	-0.250	CNTNAP1	0.236	~amyloid_beta+(1|manifest)+cngeneson+pc_mito+brain_region+Sex	3.002	1542.000
ENSG00000114030	0.056	0.039	0.186	-0.108	KPNA1	0.106	~amyloid_beta+(1|manifest)+cngeneson+pc_mito+brain_region+Sex	1.747	2751.000
ENSG00000139636	0.059	0.039	0.120	-0.042	LMBR1L	0.110	~amyloid_beta+(1|manifest)+cngeneson+pc_mito+brain_region+Sex	0.856	4477.000
ENSG00000165449	0.519	0.039	0.489	-0.411	SLC16A9	0.562	~amyloid_beta+(1|manifest)+cngeneson+pc_mito+brain_region+Sex	2.059	2357.000
ENSG00000165006	0.325	0.039	0.301	-0.223	UBAP1	0.377	~amyloid_beta+(1|manifest)+cngeneson+pc_mito+brain_region+Sex	3.739	1146.000
ENSG00000115364	0.031	0.039	0.142	-0.064	MRPL19	0.075	~amyloid_beta+(1|manifest)+cngeneson+pc_mito+brain_region+Sex	0.352	5648.000
ENSG00000002549	0.036	0.039	0.151	-0.074	LAP3	0.082	~amyloid_beta+(1|manifest)+cngeneson+pc_mito+brain_region+Sex	1.925	2531.000
ENSG00000167978	0.647	0.039	0.608	-0.530	SRRM2	0.680	~amyloid_beta+(1|manifest)+cngeneson+pc_mito+brain_region+Sex	12.449	164.000
ENSG00000167766	0.071	0.039	0.283	-0.205	ZNF83	0.125	~amyloid_beta+(1|manifest)+cngeneson+pc_mito+brain_region+Sex	1.634	2934.000
ENSG00000196263	0.044	0.038	0.185	-0.108	ZNF471	0.093	~amyloid_beta+(1|manifest)+cngeneson+pc_mito+brain_region+Sex	0.700	4886.000
ENSG00000176542	0.007	0.038	0.141	-0.064	USF3	0.031	~amyloid_beta+(1|manifest)+cngeneson+pc_mito+brain_region+Sex	0.958	4230.000
ENSG00000113916	0.937	0.038	0.377	-0.300	BCL6	0.944	~amyloid_beta+(1|manifest)+cngeneson+pc_mito+brain_region+Sex	23.828	33.000
ENSG00000176853	0.012	0.038	0.096	-0.020	FAM91A1	0.044	~amyloid_beta+(1|manifest)+cngeneson+pc_mito+brain_region+Sex	0.425	5516.000
ENSG00000105221	0.411	0.038	0.261	-0.185	AKT2	0.458	~amyloid_beta+(1|manifest)+cngeneson+pc_mito+brain_region+Sex	3.913	1086.000
ENSG00000105887	0.031	0.038	0.153	-0.077	MTPN	0.075	~amyloid_beta+(1|manifest)+cngeneson+pc_mito+brain_region+Sex	0.362	5631.000
ENSG00000070413	0.143	0.038	0.152	-0.075	DGCR2	0.199	~amyloid_beta+(1|manifest)+cngeneson+pc_mito+brain_region+Sex	0.935	4290.000
ENSG00000145824	0.168	0.038	0.380	-0.304	CXCL14	0.224	~amyloid_beta+(1|manifest)+cngeneson+pc_mito+brain_region+Sex	3.673	1175.000
ENSG00000115652	0.060	0.038	0.095	-0.019	UXS1	0.111	~amyloid_beta+(1|manifest)+cngeneson+pc_mito+brain_region+Sex	0.613	5103.000
ENSG00000060762	0.111	0.038	0.222	-0.146	MPC1	0.167	~amyloid_beta+(1|manifest)+cngeneson+pc_mito+brain_region+Sex	1.661	2885.000
ENSG00000163281	0.021	0.038	0.150	-0.074	GNPDA2	0.060	~amyloid_beta+(1|manifest)+cngeneson+pc_mito+brain_region+Sex	0.195	5877.000
ENSG00000110074	0.091	0.038	0.183	-0.107	FOXRED1	0.147	~amyloid_beta+(1|manifest)+cngeneson+pc_mito+brain_region+Sex	1.846	2633.000
ENSG00000153291	0.026	0.038	0.196	-0.121	SLC25A27	0.069	~amyloid_beta+(1|manifest)+cngeneson+pc_mito+brain_region+Sex	1.512	3120.000
ENSG00000136682	0.080	0.038	0.132	-0.056	CBWD2	0.135	~amyloid_beta+(1|manifest)+cngeneson+pc_mito+brain_region+Sex	0.318	5697.000
ENSG00000203965	0.026	0.038	0.188	-0.113	EFCAB7	0.069	~amyloid_beta+(1|manifest)+cngeneson+pc_mito+brain_region+Sex	0.432	5504.000
ENSG00000185736	0.333	0.037	0.748	-0.673	ADARB2	0.385	~amyloid_beta+(1|manifest)+cngeneson+pc_mito+brain_region+Sex	6.897	479.000
ENSG00000258484	0.072	0.037	0.194	-0.119	SPESP1	0.126	~amyloid_beta+(1|manifest)+cngeneson+pc_mito+brain_region+Sex	0.859	4468.000
ENSG00000155975	0.019	0.037	0.120	-0.046	VPS37A	0.057	~amyloid_beta+(1|manifest)+cngeneson+pc_mito+brain_region+Sex	0.529	5289.000
ENSG00000137693	0.738	0.037	0.227	-0.152	YAP1	0.764	~amyloid_beta+(1|manifest)+cngeneson+pc_mito+brain_region+Sex	4.004	1039.000
ENSG00000067182	0.253	0.037	0.347	-0.273	TNFRSF1A	0.307	~amyloid_beta+(1|manifest)+cngeneson+pc_mito+brain_region+Sex	2.848	1636.000
ENSG00000196074	0.176	0.037	0.195	-0.121	SYCP2	0.231	~amyloid_beta+(1|manifest)+cngeneson+pc_mito+brain_region+Sex	1.248	3607.000
ENSG00000142082	0.075	0.037	0.161	-0.087	SIRT3	0.128	~amyloid_beta+(1|manifest)+cngeneson+pc_mito+brain_region+Sex	1.576	3016.000
ENSG00000122786	0.172	0.037	0.483	-0.409	CALD1	0.227	~amyloid_beta+(1|manifest)+cngeneson+pc_mito+brain_region+Sex	0.299	5735.000
ENSG00000164305	0.089	0.037	0.150	-0.077	CASP3	0.144	~amyloid_beta+(1|manifest)+cngeneson+pc_mito+brain_region+Sex	0.708	4857.000
ENSG00000168374	0.083	0.037	0.102	-0.029	ARF4	0.138	~amyloid_beta+(1|manifest)+cngeneson+pc_mito+brain_region+Sex	0.159	5914.000
ENSG00000169871	0.049	0.037	0.270	-0.197	TRIM56	0.098	~amyloid_beta+(1|manifest)+cngeneson+pc_mito+brain_region+Sex	3.294	1372.000
ENSG00000126012	0.091	0.036	0.136	-0.063	KDM5C	0.146	~amyloid_beta+(1|manifest)+cngeneson+pc_mito+brain_region+Sex	3.445	1277.000
ENSG00000206530	0.168	0.036	0.165	-0.092	CFAP44	0.224	~amyloid_beta+(1|manifest)+cngeneson+pc_mito+brain_region+Sex	0.596	5139.000
ENSG00000165055	0.338	0.036	0.245	-0.172	METTL2B	0.389	~amyloid_beta+(1|manifest)+cngeneson+pc_mito+brain_region+Sex	3.768	1135.000
ENSG00000121289	0.019	0.036	0.179	-0.106	CEP89	0.057	~amyloid_beta+(1|manifest)+cngeneson+pc_mito+brain_region+Sex	0.932	4297.000
ENSG00000038382	0.807	0.036	0.228	-0.156	TRIO	0.826	~amyloid_beta+(1|manifest)+cngeneson+pc_mito+brain_region+Sex	15.581	93.000
ENSG00000136770	0.235	0.036	0.255	-0.182	DNAJC1	0.289	~amyloid_beta+(1|manifest)+cngeneson+pc_mito+brain_region+Sex	2.369	2028.000
ENSG00000070770	0.008	0.036	0.105	-0.032	CSNK2A2	0.035	~amyloid_beta+(1|manifest)+cngeneson+pc_mito+brain_region+Sex	0.387	5584.000
ENSG00000167986	0.063	0.036	0.162	-0.089	DDB1	0.116	~amyloid_beta+(1|manifest)+cngeneson+pc_mito+brain_region+Sex	0.920	4331.000
ENSG00000135144	0.390	0.036	0.367	-0.295	DTX1	0.439	~amyloid_beta+(1|manifest)+cngeneson+pc_mito+brain_region+Sex	4.558	875.000
ENSG00000079691	0.672	0.036	0.137	-0.064	CARMIL1	0.703	~amyloid_beta+(1|manifest)+cngeneson+pc_mito+brain_region+Sex	2.165	2245.000
ENSG00000018625	0.942	0.036	0.238	-0.167	ATP1A2	0.948	~amyloid_beta+(1|manifest)+cngeneson+pc_mito+brain_region+Sex	16.280	83.000
ENSG00000175470	0.005	0.036	0.134	-0.062	PPP2R2D	0.027	~amyloid_beta+(1|manifest)+cngeneson+pc_mito+brain_region+Sex	0.812	4594.000
ENSG00000120533	0.042	0.036	0.150	-0.078	ENY2	0.089	~amyloid_beta+(1|manifest)+cngeneson+pc_mito+brain_region+Sex	0.206	5866.000
ENSG00000148484	0.017	0.036	0.201	-0.129	RSU1	0.054	~amyloid_beta+(1|manifest)+cngeneson+pc_mito+brain_region+Sex	2.386	2007.000
ENSG00000075826	0.127	0.036	0.157	-0.086	SEC31B	0.183	~amyloid_beta+(1|manifest)+cngeneson+pc_mito+brain_region+Sex	1.424	3279.000
ENSG00000078487	0.117	0.036	0.162	-0.091	ZCWPW1	0.173	~amyloid_beta+(1|manifest)+cngeneson+pc_mito+brain_region+Sex	1.322	3460.000
ENSG00000017797	0.031	0.036	0.113	-0.042	RALBP1	0.075	~amyloid_beta+(1|manifest)+cngeneson+pc_mito+brain_region+Sex	0.180	5899.000
ENSG00000151835	0.165	0.035	0.359	-0.288	SACS	0.220	~amyloid_beta+(1|manifest)+cngeneson+pc_mito+brain_region+Sex	3.354	1339.000
ENSG00000161021	0.056	0.035	0.121	-0.050	MAML1	0.107	~amyloid_beta+(1|manifest)+cngeneson+pc_mito+brain_region+Sex	0.309	5711.000
ENSG00000152936	0.954	0.035	0.499	-0.429	LMNTD1	0.959	~amyloid_beta+(1|manifest)+cngeneson+pc_mito+brain_region+Sex	4.927	792.000
ENSG00000130449	0.839	0.035	0.171	-0.101	ZSWIM6	0.856	~amyloid_beta+(1|manifest)+cngeneson+pc_mito+brain_region+Sex	4.319	943.000
ENSG00000104687	0.042	0.035	0.157	-0.087	GSR	0.089	~amyloid_beta+(1|manifest)+cngeneson+pc_mito+brain_region+Sex	1.081	3967.000
ENSG00000121413	0.196	0.035	0.271	-0.200	ZSCAN18	0.251	~amyloid_beta+(1|manifest)+cngeneson+pc_mito+brain_region+Sex	3.405	1312.000
ENSG00000214595	0.934	0.035	0.308	-0.237	EML6	0.942	~amyloid_beta+(1|manifest)+cngeneson+pc_mito+brain_region+Sex	1.833	2652.000
ENSG00000089597	0.148	0.035	0.191	-0.121	GANAB	0.204	~amyloid_beta+(1|manifest)+cngeneson+pc_mito+brain_region+Sex	1.514	3113.000
ENSG00000000419	0.025	0.035	0.121	-0.051	DPM1	0.067	~amyloid_beta+(1|manifest)+cngeneson+pc_mito+brain_region+Sex	0.435	5496.000
ENSG00000101298	0.075	0.035	0.159	-0.089	SNPH	0.128	~amyloid_beta+(1|manifest)+cngeneson+pc_mito+brain_region+Sex	0.599	5133.000
ENSG00000153774	0.230	0.035	0.156	-0.086	CFDP1	0.284	~amyloid_beta+(1|manifest)+cngeneson+pc_mito+brain_region+Sex	0.917	4335.000
ENSG00000121644	0.064	0.035	0.119	-0.049	DESI2	0.116	~amyloid_beta+(1|manifest)+cngeneson+pc_mito+brain_region+Sex	0.480	5402.000
ENSG00000170832	0.140	0.035	0.244	-0.174	USP32	0.197	~amyloid_beta+(1|manifest)+cngeneson+pc_mito+brain_region+Sex	3.637	1187.000
ENSG00000114544	0.063	0.035	0.135	-0.065	SLC41A3	0.115	~amyloid_beta+(1|manifest)+cngeneson+pc_mito+brain_region+Sex	0.670	4962.000
ENSG00000166979	0.599	0.035	0.345	-0.275	EVA1C	0.635	~amyloid_beta+(1|manifest)+cngeneson+pc_mito+brain_region+Sex	4.207	981.000
ENSG00000108239	0.016	0.035	0.134	-0.064	TBC1D12	0.053	~amyloid_beta+(1|manifest)+cngeneson+pc_mito+brain_region+Sex	0.640	5031.000
ENSG00000145246	0.085	0.035	0.138	-0.068	ATP10D	0.140	~amyloid_beta+(1|manifest)+cngeneson+pc_mito+brain_region+Sex	1.375	3369.000
ENSG00000161202	0.161	0.035	0.165	-0.095	DVL3	0.217	~amyloid_beta+(1|manifest)+cngeneson+pc_mito+brain_region+Sex	1.233	3626.000
ENSG00000047365	0.788	0.035	0.132	-0.062	ARAP2	0.808	~amyloid_beta+(1|manifest)+cngeneson+pc_mito+brain_region+Sex	4.812	820.000
ENSG00000115239	0.192	0.035	0.265	-0.196	ASB3	0.246	~amyloid_beta+(1|manifest)+cngeneson+pc_mito+brain_region+Sex	3.475	1258.000
ENSG00000117118	0.097	0.035	0.170	-0.101	SDHB	0.152	~amyloid_beta+(1|manifest)+cngeneson+pc_mito+brain_region+Sex	1.281	3543.000
ENSG00000128268	0.078	0.034	0.207	-0.138	MGAT3	0.133	~amyloid_beta+(1|manifest)+cngeneson+pc_mito+brain_region+Sex	0.217	5850.000
ENSG00000180957	0.019	0.034	0.137	-0.068	PITPNB	0.057	~amyloid_beta+(1|manifest)+cngeneson+pc_mito+brain_region+Sex	0.567	5190.000
ENSG00000133703	0.077	0.034	0.080	-0.011	KRAS	0.132	~amyloid_beta+(1|manifest)+cngeneson+pc_mito+brain_region+Sex	0.313	5705.000
ENSG00000112033	0.039	0.034	0.102	-0.033	PPARD	0.086	~amyloid_beta+(1|manifest)+cngeneson+pc_mito+brain_region+Sex	0.192	5882.000
ENSG00000100239	0.075	0.034	0.217	-0.148	PPP6R2	0.129	~amyloid_beta+(1|manifest)+cngeneson+pc_mito+brain_region+Sex	2.630	1788.000
ENSG00000007541	0.155	0.034	0.285	-0.216	PIGQ	0.211	~amyloid_beta+(1|manifest)+cngeneson+pc_mito+brain_region+Sex	3.198	1426.000
ENSG00000122257	0.011	0.034	0.198	-0.130	RBBP6	0.042	~amyloid_beta+(1|manifest)+cngeneson+pc_mito+brain_region+Sex	1.998	2433.000
ENSG00000128654	0.252	0.034	0.209	-0.141	MTX2	0.306	~amyloid_beta+(1|manifest)+cngeneson+pc_mito+brain_region+Sex	0.886	4411.000
ENSG00000151276	0.982	0.034	0.420	-0.352	MAGI1	0.984	~amyloid_beta+(1|manifest)+cngeneson+pc_mito+brain_region+Sex	19.987	53.000
ENSG00000183808	0.083	0.034	0.132	-0.064	RBM12B	0.138	~amyloid_beta+(1|manifest)+cngeneson+pc_mito+brain_region+Sex	1.306	3489.000
ENSG00000101871	0.319	0.034	0.299	-0.232	MID1	0.371	~amyloid_beta+(1|manifest)+cngeneson+pc_mito+brain_region+Sex	2.311	2089.000
ENSG00000135899	0.093	0.034	0.304	-0.236	SP110	0.149	~amyloid_beta+(1|manifest)+cngeneson+pc_mito+brain_region+Sex	2.563	1845.000
ENSG00000267680	0.030	0.034	0.110	-0.042	ZNF224	0.074	~amyloid_beta+(1|manifest)+cngeneson+pc_mito+brain_region+Sex	0.118	5944.000
ENSG00000074319	0.066	0.034	0.129	-0.061	TSG101	0.119	~amyloid_beta+(1|manifest)+cngeneson+pc_mito+brain_region+Sex	0.502	5354.000
ENSG00000069966	0.022	0.034	0.143	-0.076	GNB5	0.063	~amyloid_beta+(1|manifest)+cngeneson+pc_mito+brain_region+Sex	0.711	4845.000
ENSG00000119698	0.017	0.034	0.299	-0.231	PPP4R4	0.053	~amyloid_beta+(1|manifest)+cngeneson+pc_mito+brain_region+Sex	1.470	3183.000
ENSG00000166619	0.170	0.034	0.128	-0.061	BLCAP	0.226	~amyloid_beta+(1|manifest)+cngeneson+pc_mito+brain_region+Sex	0.694	4909.000
ENSG00000011258	0.033	0.034	0.182	-0.115	MBTD1	0.078	~amyloid_beta+(1|manifest)+cngeneson+pc_mito+brain_region+Sex	0.477	5406.000
ENSG00000162980	0.092	0.034	0.171	-0.103	ARL5A	0.148	~amyloid_beta+(1|manifest)+cngeneson+pc_mito+brain_region+Sex	0.694	4910.000
ENSG00000176102	0.076	0.034	0.192	-0.125	CSTF3	0.130	~amyloid_beta+(1|manifest)+cngeneson+pc_mito+brain_region+Sex	0.267	5785.000
ENSG00000182901	0.693	0.034	0.570	-0.503	RGS7	0.723	~amyloid_beta+(1|manifest)+cngeneson+pc_mito+brain_region+Sex	8.955	317.000
ENSG00000101350	0.010	0.034	0.092	-0.025	KIF3B	0.039	~amyloid_beta+(1|manifest)+cngeneson+pc_mito+brain_region+Sex	0.378	5599.000
ENSG00000187609	0.101	0.033	0.280	-0.214	EXD3	0.156	~amyloid_beta+(1|manifest)+cngeneson+pc_mito+brain_region+Sex	3.161	1447.000
ENSG00000156687	0.802	0.033	0.346	-0.279	UNC5D	0.822	~amyloid_beta+(1|manifest)+cngeneson+pc_mito+brain_region+Sex	2.388	2004.000
ENSG00000167548	0.104	0.033	0.151	-0.085	KMT2D	0.160	~amyloid_beta+(1|manifest)+cngeneson+pc_mito+brain_region+Sex	1.359	3394.000
ENSG00000174718	0.021	0.033	0.168	-0.102	RESF1	0.061	~amyloid_beta+(1|manifest)+cngeneson+pc_mito+brain_region+Sex	1.156	3800.000
ENSG00000060749	0.037	0.033	0.134	-0.068	QSER1	0.083	~amyloid_beta+(1|manifest)+cngeneson+pc_mito+brain_region+Sex	0.929	4304.000
ENSG00000119383	0.143	0.033	0.239	-0.173	PTPA	0.199	~amyloid_beta+(1|manifest)+cngeneson+pc_mito+brain_region+Sex	1.338	3431.000
ENSG00000100227	0.093	0.033	0.142	-0.077	POLDIP3	0.149	~amyloid_beta+(1|manifest)+cngeneson+pc_mito+brain_region+Sex	1.101	3929.000
ENSG00000188554	0.034	0.033	0.090	-0.024	NBR1	0.080	~amyloid_beta+(1|manifest)+cngeneson+pc_mito+brain_region+Sex	0.441	5480.000
ENSG00000213020	0.032	0.033	0.136	-0.071	ZNF611	0.077	~amyloid_beta+(1|manifest)+cngeneson+pc_mito+brain_region+Sex	0.994	4152.000
ENSG00000156113	0.750	0.033	0.164	-0.099	KCNMA1	0.775	~amyloid_beta+(1|manifest)+cngeneson+pc_mito+brain_region+Sex	6.615	514.000
ENSG00000115808	0.017	0.033	0.147	-0.082	STRN	0.053	~amyloid_beta+(1|manifest)+cngeneson+pc_mito+brain_region+Sex	0.989	4167.000
ENSG00000105607	0.197	0.032	0.237	-0.172	GCDH	0.251	~amyloid_beta+(1|manifest)+cngeneson+pc_mito+brain_region+Sex	2.389	2003.000
ENSG00000198818	0.050	0.032	0.114	-0.050	SFT2D1	0.099	~amyloid_beta+(1|manifest)+cngeneson+pc_mito+brain_region+Sex	0.201	5870.000
ENSG00000166402	0.033	0.032	0.179	-0.114	TUB	0.078	~amyloid_beta+(1|manifest)+cngeneson+pc_mito+brain_region+Sex	0.380	5598.000
ENSG00000255872	0.135	0.032	0.132	-0.068	[illegible]	0.192	~amyloid_beta+(1|manifest)+cngeneson+pc_mito+brain_region+Sex	1.167	3776.000
ENSG00000198492	0.086	0.032	0.121	-0.056	YTHDF2	0.141	~amyloid_beta+(1|manifest)+cngeneson+pc_mito+brain_region+Sex	0.151	5921.000
ENSG00000111203	0.183	0.032	0.272	-0.207	ITFG2	0.237	~amyloid_beta+(1|manifest)+cngeneson+pc_mito+brain_region+Sex	3.084	1492.000
ENSG00000156026	0.133	0.032	0.134	-0.069	MCU	0.190	~amyloid_beta+(1|manifest)+cngeneson+pc_mito+brain_region+Sex	0.981	4184.000
ENSG00000136205	0.436	0.032	0.465	-0.401	TNS3	0.482	~amyloid_beta+(1|manifest)+cngeneson+pc_mito+brain_region+Sex	5.795	625.000
ENSG00000151247	0.087	0.032	0.132	-0.068	EIF4E	0.142	~amyloid_beta+(1|manifest)+cngeneson+pc_mito+brain_region+Sex	0.711	4842.000
ENSG00000165097	0.034	0.032	0.076	-0.012	KDM1B	0.079	~amyloid_beta+(1|manifest)+cngeneson+pc_mito+brain_region+Sex	0.543	5250.000
ENSG00000197442	0.780	0.032	0.124	-0.060	MAP3K5	0.801	~amyloid_beta+(1|manifest)+cngeneson+pc_mito+brain_region+Sex	6.486	526.000
ENSG00000010818	0.409	0.032	0.337	-0.273	HIVEP2	0.456	~amyloid_beta+(1|manifest)+cngeneson+pc_mito+brain_region+Sex	2.214	2186.000
ENSG00000154856	0.531	0.032	0.519	-0.455	APCDD1	0.573	~amyloid_beta+(1|manifest)+cngeneson+pc_mito+brain_region+Sex	6.120	578.000
ENSG00000064419	0.012	0.032	0.163	-0.100	TNPO3	0.044	~amyloid_beta+(1|manifest)+cngeneson+pc_mito+brain_region+Sex	0.600	5131.000
ENSG00000014164	0.035	0.032	0.092	-0.029	ZC3H3	0.081	~amyloid_beta+(1|manifest)+cngeneson+pc_mito+brain_region+Sex	0.411	5546.000
ENSG00000171016	0.139	0.032	0.146	-0.082	PYGO1	0.196	~amyloid_beta+(1|manifest)+cngeneson+pc_mito+brain_region+Sex	0.319	5696.000
ENSG00000172992	0.050	0.032	0.231	-0.168	DCAKD	0.099	~amyloid_beta+(1|manifest)+cngeneson+pc_mito+brain_region+Sex	1.585	3005.000
ENSG00000204178	0.019	0.032	0.096	-0.033	MACO1	0.057	~amyloid_beta+(1|manifest)+cngeneson+pc_mito+brain_region+Sex	0.638	5033.000
ENSG00000108306	0.000	0.032	0.158	-0.095	FBXL20	0.005	~amyloid_beta+(1|manifest)+cngeneson+pc_mito+brain_region+Sex	0.649	5002.000
ENSG00000130348	0.024	0.032	0.106	-0.043	QRSL1	0.066	~amyloid_beta+(1|manifest)+cngeneson+pc_mito+brain_region+Sex	0.962	4217.000
ENSG00000241973	0.089	0.031	0.231	-0.168	PI4KA	0.144	~amyloid_beta+(1|manifest)+cngeneson+pc_mito+brain_region+Sex	2.852	1634.000
ENSG00000095015	0.028	0.031	0.087	-0.024	MAP3K1	0.071	~amyloid_beta+(1|manifest)+cngeneson+pc_mito+brain_region+Sex	0.319	5694.000
ENSG00000146143	0.530	0.031	0.206	-0.144	PRIM2	0.572	~amyloid_beta+(1|manifest)+cngeneson+pc_mito+brain_region+Sex	1.032	4067.000
ENSG00000142864	0.051	0.031	0.164	-0.101	SERBP1	0.101	~amyloid_beta+(1|manifest)+cngeneson+pc_mito+brain_region+Sex	1.125	3874.000
ENSG00000198663	0.063	0.031	0.142	-0.080	C6orf89	0.116	~amyloid_beta+(1|manifest)+cngeneson+pc_mito+brain_region+Sex	1.031	4072.000
ENSG00000149499	0.125	0.031	0.325	-0.262	EML3	0.181	~amyloid_beta+(1|manifest)+cngeneson+pc_mito+brain_region+Sex	2.621	1797.000
ENSG00000183726	0.036	0.031	0.127	-0.064	TMEM50A	0.082	~amyloid_beta+(1|manifest)+cngeneson+pc_mito+brain_region+Sex	0.167	5909.000
ENSG00000164054	0.185	0.031	0.452	-0.390	SHISA5	0.239	~amyloid_beta+(1|manifest)+cngeneson+pc_mito+brain_region+Sex	5.280	713.000
ENSG00000133393	0.031	0.031	0.080	-0.018	FOPNL	0.075	~amyloid_beta+(1|manifest)+cngeneson+pc_mito+brain_region+Sex	0.287	5750.000
ENSG00000153214	0.062	0.031	0.216	-0.154	TMEM87B	0.114	~amyloid_beta+(1|manifest)+cngeneson+pc_mito+brain_region+Sex	2.648	1771.000
ENSG00000244405	0.505	0.031	0.195	-0.133	ETV5	0.548	~amyloid_beta+(1|manifest)+cngeneson+pc_mito+brain_region+Sex	0.665	4968.000
ENSG00000143603	0.955	0.031	0.255	-0.193	KCNN3	0.960	~amyloid_beta+(1|manifest)+cngeneson+pc_mito+brain_region+Sex	13.716	129.000
ENSG00000090316	0.019	0.031	0.117	-0.055	MAEA	0.057	~amyloid_beta+(1|manifest)+cngeneson+pc_mito+brain_region+Sex	0.224	5843.000
ENSG00000059145	0.032	0.031	0.191	-0.130	UNKL	0.076	~amyloid_beta+(1|manifest)+cngeneson+pc_mito+brain_region+Sex	1.763	2732.000
ENSG00000104442	0.065	0.031	0.121	-0.060	ARMC1	0.117	~amyloid_beta+(1|manifest)+cngeneson+pc_mito+brain_region+Sex	0.328	5678.000
ENSG00000131374	0.716	0.031	0.120	-0.058	TBC1D5	0.743	~amyloid_beta+(1|manifest)+cngeneson+pc_mito+brain_region+Sex	3.265	1388.000
ENSG00000164124	0.154	0.031	0.394	-0.333	TMEM144	0.209	~amyloid_beta+(1|manifest)+cngeneson+pc_mito+brain_region+Sex	1.618	2955.000
ENSG00000046651	0.187	0.030	0.202	-0.141	OFD1	0.241	~amyloid_beta+(1|manifest)+cngeneson+pc_mito+brain_region+Sex	0.471	5416.000
ENSG00000159461	0.038	0.030	0.145	-0.084	AMFR	0.085	~amyloid_beta+(1|manifest)+cngeneson+pc_mito+brain_region+Sex	0.742	4762.000
ENSG00000070367	0.085	0.030	0.143	-0.082	EXOC5	0.140	~amyloid_beta+(1|manifest)+cngeneson+pc_mito+brain_region+Sex	0.464	5429.000
ENSG00000134532	0.644	0.030	0.160	-0.100	SOX5	0.677	~amyloid_beta+(1|manifest)+cngeneson+pc_mito+brain_region+Sex	8.802	325.000
ENSG00000132405	0.032	0.030	0.143	-0.083	TBC1D14	0.077	~amyloid_beta+(1|manifest)+cngeneson+pc_mito+brain_region+Sex	1.056	4011.000
ENSG00000130338	0.100	0.030	0.129	-0.069	TULP4	0.155	~amyloid_beta+(1|manifest)+cngeneson+pc_mito+brain_region+Sex	0.624	5073.000
ENSG00000134461	0.024	0.030	0.141	-0.081	ANKRD16	0.065	~amyloid_beta+(1|manifest)+cngeneson+pc_mito+brain_region+Sex	1.134	3853.000
ENSG00000111271	0.047	0.030	0.148	-0.088	ACAD10	0.096	~amyloid_beta+(1|manifest)+cngeneson+pc_mito+brain_region+Sex	1.091	3946.000
ENSG00000152484	0.013	0.030	0.153	-0.093	USP12	0.047	~amyloid_beta+(1|manifest)+cngeneson+pc_mito+brain_region+Sex	1.131	3861.000
ENSG00000138777	0.387	0.030	0.226	-0.167	PPA2	0.436	~amyloid_beta+(1|manifest)+cngeneson+pc_mito+brain_region+Sex	0.642	5027.000
ENSG00000183044	0.217	0.030	0.352	-0.293	ABAT	0.272	~amyloid_beta+(1|manifest)+cngeneson+pc_mito+brain_region+Sex	3.043	1518.000
ENSG00000089472	0.071	0.030	0.239	-0.180	HEPH	0.125	~amyloid_beta+(1|manifest)+cngeneson+pc_mito+brain_region+Sex	1.313	3475.000
ENSG00000175224	0.005	0.030	0.132	-0.073	ATG13	0.027	~amyloid_beta+(1|manifest)+cngeneson+pc_mito+brain_region+Sex	1.036	4057.000
ENSG00000232119	0.028	0.029	0.118	-0.059	MCTS1	0.071	~amyloid_beta+(1|manifest)+cngeneson+pc_mito+brain_region+Sex	0.507	5334.000
ENSG00000175105	0.022	0.029	0.162	-0.104	ZNF654	0.063	~amyloid_beta+(1|manifest)+cngeneson+pc_mito+brain_region+Sex	1.834	2651.000
ENSG00000137642	0.563	0.029	0.196	-0.138	SORL1	0.603	~amyloid_beta+(1|manifest)+cngeneson+pc_mito+brain_region+Sex	2.265	2138.000
ENSG00000114988	0.020	0.029	0.099	-0.040	LMAN2L	0.060	~amyloid_beta+(1|manifest)+cngeneson+pc_mito+brain_region+Sex	0.255	5798.000
ENSG00000126067	0.025	0.029	0.083	-0.025	PSMB2	0.067	~amyloid_beta+(1|manifest)+cngeneson+pc_mito+brain_region+Sex	0.206	5867.000
ENSG00000109790	0.026	0.029	0.186	-0.128	KLHL5	0.068	~amyloid_beta+(1|manifest)+cngeneson+pc_mito+brain_region+Sex	1.188	3733.000
ENSG00000122958	0.028	0.029	0.089	-0.031	VPS26A	0.072	~amyloid_beta+(1|manifest)+cngeneson+pc_mito+brain_region+Sex	0.478	5404.000
ENSG00000136807	0.278	0.029	0.306	-0.248	CDK9	0.331	~amyloid_beta+(1|manifest)+cngeneson+pc_mito+brain_region+Sex	7.203	451.000
ENSG00000122376	0.440	0.029	0.115	-0.057	SHLD2	0.485	~amyloid_beta+(1|manifest)+cngeneson+pc_mito+brain_region+Sex	0.327	5681.000
ENSG00000132326	0.030	0.029	0.221	-0.164	PER2	0.074	~amyloid_beta+(1|manifest)+cngeneson+pc_mito+brain_region+Sex	2.294	2115.000
ENSG00000175155	0.030	0.029	0.122	-0.065	YPEL2	0.074	~amyloid_beta+(1|manifest)+cngeneson+pc_mito+brain_region+Sex	0.188	5891.000
ENSG00000152700	0.172	0.029	0.120	-0.063	SAR1B	0.227	~amyloid_beta+(1|manifest)+cngeneson+pc_mito+brain_region+Sex	0.680	4941.000
ENSG00000205707	0.048	0.029	0.110	-0.052	ETFRF1	0.098	~amyloid_beta+(1|manifest)+cngeneson+pc_mito+brain_region+Sex	0.229	5836.000
ENSG00000091164	0.019	0.028	0.118	-0.061	TXNL1	0.057	~amyloid_beta+(1|manifest)+cngeneson+pc_mito+brain_region+Sex	0.644	5020.000
ENSG00000128891	0.056	0.028	0.193	-0.136	CCDC32	0.107	~amyloid_beta+(1|manifest)+cngeneson+pc_mito+brain_region+Sex	2.876	1623.000
ENSG00000159788	0.212	0.028	0.315	-0.258	RGS12	0.267	~amyloid_beta+(1|manifest)+cngeneson+pc_mito+brain_region+Sex	2.687	1738.000
ENSG00000163406	0.806	0.028	0.460	-0.403	SLC15A2	0.826	~amyloid_beta+(1|manifest)+cngeneson+pc_mito+brain_region+Sex	7.122	459.000
ENSG00000101544	0.014	0.028	0.176	-0.119	ADNP2	0.048	~amyloid_beta+(1|manifest)+cngeneson+pc_mito+brain_region+Sex	0.246	5813.000
ENSG00000135090	0.696	0.028	0.161	-0.104	TAOK3	0.726	~amyloid_beta+(1|manifest)+cngeneson+pc_mito+brain_region+Sex	2.212	2190.000
ENSG00000169084	0.279	0.028	0.267	-0.210	DHRSX	0.332	~amyloid_beta+(1|manifest)+cngeneson+pc_mito+brain_region+Sex	4.355	930.000
ENSG00000148153	0.094	0.028	0.067	-0.012	INIP	0.149	~amyloid_beta+(1|manifest)+cngeneson+pc_mito+brain_region+Sex	0.294	5742.000
ENSG00000061794	0.056	0.027	0.054	0.001	MRPS35	0.106	~amyloid_beta+(1|manifest)+cngeneson+pc_mito+brain_region+Sex	0.316	5702.000
ENSG00000148154	0.552	0.027	0.358	-0.304	UGCG	0.593	~amyloid_beta+(1|manifest)+cngeneson+pc_mito+brain_region+Sex	6.862	485.000
ENSG00000197969	0.008	0.027	0.155	-0.101	VPS13A	0.035	~amyloid_beta+(1|manifest)+cngeneson+pc_mito+brain_region+Sex	1.486	3167.000
ENSG00000123130	0.032	0.027	0.131	-0.077	ACOT9	0.077	~amyloid_beta+(1|manifest)+cngeneson+pc_mito+brain_region+Sex	0.854	4488.000
ENSG00000104447	0.469	0.027	0.169	-0.115	TRPS1	0.514	~amyloid_beta+(1|manifest)+cngeneson+pc_mito+brain_region+Sex	6.724	504.000
ENSG00000105948	0.045	0.027	0.124	-0.071	TTC26	0.093	~amyloid_beta+(1|manifest)+cngeneson+pc_mito+brain_region+Sex	0.225	5841.000
ENSG00000186001	0.028	0.027	0.154	-0.101	LRCH3	0.071	~amyloid_beta+(1|manifest)+cngeneson+pc_mito+brain_region+Sex	1.314	3473.000
ENSG00000197798	0.123	0.027	0.114	-0.060	FAM118B	0.179	~amyloid_beta+(1|manifest)+cngeneson+pc_mito+brain_region+Sex	1.420	3293.000
ENSG00000119711	0.105	0.026	0.189	-0.137	ALDH6A1	0.161	~amyloid_beta+(1|manifest)+cngeneson+pc_mito+brain_region+Sex	1.186	3736.000
ENSG00000090975	0.546	0.026	0.395	-0.343	PITPNM2	0.586	~amyloid_beta+(1|manifest)+cngeneson+pc_mito+brain_region+Sex	8.023	375.000
ENSG00000135829	0.041	0.026	0.144	-0.092	DHX9	0.089	~amyloid_beta+(1|manifest)+cngeneson+pc_mito+brain_region+Sex	1.552	3047.000
ENSG00000175073	0.019	0.026	0.088	-0.036	VCPIP1	0.057	~amyloid_beta+(1|manifest)+cngeneson+pc_mito+brain_region+Sex	0.106	5949.000
ENSG00000147996	0.128	0.026	0.098	-0.046	CBWD5	0.184	~amyloid_beta+(1|manifest)+cngeneson+pc_mito+brain_region+Sex	0.574	5176.000
ENSG00000155034	0.137	0.026	0.121	-0.069	FBXL18	0.194	~amyloid_beta+(1|manifest)+cngeneson+pc_mito+brain_region+Sex	0.777	4673.000
ENSG00000163820	0.011	0.026	0.093	-0.041	FYCO1	0.043	~amyloid_beta+(1|manifest)+cngeneson+pc_mito+brain_region+Sex	0.317	5699.000
ENSG00000113068	0.073	0.026	0.115	-0.063	PFDN1	0.127	~amyloid_beta+(1|manifest)+cngeneson+pc_mito+brain_region+Sex	0.780	4667.000
ENSG00000163961	0.050	0.026	0.179	-0.127	RNF168	0.100	~amyloid_beta+(1|manifest)+cngeneson+pc_mito+brain_region+Sex	1.577	3015.000
ENSG00000102710	0.085	0.026	0.146	-0.095	SUPT20H	0.140	~amyloid_beta+(1|manifest)+cngeneson+pc_mito+brain_region+Sex	0.273	5776.000
ENSG00000119820	0.213	0.025	0.175	-0.124	YIPF4	0.267	~amyloid_beta+(1|manifest)+cngeneson+pc_mito+brain_region+Sex	0.962	4221.000
ENSG00000127080	0.040	0.025	0.093	-0.042	IPPK	0.088	~amyloid_beta+(1|manifest)+cngeneson+pc_mito+brain_region+Sex	0.445	5467.000
ENSG00000102038	0.205	0.025	0.265	-0.215	SMARCA1	0.260	~amyloid_beta+(1|manifest)+cngeneson+pc_mito+brain_region+Sex	2.087	2327.000
ENSG00000143457	0.092	0.025	0.101	-0.050	GOLPH3L	0.147	~amyloid_beta+(1|manifest)+cngeneson+pc_mito+brain_region+Sex	0.404	5558.000
ENSG00000143870	0.118	0.025	0.203	-0.152	PDIA6	0.174	~amyloid_beta+(1|manifest)+cngeneson+pc_mito+brain_region+Sex	0.241	5822.000
ENSG00000181929	0.008	0.025	0.116	-0.066	PRKAG1	0.035	~amyloid_beta+(1|manifest)+cngeneson+pc_mito+brain_region+Sex	0.700	4888.000
ENSG00000143437	0.066	0.025	0.168	-0.118	ARNT	0.119	~amyloid_beta+(1|manifest)+cngeneson+pc_mito+brain_region+Sex	0.713	4836.000
ENSG00000175556	0.165	0.025	0.144	-0.094	LONRF3	0.221	~amyloid_beta+(1|manifest)+cngeneson+pc_mito+brain_region+Sex	1.542	3061.000
ENSG00000204852	0.034	0.025	0.183	-0.133	TCTN1	0.080	~amyloid_beta+(1|manifest)+cngeneson+pc_mito+brain_region+Sex	0.211	5861.000
ENSG00000174579	0.018	0.025	0.146	-0.097	MSL2	0.056	~amyloid_beta+(1|manifest)+cngeneson+pc_mito+brain_region+Sex	0.729	4796.000
ENSG00000165209	0.279	0.025	0.253	-0.204	STRBP	0.332	~amyloid_beta+(1|manifest)+cngeneson+pc_mito+brain_region+Sex	1.645	2914.000
ENSG00000156304	0.018	0.025	0.115	-0.066	SCAF4	0.055	~amyloid_beta+(1|manifest)+cngeneson+pc_mito+brain_region+Sex	0.643	5025.000
ENSG00000040199	0.168	0.025	0.146	-0.096	PHLPP2	0.223	~amyloid_beta+(1|manifest)+cngeneson+pc_mito+brain_region+Sex	1.213	3675.000
ENSG00000189283	0.852	0.025	0.135	-0.085	FHIT	0.867	~amyloid_beta+(1|manifest)+cngeneson+pc_mito+brain_region+Sex	2.200	2203.000
ENSG00000102781	0.030	0.025	0.236	-0.187	KATNAL1	0.074	~amyloid_beta+(1|manifest)+cngeneson+pc_mito+brain_region+Sex	1.159	3792.000
ENSG00000179532	0.299	0.024	0.342	-0.293	DNHD1	0.352	~amyloid_beta+(1|manifest)+cngeneson+pc_mito+brain_region+Sex	3.621	1194.000
ENSG00000113615	0.040	0.024	0.124	-0.075	SEC24A	0.087	~amyloid_beta+(1|manifest)+cngeneson+pc_mito+brain_region+Sex	0.725	4808.000
ENSG00000122406	0.046	0.024	0.185	-0.136	RPL5	0.095	~amyloid_beta+(1|manifest)+cngeneson+pc_mito+brain_region+Sex	2.042	2379.000
ENSG00000006451	0.068	0.024	0.097	-0.048	RALA	0.122	~amyloid_beta+(1|manifest)+cngeneson+pc_mito+brain_region+Sex	0.317	5700.000
ENSG00000187676	0.412	0.024	0.352	-0.304	B3GLCT	0.458	~amyloid_beta+(1|manifest)+cngeneson+pc_mito+brain_region+Sex	3.245	1399.000
ENSG00000105983	0.038	0.024	0.197	-0.148	LMBR1	0.086	~amyloid_beta+(1|manifest)+cngeneson+pc_mito+brain_region+Sex	2.436	1947.000
ENSG00000120705	0.013	0.024	0.095	-0.047	ETF1	0.047	~amyloid_beta+(1|manifest)+cngeneson+pc_mito+brain_region+Sex	0.313	5709.000
ENSG00000113638	0.023	0.024	0.100	-0.052	TTC33	0.065	~amyloid_beta+(1|manifest)+cngeneson+pc_mito+brain_region+Sex	0.551	5228.000
ENSG00000177311	0.029	0.024	0.191	-0.143	ZBTB38	0.073	~amyloid_beta+(1|manifest)+cngeneson+pc_mito+brain_region+Sex	1.238	3620.000
ENSG00000198947	0.584	0.024	0.282	-0.234	DMD	0.622	~amyloid_beta+(1|manifest)+cngeneson+pc_mito+brain_region+Sex	2.411	1973.000
ENSG00000270647	0.248	0.024	0.194	-0.146	TAF15	0.302	~amyloid_beta+(1|manifest)+cngeneson+pc_mito+brain_region+Sex	5.499	680.000
ENSG00000131100	0.052	0.024	0.102	-0.054	ATP6V1E1	0.102	~amyloid_beta+(1|manifest)+cngeneson+pc_mito+brain_region+Sex	0.719	4825.000
ENSG00000132792	0.016	0.024	0.122	-0.075	CTNNBL1	0.052	~amyloid_beta+(1|manifest)+cngeneson+pc_mito+brain_region+Sex	0.674	4955.000
ENSG00000177879	0.176	0.024	0.186	-0.139	AP3S1	0.230	~amyloid_beta+(1|manifest)+cngeneson+pc_mito+brain_region+Sex	0.111	5946.000
ENSG00000124479	0.018	0.024	0.187	-0.140	NDP	0.055	~amyloid_beta+(1|manifest)+cngeneson+pc_mito+brain_region+Sex	1.340	3429.000
ENSG00000129003	0.113	0.024	0.135	-0.088	VPS13C	0.169	~amyloid_beta+(1|manifest)+cngeneson+pc_mito+brain_region+Sex	0.813	4591.000
ENSG00000108395	0.074	0.023	0.123	-0.076	TRIM37	0.128	~amyloid_beta+(1|manifest)+cngeneson+pc_mito+brain_region+Sex	0.875	4426.000
ENSG00000113583	0.093	0.023	0.104	-0.057	C5orf15	0.149	~amyloid_beta+(1|manifest)+cngeneson+pc_mito+brain_region+Sex	0.521	5302.000
ENSG00000163754	0.003	0.023	0.142	-0.096	GYG1	0.020	~amyloid_beta+(1|manifest)+cngeneson+pc_mito+brain_region+Sex	0.835	4529.000
ENSG00000107242	0.144	0.023	0.206	-0.160	PIP5K1B	0.200	~amyloid_beta+(1|manifest)+cngeneson+pc_mito+brain_region+Sex	2.627	1791.000
ENSG00000139645	0.047	0.023	0.130	-0.084	ANKRD52	0.096	~amyloid_beta+(1|manifest)+cngeneson+pc_mito+brain_region+Sex	1.335	3440.000
ENSG00000167004	0.033	0.023	0.154	-0.108	PDIA3	0.078	~amyloid_beta+(1|manifest)+cngeneson+pc_mito+brain_region+Sex	0.437	5491.000
ENSG00000157538	0.078	0.023	0.137	-0.092	VPS26C	0.132	~amyloid_beta+(1|manifest)+cngeneson+pc_mito+brain_region+Sex	1.178	3749.000
ENSG00000168781	0.022	0.023	0.115	-0.069	PPIP5K1	0.063	~amyloid_beta+(1|manifest)+cngeneson+pc_mito+brain_region+Sex	0.067	5969.000
ENSG00000131375	0.018	0.023	0.147	-0.102	CAPN7	0.055	~amyloid_beta+(1|manifest)+cngeneson+pc_mito+brain_region+Sex	2.096	2318.000
ENSG00000197892	0.018	0.023	0.145	-0.100	KIF13B	0.057	~amyloid_beta+(1|manifest)+cngeneson+pc_mito+brain_region+Sex	1.681	2856.000
ENSG00000177150	0.114	0.023	0.089	-0.043	FAM210A	0.170	~amyloid_beta+(1|manifest)+cngeneson+pc_mito+brain_region+Sex	0.501	5356.000
ENSG00000103811	0.370	0.023	0.374	-0.329	CTSH	0.420	~amyloid_beta+(1|manifest)+cngeneson+pc_mito+brain_region+Sex	5.777	630.000
ENSG00000125686	0.033	0.022	0.080	-0.035	MED1	0.078	~amyloid_beta+(1|manifest)+cngeneson+pc_mito+brain_region+Sex	0.396	5570.000
ENSG00000175264	0.120	0.022	0.366	-0.321	CHST1	0.176	~amyloid_beta+(1|manifest)+cngeneson+pc_mito+brain_region+Sex	3.109	1478.000
ENSG00000197217	0.033	0.022	0.116	-0.072	ENTPD4	0.078	~amyloid_beta+(1|manifest)+cngeneson+pc_mito+brain_region+Sex	0.609	5110.000
ENSG00000065029	0.109	0.022	0.208	-0.164	ZNF76	0.165	~amyloid_beta+(1|manifest)+cngeneson+pc_mito+brain_region+Sex	2.215	2185.000
ENSG00000180304	0.095	0.022	0.083	-0.039	OAZ2	0.150	~amyloid_beta+(1|manifest)+cngeneson+pc_mito+brain_region+Sex	0.483	5398.000
ENSG00000125266	0.146	0.022	0.198	-0.154	EFNB2	0.202	~amyloid_beta+(1|manifest)+cngeneson+pc_mito+brain_region+Sex	1.643	2916.000
ENSG00000178252	0.323	0.022	0.391	-0.347	WDR6	0.375	~amyloid_beta+(1|manifest)+cngeneson+pc_mito+brain_region+Sex	6.137	575.000
ENSG00000140948	0.007	0.022	0.127	-0.083	ZCCHC14	0.033	~amyloid_beta+(1|manifest)+cngeneson+pc_mito+brain_region+Sex	0.916	4336.000
ENSG00000134884	0.472	0.022	0.290	-0.246	ARGLU1	0.517	~amyloid_beta+(1|manifest)+cngeneson+pc_mito+brain_region+Sex	10.271	260.000
ENSG00000129197	0.105	0.022	0.114	-0.070	RPAIN	0.161	~amyloid_beta+(1|manifest)+cngeneson+pc_mito+brain_region+Sex	0.896	4382.000
ENSG00000204628	0.245	0.022	0.187	-0.143	RACK1	0.299	~amyloid_beta+(1|manifest)+cngeneson+pc_mito+brain_region+Sex	1.998	2435.000
ENSG00000108349	0.038	0.022	0.160	-0.116	CASC3	0.085	~amyloid_beta+(1|manifest)+cngeneson+pc_mito+brain_region+Sex	1.101	3928.000
ENSG00000090487	0.054	0.022	0.135	-0.092	SPG21	0.104	~amyloid_beta+(1|manifest)+cngeneson+pc_mito+brain_region+Sex	1.095	3938.000
ENSG00000167491	0.189	0.022	0.200	-0.157	GATAD2A	0.244	~amyloid_beta+(1|manifest)+cngeneson+pc_mito+brain_region+Sex	2.055	2361.000
ENSG00000010270	0.029	0.022	0.084	-0.041	STARD3NL	0.073	~amyloid_beta+(1|manifest)+cngeneson+pc_mito+brain_region+Sex	0.305	5725.000
ENSG00000141699	0.138	0.022	0.121	-0.077	RETREG3	0.195	~amyloid_beta+(1|manifest)+cngeneson+pc_mito+brain_region+Sex	0.734	4784.000
ENSG00000108679	0.077	0.021	0.170	-0.128	LGALS3BP	0.132	~amyloid_beta+(1|manifest)+cngeneson+pc_mito+brain_region+Sex	0.464	5431.000
ENSG00000172466	0.052	0.021	0.156	-0.114	ZNF24	0.102	~amyloid_beta+(1|manifest)+cngeneson+pc_mito+brain_region+Sex	0.634	5049.000
ENSG00000258643	0.016	0.021	0.147	-0.104	[illegible]	0.053	~amyloid_beta+(1|manifest)+cngeneson+pc_mito+brain_region+Sex	0.848	4505.000
ENSG00000185246	0.024	0.021	0.081	-0.039	PRPF39	0.066	~amyloid_beta+(1|manifest)+cngeneson+pc_mito+brain_region+Sex	0.414	5537.000
ENSG00000169032	0.050	0.021	0.131	-0.089	MAP2K1	0.099	~amyloid_beta+(1|manifest)+cngeneson+pc_mito+brain_region+Sex	0.681	4936.000
ENSG00000100852	0.927	0.021	0.154	-0.112	ARHGAP5	0.936	~amyloid_beta+(1|manifest)+cngeneson+pc_mito+brain_region+Sex	4.699	839.000
ENSG00000105926	0.889	0.021	0.323	-0.281	MPP6	0.901	~amyloid_beta+(1|manifest)+cngeneson+pc_mito+brain_region+Sex	4.809	822.000
ENSG00000165091	0.833	0.021	0.175	-0.133	TMC1	0.850	~amyloid_beta+(1|manifest)+cngeneson+pc_mito+brain_region+Sex	0.675	4949.000
ENSG00000169139	0.015	0.021	0.092	-0.050	UBE2V2	0.050	~amyloid_beta+(1|manifest)+cngeneson+pc_mito+brain_region+Sex	0.086	5964.000
ENSG00000022277	0.018	0.021	0.068	-0.027	RTF2	0.056	~amyloid_beta+(1|manifest)+cngeneson+pc_mito+brain_region+Sex	0.328	5677.000
ENSG00000107949	0.126	0.021	0.088	-0.046	BCCIP	0.183	~amyloid_beta+(1|manifest)+cngeneson+pc_mito+brain_region+Sex	0.770	4690.000
ENSG00000184708	0.082	0.021	0.114	-0.072	EIF4ENIF1	0.137	~amyloid_beta+(1|manifest)+cngeneson+pc_mito+brain_region+Sex	1.011	4113.000
ENSG00000149639	0.043	0.021	0.205	-0.163	SOGA1	0.091	~amyloid_beta+(1|manifest)+cngeneson+pc_mito+brain_region+Sex	0.623	5074.000
ENSG00000100599	0.020	0.021	0.171	-0.129	RIN3	0.060	~amyloid_beta+(1|manifest)+cngeneson+pc_mito+brain_region+Sex	0.242	5818.000
ENSG00000156232	0.052	0.021	0.098	-0.057	WHAMM	0.101	~amyloid_beta+(1|manifest)+cngeneson+pc_mito+brain_region+Sex	0.871	4435.000
ENSG00000089234	0.049	0.021	0.069	-0.027	BRAP	0.098	~amyloid_beta+(1|manifest)+cngeneson+pc_mito+brain_region+Sex	0.370	5620.000
ENSG00000162869	0.108	0.021	0.108	-0.067	PPP1R21	0.163	~amyloid_beta+(1|manifest)+cngeneson+pc_mito+brain_region+Sex	1.451	3225.000
ENSG00000074054	0.930	0.021	0.131	-0.090	CLASP1	0.938	~amyloid_beta+(1|manifest)+cngeneson+pc_mito+brain_region+Sex	0.961	4223.000
ENSG00000163541	0.050	0.021	0.077	-0.036	SUCLG1	0.100	~amyloid_beta+(1|manifest)+cngeneson+pc_mito+brain_region+Sex	0.373	5610.000
ENSG00000100280	0.102	0.020	0.182	-0.141	AP1B1	0.157	~amyloid_beta+(1|manifest)+cngeneson+pc_mito+brain_region+Sex	1.278	3549.000
ENSG00000080815	0.069	0.020	0.124	-0.083	PSEN1	0.123	~amyloid_beta+(1|manifest)+cngeneson+pc_mito+brain_region+Sex	0.752	4736.000
ENSG00000006695	0.122	0.020	0.131	-0.090	COX10	0.179	~amyloid_beta+(1|manifest)+cngeneson+pc_mito+brain_region+Sex	1.261	3579.000
ENSG00000145782	0.093	0.020	0.066	-0.026	ATG12	0.148	~amyloid_beta+(1|manifest)+cngeneson+pc_mito+brain_region+Sex	0.282	5761.000
ENSG00000150510	0.088	0.020	0.275	-0.235	FAM124A	0.143	~amyloid_beta+(1|manifest)+cngeneson+pc_mito+brain_region+Sex	2.547	1858.000
ENSG00000124523	0.023	0.020	0.129	-0.089	SIRT5	0.065	~amyloid_beta+(1|manifest)+cngeneson+pc_mito+brain_region+Sex	1.230	3643.000
ENSG00000065609	0.061	0.020	0.172	-0.133	SNAP91	0.113	~amyloid_beta+(1|manifest)+cngeneson+pc_mito+brain_region+Sex	0.194	5878.000
ENSG00000081019	0.114	0.020	0.129	-0.089	RSBN1	0.170	~amyloid_beta+(1|manifest)+cngeneson+pc_mito+brain_region+Sex	0.532	5279.000
ENSG00000162337	0.161	0.020	0.153	-0.113	LRP5	0.216	~amyloid_beta+(1|manifest)+cngeneson+pc_mito+brain_region+Sex	1.680	2857.000
ENSG00000058866	0.911	0.020	0.266	-0.226	DGKG	0.921	~amyloid_beta+(1|manifest)+cngeneson+pc_mito+brain_region+Sex	11.269	209.000
ENSG00000187118	0.284	0.020	0.148	-0.109	CMC1	0.337	~amyloid_beta+(1|manifest)+cngeneson+pc_mito+brain_region+Sex	0.586	5158.000
ENSG00000113621	0.106	0.020	0.208	-0.169	TXNDC15	0.162	~amyloid_beta+(1|manifest)+cngeneson+pc_mito+brain_region+Sex	2.655	1765.000
ENSG00000168246	0.036	0.020	0.151	-0.111	UBTD2	0.082	~amyloid_beta+(1|manifest)+cngeneson+pc_mito+brain_region+Sex	1.010	4116.000
ENSG00000104812	0.158	0.020	0.223	-0.183	GYS1	0.213	~amyloid_beta+(1|manifest)+cngeneson+pc_mito+brain_region+Sex	2.265	2137.000
ENSG00000128059	0.024	0.020	0.091	-0.051	PPAT	0.065	~amyloid_beta+(1|manifest)+cngeneson+pc_mito+brain_region+Sex	0.592	5150.000
ENSG00000117713	0.630	0.019	0.128	-0.089	ARID1A	0.665	~amyloid_beta+(1|manifest)+cngeneson+pc_mito+brain_region+Sex	2.585	1823.000
ENSG00000116539	0.699	0.019	0.123	-0.084	ASH1L	0.728	~amyloid_beta+(1|manifest)+cngeneson+pc_mito+brain_region+Sex	3.119	1471.000
ENSG00000163512	0.037	0.019	0.142	-0.103	AZI2	0.084	~amyloid_beta+(1|manifest)+cngeneson+pc_mito+brain_region+Sex	0.178	5900.000
ENSG00000072364	0.106	0.019	0.233	-0.194	AFF4	0.162	~amyloid_beta+(1|manifest)+cngeneson+pc_mito+brain_region+Sex	3.341	1344.000
ENSG00000144468	0.124	0.019	0.223	-0.185	RHBDD1	0.181	~amyloid_beta+(1|manifest)+cngeneson+pc_mito+brain_region+Sex	1.215	3673.000
ENSG00000142207	0.080	0.019	0.121	-0.083	URB1	0.135	~amyloid_beta+(1|manifest)+cngeneson+pc_mito+brain_region+Sex	1.256	3588.000
ENSG00000086712	0.047	0.019	0.091	-0.053	TXLNG	0.095	~amyloid_beta+(1|manifest)+cngeneson+pc_mito+brain_region+Sex	1.104	3916.000
ENSG00000007866	0.055	0.019	0.151	-0.113	TEAD3	0.105	~amyloid_beta+(1|manifest)+cngeneson+pc_mito+brain_region+Sex	0.955	4239.000
ENSG00000013374	0.038	0.019	0.086	-0.048	NUB1	0.085	~amyloid_beta+(1|manifest)+cngeneson+pc_mito+brain_region+Sex	0.727	4803.000
ENSG00000138698	0.484	0.019	0.264	-0.226	RAP1GDS1	0.529	~amyloid_beta+(1|manifest)+cngeneson+pc_mito+brain_region+Sex	1.260	3584.000
ENSG00000137936	0.673	0.019	0.338	-0.300	BCAR3	0.704	~amyloid_beta+(1|manifest)+cngeneson+pc_mito+brain_region+Sex	2.431	1956.000
ENSG00000122779	0.027	0.019	0.152	-0.114	TRIM24	0.070	~amyloid_beta+(1|manifest)+cngeneson+pc_mito+brain_region+Sex	1.541	3062.000
ENSG00000130559	0.051	0.019	0.093	-0.055	CAMSAP1	0.101	~amyloid_beta+(1|manifest)+cngeneson+pc_mito+brain_region+Sex	0.655	4986.000
ENSG00000090581	0.053	0.019	0.113	-0.076	GNPTG	0.104	~amyloid_beta+(1|manifest)+cngeneson+pc_mito+brain_region+Sex	0.723	4814.000
ENSG00000163960	0.045	0.019	0.134	-0.097	UBXN7	0.094	~amyloid_beta+(1|manifest)+cngeneson+pc_mito+brain_region+Sex	0.927	4315.000
ENSG00000133657	0.261	0.019	0.279	-0.242	ATP13A3	0.315	~amyloid_beta+(1|manifest)+cngeneson+pc_mito+brain_region+Sex	5.108	751.000
ENSG00000043591	0.128	0.019	0.160	-0.123	ADRB1	0.184	~amyloid_beta+(1|manifest)+cngeneson+pc_mito+brain_region+Sex	1.361	3388.000
ENSG00000066629	0.008	0.018	0.110	-0.073	EML1	0.035	~amyloid_beta+(1|manifest)+cngeneson+pc_mito+brain_region+Sex	0.669	4964.000
ENSG00000175931	0.073	0.018	0.091	-0.055	UBE2O	0.126	~amyloid_beta+(1|manifest)+cngeneson+pc_mito+brain_region+Sex	0.736	4780.000
ENSG00000123636	0.779	0.018	0.127	-0.090	BAZ2B	0.800	~amyloid_beta+(1|manifest)+cngeneson+pc_mito+brain_region+Sex	4.045	1027.000
ENSG00000135932	0.028	0.018	0.142	-0.106	CAB39	0.071	~amyloid_beta+(1|manifest)+cngeneson+pc_mito+brain_region+Sex	1.000	4137.000
ENSG00000132153	0.179	0.018	0.132	-0.095	DHX30	0.233	~amyloid_beta+(1|manifest)+cngeneson+pc_mito+brain_region+Sex	0.817	4582.000
ENSG00000112941	0.184	0.018	0.167	-0.131	TENT4A	0.238	~amyloid_beta+(1|manifest)+cngeneson+pc_mito+brain_region+Sex	1.438	3251.000
ENSG00000163935	0.033	0.018	0.102	-0.066	SFMBT1	0.078	~amyloid_beta+(1|manifest)+cngeneson+pc_mito+brain_region+Sex	0.835	4530.000
ENSG00000065154	0.226	0.018	0.192	-0.156	OAT	0.281	~amyloid_beta+(1|manifest)+cngeneson+pc_mito+brain_region+Sex	1.803	2691.000
ENSG00000057935	0.099	0.018	0.151	-0.116	MTA3	0.154	~amyloid_beta+(1|manifest)+cngeneson+pc_mito+brain_region+Sex	0.590	5152.000
ENSG00000140545	0.443	0.018	0.362	-0.326	MFGE8	0.488	~amyloid_beta+(1|manifest)+cngeneson+pc_mito+brain_region+Sex	3.404	1314.000
ENSG00000148690	0.148	0.018	0.174	-0.139	FRA10AC1	0.204	~amyloid_beta+(1|manifest)+cngeneson+pc_mito+brain_region+Sex	0.584	5162.000
ENSG00000178691	0.069	0.018	0.100	-0.065	SUZ12	0.122	~amyloid_beta+(1|manifest)+cngeneson+pc_mito+brain_region+Sex	0.679	4942.000
ENSG00000124571	0.017	0.017	0.117	-0.082	XPO5	0.053	~amyloid_beta+(1|manifest)+cngeneson+pc_mito+brain_region+Sex	0.822	4564.000
ENSG00000158545	0.055	0.017	0.060	-0.026	ZC3H18	0.105	~amyloid_beta+(1|manifest)+cngeneson+pc_mito+brain_region+Sex	0.657	4982.000
ENSG00000109180	0.191	0.017	0.197	-0.162	OCIAD1	0.245	~amyloid_beta+(1|manifest)+cngeneson+pc_mito+brain_region+Sex	0.331	5672.000
ENSG00000100592	0.979	0.017	0.255	-0.220	DAAM1	0.982	~amyloid_beta+(1|manifest)+cngeneson+pc_mito+brain_region+Sex	14.538	112.000
ENSG00000140548	0.199	0.017	0.211	-0.176	ZNF710	0.253	~amyloid_beta+(1|manifest)+cngeneson+pc_mito+brain_region+Sex	1.187	3734.000
ENSG00000169398	0.843	0.017	0.077	-0.042	PTK2	0.858	~amyloid_beta+(1|manifest)+cngeneson+pc_mito+brain_region+Sex	1.962	2475.000
ENSG00000143416	0.217	0.017	0.387	-0.352	SELENBP1	0.272	~amyloid_beta+(1|manifest)+cngeneson+pc_mito+brain_region+Sex	4.075	1021.000
ENSG00000013561	0.072	0.017	0.094	-0.059	RNF14	0.126	~amyloid_beta+(1|manifest)+cngeneson+pc_mito+brain_region+Sex	0.537	5264.000
ENSG00000141232	0.638	0.017	0.238	-0.204	TOB1	0.672	~amyloid_beta+(1|manifest)+cngeneson+pc_mito+brain_region+Sex	1.043	4041.000
ENSG00000101605	0.153	0.017	0.485	-0.451	MYOM1	0.208	~amyloid_beta+(1|manifest)+cngeneson+pc_mito+brain_region+Sex	4.154	998.000
ENSG00000140750	0.025	0.017	0.176	-0.142	ARHGAP17	0.066	~amyloid_beta+(1|manifest)+cngeneson+pc_mito+brain_region+Sex	1.633	2935.000
ENSG00000070081	0.060	0.017	0.115	-0.081	NUCB2	0.111	~amyloid_beta+(1|manifest)+cngeneson+pc_mito+brain_region+Sex	0.931	4299.000
ENSG00000178913	0.053	0.017	0.111	-0.077	TAF7	0.103	~amyloid_beta+(1|manifest)+cngeneson+pc_mito+brain_region+Sex	0.488	5386.000
ENSG00000111897	0.072	0.017	0.137	-0.104	SERINC1	0.126	~amyloid_beta+(1|manifest)+cngeneson+pc_mito+brain_region+Sex	0.630	5059.000
ENSG00000130226	0.952	0.017	0.647	-0.613	DPP6	0.957	~amyloid_beta+(1|manifest)+cngeneson+pc_mito+brain_region+Sex	5.026	768.000
ENSG00000265491	0.081	0.017	0.126	-0.092	RNF115	0.135	~amyloid_beta+(1|manifest)+cngeneson+pc_mito+brain_region+Sex	1.123	3879.000
ENSG00000157212	0.102	0.017	0.054	-0.021	PAXIP1	0.157	~amyloid_beta+(1|manifest)+cngeneson+pc_mito+brain_region+Sex	0.668	4965.000
ENSG00000099889	0.177	0.017	0.255	-0.222	ARVCF	0.231	~amyloid_beta+(1|manifest)+cngeneson+pc_mito+brain_region+Sex	2.802	1665.000
ENSG00000221932	0.595	0.017	0.736	-0.703	HEPN1	0.632	~amyloid_beta+(1|manifest)+cngeneson+pc_mito+brain_region+Sex	10.583	243.000
ENSG00000090061	0.072	0.016	0.046	-0.013	CCNK	0.126	~amyloid_beta+(1|manifest)+cngeneson+pc_mito+brain_region+Sex	0.387	5583.000
ENSG00000149294	0.852	0.016	0.151	-0.118	NCAM1	0.867	~amyloid_beta+(1|manifest)+cngeneson+pc_mito+brain_region+Sex	6.890	480.000
ENSG00000177728	0.323	0.016	0.357	-0.324	TMEM94	0.375	~amyloid_beta+(1|manifest)+cngeneson+pc_mito+brain_region+Sex	4.317	944.000
ENSG00000253729	0.021	0.016	0.140	-0.107	PRKDC	0.062	~amyloid_beta+(1|manifest)+cngeneson+pc_mito+brain_region+Sex	1.540	3066.000
ENSG00000170581	0.279	0.016	0.200	-0.168	STAT2	0.332	~amyloid_beta+(1|manifest)+cngeneson+pc_mito+brain_region+Sex	3.468	1266.000
ENSG00000139505	0.007	0.016	0.078	-0.045	MTMR6	0.033	~amyloid_beta+(1|manifest)+cngeneson+pc_mito+brain_region+Sex	0.166	5910.000
ENSG00000100003	0.092	0.016	0.123	-0.091	SEC14L2	0.148	~amyloid_beta+(1|manifest)+cngeneson+pc_mito+brain_region+Sex	1.068	3994.000
ENSG00000084093	0.084	0.016	0.113	-0.081	REST	0.138	~amyloid_beta+(1|manifest)+cngeneson+pc_mito+brain_region+Sex	0.666	4967.000
ENSG00000198466	0.106	0.016	0.078	-0.046	ZNF587	0.162	~amyloid_beta+(1|manifest)+cngeneson+pc_mito+brain_region+Sex	0.515	5315.000
ENSG00000196419	0.054	0.016	0.094	-0.062	XRCC6	0.104	~amyloid_beta+(1|manifest)+cngeneson+pc_mito+brain_region+Sex	0.916	4337.000
ENSG00000138663	0.012	0.016	0.069	-0.037	COPS4	0.044	~amyloid_beta+(1|manifest)+cngeneson+pc_mito+brain_region+Sex	0.305	5724.000
ENSG00000121057	0.132	0.016	0.228	-0.196	AKAP1	0.189	~amyloid_beta+(1|manifest)+cngeneson+pc_mito+brain_region+Sex	1.292	3523.000
ENSG00000168118	0.354	0.016	0.195	-0.163	RAB4A	0.405	~amyloid_beta+(1|manifest)+cngeneson+pc_mito+brain_region+Sex	1.817	2675.000
ENSG00000159082	0.059	0.016	0.075	-0.043	SYNJ1	0.110	~amyloid_beta+(1|manifest)+cngeneson+pc_mito+brain_region+Sex	0.726	4804.000
ENSG00000166716	0.025	0.016	0.118	-0.086	ZNF592	0.066	~amyloid_beta+(1|manifest)+cngeneson+pc_mito+brain_region+Sex	0.834	4532.000
ENSG00000083168	0.481	0.016	0.221	-0.190	KAT6A	0.526	~amyloid_beta+(1|manifest)+cngeneson+pc_mito+brain_region+Sex	3.604	1204.000
ENSG00000110330	0.036	0.016	0.096	-0.065	BIRC2	0.083	~amyloid_beta+(1|manifest)+cngeneson+pc_mito+brain_region+Sex	0.620	5085.000
ENSG00000138069	0.185	0.016	0.246	-0.215	RAB1A	0.240	~amyloid_beta+(1|manifest)+cngeneson+pc_mito+brain_region+Sex	2.703	1725.000
ENSG00000188342	0.118	0.016	0.133	-0.101	GTF2F2	0.174	~amyloid_beta+(1|manifest)+cngeneson+pc_mito+brain_region+Sex	0.468	5424.000
ENSG00000153832	0.006	0.016	0.086	-0.054	FBXO36	0.030	~amyloid_beta+(1|manifest)+cngeneson+pc_mito+brain_region+Sex	0.327	5680.000
ENSG00000097007	0.266	0.016	0.158	-0.127	ABL1	0.320	~amyloid_beta+(1|manifest)+cngeneson+pc_mito+brain_region+Sex	1.454	3219.000
ENSG00000103351	0.086	0.016	0.099	-0.068	CLUAP1	0.141	~amyloid_beta+(1|manifest)+cngeneson+pc_mito+brain_region+Sex	0.762	4709.000
ENSG00000138442	0.211	0.016	0.083	-0.052	WDR12	0.266	~amyloid_beta+(1|manifest)+cngeneson+pc_mito+brain_region+Sex	0.727	4801.000
ENSG00000100603	0.018	0.015	0.072	-0.041	SNW1	0.056	~amyloid_beta+(1|manifest)+cngeneson+pc_mito+brain_region+Sex	0.675	4947.000
ENSG00000009954	0.023	0.015	0.218	-0.188	BAZ1B	0.065	~amyloid_beta+(1|manifest)+cngeneson+pc_mito+brain_region+Sex	1.698	2827.000
ENSG00000165322	0.230	0.015	0.123	-0.093	ARHGAP12	0.285	~amyloid_beta+(1|manifest)+cngeneson+pc_mito+brain_region+Sex	0.908	4358.000
ENSG00000138018	0.077	0.015	0.109	-0.079	SELENOI	0.131	~amyloid_beta+(1|manifest)+cngeneson+pc_mito+brain_region+Sex	1.468	3190.000
ENSG00000050130	0.083	0.015	0.107	-0.077	JKAMP	0.138	~amyloid_beta+(1|manifest)+cngeneson+pc_mito+brain_region+Sex	0.653	4992.000
ENSG00000134452	0.073	0.015	0.072	-0.042	FBH1	0.127	~amyloid_beta+(1|manifest)+cngeneson+pc_mito+brain_region+Sex	0.858	4471.000
ENSG00000176095	0.035	0.015	0.097	-0.068	IP6K1	0.081	~amyloid_beta+(1|manifest)+cngeneson+pc_mito+brain_region+Sex	0.499	5362.000
ENSG00000162378	0.036	0.015	0.116	-0.087	ZYG11B	0.082	~amyloid_beta+(1|manifest)+cngeneson+pc_mito+brain_region+Sex	1.025	4089.000
ENSG00000025800	0.032	0.015	0.104	-0.075	KPNA6	0.076	~amyloid_beta+(1|manifest)+cngeneson+pc_mito+brain_region+Sex	0.800	4630.000
ENSG00000131558	0.858	0.015	0.105	-0.075	EXOC4	0.872	~amyloid_beta+(1|manifest)+cngeneson+pc_mito+brain_region+Sex	1.448	3234.000
ENSG00000162520	0.045	0.014	0.116	-0.087	SYNC	0.094	~amyloid_beta+(1|manifest)+cngeneson+pc_mito+brain_region+Sex	1.315	3470.000
ENSG00000033627	0.016	0.014	0.214	-0.186	ATP6V0A1	0.052	~amyloid_beta+(1|manifest)+cngeneson+pc_mito+brain_region+Sex	0.842	4515.000
ENSG00000143384	0.170	0.014	0.240	-0.212	MCL1	0.226	~amyloid_beta+(1|manifest)+cngeneson+pc_mito+brain_region+Sex	1.801	2693.000
ENSG00000089094	0.155	0.014	0.183	-0.155	KDM2B	0.211	~amyloid_beta+(1|manifest)+cngeneson+pc_mito+brain_region+Sex	1.693	2832.000
ENSG00000099968	0.176	0.014	0.182	-0.154	BCL2L13	0.231	~amyloid_beta+(1|manifest)+cngeneson+pc_mito+brain_region+Sex	1.797	2705.000
ENSG00000112339	0.104	0.014	0.121	-0.093	HBS1L	0.160	~amyloid_beta+(1|manifest)+cngeneson+pc_mito+brain_region+Sex	0.810	4602.000
ENSG00000064726	0.042	0.014	0.098	-0.070	BTBD1	0.090	~amyloid_beta+(1|manifest)+cngeneson+pc_mito+brain_region+Sex	0.966	4212.000
ENSG00000154928	0.968	0.014	0.373	-0.345	EPHB1	0.971	~amyloid_beta+(1|manifest)+cngeneson+pc_mito+brain_region+Sex	8.509	343.000
ENSG00000140598	0.038	0.014	0.088	-0.060	EFL1	0.085	~amyloid_beta+(1|manifest)+cngeneson+pc_mito+brain_region+Sex	0.435	5493.000
ENSG00000154080	0.669	0.014	0.270	-0.242	CHST9	0.700	~amyloid_beta+(1|manifest)+cngeneson+pc_mito+brain_region+Sex	3.558	1223.000
ENSG00000131378	0.499	0.014	0.344	-0.316	RFTN1	0.543	~amyloid_beta+(1|manifest)+cngeneson+pc_mito+brain_region+Sex	4.798	824.000
ENSG00000106012	0.039	0.014	0.142	-0.114	IQCE	0.086	~amyloid_beta+(1|manifest)+cngeneson+pc_mito+brain_region+Sex	1.893	2567.000
ENSG00000162929	0.019	0.014	0.152	-0.124	KIAA1841	0.057	~amyloid_beta+(1|manifest)+cngeneson+pc_mito+brain_region+Sex	0.973	4197.000
ENSG00000148308	0.122	0.014	0.132	-0.104	GTF3C5	0.179	~amyloid_beta+(1|manifest)+cngeneson+pc_mito+brain_region+Sex	1.068	3995.000
ENSG00000153046	0.104	0.014	0.232	-0.205	CDYL	0.159	~amyloid_beta+(1|manifest)+cngeneson+pc_mito+brain_region+Sex	3.124	1467.000
ENSG00000115525	0.373	0.014	0.164	-0.137	ST3GAL5	0.423	~amyloid_beta+(1|manifest)+cngeneson+pc_mito+brain_region+Sex	2.414	1968.000
ENSG00000215301	0.425	0.013	0.213	-0.187	DDX3X	0.471	~amyloid_beta+(1|manifest)+cngeneson+pc_mito+brain_region+Sex	3.686	1169.000
ENSG00000169660	0.087	0.013	0.203	-0.177	HEXD	0.142	~amyloid_beta+(1|manifest)+cngeneson+pc_mito+brain_region+Sex	1.993	2443.000
ENSG00000099840	0.189	0.013	0.394	-0.367	IZUMO4	0.243	~amyloid_beta+(1|manifest)+cngeneson+pc_mito+brain_region+Sex	3.202	1423.000
ENSG00000145414	0.009	0.013	0.127	-0.101	NAF1	0.038	~amyloid_beta+(1|manifest)+cngeneson+pc_mito+brain_region+Sex	0.824	4555.000
ENSG00000168036	0.475	0.013	0.253	-0.227	CTNNB1	0.520	~amyloid_beta+(1|manifest)+cngeneson+pc_mito+brain_region+Sex	1.549	3050.000
ENSG00000108055	0.110	0.013	0.073	-0.046	SMC3	0.165	~amyloid_beta+(1|manifest)+cngeneson+pc_mito+brain_region+Sex	0.402	5561.000
ENSG00000115216	0.195	0.013	0.125	-0.099	NRBP1	0.250	~amyloid_beta+(1|manifest)+cngeneson+pc_mito+brain_region+Sex	1.261	3580.000
ENSG00000104133	0.069	0.013	0.129	-0.103	SPG11	0.122	~amyloid_beta+(1|manifest)+cngeneson+pc_mito+brain_region+Sex	2.149	2263.000
ENSG00000142599	0.919	0.013	0.077	-0.051	RERE	0.928	~amyloid_beta+(1|manifest)+cngeneson+pc_mito+brain_region+Sex	1.882	2586.000
ENSG00000178761	0.128	0.013	0.180	-0.154	FAM219B	0.185	~amyloid_beta+(1|manifest)+cngeneson+pc_mito+brain_region+Sex	1.199	3710.000
ENSG00000151327	0.093	0.013	0.103	-0.077	[illegible]	0.149	~amyloid_beta+(1|manifest)+cngeneson+pc_mito+brain_region+Sex	0.806	4610.000
ENSG00000169599	0.025	0.013	0.067	-0.041	NFU1	0.067	~amyloid_beta+(1|manifest)+cngeneson+pc_mito+brain_region+Sex	0.224	5844.000
ENSG00000154917	0.067	0.013	0.134	-0.109	RAB6B	0.120	~amyloid_beta+(1|manifest)+cngeneson+pc_mito+brain_region+Sex	0.450	5454.000
ENSG00000170633	0.132	0.013	0.065	-0.039	RNF34	0.189	~amyloid_beta+(1|manifest)+cngeneson+pc_mito+brain_region+Sex	0.549	5232.000
ENSG00000078124	0.215	0.013	0.167	-0.141	ACER3	0.269	~amyloid_beta+(1|manifest)+cngeneson+pc_mito+brain_region+Sex	1.840	2641.000
ENSG00000089289	0.073	0.013	0.076	-0.051	IGBP1	0.127	~amyloid_beta+(1|manifest)+cngeneson+pc_mito+brain_region+Sex	0.701	4883.000
ENSG00000137070	0.225	0.013	0.294	-0.268	IL11RA	0.280	~amyloid_beta+(1|manifest)+cngeneson+pc_mito+brain_region+Sex	2.804	1662.000
ENSG00000009844	0.027	0.013	0.094	-0.069	VTA1	0.071	~amyloid_beta+(1|manifest)+cngeneson+pc_mito+brain_region+Sex	0.648	5006.000
ENSG00000135698	0.102	0.013	0.124	-0.099	[illegible]	0.157	~amyloid_beta+(1|manifest)+cngeneson+pc_mito+brain_region+Sex	0.482	5399.000
ENSG00000196312	0.173	0.013	0.144	-0.119	MFSD14C	0.228	~amyloid_beta+(1|manifest)+cngeneson+pc_mito+brain_region+Sex	0.823	4559.000
ENSG00000135837	0.219	0.012	0.293	-0.268	CEP350	0.274	~amyloid_beta+(1|manifest)+cngeneson+pc_mito+brain_region+Sex	6.869	482.000
ENSG00000078140	0.142	0.012	0.173	-0.148	UBE2K	0.198	~amyloid_beta+(1|manifest)+cngeneson+pc_mito+brain_region+Sex	1.559	3040.000
ENSG00000074935	0.066	0.012	0.142	-0.117	TUBE1	0.118	~amyloid_beta+(1|manifest)+cngeneson+pc_mito+brain_region+Sex	0.176	5903.000
ENSG00000172845	0.091	0.012	0.137	-0.112	SP3	0.147	~amyloid_beta+(1|manifest)+cngeneson+pc_mito+brain_region+Sex	0.518	5308.000
ENSG00000134330	0.014	0.012	0.087	-0.063	IAH1	0.048	~amyloid_beta+(1|manifest)+cngeneson+pc_mito+brain_region+Sex	0.535	5269.000
ENSG00000114331	0.772	0.012	0.288	-0.263	ACAP2	0.795	~amyloid_beta+(1|manifest)+cngeneson+pc_mito+brain_region+Sex	7.225	448.000
ENSG00000114850	0.071	0.012	0.136	-0.112	SSR3	0.125	~amyloid_beta+(1|manifest)+cngeneson+pc_mito+brain_region+Sex	0.824	4558.000
ENSG00000113845	0.071	0.012	0.078	-0.054	TIMMDC1	0.125	~amyloid_beta+(1|manifest)+cngeneson+pc_mito+brain_region+Sex	0.549	5231.000
ENSG00000137513	0.210	0.012	0.096	-0.073	NARS2	0.264	~amyloid_beta+(1|manifest)+cngeneson+pc_mito+brain_region+Sex	0.690	4918.000
ENSG00000120616	0.586	0.012	0.142	-0.119	EPC1	0.623	~amyloid_beta+(1|manifest)+cngeneson+pc_mito+brain_region+Sex	2.635	1782.000
ENSG00000165507	0.943	0.012	0.345	-0.321	DEPP1	0.949	~amyloid_beta+(1|manifest)+cngeneson+pc_mito+brain_region+Sex	6.006	591.000
ENSG00000286185	0.302	0.012	0.332	-0.309	[illegible]	0.355	~amyloid_beta+(1|manifest)+cngeneson+pc_mito+brain_region+Sex	5.353	700.000
ENSG00000241962	0.939	0.012	0.228	-0.204	[illegible]	0.946	~amyloid_beta+(1|manifest)+cngeneson+pc_mito+brain_region+Sex	0.873	4429.000
ENSG00000093167	0.360	0.012	0.146	-0.123	LRRFIP2	0.411	~amyloid_beta+(1|manifest)+cngeneson+pc_mito+brain_region+Sex	1.116	3895.000
ENSG00000157800	0.030	0.012	0.126	-0.103	SLC37A3	0.074	~amyloid_beta+(1|manifest)+cngeneson+pc_mito+brain_region+Sex	0.987	4173.000
ENSG00000101849	0.709	0.011	0.223	-0.200	TBL1X	0.738	~amyloid_beta+(1|manifest)+cngeneson+pc_mito+brain_region+Sex	1.293	3520.000

ENSG00000114354	0.010	0.011	0.101	-0.078	TFG	0.040	~amyloid_beta+(1|manifest)+cngeneson+pc_mito+brain_region+Sex	0.987	4171.000
ENSG00000067167	0.045	0.011	0.132	-0.109	TRAM1	0.094	~amyloid_beta+(1|manifest)+cngeneson+pc_mito+brain_region+Sex	0.226	5838.000
ENSG00000123091	0.076	0.011	0.098	-0.075	RNF11	0.130	~amyloid_beta+(1|manifest)+cngeneson+pc_mito+brain_region+Sex	0.126	5934.000
ENSG00000174233	0.173	0.011	0.134	-0.112	ADCY6	0.228	~amyloid_beta+(1|manifest)+cngeneson+pc_mito+brain_region+Sex	1.403	3315.000
ENSG00000157827	0.731	0.011	0.090	-0.068	FMNL2	0.757	~amyloid_beta+(1|manifest)+cngeneson+pc_mito+brain_region+Sex	2.562	1846.000
ENSG00000138375	0.101	0.011	0.086	-0.064	[illegible]	0.157	~amyloid_beta+(1|manifest)+cngeneson+pc_mito+brain_region+Sex	0.339	5665.000
ENSG00000110844	0.109	0.011	0.155	-0.133	PRPF40B	0.165	~amyloid_beta+(1|manifest)+cngeneson+pc_mito+brain_region+Sex	1.301	3504.000
ENSG00000159256	0.053	0.011	0.135	-0.113	MORC3	0.103	~amyloid_beta+(1|manifest)+cngeneson+pc_mito+brain_region+Sex	1.000	4140.000
ENSG00000111785	0.354	0.011	0.180	-0.159	RIC8B	0.405	~amyloid_beta+(1|manifest)+cngeneson+pc_mito+brain_region+Sex	2.107	2303.000
ENSG00000106993	0.018	0.011	0.093	-0.071	CDC37L1	0.056	~amyloid_beta+(1|manifest)+cngeneson+pc_mito+brain_region+Sex	0.801	4628.000
ENSG00000283977	0.037	0.010	0.172	-0.151	[illegible]	0.083	~amyloid_beta+(1|manifest)+cngeneson+pc_mito+brain_region+Sex	1.444	3241.000
ENSG00000135972	0.051	0.010	0.093	-0.072	MRPS9	0.101	~amyloid_beta+(1|manifest)+cngeneson+pc_mito+brain_region+Sex	0.877	4424.000
ENSG00000087338	0.371	0.010	0.045	-0.024	GMCL1	0.421	~amyloid_beta+(1|manifest)+cngeneson+pc_mito+brain_region+Sex	0.527	5293.000
ENSG00000185619	0.041	0.010	0.121	-0.100	PCGF3	0.089	~amyloid_beta+(1|manifest)+cngeneson+pc_mito+brain_region+Sex	1.178	3748.000
ENSG00000122378	0.256	0.010	0.198	-0.177	PRXL2A	0.310	~amyloid_beta+(1|manifest)+cngeneson+pc_mito+brain_region+Sex	2.895	1607.000
ENSG00000116750	0.034	0.010	0.062	-0.042	UCHL5	0.080	~amyloid_beta+(1|manifest)+cngeneson+pc_mito+brain_region+Sex	0.190	5889.000
ENSG00000112715	0.888	0.010	0.659	-0.638	VEGFA	0.900	~amyloid_beta+(1|manifest)+cngeneson+pc_mito+brain_region+Sex	8.622	338.000
ENSG00000153395	0.081	0.010	0.134	-0.114	LPCAT1	0.135	~amyloid_beta+(1|manifest)+cngeneson+pc_mito+brain_region+Sex	1.123	3878.000
ENSG00000089006	0.087	0.010	0.064	-0.044	SNX5	0.142	~amyloid_beta+(1|manifest)+cngeneson+pc_mito+brain_region+Sex	0.241	5821.000
ENSG00000183475	0.097	0.010	0.070	-0.050	ASB7	0.152	~amyloid_beta+(1|manifest)+cngeneson+pc_mito+brain_region+Sex	0.406	5554.000
ENSG00000015133	0.111	0.010	0.183	-0.163	CCDC88C	0.167	~amyloid_beta+(1|manifest)+cngeneson+pc_mito+brain_region+Sex	0.847	4508.000
ENSG00000150760	0.557	0.010	0.137	-0.118	DOCK1	0.597	~amyloid_beta+(1|manifest)+cngeneson+pc_mito+brain_region+Sex	2.373	2026.000
ENSG00000127481	0.002	0.010	0.156	-0.137	UBR4	0.016	~amyloid_beta+(1|manifest)+cngeneson+pc_mito+brain_region+Sex	1.396	3330.000
ENSG00000152683	0.073	0.010	0.067	-0.048	SLC30A6	0.127	~amyloid_beta+(1|manifest)+cngeneson+pc_mito+brain_region+Sex	0.091	5962.000
ENSG00000102172	0.116	0.010	0.273	-0.254	SMS	0.172	~amyloid_beta+(1|manifest)+cngeneson+pc_mito+brain_region+Sex	0.747	4747.000
ENSG00000151881	0.029	0.010	0.106	-0.087	TMEM267	0.073	~amyloid_beta+(1|manifest)+cngeneson+pc_mito+brain_region+Sex	0.451	5453.000
ENSG00000134686	0.752	0.010	0.183	-0.164	PHC2	0.777	~amyloid_beta+(1|manifest)+cngeneson+pc_mito+brain_region+Sex	2.567	1841.000
ENSG00000112333	0.360	0.009	0.126	-0.107	NR2E1	0.411	~amyloid_beta+(1|manifest)+cngeneson+pc_mito+brain_region+Sex	0.215	5854.000
ENSG00000110675	0.532	0.009	0.118	-0.099	ELMOD1	0.574	~amyloid_beta+(1|manifest)+cngeneson+pc_mito+brain_region+Sex	2.511	1887.000
ENSG00000010322	0.166	0.009	0.155	-0.136	NISCH	0.221	~amyloid_beta+(1|manifest)+cngeneson+pc_mito+brain_region+Sex	1.683	2853.000
ENSG00000169905	0.070	0.009	0.074	-0.056	TOR1AIP2	0.123	~amyloid_beta+(1|manifest)+cngeneson+pc_mito+brain_region+Sex	0.690	4920.000
ENSG00000189308	0.017	0.009	0.083	-0.065	LIN54	0.054	~amyloid_beta+(1|manifest)+cngeneson+pc_mito+brain_region+Sex	0.687	4925.000
ENSG00000198898	0.089	0.009	0.210	-0.193	CAPZA2	0.143	~amyloid_beta+(1|manifest)+cngeneson+pc_mito+brain_region+Sex	2.502	1903.000
ENSG00000172379	0.452	0.009	0.136	-0.119	ARNT2	0.496	~amyloid_beta+(1|manifest)+cngeneson+pc_mito+brain_region+Sex	0.992	4160.000
ENSG00000154889	0.100	0.009	0.132	-0.115	MPPE1	0.156	~amyloid_beta+(1|manifest)+cngeneson+pc_mito+brain_region+Sex	1.191	3724.000
ENSG00000115827	0.036	0.009	0.081	-0.064	DCAF17	0.082	~amyloid_beta+(1|manifest)+cngeneson+pc_mito+brain_region+Sex	0.376	5606.000
ENSG00000132383	0.039	0.009	0.080	-0.063	RPA1	0.086	~amyloid_beta+(1|manifest)+cngeneson+pc_mito+brain_region+Sex	0.069	5968.000
ENSG00000163683	0.037	0.009	0.082	-0.065	SMIM14	0.083	~amyloid_beta+(1|manifest)+cngeneson+pc_mito+brain_region+Sex	0.893	4389.000
ENSG00000151465	0.181	0.009	0.059	-0.042	CDC123	0.235	~amyloid_beta+(1|manifest)+cngeneson+pc_mito+brain_region+Sex	0.505	5338.000
ENSG00000170903	0.074	0.008	0.244	-0.227	MSANTD4	0.128	~amyloid_beta+(1|manifest)+cngeneson+pc_mito+brain_region+Sex	3.256	1392.000
ENSG00000167074	0.191	0.008	0.298	-0.281	TEF	0.245	~amyloid_beta+(1|manifest)+cngeneson+pc_mito+brain_region+Sex	3.961	1057.000
ENSG00000272391	0.156	0.008	0.090	-0.073	POM121C	0.212	~amyloid_beta+(1|manifest)+cngeneson+pc_mito+brain_region+Sex	0.427	5513.000
ENSG00000143353	0.239	0.008	0.147	-0.130	LYPLAL1	0.293	~amyloid_beta+(1|manifest)+cngeneson+pc_mito+brain_region+Sex	1.612	2964.000
ENSG00000178467	0.272	0.008	0.245	-0.229	P4HTM	0.325	~amyloid_beta+(1|manifest)+cngeneson+pc_mito+brain_region+Sex	2.572	1834.000
ENSG00000105483	0.110	0.008	0.056	-0.040	CARD8	0.166	~amyloid_beta+(1|manifest)+cngeneson+pc_mito+brain_region+Sex	0.560	5213.000
ENSG00000110367	0.053	0.008	0.126	-0.110	DDX6	0.103	~amyloid_beta+(1|manifest)+cngeneson+pc_mito+brain_region+Sex	0.619	5087.000
ENSG00000149115	0.056	0.008	0.166	-0.149	TNKS1BP1	0.106	~amyloid_beta+(1|manifest)+cngeneson+pc_mito+brain_region+Sex	1.037	4052.000
ENSG00000154654	0.884	0.008	0.165	-0.150	NCAM2	0.897	~amyloid_beta+(1|manifest)+cngeneson+pc_mito+brain_region+Sex	8.685	332.000
ENSG00000145012	0.842	0.008	0.152	-0.136	LPP	0.858	~amyloid_beta+(1|manifest)+cngeneson+pc_mito+brain_region+Sex	11.632	194.000
ENSG00000081791	0.124	0.008	0.125	-0.110	DELE1	0.181	~amyloid_beta+(1|manifest)+cngeneson+pc_mito+brain_region+Sex	0.918	4333.000
ENSG00000139323	0.419	0.008	0.313	-0.297	POC1B	0.466	~amyloid_beta+(1|manifest)+cngeneson+pc_mito+brain_region+Sex	5.133	743.000
ENSG00000043514	0.149	0.008	0.052	-0.036	TRIT1	0.204	~amyloid_beta+(1|manifest)+cngeneson+pc_mito+brain_region+Sex	0.332	5670.000
ENSG00000178385	0.011	0.008	0.164	-0.149	PLEKHM3	0.042	~amyloid_beta+(1|manifest)+cngeneson+pc_mito+brain_region+Sex	0.349	5656.000
ENSG00000270011	0.204	0.008	0.153	-0.138	[illegible]	0.259	~amyloid_beta+(1|manifest)+cngeneson+pc_mito+brain_region+Sex	1.689	2845.000
ENSG00000115464	0.367	0.007	0.086	-0.071	USP34	0.418	~amyloid_beta+(1|manifest)+cngeneson+pc_mito+brain_region+Sex	4.838	810.000
ENSG00000131941	0.089	0.007	0.250	-0.235	RHPN2	0.143	~amyloid_beta+(1|manifest)+cngeneson+pc_mito+brain_region+Sex	2.977	1554.000
ENSG00000138688	0.361	0.007	0.151	-0.136	KIAA1109	0.412	~amyloid_beta+(1|manifest)+cngeneson+pc_mito+brain_region+Sex	1.702	2819.000
ENSG00000163536	0.152	0.007	0.095	-0.080	SERPINI1	0.207	~amyloid_beta+(1|manifest)+cngeneson+pc_mito+brain_region+Sex	0.375	5608.000
ENSG00000099991	0.153	0.007	0.220	-0.206	CABIN1	0.208	~amyloid_beta+(1|manifest)+cngeneson+pc_mito+brain_region+Sex	3.301	1368.000
ENSG00000124209	0.105	0.007	0.066	-0.052	RAB22A	0.161	~amyloid_beta+(1|manifest)+cngeneson+pc_mito+brain_region+Sex	0.719	4826.000
ENSG00000157020	0.132	0.007	0.085	-0.071	SEC13	0.189	~amyloid_beta+(1|manifest)+cngeneson+pc_mito+brain_region+Sex	0.697	4900.000
ENSG00000184831	0.047	0.007	0.079	-0.066	APOO	0.096	~amyloid_beta+(1|manifest)+cngeneson+pc_mito+brain_region+Sex	0.522	5300.000
ENSG00000105245	0.031	0.007	0.270	-0.257	NUMBL	0.075	~amyloid_beta+(1|manifest)+cngeneson+pc_mito+brain_region+Sex	2.070	2345.000
ENSG00000119042	0.591	0.007	0.125	-0.112	SATB2	0.627	~amyloid_beta+(1|manifest)+cngeneson+pc_mito+brain_region+Sex	1.512	3119.000
ENSG00000151883	0.141	0.007	0.253	-0.239	PARP8	0.197	~amyloid_beta+(1|manifest)+cngeneson+pc_mito+brain_region+Sex	1.226	3654.000
ENSG00000144136	0.205	0.007	0.182	-0.169	SLC20A1	0.260	~amyloid_beta+(1|manifest)+cngeneson+pc_mito+brain_region+Sex	2.816	1654.000
ENSG00000067141	0.990	0.006	0.157	-0.144	NEO1	0.991	~amyloid_beta+(1|manifest)+cngeneson+pc_mito+brain_region+Sex	9.909	274.000
ENSG00000115762	0.064	0.006	0.090	-0.077	PLEKHB2	0.117	~amyloid_beta+(1|manifest)+cngeneson+pc_mito+brain_region+Sex	0.627	5067.000
ENSG00000107249	0.605	0.006	0.243	-0.230	GLIS3	0.641	~amyloid_beta+(1|manifest)+cngeneson+pc_mito+brain_region+Sex	26.156	27.000
ENSG00000077585	0.114	0.006	0.249	-0.236	GPR137B	0.170	~amyloid_beta+(1|manifest)+cngeneson+pc_mito+brain_region+Sex	0.942	4272.000
ENSG00000171291	0.065	0.006	0.099	-0.087	ZNF439	0.118	~amyloid_beta+(1|manifest)+cngeneson+pc_mito+brain_region+Sex	0.554	5224.000
ENSG00000159403	0.378	0.006	0.358	-0.346	C1R	0.428	~amyloid_beta+(1|manifest)+cngeneson+pc_mito+brain_region+Sex	5.753	634.000
ENSG00000182903	0.018	0.006	0.125	-0.113	ZNF721	0.056	~amyloid_beta+(1|manifest)+cngeneson+pc_mito+brain_region+Sex	0.655	4989.000
ENSG00000205060	0.094	0.006	0.121	-0.109	SLC35B4	0.149	~amyloid_beta+(1|manifest)+cngeneson+pc_mito+brain_region+Sex	0.648	5007.000
ENSG00000077522	0.994	0.006	0.127	-0.115	ACTN2	0.994	~amyloid_beta+(1|manifest)+cngeneson+pc_mito+brain_region+Sex	3.358	1337.000
ENSG00000049769	0.047	0.006	0.075	-0.063	PPP1R3F	0.096	~amyloid_beta+(1|manifest)+cngeneson+pc_mito+brain_region+Sex	0.843	4513.000
ENSG00000118855	0.046	0.006	0.048	-0.036	MFSD1	0.095	~amyloid_beta+(1|manifest)+cngeneson+pc_mito+brain_region+Sex	0.529	5287.000
ENSG00000079482	0.714	0.006	0.127	-0.115	OPHN1	0.742	~amyloid_beta+(1|manifest)+cngeneson+pc_mito+brain_region+Sex	0.837	4524.000
ENSG00000063046	0.089	0.006	0.100	-0.089	EIF4B	0.143	~amyloid_beta+(1|manifest)+cngeneson+pc_mito+brain_region+Sex	1.116	3894.000
ENSG00000135250	0.534	0.006	0.108	-0.096	SRPK2	0.575	~amyloid_beta+(1|manifest)+cngeneson+pc_mito+brain_region+Sex	2.041	2381.000
ENSG00000101266	0.054	0.006	0.112	-0.101	CSNK2A1	0.104	~amyloid_beta+(1|manifest)+cngeneson+pc_mito+brain_region+Sex	1.800	2699.000
ENSG00000169018	0.050	0.006	0.092	-0.080	FEM1B	0.099	~amyloid_beta+(1|manifest)+cngeneson+pc_mito+brain_region+Sex	0.123	5939.000
ENSG00000111269	0.090	0.006	0.104	-0.092	CREBL2	0.145	~amyloid_beta+(1|manifest)+cngeneson+pc_mito+brain_region+Sex	0.629	5060.000
ENSG00000107854	0.101	0.006	0.102	-0.091	TNKS2	0.156	~amyloid_beta+(1|manifest)+cngeneson+pc_mito+brain_region+Sex	0.868	4447.000
ENSG00000121741	0.315	0.005	0.182	-0.171	ZMYM2	0.368	~amyloid_beta+(1|manifest)+cngeneson+pc_mito+brain_region+Sex	1.539	3067.000
ENSG00000176407	0.188	0.005	0.140	-0.130	KCMF1	0.242	~amyloid_beta+(1|manifest)+cngeneson+pc_mito+brain_region+Sex	1.366	3381.000
ENSG00000133800	0.183	0.005	0.330	-0.319	LYVE1	0.238	~amyloid_beta+(1|manifest)+cngeneson+pc_mito+brain_region+Sex	3.687	1168.000
ENSG00000164134	0.087	0.005	0.153	-0.142	NAA15	0.141	~amyloid_beta+(1|manifest)+cngeneson+pc_mito+brain_region+Sex	2.208	2196.000
ENSG00000173114	0.447	0.005	0.244	-0.234	LRRN3	0.492	~amyloid_beta+(1|manifest)+cngeneson+pc_mito+brain_region+Sex	1.994	2441.000
ENSG00000198040	0.080	0.005	0.097	-0.088	ZNF84	0.135	~amyloid_beta+(1|manifest)+cngeneson+pc_mito+brain_region+Sex	1.238	3619.000
ENSG00000111802	0.041	0.005	0.098	-0.089	TDP2	0.089	~amyloid_beta+(1|manifest)+cngeneson+pc_mito+brain_region+Sex	0.191	5886.000
ENSG00000114473	0.037	0.005	0.115	-0.105	IQCG	0.083	~amyloid_beta+(1|manifest)+cngeneson+pc_mito+brain_region+Sex	1.037	4054.000
ENSG00000077380	0.011	0.005	0.156	-0.147	DYNC1I2	0.042	~amyloid_beta+(1|manifest)+cngeneson+pc_mito+brain_region+Sex	1.401	3320.000
ENSG00000172171	0.050	0.005	0.112	-0.102	TEFM	0.100	~amyloid_beta+(1|manifest)+cngeneson+pc_mito+brain_region+Sex	0.993	4158.000
ENSG00000111364	0.123	0.005	0.085	-0.076	DDX55	0.180	~amyloid_beta+(1|manifest)+cngeneson+pc_mito+brain_region+Sex	0.722	4819.000
ENSG00000129824	0.005	0.004	0.010	-0.001	RPS4Y1	0.026	~amyloid_beta+(1|manifest)+cngeneson+pc_mito+brain_region+Sex	8.120	370.000
ENSG00000135775	0.195	0.004	0.099	-0.090	COG2	0.249	~amyloid_beta+(1|manifest)+cngeneson+pc_mito+brain_region+Sex	0.501	5355.000
ENSG00000134107	0.950	0.004	0.268	-0.259	BHLHE40	0.955	~amyloid_beta+(1|manifest)+cngeneson+pc_mito+brain_region+Sex	3.288	1380.000
ENSG00000150712	0.171	0.004	0.095	-0.086	MTMR12	0.226	~amyloid_beta+(1|manifest)+cngeneson+pc_mito+brain_region+Sex	0.346	5659.000
ENSG00000185236	0.197	0.004	0.232	-0.224	RAB11B	0.251	~amyloid_beta+(1|manifest)+cngeneson+pc_mito+brain_region+Sex	2.410	1975.000
ENSG00000068024	0.298	0.004	0.198	-0.190	HDAC4	0.351	~amyloid_beta+(1|manifest)+cngeneson+pc_mito+brain_region+Sex	1.481	3174.000
ENSG00000075711	0.497	0.004	0.159	-0.150	DLG1	0.541	~amyloid_beta+(1|manifest)+cngeneson+pc_mito+brain_region+Sex	1.374	3371.000
ENSG00000072840	0.224	0.004	NA	NA	EVC	0.278	~amyloid_beta+(1|manifest)+cngeneson+pc_mito+brain_region+Sex	0.681	4937.000
ENSG00000085998	0.035	0.004	0.152	-0.144	POMGNT1	0.081	~amyloid_beta+(1|manifest)+cngeneson+pc_mito+brain_region+Sex	1.443	3243.000
ENSG00000101199	0.216	0.004	0.364	-0.356	ARFGAP1	0.270	~amyloid_beta+(1|manifest)+cngeneson+pc_mito+brain_region+Sex	4.276	954.000
ENSG00000108039	0.032	0.004	0.087	-0.079	XPNPEP1	0.077	~amyloid_beta+(1|manifest)+cngeneson+pc_mito+brain_region+Sex	0.543	5249.000
ENSG00000156735	0.143	0.004	0.046	-0.039	BAG4	0.199	~amyloid_beta+(1|manifest)+cngeneson+pc_mito+brain_region+Sex	0.430	5508.000
ENSG00000166813	0.155	0.004	0.144	-0.136	KIF7	0.210	~amyloid_beta+(1|manifest)+cngeneson+pc_mito+brain_region+Sex	1.744	2758.000
ENSG00000196814	0.385	0.004	0.279	-0.272	MVB12B	0.434	~amyloid_beta+(1|manifest)+cngeneson+pc_mito+brain_region+Sex	2.177	2228.000
ENSG00000164091	0.124	0.004	0.070	-0.062	WDR82	0.180	~amyloid_beta+(1|manifest)+cngeneson+pc_mito+brain_region+Sex	0.173	5906.000
ENSG00000155545	0.018	0.004	0.065	-0.057	MIER3	0.056	~amyloid_beta+(1|manifest)+cngeneson+pc_mito+brain_region+Sex	0.155	5918.000
ENSG00000119682	0.054	0.004	0.108	-0.101	AREL1	0.104	~amyloid_beta+(1|manifest)+cngeneson+pc_mito+brain_region+Sex	0.915	4340.000
ENSG00000124788	0.964	0.004	0.151	-0.144	ATXN1	0.968	~amyloid_beta+(1|manifest)+cngeneson+pc_mito+brain_region+Sex	1.035	4061.000
ENSG00000141562	0.218	0.003	0.231	-0.224	NARF	0.273	~amyloid_beta+(1|manifest)+cngeneson+pc_mito+brain_region+Sex	3.116	1473.000
ENSG00000163866	0.417	0.003	0.184	-0.177	SMIM12	0.463	~amyloid_beta+(1|manifest)+cngeneson+pc_mito+brain_region+Sex	3.634	1188.000
ENSG00000168395	0.101	0.003	0.201	-0.194	ING5	0.156	~amyloid_beta+(1|manifest)+cngeneson+pc_mito+brain_region+Sex	2.316	2084.000
ENSG00000109323	0.002	0.003	0.125	-0.118	MANBA	0.018	~amyloid_beta+(1|manifest)+cngeneson+pc_mito+brain_region+Sex	0.769	4694.000
ENSG00000128872	0.375	0.003	0.300	-0.293	TMOD2	0.425	~amyloid_beta+(1|manifest)+cngeneson+pc_mito+brain_region+Sex	4.309	946.000
ENSG00000196236	0.101	0.003	0.098	-0.091	XPNPEP3	0.156	~amyloid_beta+(1|manifest)+cngeneson+pc_mito+brain_region+Sex	0.815	4583.000
ENSG00000147894	0.079	0.003	0.164	-0.158	C9orf72	0.134	~amyloid_beta+(1|manifest)+cngeneson+pc_mito+brain_region+Sex	1.830	2653.000
ENSG00000122643	0.177	0.003	0.096	-0.090	NT5C3A	0.231	~amyloid_beta+(1|manifest)+cngeneson+pc_mito+brain_region+Sex	0.969	4208.000
ENSG00000136930	0.024	0.003	0.103	-0.096	PSMB7	0.066	~amyloid_beta+(1|manifest)+cngeneson+pc_mito+brain_region+Sex	0.856	4475.000
ENSG00000170322	0.185	0.003	0.151	-0.144	NFRKB	0.240	~amyloid_beta+(1|manifest)+cngeneson+pc_mito+brain_region+Sex	1.508	3128.000
ENSG00000071127	0.116	0.003	0.195	-0.189	WDR1	0.172	~amyloid_beta+(1|manifest)+cngeneson+pc_mito+brain_region+Sex	4.158	996.000
ENSG00000168214	0.166	0.003	0.183	-0.177	RBPJ	0.222	~amyloid_beta+(1|manifest)+cngeneson+pc_mito+brain_region+Sex	1.030	4074.000
ENSG00000120158	0.113	0.003	0.079	-0.073	RCL1	0.169	~amyloid_beta+(1|manifest)+cngeneson+pc_mito+brain_region+Sex	0.856	4476.000
ENSG00000134779	0.005	0.003	0.123	-0.118	TPGS2	0.028	~amyloid_beta+(1|manifest)+cngeneson+pc_mito+brain_region+Sex	1.134	3851.000
ENSG00000123124	0.021	0.003	0.173	-0.168	WWP1	0.060	~amyloid_beta+(1|manifest)+cngeneson+pc_mito+brain_region+Sex	0.444	5468.000
ENSG00000095637	0.779	0.003	0.188	-0.182	SORBS1	0.801	~amyloid_beta+(1|manifest)+cngeneson+pc_mito+brain_region+Sex	27.030	25.000
ENSG00000082996	0.144	0.003	0.118	-0.113	RNF13	0.200	~amyloid_beta+(1|manifest)+cngeneson+pc_mito+brain_region+Sex	1.643	2917.000
ENSG00000101452	0.146	0.003	0.036	-0.031	DHX35	0.202	~amyloid_beta+(1|manifest)+cngeneson+pc_mito+brain_region+Sex	0.416	5535.000
ENSG00000165934	0.072	0.003	0.077	-0.071	CPSF2	0.126	~amyloid_beta+(1|manifest)+cngeneson+pc_mito+brain_region+Sex	0.516	5312.000
ENSG00000082805	0.706	0.003	0.136	-0.130	ERC1	0.734	~amyloid_beta+(1|manifest)+cngeneson+pc_mito+brain_region+Sex	0.495	5374.000
ENSG00000185420	0.994	0.002	0.193	-0.188	SMYD3	0.994	~amyloid_beta+(1|manifest)+cngeneson+pc_mito+brain_region+Sex	2.703	1724.000
ENSG00000101052	0.023	0.002	0.094	-0.089	IFT52	0.065	~amyloid_beta+(1|manifest)+cngeneson+pc_mito+brain_region+Sex	0.650	4997.000
ENSG00000125814	0.051	0.002	0.121	-0.116	NAPB	0.101	~amyloid_beta+(1|manifest)+cngeneson+pc_mito+brain_region+Sex	1.003	4131.000
ENSG00000219481	0.338	0.002	0.239	-0.234	NBPF1	0.389	~amyloid_beta+(1|manifest)+cngeneson+pc_mito+brain_region+Sex	1.727	2785.000
ENSG00000196628	0.997	0.002	0.097	-0.093	TCF4	0.997	~amyloid_beta+(1|manifest)+cngeneson+pc_mito+brain_region+Sex	4.045	1028.000
ENSG00000154274	0.607	0.002	0.445	-0.441	C4orf19	0.643	~amyloid_beta+(1|manifest)+cngeneson+pc_mito+brain_region+Sex	11.515	197.000
ENSG00000083844	0.072	0.002	0.077	-0.074	ZNF264	0.126	~amyloid_beta+(1|manifest)+cngeneson+pc_mito+brain_region+Sex	0.500	5361.000
ENSG00000072832	0.083	0.002	0.090	-0.087	CRMP1	0.138	~amyloid_beta+(1|manifest)+cngeneson+pc_mito+brain_region+Sex	0.512	5323.000
ENSG00000159433	0.031	0.002	0.096	-0.092	STARD9	0.075	~amyloid_beta+(1|manifest)+cngeneson+pc_mito+brain_region+Sex	0.351	5650.000
ENSG00000188338	0.099	0.002	0.196	-0.193	SLC38A3	0.154	~amyloid_beta+(1|manifest)+cngeneson+pc_mito+brain_region+Sex	2.860	1627.000
ENSG00000164253	0.030	0.002	0.153	-0.150	WDR41	0.074	~amyloid_beta+(1|manifest)+cngeneson+pc_mito+brain_region+Sex	1.637	2927.000
ENSG00000117000	0.073	0.002	0.177	-0.174	RLF	0.126	~amyloid_beta+(1|manifest)+cngeneson+pc_mito+brain_region+Sex	1.490	3156.000
ENSG00000165671	0.169	0.002	0.151	-0.148	NSD1	0.224	~amyloid_beta+(1|manifest)+cngeneson+pc_mito+brain_region+Sex	1.769	2725.000
ENSG00000256771	0.016	0.002	0.050	-0.047	ZNF253	0.053	~amyloid_beta+(1|manifest)+cngeneson+pc_mito+brain_region+Sex	0.370	5619.000
ENSG00000162670	0.983	0.001	0.492	-0.490	BRINP3	0.984	~amyloid_beta+(1|manifest)+cngeneson+pc_mito+brain_region+Sex	8.399	353.000
ENSG00000118705	0.070	0.001	0.096	-0.094	RPN2	0.124	~amyloid_beta+(1|manifest)+cngeneson+pc_mito+brain_region+Sex	0.630	5057.000
ENSG00000275700	0.060	0.001	0.099	-0.096	AATF	0.112	~amyloid_beta+(1|manifest)+cngeneson+pc_mito+brain_region+Sex	0.665	4970.000
ENSG00000059691	0.042	0.001	0.084	-0.082	GATB	0.090	~amyloid_beta+(1|manifest)+cngeneson+pc_mito+brain_region+Sex	0.063	5970.000
ENSG00000091140	0.054	0.001	0.046	-0.044	DLD	0.104	~amyloid_beta+(1|manifest)+cngeneson+pc_mito+brain_region+Sex	0.265	5788.000
ENSG00000100519	0.019	0.001	0.073	-0.071	PSMC6	0.058	~amyloid_beta+(1|manifest)+cngeneson+pc_mito+brain_region+Sex	0.787	4658.000
ENSG00000167658	0.152	0.001	0.116	-0.114	EEF2	0.207	~amyloid_beta+(1|manifest)+cngeneson+pc_mito+brain_region+Sex	0.572	5184.000
ENSG00000136141	0.432	0.001	0.120	-0.118	LRCH1	0.478	~amyloid_beta+(1|manifest)+cngeneson+pc_mito+brain_region+Sex	0.969	4204.000
ENSG00000118946	0.752	0.001	0.308	-0.306	PCDH17	0.776	~amyloid_beta+(1|manifest)+cngeneson+pc_mito+brain_region+Sex	1.388	3343.000
ENSG00000281593	0.055	0.001	0.262	-0.260	[illegible]	0.106	~amyloid_beta+(1|manifest)+cngeneson+pc_mito+brain_region+Sex	4.203	983.000
ENSG00000249240	0.570	0.001	0.428	-0.427	[illegible]	0.609	~amyloid_beta+(1|manifest)+cngeneson+pc_mito+brain_region+Sex	2.816	1655.000
ENSG00000080371	0.062	0.001	0.114	-0.112	RAB21	0.114	~amyloid_beta+(1|manifest)+cngeneson+pc_mito+brain_region+Sex	1.051	4021.000
ENSG00000099783	0.135	0.001	0.116	-0.115	HNRNPM	0.191	~amyloid_beta+(1|manifest)+cngeneson+pc_mito+brain_region+Sex	1.988	2449.000
ENSG00000139631	0.068	0.001	0.170	-0.169	CSAD	0.121	~amyloid_beta+(1|manifest)+cngeneson+pc_mito+brain_region+Sex	2.121	2287.000
ENSG00000018189	0.920	0.001	0.105	-0.103	RUFY3	0.929	~amyloid_beta+(1|manifest)+cngeneson+pc_mito+brain_region+Sex	5.130	744.000
ENSG00000167258	0.010	0.001	0.111	-0.110	CDK12	0.039	~amyloid_beta+(1|manifest)+cngeneson+pc_mito+brain_region+Sex	1.065	4000.000
ENSG00000106299	0.082	0.001	0.106	-0.105	WASL	0.137	~amyloid_beta+(1|manifest)+cngeneson+pc_mito+brain_region+Sex	0.984	4181.000
ENSG00000115902	0.518	0.001	0.399	-0.398	SLC1A4	0.561	~amyloid_beta+(1|manifest)+cngeneson+pc_mito+brain_region+Sex	4.478	896.000
ENSG00000163625	0.956	0.000	0.098	-0.097	WDFY3	0.960	~amyloid_beta+(1|manifest)+cngeneson+pc_mito+brain_region+Sex	1.232	3634.000
ENSG00000198721	0.114	0.000	0.188	-0.187	ECI2	0.170	~amyloid_beta+(1|manifest)+cngeneson+pc_mito+brain_region+Sex	1.585	3007.000
ENSG00000204514	0.117	0.000	0.158	-0.157	ZNF814	0.174	~amyloid_beta+(1|manifest)+cngeneson+pc_mito+brain_region+Sex	1.892	2570.000
ENSG00000112679	0.025	0.000	0.134	-0.133	DUSP22	0.066	~amyloid_beta+(1|manifest)+cngeneson+pc_mito+brain_region+Sex	1.438	3250.000
ENSG00000004864	0.162	0.000	0.127	-0.127	SLC25A13	0.217	~amyloid_beta+(1|manifest)+cngeneson+pc_mito+brain_region+Sex	1.691	2841.000
ENSG00000138071	0.058	0.000	0.066	-0.065	ACTR2	0.110	~amyloid_beta+(1|manifest)+cngeneson+pc_mito+brain_region+Sex	0.622	5079.000
ENSG00000055332	0.146	0.000	0.066	-0.066	EIF2AK2	0.202	~amyloid_beta+(1|manifest)+cngeneson+pc_mito+brain_region+Sex	0.776	4674.000
ENSG00000198909	0.011	0.000	0.119	-0.119	MAP3K3	0.041	~amyloid_beta+(1|manifest)+cngeneson+pc_mito+brain_region+Sex	0.863	4458.000
ENSG00000026103	0.199	0.000	0.265	-0.265	FAS	0.253	~amyloid_beta+(1|manifest)+cngeneson+pc_mito+brain_region+Sex	2.333	2066.000
ENSG00000067048	0.003	0.000	0.000	0.000	DDX3Y	0.021	~amyloid_beta+(1|manifest)+cngeneson+pc_mito+brain_region+Sex	7.178	455.000
ENSG00000164190	0.415	0.000	0.118	-0.118	NIPBL	0.461	~amyloid_beta+(1|manifest)+cngeneson+pc_mito+brain_region+Sex	1.692	2834.000
ENSG00000012817	0.025	0.000	0.000	0.000	KDM5D	0.068	~amyloid_beta+(1|manifest)+cngeneson+pc_mito+brain_region+Sex	11.188	214.000
ENSG00000087258	0.988	0.000	0.212	-0.213	GNAO1	0.989	~amyloid_beta+(1|manifest)+cngeneson+pc_mito+brain_region+Sex	2.738	1710.000
ENSG00000050748	0.085	0.000	0.096	-0.096	MAPK9	0.140	~amyloid_beta+(1|manifest)+cngeneson+pc_mito+brain_region+Sex	0.870	4438.000
ENSG00000064042	0.699	-0.001	0.309	-0.310	LIMCH1	0.728	~amyloid_beta+(1|manifest)+cngeneson+pc_mito+brain_region+Sex	3.425	1296.000
ENSG00000128708	0.017	-0.001	0.062	-0.064	HAT1	0.055	~amyloid_beta+(1|manifest)+cngeneson+pc_mito+brain_region+Sex	0.484	5395.000
ENSG00000196591	0.068	-0.001	0.045	-0.047	HDAC2	0.121	~amyloid_beta+(1|manifest)+cngeneson+pc_mito+brain_region+Sex	0.363	5627.000
ENSG00000183621	0.421	-0.001	0.172	-0.174	ZNF438	0.467	~amyloid_beta+(1|manifest)+cngeneson+pc_mito+brain_region+Sex	0.956	4234.000
ENSG00000137073	0.084	-0.001	0.146	-0.148	UBAP2	0.139	~amyloid_beta+(1|manifest)+cngeneson+pc_mito+brain_region+Sex	1.293	3518.000
ENSG00000072062	0.057	-0.001	0.117	-0.118	PRKACA	0.109	~amyloid_beta+(1|manifest)+cngeneson+pc_mito+brain_region+Sex	1.013	4109.000
ENSG00000161904	0.096	-0.001	0.136	-0.138	LEMD2	0.151	~amyloid_beta+(1|manifest)+cngeneson+pc_mito+brain_region+Sex	1.435	3254.000
ENSG00000182400	0.059	-0.001	0.075	-0.077	TRAPPC6B	0.110	~amyloid_beta+(1|manifest)+cngeneson+pc_mito+brain_region+Sex	0.322	5690.000
ENSG00000153956	0.415	-0.001	0.250	-0.252	CACNA2D1	0.461	~amyloid_beta+(1|manifest)+cngeneson+pc_mito+brain_region+Sex	4.821	818.000
ENSG00000188596	0.031	-0.001	0.445	-0.448	CFAP54	0.075	~amyloid_beta+(1|manifest)+cngeneson+pc_mito+brain_region+Sex	0.623	5075.000
ENSG00000173226	0.025	-0.001	0.141	-0.144	IQCB1	0.067	~amyloid_beta+(1|manifest)+cngeneson+pc_mito+brain_region+Sex	1.357	3398.000
ENSG00000164953	0.049	-0.001	0.139	-0.141	TMEM67	0.099	~amyloid_beta+(1|manifest)+cngeneson+pc_mito+brain_region+Sex	0.744	4759.000
ENSG00000198814	0.046	-0.001	0.042	-0.044	GK	0.095	~amyloid_beta+(1|manifest)+cngeneson+pc_mito+brain_region+Sex	0.331	5673.000
ENSG00000101966	0.025	-0.001	0.129	-0.132	XIAP	0.067	~amyloid_beta+(1|manifest)+cngeneson+pc_mito+brain_region+Sex	1.299	3508.000
ENSG00000146005	0.980	-0.001	0.538	-0.540	PSD2	0.982	~amyloid_beta+(1|manifest)+cngeneson+pc_mito+brain_region+Sex	14.792	105.000
ENSG00000108423	0.097	-0.001	0.068	-0.070	TUBD1	0.152	~amyloid_beta+(1|manifest)+cngeneson+pc_mito+brain_region+Sex	0.680	4939.000
ENSG00000197324	0.178	-0.001	0.179	-0.181	LRP10	0.233	~amyloid_beta+(1|manifest)+cngeneson+pc_mito+brain_region+Sex	0.615	5098.000
ENSG00000177707	0.312	-0.001	0.175	-0.177	NECTIN3	0.365	~amyloid_beta+(1|manifest)+cngeneson+pc_mito+brain_region+Sex	0.488	5387.000
ENSG00000130227	0.011	-0.001	0.113	-0.116	XPO7	0.042	~amyloid_beta+(1|manifest)+cngeneson+pc_mito+brain_region+Sex	0.411	5544.000
ENSG00000149212	0.870	-0.001	0.432	-0.435	SESN3	0.884	~amyloid_beta+(1|manifest)+cngeneson+pc_mito+brain_region+Sex	5.323	704.000
ENSG00000183878	0.332	-0.001	0.002	-0.005	UTY	0.384	~amyloid_beta+(1|manifest)+cngeneson+pc_mito+brain_region+Sex	49.870	5.000
ENSG00000003402	0.033	-0.002	0.178	-0.181	CFLAR	0.078	~amyloid_beta+(1|manifest)+cngeneson+pc_mito+brain_region+Sex	0.931	4300.000
ENSG00000136485	0.050	-0.002	0.079	-0.082	DCAF7	0.100	~amyloid_beta+(1|manifest)+cngeneson+pc_mito+brain_region+Sex	0.999	4142.000
ENSG00000146540	0.138	-0.002	0.092	-0.096	C7orf50	0.194	~amyloid_beta+(1|manifest)+cngeneson+pc_mito+brain_region+Sex	0.360	5635.000
ENSG00000148248	0.223	-0.002	0.127	-0.131	SURF4	0.278	~amyloid_beta+(1|manifest)+cngeneson+pc_mito+brain_region+Sex	0.521	5303.000
ENSG00000138175	0.164	-0.002	0.174	-0.178	ARL3	0.220	~amyloid_beta+(1|manifest)+cngeneson+pc_mito+brain_region+Sex	2.819	1653.000
ENSG00000173757	0.036	-0.002	0.162	-0.165	STAT5B	0.082	~amyloid_beta+(1|manifest)+cngeneson+pc_mito+brain_region+Sex	1.622	2950.000
ENSG00000177917	0.157	-0.002	0.085	-0.089	ARL6IP6	0.212	~amyloid_beta+(1|manifest)+cngeneson+pc_mito+brain_region+Sex	0.621	5081.000
ENSG00000108924	0.209	-0.002	0.195	-0.200	HLF	0.264	~amyloid_beta+(1|manifest)+cngeneson+pc_mito+brain_region+Sex	2.458	1931.000
ENSG00000114853	0.145	-0.002	0.170	-0.174	ZBTB47	0.201	~amyloid_beta+(1|manifest)+cngeneson+pc_mito+brain_region+Sex	2.151	2258.000
ENSG00000116704	0.013	-0.002	0.112	-0.117	SLC35D1	0.047	~amyloid_beta+(1|manifest)+cngeneson+pc_mito+brain_region+Sex	0.886	4409.000
ENSG00000167323	0.094	-0.002	0.208	-0.213	STIM1	0.150	~amyloid_beta+(1|manifest)+cngeneson+pc_mito+brain_region+Sex	0.683	4929.000
ENSG00000153446	0.138	-0.002	0.140	-0.145	C16orf89	0.195	~amyloid_beta+(1|manifest)+cngeneson+pc_mito+brain_region+Sex	1.353	3403.000
ENSG00000092201	0.041	-0.002	0.079	-0.084	SUPT16H	0.089	~amyloid_beta+(1|manifest)+cngeneson+pc_mito+brain_region+Sex	0.911	4349.000
ENSG00000165246	0.770	-0.002	0.005	-0.010	NLGN4Y	0.793	~amyloid_beta+(1|manifest)+cngeneson+pc_mito+brain_region+Sex	29.636	21.000
ENSG00000093183	0.134	-0.002	0.072	-0.077	SEC22C	0.190	~amyloid_beta+(1|manifest)+cngeneson+pc_mito+brain_region+Sex	0.928	4306.000
ENSG00000080839	0.025	-0.002	0.070	-0.075	RBL1	0.067	~amyloid_beta+(1|manifest)+cngeneson+pc_mito+brain_region+Sex	0.655	4988.000
ENSG00000072195	0.079	-0.002	0.144	-0.149	SPEG	0.134	~amyloid_beta+(1|manifest)+cngeneson+pc_mito+brain_region+Sex	1.893	2566.000
ENSG00000115993	0.053	-0.003	0.134	-0.139	TRAK2	0.103	~amyloid_beta+(1|manifest)+cngeneson+pc_mito+brain_region+Sex	0.872	4431.000
ENSG00000163104	0.079	-0.003	0.148	-0.153	[illegible]	0.134	~amyloid_beta+(1|manifest)+cngeneson+pc_mito+brain_region+Sex	1.057	4008.000
ENSG00000140650	0.112	-0.003	0.093	-0.098	PMM2	0.168	~amyloid_beta+(1|manifest)+cngeneson+pc_mito+brain_region+Sex	1.688	2848.000
ENSG00000124198	0.012	-0.003	0.149	-0.154	ARFGEF2	0.043	~amyloid_beta+(1|manifest)+cngeneson+pc_mito+brain_region+Sex	1.720	2795.000
ENSG00000060491	0.113	-0.003	0.187	-0.192	OGFR	0.169	~amyloid_beta+(1|manifest)+cngeneson+pc_mito+brain_region+Sex	2.626	1792.000
ENSG00000078177	0.074	-0.003	0.085	-0.091	N4BP2	0.128	~amyloid_beta+(1|manifest)+cngeneson+pc_mito+brain_region+Sex	0.862	4461.000
ENSG00000154305	0.196	-0.003	0.082	-0.088	MIA3	0.250	~amyloid_beta+(1|manifest)+cngeneson+pc_mito+brain_region+Sex	0.926	4316.000
ENSG00000197894	0.074	-0.003	0.105	-0.111	ADH5	0.128	~amyloid_beta+(1|manifest)+cngeneson+pc_mito+brain_region+Sex	0.633	5051.000
ENSG00000103018	0.885	-0.003	0.292	-0.298	CYB5B	0.897	~amyloid_beta+(1|manifest)+cngeneson+pc_mito+brain_region+Sex	7.652	405.000
ENSG00000139624	0.052	-0.003	0.125	-0.132	CERS5	0.102	~amyloid_beta+(1|manifest)+cngeneson+pc_mito+brain_region+Sex	0.463	5432.000
ENSG00000117528	0.332	-0.003	0.165	-0.171	ABCD3	0.384	~amyloid_beta+(1|manifest)+cngeneson+pc_mito+brain_region+Sex	1.205	3694.000
ENSG00000065665	0.053	-0.003	0.070	-0.076	SEC61A2	0.103	~amyloid_beta+(1|manifest)+cngeneson+pc_mito+brain_region+Sex	0.691	4916.000
ENSG00000136738	0.161	-0.003	0.077	-0.083	STAM	0.216	~amyloid_beta+(1|manifest)+cngeneson+pc_mito+brain_region+Sex	0.608	5115.000
ENSG00000129566	0.021	-0.003	0.072	-0.079	TEP1	0.061	~amyloid_beta+(1|manifest)+cngeneson+pc_mito+brain_region+Sex	0.821	4568.000
ENSG00000197724	0.025	-0.003	0.037	-0.044	PHF2	0.067	~amyloid_beta+(1|manifest)+cngeneson+pc_mito+brain_region+Sex	0.377	5602.000
ENSG00000135093	0.148	-0.003	0.080	-0.087	USP30	0.203	~amyloid_beta+(1|manifest)+cngeneson+pc_mito+brain_region+Sex	0.267	5786.000
ENSG00000157916	0.174	-0.003	0.155	-0.162	RER1	0.229	~amyloid_beta+(1|manifest)+cngeneson+pc_mito+brain_region+Sex	1.654	2896.000
ENSG00000177885	0.061	-0.003	0.080	-0.086	GRB2	0.113	~amyloid_beta+(1|manifest)+cngeneson+pc_mito+brain_region+Sex	0.646	5014.000
ENSG00000082269	0.875	-0.003	0.254	-0.261	FAM135A	0.888	~amyloid_beta+(1|manifest)+cngeneson+pc_mito+brain_region+Sex	5.996	592.000
ENSG00000120675	0.027	-0.004	0.131	-0.138	DNAJC15	0.069	~amyloid_beta+(1|manifest)+cngeneson+pc_mito+brain_region+Sex	1.202	3702.000
ENSG00000159917	0.165	-0.004	0.096	-0.103	ZNF235	0.221	~amyloid_beta+(1|manifest)+cngeneson+pc_mito+brain_region+Sex	0.803	4623.000
ENSG00000147677	0.115	-0.004	0.124	-0.131	EIF3H	0.171	~amyloid_beta+(1|manifest)+cngeneson+pc_mito+brain_region+Sex	0.597	5138.000
ENSG00000225830	0.048	-0.004	0.104	-0.111	ERCC6	0.097	~amyloid_beta+(1|manifest)+cngeneson+pc_mito+brain_region+Sex	0.852	4493.000
ENSG00000167904	0.075	-0.004	0.041	-0.049	TMEM68	0.128	~amyloid_beta+(1|manifest)+cngeneson+pc_mito+brain_region+Sex	0.553	5225.000
ENSG00000156017	0.294	-0.004	0.071	-0.079	CARNMT1	0.347	~amyloid_beta+(1|manifest)+cngeneson+pc_mito+brain_region+Sex	0.735	4782.000
ENSG00000107829	0.136	-0.004	0.160	-0.169	FBXW4	0.192	~amyloid_beta+(1|manifest)+cngeneson+pc_mito+brain_region+Sex	0.834	4533.000
ENSG00000163913	0.047	-0.004	0.160	-0.168	IFT122	0.096	~amyloid_beta+(1|manifest)+cngeneson+pc_mito+brain_region+Sex	0.721	4821.000
ENSG00000164347	0.085	-0.004	0.094	-0.103	GFM2	0.140	~amyloid_beta+(1|manifest)+cngeneson+pc_mito+brain_region+Sex	0.531	5281.000
ENSG00000197081	0.402	-0.004	0.210	-0.218	IGF2R	0.450	~amyloid_beta+(1|manifest)+cngeneson+pc_mito+brain_region+Sex	3.226	1412.000
ENSG00000185024	0.181	-0.004	0.081	-0.090	BRF1	0.236	~amyloid_beta+(1|manifest)+cngeneson+pc_mito+brain_region+Sex	0.713	4837.000
ENSG00000103064	0.211	-0.005	0.122	-0.131	SLC7A6	0.266	~amyloid_beta+(1|manifest)+cngeneson+pc_mito+brain_region+Sex	1.532	3085.000
ENSG00000101191	0.084	-0.005	0.123	-0.132	DIDO1	0.139	~amyloid_beta+(1|manifest)+cngeneson+pc_mito+brain_region+Sex	1.659	2892.000
ENSG00000005436	0.169	-0.005	0.108	-0.117	GCFC2	0.225	~amyloid_beta+(1|manifest)+cngeneson+pc_mito+brain_region+Sex	0.952	4250.000
ENSG00000188191	0.057	-0.005	0.142	-0.152	PRKAR1B	0.108	~amyloid_beta+(1|manifest)+cngeneson+pc_mito+brain_region+Sex	0.996	4147.000
ENSG00000138757	0.111	-0.005	0.034	-0.044	G3BP2	0.167	~amyloid_beta+(1|manifest)+cngeneson+pc_mito+brain_region+Sex	0.387	5586.000
ENSG00000082146	0.013	-0.005	0.064	-0.074	STRADB	0.046	~amyloid_beta+(1|manifest)+cngeneson+pc_mito+brain_region+Sex	0.711	4841.000
ENSG00000187605	0.563	-0.005	0.151	-0.161	TET3	0.603	~amyloid_beta+(1|manifest)+cngeneson+pc_mito+brain_region+Sex	0.744	4760.000
ENSG00000148459	0.036	-0.005	0.060	-0.070	PDSS1	0.083	~amyloid_beta+(1|manifest)+cngeneson+pc_mito+brain_region+Sex	0.246	5812.000
ENSG00000127990	0.360	-0.005	0.220	-0.230	SGCE	0.411	~amyloid_beta+(1|manifest)+cngeneson+pc_mito+brain_region+Sex	0.020	5979.000
ENSG00000119321	0.019	-0.005	0.052	-0.063	FKBP15	0.058	~amyloid_beta+(1|manifest)+cngeneson+pc_mito+brain_region+Sex	0.752	4738.000
ENSG00000116786	0.076	-0.005	0.160	-0.171	PLEKHM2	0.131	~amyloid_beta+(1|manifest)+cngeneson+pc_mito+brain_region+Sex	1.733	2774.000
ENSG00000173950	0.079	-0.005	0.096	-0.107	XXYLT1	0.133	~amyloid_beta+(1|manifest)+cngeneson+pc_mito+brain_region+Sex	1.331	3445.000
ENSG00000101782	0.136	-0.005	0.066	-0.077	RIOK3	0.192	~amyloid_beta+(1|manifest)+cngeneson+pc_mito+brain_region+Sex	0.874	4428.000
ENSG00000125834	0.047	-0.005	0.066	-0.077	STK35	0.096	~amyloid_beta+(1|manifest)+cngeneson+pc_mito+brain_region+Sex	0.530	5286.000
ENSG00000136504	0.072	-0.006	0.070	-0.081	KAT7	0.126	~amyloid_beta+(1|manifest)+cngeneson+pc_mito+brain_region+Sex	0.969	4206.000
ENSG00000125534	0.355	-0.006	0.500	-0.511	PPDPF	0.406	~amyloid_beta+(1|manifest)+cngeneson+pc_mito+brain_region+Sex	3.197	1427.000
ENSG00000197050	0.046	-0.006	0.067	-0.079	ZNF420	0.095	~amyloid_beta+(1|manifest)+cngeneson+pc_mito+brain_region+Sex	0.729	4795.000
ENSG00000173744	0.055	-0.006	0.148	-0.160	AGFG1	0.106	~amyloid_beta+(1|manifest)+cngeneson+pc_mito+brain_region+Sex	1.439	3248.000
ENSG00000120784	0.152	-0.006	0.123	-0.135	ZFP30	0.207	~amyloid_beta+(1|manifest)+cngeneson+pc_mito+brain_region+Sex	1.402	3318.000
ENSG00000273749	0.175	-0.006	0.172	-0.184	CYFIP1	0.229	~amyloid_beta+(1|manifest)+cngeneson+pc_mito+brain_region+Sex	1.664	2882.000
ENSG00000131373	0.084	-0.006	0.100	-0.112	HACL1	0.139	~amyloid_beta+(1|manifest)+cngeneson+pc_mito+brain_region+Sex	1.111	3906.000
ENSG00000147687	0.030	-0.006	0.150	-0.162	TATDN1	0.074	~amyloid_beta+(1|manifest)+cngeneson+pc_mito+brain_region+Sex	0.757	4723.000
ENSG00000138785	0.211	-0.006	0.126	-0.138	INTS12	0.265	~amyloid_beta+(1|manifest)+cngeneson+pc_mito+brain_region+Sex	1.341	3427.000
ENSG00000172175	0.019	-0.006	0.151	-0.164	MALT1	0.057	~amyloid_beta+(1|manifest)+cngeneson+pc_mito+brain_region+Sex	1.933	2519.000
ENSG00000170448	0.027	-0.006	0.044	-0.056	NFXL1	0.069	~amyloid_beta+(1|manifest)+cngeneson+pc_mito+brain_region+Sex	0.463	5434.000
ENSG00000100023	0.147	-0.006	0.114	-0.127	PPIL2	0.202	~amyloid_beta+(1|manifest)+cngeneson+pc_mito+brain_region+Sex	1.891	2571.000
ENSG00000141002	0.215	-0.006	0.381	-0.393	TCF25	0.269	~amyloid_beta+(1|manifest)+cngeneson+pc_mito+brain_region+Sex	6.171	570.000
ENSG00000264324	0.022	-0.006	0.116	-0.129	[illegible]	0.063	~amyloid_beta+(1|manifest)+cngeneson+pc_mito+brain_region+Sex	1.860	2614.000
ENSG00000102900	0.052	-0.006	0.081	-0.093	NUP93	0.102	~amyloid_beta+(1|manifest)+cngeneson+pc_mito+brain_region+Sex	0.855	4483.000
ENSG00000178125	0.002	-0.006	0.117	-0.130	PPP1R42	0.017	~amyloid_beta+(1|manifest)+cngeneson+pc_mito+brain_region+Sex	0.262	5793.000
ENSG00000156171	0.029	-0.006	0.091	-0.104	DRAM2	0.073	~amyloid_beta+(1|manifest)+cngeneson+pc_mito+brain_region+Sex	0.848	4506.000
ENSG00000120915	0.177	-0.006	0.170	-0.183	EPHX2	0.231	~amyloid_beta+(1|manifest)+cngeneson+pc_mito+brain_region+Sex	3.243	1401.000
ENSG00000204217	0.240	-0.007	0.154	-0.168	BMPR2	0.294	~amyloid_beta+(1|manifest)+cngeneson+pc_mito+brain_region+Sex	2.343	2058.000
ENSG00000176946	0.112	-0.007	0.093	-0.106	THAP4	0.168	~amyloid_beta+(1|manifest)+cngeneson+pc_mito+brain_region+Sex	0.558	5219.000
ENSG00000130055	0.051	-0.007	0.172	-0.186	GDPD2	0.101	~amyloid_beta+(1|manifest)+cngeneson+pc_mito+brain_region+Sex	1.130	3863.000
ENSG00000148700	0.980	-0.007	0.095	-0.109	ADD3	0.982	~amyloid_beta+(1|manifest)+cngeneson+pc_mito+brain_region+Sex	3.098	1484.000
ENSG00000196639	0.172	-0.007	0.204	-0.218	HRH1	0.227	~amyloid_beta+(1|manifest)+cngeneson+pc_mito+brain_region+Sex	0.108	5948.000
ENSG00000152127	0.975	-0.007	0.424	-0.438	MGAT5	0.978	~amyloid_beta+(1|manifest)+cngeneson+pc_mito+brain_region+Sex	14.427	117.000
ENSG00000133687	0.991	-0.007	0.463	-0.477	TMTC1	0.991	~amyloid_beta+(1|manifest)+cngeneson+pc_mito+brain_region+Sex	11.297	207.000
ENSG00000153006	0.136	-0.007	0.073	-0.087	SREK1IP1	0.193	~amyloid_beta+(1|manifest)+cngeneson+pc_mito+brain_region+Sex	0.631	5054.000
ENSG00000100836	0.603	-0.007	0.523	-0.538	PABPN1	0.640	~amyloid_beta+(1|manifest)+cngeneson+pc_mito+brain_region+Sex	12.131	172.000
ENSG00000100813	0.092	-0.007	0.090	-0.105	ACIN1	0.147	~amyloid_beta+(1|manifest)+cngeneson+pc_mito+brain_region+Sex	0.977	4190.000
ENSG00000121774	0.083	-0.007	0.107	-0.122	KHDRBS1	0.138	~amyloid_beta+(1|manifest)+cngeneson+pc_mito+brain_region+Sex	0.352	5649.000
ENSG00000166086	0.223	-0.007	0.090	-0.104	JAM3	0.278	~amyloid_beta+(1|manifest)+cngeneson+pc_mito+brain_region+Sex	0.522	5301.000
ENSG00000068745	0.270	-0.007	0.259	-0.274	IP6K2	0.323	~amyloid_beta+(1|manifest)+cngeneson+pc_mito+brain_region+Sex	1.972	2465.000
ENSG00000185344	0.041	-0.007	0.043	-0.058	ATP6V0A2	0.089	~amyloid_beta+(1|manifest)+cngeneson+pc_mito+brain_region+Sex	0.565	5198.000
ENSG00000150967	0.234	-0.007	0.221	-0.236	ABCB9	0.288	~amyloid_beta+(1|manifest)+cngeneson+pc_mito+brain_region+Sex	2.310	2092.000
ENSG00000177646	0.089	-0.008	0.075	-0.090	ACAD9	0.143	~amyloid_beta+(1|manifest)+cngeneson+pc_mito+brain_region+Sex	0.533	5276.000
ENSG00000112305	0.108	-0.008	0.199	-0.214	SMAP1	0.163	~amyloid_beta+(1|manifest)+cngeneson+pc_mito+brain_region+Sex	2.392	1996.000
ENSG00000119242	0.073	-0.008	0.173	-0.188	CCDC92	0.127	~amyloid_beta+(1|manifest)+cngeneson+pc_mito+brain_region+Sex	1.309	3484.000
ENSG00000083750	0.127	-0.008	0.115	-0.130	RRAGB	0.183	~amyloid_beta+(1|manifest)+cngeneson+pc_mito+brain_region+Sex	0.184	5896.000
ENSG00000102910	0.820	-0.008	0.126	-0.142	LONP2	0.839	~amyloid_beta+(1|manifest)+cngeneson+pc_mito+brain_region+Sex	4.235	971.000
ENSG00000177058	0.006	-0.008	0.073	-0.089	SLC38A9	0.029	~amyloid_beta+(1|manifest)+cngeneson+pc_mito+brain_region+Sex	0.308	5714.000
ENSG00000113716	0.146	-0.008	0.085	-0.101	HMGXB3	0.202	~amyloid_beta+(1|manifest)+cngeneson+pc_mito+brain_region+Sex	0.990	4164.000
ENSG00000188690	0.047	-0.008	0.118	-0.134	UROS	0.096	~amyloid_beta+(1|manifest)+cngeneson+pc_mito+brain_region+Sex	1.218	3666.000
ENSG00000113597	0.102	-0.008	0.054	-0.070	TRAPPC13	0.158	~amyloid_beta+(1|manifest)+cngeneson+pc_mito+brain_region+Sex	0.780	4669.000
ENSG00000107897	0.031	-0.008	0.023	-0.039	ACBD5	0.076	~amyloid_beta+(1|manifest)+cngeneson+pc_mito+brain_region+Sex	0.629	5062.000
ENSG00000164494	0.392	-0.008	0.147	-0.163	PDSS2	0.441	~amyloid_beta+(1|manifest)+cngeneson+pc_mito+brain_region+Sex	0.746	4752.000
ENSG00000165355	0.092	-0.008	0.068	-0.084	FBXO33	0.148	~amyloid_beta+(1|manifest)+cngeneson+pc_mito+brain_region+Sex	0.660	4980.000
ENSG00000149262	0.056	-0.008	0.049	-0.065	INTS4	0.106	~amyloid_beta+(1|manifest)+cngeneson+pc_mito+brain_region+Sex	0.440	5484.000
ENSG00000149100	0.092	-0.008	0.069	-0.085	EIF3M	0.147	~amyloid_beta+(1|manifest)+cngeneson+pc_mito+brain_region+Sex	0.236	5830.000
ENSG00000179152	0.439	-0.008	0.143	-0.159	TCAIM	0.484	~amyloid_beta+(1|manifest)+cngeneson+pc_mito+brain_region+Sex	1.844	2635.000
ENSG00000134369	0.965	-0.008	0.614	-0.631	NAV1	0.969	~amyloid_beta+(1|manifest)+cngeneson+pc_mito+brain_region+Sex	18.570	61.000
ENSG00000167637	0.020	-0.008	0.053	-0.069	ZNF283	0.060	~amyloid_beta+(1|manifest)+cngeneson+pc_mito+brain_region+Sex	0.488	5389.000
ENSG00000132589	0.326	-0.008	0.244	-0.261	FLOT2	0.378	~amyloid_beta+(1|manifest)+cngeneson+pc_mito+brain_region+Sex	1.434	3258.000
ENSG00000081087	0.060	-0.008	0.089	-0.106	OSTM1	0.112	~amyloid_beta+(1|manifest)+cngeneson+pc_mito+brain_region+Sex	0.702	4877.000
ENSG00000143569	0.054	-0.008	0.077	-0.094	UBAP2L	0.104	~amyloid_beta+(1|manifest)+cngeneson+pc_mito+brain_region+Sex	0.734	4783.000
ENSG00000119661	0.040	-0.008	0.037	-0.053	DNAL1	0.087	~amyloid_beta+(1|manifest)+cngeneson+pc_mito+brain_region+Sex	0.262	5794.000
ENSG00000149577	0.123	-0.008	0.102	-0.119	SIDT2	0.180	~amyloid_beta+(1|manifest)+cngeneson+pc_mito+brain_region+Sex	1.037	4056.000
ENSG00000128609	0.210	-0.008	0.070	-0.087	NDUFA5	0.265	~amyloid_beta+(1|manifest)+cngeneson+pc_mito+brain_region+Sex	0.439	5486.000
ENSG00000175354	0.124	-0.009	0.260	-0.277	PTPN2	0.181	~amyloid_beta+(1|manifest)+cngeneson+pc_mito+brain_region+Sex	2.682	1743.000
ENSG00000092969	0.980	-0.009	0.324	-0.341	TGFB2	0.982	~amyloid_beta+(1|manifest)+cngeneson+pc_mito+brain_region+Sex	7.715	398.000
ENSG00000074201	0.095	-0.009	0.080	-0.097	CLNS1A	0.150	~amyloid_beta+(1|manifest)+cngeneson+pc_mito+brain_region+Sex	0.326	5684.000
ENSG00000197548	0.915	-0.009	0.100	-0.118	ATG7	0.925	~amyloid_beta+(1|manifest)+cngeneson+pc_mito+brain_region+Sex	2.552	1855.000
ENSG00000143420	0.751	-0.009	0.410	-0.428	ENSA	0.776	~amyloid_beta+(1|manifest)+cngeneson+pc_mito+brain_region+Sex	5.455	683.000
ENSG00000116353	0.060	-0.009	0.086	-0.103	MECR	0.111	~amyloid_beta+(1|manifest)+cngeneson+pc_mito+brain_region+Sex	1.103	3921.000
ENSG00000104805	0.122	-0.009	0.140	-0.158	NUCB1	0.178	~amyloid_beta+(1|manifest)+cngeneson+pc_mito+brain_region+Sex	0.765	4703.000
ENSG00000115020	0.049	-0.009	0.144	-0.162	PIKFYVE	0.098	~amyloid_beta+(1|manifest)+cngeneson+pc_mito+brain_region+Sex	1.511	3122.000
ENSG00000068308	0.059	-0.009	0.048	-0.067	OTUD5	0.111	~amyloid_beta+(1|manifest)+cngeneson+pc_mito+brain_region+Sex	0.738	4775.000
ENSG00000170088	0.069	-0.009	0.173	-0.191	TMEM192	0.122	~amyloid_beta+(1|manifest)+cngeneson+pc_mito+brain_region+Sex	1.697	2828.000
ENSG00000106263	0.247	-0.009	0.156	-0.175	EIF3B	0.300	~amyloid_beta+(1|manifest)+cngeneson+pc_mito+brain_region+Sex	1.613	2961.000
ENSG00000163655	0.121	-0.009	0.100	-0.119	GMPS	0.177	~amyloid_beta+(1|manifest)+cngeneson+pc_mito+brain_region+Sex	1.030	4076.000
ENSG00000148737	0.936	-0.009	0.075	-0.094	TCF7L2	0.943	~amyloid_beta+(1|manifest)+cngeneson+pc_mito+brain_region+Sex	4.677	847.000
ENSG00000067248	0.092	-0.009	0.100	-0.119	DHX29	0.147	~amyloid_beta+(1|manifest)+cngeneson+pc_mito+brain_region+Sex	1.220	3663.000
ENSG00000047230	0.003	-0.010	0.123	-0.142	CTPS2	0.021	~amyloid_beta+(1|manifest)+cngeneson+pc_mito+brain_region+Sex	0.213	5856.000
ENSG00000187555	0.085	-0.010	0.077	-0.096	USP7	0.140	~amyloid_beta+(1|manifest)+cngeneson+pc_mito+brain_region+Sex	0.582	5165.000
ENSG00000115694	0.304	-0.010	0.158	-0.177	STK25	0.356	~amyloid_beta+(1|manifest)+cngeneson+pc_mito+brain_region+Sex	1.850	2626.000
ENSG00000043093	0.528	-0.010	0.311	-0.330	DCUN1D1	0.570	~amyloid_beta+(1|manifest)+cngeneson+pc_mito+brain_region+Sex	2.381	2012.000
ENSG00000115295	0.816	-0.010	0.278	-0.297	CLIP4	0.835	~amyloid_beta+(1|manifest)+cngeneson+pc_mito+brain_region+Sex	1.873	2597.000
ENSG00000165275	0.360	-0.010	0.150	-0.170	TRMT10B	0.411	~amyloid_beta+(1|manifest)+cngeneson+pc_mito+brain_region+Sex	2.398	1987.000
ENSG00000122218	0.115	-0.010	0.117	-0.136	COPA	0.171	~amyloid_beta+(1|manifest)+cngeneson+pc_mito+brain_region+Sex	0.389	5582.000
ENSG00000117139	0.061	-0.010	0.108	-0.128	KDM5B	0.112	~amyloid_beta+(1|manifest)+cngeneson+pc_mito+brain_region+Sex	1.433	3262.000
ENSG00000166847	0.116	-0.010	0.074	-0.094	DCTN5	0.172	~amyloid_beta+(1|manifest)+cngeneson+pc_mito+brain_region+Sex	0.649	5003.000
ENSG00000134313	0.464	-0.010	0.180	-0.200	KIDINS220	0.509	~amyloid_beta+(1|manifest)+cngeneson+pc_mito+brain_region+Sex	1.734	2773.000
ENSG00000135870	0.041	-0.010	0.096	-0.116	RC3H1	0.089	~amyloid_beta+(1|manifest)+cngeneson+pc_mito+brain_region+Sex	1.190	3728.000
ENSG00000119636	0.032	-0.010	0.119	-0.139	BBOF1	0.077	~amyloid_beta+(1|manifest)+cngeneson+pc_mito+brain_region+Sex	1.310	3483.000
ENSG00000108651	0.096	-0.010	0.090	-0.110	UTP6	0.151	~amyloid_beta+(1|manifest)+cngeneson+pc_mito+brain_region+Sex	1.147	3819.000
ENSG00000117899	0.228	-0.010	0.099	-0.120	MESD	0.282	~amyloid_beta+(1|manifest)+cngeneson+pc_mito+brain_region+Sex	0.948	4257.000
ENSG00000182326	0.446	-0.010	0.318	-0.339	C1S	0.491	~amyloid_beta+(1|manifest)+cngeneson+pc_mito+brain_region+Sex	3.005	1541.000
ENSG00000138443	0.763	-0.010	0.162	-0.182	ABI2	0.787	~amyloid_beta+(1|manifest)+cngeneson+pc_mito+brain_region+Sex	1.227	3653.000
ENSG00000186918	0.100	-0.010	0.121	-0.142	ZNF395	0.156	~amyloid_beta+(1|manifest)+cngeneson+pc_mito+brain_region+Sex	1.453	3223.000
ENSG00000148187	0.068	-0.010	0.065	-0.086	MRRF	0.122	~amyloid_beta+(1|manifest)+cngeneson+pc_mito+brain_region+Sex	0.998	4146.000
ENSG00000128791	0.086	-0.010	0.080	-0.101	TWSG1	0.141	~amyloid_beta+(1|manifest)+cngeneson+pc_mito+brain_region+Sex	0.711	4843.000
ENSG00000145982	0.468	-0.011	0.207	-0.228	FARS2	0.513	~amyloid_beta+(1|manifest)+cngeneson+pc_mito+brain_region+Sex	0.953	4244.000
ENSG00000102309	0.250	-0.011	0.123	-0.144	PIN4	0.304	~amyloid_beta+(1|manifest)+cngeneson+pc_mito+brain_region+Sex	1.683	2852.000
ENSG00000277149	0.079	-0.011	0.075	-0.096	TYW1B	0.134	~amyloid_beta+(1|manifest)+cngeneson+pc_mito+brain_region+Sex	0.729	4797.000
ENSG00000152520	0.343	-0.011	0.155	-0.177	PAN3	0.394	~amyloid_beta+(1|manifest)+cngeneson+pc_mito+brain_region+Sex	2.187	2219.000
ENSG00000107902	0.084	-0.011	0.147	-0.169	LHPP	0.139	~amyloid_beta+(1|manifest)+cngeneson+pc_mito+brain_region+Sex	1.175	3760.000
ENSG00000151240	0.957	-0.011	0.165	-0.187	DIP2C	0.961	~amyloid_beta+(1|manifest)+cngeneson+pc_mito+brain_region+Sex	7.333	436.000
ENSG00000115875	0.096	-0.011	0.137	-0.159	SRSF7	0.151	~amyloid_beta+(1|manifest)+cngeneson+pc_mito+brain_region+Sex	1.277	3553.000
ENSG00000140632	0.177	-0.011	0.116	-0.138	GLYR1	0.231	~amyloid_beta+(1|manifest)+cngeneson+pc_mito+brain_region+Sex	1.455	3214.000
ENSG00000122970	0.030	-0.011	0.049	-0.071	IFT81	0.074	~amyloid_beta+(1|manifest)+cngeneson+pc_mito+brain_region+Sex	0.366	5624.000
ENSG00000108384	0.191	-0.011	0.063	-0.085	RAD51C	0.245	~amyloid_beta+(1|manifest)+cngeneson+pc_mito+brain_region+Sex	0.297	5738.000
ENSG00000015676	0.059	-0.011	0.083	-0.105	NUDCD3	0.110	~amyloid_beta+(1|manifest)+cngeneson+pc_mito+brain_region+Sex	1.210	3681.000
ENSG00000156671	0.031	-0.011	0.120	-0.142	SAMD8	0.075	~amyloid_beta+(1|manifest)+cngeneson+pc_mito+brain_region+Sex	1.010	4115.000
ENSG00000134759	0.086	-0.011	0.117	-0.140	ELP2	0.141	~amyloid_beta+(1|manifest)+cngeneson+pc_mito+brain_region+Sex	0.630	5056.000
ENSG00000101421	0.036	-0.011	0.063	-0.085	CHMP4B	0.083	~amyloid_beta+(1|manifest)+cngeneson+pc_mito+brain_region+Sex	0.283	5759.000
ENSG00000084774	0.139	-0.012	0.097	-0.120	CAD	0.195	~amyloid_beta+(1|manifest)+cngeneson+pc_mito+brain_region+Sex	1.232	3633.000
ENSG00000164506	0.933	-0.012	0.168	-0.191	STXBP5	0.941	~amyloid_beta+(1|manifest)+cngeneson+pc_mito+brain_region+Sex	6.271	553.000
ENSG00000150527	0.017	-0.012	0.129	-0.152	MIA2	0.054	~amyloid_beta+(1|manifest)+cngeneson+pc_mito+brain_region+Sex	1.640	2922.000
ENSG00000033030	0.048	-0.012	0.078	-0.101	ZCCHC8	0.097	~amyloid_beta+(1|manifest)+cngeneson+pc_mito+brain_region+Sex	0.512	5324.000
ENSG00000179295	0.024	-0.012	0.080	-0.103	PTPN11	0.066	~amyloid_beta+(1|manifest)+cngeneson+pc_mito+brain_region+Sex	0.845	4510.000
ENSG00000176148	0.054	-0.012	0.062	-0.086	TCP11L1	0.104	~amyloid_beta+(1|manifest)+cngeneson+pc_mito+brain_region+Sex	0.545	5242.000
ENSG00000197448	0.145	-0.012	0.138	-0.161	GSTK1	0.201	~amyloid_beta+(1|manifest)+cngeneson+pc_mito+brain_region+Sex	1.433	3260.000
ENSG00000072501	0.141	-0.012	0.114	-0.137	SMC1A	0.198	~amyloid_beta+(1|manifest)+cngeneson+pc_mito+brain_region+Sex	0.956	4235.000
ENSG00000131626	0.839	-0.012	0.198	-0.222	PPFIA1	0.855	~amyloid_beta+(1|manifest)+cngeneson+pc_mito+brain_region+Sex	10.702	234.000
ENSG00000141084	0.059	-0.012	0.088	-0.112	RANBP10	0.111	~amyloid_beta+(1|manifest)+cngeneson+pc_mito+brain_region+Sex	0.944	4268.000
ENSG00000055917	0.302	-0.012	0.152	-0.176	PUM2	0.354	~amyloid_beta+(1|manifest)+cngeneson+pc_mito+brain_region+Sex	1.199	3707.000
ENSG00000136731	0.044	-0.012	0.052	-0.076	UGGT1	0.093	~amyloid_beta+(1|manifest)+cngeneson+pc_mito+brain_region+Sex	0.765	4704.000
ENSG00000116852	0.262	-0.012	0.177	-0.201	KIF21B	0.316	~amyloid_beta+(1|manifest)+cngeneson+pc_mito+brain_region+Sex	2.389	2000.000
ENSG00000120458	0.059	-0.012	0.065	-0.090	MSANTD2	0.110	~amyloid_beta+(1|manifest)+cngeneson+pc_mito+brain_region+Sex	0.614	5100.000
ENSG00000118900	0.035	-0.012	0.103	-0.127	UBN1	0.081	~amyloid_beta+(1|manifest)+cngeneson+pc_mito+brain_region+Sex	1.136	3847.000
ENSG00000079785	0.034	-0.012	0.047	-0.071	DDX1	0.079	~amyloid_beta+(1|manifest)+cngeneson+pc_mito+brain_region+Sex	0.779	4672.000
ENSG00000114686	0.070	-0.012	0.043	-0.067	MRPL3	0.124	~amyloid_beta+(1|manifest)+cngeneson+pc_mito+brain_region+Sex	0.584	5161.000
ENSG00000115750	0.156	-0.013	0.089	-0.114	TAF1B	0.212	~amyloid_beta+(1|manifest)+cngeneson+pc_mito+brain_region+Sex	0.408	5550.000
ENSG00000092847	0.090	-0.013	0.106	-0.131	AGO1	0.145	~amyloid_beta+(1|manifest)+cngeneson+pc_mito+brain_region+Sex	0.933	4292.000
ENSG00000185985	0.275	-0.013	0.262	-0.287	SLITRK2	0.329	~amyloid_beta+(1|manifest)+cngeneson+pc_mito+brain_region+Sex	2.350	2048.000
ENSG00000006459	0.152	-0.013	0.158	-0.183	KDM7A	0.207	~amyloid_beta+(1|manifest)+cngeneson+pc_mito+brain_region+Sex	2.686	1739.000
ENSG00000136848	0.144	-0.013	0.217	-0.242	DAB2IP	0.200	~amyloid_beta+(1|manifest)+cngeneson+pc_mito+brain_region+Sex	2.847	1639.000
ENSG00000141759	0.147	-0.013	0.094	-0.119	TXNL4A	0.202	~amyloid_beta+(1|manifest)+cngeneson+pc_mito+brain_region+Sex	1.138	3844.000
ENSG00000197150	0.078	-0.013	0.107	-0.133	ABCB8	0.133	~amyloid_beta+(1|manifest)+cngeneson+pc_mito+brain_region+Sex	1.547	3056.000
ENSG00000117519	0.956	-0.013	0.425	-0.451	CNN3	0.960	~amyloid_beta+(1|manifest)+cngeneson+pc_mito+brain_region+Sex	13.371	141.000
ENSG00000147364	0.040	-0.013	0.036	-0.061	FBXO25	0.088	~amyloid_beta+(1|manifest)+cngeneson+pc_mito+brain_region+Sex	0.501	5357.000
ENSG00000132305	0.173	-0.013	0.086	-0.112	IMMT	0.228	~amyloid_beta+(1|manifest)+cngeneson+pc_mito+brain_region+Sex	1.155	3802.000
ENSG00000157954	0.034	-0.013	0.050	-0.076	WIPI2	0.079	~amyloid_beta+(1|manifest)+cngeneson+pc_mito+brain_region+Sex	0.645	5019.000
ENSG00000143772	0.966	-0.013	0.172	-0.198	ITPKB	0.969	~amyloid_beta+(1|manifest)+cngeneson+pc_mito+brain_region+Sex	10.860	229.000
ENSG00000186153	0.847	-0.013	0.157	-0.183	WWOX	0.862	~amyloid_beta+(1|manifest)+cngeneson+pc_mito+brain_region+Sex	8.632	337.000
ENSG00000165525	0.038	-0.013	0.145	-0.171	NEMF	0.086	~amyloid_beta+(1|manifest)+cngeneson+pc_mito+brain_region+Sex	2.276	2128.000
ENSG00000131507	0.328	-0.013	0.116	-0.142	NDFIP1	0.380	~amyloid_beta+(1|manifest)+cngeneson+pc_mito+brain_region+Sex	1.954	2489.000
ENSG00000273136	0.071	-0.013	0.371	-0.397	NBPF26	0.125	~amyloid_beta+(1|manifest)+cngeneson+pc_mito+brain_region+Sex	1.334	3442.000
ENSG00000128829	0.041	-0.013	0.138	-0.164	EIF2AK4	0.089	~amyloid_beta+(1|manifest)+cngeneson+pc_mito+brain_region+Sex	0.308	5715.000
ENSG00000049618	0.936	-0.013	0.065	-0.092	ARID1B	0.943	~amyloid_beta+(1|manifest)+cngeneson+pc_mito+brain_region+Sex	1.113	3903.000
ENSG00000119414	0.054	-0.014	0.055	-0.082	PPP6C	0.104	~amyloid_beta+(1|manifest)+cngeneson+pc_mito+brain_region+Sex	0.764	4708.000
ENSG00000177239	0.081	-0.014	0.233	-0.261	MAN1B1	0.136	~amyloid_beta+(1|manifest)+cngeneson+pc_mito+brain_region+Sex	2.968	1561.000
ENSG00000166200	0.044	-0.014	0.054	-0.081	COPS2	0.092	~amyloid_beta+(1|manifest)+cngeneson+pc_mito+brain_region+Sex	0.508	5332.000
ENSG00000130714	0.241	-0.014	0.118	-0.145	POMT1	0.295	~amyloid_beta+(1|manifest)+cngeneson+pc_mito+brain_region+Sex	1.252	3593.000
ENSG00000160145	0.907	-0.014	0.234	-0.262	KALRN	0.918	~amyloid_beta+(1|manifest)+cngeneson+pc_mito+brain_region+Sex	10.607	240.000
ENSG00000144747	0.107	-0.014	0.040	-0.068	TMF1	0.163	~amyloid_beta+(1|manifest)+cngeneson+pc_mito+brain_region+Sex	0.561	5210.000
ENSG00000111358	0.091	-0.014	0.062	-0.090	GTF2H3	0.146	~amyloid_beta+(1|manifest)+cngeneson+pc_mito+brain_region+Sex	0.814	4589.000
ENSG00000157450	0.059	-0.014	0.085	-0.113	RNF111	0.111	~amyloid_beta+(1|manifest)+cngeneson+pc_mito+brain_region+Sex	1.427	3270.000
ENSG00000103549	0.088	-0.014	0.076	-0.104	RNF40	0.143	~amyloid_beta+(1|manifest)+cngeneson+pc_mito+brain_region+Sex	0.890	4396.000
ENSG00000176105	0.057	-0.014	0.043	-0.071	YES1	0.109	~amyloid_beta+(1|manifest)+cngeneson+pc_mito+brain_region+Sex	0.732	4786.000
ENSG00000109670	0.951	-0.014	0.181	-0.209	FBXW7	0.957	~amyloid_beta+(1|manifest)+cngeneson+pc_mito+brain_region+Sex	3.695	1164.000
ENSG00000138162	0.107	-0.014	0.145	-0.173	TACC2	0.162	~amyloid_beta+(1|manifest)+cngeneson+pc_mito+brain_region+Sex	0.782	4662.000
ENSG00000119392	0.088	-0.014	0.073	-0.101	GLE1	0.143	~amyloid_beta+(1|manifest)+cngeneson+pc_mito+brain_region+Sex	0.952	4251.000
ENSG00000151491	0.635	-0.015	0.158	-0.187	EPS8	0.669	~amyloid_beta+(1|manifest)+cngeneson+pc_mito+brain_region+Sex	0.533	5277.000
ENSG00000170989	0.389	-0.015	0.222	-0.251	S1PR1	0.438	~amyloid_beta+(1|manifest)+cngeneson+pc_mito+brain_region+Sex	1.488	3162.000
ENSG00000139734	0.501	-0.015	0.371	-0.400	DIAPH3	0.545	~amyloid_beta+(1|manifest)+cngeneson+pc_mito+brain_region+Sex	5.106	752.000
ENSG00000107669	0.384	-0.015	0.106	-0.135	ATE1	0.433	~amyloid_beta+(1|manifest)+cngeneson+pc_mito+brain_region+Sex	1.135	3849.000
ENSG00000117906	0.375	-0.015	0.276	-0.305	RCN2	0.425	~amyloid_beta+(1|manifest)+cngeneson+pc_mito+brain_region+Sex	3.645	1184.000
ENSG00000124356	0.026	-0.015	0.047	-0.077	STAMBP	0.069	~amyloid_beta+(1|manifest)+cngeneson+pc_mito+brain_region+Sex	0.733	4785.000
ENSG00000134001	0.064	-0.015	0.033	-0.063	EIF2S1	0.116	~amyloid_beta+(1|manifest)+cngeneson+pc_mito+brain_region+Sex	0.590	5155.000
ENSG00000081870	0.146	-0.015	0.054	-0.084	HSPB11	0.202	~amyloid_beta+(1|manifest)+cngeneson+pc_mito+brain_region+Sex	0.569	5185.000
ENSG00000132300	0.115	-0.015	0.117	-0.147	PTCD3	0.171	~amyloid_beta+(1|manifest)+cngeneson+pc_mito+brain_region+Sex	1.797	2706.000
ENSG00000067704	0.023	-0.015	0.052	-0.082	IARS2	0.064	~amyloid_beta+(1|manifest)+cngeneson+pc_mito+brain_region+Sex	0.493	5380.000
ENSG00000118412	0.103	-0.015	0.100	-0.130	CASP8AP2	0.158	~amyloid_beta+(1|manifest)+cngeneson+pc_mito+brain_region+Sex	0.582	5166.000
ENSG00000175309	0.329	-0.015	0.249	-0.279	PHYKPL	0.381	~amyloid_beta+(1|manifest)+cngeneson+pc_mito+brain_region+Sex	3.685	1172.000
ENSG00000137275	0.078	-0.015	0.124	-0.154	RIPK1	0.133	~amyloid_beta+(1|manifest)+cngeneson+pc_mito+brain_region+Sex	1.634	2933.000
ENSG00000164168	0.132	-0.015	0.060	-0.090	[illegible]	0.189	~amyloid_beta+(1|manifest)+cngeneson+pc_mito+brain_region+Sex	0.634	5050.000
ENSG00000047188	0.061	-0.015	0.145	-0.175	YTHDC2	0.113	~amyloid_beta+(1|manifest)+cngeneson+pc_mito+brain_region+Sex	0.965	4213.000
ENSG00000162415	0.645	-0.015	0.181	-0.211	ZSWIM5	0.678	~amyloid_beta+(1|manifest)+cngeneson+pc_mito+brain_region+Sex	2.082	2333.000
ENSG00000116191	0.079	-0.015	0.081	-0.111	RALGPS2	0.134	~amyloid_beta+(1|manifest)+cngeneson+pc_mito+brain_region+Sex	0.697	4903.000
ENSG00000179021	0.026	-0.015	0.099	-0.129	C3orf38	0.068	~amyloid_beta+(1|manifest)+cngeneson+pc_mito+brain_region+Sex	0.867	4449.000
ENSG00000179454	0.089	-0.015	0.078	-0.108	KLHL28	0.144	~amyloid_beta+(1|manifest)+cngeneson+pc_mito+brain_region+Sex	1.002	4131.000
ENSG00000087266	0.287	-0.015	0.280	-0.311	SH3BP2	0.340	~amyloid_beta+(1|manifest)+cngeneson+pc_mito+brain_region+Sex	3.187	1433.000
ENSG00000089022	0.081	-0.015	0.055	-0.086	[illegible]	0.136	~amyloid_beta+(1|manifest)+cngeneson+pc_mito+brain_region+Sex	0.451	5451.000
ENSG00000141376	0.946	-0.015	0.186	-0.217	BCAS3	0.952	~amyloid_beta+(1|manifest)+cngeneson+pc_mito+brain_region+Sex	3.072	1496.000
ENSG00000114374	0.350	-0.015	0.005	-0.036	USP9Y	0.400	~amyloid_beta+(1|manifest)+cngeneson+pc_mito+brain_region+Sex	33.629	14.000
ENSG00000103051	0.143	-0.016	0.124	-0.155	COG4	0.199	~amyloid_beta+(1|manifest)+cngeneson+pc_mito+brain_region+Sex	1.360	3389.000
ENSG00000204271	0.011	-0.016	0.024	-0.055	SPIN3	0.042	~amyloid_beta+(1|manifest)+cngeneson+pc_mito+brain_region+Sex	0.287	5749.000
ENSG00000112697	0.061	-0.016	0.097	-0.129	TMEM30A	0.113	~amyloid_beta+(1|manifest)+cngeneson+pc_mito+brain_region+Sex	0.411	5545.000
ENSG00000160679	0.111	-0.016	0.076	-0.108	CHTOP	0.167	~amyloid_beta+(1|manifest)+cngeneson+pc_mito+brain_region+Sex	1.139	3840.000
ENSG00000176953	0.177	-0.016	0.142	-0.174	NFATC2IP	0.231	~amyloid_beta+(1|manifest)+cngeneson+pc_mito+brain_region+Sex	1.669	2870.000
ENSG00000123575	0.268	-0.016	0.134	-0.166	FAM199X	0.321	~amyloid_beta+(1|manifest)+cngeneson+pc_mito+brain_region+Sex	1.368	3378.000
ENSG00000145740	0.105	-0.016	0.055	-0.087	SLC30A5	0.161	~amyloid_beta+(1|manifest)+cngeneson+pc_mito+brain_region+Sex	0.765	4705.000
ENSG00000197586	0.106	-0.016	0.099	-0.132	ENTPD6	0.162	~amyloid_beta+(1|manifest)+cngeneson+pc_mito+brain_region+Sex	0.854	4486.000
ENSG00000003509	0.097	-0.016	0.036	-0.068	NDUFAF7	0.153	~amyloid_beta+(1|manifest)+cngeneson+pc_mito+brain_region+Sex	0.886	4408.000
ENSG00000100425	0.023	-0.016	0.081	-0.114	BRD1	0.064	~amyloid_beta+(1|manifest)+cngeneson+pc_mito+brain_region+Sex	1.169	3767.000
ENSG00000182628	0.043	-0.016	0.057	-0.090	SKA2	0.091	~amyloid_beta+(1|manifest)+cngeneson+pc_mito+brain_region+Sex	0.470	5417.000
ENSG00000131791	0.064	-0.017	0.064	-0.098	PRKAB2	0.117	~amyloid_beta+(1|manifest)+cngeneson+pc_mito+brain_region+Sex	0.498	5363.000
ENSG00000005486	0.232	-0.017	0.150	-0.184	RHBDD2	0.286	~amyloid_beta+(1|manifest)+cngeneson+pc_mito+brain_region+Sex	0.100	5953.000
ENSG00000178662	0.439	-0.017	0.252	-0.286	CSRNP3	0.484	~amyloid_beta+(1|manifest)+cngeneson+pc_mito+brain_region+Sex	1.456	3211.000
ENSG00000044115	0.736	-0.017	0.229	-0.263	CTNNA1	0.762	~amyloid_beta+(1|manifest)+cngeneson+pc_mito+brain_region+Sex	1.691	2843.000
ENSG00000139132	0.885	-0.017	0.202	-0.235	FGD4	0.898	~amyloid_beta+(1|manifest)+cngeneson+pc_mito+brain_region+Sex	4.369	927.000
ENSG00000166855	0.118	-0.017	0.031	-0.064	CLPX	0.175	~amyloid_beta+(1|manifest)+cngeneson+pc_mito+brain_region+Sex	0.443	5473.000
ENSG00000180228	0.089	-0.017	0.090	-0.124	PRKRA	0.144	~amyloid_beta+(1|manifest)+cngeneson+pc_mito+brain_region+Sex	1.092	3945.000
ENSG00000079335	0.781	-0.017	0.255	-0.289	CDC14A	0.802	~amyloid_beta+(1|manifest)+cngeneson+pc_mito+brain_region+Sex	0.661	4976.000
ENSG00000189091	0.105	-0.017	0.063	-0.097	SF3B3	0.161	~amyloid_beta+(1|manifest)+cngeneson+pc_mito+brain_region+Sex	0.962	4219.000
ENSG00000138399	0.208	-0.017	0.189	-0.223	FASTKD1	0.263	~amyloid_beta+(1|manifest)+cngeneson+pc_mito+brain_region+Sex	2.626	1794.000
ENSG00000116141	0.044	-0.017	0.251	-0.286	MARK1	0.093	~amyloid_beta+(1|manifest)+cngeneson+pc_mito+brain_region+Sex	2.265	2136.000
ENSG00000182504	0.107	-0.017	0.054	-0.089	CEP97	0.163	~amyloid_beta+(1|manifest)+cngeneson+pc_mito+brain_region+Sex	1.073	3984.000
ENSG00000189350	0.255	-0.017	0.459	-0.494	[illegible]	0.309	~amyloid_beta+(1|manifest)+cngeneson+pc_mito+brain_region+Sex	5.985	593.000
ENSG00000169718	0.106	-0.017	0.134	-0.169	DUS1L	0.162	~amyloid_beta+(1|manifest)+cngeneson+pc_mito+brain_region+Sex	1.592	2996.000
ENSG00000147065	0.356	-0.018	0.169	-0.204	MSN	0.407	~amyloid_beta+(1|manifest)+cngeneson+pc_mito+brain_region+Sex	3.957	1061.000
ENSG00000007923	0.076	-0.018	0.058	-0.093	DNAJC11	0.130	~amyloid_beta+(1|manifest)+cngeneson+pc_mito+brain_region+Sex	0.909	4356.000
ENSG00000172262	0.059	-0.018	0.153	-0.188	ZNF131	0.111	~amyloid_beta+(1|manifest)+cngeneson+pc_mito+brain_region+Sex	2.002	2426.000
ENSG00000075234	0.133	-0.018	0.124	-0.159	TTC38	0.190	~amyloid_beta+(1|manifest)+cngeneson+pc_mito+brain_region+Sex	1.638	2926.000
ENSG00000100722	0.128	-0.018	0.094	-0.130	ZC3H14	0.184	~amyloid_beta+(1|manifest)+cngeneson+pc_mito+brain_region+Sex	0.897	4378.000
ENSG00000079332	0.048	-0.018	0.030	-0.066	SAR1A	0.098	~amyloid_beta+(1|manifest)+cngeneson+pc_mito+brain_region+Sex	0.124	5936.000
ENSG00000150093	0.455	-0.018	0.235	-0.271	ITGB1	0.500	~amyloid_beta+(1|manifest)+cngeneson+pc_mito+brain_region+Sex	5.875	612.000
ENSG00000148399	0.225	-0.018	0.125	-0.161	DPH7	0.280	~amyloid_beta+(1|manifest)+cngeneson+pc_mito+brain_region+Sex	1.487	3164.000
ENSG00000159214	0.065	-0.018	0.167	-0.203	CCDC24	0.118	~amyloid_beta+(1|manifest)+cngeneson+pc_mito+brain_region+Sex	3.156	1450.000
ENSG00000137996	0.040	-0.018	0.025	-0.062	RTCA	0.088	~amyloid_beta+(1|manifest)+cngeneson+pc_mito+brain_region+Sex	0.583	5163.000

ENSG00000106723	0.481	-0.019	0.232	-0.270	SPIN1	0.525	~amyloid_beta+(1|manifest)+cngeneson+pc_mito+brain_region+Sex	2.671	1752.000
ENSG00000096401	0.060	-0.019	0.047	-0.084	CDC5L	0.111	~amyloid_beta+(1|manifest)+cngeneson+pc_mito+brain_region+Sex	0.444	5471.000
ENSG00000128596	0.067	-0.019	0.108	-0.145	CCDC136	0.120	~amyloid_beta+(1|manifest)+cngeneson+pc_mito+brain_region+Sex	1.017	4104.000
ENSG00000129083	0.022	-0.019	0.037	-0.075	COPB1	0.063	~amyloid_beta+(1|manifest)+cngeneson+pc_mito+brain_region+Sex	0.709	4851.000
ENSG00000116198	0.282	-0.019	0.093	-0.131	CEP104	0.335	~amyloid_beta+(1|manifest)+cngeneson+pc_mito+brain_region+Sex	1.067	3996.000
ENSG00000003756	0.234	-0.019	0.140	-0.178	RBM5	0.288	~amyloid_beta+(1|manifest)+cngeneson+pc_mito+brain_region+Sex	2.553	1854.000
ENSG00000163798	0.092	-0.019	0.026	-0.064	SLC4A1AP	0.147	~amyloid_beta+(1|manifest)+cngeneson+pc_mito+brain_region+Sex	0.199	5872.000
ENSG00000047315	0.055	-0.019	0.048	-0.086	POLR2B	0.105	~amyloid_beta+(1|manifest)+cngeneson+pc_mito+brain_region+Sex	0.831	4538.000
ENSG00000184432	0.041	-0.019	0.063	-0.101	COPB2	0.088	~amyloid_beta+(1|manifest)+cngeneson+pc_mito+brain_region+Sex	0.817	4581.000
ENSG00000148429	0.112	-0.019	0.169	-0.207	USP6NL	0.168	~amyloid_beta+(1|manifest)+cngeneson+pc_mito+brain_region+Sex	1.554	3043.000
ENSG00000221978	0.477	-0.019	0.380	-0.419	CCNL2	0.522	~amyloid_beta+(1|manifest)+cngeneson+pc_mito+brain_region+Sex	8.605	339.000
ENSG00000094914	0.240	-0.019	0.131	-0.169	AAAS	0.293	~amyloid_beta+(1|manifest)+cngeneson+pc_mito+brain_region+Sex	2.101	2312.000
ENSG00000117305	0.035	-0.019	0.052	-0.091	HMGCL	0.081	~amyloid_beta+(1|manifest)+cngeneson+pc_mito+brain_region+Sex	0.768	4697.000
ENSG00000080298	0.837	-0.019	0.097	-0.135	RFX3	0.854	~amyloid_beta+(1|manifest)+cngeneson+pc_mito+brain_region+Sex	1.761	2735.000
ENSG00000068654	0.080	-0.019	0.063	-0.102	POLR1A	0.134	~amyloid_beta+(1|manifest)+cngeneson+pc_mito+brain_region+Sex	0.741	4767.000
ENSG00000109339	0.774	-0.019	0.072	-0.111	MAPK10	0.796	~amyloid_beta+(1|manifest)+cngeneson+pc_mito+brain_region+Sex	2.827	1649.000
ENSG00000081014	0.016	-0.019	0.025	-0.064	AP4E1	0.053	~amyloid_beta+(1|manifest)+cngeneson+pc_mito+brain_region+Sex	0.659	4981.000
ENSG00000178188	0.219	-0.020	0.207	-0.246	SH2B1	0.274	~amyloid_beta+(1|manifest)+cngeneson+pc_mito+brain_region+Sex	2.303	2102.000
ENSG00000136267	0.714	-0.020	0.493	-0.532	DGKB	0.742	~amyloid_beta+(1|manifest)+cngeneson+pc_mito+brain_region+Sex	3.380	1330.000
ENSG00000111859	0.401	-0.020	0.136	-0.175	NEDD9	0.448	~amyloid_beta+(1|manifest)+cngeneson+pc_mito+brain_region+Sex	1.260	3586.000
ENSG00000102974	0.047	-0.020	0.117	-0.156	CTCF	0.096	~amyloid_beta+(1|manifest)+cngeneson+pc_mito+brain_region+Sex	1.086	3959.000
ENSG00000127804	0.035	-0.020	0.065	-0.104	METTL16	0.081	~amyloid_beta+(1|manifest)+cngeneson+pc_mito+brain_region+Sex	0.565	5197.000
ENSG00000147100	0.019	-0.020	0.138	-0.178	SLC16A2	0.058	~amyloid_beta+(1|manifest)+cngeneson+pc_mito+brain_region+Sex	1.276	3555.000
ENSG00000123607	0.012	-0.020	0.048	-0.088	TTC21B	0.045	~amyloid_beta+(1|manifest)+cngeneson+pc_mito+brain_region+Sex	0.746	4750.000
ENSG00000055211	0.052	-0.020	0.034	-0.074	GINM1	0.101	~amyloid_beta+(1|manifest)+cngeneson+pc_mito+brain_region+Sex	0.740	4768.000
ENSG00000097033	0.400	-0.020	0.175	-0.215	SH3GLB1	0.448	~amyloid_beta+(1|manifest)+cngeneson+pc_mito+brain_region+Sex	2.930	1589.000
ENSG00000133640	0.070	-0.020	0.185	-0.225	LRRIQ1	0.124	~amyloid_beta+(1|manifest)+cngeneson+pc_mito+brain_region+Sex	1.287	3531.000
ENSG00000118363	0.205	-0.020	0.090	-0.130	SPCS2	0.260	~amyloid_beta+(1|manifest)+cngeneson+pc_mito+brain_region+Sex	0.184	5894.000
ENSG00000171467	0.323	-0.020	0.146	-0.186	ZNF318	0.375	~amyloid_beta+(1|manifest)+cngeneson+pc_mito+brain_region+Sex	1.607	2973.000
ENSG00000135778	0.123	-0.020	0.054	-0.094	NTPCR	0.180	~amyloid_beta+(1|manifest)+cngeneson+pc_mito+brain_region+Sex	0.952	4249.000
ENSG00000188811	0.072	-0.020	0.102	-0.142	NHLRC3	0.126	~amyloid_beta+(1|manifest)+cngeneson+pc_mito+brain_region+Sex	1.050	4025.000
ENSG00000149084	0.175	-0.020	0.135	-0.175	HSD17B12	0.230	~amyloid_beta+(1|manifest)+cngeneson+pc_mito+brain_region+Sex	1.147	3818.000
ENSG00000188312	0.862	-0.020	0.165	-0.206	CENPP	0.875	~amyloid_beta+(1|manifest)+cngeneson+pc_mito+brain_region+Sex	1.423	3285.000
ENSG00000071054	0.916	-0.020	0.132	-0.173	MAP4K4	0.926	~amyloid_beta+(1|manifest)+cngeneson+pc_mito+brain_region+Sex	5.740	639.000
ENSG00000126456	0.140	-0.020	0.132	-0.173	IRF3	0.197	~amyloid_beta+(1|manifest)+cngeneson+pc_mito+brain_region+Sex	2.141	2271.000
ENSG00000152242	0.083	-0.020	0.067	-0.107	C18orf25	0.138	~amyloid_beta+(1|manifest)+cngeneson+pc_mito+brain_region+Sex	0.303	5730.000
ENSG00000175727	0.131	-0.020	0.141	-0.182	MLXIP	0.188	~amyloid_beta+(1|manifest)+cngeneson+pc_mito+brain_region+Sex	1.907	2546.000
ENSG00000197056	0.186	-0.020	0.074	-0.115	ZMYM1	0.240	~amyloid_beta+(1|manifest)+cngeneson+pc_mito+brain_region+Sex	0.810	4601.000
ENSG00000005238	0.131	-0.020	0.125	-0.166	FAM214B	0.187	~amyloid_beta+(1|manifest)+cngeneson+pc_mito+brain_region+Sex	1.149	3816.000
ENSG00000122692	0.050	-0.021	0.021	-0.062	SMU1	0.100	~amyloid_beta+(1|manifest)+cngeneson+pc_mito+brain_region+Sex	0.242	5817.000
ENSG00000153107	0.126	-0.021	0.046	-0.087	ANAPC1	0.182	~amyloid_beta+(1|manifest)+cngeneson+pc_mito+brain_region+Sex	0.955	4238.000
ENSG00000125633	0.087	-0.021	0.109	-0.150	CCDC93	0.142	~amyloid_beta+(1|manifest)+cngeneson+pc_mito+brain_region+Sex	1.837	2645.000
ENSG00000132824	0.190	-0.021	0.076	-0.117	SERINC3	0.245	~amyloid_beta+(1|manifest)+cngeneson+pc_mito+brain_region+Sex	0.278	5767.000
ENSG00000119638	0.123	-0.021	0.110	-0.152	NEK9	0.180	~amyloid_beta+(1|manifest)+cngeneson+pc_mito+brain_region+Sex	1.867	2606.000
ENSG00000155008	0.166	-0.021	0.045	-0.088	APOOL	0.221	~amyloid_beta+(1|manifest)+cngeneson+pc_mito+brain_region+Sex	0.358	5637.000
ENSG00000055044	0.088	-0.021	0.056	-0.098	NOP58	0.143	~amyloid_beta+(1|manifest)+cngeneson+pc_mito+brain_region+Sex	0.346	5658.000
ENSG00000139737	0.881	-0.021	0.183	-0.225	SLAIN1	0.894	~amyloid_beta+(1|manifest)+cngeneson+pc_mito+brain_region+Sex	6.788	494.000
ENSG00000163393	0.614	-0.021	0.195	-0.238	SLC22A15	0.651	~amyloid_beta+(1|manifest)+cngeneson+pc_mito+brain_region+Sex	1.823	2664.000
ENSG00000164258	0.133	-0.021	0.088	-0.130	NDUFS4	0.190	~amyloid_beta+(1|manifest)+cngeneson+pc_mito+brain_region+Sex	1.077	3972.000
ENSG00000076108	0.063	-0.021	0.122	-0.164	BAZ2A	0.115	~amyloid_beta+(1|manifest)+cngeneson+pc_mito+brain_region+Sex	1.138	3841.000
ENSG00000055070	0.080	-0.021	0.061	-0.104	SZRD1	0.135	~amyloid_beta+(1|manifest)+cngeneson+pc_mito+brain_region+Sex	0.419	5527.000
ENSG00000123505	0.030	-0.021	0.088	-0.131	AMD1	0.075	~amyloid_beta+(1|manifest)+cngeneson+pc_mito+brain_region+Sex	1.195	3716.000
ENSG00000076604	0.381	-0.021	0.135	-0.177	TRAF4	0.431	~amyloid_beta+(1|manifest)+cngeneson+pc_mito+brain_region+Sex	1.883	2585.000
ENSG00000188938	0.231	-0.021	0.186	-0.229	[illegible]	0.285	~amyloid_beta+(1|manifest)+cngeneson+pc_mito+brain_region+Sex	2.095	2320.000
ENSG00000164323	0.140	-0.021	0.044	-0.086	CFAP97	0.196	~amyloid_beta+(1|manifest)+cngeneson+pc_mito+brain_region+Sex	0.534	5271.000
ENSG00000143549	0.040	-0.022	0.074	-0.117	TPM3	0.087	~amyloid_beta+(1|manifest)+cngeneson+pc_mito+brain_region+Sex	0.856	4478.000
ENSG00000049883	0.028	-0.022	0.039	-0.082	PTCD2	0.072	~amyloid_beta+(1|manifest)+cngeneson+pc_mito+brain_region+Sex	0.326	5683.000
ENSG00000108578	0.118	-0.022	0.042	-0.086	BLMH	0.174	~amyloid_beta+(1|manifest)+cngeneson+pc_mito+brain_region+Sex	0.799	4637.000
ENSG00000112531	0.541	-0.022	0.076	-0.119	QKI	0.582	~amyloid_beta+(1|manifest)+cngeneson+pc_mito+brain_region+Sex	3.690	1166.000
ENSG00000182359	0.160	-0.022	0.066	-0.109	KBTBD3	0.215	~amyloid_beta+(1|manifest)+cngeneson+pc_mito+brain_region+Sex	0.504	5344.000
ENSG00000162129	0.065	-0.022	0.135	-0.179	CLPB	0.118	~amyloid_beta+(1|manifest)+cngeneson+pc_mito+brain_region+Sex	2.171	2235.000
ENSG00000169554	0.922	-0.022	0.208	-0.252	ZEB2	0.931	~amyloid_beta+(1|manifest)+cngeneson+pc_mito+brain_region+Sex	1.730	2780.000
ENSG00000168813	0.015	-0.022	0.057	-0.101	ZNF507	0.050	~amyloid_beta+(1|manifest)+cngeneson+pc_mito+brain_region+Sex	0.546	5239.000
ENSG00000065150	0.021	-0.022	0.106	-0.150	IPO5	0.060	~amyloid_beta+(1|manifest)+cngeneson+pc_mito+brain_region+Sex	0.667	4966.000
ENSG00000137267	0.370	-0.022	0.167	-0.212	TUBB2A	0.420	~amyloid_beta+(1|manifest)+cngeneson+pc_mito+brain_region+Sex	1.511	3125.000
ENSG00000197226	0.235	-0.022	0.124	-0.169	TBC1D9B	0.289	~amyloid_beta+(1|manifest)+cngeneson+pc_mito+brain_region+Sex	1.675	2861.000
ENSG00000114650	0.130	-0.022	0.172	-0.217	SCAP	0.186	~amyloid_beta+(1|manifest)+cngeneson+pc_mito+brain_region+Sex	1.975	2461.000
ENSG00000128595	0.127	-0.022	0.138	-0.183	CALU	0.183	~amyloid_beta+(1|manifest)+cngeneson+pc_mito+brain_region+Sex	2.324	2077.000
ENSG00000120686	0.026	-0.022	0.026	-0.071	UFM1	0.068	~amyloid_beta+(1|manifest)+cngeneson+pc_mito+brain_region+Sex	0.123	5940.000
ENSG00000170075	0.675	-0.022	0.394	-0.439	GPR37L1	0.706	~amyloid_beta+(1|manifest)+cngeneson+pc_mito+brain_region+Sex	2.604	1810.000
ENSG00000125304	0.063	-0.022	0.061	-0.106	TM9SF2	0.115	~amyloid_beta+(1|manifest)+cngeneson+pc_mito+brain_region+Sex	0.602	5124.000
ENSG00000184939	0.035	-0.023	0.062	-0.107	ZFP90	0.081	~amyloid_beta+(1|manifest)+cngeneson+pc_mito+brain_region+Sex	0.652	4993.000
ENSG00000174469	0.115	-0.023	0.085	-0.130	CNTNAP2	0.172	~amyloid_beta+(1|manifest)+cngeneson+pc_mito+brain_region+Sex	0.897	4381.000
ENSG00000069956	0.128	-0.023	0.098	-0.144	MAPK6	0.184	~amyloid_beta+(1|manifest)+cngeneson+pc_mito+brain_region+Sex	0.726	4805.000
ENSG00000133056	0.119	-0.023	0.126	-0.172	PIK3C2B	0.176	~amyloid_beta+(1|manifest)+cngeneson+pc_mito+brain_region+Sex	1.990	2448.000
ENSG00000187735	0.043	-0.023	0.069	-0.115	TCEA1	0.092	~amyloid_beta+(1|manifest)+cngeneson+pc_mito+brain_region+Sex	0.158	5916.000
ENSG00000117475	0.109	-0.023	0.093	-0.139	BLZF1	0.165	~amyloid_beta+(1|manifest)+cngeneson+pc_mito+brain_region+Sex	0.955	4237.000
ENSG00000005812	0.033	-0.023	0.055	-0.101	FBXL3	0.079	~amyloid_beta+(1|manifest)+cngeneson+pc_mito+brain_region+Sex	0.772	4683.000
ENSG00000124243	0.021	-0.023	0.100	-0.146	BCAS4	0.060	~amyloid_beta+(1|manifest)+cngeneson+pc_mito+brain_region+Sex	0.292	5743.000
ENSG00000083223	0.032	-0.023	0.082	-0.129	TUT7	0.077	~amyloid_beta+(1|manifest)+cngeneson+pc_mito+brain_region+Sex	1.104	3915.000
ENSG00000118292	0.272	-0.023	0.180	-0.227	C1orf54	0.325	~amyloid_beta+(1|manifest)+cngeneson+pc_mito+brain_region+Sex	2.827	1648.000
ENSG00000134138	0.782	-0.023	0.080	-0.127	MEIS2	0.803	~amyloid_beta+(1|manifest)+cngeneson+pc_mito+brain_region+Sex	3.537	1229.000
ENSG00000134250	0.786	-0.023	0.144	-0.191	NOTCH2	0.807	~amyloid_beta+(1|manifest)+cngeneson+pc_mito+brain_region+Sex	1.228	3650.000
ENSG00000181007	0.040	-0.023	0.030	-0.077	ZFP82	0.088	~amyloid_beta+(1|manifest)+cngeneson+pc_mito+brain_region+Sex	0.474	5414.000
ENSG00000139697	0.130	-0.023	0.138	-0.185	SBNO1	0.186	~amyloid_beta+(1|manifest)+cngeneson+pc_mito+brain_region+Sex	1.928	2525.000
ENSG00000057019	0.071	-0.023	0.100	-0.147	DCBLD2	0.125	~amyloid_beta+(1|manifest)+cngeneson+pc_mito+brain_region+Sex	1.375	3370.000
ENSG00000127952	0.033	-0.024	0.105	-0.152	STYXL1	0.078	~amyloid_beta+(1|manifest)+cngeneson+pc_mito+brain_region+Sex	1.069	3990.000
ENSG00000113712	0.093	-0.024	0.101	-0.148	CSNK1A1	0.148	~amyloid_beta+(1|manifest)+cngeneson+pc_mito+brain_region+Sex	3.330	1353.000
ENSG00000143776	0.811	-0.024	0.130	-0.178	CDC42BPA	0.830	~amyloid_beta+(1|manifest)+cngeneson+pc_mito+brain_region+Sex	3.619	1196.000
ENSG00000071243	0.197	-0.024	0.072	-0.119	ING3	0.251	~amyloid_beta+(1|manifest)+cngeneson+pc_mito+brain_region+Sex	0.528	5290.000
ENSG00000122591	0.379	-0.024	0.051	-0.099	FAM126A	0.429	~amyloid_beta+(1|manifest)+cngeneson+pc_mito+brain_region+Sex	1.387	3346.000
ENSG00000138674	0.025	-0.024	0.088	-0.136	SEC31A	0.067	~amyloid_beta+(1|manifest)+cngeneson+pc_mito+brain_region+Sex	1.345	3418.000
ENSG00000048740	0.908	-0.024	0.096	-0.144	CELF2	0.919	~amyloid_beta+(1|manifest)+cngeneson+pc_mito+brain_region+Sex	7.364	431.000
ENSG00000135315	0.084	-0.024	0.138	-0.186	CEP162	0.138	~amyloid_beta+(1|manifest)+cngeneson+pc_mito+brain_region+Sex	1.291	3526.000
ENSG00000009413	0.662	-0.024	0.073	-0.121	REV3L	0.694	~amyloid_beta+(1|manifest)+cngeneson+pc_mito+brain_region+Sex	1.180	3744.000
ENSG00000171606	0.100	-0.024	0.102	-0.150	ZNF274	0.155	~amyloid_beta+(1|manifest)+cngeneson+pc_mito+brain_region+Sex	0.224	5842.000
ENSG00000166128	0.162	-0.024	0.069	-0.117	RAB8B	0.217	~amyloid_beta+(1|manifest)+cngeneson+pc_mito+brain_region+Sex	0.474	5410.000
ENSG00000135823	0.043	-0.024	0.045	-0.094	STX6	0.091	~amyloid_beta+(1|manifest)+cngeneson+pc_mito+brain_region+Sex	0.627	5068.000
ENSG00000186687	0.031	-0.024	0.021	-0.069	LYRM7	0.075	~amyloid_beta+(1|manifest)+cngeneson+pc_mito+brain_region+Sex	0.540	5261.000
ENSG00000185864	0.041	-0.024	0.124	-0.172	NPIPB4	0.089	~amyloid_beta+(1|manifest)+cngeneson+pc_mito+brain_region+Sex	1.080	3968.000
ENSG00000048707	0.587	-0.024	0.199	-0.248	VPS13D	0.624	~amyloid_beta+(1|manifest)+cngeneson+pc_mito+brain_region+Sex	4.189	991.000
ENSG00000101161	0.039	-0.025	0.098	-0.147	PRPF6	0.086	~amyloid_beta+(1|manifest)+cngeneson+pc_mito+brain_region+Sex	1.261	3582.000
ENSG00000102471	0.041	-0.025	0.049	-0.098	NDFIP2	0.089	~amyloid_beta+(1|manifest)+cngeneson+pc_mito+brain_region+Sex	0.504	5346.000
ENSG00000135424	0.730	-0.025	0.364	-0.414	ITGA7	0.757	~amyloid_beta+(1|manifest)+cngeneson+pc_mito+brain_region+Sex	10.303	257.000
ENSG00000134490	0.420	-0.025	0.401	-0.450	TMEM241	0.467	~amyloid_beta+(1|manifest)+cngeneson+pc_mito+brain_region+Sex	7.858	385.000
ENSG00000118420	0.368	-0.025	0.039	-0.089	UBE3D	0.418	~amyloid_beta+(1|manifest)+cngeneson+pc_mito+brain_region+Sex	0.861	4463.000
ENSG00000069329	0.018	-0.025	0.041	-0.091	VPS35	0.055	~amyloid_beta+(1|manifest)+cngeneson+pc_mito+brain_region+Sex	0.253	5804.000
ENSG00000144895	0.110	-0.025	0.067	-0.117	EIF2A	0.166	~amyloid_beta+(1|manifest)+cngeneson+pc_mito+brain_region+Sex	0.474	5411.000
ENSG00000185818	0.957	-0.025	0.627	-0.677	NAT8L	0.961	~amyloid_beta+(1|manifest)+cngeneson+pc_mito+brain_region+Sex	10.277	259.000
ENSG00000221994	0.004	-0.025	0.043	-0.093	ZNF630	0.025	~amyloid_beta+(1|manifest)+cngeneson+pc_mito+brain_region+Sex	0.820	4571.000
ENSG00000089053	0.275	-0.025	0.170	-0.220	ANAPC5	0.328	~amyloid_beta+(1|manifest)+cngeneson+pc_mito+brain_region+Sex	2.626	1793.000
ENSG00000140830	0.064	-0.025	0.089	-0.139	TXNL4B	0.117	~amyloid_beta+(1|manifest)+cngeneson+pc_mito+brain_region+Sex	0.752	4737.000
ENSG00000119541	0.031	-0.025	0.064	-0.114	VPS4B	0.075	~amyloid_beta+(1|manifest)+cngeneson+pc_mito+brain_region+Sex	1.130	3862.000
ENSG00000160191	0.304	-0.025	0.237	-0.287	PDE9A	0.356	~amyloid_beta+(1|manifest)+cngeneson+pc_mito+brain_region+Sex	2.254	2149.000
ENSG00000130363	0.080	-0.025	0.098	-0.149	RSPH3	0.135	~amyloid_beta+(1|manifest)+cngeneson+pc_mito+brain_region+Sex	0.280	5765.000
ENSG00000185624	0.246	-0.026	0.203	-0.254	P4HB	0.300	~amyloid_beta+(1|manifest)+cngeneson+pc_mito+brain_region+Sex	0.915	4341.000
ENSG00000133863	0.328	-0.026	0.130	-0.182	TEX15	0.380	~amyloid_beta+(1|manifest)+cngeneson+pc_mito+brain_region+Sex	1.403	3313.000
ENSG00000116199	0.057	-0.026	0.045	-0.096	FAM20B	0.108	~amyloid_beta+(1|manifest)+cngeneson+pc_mito+brain_region+Sex	0.756	4725.000
ENSG00000048342	0.127	-0.026	0.192	-0.243	CC2D2A	0.184	~amyloid_beta+(1|manifest)+cngeneson+pc_mito+brain_region+Sex	1.881	2588.000
ENSG00000206560	0.095	-0.026	0.385	-0.436	ANKRD28	0.150	~amyloid_beta+(1|manifest)+cngeneson+pc_mito+brain_region+Sex	5.626	661.000
ENSG00000106701	0.048	-0.026	0.119	-0.171	FSD1L	0.097	~amyloid_beta+(1|manifest)+cngeneson+pc_mito+brain_region+Sex	1.595	2992.000
ENSG00000131943	0.089	-0.026	0.081	-0.132	C19orf12	0.144	~amyloid_beta+(1|manifest)+cngeneson+pc_mito+brain_region+Sex	0.754	4731.000
ENSG00000100714	0.031	-0.026	0.051	-0.103	MTHFD1	0.076	~amyloid_beta+(1|manifest)+cngeneson+pc_mito+brain_region+Sex	1.022	4095.000
ENSG00000008282	0.152	-0.026	0.061	-0.113	SYPL1	0.208	~amyloid_beta+(1|manifest)+cngeneson+pc_mito+brain_region+Sex	0.261	5795.000
ENSG00000112320	0.578	-0.026	0.150	-0.202	SOBP	0.616	~amyloid_beta+(1|manifest)+cngeneson+pc_mito+brain_region+Sex	1.635	2930.000
ENSG00000154124	0.014	-0.026	0.013	-0.065	OTULIN	0.048	~amyloid_beta+(1|manifest)+cngeneson+pc_mito+brain_region+Sex	0.279	5766.000
ENSG00000137692	0.035	-0.026	0.087	-0.139	DCUN1D5	0.080	~amyloid_beta+(1|manifest)+cngeneson+pc_mito+brain_region+Sex	0.894	4388.000
ENSG00000152270	0.849	-0.026	0.521	-0.573	PDE3B	0.864	~amyloid_beta+(1|manifest)+cngeneson+pc_mito+brain_region+Sex	6.127	576.000
ENSG00000164663	0.137	-0.026	0.112	-0.164	USP49	0.194	~amyloid_beta+(1|manifest)+cngeneson+pc_mito+brain_region+Sex	1.094	3942.000
ENSG00000180628	0.904	-0.026	0.230	-0.283	PCGF5	0.915	~amyloid_beta+(1|manifest)+cngeneson+pc_mito+brain_region+Sex	4.783	828.000
ENSG00000088808	0.075	-0.026	0.138	-0.190	PPP1R13B	0.129	~amyloid_beta+(1|manifest)+cngeneson+pc_mito+brain_region+Sex	1.209	3685.000
ENSG00000110048	0.060	-0.026	0.085	-0.138	OSBP	0.112	~amyloid_beta+(1|manifest)+cngeneson+pc_mito+brain_region+Sex	1.198	3712.000
ENSG00000135506	0.265	-0.027	0.082	-0.135	OS9	0.318	~amyloid_beta+(1|manifest)+cngeneson+pc_mito+brain_region+Sex	1.017	4103.000
ENSG00000073969	0.021	-0.027	0.020	-0.073	NSF	0.061	~amyloid_beta+(1|manifest)+cngeneson+pc_mito+brain_region+Sex	0.275	5773.000
ENSG00000167740	0.187	-0.027	0.174	-0.227	CYB5D2	0.241	~amyloid_beta+(1|manifest)+cngeneson+pc_mito+brain_region+Sex	2.775	1687.000
ENSG00000078747	0.202	-0.027	0.183	-0.237	ITCH	0.257	~amyloid_beta+(1|manifest)+cngeneson+pc_mito+brain_region+Sex	3.427	1295.000
ENSG00000136052	0.131	-0.027	0.098	-0.151	SLC41A2	0.188	~amyloid_beta+(1|manifest)+cngeneson+pc_mito+brain_region+Sex	2.500	1904.000
ENSG00000018699	0.133	-0.027	0.097	-0.150	TTC27	0.189	~amyloid_beta+(1|manifest)+cngeneson+pc_mito+brain_region+Sex	0.632	5052.000
ENSG00000103942	0.115	-0.027	0.082	-0.136	HOMER2	0.172	~amyloid_beta+(1|manifest)+cngeneson+pc_mito+brain_region+Sex	0.467	5427.000
ENSG00000151743	0.314	-0.027	0.065	-0.118	AMN1	0.366	~amyloid_beta+(1|manifest)+cngeneson+pc_mito+brain_region+Sex	0.986	4176.000
ENSG00000205937	0.158	-0.027	0.109	-0.163	RNPS1	0.213	~amyloid_beta+(1|manifest)+cngeneson+pc_mito+brain_region+Sex	1.494	3147.000
ENSG00000168917	0.384	-0.027	0.402	-0.456	SLC35G2	0.433	~amyloid_beta+(1|manifest)+cngeneson+pc_mito+brain_region+Sex	4.407	913.000
ENSG00000076242	0.014	-0.027	0.049	-0.103	MLH1	0.047	~amyloid_beta+(1|manifest)+cngeneson+pc_mito+brain_region+Sex	0.241	5820.000
ENSG00000139263	0.012	-0.027	0.042	-0.097	LRIG3	0.043	~amyloid_beta+(1|manifest)+cngeneson+pc_mito+brain_region+Sex	0.282	5762.000
ENSG00000106346	0.038	-0.027	0.084	-0.138	USP42	0.085	~amyloid_beta+(1|manifest)+cngeneson+pc_mito+brain_region+Sex	1.155	3803.000
ENSG00000173786	0.150	-0.027	0.060	-0.115	CNP	0.206	~amyloid_beta+(1|manifest)+cngeneson+pc_mito+brain_region+Sex	0.339	5664.000
ENSG00000086619	0.029	-0.027	0.067	-0.122	ERO1B	0.073	~amyloid_beta+(1|manifest)+cngeneson+pc_mito+brain_region+Sex	0.906	4367.000
ENSG00000198586	0.701	-0.027	0.152	-0.207	TLK1	0.730	~amyloid_beta+(1|manifest)+cngeneson+pc_mito+brain_region+Sex	4.325	941.000
ENSG00000062716	0.038	-0.027	0.058	-0.113	VMP1	0.086	~amyloid_beta+(1|manifest)+cngeneson+pc_mito+brain_region+Sex	0.788	4657.000
ENSG00000182670	0.315	-0.027	0.140	-0.194	TTC3	0.367	~amyloid_beta+(1|manifest)+cngeneson+pc_mito+brain_region+Sex	2.110	2300.000
ENSG00000101004	0.103	-0.027	0.206	-0.261	NINL	0.158	~amyloid_beta+(1|manifest)+cngeneson+pc_mito+brain_region+Sex	3.592	1210.000
ENSG00000059758	0.395	-0.027	0.156	-0.211	CDK17	0.444	~amyloid_beta+(1|manifest)+cngeneson+pc_mito+brain_region+Sex	1.101	3926.000
ENSG00000056487	0.749	-0.027	0.182	-0.237	PHF21B	0.775	~amyloid_beta+(1|manifest)+cngeneson+pc_mito+brain_region+Sex	1.445	3239.000
ENSG00000197170	0.020	-0.028	0.012	-0.067	PSMD12	0.060	~amyloid_beta+(1|manifest)+cngeneson+pc_mito+brain_region+Sex	0.264	5791.000
ENSG00000126581	0.033	-0.028	0.015	-0.071	BECN1	0.078	~amyloid_beta+(1|manifest)+cngeneson+pc_mito+brain_region+Sex	0.372	5614.000
ENSG00000179387	0.010	-0.028	0.019	-0.075	ELMOD2	0.041	~amyloid_beta+(1|manifest)+cngeneson+pc_mito+brain_region+Sex	0.370	5618.000
ENSG00000072415	0.050	-0.028	0.074	-0.129	MPP5	0.100	~amyloid_beta+(1|manifest)+cngeneson+pc_mito+brain_region+Sex	1.399	3324.000
ENSG00000153707	0.962	-0.028	0.282	-0.338	PTPRD	0.966	~amyloid_beta+(1|manifest)+cngeneson+pc_mito+brain_region+Sex	6.190	564.000
ENSG00000205683	0.785	-0.028	0.302	-0.357	DPF3	0.806	~amyloid_beta+(1|manifest)+cngeneson+pc_mito+brain_region+Sex	3.551	1225.000
ENSG00000031003	0.319	-0.028	0.061	-0.117	FAM13B	0.371	~amyloid_beta+(1|manifest)+cngeneson+pc_mito+brain_region+Sex	1.867	2607.000
ENSG00000134909	0.648	-0.028	0.099	-0.156	ARHGAP32	0.680	~amyloid_beta+(1|manifest)+cngeneson+pc_mito+brain_region+Sex	1.950	2493.000
ENSG00000057252	0.110	-0.028	0.083	-0.140	SOAT1	0.166	~amyloid_beta+(1|manifest)+cngeneson+pc_mito+brain_region+Sex	1.098	3933.000
ENSG00000169857	0.497	-0.028	0.111	-0.167	AVEN	0.541	~amyloid_beta+(1|manifest)+cngeneson+pc_mito+brain_region+Sex	1.961	2479.000
ENSG00000109775	0.129	-0.028	0.026	-0.082	UFSP2	0.186	~amyloid_beta+(1|manifest)+cngeneson+pc_mito+brain_region+Sex	0.431	5507.000
ENSG00000068366	0.070	-0.028	0.062	-0.118	ACSL4	0.124	~amyloid_beta+(1|manifest)+cngeneson+pc_mito+brain_region+Sex	1.463	3200.000
ENSG00000171103	0.045	-0.028	0.040	-0.096	TRMT61B	0.094	~amyloid_beta+(1|manifest)+cngeneson+pc_mito+brain_region+Sex	0.225	5840.000
ENSG00000065548	0.074	-0.028	0.059	-0.116	ZC3H15	0.128	~amyloid_beta+(1|manifest)+cngeneson+pc_mito+brain_region+Sex	1.126	3868.000
ENSG00000142655	0.082	-0.028	0.081	-0.137	PEX14	0.137	~amyloid_beta+(1|manifest)+cngeneson+pc_mito+brain_region+Sex	0.823	4561.000
ENSG00000152558	0.050	-0.028	0.065	-0.121	TMEM123	0.099	~amyloid_beta+(1|manifest)+cngeneson+pc_mito+brain_region+Sex	0.417	5530.000
ENSG00000177889	0.164	-0.028	0.041	-0.097	UBE2N	0.219	~amyloid_beta+(1|manifest)+cngeneson+pc_mito+brain_region+Sex	0.496	5371.000
ENSG00000180376	0.052	-0.028	0.116	-0.173	CCDC66	0.102	~amyloid_beta+(1|manifest)+cngeneson+pc_mito+brain_region+Sex	1.877	2592.000
ENSG00000184060	0.188	-0.028	0.131	-0.188	ADAP2	0.242	~amyloid_beta+(1|manifest)+cngeneson+pc_mito+brain_region+Sex	1.490	3158.000
ENSG00000186432	0.060	-0.028	0.080	-0.137	KPNA4	0.112	~amyloid_beta+(1|manifest)+cngeneson+pc_mito+brain_region+Sex	1.437	3252.000
ENSG00000183690	0.054	-0.029	0.118	-0.175	EFHC2	0.104	~amyloid_beta+(1|manifest)+cngeneson+pc_mito+brain_region+Sex	0.590	5154.000
ENSG00000198677	0.072	-0.029	0.090	-0.148	TTC37	0.126	~amyloid_beta+(1|manifest)+cngeneson+pc_mito+brain_region+Sex	0.826	4548.000
ENSG00000108219	0.073	-0.029	0.091	-0.149	TSPAN14	0.126	~amyloid_beta+(1|manifest)+cngeneson+pc_mito+brain_region+Sex	0.815	4586.000
ENSG00000070061	0.108	-0.029	0.072	-0.129	ELP1	0.164	~amyloid_beta+(1|manifest)+cngeneson+pc_mito+brain_region+Sex	1.537	3073.000
ENSG00000070371	0.199	-0.029	0.112	-0.170	CLTCL1	0.253	~amyloid_beta+(1|manifest)+cngeneson+pc_mito+brain_region+Sex	1.259	3587.000
ENSG00000144635	0.031	-0.029	0.027	-0.085	DYNC1LI1	0.075	~amyloid_beta+(1|manifest)+cngeneson+pc_mito+brain_region+Sex	0.643	5024.000
ENSG00000067177	0.366	-0.029	0.343	-0.401	PHKA1	0.417	~amyloid_beta+(1|manifest)+cngeneson+pc_mito+brain_region+Sex	6.598	515.000
ENSG00000176204	0.978	-0.029	0.287	-0.345	LRRTM4	0.980	~amyloid_beta+(1|manifest)+cngeneson+pc_mito+brain_region+Sex	3.046	1515.000
ENSG00000159658	0.100	-0.029	0.072	-0.130	EFCAB14	0.155	~amyloid_beta+(1|manifest)+cngeneson+pc_mito+brain_region+Sex	0.440	5483.000
ENSG00000138459	0.016	-0.029	0.023	-0.081	SLC35A5	0.053	~amyloid_beta+(1|manifest)+cngeneson+pc_mito+brain_region+Sex	0.500	5358.000
ENSG00000162852	0.006	-0.029	0.030	-0.089	CNST	0.030	~amyloid_beta+(1|manifest)+cngeneson+pc_mito+brain_region+Sex	0.595	5144.000
ENSG00000134698	0.107	-0.029	0.098	-0.157	AGO4	0.163	~amyloid_beta+(1|manifest)+cngeneson+pc_mito+brain_region+Sex	0.620	5083.000
ENSG00000075131	0.069	-0.029	0.059	-0.118	TIPIN	0.122	~amyloid_beta+(1|manifest)+cngeneson+pc_mito+brain_region+Sex	0.269	5782.000
ENSG00000110429	0.029	-0.029	0.042	-0.101	FBXO3	0.074	~amyloid_beta+(1|manifest)+cngeneson+pc_mito+brain_region+Sex	0.537	5265.000
ENSG00000144228	0.032	-0.030	0.054	-0.113	SPOPL	0.076	~amyloid_beta+(1|manifest)+cngeneson+pc_mito+brain_region+Sex	0.574	5177.000
ENSG00000100532	0.098	-0.030	0.042	-0.102	CGRRF1	0.153	~amyloid_beta+(1|manifest)+cngeneson+pc_mito+brain_region+Sex	0.818	4576.000
ENSG00000048405	0.072	-0.030	0.114	-0.173	ZNF800	0.126	~amyloid_beta+(1|manifest)+cngeneson+pc_mito+brain_region+Sex	1.761	2736.000
ENSG00000265808	0.079	-0.030	0.088	-0.148	SEC22B	0.134	~amyloid_beta+(1|manifest)+cngeneson+pc_mito+brain_region+Sex	0.802	4625.000
ENSG00000107742	0.378	-0.030	0.257	-0.317	SPOCK2	0.428	~amyloid_beta+(1|manifest)+cngeneson+pc_mito+brain_region+Sex	2.975	1555.000
ENSG00000102445	0.156	-0.030	0.060	-0.120	RUBCNL	0.211	~amyloid_beta+(1|manifest)+cngeneson+pc_mito+brain_region+Sex	0.488	5388.000
ENSG00000151893	0.002	-0.030	0.089	-0.148	CACUL1	0.017	~amyloid_beta+(1|manifest)+cngeneson+pc_mito+brain_region+Sex	1.151	3811.000
ENSG00000181350	0.195	-0.030	0.103	-0.163	LRRC75A	0.249	~amyloid_beta+(1|manifest)+cngeneson+pc_mito+brain_region+Sex	1.494	3149.000
ENSG00000198833	0.002	-0.030	0.001	-0.061	UBE2J1	0.015	~amyloid_beta+(1|manifest)+cngeneson+pc_mito+brain_region+Sex	0.361	5633.000
ENSG00000129315	0.025	-0.030	0.046	-0.107	CCNT1	0.067	~amyloid_beta+(1|manifest)+cngeneson+pc_mito+brain_region+Sex	0.712	4840.000
ENSG00000154781	0.086	-0.030	0.067	-0.128	CCDC174	0.141	~amyloid_beta+(1|manifest)+cngeneson+pc_mito+brain_region+Sex	1.272	3561.000
ENSG00000168924	0.032	-0.030	0.045	-0.106	LETM1	0.076	~amyloid_beta+(1|manifest)+cngeneson+pc_mito+brain_region+Sex	0.539	5262.000
ENSG00000177225	0.176	-0.030	0.125	-0.186	GATD1	0.231	~amyloid_beta+(1|manifest)+cngeneson+pc_mito+brain_region+Sex	1.800	2697.000
ENSG00000182218	0.164	-0.030	0.182	-0.243	HHIPL1	0.220	~amyloid_beta+(1|manifest)+cngeneson+pc_mito+brain_region+Sex	2.305	2099.000
ENSG00000056586	0.068	-0.030	0.089	-0.150	RC3H2	0.121	~amyloid_beta+(1|manifest)+cngeneson+pc_mito+brain_region+Sex	1.124	3876.000
ENSG00000196712	0.349	-0.030	0.027	-0.088	NF1	0.400	~amyloid_beta+(1|manifest)+cngeneson+pc_mito+brain_region+Sex	2.001	2429.000
ENSG00000134574	0.637	-0.031	0.189	-0.250	DDB2	0.671	~amyloid_beta+(1|manifest)+cngeneson+pc_mito+brain_region+Sex	1.600	2984.000
ENSG00000146021	0.104	-0.031	0.142	-0.203	KLHL3	0.159	~amyloid_beta+(1|manifest)+cngeneson+pc_mito+brain_region+Sex	1.423	3282.000
ENSG00000134283	0.349	-0.031	0.108	-0.170	PPHLN1	0.399	~amyloid_beta+(1|manifest)+cngeneson+pc_mito+brain_region+Sex	0.921	4327.000
ENSG00000085365	0.306	-0.031	0.091	-0.153	SCAMP1	0.359	~amyloid_beta+(1|manifest)+cngeneson+pc_mito+brain_region+Sex	1.163	3779.000
ENSG00000081154	0.019	-0.031	0.051	-0.112	PCNP	0.058	~amyloid_beta+(1|manifest)+cngeneson+pc_mito+brain_region+Sex	0.349	5655.000
ENSG00000065534	0.158	-0.031	0.272	-0.334	MYLK	0.213	~amyloid_beta+(1|manifest)+cngeneson+pc_mito+brain_region+Sex	2.235	2168.000
ENSG00000132680	0.121	-0.031	0.074	-0.136	KHDC4	0.178	~amyloid_beta+(1|manifest)+cngeneson+pc_mito+brain_region+Sex	1.384	3350.000
ENSG00000156374	0.057	-0.031	0.051	-0.113	PCGF6	0.109	~amyloid_beta+(1|manifest)+cngeneson+pc_mito+brain_region+Sex	0.574	5173.000
ENSG00000100528	0.005	-0.031	0.024	-0.087	CNIH1	0.026	~amyloid_beta+(1|manifest)+cngeneson+pc_mito+brain_region+Sex	0.413	5540.000
ENSG00000066422	0.006	-0.031	0.006	-0.069	ZBTB11	0.030	~amyloid_beta+(1|manifest)+cngeneson+pc_mito+brain_region+Sex	0.445	5464.000
ENSG00000067369	0.010	-0.031	0.086	-0.148	TP53BP1	0.040	~amyloid_beta+(1|manifest)+cngeneson+pc_mito+brain_region+Sex	1.360	3391.000
ENSG00000141258	0.162	-0.032	0.242	-0.305	SGSM2	0.217	~amyloid_beta+(1|manifest)+cngeneson+pc_mito+brain_region+Sex	3.311	1363.000
ENSG00000105127	0.083	-0.032	0.080	-0.144	AKAP8	0.138	~amyloid_beta+(1|manifest)+cngeneson+pc_mito+brain_region+Sex	1.532	3083.000
ENSG00000158528	0.926	-0.032	0.166	-0.230	PPP1R9A	0.934	~amyloid_beta+(1|manifest)+cngeneson+pc_mito+brain_region+Sex	1.325	3453.000
ENSG00000081177	0.022	-0.032	0.007	-0.070	EXD2	0.063	~amyloid_beta+(1|manifest)+cngeneson+pc_mito+brain_region+Sex	0.284	5755.000
ENSG00000063978	0.051	-0.032	0.068	-0.132	RNF4	0.101	~amyloid_beta+(1|manifest)+cngeneson+pc_mito+brain_region+Sex	0.572	5183.000
ENSG00000087087	0.153	-0.032	0.160	-0.223	SRRT	0.208	~amyloid_beta+(1|manifest)+cngeneson+pc_mito+brain_region+Sex	2.169	2236.000
ENSG00000124275	0.081	-0.032	0.023	-0.087	MTRR	0.136	~amyloid_beta+(1|manifest)+cngeneson+pc_mito+brain_region+Sex	0.407	5552.000
ENSG00000198521	0.169	-0.032	0.140	-0.204	ZNF43	0.225	~amyloid_beta+(1|manifest)+cngeneson+pc_mito+brain_region+Sex	2.505	1897.000
ENSG00000203546	0.020	-0.032	0.054	-0.119	[illegible]	0.060	~amyloid_beta+(1|manifest)+cngeneson+pc_mito+brain_region+Sex	0.637	5040.000
ENSG00000136379	0.144	-0.032	0.082	-0.146	ABHD17C	0.200	~amyloid_beta+(1|manifest)+cngeneson+pc_mito+brain_region+Sex	1.194	3718.000
ENSG00000164089	0.969	-0.032	0.386	-0.450	ETNPPL	0.972	~amyloid_beta+(1|manifest)+cngeneson+pc_mito+brain_region+Sex	18.979	57.000
ENSG00000113360	0.157	-0.032	0.094	-0.159	DROSHA	0.212	~amyloid_beta+(1|manifest)+cngeneson+pc_mito+brain_region+Sex	1.285	3534.000
ENSG00000197013	0.023	-0.032	0.048	-0.113	ZNF429	0.065	~amyloid_beta+(1|manifest)+cngeneson+pc_mito+brain_region+Sex	0.398	5565.000
ENSG00000143147	0.229	-0.032	0.123	-0.188	GPR161	0.283	~amyloid_beta+(1|manifest)+cngeneson+pc_mito+brain_region+Sex	0.660	4978.000
ENSG00000204104	0.029	-0.032	0.085	-0.150	TRAF3IP1	0.073	~amyloid_beta+(1|manifest)+cngeneson+pc_mito+brain_region+Sex	0.369	5621.000
ENSG00000078142	0.318	-0.032	0.146	-0.211	PIK3C3	0.370	~amyloid_beta+(1|manifest)+cngeneson+pc_mito+brain_region+Sex	1.254	3589.000
ENSG00000035862	0.116	-0.032	0.173	-0.238	TIMP2	0.172	~amyloid_beta+(1|manifest)+cngeneson+pc_mito+brain_region+Sex	0.628	5064.000
ENSG00000197496	0.059	-0.033	0.094	-0.159	SLC2A10	0.111	~amyloid_beta+(1|manifest)+cngeneson+pc_mito+brain_region+Sex	0.545	5240.000
ENSG00000135776	0.091	-0.033	0.057	-0.122	ABCB10	0.147	~amyloid_beta+(1|manifest)+cngeneson+pc_mito+brain_region+Sex	0.884	4415.000
ENSG00000197863	0.022	-0.033	0.119	-0.185	ZNF790	0.063	~amyloid_beta+(1|manifest)+cngeneson+pc_mito+brain_region+Sex	2.665	1758.000
ENSG00000144061	0.042	-0.033	0.078	-0.143	NPHP1	0.090	~amyloid_beta+(1|manifest)+cngeneson+pc_mito+brain_region+Sex	0.732	4789.000
ENSG00000172890	0.062	-0.033	0.060	-0.126	NADSYN1	0.114	~amyloid_beta+(1|manifest)+cngeneson+pc_mito+brain_region+Sex	1.043	4040.000
ENSG00000263956	0.323	-0.033	0.171	-0.237	NBPF11	0.375	~amyloid_beta+(1|manifest)+cngeneson+pc_mito+brain_region+Sex	2.004	2423.000
ENSG00000116731	0.153	-0.033	0.044	-0.111	PRDM2	0.208	~amyloid_beta+(1|manifest)+cngeneson+pc_mito+brain_region+Sex	0.350	5652.000
ENSG00000196705	0.139	-0.033	0.147	-0.213	ZNF431	0.195	~amyloid_beta+(1|manifest)+cngeneson+pc_mito+brain_region+Sex	2.168	2239.000
ENSG00000133026	0.108	-0.033	0.085	-0.151	MYH10	0.164	~amyloid_beta+(1|manifest)+cngeneson+pc_mito+brain_region+Sex	1.041	4046.000
ENSG00000276644	0.412	-0.033	0.198	-0.265	DACH1	0.459	~amyloid_beta+(1|manifest)+cngeneson+pc_mito+brain_region+Sex	1.506	3129.000
ENSG00000159459	0.055	-0.034	0.085	-0.152	UBR1	0.105	~amyloid_beta+(1|manifest)+cngeneson+pc_mito+brain_region+Sex	1.713	2805.000
ENSG00000163638	0.580	-0.034	0.256	-0.324	ADAMTS9	0.618	~amyloid_beta+(1|manifest)+cngeneson+pc_mito+brain_region+Sex	1.466	3193.000
ENSG00000122085	0.227	-0.034	0.162	-0.229	MTERF4	0.282	~amyloid_beta+(1|manifest)+cngeneson+pc_mito+brain_region+Sex	2.441	1941.000
ENSG00000125991	0.174	-0.034	0.088	-0.155	ERGIC3	0.229	~amyloid_beta+(1|manifest)+cngeneson+pc_mito+brain_region+Sex	0.806	4612.000
ENSG00000163249	0.075	-0.034	0.055	-0.123	CCNYL1	0.128	~amyloid_beta+(1|manifest)+cngeneson+pc_mito+brain_region+Sex	1.412	3303.000
ENSG00000198689	0.137	-0.034	0.124	-0.192	SLC9A6	0.194	~amyloid_beta+(1|manifest)+cngeneson+pc_mito+brain_region+Sex	1.592	2997.000
ENSG00000131269	0.365	-0.034	0.140	-0.208	ABCB7	0.416	~amyloid_beta+(1|manifest)+cngeneson+pc_mito+brain_region+Sex	1.168	3774.000
ENSG00000104129	0.152	-0.034	0.059	-0.127	DNAJC17	0.207	~amyloid_beta+(1|manifest)+cngeneson+pc_mito+brain_region+Sex	1.043	4042.000
ENSG00000196141	0.758	-0.034	0.191	-0.259	SPATS2L	0.781	~amyloid_beta+(1|manifest)+cngeneson+pc_mito+brain_region+Sex	0.901	4373.000
ENSG00000161057	0.058	-0.034	0.051	-0.119	PSMC2	0.109	~amyloid_beta+(1|manifest)+cngeneson+pc_mito+brain_region+Sex	0.600	5129.000
ENSG00000081307	0.077	-0.034	0.136	-0.204	UBA5	0.131	~amyloid_beta+(1|manifest)+cngeneson+pc_mito+brain_region+Sex	0.503	5349.000
ENSG00000181704	0.013	-0.034	0.007	-0.075	YIPF6	0.046	~amyloid_beta+(1|manifest)+cngeneson+pc_mito+brain_region+Sex	0.178	5901.000
ENSG00000152977	0.811	-0.034	0.491	-0.559	ZIC1	0.830	~amyloid_beta+(1|manifest)+cngeneson+pc_mito+brain_region+Sex	14.488	113.000
ENSG00000205189	0.085	-0.034	0.049	-0.117	ZBTB10	0.140	~amyloid_beta+(1|manifest)+cngeneson+pc_mito+brain_region+Sex	0.716	4832.000
ENSG00000161791	0.221	-0.034	0.155	-0.224	FMNL3	0.276	~amyloid_beta+(1|manifest)+cngeneson+pc_mito+brain_region+Sex	2.014	2415.000
ENSG00000090565	0.375	-0.034	0.226	-0.295	RAB11FIP3	0.425	~amyloid_beta+(1|manifest)+cngeneson+pc_mito+brain_region+Sex	3.790	1129.000
ENSG00000065559	0.374	-0.034	0.150	-0.219	MAP2K4	0.423	~amyloid_beta+(1|manifest)+cngeneson+pc_mito+brain_region+Sex	1.480	3176.000
ENSG00000120800	0.009	-0.034	0.030	-0.098	UTP20	0.037	~amyloid_beta+(1|manifest)+cngeneson+pc_mito+brain_region+Sex	0.800	4634.000
ENSG00000119844	0.060	-0.034	0.081	-0.150	AFTPH	0.111	~amyloid_beta+(1|manifest)+cngeneson+pc_mito+brain_region+Sex	1.453	3222.000
ENSG00000158604	0.071	-0.034	0.057	-0.126	TMED4	0.125	~amyloid_beta+(1|manifest)+cngeneson+pc_mito+brain_region+Sex	0.594	5145.000
ENSG00000129219	0.205	-0.034	0.119	-0.188	PLD2	0.260	~amyloid_beta+(1|manifest)+cngeneson+pc_mito+brain_region+Sex	2.092	2323.000
ENSG00000146966	0.290	-0.035	0.211	-0.280	DENND2A	0.343	~amyloid_beta+(1|manifest)+cngeneson+pc_mito+brain_region+Sex	2.239	2164.000
ENSG00000183783	0.981	-0.035	0.367	-0.436	KCTD8	0.982	~amyloid_beta+(1|manifest)+cngeneson+pc_mito+brain_region+Sex	4.241	968.000
ENSG00000134987	0.014	-0.035	0.013	-0.082	WDR36	0.049	~amyloid_beta+(1|manifest)+cngeneson+pc_mito+brain_region+Sex	0.728	4799.000
ENSG00000009335	0.078	-0.035	0.137	-0.207	UBE3C	0.133	~amyloid_beta+(1|manifest)+cngeneson+pc_mito+brain_region+Sex	1.408	3309.000
ENSG00000066557	0.006	-0.035	0.061	-0.131	LRRC40	0.028	~amyloid_beta+(1|manifest)+cngeneson+pc_mito+brain_region+Sex	0.545	5244.000
ENSG00000164024	0.034	-0.035	0.009	-0.079	METAP1	0.080	~amyloid_beta+(1|manifest)+cngeneson+pc_mito+brain_region+Sex	0.537	5263.000
ENSG00000132424	0.367	-0.035	0.120	-0.190	PNISR	0.417	~amyloid_beta+(1|manifest)+cngeneson+pc_mito+brain_region+Sex	11.013	220.000
ENSG00000162928	0.044	-0.035	0.033	-0.103	PEX13	0.093	~amyloid_beta+(1|manifest)+cngeneson+pc_mito+brain_region+Sex	0.690	4919.000
ENSG00000157423	0.031	-0.035	0.219	-0.289	HYDIN	0.076	~amyloid_beta+(1|manifest)+cngeneson+pc_mito+brain_region+Sex	1.549	3051.000
ENSG00000168724	0.044	-0.035	0.034	-0.105	DNAJC21	0.093	~amyloid_beta+(1|manifest)+cngeneson+pc_mito+brain_region+Sex	0.599	5132.000
ENSG00000136381	0.006	-0.036	0.086	-0.157	IREB2	0.030	~amyloid_beta+(1|manifest)+cngeneson+pc_mito+brain_region+Sex	1.807	2684.000
ENSG00000151657	0.021	-0.036	0.012	-0.083	KIN	0.061	~amyloid_beta+(1|manifest)+cngeneson+pc_mito+brain_region+Sex	0.700	4885.000
ENSG00000198420	0.204	-0.036	0.101	-0.172	TCAF1	0.259	~amyloid_beta+(1|manifest)+cngeneson+pc_mito+brain_region+Sex	1.286	3533.000
ENSG00000111727	0.004	-0.036	0.000	-0.072	HCFC2	0.022	~amyloid_beta+(1|manifest)+cngeneson+pc_mito+brain_region+Sex	0.562	5203.000
ENSG00000172943	0.043	-0.036	0.045	-0.117	PHF8	0.091	~amyloid_beta+(1|manifest)+cngeneson+pc_mito+brain_region+Sex	0.564	5199.000
ENSG00000084710	0.784	-0.036	0.257	-0.329	EFR3B	0.806	~amyloid_beta+(1|manifest)+cngeneson+pc_mito+brain_region+Sex	3.589	1212.000
ENSG00000065615	0.119	-0.036	0.055	-0.126	CYB5R4	0.175	~amyloid_beta+(1|manifest)+cngeneson+pc_mito+brain_region+Sex	0.754	4733.000
ENSG00000214517	0.075	-0.036	0.114	-0.186	PPME1	0.129	~amyloid_beta+(1|manifest)+cngeneson+pc_mito+brain_region+Sex	1.941	2505.000
ENSG00000168827	0.021	-0.036	0.028	-0.100	GFM1	0.062	~amyloid_beta+(1|manifest)+cngeneson+pc_mito+brain_region+Sex	0.792	4648.000
ENSG00000106603	0.284	-0.036	0.085	-0.157	COA1	0.337	~amyloid_beta+(1|manifest)+cngeneson+pc_mito+brain_region+Sex	1.606	2976.000
ENSG00000180626	0.054	-0.036	0.040	-0.112	ZNF594	0.105	~amyloid_beta+(1|manifest)+cngeneson+pc_mito+brain_region+Sex	0.384	5589.000
ENSG00000107581	0.029	-0.036	0.073	-0.146	EIF3A	0.073	~amyloid_beta+(1|manifest)+cngeneson+pc_mito+brain_region+Sex	1.584	3008.000
ENSG00000108641	0.030	-0.036	0.055	-0.128	B9D1	0.074	~amyloid_beta+(1|manifest)+cngeneson+pc_mito+brain_region+Sex	0.755	4728.000
ENSG00000100934	0.123	-0.036	0.077	-0.150	SEC23A	0.180	~amyloid_beta+(1|manifest)+cngeneson+pc_mito+brain_region+Sex	1.372	3373.000
ENSG00000160094	0.008	-0.036	0.062	-0.134	ZNF362	0.036	~amyloid_beta+(1|manifest)+cngeneson+pc_mito+brain_region+Sex	0.821	4567.000
ENSG00000138050	0.010	-0.037	0.059	-0.133	THUMPD2	0.041	~amyloid_beta+(1|manifest)+cngeneson+pc_mito+brain_region+Sex	1.231	3635.000
ENSG00000144840	0.018	-0.037	0.019	-0.092	RABL3	0.056	~amyloid_beta+(1|manifest)+cngeneson+pc_mito+brain_region+Sex	0.645	5017.000
ENSG00000101868	0.502	-0.037	0.164	-0.237	POLA1	0.546	~amyloid_beta+(1|manifest)+cngeneson+pc_mito+brain_region+Sex	2.106	2305.000
ENSG00000100320	0.671	-0.037	0.133	-0.206	RBFOX2	0.702	~amyloid_beta+(1|manifest)+cngeneson+pc_mito+brain_region+Sex	5.650	655.000
ENSG00000197312	0.058	-0.037	0.030	-0.103	DDI2	0.109	~amyloid_beta+(1|manifest)+cngeneson+pc_mito+brain_region+Sex	0.702	4880.000
ENSG00000151376	0.544	-0.037	0.187	-0.260	ME3	0.585	~amyloid_beta+(1|manifest)+cngeneson+pc_mito+brain_region+Sex	5.248	716.000
ENSG00000162769	0.062	-0.037	0.058	-0.131	FLVCR1	0.115	~amyloid_beta+(1|manifest)+cngeneson+pc_mito+brain_region+Sex	1.246	3610.000
ENSG00000136051	0.038	-0.037	0.099	-0.173	WASHC4	0.085	~amyloid_beta+(1|manifest)+cngeneson+pc_mito+brain_region+Sex	0.622	5077.000
ENSG00000024048	0.247	-0.037	0.083	-0.157	UBR2	0.301	~amyloid_beta+(1|manifest)+cngeneson+pc_mito+brain_region+Sex	1.431	3263.000
ENSG00000145041	0.009	-0.037	0.036	-0.110	DCAF1	0.037	~amyloid_beta+(1|manifest)+cngeneson+pc_mito+brain_region+Sex	0.928	4310.000
ENSG00000027001	0.017	-0.037	0.040	-0.113	MIPEP	0.054	~amyloid_beta+(1|manifest)+cngeneson+pc_mito+brain_region+Sex	0.651	4995.000
ENSG00000174498	0.193	-0.037	0.210	-0.284	IGDCC3	0.247	~amyloid_beta+(1|manifest)+cngeneson+pc_mito+brain_region+Sex	1.745	2756.000
ENSG00000145332	0.045	-0.037	0.081	-0.155	KLHL8	0.093	~amyloid_beta+(1|manifest)+cngeneson+pc_mito+brain_region+Sex	0.669	4963.000
ENSG00000221914	0.070	-0.037	0.145	-0.219	PPP2R2A	0.124	~amyloid_beta+(1|manifest)+cngeneson+pc_mito+brain_region+Sex	1.277	3552.000
ENSG00000170471	0.036	-0.037	0.056	-0.131	RALGAPB	0.082	~amyloid_beta+(1|manifest)+cngeneson+pc_mito+brain_region+Sex	1.250	3600.000
ENSG00000156958	0.047	-0.037	0.047	-0.121	GALK2	0.096	~amyloid_beta+(1|manifest)+cngeneson+pc_mito+brain_region+Sex	1.198	3711.000
ENSG00000122033	0.017	-0.037	0.085	-0.160	MTIF3	0.054	~amyloid_beta+(1|manifest)+cngeneson+pc_mito+brain_region+Sex	1.323	3459.000
ENSG00000112062	0.019	-0.037	0.110	-0.185	MAPK14	0.057	~amyloid_beta+(1|manifest)+cngeneson+pc_mito+brain_region+Sex	2.092	2324.000
ENSG00000151023	0.278	-0.038	0.091	-0.166	ENKUR	0.331	~amyloid_beta+(1|manifest)+cngeneson+pc_mito+brain_region+Sex	0.593	5146.000
ENSG00000100030	0.349	-0.038	0.083	-0.158	MAPK1	0.400	~amyloid_beta+(1|manifest)+cngeneson+pc_mito+brain_region+Sex	1.248	3605.000
ENSG00000196547	0.147	-0.038	0.093	-0.168	MAN2A2	0.203	~amyloid_beta+(1|manifest)+cngeneson+pc_mito+brain_region+Sex	1.489	3159.000
ENSG00000198198	0.063	-0.038	0.055	-0.131	SZT2	0.115	~amyloid_beta+(1|manifest)+cngeneson+pc_mito+brain_region+Sex	0.748	4746.000
ENSG00000198478	0.089	-0.038	0.115	-0.190	SH3BGRL2	0.144	~amyloid_beta+(1|manifest)+cngeneson+pc_mito+brain_region+Sex	0.682	4933.000
ENSG00000033170	0.948	-0.038	0.200	-0.275	FUT8	0.954	~amyloid_beta+(1|manifest)+cngeneson+pc_mito+brain_region+Sex	5.531	676.000
ENSG00000186318	0.122	-0.038	0.058	-0.133	BACE1	0.179	~amyloid_beta+(1|manifest)+cngeneson+pc_mito+brain_region+Sex	1.205	3691.000
ENSG00000139324	0.065	-0.038	0.025	-0.101	TMTC3	0.118	~amyloid_beta+(1|manifest)+cngeneson+pc_mito+brain_region+Sex	0.469	5423.000
ENSG00000172728	0.598	-0.038	0.284	-0.359	FUT10	0.634	~amyloid_beta+(1|manifest)+cngeneson+pc_mito+brain_region+Sex	2.555	1851.000
ENSG00000187239	0.407	-0.038	0.054	-0.130	FNBP1	0.454	~amyloid_beta+(1|manifest)+cngeneson+pc_mito+brain_region+Sex	10.223	261.000
ENSG00000123106	0.888	-0.038	0.224	-0.301	CCDC91	0.901	~amyloid_beta+(1|manifest)+cngeneson+pc_mito+brain_region+Sex	1.390	3337.000
ENSG00000172638	0.151	-0.038	0.206	-0.282	EFEMP2	0.207	~amyloid_beta+(1|manifest)+cngeneson+pc_mito+brain_region+Sex	4.105	1012.000
ENSG00000115392	0.037	-0.038	0.167	-0.244	FANCL	0.084	~amyloid_beta+(1|manifest)+cngeneson+pc_mito+brain_region+Sex	2.214	2187.000
ENSG00000162927	0.024	-0.038	0.066	-0.143	PUS10	0.066	~amyloid_beta+(1|manifest)+cngeneson+pc_mito+brain_region+Sex	0.148	5926.000
ENSG00000111728	0.941	-0.038	0.524	-0.601	ST8SIA1	0.947	~amyloid_beta+(1|manifest)+cngeneson+pc_mito+brain_region+Sex	1.602	2981.000
ENSG00000145388	0.004	-0.039	0.005	-0.082	METTL14	0.025	~amyloid_beta+(1|manifest)+cngeneson+pc_mito+brain_region+Sex	0.275	5772.000

ENSG00000134873	0.438	-0.048	0.289	-0.385	CLDN10	0.483	~amyloid_beta+(1|manifest)+cngeneson+pc_mito+brain_region+Sex	1.032	4066.000
ENSG00000164627	0.406	-0.048	0.138	-0.234	KIF6	0.453	~amyloid_beta+(1|manifest)+cngeneson+pc_mito+brain_region+Sex	1.264	3572.000
ENSG00000127124	0.767	-0.048	0.266	-0.363	HIVEP3	0.790	~amyloid_beta+(1|manifest)+cngeneson+pc_mito+brain_region+Sex	5.535	673.000
ENSG00000117543	0.021	-0.049	-0.002	-0.095	DPH5	0.060	~amyloid_beta+(1|manifest)+cngeneson+pc_mito+brain_region+Sex	0.329	5676.000
ENSG00000144426	0.127	-0.049	0.099	-0.196	NBEAL1	0.183	~amyloid_beta+(1|manifest)+cngeneson+pc_mito+brain_region+Sex	2.371	2027.000
ENSG00000169057	0.239	-0.049	0.102	-0.199	MECP2	0.292	~amyloid_beta+(1|manifest)+cngeneson+pc_mito+brain_region+Sex	1.052	4019.000
ENSG00000143643	0.001	-0.049	0.008	-0.106	TTC13	0.010	~amyloid_beta+(1|manifest)+cngeneson+pc_mito+brain_region+Sex	0.772	4682.000
ENSG00000107362	0.055	-0.049	0.011	-0.109	ABHD17B	0.105	~amyloid_beta+(1|manifest)+cngeneson+pc_mito+brain_region+Sex	0.411	5543.000
ENSG00000107938	0.148	-0.049	0.036	-0.134	EDRF1	0.203	~amyloid_beta+(1|manifest)+cngeneson+pc_mito+brain_region+Sex	0.945	4266.000
ENSG00000165629	0.009	-0.049	0.116	-0.214	ATP5F1C	0.038	~amyloid_beta+(1|manifest)+cngeneson+pc_mito+brain_region+Sex	1.320	3464.000
ENSG00000145390	0.833	-0.049	0.194	-0.292	USP53	0.850	~amyloid_beta+(1|manifest)+cngeneson+pc_mito+brain_region+Sex	4.351	933.000
ENSG00000133142	0.080	-0.049	0.039	-0.137	TCEAL4	0.135	~amyloid_beta+(1|manifest)+cngeneson+pc_mito+brain_region+Sex	0.730	4793.000
ENSG00000106617	0.077	-0.049	0.178	-0.276	PRKAG2	0.132	~amyloid_beta+(1|manifest)+cngeneson+pc_mito+brain_region+Sex	1.672	2867.000
ENSG00000127419	0.216	-0.049	0.140	-0.238	TMEM175	0.271	~amyloid_beta+(1|manifest)+cngeneson+pc_mito+brain_region+Sex	1.926	2529.000
ENSG00000163001	0.062	-0.049	0.101	-0.200	CFAP36	0.114	~amyloid_beta+(1|manifest)+cngeneson+pc_mito+brain_region+Sex	2.748	1707.000
ENSG00000151116	0.014	-0.049	0.006	-0.105	UEVLD	0.049	~amyloid_beta+(1|manifest)+cngeneson+pc_mito+brain_region+Sex	0.745	4758.000
ENSG00000078725	0.900	-0.049	0.174	-0.272	BRINP1	0.911	~amyloid_beta+(1|manifest)+cngeneson+pc_mito+brain_region+Sex	3.274	1385.000
ENSG00000185917	0.011	-0.049	0.019	-0.118	SETD4	0.042	~amyloid_beta+(1|manifest)+cngeneson+pc_mito+brain_region+Sex	1.026	4087.000
ENSG00000007341	0.092	-0.049	0.121	-0.220	ST7L	0.148	~amyloid_beta+(1|manifest)+cngeneson+pc_mito+brain_region+Sex	0.705	4867.000
ENSG00000265354	0.010	-0.049	0.005	-0.104	TIMM23	0.039	~amyloid_beta+(1|manifest)+cngeneson+pc_mito+brain_region+Sex	0.469	5421.000
ENSG00000116991	0.826	-0.049	0.128	-0.226	SIPA1L2	0.844	~amyloid_beta+(1|manifest)+cngeneson+pc_mito+brain_region+Sex	5.308	709.000
ENSG00000116138	0.004	-0.050	0.007	-0.106	DNAJC16	0.025	~amyloid_beta+(1|manifest)+cngeneson+pc_mito+brain_region+Sex	0.635	5046.000
ENSG00000143624	0.172	-0.050	0.102	-0.201	INTS3	0.227	~amyloid_beta+(1|manifest)+cngeneson+pc_mito+brain_region+Sex	2.759	1697.000
ENSG00000124151	0.059	-0.050	0.068	-0.167	NCOA3	0.110	~amyloid_beta+(1|manifest)+cngeneson+pc_mito+brain_region+Sex	1.121	3885.000
ENSG00000221963	0.376	-0.050	0.204	-0.303	APOL6	0.426	~amyloid_beta+(1|manifest)+cngeneson+pc_mito+brain_region+Sex	4.009	1038.000
ENSG00000163527	0.082	-0.050	0.172	-0.272	STT3B	0.137	~amyloid_beta+(1|manifest)+cngeneson+pc_mito+brain_region+Sex	2.538	1870.000
ENSG00000144580	0.009	-0.050	0.009	-0.109	CNOT9	0.038	~amyloid_beta+(1|manifest)+cngeneson+pc_mito+brain_region+Sex	0.553	5226.000
ENSG00000112130	0.115	-0.050	0.057	-0.157	RNF8	0.171	~amyloid_beta+(1|manifest)+cngeneson+pc_mito+brain_region+Sex	1.291	3524.000
ENSG00000185634	0.244	-0.050	0.058	-0.158	SHC4	0.298	~amyloid_beta+(1|manifest)+cngeneson+pc_mito+brain_region+Sex	0.975	4195.000
ENSG00000054118	0.078	-0.050	0.063	-0.163	THRAP3	0.133	~amyloid_beta+(1|manifest)+cngeneson+pc_mito+brain_region+Sex	1.709	2812.000
ENSG00000119285	0.039	-0.050	0.022	-0.123	HEATR1	0.086	~amyloid_beta+(1|manifest)+cngeneson+pc_mito+brain_region+Sex	0.903	4372.000
ENSG00000156831	0.266	-0.050	0.065	-0.165	NSMCE2	0.319	~amyloid_beta+(1|manifest)+cngeneson+pc_mito+brain_region+Sex	0.396	5568.000
ENSG00000090857	0.257	-0.050	0.228	-0.329	PDPR	0.311	~amyloid_beta+(1|manifest)+cngeneson+pc_mito+brain_region+Sex	4.247	965.000
ENSG00000164118	0.138	-0.050	0.051	-0.152	CEP44	0.195	~amyloid_beta+(1|manifest)+cngeneson+pc_mito+brain_region+Sex	1.002	4132.000
ENSG00000174306	0.715	-0.051	0.093	-0.195	ZHX3	0.743	~amyloid_beta+(1|manifest)+cngeneson+pc_mito+brain_region+Sex	6.036	588.000
ENSG00000157036	0.043	-0.051	0.045	-0.148	EXOG	0.091	~amyloid_beta+(1|manifest)+cngeneson+pc_mito+brain_region+Sex	1.395	3332.000
ENSG00000088179	0.445	-0.051	0.185	-0.287	PTPN4	0.490	~amyloid_beta+(1|manifest)+cngeneson+pc_mito+brain_region+Sex	4.022	1033.000
ENSG00000122882	0.006	-0.051	-0.003	-0.099	ECD	0.029	~amyloid_beta+(1|manifest)+cngeneson+pc_mito+brain_region+Sex	0.420	5525.000
ENSG00000071189	0.132	-0.051	0.204	-0.306	SNX13	0.188	~amyloid_beta+(1|manifest)+cngeneson+pc_mito+brain_region+Sex	2.175	2230.000
ENSG00000100884	0.259	-0.051	0.247	-0.350	CPNE6	0.313	~amyloid_beta+(1|manifest)+cngeneson+pc_mito+brain_region+Sex	2.044	2378.000
ENSG00000039560	0.122	-0.051	0.065	-0.168	RAI14	0.179	~amyloid_beta+(1|manifest)+cngeneson+pc_mito+brain_region+Sex	1.666	2880.000
ENSG00000131725	0.101	-0.051	0.077	-0.179	WDR44	0.156	~amyloid_beta+(1|manifest)+cngeneson+pc_mito+brain_region+Sex	2.022	2407.000
ENSG00000103540	0.005	-0.051	0.033	-0.136	CCP110	0.027	~amyloid_beta+(1|manifest)+cngeneson+pc_mito+brain_region+Sex	0.661	4977.000
ENSG00000114098	0.121	-0.051	0.084	-0.187	ARMC8	0.177	~amyloid_beta+(1|manifest)+cngeneson+pc_mito+brain_region+Sex	1.725	2789.000
ENSG00000142065	0.023	-0.051	0.062	-0.165	ZFP14	0.063	~amyloid_beta+(1|manifest)+cngeneson+pc_mito+brain_region+Sex	1.160	3790.000
ENSG00000077254	0.073	-0.051	0.038	-0.141	USP33	0.127	~amyloid_beta+(1|manifest)+cngeneson+pc_mito+brain_region+Sex	1.672	2868.000
ENSG00000095002	0.118	-0.051	0.053	-0.156	MSH2	0.175	~amyloid_beta+(1|manifest)+cngeneson+pc_mito+brain_region+Sex	0.708	4854.000
ENSG00000095485	0.022	-0.052	0.021	-0.124	CWF19L1	0.062	~amyloid_beta+(1|manifest)+cngeneson+pc_mito+brain_region+Sex	0.724	4812.000
ENSG00000158092	0.107	-0.052	0.040	-0.143	NCK1	0.163	~amyloid_beta+(1|manifest)+cngeneson+pc_mito+brain_region+Sex	1.218	3665.000
ENSG00000259417	0.867	-0.052	0.471	-0.575	CTXND1	0.881	~amyloid_beta+(1|manifest)+cngeneson+pc_mito+brain_region+Sex	9.560	292.000
ENSG00000170275	0.173	-0.052	0.084	-0.187	CRTAP	0.228	~amyloid_beta+(1|manifest)+cngeneson+pc_mito+brain_region+Sex	1.069	3992.000
ENSG00000086666	0.528	-0.052	0.097	-0.201	ZFAND6	0.570	~amyloid_beta+(1|manifest)+cngeneson+pc_mito+brain_region+Sex	1.115	3899.000
ENSG00000185386	0.175	-0.052	0.178	-0.282	MAPK11	0.230	~amyloid_beta+(1|manifest)+cngeneson+pc_mito+brain_region+Sex	0.703	4873.000
ENSG00000149187	0.381	-0.052	0.107	-0.211	CELF1	0.430	~amyloid_beta+(1|manifest)+cngeneson+pc_mito+brain_region+Sex	0.929	4303.000
ENSG00000131368	0.099	-0.052	0.166	-0.270	MRPS25	0.154	~amyloid_beta+(1|manifest)+cngeneson+pc_mito+brain_region+Sex	2.338	2063.000
ENSG00000164187	0.221	-0.052	0.049	-0.153	LMBRD2	0.276	~amyloid_beta+(1|manifest)+cngeneson+pc_mito+brain_region+Sex	1.144	3825.000
ENSG00000105568	0.030	-0.052	0.028	-0.132	PPP2R1A	0.075	~amyloid_beta+(1|manifest)+cngeneson+pc_mito+brain_region+Sex	0.424	5519.000
ENSG00000178950	0.105	-0.052	0.163	-0.268	GAK	0.161	~amyloid_beta+(1|manifest)+cngeneson+pc_mito+brain_region+Sex	2.274	2129.000
ENSG00000173482	0.781	-0.052	0.133	-0.237	PTPRM	0.802	~amyloid_beta+(1|manifest)+cngeneson+pc_mito+brain_region+Sex	1.635	2929.000
ENSG00000113658	0.084	-0.052	0.059	-0.164	SMAD5	0.140	~amyloid_beta+(1|manifest)+cngeneson+pc_mito+brain_region+Sex	0.724	4810.000
ENSG00000132906	0.067	-0.052	0.056	-0.161	CASP9	0.120	~amyloid_beta+(1|manifest)+cngeneson+pc_mito+brain_region+Sex	1.635	2931.000
ENSG00000156502	0.001	-0.052	-0.012	-0.092	SUPV3L1	0.013	~amyloid_beta+(1|manifest)+cngeneson+pc_mito+brain_region+Sex	0.491	5384.000
ENSG00000160961	0.059	-0.052	0.026	-0.131	ZNF333	0.111	~amyloid_beta+(1|manifest)+cngeneson+pc_mito+brain_region+Sex	1.103	3918.000
ENSG00000141646	0.045	-0.052	0.020	-0.125	SMAD4	0.093	~amyloid_beta+(1|manifest)+cngeneson+pc_mito+brain_region+Sex	0.445	5465.000
ENSG00000184402	0.017	-0.052	0.056	-0.160	SS18L1	0.054	~amyloid_beta+(1|manifest)+cngeneson+pc_mito+brain_region+Sex	1.082	3966.000
ENSG00000171612	0.743	-0.052	0.262	-0.367	SLC25A33	0.768	~amyloid_beta+(1|manifest)+cngeneson+pc_mito+brain_region+Sex	3.118	1472.000
ENSG00000126775	0.052	-0.052	0.055	-0.160	ATG14	0.102	~amyloid_beta+(1|manifest)+cngeneson+pc_mito+brain_region+Sex	0.819	4575.000
ENSG00000114480	0.440	-0.052	0.237	-0.342	GBE1	0.486	~amyloid_beta+(1|manifest)+cngeneson+pc_mito+brain_region+Sex	5.356	698.000
ENSG00000055483	0.371	-0.053	0.108	-0.213	USP36	0.421	~amyloid_beta+(1|manifest)+cngeneson+pc_mito+brain_region+Sex	2.131	2277.000
ENSG00000149308	0.010	-0.053	0.022	-0.128	NPAT	0.040	~amyloid_beta+(1|manifest)+cngeneson+pc_mito+brain_region+Sex	0.674	4951.000
ENSG00000165886	0.167	-0.053	0.101	-0.206	UBTD1	0.223	~amyloid_beta+(1|manifest)+cngeneson+pc_mito+brain_region+Sex	1.365	3384.000
ENSG00000173473	0.386	-0.053	0.174	-0.279	SMARCC1	0.435	~amyloid_beta+(1|manifest)+cngeneson+pc_mito+brain_region+Sex	2.979	1552.000
ENSG00000136147	0.088	-0.053	0.080	-0.186	PHF11	0.143	~amyloid_beta+(1|manifest)+cngeneson+pc_mito+brain_region+Sex	1.102	3924.000
ENSG00000115091	0.172	-0.053	0.118	-0.224	ACTR3	0.227	~amyloid_beta+(1|manifest)+cngeneson+pc_mito+brain_region+Sex	2.885	1613.000
ENSG00000123179	0.035	-0.053	0.057	-0.163	EBPL	0.081	~amyloid_beta+(1|manifest)+cngeneson+pc_mito+brain_region+Sex	1.179	3747.000
ENSG00000143337	0.016	-0.053	0.029	-0.134	TOR1AIP1	0.053	~amyloid_beta+(1|manifest)+cngeneson+pc_mito+brain_region+Sex	0.635	5045.000
ENSG00000157077	0.054	-0.053	0.054	-0.160	ZFYVE9	0.105	~amyloid_beta+(1|manifest)+cngeneson+pc_mito+brain_region+Sex	0.609	5109.000
ENSG00000054983	0.052	-0.053	0.058	-0.164	GALC	0.102	~amyloid_beta+(1|manifest)+cngeneson+pc_mito+brain_region+Sex	1.433	3259.000
ENSG00000132640	0.169	-0.053	0.083	-0.189	BTBD3	0.225	~amyloid_beta+(1|manifest)+cngeneson+pc_mito+brain_region+Sex	0.679	4943.000
ENSG00000131115	0.031	-0.053	0.023	-0.129	ZNF227	0.076	~amyloid_beta+(1|manifest)+cngeneson+pc_mito+brain_region+Sex	0.216	5852.000
ENSG00000114739	0.058	-0.053	0.052	-0.158	ACVR2B	0.109	~amyloid_beta+(1|manifest)+cngeneson+pc_mito+brain_region+Sex	0.621	5082.000
ENSG00000070961	0.360	-0.053	0.110	-0.217	ATP2B1	0.411	~amyloid_beta+(1|manifest)+cngeneson+pc_mito+brain_region+Sex	1.284	3535.000
ENSG00000056972	0.790	-0.053	0.189	-0.296	TRAF3IP2	0.810	~amyloid_beta+(1|manifest)+cngeneson+pc_mito+brain_region+Sex	4.962	785.000
ENSG00000182985	0.795	-0.053	0.104	-0.211	CADM1	0.815	~amyloid_beta+(1|manifest)+cngeneson+pc_mito+brain_region+Sex	14.407	118.000
ENSG00000047579	0.301	-0.054	0.144	-0.251	DTNBP1	0.354	~amyloid_beta+(1|manifest)+cngeneson+pc_mito+brain_region+Sex	3.436	1286.000
ENSG00000108506	0.020	-0.054	0.005	-0.113	INTS2	0.058	~amyloid_beta+(1|manifest)+cngeneson+pc_mito+brain_region+Sex	0.702	4876.000
ENSG00000204130	0.079	-0.054	0.111	-0.219	RUFY2	0.134	~amyloid_beta+(1|manifest)+cngeneson+pc_mito+brain_region+Sex	0.781	4664.000
ENSG00000138802	0.014	-0.054	0.068	-0.176	SEC24B	0.048	~amyloid_beta+(1|manifest)+cngeneson+pc_mito+brain_region+Sex	1.272	3559.000
ENSG00000136933	0.017	-0.054	0.051	-0.159	RABEPK	0.054	~amyloid_beta+(1|manifest)+cngeneson+pc_mito+brain_region+Sex	1.178	3750.000
ENSG00000143183	0.169	-0.054	0.075	-0.184	TMCO1	0.225	~amyloid_beta+(1|manifest)+cngeneson+pc_mito+brain_region+Sex	1.097	3935.000
ENSG00000227345	0.085	-0.054	0.061	-0.169	PARG	0.140	~amyloid_beta+(1|manifest)+cngeneson+pc_mito+brain_region+Sex	1.050	4026.000
ENSG00000139083	0.980	-0.054	0.513	-0.622	ETV6	0.982	~amyloid_beta+(1|manifest)+cngeneson+pc_mito+brain_region+Sex	16.717	79.000
ENSG00000115816	0.020	-0.054	0.015	-0.123	CEBPZ	0.059	~amyloid_beta+(1|manifest)+cngeneson+pc_mito+brain_region+Sex	0.600	5130.000
ENSG00000103740	0.858	-0.054	0.296	-0.405	ACSBG1	0.872	~amyloid_beta+(1|manifest)+cngeneson+pc_mito+brain_region+Sex	9.313	305.000
ENSG00000174437	0.374	-0.054	0.103	-0.212	ATP2A2	0.424	~amyloid_beta+(1|manifest)+cngeneson+pc_mito+brain_region+Sex	1.773	2723.000
ENSG00000113108	0.190	-0.055	0.107	-0.216	APBB3	0.245	~amyloid_beta+(1|manifest)+cngeneson+pc_mito+brain_region+Sex	3.093	1488.000
ENSG00000158161	0.031	-0.055	0.036	-0.146	EYA3	0.075	~amyloid_beta+(1|manifest)+cngeneson+pc_mito+brain_region+Sex	0.383	5591.000
ENSG00000135298	0.788	-0.055	0.149	-0.259	ADGRB3	0.808	~amyloid_beta+(1|manifest)+cngeneson+pc_mito+brain_region+Sex	25.070	29.000
ENSG00000197121	0.406	-0.055	0.182	-0.292	PGAP1	0.454	~amyloid_beta+(1|manifest)+cngeneson+pc_mito+brain_region+Sex	3.875	1095.000
ENSG00000196455	0.001	-0.055	-0.021	-0.089	PIK3R4	0.008	~amyloid_beta+(1|manifest)+cngeneson+pc_mito+brain_region+Sex	0.393	5573.000
ENSG00000156052	0.332	-0.055	0.022	-0.132	GNAQ	0.384	~amyloid_beta+(1|manifest)+cngeneson+pc_mito+brain_region+Sex	2.751	1705.000
ENSG00000104299	0.031	-0.055	0.160	-0.270	INTS9	0.076	~amyloid_beta+(1|manifest)+cngeneson+pc_mito+brain_region+Sex	2.617	1803.000
ENSG00000164741	0.635	-0.055	0.265	-0.374	DLC1	0.669	~amyloid_beta+(1|manifest)+cngeneson+pc_mito+brain_region+Sex	5.860	614.000
ENSG00000120008	0.687	-0.055	0.155	-0.265	WDR11	0.717	~amyloid_beta+(1|manifest)+cngeneson+pc_mito+brain_region+Sex	1.044	4037.000
ENSG00000101935	0.182	-0.055	0.034	-0.144	AMMECR1	0.236	~amyloid_beta+(1|manifest)+cngeneson+pc_mito+brain_region+Sex	1.809	2680.000
ENSG00000187391	0.650	-0.055	0.080	-0.190	MAGI2	0.682	~amyloid_beta+(1|manifest)+cngeneson+pc_mito+brain_region+Sex	11.996	176.000
ENSG00000163904	0.043	-0.055	0.012	-0.123	SENP2	0.091	~amyloid_beta+(1|manifest)+cngeneson+pc_mito+brain_region+Sex	0.574	5174.000
ENSG00000087299	0.026	-0.055	0.049	-0.160	L2HGDH	0.068	~amyloid_beta+(1|manifest)+cngeneson+pc_mito+brain_region+Sex	0.985	4179.000
ENSG00000104643	0.013	-0.055	0.033	-0.143	MTMR9	0.047	~amyloid_beta+(1|manifest)+cngeneson+pc_mito+brain_region+Sex	1.132	3859.000
ENSG00000134046	0.054	-0.055	0.070	-0.181	MBD2	0.104	~amyloid_beta+(1|manifest)+cngeneson+pc_mito+brain_region+Sex	1.835	2649.000
ENSG00000154240	0.575	-0.055	0.219	-0.330	CEP112	0.613	~amyloid_beta+(1|manifest)+cngeneson+pc_mito+brain_region+Sex	1.837	2646.000
ENSG00000172071	0.119	-0.056	0.040	-0.151	EIF2AK3	0.176	~amyloid_beta+(1|manifest)+cngeneson+pc_mito+brain_region+Sex	1.069	3988.000
ENSG00000131023	0.066	-0.056	0.091	-0.202	LATS1	0.119	~amyloid_beta+(1|manifest)+cngeneson+pc_mito+brain_region+Sex	1.246	3609.000
ENSG00000096717	0.058	-0.056	0.033	-0.144	SIRT1	0.109	~amyloid_beta+(1|manifest)+cngeneson+pc_mito+brain_region+Sex	0.694	4908.000
ENSG00000147854	0.146	-0.056	0.159	-0.270	UHRF2	0.202	~amyloid_beta+(1|manifest)+cngeneson+pc_mito+brain_region+Sex	3.939	1069.000
ENSG00000138756	0.758	-0.056	0.102	-0.214	BMP2K	0.781	~amyloid_beta+(1|manifest)+cngeneson+pc_mito+brain_region+Sex	4.838	811.000
ENSG00000272325	0.622	-0.056	0.388	-0.499	NUDT3	0.657	~amyloid_beta+(1|manifest)+cngeneson+pc_mito+brain_region+Sex	3.724	1151.000
ENSG00000146776	0.292	-0.056	0.116	-0.227	ATXN7L1	0.345	~amyloid_beta+(1|manifest)+cngeneson+pc_mito+brain_region+Sex	0.911	4353.000
ENSG00000130177	0.053	-0.056	0.088	-0.200	CDC16	0.103	~amyloid_beta+(1|manifest)+cngeneson+pc_mito+brain_region+Sex	1.511	3124.000
ENSG00000121931	0.046	-0.056	0.064	-0.177	LRIF1	0.095	~amyloid_beta+(1|manifest)+cngeneson+pc_mito+brain_region+Sex	0.422	5523.000
ENSG00000169967	0.205	-0.056	0.075	-0.187	MAP3K2	0.260	~amyloid_beta+(1|manifest)+cngeneson+pc_mito+brain_region+Sex	2.260	2144.000
ENSG00000127511	0.176	-0.056	0.078	-0.190	SIN3B	0.230	~amyloid_beta+(1|manifest)+cngeneson+pc_mito+brain_region+Sex	2.113	2295.000
ENSG00000162642	0.150	-0.056	0.071	-0.183	C1orf52	0.206	~amyloid_beta+(1|manifest)+cngeneson+pc_mito+brain_region+Sex	0.880	4419.000
ENSG00000089775	0.021	-0.056	0.091	-0.204	ZBTB25	0.061	~amyloid_beta+(1|manifest)+cngeneson+pc_mito+brain_region+Sex	1.937	2513.000
ENSG00000127314	0.096	-0.057	0.136	-0.249	RAP1B	0.151	~amyloid_beta+(1|manifest)+cngeneson+pc_mito+brain_region+Sex	2.876	1620.000
ENSG00000136100	0.030	-0.057	0.072	-0.186	VPS36	0.074	~amyloid_beta+(1|manifest)+cngeneson+pc_mito+brain_region+Sex	1.295	3515.000
ENSG00000147872	0.070	-0.057	0.038	-0.151	PLIN2	0.123	~amyloid_beta+(1|manifest)+cngeneson+pc_mito+brain_region+Sex	0.893	4390.000
ENSG00000058056	0.016	-0.057	0.017	-0.131	USP13	0.051	~amyloid_beta+(1|manifest)+cngeneson+pc_mito+brain_region+Sex	0.454	5447.000
ENSG00000006530	0.005	-0.057	0.021	-0.135	AGK	0.026	~amyloid_beta+(1|manifest)+cngeneson+pc_mito+brain_region+Sex	0.568	5189.000
ENSG00000100796	0.022	-0.057	0.053	-0.166	PPP4R3A	0.062	~amyloid_beta+(1|manifest)+cngeneson+pc_mito+brain_region+Sex	1.160	3791.000
ENSG00000086015	0.071	-0.057	0.151	-0.265	MAST2	0.125	~amyloid_beta+(1|manifest)+cngeneson+pc_mito+brain_region+Sex	3.229	1410.000
ENSG00000088930	0.096	-0.057	0.092	-0.206	XRN2	0.152	~amyloid_beta+(1|manifest)+cngeneson+pc_mito+brain_region+Sex	2.640	1780.000
ENSG00000124313	0.071	-0.057	0.078	-0.192	IQSEC2	0.125	~amyloid_beta+(1|manifest)+cngeneson+pc_mito+brain_region+Sex	1.265	3567.000
ENSG00000164180	0.155	-0.057	0.044	-0.158	[illegible]	0.211	~amyloid_beta+(1|manifest)+cngeneson+pc_mito+brain_region+Sex	1.057	4007.000
ENSG00000146411	0.082	-0.057	0.057	-0.171	SLC2A12	0.137	~amyloid_beta+(1|manifest)+cngeneson+pc_mito+brain_region+Sex	1.022	4094.000
ENSG00000162408	0.036	-0.057	0.000	-0.114	NOL9	0.082	~amyloid_beta+(1|manifest)+cngeneson+pc_mito+brain_region+Sex	0.608	5113.000
ENSG00000178585	0.104	-0.057	0.150	-0.265	CTNNBIP1	0.160	~amyloid_beta+(1|manifest)+cngeneson+pc_mito+brain_region+Sex	2.158	2251.000
ENSG00000011376	0.014	-0.058	0.015	-0.130	LARS2	0.048	~amyloid_beta+(1|manifest)+cngeneson+pc_mito+brain_region+Sex	0.637	5037.000
ENSG00000085382	0.164	-0.058	0.087	-0.202	HACE1	0.220	~amyloid_beta+(1|manifest)+cngeneson+pc_mito+brain_region+Sex	1.195	3715.000
ENSG00000170946	0.006	-0.058	0.009	-0.125	DNAJC24	0.028	~amyloid_beta+(1|manifest)+cngeneson+pc_mito+brain_region+Sex	1.000	4138.000
ENSG00000177034	0.017	-0.058	0.008	-0.123	MTX3	0.054	~amyloid_beta+(1|manifest)+cngeneson+pc_mito+brain_region+Sex	0.497	5370.000
ENSG00000070404	0.288	-0.058	0.342	-0.458	FSTL3	0.340	~amyloid_beta+(1|manifest)+cngeneson+pc_mito+brain_region+Sex	6.292	551.000
ENSG00000100601	0.013	-0.058	0.013	-0.129	ALKBH1	0.046	~amyloid_beta+(1|manifest)+cngeneson+pc_mito+brain_region+Sex	0.514	5319.000
ENSG00000104388	0.004	-0.058	0.063	-0.179	RAB2A	0.024	~amyloid_beta+(1|manifest)+cngeneson+pc_mito+brain_region+Sex	0.868	4444.000
ENSG00000115841	0.103	-0.058	0.011	-0.127	RMDN2	0.158	~amyloid_beta+(1|manifest)+cngeneson+pc_mito+brain_region+Sex	0.646	5013.000
ENSG00000145362	0.635	-0.058	0.083	-0.199	ANK2	0.669	~amyloid_beta+(1|manifest)+cngeneson+pc_mito+brain_region+Sex	7.933	381.000
ENSG00000083799	0.013	-0.058	0.065	-0.181	CYLD	0.046	~amyloid_beta+(1|manifest)+cngeneson+pc_mito+brain_region+Sex	1.177	3752.000
ENSG00000090054	0.072	-0.058	0.024	-0.140	SPTLC1	0.126	~amyloid_beta+(1|manifest)+cngeneson+pc_mito+brain_region+Sex	0.945	4264.000
ENSG00000198160	0.023	-0.058	0.092	-0.208	MIER1	0.064	~amyloid_beta+(1|manifest)+cngeneson+pc_mito+brain_region+Sex	1.927	2526.000
ENSG00000071073	0.181	-0.058	0.042	-0.159	MGAT4A	0.236	~amyloid_beta+(1|manifest)+cngeneson+pc_mito+brain_region+Sex	0.705	4865.000
ENSG00000164332	0.024	-0.058	0.008	-0.124	UBLCP1	0.065	~amyloid_beta+(1|manifest)+cngeneson+pc_mito+brain_region+Sex	0.192	5881.000
ENSG00000153914	0.332	-0.058	0.141	-0.257	SREK1	0.384	~amyloid_beta+(1|manifest)+cngeneson+pc_mito+brain_region+Sex	1.648	2910.000
ENSG00000157483	0.231	-0.058	0.158	-0.274	MYO1E	0.285	~amyloid_beta+(1|manifest)+cngeneson+pc_mito+brain_region+Sex	1.962	2474.000
ENSG00000154511	0.717	-0.058	0.135	-0.251	DIPK1A	0.744	~amyloid_beta+(1|manifest)+cngeneson+pc_mito+brain_region+Sex	2.395	1990.000
ENSG00000120798	0.023	-0.058	0.075	-0.192	NR2C1	0.065	~amyloid_beta+(1|manifest)+cngeneson+pc_mito+brain_region+Sex	1.829	2657.000
ENSG00000156298	0.502	-0.059	0.285	-0.402	TSPAN7	0.546	~amyloid_beta+(1|manifest)+cngeneson+pc_mito+brain_region+Sex	2.508	1890.000
ENSG00000151917	0.017	-0.059	0.048	-0.165	BEND6	0.054	~amyloid_beta+(1|manifest)+cngeneson+pc_mito+brain_region+Sex	0.723	4813.000
ENSG00000196247	0.044	-0.059	0.023	-0.140	ZNF107	0.093	~amyloid_beta+(1|manifest)+cngeneson+pc_mito+brain_region+Sex	1.074	3980.000
ENSG00000146535	0.743	-0.059	0.256	-0.373	GNA12	0.768	~amyloid_beta+(1|manifest)+cngeneson+pc_mito+brain_region+Sex	6.298	550.000
ENSG00000111912	0.231	-0.059	0.097	-0.214	NCOA7	0.285	~amyloid_beta+(1|manifest)+cngeneson+pc_mito+brain_region+Sex	1.384	3351.000
ENSG00000133114	0.002	-0.059	0.001	-0.118	GPALPP1	0.017	~amyloid_beta+(1|manifest)+cngeneson+pc_mito+brain_region+Sex	0.564	5200.000
ENSG00000127418	0.392	-0.059	0.145	-0.263	FGFRL1	0.441	~amyloid_beta+(1|manifest)+cngeneson+pc_mito+brain_region+Sex	1.107	3912.000
ENSG00000170522	0.284	-0.059	0.097	-0.215	ELOVL6	0.337	~amyloid_beta+(1|manifest)+cngeneson+pc_mito+brain_region+Sex	1.464	3198.000
ENSG00000160293	0.098	-0.059	0.091	-0.209	VAV2	0.153	~amyloid_beta+(1|manifest)+cngeneson+pc_mito+brain_region+Sex	1.383	3352.000
ENSG00000166199	0.026	-0.059	0.020	-0.138	ALKBH3	0.069	~amyloid_beta+(1|manifest)+cngeneson+pc_mito+brain_region+Sex	0.951	4252.000
ENSG00000145241	0.079	-0.059	0.092	-0.210	CENPC	0.134	~amyloid_beta+(1|manifest)+cngeneson+pc_mito+brain_region+Sex	0.391	5579.000
ENSG00000198315	0.005	-0.059	0.003	-0.121	ZKSCAN8	0.025	~amyloid_beta+(1|manifest)+cngeneson+pc_mito+brain_region+Sex	0.566	5195.000
ENSG00000261115	0.520	-0.059	0.167	-0.286	[illegible]	0.563	~amyloid_beta+(1|manifest)+cngeneson+pc_mito+brain_region+Sex	1.734	2771.000
ENSG00000116128	0.109	-0.059	0.076	-0.194	BCL9	0.164	~amyloid_beta+(1|manifest)+cngeneson+pc_mito+brain_region+Sex	1.161	3788.000
ENSG00000197102	0.202	-0.059	0.128	-0.247	DYNC1H1	0.256	~amyloid_beta+(1|manifest)+cngeneson+pc_mito+brain_region+Sex	3.702	1161.000
ENSG00000087502	0.002	-0.059	-0.016	-0.103	ERGIC2	0.017	~amyloid_beta+(1|manifest)+cngeneson+pc_mito+brain_region+Sex	0.509	5329.000
ENSG00000168438	0.018	-0.059	-0.006	-0.113	CDC40	0.055	~amyloid_beta+(1|manifest)+cngeneson+pc_mito+brain_region+Sex	0.691	4915.000
ENSG00000197930	0.116	-0.059	0.052	-0.171	ERO1A	0.172	~amyloid_beta+(1|manifest)+cngeneson+pc_mito+brain_region+Sex	1.159	3793.000
ENSG00000155287	0.091	-0.059	0.109	-0.228	SLC25A28	0.146	~amyloid_beta+(1|manifest)+cngeneson+pc_mito+brain_region+Sex	1.201	3703.000
ENSG00000137770	0.032	-0.060	0.051	-0.170	CTDSPL2	0.077	~amyloid_beta+(1|manifest)+cngeneson+pc_mito+brain_region+Sex	1.389	3340.000
ENSG00000157625	0.002	-0.060	-0.013	-0.107	TAB3	0.017	~amyloid_beta+(1|manifest)+cngeneson+pc_mito+brain_region+Sex	0.308	5716.000
ENSG00000125629	0.011	-0.060	-0.015	-0.105	INSIG2	0.042	~amyloid_beta+(1|manifest)+cngeneson+pc_mito+brain_region+Sex	0.264	5789.000
ENSG00000114446	0.022	-0.060	0.007	-0.127	IFT57	0.063	~amyloid_beta+(1|manifest)+cngeneson+pc_mito+brain_region+Sex	0.352	5646.000
ENSG00000005889	0.077	-0.060	0.056	-0.176	ZFX	0.132	~amyloid_beta+(1|manifest)+cngeneson+pc_mito+brain_region+Sex	1.840	2642.000
ENSG00000113580	0.268	-0.060	0.078	-0.198	NR3C1	0.322	~amyloid_beta+(1|manifest)+cngeneson+pc_mito+brain_region+Sex	0.786	4659.000
ENSG00000135845	0.112	-0.060	0.071	-0.191	PIGC	0.168	~amyloid_beta+(1|manifest)+cngeneson+pc_mito+brain_region+Sex	1.917	2539.000
ENSG00000072121	0.016	-0.060	0.049	-0.170	ZFYVE26	0.053	~amyloid_beta+(1|manifest)+cngeneson+pc_mito+brain_region+Sex	2.387	2005.000
ENSG00000075292	0.529	-0.061	0.062	-0.183	ZNF638	0.571	~amyloid_beta+(1|manifest)+cngeneson+pc_mito+brain_region+Sex	2.306	2096.000
ENSG00000044446	0.060	-0.061	0.080	-0.201	PHKA2	0.111	~amyloid_beta+(1|manifest)+cngeneson+pc_mito+brain_region+Sex	1.085	3960.000
ENSG00000089060	0.114	-0.061	0.086	-0.207	SLC8B1	0.170	~amyloid_beta+(1|manifest)+cngeneson+pc_mito+brain_region+Sex	1.376	3364.000
ENSG00000120029	0.053	-0.061	0.091	-0.213	ARMH3	0.103	~amyloid_beta+(1|manifest)+cngeneson+pc_mito+brain_region+Sex	1.540	3065.000
ENSG00000151553	0.131	-0.061	0.090	-0.212	[illegible]	0.188	~amyloid_beta+(1|manifest)+cngeneson+pc_mito+brain_region+Sex	0.479	5403.000
ENSG00000135540	0.835	-0.061	0.143	-0.265	NHSL1	0.852	~amyloid_beta+(1|manifest)+cngeneson+pc_mito+brain_region+Sex	9.751	282.000
ENSG00000198843	0.039	-0.061	0.030	-0.152	SELENOT	0.086	~amyloid_beta+(1|manifest)+cngeneson+pc_mito+brain_region+Sex	0.356	5641.000
ENSG00000082258	0.058	-0.061	0.092	-0.215	CCNT2	0.109	~amyloid_beta+(1|manifest)+cngeneson+pc_mito+brain_region+Sex	1.102	3925.000
ENSG00000136146	0.034	-0.062	0.017	-0.141	MED4	0.080	~amyloid_beta+(1|manifest)+cngeneson+pc_mito+brain_region+Sex	0.363	5628.000
ENSG00000122507	0.815	-0.062	0.202	-0.325	BBS9	0.834	~amyloid_beta+(1|manifest)+cngeneson+pc_mito+brain_region+Sex	2.471	1922.000
ENSG00000055609	0.110	-0.062	0.010	-0.134	KMT2C	0.166	~amyloid_beta+(1|manifest)+cngeneson+pc_mito+brain_region+Sex	2.812	1657.000
ENSG00000143322	0.803	-0.062	0.145	-0.268	ABL2	0.822	~amyloid_beta+(1|manifest)+cngeneson+pc_mito+brain_region+Sex	2.516	1883.000
ENSG00000062725	0.032	-0.062	0.075	-0.199	APPBP2	0.076	~amyloid_beta+(1|manifest)+cngeneson+pc_mito+brain_region+Sex	1.194	3719.000
ENSG00000164022	0.012	-0.062	0.000	-0.123	AIMP1	0.043	~amyloid_beta+(1|manifest)+cngeneson+pc_mito+brain_region+Sex	0.715	4834.000
ENSG00000164761	0.065	-0.062	0.066	-0.190	[illegible]	0.118	~amyloid_beta+(1|manifest)+cngeneson+pc_mito+brain_region+Sex	1.983	2455.000
ENSG00000136111	0.436	-0.062	0.121	-0.245	TBC1D4	0.482	~amyloid_beta+(1|manifest)+cngeneson+pc_mito+brain_region+Sex	8.411	351.000
ENSG00000140199	0.178	-0.062	0.114	-0.239	SLC12A6	0.233	~amyloid_beta+(1|manifest)+cngeneson+pc_mito+brain_region+Sex	1.751	2747.000
ENSG00000135912	0.052	-0.062	0.086	-0.210	TTLL4	0.102	~amyloid_beta+(1|manifest)+cngeneson+pc_mito+brain_region+Sex	2.029	2397.000
ENSG00000106799	0.142	-0.062	0.010	-0.134	TGFBR1	0.198	~amyloid_beta+(1|manifest)+cngeneson+pc_mito+brain_region+Sex	0.462	5438.000
ENSG00000143013	0.325	-0.062	0.203	-0.328	LMO4	0.377	~amyloid_beta+(1|manifest)+cngeneson+pc_mito+brain_region+Sex	2.242	2163.000
ENSG00000147475	0.124	-0.062	0.052	-0.177	ERLIN2	0.181	~amyloid_beta+(1|manifest)+cngeneson+pc_mito+brain_region+Sex	1.109	3911.000
ENSG00000061676	0.729	-0.062	0.115	-0.240	NCKAP1	0.756	~amyloid_beta+(1|manifest)+cngeneson+pc_mito+brain_region+Sex	3.632	1190.000
ENSG00000008294	0.065	-0.062	0.014	-0.138	SPAG9	0.118	~amyloid_beta+(1|manifest)+cngeneson+pc_mito+brain_region+Sex	3.348	1340.000
ENSG00000100888	0.050	-0.063	0.035	-0.160	CHD8	0.100	~amyloid_beta+(1|manifest)+cngeneson+pc_mito+brain_region+Sex	1.074	3983.000
ENSG00000148339	0.032	-0.063	0.146	-0.271	SLC25A25	0.077	~amyloid_beta+(1|manifest)+cngeneson+pc_mito+brain_region+Sex	2.099	2315.000
ENSG00000168066	0.350	-0.063	0.148	-0.273	SF1	0.400	~amyloid_beta+(1|manifest)+cngeneson+pc_mito+brain_region+Sex	3.409	1309.000
ENSG00000047621	0.079	-0.063	0.111	-0.236	C12orf4	0.134	~amyloid_beta+(1|manifest)+cngeneson+pc_mito+brain_region+Sex	2.688	1735.000
ENSG00000138376	0.256	-0.063	0.194	-0.320	BARD1	0.310	~amyloid_beta+(1|manifest)+cngeneson+pc_mito+brain_region+Sex	4.671	850.000
ENSG00000088367	0.104	-0.063	0.204	-0.330	EPB41L1	0.160	~amyloid_beta+(1|manifest)+cngeneson+pc_mito+brain_region+Sex	1.385	3348.000
ENSG00000114999	0.017	-0.063	0.054	-0.180	TTL	0.054	~amyloid_beta+(1|manifest)+cngeneson+pc_mito+brain_region+Sex	1.688	2846.000
ENSG00000164182	0.559	-0.063	0.092	-0.218	NDUFAF2	0.599	~amyloid_beta+(1|manifest)+cngeneson+pc_mito+brain_region+Sex	2.243	2161.000
ENSG00000131697	0.010	-0.063	0.035	-0.162	NPHP4	0.040	~amyloid_beta+(1|manifest)+cngeneson+pc_mito+brain_region+Sex	1.051	4020.000
ENSG00000138036	0.044	-0.063	0.132	-0.259	DYNC2LI1	0.092	~amyloid_beta+(1|manifest)+cngeneson+pc_mito+brain_region+Sex	2.207	2198.000
ENSG00000150347	0.632	-0.063	0.239	-0.366	ARID5B	0.667	~amyloid_beta+(1|manifest)+cngeneson+pc_mito+brain_region+Sex	3.975	1049.000
ENSG00000131127	0.103	-0.063	0.159	-0.286	ZNF141	0.158	~amyloid_beta+(1|manifest)+cngeneson+pc_mito+brain_region+Sex	2.606	1806.000
ENSG00000089902	0.169	-0.064	0.055	-0.182	RCOR1	0.225	~amyloid_beta+(1|manifest)+cngeneson+pc_mito+brain_region+Sex	1.323	3458.000
ENSG00000143442	0.380	-0.064	0.040	-0.168	POGZ	0.429	~amyloid_beta+(1|manifest)+cngeneson+pc_mito+brain_region+Sex	5.605	665.000
ENSG00000128191	0.070	-0.064	0.020	-0.148	DGCR8	0.123	~amyloid_beta+(1|manifest)+cngeneson+pc_mito+brain_region+Sex	0.994	4154.000
ENSG00000166181	0.013	-0.064	-0.004	-0.124	API5	0.047	~amyloid_beta+(1|manifest)+cngeneson+pc_mito+brain_region+Sex	0.281	5764.000
ENSG00000069275	0.082	-0.064	0.110	-0.237	NUCKS1	0.137	~amyloid_beta+(1|manifest)+cngeneson+pc_mito+brain_region+Sex	2.647	1772.000
ENSG00000176142	0.004	-0.064	-0.019	-0.108	TMEM39A	0.025	~amyloid_beta+(1|manifest)+cngeneson+pc_mito+brain_region+Sex	0.255	5801.000
ENSG00000176244	0.269	-0.064	0.098	-0.226	ACBD7	0.322	~amyloid_beta+(1|manifest)+cngeneson+pc_mito+brain_region+Sex	1.957	2485.000
ENSG00000131171	0.454	-0.064	0.170	-0.298	SH3BGRL	0.499	~amyloid_beta+(1|manifest)+cngeneson+pc_mito+brain_region+Sex	1.795	2710.000
ENSG00000119938	0.905	-0.064	0.215	-0.343	PPP1R3C	0.916	~amyloid_beta+(1|manifest)+cngeneson+pc_mito+brain_region+Sex	1.808	2682.000
ENSG00000168310	0.225	-0.064	0.107	-0.235	IRF2	0.280	~amyloid_beta+(1|manifest)+cngeneson+pc_mito+brain_region+Sex	1.605	2978.000
ENSG00000086232	0.006	-0.064	-0.012	-0.116	EIF2AK1	0.028	~amyloid_beta+(1|manifest)+cngeneson+pc_mito+brain_region+Sex	0.584	5160.000
ENSG00000113966	0.067	-0.064	0.016	-0.145	ARL6	0.120	~amyloid_beta+(1|manifest)+cngeneson+pc_mito+brain_region+Sex	0.975	4194.000
ENSG00000056097	0.098	-0.064	0.056	-0.185	ZFR	0.153	~amyloid_beta+(1|manifest)+cngeneson+pc_mito+brain_region+Sex	1.350	3413.000
ENSG00000148110	0.023	-0.065	0.004	-0.133	MFSD14B	0.065	~amyloid_beta+(1|manifest)+cngeneson+pc_mito+brain_region+Sex	0.502	5353.000
ENSG00000061936	0.066	-0.065	0.093	-0.222	SFSWAP	0.120	~amyloid_beta+(1|manifest)+cngeneson+pc_mito+brain_region+Sex	1.971	2466.000
ENSG00000163812	0.012	-0.065	0.094	-0.223	ZDHHC3	0.043	~amyloid_beta+(1|manifest)+cngeneson+pc_mito+brain_region+Sex	1.545	3059.000
ENSG00000115966	0.253	-0.065	0.135	-0.264	ATF2	0.307	~amyloid_beta+(1|manifest)+cngeneson+pc_mito+brain_region+Sex	0.556	5220.000
ENSG00000141401	0.052	-0.065	0.070	-0.199	IMPA2	0.102	~amyloid_beta+(1|manifest)+cngeneson+pc_mito+brain_region+Sex	1.381	3354.000
ENSG00000100596	0.127	-0.065	0.061	-0.190	SPTLC2	0.183	~amyloid_beta+(1|manifest)+cngeneson+pc_mito+brain_region+Sex	2.053	2362.000
ENSG00000017260	0.895	-0.065	0.409	-0.539	ATP2C1	0.907	~amyloid_beta+(1|manifest)+cngeneson+pc_mito+brain_region+Sex	11.724	190.000
ENSG00000105793	0.028	-0.065	0.009	-0.139	GTPBP10	0.072	~amyloid_beta+(1|manifest)+cngeneson+pc_mito+brain_region+Sex	1.316	3469.000
ENSG00000135341	0.029	-0.065	0.100	-0.230	MAP3K7	0.073	~amyloid_beta+(1|manifest)+cngeneson+pc_mito+brain_region+Sex	1.838	2644.000
ENSG00000171223	0.895	-0.065	0.637	-0.766	JUNB	0.907	~amyloid_beta+(1|manifest)+cngeneson+pc_mito+brain_region+Sex	7.759	394.000
ENSG00000113194	0.019	-0.065	0.036	-0.167	FAF2	0.058	~amyloid_beta+(1|manifest)+cngeneson+pc_mito+brain_region+Sex	1.170	3765.000
ENSG00000153015	0.190	-0.065	0.137	-0.267	CWC27	0.245	~amyloid_beta+(1|manifest)+cngeneson+pc_mito+brain_region+Sex	1.133	3856.000
ENSG00000172939	0.029	-0.065	0.038	-0.168	OXSR1	0.073	~amyloid_beta+(1|manifest)+cngeneson+pc_mito+brain_region+Sex	0.748	4745.000
ENSG00000153140	0.089	-0.065	0.046	-0.177	CETN3	0.143	~amyloid_beta+(1|manifest)+cngeneson+pc_mito+brain_region+Sex	0.547	5236.000
ENSG00000135541	0.800	-0.065	0.192	-0.323	AHI1	0.820	~amyloid_beta+(1|manifest)+cngeneson+pc_mito+brain_region+Sex	7.750	395.000
ENSG00000155313	0.146	-0.066	0.132	-0.263	USP25	0.202	~amyloid_beta+(1|manifest)+cngeneson+pc_mito+brain_region+Sex	2.342	2060.000
ENSG00000133678	0.060	-0.066	0.000	-0.131	TMEM254	0.112	~amyloid_beta+(1|manifest)+cngeneson+pc_mito+brain_region+Sex	0.675	4948.000
ENSG00000171984	0.039	-0.066	0.001	-0.132	SHLD1	0.087	~amyloid_beta+(1|manifest)+cngeneson+pc_mito+brain_region+Sex	0.813	4592.000
ENSG00000140386	0.421	-0.066	0.058	-0.190	SCAPER	0.468	~amyloid_beta+(1|manifest)+cngeneson+pc_mito+brain_region+Sex	2.945	1579.000
ENSG00000036448	0.081	-0.066	0.018	-0.150	MYOM2	0.136	~amyloid_beta+(1|manifest)+cngeneson+pc_mito+brain_region+Sex	1.139	3839.000
ENSG00000125818	0.007	-0.066	0.061	-0.193	PSMF1	0.031	~amyloid_beta+(1|manifest)+cngeneson+pc_mito+brain_region+Sex	1.524	3099.000
ENSG00000131966	0.030	-0.066	0.085	-0.218	ACTR10	0.074	~amyloid_beta+(1|manifest)+cngeneson+pc_mito+brain_region+Sex	0.498	5366.000
ENSG00000205659	0.065	-0.066	0.090	-0.222	LIN52	0.118	~amyloid_beta+(1|manifest)+cngeneson+pc_mito+brain_region+Sex	0.860	4466.000
ENSG00000132950	0.026	-0.066	0.087	-0.219	ZMYM5	0.068	~amyloid_beta+(1|manifest)+cngeneson+pc_mito+brain_region+Sex	1.637	2928.000
ENSG00000175387	0.046	-0.066	0.085	-0.217	SMAD2	0.095	~amyloid_beta+(1|manifest)+cngeneson+pc_mito+brain_region+Sex	1.626	2943.000
ENSG00000027697	0.077	-0.066	0.017	-0.149	IFNGR1	0.131	~amyloid_beta+(1|manifest)+cngeneson+pc_mito+brain_region+Sex	0.861	4462.000
ENSG00000151233	0.026	-0.067	0.002	-0.135	GXYLT1	0.068	~amyloid_beta+(1|manifest)+cngeneson+pc_mito+brain_region+Sex	0.611	5107.000
ENSG00000119596	0.193	-0.067	0.090	-0.223	YLPM1	0.248	~amyloid_beta+(1|manifest)+cngeneson+pc_mito+brain_region+Sex	2.301	2106.000
ENSG00000122729	0.096	-0.067	0.122	-0.256	ACO1	0.151	~amyloid_beta+(1|manifest)+cngeneson+pc_mito+brain_region+Sex	2.680	1746.000
ENSG00000077514	0.288	-0.067	0.081	-0.214	POLD3	0.341	~amyloid_beta+(1|manifest)+cngeneson+pc_mito+brain_region+Sex	1.659	2891.000
ENSG00000105792	0.006	-0.067	0.019	-0.153	CFAP69	0.030	~amyloid_beta+(1|manifest)+cngeneson+pc_mito+brain_region+Sex	0.303	5731.000
ENSG00000146263	0.403	-0.067	0.029	-0.163	MMS22L	0.450	~amyloid_beta+(1|manifest)+cngeneson+pc_mito+brain_region+Sex	0.458	5443.000
ENSG00000139116	0.361	-0.067	0.121	-0.255	KIF21A	0.412	~amyloid_beta+(1|manifest)+cngeneson+pc_mito+brain_region+Sex	5.840	619.000
ENSG00000109118	0.027	-0.067	0.028	-0.162	PHF12	0.071	~amyloid_beta+(1|manifest)+cngeneson+pc_mito+brain_region+Sex	0.973	4198.000
ENSG00000065613	0.023	-0.067	0.082	-0.216	SLK	0.065	~amyloid_beta+(1|manifest)+cngeneson+pc_mito+brain_region+Sex	1.911	2542.000
ENSG00000138767	0.329	-0.067	0.160	-0.295	CNOT6L	0.381	~amyloid_beta+(1|manifest)+cngeneson+pc_mito+brain_region+Sex	3.074	1495.000
ENSG00000140367	0.018	-0.067	0.004	-0.139	UBE2Q2	0.056	~amyloid_beta+(1|manifest)+cngeneson+pc_mito+brain_region+Sex	0.541	5255.000
ENSG00000156500	0.025	-0.067	0.036	-0.170	FAM122C	0.067	~amyloid_beta+(1|manifest)+cngeneson+pc_mito+brain_region+Sex	0.589	5156.000
ENSG00000021574	0.030	-0.067	0.028	-0.163	SPAST	0.074	~amyloid_beta+(1|manifest)+cngeneson+pc_mito+brain_region+Sex	0.800	4631.000
ENSG00000011523	0.041	-0.067	0.022	-0.157	CEP68	0.089	~amyloid_beta+(1|manifest)+cngeneson+pc_mito+brain_region+Sex	1.033	4065.000
ENSG00000076641	0.196	-0.068	0.106	-0.241	PAG1	0.250	~amyloid_beta+(1|manifest)+cngeneson+pc_mito+brain_region+Sex	2.149	2264.000
ENSG00000115524	0.130	-0.068	0.107	-0.242	SF3B1	0.187	~amyloid_beta+(1|manifest)+cngeneson+pc_mito+brain_region+Sex	3.047	1512.000
ENSG00000166734	0.253	-0.068	0.079	-0.214	CASC4	0.307	~amyloid_beta+(1|manifest)+cngeneson+pc_mito+brain_region+Sex	2.114	2294.000
ENSG00000175216	0.030	-0.068	0.029	-0.164	CKAP5	0.075	~amyloid_beta+(1|manifest)+cngeneson+pc_mito+brain_region+Sex	1.423	3283.000
ENSG00000166471	0.081	-0.068	0.063	-0.198	TMEM41B	0.136	~amyloid_beta+(1|manifest)+cngeneson+pc_mito+brain_region+Sex	1.254	3590.000
ENSG00000089847	0.063	-0.068	0.050	-0.186	ANKRD24	0.115	~amyloid_beta+(1|manifest)+cngeneson+pc_mito+brain_region+Sex	1.032	4068.000
ENSG00000153815	0.825	-0.068	0.253	-0.389	CMIP	0.843	~amyloid_beta+(1|manifest)+cngeneson+pc_mito+brain_region+Sex	7.800	391.000
ENSG00000151445	0.015	-0.069	0.000	-0.137	VIPAS39	0.050	~amyloid_beta+(1|manifest)+cngeneson+pc_mito+brain_region+Sex	0.338	5666.000
ENSG00000141580	0.048	-0.069	0.028	-0.165	WDR45B	0.097	~amyloid_beta+(1|manifest)+cngeneson+pc_mito+brain_region+Sex	0.739	4770.000
ENSG00000135372	0.048	-0.069	0.016	-0.153	NAT10	0.097	~amyloid_beta+(1|manifest)+cngeneson+pc_mito+brain_region+Sex	0.833	4537.000
ENSG00000108424	0.174	-0.069	0.112	-0.249	KPNB1	0.229	~amyloid_beta+(1|manifest)+cngeneson+pc_mito+brain_region+Sex	2.233	2170.000
ENSG00000110696	0.115	-0.069	0.069	-0.206	C11orf58	0.171	~amyloid_beta+(1|manifest)+cngeneson+pc_mito+brain_region+Sex	0.858	4470.000
ENSG00000182827	0.002	-0.069	-0.016	-0.121	ACBD3	0.018	~amyloid_beta+(1|manifest)+cngeneson+pc_mito+brain_region+Sex	0.617	5094.000
ENSG00000113300	0.025	-0.069	-0.003	-0.135	CNOT6	0.067	~amyloid_beta+(1|manifest)+cngeneson+pc_mito+brain_region+Sex	0.357	5639.000
ENSG00000148516	0.669	-0.069	0.087	-0.225	ZEB1	0.700	~amyloid_beta+(1|manifest)+cngeneson+pc_mito+brain_region+Sex	3.494	1252.000
ENSG00000198270	0.012	-0.069	-0.020	-0.118	TMEM116	0.044	~amyloid_beta+(1|manifest)+cngeneson+pc_mito+brain_region+Sex	0.518	5307.000
ENSG00000083896	0.082	-0.070	0.128	-0.267	YTHDC1	0.137	~amyloid_beta+(1|manifest)+cngeneson+pc_mito+brain_region+Sex	0.328	5679.000
ENSG00000196911	0.095	-0.070	0.046	-0.185	KPNA5	0.151	~amyloid_beta+(1|manifest)+cngeneson+pc_mito+brain_region+Sex	0.928	4308.000
ENSG00000196605	0.086	-0.070	0.048	-0.187	ZNF846	0.141	~amyloid_beta+(1|manifest)+cngeneson+pc_mito+brain_region+Sex	0.833	4536.000
ENSG00000170743	0.174	-0.070	0.183	-0.322	SYT9	0.229	~amyloid_beta+(1|manifest)+cngeneson+pc_mito+brain_region+Sex	0.637	5036.000
ENSG00000163558	0.004	-0.070	0.094	-0.233	PRKCI	0.022	~amyloid_beta+(1|manifest)+cngeneson+pc_mito+brain_region+Sex	2.210	2193.000
ENSG00000138246	0.002	-0.070	0.067	-0.207	DNAJC13	0.017	~amyloid_beta+(1|manifest)+cngeneson+pc_mito+brain_region+Sex	0.907	4361.000
ENSG00000170271	0.059	-0.070	0.279	-0.419	FAXDC2	0.110	~amyloid_beta+(1|manifest)+cngeneson+pc_mito+brain_region+Sex	2.996	1545.000
ENSG00000118922	0.930	-0.070	0.298	-0.438	KLF12	0.938	~amyloid_beta+(1|manifest)+cngeneson+pc_mito+brain_region+Sex	7.384	427.000
ENSG00000084754	0.061	-0.070	0.066	-0.206	HADHA	0.112	~amyloid_beta+(1|manifest)+cngeneson+pc_mito+brain_region+Sex	1.732	2779.000
ENSG00000162688	0.390	-0.070	0.136	-0.276	AGL	0.439	~amyloid_beta+(1|manifest)+cngeneson+pc_mito+brain_region+Sex	1.995	2439.000
ENSG00000117335	0.046	-0.070	0.062	-0.203	CD46	0.095	~amyloid_beta+(1|manifest)+cngeneson+pc_mito+brain_region+Sex	1.930	2522.000
ENSG00000004455	0.042	-0.070	0.030	-0.171	AK2	0.090	~amyloid_beta+(1|manifest)+cngeneson+pc_mito+brain_region+Sex	1.217	3668.000
ENSG00000132694	0.410	-0.070	0.157	-0.298	ARHGEF11	0.456	~amyloid_beta+(1|manifest)+cngeneson+pc_mito+brain_region+Sex	4.925	793.000
ENSG00000136045	0.006	-0.071	-0.015	-0.126	PWP1	0.029	~amyloid_beta+(1|manifest)+cngeneson+pc_mito+brain_region+Sex	0.597	5137.000
ENSG00000107554	0.095	-0.071	0.023	-0.164	DNMBP	0.150	~amyloid_beta+(1|manifest)+cngeneson+pc_mito+brain_region+Sex	2.377	2020.000
ENSG00000104957	0.074	-0.071	0.114	-0.255	CCDC130	0.127	~amyloid_beta+(1|manifest)+cngeneson+pc_mito+brain_region+Sex	2.352	2043.000
ENSG00000162971	0.019	-0.071	0.020	-0.161	TYW5	0.057	~amyloid_beta+(1|manifest)+cngeneson+pc_mito+brain_region+Sex	0.410	5547.000
ENSG00000133943	0.630	-0.071	0.224	-0.366	DGLUCY	0.665	~amyloid_beta+(1|manifest)+cngeneson+pc_mito+brain_region+Sex	4.606	861.000
ENSG00000142794	0.097	-0.071	0.011	-0.153	NBPF3	0.152	~amyloid_beta+(1|manifest)+cngeneson+pc_mito+brain_region+Sex	0.498	5364.000
ENSG00000165119	0.058	-0.071	0.082	-0.224	HNRNPK	0.109	~amyloid_beta+(1|manifest)+cngeneson+pc_mito+brain_region+Sex	1.341	3426.000
ENSG00000135913	0.044	-0.071	0.061	-0.202	USP37	0.093	~amyloid_beta+(1|manifest)+cngeneson+pc_mito+brain_region+Sex	1.381	3355.000
ENSG00000183576	0.089	-0.071	0.051	-0.193	SETD3	0.144	~amyloid_beta+(1|manifest)+cngeneson+pc_mito+brain_region+Sex	1.172	3763.000
ENSG00000114209	0.009	-0.071	0.020	-0.162	PDCD10	0.039	~amyloid_beta+(1|manifest)+cngeneson+pc_mito+brain_region+Sex	0.810	4600.000
ENSG00000166033	0.739	-0.071	0.329	-0.471	HTRA1	0.765	~amyloid_beta+(1|manifest)+cngeneson+pc_mito+brain_region+Sex	5.000	775.000
ENSG00000170802	0.309	-0.071	0.172	-0.314	FOXN2	0.361	~amyloid_beta+(1|manifest)+cngeneson+pc_mito+brain_region+Sex	3.389	1323.000
ENSG00000141385	0.185	-0.071	0.075	-0.217	AFG3L2	0.240	~amyloid_beta+(1|manifest)+cngeneson+pc_mito+brain_region+Sex	1.695	2830.000
ENSG00000033122	0.401	-0.071	0.131	-0.274	LRRC7	0.449	~amyloid_beta+(1|manifest)+cngeneson+pc_mito+brain_region+Sex	2.722	1718.000
ENSG00000152749	0.054	-0.071	0.009	-0.152	GPR180	0.104	~amyloid_beta+(1|manifest)+cngeneson+pc_mito+brain_region+Sex	0.159	5915.000
ENSG00000144959	0.144	-0.071	0.068	-0.211	NCEH1	0.200	~amyloid_beta+(1|manifest)+cngeneson+pc_mito+brain_region+Sex	1.144	3829.000
ENSG00000066777	0.106	-0.072	0.078	-0.221	ARFGEF1	0.162	~amyloid_beta+(1|manifest)+cngeneson+pc_mito+brain_region+Sex	2.057	2360.000
ENSG00000185483	0.374	-0.072	0.433	-0.577	ROR1	0.423	~amyloid_beta+(1|manifest)+cngeneson+pc_mito+brain_region+Sex	7.264	447.000
ENSG00000130340	0.050	-0.072	0.127	-0.270	SNX9	0.099	~amyloid_beta+(1|manifest)+cngeneson+pc_mito+brain_region+Sex	2.031	2394.000
ENSG00000123159	0.095	-0.072	0.074	-0.217	GIPC1	0.150	~amyloid_beta+(1|manifest)+cngeneson+pc_mito+brain_region+Sex	1.889	2579.000
ENSG00000108094	0.002	-0.072	-0.024	-0.119	CUL2	0.015	~amyloid_beta+(1|manifest)+cngeneson+pc_mito+brain_region+Sex	0.645	5018.000
ENSG00000164654	0.010	-0.072	-0.010	-0.134	MIOS	0.039	~amyloid_beta+(1|manifest)+cngeneson+pc_mito+brain_region+Sex	1.141	3833.000
ENSG00000143878	0.622	-0.072	0.364	-0.507	RHOB	0.657	~amyloid_beta+(1|manifest)+cngeneson+pc_mito+brain_region+Sex	11.271	208.000
ENSG00000137872	0.171	-0.072	0.086	-0.230	SEMA6D	0.226	~amyloid_beta+(1|manifest)+cngeneson+pc_mito+brain_region+Sex	1.529	3089.000
ENSG00000205593	0.101	-0.072	0.112	-0.256	DENND6B	0.156	~amyloid_beta+(1|manifest)+cngeneson+pc_mito+brain_region+Sex	1.729	2783.000
ENSG00000131013	0.061	-0.072	0.050	-0.194	PPIL4	0.112	~amyloid_beta+(1|manifest)+cngeneson+pc_mito+brain_region+Sex	0.401	5564.000
ENSG00000124588	0.276	-0.072	0.081	-0.225	NQO2	0.329	~amyloid_beta+(1|manifest)+cngeneson+pc_mito+brain_region+Sex	1.423	3284.000
ENSG00000279170	0.064	-0.072	0.075	-0.220	TSTD3	0.116	~amyloid_beta+(1|manifest)+cngeneson+pc_mito+brain_region+Sex	1.234	3625.000
ENSG00000006125	0.134	-0.072	0.081	-0.226	AP2B1	0.191	~amyloid_beta+(1|manifest)+cngeneson+pc_mito+brain_region+Sex	1.604	2979.000
ENSG00000110713	0.016	-0.072	0.068	-0.213	NUP98	0.053	~amyloid_beta+(1|manifest)+cngeneson+pc_mito+brain_region+Sex	1.301	3505.000
ENSG00000178105	0.207	-0.073	0.024	-0.169	DDX10	0.262	~amyloid_beta+(1|manifest)+cngeneson+pc_mito+brain_region+Sex	1.674	2864.000
ENSG00000222011	0.138	-0.073	0.017	-0.162	FAM185A	0.195	~amyloid_beta+(1|manifest)+cngeneson+pc_mito+brain_region+Sex	0.377	5603.000
ENSG00000118263	0.825	-0.073	0.255	-0.400	KLF7	0.843	~amyloid_beta+(1|manifest)+cngeneson+pc_mito+brain_region+Sex	2.621	1798.000
ENSG00000159592	0.126	-0.073	0.093	-0.238	GPBP1L1	0.182	~amyloid_beta+(1|manifest)+cngeneson+pc_mito+brain_region+Sex	2.543	1861.000
ENSG00000033800	0.609	-0.073	0.108	-0.254	PIAS1	0.645	~amyloid_beta+(1|manifest)+cngeneson+pc_mito+brain_region+Sex	1.867	2605.000
ENSG00000114982	0.141	-0.073	0.050	-0.196	KANSL3	0.198	~amyloid_beta+(1|manifest)+cngeneson+pc_mito+brain_region+Sex	1.538	3070.000
ENSG00000156535	0.094	-0.073	0.250	-0.396	CD109	0.150	~amyloid_beta+(1|manifest)+cngeneson+pc_mito+brain_region+Sex	0.605	5117.000
ENSG00000123066	0.578	-0.073	0.075	-0.221	MED13L	0.616	~amyloid_beta+(1|manifest)+cngeneson+pc_mito+brain_region+Sex	8.826	323.000
ENSG00000174405	0.015	-0.073	0.037	-0.183	LIG4	0.050	~amyloid_beta+(1|manifest)+cngeneson+pc_mito+brain_region+Sex	1.495	3144.000
ENSG00000149541	0.148	-0.073	0.087	-0.233	B3GAT3	0.204	~amyloid_beta+(1|manifest)+cngeneson+pc_mito+brain_region+Sex	0.888	4404.000
ENSG00000170325	0.217	-0.073	0.071	-0.217	PRDM10	0.272	~amyloid_beta+(1|manifest)+cngeneson+pc_mito+brain_region+Sex	1.874	2596.000
ENSG00000133048	0.913	-0.073	1.348	-1.495	CHI3L1	0.923	~amyloid_beta+(1|manifest)+cngeneson+pc_mito+brain_region+Sex	49.076	7.000
ENSG00000164879	0.581	-0.073	0.080	-0.227	CA3	0.618	~amyloid_beta+(1|manifest)+cngeneson+pc_mito+brain_region+Sex	3.238	1404.000
ENSG00000135049	0.141	-0.073	0.106	-0.253	AGTPBP1	0.198	~amyloid_beta+(1|manifest)+cngeneson+pc_mito+brain_region+Sex	1.564	3033.000
ENSG00000184743	0.106	-0.074	0.044	-0.192	ATL3	0.162	~amyloid_beta+(1|manifest)+cngeneson+pc_mito+brain_region+Sex	0.689	4921.000
ENSG00000103227	0.167	-0.074	0.210	-0.357	LMF1	0.222	~amyloid_beta+(1|manifest)+cngeneson+pc_mito+brain_region+Sex	2.503	1901.000
ENSG00000134121	0.912	-0.074	0.313	-0.461	CHL1	0.922	~amyloid_beta+(1|manifest)+cngeneson+pc_mito+brain_region+Sex	13.458	138.000
ENSG00000145833	0.231	-0.074	0.137	-0.285	DDX46	0.285	~amyloid_beta+(1|manifest)+cngeneson+pc_mito+brain_region+Sex	2.178	2226.000
ENSG00000106028	0.023	-0.074	0.051	-0.199	SSBP1	0.064	~amyloid_beta+(1|manifest)+cngeneson+pc_mito+brain_region+Sex	0.438	5489.000
ENSG00000132359	0.074	-0.074	0.123	-0.271	RAP1GAP2	0.128	~amyloid_beta+(1|manifest)+cngeneson+pc_mito+brain_region+Sex	1.156	3798.000
ENSG00000133401	0.445	-0.074	0.243	-0.391	PDZD2	0.490	~amyloid_beta+(1|manifest)+cngeneson+pc_mito+brain_region+Sex	2.703	1726.000
ENSG00000144036	0.391	-0.074	0.037	-0.186	EXOC6B	0.440	~amyloid_beta+(1|manifest)+cngeneson+pc_mito+brain_region+Sex	3.236	1406.000
ENSG00000136925	0.016	-0.074	0.030	-0.178	TSTD2	0.053	~amyloid_beta+(1|manifest)+cngeneson+pc_mito+brain_region+Sex	1.561	3039.000
ENSG00000185565	0.250	-0.074	0.011	-0.160	LSAMP	0.304	~amyloid_beta+(1|manifest)+cngeneson+pc_mito+brain_region+Sex	14.626	109.000
ENSG00000112146	0.143	-0.074	0.076	-0.225	FBXO9	0.199	~amyloid_beta+(1|manifest)+cngeneson+pc_mito+brain_region+Sex	1.614	2959.000
ENSG00000152409	0.126	-0.075	0.054	-0.203	JMY	0.182	~amyloid_beta+(1|manifest)+cngeneson+pc_mito+brain_region+Sex	0.772	4681.000
ENSG00000198265	0.040	-0.075	0.029	-0.178	HELZ	0.088	~amyloid_beta+(1|manifest)+cngeneson+pc_mito+brain_region+Sex	1.175	3758.000
ENSG00000230124	0.244	-0.075	0.107	-0.257	ACBD6	0.298	~amyloid_beta+(1|manifest)+cngeneson+pc_mito+brain_region+Sex	0.318	5698.000
ENSG00000169180	0.061	-0.075	0.045	-0.194	XPO6	0.113	~amyloid_beta+(1|manifest)+cngeneson+pc_mito+brain_region+Sex	1.334	3443.000
ENSG00000121742	0.247	-0.075	0.261	-0.411	GJB6	0.301	~amyloid_beta+(1|manifest)+cngeneson+pc_mito+brain_region+Sex	2.245	2160.000
ENSG00000125107	0.050	-0.075	0.050	-0.200	CNOT1	0.099	~amyloid_beta+(1|manifest)+cngeneson+pc_mito+brain_region+Sex	1.777	2719.000
ENSG00000152223	0.002	-0.075	0.023	-0.173	EPG5	0.016	~amyloid_beta+(1|manifest)+cngeneson+pc_mito+brain_region+Sex	0.842	4516.000
ENSG00000135048	0.108	-0.075	0.062	-0.212	CEMIP2	0.164	~amyloid_beta+(1|manifest)+cngeneson+pc_mito+brain_region+Sex	2.185	2221.000
ENSG00000116906	0.042	-0.075	0.009	-0.159	GNPAT	0.089	~amyloid_beta+(1|manifest)+cngeneson+pc_mito+brain_region+Sex	0.999	4144.000
ENSG00000100427	0.753	-0.075	0.310	-0.460	MLC1	0.777	~amyloid_beta+(1|manifest)+cngeneson+pc_mito+brain_region+Sex	4.881	804.000
ENSG00000268350	0.016	-0.075	0.009	-0.159	FAM156A	0.053	~amyloid_beta+(1|manifest)+cngeneson+pc_mito+brain_region+Sex	0.665	4969.000
ENSG00000189144	0.007	-0.075	-0.001	-0.149	ZNF573	0.032	~amyloid_beta+(1|manifest)+cngeneson+pc_mito+brain_region+Sex	0.936	4286.000
ENSG00000148053	0.619	-0.075	0.117	-0.268	NTRK2	0.655	~amyloid_beta+(1|manifest)+cngeneson+pc_mito+brain_region+Sex	18.300	66.000
ENSG00000165669	0.176	-0.075	0.079	-0.230	FAM204A	0.231	~amyloid_beta+(1|manifest)+cngeneson+pc_mito+brain_region+Sex	1.242	3615.000
ENSG00000101542	0.640	-0.075	0.092	-0.243	CDH20	0.674	~amyloid_beta+(1|manifest)+cngeneson+pc_mito+brain_region+Sex	12.712	162.000
ENSG00000165813	0.015	-0.076	0.006	-0.157	CCDC186	0.050	~amyloid_beta+(1|manifest)+cngeneson+pc_mito+brain_region+Sex	0.759	4720.000
ENSG00000099326	0.205	-0.076	0.166	-0.318	MZF1	0.260	~amyloid_beta+(1|manifest)+cngeneson+pc_mito+brain_region+Sex	3.341	1346.000
ENSG00000113761	0.089	-0.076	0.145	-0.296	ZNF346	0.143	~amyloid_beta+(1|manifest)+cngeneson+pc_mito+brain_region+Sex	2.020	2410.000
ENSG00000111530	0.031	-0.076	0.022	-0.174	CAND1	0.075	~amyloid_beta+(1|manifest)+cngeneson+pc_mito+brain_region+Sex	0.999	4143.000
ENSG00000141551	0.047	-0.076	0.070	-0.222	CSNK1D	0.096	~amyloid_beta+(1|manifest)+cngeneson+pc_mito+brain_region+Sex	1.957	2484.000
ENSG00000171862	0.259	-0.076	0.088	-0.241	PTEN	0.313	~amyloid_beta+(1|manifest)+cngeneson+pc_mito+brain_region+Sex	0.886	4410.000
ENSG00000170525	0.938	-0.076	0.455	-0.608	PFKFB3	0.945	~amyloid_beta+(1|manifest)+cngeneson+pc_mito+brain_region+Sex	20.358	50.000
ENSG00000178568	0.448	-0.076	0.053	-0.205	ERBB4	0.493	~amyloid_beta+(1|manifest)+cngeneson+pc_mito+brain_region+Sex	9.889	275.000
ENSG00000143324	0.569	-0.077	0.199	-0.352	XPR1	0.607	~amyloid_beta+(1|manifest)+cngeneson+pc_mito+brain_region+Sex	0.672	4960.000
ENSG00000203667	0.136	-0.077	0.090	-0.243	COX20	0.193	~amyloid_beta+(1|manifest)+cngeneson+pc_mito+brain_region+Sex	1.944	2503.000
ENSG00000162434	0.786	-0.077	0.207	-0.360	JAK1	0.807	~amyloid_beta+(1|manifest)+cngeneson+pc_mito+brain_region+Sex	6.491	525.000
ENSG00000153147	0.051	-0.077	0.032	-0.186	SMARCA5	0.101	~amyloid_beta+(1|manifest)+cngeneson+pc_mito+brain_region+Sex	0.867	4451.000
ENSG00000005206	0.662	-0.077	0.444	-0.598	SPPL2B	0.694	~amyloid_beta+(1|manifest)+cngeneson+pc_mito+brain_region+Sex	9.654	284.000
ENSG00000005175	0.078	-0.077	0.022	-0.175	RPAP3	0.133	~amyloid_beta+(1|manifest)+cngeneson+pc_mito+brain_region+Sex	1.894	2560.000
ENSG00000175215	0.129	-0.077	0.082	-0.236	CTDSP2	0.186	~amyloid_beta+(1|manifest)+cngeneson+pc_mito+brain_region+Sex	0.987	4172.000
ENSG00000109111	0.085	-0.077	0.149	-0.303	SUPT6H	0.140	~amyloid_beta+(1|manifest)+cngeneson+pc_mito+brain_region+Sex	2.345	2054.000
ENSG00000182600	0.240	-0.077	0.191	-0.346	SNORC	0.293	~amyloid_beta+(1|manifest)+cngeneson+pc_mito+brain_region+Sex	2.228	2174.000
ENSG00000106392	0.081	-0.077	0.075	-0.229	C1GALT1	0.135	~amyloid_beta+(1|manifest)+cngeneson+pc_mito+brain_region+Sex	1.739	2764.000
ENSG00000136936	0.055	-0.077	0.051	-0.206	XPA	0.106	~amyloid_beta+(1|manifest)+cngeneson+pc_mito+brain_region+Sex	1.871	2598.000
ENSG00000054690	0.089	-0.077	0.073	-0.227	PLEKHH1	0.143	~amyloid_beta+(1|manifest)+cngeneson+pc_mito+brain_region+Sex	1.534	3078.000
ENSG00000130988	0.844	-0.077	0.304	-0.459	RGN	0.860	~amyloid_beta+(1|manifest)+cngeneson+pc_mito+brain_region+Sex	8.025	374.000
ENSG00000172671	0.010	-0.077	-0.010	-0.145	ZFAND4	0.040	~amyloid_beta+(1|manifest)+cngeneson+pc_mito+brain_region+Sex	0.591	5151.000
ENSG00000132964	0.134	-0.077	0.083	-0.238	CDK8	0.190	~amyloid_beta+(1|manifest)+cngeneson+pc_mito+brain_region+Sex	2.029	2396.000
ENSG00000163624	0.472	-0.078	0.178	-0.333	CDS1	0.517	~amyloid_beta+(1|manifest)+cngeneson+pc_mito+brain_region+Sex	1.869	2603.000
ENSG00000140153	0.337	-0.078	0.068	-0.223	WDR20	0.389	~amyloid_beta+(1|manifest)+cngeneson+pc_mito+brain_region+Sex	1.660	2890.000
ENSG00000273559	0.163	-0.078	0.101	-0.256	CWC25	0.218	~amyloid_beta+(1|manifest)+cngeneson+pc_mito+brain_region+Sex	1.336	3436.000
ENSG00000114423	0.272	-0.078	0.067	-0.223	CBLB	0.326	~amyloid_beta+(1|manifest)+cngeneson+pc_mito+brain_region+Sex	0.703	4874.000
ENSG00000176473	0.041	-0.078	0.002	-0.158	WDR25	0.088	~amyloid_beta+(1|manifest)+cngeneson+pc_mito+brain_region+Sex	0.148	5924.000
ENSG00000198218	0.056	-0.078	0.035	-0.191	QRICH1	0.106	~amyloid_beta+(1|manifest)+cngeneson+pc_mito+brain_region+Sex	0.960	4226.000
ENSG00000111581	0.010	-0.078	0.012	-0.168	NUP107	0.041	~amyloid_beta+(1|manifest)+cngeneson+pc_mito+brain_region+Sex	0.614	5101.000
ENSG00000131263	0.041	-0.078	0.024	-0.180	RLIM	0.089	~amyloid_beta+(1|manifest)+cngeneson+pc_mito+brain_region+Sex	0.941	4275.000
ENSG00000163110	0.499	-0.078	0.054	-0.211	PDLIM5	0.543	~amyloid_beta+(1|manifest)+cngeneson+pc_mito+brain_region+Sex	1.429	3266.000
ENSG00000135407	0.616	-0.078	0.101	-0.257	AVIL	0.652	~amyloid_beta+(1|manifest)+cngeneson+pc_mito+brain_region+Sex	3.708	1159.000
ENSG00000203685	0.956	-0.078	0.441	-0.598	STUM	0.960	~amyloid_beta+(1|manifest)+cngeneson+pc_mito+brain_region+Sex	4.371	926.000
ENSG00000068784	0.156	-0.078	0.028	-0.184	SRBD1	0.211	~amyloid_beta+(1|manifest)+cngeneson+pc_mito+brain_region+Sex	0.810	4599.000
ENSG00000132294	0.516	-0.078	0.180	-0.336	EFR3A	0.559	~amyloid_beta+(1|manifest)+cngeneson+pc_mito+brain_region+Sex	2.769	1692.000
ENSG00000123213	0.045	-0.078	0.045	-0.202	NLN	0.094	~amyloid_beta+(1|manifest)+cngeneson+pc_mito+brain_region+Sex	1.167	3775.000
ENSG00000120519	0.019	-0.078	0.095	-0.252	SLC10A7	0.057	~amyloid_beta+(1|manifest)+cngeneson+pc_mito+brain_region+Sex	1.283	3537.000
ENSG00000104047	0.072	-0.078	0.034	-0.191	DTWD1	0.126	~amyloid_beta+(1|manifest)+cngeneson+pc_mito+brain_region+Sex	0.592	5149.000
ENSG00000128512	0.433	-0.078	0.049	-0.206	DOCK4	0.479	~amyloid_beta+(1|manifest)+cngeneson+pc_mito+brain_region+Sex	15.636	92.000
ENSG00000174428	0.076	-0.079	0.128	-0.285	[illegible]	0.130	~amyloid_beta+(1|manifest)+cngeneson+pc_mito+brain_region+Sex	1.626	2944.000
ENSG00000158079	0.045	-0.079	0.009	-0.166	PTPDC1	0.094	~amyloid_beta+(1|manifest)+cngeneson+pc_mito+brain_region+Sex	1.033	4064.000
ENSG00000155636	0.002	-0.079	-0.022	-0.136	RBM45	0.018	~amyloid_beta+(1|manifest)+cngeneson+pc_mito+brain_region+Sex	0.790	4653.000
ENSG00000141027	0.454	-0.079	0.072	-0.230	NCOR1	0.499	~amyloid_beta+(1|manifest)+cngeneson+pc_mito+brain_region+Sex	4.994	776.000
ENSG00000132471	0.253	-0.079	0.137	-0.295	WBP2	0.307	~amyloid_beta+(1|manifest)+cngeneson+pc_mito+brain_region+Sex	2.969	1560.000
ENSG00000138496	0.357	-0.079	0.253	-0.410	PARP9	0.408	~amyloid_beta+(1|manifest)+cngeneson+pc_mito+brain_region+Sex	4.679	844.000
ENSG00000120910	0.223	-0.079	0.127	-0.285	PPP3CC	0.278	~amyloid_beta+(1|manifest)+cngeneson+pc_mito+brain_region+Sex	1.597	2988.000
ENSG00000214013	0.211	-0.079	0.100	-0.259	GANC	0.266	~amyloid_beta+(1|manifest)+cngeneson+pc_mito+brain_region+Sex	1.492	3151.000
ENSG00000185627	0.038	-0.079	0.023	-0.181	PSMD13	0.086	~amyloid_beta+(1|manifest)+cngeneson+pc_mito+brain_region+Sex	1.660	2888.000
ENSG00000127980	0.010	-0.079	-0.002	-0.156	PEX1	0.041	~amyloid_beta+(1|manifest)+cngeneson+pc_mito+brain_region+Sex	0.860	4467.000
ENSG00000049239	0.100	-0.079	0.061	-0.219	H6PD	0.156	~amyloid_beta+(1|manifest)+cngeneson+pc_mito+brain_region+Sex	1.310	3479.000
ENSG00000182158	0.046	-0.079	0.051	-0.209	CREB3L2	0.095	~amyloid_beta+(1|manifest)+cngeneson+pc_mito+brain_region+Sex	0.845	4509.000
ENSG00000171262	0.007	-0.079	-0.024	-0.135	FAM98B	0.033	~amyloid_beta+(1|manifest)+cngeneson+pc_mito+brain_region+Sex	0.504	5347.000
ENSG00000172765	0.312	-0.079	0.055	-0.214	TMCC1	0.365	~amyloid_beta+(1|manifest)+cngeneson+pc_mito+brain_region+Sex	2.285	2119.000
ENSG00000196313	0.003	-0.079	-0.029	-0.130	POM121	0.020	~amyloid_beta+(1|manifest)+cngeneson+pc_mito+brain_region+Sex	0.288	5748.000
ENSG00000117054	0.022	-0.080	0.015	-0.174	ACADM	0.062	~amyloid_beta+(1|manifest)+cngeneson+pc_mito+brain_region+Sex	1.229	3646.000
ENSG00000124177	0.143	-0.080	0.015	-0.174	CHD6	0.199	~amyloid_beta+(1|manifest)+cngeneson+pc_mito+brain_region+Sex	1.623	2949.000
ENSG00000103647	0.225	-0.080	0.151	-0.310	CORO2B	0.280	~amyloid_beta+(1|manifest)+cngeneson+pc_mito+brain_region+Sex	1.069	3993.000
ENSG00000094631	0.085	-0.080	0.117	-0.277	HDAC6	0.140	~amyloid_beta+(1|manifest)+cngeneson+pc_mito+brain_region+Sex	2.289	2117.000
ENSG00000198742	0.300	-0.080	0.052	-0.212	SMURF1	0.353	~amyloid_beta+(1|manifest)+cngeneson+pc_mito+brain_region+Sex	1.424	3281.000
ENSG00000160551	0.696	-0.080	0.143	-0.303	TAOK1	0.726	~amyloid_beta+(1|manifest)+cngeneson+pc_mito+brain_region+Sex	6.015	589.000
ENSG00000155657	0.807	-0.080	0.256	-0.417	TTN	0.826	~amyloid_beta+(1|manifest)+cngeneson+pc_mito+brain_region+Sex	5.176	733.000
ENSG00000171634	0.356	-0.080	0.046	-0.207	BPTF	0.407	~amyloid_beta+(1|manifest)+cngeneson+pc_mito+brain_region+Sex	3.966	1055.000
ENSG00000131323	0.083	-0.080	0.116	-0.277	TRAF3	0.138	~amyloid_beta+(1|manifest)+cngeneson+pc_mito+brain_region+Sex	1.571	3022.000
ENSG00000132716	0.163	-0.080	0.108	-0.269	DCAF8	0.218	~amyloid_beta+(1|manifest)+cngeneson+pc_mito+brain_region+Sex	2.522	1879.000
ENSG00000123684	0.011	-0.081	-0.003	-0.159	LPGAT1	0.042	~amyloid_beta+(1|manifest)+cngeneson+pc_mito+brain_region+Sex	0.920	4329.000
ENSG00000148541	0.595	-0.081	0.337	-0.499	FAM13C	0.631	~amyloid_beta+(1|manifest)+cngeneson+pc_mito+brain_region+Sex	2.756	1699.000
ENSG00000147642	0.393	-0.081	0.206	-0.367	SYBU	0.441	~amyloid_beta+(1|manifest)+cngeneson+pc_mito+brain_region+Sex	2.290	2116.000
ENSG00000058063	0.006	-0.081	0.027	-0.189	ATP11B	0.029	~amyloid_beta+(1|manifest)+cngeneson+pc_mito+brain_region+Sex	1.230	3638.000
ENSG00000163029	0.016	-0.081	0.013	-0.175	SMC6	0.052	~amyloid_beta+(1|manifest)+cngeneson+pc_mito+brain_region+Sex	1.483	3170.000
ENSG00000101126	0.281	-0.081	0.074	-0.236	ADNP	0.334	~amyloid_beta+(1|manifest)+cngeneson+pc_mito+brain_region+Sex	2.269	2134.000
ENSG00000037474	0.062	-0.081	0.072	-0.235	NSUN2	0.115	~amyloid_beta+(1|manifest)+cngeneson+pc_mito+brain_region+Sex	3.377	1332.000
ENSG00000143294	0.006	-0.081	-0.018	-0.145	PRCC	0.029	~amyloid_beta+(1|manifest)+cngeneson+pc_mito+brain_region+Sex	0.637	5041.000
ENSG00000164219	0.008	-0.081	-0.022	-0.141	PGGT1B	0.035	~amyloid_beta+(1|manifest)+cngeneson+pc_mito+brain_region+Sex	0.853	4491.000
ENSG00000165219	0.012	-0.081	0.022	-0.185	GAPVD1	0.044	~amyloid_beta+(1|manifest)+cngeneson+pc_mito+brain_region+Sex	1.090	3950.000
ENSG00000066739	0.014	-0.081	0.022	-0.184	ATG2B	0.047	~amyloid_beta+(1|manifest)+cngeneson+pc_mito+brain_region+Sex	0.856	4479.000
ENSG00000180263	0.382	-0.082	0.118	-0.282	FGD6	0.432	~amyloid_beta+(1|manifest)+cngeneson+pc_mito+brain_region+Sex	2.572	1836.000
ENSG00000120832	0.022	-0.082	0.025	-0.188	MTERF2	0.063	~amyloid_beta+(1|manifest)+cngeneson+pc_mito+brain_region+Sex	1.111	3905.000
ENSG00000166123	0.852	-0.082	0.367	-0.530	GPT2	0.867	~amyloid_beta+(1|manifest)+cngeneson+pc_mito+brain_region+Sex	2.915	1598.000
ENSG00000111647	0.227	-0.082	0.136	-0.299	[illegible]	0.282	~amyloid_beta+(1|manifest)+cngeneson+pc_mito+brain_region+Sex	4.288	951.000
ENSG00000143748	0.009	-0.082	0.000	-0.164	NVL	0.038	~amyloid_beta+(1|manifest)+cngeneson+pc_mito+brain_region+Sex	0.715	4833.000
ENSG00000078070	0.026	-0.082	0.082	-0.245	MCCC1	0.069	~amyloid_beta+(1|manifest)+cngeneson+pc_mito+brain_region+Sex	1.229	3647.000
ENSG00000086200	0.012	-0.082	0.031	-0.195	IPO11	0.043	~amyloid_beta+(1|manifest)+cngeneson+pc_mito+brain_region+Sex	1.090	3949.000
ENSG00000137502	0.038	-0.082	0.044	-0.208	RAB30	0.086	~amyloid_beta+(1|manifest)+cngeneson+pc_mito+brain_region+Sex	1.028	4083.000
ENSG00000153560	0.004	-0.082	-0.006	-0.159	UBP1	0.024	~amyloid_beta+(1|manifest)+cngeneson+pc_mito+brain_region+Sex	0.813	4590.000
ENSG00000165633	0.009	-0.082	0.030	-0.195	VSTM4	0.037	~amyloid_beta+(1|manifest)+cngeneson+pc_mito+brain_region+Sex	0.493	5382.000
ENSG00000130956	0.009	-0.082	0.055	-0.220	HABP4	0.037	~amyloid_beta+(1|manifest)+cngeneson+pc_mito+brain_region+Sex	1.363	3386.000
ENSG00000122707	0.046	-0.082	0.024	-0.188	RECK	0.095	~amyloid_beta+(1|manifest)+cngeneson+pc_mito+brain_region+Sex	0.928	4312.000
ENSG00000172795	0.032	-0.082	0.012	-0.177	DCP2	0.076	~amyloid_beta+(1|manifest)+cngeneson+pc_mito+brain_region+Sex	1.199	3709.000
ENSG00000080200	0.091	-0.082	0.111	-0.276	CRYBG3	0.147	~amyloid_beta+(1|manifest)+cngeneson+pc_mito+brain_region+Sex	1.889	2577.000
ENSG00000182973	0.035	-0.082	0.033	-0.198	CNOT10	0.081	~amyloid_beta+(1|manifest)+cngeneson+pc_mito+brain_region+Sex	1.047	4032.000
ENSG00000161526	0.033	-0.082	0.080	-0.245	SAP30BP	0.078	~amyloid_beta+(1|manifest)+cngeneson+pc_mito+brain_region+Sex	2.185	2222.000
ENSG00000197622	0.123	-0.082	0.065	-0.230	CDC42SE1	0.180	~amyloid_beta+(1|manifest)+cngeneson+pc_mito+brain_region+Sex	1.300	3506.000
ENSG00000185052	0.930	-0.083	0.354	-0.520	SLC24A3	0.938	~amyloid_beta+(1|manifest)+cngeneson+pc_mito+brain_region+Sex	3.753	1141.000
ENSG00000198799	0.015	-0.083	-0.010	-0.155	LRIG2	0.051	~amyloid_beta+(1|manifest)+cngeneson+pc_mito+brain_region+Sex	1.276	3554.000
ENSG00000164574	0.682	-0.083	0.191	-0.356	GALNT10	0.712	~amyloid_beta+(1|manifest)+cngeneson+pc_mito+brain_region+Sex	5.916	604.000
ENSG00000143653	0.006	-0.083	-0.018	-0.148	SCCPDH	0.030	~amyloid_beta+(1|manifest)+cngeneson+pc_mito+brain_region+Sex	0.333	5668.000
ENSG00000022976	0.042	-0.083	0.025	-0.191	ZNF839	0.089	~amyloid_beta+(1|manifest)+cngeneson+pc_mito+brain_region+Sex	1.359	3393.000
ENSG00000133704	0.023	-0.083	0.005	-0.171	IPO8	0.065	~amyloid_beta+(1|manifest)+cngeneson+pc_mito+brain_region+Sex	1.082	3965.000
ENSG00000169604	0.202	-0.083	0.330	-0.496	ANTXR1	0.257	~amyloid_beta+(1|manifest)+cngeneson+pc_mito+brain_region+Sex	5.747	636.000
ENSG00000117697	0.018	-0.083	0.008	-0.174	NSL1	0.056	~amyloid_beta+(1|manifest)+cngeneson+pc_mito+brain_region+Sex	0.441	5481.000

ENSG00000164144	0.233	-0.083	0.103	-0.269	ARFIP1	0.287	~amyloid_beta+(1|manifest)+cngeneson+pc_mito+brain_region+Sex	3.076	1494.000
ENSG00000060339	0.198	-0.083	0.146	-0.313	CCAR1	0.252	~amyloid_beta+(1|manifest)+cngeneson+pc_mito+brain_region+Sex	2.921	1594.000
ENSG00000137815	0.127	-0.083	0.074	-0.240	RTF1	0.183	~amyloid_beta+(1|manifest)+cngeneson+pc_mito+brain_region+Sex	1.331	3444.000
ENSG00000107890	0.328	-0.083	0.161	-0.328	ANKRD26	0.380	~amyloid_beta+(1|manifest)+cngeneson+pc_mito+brain_region+Sex	3.120	1470.000
ENSG00000127249	0.840	-0.084	0.209	-0.377	ATP13A4	0.856	~amyloid_beta+(1|manifest)+cngeneson+pc_mito+brain_region+Sex	12.605	163.000
ENSG00000152492	0.117	-0.084	0.068	-0.235	CCDC50	0.174	~amyloid_beta+(1|manifest)+cngeneson+pc_mito+brain_region+Sex	0.944	4270.000
ENSG00000150401	0.119	-0.084	0.068	-0.236	DCUN1D2	0.175	~amyloid_beta+(1|manifest)+cngeneson+pc_mito+brain_region+Sex	1.614	2960.000
ENSG00000148606	0.020	-0.084	0.017	-0.185	POLR3A	0.059	~amyloid_beta+(1|manifest)+cngeneson+pc_mito+brain_region+Sex	1.321	3462.000
ENSG00000129675	0.436	-0.084	0.146	-0.314	ARHGEF6	0.482	~amyloid_beta+(1|manifest)+cngeneson+pc_mito+brain_region+Sex	2.327	2073.000
ENSG00000153944	0.275	-0.084	0.038	-0.206	MSI2	0.328	~amyloid_beta+(1|manifest)+cngeneson+pc_mito+brain_region+Sex	8.926	320.000
ENSG00000185973	0.061	-0.084	0.040	-0.207	TMLHE	0.113	~amyloid_beta+(1|manifest)+cngeneson+pc_mito+brain_region+Sex	0.941	4276.000
ENSG00000140859	0.128	-0.084	0.053	-0.221	KIFC3	0.184	~amyloid_beta+(1|manifest)+cngeneson+pc_mito+brain_region+Sex	0.935	4289.000
ENSG00000154001	0.048	-0.084	0.053	-0.221	PPP2R5E	0.097	~amyloid_beta+(1|manifest)+cngeneson+pc_mito+brain_region+Sex	1.326	3451.000
ENSG00000172733	0.410	-0.084	0.185	-0.353	PURG	0.457	~amyloid_beta+(1|manifest)+cngeneson+pc_mito+brain_region+Sex	1.707	2813.000
ENSG00000188647	0.868	-0.084	0.299	-0.467	PTAR1	0.882	~amyloid_beta+(1|manifest)+cngeneson+pc_mito+brain_region+Sex	5.179	732.000
ENSG00000103034	0.141	-0.084	0.116	-0.284	NDRG4	0.197	~amyloid_beta+(1|manifest)+cngeneson+pc_mito+brain_region+Sex	1.492	3153.000
ENSG00000182993	0.610	-0.084	0.074	-0.242	C12orf60	0.647	~amyloid_beta+(1|manifest)+cngeneson+pc_mito+brain_region+Sex	4.485	894.000
ENSG00000144306	0.034	-0.084	0.016	-0.185	SCRN3	0.080	~amyloid_beta+(1|manifest)+cngeneson+pc_mito+brain_region+Sex	0.855	4484.000
ENSG00000053900	0.012	-0.084	0.011	-0.180	ANAPC4	0.045	~amyloid_beta+(1|manifest)+cngeneson+pc_mito+brain_region+Sex	1.298	3510.000
ENSG00000111684	0.417	-0.085	0.097	-0.266	LPCAT3	0.463	~amyloid_beta+(1|manifest)+cngeneson+pc_mito+brain_region+Sex	2.695	1734.000
ENSG00000128915	0.029	-0.085	0.053	-0.222	ICE2	0.073	~amyloid_beta+(1|manifest)+cngeneson+pc_mito+brain_region+Sex	0.531	5285.000
ENSG00000112308	0.151	-0.085	0.150	-0.319	C6orf62	0.207	~amyloid_beta+(1|manifest)+cngeneson+pc_mito+brain_region+Sex	1.069	3989.000
ENSG00000107164	0.028	-0.085	0.022	-0.191	FUBP3	0.072	~amyloid_beta+(1|manifest)+cngeneson+pc_mito+brain_region+Sex	0.828	4544.000
ENSG00000130640	0.192	-0.085	0.112	-0.282	TUBGCP2	0.246	~amyloid_beta+(1|manifest)+cngeneson+pc_mito+brain_region+Sex	2.743	1709.000
ENSG00000143379	0.040	-0.085	0.034	-0.204	SETDB1	0.087	~amyloid_beta+(1|manifest)+cngeneson+pc_mito+brain_region+Sex	1.630	2939.000
ENSG00000170921	0.520	-0.085	0.125	-0.295	TANC2	0.563	~amyloid_beta+(1|manifest)+cngeneson+pc_mito+brain_region+Sex	2.582	1828.000
ENSG00000142892	0.006	-0.085	-0.005	-0.165	PIGK	0.030	~amyloid_beta+(1|manifest)+cngeneson+pc_mito+brain_region+Sex	0.235	5831.000
ENSG00000109572	0.118	-0.085	0.045	-0.215	CLCN3	0.174	~amyloid_beta+(1|manifest)+cngeneson+pc_mito+brain_region+Sex	0.573	5180.000
ENSG00000151461	0.145	-0.085	0.073	-0.243	UPF2	0.201	~amyloid_beta+(1|manifest)+cngeneson+pc_mito+brain_region+Sex	1.640	2923.000
ENSG00000163374	0.617	-0.085	0.213	-0.383	YY1AP1	0.653	~amyloid_beta+(1|manifest)+cngeneson+pc_mito+brain_region+Sex	9.269	309.000
ENSG00000186017	0.026	-0.085	-0.006	-0.164	ZNF566	0.068	~amyloid_beta+(1|manifest)+cngeneson+pc_mito+brain_region+Sex	0.948	4259.000
ENSG00000116698	0.439	-0.085	0.122	-0.293	SMG7	0.484	~amyloid_beta+(1|manifest)+cngeneson+pc_mito+brain_region+Sex	2.168	2241.000
ENSG00000104626	0.159	-0.085	0.019	-0.190	ERI1	0.214	~amyloid_beta+(1|manifest)+cngeneson+pc_mito+brain_region+Sex	1.428	3267.000
ENSG00000196437	0.008	-0.085	-0.007	-0.164	ZNF569	0.036	~amyloid_beta+(1|manifest)+cngeneson+pc_mito+brain_region+Sex	1.148	3817.000
ENSG00000107959	0.023	-0.085	0.014	-0.185	PITRM1	0.064	~amyloid_beta+(1|manifest)+cngeneson+pc_mito+brain_region+Sex	1.399	3325.000
ENSG00000138592	0.118	-0.086	0.064	-0.235	USP8	0.174	~amyloid_beta+(1|manifest)+cngeneson+pc_mito+brain_region+Sex	1.129	3865.000
ENSG00000121989	0.095	-0.086	0.021	-0.193	ACVR2A	0.150	~amyloid_beta+(1|manifest)+cngeneson+pc_mito+brain_region+Sex	1.181	3742.000
ENSG00000106443	0.480	-0.086	0.087	-0.258	PHF14	0.525	~amyloid_beta+(1|manifest)+cngeneson+pc_mito+brain_region+Sex	1.802	2692.000
ENSG00000164815	0.002	-0.086	-0.023	-0.148	ORC5	0.017	~amyloid_beta+(1|manifest)+cngeneson+pc_mito+brain_region+Sex	0.302	5733.000
ENSG00000074695	0.006	-0.086	-0.012	-0.160	LMAN1	0.030	~amyloid_beta+(1|manifest)+cngeneson+pc_mito+brain_region+Sex	0.149	5923.000
ENSG00000123472	0.061	-0.086	0.036	-0.208	ATPAF1	0.112	~amyloid_beta+(1|manifest)+cngeneson+pc_mito+brain_region+Sex	0.533	5275.000
ENSG00000005810	0.107	-0.086	0.031	-0.204	MYCBP2	0.163	~amyloid_beta+(1|manifest)+cngeneson+pc_mito+brain_region+Sex	1.377	3361.000
ENSG00000065833	0.512	-0.086	0.333	-0.505	ME1	0.554	~amyloid_beta+(1|manifest)+cngeneson+pc_mito+brain_region+Sex	1.233	3628.000
ENSG00000088448	0.259	-0.086	0.135	-0.308	ANKRD10	0.313	~amyloid_beta+(1|manifest)+cngeneson+pc_mito+brain_region+Sex	4.508	890.000
ENSG00000141425	0.025	-0.086	0.042	-0.215	RPRD1A	0.068	~amyloid_beta+(1|manifest)+cngeneson+pc_mito+brain_region+Sex	1.064	4001.000
ENSG00000121486	0.000	-0.087	-0.046	-0.127	TRMT1L	0.003	~amyloid_beta+(1|manifest)+cngeneson+pc_mito+brain_region+Sex	0.357	5640.000
ENSG00000197008	0.089	-0.087	0.039	-0.212	ZNF138	0.144	~amyloid_beta+(1|manifest)+cngeneson+pc_mito+brain_region+Sex	1.054	4014.000
ENSG00000104331	0.041	-0.087	-0.015	-0.158	IMPAD1	0.089	~amyloid_beta+(1|manifest)+cngeneson+pc_mito+brain_region+Sex	0.386	5587.000
ENSG00000006016	0.629	-0.087	0.250	-0.423	CRLF1	0.665	~amyloid_beta+(1|manifest)+cngeneson+pc_mito+brain_region+Sex	7.556	409.000
ENSG00000137145	0.113	-0.087	0.076	-0.249	DENND4C	0.168	~amyloid_beta+(1|manifest)+cngeneson+pc_mito+brain_region+Sex	3.471	1263.000
ENSG00000118007	0.656	-0.087	0.116	-0.290	STAG1	0.688	~amyloid_beta+(1|manifest)+cngeneson+pc_mito+brain_region+Sex	1.365	3382.000
ENSG00000131591	0.030	-0.087	0.049	-0.223	C1orf159	0.074	~amyloid_beta+(1|manifest)+cngeneson+pc_mito+brain_region+Sex	0.907	4362.000
ENSG00000166821	0.052	-0.087	0.027	-0.201	PEX11A	0.102	~amyloid_beta+(1|manifest)+cngeneson+pc_mito+brain_region+Sex	1.597	2989.000
ENSG00000090863	0.423	-0.087	0.055	-0.230	GLG1	0.469	~amyloid_beta+(1|manifest)+cngeneson+pc_mito+brain_region+Sex	3.062	1502.000
ENSG00000141380	0.019	-0.087	0.052	-0.227	SS18	0.057	~amyloid_beta+(1|manifest)+cngeneson+pc_mito+brain_region+Sex	2.124	2284.000
ENSG00000068305	0.180	-0.087	0.079	-0.254	MEF2A	0.234	~amyloid_beta+(1|manifest)+cngeneson+pc_mito+brain_region+Sex	2.847	1638.000
ENSG00000103194	0.041	-0.087	0.017	-0.192	USP10	0.088	~amyloid_beta+(1|manifest)+cngeneson+pc_mito+brain_region+Sex	1.029	4080.000
ENSG00000047249	0.257	-0.088	0.111	-0.286	ATP6V1H	0.310	~amyloid_beta+(1|manifest)+cngeneson+pc_mito+brain_region+Sex	0.524	5298.000
ENSG00000108582	0.040	-0.088	0.061	-0.236	CPD	0.087	~amyloid_beta+(1|manifest)+cngeneson+pc_mito+brain_region+Sex	1.188	3732.000
ENSG00000196597	0.146	-0.088	0.063	-0.238	ZNF782	0.202	~amyloid_beta+(1|manifest)+cngeneson+pc_mito+brain_region+Sex	2.223	2178.000
ENSG00000029364	0.040	-0.088	0.014	-0.189	SLC39A9	0.088	~amyloid_beta+(1|manifest)+cngeneson+pc_mito+brain_region+Sex	1.035	4060.000
ENSG00000108671	0.051	-0.088	0.085	-0.261	PSMD11	0.101	~amyloid_beta+(1|manifest)+cngeneson+pc_mito+brain_region+Sex	1.329	3447.000
ENSG00000102081	0.002	-0.088	-0.034	-0.143	FMR1	0.015	~amyloid_beta+(1|manifest)+cngeneson+pc_mito+brain_region+Sex	0.286	5751.000
ENSG00000170390	0.319	-0.088	0.049	-0.226	DCLK2	0.371	~amyloid_beta+(1|manifest)+cngeneson+pc_mito+brain_region+Sex	4.367	928.000
ENSG00000153207	0.144	-0.088	0.167	-0.344	AHCTF1	0.200	~amyloid_beta+(1|manifest)+cngeneson+pc_mito+brain_region+Sex	2.444	1940.000
ENSG00000090686	0.002	-0.088	0.032	-0.209	USP48	0.015	~amyloid_beta+(1|manifest)+cngeneson+pc_mito+brain_region+Sex	0.990	4165.000
ENSG00000148925	0.025	-0.088	0.026	-0.202	BTBD10	0.067	~amyloid_beta+(1|manifest)+cngeneson+pc_mito+brain_region+Sex	1.190	3729.000
ENSG00000167257	0.004	-0.088	-0.012	-0.165	RNF214	0.024	~amyloid_beta+(1|manifest)+cngeneson+pc_mito+brain_region+Sex	0.911	4354.000
ENSG00000109016	0.268	-0.088	0.093	-0.269	DHRS7B	0.322	~amyloid_beta+(1|manifest)+cngeneson+pc_mito+brain_region+Sex	1.821	2667.000
ENSG00000128923	0.014	-0.088	0.019	-0.196	MINDY2	0.048	~amyloid_beta+(1|manifest)+cngeneson+pc_mito+brain_region+Sex	0.856	4481.000
ENSG00000136937	0.100	-0.088	0.026	-0.203	NCBP1	0.156	~amyloid_beta+(1|manifest)+cngeneson+pc_mito+brain_region+Sex	0.852	4494.000
ENSG00000167202	0.040	-0.089	0.054	-0.231	TBC1D2B	0.087	~amyloid_beta+(1|manifest)+cngeneson+pc_mito+brain_region+Sex	1.631	2936.000
ENSG00000182473	0.205	-0.089	0.125	-0.302	EXOC7	0.260	~amyloid_beta+(1|manifest)+cngeneson+pc_mito+brain_region+Sex	3.259	1389.000
ENSG00000285932	0.058	-0.089	0.082	-0.259	--------	0.109	~amyloid_beta+(1|manifest)+cngeneson+pc_mito+brain_region+Sex	0.962	4220.000
ENSG00000108443	0.040	-0.089	0.018	-0.196	RPS6KB1	0.087	~amyloid_beta+(1|manifest)+cngeneson+pc_mito+brain_region+Sex	1.018	4099.000
ENSG00000132849	0.287	-0.089	0.079	-0.257	PATJ	0.340	~amyloid_beta+(1|manifest)+cngeneson+pc_mito+brain_region+Sex	6.669	511.000
ENSG00000107077	0.751	-0.089	0.198	-0.375	KDM4C	0.776	~amyloid_beta+(1|manifest)+cngeneson+pc_mito+brain_region+Sex	4.416	910.000
ENSG00000157764	0.234	-0.089	0.035	-0.213	BRAF	0.288	~amyloid_beta+(1|manifest)+cngeneson+pc_mito+brain_region+Sex	3.022	1529.000
ENSG00000034693	0.009	-0.089	-0.005	-0.172	PEX3	0.037	~amyloid_beta+(1|manifest)+cngeneson+pc_mito+brain_region+Sex	0.728	4798.000
ENSG00000170324	0.228	-0.089	0.338	-0.516	FRMPD2	0.282	~amyloid_beta+(1|manifest)+cngeneson+pc_mito+brain_region+Sex	3.100	1482.000
ENSG00000151148	0.040	-0.089	0.017	-0.195	UBE3B	0.088	~amyloid_beta+(1|manifest)+cngeneson+pc_mito+brain_region+Sex	1.598	2986.000
ENSG00000100416	0.057	-0.089	0.031	-0.209	TRMU	0.108	~amyloid_beta+(1|manifest)+cngeneson+pc_mito+brain_region+Sex	1.126	3869.000
ENSG00000153208	0.885	-0.089	0.304	-0.482	MERTK	0.898	~amyloid_beta+(1|manifest)+cngeneson+pc_mito+brain_region+Sex	4.729	835.000
ENSG00000102226	0.095	-0.089	0.028	-0.206	USP11	0.150	~amyloid_beta+(1|manifest)+cngeneson+pc_mito+brain_region+Sex	1.700	2820.000
ENSG00000137274	0.017	-0.089	-0.014	-0.164	BPHL	0.054	~amyloid_beta+(1|manifest)+cngeneson+pc_mito+brain_region+Sex	0.534	5270.000
ENSG00000138286	0.030	-0.089	0.025	-0.204	--------	0.075	~amyloid_beta+(1|manifest)+cngeneson+pc_mito+brain_region+Sex	1.306	3493.000
ENSG00000109220	0.081	-0.089	0.052	-0.230	CHIC2	0.136	~amyloid_beta+(1|manifest)+cngeneson+pc_mito+brain_region+Sex	0.468	5425.000
ENSG00000120159	0.007	-0.090	-0.008	-0.172	CAAP1	0.031	~amyloid_beta+(1|manifest)+cngeneson+pc_mito+brain_region+Sex	0.909	4357.000
ENSG00000154359	0.104	-0.090	0.067	-0.246	LONRF1	0.159	~amyloid_beta+(1|manifest)+cngeneson+pc_mito+brain_region+Sex	2.097	2317.000
ENSG00000143079	0.037	-0.090	0.062	-0.242	--------	0.084	~amyloid_beta+(1|manifest)+cngeneson+pc_mito+brain_region+Sex	2.303	2101.000
ENSG00000168734	0.053	-0.090	0.031	-0.211	PKIG	0.103	~amyloid_beta+(1|manifest)+cngeneson+pc_mito+brain_region+Sex	0.517	5309.000
ENSG00000048649	0.132	-0.090	0.047	-0.227	RSF1	0.189	~amyloid_beta+(1|manifest)+cngeneson+pc_mito+brain_region+Sex	2.445	1939.000
ENSG00000109606	0.020	-0.090	0.015	-0.195	DHX15	0.060	~amyloid_beta+(1|manifest)+cngeneson+pc_mito+brain_region+Sex	1.074	3981.000
ENSG00000182185	0.679	-0.090	0.112	-0.292	RAD51B	0.710	~amyloid_beta+(1|manifest)+cngeneson+pc_mito+brain_region+Sex	0.862	4459.000
ENSG00000121064	0.065	-0.090	0.104	-0.284	SCPEP1	0.118	~amyloid_beta+(1|manifest)+cngeneson+pc_mito+brain_region+Sex	2.597	1815.000
ENSG00000141485	0.265	-0.090	0.127	-0.306	SLC13A5	0.319	~amyloid_beta+(1|manifest)+cngeneson+pc_mito+brain_region+Sex	2.477	1916.000
ENSG00000092421	0.149	-0.090	0.186	-0.366	SEMA6A	0.204	~amyloid_beta+(1|manifest)+cngeneson+pc_mito+brain_region+Sex	1.122	3883.000
ENSG00000103494	0.012	-0.090	0.002	-0.182	RPGRIP1L	0.044	~amyloid_beta+(1|manifest)+cngeneson+pc_mito+brain_region+Sex	0.443	5478.000
ENSG00000069974	0.135	-0.090	0.081	-0.262	RAB27A	0.191	~amyloid_beta+(1|manifest)+cngeneson+pc_mito+brain_region+Sex	2.400	1982.000
ENSG00000173258	0.049	-0.090	0.070	-0.250	ZNF483	0.098	~amyloid_beta+(1|manifest)+cngeneson+pc_mito+brain_region+Sex	1.190	3727.000
ENSG00000144040	0.265	-0.090	0.039	-0.219	SFXN5	0.319	~amyloid_beta+(1|manifest)+cngeneson+pc_mito+brain_region+Sex	6.092	581.000
ENSG00000071794	0.173	-0.091	0.058	-0.239	HLTF	0.228	~amyloid_beta+(1|manifest)+cngeneson+pc_mito+brain_region+Sex	1.205	3695.000
ENSG00000160584	0.034	-0.091	-0.021	-0.160	SIK3	0.080	~amyloid_beta+(1|manifest)+cngeneson+pc_mito+brain_region+Sex	2.322	2078.000
ENSG00000140382	0.091	-0.091	0.033	-0.214	HMG20A	0.146	~amyloid_beta+(1|manifest)+cngeneson+pc_mito+brain_region+Sex	1.103	3919.000
ENSG00000112237	0.018	-0.091	0.009	-0.191	CCNC	0.056	~amyloid_beta+(1|manifest)+cngeneson+pc_mito+brain_region+Sex	0.727	4802.000
ENSG00000125485	0.060	-0.091	0.004	-0.186	DDX31	0.111	~amyloid_beta+(1|manifest)+cngeneson+pc_mito+brain_region+Sex	0.807	4608.000
ENSG00000139218	0.206	-0.091	0.072	-0.253	SCAF11	0.260	~amyloid_beta+(1|manifest)+cngeneson+pc_mito+brain_region+Sex	2.962	1563.000
ENSG00000151623	0.663	-0.091	0.159	-0.340	NR3C2	0.695	~amyloid_beta+(1|manifest)+cngeneson+pc_mito+brain_region+Sex	5.747	635.000
ENSG00000158290	0.002	-0.091	-0.023	-0.159	CUL4B	0.015	~amyloid_beta+(1|manifest)+cngeneson+pc_mito+brain_region+Sex	0.717	4829.000
ENSG00000143401	0.002	-0.091	-0.035	-0.148	ANP32E	0.015	~amyloid_beta+(1|manifest)+cngeneson+pc_mito+brain_region+Sex	0.480	5401.000
ENSG00000102158	0.037	-0.092	-0.009	-0.174	MAGT1	0.083	~amyloid_beta+(1|manifest)+cngeneson+pc_mito+brain_region+Sex	0.443	5474.000
ENSG00000013619	0.268	-0.092	0.144	-0.327	MAMLD1	0.322	~amyloid_beta+(1|manifest)+cngeneson+pc_mito+brain_region+Sex	1.903	2550.000
ENSG00000119280	0.719	-0.092	0.347	-0.530	C1orf198	0.746	~amyloid_beta+(1|manifest)+cngeneson+pc_mito+brain_region+Sex	6.173	569.000
ENSG00000074603	0.185	-0.092	0.173	-0.357	DPP8	0.240	~amyloid_beta+(1|manifest)+cngeneson+pc_mito+brain_region+Sex	3.182	1435.000
ENSG00000106278	0.049	-0.092	0.024	-0.207	PTPRZ1	0.098	~amyloid_beta+(1|manifest)+cngeneson+pc_mito+brain_region+Sex	13.340	142.000
ENSG00000166747	0.024	-0.092	0.024	-0.208	AP1G1	0.065	~amyloid_beta+(1|manifest)+cngeneson+pc_mito+brain_region+Sex	1.327	3449.000
ENSG00000149809	0.140	-0.092	0.124	-0.307	TM7SF2	0.197	~amyloid_beta+(1|manifest)+cngeneson+pc_mito+brain_region+Sex	1.351	3410.000
ENSG00000140612	0.004	-0.092	-0.011	-0.173	SEC11A	0.024	~amyloid_beta+(1|manifest)+cngeneson+pc_mito+brain_region+Sex	0.917	4334.000
ENSG00000198707	0.064	-0.092	0.142	-0.326	CEP290	0.117	~amyloid_beta+(1|manifest)+cngeneson+pc_mito+brain_region+Sex	0.576	5172.000
ENSG00000186716	0.511	-0.092	0.210	-0.394	BCR	0.554	~amyloid_beta+(1|manifest)+cngeneson+pc_mito+brain_region+Sex	3.978	1047.000
ENSG00000078295	0.621	-0.092	0.094	-0.278	ADCY2	0.657	~amyloid_beta+(1|manifest)+cngeneson+pc_mito+brain_region+Sex	2.867	1624.000
ENSG00000057663	0.038	-0.092	0.015	-0.199	ATG5	0.085	~amyloid_beta+(1|manifest)+cngeneson+pc_mito+brain_region+Sex	0.911	4352.000
ENSG00000099385	0.228	-0.092	0.074	-0.258	BCL7C	0.282	~amyloid_beta+(1|manifest)+cngeneson+pc_mito+brain_region+Sex	1.472	3182.000
ENSG00000163811	0.016	-0.092	-0.008	-0.177	WDR43	0.053	~amyloid_beta+(1|manifest)+cngeneson+pc_mito+brain_region+Sex	1.596	2990.000
ENSG00000146872	0.041	-0.092	0.083	-0.268	TLK2	0.088	~amyloid_beta+(1|manifest)+cngeneson+pc_mito+brain_region+Sex	1.381	3357.000
ENSG00000168904	0.107	-0.092	0.038	-0.223	LRRC28	0.163	~amyloid_beta+(1|manifest)+cngeneson+pc_mito+brain_region+Sex	1.454	3221.000
ENSG00000133812	0.092	-0.093	-0.006	-0.179	SBF2	0.147	~amyloid_beta+(1|manifest)+cngeneson+pc_mito+brain_region+Sex	4.586	867.000
ENSG00000161551	0.085	-0.093	0.028	-0.213	ZNF577	0.140	~amyloid_beta+(1|manifest)+cngeneson+pc_mito+brain_region+Sex	0.939	4281.000
ENSG00000169410	0.807	-0.093	0.398	-0.583	PTPN9	0.826	~amyloid_beta+(1|manifest)+cngeneson+pc_mito+brain_region+Sex	14.729	106.000
ENSG00000104714	0.017	-0.093	0.004	-0.189	ERICH1	0.054	~amyloid_beta+(1|manifest)+cngeneson+pc_mito+brain_region+Sex	0.780	4666.000
ENSG00000138650	0.338	-0.093	0.136	-0.321	PCDH10	0.389	~amyloid_beta+(1|manifest)+cngeneson+pc_mito+brain_region+Sex	1.180	3745.000
ENSG00000132530	0.035	-0.093	0.234	-0.419	XAF1	0.081	~amyloid_beta+(1|manifest)+cngeneson+pc_mito+brain_region+Sex	1.940	2508.000
ENSG00000163116	0.775	-0.093	0.161	-0.346	STPG2	0.797	~amyloid_beta+(1|manifest)+cngeneson+pc_mito+brain_region+Sex	0.911	4350.000
ENSG00000145284	0.281	-0.093	0.031	-0.216	SCD5	0.334	~amyloid_beta+(1|manifest)+cngeneson+pc_mito+brain_region+Sex	0.458	5442.000
ENSG00000119650	0.123	-0.093	0.095	-0.281	IFT43	0.180	~amyloid_beta+(1|manifest)+cngeneson+pc_mito+brain_region+Sex	2.492	1910.000
ENSG00000163946	0.028	-0.093	0.022	-0.208	TASOR	0.072	~amyloid_beta+(1|manifest)+cngeneson+pc_mito+brain_region+Sex	0.771	4689.000
ENSG00000107758	0.118	-0.093	0.086	-0.272	PPP3CB	0.175	~amyloid_beta+(1|manifest)+cngeneson+pc_mito+brain_region+Sex	2.212	2189.000
ENSG00000006576	0.001	-0.093	0.010	-0.197	PHTF2	0.012	~amyloid_beta+(1|manifest)+cngeneson+pc_mito+brain_region+Sex	1.392	3335.000
ENSG00000198408	0.511	-0.093	0.138	-0.325	OGA	0.554	~amyloid_beta+(1|manifest)+cngeneson+pc_mito+brain_region+Sex	1.396	3331.000
ENSG00000176915	0.046	-0.093	0.044	-0.231	ANKLE2	0.095	~amyloid_beta+(1|manifest)+cngeneson+pc_mito+brain_region+Sex	2.046	2374.000
ENSG00000164975	0.001	-0.093	-0.017	-0.170	SNAPC3	0.008	~amyloid_beta+(1|manifest)+cngeneson+pc_mito+brain_region+Sex	0.687	4926.000
ENSG00000268163	0.090	-0.094	0.057	-0.244	--------	0.144	~amyloid_beta+(1|manifest)+cngeneson+pc_mito+brain_region+Sex	1.163	3781.000
ENSG00000100647	0.309	-0.094	0.094	-0.282	SUSD6	0.361	~amyloid_beta+(1|manifest)+cngeneson+pc_mito+brain_region+Sex	3.570	1219.000
ENSG00000170242	0.513	-0.094	0.085	-0.272	USP47	0.556	~amyloid_beta+(1|manifest)+cngeneson+pc_mito+brain_region+Sex	4.374	923.000
ENSG00000197912	0.194	-0.094	0.100	-0.288	SPG7	0.249	~amyloid_beta+(1|manifest)+cngeneson+pc_mito+brain_region+Sex	2.898	1604.000
ENSG00000157837	0.177	-0.094	0.047	-0.234	SPPL3	0.231	~amyloid_beta+(1|manifest)+cngeneson+pc_mito+brain_region+Sex	1.038	4049.000
ENSG00000137522	0.028	-0.094	0.017	-0.204	RNF121	0.071	~amyloid_beta+(1|manifest)+cngeneson+pc_mito+brain_region+Sex	1.412	3304.000
ENSG00000100767	0.693	-0.094	0.490	-0.678	PAPLN	0.723	~amyloid_beta+(1|manifest)+cngeneson+pc_mito+brain_region+Sex	13.529	136.000
ENSG00000171033	0.753	-0.094	0.238	-0.426	PKIA	0.777	~amyloid_beta+(1|manifest)+cngeneson+pc_mito+brain_region+Sex	3.564	1222.000
ENSG00000105220	0.698	-0.094	0.270	-0.458	GPI	0.727	~amyloid_beta+(1|manifest)+cngeneson+pc_mito+brain_region+Sex	3.800	1121.000
ENSG00000142197	0.008	-0.094	0.002	-0.190	DOP1B	0.033	~amyloid_beta+(1|manifest)+cngeneson+pc_mito+brain_region+Sex	0.984	4180.000
ENSG00000183354	0.396	-0.094	0.127	-0.316	KIAA2026	0.444	~amyloid_beta+(1|manifest)+cngeneson+pc_mito+brain_region+Sex	3.211	1418.000
ENSG00000138668	0.029	-0.095	-0.001	-0.188	HNRNPD	0.073	~amyloid_beta+(1|manifest)+cngeneson+pc_mito+brain_region+Sex	2.117	2291.000
ENSG00000064313	0.021	-0.095	0.001	-0.191	TAF2	0.060	~amyloid_beta+(1|manifest)+cngeneson+pc_mito+brain_region+Sex	1.501	3137.000
ENSG00000118246	0.002	-0.095	-0.036	-0.153	FASTKD2	0.015	~amyloid_beta+(1|manifest)+cngeneson+pc_mito+brain_region+Sex	0.593	5147.000
ENSG00000116497	0.052	-0.095	0.038	-0.227	S100PBP	0.102	~amyloid_beta+(1|manifest)+cngeneson+pc_mito+brain_region+Sex	1.822	2665.000
ENSG00000197265	0.003	-0.095	-0.002	-0.188	GTF2E2	0.018	~amyloid_beta+(1|manifest)+cngeneson+pc_mito+brain_region+Sex	1.141	3834.000
ENSG00000150627	0.555	-0.095	0.296	-0.486	WDR17	0.595	~amyloid_beta+(1|manifest)+cngeneson+pc_mito+brain_region+Sex	2.928	1590.000
ENSG00000112367	0.002	-0.095	-0.023	-0.168	FIG4	0.017	~amyloid_beta+(1|manifest)+cngeneson+pc_mito+brain_region+Sex	0.632	5053.000
ENSG00000064933	0.070	-0.095	0.037	-0.228	PMS1	0.124	~amyloid_beta+(1|manifest)+cngeneson+pc_mito+brain_region+Sex	2.207	2197.000
ENSG00000141540	0.119	-0.095	0.208	-0.398	TTYH2	0.175	~amyloid_beta+(1|manifest)+cngeneson+pc_mito+brain_region+Sex	1.343	3422.000
ENSG00000120697	0.001	-0.095	-0.047	-0.144	ALG5	0.012	~amyloid_beta+(1|manifest)+cngeneson+pc_mito+brain_region+Sex	0.561	5205.000
ENSG00000013503	0.001	-0.096	-0.037	-0.154	POLR3B	0.009	~amyloid_beta+(1|manifest)+cngeneson+pc_mito+brain_region+Sex	0.389	5581.000
ENSG00000198795	0.629	-0.096	0.102	-0.293	ZNF521	0.664	~amyloid_beta+(1|manifest)+cngeneson+pc_mito+brain_region+Sex	2.282	2122.000
ENSG00000204406	0.018	-0.096	-0.026	-0.166	MBD5	0.056	~amyloid_beta+(1|manifest)+cngeneson+pc_mito+brain_region+Sex	2.753	1700.000
ENSG00000198836	0.058	-0.096	0.059	-0.251	OPA1	0.109	~amyloid_beta+(1|manifest)+cngeneson+pc_mito+brain_region+Sex	1.354	3402.000
ENSG00000110768	0.007	-0.096	-0.017	-0.175	GTF2H1	0.032	~amyloid_beta+(1|manifest)+cngeneson+pc_mito+brain_region+Sex	0.834	4534.000
ENSG00000112685	0.020	-0.096	0.102	-0.294	EXOC2	0.059	~amyloid_beta+(1|manifest)+cngeneson+pc_mito+brain_region+Sex	2.105	2308.000
ENSG00000187957	0.843	-0.096	0.353	-0.545	DNER	0.859	~amyloid_beta+(1|manifest)+cngeneson+pc_mito+brain_region+Sex	10.839	230.000
ENSG00000166783	0.018	-0.096	0.009	-0.201	MARF1	0.055	~amyloid_beta+(1|manifest)+cngeneson+pc_mito+brain_region+Sex	1.216	3672.000
ENSG00000145868	0.001	-0.096	-0.026	-0.166	FBXO38	0.012	~amyloid_beta+(1|manifest)+cngeneson+pc_mito+brain_region+Sex	0.598	5135.000
ENSG00000114450	0.196	-0.096	0.018	-0.210	GNB4	0.250	~amyloid_beta+(1|manifest)+cngeneson+pc_mito+brain_region+Sex	1.064	4002.000
ENSG00000169621	0.222	-0.096	0.057	-0.250	APLF	0.277	~amyloid_beta+(1|manifest)+cngeneson+pc_mito+brain_region+Sex	2.688	1736.000
ENSG00000188994	0.370	-0.097	0.070	-0.263	ZNF292	0.420	~amyloid_beta+(1|manifest)+cngeneson+pc_mito+brain_region+Sex	3.206	1422.000
ENSG00000135317	0.013	-0.097	0.019	-0.213	SNX14	0.046	~amyloid_beta+(1|manifest)+cngeneson+pc_mito+brain_region+Sex	0.945	4263.000
ENSG00000162086	0.059	-0.097	0.048	-0.242	ZNF75A	0.110	~amyloid_beta+(1|manifest)+cngeneson+pc_mito+brain_region+Sex	1.103	3923.000
ENSG00000099219	0.042	-0.097	0.009	-0.203	ERMP1	0.089	~amyloid_beta+(1|manifest)+cngeneson+pc_mito+brain_region+Sex	1.184	3737.000
ENSG00000100360	0.159	-0.097	0.107	-0.300	IFT27	0.215	~amyloid_beta+(1|manifest)+cngeneson+pc_mito+brain_region+Sex	3.756	1139.000
ENSG00000186566	0.152	-0.097	0.025	-0.219	GPATCH8	0.207	~amyloid_beta+(1|manifest)+cngeneson+pc_mito+brain_region+Sex	1.277	3551.000
ENSG00000129472	0.003	-0.097	-0.028	-0.166	RAB2B	0.019	~amyloid_beta+(1|manifest)+cngeneson+pc_mito+brain_region+Sex	0.877	4423.000
ENSG00000186815	0.955	-0.097	0.582	-0.776	TPCN1	0.960	~amyloid_beta+(1|manifest)+cngeneson+pc_mito+brain_region+Sex	22.335	39.000
ENSG00000116729	0.635	-0.097	0.111	-0.305	WLS	0.669	~amyloid_beta+(1|manifest)+cngeneson+pc_mito+brain_region+Sex	6.229	560.000
ENSG00000066427	0.144	-0.097	0.152	-0.346	ATXN3	0.200	~amyloid_beta+(1|manifest)+cngeneson+pc_mito+brain_region+Sex	2.314	2087.000
ENSG00000074211	0.615	-0.097	0.312	-0.507	PPP2R2C	0.651	~amyloid_beta+(1|manifest)+cngeneson+pc_mito+brain_region+Sex	5.173	735.000
ENSG00000168300	0.266	-0.097	0.103	-0.298	PCMTD1	0.319	~amyloid_beta+(1|manifest)+cngeneson+pc_mito+brain_region+Sex	1.318	3465.000
ENSG00000150403	0.006	-0.097	0.000	-0.195	TMCO3	0.028	~amyloid_beta+(1|manifest)+cngeneson+pc_mito+brain_region+Sex	0.986	4177.000
ENSG00000115368	0.009	-0.097	-0.024	-0.171	WDR75	0.037	~amyloid_beta+(1|manifest)+cngeneson+pc_mito+brain_region+Sex	0.925	4318.000
ENSG00000033178	0.039	-0.098	0.063	-0.258	UBA6	0.086	~amyloid_beta+(1|manifest)+cngeneson+pc_mito+brain_region+Sex	1.850	2625.000
ENSG00000032389	0.004	-0.098	-0.022	-0.174	EIPR1	0.023	~amyloid_beta+(1|manifest)+cngeneson+pc_mito+brain_region+Sex	0.354	5644.000
ENSG00000157107	0.029	-0.098	0.159	-0.354	FCHO2	0.073	~amyloid_beta+(1|manifest)+cngeneson+pc_mito+brain_region+Sex	0.566	5193.000
ENSG00000116171	0.073	-0.098	0.068	-0.263	SCP2	0.127	~amyloid_beta+(1|manifest)+cngeneson+pc_mito+brain_region+Sex	1.306	3490.000
ENSG00000125841	0.153	-0.098	0.076	-0.272	NRSN2	0.208	~amyloid_beta+(1|manifest)+cngeneson+pc_mito+brain_region+Sex	2.025	2403.000
ENSG00000171723	0.039	-0.098	-0.019	-0.177	GPHN	0.086	~amyloid_beta+(1|manifest)+cngeneson+pc_mito+brain_region+Sex	3.159	1449.000
ENSG00000067798	0.443	-0.098	0.051	-0.248	NAV3	0.488	~amyloid_beta+(1|manifest)+cngeneson+pc_mito+brain_region+Sex	11.336	204.000
ENSG00000099917	0.237	-0.098	0.147	-0.344	MED15	0.291	~amyloid_beta+(1|manifest)+cngeneson+pc_mito+brain_region+Sex	3.801	1120.000
ENSG00000118873	0.032	-0.098	0.056	-0.253	RAB3GAP2	0.076	~amyloid_beta+(1|manifest)+cngeneson+pc_mito+brain_region+Sex	2.405	1978.000
ENSG00000023516	0.098	-0.098	0.030	-0.227	AKAP11	0.154	~amyloid_beta+(1|manifest)+cngeneson+pc_mito+brain_region+Sex	1.243	3613.000
ENSG00000112584	0.216	-0.099	0.121	-0.318	FAM120B	0.271	~amyloid_beta+(1|manifest)+cngeneson+pc_mito+brain_region+Sex	2.338	2062.000
ENSG00000171109	0.000	-0.099	-0.065	-0.133	MFN1	0.001	~amyloid_beta+(1|manifest)+cngeneson+pc_mito+brain_region+Sex	1.169	3768.000
ENSG00000092140	0.010	-0.099	-0.017	-0.180	G2E3	0.041	~amyloid_beta+(1|manifest)+cngeneson+pc_mito+brain_region+Sex	0.237	5829.000
ENSG00000175265	0.576	-0.099	0.372	-0.569	GOLGA8A	0.614	~amyloid_beta+(1|manifest)+cngeneson+pc_mito+brain_region+Sex	8.146	366.000
ENSG00000163848	0.578	-0.099	0.093	-0.290	ZNF148	0.616	~amyloid_beta+(1|manifest)+cngeneson+pc_mito+brain_region+Sex	2.000	2431.000
ENSG00000186132	0.010	-0.099	-0.023	-0.174	C2orf76	0.039	~amyloid_beta+(1|manifest)+cngeneson+pc_mito+brain_region+Sex	0.896	4384.000
ENSG00000112981	0.021	-0.099	0.135	-0.333	NME5	0.061	~amyloid_beta+(1|manifest)+cngeneson+pc_mito+brain_region+Sex	1.466	3195.000
ENSG00000141446	0.026	-0.099	0.015	-0.213	ESCO1	0.068	~amyloid_beta+(1|manifest)+cngeneson+pc_mito+brain_region+Sex	1.281	3542.000
ENSG00000240038	0.300	-0.099	0.236	-0.434	AMY2B	0.353	~amyloid_beta+(1|manifest)+cngeneson+pc_mito+brain_region+Sex	4.131	1006.000
ENSG00000182923	0.007	-0.099	-0.010	-0.188	CEP63	0.033	~amyloid_beta+(1|manifest)+cngeneson+pc_mito+brain_region+Sex	1.203	3698.000
ENSG00000104324	0.626	-0.099	0.112	-0.310	CPQ	0.661	~amyloid_beta+(1|manifest)+cngeneson+pc_mito+brain_region+Sex	1.978	2460.000
ENSG00000204138	0.174	-0.099	0.050	-0.249	PHACTR4	0.229	~amyloid_beta+(1|manifest)+cngeneson+pc_mito+brain_region+Sex	1.852	2621.000
ENSG00000196632	0.151	-0.099	0.041	-0.240	WNK3	0.207	~amyloid_beta+(1|manifest)+cngeneson+pc_mito+brain_region+Sex	1.462	3203.000
ENSG00000151413	0.527	-0.100	0.140	-0.339	NUBPL	0.569	~amyloid_beta+(1|manifest)+cngeneson+pc_mito+brain_region+Sex	0.656	4984.000
ENSG00000084072	0.144	-0.100	0.071	-0.271	PPIE	0.200	~amyloid_beta+(1|manifest)+cngeneson+pc_mito+brain_region+Sex	2.985	1549.000
ENSG00000138709	0.568	-0.100	0.184	-0.383	LARP1B	0.607	~amyloid_beta+(1|manifest)+cngeneson+pc_mito+brain_region+Sex	4.832	812.000
ENSG00000110013	0.016	-0.100	-0.026	-0.174	SIAE	0.053	~amyloid_beta+(1|manifest)+cngeneson+pc_mito+brain_region+Sex	1.150	3813.000
ENSG00000069943	0.002	-0.100	-0.016	-0.183	PIGB	0.017	~amyloid_beta+(1|manifest)+cngeneson+pc_mito+brain_region+Sex	0.629	5063.000
ENSG00000167380	0.012	-0.100	-0.014	-0.185	ZNF226	0.045	~amyloid_beta+(1|manifest)+cngeneson+pc_mito+brain_region+Sex	0.972	4201.000
ENSG00000120725	0.094	-0.100	0.024	-0.224	SIL1	0.149	~amyloid_beta+(1|manifest)+cngeneson+pc_mito+brain_region+Sex	1.054	4017.000
ENSG00000109099	0.510	-0.100	0.235	-0.435	PMP22	0.553	~amyloid_beta+(1|manifest)+cngeneson+pc_mito+brain_region+Sex	2.481	1913.000
ENSG00000147050	0.554	-0.100	0.088	-0.288	KDM6A	0.594	~amyloid_beta+(1|manifest)+cngeneson+pc_mito+brain_region+Sex	2.151	2259.000
ENSG00000153922	0.225	-0.100	0.038	-0.238	CHD1	0.280	~amyloid_beta+(1|manifest)+cngeneson+pc_mito+brain_region+Sex	1.446	3237.000
ENSG00000135945	0.047	-0.100	0.027	-0.227	REV1	0.096	~amyloid_beta+(1|manifest)+cngeneson+pc_mito+brain_region+Sex	1.338	3434.000
ENSG00000013364	0.040	-0.100	0.077	-0.278	MVP	0.087	~amyloid_beta+(1|manifest)+cngeneson+pc_mito+brain_region+Sex	1.964	2471.000
ENSG00000075945	0.030	-0.100	0.061	-0.261	KIFAP3	0.074	~amyloid_beta+(1|manifest)+cngeneson+pc_mito+brain_region+Sex	0.649	5005.000
ENSG00000114742	0.038	-0.100	0.045	-0.246	WDR48	0.085	~amyloid_beta+(1|manifest)+cngeneson+pc_mito+brain_region+Sex	1.668	2871.000
ENSG00000085832	0.262	-0.100	0.030	-0.230	EPS15	0.316	~amyloid_beta+(1|manifest)+cngeneson+pc_mito+brain_region+Sex	1.884	2584.000
ENSG00000128908	0.036	-0.100	0.042	-0.243	INO80	0.082	~amyloid_beta+(1|manifest)+cngeneson+pc_mito+brain_region+Sex	1.864	2609.000
ENSG00000131981	0.382	-0.100	0.356	-0.557	LGALS3	0.432	~amyloid_beta+(1|manifest)+cngeneson+pc_mito+brain_region+Sex	6.371	540.000
ENSG00000107862	0.011	-0.100	0.032	-0.233	GBF1	0.043	~amyloid_beta+(1|manifest)+cngeneson+pc_mito+brain_region+Sex	1.822	2666.000
ENSG00000204116	0.402	-0.100	0.087	-0.287	CHIC1	0.449	~amyloid_beta+(1|manifest)+cngeneson+pc_mito+brain_region+Sex	1.626	2945.000
ENSG00000136021	0.076	-0.100	0.041	-0.242	SCYL2	0.130	~amyloid_beta+(1|manifest)+cngeneson+pc_mito+brain_region+Sex	2.176	2229.000
ENSG00000086544	0.833	-0.101	0.334	-0.535	ITPKC	0.850	~amyloid_beta+(1|manifest)+cngeneson+pc_mito+brain_region+Sex	9.787	280.000
ENSG00000172461	0.221	-0.101	0.029	-0.230	FUT9	0.276	~amyloid_beta+(1|manifest)+cngeneson+pc_mito+brain_region+Sex	3.825	1112.000
ENSG00000157426	0.116	-0.101	0.090	-0.292	AASDH	0.172	~amyloid_beta+(1|manifest)+cngeneson+pc_mito+brain_region+Sex	2.052	2368.000
ENSG00000188419	0.056	-0.101	0.052	-0.254	CHM	0.107	~amyloid_beta+(1|manifest)+cngeneson+pc_mito+brain_region+Sex	1.851	2622.000
ENSG00000181192	0.116	-0.101	0.064	-0.265	DHTKD1	0.172	~amyloid_beta+(1|manifest)+cngeneson+pc_mito+brain_region+Sex	1.262	3576.000
ENSG00000005469	0.000	-0.101	-0.051	-0.151	CROT	0.004	~amyloid_beta+(1|manifest)+cngeneson+pc_mito+brain_region+Sex	0.313	5706.000
ENSG00000101746	0.723	-0.101	0.164	-0.366	NOL4	0.750	~amyloid_beta+(1|manifest)+cngeneson+pc_mito+brain_region+Sex	3.159	1448.000
ENSG00000185946	0.344	-0.101	0.181	-0.383	RNPC3	0.395	~amyloid_beta+(1|manifest)+cngeneson+pc_mito+brain_region+Sex	6.803	491.000
ENSG00000181982	0.011	-0.101	-0.025	-0.177	CCDC149	0.041	~amyloid_beta+(1|manifest)+cngeneson+pc_mito+brain_region+Sex	0.700	4887.000
ENSG00000108389	0.035	-0.101	0.003	-0.205	MTMR4	0.081	~amyloid_beta+(1|manifest)+cngeneson+pc_mito+brain_region+Sex	1.341	3428.000
ENSG00000111605	0.064	-0.101	0.080	-0.282	CPSF6	0.117	~amyloid_beta+(1|manifest)+cngeneson+pc_mito+brain_region+Sex	2.610	1805.000
ENSG00000085511	0.005	-0.101	-0.007	-0.196	MAP3K4	0.026	~amyloid_beta+(1|manifest)+cngeneson+pc_mito+brain_region+Sex	1.397	3327.000
ENSG00000174227	0.040	-0.101	0.026	-0.228	PIGG	0.087	~amyloid_beta+(1|manifest)+cngeneson+pc_mito+brain_region+Sex	1.399	3322.000
ENSG00000113048	0.020	-0.101	-0.006	-0.197	MRPS27	0.058	~amyloid_beta+(1|manifest)+cngeneson+pc_mito+brain_region+Sex	1.007	4125.000
ENSG00000196396	0.030	-0.101	-0.009	-0.194	PTPN1	0.075	~amyloid_beta+(1|manifest)+cngeneson+pc_mito+brain_region+Sex	0.987	4174.000
ENSG00000166206	0.026	-0.101	-0.017	-0.186	GABRB3	0.068	~amyloid_beta+(1|manifest)+cngeneson+pc_mito+brain_region+Sex	0.768	4698.000
ENSG00000114520	0.004	-0.101	-0.033	-0.170	SNX4	0.024	~amyloid_beta+(1|manifest)+cngeneson+pc_mito+brain_region+Sex	1.050	4023.000
ENSG00000121310	0.481	-0.101	0.167	-0.369	ECHDC2	0.525	~amyloid_beta+(1|manifest)+cngeneson+pc_mito+brain_region+Sex	13.067	148.000
ENSG00000115947	0.026	-0.101	0.000	-0.203	ORC4	0.068	~amyloid_beta+(1|manifest)+cngeneson+pc_mito+brain_region+Sex	0.452	5450.000
ENSG00000136715	0.153	-0.101	0.050	-0.253	SAP130	0.208	~amyloid_beta+(1|manifest)+cngeneson+pc_mito+brain_region+Sex	1.652	2901.000
ENSG00000083312	0.077	-0.101	0.043	-0.246	TNPO1	0.132	~amyloid_beta+(1|manifest)+cngeneson+pc_mito+brain_region+Sex	1.865	2608.000
ENSG00000035681	0.038	-0.102	0.116	-0.319	NSMAF	0.085	~amyloid_beta+(1|manifest)+cngeneson+pc_mito+brain_region+Sex	3.384	1328.000
ENSG00000143149	0.004	-0.102	-0.024	-0.179	ALDH9A1	0.023	~amyloid_beta+(1|manifest)+cngeneson+pc_mito+brain_region+Sex	0.105	5950.000
ENSG00000124164	0.000	-0.102	-0.048	-0.155	VAPB	0.006	~amyloid_beta+(1|manifest)+cngeneson+pc_mito+brain_region+Sex	0.363	5629.000
ENSG00000111052	0.679	-0.102	0.171	-0.374	LIN7A	0.710	~amyloid_beta+(1|manifest)+cngeneson+pc_mito+brain_region+Sex	2.367	2031.000
ENSG00000155189	0.057	-0.102	0.003	-0.207	AGPAT5	0.108	~amyloid_beta+(1|manifest)+cngeneson+pc_mito+brain_region+Sex	1.013	4108.000
ENSG00000115137	0.001	-0.102	-0.051	-0.153	DNAJC27	0.007	~amyloid_beta+(1|manifest)+cngeneson+pc_mito+brain_region+Sex	0.720	4824.000
ENSG00000232593	0.787	-0.102	0.190	-0.395	KANTR	0.808	~amyloid_beta+(1|manifest)+cngeneson+pc_mito+brain_region+Sex	6.545	521.000
ENSG00000104635	0.956	-0.102	0.566	-0.770	SLC39A14	0.960	~amyloid_beta+(1|manifest)+cngeneson+pc_mito+brain_region+Sex	10.603	242.000
ENSG00000162694	0.041	-0.102	0.016	-0.221	EXTL2	0.089	~amyloid_beta+(1|manifest)+cngeneson+pc_mito+brain_region+Sex	1.338	3432.000
ENSG00000177025	0.313	-0.102	0.211	-0.416	C19orf18	0.366	~amyloid_beta+(1|manifest)+cngeneson+pc_mito+brain_region+Sex	5.770	631.000
ENSG00000197386	0.035	-0.102	0.042	-0.247	HTT	0.081	~amyloid_beta+(1|manifest)+cngeneson+pc_mito+brain_region+Sex	2.634	1783.000
ENSG00000141564	0.052	-0.102	0.077	-0.282	RPTOR	0.102	~amyloid_beta+(1|manifest)+cngeneson+pc_mito+brain_region+Sex	1.380	3358.000
ENSG00000092094	0.076	-0.102	0.048	-0.253	OSGEP	0.130	~amyloid_beta+(1|manifest)+cngeneson+pc_mito+brain_region+Sex	2.469	1923.000
ENSG00000127928	0.124	-0.102	0.042	-0.247	GNGT1	0.181	~amyloid_beta+(1|manifest)+cngeneson+pc_mito+brain_region+Sex	0.957	4233.000
ENSG00000131381	0.008	-0.103	-0.020	-0.185	RBSN	0.035	~amyloid_beta+(1|manifest)+cngeneson+pc_mito+brain_region+Sex	0.933	4296.000
ENSG00000054611	0.064	-0.103	0.000	-0.206	TBC1D22A	0.117	~amyloid_beta+(1|manifest)+cngeneson+pc_mito+brain_region+Sex	0.865	4454.000
ENSG00000180287	0.164	-0.103	0.128	-0.334	PLD5	0.219	~amyloid_beta+(1|manifest)+cngeneson+pc_mito+brain_region+Sex	8.587	341.000
ENSG00000140577	0.019	-0.103	0.071	-0.277	CRTC3	0.057	~amyloid_beta+(1|manifest)+cngeneson+pc_mito+brain_region+Sex	2.100	2313.000
ENSG00000081320	0.351	-0.103	0.187	-0.393	STK17B	0.402	~amyloid_beta+(1|manifest)+cngeneson+pc_mito+brain_region+Sex	1.174	3762.000
ENSG00000189221	0.120	-0.103	0.003	-0.209	MAOA	0.176	~amyloid_beta+(1|manifest)+cngeneson+pc_mito+brain_region+Sex	0.895	4387.000
ENSG00000163257	0.009	-0.103	-0.029	-0.178	DCAF16	0.038	~amyloid_beta+(1|manifest)+cngeneson+pc_mito+brain_region+Sex	0.590	5153.000
ENSG00000197713	0.004	-0.103	-0.032	-0.175	RPE	0.023	~amyloid_beta+(1|manifest)+cngeneson+pc_mito+brain_region+Sex	0.949	4254.000
ENSG00000164941	0.010	-0.103	-0.019	-0.188	INTS8	0.040	~amyloid_beta+(1|manifest)+cngeneson+pc_mito+brain_region+Sex	1.044	4038.000
ENSG00000092964	0.429	-0.103	0.055	-0.262	DPYSL2	0.475	~amyloid_beta+(1|manifest)+cngeneson+pc_mito+brain_region+Sex	7.802	390.000
ENSG00000172164	0.753	-0.103	0.378	-0.585	SNTB1	0.777	~amyloid_beta+(1|manifest)+cngeneson+pc_mito+brain_region+Sex	2.499	1906.000
ENSG00000180357	0.466	-0.103	0.115	-0.322	ZNF609	0.511	~amyloid_beta+(1|manifest)+cngeneson+pc_mito+brain_region+Sex	2.629	1790.000
ENSG00000171566	0.037	-0.104	-0.004	-0.203	PLRG1	0.083	~amyloid_beta+(1|manifest)+cngeneson+pc_mito+brain_region+Sex	0.650	4998.000
ENSG00000184985	0.638	-0.104	0.120	-0.327	SORCS2	0.672	~amyloid_beta+(1|manifest)+cngeneson+pc_mito+brain_region+Sex	4.851	808.000
ENSG00000166575	0.531	-0.104	0.099	-0.307	TMEM135	0.573	~amyloid_beta+(1|manifest)+cngeneson+pc_mito+brain_region+Sex	3.808	1116.000
ENSG00000149289	0.662	-0.104	0.131	-0.339	ZC3H12C	0.694	~amyloid_beta+(1|manifest)+cngeneson+pc_mito+brain_region+Sex	3.197	1428.000
ENSG00000172878	0.017	-0.104	-0.011	-0.197	METAP1D	0.054	~amyloid_beta+(1|manifest)+cngeneson+pc_mito+brain_region+Sex	0.872	4433.000
ENSG00000136169	0.036	-0.104	0.028	-0.235	SETDB2	0.082	~amyloid_beta+(1|manifest)+cngeneson+pc_mito+brain_region+Sex	1.378	3360.000
ENSG00000103489	0.399	-0.104	0.162	-0.370	XYLT1	0.447	~amyloid_beta+(1|manifest)+cngeneson+pc_mito+brain_region+Sex	0.423	5521.000
ENSG00000165675	0.124	-0.104	-0.013	-0.195	ENOX2	0.180	~amyloid_beta+(1|manifest)+cngeneson+pc_mito+brain_region+Sex	1.120	3887.000
ENSG00000140464	0.139	-0.104	0.064	-0.272	PML	0.195	~amyloid_beta+(1|manifest)+cngeneson+pc_mito+brain_region+Sex	2.392	1995.000
ENSG00000050030	0.575	-0.104	0.102	-0.310	NEXMIF	0.613	~amyloid_beta+(1|manifest)+cngeneson+pc_mito+brain_region+Sex	1.492	3154.000
ENSG00000116761	0.028	-0.104	-0.010	-0.198	CTH	0.071	~amyloid_beta+(1|manifest)+cngeneson+pc_mito+brain_region+Sex	0.940	4278.000
ENSG00000120688	0.028	-0.105	0.067	-0.276	WBP4	0.071	~amyloid_beta+(1|manifest)+cngeneson+pc_mito+brain_region+Sex	2.262	2141.000
ENSG00000100478	0.002	-0.105	-0.041	-0.168	AP4S1	0.016	~amyloid_beta+(1|manifest)+cngeneson+pc_mito+brain_region+Sex	0.190	5890.000
ENSG00000101557	0.035	-0.105	0.007	-0.217	USP14	0.081	~amyloid_beta+(1|manifest)+cngeneson+pc_mito+brain_region+Sex	1.755	2744.000
ENSG00000168538	0.007	-0.105	-0.027	-0.183	TRAPPC11	0.032	~amyloid_beta+(1|manifest)+cngeneson+pc_mito+brain_region+Sex	0.707	4858.000
ENSG00000147202	0.107	-0.105	0.085	-0.295	DIAPH2	0.162	~amyloid_beta+(1|manifest)+cngeneson+pc_mito+brain_region+Sex	0.403	5560.000
ENSG00000110911	0.142	-0.105	0.089	-0.300	SLC11A2	0.199	~amyloid_beta+(1|manifest)+cngeneson+pc_mito+brain_region+Sex	2.943	1581.000
ENSG00000169288	0.002	-0.105	-0.038	-0.173	MRPL1	0.017	~amyloid_beta+(1|manifest)+cngeneson+pc_mito+brain_region+Sex	0.714	4835.000
ENSG00000128699	0.111	-0.105	0.048	-0.258	ORMDL1	0.167	~amyloid_beta+(1|manifest)+cngeneson+pc_mito+brain_region+Sex	1.469	3186.000
ENSG00000101019	0.014	-0.105	-0.025	-0.186	UQCC1	0.048	~amyloid_beta+(1|manifest)+cngeneson+pc_mito+brain_region+Sex	0.995	4151.000
ENSG00000035928	0.007	-0.106	-0.010	-0.201	RFC1	0.033	~amyloid_beta+(1|manifest)+cngeneson+pc_mito+brain_region+Sex	1.101	3927.000
ENSG00000138760	0.050	-0.106	0.032	-0.243	SCARB2	0.100	~amyloid_beta+(1|manifest)+cngeneson+pc_mito+brain_region+Sex	1.104	3917.000
ENSG00000188895	0.173	-0.106	0.107	-0.319	MSL1	0.228	~amyloid_beta+(1|manifest)+cngeneson+pc_mito+brain_region+Sex	2.794	1673.000
ENSG00000086758	0.131	-0.106	0.055	-0.266	HUWE1	0.188	~amyloid_beta+(1|manifest)+cngeneson+pc_mito+brain_region+Sex	3.250	1396.000
ENSG00000051382	0.118	-0.106	0.087	-0.298	PIK3CB	0.175	~amyloid_beta+(1|manifest)+cngeneson+pc_mito+brain_region+Sex	2.620	1799.000
ENSG00000047410	0.027	-0.106	0.028	-0.240	TPR	0.071	~amyloid_beta+(1|manifest)+cngeneson+pc_mito+brain_region+Sex	0.732	4787.000
ENSG00000102189	0.235	-0.106	0.116	-0.328	EEA1	0.289	~amyloid_beta+(1|manifest)+cngeneson+pc_mito+brain_region+Sex	4.080	1020.000
ENSG00000165494	0.029	-0.106	-0.002	-0.211	PCF11	0.072	~amyloid_beta+(1|manifest)+cngeneson+pc_mito+brain_region+Sex	0.576	5171.000
ENSG00000066651	0.338	-0.106	0.101	-0.314	TRMT11	0.390	~amyloid_beta+(1|manifest)+cngeneson+pc_mito+brain_region+Sex	2.112	2296.000
ENSG00000275835	0.040	-0.106	0.027	-0.240	TUBGCP5	0.088	~amyloid_beta+(1|manifest)+cngeneson+pc_mito+brain_region+Sex	1.479	3177.000
ENSG00000110693	0.111	-0.106	-0.007	-0.206	SOX6	0.167	~amyloid_beta+(1|manifest)+cngeneson+pc_mito+brain_region+Sex	1.870	2601.000
ENSG00000166887	0.032	-0.106	-0.019	-0.194	VPS39	0.077	~amyloid_beta+(1|manifest)+cngeneson+pc_mito+brain_region+Sex	1.164	3778.000
ENSG00000136143	0.032	-0.107	-0.004	-0.209	SUCLA2	0.076	~amyloid_beta+(1|manifest)+cngeneson+pc_mito+brain_region+Sex	1.058	4006.000
ENSG00000164010	0.126	-0.107	0.060	-0.273	ERMAP	0.182	~amyloid_beta+(1|manifest)+cngeneson+pc_mito+brain_region+Sex	2.434	1951.000
ENSG00000189180	0.097	-0.107	0.068	-0.281	ZNF33A	0.152	~amyloid_beta+(1|manifest)+cngeneson+pc_mito+brain_region+Sex	1.981	2457.000
ENSG00000092148	0.094	-0.107	0.023	-0.237	HECTD1	0.150	~amyloid_beta+(1|manifest)+cngeneson+pc_mito+brain_region+Sex	2.811	1658.000
ENSG00000134152	0.007	-0.107	0.007	-0.220	KATNBL1	0.033	~amyloid_beta+(1|manifest)+cngeneson+pc_mito+brain_region+Sex	2.248	2157.000
ENSG00000251247	0.500	-0.107	0.206	-0.420	ZNF345	0.544	~amyloid_beta+(1|manifest)+cngeneson+pc_mito+brain_region+Sex	6.283	552.000
ENSG00000108669	0.166	-0.107	0.053	-0.267	CYTH1	0.222	~amyloid_beta+(1|manifest)+cngeneson+pc_mito+brain_region+Sex	1.518	3108.000
ENSG00000151151	0.002	-0.107	-0.037	-0.177	IPMK	0.017	~amyloid_beta+(1|manifest)+cngeneson+pc_mito+brain_region+Sex	0.749	4743.000
ENSG00000185737	0.053	-0.107	-0.009	-0.205	NRG3	0.103	~amyloid_beta+(1|manifest)+cngeneson+pc_mito+brain_region+Sex	10.522	245.000
ENSG00000198879	0.242	-0.107	0.020	-0.234	SFMBT2	0.296	~amyloid_beta+(1|manifest)+cngeneson+pc_mito+brain_region+Sex	1.274	3557.000
ENSG00000139974	0.017	-0.107	0.047	-0.261	SLC38A6	0.054	~amyloid_beta+(1|manifest)+cngeneson+pc_mito+brain_region+Sex	0.794	4645.000
ENSG00000102893	0.088	-0.107	0.047	-0.262	PHKB	0.143	~amyloid_beta+(1|manifest)+cngeneson+pc_mito+brain_region+Sex	0.869	4443.000
ENSG00000134905	0.007	-0.108	-0.016	-0.199	CARS2	0.032	~amyloid_beta+(1|manifest)+cngeneson+pc_mito+brain_region+Sex	0.838	4523.000
ENSG00000163285	0.891	-0.108	0.384	-0.599	GABRG1	0.903	~amyloid_beta+(1|manifest)+cngeneson+pc_mito+brain_region+Sex	8.508	344.000
ENSG00000140471	0.058	-0.108	0.009	-0.224	LINS1	0.109	~amyloid_beta+(1|manifest)+cngeneson+pc_mito+brain_region+Sex	2.877	1617.000
ENSG00000145730	0.910	-0.108	0.374	-0.589	PAM	0.920	~amyloid_beta+(1|manifest)+cngeneson+pc_mito+brain_region+Sex	11.012	221.000
ENSG00000132541	0.393	-0.108	0.101	-0.317	RIDA	0.441	~amyloid_beta+(1|manifest)+cngeneson+pc_mito+brain_region+Sex	2.655	1766.000
ENSG00000196177	0.074	-0.108	0.062	-0.278	ACADSB	0.127	~amyloid_beta+(1|manifest)+cngeneson+pc_mito+brain_region+Sex	1.674	2863.000
ENSG00000083520	0.020	-0.108	-0.013	-0.204	DIS3	0.059	~amyloid_beta+(1|manifest)+cngeneson+pc_mito+brain_region+Sex	0.849	4504.000
ENSG00000196975	0.230	-0.108	0.069	-0.285	ANXA4	0.284	~amyloid_beta+(1|manifest)+cngeneson+pc_mito+brain_region+Sex	1.658	2893.000
ENSG00000149970	0.909	-0.108	0.379	-0.596	CNKSR2	0.919	~amyloid_beta+(1|manifest)+cngeneson+pc_mito+brain_region+Sex	6.238	558.000
ENSG00000112078	0.020	-0.108	0.028	-0.245	KCTD20	0.058	~amyloid_beta+(1|manifest)+cngeneson+pc_mito+brain_region+Sex	1.450	3230.000
ENSG00000159322	0.016	-0.108	-0.026	-0.191	ADPGK	0.052	~amyloid_beta+(1|manifest)+cngeneson+pc_mito+brain_region+Sex	1.341	3425.000
ENSG00000101751	0.012	-0.109	-0.025	-0.192	POLI	0.043	~amyloid_beta+(1|manifest)+cngeneson+pc_mito+brain_region+Sex	0.799	4638.000
ENSG00000166263	0.038	-0.109	-0.014	-0.203	STXBP4	0.085	~amyloid_beta+(1|manifest)+cngeneson+pc_mito+brain_region+Sex	0.392	5578.000
ENSG00000102181	0.008	-0.109	-0.024	-0.193	CD99L2	0.036	~amyloid_beta+(1|manifest)+cngeneson+pc_mito+brain_region+Sex	0.735	4781.000
ENSG00000074755	0.090	-0.109	0.057	-0.275	ZZEF1	0.145	~amyloid_beta+(1|manifest)+cngeneson+pc_mito+brain_region+Sex	2.591	1819.000
ENSG00000081803	0.743	-0.109	0.245	-0.463	CADPS2	0.769	~amyloid_beta+(1|manifest)+cngeneson+pc_mito+brain_region+Sex	0.928	4307.000
ENSG00000165868	0.227	-0.109	0.077	-0.295	HSPA12A	0.281	~amyloid_beta+(1|manifest)+cngeneson+pc_mito+brain_region+Sex	0.412	5541.000
ENSG00000144724	0.629	-0.109	0.136	-0.354	PTPRG	0.665	~amyloid_beta+(1|manifest)+cngeneson+pc_mito+brain_region+Sex	6.740	501.000
ENSG00000156650	0.343	-0.110	0.078	-0.298	KAT6B	0.394	~amyloid_beta+(1|manifest)+cngeneson+pc_mito+brain_region+Sex	2.024	2405.000
ENSG00000132182	0.032	-0.110	0.056	-0.276	NUP210	0.076	~amyloid_beta+(1|manifest)+cngeneson+pc_mito+brain_region+Sex	1.307	3487.000
ENSG00000164209	0.046	-0.110	0.003	-0.222	SLC25A46	0.095	~amyloid_beta+(1|manifest)+cngeneson+pc_mito+brain_region+Sex	1.016	4105.000
ENSG00000023902	0.056	-0.110	0.031	-0.251	PLEKHO1	0.107	~amyloid_beta+(1|manifest)+cngeneson+pc_mito+brain_region+Sex	0.818	4578.000
ENSG00000148019	0.062	-0.110	0.084	-0.305	CEP78	0.114	~amyloid_beta+(1|manifest)+cngeneson+pc_mito+brain_region+Sex	1.230	3641.000
ENSG00000204920	0.032	-0.110	0.073	-0.294	ZNF155	0.076	~amyloid_beta+(1|manifest)+cngeneson+pc_mito+brain_region+Sex	0.899	4375.000
ENSG00000085117	0.254	-0.110	0.109	-0.329	CD82	0.307	~amyloid_beta+(1|manifest)+cngeneson+pc_mito+brain_region+Sex	4.211	978.000
ENSG00000109171	0.045	-0.110	0.008	-0.229	SLAIN2	0.094	~amyloid_beta+(1|manifest)+cngeneson+pc_mito+brain_region+Sex	1.034	4063.000
ENSG00000094841	0.010	-0.110	-0.047	-0.173	UPRT	0.039	~amyloid_beta+(1|manifest)+cngeneson+pc_mito+brain_region+Sex	0.563	5201.000
ENSG00000135929	0.133	-0.110	0.072	-0.293	CYP27A1	0.190	~amyloid_beta+(1|manifest)+cngeneson+pc_mito+brain_region+Sex	1.451	3229.000
ENSG00000183495	0.096	-0.110	0.036	-0.256	EP400	0.151	~amyloid_beta+(1|manifest)+cngeneson+pc_mito+brain_region+Sex	1.923	2534.000
ENSG00000141736	0.101	-0.111	0.091	-0.312	ERBB2	0.156	~amyloid_beta+(1|manifest)+cngeneson+pc_mito+brain_region+Sex	1.755	2745.000
ENSG00000135931	0.089	-0.111	0.018	-0.239	ARMC9	0.144	~amyloid_beta+(1|manifest)+cngeneson+pc_mito+brain_region+Sex	0.740	4769.000
ENSG00000143641	0.719	-0.111	0.166	-0.387	GALNT2	0.747	~amyloid_beta+(1|manifest)+cngeneson+pc_mito+brain_region+Sex	4.933	791.000
ENSG00000116962	0.152	-0.111	0.058	-0.280	NID1	0.207	~amyloid_beta+(1|manifest)+cngeneson+pc_mito+brain_region+Sex	1.617	2956.000
ENSG00000197776	0.171	-0.111	0.072	-0.293	KLHDC1	0.226	~amyloid_beta+(1|manifest)+cngeneson+pc_mito+brain_region+Sex	2.259	2145.000
ENSG00000143458	0.072	-0.111	0.077	-0.299	GABPB2	0.126	~amyloid_beta+(1|manifest)+cngeneson+pc_mito+brain_region+Sex	0.384	5588.000
ENSG00000166004	0.056	-0.111	0.088	-0.310	CEP295	0.107	~amyloid_beta+(1|manifest)+cngeneson+pc_mito+brain_region+Sex	2.389	2002.000
ENSG00000107798	0.054	-0.111	0.045	-0.267	LIPA	0.104	~amyloid_beta+(1|manifest)+cngeneson+pc_mito+brain_region+Sex	1.093	3944.000
ENSG00000163864	0.059	-0.111	0.061	-0.283	NMNAT3	0.110	~amyloid_beta+(1|manifest)+cngeneson+pc_mito+brain_region+Sex	0.462	5435.000
ENSG00000198589	0.521	-0.111	0.082	-0.305	LRBA	0.563	~amyloid_beta+(1|manifest)+cngeneson+pc_mito+brain_region+Sex	4.853	807.000
ENSG00000130684	0.043	-0.111	0.069	-0.292	ZNF337	0.091	~amyloid_beta+(1|manifest)+cngeneson+pc_mito+brain_region+Sex	0.259	5796.000
ENSG00000196865	0.004	-0.111	-0.002	-0.220	NHLRC2	0.024	~amyloid_beta+(1|manifest)+cngeneson+pc_mito+brain_region+Sex	1.372	3374.000
ENSG00000048991	0.091	-0.111	0.011	-0.234	R3HDM1	0.146	~amyloid_beta+(1|manifest)+cngeneson+pc_mito+brain_region+Sex	2.778	1684.000
ENSG00000240344	0.021	-0.111	-0.014	-0.209	PPIL3	0.061	~amyloid_beta+(1|manifest)+cngeneson+pc_mito+brain_region+Sex	1.513	3115.000
ENSG00000023318	0.101	-0.112	0.012	-0.235	ERP44	0.156	~amyloid_beta+(1|manifest)+cngeneson+pc_mito+brain_region+Sex	1.710	2810.000
ENSG00000144741	0.347	-0.112	0.044	-0.268	SLC25A26	0.398	~amyloid_beta+(1|manifest)+cngeneson+pc_mito+brain_region+Sex	1.800	2698.000
ENSG00000158270	0.924	-0.112	0.450	-0.674	COLEC12	0.932	~amyloid_beta+(1|manifest)+cngeneson+pc_mito+brain_region+Sex	11.965	179.000
ENSG00000183137	0.043	-0.112	-0.023	-0.200	CEP57L1	0.092	~amyloid_beta+(1|manifest)+cngeneson+pc_mito+brain_region+Sex	1.524	3100.000
ENSG00000143669	0.511	-0.112	0.212	-0.435	LYST	0.554	~amyloid_beta+(1|manifest)+cngeneson+pc_mito+brain_region+Sex	7.718	397.000
ENSG00000144524	0.005	-0.112	-0.043	-0.181	COPS7B	0.025	~amyloid_beta+(1|manifest)+cngeneson+pc_mito+brain_region+Sex	1.009	4120.000
ENSG00000103544	0.049	-0.112	0.072	-0.296	VPS35L	0.098	~amyloid_beta+(1|manifest)+cngeneson+pc_mito+brain_region+Sex	1.758	2739.000
ENSG00000071537	0.027	-0.112	0.014	-0.238	SEL1L	0.070	~amyloid_beta+(1|manifest)+cngeneson+pc_mito+brain_region+Sex	0.370	5615.000
ENSG00000155561	0.005	-0.112	-0.009	-0.216	NUP205	0.027	~amyloid_beta+(1|manifest)+cngeneson+pc_mito+brain_region+Sex	1.053	4018.000
ENSG00000111011	0.052	-0.113	0.026	-0.251	RSRC2	0.102	~amyloid_beta+(1|manifest)+cngeneson+pc_mito+brain_region+Sex	1.948	2496.000
ENSG00000174989	0.006	-0.113	-0.010	-0.215	FBXW8	0.030	~amyloid_beta+(1|manifest)+cngeneson+pc_mito+brain_region+Sex	0.566	5192.000
ENSG00000179715	0.436	-0.113	0.343	-0.569	PCED1B	0.482	~amyloid_beta+(1|manifest)+cngeneson+pc_mito+brain_region+Sex	6.178	565.000
ENSG00000174945	0.356	-0.113	0.037	-0.263	AMZ1	0.407	~amyloid_beta+(1|manifest)+cngeneson+pc_mito+brain_region+Sex	4.275	955.000
ENSG00000168702	0.480	-0.113	0.071	-0.296	LRP1B	0.525	~amyloid_beta+(1|manifest)+cngeneson+pc_mito+brain_region+Sex	28.404	24.000
ENSG00000167005	0.003	-0.113	-0.040	-0.186	NUDT21	0.019	~amyloid_beta+(1|manifest)+cngeneson+pc_mito+brain_region+Sex	0.543	5251.000
ENSG00000174780	0.001	-0.113	-0.051	-0.175	SRP72	0.011	~amyloid_beta+(1|manifest)+cngeneson+pc_mito+brain_region+Sex	0.516	5313.000
ENSG00000141627	0.457	-0.113	0.074	-0.300	DYM	0.502	~amyloid_beta+(1|manifest)+cngeneson+pc_mito+brain_region+Sex	4.099	1015.000
ENSG00000112294	0.013	-0.113	-0.032	-0.194	ALDH5A1	0.046	~amyloid_beta+(1|manifest)+cngeneson+pc_mito+brain_region+Sex	0.745	4754.000
ENSG00000164309	0.159	-0.113	0.227	-0.453	CMYA5	0.214	~amyloid_beta+(1|manifest)+cngeneson+pc_mito+brain_region+Sex	3.437	1284.000
ENSG00000085788	0.065	-0.113	0.005	-0.231	DDHD2	0.118	~amyloid_beta+(1|manifest)+cngeneson+pc_mito+brain_region+Sex	1.421	3290.000
ENSG00000149532	0.339	-0.113	0.179	-0.406	CPSF7	0.390	~amyloid_beta+(1|manifest)+cngeneson+pc_mito+brain_region+Sex	5.266	715.000
ENSG00000254087	0.191	-0.114	0.046	-0.273	LYN	0.245	~amyloid_beta+(1|manifest)+cngeneson+pc_mito+brain_region+Sex	1.158	3794.000
ENSG00000171988	0.065	-0.114	-0.015	-0.212	JMJD1C	0.118	~amyloid_beta+(1|manifest)+cngeneson+pc_mito+brain_region+Sex	1.952	2491.000
ENSG00000112159	0.031	-0.114	0.064	-0.291	MDN1	0.075	~amyloid_beta+(1|manifest)+cngeneson+pc_mito+brain_region+Sex	1.547	3054.000
ENSG00000134982	0.035	-0.114	-0.028	-0.199	APC	0.081	~amyloid_beta+(1|manifest)+cngeneson+pc_mito+brain_region+Sex	5.907	606.000
ENSG00000107201	0.231	-0.114	0.102	-0.330	DDX58	0.285	~amyloid_beta+(1|manifest)+cngeneson+pc_mito+brain_region+Sex	3.009	1537.000
ENSG00000134247	0.062	-0.114	-0.008	-0.220	PTGFRN	0.114	~amyloid_beta+(1|manifest)+cngeneson+pc_mito+brain_region+Sex	1.691	2840.000
ENSG00000183579	0.322	-0.114	0.034	-0.262	ZNRF3	0.374	~amyloid_beta+(1|manifest)+cngeneson+pc_mito+brain_region+Sex	11.242	210.000
ENSG00000109062	0.306	-0.114	0.086	-0.314	SLC9A3R1	0.359	~amyloid_beta+(1|manifest)+cngeneson+pc_mito+brain_region+Sex	1.114	3900.000
ENSG00000198752	0.067	-0.114	0.047	-0.275	CDC42BPB	0.120	~amyloid_beta+(1|manifest)+cngeneson+pc_mito+brain_region+Sex	1.435	3255.000
ENSG00000141013	0.047	-0.114	0.018	-0.247	GAS8	0.096	~amyloid_beta+(1|manifest)+cngeneson+pc_mito+brain_region+Sex	3.307	1365.000
ENSG00000076321	0.039	-0.114	0.004	-0.233	KLHL20	0.086	~amyloid_beta+(1|manifest)+cngeneson+pc_mito+brain_region+Sex	1.367	3379.000
ENSG00000187097	0.001	-0.115	-0.036	-0.193	ENTPD5	0.013	~amyloid_beta+(1|manifest)+cngeneson+pc_mito+brain_region+Sex	0.937	4285.000
ENSG00000155229	0.087	-0.115	0.014	-0.244	MMS19	0.142	~amyloid_beta+(1|manifest)+cngeneson+pc_mito+brain_region+Sex	1.567	3027.000
ENSG00000109686	0.318	-0.115	0.105	-0.335	SH3D19	0.371	~amyloid_beta+(1|manifest)+cngeneson+pc_mito+brain_region+Sex	1.156	3797.000
ENSG00000107651	0.016	-0.115	-0.021	-0.209	SEC23IP	0.053	~amyloid_beta+(1|manifest)+cngeneson+pc_mito+brain_region+Sex	1.117	3893.000
ENSG00000162437	0.345	-0.115	0.111	-0.341	RAVER2	0.396	~amyloid_beta+(1|manifest)+cngeneson+pc_mito+brain_region+Sex	1.310	3480.000
ENSG00000094975	0.042	-0.115	0.009	-0.239	SUCO	0.091	~amyloid_beta+(1|manifest)+cngeneson+pc_mito+brain_region+Sex	1.891	2574.000
ENSG00000198015	0.004	-0.115	-0.040	-0.191	MRPL42	0.025	~amyloid_beta+(1|manifest)+cngeneson+pc_mito+brain_region+Sex	0.826	4550.000
ENSG00000175395	0.008	-0.115	-0.025	-0.206	ZNF25	0.035	~amyloid_beta+(1|manifest)+cngeneson+pc_mito+brain_region+Sex	1.330	3446.000
ENSG00000100991	0.121	-0.115	0.064	-0.295	TRPC4AP	0.178	~amyloid_beta+(1|manifest)+cngeneson+pc_mito+brain_region+Sex	2.223	2179.000
ENSG00000100393	0.120	-0.115	0.038	-0.269	EP300	0.176	~amyloid_beta+(1|manifest)+cngeneson+pc_mito+brain_region+Sex	2.193	2211.000
ENSG00000181555	0.086	-0.116	0.049	-0.280	SETD2	0.141	~amyloid_beta+(1|manifest)+cngeneson+pc_mito+brain_region+Sex	2.702	1727.000
ENSG00000050405	0.005	-0.116	-0.027	-0.204	LIMA1	0.027	~amyloid_beta+(1|manifest)+cngeneson+pc_mito+brain_region+Sex	0.622	5078.000
ENSG00000135537	0.142	-0.116	0.021	-0.252	AFG1L	0.199	~amyloid_beta+(1|manifest)+cngeneson+pc_mito+brain_region+Sex	1.451	3228.000
ENSG00000175198	0.456	-0.116	0.075	-0.306	PCCA	0.501	~amyloid_beta+(1|manifest)+cngeneson+pc_mito+brain_region+Sex	3.808	1117.000
ENSG00000241058	0.228	-0.116	0.127	-0.358	NSUN6	0.282	~amyloid_beta+(1|manifest)+cngeneson+pc_mito+brain_region+Sex	3.258	1390.000
ENSG00000140836	0.347	-0.116	0.044	-0.275	ZFHX3	0.398	~amyloid_beta+(1|manifest)+cngeneson+pc_mito+brain_region+Sex	2.378	2019.000
ENSG00000122515	0.175	-0.116	0.105	-0.336	ZMIZ2	0.229	~amyloid_beta+(1|manifest)+cngeneson+pc_mito+brain_region+Sex	3.180	1437.000
ENSG00000106052	0.230	-0.116	0.054	-0.286	TAX1BP1	0.284	~amyloid_beta+(1|manifest)+cngeneson+pc_mito+brain_region+Sex	2.179	2224.000
ENSG00000144161	0.007	-0.116	-0.039	-0.194	ZC3H8	0.033	~amyloid_beta+(1|manifest)+cngeneson+pc_mito+brain_region+Sex	1.553	3045.000
ENSG00000125703	0.416	-0.116	0.083	-0.316	ATG4C	0.462	~amyloid_beta+(1|manifest)+cngeneson+pc_mito+brain_region+Sex	3.125	1466.000
ENSG00000116830	0.075	-0.116	0.032	-0.265	TTF2	0.129	~amyloid_beta+(1|manifest)+cngeneson+pc_mito+brain_region+Sex	1.557	3041.000
ENSG00000085719	0.005	-0.117	-0.041	-0.192	CPNE3	0.028	~amyloid_beta+(1|manifest)+cngeneson+pc_mito+brain_region+Sex	0.573	5181.000
ENSG00000116117	0.053	-0.117	-0.027	-0.206	PARD3B	0.103	~amyloid_beta+(1|manifest)+cngeneson+pc_mito+brain_region+Sex	1.402	3317.000
ENSG00000198373	0.006	-0.117	-0.019	-0.214	WWP2	0.030	~amyloid_beta+(1|manifest)+cngeneson+pc_mito+brain_region+Sex	0.806	4611.000
ENSG00000089177	0.126	-0.117	0.084	-0.318	KIF16B	0.182	~amyloid_beta+(1|manifest)+cngeneson+pc_mito+brain_region+Sex	1.896	2555.000
ENSG00000148396	0.003	-0.117	-0.027	-0.207	SEC16A	0.021	~amyloid_beta+(1|manifest)+cngeneson+pc_mito+brain_region+Sex	1.031	4070.000
ENSG00000133835	0.032	-0.117	0.001	-0.234	HSD17B4	0.077	~amyloid_beta+(1|manifest)+cngeneson+pc_mito+brain_region+Sex	1.342	3424.000
ENSG00000129292	0.053	-0.117	0.032	-0.266	PHF20L1	0.103	~amyloid_beta+(1|manifest)+cngeneson+pc_mito+brain_region+Sex	1.394	3333.000
ENSG00000135821	0.755	-0.117	0.204	-0.438	GLUL	0.779	~amyloid_beta+(1|manifest)+cngeneson+pc_mito+brain_region+Sex	5.612	664.000
ENSG00000126773	0.215	-0.117	0.113	-0.347	PCNX4	0.270	~amyloid_beta+(1|manifest)+cngeneson+pc_mito+brain_region+Sex	2.503	1902.000
ENSG00000160563	0.269	-0.117	0.073	-0.307	MED27	0.323	~amyloid_beta+(1|manifest)+cngeneson+pc_mito+brain_region+Sex	3.347	1342.000
ENSG00000135457	0.182	-0.117	0.057	-0.292	TFCP2	0.236	~amyloid_beta+(1|manifest)+cngeneson+pc_mito+brain_region+Sex	1.362	3387.000
ENSG00000164300	0.386	-0.118	0.073	-0.309	SERINC5	0.436	~amyloid_beta+(1|manifest)+cngeneson+pc_mito+brain_region+Sex	0.851	4496.000
ENSG00000112530	0.705	-0.118	0.241	-0.476	PACRG	0.733	~amyloid_beta+(1|manifest)+cngeneson+pc_mito+brain_region+Sex	1.316	3468.000
ENSG00000198794	0.007	-0.118	-0.033	-0.203	SCAMP5	0.032	~amyloid_beta+(1|manifest)+cngeneson+pc_mito+brain_region+Sex	0.987	4175.000
ENSG00000145555	0.764	-0.118	0.278	-0.514	MYO10	0.787	~amyloid_beta+(1|manifest)+cngeneson+pc_mito+brain_region+Sex	2.588	1821.000
ENSG00000167635	0.011	-0.118	-0.013	-0.223	ZNF146	0.041	~amyloid_beta+(1|manifest)+cngeneson+pc_mito+brain_region+Sex	1.504	3132.000
ENSG00000163565	0.840	-0.118	0.283	-0.519	IFI16	0.856	~amyloid_beta+(1|manifest)+cngeneson+pc_mito+brain_region+Sex	2.212	2191.000
ENSG00000198964	0.570	-0.118	0.108	-0.344	SGMS1	0.608	~amyloid_beta+(1|manifest)+cngeneson+pc_mito+brain_region+Sex	2.343	2056.000
ENSG00000146085	0.035	-0.118	0.017	-0.253	MMUT	0.081	~amyloid_beta+(1|manifest)+cngeneson+pc_mito+brain_region+Sex	0.527	5294.000
ENSG00000135272	0.200	-0.118	0.140	-0.376	MDFIC	0.254	~amyloid_beta+(1|manifest)+cngeneson+pc_mito+brain_region+Sex	0.766	4701.000
ENSG00000133103	0.187	-0.118	0.047	-0.283	COG6	0.241	~amyloid_beta+(1|manifest)+cngeneson+pc_mito+brain_region+Sex	1.757	2741.000
ENSG00000118620	0.019	-0.118	-0.031	-0.205	ZNF430	0.057	~amyloid_beta+(1|manifest)+cngeneson+pc_mito+brain_region+Sex	0.958	4229.000
ENSG00000147905	0.556	-0.118	0.102	-0.338	ZCCHC7	0.596	~amyloid_beta+(1|manifest)+cngeneson+pc_mito+brain_region+Sex	2.737	1712.000
ENSG00000114127	0.033	-0.118	0.036	-0.272	XRN1	0.077	~amyloid_beta+(1|manifest)+cngeneson+pc_mito+brain_region+Sex	1.537	3072.000
ENSG00000184014	0.160	-0.118	0.047	-0.283	DENND5A	0.215	~amyloid_beta+(1|manifest)+cngeneson+pc_mito+brain_region+Sex	2.410	1974.000
ENSG00000170759	0.065	-0.118	0.047	-0.284	KIF5B	0.118	~amyloid_beta+(1|manifest)+cngeneson+pc_mito+brain_region+Sex	2.110	2299.000
ENSG00000174231	0.088	-0.118	0.061	-0.298	PRPF8	0.143	~amyloid_beta+(1|manifest)+cngeneson+pc_mito+brain_region+Sex	2.979	1553.000
ENSG00000110031	0.005	-0.119	-0.019	-0.218	LPXN	0.028	~amyloid_beta+(1|manifest)+cngeneson+pc_mito+brain_region+Sex	0.945	4267.000
ENSG00000127603	0.292	-0.119	0.043	-0.280	MACF1	0.344	~amyloid_beta+(1|manifest)+cngeneson+pc_mito+brain_region+Sex	29.561	22.000
ENSG00000069248	0.003	-0.119	-0.038	-0.200	NUP133	0.019	~amyloid_beta+(1|manifest)+cngeneson+pc_mito+brain_region+Sex	0.989	4166.000
ENSG00000166579	0.259	-0.119	0.036	-0.274	NDEL1	0.313	~amyloid_beta+(1|manifest)+cngeneson+pc_mito+brain_region+Sex	2.045	2376.000
ENSG00000138138	0.004	-0.119	-0.038	-0.200	ATAD1	0.025	~amyloid_beta+(1|manifest)+cngeneson+pc_mito+brain_region+Sex	1.344	3420.000
ENSG00000140538	0.277	-0.119	0.042	-0.280	NTRK3	0.330	~amyloid_beta+(1|manifest)+cngeneson+pc_mito+brain_region+Sex	14.545	111.000
ENSG00000135835	0.031	-0.119	0.029	-0.267	KIAA1614	0.075	~amyloid_beta+(1|manifest)+cngeneson+pc_mito+brain_region+Sex	1.372	3376.000
ENSG00000151338	0.416	-0.119	0.139	-0.377	MIPOL1	0.462	~amyloid_beta+(1|manifest)+cngeneson+pc_mito+brain_region+Sex	0.755	4727.000
ENSG00000159445	0.040	-0.119	-0.009	-0.230	THEM4	0.087	~amyloid_beta+(1|manifest)+cngeneson+pc_mito+brain_region+Sex	1.398	3326.000
ENSG00000162695	0.021	-0.119	0.005	-0.243	SLC30A7	0.060	~amyloid_beta+(1|manifest)+cngeneson+pc_mito+brain_region+Sex	2.175	2231.000
ENSG00000124370	0.004	-0.119	-0.043	-0.195	MCEE	0.025	~amyloid_beta+(1|manifest)+cngeneson+pc_mito+brain_region+Sex	1.000	4136.000
ENSG00000038532	0.645	-0.119	0.152	-0.391	CLEC16A	0.678	~amyloid_beta+(1|manifest)+cngeneson+pc_mito+brain_region+Sex	10.022	269.000
ENSG00000157500	0.030	-0.119	-0.006	-0.233	APPL1	0.074	~amyloid_beta+(1|manifest)+cngeneson+pc_mito+brain_region+Sex	1.253	3591.000
ENSG00000141127	0.003	-0.119	-0.036	-0.203	PRPSAP2	0.021	~amyloid_beta+(1|manifest)+cngeneson+pc_mito+brain_region+Sex	0.943	4271.000
ENSG00000165632	0.270	-0.120	0.022	-0.261	TAF3	0.324	~amyloid_beta+(1|manifest)+cngeneson+pc_mito+brain_region+Sex	1.445	3240.000
ENSG00000125520	0.177	-0.120	0.079	-0.318	SLC2A4RG	0.232	~amyloid_beta+(1|manifest)+cngeneson+pc_mito+brain_region+Sex	2.361	2038.000
ENSG00000188227	0.016	-0.120	0.022	-0.261	ZNF793	0.053	~amyloid_beta+(1|manifest)+cngeneson+pc_mito+brain_region+Sex	0.952	4248.000
ENSG00000139726	0.010	-0.120	-0.016	-0.224	DENR	0.039	~amyloid_beta+(1|manifest)+cngeneson+pc_mito+brain_region+Sex	1.445	3238.000
ENSG00000171408	0.702	-0.120	0.181	-0.420	PDE7B	0.730	~amyloid_beta+(1|manifest)+cngeneson+pc_mito+brain_region+Sex	8.098	372.000
ENSG00000163875	0.015	-0.120	-0.021	-0.219	MEAF6	0.050	~amyloid_beta+(1|manifest)+cngeneson+pc_mito+brain_region+Sex	1.320	3463.000
ENSG00000127241	0.052	-0.120	0.097	-0.337	MASP1	0.102	~amyloid_beta+(1|manifest)+cngeneson+pc_mito+brain_region+Sex	1.441	3247.000
ENSG00000074356	0.006	-0.120	-0.023	-0.216	NCBP3	0.030	~amyloid_beta+(1|manifest)+cngeneson+pc_mito+brain_region+Sex	1.117	3892.000
ENSG00000198625	0.078	-0.120	0.038	-0.278	MDM4	0.133	~amyloid_beta+(1|manifest)+cngeneson+pc_mito+brain_region+Sex	1.824	2661.000
ENSG00000122550	0.089	-0.120	0.004	-0.245	KLHL7	0.143	~amyloid_beta+(1|manifest)+cngeneson+pc_mito+brain_region+Sex	1.535	3077.000
ENSG00000119402	0.002	-0.120	-0.051	-0.189	FBXW2	0.017	~amyloid_beta+(1|manifest)+cngeneson+pc_mito+brain_region+Sex	0.761	4714.000
ENSG00000132670	0.140	-0.120	0.029	-0.269	PTPRA	0.197	~amyloid_beta+(1|manifest)+cngeneson+pc_mito+brain_region+Sex	2.506	1895.000

ENSG00000166233	0.276	-0.120	0.070	-0.311	ARIH1	0.329	~amyloid_beta+(1|manifest)+cngeneson+pc_mito+brain_region+Sex	3.409	1310.000
ENSG00000122008	0.026	-0.120	0.025	-0.266	POLK	0.068	~amyloid_beta+(1|manifest)+cngeneson+pc_mito+brain_region+Sex	0.942	4273.000
ENSG00000198382	0.008	-0.121	-0.022	-0.219	UVRAG	0.035	~amyloid_beta+(1|manifest)+cngeneson+pc_mito+brain_region+Sex	1.177	3755.000
ENSG00000060138	0.793	-0.121	0.427	-0.668	YBX3	0.813	~amyloid_beta+(1|manifest)+cngeneson+pc_mito+brain_region+Sex	10.448	249.000
ENSG00000122783	0.096	-0.121	-0.021	-0.220	CYREN	0.151	~amyloid_beta+(1|manifest)+cngeneson+pc_mito+brain_region+Sex	0.272	5779.000
ENSG00000167555	0.014	-0.121	-0.014	-0.227	ZNF528	0.048	~amyloid_beta+(1|manifest)+cngeneson+pc_mito+brain_region+Sex	0.849	4503.000
ENSG00000103591	0.010	-0.121	-0.033	-0.209	AAGAB	0.039	~amyloid_beta+(1|manifest)+cngeneson+pc_mito+brain_region+Sex	1.049	4028.000
ENSG00000137075	0.067	-0.121	0.049	-0.292	RNF38	0.121	~amyloid_beta+(1|manifest)+cngeneson+pc_mito+brain_region+Sex	1.717	2801.000
ENSG00000029639	0.001	-0.121	-0.061	-0.182	TFB1M	0.013	~amyloid_beta+(1|manifest)+cngeneson+pc_mito+brain_region+Sex	0.635	5047.000
ENSG00000147548	0.561	-0.121	0.130	-0.373	NSD3	0.600	~amyloid_beta+(1|manifest)+cngeneson+pc_mito+brain_region+Sex	3.928	1077.000
ENSG00000126216	0.009	-0.121	-0.006	-0.236	TUBGCP3	0.037	~amyloid_beta+(1|manifest)+cngeneson+pc_mito+brain_region+Sex	1.476	3178.000
ENSG00000186908	0.020	-0.121	0.002	-0.245	ZDHHC17	0.058	~amyloid_beta+(1|manifest)+cngeneson+pc_mito+brain_region+Sex	0.800	4633.000
ENSG00000138814	0.347	-0.122	0.051	-0.294	PPP3CA	0.397	~amyloid_beta+(1|manifest)+cngeneson+pc_mito+brain_region+Sex	8.958	316.000
ENSG00000134744	0.082	-0.122	0.009	-0.252	TUT4	0.137	~amyloid_beta+(1|manifest)+cngeneson+pc_mito+brain_region+Sex	1.736	2768.000
ENSG00000154767	0.031	-0.122	0.010	-0.254	XPC	0.075	~amyloid_beta+(1|manifest)+cngeneson+pc_mito+brain_region+Sex	1.542	3060.000
ENSG00000101310	0.012	-0.122	-0.013	-0.230	SEC23B	0.044	~amyloid_beta+(1|manifest)+cngeneson+pc_mito+brain_region+Sex	0.914	4343.000
ENSG00000163214	0.017	-0.122	-0.001	-0.242	DHX57	0.054	~amyloid_beta+(1|manifest)+cngeneson+pc_mito+brain_region+Sex	0.976	4193.000
ENSG00000018510	0.004	-0.122	-0.025	-0.218	AGPS	0.023	~amyloid_beta+(1|manifest)+cngeneson+pc_mito+brain_region+Sex	0.938	4282.000
ENSG00000077147	0.040	-0.122	-0.014	-0.229	TM9SF3	0.087	~amyloid_beta+(1|manifest)+cngeneson+pc_mito+brain_region+Sex	1.714	2803.000
ENSG00000162599	0.192	-0.122	0.007	-0.251	NFIA	0.246	~amyloid_beta+(1|manifest)+cngeneson+pc_mito+brain_region+Sex	10.711	232.000
ENSG00000137776	0.205	-0.122	0.069	-0.313	SLTM	0.260	~amyloid_beta+(1|manifest)+cngeneson+pc_mito+brain_region+Sex	3.151	1451.000
ENSG00000144357	0.171	-0.122	0.007	-0.251	UBR3	0.227	~amyloid_beta+(1|manifest)+cngeneson+pc_mito+brain_region+Sex	3.472	1260.000
ENSG00000115760	0.026	-0.122	-0.032	-0.212	BIRC6	0.069	~amyloid_beta+(1|manifest)+cngeneson+pc_mito+brain_region+Sex	5.744	638.000
ENSG00000075790	0.000	-0.122	-0.077	-0.167	BCAP29	0.001	~amyloid_beta+(1|manifest)+cngeneson+pc_mito+brain_region+Sex	0.459	5441.000
ENSG00000125944	0.063	-0.122	0.023	-0.267	HNRNPR	0.116	~amyloid_beta+(1|manifest)+cngeneson+pc_mito+brain_region+Sex	2.192	2212.000
ENSG00000125871	0.001	-0.122	-0.057	-0.188	MGME1	0.010	~amyloid_beta+(1|manifest)+cngeneson+pc_mito+brain_region+Sex	0.608	5114.000
ENSG00000188641	0.412	-0.122	0.071	-0.315	DPYD	0.458	~amyloid_beta+(1|manifest)+cngeneson+pc_mito+brain_region+Sex	2.168	2240.000
ENSG00000021645	0.172	-0.122	0.013	-0.257	NRXN3	0.227	~amyloid_beta+(1|manifest)+cngeneson+pc_mito+brain_region+Sex	2.208	2194.000
ENSG00000137103	0.029	-0.123	-0.011	-0.235	TMEM8B	0.073	~amyloid_beta+(1|manifest)+cngeneson+pc_mito+brain_region+Sex	0.709	4849.000
ENSG00000100234	0.623	-0.123	0.261	-0.506	TIMP3	0.658	~amyloid_beta+(1|manifest)+cngeneson+pc_mito+brain_region+Sex	5.003	774.000
ENSG00000015171	0.289	-0.123	0.072	-0.318	ZMYND11	0.342	~amyloid_beta+(1|manifest)+cngeneson+pc_mito+brain_region+Sex	2.492	1909.000
ENSG00000134769	0.220	-0.123	0.065	-0.311	DTNA	0.275	~amyloid_beta+(1|manifest)+cngeneson+pc_mito+brain_region+Sex	33.730	13.000
ENSG00000106003	0.499	-0.123	0.159	-0.405	LFNG	0.543	~amyloid_beta+(1|manifest)+cngeneson+pc_mito+brain_region+Sex	3.914	1084.000
ENSG00000110422	0.698	-0.123	0.297	-0.544	HIPK3	0.727	~amyloid_beta+(1|manifest)+cngeneson+pc_mito+brain_region+Sex	7.447	420.000
ENSG00000151348	0.025	-0.123	0.028	-0.275	EXT2	0.067	~amyloid_beta+(1|manifest)+cngeneson+pc_mito+brain_region+Sex	1.829	2655.000
ENSG00000086102	0.026	-0.123	0.026	-0.273	NFX1	0.068	~amyloid_beta+(1|manifest)+cngeneson+pc_mito+brain_region+Sex	1.660	2889.000
ENSG00000171824	0.087	-0.123	0.170	-0.417	EXOSC10	0.142	~amyloid_beta+(1|manifest)+cngeneson+pc_mito+brain_region+Sex	3.139	1458.000
ENSG00000179761	0.244	-0.123	0.051	-0.298	PIPOX	0.298	~amyloid_beta+(1|manifest)+cngeneson+pc_mito+brain_region+Sex	2.685	1741.000
ENSG00000078018	0.590	-0.124	0.504	-0.751	MAP2	0.627	~amyloid_beta+(1|manifest)+cngeneson+pc_mito+brain_region+Sex	11.236	211.000
ENSG00000138433	0.009	-0.124	0.001	-0.248	CIR1	0.037	~amyloid_beta+(1|manifest)+cngeneson+pc_mito+brain_region+Sex	0.674	4956.000
ENSG00000162601	0.027	-0.124	0.010	-0.258	MYSM1	0.070	~amyloid_beta+(1|manifest)+cngeneson+pc_mito+brain_region+Sex	2.734	1714.000
ENSG00000150471	0.136	-0.124	-0.006	-0.242	ADGRL3	0.193	~amyloid_beta+(1|manifest)+cngeneson+pc_mito+brain_region+Sex	7.369	429.000
ENSG00000051825	0.129	-0.124	0.094	-0.342	[illegible]	0.185	~amyloid_beta+(1|manifest)+cngeneson+pc_mito+brain_region+Sex	2.315	2085.000
ENSG00000153823	0.681	-0.124	0.154	-0.402	PID1	0.712	~amyloid_beta+(1|manifest)+cngeneson+pc_mito+brain_region+Sex	3.608	1202.000
ENSG00000105401	0.476	-0.124	0.244	-0.492	CDC37	0.521	~amyloid_beta+(1|manifest)+cngeneson+pc_mito+brain_region+Sex	4.479	895.000
ENSG00000063660	0.539	-0.124	0.218	-0.466	GPC1	0.580	~amyloid_beta+(1|manifest)+cngeneson+pc_mito+brain_region+Sex	3.795	1127.000
ENSG00000196376	0.748	-0.124	0.203	-0.451	SLC35F1	0.773	~amyloid_beta+(1|manifest)+cngeneson+pc_mito+brain_region+Sex	7.504	419.000
ENSG00000134014	0.023	-0.124	-0.008	-0.241	ELP3	0.064	~amyloid_beta+(1|manifest)+cngeneson+pc_mito+brain_region+Sex	1.154	3807.000
ENSG00000119720	0.147	-0.124	0.028	-0.277	NRDE2	0.202	~amyloid_beta+(1|manifest)+cngeneson+pc_mito+brain_region+Sex	1.847	2631.000
ENSG00000198887	0.624	-0.124	0.259	-0.508	SMC5	0.660	~amyloid_beta+(1|manifest)+cngeneson+pc_mito+brain_region+Sex	8.588	340.000
ENSG00000111913	0.095	-0.125	0.153	-0.403	RIPOR2	0.150	~amyloid_beta+(1|manifest)+cngeneson+pc_mito+brain_region+Sex	3.610	1201.000
ENSG00000144320	0.008	-0.125	-0.028	-0.222	LNPK	0.033	~amyloid_beta+(1|manifest)+cngeneson+pc_mito+brain_region+Sex	1.436	3253.000
ENSG00000174485	0.769	-0.125	0.229	-0.479	DENND4A	0.791	~amyloid_beta+(1|manifest)+cngeneson+pc_mito+brain_region+Sex	11.685	192.000
ENSG00000103852	0.098	-0.125	0.042	-0.291	TTC23	0.153	~amyloid_beta+(1|manifest)+cngeneson+pc_mito+brain_region+Sex	2.048	2371.000
ENSG00000085433	0.040	-0.125	0.017	-0.266	WDR47	0.088	~amyloid_beta+(1|manifest)+cngeneson+pc_mito+brain_region+Sex	1.787	2712.000
ENSG00000198718	0.001	-0.125	-0.054	-0.196	[illegible]	0.011	~amyloid_beta+(1|manifest)+cngeneson+pc_mito+brain_region+Sex	1.371	3377.000
ENSG00000136152	0.002	-0.125	-0.038	-0.212	COG3	0.016	~amyloid_beta+(1|manifest)+cngeneson+pc_mito+brain_region+Sex	0.933	4293.000
ENSG00000161547	0.139	-0.125	0.053	-0.304	SRSF2	0.196	~amyloid_beta+(1|manifest)+cngeneson+pc_mito+brain_region+Sex	2.619	1801.000
ENSG00000147535	0.085	-0.125	0.034	-0.284	PLPP5	0.140	~amyloid_beta+(1|manifest)+cngeneson+pc_mito+brain_region+Sex	2.380	2015.000
ENSG00000198846	0.497	-0.125	0.128	-0.379	TOX	0.541	~amyloid_beta+(1|manifest)+cngeneson+pc_mito+brain_region+Sex	2.347	2052.000
ENSG00000103599	0.054	-0.126	0.006	-0.257	IQCH	0.104	~amyloid_beta+(1|manifest)+cngeneson+pc_mito+brain_region+Sex	1.314	3472.000
ENSG00000143924	0.125	-0.126	0.009	-0.261	EML4	0.181	~amyloid_beta+(1|manifest)+cngeneson+pc_mito+brain_region+Sex	2.189	2216.000
ENSG00000167196	0.024	-0.126	-0.015	-0.237	FBXO22	0.066	~amyloid_beta+(1|manifest)+cngeneson+pc_mito+brain_region+Sex	1.397	3328.000
ENSG00000088205	0.083	-0.126	0.025	-0.277	DDX18	0.138	~amyloid_beta+(1|manifest)+cngeneson+pc_mito+brain_region+Sex	1.698	2826.000
ENSG00000156011	0.181	-0.126	0.008	-0.260	PSD3	0.236	~amyloid_beta+(1|manifest)+cngeneson+pc_mito+brain_region+Sex	10.126	254.000
ENSG00000078674	0.093	-0.126	0.001	-0.253	PCM1	0.148	~amyloid_beta+(1|manifest)+cngeneson+pc_mito+brain_region+Sex	2.047	2372.000
ENSG00000124783	0.120	-0.126	0.030	-0.283	SSR1	0.176	~amyloid_beta+(1|manifest)+cngeneson+pc_mito+brain_region+Sex	1.281	3544.000
ENSG00000185658	0.297	-0.126	0.034	-0.287	BRWD1	0.350	~amyloid_beta+(1|manifest)+cngeneson+pc_mito+brain_region+Sex	4.209	980.000
ENSG00000138095	0.117	-0.126	0.039	-0.292	LRPPRC	0.174	~amyloid_beta+(1|manifest)+cngeneson+pc_mito+brain_region+Sex	0.772	4684.000
ENSG00000124006	0.144	-0.126	0.133	-0.386	OBSL1	0.200	~amyloid_beta+(1|manifest)+cngeneson+pc_mito+brain_region+Sex	4.750	833.000
ENSG00000166272	0.015	-0.127	-0.024	-0.230	WBP1L	0.051	~amyloid_beta+(1|manifest)+cngeneson+pc_mito+brain_region+Sex	0.476	5409.000
ENSG00000108061	0.195	-0.127	0.076	-0.330	SHOC2	0.249	~amyloid_beta+(1|manifest)+cngeneson+pc_mito+brain_region+Sex	2.814	1656.000
ENSG00000143614	0.112	-0.127	0.072	-0.326	GATAD2B	0.168	~amyloid_beta+(1|manifest)+cngeneson+pc_mito+brain_region+Sex	2.182	2223.000
ENSG00000125351	0.156	-0.127	0.120	-0.373	UPF3B	0.211	~amyloid_beta+(1|manifest)+cngeneson+pc_mito+brain_region+Sex	2.326	2074.000
ENSG00000078668	0.243	-0.127	0.090	-0.344	VDAC3	0.297	~amyloid_beta+(1|manifest)+cngeneson+pc_mito+brain_region+Sex	2.933	1585.000
ENSG00000171055	0.022	-0.127	0.021	-0.275	FEZ2	0.062	~amyloid_beta+(1|manifest)+cngeneson+pc_mito+brain_region+Sex	2.061	2356.000
ENSG00000052841	0.067	-0.127	-0.021	-0.233	TTC17	0.120	~amyloid_beta+(1|manifest)+cngeneson+pc_mito+brain_region+Sex	2.990	1547.000
ENSG00000136295	0.403	-0.127	0.125	-0.380	TTYH3	0.450	~amyloid_beta+(1|manifest)+cngeneson+pc_mito+brain_region+Sex	2.140	2274.000
ENSG00000175161	0.537	-0.127	0.094	-0.348	CADM2	0.578	~amyloid_beta+(1|manifest)+cngeneson+pc_mito+brain_region+Sex	26.601	26.000
ENSG00000115526	0.075	-0.127	-0.007	-0.247	CHST10	0.129	~amyloid_beta+(1|manifest)+cngeneson+pc_mito+brain_region+Sex	0.836	4526.000
ENSG00000152583	0.062	-0.128	0.014	-0.269	SPARCL1	0.114	~amyloid_beta+(1|manifest)+cngeneson+pc_mito+brain_region+Sex	4.226	973.000
ENSG00000166348	0.429	-0.128	0.202	-0.457	USP54	0.475	~amyloid_beta+(1|manifest)+cngeneson+pc_mito+brain_region+Sex	4.558	876.000
ENSG00000115365	0.007	-0.128	-0.033	-0.223	LANCL1	0.032	~amyloid_beta+(1|manifest)+cngeneson+pc_mito+brain_region+Sex	1.302	3503.000
ENSG00000124786	0.017	-0.128	-0.009	-0.247	SLC35B3	0.054	~amyloid_beta+(1|manifest)+cngeneson+pc_mito+brain_region+Sex	1.533	3081.000
ENSG00000165029	0.445	-0.128	0.184	-0.440	ABCA1	0.491	~amyloid_beta+(1|manifest)+cngeneson+pc_mito+brain_region+Sex	1.642	2921.000
ENSG00000169946	0.396	-0.128	0.061	-0.317	ZFPM2	0.444	~amyloid_beta+(1|manifest)+cngeneson+pc_mito+brain_region+Sex	1.894	2561.000
ENSG00000132912	0.005	-0.128	-0.037	-0.219	DCTN4	0.026	~amyloid_beta+(1|manifest)+cngeneson+pc_mito+brain_region+Sex	1.317	3467.000
ENSG00000149218	0.331	-0.128	0.118	-0.375	ENDOD1	0.383	~amyloid_beta+(1|manifest)+cngeneson+pc_mito+brain_region+Sex	5.781	629.000
ENSG00000070269	0.012	-0.128	-0.018	-0.238	TMEM260	0.043	~amyloid_beta+(1|manifest)+cngeneson+pc_mito+brain_region+Sex	1.096	3937.000
ENSG00000151914	0.057	-0.128	0.082	-0.339	DST	0.109	~amyloid_beta+(1|manifest)+cngeneson+pc_mito+brain_region+Sex	30.378	20.000
ENSG00000092203	0.060	-0.128	-0.005	-0.252	TOX4	0.111	~amyloid_beta+(1|manifest)+cngeneson+pc_mito+brain_region+Sex	1.451	3227.000
ENSG00000180773	0.019	-0.128	-0.006	-0.251	SLC36A4	0.058	~amyloid_beta+(1|manifest)+cngeneson+pc_mito+brain_region+Sex	1.087	3955.000
ENSG00000128607	0.110	-0.128	0.030	-0.287	KLHDC10	0.166	~amyloid_beta+(1|manifest)+cngeneson+pc_mito+brain_region+Sex	1.402	3319.000
ENSG00000164151	0.049	-0.129	0.026	-0.283	ICE1	0.098	~amyloid_beta+(1|manifest)+cngeneson+pc_mito+brain_region+Sex	1.175	3759.000
ENSG00000134371	0.172	-0.129	0.080	-0.338	CDC73	0.227	~amyloid_beta+(1|manifest)+cngeneson+pc_mito+brain_region+Sex	1.894	2563.000
ENSG00000008869	0.005	-0.129	-0.044	-0.214	HEATR5B	0.027	~amyloid_beta+(1|manifest)+cngeneson+pc_mito+brain_region+Sex	1.139	3838.000
ENSG00000004766	0.134	-0.129	0.027	-0.285	VPS50	0.191	~amyloid_beta+(1|manifest)+cngeneson+pc_mito+brain_region+Sex	0.976	4192.000
ENSG00000023171	0.287	-0.129	0.246	-0.504	GRAMD1B	0.340	~amyloid_beta+(1|manifest)+cngeneson+pc_mito+brain_region+Sex	2.892	1610.000
ENSG00000198780	0.078	-0.129	0.038	-0.296	FAM169A	0.133	~amyloid_beta+(1|manifest)+cngeneson+pc_mito+brain_region+Sex	2.167	2242.000
ENSG00000145715	0.402	-0.129	0.078	-0.337	RASA1	0.450	~amyloid_beta+(1|manifest)+cngeneson+pc_mito+brain_region+Sex	2.108	2301.000
ENSG00000152926	0.142	-0.129	0.120	-0.378	ZNF117	0.199	~amyloid_beta+(1|manifest)+cngeneson+pc_mito+brain_region+Sex	1.553	3046.000
ENSG00000115942	0.085	-0.129	0.043	-0.302	ORC2	0.140	~amyloid_beta+(1|manifest)+cngeneson+pc_mito+brain_region+Sex	1.963	2473.000
ENSG00000105835	0.539	-0.129	0.084	-0.342	NAMPT	0.580	~amyloid_beta+(1|manifest)+cngeneson+pc_mito+brain_region+Sex	6.419	534.000
ENSG00000203705	0.022	-0.129	-0.037	-0.222	TATDN3	0.062	~amyloid_beta+(1|manifest)+cngeneson+pc_mito+brain_region+Sex	1.271	3563.000
ENSG00000148358	0.050	-0.129	0.016	-0.274	GPR107	0.099	~amyloid_beta+(1|manifest)+cngeneson+pc_mito+brain_region+Sex	2.038	2383.000
ENSG00000107625	0.022	-0.130	-0.005	-0.254	DDX50	0.062	~amyloid_beta+(1|manifest)+cngeneson+pc_mito+brain_region+Sex	1.818	2673.000
ENSG00000067900	0.176	-0.130	0.039	-0.299	ROCK1	0.231	~amyloid_beta+(1|manifest)+cngeneson+pc_mito+brain_region+Sex	1.818	2671.000
ENSG00000096872	0.009	-0.130	0.013	-0.273	IFT74	0.038	~amyloid_beta+(1|manifest)+cngeneson+pc_mito+brain_region+Sex	0.603	5119.000
ENSG00000130396	0.211	-0.130	0.059	-0.319	AFDN	0.265	~amyloid_beta+(1|manifest)+cngeneson+pc_mito+brain_region+Sex	1.511	3123.000
ENSG00000126861	0.198	-0.130	0.063	-0.323	OMG	0.253	~amyloid_beta+(1|manifest)+cngeneson+pc_mito+brain_region+Sex	1.221	3661.000
ENSG00000180182	0.049	-0.130	0.018	-0.278	MED14	0.099	~amyloid_beta+(1|manifest)+cngeneson+pc_mito+brain_region+Sex	1.463	3201.000
ENSG00000164125	0.221	-0.130	0.095	-0.356	GASK1B	0.276	~amyloid_beta+(1|manifest)+cngeneson+pc_mito+brain_region+Sex	1.140	3835.000
ENSG00000142875	0.024	-0.130	-0.036	-0.225	PRKACB	0.066	~amyloid_beta+(1|manifest)+cngeneson+pc_mito+brain_region+Sex	1.103	3920.000
ENSG00000196123	0.251	-0.130	0.132	-0.392	KIAA0895L	0.305	~amyloid_beta+(1|manifest)+cngeneson+pc_mito+brain_region+Sex	3.385	1326.000
ENSG00000100650	0.661	-0.130	0.260	-0.521	SRSF5	0.693	~amyloid_beta+(1|manifest)+cngeneson+pc_mito+brain_region+Sex	11.912	180.000
ENSG00000134255	0.009	-0.131	-0.037	-0.224	CEPT1	0.039	~amyloid_beta+(1|manifest)+cngeneson+pc_mito+brain_region+Sex	0.648	5009.000
ENSG00000130741	0.062	-0.131	0.039	-0.300	EIF2S3	0.114	~amyloid_beta+(1|manifest)+cngeneson+pc_mito+brain_region+Sex	2.476	1918.000
ENSG00000119227	0.090	-0.131	0.040	-0.301	PIGZ	0.145	~amyloid_beta+(1|manifest)+cngeneson+pc_mito+brain_region+Sex	1.657	2894.000
ENSG00000152767	0.525	-0.131	0.124	-0.386	FARP1	0.567	~amyloid_beta+(1|manifest)+cngeneson+pc_mito+brain_region+Sex	9.480	298.000
ENSG00000129351	0.068	-0.131	0.017	-0.279	ILF3	0.121	~amyloid_beta+(1|manifest)+cngeneson+pc_mito+brain_region+Sex	1.944	2504.000
ENSG00000173273	0.393	-0.131	0.062	-0.324	TNKS	0.441	~amyloid_beta+(1|manifest)+cngeneson+pc_mito+brain_region+Sex	1.894	2562.000
ENSG00000112293	0.015	-0.131	-0.001	-0.261	GPLD1	0.050	~amyloid_beta+(1|manifest)+cngeneson+pc_mito+brain_region+Sex	1.535	3076.000
ENSG00000078687	0.071	-0.132	0.002	-0.266	TNRC6C	0.125	~amyloid_beta+(1|manifest)+cngeneson+pc_mito+brain_region+Sex	1.233	3632.000
ENSG00000181826	0.495	-0.132	0.133	-0.396	RELL1	0.539	~amyloid_beta+(1|manifest)+cngeneson+pc_mito+brain_region+Sex	10.617	239.000
ENSG00000152422	0.170	-0.132	-0.004	-0.260	XRCC4	0.226	~amyloid_beta+(1|manifest)+cngeneson+pc_mito+brain_region+Sex	1.229	3648.000
ENSG00000142166	0.007	-0.132	-0.023	-0.241	IFNAR1	0.033	~amyloid_beta+(1|manifest)+cngeneson+pc_mito+brain_region+Sex	1.248	3606.000
ENSG00000205133	0.015	-0.132	-0.015	-0.249	TRIQK	0.051	~amyloid_beta+(1|manifest)+cngeneson+pc_mito+brain_region+Sex	1.547	3053.000
ENSG00000130856	0.061	-0.133	0.023	-0.289	ZNF236	0.113	~amyloid_beta+(1|manifest)+cngeneson+pc_mito+brain_region+Sex	1.679	2859.000
ENSG00000000457	0.002	-0.133	-0.043	-0.222	SCYL3	0.016	~amyloid_beta+(1|manifest)+cngeneson+pc_mito+brain_region+Sex	0.895	4386.000
ENSG00000171729	0.025	-0.133	-0.029	-0.237	TMEM51	0.067	~amyloid_beta+(1|manifest)+cngeneson+pc_mito+brain_region+Sex	0.314	5704.000
ENSG00000165699	0.027	-0.133	0.018	-0.284	TSC1	0.069	~amyloid_beta+(1|manifest)+cngeneson+pc_mito+brain_region+Sex	1.125	3872.000
ENSG00000141429	0.132	-0.133	0.017	-0.283	GALNT1	0.188	~amyloid_beta+(1|manifest)+cngeneson+pc_mito+brain_region+Sex	2.224	2176.000
ENSG00000103047	0.009	-0.133	-0.022	-0.244	TANGO6	0.037	~amyloid_beta+(1|manifest)+cngeneson+pc_mito+brain_region+Sex	0.890	4397.000
ENSG00000151092	0.074	-0.133	0.004	-0.271	NGLY1	0.128	~amyloid_beta+(1|manifest)+cngeneson+pc_mito+brain_region+Sex	1.610	2965.000
ENSG00000128928	0.098	-0.133	0.059	-0.326	IVD	0.153	~amyloid_beta+(1|manifest)+cngeneson+pc_mito+brain_region+Sex	2.251	2152.000
ENSG00000205209	0.101	-0.134	0.051	-0.318	SCGB2B2	0.156	~amyloid_beta+(1|manifest)+cngeneson+pc_mito+brain_region+Sex	1.407	3311.000
ENSG00000064115	0.017	-0.134	-0.021	-0.246	TM7SF3	0.053	~amyloid_beta+(1|manifest)+cngeneson+pc_mito+brain_region+Sex	1.181	3743.000
ENSG00000004487	0.009	-0.134	-0.029	-0.239	KDM1A	0.038	~amyloid_beta+(1|manifest)+cngeneson+pc_mito+brain_region+Sex	1.476	3179.000
ENSG00000169375	0.009	-0.134	0.023	-0.291	SIN3A	0.037	~amyloid_beta+(1|manifest)+cngeneson+pc_mito+brain_region+Sex	1.699	2823.000
ENSG00000270629	0.278	-0.134	0.108	-0.376	NBPF14	0.331	~amyloid_beta+(1|manifest)+cngeneson+pc_mito+brain_region+Sex	3.016	1533.000
ENSG00000185515	0.003	-0.134	-0.053	-0.216	BRCC3	0.019	~amyloid_beta+(1|manifest)+cngeneson+pc_mito+brain_region+Sex	1.352	3408.000
ENSG00000112701	0.125	-0.135	-0.006	-0.263	SENP6	0.181	~amyloid_beta+(1|manifest)+cngeneson+pc_mito+brain_region+Sex	2.572	1833.000
ENSG00000080345	0.039	-0.135	0.000	-0.269	RIF1	0.086	~amyloid_beta+(1|manifest)+cngeneson+pc_mito+brain_region+Sex	0.822	4562.000
ENSG00000012174	0.000	-0.135	-0.060	-0.210	MBTPS2	0.002	~amyloid_beta+(1|manifest)+cngeneson+pc_mito+brain_region+Sex	0.654	4990.000
ENSG00000147119	0.172	-0.135	-0.009	-0.261	CHST7	0.227	~amyloid_beta+(1|manifest)+cngeneson+pc_mito+brain_region+Sex	2.019	2413.000
ENSG00000138750	0.000	-0.135	-0.056	-0.215	NUP54	0.006	~amyloid_beta+(1|manifest)+cngeneson+pc_mito+brain_region+Sex	1.205	3692.000
ENSG00000151292	0.030	-0.136	-0.030	-0.241	CSNK1G3	0.075	~amyloid_beta+(1|manifest)+cngeneson+pc_mito+brain_region+Sex	1.074	3978.000
ENSG00000076067	0.131	-0.136	0.026	-0.297	RBMS2	0.188	~amyloid_beta+(1|manifest)+cngeneson+pc_mito+brain_region+Sex	1.430	3265.000
ENSG00000075568	0.459	-0.136	0.081	-0.352	TMEM131	0.504	~amyloid_beta+(1|manifest)+cngeneson+pc_mito+brain_region+Sex	4.456	903.000
ENSG00000021776	0.017	-0.136	-0.027	-0.245	AQR	0.054	~amyloid_beta+(1|manifest)+cngeneson+pc_mito+brain_region+Sex	0.732	4790.000
ENSG00000178075	0.540	-0.136	0.162	-0.434	GRAMD1C	0.581	~amyloid_beta+(1|manifest)+cngeneson+pc_mito+brain_region+Sex	4.191	989.000
ENSG00000100325	0.008	-0.136	-0.036	-0.235	ASCC2	0.035	~amyloid_beta+(1|manifest)+cngeneson+pc_mito+brain_region+Sex	1.127	3867.000
ENSG00000144451	0.208	-0.136	0.039	-0.311	SPAG16	0.263	~amyloid_beta+(1|manifest)+cngeneson+pc_mito+brain_region+Sex	1.057	4009.000
ENSG00000183580	0.012	-0.136	-0.052	-0.220	FBXL7	0.044	~amyloid_beta+(1|manifest)+cngeneson+pc_mito+brain_region+Sex	3.589	1211.000
ENSG00000164080	0.171	-0.136	0.019	-0.291	RAD54L2	0.226	~amyloid_beta+(1|manifest)+cngeneson+pc_mito+brain_region+Sex	2.981	1551.000
ENSG00000111707	0.006	-0.136	-0.046	-0.226	SUDS3	0.029	~amyloid_beta+(1|manifest)+cngeneson+pc_mito+brain_region+Sex	1.233	3630.000
ENSG00000110090	0.146	-0.136	0.042	-0.314	CPT1A	0.202	~amyloid_beta+(1|manifest)+cngeneson+pc_mito+brain_region+Sex	1.984	2454.000
ENSG00000186073	0.051	-0.136	-0.038	-0.234	C15orf41	0.101	~amyloid_beta+(1|manifest)+cngeneson+pc_mito+brain_region+Sex	1.642	2919.000
ENSG00000165115	0.143	-0.136	0.058	-0.331	KIF27	0.199	~amyloid_beta+(1|manifest)+cngeneson+pc_mito+brain_region+Sex	2.557	1850.000
ENSG00000173889	0.119	-0.136	0.015	-0.287	PHC3	0.175	~amyloid_beta+(1|manifest)+cngeneson+pc_mito+brain_region+Sex	2.086	2328.000
ENSG00000109586	0.503	-0.137	0.113	-0.386	GALNT7	0.547	~amyloid_beta+(1|manifest)+cngeneson+pc_mito+brain_region+Sex	5.105	754.000
ENSG00000198561	0.005	-0.137	-0.043	-0.230	CTNND1	0.027	~amyloid_beta+(1|manifest)+cngeneson+pc_mito+brain_region+Sex	1.152	3808.000
ENSG00000161692	0.073	-0.137	0.041	-0.315	DBF4B	0.126	~amyloid_beta+(1|manifest)+cngeneson+pc_mito+brain_region+Sex	2.384	2009.000
ENSG00000101144	0.569	-0.137	0.204	-0.478	BMP7	0.608	~amyloid_beta+(1|manifest)+cngeneson+pc_mito+brain_region+Sex	3.284	1382.000
ENSG00000149131	0.291	-0.137	0.133	-0.407	SERPING1	0.343	~amyloid_beta+(1|manifest)+cngeneson+pc_mito+brain_region+Sex	6.476	530.000
ENSG00000170348	0.485	-0.137	0.179	-0.453	TMED10	0.530	~amyloid_beta+(1|manifest)+cngeneson+pc_mito+brain_region+Sex	3.172	1441.000
ENSG00000087095	0.113	-0.137	0.005	-0.279	NLK	0.169	~amyloid_beta+(1|manifest)+cngeneson+pc_mito+brain_region+Sex	2.554	1852.000
ENSG00000142949	0.344	-0.137	0.144	-0.419	PTPRF	0.395	~amyloid_beta+(1|manifest)+cngeneson+pc_mito+brain_region+Sex	1.375	3366.000
ENSG00000038219	0.117	-0.137	0.157	-0.432	BOD1L1	0.173	~amyloid_beta+(1|manifest)+cngeneson+pc_mito+brain_region+Sex	0.728	4800.000
ENSG00000102230	0.147	-0.137	0.112	-0.386	PCYT1B	0.202	~amyloid_beta+(1|manifest)+cngeneson+pc_mito+brain_region+Sex	2.082	2334.000
ENSG00000197694	0.244	-0.137	0.050	-0.324	SPTAN1	0.297	~amyloid_beta+(1|manifest)+cngeneson+pc_mito+brain_region+Sex	4.214	974.000
ENSG00000111880	0.404	-0.137	0.090	-0.365	RNGTT	0.451	~amyloid_beta+(1|manifest)+cngeneson+pc_mito+brain_region+Sex	3.804	1119.000
ENSG00000124486	0.040	-0.137	0.020	-0.295	USP9X	0.087	~amyloid_beta+(1|manifest)+cngeneson+pc_mito+brain_region+Sex	2.004	2424.000
ENSG00000196505	0.003	-0.138	-0.053	-0.222	GDAP2	0.021	~amyloid_beta+(1|manifest)+cngeneson+pc_mito+brain_region+Sex	1.150	3815.000
ENSG00000006740	0.002	-0.138	0.030	-0.305	ARHGAP44	0.015	~amyloid_beta+(1|manifest)+cngeneson+pc_mito+brain_region+Sex	0.330	5674.000
ENSG00000172869	0.289	-0.138	0.048	-0.324	DMXL1	0.342	~amyloid_beta+(1|manifest)+cngeneson+pc_mito+brain_region+Sex	2.795	1671.000
ENSG00000167113	0.065	-0.138	0.025	-0.302	COQ4	0.117	~amyloid_beta+(1|manifest)+cngeneson+pc_mito+brain_region+Sex	2.352	2044.000
ENSG00000131089	0.099	-0.138	0.030	-0.307	ARHGEF9	0.155	~amyloid_beta+(1|manifest)+cngeneson+pc_mito+brain_region+Sex	2.222	2181.000
ENSG00000171130	0.418	-0.138	0.145	-0.421	ATP6V0E2	0.464	~amyloid_beta+(1|manifest)+cngeneson+pc_mito+brain_region+Sex	4.239	969.000
ENSG00000143469	0.137	-0.139	-0.004	-0.273	SYT14	0.194	~amyloid_beta+(1|manifest)+cngeneson+pc_mito+brain_region+Sex	0.353	5645.000
ENSG00000168818	0.041	-0.139	0.003	-0.280	STX18	0.088	~amyloid_beta+(1|manifest)+cngeneson+pc_mito+brain_region+Sex	1.069	3991.000
ENSG00000057608	0.002	-0.139	-0.053	-0.225	GDI2	0.018	~amyloid_beta+(1|manifest)+cngeneson+pc_mito+brain_region+Sex	1.224	3658.000
ENSG00000178445	0.012	-0.139	-0.012	-0.267	GLDC	0.044	~amyloid_beta+(1|manifest)+cngeneson+pc_mito+brain_region+Sex	0.762	4711.000
ENSG00000150787	0.022	-0.140	-0.027	-0.252	PTS	0.063	~amyloid_beta+(1|manifest)+cngeneson+pc_mito+brain_region+Sex	1.870	2600.000
ENSG00000198722	0.241	-0.140	0.042	-0.321	UNC13B	0.295	~amyloid_beta+(1|manifest)+cngeneson+pc_mito+brain_region+Sex	1.819	2669.000
ENSG00000173611	0.035	-0.140	-0.028	-0.252	SCAI	0.081	~amyloid_beta+(1|manifest)+cngeneson+pc_mito+brain_region+Sex	1.037	4055.000
ENSG00000177054	0.055	-0.140	0.020	-0.300	ZDHHC13	0.105	~amyloid_beta+(1|manifest)+cngeneson+pc_mito+brain_region+Sex	2.497	1907.000
ENSG00000113391	0.170	-0.140	0.010	-0.291	FAM172A	0.225	~amyloid_beta+(1|manifest)+cngeneson+pc_mito+brain_region+Sex	1.527	3092.000
ENSG00000183166	0.442	-0.140	0.058	-0.338	CALN1	0.487	~amyloid_beta+(1|manifest)+cngeneson+pc_mito+brain_region+Sex	14.938	102.000
ENSG00000215114	0.002	-0.141	-0.064	-0.217	UBXN2B	0.014	~amyloid_beta+(1|manifest)+cngeneson+pc_mito+brain_region+Sex	0.397	5567.000
ENSG00000198862	0.001	-0.141	-0.037	-0.244	LTN1	0.011	~amyloid_beta+(1|manifest)+cngeneson+pc_mito+brain_region+Sex	1.241	3617.000
ENSG00000121749	0.036	-0.141	0.012	-0.294	TBC1D15	0.082	~amyloid_beta+(1|manifest)+cngeneson+pc_mito+brain_region+Sex	1.244	3612.000
ENSG00000163738	0.503	-0.141	0.119	-0.401	MTHFD2L	0.547	~amyloid_beta+(1|manifest)+cngeneson+pc_mito+brain_region+Sex	1.710	2808.000
ENSG00000091129	0.077	-0.141	0.013	-0.295	NRCAM	0.131	~amyloid_beta+(1|manifest)+cngeneson+pc_mito+brain_region+Sex	22.346	38.000
ENSG00000150867	0.826	-0.141	0.313	-0.596	PIP4K2A	0.844	~amyloid_beta+(1|manifest)+cngeneson+pc_mito+brain_region+Sex	6.729	503.000
ENSG00000179455	0.415	-0.142	0.092	-0.376	MKRN3	0.461	~amyloid_beta+(1|manifest)+cngeneson+pc_mito+brain_region+Sex	1.849	2627.000
ENSG00000166037	0.045	-0.142	0.005	-0.289	CEP57	0.094	~amyloid_beta+(1|manifest)+cngeneson+pc_mito+brain_region+Sex	1.426	3273.000
ENSG00000160746	0.018	-0.143	0.015	-0.300	ANO10	0.056	~amyloid_beta+(1|manifest)+cngeneson+pc_mito+brain_region+Sex	1.805	2687.000
ENSG00000155093	0.052	-0.143	-0.010	-0.276	PTPRN2	0.102	~amyloid_beta+(1|manifest)+cngeneson+pc_mito+brain_region+Sex	0.969	4209.000
ENSG00000107020	0.007	-0.143	-0.060	-0.225	PLGRKT	0.033	~amyloid_beta+(1|manifest)+cngeneson+pc_mito+brain_region+Sex	0.755	4726.000
ENSG00000184588	0.144	-0.143	0.005	-0.290	PDE4B	0.200	~amyloid_beta+(1|manifest)+cngeneson+pc_mito+brain_region+Sex	11.014	219.000
ENSG00000136280	0.053	-0.143	-0.040	-0.246	CCM2	0.103	~amyloid_beta+(1|manifest)+cngeneson+pc_mito+brain_region+Sex	1.357	3397.000
ENSG00000008952	0.125	-0.143	0.037	-0.323	SEC62	0.181	~amyloid_beta+(1|manifest)+cngeneson+pc_mito+brain_region+Sex	0.980	4186.000
ENSG00000071575	0.205	-0.143	0.104	-0.390	TRIB2	0.260	~amyloid_beta+(1|manifest)+cngeneson+pc_mito+brain_region+Sex	3.599	1207.000
ENSG00000213079	0.294	-0.143	0.050	-0.336	SCAF8	0.347	~amyloid_beta+(1|manifest)+cngeneson+pc_mito+brain_region+Sex	3.027	1526.000
ENSG00000123384	0.256	-0.143	0.056	-0.342	LRP1	0.310	~amyloid_beta+(1|manifest)+cngeneson+pc_mito+brain_region+Sex	3.520	1241.000
ENSG00000151065	0.023	-0.144	-0.013	-0.274	DCP1B	0.064	~amyloid_beta+(1|manifest)+cngeneson+pc_mito+brain_region+Sex	0.981	4185.000
ENSG00000169860	0.136	-0.144	0.045	-0.332	P2RY1	0.193	~amyloid_beta+(1|manifest)+cngeneson+pc_mito+brain_region+Sex	2.393	1994.000
ENSG00000198363	0.079	-0.144	-0.014	-0.274	ASPH	0.133	~amyloid_beta+(1|manifest)+cngeneson+pc_mito+brain_region+Sex	9.480	299.000
ENSG00000125827	0.029	-0.144	-0.009	-0.279	TMX4	0.073	~amyloid_beta+(1|manifest)+cngeneson+pc_mito+brain_region+Sex	1.625	2946.000
ENSG00000132356	0.021	-0.144	-0.035	-0.253	PRKAA1	0.062	~amyloid_beta+(1|manifest)+cngeneson+pc_mito+brain_region+Sex	1.434	3257.000
ENSG00000256591	0.051	-0.144	0.017	-0.305	[illegible]	0.101	~amyloid_beta+(1|manifest)+cngeneson+pc_mito+brain_region+Sex	1.567	3029.000
ENSG00000196715	0.008	-0.144	-0.054	-0.235	VKORC1L1	0.034	~amyloid_beta+(1|manifest)+cngeneson+pc_mito+brain_region+Sex	1.047	4034.000
ENSG00000123104	0.332	-0.145	0.058	-0.347	ITPR2	0.384	~amyloid_beta+(1|manifest)+cngeneson+pc_mito+brain_region+Sex	33.228	16.000
ENSG00000149231	0.004	-0.145	-0.036	-0.253	CCDC82	0.024	~amyloid_beta+(1|manifest)+cngeneson+pc_mito+brain_region+Sex	1.211	3679.000
ENSG00000148814	0.005	-0.145	-0.032	-0.257	LRRC27	0.025	~amyloid_beta+(1|manifest)+cngeneson+pc_mito+brain_region+Sex	1.262	3577.000
ENSG00000103707	0.000	-0.145	-0.078	-0.211	MTFMT	0.003	~amyloid_beta+(1|manifest)+cngeneson+pc_mito+brain_region+Sex	0.889	4400.000
ENSG00000111412	0.078	-0.145	0.000	-0.290	C12orf49	0.133	~amyloid_beta+(1|manifest)+cngeneson+pc_mito+brain_region+Sex	2.300	2108.000
ENSG00000070366	0.143	-0.145	0.046	-0.335	SMG6	0.199	~amyloid_beta+(1|manifest)+cngeneson+pc_mito+brain_region+Sex	2.141	2270.000
ENSG00000068650	0.536	-0.145	0.125	-0.415	ATP11A	0.577	~amyloid_beta+(1|manifest)+cngeneson+pc_mito+brain_region+Sex	5.920	603.000
ENSG00000131848	0.261	-0.145	0.066	-0.356	ZSCAN5A	0.315	~amyloid_beta+(1|manifest)+cngeneson+pc_mito+brain_region+Sex	1.762	2733.000
ENSG00000156162	0.001	-0.145	-0.052	-0.238	DPY19L4	0.010	~amyloid_beta+(1|manifest)+cngeneson+pc_mito+brain_region+Sex	0.922	4322.000
ENSG00000118762	0.635	-0.145	0.208	-0.498	PKD2	0.669	~amyloid_beta+(1|manifest)+cngeneson+pc_mito+brain_region+Sex	8.725	327.000
ENSG00000115896	0.337	-0.145	0.036	-0.326	PLCL1	0.389	~amyloid_beta+(1|manifest)+cngeneson+pc_mito+brain_region+Sex	1.688	2847.000
ENSG00000182771	0.266	-0.145	0.036	-0.327	GRID1	0.320	~amyloid_beta+(1|manifest)+cngeneson+pc_mito+brain_region+Sex	3.136	1461.000
ENSG00000128513	0.013	-0.145	-0.018	-0.273	POT1	0.047	~amyloid_beta+(1|manifest)+cngeneson+pc_mito+brain_region+Sex	1.314	3474.000
ENSG00000206562	0.008	-0.146	-0.035	-0.256	METTL6	0.036	~amyloid_beta+(1|manifest)+cngeneson+pc_mito+brain_region+Sex	0.804	4620.000
ENSG00000165714	0.002	-0.146	-0.043	-0.248	BORCS5	0.017	~amyloid_beta+(1|manifest)+cngeneson+pc_mito+brain_region+Sex	0.505	5339.000
ENSG00000171798	0.586	-0.146	0.358	-0.649	KNDC1	0.623	~amyloid_beta+(1|manifest)+cngeneson+pc_mito+brain_region+Sex	8.133	368.000
ENSG00000082212	0.017	-0.146	-0.037	-0.255	ME2	0.055	~amyloid_beta+(1|manifest)+cngeneson+pc_mito+brain_region+Sex	0.307	5720.000
ENSG00000122863	0.342	-0.146	0.089	-0.381	CHST3	0.394	~amyloid_beta+(1|manifest)+cngeneson+pc_mito+brain_region+Sex	4.457	901.000
ENSG00000173852	0.574	-0.146	0.187	-0.479	DPY19L1	0.612	~amyloid_beta+(1|manifest)+cngeneson+pc_mito+brain_region+Sex	7.914	383.000
ENSG00000101464	0.100	-0.146	-0.007	-0.285	PIGU	0.155	~amyloid_beta+(1|manifest)+cngeneson+pc_mito+brain_region+Sex	1.264	3570.000
ENSG00000111224	0.016	-0.146	-0.030	-0.261	PARP11	0.053	~amyloid_beta+(1|manifest)+cngeneson+pc_mito+brain_region+Sex	1.979	2458.000
ENSG00000112624	0.170	-0.146	0.063	-0.355	BICRAL	0.226	~amyloid_beta+(1|manifest)+cngeneson+pc_mito+brain_region+Sex	2.830	1646.000
ENSG00000177453	0.246	-0.146	0.018	-0.311	NIM1K	0.300	~amyloid_beta+(1|manifest)+cngeneson+pc_mito+brain_region+Sex	1.893	2565.000
ENSG00000110427	0.094	-0.147	-0.005	-0.288	KIAA1549L	0.149	~amyloid_beta+(1|manifest)+cngeneson+pc_mito+brain_region+Sex	0.511	5327.000
ENSG00000165443	0.699	-0.147	0.213	-0.506	PHYHIPL	0.728	~amyloid_beta+(1|manifest)+cngeneson+pc_mito+brain_region+Sex	2.547	1859.000
ENSG00000111142	0.002	-0.147	-0.066	-0.228	METAP2	0.017	~amyloid_beta+(1|manifest)+cngeneson+pc_mito+brain_region+Sex	0.963	4216.000
ENSG00000165495	0.703	-0.147	0.202	-0.496	PKNOX2	0.732	~amyloid_beta+(1|manifest)+cngeneson+pc_mito+brain_region+Sex	10.876	228.000
ENSG00000138078	0.093	-0.147	0.040	-0.335	PREPL	0.148	~amyloid_beta+(1|manifest)+cngeneson+pc_mito+brain_region+Sex	2.412	1972.000
ENSG00000184083	0.044	-0.147	-0.026	-0.268	FAM120C	0.093	~amyloid_beta+(1|manifest)+cngeneson+pc_mito+brain_region+Sex	1.306	3492.000
ENSG00000138685	0.406	-0.147	0.075	-0.369	FGF2	0.453	~amyloid_beta+(1|manifest)+cngeneson+pc_mito+brain_region+Sex	4.366	929.000
ENSG00000186166	0.099	-0.147	0.033	-0.327	CCDC84	0.155	~amyloid_beta+(1|manifest)+cngeneson+pc_mito+brain_region+Sex	2.434	1952.000
ENSG00000110888	0.128	-0.147	0.050	-0.345	CAPRIN2	0.184	~amyloid_beta+(1|manifest)+cngeneson+pc_mito+brain_region+Sex	2.549	1856.000
ENSG00000109113	0.243	-0.147	0.109	-0.404	RAB34	0.297	~amyloid_beta+(1|manifest)+cngeneson+pc_mito+brain_region+Sex	4.195	987.000
ENSG00000066933	0.547	-0.147	0.133	-0.428	MYO9A	0.588	~amyloid_beta+(1|manifest)+cngeneson+pc_mito+brain_region+Sex	9.701	283.000
ENSG00000163788	0.450	-0.148	0.080	-0.376	SNRK	0.495	~amyloid_beta+(1|manifest)+cngeneson+pc_mito+brain_region+Sex	2.799	1667.000
ENSG00000152990	0.138	-0.148	0.003	-0.298	ADGRA3	0.194	~amyloid_beta+(1|manifest)+cngeneson+pc_mito+brain_region+Sex	3.899	1090.000
ENSG00000158458	0.568	-0.148	0.136	-0.431	NRG2	0.607	~amyloid_beta+(1|manifest)+cngeneson+pc_mito+brain_region+Sex	5.834	620.000
ENSG00000118058	0.034	-0.148	0.010	-0.306	KMT2A	0.080	~amyloid_beta+(1|manifest)+cngeneson+pc_mito+brain_region+Sex	1.589	3000.000
ENSG00000176225	0.012	-0.148	-0.038	-0.258	RTTN	0.045	~amyloid_beta+(1|manifest)+cngeneson+pc_mito+brain_region+Sex	0.986	4178.000
ENSG00000144649	0.018	-0.148	-0.012	-0.284	GASK1A	0.056	~amyloid_beta+(1|manifest)+cngeneson+pc_mito+brain_region+Sex	1.323	3456.000
ENSG00000111860	0.318	-0.148	0.055	-0.351	CEP85L	0.370	~amyloid_beta+(1|manifest)+cngeneson+pc_mito+brain_region+Sex	3.901	1089.000
ENSG00000137497	0.260	-0.148	0.079	-0.375	NUMA1	0.313	~amyloid_beta+(1|manifest)+cngeneson+pc_mito+brain_region+Sex	2.734	1713.000
ENSG00000067191	0.191	-0.148	0.118	-0.415	CACNB1	0.245	~amyloid_beta+(1|manifest)+cngeneson+pc_mito+brain_region+Sex	3.122	1469.000
ENSG00000149658	0.472	-0.149	0.205	-0.503	YTHDF1	0.517	~amyloid_beta+(1|manifest)+cngeneson+pc_mito+brain_region+Sex	5.012	771.000
ENSG00000111850	0.184	-0.149	0.105	-0.403	SMIM8	0.239	~amyloid_beta+(1|manifest)+cngeneson+pc_mito+brain_region+Sex	3.251	1395.000
ENSG00000105499	0.160	-0.149	0.088	-0.386	PLA2G4C	0.215	~amyloid_beta+(1|manifest)+cngeneson+pc_mito+brain_region+Sex	2.325	2075.000
ENSG00000196338	0.098	-0.149	0.045	-0.344	NLGN3	0.153	~amyloid_beta+(1|manifest)+cngeneson+pc_mito+brain_region+Sex	1.189	3730.000
ENSG00000125846	0.010	-0.149	-0.029	-0.270	ZNF133	0.040	~amyloid_beta+(1|manifest)+cngeneson+pc_mito+brain_region+Sex	1.004	4129.000
ENSG00000108010	0.005	-0.149	-0.046	-0.253	GLRX3	0.028	~amyloid_beta+(1|manifest)+cngeneson+pc_mito+brain_region+Sex	2.039	2382.000
ENSG00000135318	0.179	-0.150	0.004	-0.304	NT5E	0.233	~amyloid_beta+(1|manifest)+cngeneson+pc_mito+brain_region+Sex	1.987	2451.000
ENSG00000198920	0.003	-0.150	-0.057	-0.242	KIAA0753	0.021	~amyloid_beta+(1|manifest)+cngeneson+pc_mito+brain_region+Sex	1.025	4091.000
ENSG00000119138	0.013	-0.150	0.031	-0.330	KLF9	0.046	~amyloid_beta+(1|manifest)+cngeneson+pc_mito+brain_region+Sex	0.806	4613.000
ENSG00000157106	0.134	-0.150	0.033	-0.332	SMG1	0.191	~amyloid_beta+(1|manifest)+cngeneson+pc_mito+brain_region+Sex	3.414	1307.000
ENSG00000180233	0.301	-0.150	0.078	-0.378	ZNRF2	0.353	~amyloid_beta+(1|manifest)+cngeneson+pc_mito+brain_region+Sex	5.121	745.000
ENSG00000089682	0.032	-0.150	-0.027	-0.273	RBM41	0.076	~amyloid_beta+(1|manifest)+cngeneson+pc_mito+brain_region+Sex	1.038	4051.000
ENSG00000130638	0.168	-0.150	0.010	-0.310	ATXN10	0.223	~amyloid_beta+(1|manifest)+cngeneson+pc_mito+brain_region+Sex	3.137	1460.000
ENSG00000114670	0.186	-0.150	0.032	-0.332	NEK11	0.240	~amyloid_beta+(1|manifest)+cngeneson+pc_mito+brain_region+Sex	0.881	4418.000
ENSG00000101901	0.006	-0.150	-0.053	-0.247	ALG13	0.029	~amyloid_beta+(1|manifest)+cngeneson+pc_mito+brain_region+Sex	0.788	4655.000
ENSG00000162512	0.316	-0.150	0.133	-0.434	SDC3	0.368	~amyloid_beta+(1|manifest)+cngeneson+pc_mito+brain_region+Sex	8.163	364.000
ENSG00000186472	0.843	-0.150	0.355	-0.655	PCLO	0.858	~amyloid_beta+(1|manifest)+cngeneson+pc_mito+brain_region+Sex	5.085	757.000
ENSG00000119684	0.047	-0.150	0.024	-0.325	MLH3	0.095	~amyloid_beta+(1|manifest)+cngeneson+pc_mito+brain_region+Sex	1.843	2636.000
ENSG00000171094	0.553	-0.151	0.200	-0.501	ALK	0.593	~amyloid_beta+(1|manifest)+cngeneson+pc_mito+brain_region+Sex	4.825	815.000
ENSG00000120925	0.097	-0.151	0.026	-0.327	RNF170	0.152	~amyloid_beta+(1|manifest)+cngeneson+pc_mito+brain_region+Sex	2.169	2237.000
ENSG00000172888	0.125	-0.151	0.192	-0.493	ZNF621	0.181	~amyloid_beta+(1|manifest)+cngeneson+pc_mito+brain_region+Sex	3.115	1475.000
ENSG00000093144	0.003	-0.151	-0.061	-0.241	ECHDC1	0.018	~amyloid_beta+(1|manifest)+cngeneson+pc_mito+brain_region+Sex	1.163	3783.000
ENSG00000166532	0.230	-0.151	0.055	-0.357	RIMKLB	0.285	~amyloid_beta+(1|manifest)+cngeneson+pc_mito+brain_region+Sex	2.898	1605.000
ENSG00000143919	0.333	-0.151	0.048	-0.351	CAMKMT	0.384	~amyloid_beta+(1|manifest)+cngeneson+pc_mito+brain_region+Sex	1.887	2581.000
ENSG00000174839	0.006	-0.151	-0.036	-0.267	DENND6A	0.029	~amyloid_beta+(1|manifest)+cngeneson+pc_mito+brain_region+Sex	1.085	3961.000
ENSG00000117360	0.046	-0.152	0.005	-0.308	PRPF3	0.095	~amyloid_beta+(1|manifest)+cngeneson+pc_mito+brain_region+Sex	2.063	2354.000
ENSG00000105767	0.235	-0.152	0.085	-0.388	CADM4	0.289	~amyloid_beta+(1|manifest)+cngeneson+pc_mito+brain_region+Sex	2.333	2065.000
ENSG00000275052	0.067	-0.152	0.012	-0.315	PPP4R3B	0.120	~amyloid_beta+(1|manifest)+cngeneson+pc_mito+brain_region+Sex	2.570	1837.000
ENSG00000171714	0.013	-0.152	0.258	-0.561	ANO5	0.046	~amyloid_beta+(1|manifest)+cngeneson+pc_mito+brain_region+Sex	10.918	227.000
ENSG00000146833	0.001	-0.152	-0.071	-0.233	TRIM4	0.013	~amyloid_beta+(1|manifest)+cngeneson+pc_mito+brain_region+Sex	1.021	4096.000
ENSG00000168591	0.073	-0.152	0.015	-0.319	TMUB2	0.127	~amyloid_beta+(1|manifest)+cngeneson+pc_mito+brain_region+Sex	2.314	2086.000
ENSG00000137261	0.557	-0.152	0.183	-0.487	KIAA0319	0.596	~amyloid_beta+(1|manifest)+cngeneson+pc_mito+brain_region+Sex	2.160	2249.000
ENSG00000144369	0.554	-0.152	0.133	-0.437	FAM171B	0.594	~amyloid_beta+(1|manifest)+cngeneson+pc_mito+brain_region+Sex	7.283	445.000
ENSG00000165650	0.019	-0.153	-0.032	-0.273	PDZD8	0.058	~amyloid_beta+(1|manifest)+cngeneson+pc_mito+brain_region+Sex	2.027	2399.000
ENSG00000118217	0.231	-0.153	0.034	-0.339	ATF6	0.285	~amyloid_beta+(1|manifest)+cngeneson+pc_mito+brain_region+Sex	1.621	2951.000
ENSG00000136631	0.192	-0.153	0.014	-0.320	VPS45	0.246	~amyloid_beta+(1|manifest)+cngeneson+pc_mito+brain_region+Sex	0.888	4402.000
ENSG00000113296	0.059	-0.153	0.060	-0.366	THBS4	0.110	~amyloid_beta+(1|manifest)+cngeneson+pc_mito+brain_region+Sex	1.461	3205.000
ENSG00000065243	0.034	-0.153	-0.002	-0.304	PKN2	0.080	~amyloid_beta+(1|manifest)+cngeneson+pc_mito+brain_region+Sex	1.739	2766.000
ENSG00000135241	0.765	-0.153	0.266	-0.572	PNPLA8	0.788	~amyloid_beta+(1|manifest)+cngeneson+pc_mito+brain_region+Sex	10.412	252.000
ENSG00000127914	0.237	-0.153	0.099	-0.405	AKAP9	0.291	~amyloid_beta+(1|manifest)+cngeneson+pc_mito+brain_region+Sex	3.150	1452.000
ENSG00000198087	0.040	-0.154	0.042	-0.349	CD2AP	0.087	~amyloid_beta+(1|manifest)+cngeneson+pc_mito+brain_region+Sex	2.642	1776.000
ENSG00000147133	0.008	-0.154	-0.052	-0.256	TAF1	0.035	~amyloid_beta+(1|manifest)+cngeneson+pc_mito+brain_region+Sex	1.005	4127.000
ENSG00000168575	0.741	-0.154	0.235	-0.544	SLC20A2	0.767	~amyloid_beta+(1|manifest)+cngeneson+pc_mito+brain_region+Sex	6.363	542.000
ENSG00000122482	0.305	-0.154	0.049	-0.357	ZNF644	0.357	~amyloid_beta+(1|manifest)+cngeneson+pc_mito+brain_region+Sex	3.097	1485.000
ENSG00000108654	0.611	-0.154	0.218	-0.527	DDX5	0.647	~amyloid_beta+(1|manifest)+cngeneson+pc_mito+brain_region+Sex	12.861	154.000
ENSG00000170035	0.487	-0.154	0.096	-0.405	UBE2E3	0.531	~amyloid_beta+(1|manifest)+cngeneson+pc_mito+brain_region+Sex	1.055	4013.000
ENSG00000160216	0.059	-0.154	0.038	-0.347	AGPAT3	0.110	~amyloid_beta+(1|manifest)+cngeneson+pc_mito+brain_region+Sex	1.998	2436.000
ENSG00000101654	0.016	-0.155	-0.032	-0.277	RNMT	0.052	~amyloid_beta+(1|manifest)+cngeneson+pc_mito+brain_region+Sex	0.949	4256.000
ENSG00000117868	0.118	-0.155	0.028	-0.337	ESYT2	0.174	~amyloid_beta+(1|manifest)+cngeneson+pc_mito+brain_region+Sex	2.649	1769.000
ENSG00000164944	0.002	-0.155	-0.066	-0.244	VIRMA	0.016	~amyloid_beta+(1|manifest)+cngeneson+pc_mito+brain_region+Sex	1.261	3578.000
ENSG00000138613	0.043	-0.155	-0.019	-0.291	APH1B	0.091	~amyloid_beta+(1|manifest)+cngeneson+pc_mito+brain_region+Sex	1.527	3091.000
ENSG00000183775	0.022	-0.155	-0.060	-0.250	KCTD16	0.062	~amyloid_beta+(1|manifest)+cngeneson+pc_mito+brain_region+Sex	0.614	5099.000
ENSG00000111596	0.205	-0.155	0.041	-0.351	CNOT2	0.259	~amyloid_beta+(1|manifest)+cngeneson+pc_mito+brain_region+Sex	0.936	4287.000
ENSG00000152061	0.223	-0.155	0.030	-0.340	RABGAP1L	0.278	~amyloid_beta+(1|manifest)+cngeneson+pc_mito+brain_region+Sex	4.824	816.000
ENSG00000197959	0.708	-0.156	0.229	-0.540	DNM3	0.737	~amyloid_beta+(1|manifest)+cngeneson+pc_mito+brain_region+Sex	12.261	168.000
ENSG00000111877	0.026	-0.156	-0.025	-0.287	MCM9	0.068	~amyloid_beta+(1|manifest)+cngeneson+pc_mito+brain_region+Sex	2.052	2366.000
ENSG00000132434	0.043	-0.156	-0.010	-0.302	LANCL2	0.091	~amyloid_beta+(1|manifest)+cngeneson+pc_mito+brain_region+Sex	1.043	4041.000
ENSG00000090621	0.091	-0.156	0.036	-0.349	PABPC4	0.146	~amyloid_beta+(1|manifest)+cngeneson+pc_mito+brain_region+Sex	1.668	2872.000
ENSG00000146282	0.035	-0.156	0.003	-0.315	RARS2	0.081	~amyloid_beta+(1|manifest)+cngeneson+pc_mito+brain_region+Sex	0.622	5080.000
ENSG00000107611	0.037	-0.156	-0.048	-0.265	CUBN	0.083	~amyloid_beta+(1|manifest)+cngeneson+pc_mito+brain_region+Sex	1.144	3827.000
ENSG00000072803	0.165	-0.157	0.018	-0.331	FBXW11	0.221	~amyloid_beta+(1|manifest)+cngeneson+pc_mito+brain_region+Sex	2.215	2184.000
ENSG00000147601	0.005	-0.157	-0.036	-0.278	TERF1	0.028	~amyloid_beta+(1|manifest)+cngeneson+pc_mito+brain_region+Sex	1.490	3157.000
ENSG00000166450	0.072	-0.157	0.004	-0.318	PRTG	0.126	~amyloid_beta+(1|manifest)+cngeneson+pc_mito+brain_region+Sex	1.650	2906.000
ENSG00000085224	0.132	-0.157	0.009	-0.323	ATRX	0.188	~amyloid_beta+(1|manifest)+cngeneson+pc_mito+brain_region+Sex	3.207	1421.000
ENSG00000109046	0.400	-0.157	0.090	-0.404	WSB1	0.448	~amyloid_beta+(1|manifest)+cngeneson+pc_mito+brain_region+Sex	13.608	132.000
ENSG00000137504	0.112	-0.157	0.034	-0.348	CREBZF	0.168	~amyloid_beta+(1|manifest)+cngeneson+pc_mito+brain_region+Sex	2.876	1621.000
ENSG00000167522	0.329	-0.157	0.068	-0.383	ANKRD11	0.381	~amyloid_beta+(1|manifest)+cngeneson+pc_mito+brain_region+Sex	1.847	2630.000
ENSG00000234444	0.012	-0.158	-0.002	-0.314	ZNF736	0.044	~amyloid_beta+(1|manifest)+cngeneson+pc_mito+brain_region+Sex	0.448	5459.000
ENSG00000186868	0.266	-0.158	0.056	-0.371	MAPT	0.320	~amyloid_beta+(1|manifest)+cngeneson+pc_mito+brain_region+Sex	11.450	201.000
ENSG00000148655	0.885	-0.158	0.488	-0.804	LRMDA	0.898	~amyloid_beta+(1|manifest)+cngeneson+pc_mito+brain_region+Sex	12.889	152.000
ENSG00000083535	0.102	-0.158	-0.013	-0.303	PIBF1	0.158	~amyloid_beta+(1|manifest)+cngeneson+pc_mito+brain_region+Sex	2.544	1862.000
ENSG00000117569	0.159	-0.158	0.023	-0.339	PTBP2	0.214	~amyloid_beta+(1|manifest)+cngeneson+pc_mito+brain_region+Sex	2.197	2205.000
ENSG00000070018	0.272	-0.159	0.098	-0.415	LRP6	0.325	~amyloid_beta+(1|manifest)+cngeneson+pc_mito+brain_region+Sex	3.033	1523.000
ENSG00000101413	0.002	-0.159	-0.065	-0.252	RPRD1B	0.016	~amyloid_beta+(1|manifest)+cngeneson+pc_mito+brain_region+Sex	1.163	3780.000
ENSG00000197965	0.275	-0.159	0.065	-0.383	MPZL1	0.328	~amyloid_beta+(1|manifest)+cngeneson+pc_mito+brain_region+Sex	4.546	883.000
ENSG00000173320	0.030	-0.159	-0.002	-0.316	STOX2	0.075	~amyloid_beta+(1|manifest)+cngeneson+pc_mito+brain_region+Sex	1.457	3209.000
ENSG00000211456	0.042	-0.159	-0.009	-0.309	SACM1L	0.090	~amyloid_beta+(1|manifest)+cngeneson+pc_mito+brain_region+Sex	3.028	1525.000
ENSG00000102466	0.225	-0.159	0.013	-0.331	FGF14	0.280	~amyloid_beta+(1|manifest)+cngeneson+pc_mito+brain_region+Sex	1.902	2551.000
ENSG00000137478	0.349	-0.159	0.062	-0.380	FCHSD2	0.399	~amyloid_beta+(1|manifest)+cngeneson+pc_mito+brain_region+Sex	5.147	739.000
ENSG00000113240	0.021	-0.159	-0.030	-0.288	CLK4	0.061	~amyloid_beta+(1|manifest)+cngeneson+pc_mito+brain_region+Sex	1.573	3018.000
ENSG00000144224	0.053	-0.159	0.038	-0.356	UBXN4	0.103	~amyloid_beta+(1|manifest)+cngeneson+pc_mito+brain_region+Sex	2.779	1683.000
ENSG00000197601	0.143	-0.159	0.084	-0.403	FAR1	0.199	~amyloid_beta+(1|manifest)+cngeneson+pc_mito+brain_region+Sex	1.048	4030.000
ENSG00000121766	0.001	-0.160	-0.080	-0.239	ZCCHC17	0.008	~amyloid_beta+(1|manifest)+cngeneson+pc_mito+brain_region+Sex	1.001	4134.000
ENSG00000143514	0.096	-0.160	0.000	-0.320	TP53BP2	0.151	~amyloid_beta+(1|manifest)+cngeneson+pc_mito+brain_region+Sex	1.912	2541.000
ENSG00000139178	0.534	-0.160	0.317	-0.637	C1RL	0.575	~amyloid_beta+(1|manifest)+cngeneson+pc_mito+brain_region+Sex	10.628	238.000
ENSG00000082068	0.137	-0.160	-0.005	-0.315	WDR70	0.194	~amyloid_beta+(1|manifest)+cngeneson+pc_mito+brain_region+Sex	3.051	1508.000
ENSG00000118518	0.005	-0.160	-0.055	-0.266	RNF146	0.027	~amyloid_beta+(1|manifest)+cngeneson+pc_mito+brain_region+Sex	1.045	4036.000
ENSG00000169756	0.698	-0.161	0.217	-0.538	LIMS1	0.727	~amyloid_beta+(1|manifest)+cngeneson+pc_mito+brain_region+Sex	11.112	215.000
ENSG00000136874	0.022	-0.161	-0.032	-0.289	STX17	0.063	~amyloid_beta+(1|manifest)+cngeneson+pc_mito+brain_region+Sex	1.116	3896.000
ENSG00000129317	0.011	-0.161	-0.053	-0.268	PUS7L	0.042	~amyloid_beta+(1|manifest)+cngeneson+pc_mito+brain_region+Sex	0.453	5448.000
ENSG00000118965	0.044	-0.161	0.020	-0.341	WDR35	0.093	~amyloid_beta+(1|manifest)+cngeneson+pc_mito+brain_region+Sex	1.481	3173.000
ENSG00000170017	0.212	-0.161	0.020	-0.341	ALCAM	0.266	~amyloid_beta+(1|manifest)+cngeneson+pc_mito+brain_region+Sex	2.084	2329.000
ENSG00000198162	0.072	-0.161	-0.023	-0.299	MAN1A2	0.126	~amyloid_beta+(1|manifest)+cngeneson+pc_mito+brain_region+Sex	2.399	1984.000
ENSG00000170430	0.160	-0.161	0.032	-0.354	MGMT	0.215	~amyloid_beta+(1|manifest)+cngeneson+pc_mito+brain_region+Sex	0.428	5511.000
ENSG00000100629	0.209	-0.161	0.034	-0.356	CEP128	0.263	~amyloid_beta+(1|manifest)+cngeneson+pc_mito+brain_region+Sex	1.350	3412.000
ENSG00000116678	0.185	-0.161	0.000	-0.322	LEPR	0.240	~amyloid_beta+(1|manifest)+cngeneson+pc_mito+brain_region+Sex	1.892	2569.000
ENSG00000100330	0.252	-0.161	0.031	-0.354	MTMR3	0.306	~amyloid_beta+(1|manifest)+cngeneson+pc_mito+brain_region+Sex	6.140	574.000
ENSG00000164619	0.156	-0.161	-0.009	-0.314	BMPER	0.212	~amyloid_beta+(1|manifest)+cngeneson+pc_mito+brain_region+Sex	1.201	3704.000
ENSG00000107771	0.281	-0.161	0.040	-0.363	CCSER2	0.334	~amyloid_beta+(1|manifest)+cngeneson+pc_mito+brain_region+Sex	5.346	702.000
ENSG00000107929	0.064	-0.162	-0.010	-0.313	LARP4B	0.116	~amyloid_beta+(1|manifest)+cngeneson+pc_mito+brain_region+Sex	2.062	2355.000
ENSG00000150938	0.648	-0.162	0.183	-0.506	CRIM1	0.680	~amyloid_beta+(1|manifest)+cngeneson+pc_mito+brain_region+Sex	5.880	611.000
ENSG00000088854	0.044	-0.162	-0.026	-0.298	C20orf194	0.092	~amyloid_beta+(1|manifest)+cngeneson+pc_mito+brain_region+Sex	8.256	360.000
ENSG00000213047	0.009	-0.162	-0.037	-0.287	DENND1B	0.037	~amyloid_beta+(1|manifest)+cngeneson+pc_mito+brain_region+Sex	0.635	5043.000
ENSG00000168038	0.309	-0.162	0.045	-0.369	ULK4	0.362	~amyloid_beta+(1|manifest)+cngeneson+pc_mito+brain_region+Sex	2.251	2153.000
ENSG00000145623	0.919	-0.162	0.613	-0.938	OSMR	0.928	~amyloid_beta+(1|manifest)+cngeneson+pc_mito+brain_region+Sex	22.179	40.000
ENSG00000164597	0.344	-0.162	0.061	-0.385	COG5	0.395	~amyloid_beta+(1|manifest)+cngeneson+pc_mito+brain_region+Sex	5.717	643.000
ENSG00000150687	0.091	-0.162	-0.036	-0.288	PRSS23	0.146	~amyloid_beta+(1|manifest)+cngeneson+pc_mito+brain_region+Sex	2.350	2049.000
ENSG00000135919	0.048	-0.163	-0.032	-0.293	SERPINE2	0.097	~amyloid_beta+(1|manifest)+cngeneson+pc_mito+brain_region+Sex	0.838	4521.000
ENSG00000169933	0.593	-0.163	0.215	-0.541	FRMPD4	0.630	~amyloid_beta+(1|manifest)+cngeneson+pc_mito+brain_region+Sex	1.836	2647.000
ENSG00000130224	0.002	-0.163	0.003	-0.329	LRCH2	0.015	~amyloid_beta+(1|manifest)+cngeneson+pc_mito+brain_region+Sex	0.010	5980.000
ENSG00000154319	0.175	-0.163	0.007	-0.333	FAM167A	0.229	~amyloid_beta+(1|manifest)+cngeneson+pc_mito+brain_region+Sex	1.009	4119.000
ENSG00000113273	0.275	-0.163	0.023	-0.350	ARSB	0.329	~amyloid_beta+(1|manifest)+cngeneson+pc_mito+brain_region+Sex	2.576	1830.000
ENSG00000151665	0.002	-0.163	-0.067	-0.260	PIGF	0.015	~amyloid_beta+(1|manifest)+cngeneson+pc_mito+brain_region+Sex	0.531	5282.000
ENSG00000162885	0.008	-0.164	-0.039	-0.289	B3GALNT2	0.034	~amyloid_beta+(1|manifest)+cngeneson+pc_mito+brain_region+Sex	1.247	3608.000
ENSG00000106100	0.254	-0.164	0.095	-0.423	NOD1	0.308	~amyloid_beta+(1|manifest)+cngeneson+pc_mito+brain_region+Sex	6.782	495.000
ENSG00000101353	0.002	-0.164	-0.056	-0.271	MROH8	0.015	~amyloid_beta+(1|manifest)+cngeneson+pc_mito+brain_region+Sex	0.837	4525.000
ENSG00000174373	0.109	-0.164	-0.009	-0.320	RALGAPA1	0.165	~amyloid_beta+(1|manifest)+cngeneson+pc_mito+brain_region+Sex	2.625	1795.000
ENSG00000164167	0.002	-0.164	-0.048	-0.280	LSM6	0.016	~amyloid_beta+(1|manifest)+cngeneson+pc_mito+brain_region+Sex	1.660	2887.000
ENSG00000107036	0.182	-0.164	0.008	-0.336	RIC1	0.236	~amyloid_beta+(1|manifest)+cngeneson+pc_mito+brain_region+Sex	2.852	1632.000
ENSG00000175054	0.023	-0.164	-0.028	-0.300	ATR	0.065	~amyloid_beta+(1|manifest)+cngeneson+pc_mito+brain_region+Sex	2.154	2255.000
ENSG00000036530	0.161	-0.164	0.091	-0.419	CYP46A1	0.216	~amyloid_beta+(1|manifest)+cngeneson+pc_mito+brain_region+Sex	2.439	1942.000
ENSG00000159579	0.009	-0.165	-0.051	-0.279	RSPRY1	0.037	~amyloid_beta+(1|manifest)+cngeneson+pc_mito+brain_region+Sex	1.526	3094.000
ENSG00000047932	0.011	-0.165	-0.060	-0.269	GOPC	0.041	~amyloid_beta+(1|manifest)+cngeneson+pc_mito+brain_region+Sex	1.305	3497.000
ENSG00000119471	0.280	-0.165	0.088	-0.418	HSDL2	0.333	~amyloid_beta+(1|manifest)+cngeneson+pc_mito+brain_region+Sex	3.695	1165.000
ENSG00000180488	0.001	-0.165	-0.085	-0.245	MIGA1	0.008	~amyloid_beta+(1|manifest)+cngeneson+pc_mito+brain_region+Sex	1.220	3662.000
ENSG00000133997	0.000	-0.165	-0.116	-0.215	MED6	0.000	~amyloid_beta+(1|manifest)+cngeneson+pc_mito+brain_region+Sex	0.536	5267.000
ENSG00000004866	0.013	-0.165	-0.057	-0.274	ST7	0.047	~amyloid_beta+(1|manifest)+cngeneson+pc_mito+brain_region+Sex	1.205	3693.000
ENSG00000146350	0.302	-0.166	0.086	-0.417	TBC1D32	0.355	~amyloid_beta+(1|manifest)+cngeneson+pc_mito+brain_region+Sex	0.425	5518.000
ENSG00000145743	0.086	-0.166	-0.015	-0.316	FBXL17	0.140	~amyloid_beta+(1|manifest)+cngeneson+pc_mito+brain_region+Sex	2.931	1586.000
ENSG00000172264	0.802	-0.166	0.348	-0.680	MACROD2	0.822	~amyloid_beta+(1|manifest)+cngeneson+pc_mito+brain_region+Sex	6.501	524.000
ENSG00000166405	0.197	-0.166	0.041	-0.373	RIC3	0.251	~amyloid_beta+(1|manifest)+cngeneson+pc_mito+brain_region+Sex	3.553	1224.000
ENSG00000144847	0.384	-0.166	0.075	-0.408	IGSF11	0.434	~amyloid_beta+(1|manifest)+cngeneson+pc_mito+brain_region+Sex	1.864	2610.000
ENSG00000100614	0.008	-0.167	-0.063	-0.270	PPM1A	0.036	~amyloid_beta+(1|manifest)+cngeneson+pc_mito+brain_region+Sex	1.523	3102.000
ENSG00000020426	0.021	-0.167	-0.034	-0.299	MNAT1	0.061	~amyloid_beta+(1|manifest)+cngeneson+pc_mito+brain_region+Sex	1.608	2969.000
ENSG00000113593	0.000	-0.167	-0.097	-0.237	PPWD1	0.002	~amyloid_beta+(1|manifest)+cngeneson+pc_mito+brain_region+Sex	0.826	4547.000
ENSG00000079246	0.287	-0.167	0.044	-0.378	XRCC5	0.340	~amyloid_beta+(1|manifest)+cngeneson+pc_mito+brain_region+Sex	4.738	834.000
ENSG00000198838	0.026	-0.167	-0.044	-0.290	RYR3	0.069	~amyloid_beta+(1|manifest)+cngeneson+pc_mito+brain_region+Sex	8.913	321.000
ENSG00000124596	0.112	-0.167	0.006	-0.340	OARD1	0.168	~amyloid_beta+(1|manifest)+cngeneson+pc_mito+brain_region+Sex	1.382	3353.000
ENSG00000163872	0.009	-0.167	-0.060	-0.275	YEATS2	0.037	~amyloid_beta+(1|manifest)+cngeneson+pc_mito+brain_region+Sex	1.273	3558.000
ENSG00000108984	0.273	-0.167	0.054	-0.389	MAP2K6	0.326	~amyloid_beta+(1|manifest)+cngeneson+pc_mito+brain_region+Sex	4.316	945.000
ENSG00000204120	0.214	-0.167	0.019	-0.353	GIGYF2	0.268	~amyloid_beta+(1|manifest)+cngeneson+pc_mito+brain_region+Sex	4.150	1000.000
ENSG00000100505	0.068	-0.167	-0.017	-0.318	TRIM9	0.121	~amyloid_beta+(1|manifest)+cngeneson+pc_mito+brain_region+Sex	4.889	801.000
ENSG00000147588	0.645	-0.167	0.285	-0.620	PMP2	0.678	~amyloid_beta+(1|manifest)+cngeneson+pc_mito+brain_region+Sex	7.014	468.000
ENSG00000109771	0.458	-0.168	0.133	-0.468	LRP2BP	0.502	~amyloid_beta+(1|manifest)+cngeneson+pc_mito+brain_region+Sex	1.805	2689.000
ENSG00000197302	0.060	-0.168	-0.023	-0.312	ZNF720	0.112	~amyloid_beta+(1|manifest)+cngeneson+pc_mito+brain_region+Sex	2.127	2283.000
ENSG00000266714	0.330	-0.168	0.184	-0.520	MYO15B	0.381	~amyloid_beta+(1|manifest)+cngeneson+pc_mito+brain_region+Sex	3.795	1126.000
ENSG00000111731	0.012	-0.168	-0.054	-0.282	C2CD5	0.043	~amyloid_beta+(1|manifest)+cngeneson+pc_mito+brain_region+Sex	1.083	3964.000
ENSG00000121390	0.392	-0.168	0.070	-0.406	PSPC1	0.441	~amyloid_beta+(1|manifest)+cngeneson+pc_mito+brain_region+Sex	2.390	1999.000
ENSG00000145721	0.050	-0.168	0.082	-0.418	LIX1	0.099	~amyloid_beta+(1|manifest)+cngeneson+pc_mito+brain_region+Sex	3.639	1185.000
ENSG00000066032	0.041	-0.168	-0.042	-0.294	CTNNA2	0.088	~amyloid_beta+(1|manifest)+cngeneson+pc_mito+brain_region+Sex	11.326	205.000
ENSG00000136643	0.252	-0.168	0.030	-0.367	RPS6KC1	0.306	~amyloid_beta+(1|manifest)+cngeneson+pc_mito+brain_region+Sex	2.268	2135.000
ENSG00000166479	0.017	-0.168	-0.050	-0.287	TMX3	0.054	~amyloid_beta+(1|manifest)+cngeneson+pc_mito+brain_region+Sex	1.579	3014.000
ENSG00000113369	0.068	-0.168	0.040	-0.377	ARRDC3	0.121	~amyloid_beta+(1|manifest)+cngeneson+pc_mito+brain_region+Sex	0.391	5580.000
ENSG00000165359	0.041	-0.168	-0.018	-0.319	INTS6L	0.089	~amyloid_beta+(1|manifest)+cngeneson+pc_mito+brain_region+Sex	2.430	1957.000
ENSG00000171853	0.049	-0.169	-0.005	-0.332	TRAPPC12	0.099	~amyloid_beta+(1|manifest)+cngeneson+pc_mito+brain_region+Sex	1.694	2831.000
ENSG00000163430	0.340	-0.169	0.086	-0.424	FSTL1	0.392	~amyloid_beta+(1|manifest)+cngeneson+pc_mito+brain_region+Sex	3.663	1179.000
ENSG00000132485	0.181	-0.169	0.066	-0.404	ZRANB2	0.236	~amyloid_beta+(1|manifest)+cngeneson+pc_mito+brain_region+Sex	5.557	670.000
ENSG00000064652	0.039	-0.169	-0.034	-0.304	SNX24	0.086	~amyloid_beta+(1|manifest)+cngeneson+pc_mito+brain_region+Sex	1.668	2873.000
ENSG00000088812	0.057	-0.169	-0.032	-0.307	ATRN	0.108	~amyloid_beta+(1|manifest)+cngeneson+pc_mito+brain_region+Sex	1.993	2442.000
ENSG00000006071	0.002	-0.169	0.163	-0.502	ABCC8	0.017	~amyloid_beta+(1|manifest)+cngeneson+pc_mito+brain_region+Sex	1.011	4114.000
ENSG00000051009	0.051	-0.170	-0.010	-0.329	[illegible]	0.101	~amyloid_beta+(1|manifest)+cngeneson+pc_mito+brain_region+Sex	2.604	1811.000
ENSG00000163660	0.233	-0.170	0.071	-0.411	CCNL1	0.287	~amyloid_beta+(1|manifest)+cngeneson+pc_mito+brain_region+Sex	4.153	999.000
ENSG00000096968	0.038	-0.170	-0.018	-0.322	JAK2	0.085	~amyloid_beta+(1|manifest)+cngeneson+pc_mito+brain_region+Sex	2.454	1932.000
ENSG00000158417	0.012	-0.170	-0.060	-0.280	EIF5B	0.044	~amyloid_beta+(1|manifest)+cngeneson+pc_mito+brain_region+Sex	1.187	3735.000
ENSG00000180881	0.314	-0.170	0.049	-0.389	CAPS2	0.367	~amyloid_beta+(1|manifest)+cngeneson+pc_mito+brain_region+Sex	3.111	1477.000
ENSG00000148143	0.396	-0.170	0.077	-0.417	ZNF462	0.444	~amyloid_beta+(1|manifest)+cngeneson+pc_mito+brain_region+Sex	2.468	1924.000
ENSG00000114698	0.501	-0.170	0.126	-0.467	PLSCR4	0.545	~amyloid_beta+(1|manifest)+cngeneson+pc_mito+brain_region+Sex	5.535	674.000
ENSG00000154217	0.040	-0.171	-0.046	-0.295	PITPNC1	0.087	~amyloid_beta+(1|manifest)+cngeneson+pc_mito+brain_region+Sex	2.705	1722.000
ENSG00000178694	0.014	-0.171	-0.040	-0.302	NSUN3	0.049	~amyloid_beta+(1|manifest)+cngeneson+pc_mito+brain_region+Sex	1.494	3150.000
ENSG00000113649	0.058	-0.171	-0.019	-0.323	TCERG1	0.109	~amyloid_beta+(1|manifest)+cngeneson+pc_mito+brain_region+Sex	1.938	2511.000
ENSG00000115840	0.106	-0.171	-0.005	-0.337	SLC25A12	0.161	~amyloid_beta+(1|manifest)+cngeneson+pc_mito+brain_region+Sex	2.262	2142.000
ENSG00000072310	0.693	-0.171	0.409	-0.751	SREBF1	0.723	~amyloid_beta+(1|manifest)+cngeneson+pc_mito+brain_region+Sex	14.637	108.000
ENSG00000011201	0.571	-0.171	0.176	-0.519	ANOS1	0.610	~amyloid_beta+(1|manifest)+cngeneson+pc_mito+brain_region+Sex	2.634	1784.000
ENSG00000125375	0.002	-0.171	-0.078	-0.265	DMAC2L	0.016	~amyloid_beta+(1|manifest)+cngeneson+pc_mito+brain_region+Sex	1.011	4112.000
ENSG00000154553	0.047	-0.172	0.037	-0.380	PDLIM3	0.096	~amyloid_beta+(1|manifest)+cngeneson+pc_mito+brain_region+Sex	2.466	1925.000
ENSG00000078246	0.564	-0.172	0.239	-0.582	TULP3	0.603	~amyloid_beta+(1|manifest)+cngeneson+pc_mito+brain_region+Sex	3.230	1409.000
ENSG00000196950	0.165	-0.172	0.019	-0.364	SLC39A10	0.220	~amyloid_beta+(1|manifest)+cngeneson+pc_mito+brain_region+Sex	2.143	2268.000
ENSG00000062650	0.017	-0.172	-0.050	-0.295	WAPL	0.054	~amyloid_beta+(1|manifest)+cngeneson+pc_mito+brain_region+Sex	1.730	2781.000
ENSG00000149485	0.661	-0.173	0.192	-0.538	FADS1	0.693	~amyloid_beta+(1|manifest)+cngeneson+pc_mito+brain_region+Sex	5.615	663.000
ENSG00000148950	0.123	-0.173	0.007	-0.352	IMMP1L	0.180	~amyloid_beta+(1|manifest)+cngeneson+pc_mito+brain_region+Sex	1.521	3104.000
ENSG00000110435	0.021	-0.173	-0.003	-0.342	PDHX	0.061	~amyloid_beta+(1|manifest)+cngeneson+pc_mito+brain_region+Sex	2.982	1550.000
ENSG00000196275	0.058	-0.173	0.023	-0.369	GTF2IRD2	0.110	~amyloid_beta+(1|manifest)+cngeneson+pc_mito+brain_region+Sex	7.408	424.000
ENSG00000128536	0.237	-0.173	0.066	-0.412	CDHR3	0.291	~amyloid_beta+(1|manifest)+cngeneson+pc_mito+brain_region+Sex	12.757	158.000
ENSG00000164327	0.126	-0.173	-0.002	-0.344	RICTOR	0.182	~amyloid_beta+(1|manifest)+cngeneson+pc_mito+brain_region+Sex	2.357	2042.000
ENSG00000132549	0.156	-0.173	0.008	-0.354	VPS13B	0.211	~amyloid_beta+(1|manifest)+cngeneson+pc_mito+brain_region+Sex	2.436	1948.000
ENSG00000166266	0.003	-0.174	-0.053	-0.294	CUL5	0.020	~amyloid_beta+(1|manifest)+cngeneson+pc_mito+brain_region+Sex	1.936	2516.000
ENSG00000100225	0.005	-0.174	-0.059	-0.288	FBXO7	0.025	~amyloid_beta+(1|manifest)+cngeneson+pc_mito+brain_region+Sex	0.601	5125.000
ENSG00000132394	0.001	-0.174	-0.061	-0.286	EEFSEC	0.010	~amyloid_beta+(1|manifest)+cngeneson+pc_mito+brain_region+Sex	0.772	4685.000
ENSG00000197535	0.119	-0.174	0.010	-0.358	MYO5A	0.175	~amyloid_beta+(1|manifest)+cngeneson+pc_mito+brain_region+Sex	0.768	4696.000
ENSG00000179399	0.049	-0.174	-0.037	-0.312	GPC5	0.099	~amyloid_beta+(1|manifest)+cngeneson+pc_mito+brain_region+Sex	3.531	1232.000
ENSG00000072274	0.544	-0.175	0.123	-0.473	TFRC	0.585	~amyloid_beta+(1|manifest)+cngeneson+pc_mito+brain_region+Sex	4.765	830.000
ENSG00000133858	0.008	-0.175	-0.045	-0.305	ZFC3H1	0.036	~amyloid_beta+(1|manifest)+cngeneson+pc_mito+brain_region+Sex	1.502	3135.000
ENSG00000104824	0.264	-0.175	0.175	-0.525	HNRNPL	0.318	~amyloid_beta+(1|manifest)+cngeneson+pc_mito+brain_region+Sex	2.379	2016.000
ENSG00000134986	0.024	-0.175	-0.040	-0.310	NREP	0.066	~amyloid_beta+(1|manifest)+cngeneson+pc_mito+brain_region+Sex	1.517	3109.000
ENSG00000262655	0.425	-0.175	0.107	-0.457	SPON1	0.471	~amyloid_beta+(1|manifest)+cngeneson+pc_mito+brain_region+Sex	6.141	573.000
ENSG00000167470	0.553	-0.175	0.173	-0.523	MIDN	0.593	~amyloid_beta+(1|manifest)+cngeneson+pc_mito+brain_region+Sex	5.142	740.000
ENSG00000100883	0.014	-0.175	-0.039	-0.312	SRP54	0.049	~amyloid_beta+(1|manifest)+cngeneson+pc_mito+brain_region+Sex	1.495	3143.000
ENSG00000080189	0.334	-0.175	0.150	-0.501	SLC35C2	0.386	~amyloid_beta+(1|manifest)+cngeneson+pc_mito+brain_region+Sex	3.579	1218.000
ENSG00000115415	0.400	-0.176	0.057	-0.408	STAT1	0.448	~amyloid_beta+(1|manifest)+cngeneson+pc_mito+brain_region+Sex	10.665	236.000
ENSG00000125247	0.016	-0.176	-0.045	-0.307	TMTC4	0.053	~amyloid_beta+(1|manifest)+cngeneson+pc_mito+brain_region+Sex	2.104	2311.000
ENSG00000129103	0.100	-0.176	0.045	-0.397	SUMF2	0.155	~amyloid_beta+(1|manifest)+cngeneson+pc_mito+brain_region+Sex	2.237	2166.000
ENSG00000132600	0.002	-0.176	-0.053	-0.299	PRMT7	0.017	~amyloid_beta+(1|manifest)+cngeneson+pc_mito+brain_region+Sex	1.136	3845.000
ENSG00000103528	0.540	-0.176	0.145	-0.497	SYT17	0.581	~amyloid_beta+(1|manifest)+cngeneson+pc_mito+brain_region+Sex	3.045	1517.000
ENSG00000079102	0.343	-0.177	0.055	-0.408	RUNX1T1	0.394	~amyloid_beta+(1|manifest)+cngeneson+pc_mito+brain_region+Sex	1.561	3037.000
ENSG00000164808	0.136	-0.177	-0.002	-0.352	SPIDR	0.192	~amyloid_beta+(1|manifest)+cngeneson+pc_mito+brain_region+Sex	2.544	1860.000
ENSG00000141179	0.298	-0.177	0.025	-0.379	PCTP	0.351	~amyloid_beta+(1|manifest)+cngeneson+pc_mito+brain_region+Sex	1.850	2624.000
ENSG00000198839	0.125	-0.177	-0.003	-0.351	ZNF277	0.181	~amyloid_beta+(1|manifest)+cngeneson+pc_mito+brain_region+Sex	1.563	3035.000
ENSG00000164197	0.042	-0.177	-0.051	-0.303	RNF180	0.090	~amyloid_beta+(1|manifest)+cngeneson+pc_mito+brain_region+Sex	0.964	4215.000
ENSG00000213160	0.317	-0.177	0.111	-0.465	KLHL23	0.370	~amyloid_beta+(1|manifest)+cngeneson+pc_mito+brain_region+Sex	3.602	1206.000
ENSG00000165392	0.394	-0.177	0.080	-0.435	WRN	0.443	~amyloid_beta+(1|manifest)+cngeneson+pc_mito+brain_region+Sex	4.720	837.000
ENSG00000124496	0.591	-0.178	0.211	-0.567	TRERF1	0.628	~amyloid_beta+(1|manifest)+cngeneson+pc_mito+brain_region+Sex	7.334	435.000
ENSG00000143190	0.034	-0.178	-0.041	-0.315	POU2F1	0.080	~amyloid_beta+(1|manifest)+cngeneson+pc_mito+brain_region+Sex	5.201	728.000
ENSG00000161533	0.002	-0.179	-0.086	-0.271	ACOX1	0.015	~amyloid_beta+(1|manifest)+cngeneson+pc_mito+brain_region+Sex	1.018	4102.000
ENSG00000169379	0.023	-0.179	-0.050	-0.307	ARL13B	0.064	~amyloid_beta+(1|manifest)+cngeneson+pc_mito+brain_region+Sex	1.773	2724.000

ENSG00000116120	0.000	-0.179	-0.097	-0.261	FARSB	0.007	~amyloid_beta+(1|manifest)+cngeneson+pc_mito+brain_region+Sex	1.454	3217.000
ENSG00000132718	0.378	-0.179	0.098	-0.456	SYT11	0.428	~amyloid_beta+(1|manifest)+cngeneson+pc_mito+brain_region+Sex	2.092	2322.000
ENSG00000165288	0.077	-0.179	-0.029	-0.329	BRWD3	0.131	~amyloid_beta+(1|manifest)+cngeneson+pc_mito+brain_region+Sex	2.119	2290.000
ENSG00000184863	0.064	-0.179	-0.021	-0.338	RBM33	0.117	~amyloid_beta+(1|manifest)+cngeneson+pc_mito+brain_region+Sex	1.893	2564.000
ENSG00000186376	0.001	-0.180	-0.102	-0.257	ZNF75D	0.013	~amyloid_beta+(1|manifest)+cngeneson+pc_mito+brain_region+Sex	0.702	4878.000
ENSG00000121542	0.001	-0.180	-0.069	-0.291	SEC22A	0.013	~amyloid_beta+(1|manifest)+cngeneson+pc_mito+brain_region+Sex	0.835	4531.000
ENSG00000153936	0.337	-0.180	0.057	-0.417	HS2ST1	0.389	~amyloid_beta+(1|manifest)+cngeneson+pc_mito+brain_region+Sex	3.544	1227.000
ENSG00000271723	0.710	-0.180	0.378	-0.739	[illegible]	0.739	~amyloid_beta+(1|manifest)+cngeneson+pc_mito+brain_region+Sex	8.642	334.000
ENSG00000187231	0.373	-0.181	0.094	-0.455	SESTD1	0.423	~amyloid_beta+(1|manifest)+cngeneson+pc_mito+brain_region+Sex	3.932	1075.000
ENSG00000188130	0.151	-0.181	0.026	-0.387	MAPK12	0.207	~amyloid_beta+(1|manifest)+cngeneson+pc_mito+brain_region+Sex	2.002	2428.000
ENSG00000144218	0.144	-0.181	-0.008	-0.354	AFF3	0.200	~amyloid_beta+(1|manifest)+cngeneson+pc_mito+brain_region+Sex	1.667	2875.000
ENSG00000115355	0.212	-0.181	0.035	-0.396	CCDC88A	0.266	~amyloid_beta+(1|manifest)+cngeneson+pc_mito+brain_region+Sex	6.630	513.000
ENSG00000175066	0.011	-0.181	-0.022	-0.339	GK5	0.041	~amyloid_beta+(1|manifest)+cngeneson+pc_mito+brain_region+Sex	1.870	2602.000
ENSG00000111962	0.542	-0.181	0.224	-0.586	UST	0.583	~amyloid_beta+(1|manifest)+cngeneson+pc_mito+brain_region+Sex	2.512	1886.000
ENSG00000137831	0.073	-0.181	0.005	-0.367	UACA	0.126	~amyloid_beta+(1|manifest)+cngeneson+pc_mito+brain_region+Sex	2.099	2314.000
ENSG00000091157	0.022	-0.181	-0.050	-0.312	WDR7	0.063	~amyloid_beta+(1|manifest)+cngeneson+pc_mito+brain_region+Sex	1.645	2915.000
ENSG00000171444	0.414	-0.181	0.094	-0.456	MCC	0.460	~amyloid_beta+(1|manifest)+cngeneson+pc_mito+brain_region+Sex	2.215	2183.000
ENSG00000145348	0.105	-0.182	-0.013	-0.350	TBCK	0.161	~amyloid_beta+(1|manifest)+cngeneson+pc_mito+brain_region+Sex	1.932	2521.000
ENSG00000112242	0.193	-0.182	0.070	-0.433	E2F3	0.247	~amyloid_beta+(1|manifest)+cngeneson+pc_mito+brain_region+Sex	3.090	1489.000
ENSG00000170776	0.667	-0.182	0.223	-0.586	AKAP13	0.698	~amyloid_beta+(1|manifest)+cngeneson+pc_mito+brain_region+Sex	13.687	130.000
ENSG00000120071	0.053	-0.182	-0.027	-0.338	KANSL1	0.103	~amyloid_beta+(1|manifest)+cngeneson+pc_mito+brain_region+Sex	15.462	95.000
ENSG00000092199	0.474	-0.182	0.108	-0.473	HNRNPC	0.519	~amyloid_beta+(1|manifest)+cngeneson+pc_mito+brain_region+Sex	4.415	911.000
ENSG00000147650	0.072	-0.182	0.014	-0.379	LRP12	0.126	~amyloid_beta+(1|manifest)+cngeneson+pc_mito+brain_region+Sex	2.199	2204.000
ENSG00000166024	0.001	-0.183	-0.096	-0.269	R3HCC1L	0.013	~amyloid_beta+(1|manifest)+cngeneson+pc_mito+brain_region+Sex	1.195	3717.000
ENSG00000138398	0.005	-0.183	-0.062	-0.303	PPIG	0.025	~amyloid_beta+(1|manifest)+cngeneson+pc_mito+brain_region+Sex	1.403	3314.000
ENSG00000167601	0.114	-0.183	0.021	-0.386	AXL	0.170	~amyloid_beta+(1|manifest)+cngeneson+pc_mito+brain_region+Sex	1.972	2464.000
ENSG00000166225	0.181	-0.183	0.015	-0.381	FRS2	0.235	~amyloid_beta+(1|manifest)+cngeneson+pc_mito+brain_region+Sex	2.084	2331.000
ENSG00000114107	0.014	-0.183	-0.002	-0.363	CEP70	0.048	~amyloid_beta+(1|manifest)+cngeneson+pc_mito+brain_region+Sex	1.430	3264.000
ENSG00000183291	0.001	-0.183	-0.086	-0.280	SELENOF	0.010	~amyloid_beta+(1|manifest)+cngeneson+pc_mito+brain_region+Sex	1.427	3272.000
ENSG00000164073	0.003	-0.183	-0.084	-0.282	MFSD8	0.020	~amyloid_beta+(1|manifest)+cngeneson+pc_mito+brain_region+Sex	1.664	2883.000
ENSG00000144535	0.051	-0.183	-0.025	-0.341	DIS3L2	0.101	~amyloid_beta+(1|manifest)+cngeneson+pc_mito+brain_region+Sex	0.912	4346.000
ENSG00000164306	0.026	-0.183	-0.026	-0.341	PRIMPOL	0.068	~amyloid_beta+(1|manifest)+cngeneson+pc_mito+brain_region+Sex	1.842	2638.000
ENSG00000115935	0.136	-0.184	-0.003	-0.364	WIPF1	0.193	~amyloid_beta+(1|manifest)+cngeneson+pc_mito+brain_region+Sex	2.111	2298.000
ENSG00000108599	0.632	-0.184	0.231	-0.598	AKAP10	0.667	~amyloid_beta+(1|manifest)+cngeneson+pc_mito+brain_region+Sex	4.467	900.000
ENSG00000162714	0.001	-0.184	-0.092	-0.275	ZNF496	0.014	~amyloid_beta+(1|manifest)+cngeneson+pc_mito+brain_region+Sex	0.812	4595.000
ENSG00000100580	0.014	-0.184	-0.060	-0.308	TMED8	0.047	~amyloid_beta+(1|manifest)+cngeneson+pc_mito+brain_region+Sex	1.123	3880.000
ENSG00000143995	0.187	-0.184	0.000	-0.368	MEIS1	0.241	~amyloid_beta+(1|manifest)+cngeneson+pc_mito+brain_region+Sex	2.841	1643.000
ENSG00000155363	0.014	-0.184	-0.036	-0.332	MOV10	0.048	~amyloid_beta+(1|manifest)+cngeneson+pc_mito+brain_region+Sex	1.721	2794.000
ENSG00000184719	0.059	-0.184	-0.049	-0.319	RNLS	0.110	~amyloid_beta+(1|manifest)+cngeneson+pc_mito+brain_region+Sex	0.904	4370.000
ENSG00000121957	0.368	-0.184	0.070	-0.439	GPSM2	0.418	~amyloid_beta+(1|manifest)+cngeneson+pc_mito+brain_region+Sex	5.583	666.000
ENSG00000119778	0.065	-0.185	-0.035	-0.334	ATAD2B	0.118	~amyloid_beta+(1|manifest)+cngeneson+pc_mito+brain_region+Sex	1.946	2501.000
ENSG00000078043	0.005	-0.185	-0.051	-0.319	PIAS2	0.028	~amyloid_beta+(1|manifest)+cngeneson+pc_mito+brain_region+Sex	1.959	2482.000
ENSG00000116350	0.079	-0.186	0.013	-0.385	SRSF4	0.134	~amyloid_beta+(1|manifest)+cngeneson+pc_mito+brain_region+Sex	2.649	1770.000
ENSG00000079308	0.304	-0.186	0.186	-0.557	TNS1	0.356	~amyloid_beta+(1|manifest)+cngeneson+pc_mito+brain_region+Sex	5.519	677.000
ENSG00000100815	0.049	-0.186	-0.028	-0.343	TRIP11	0.098	~amyloid_beta+(1|manifest)+cngeneson+pc_mito+brain_region+Sex	1.054	4016.000
ENSG00000091656	0.298	-0.186	0.064	-0.437	ZFHX4	0.351	~amyloid_beta+(1|manifest)+cngeneson+pc_mito+brain_region+Sex	9.443	301.000
ENSG00000171533	0.003	-0.186	-0.085	-0.288	MAP6	0.019	~amyloid_beta+(1|manifest)+cngeneson+pc_mito+brain_region+Sex	0.739	4773.000
ENSG00000011454	0.050	-0.186	-0.036	-0.337	RABGAP1	0.100	~amyloid_beta+(1|manifest)+cngeneson+pc_mito+brain_region+Sex	3.966	1056.000
ENSG00000181222	0.086	-0.186	-0.011	-0.362	POLR2A	0.141	~amyloid_beta+(1|manifest)+cngeneson+pc_mito+brain_region+Sex	1.814	2676.000
ENSG00000135679	0.244	-0.187	0.019	-0.392	MDM2	0.298	~amyloid_beta+(1|manifest)+cngeneson+pc_mito+brain_region+Sex	4.347	934.000
ENSG00000135744	0.677	-0.187	0.287	-0.660	AGT	0.708	~amyloid_beta+(1|manifest)+cngeneson+pc_mito+brain_region+Sex	2.104	2309.000
ENSG00000155621	0.004	-0.187	-0.082	-0.292	C9orf85	0.024	~amyloid_beta+(1|manifest)+cngeneson+pc_mito+brain_region+Sex	0.871	4436.000
ENSG00000166147	0.026	-0.187	0.145	-0.518	FBN1	0.069	~amyloid_beta+(1|manifest)+cngeneson+pc_mito+brain_region+Sex	2.901	1601.000
ENSG00000126870	0.534	-0.187	0.221	-0.595	WDR60	0.575	~amyloid_beta+(1|manifest)+cngeneson+pc_mito+brain_region+Sex	5.309	707.000
ENSG00000198874	0.016	-0.187	-0.049	-0.325	TYW1	0.053	~amyloid_beta+(1|manifest)+cngeneson+pc_mito+brain_region+Sex	1.667	2876.000
ENSG00000079134	0.005	-0.187	-0.057	-0.317	THOC1	0.028	~amyloid_beta+(1|manifest)+cngeneson+pc_mito+brain_region+Sex	1.956	2486.000
ENSG00000116747	0.001	-0.187	-0.096	-0.279	RO60	0.013	~amyloid_beta+(1|manifest)+cngeneson+pc_mito+brain_region+Sex	0.954	4241.000
ENSG00000197157	0.437	-0.187	0.099	-0.474	SND1	0.482	~amyloid_beta+(1|manifest)+cngeneson+pc_mito+brain_region+Sex	4.701	838.000
ENSG00000110274	0.073	-0.187	-0.014	-0.361	CEP164	0.127	~amyloid_beta+(1|manifest)+cngeneson+pc_mito+brain_region+Sex	2.972	1557.000
ENSG00000137992	0.085	-0.188	-0.011	-0.364	DBT	0.140	~amyloid_beta+(1|manifest)+cngeneson+pc_mito+brain_region+Sex	1.996	2437.000
ENSG00000064995	0.003	-0.188	-0.085	-0.291	TAF11	0.021	~amyloid_beta+(1|manifest)+cngeneson+pc_mito+brain_region+Sex	1.099	3932.000
ENSG00000067208	0.174	-0.188	0.003	-0.379	EVI5	0.229	~amyloid_beta+(1|manifest)+cngeneson+pc_mito+brain_region+Sex	3.028	1524.000
ENSG00000125676	0.024	-0.188	-0.033	-0.343	THOC2	0.066	~amyloid_beta+(1|manifest)+cngeneson+pc_mito+brain_region+Sex	2.596	1816.000
ENSG00000081026	0.259	-0.188	0.060	-0.437	MAGI3	0.313	~amyloid_beta+(1|manifest)+cngeneson+pc_mito+brain_region+Sex	3.663	1180.000
ENSG00000078304	0.051	-0.189	-0.029	-0.349	PPP2R5C	0.101	~amyloid_beta+(1|manifest)+cngeneson+pc_mito+brain_region+Sex	1.464	3197.000
ENSG00000176623	0.002	-0.189	-0.073	-0.305	RMDN1	0.016	~amyloid_beta+(1|manifest)+cngeneson+pc_mito+brain_region+Sex	0.962	4218.000
ENSG00000164031	0.000	-0.189	-0.113	-0.265	DNAJB14	0.002	~amyloid_beta+(1|manifest)+cngeneson+pc_mito+brain_region+Sex	1.468	3191.000
ENSG00000114757	0.173	-0.189	-0.008	-0.371	PEX5L	0.228	~amyloid_beta+(1|manifest)+cngeneson+pc_mito+brain_region+Sex	0.505	5340.000
ENSG00000111300	0.140	-0.189	0.023	-0.402	NAA25	0.196	~amyloid_beta+(1|manifest)+cngeneson+pc_mito+brain_region+Sex	3.295	1370.000
ENSG00000125246	0.509	-0.190	0.235	-0.614	CLYBL	0.552	~amyloid_beta+(1|manifest)+cngeneson+pc_mito+brain_region+Sex	7.442	421.000
ENSG00000152092	0.234	-0.190	0.040	-0.419	ASTN1	0.288	~amyloid_beta+(1|manifest)+cngeneson+pc_mito+brain_region+Sex	2.474	1919.000
ENSG00000174606	0.000	-0.190	-0.128	-0.252	ANGEL2	0.001	~amyloid_beta+(1|manifest)+cngeneson+pc_mito+brain_region+Sex	1.084	3962.000
ENSG00000102317	0.020	-0.190	-0.046	-0.334	RBM3	0.060	~amyloid_beta+(1|manifest)+cngeneson+pc_mito+brain_region+Sex	1.889	2580.000
ENSG00000148935	0.057	-0.190	-0.053	-0.327	GAS2	0.109	~amyloid_beta+(1|manifest)+cngeneson+pc_mito+brain_region+Sex	1.892	2568.000
ENSG00000173588	0.004	-0.191	-0.058	-0.323	CEP83	0.022	~amyloid_beta+(1|manifest)+cngeneson+pc_mito+brain_region+Sex	1.742	2762.000
ENSG00000155158	0.097	-0.191	-0.018	-0.364	TTC39B	0.152	~amyloid_beta+(1|manifest)+cngeneson+pc_mito+brain_region+Sex	4.948	787.000
ENSG00000006062	0.708	-0.191	0.341	-0.722	MAP3K14	0.736	~amyloid_beta+(1|manifest)+cngeneson+pc_mito+brain_region+Sex	10.106	264.000
ENSG00000105176	0.040	-0.191	-0.044	-0.338	URI1	0.087	~amyloid_beta+(1|manifest)+cngeneson+pc_mito+brain_region+Sex	2.392	1997.000
ENSG00000166848	0.388	-0.191	0.072	-0.454	TERF2IP	0.437	~amyloid_beta+(1|manifest)+cngeneson+pc_mito+brain_region+Sex	2.928	1591.000
ENSG00000116266	0.022	-0.191	-0.051	-0.332	STXBP3	0.062	~amyloid_beta+(1|manifest)+cngeneson+pc_mito+brain_region+Sex	0.600	5127.000
ENSG00000127720	0.082	-0.191	-0.035	-0.348	METTL25	0.137	~amyloid_beta+(1|manifest)+cngeneson+pc_mito+brain_region+Sex	1.391	3336.000
ENSG00000116874	0.010	-0.191	-0.077	-0.305	WARS2	0.041	~amyloid_beta+(1|manifest)+cngeneson+pc_mito+brain_region+Sex	1.424	3278.000
ENSG00000123200	0.012	-0.191	-0.061	-0.321	ZC3H13	0.044	~amyloid_beta+(1|manifest)+cngeneson+pc_mito+brain_region+Sex	1.143	3831.000
ENSG00000135597	0.002	-0.191	-0.086	-0.296	REPS1	0.016	~amyloid_beta+(1|manifest)+cngeneson+pc_mito+brain_region+Sex	3.940	1067.000
ENSG00000047056	0.236	-0.191	0.024	-0.407	WDR37	0.290	~amyloid_beta+(1|manifest)+cngeneson+pc_mito+brain_region+Sex	3.778	1131.000
ENSG00000165417	0.006	-0.192	-0.088	-0.296	GTF2A1	0.029	~amyloid_beta+(1|manifest)+cngeneson+pc_mito+brain_region+Sex	1.745	2755.000
ENSG00000172915	0.427	-0.192	0.099	-0.483	NBEA	0.473	~amyloid_beta+(1|manifest)+cngeneson+pc_mito+brain_region+Sex	0.890	4398.000
ENSG00000112200	0.071	-0.192	-0.009	-0.376	ZNF451	0.125	~amyloid_beta+(1|manifest)+cngeneson+pc_mito+brain_region+Sex	2.311	2090.000
ENSG00000122642	0.022	-0.193	-0.068	-0.317	FKBP9	0.062	~amyloid_beta+(1|manifest)+cngeneson+pc_mito+brain_region+Sex	1.880	2589.000
ENSG00000138430	0.055	-0.193	-0.046	-0.339	OLA1	0.106	~amyloid_beta+(1|manifest)+cngeneson+pc_mito+brain_region+Sex	2.037	2385.000
ENSG00000196678	0.001	-0.193	-0.092	-0.293	ERI2	0.009	~amyloid_beta+(1|manifest)+cngeneson+pc_mito+brain_region+Sex	1.487	3163.000
ENSG00000089737	0.139	-0.193	0.031	-0.417	DDX24	0.195	~amyloid_beta+(1|manifest)+cngeneson+pc_mito+brain_region+Sex	3.020	1531.000
ENSG00000077232	0.025	-0.193	-0.046	-0.340	DNAJC10	0.068	~amyloid_beta+(1|manifest)+cngeneson+pc_mito+brain_region+Sex	1.547	3055.000
ENSG00000165533	0.002	-0.193	-0.079	-0.306	TTC8	0.017	~amyloid_beta+(1|manifest)+cngeneson+pc_mito+brain_region+Sex	0.752	4739.000
ENSG00000196511	0.192	-0.193	0.021	-0.406	TPK1	0.246	~amyloid_beta+(1|manifest)+cngeneson+pc_mito+brain_region+Sex	2.191	2214.000
ENSG00000143207	0.080	-0.193	-0.027	-0.360	COP1	0.135	~amyloid_beta+(1|manifest)+cngeneson+pc_mito+brain_region+Sex	3.565	1221.000
ENSG00000137601	0.157	-0.193	0.050	-0.436	NEK1	0.212	~amyloid_beta+(1|manifest)+cngeneson+pc_mito+brain_region+Sex	3.820	1114.000
ENSG00000155754	0.055	-0.193	-0.048	-0.338	C2CD6	0.105	~amyloid_beta+(1|manifest)+cngeneson+pc_mito+brain_region+Sex	0.855	4482.000
ENSG00000109466	0.028	-0.194	-0.071	-0.317	KLHL2	0.072	~amyloid_beta+(1|manifest)+cngeneson+pc_mito+brain_region+Sex	2.364	2035.000
ENSG00000180104	0.040	-0.194	-0.035	-0.352	EXOC3	0.087	~amyloid_beta+(1|manifest)+cngeneson+pc_mito+brain_region+Sex	1.712	2807.000
ENSG00000185345	0.007	-0.194	-0.068	-0.320	PRKN	0.032	~amyloid_beta+(1|manifest)+cngeneson+pc_mito+brain_region+Sex	6.094	580.000
ENSG00000187715	0.398	-0.194	0.074	-0.462	KBTBD12	0.446	~amyloid_beta+(1|manifest)+cngeneson+pc_mito+brain_region+Sex	3.275	1384.000
ENSG00000134900	0.002	-0.194	-0.088	-0.301	TPP2	0.015	~amyloid_beta+(1|manifest)+cngeneson+pc_mito+brain_region+Sex	1.188	3731.000
ENSG00000102531	0.238	-0.195	0.025	-0.415	FNDC3A	0.292	~amyloid_beta+(1|manifest)+cngeneson+pc_mito+brain_region+Sex	5.730	641.000
ENSG00000188906	0.004	-0.195	-0.063	-0.327	LRRK2	0.024	~amyloid_beta+(1|manifest)+cngeneson+pc_mito+brain_region+Sex	0.779	4671.000
ENSG00000177706	0.824	-0.195	0.424	-0.814	FAM20C	0.842	~amyloid_beta+(1|manifest)+cngeneson+pc_mito+brain_region+Sex	12.771	156.000
ENSG00000184005	0.732	-0.195	0.309	-0.700	[illegible]	0.759	~amyloid_beta+(1|manifest)+cngeneson+pc_mito+brain_region+Sex	6.526	523.000
ENSG00000103479	0.010	-0.196	0.004	-0.395	RBL2	0.040	~amyloid_beta+(1|manifest)+cngeneson+pc_mito+brain_region+Sex	1.904	2549.000
ENSG00000119487	0.034	-0.196	-0.034	-0.357	MAPKAP1	0.080	~amyloid_beta+(1|manifest)+cngeneson+pc_mito+brain_region+Sex	1.735	2770.000
ENSG00000120860	0.003	-0.196	-0.044	-0.349	WASHC3	0.020	~amyloid_beta+(1|manifest)+cngeneson+pc_mito+brain_region+Sex	1.204	3696.000
ENSG00000106571	0.159	-0.196	0.013	-0.405	GLI3	0.214	~amyloid_beta+(1|manifest)+cngeneson+pc_mito+brain_region+Sex	2.650	1768.000
ENSG00000184226	0.003	-0.196	-0.093	-0.299	PCDH9	0.019	~amyloid_beta+(1|manifest)+cngeneson+pc_mito+brain_region+Sex	10.700	235.000
ENSG00000171100	0.105	-0.196	-0.003	-0.390	MTM1	0.161	~amyloid_beta+(1|manifest)+cngeneson+pc_mito+brain_region+Sex	1.592	2998.000
ENSG00000136935	0.074	-0.196	-0.005	-0.388	GOLGA1	0.128	~amyloid_beta+(1|manifest)+cngeneson+pc_mito+brain_region+Sex	2.505	1898.000
ENSG00000089916	0.001	-0.197	-0.100	-0.294	GPATCH2L	0.011	~amyloid_beta+(1|manifest)+cngeneson+pc_mito+brain_region+Sex	1.139	3837.000
ENSG00000184232	0.635	-0.197	0.255	-0.650	OAF	0.669	~amyloid_beta+(1|manifest)+cngeneson+pc_mito+brain_region+Sex	7.122	457.000
ENSG00000138780	0.085	-0.197	-0.039	-0.356	GSTCD	0.140	~amyloid_beta+(1|manifest)+cngeneson+pc_mito+brain_region+Sex	1.993	2444.000
ENSG00000154310	0.004	-0.198	-0.090	-0.305	TNIK	0.022	~amyloid_beta+(1|manifest)+cngeneson+pc_mito+brain_region+Sex	6.656	512.000
ENSG00000159733	0.829	-0.198	0.439	-0.835	ZFYVE28	0.847	~amyloid_beta+(1|manifest)+cngeneson+pc_mito+brain_region+Sex	16.371	81.000
ENSG00000171903	0.011	-0.198	-0.024	-0.372	CYP4F11	0.042	~amyloid_beta+(1|manifest)+cngeneson+pc_mito+brain_region+Sex	2.367	2029.000
ENSG00000188559	0.412	-0.198	0.120	-0.516	RALGAPA2	0.458	~amyloid_beta+(1|manifest)+cngeneson+pc_mito+brain_region+Sex	12.014	175.000
ENSG00000136560	0.371	-0.198	0.083	-0.479	TANK	0.421	~amyloid_beta+(1|manifest)+cngeneson+pc_mito+brain_region+Sex	3.066	1498.000
ENSG00000163659	0.334	-0.198	0.051	-0.447	TIPARP	0.386	~amyloid_beta+(1|manifest)+cngeneson+pc_mito+brain_region+Sex	4.107	1011.000
ENSG00000137944	0.006	-0.199	-0.056	-0.341	KYAT3	0.030	~amyloid_beta+(1|manifest)+cngeneson+pc_mito+brain_region+Sex	1.748	2749.000
ENSG00000174842	0.001	-0.199	-0.101	-0.296	GLMN	0.013	~amyloid_beta+(1|manifest)+cngeneson+pc_mito+brain_region+Sex	1.780	2715.000
ENSG00000089123	0.084	-0.199	-0.017	-0.381	TASP1	0.139	~amyloid_beta+(1|manifest)+cngeneson+pc_mito+brain_region+Sex	1.541	3061.000
ENSG00000196792	0.131	-0.199	-0.010	-0.387	STRN3	0.187	~amyloid_beta+(1|manifest)+cngeneson+pc_mito+brain_region+Sex	2.861	1626.000
ENSG00000053328	0.044	-0.199	-0.042	-0.356	METTL24	0.092	~amyloid_beta+(1|manifest)+cngeneson+pc_mito+brain_region+Sex	1.573	3019.000
ENSG00000164199	0.006	-0.199	-0.079	-0.320	ADGRV1	0.030	~amyloid_beta+(1|manifest)+cngeneson+pc_mito+brain_region+Sex	5.038	764.000
ENSG00000100281	0.012	-0.200	-0.033	-0.366	HMGXB4	0.043	~amyloid_beta+(1|manifest)+cngeneson+pc_mito+brain_region+Sex	2.270	2131.000
ENSG00000147408	0.001	-0.200	-0.104	-0.296	[illegible]	0.008	~amyloid_beta+(1|manifest)+cngeneson+pc_mito+brain_region+Sex	4.599	863.000
ENSG00000116754	0.112	-0.200	0.026	-0.427	SRSF11	0.168	~amyloid_beta+(1|manifest)+cngeneson+pc_mito+brain_region+Sex	4.353	931.000
ENSG00000141510	0.346	-0.200	0.054	-0.454	TP53	0.397	~amyloid_beta+(1|manifest)+cngeneson+pc_mito+brain_region+Sex	2.738	1711.000
ENSG00000184408	0.744	-0.200	0.434	-0.834	KCND2	0.769	~amyloid_beta+(1|manifest)+cngeneson+pc_mito+brain_region+Sex	12.224	170.000
ENSG00000008394	0.228	-0.200	0.048	-0.449	MGST1	0.282	~amyloid_beta+(1|manifest)+cngeneson+pc_mito+brain_region+Sex	9.915	273.000
ENSG00000137764	0.073	-0.201	-0.030	-0.371	MAP2K5	0.126	~amyloid_beta+(1|manifest)+cngeneson+pc_mito+brain_region+Sex	0.742	4764.000
ENSG00000151779	0.090	-0.201	-0.026	-0.376	NBAS	0.145	~amyloid_beta+(1|manifest)+cngeneson+pc_mito+brain_region+Sex	2.263	2140.000
ENSG00000135655	0.010	-0.201	-0.059	-0.343	USP15	0.041	~amyloid_beta+(1|manifest)+cngeneson+pc_mito+brain_region+Sex	2.484	1912.000
ENSG00000005379	0.367	-0.201	0.244	-0.645	TSPOAP1	0.417	~amyloid_beta+(1|manifest)+cngeneson+pc_mito+brain_region+Sex	7.044	465.000
ENSG00000049167	0.007	-0.201	-0.077	-0.325	ERCC8	0.032	~amyloid_beta+(1|manifest)+cngeneson+pc_mito+brain_region+Sex	1.501	3136.000
ENSG00000152620	0.097	-0.201	-0.014	-0.388	NADK2	0.152	~amyloid_beta+(1|manifest)+cngeneson+pc_mito+brain_region+Sex	3.343	1341.000
ENSG00000176834	0.160	-0.201	0.055	-0.458	VSIG10	0.215	~amyloid_beta+(1|manifest)+cngeneson+pc_mito+brain_region+Sex	3.316	1361.000
ENSG00000080608	0.024	-0.202	-0.055	-0.348	PUM3	0.066	~amyloid_beta+(1|manifest)+cngeneson+pc_mito+brain_region+Sex	2.343	2057.000
ENSG00000138771	0.632	-0.202	0.253	-0.656	SHROOM3	0.667	~amyloid_beta+(1|manifest)+cngeneson+pc_mito+brain_region+Sex	3.319	1358.000
ENSG00000196502	0.125	-0.202	0.037	-0.440	SULT1A1	0.181	~amyloid_beta+(1|manifest)+cngeneson+pc_mito+brain_region+Sex	3.421	1299.000
ENSG00000178234	0.008	-0.202	-0.070	-0.333	GALNT11	0.035	~amyloid_beta+(1|manifest)+cngeneson+pc_mito+brain_region+Sex	1.150	3814.000
ENSG00000078328	0.469	-0.202	0.118	-0.522	RBFOX1	0.514	~amyloid_beta+(1|manifest)+cngeneson+pc_mito+brain_region+Sex	1.433	3261.000
ENSG00000101150	0.025	-0.202	-0.049	-0.355	TPD52L2	0.067	~amyloid_beta+(1|manifest)+cngeneson+pc_mito+brain_region+Sex	2.553	1853.000
ENSG00000138696	0.002	-0.202	-0.093	-0.311	BMPR1B	0.015	~amyloid_beta+(1|manifest)+cngeneson+pc_mito+brain_region+Sex	2.952	1571.000
ENSG00000154447	0.483	-0.202	0.135	-0.539	SH3RF1	0.527	~amyloid_beta+(1|manifest)+cngeneson+pc_mito+brain_region+Sex	4.176	992.000
ENSG00000121691	0.113	-0.202	-0.013	-0.391	CAT	0.169	~amyloid_beta+(1|manifest)+cngeneson+pc_mito+brain_region+Sex	1.163	3782.000
ENSG00000069535	0.775	-0.203	0.362	-0.767	MAOB	0.797	~amyloid_beta+(1|manifest)+cngeneson+pc_mito+brain_region+Sex	17.756	71.000
ENSG00000140463	0.000	-0.203	-0.119	-0.287	BBS4	0.003	~amyloid_beta+(1|manifest)+cngeneson+pc_mito+brain_region+Sex	1.147	3821.000
ENSG00000157741	0.004	-0.203	-0.074	-0.333	UBN2	0.025	~amyloid_beta+(1|manifest)+cngeneson+pc_mito+brain_region+Sex	1.648	2909.000
ENSG00000106261	0.202	-0.204	0.088	-0.495	ZKSCAN1	0.257	~amyloid_beta+(1|manifest)+cngeneson+pc_mito+brain_region+Sex	3.719	1156.000
ENSG00000186314	0.239	-0.204	0.013	-0.420	PRELID2	0.293	~amyloid_beta+(1|manifest)+cngeneson+pc_mito+brain_region+Sex	0.948	4258.000
ENSG00000151322	0.001	-0.204	-0.110	-0.298	NPAS3	0.012	~amyloid_beta+(1|manifest)+cngeneson+pc_mito+brain_region+Sex	13.051	149.000
ENSG00000073712	0.045	-0.204	-0.042	-0.366	FERMT2	0.094	~amyloid_beta+(1|manifest)+cngeneson+pc_mito+brain_region+Sex	3.916	1081.000
ENSG00000156256	0.000	-0.204	-0.118	-0.290	USP16	0.004	~amyloid_beta+(1|manifest)+cngeneson+pc_mito+brain_region+Sex	0.910	4355.000
ENSG00000111817	0.265	-0.204	0.033	-0.442	DSE	0.318	~amyloid_beta+(1|manifest)+cngeneson+pc_mito+brain_region+Sex	1.305	3496.000
ENSG00000151414	0.206	-0.205	0.026	-0.436	NEK7	0.261	~amyloid_beta+(1|manifest)+cngeneson+pc_mito+brain_region+Sex	1.475	3180.000
ENSG00000165478	0.379	-0.205	0.106	-0.516	HEPACAM	0.429	~amyloid_beta+(1|manifest)+cngeneson+pc_mito+brain_region+Sex	7.334	434.000
ENSG00000162613	0.006	-0.205	-0.058	-0.352	FUBP1	0.030	~amyloid_beta+(1|manifest)+cngeneson+pc_mito+brain_region+Sex	1.565	3032.000
ENSG00000183117	0.666	-0.205	0.288	-0.699	CSMD1	0.698	~amyloid_beta+(1|manifest)+cngeneson+pc_mito+brain_region+Sex	17.282	78.000
ENSG00000158195	0.195	-0.205	0.012	-0.422	WASF2	0.249	~amyloid_beta+(1|manifest)+cngeneson+pc_mito+brain_region+Sex	3.594	1209.000
ENSG00000144749	0.121	-0.206	0.000	-0.411	LRIG1	0.178	~amyloid_beta+(1|manifest)+cngeneson+pc_mito+brain_region+Sex	20.593	47.000
ENSG00000114933	0.553	-0.206	0.210	-0.622	INO80D	0.593	~amyloid_beta+(1|manifest)+cngeneson+pc_mito+brain_region+Sex	49.199	6.000
ENSG00000178538	0.547	-0.206	0.174	-0.587	CA8	0.587	~amyloid_beta+(1|manifest)+cngeneson+pc_mito+brain_region+Sex	2.603	1812.000
ENSG00000120899	0.015	-0.207	-0.075	-0.338	PTK2B	0.050	~amyloid_beta+(1|manifest)+cngeneson+pc_mito+brain_region+Sex	1.122	3882.000
ENSG00000188352	0.261	-0.207	0.037	-0.450	FOCAD	0.315	~amyloid_beta+(1|manifest)+cngeneson+pc_mito+brain_region+Sex	5.349	701.000
ENSG00000116580	0.036	-0.207	-0.038	-0.376	GON4L	0.082	~amyloid_beta+(1|manifest)+cngeneson+pc_mito+brain_region+Sex	2.771	1688.000
ENSG00000128563	0.080	-0.207	0.035	-0.449	PRKRIP1	0.134	~amyloid_beta+(1|manifest)+cngeneson+pc_mito+brain_region+Sex	2.282	2124.000
ENSG00000152404	0.005	-0.207	-0.067	-0.347	CWF19L2	0.028	~amyloid_beta+(1|manifest)+cngeneson+pc_mito+brain_region+Sex	0.814	4587.000
ENSG00000046889	0.001	-0.207	-0.105	-0.309	PREX2	0.008	~amyloid_beta+(1|manifest)+cngeneson+pc_mito+brain_region+Sex	2.677	1748.000
ENSG00000170899	0.012	-0.207	-0.064	-0.350	GSTA4	0.043	~amyloid_beta+(1|manifest)+cngeneson+pc_mito+brain_region+Sex	1.029	4082.000
ENSG00000062598	0.083	-0.207	-0.018	-0.396	ELMO2	0.138	~amyloid_beta+(1|manifest)+cngeneson+pc_mito+brain_region+Sex	2.349	2050.000
ENSG00000042317	0.039	-0.207	-0.018	-0.397	SPATA7	0.087	~amyloid_beta+(1|manifest)+cngeneson+pc_mito+brain_region+Sex	2.224	2177.000
ENSG00000186448	0.017	-0.207	-0.078	-0.337	ZNF197	0.054	~amyloid_beta+(1|manifest)+cngeneson+pc_mito+brain_region+Sex	1.512	3118.000
ENSG00000129691	0.010	-0.208	-0.075	-0.340	ASH2L	0.040	~amyloid_beta+(1|manifest)+cngeneson+pc_mito+brain_region+Sex	1.533	3082.000
ENSG00000138593	0.039	-0.208	-0.038	-0.377	SECISBP2L	0.086	~amyloid_beta+(1|manifest)+cngeneson+pc_mito+brain_region+Sex	2.396	1988.000
ENSG00000146151	0.516	-0.208	0.147	-0.563	HMGCLL1	0.559	~amyloid_beta+(1|manifest)+cngeneson+pc_mito+brain_region+Sex	2.787	1678.000
ENSG00000092108	0.003	-0.208	-0.079	-0.337	SCFD1	0.018	~amyloid_beta+(1|manifest)+cngeneson+pc_mito+brain_region+Sex	2.104	2310.000
ENSG00000075213	0.357	-0.208	0.098	-0.514	SEMA3A	0.408	~amyloid_beta+(1|manifest)+cngeneson+pc_mito+brain_region+Sex	1.937	2514.000
ENSG00000134444	0.078	-0.208	-0.021	-0.395	RELCH	0.133	~amyloid_beta+(1|manifest)+cngeneson+pc_mito+brain_region+Sex	1.151	3810.000
ENSG00000181827	0.074	-0.208	-0.030	-0.386	RFX7	0.128	~amyloid_beta+(1|manifest)+cngeneson+pc_mito+brain_region+Sex	3.000	1543.000
ENSG00000164687	0.150	-0.208	0.056	-0.472	FABP5	0.206	~amyloid_beta+(1|manifest)+cngeneson+pc_mito+brain_region+Sex	3.952	1063.000
ENSG00000111670	0.035	-0.208	-0.049	-0.368	GNPTAB	0.081	~amyloid_beta+(1|manifest)+cngeneson+pc_mito+brain_region+Sex	2.320	2081.000
ENSG00000145476	0.110	-0.209	0.067	-0.484	CYP4V2	0.166	~amyloid_beta+(1|manifest)+cngeneson+pc_mito+brain_region+Sex	3.526	1235.000
ENSG00000114120	0.041	-0.209	-0.029	-0.389	SLC25A36	0.088	~amyloid_beta+(1|manifest)+cngeneson+pc_mito+brain_region+Sex	2.593	1817.000
ENSG00000162994	0.142	-0.209	0.010	-0.428	CLHC1	0.199	~amyloid_beta+(1|manifest)+cngeneson+pc_mito+brain_region+Sex	3.168	1443.000
ENSG00000130023	0.009	-0.210	-0.085	-0.335	ERMARD	0.038	~amyloid_beta+(1|manifest)+cngeneson+pc_mito+brain_region+Sex	1.420	3294.000
ENSG00000183826	0.100	-0.210	-0.014	-0.407	BTBD9	0.156	~amyloid_beta+(1|manifest)+cngeneson+pc_mito+brain_region+Sex	2.899	1602.000
ENSG00000129353	0.138	-0.211	0.035	-0.456	SLC44A2	0.195	~amyloid_beta+(1|manifest)+cngeneson+pc_mito+brain_region+Sex	1.616	2957.000
ENSG00000139496	0.024	-0.211	-0.053	-0.369	NUP58	0.066	~amyloid_beta+(1|manifest)+cngeneson+pc_mito+brain_region+Sex	1.249	3602.000
ENSG00000088280	0.505	-0.211	0.190	-0.612	ASAP3	0.548	~amyloid_beta+(1|manifest)+cngeneson+pc_mito+brain_region+Sex	7.071	462.000
ENSG00000010244	0.119	-0.211	0.006	-0.428	ZNF207	0.176	~amyloid_beta+(1|manifest)+cngeneson+pc_mito+brain_region+Sex	2.172	2234.000
ENSG00000054179	0.505	-0.211	0.254	-0.676	ENTPD2	0.548	~amyloid_beta+(1|manifest)+cngeneson+pc_mito+brain_region+Sex	4.533	886.000
ENSG00000173230	0.302	-0.212	0.153	-0.577	GOLGB1	0.354	~amyloid_beta+(1|manifest)+cngeneson+pc_mito+brain_region+Sex	3.973	1050.000
ENSG00000106692	0.000	-0.212	-0.106	-0.318	FKTN	0.006	~amyloid_beta+(1|manifest)+cngeneson+pc_mito+brain_region+Sex	0.567	5191.000
ENSG00000134851	0.012	-0.212	-0.056	-0.368	TMEM165	0.043	~amyloid_beta+(1|manifest)+cngeneson+pc_mito+brain_region+Sex	2.132	2276.000
ENSG00000047634	0.304	-0.212	0.059	-0.483	SCML1	0.356	~amyloid_beta+(1|manifest)+cngeneson+pc_mito+brain_region+Sex	6.789	493.000
ENSG00000113790	0.028	-0.212	-0.073	-0.352	EHHADH	0.072	~amyloid_beta+(1|manifest)+cngeneson+pc_mito+brain_region+Sex	1.469	3185.000
ENSG00000135164	0.088	-0.212	-0.011	-0.414	DMTF1	0.143	~amyloid_beta+(1|manifest)+cngeneson+pc_mito+brain_region+Sex	0.993	4156.000
ENSG00000156475	0.004	-0.213	-0.100	-0.325	PPP2R2B	0.023	~amyloid_beta+(1|manifest)+cngeneson+pc_mito+brain_region+Sex	9.452	300.000
ENSG00000080823	0.038	-0.213	-0.046	-0.379	MOK	0.085	~amyloid_beta+(1|manifest)+cngeneson+pc_mito+brain_region+Sex	1.497	3141.000
ENSG00000134716	0.144	-0.213	0.007	-0.433	CYP2J2	0.200	~amyloid_beta+(1|manifest)+cngeneson+pc_mito+brain_region+Sex	0.887	4406.000
ENSG00000122870	0.342	-0.213	0.055	-0.481	BICC1	0.393	~amyloid_beta+(1|manifest)+cngeneson+pc_mito+brain_region+Sex	6.840	487.000
ENSG00000139364	0.755	-0.213	0.377	-0.804	[illegible]	0.779	~amyloid_beta+(1|manifest)+cngeneson+pc_mito+brain_region+Sex	7.063	463.000
ENSG00000146463	0.051	-0.213	-0.045	-0.382	ZMYM4	0.101	~amyloid_beta+(1|manifest)+cngeneson+pc_mito+brain_region+Sex	1.422	3288.000
ENSG00000168234	0.039	-0.214	-0.004	-0.424	TTC39C	0.086	~amyloid_beta+(1|manifest)+cngeneson+pc_mito+brain_region+Sex	2.678	1747.000
ENSG00000128656	0.162	-0.214	0.008	-0.435	CHN1	0.218	~amyloid_beta+(1|manifest)+cngeneson+pc_mito+brain_region+Sex	1.365	3383.000
ENSG00000164066	0.029	-0.214	-0.049	-0.379	INTU	0.073	~amyloid_beta+(1|manifest)+cngeneson+pc_mito+brain_region+Sex	3.244	1400.000
ENSG00000170624	0.315	-0.214	0.130	-0.558	SGCD	0.367	~amyloid_beta+(1|manifest)+cngeneson+pc_mito+brain_region+Sex	4.894	800.000
ENSG00000041982	0.070	-0.214	0.372	-0.800	TNC	0.123	~amyloid_beta+(1|manifest)+cngeneson+pc_mito+brain_region+Sex	7.511	414.000
ENSG00000100578	0.224	-0.214	0.027	-0.455	KIAA0586	0.279	~amyloid_beta+(1|manifest)+cngeneson+pc_mito+brain_region+Sex	5.419	691.000
ENSG00000271425	0.003	-0.215	-0.098	-0.331	NBPF10	0.019	~amyloid_beta+(1|manifest)+cngeneson+pc_mito+brain_region+Sex	1.311	3478.000
ENSG00000211584	0.117	-0.215	0.016	-0.447	SLC48A1	0.174	~amyloid_beta+(1|manifest)+cngeneson+pc_mito+brain_region+Sex	2.120	2288.000
ENSG00000133627	0.044	-0.215	-0.059	-0.371	ACTR3B	0.093	~amyloid_beta+(1|manifest)+cngeneson+pc_mito+brain_region+Sex	2.017	2414.000
ENSG00000148225	0.007	-0.215	-0.093	-0.338	WDR31	0.032	~amyloid_beta+(1|manifest)+cngeneson+pc_mito+brain_region+Sex	1.458	3208.000
ENSG00000126653	0.055	-0.216	-0.042	-0.389	NSRP1	0.105	~amyloid_beta+(1|manifest)+cngeneson+pc_mito+brain_region+Sex	2.839	1644.000
ENSG00000112137	0.239	-0.216	0.075	-0.507	PHACTR1	0.292	~amyloid_beta+(1|manifest)+cngeneson+pc_mito+brain_region+Sex	2.954	1570.000
ENSG00000104313	0.669	-0.216	0.268	-0.700	EYA1	0.700	~amyloid_beta+(1|manifest)+cngeneson+pc_mito+brain_region+Sex	5.843	616.000
ENSG00000109920	0.311	-0.216	0.083	-0.515	FNBP4	0.364	~amyloid_beta+(1|manifest)+cngeneson+pc_mito+brain_region+Sex	5.382	695.000
ENSG00000163328	0.306	-0.216	0.079	-0.512	GPR155	0.358	~amyloid_beta+(1|manifest)+cngeneson+pc_mito+brain_region+Sex	4.935	789.000
ENSG00000185942	0.121	-0.217	0.005	-0.438	NKAIN3	0.177	~amyloid_beta+(1|manifest)+cngeneson+pc_mito+brain_region+Sex	14.001	124.000
ENSG00000167632	0.083	-0.217	-0.026	-0.409	TRAPPC9	0.138	~amyloid_beta+(1|manifest)+cngeneson+pc_mito+brain_region+Sex	3.326	1355.000
ENSG00000145996	0.006	-0.218	-0.089	-0.347	CDKAL1	0.030	~amyloid_beta+(1|manifest)+cngeneson+pc_mito+brain_region+Sex	1.473	3181.000
ENSG00000138190	0.011	-0.219	-0.088	-0.350	EXOC6	0.041	~amyloid_beta+(1|manifest)+cngeneson+pc_mito+brain_region+Sex	2.188	2218.000
ENSG00000082293	0.370	-0.219	0.123	-0.561	COL19A1	0.420	~amyloid_beta+(1|manifest)+cngeneson+pc_mito+brain_region+Sex	1.491	3155.000
ENSG00000188313	0.752	-0.219	0.339	-0.776	PLSCR1	0.777	~amyloid_beta+(1|manifest)+cngeneson+pc_mito+brain_region+Sex	14.335	121.000
ENSG00000117859	0.146	-0.219	0.015	-0.435	OSBPL9	0.202	~amyloid_beta+(1|manifest)+cngeneson+pc_mito+brain_region+Sex	3.914	1085.000
ENSG00000176222	0.043	-0.219	-0.036	-0.403	ZNF404	0.091	~amyloid_beta+(1|manifest)+cngeneson+pc_mito+brain_region+Sex	2.283	2121.000
ENSG00000156049	0.565	-0.220	0.212	-0.652	GNA14	0.604	~amyloid_beta+(1|manifest)+cngeneson+pc_mito+brain_region+Sex	9.308	306.000
ENSG00000151612	0.098	-0.220	-0.024	-0.416	ZNF827	0.153	~amyloid_beta+(1|manifest)+cngeneson+pc_mito+brain_region+Sex	3.166	1445.000
ENSG00000100626	0.195	-0.220	0.008	-0.448	GALNT16	0.250	~amyloid_beta+(1|manifest)+cngeneson+pc_mito+brain_region+Sex	9.941	272.000
ENSG00000163138	0.002	-0.220	-0.097	-0.344	PACRGL	0.015	~amyloid_beta+(1|manifest)+cngeneson+pc_mito+brain_region+Sex	1.407	3310.000
ENSG00000146938	0.390	-0.220	0.101	-0.542	NLGN4X	0.439	~amyloid_beta+(1|manifest)+cngeneson+pc_mito+brain_region+Sex	2.185	2220.000
ENSG00000126883	0.002	-0.221	-0.111	-0.331	NUP214	0.018	~amyloid_beta+(1|manifest)+cngeneson+pc_mito+brain_region+Sex	1.310	3481.000
ENSG00000106049	0.022	-0.221	-0.056	-0.386	HIBADH	0.062	~amyloid_beta+(1|manifest)+cngeneson+pc_mito+brain_region+Sex	0.514	5317.000
ENSG00000143036	0.701	-0.221	0.285	-0.728	SLC44A3	0.730	~amyloid_beta+(1|manifest)+cngeneson+pc_mito+brain_region+Sex	8.516	342.000
ENSG00000132570	0.019	-0.221	-0.085	-0.358	PCBD2	0.058	~amyloid_beta+(1|manifest)+cngeneson+pc_mito+brain_region+Sex	1.536	3074.000
ENSG00000185989	0.039	-0.221	-0.042	-0.400	RASA3	0.087	~amyloid_beta+(1|manifest)+cngeneson+pc_mito+brain_region+Sex	1.734	2772.000
ENSG00000162105	0.097	-0.221	-0.042	-0.401	SHANK2	0.152	~amyloid_beta+(1|manifest)+cngeneson+pc_mito+brain_region+Sex	2.500	1905.000
ENSG00000118690	0.153	-0.222	0.037	-0.481	ARMC2	0.208	~amyloid_beta+(1|manifest)+cngeneson+pc_mito+brain_region+Sex	3.007	1539.000
ENSG00000142856	0.296	-0.222	0.040	-0.484	ITGB3BP	0.348	~amyloid_beta+(1|manifest)+cngeneson+pc_mito+brain_region+Sex	3.763	1137.000
ENSG00000083123	0.088	-0.223	-0.009	-0.436	BCKDHB	0.143	~amyloid_beta+(1|manifest)+cngeneson+pc_mito+brain_region+Sex	4.668	852.000
ENSG00000169006	0.109	-0.223	0.070	-0.517	NTSR2	0.165	~amyloid_beta+(1|manifest)+cngeneson+pc_mito+brain_region+Sex	3.686	1171.000
ENSG00000139746	0.006	-0.223	-0.092	-0.354	RBM26	0.028	~amyloid_beta+(1|manifest)+cngeneson+pc_mito+brain_region+Sex	2.808	1659.000
ENSG00000119397	0.020	-0.223	-0.073	-0.374	CNTRL	0.060	~amyloid_beta+(1|manifest)+cngeneson+pc_mito+brain_region+Sex	0.870	4439.000
ENSG00000136813	0.050	-0.224	-0.040	-0.407	ECPAS	0.099	~amyloid_beta+(1|manifest)+cngeneson+pc_mito+brain_region+Sex	3.476	1257.000
ENSG00000039123	0.004	-0.224	-0.100	-0.348	MTREX	0.024	~amyloid_beta+(1|manifest)+cngeneson+pc_mito+brain_region+Sex	1.666	2879.000
ENSG00000100994	0.194	-0.224	0.138	-0.586	PYGB	0.248	~amyloid_beta+(1|manifest)+cngeneson+pc_mito+brain_region+Sex	5.228	721.000
ENSG00000158966	0.013	-0.225	-0.073	-0.377	CACHD1	0.046	~amyloid_beta+(1|manifest)+cngeneson+pc_mito+brain_region+Sex	5.026	769.000
ENSG00000159267	0.079	-0.225	-0.038	-0.413	HLCS	0.134	~amyloid_beta+(1|manifest)+cngeneson+pc_mito+brain_region+Sex	1.736	2769.000
ENSG00000168172	0.004	-0.225	-0.057	-0.394	HOOK3	0.024	~amyloid_beta+(1|manifest)+cngeneson+pc_mito+brain_region+Sex	2.348	2051.000
ENSG00000134042	0.141	-0.225	0.006	-0.457	MRO	0.197	~amyloid_beta+(1|manifest)+cngeneson+pc_mito+brain_region+Sex	2.956	1567.000
ENSG00000138463	0.001	-0.226	-0.113	-0.339	SLC49A4	0.010	~amyloid_beta+(1|manifest)+cngeneson+pc_mito+brain_region+Sex	1.410	3307.000
ENSG00000048471	0.294	-0.226	0.056	-0.508	SNX29	0.347	~amyloid_beta+(1|manifest)+cngeneson+pc_mito+brain_region+Sex	3.939	1068.000
ENSG00000161040	0.179	-0.226	-0.002	-0.451	FBXL13	0.233	~amyloid_beta+(1|manifest)+cngeneson+pc_mito+brain_region+Sex	3.492	1253.000
ENSG00000180902	0.213	-0.226	0.025	-0.478	D2HGDH	0.267	~amyloid_beta+(1|manifest)+cngeneson+pc_mito+brain_region+Sex	4.586	868.000
ENSG00000197323	0.042	-0.227	-0.056	-0.398	TRIM33	0.090	~amyloid_beta+(1|manifest)+cngeneson+pc_mito+brain_region+Sex	2.210	2192.000
ENSG00000168710	0.120	-0.227	0.002	-0.455	AHCYL1	0.176	~amyloid_beta+(1|manifest)+cngeneson+pc_mito+brain_region+Sex	24.755	31.000
ENSG00000014824	0.027	-0.227	-0.030	-0.424	SLC30A9	0.070	~amyloid_beta+(1|manifest)+cngeneson+pc_mito+brain_region+Sex	2.549	1857.000
ENSG00000054392	0.019	-0.227	-0.065	-0.388	HHAT	0.058	~amyloid_beta+(1|manifest)+cngeneson+pc_mito+brain_region+Sex	2.024	2406.000
ENSG00000196663	0.172	-0.227	0.021	-0.475	TECPR2	0.227	~amyloid_beta+(1|manifest)+cngeneson+pc_mito+brain_region+Sex	2.173	2231.000
ENSG00000183337	0.293	-0.227	0.127	-0.581	BCOR	0.346	~amyloid_beta+(1|manifest)+cngeneson+pc_mito+brain_region+Sex	13.463	137.000
ENSG00000196782	0.124	-0.227	-0.030	-0.424	MAML3	0.181	~amyloid_beta+(1|manifest)+cngeneson+pc_mito+brain_region+Sex	1.702	2818.000
ENSG00000187824	0.013	-0.228	-0.076	-0.379	TMEM220	0.046	~amyloid_beta+(1|manifest)+cngeneson+pc_mito+brain_region+Sex	1.486	3165.000
ENSG00000115504	0.201	-0.228	0.029	-0.484	EHBP1	0.256	~amyloid_beta+(1|manifest)+cngeneson+pc_mito+brain_region+Sex	25.420	28.000
ENSG00000188158	0.264	-0.228	0.048	-0.505	NHS	0.317	~amyloid_beta+(1|manifest)+cngeneson+pc_mito+brain_region+Sex	7.818	388.000
ENSG00000162409	0.010	-0.228	-0.099	-0.358	PRKAA2	0.040	~amyloid_beta+(1|manifest)+cngeneson+pc_mito+brain_region+Sex	1.520	3105.000
ENSG00000146963	0.029	-0.229	-0.058	-0.399	LUC7L2	0.073	~amyloid_beta+(1|manifest)+cngeneson+pc_mito+brain_region+Sex	2.544	1861.000
ENSG00000164074	0.120	-0.229	0.006	-0.464	ABHD18	0.176	~amyloid_beta+(1|manifest)+cngeneson+pc_mito+brain_region+Sex	2.802	1664.000
ENSG00000113645	0.045	-0.229	-0.039	-0.419	WWC1	0.093	~amyloid_beta+(1|manifest)+cngeneson+pc_mito+brain_region+Sex	3.771	1132.000
ENSG00000196199	0.159	-0.229	0.080	-0.538	[illegible]	0.214	~amyloid_beta+(1|manifest)+cngeneson+pc_mito+brain_region+Sex	4.116	1009.000
ENSG00000123096	0.316	-0.230	0.130	-0.589	SSPN	0.368	~amyloid_beta+(1|manifest)+cngeneson+pc_mito+brain_region+Sex	3.859	1101.000
ENSG00000157869	0.109	-0.230	-0.015	-0.444	RAB28	0.165	~amyloid_beta+(1|manifest)+cngeneson+pc_mito+brain_region+Sex	3.503	1250.000
ENSG00000178927	0.146	-0.230	0.036	-0.497	CYBC1	0.202	~amyloid_beta+(1|manifest)+cngeneson+pc_mito+brain_region+Sex	2.973	1556.000
ENSG00000148604	0.238	-0.231	0.012	-0.473	RGR	0.291	~amyloid_beta+(1|manifest)+cngeneson+pc_mito+brain_region+Sex	5.160	737.000
ENSG00000069431	0.696	-0.231	0.306	-0.767	ABCC9	0.726	~amyloid_beta+(1|manifest)+cngeneson+pc_mito+brain_region+Sex	12.713	161.000
ENSG00000077097	0.001	-0.231	-0.114	-0.348	TOP2B	0.013	~amyloid_beta+(1|manifest)+cngeneson+pc_mito+brain_region+Sex	1.503	3133.000
ENSG00000116406	0.096	-0.232	-0.037	-0.427	EDEM3	0.151	~amyloid_beta+(1|manifest)+cngeneson+pc_mito+brain_region+Sex	3.085	1491.000
ENSG00000144566	0.008	-0.232	-0.080	-0.384	RAB5A	0.036	~amyloid_beta+(1|manifest)+cngeneson+pc_mito+brain_region+Sex	2.028	2398.000
ENSG00000152582	0.048	-0.232	-0.064	-0.400	SPEF2	0.097	~amyloid_beta+(1|manifest)+cngeneson+pc_mito+brain_region+Sex	1.245	3611.000
ENSG00000023697	0.022	-0.233	-0.091	-0.374	DERA	0.063	~amyloid_beta+(1|manifest)+cngeneson+pc_mito+brain_region+Sex	3.231	1408.000
ENSG00000162402	0.112	-0.233	-0.019	-0.447	USP24	0.168	~amyloid_beta+(1|manifest)+cngeneson+pc_mito+brain_region+Sex	3.798	1122.000
ENSG00000172954	0.002	-0.233	-0.124	-0.343	LCLAT1	0.016	~amyloid_beta+(1|manifest)+cngeneson+pc_mito+brain_region+Sex	0.808	4606.000
ENSG00000129244	0.236	-0.233	0.060	-0.527	ATP1B2	0.290	~amyloid_beta+(1|manifest)+cngeneson+pc_mito+brain_region+Sex	13.021	150.000
ENSG00000182667	0.006	-0.234	-0.103	-0.364	NTM	0.029	~amyloid_beta+(1|manifest)+cngeneson+pc_mito+brain_region+Sex	10.804	231.000
ENSG00000119509	0.000	-0.235	-0.133	-0.337	INVS	0.004	~amyloid_beta+(1|manifest)+cngeneson+pc_mito+brain_region+Sex	1.630	2937.000
ENSG00000157259	0.117	-0.235	0.030	-0.500	GATAD1	0.174	~amyloid_beta+(1|manifest)+cngeneson+pc_mito+brain_region+Sex	3.513	1241.000
ENSG00000185046	0.077	-0.235	-0.007	-0.463	ANKS1B	0.132	~amyloid_beta+(1|manifest)+cngeneson+pc_mito+brain_region+Sex	0.850	4499.000
ENSG00000198961	0.108	-0.235	-0.028	-0.443	PJA2	0.163	~amyloid_beta+(1|manifest)+cngeneson+pc_mito+brain_region+Sex	3.621	1195.000
ENSG00000169570	0.047	-0.236	-0.073	-0.398	DTWD2	0.096	~amyloid_beta+(1|manifest)+cngeneson+pc_mito+brain_region+Sex	1.706	2815.000
ENSG00000179104	0.124	-0.236	0.001	-0.473	TMTC2	0.181	~amyloid_beta+(1|manifest)+cngeneson+pc_mito+brain_region+Sex	9.107	314.000
ENSG00000118482	0.279	-0.236	0.049	-0.520	PHF3	0.332	~amyloid_beta+(1|manifest)+cngeneson+pc_mito+brain_region+Sex	4.161	995.000
ENSG00000141469	0.685	-0.236	0.331	-0.803	SLC14A1	0.716	~amyloid_beta+(1|manifest)+cngeneson+pc_mito+brain_region+Sex	9.781	281.000
ENSG00000141837	0.185	-0.236	-0.013	-0.459	CACNA1A	0.239	~amyloid_beta+(1|manifest)+cngeneson+pc_mito+brain_region+Sex	4.212	976.000
ENSG00000113448	0.284	-0.236	0.081	-0.553	PDE4D	0.337	~amyloid_beta+(1|manifest)+cngeneson+pc_mito+brain_region+Sex	9.847	277.000
ENSG00000196284	0.002	-0.236	-0.121	-0.351	SUPT3H	0.017	~amyloid_beta+(1|manifest)+cngeneson+pc_mito+brain_region+Sex	1.876	2595.000
ENSG00000076344	0.209	-0.237	0.183	-0.656	RGS11	0.263	~amyloid_beta+(1|manifest)+cngeneson+pc_mito+brain_region+Sex	7.592	406.000
ENSG00000175455	0.182	-0.237	0.066	-0.540	CCDC14	0.237	~amyloid_beta+(1|manifest)+cngeneson+pc_mito+brain_region+Sex	2.461	1929.000
ENSG00000162407	0.408	-0.237	0.154	-0.627	PLPP3	0.455	~amyloid_beta+(1|manifest)+cngeneson+pc_mito+brain_region+Sex	6.165	571.000
ENSG00000153113	0.299	-0.237	0.094	-0.568	CAST	0.351	~amyloid_beta+(1|manifest)+cngeneson+pc_mito+brain_region+Sex	4.762	831.000
ENSG00000169239	0.045	-0.237	-0.043	-0.432	CA5B	0.094	~amyloid_beta+(1|manifest)+cngeneson+pc_mito+brain_region+Sex	2.480	1915.000
ENSG00000169641	0.102	-0.238	-0.029	-0.447	LUZP1	0.157	~amyloid_beta+(1|manifest)+cngeneson+pc_mito+brain_region+Sex	3.529	1233.000
ENSG00000160712	0.088	-0.238	-0.046	-0.431	IL6R	0.143	~amyloid_beta+(1|manifest)+cngeneson+pc_mito+brain_region+Sex	2.333	2067.000
ENSG00000005700	0.130	-0.238	0.011	-0.488	IBTK	0.187	~amyloid_beta+(1|manifest)+cngeneson+pc_mito+brain_region+Sex	4.976	782.000
ENSG00000074410	0.066	-0.238	-0.053	-0.423	CA12	0.120	~amyloid_beta+(1|manifest)+cngeneson+pc_mito+brain_region+Sex	8.996	315.000
ENSG00000127995	0.002	-0.238	-0.120	-0.357	CASD1	0.017	~amyloid_beta+(1|manifest)+cngeneson+pc_mito+brain_region+Sex	1.862	2611.000
ENSG00000128000	0.079	-0.239	0.034	-0.512	ZNF780B	0.134	~amyloid_beta+(1|manifest)+cngeneson+pc_mito+brain_region+Sex	1.861	2612.000
ENSG00000101938	0.040	-0.239	-0.079	-0.398	CHRDL1	0.088	~amyloid_beta+(1|manifest)+cngeneson+pc_mito+brain_region+Sex	0.706	4862.000
ENSG00000104325	0.237	-0.239	0.065	-0.543	DECR1	0.291	~amyloid_beta+(1|manifest)+cngeneson+pc_mito+brain_region+Sex	3.334	1351.000
ENSG00000137561	0.135	-0.239	0.028	-0.506	TTPA	0.191	~amyloid_beta+(1|manifest)+cngeneson+pc_mito+brain_region+Sex	2.188	2217.000
ENSG00000162604	0.000	-0.240	-0.147	-0.333	TM2D1	0.004	~amyloid_beta+(1|manifest)+cngeneson+pc_mito+brain_region+Sex	1.235	3624.000
ENSG00000053524	0.483	-0.240	0.176	-0.656	MCF2L2	0.527	~amyloid_beta+(1|manifest)+cngeneson+pc_mito+brain_region+Sex	1.349	3414.000
ENSG00000106804	0.001	-0.240	-0.103	-0.377	C5	0.013	~amyloid_beta+(1|manifest)+cngeneson+pc_mito+brain_region+Sex	1.095	3940.000
ENSG00000148481	0.000	-0.240	-0.153	-0.327	MINDY3	0.001	~amyloid_beta+(1|manifest)+cngeneson+pc_mito+brain_region+Sex	0.939	4280.000
ENSG00000164114	0.001	-0.241	-0.116	-0.365	MAP9	0.007	~amyloid_beta+(1|manifest)+cngeneson+pc_mito+brain_region+Sex	0.976	4191.000
ENSG00000285447	0.105	-0.241	0.035	-0.516	ZNF883	0.161	~amyloid_beta+(1|manifest)+cngeneson+pc_mito+brain_region+Sex	4.519	889.000
ENSG00000108848	0.240	-0.242	0.073	-0.556	LUC7L3	0.294	~amyloid_beta+(1|manifest)+cngeneson+pc_mito+brain_region+Sex	15.276	99.000
ENSG00000275342	0.009	-0.242	-0.111	-0.372	PRAG1	0.037	~amyloid_beta+(1|manifest)+cngeneson+pc_mito+brain_region+Sex	1.355	3401.000
ENSG00000143156	0.011	-0.242	-0.094	-0.389	NME7	0.042	~amyloid_beta+(1|manifest)+cngeneson+pc_mito+brain_region+Sex	0.949	4255.000
ENSG00000114315	0.326	-0.243	0.091	-0.576	HES1	0.378	~amyloid_beta+(1|manifest)+cngeneson+pc_mito+brain_region+Sex	4.907	795.000
ENSG00000135999	0.254	-0.243	0.037	-0.523	EPC2	0.308	~amyloid_beta+(1|manifest)+cngeneson+pc_mito+brain_region+Sex	4.585	869.000
ENSG00000110066	0.014	-0.243	-0.088	-0.398	KMT5B	0.047	~amyloid_beta+(1|manifest)+cngeneson+pc_mito+brain_region+Sex	1.804	2690.000
ENSG00000044459	0.303	-0.243	0.068	-0.555	CNTLN	0.356	~amyloid_beta+(1|manifest)+cngeneson+pc_mito+brain_region+Sex	2.010	2419.000
ENSG00000166068	0.280	-0.244	0.057	-0.545	SPRED1	0.333	~amyloid_beta+(1|manifest)+cngeneson+pc_mito+brain_region+Sex	3.686	1170.000
ENSG00000162836	0.108	-0.245	-0.020	-0.469	ACP6	0.164	~amyloid_beta+(1|manifest)+cngeneson+pc_mito+brain_region+Sex	2.843	1642.000
ENSG00000160781	0.355	-0.245	0.108	-0.599	PAQR6	0.406	~amyloid_beta+(1|manifest)+cngeneson+pc_mito+brain_region+Sex	6.268	554.000
ENSG00000143344	0.199	-0.245	0.048	-0.539	RGL1	0.253	~amyloid_beta+(1|manifest)+cngeneson+pc_mito+brain_region+Sex	10.336	253.000
ENSG00000073803	0.243	-0.245	0.033	-0.524	MAP3K13	0.297	~amyloid_beta+(1|manifest)+cngeneson+pc_mito+brain_region+Sex	2.294	2114.000
ENSG00000173210	0.365	-0.246	0.060	-0.552	ABLIM3	0.416	~amyloid_beta+(1|manifest)+cngeneson+pc_mito+brain_region+Sex	6.178	567.000
ENSG00000184220	0.143	-0.246	-0.017	-0.475	CMSS1	0.199	~amyloid_beta+(1|manifest)+cngeneson+pc_mito+brain_region+Sex	3.175	1439.000
ENSG00000138380	0.011	-0.246	-0.079	-0.413	CARF	0.042	~amyloid_beta+(1|manifest)+cngeneson+pc_mito+brain_region+Sex	2.076	2338.000
ENSG00000154529	0.770	-0.246	0.434	-0.925	CNTNAP3B	0.793	~amyloid_beta+(1|manifest)+cngeneson+pc_mito+brain_region+Sex	18.062	70.000
ENSG00000150625	0.029	-0.246	-0.076	-0.416	GPM6A	0.073	~amyloid_beta+(1|manifest)+cngeneson+pc_mito+brain_region+Sex	17.444	75.000
ENSG00000103978	0.027	-0.246	-0.069	-0.423	TMEM87A	0.069	~amyloid_beta+(1|manifest)+cngeneson+pc_mito+brain_region+Sex	1.834	2650.000
ENSG00000168216	0.010	-0.246	-0.099	-0.394	LMBRD1	0.039	~amyloid_beta+(1|manifest)+cngeneson+pc_mito+brain_region+Sex	1.566	3030.000
ENSG00000107551	0.553	-0.246	0.203	-0.696	RASSF4	0.593	~amyloid_beta+(1|manifest)+cngeneson+pc_mito+brain_region+Sex	7.570	407.000
ENSG00000154743	0.003	-0.247	-0.120	-0.374	TSEN2	0.018	~amyloid_beta+(1|manifest)+cngeneson+pc_mito+brain_region+Sex	0.792	4650.000
ENSG00000109680	0.030	-0.247	-0.033	-0.461	TBC1D19	0.074	~amyloid_beta+(1|manifest)+cngeneson+pc_mito+brain_region+Sex	1.162	3785.000
ENSG00000115084	0.006	-0.247	-0.104	-0.390	SLC35F5	0.029	~amyloid_beta+(1|manifest)+cngeneson+pc_mito+brain_region+Sex	1.406	3312.000
ENSG00000115266	0.662	-0.248	0.321	-0.817	APC2	0.694	~amyloid_beta+(1|manifest)+cngeneson+pc_mito+brain_region+Sex	16.330	82.000
ENSG00000166669	0.141	-0.248	-0.022	-0.473	ATF7IP2	0.197	~amyloid_beta+(1|manifest)+cngeneson+pc_mito+brain_region+Sex	1.515	3112.000
ENSG00000073711	0.147	-0.248	0.017	-0.512	PPP2R3A	0.203	~amyloid_beta+(1|manifest)+cngeneson+pc_mito+brain_region+Sex	4.202	984.000
ENSG00000181744	0.013	-0.248	-0.071	-0.425	DIPK2A	0.047	~amyloid_beta+(1|manifest)+cngeneson+pc_mito+brain_region+Sex	1.963	2472.000
ENSG00000030419	0.251	-0.248	0.031	-0.527	IKZF2	0.305	~amyloid_beta+(1|manifest)+cngeneson+pc_mito+brain_region+Sex	4.609	860.000
ENSG00000087470	0.124	-0.248	-0.021	-0.476	DNM1L	0.180	~amyloid_beta+(1|manifest)+cngeneson+pc_mito+brain_region+Sex	3.954	1062.000
ENSG00000137817	0.209	-0.249	0.090	-0.589	PARP6	0.263	~amyloid_beta+(1|manifest)+cngeneson+pc_mito+brain_region+Sex	4.806	823.000
ENSG00000137821	0.017	-0.250	-0.093	-0.406	LRRC49	0.054	~amyloid_beta+(1|manifest)+cngeneson+pc_mito+brain_region+Sex	1.252	3592.000
ENSG00000147274	0.038	-0.250	-0.067	-0.432	RBMX	0.085	~amyloid_beta+(1|manifest)+cngeneson+pc_mito+brain_region+Sex	3.669	1176.000
ENSG00000083290	0.336	-0.250	0.131	-0.630	ULK2	0.388	~amyloid_beta+(1|manifest)+cngeneson+pc_mito+brain_region+Sex	1.600	2985.000
ENSG00000115109	0.077	-0.250	-0.048	-0.452	EPB41L5	0.132	~amyloid_beta+(1|manifest)+cngeneson+pc_mito+brain_region+Sex	3.404	1313.000
ENSG00000160352	0.002	-0.250	-0.133	-0.368	ZNF714	0.016	~amyloid_beta+(1|manifest)+cngeneson+pc_mito+brain_region+Sex	1.567	3026.000
ENSG00000131016	0.542	-0.250	0.222	-0.722	AKAP12	0.583	~amyloid_beta+(1|manifest)+cngeneson+pc_mito+brain_region+Sex	1.336	3435.000
ENSG00000257335	0.034	-0.250	-0.048	-0.453	MGAM	0.079	~amyloid_beta+(1|manifest)+cngeneson+pc_mito+brain_region+Sex	3.615	1200.000
ENSG00000187672	0.024	-0.250	-0.093	-0.408	ERC2	0.066	~amyloid_beta+(1|manifest)+cngeneson+pc_mito+brain_region+Sex	2.437	1946.000
ENSG00000164344	0.563	-0.250	0.192	-0.693	KLKB1	0.603	~amyloid_beta+(1|manifest)+cngeneson+pc_mito+brain_region+Sex	7.183	453.000
ENSG00000101745	0.306	-0.251	0.087	-0.588	ANKRD12	0.358	~amyloid_beta+(1|manifest)+cngeneson+pc_mito+brain_region+Sex	5.355	699.000
ENSG00000143786	0.389	-0.251	0.091	-0.594	CNIH3	0.438	~amyloid_beta+(1|manifest)+cngeneson+pc_mito+brain_region+Sex	4.191	988.000
ENSG00000119408	0.235	-0.251	0.071	-0.573	NEK6	0.289	~amyloid_beta+(1|manifest)+cngeneson+pc_mito+brain_region+Sex	2.771	1689.000
ENSG00000138448	0.224	-0.252	0.034	-0.537	ITGAV	0.279	~amyloid_beta+(1|manifest)+cngeneson+pc_mito+brain_region+Sex	8.427	350.000
ENSG00000173193	0.036	-0.252	-0.088	-0.416	PARP14	0.082	~amyloid_beta+(1|manifest)+cngeneson+pc_mito+brain_region+Sex	3.845	1106.000
ENSG00000104412	0.004	-0.253	-0.106	-0.400	EMC2	0.024	~amyloid_beta+(1|manifest)+cngeneson+pc_mito+brain_region+Sex	1.466	3194.000
ENSG00000139117	0.747	-0.253	0.422	-0.929	CPNE8	0.772	~amyloid_beta+(1|manifest)+cngeneson+pc_mito+brain_region+Sex	16.002	87.000
ENSG00000164749	0.060	-0.253	-0.068	-0.439	HNF4G	0.112	~amyloid_beta+(1|manifest)+cngeneson+pc_mito+brain_region+Sex	1.680	2858.000
ENSG00000131873	0.050	-0.254	-0.056	-0.452	CHSY1	0.099	~amyloid_beta+(1|manifest)+cngeneson+pc_mito+brain_region+Sex	1.043	4039.000
ENSG00000148672	0.018	-0.254	-0.067	-0.441	GLUD1	0.056	~amyloid_beta+(1|manifest)+cngeneson+pc_mito+brain_region+Sex	5.117	749.000
ENSG00000104375	0.269	-0.254	0.080	-0.588	STK3	0.322	~amyloid_beta+(1|manifest)+cngeneson+pc_mito+brain_region+Sex	11.704	191.000
ENSG00000107643	0.085	-0.255	-0.013	-0.496	MAPK8	0.140	~amyloid_beta+(1|manifest)+cngeneson+pc_mito+brain_region+Sex	4.101	1014.000
ENSG00000185621	0.002	-0.255	-0.131	-0.378	LMLN	0.017	~amyloid_beta+(1|manifest)+cngeneson+pc_mito+brain_region+Sex	1.426	3274.000
ENSG00000122484	0.030	-0.255	-0.067	-0.443	RPAP2	0.075	~amyloid_beta+(1|manifest)+cngeneson+pc_mito+brain_region+Sex	2.646	1774.000
ENSG00000166682	0.039	-0.255	-0.054	-0.456	TMPRSS5	0.086	~amyloid_beta+(1|manifest)+cngeneson+pc_mito+brain_region+Sex	3.086	1490.000
ENSG00000106415	0.400	-0.255	0.102	-0.613	GLCCI1	0.448	~amyloid_beta+(1|manifest)+cngeneson+pc_mito+brain_region+Sex	5.705	648.000
ENSG00000113578	0.171	-0.255	-0.007	-0.504	FGF1	0.227	~amyloid_beta+(1|manifest)+cngeneson+pc_mito+brain_region+Sex	2.359	2039.000
ENSG00000169783	0.369	-0.255	0.223	-0.734	LINGO1	0.419	~amyloid_beta+(1|manifest)+cngeneson+pc_mito+brain_region+Sex	78.034	1.000
ENSG00000155903	0.026	-0.256	-0.083	-0.428	RASA2	0.069	~amyloid_beta+(1|manifest)+cngeneson+pc_mito+brain_region+Sex	2.591	1818.000
ENSG00000075539	0.002	-0.256	-0.099	-0.413	FRYL	0.017	~amyloid_beta+(1|manifest)+cngeneson+pc_mito+brain_region+Sex	3.367	1335.000
ENSG00000138336	0.100	-0.256	-0.013	-0.498	TET1	0.156	~amyloid_beta+(1|manifest)+cngeneson+pc_mito+brain_region+Sex	2.958	1564.000
ENSG00000196405	0.029	-0.256	-0.074	-0.438	EVL	0.073	~amyloid_beta+(1|manifest)+cngeneson+pc_mito+brain_region+Sex	1.533	3080.000
ENSG00000117625	0.102	-0.256	-0.031	-0.481	RCOR3	0.157	~amyloid_beta+(1|manifest)+cngeneson+pc_mito+brain_region+Sex	3.295	1371.000
ENSG00000175600	0.180	-0.256	0.002	-0.515	SUGCT	0.234	~amyloid_beta+(1|manifest)+cngeneson+pc_mito+brain_region+Sex	2.599	1813.000
ENSG00000144674	0.153	-0.257	0.011	-0.525	GOLGA4	0.208	~amyloid_beta+(1|manifest)+cngeneson+pc_mito+brain_region+Sex	5.346	703.000
ENSG00000198648	0.010	-0.257	-0.108	-0.407	STK39	0.039	~amyloid_beta+(1|manifest)+cngeneson+pc_mito+brain_region+Sex	0.495	5373.000
ENSG00000174776	0.460	-0.258	0.328	-0.843	WDR49	0.505	~amyloid_beta+(1|manifest)+cngeneson+pc_mito+brain_region+Sex	1.795	2708.000
ENSG00000153904	0.005	-0.258	-0.089	-0.426	DDAH1	0.026	~amyloid_beta+(1|manifest)+cngeneson+pc_mito+brain_region+Sex	1.715	2802.000
ENSG00000108587	0.010	-0.258	-0.087	-0.430	GOSR1	0.039	~amyloid_beta+(1|manifest)+cngeneson+pc_mito+brain_region+Sex	2.682	1742.000
ENSG00000119231	0.123	-0.258	-0.011	-0.506	SENP5	0.180	~amyloid_beta+(1|manifest)+cngeneson+pc_mito+brain_region+Sex	3.891	1093.000
ENSG00000154493	0.068	-0.258	-0.054	-0.463	C10orf90	0.122	~amyloid_beta+(1|manifest)+cngeneson+pc_mito+brain_region+Sex	2.965	1562.000
ENSG00000143398	0.198	-0.259	0.015	-0.534	PIP5K1A	0.252	~amyloid_beta+(1|manifest)+cngeneson+pc_mito+brain_region+Sex	3.967	1053.000
ENSG00000073614	0.027	-0.259	-0.084	-0.435	KDM5A	0.071	~amyloid_beta+(1|manifest)+cngeneson+pc_mito+brain_region+Sex	1.671	2869.000
ENSG00000064692	0.098	-0.259	-0.045	-0.474	SNCAIP	0.153	~amyloid_beta+(1|manifest)+cngeneson+pc_mito+brain_region+Sex	2.166	2244.000
ENSG00000118454	0.027	-0.260	-0.082	-0.438	ANKRD13C	0.070	~amyloid_beta+(1|manifest)+cngeneson+pc_mito+brain_region+Sex	2.379	2017.000
ENSG00000079156	0.263	-0.260	0.055	-0.575	OSBPL6	0.317	~amyloid_beta+(1|manifest)+cngeneson+pc_mito+brain_region+Sex	2.257	2146.000
ENSG00000197044	0.000	-0.260	-0.135	-0.385	ZNF441	0.007	~amyloid_beta+(1|manifest)+cngeneson+pc_mito+brain_region+Sex	2.046	2373.000
ENSG00000163075	0.023	-0.260	-0.077	-0.443	CFAP221	0.065	~amyloid_beta+(1|manifest)+cngeneson+pc_mito+brain_region+Sex	1.940	2506.000
ENSG00000184500	0.289	-0.260	0.030	-0.551	PROS1	0.342	~amyloid_beta+(1|manifest)+cngeneson+pc_mito+brain_region+Sex	10.710	233.000
ENSG00000079819	0.159	-0.261	0.008	-0.529	EPB41L2	0.214	~amyloid_beta+(1|manifest)+cngeneson+pc_mito+brain_region+Sex	3.113	1476.000
ENSG00000100731	0.025	-0.261	-0.074	-0.448	PCNX1	0.068	~amyloid_beta+(1|manifest)+cngeneson+pc_mito+brain_region+Sex	3.398	1319.000
ENSG00000163590	0.004	-0.261	-0.113	-0.410	PPM1L	0.025	~amyloid_beta+(1|manifest)+cngeneson+pc_mito+brain_region+Sex	1.760	2737.000
ENSG00000170248	0.014	-0.262	-0.100	-0.424	PDCD6IP	0.047	~amyloid_beta+(1|manifest)+cngeneson+pc_mito+brain_region+Sex	2.463	1928.000
ENSG00000101639	0.028	-0.262	-0.079	-0.446	CEP192	0.072	~amyloid_beta+(1|manifest)+cngeneson+pc_mito+brain_region+Sex	2.417	1964.000
ENSG00000140470	0.414	-0.262	0.121	-0.646	[illegible]	0.461	~amyloid_beta+(1|manifest)+cngeneson+pc_mito+brain_region+Sex	4.858	806.000
ENSG00000106078	0.314	-0.263	0.079	-0.605	COBL	0.367	~amyloid_beta+(1|manifest)+cngeneson+pc_mito+brain_region+Sex	4.122	1007.000
ENSG00000182197	0.023	-0.263	-0.084	-0.442	EXT1	0.064	~amyloid_beta+(1|manifest)+cngeneson+pc_mito+brain_region+Sex	1.385	3347.000
ENSG00000185189	0.121	-0.264	0.063	-0.590	NRBP2	0.178	~amyloid_beta+(1|manifest)+cngeneson+pc_mito+brain_region+Sex	6.562	519.000
ENSG00000120733	0.000	-0.264	-0.147	-0.381	KDM3B	0.006	~amyloid_beta+(1|manifest)+cngeneson+pc_mito+brain_region+Sex	1.146	3822.000
ENSG00000162706	0.032	-0.264	-0.020	-0.507	CADM3	0.077	~amyloid_beta+(1|manifest)+cngeneson+pc_mito+brain_region+Sex	2.821	1651.000
ENSG00000143952	0.008	-0.264	-0.115	-0.413	VPS54	0.035	~amyloid_beta+(1|manifest)+cngeneson+pc_mito+brain_region+Sex	2.298	2111.000
ENSG00000100100	0.134	-0.264	0.017	-0.545	PIK3IP1	0.191	~amyloid_beta+(1|manifest)+cngeneson+pc_mito+brain_region+Sex	2.058	2358.000
ENSG00000104419	0.507	-0.264	0.187	-0.716	NDRG1	0.551	~amyloid_beta+(1|manifest)+cngeneson+pc_mito+brain_region+Sex	2.302	2104.000
ENSG00000154065	0.026	-0.264	-0.078	-0.451	ANKRD29	0.068	~amyloid_beta+(1|manifest)+cngeneson+pc_mito+brain_region+Sex	1.417	3298.000
ENSG00000171503	0.006	-0.265	-0.111	-0.418	ETFDH	0.029	~amyloid_beta+(1|manifest)+cngeneson+pc_mito+brain_region+Sex	2.367	2030.000
ENSG00000165424	0.422	-0.265	0.150	-0.680	ZCCHC24	0.468	~amyloid_beta+(1|manifest)+cngeneson+pc_mito+brain_region+Sex	5.365	696.000
ENSG00000148090	0.334	-0.265	0.085	-0.615	AUH	0.386	~amyloid_beta+(1|manifest)+cngeneson+pc_mito+brain_region+Sex	8.635	335.000
ENSG00000055163	0.146	-0.266	0.006	-0.537	CYFIP2	0.202	~amyloid_beta+(1|manifest)+cngeneson+pc_mito+brain_region+Sex	4.274	956.000
ENSG00000184194	0.027	-0.267	-0.030	-0.503	GPR173	0.069	~amyloid_beta+(1|manifest)+cngeneson+pc_mito+brain_region+Sex	2.331	2069.000
ENSG00000107560	0.120	-0.267	-0.016	-0.519	RAB11FIP2	0.176	~amyloid_beta+(1|manifest)+cngeneson+pc_mito+brain_region+Sex	2.759	1698.000
ENSG00000164037	0.015	-0.268	-0.110	-0.425	SLC9B1	0.051	~amyloid_beta+(1|manifest)+cngeneson+pc_mito+brain_region+Sex	1.718	2800.000
ENSG00000081377	0.173	-0.268	0.014	-0.550	CDC14B	0.228	~amyloid_beta+(1|manifest)+cngeneson+pc_mito+brain_region+Sex	2.943	1580.000
ENSG00000085982	0.018	-0.268	-0.071	-0.465	USP40	0.055	~amyloid_beta+(1|manifest)+cngeneson+pc_mito+brain_region+Sex	2.301	2107.000
ENSG00000153902	0.363	-0.269	0.122	-0.660	LGI4	0.414	~amyloid_beta+(1|manifest)+cngeneson+pc_mito+brain_region+Sex	4.333	938.000
ENSG00000162664	0.003	-0.269	-0.119	-0.420	ZNF326	0.021	~amyloid_beta+(1|manifest)+cngeneson+pc_mito+brain_region+Sex	2.013	2416.000
ENSG00000151892	0.096	-0.269	-0.055	-0.484	GFRA1	0.151	~amyloid_beta+(1|manifest)+cngeneson+pc_mito+brain_region+Sex	3.194	1430.000
ENSG00000136754	0.002	-0.269	-0.121	-0.418	ABI1	0.016	~amyloid_beta+(1|manifest)+cngeneson+pc_mito+brain_region+Sex	1.467	3192.000
ENSG00000129151	0.190	-0.270	0.011	-0.551	BBOX1	0.245	~amyloid_beta+(1|manifest)+cngeneson+pc_mito+brain_region+Sex	2.561	1847.000
ENSG00000148057	0.009	-0.270	-0.103	-0.437	IDNK	0.038	~amyloid_beta+(1|manifest)+cngeneson+pc_mito+brain_region+Sex	1.609	2968.000
ENSG00000135063	0.081	-0.270	-0.024	-0.516	[illegible]	0.136	~amyloid_beta+(1|manifest)+cngeneson+pc_mito+brain_region+Sex	7.564	408.000
ENSG00000089280	0.123	-0.270	-0.006	-0.535	FUS	0.179	~amyloid_beta+(1|manifest)+cngeneson+pc_mito+brain_region+Sex	3.052	1507.000
ENSG00000144504	0.110	-0.271	-0.009	-0.532	ANKMY1	0.166	~amyloid_beta+(1|manifest)+cngeneson+pc_mito+brain_region+Sex	2.895	1606.000
ENSG00000139173	0.089	-0.271	-0.040	-0.502	TMEM117	0.144	~amyloid_beta+(1|manifest)+cngeneson+pc_mito+brain_region+Sex	2.664	1759.000
ENSG00000181450	0.043	-0.271	-0.084	-0.458	ZNF678	0.092	~amyloid_beta+(1|manifest)+cngeneson+pc_mito+brain_region+Sex	6.050	585.000
ENSG00000196358	0.204	-0.271	0.129	-0.672	NTNG2	0.258	~amyloid_beta+(1|manifest)+cngeneson+pc_mito+brain_region+Sex	5.315	705.000
ENSG00000111058	0.233	-0.271	0.064	-0.607	ACSS3	0.287	~amyloid_beta+(1|manifest)+cngeneson+pc_mito+brain_region+Sex	10.292	258.000
ENSG00000158623	0.002	-0.272	-0.152	-0.392	COPG2	0.015	~amyloid_beta+(1|manifest)+cngeneson+pc_mito+brain_region+Sex	1.946	2500.000
ENSG00000169918	0.252	-0.272	0.081	-0.625	OTUD7A	0.307	~amyloid_beta+(1|manifest)+cngeneson+pc_mito+brain_region+Sex	9.431	302.000
ENSG00000135976	0.081	-0.272	-0.004	-0.540	ANKRD36	0.136	~amyloid_beta+(1|manifest)+cngeneson+pc_mito+brain_region+Sex	1.652	2902.000
ENSG00000182168	0.583	-0.273	0.246	-0.792	UNC5C	0.620	~amyloid_beta+(1|manifest)+cngeneson+pc_mito+brain_region+Sex	11.821	185.000
ENSG00000169122	0.000	-0.273	-0.173	-0.373	FAM110B	0.004	~amyloid_beta+(1|manifest)+cngeneson+pc_mito+brain_region+Sex	0.701	4884.000
ENSG00000198185	0.016	-0.274	-0.038	-0.510	ZNF334	0.053	~amyloid_beta+(1|manifest)+cngeneson+pc_mito+brain_region+Sex	0.780	4668.000
ENSG00000107614	0.000	-0.274	-0.163	-0.386	TRDMT1	0.006	~amyloid_beta+(1|manifest)+cngeneson+pc_mito+brain_region+Sex	0.822	4565.000
ENSG00000182150	0.068	-0.275	-0.049	-0.502	ERCC6L2	0.121	~amyloid_beta+(1|manifest)+cngeneson+pc_mito+brain_region+Sex	4.149	1001.000
ENSG00000055208	0.200	-0.276	0.045	-0.596	TAB2	0.254	~amyloid_beta+(1|manifest)+cngeneson+pc_mito+brain_region+Sex	8.127	369.000
ENSG00000121067	0.150	-0.276	-0.008	-0.543	SPOP	0.205	~amyloid_beta+(1|manifest)+cngeneson+pc_mito+brain_region+Sex	3.626	1192.000
ENSG00000118564	0.020	-0.278	-0.091	-0.464	FBXL5	0.059	~amyloid_beta+(1|manifest)+cngeneson+pc_mito+brain_region+Sex	3.762	1138.000
ENSG00000198815	0.111	-0.279	-0.023	-0.534	FOXJ3	0.167	~amyloid_beta+(1|manifest)+cngeneson+pc_mito+brain_region+Sex	6.712	507.000
ENSG00000029363	0.140	-0.279	-0.018	-0.541	BCLAF1	0.196	~amyloid_beta+(1|manifest)+cngeneson+pc_mito+brain_region+Sex	3.638	1186.000
ENSG00000050426	0.051	-0.280	-0.033	-0.528	LETMD1	0.101	~amyloid_beta+(1|manifest)+cngeneson+pc_mito+brain_region+Sex	2.993	1546.000
ENSG00000135968	0.024	-0.281	-0.073	-0.488	GCC2	0.065	~amyloid_beta+(1|manifest)+cngeneson+pc_mito+brain_region+Sex	1.683	2854.000
ENSG00000187742	0.023	-0.281	-0.090	-0.472	SECISBP2	0.065	~amyloid_beta+(1|manifest)+cngeneson+pc_mito+brain_region+Sex	2.432	1955.000
ENSG00000136636	0.377	-0.281	0.121	-0.683	KCTD3	0.427	~amyloid_beta+(1|manifest)+cngeneson+pc_mito+brain_region+Sex	4.664	854.000
ENSG00000147324	0.157	-0.281	0.046	-0.609	MFHAS1	0.212	~amyloid_beta+(1|manifest)+cngeneson+pc_mito+brain_region+Sex	8.181	363.000
ENSG00000197857	0.060	-0.281	-0.070	-0.493	ZNF44	0.112	~amyloid_beta+(1|manifest)+cngeneson+pc_mito+brain_region+Sex	2.163	2246.000
ENSG00000180822	0.155	-0.282	-0.014	-0.549	PSMG4	0.210	~amyloid_beta+(1|manifest)+cngeneson+pc_mito+brain_region+Sex	3.834	1109.000
ENSG00000103657	0.013	-0.282	-0.102	-0.461	HERC1	0.047	~amyloid_beta+(1|manifest)+cngeneson+pc_mito+brain_region+Sex	3.427	1294.000
ENSG00000037749	0.004	-0.282	-0.144	-0.419	MFAP3	0.023	~amyloid_beta+(1|manifest)+cngeneson+pc_mito+brain_region+Sex	1.661	2886.000
ENSG00000134882	0.398	-0.283	0.118	-0.683	UBAC2	0.446	~amyloid_beta+(1|manifest)+cngeneson+pc_mito+brain_region+Sex	13.304	144.000
ENSG00000189195	0.105	-0.283	0.123	-0.689	BTBD8	0.161	~amyloid_beta+(1|manifest)+cngeneson+pc_mito+brain_region+Sex	1.739	2765.000
ENSG00000135336	0.004	-0.284	-0.116	-0.452	ORC3	0.024	~amyloid_beta+(1|manifest)+cngeneson+pc_mito+brain_region+Sex	0.502	5351.000
ENSG00000145725	0.005	-0.284	-0.116	-0.453	PPIP5K2	0.027	~amyloid_beta+(1|manifest)+cngeneson+pc_mito+brain_region+Sex	2.319	2083.000
ENSG00000007392	0.228	-0.285	0.055	-0.625	LUC7L	0.282	~amyloid_beta+(1|manifest)+cngeneson+pc_mito+brain_region+Sex	3.723	1153.000
ENSG00000132837	0.269	-0.285	0.046	-0.617	DMGDH	0.322	~amyloid_beta+(1|manifest)+cngeneson+pc_mito+brain_region+Sex	3.472	1261.000
ENSG00000171476	0.344	-0.286	0.077	-0.648	HOPX	0.395	~amyloid_beta+(1|manifest)+cngeneson+pc_mito+brain_region+Sex	9.559	293.000
ENSG00000169902	0.843	-0.286	0.746	-1.318	TPST1	0.859	~amyloid_beta+(1|manifest)+cngeneson+pc_mito+brain_region+Sex	36.528	9.000
ENSG00000090989	0.001	-0.286	-0.138	-0.434	EXOC1	0.013	~amyloid_beta+(1|manifest)+cngeneson+pc_mito+brain_region+Sex	1.263	3573.000
ENSG00000100503	0.048	-0.287	-0.064	-0.509	NIN	0.098	~amyloid_beta+(1|manifest)+cngeneson+pc_mito+brain_region+Sex	2.783	1679.000
ENSG00000174501	0.088	-0.287	0.008	-0.583	ANKRD36C	0.143	~amyloid_beta+(1|manifest)+cngeneson+pc_mito+brain_region+Sex	1.515	3111.000
ENSG00000174796	0.002	-0.287	-0.142	-0.433	THAP6	0.017	~amyloid_beta+(1|manifest)+cngeneson+pc_mito+brain_region+Sex	1.524	3098.000
ENSG00000139220	0.214	-0.288	0.042	-0.617	PPFIA2	0.268	~amyloid_beta+(1|manifest)+cngeneson+pc_mito+brain_region+Sex	3.467	1267.000

ENSG00000227124	0.115	-0.288	-0.025	-0.550	ZNF717	0.171	~amyloid_beta+(1|manifest)+cngeneson+pc_mito+brain_region+Sex	3.453	1274.000
ENSG00000095564	0.070	-0.288	-0.046	-0.531	BTAF1	0.124	~amyloid_beta+(1|manifest)+cngeneson+pc_mito+brain_region+Sex	2.805	1661.000
ENSG00000083097	0.001	-0.289	-0.154	-0.424	DOP1A	0.013	~amyloid_beta+(1|manifest)+cngeneson+pc_mito+brain_region+Sex	1.951	2492.000
ENSG00000025796	0.024	-0.289	-0.104	-0.474	SEC63	0.066	~amyloid_beta+(1|manifest)+cngeneson+pc_mito+brain_region+Sex	2.435	1950.000
ENSG00000103723	0.003	-0.289	-0.152	-0.426	AP3B2	0.018	~amyloid_beta+(1|manifest)+cngeneson+pc_mito+brain_region+Sex	1.886	2583.000
ENSG00000151466	0.058	-0.289	-0.065	-0.513	SCLT1	0.110	~amyloid_beta+(1|manifest)+cngeneson+pc_mito+brain_region+Sex	2.413	1969.000
ENSG00000104112	0.162	-0.290	0.080	-0.659	SCG3	0.218	~amyloid_beta+(1|manifest)+cngeneson+pc_mito+brain_region+Sex	2.952	1572.000
ENSG00000165487	0.019	-0.290	-0.104	-0.475	MICU2	0.057	~amyloid_beta+(1|manifest)+cngeneson+pc_mito+brain_region+Sex	2.720	1719.000
ENSG00000197763	0.003	-0.290	-0.140	-0.441	TXNRD3	0.018	~amyloid_beta+(1|manifest)+cngeneson+pc_mito+brain_region+Sex	1.494	3148.000
ENSG00000168785	0.007	-0.291	-0.108	-0.473	TSPAN5	0.033	~amyloid_beta+(1|manifest)+cngeneson+pc_mito+brain_region+Sex	1.990	2446.000
ENSG00000173208	0.319	-0.291	0.122	-0.703	ABCD2	0.371	~amyloid_beta+(1|manifest)+cngeneson+pc_mito+brain_region+Sex	7.953	378.000
ENSG00000058091	0.051	-0.291	-0.062	-0.520	CDK14	0.101	~amyloid_beta+(1|manifest)+cngeneson+pc_mito+brain_region+Sex	3.132	1463.000
ENSG00000134186	0.018	-0.291	-0.108	-0.475	PRPF38B	0.056	~amyloid_beta+(1|manifest)+cngeneson+pc_mito+brain_region+Sex	1.692	2838.000
ENSG00000198963	0.091	-0.291	0.008	-0.590	RORB	0.146	~amyloid_beta+(1|manifest)+cngeneson+pc_mito+brain_region+Sex	4.207	982.000
ENSG00000107290	0.079	-0.292	-0.037	-0.546	SETX	0.134	~amyloid_beta+(1|manifest)+cngeneson+pc_mito+brain_region+Sex	3.754	1140.000
ENSG00000188580	0.125	-0.293	0.102	-0.687	NKAIN2	0.182	~amyloid_beta+(1|manifest)+cngeneson+pc_mito+brain_region+Sex	3.025	1527.000
ENSG00000175893	0.160	-0.293	0.010	-0.596	ZDHHC21	0.215	~amyloid_beta+(1|manifest)+cngeneson+pc_mito+brain_region+Sex	10.958	223.000
ENSG00000060718	0.358	-0.293	0.109	-0.696	COL11A1	0.409	~amyloid_beta+(1|manifest)+cngeneson+pc_mito+brain_region+Sex	3.194	1431.000
ENSG00000215421	0.035	-0.294	-0.068	-0.520	ZNF407	0.081	~amyloid_beta+(1|manifest)+cngeneson+pc_mito+brain_region+Sex	3.405	1311.000
ENSG00000137871	0.085	-0.295	-0.009	-0.580	ZNF280D	0.140	~amyloid_beta+(1|manifest)+cngeneson+pc_mito+brain_region+Sex	3.225	1413.000
ENSG00000117069	0.012	-0.295	-0.115	-0.475	[illegible]	0.043	~amyloid_beta+(1|manifest)+cngeneson+pc_mito+brain_region+Sex	2.345	2053.000
ENSG00000136114	0.028	-0.295	-0.065	-0.525	THSD1	0.072	~amyloid_beta+(1|manifest)+cngeneson+pc_mito+brain_region+Sex	2.134	2275.000
ENSG00000125124	0.140	-0.295	0.003	-0.594	BBS2	0.196	~amyloid_beta+(1|manifest)+cngeneson+pc_mito+brain_region+Sex	3.901	1088.000
ENSG00000134824	0.489	-0.296	0.224	-0.815	FADS2	0.533	~amyloid_beta+(1|manifest)+cngeneson+pc_mito+brain_region+Sex	10.961	222.000
ENSG00000151687	0.049	-0.296	-0.063	-0.529	ANKAR	0.099	~amyloid_beta+(1|manifest)+cngeneson+pc_mito+brain_region+Sex	0.663	4971.000
ENSG00000054282	0.000	-0.296	-0.170	-0.422	SDCCAG8	0.003	~amyloid_beta+(1|manifest)+cngeneson+pc_mito+brain_region+Sex	2.713	1721.000
ENSG00000140937	0.025	-0.296	-0.098	-0.494	CDH11	0.066	~amyloid_beta+(1|manifest)+cngeneson+pc_mito+brain_region+Sex	2.050	2369.000
ENSG00000100697	0.034	-0.296	-0.081	-0.511	DICER1	0.080	~amyloid_beta+(1|manifest)+cngeneson+pc_mito+brain_region+Sex	2.769	1691.000
ENSG00000023287	0.031	-0.297	-0.087	-0.506	RB1CC1	0.076	~amyloid_beta+(1|manifest)+cngeneson+pc_mito+brain_region+Sex	1.678	2860.000
ENSG00000104408	0.210	-0.297	0.027	-0.620	EIF3E	0.265	~amyloid_beta+(1|manifest)+cngeneson+pc_mito+brain_region+Sex	2.916	1597.000
ENSG00000110436	0.005	-0.297	-0.132	-0.462	SLC1A2	0.025	~amyloid_beta+(1|manifest)+cngeneson+pc_mito+brain_region+Sex	5.084	758.000
ENSG00000112182	0.340	-0.298	0.117	-0.713	BACH2	0.391	~amyloid_beta+(1|manifest)+cngeneson+pc_mito+brain_region+Sex	2.792	1675.000
ENSG00000112769	0.321	-0.298	0.141	-0.738	LAMA4	0.374	~amyloid_beta+(1|manifest)+cngeneson+pc_mito+brain_region+Sex	4.845	809.000
ENSG00000119906	0.028	-0.298	-0.096	-0.501	SLF2	0.072	~amyloid_beta+(1|manifest)+cngeneson+pc_mito+brain_region+Sex	2.311	2091.000
ENSG00000139946	0.315	-0.299	0.078	-0.677	PELI2	0.368	~amyloid_beta+(1|manifest)+cngeneson+pc_mito+brain_region+Sex	4.253	959.000
ENSG00000074657	0.046	-0.300	-0.068	-0.531	ZNF532	0.095	~amyloid_beta+(1|manifest)+cngeneson+pc_mito+brain_region+Sex	1.947	2499.000
ENSG00000011405	0.095	-0.300	-0.022	-0.577	PIK3C2A	0.150	~amyloid_beta+(1|manifest)+cngeneson+pc_mito+brain_region+Sex	4.941	788.000
ENSG00000135926	0.620	-0.300	0.291	-0.890	TMBIM1	0.656	~amyloid_beta+(1|manifest)+cngeneson+pc_mito+brain_region+Sex	7.556	410.000
ENSG00000124145	0.489	-0.300	0.227	-0.827	SDC4	0.534	~amyloid_beta+(1|manifest)+cngeneson+pc_mito+brain_region+Sex	11.797	186.000
ENSG00000139597	0.153	-0.300	0.004	-0.604	N4BP2L1	0.208	~amyloid_beta+(1|manifest)+cngeneson+pc_mito+brain_region+Sex	3.936	1072.000
ENSG00000198830	0.038	-0.300	-0.065	-0.536	HMGN2	0.086	~amyloid_beta+(1|manifest)+cngeneson+pc_mito+brain_region+Sex	1.566	3031.000
ENSG00000108474	0.059	-0.300	-0.047	-0.554	PIGL	0.110	~amyloid_beta+(1|manifest)+cngeneson+pc_mito+brain_region+Sex	2.767	1694.000
ENSG00000106772	0.004	-0.302	-0.147	-0.456	PRUNE2	0.023	~amyloid_beta+(1|manifest)+cngeneson+pc_mito+brain_region+Sex	0.635	5044.000
ENSG00000166012	0.136	-0.303	-0.005	-0.600	TAF1D	0.192	~amyloid_beta+(1|manifest)+cngeneson+pc_mito+brain_region+Sex	5.282	712.000
ENSG00000116984	0.010	-0.303	-0.116	-0.490	MTR	0.039	~amyloid_beta+(1|manifest)+cngeneson+pc_mito+brain_region+Sex	2.505	1896.000
ENSG00000219626	0.158	-0.303	-0.016	-0.590	FAM228B	0.213	~amyloid_beta+(1|manifest)+cngeneson+pc_mito+brain_region+Sex	2.726	1716.000
ENSG00000115159	0.148	-0.303	0.005	-0.611	GPD2	0.204	~amyloid_beta+(1|manifest)+cngeneson+pc_mito+brain_region+Sex	6.120	579.000
ENSG00000163125	0.020	-0.303	-0.094	-0.513	RPRD2	0.059	~amyloid_beta+(1|manifest)+cngeneson+pc_mito+brain_region+Sex	2.776	1686.000
ENSG00000183066	0.245	-0.303	0.050	-0.657	WBP2NL	0.299	~amyloid_beta+(1|manifest)+cngeneson+pc_mito+brain_region+Sex	4.982	781.000
ENSG00000170153	0.388	-0.304	0.114	-0.722	RNF150	0.438	~amyloid_beta+(1|manifest)+cngeneson+pc_mito+brain_region+Sex	5.187	730.000
ENSG00000164329	0.011	-0.305	-0.115	-0.494	TENT2	0.042	~amyloid_beta+(1|manifest)+cngeneson+pc_mito+brain_region+Sex	2.358	2040.000
ENSG00000028203	0.002	-0.305	-0.154	-0.457	VEZT	0.016	~amyloid_beta+(1|manifest)+cngeneson+pc_mito+brain_region+Sex	3.137	1459.000
ENSG00000112234	0.003	-0.306	-0.129	-0.482	FBXL4	0.020	~amyloid_beta+(1|manifest)+cngeneson+pc_mito+brain_region+Sex	1.570	3023.000
ENSG00000118961	0.010	-0.307	-0.130	-0.483	LDAH	0.039	~amyloid_beta+(1|manifest)+cngeneson+pc_mito+brain_region+Sex	0.811	4598.000
ENSG00000136104	0.001	-0.307	-0.177	-0.436	RNASEH2B	0.007	~amyloid_beta+(1|manifest)+cngeneson+pc_mito+brain_region+Sex	0.435	5495.000
ENSG00000197563	0.011	-0.307	-0.122	-0.491	PIGN	0.041	~amyloid_beta+(1|manifest)+cngeneson+pc_mito+brain_region+Sex	1.562	3036.000
ENSG00000153989	0.001	-0.307	-0.187	-0.427	NUS1	0.007	~amyloid_beta+(1|manifest)+cngeneson+pc_mito+brain_region+Sex	2.301	2105.000
ENSG00000144909	0.389	-0.308	0.146	-0.761	OSBPL11	0.438	~amyloid_beta+(1|manifest)+cngeneson+pc_mito+brain_region+Sex	6.898	478.000
ENSG00000110721	0.186	-0.308	0.037	-0.652	CHKA	0.240	~amyloid_beta+(1|manifest)+cngeneson+pc_mito+brain_region+Sex	6.809	489.000
ENSG00000133226	0.153	-0.309	0.054	-0.673	SRRM1	0.208	~amyloid_beta+(1|manifest)+cngeneson+pc_mito+brain_region+Sex	3.989	1041.000
ENSG00000005108	0.002	-0.310	-0.140	-0.480	THSD7A	0.017	~amyloid_beta+(1|manifest)+cngeneson+pc_mito+brain_region+Sex	2.787	1677.000
ENSG00000130066	0.321	-0.310	0.082	-0.701	SAT1	0.373	~amyloid_beta+(1|manifest)+cngeneson+pc_mito+brain_region+Sex	6.555	520.000
ENSG00000197603	0.024	-0.310	-0.098	-0.522	CPLANE1	0.066	~amyloid_beta+(1|manifest)+cngeneson+pc_mito+brain_region+Sex	3.195	1429.000
ENSG00000187068	0.480	-0.311	0.189	-0.810	C3orf70	0.525	~amyloid_beta+(1|manifest)+cngeneson+pc_mito+brain_region+Sex	10.000	270.000
ENSG00000136758	0.008	-0.311	-0.118	-0.503	YME1L1	0.035	~amyloid_beta+(1|manifest)+cngeneson+pc_mito+brain_region+Sex	2.947	1577.000
ENSG00000101198	0.439	-0.311	0.185	-0.807	NKAIN4	0.484	~amyloid_beta+(1|manifest)+cngeneson+pc_mito+brain_region+Sex	8.773	326.000
ENSG00000163697	0.061	-0.311	-0.088	-0.535	APBB2	0.112	~amyloid_beta+(1|manifest)+cngeneson+pc_mito+brain_region+Sex	1.602	2980.000
ENSG00000122557	0.030	-0.311	-0.101	-0.522	HERPUD2	0.074	~amyloid_beta+(1|manifest)+cngeneson+pc_mito+brain_region+Sex	3.401	1316.000
ENSG00000149292	0.006	-0.311	-0.142	-0.481	TTC12	0.030	~amyloid_beta+(1|manifest)+cngeneson+pc_mito+brain_region+Sex	1.856	2618.000
ENSG00000166974	0.636	-0.312	0.388	-1.012	MAPRE2	0.670	~amyloid_beta+(1|manifest)+cngeneson+pc_mito+brain_region+Sex	18.751	58.000
ENSG00000142687	0.033	-0.313	-0.089	-0.536	KIAA0319L	0.078	~amyloid_beta+(1|manifest)+cngeneson+pc_mito+brain_region+Sex	4.307	947.000
ENSG00000186260	0.570	-0.313	0.271	-0.896	MRTFB	0.609	~amyloid_beta+(1|manifest)+cngeneson+pc_mito+brain_region+Sex	17.570	73.000
ENSG00000188177	0.062	-0.313	-0.080	-0.546	ZC3H6	0.114	~amyloid_beta+(1|manifest)+cngeneson+pc_mito+brain_region+Sex	2.401	1980.000
ENSG00000112249	0.009	-0.313	-0.119	-0.507	ASCC3	0.037	~amyloid_beta+(1|manifest)+cngeneson+pc_mito+brain_region+Sex	2.572	1835.000
ENSG00000169855	0.003	-0.314	-0.150	-0.477	ROBO1	0.019	~amyloid_beta+(1|manifest)+cngeneson+pc_mito+brain_region+Sex	2.045	2377.000
ENSG00000214456	0.365	-0.315	0.142	-0.772	PLIN5	0.416	~amyloid_beta+(1|manifest)+cngeneson+pc_mito+brain_region+Sex	7.052	464.000
ENSG00000004468	0.084	-0.317	-0.046	-0.587	CD38	0.139	~amyloid_beta+(1|manifest)+cngeneson+pc_mito+brain_region+Sex	4.694	842.000
ENSG00000069020	0.000	-0.317	-0.158	-0.476	MAST4	0.006	~amyloid_beta+(1|manifest)+cngeneson+pc_mito+brain_region+Sex	3.518	1242.000
ENSG00000185033	0.057	-0.318	-0.065	-0.571	SEMA4B	0.109	~amyloid_beta+(1|manifest)+cngeneson+pc_mito+brain_region+Sex	3.851	1103.000
ENSG00000150672	0.004	-0.319	-0.121	-0.516	DLG2	0.024	~amyloid_beta+(1|manifest)+cngeneson+pc_mito+brain_region+Sex	6.744	499.000
ENSG00000198948	0.178	-0.320	0.046	-0.686	MFAP3L	0.232	~amyloid_beta+(1|manifest)+cngeneson+pc_mito+brain_region+Sex	5.204	726.000
ENSG00000104093	0.020	-0.321	-0.119	-0.522	DMXL2	0.058	~amyloid_beta+(1|manifest)+cngeneson+pc_mito+brain_region+Sex	1.910	2543.000
ENSG00000196704	0.246	-0.321	0.032	-0.675	AMZ2	0.300	~amyloid_beta+(1|manifest)+cngeneson+pc_mito+brain_region+Sex	3.870	1098.000
ENSG00000145781	0.004	-0.322	-0.127	-0.517	[illegible]	0.025	~amyloid_beta+(1|manifest)+cngeneson+pc_mito+brain_region+Sex	3.450	1276.000
ENSG00000036549	0.005	-0.322	-0.149	-0.495	[illegible]	0.026	~amyloid_beta+(1|manifest)+cngeneson+pc_mito+brain_region+Sex	2.363	2036.000
ENSG00000168769	0.046	-0.322	-0.088	-0.557	TET2	0.095	~amyloid_beta+(1|manifest)+cngeneson+pc_mito+brain_region+Sex	2.565	1843.000
ENSG00000163617	0.013	-0.323	-0.122	-0.523	CCDC191	0.046	~amyloid_beta+(1|manifest)+cngeneson+pc_mito+brain_region+Sex	1.567	3025.000
ENSG00000266302	0.109	-0.323	-0.048	-0.597	[illegible]	0.165	~amyloid_beta+(1|manifest)+cngeneson+pc_mito+brain_region+Sex	3.418	1303.000
ENSG00000117523	0.072	-0.324	-0.046	-0.602	PRRC2C	0.126	~amyloid_beta+(1|manifest)+cngeneson+pc_mito+brain_region+Sex	3.336	1348.000
ENSG00000100523	0.096	-0.324	-0.052	-0.596	DDHD1	0.151	~amyloid_beta+(1|manifest)+cngeneson+pc_mito+brain_region+Sex	5.713	646.000
ENSG00000128849	0.124	-0.325	-0.024	-0.627	CGNL1	0.180	~amyloid_beta+(1|manifest)+cngeneson+pc_mito+brain_region+Sex	3.046	1516.000
ENSG00000196912	0.003	-0.326	-0.171	-0.480	ANKRD36B	0.020	~amyloid_beta+(1|manifest)+cngeneson+pc_mito+brain_region+Sex	1.790	2711.000
ENSG00000112379	0.005	-0.326	-0.157	-0.496	ARFGEF3	0.028	~amyloid_beta+(1|manifest)+cngeneson+pc_mito+brain_region+Sex	1.260	3585.000
ENSG00000165914	0.043	-0.326	-0.071	-0.582	TTC7B	0.091	~amyloid_beta+(1|manifest)+cngeneson+pc_mito+brain_region+Sex	2.893	1609.000
ENSG00000163596	0.041	-0.327	-0.090	-0.563	ICA1L	0.088	~amyloid_beta+(1|manifest)+cngeneson+pc_mito+brain_region+Sex	3.021	1530.000
ENSG00000111879	0.023	-0.327	-0.085	-0.569	FAM184A	0.064	~amyloid_beta+(1|manifest)+cngeneson+pc_mito+brain_region+Sex	2.280	2126.000
ENSG00000185261	0.040	-0.328	-0.103	-0.553	KIAA0825	0.088	~amyloid_beta+(1|manifest)+cngeneson+pc_mito+brain_region+Sex	1.380	3359.000
ENSG00000080224	0.210	-0.328	0.110	-0.765	EPHA6	0.265	~amyloid_beta+(1|manifest)+cngeneson+pc_mito+brain_region+Sex	3.751	1142.000
ENSG00000143951	0.008	-0.328	-0.119	-0.537	WDPCP	0.035	~amyloid_beta+(1|manifest)+cngeneson+pc_mito+brain_region+Sex	1.463	3202.000
ENSG00000138768	0.002	-0.328	-0.171	-0.486	USO1	0.016	~amyloid_beta+(1|manifest)+cngeneson+pc_mito+brain_region+Sex	2.669	1754.000
ENSG00000163288	0.000	-0.329	-0.197	-0.460	GABRB1	0.001	~amyloid_beta+(1|manifest)+cngeneson+pc_mito+brain_region+Sex	4.247	964.000
ENSG00000211448	0.224	-0.329	0.021	-0.679	DIO2	0.279	~amyloid_beta+(1|manifest)+cngeneson+pc_mito+brain_region+Sex	5.501	679.000
ENSG00000196586	0.001	-0.329	-0.146	-0.512	MYO6	0.010	~amyloid_beta+(1|manifest)+cngeneson+pc_mito+brain_region+Sex	4.097	1017.000
ENSG00000162669	0.001	-0.329	-0.190	-0.468	HFM1	0.009	~amyloid_beta+(1|manifest)+cngeneson+pc_mito+brain_region+Sex	0.836	4528.000
ENSG00000168077	0.407	-0.331	0.176	-0.838	SCARA3	0.454	~amyloid_beta+(1|manifest)+cngeneson+pc_mito+brain_region+Sex	9.988	271.000
ENSG00000118495	0.004	-0.331	-0.164	-0.499	PLAGL1	0.024	~amyloid_beta+(1|manifest)+cngeneson+pc_mito+brain_region+Sex	1.038	4050.000
ENSG00000163714	0.002	-0.331	-0.160	-0.502	U2SURP	0.017	~amyloid_beta+(1|manifest)+cngeneson+pc_mito+brain_region+Sex	2.319	2082.000
ENSG00000138670	0.363	-0.332	0.221	-0.884	RASGEF1B	0.414	~amyloid_beta+(1|manifest)+cngeneson+pc_mito+brain_region+Sex	66.925	3.000
ENSG00000143033	0.013	-0.332	-0.122	-0.542	MTF2	0.046	~amyloid_beta+(1|manifest)+cngeneson+pc_mito+brain_region+Sex	2.543	1865.000
ENSG00000166323	0.001	-0.332	-0.191	-0.473	C11orf65	0.010	~amyloid_beta+(1|manifest)+cngeneson+pc_mito+brain_region+Sex	1.008	4122.000
ENSG00000101596	0.005	-0.333	-0.153	-0.512	SMCHD1	0.025	~amyloid_beta+(1|manifest)+cngeneson+pc_mito+brain_region+Sex	2.667	1755.000
ENSG00000196482	0.552	-0.333	0.303	-0.969	ESRRG	0.593	~amyloid_beta+(1|manifest)+cngeneson+pc_mito+brain_region+Sex	15.903	89.000
ENSG00000249853	0.127	-0.334	0.000	-0.668	HS3ST5	0.183	~amyloid_beta+(1|manifest)+cngeneson+pc_mito+brain_region+Sex	2.582	1826.000
ENSG00000102452	0.204	-0.334	0.072	-0.740	NALCN	0.259	~amyloid_beta+(1|manifest)+cngeneson+pc_mito+brain_region+Sex	0.958	4231.000
ENSG00000204128	0.411	-0.335	0.152	-0.822	C2orf72	0.458	~amyloid_beta+(1|manifest)+cngeneson+pc_mito+brain_region+Sex	13.646	131.000
ENSG00000182141	0.219	-0.335	0.026	-0.696	ZNF708	0.274	~amyloid_beta+(1|manifest)+cngeneson+pc_mito+brain_region+Sex	10.424	251.000
ENSG00000114062	0.013	-0.336	-0.117	-0.554	UBE3A	0.046	~amyloid_beta+(1|manifest)+cngeneson+pc_mito+brain_region+Sex	4.131	1005.000
ENSG00000137285	0.508	-0.337	0.224	-0.899	TUBB2B	0.551	~amyloid_beta+(1|manifest)+cngeneson+pc_mito+brain_region+Sex	2.803	1663.000
ENSG00000116489	0.000	-0.337	-0.192	-0.483	CAPZA1	0.005	~amyloid_beta+(1|manifest)+cngeneson+pc_mito+brain_region+Sex	2.351	2045.000
ENSG00000170634	0.002	-0.338	-0.146	-0.529	ACYP2	0.016	~amyloid_beta+(1|manifest)+cngeneson+pc_mito+brain_region+Sex	1.686	2851.000
ENSG00000186088	0.005	-0.338	-0.147	-0.529	GSAP	0.025	~amyloid_beta+(1|manifest)+cngeneson+pc_mito+brain_region+Sex	1.454	3220.000
ENSG00000132842	0.017	-0.340	-0.110	-0.571	AP3B1	0.054	~amyloid_beta+(1|manifest)+cngeneson+pc_mito+brain_region+Sex	2.578	1829.000
ENSG00000120278	0.084	-0.341	-0.040	-0.643	PLEKHG1	0.140	~amyloid_beta+(1|manifest)+cngeneson+pc_mito+brain_region+Sex	1.103	3922.000
ENSG00000155265	0.000	-0.343	-0.194	-0.491	GOLGA7B	0.004	~amyloid_beta+(1|manifest)+cngeneson+pc_mito+brain_region+Sex	2.300	2109.000
ENSG00000163728	0.031	-0.343	-0.091	-0.595	TTC14	0.076	~amyloid_beta+(1|manifest)+cngeneson+pc_mito+brain_region+Sex	4.570	871.000
ENSG00000132321	0.222	-0.345	0.113	-0.803	IQCA1	0.277	~amyloid_beta+(1|manifest)+cngeneson+pc_mito+brain_region+Sex	10.100	265.000
ENSG00000155970	0.001	-0.346	-0.197	-0.494	MICU3	0.010	~amyloid_beta+(1|manifest)+cngeneson+pc_mito+brain_region+Sex	2.521	1880.000
ENSG00000169062	0.298	-0.347	0.094	-0.787	UPF3A	0.351	~amyloid_beta+(1|manifest)+cngeneson+pc_mito+brain_region+Sex	10.493	247.000
ENSG00000249242	0.082	-0.347	-0.044	-0.650	[illegible]	0.136	~amyloid_beta+(1|manifest)+cngeneson+pc_mito+brain_region+Sex	0.945	4265.000
ENSG00000171044	0.455	-0.347	0.183	-0.878	XKR6	0.500	~amyloid_beta+(1|manifest)+cngeneson+pc_mito+brain_region+Sex	1.649	2907.000
ENSG00000092978	0.000	-0.347	-0.214	-0.480	GPATCH2	0.004	~amyloid_beta+(1|manifest)+cngeneson+pc_mito+brain_region+Sex	2.666	1756.000
ENSG00000136297	0.534	-0.347	0.273	-0.967	MMD2	0.575	~amyloid_beta+(1|manifest)+cngeneson+pc_mito+brain_region+Sex	6.572	517.000
ENSG00000061987	0.005	-0.348	-0.140	-0.556	MON2	0.026	~amyloid_beta+(1|manifest)+cngeneson+pc_mito+brain_region+Sex	4.797	825.000
ENSG00000160271	0.396	-0.351	0.145	-0.847	RALGDS	0.444	~amyloid_beta+(1|manifest)+cngeneson+pc_mito+brain_region+Sex	8.928	319.000
ENSG00000119927	0.226	-0.354	0.041	-0.748	GPAM	0.281	~amyloid_beta+(1|manifest)+cngeneson+pc_mito+brain_region+Sex	4.831	813.000
ENSG00000183762	0.144	-0.355	-0.013	-0.696	KREMEN1	0.200	~amyloid_beta+(1|manifest)+cngeneson+pc_mito+brain_region+Sex	3.046	1514.000
ENSG00000134874	0.007	-0.355	-0.140	-0.570	DZIP1	0.032	~amyloid_beta+(1|manifest)+cngeneson+pc_mito+brain_region+Sex	2.152	2257.000
ENSG00000080493	0.001	-0.355	-0.159	-0.551	SLC4A4	0.013	~amyloid_beta+(1|manifest)+cngeneson+pc_mito+brain_region+Sex	9.609	288.000
ENSG00000174891	0.005	-0.356	-0.164	-0.547	RSRC1	0.028	~amyloid_beta+(1|manifest)+cngeneson+pc_mito+brain_region+Sex	1.510	3126.000
ENSG00000156076	0.509	-0.357	0.193	-0.907	WIF1	0.552	~amyloid_beta+(1|manifest)+cngeneson+pc_mito+brain_region+Sex	9.617	287.000
ENSG00000146247	0.002	-0.357	-0.142	-0.573	PHIP	0.015	~amyloid_beta+(1|manifest)+cngeneson+pc_mito+brain_region+Sex	3.750	1143.000
ENSG00000182621	0.069	-0.358	0.039	-0.754	PLCB1	0.122	~amyloid_beta+(1|manifest)+cngeneson+pc_mito+brain_region+Sex	5.141	741.000
ENSG00000215018	0.003	-0.358	-0.184	-0.532	COL28A1	0.021	~amyloid_beta+(1|manifest)+cngeneson+pc_mito+brain_region+Sex	1.895	2559.000
ENSG00000102595	0.002	-0.358	-0.171	-0.546	UGGT2	0.015	~amyloid_beta+(1|manifest)+cngeneson+pc_mito+brain_region+Sex	2.542	1866.000
ENSG00000121653	0.001	-0.358	-0.200	-0.516	MAPK8IP1	0.008	~amyloid_beta+(1|manifest)+cngeneson+pc_mito+brain_region+Sex	1.595	2993.000
ENSG00000125462	0.189	-0.358	0.083	-0.800	C1orf61	0.243	~amyloid_beta+(1|manifest)+cngeneson+pc_mito+brain_region+Sex	29.427	23.000
ENSG00000182541	0.400	-0.359	0.186	-0.905	LIMK2	0.448	~amyloid_beta+(1|manifest)+cngeneson+pc_mito+brain_region+Sex	8.119	371.000
ENSG00000132470	0.438	-0.361	0.235	-0.957	ITGB4	0.483	~amyloid_beta+(1|manifest)+cngeneson+pc_mito+brain_region+Sex	11.561	196.000
ENSG00000134508	0.530	-0.362	0.330	-1.053	CABLES1	0.572	~amyloid_beta+(1|manifest)+cngeneson+pc_mito+brain_region+Sex	7.290	441.000
ENSG00000151474	0.467	-0.362	0.214	-0.938	FRMD4A	0.512	~amyloid_beta+(1|manifest)+cngeneson+pc_mito+brain_region+Sex	6.866	483.000
ENSG00000168916	0.152	-0.362	0.048	-0.772	ZNF608	0.208	~amyloid_beta+(1|manifest)+cngeneson+pc_mito+brain_region+Sex	6.998	472.000
ENSG00000149346	0.020	-0.363	-0.119	-0.606	SLX4IP	0.058	~amyloid_beta+(1|manifest)+cngeneson+pc_mito+brain_region+Sex	1.156	3799.000
ENSG00000175575	0.065	-0.363	-0.051	-0.675	PAAF1	0.118	~amyloid_beta+(1|manifest)+cngeneson+pc_mito+brain_region+Sex	4.500	891.000
ENSG00000079931	0.001	-0.363	-0.191	-0.535	MOXD1	0.008	~amyloid_beta+(1|manifest)+cngeneson+pc_mito+brain_region+Sex	0.561	5208.000
ENSG00000146802	0.000	-0.364	-0.244	-0.484	TMEM168	0.002	~amyloid_beta+(1|manifest)+cngeneson+pc_mito+brain_region+Sex	2.111	2297.000
ENSG00000156042	0.228	-0.364	0.040	-0.768	CFAP70	0.282	~amyloid_beta+(1|manifest)+cngeneson+pc_mito+brain_region+Sex	5.725	642.000
ENSG00000150636	0.035	-0.364	-0.090	-0.638	CCDC102B	0.081	~amyloid_beta+(1|manifest)+cngeneson+pc_mito+brain_region+Sex	3.425	1297.000
ENSG00000179397	0.001	-0.365	-0.187	-0.543	CATSPERE	0.011	~amyloid_beta+(1|manifest)+cngeneson+pc_mito+brain_region+Sex	1.783	2713.000
ENSG00000118985	0.327	-0.365	0.185	-0.915	ELL2	0.379	~amyloid_beta+(1|manifest)+cngeneson+pc_mito+brain_region+Sex	13.819	128.000
ENSG00000156110	0.005	-0.365	-0.132	-0.598	ADK	0.027	~amyloid_beta+(1|manifest)+cngeneson+pc_mito+brain_region+Sex	4.671	849.000
ENSG00000154930	0.360	-0.365	0.208	-0.938	ACSS1	0.411	~amyloid_beta+(1|manifest)+cngeneson+pc_mito+brain_region+Sex	12.244	169.000
ENSG00000138738	0.001	-0.366	-0.208	-0.524	PRDM5	0.010	~amyloid_beta+(1|manifest)+cngeneson+pc_mito+brain_region+Sex	1.619	2953.000
ENSG00000066382	0.284	-0.367	0.079	-0.813	MPPED2	0.337	~amyloid_beta+(1|manifest)+cngeneson+pc_mito+brain_region+Sex	6.126	577.000
ENSG00000152348	0.108	-0.367	-0.047	-0.688	ATG10	0.164	~amyloid_beta+(1|manifest)+cngeneson+pc_mito+brain_region+Sex	2.002	2425.000
ENSG00000091138	0.448	-0.367	0.340	-1.075	SLC26A3	0.493	~amyloid_beta+(1|manifest)+cngeneson+pc_mito+brain_region+Sex	70.650	2.000
ENSG00000169519	0.002	-0.368	-0.156	-0.580	METTL15	0.017	~amyloid_beta+(1|manifest)+cngeneson+pc_mito+brain_region+Sex	3.526	1234.000
ENSG00000120594	0.288	-0.369	0.105	-0.844	PLXDC2	0.341	~amyloid_beta+(1|manifest)+cngeneson+pc_mito+brain_region+Sex	1.860	2617.000
ENSG00000133958	0.102	-0.370	-0.040	-0.701	UNC79	0.157	~amyloid_beta+(1|manifest)+cngeneson+pc_mito+brain_region+Sex	5.465	682.000
ENSG00000104613	0.200	-0.372	0.030	-0.774	INTS10	0.254	~amyloid_beta+(1|manifest)+cngeneson+pc_mito+brain_region+Sex	7.945	380.000
ENSG00000046647	0.001	-0.372	-0.203	-0.541	GEMIN8	0.012	~amyloid_beta+(1|manifest)+cngeneson+pc_mito+brain_region+Sex	1.360	3392.000
ENSG00000164776	0.012	-0.372	-0.168	-0.576	PHKG1	0.044	~amyloid_beta+(1|manifest)+cngeneson+pc_mito+brain_region+Sex	3.696	1163.000
ENSG00000114857	0.003	-0.374	-0.121	-0.627	NKTR	0.019	~amyloid_beta+(1|manifest)+cngeneson+pc_mito+brain_region+Sex	6.235	559.000
ENSG00000166435	0.071	-0.375	-0.070	-0.680	XRRA1	0.125	~amyloid_beta+(1|manifest)+cngeneson+pc_mito+brain_region+Sex	3.833	1110.000
ENSG00000140374	0.012	-0.375	-0.158	-0.591	ETFA	0.043	~amyloid_beta+(1|manifest)+cngeneson+pc_mito+brain_region+Sex	2.239	2165.000
ENSG00000014123	0.001	-0.375	-0.199	-0.551	UFL1	0.013	~amyloid_beta+(1|manifest)+cngeneson+pc_mito+brain_region+Sex	1.882	2587.000
ENSG00000131051	0.001	-0.376	-0.195	-0.556	RBM39	0.008	~amyloid_beta+(1|manifest)+cngeneson+pc_mito+brain_region+Sex	4.154	997.000
ENSG00000150893	0.530	-0.376	0.231	-0.984	FREM2	0.572	~amyloid_beta+(1|manifest)+cngeneson+pc_mito+brain_region+Sex	21.408	42.000
ENSG00000164418	0.009	-0.376	-0.139	-0.613	GRIK2	0.037	~amyloid_beta+(1|manifest)+cngeneson+pc_mito+brain_region+Sex	1.933	2518.000
ENSG00000176049	0.003	-0.377	-0.148	-0.605	JAKMIP2	0.019	~amyloid_beta+(1|manifest)+cngeneson+pc_mito+brain_region+Sex	1.315	3471.000
ENSG00000146409	0.110	-0.377	-0.040	-0.715	SLC18B1	0.166	~amyloid_beta+(1|manifest)+cngeneson+pc_mito+brain_region+Sex	5.548	671.000
ENSG00000147454	0.275	-0.377	0.077	-0.831	SLC25A37	0.328	~amyloid_beta+(1|manifest)+cngeneson+pc_mito+brain_region+Sex	7.677	403.000
ENSG00000173681	0.000	-0.382	-0.241	-0.524	BCLAF3	0.004	~amyloid_beta+(1|manifest)+cngeneson+pc_mito+brain_region+Sex	1.297	3511.000
ENSG00000167232	0.045	-0.383	-0.095	-0.672	ZNF91	0.094	~amyloid_beta+(1|manifest)+cngeneson+pc_mito+brain_region+Sex	3.934	1073.000
ENSG00000197818	0.011	-0.384	-0.159	-0.609	SLC9A8	0.042	~amyloid_beta+(1|manifest)+cngeneson+pc_mito+brain_region+Sex	3.583	1214.000
ENSG00000186231	0.333	-0.384	0.126	-0.894	KLHL32	0.385	~amyloid_beta+(1|manifest)+cngeneson+pc_mito+brain_region+Sex	5.453	685.000
ENSG00000197977	0.030	-0.385	-0.126	-0.644	ELOVL2	0.074	~amyloid_beta+(1|manifest)+cngeneson+pc_mito+brain_region+Sex	4.353	932.000
ENSG00000184178	0.003	-0.386	-0.188	-0.583	SCFD2	0.021	~amyloid_beta+(1|manifest)+cngeneson+pc_mito+brain_region+Sex	4.660	856.000
ENSG00000138468	0.011	-0.386	-0.155	-0.616	SENP7	0.041	~amyloid_beta+(1|manifest)+cngeneson+pc_mito+brain_region+Sex	2.525	1874.000
ENSG00000135108	0.006	-0.388	-0.185	-0.591	FBXO21	0.029	~amyloid_beta+(1|manifest)+cngeneson+pc_mito+brain_region+Sex	1.825	2659.000
ENSG00000121988	0.006	-0.389	-0.170	-0.608	ZRANB3	0.029	~amyloid_beta+(1|manifest)+cngeneson+pc_mito+brain_region+Sex	2.605	1807.000
ENSG00000182054	0.180	-0.389	0.004	-0.782	IDH2	0.234	~amyloid_beta+(1|manifest)+cngeneson+pc_mito+brain_region+Sex	6.676	509.000
ENSG00000187240	0.056	-0.389	-0.063	-0.716	DYNC2H1	0.107	~amyloid_beta+(1|manifest)+cngeneson+pc_mito+brain_region+Sex	1.217	3667.000
ENSG00000066468	0.011	-0.394	-0.069	-0.718	FGFR2	0.043	~amyloid_beta+(1|manifest)+cngeneson+pc_mito+brain_region+Sex	6.388	537.000
ENSG00000198520	0.487	-0.395	0.243	-1.033	ARMH1	0.531	~amyloid_beta+(1|manifest)+cngeneson+pc_mito+brain_region+Sex	9.288	308.000
ENSG00000079215	0.000	-0.395	-0.215	-0.576	SLC1A3	0.002	~amyloid_beta+(1|manifest)+cngeneson+pc_mito+brain_region+Sex	18.253	67.000
ENSG00000059588	0.048	-0.396	-0.104	-0.688	TARBP1	0.097	~amyloid_beta+(1|manifest)+cngeneson+pc_mito+brain_region+Sex	3.236	1405.000
ENSG00000146729	0.178	-0.396	0.018	-0.811	NIPSNAP2	0.233	~amyloid_beta+(1|manifest)+cngeneson+pc_mito+brain_region+Sex	7.011	470.000
ENSG00000132199	0.028	-0.398	-0.140	-0.656	ENOSF1	0.071	~amyloid_beta+(1|manifest)+cngeneson+pc_mito+brain_region+Sex	1.401	3321.000
ENSG00000113318	0.004	-0.398	-0.196	-0.600	MSH3	0.024	~amyloid_beta+(1|manifest)+cngeneson+pc_mito+brain_region+Sex	3.005	1540.000
ENSG00000184925	0.038	-0.399	-0.107	-0.691	LCN12	0.085	~amyloid_beta+(1|manifest)+cngeneson+pc_mito+brain_region+Sex	6.762	498.000
ENSG00000127989	0.059	-0.400	-0.041	-0.759	MTERF1	0.110	~amyloid_beta+(1|manifest)+cngeneson+pc_mito+brain_region+Sex	5.429	687.000
ENSG00000186998	0.403	-0.400	0.167	-0.967	EMID1	0.450	~amyloid_beta+(1|manifest)+cngeneson+pc_mito+brain_region+Sex	9.585	290.000
ENSG00000134533	0.073	-0.400	-0.053	-0.747	RERG	0.126	~amyloid_beta+(1|manifest)+cngeneson+pc_mito+brain_region+Sex	3.221	1415.000
ENSG00000144406	0.090	-0.402	-0.016	-0.789	UNC80	0.144	~amyloid_beta+(1|manifest)+cngeneson+pc_mito+brain_region+Sex	1.426	3275.000
ENSG00000182050	0.006	-0.403	-0.113	-0.693	MGAT4C	0.029	~amyloid_beta+(1|manifest)+cngeneson+pc_mito+brain_region+Sex	4.814	819.000
ENSG00000205464	0.162	-0.404	-0.005	-0.802	ATP6AP1L	0.217	~amyloid_beta+(1|manifest)+cngeneson+pc_mito+brain_region+Sex	4.935	790.000
ENSG00000186952	0.078	-0.406	-0.059	-0.754	TMEM232	0.133	~amyloid_beta+(1|manifest)+cngeneson+pc_mito+brain_region+Sex	3.201	1425.000
ENSG00000149089	0.183	-0.407	-0.021	-0.793	APIP	0.237	~amyloid_beta+(1|manifest)+cngeneson+pc_mito+brain_region+Sex	11.459	199.000
ENSG00000169764	0.004	-0.408	-0.178	-0.637	UGP2	0.023	~amyloid_beta+(1|manifest)+cngeneson+pc_mito+brain_region+Sex	2.004	2422.000
ENSG00000157600	0.137	-0.408	0.025	-0.841	TMEM164	0.194	~amyloid_beta+(1|manifest)+cngeneson+pc_mito+brain_region+Sex	11.457	200.000
ENSG00000145431	0.003	-0.409	-0.179	-0.640	PDGFC	0.019	~amyloid_beta+(1|manifest)+cngeneson+pc_mito+brain_region+Sex	3.441	1282.000
ENSG00000184156	0.477	-0.413	0.310	-1.137	KCNQ3	0.522	~amyloid_beta+(1|manifest)+cngeneson+pc_mito+brain_region+Sex	7.027	467.000
ENSG00000198105	0.124	-0.415	0.014	-0.843	ZNF248	0.181	~amyloid_beta+(1|manifest)+cngeneson+pc_mito+brain_region+Sex	6.308	549.000
ENSG00000104177	0.002	-0.416	-0.210	-0.622	MYEF2	0.016	~amyloid_beta+(1|manifest)+cngeneson+pc_mito+brain_region+Sex	3.289	1377.000
ENSG00000186976	0.010	-0.419	-0.184	-0.653	EFCAB6	0.041	~amyloid_beta+(1|manifest)+cngeneson+pc_mito+brain_region+Sex	1.582	3010.000
ENSG00000205581	0.008	-0.419	-0.178	-0.660	HMGN1	0.036	~amyloid_beta+(1|manifest)+cngeneson+pc_mito+brain_region+Sex	5.029	767.000
ENSG00000166436	0.064	-0.420	-0.096	-0.743	TRIM66	0.116	~amyloid_beta+(1|manifest)+cngeneson+pc_mito+brain_region+Sex	6.916	476.000
ENSG00000238083	0.040	-0.421	-0.133	-0.709	LRRC37A2	0.088	~amyloid_beta+(1|manifest)+cngeneson+pc_mito+brain_region+Sex	7.808	389.000
ENSG00000165323	0.038	-0.421	-0.049	-0.793	FAT3	0.085	~amyloid_beta+(1|manifest)+cngeneson+pc_mito+brain_region+Sex	0.927	4313.000
ENSG00000010803	0.012	-0.421	-0.131	-0.712	SCMH1	0.045	~amyloid_beta+(1|manifest)+cngeneson+pc_mito+brain_region+Sex	7.924	382.000
ENSG00000151834	0.000	-0.423	-0.237	-0.609	GABRA2	0.001	~amyloid_beta+(1|manifest)+cngeneson+pc_mito+brain_region+Sex	5.204	727.000
ENSG00000140022	0.000	-0.424	-0.206	-0.641	STON2	0.005	~amyloid_beta+(1|manifest)+cngeneson+pc_mito+brain_region+Sex	14.017	123.000
ENSG00000152495	0.028	-0.425	-0.153	-0.698	CAMK4	0.072	~amyloid_beta+(1|manifest)+cngeneson+pc_mito+brain_region+Sex	6.596	516.000
ENSG00000099256	0.010	-0.430	-0.173	-0.687	PRTFDC1	0.039	~amyloid_beta+(1|manifest)+cngeneson+pc_mito+brain_region+Sex	2.140	2273.000
ENSG00000166262	0.021	-0.431	-0.173	-0.689	FAM227B	0.060	~amyloid_beta+(1|manifest)+cngeneson+pc_mito+brain_region+Sex	3.248	1398.000
ENSG00000117616	0.056	-0.433	-0.047	-0.819	RSRP1	0.107	~amyloid_beta+(1|manifest)+cngeneson+pc_mito+brain_region+Sex	10.639	237.000
ENSG00000125772	0.013	-0.433	-0.193	-0.673	GPCPD1	0.046	~amyloid_beta+(1|manifest)+cngeneson+pc_mito+brain_region+Sex	2.777	1685.000
ENSG00000147509	0.146	-0.434	0.046	-0.913	RGS20	0.202	~amyloid_beta+(1|manifest)+cngeneson+pc_mito+brain_region+Sex	12.758	157.000
ENSG00000205213	0.001	-0.437	-0.179	-0.695	LGR4	0.011	~amyloid_beta+(1|manifest)+cngeneson+pc_mito+brain_region+Sex	6.086	582.000
ENSG00000198919	0.012	-0.438	-0.178	-0.697	DZIP3	0.044	~amyloid_beta+(1|manifest)+cngeneson+pc_mito+brain_region+Sex	1.846	2634.000
ENSG00000130779	0.030	-0.440	-0.089	-0.792	CLIP1	0.075	~amyloid_beta+(1|manifest)+cngeneson+pc_mito+brain_region+Sex	10.307	256.000
ENSG00000283782	0.160	-0.441	-0.043	-0.838	[illegible]	0.215	~amyloid_beta+(1|manifest)+cngeneson+pc_mito+brain_region+Sex	4.550	878.000
ENSG00000129636	0.000	-0.441	-0.271	-0.611	ITFG1	0.000	~amyloid_beta+(1|manifest)+cngeneson+pc_mito+brain_region+Sex	3.239	1402.000
ENSG00000146416	0.007	-0.441	-0.176	-0.707	AIG1	0.032	~amyloid_beta+(1|manifest)+cngeneson+pc_mito+brain_region+Sex	1.055	4012.000
ENSG00000100941	0.041	-0.441	-0.147	-0.736	PNN	0.088	~amyloid_beta+(1|manifest)+cngeneson+pc_mito+brain_region+Sex	5.223	723.000
ENSG00000040731	0.155	-0.442	-0.004	-0.880	CDH10	0.210	~amyloid_beta+(1|manifest)+cngeneson+pc_mito+brain_region+Sex	5.637	658.000
ENSG00000121964	0.001	-0.444	-0.236	-0.652	GTDC1	0.014	~amyloid_beta+(1|manifest)+cngeneson+pc_mito+brain_region+Sex	1.036	4059.000
ENSG00000112893	0.064	-0.448	-0.055	-0.840	MAN2A1	0.116	~amyloid_beta+(1|manifest)+cngeneson+pc_mito+brain_region+Sex	12.118	173.000
ENSG00000008513	0.013	-0.449	-0.173	-0.725	ST3GAL1	0.046	~amyloid_beta+(1|manifest)+cngeneson+pc_mito+brain_region+Sex	3.512	1244.000
ENSG00000236383	0.226	-0.453	0.013	-0.918	CCDC200	0.281	~amyloid_beta+(1|manifest)+cngeneson+pc_mito+brain_region+Sex	13.938	125.000
ENSG00000151575	0.021	-0.454	-0.180	-0.727	TEX9	0.062	~amyloid_beta+(1|manifest)+cngeneson+pc_mito+brain_region+Sex	1.154	3806.000
ENSG00000165102	0.068	-0.456	-0.108	-0.803	HGSNAT	0.121	~amyloid_beta+(1|manifest)+cngeneson+pc_mito+brain_region+Sex	5.514	678.000
ENSG00000119707	0.004	-0.456	-0.204	-0.707	RBM25	0.024	~amyloid_beta+(1|manifest)+cngeneson+pc_mito+brain_region+Sex	3.975	1048.000
ENSG00000116127	0.011	-0.458	-0.157	-0.759	ALMS1	0.042	~amyloid_beta+(1|manifest)+cngeneson+pc_mito+brain_region+Sex	4.768	829.000
ENSG00000196660	0.065	-0.460	-0.111	-0.810	SLC30A10	0.118	~amyloid_beta+(1|manifest)+cngeneson+pc_mito+brain_region+Sex	4.688	843.000
ENSG00000116560	0.000	-0.462	-0.305	-0.619	SFPQ	0.000	~amyloid_beta+(1|manifest)+cngeneson+pc_mito+brain_region+Sex	4.048	1025.000
ENSG00000048540	0.139	-0.464	0.103	-1.031	LMO3	0.196	~amyloid_beta+(1|manifest)+cngeneson+pc_mito+brain_region+Sex	15.330	97.000
ENSG00000172340	0.008	-0.468	-0.193	-0.742	SUCLG2	0.036	~amyloid_beta+(1|manifest)+cngeneson+pc_mito+brain_region+Sex	3.918	1080.000
ENSG00000136895	0.178	-0.469	0.032	-0.969	GARNL3	0.233	~amyloid_beta+(1|manifest)+cngeneson+pc_mito+brain_region+Sex	7.010	471.000
ENSG00000121671	0.024	-0.471	-0.176	-0.765	CRY2	0.066	~amyloid_beta+(1|manifest)+cngeneson+pc_mito+brain_region+Sex	3.842	1108.000
ENSG00000172270	0.068	-0.474	-0.078	-0.870	BSG	0.122	~amyloid_beta+(1|manifest)+cngeneson+pc_mito+brain_region+Sex	4.336	937.000
ENSG00000164929	0.108	-0.475	0.030	-0.981	BAALC	0.164	~amyloid_beta+(1|manifest)+cngeneson+pc_mito+brain_region+Sex	13.560	135.000
ENSG00000165730	0.000	-0.478	-0.225	-0.732	STOX1	0.005	~amyloid_beta+(1|manifest)+cngeneson+pc_mito+brain_region+Sex	1.048	4029.000
ENSG00000163618	0.360	-0.479	0.188	-1.145	CADPS	0.411	~amyloid_beta+(1|manifest)+cngeneson+pc_mito+brain_region+Sex	34.194	12.000
ENSG00000270099	0.001	-0.480	-0.236	-0.723	[illegible]	0.008	~amyloid_beta+(1|manifest)+cngeneson+pc_mito+brain_region+Sex	3.524	1238.000
ENSG00000168952	0.032	-0.481	-0.122	-0.839	STXBP6	0.077	~amyloid_beta+(1|manifest)+cngeneson+pc_mito+brain_region+Sex	10.194	262.000
ENSG00000119787	0.006	-0.483	-0.224	-0.742	ATL2	0.030	~amyloid_beta+(1|manifest)+cngeneson+pc_mito+brain_region+Sex	4.424	908.000
ENSG00000118507	0.000	-0.484	-0.281	-0.686	AKAP7	0.003	~amyloid_beta+(1|manifest)+cngeneson+pc_mito+brain_region+Sex	2.213	2188.000
ENSG00000176771	0.210	-0.493	0.143	-1.129	NCKAP5	0.265	~amyloid_beta+(1|manifest)+cngeneson+pc_mito+brain_region+Sex	18.563	62.000
ENSG00000108854	0.003	-0.498	-0.219	-0.777	SMURF2	0.021	~amyloid_beta+(1|manifest)+cngeneson+pc_mito+brain_region+Sex	6.251	556.000
ENSG00000112425	0.004	-0.500	-0.229	-0.772	EPM2A	0.023	~amyloid_beta+(1|manifest)+cngeneson+pc_mito+brain_region+Sex	3.419	1301.000
ENSG00000151320	0.247	-0.502	0.099	-1.103	AKAP6	0.300	~amyloid_beta+(1|manifest)+cngeneson+pc_mito+brain_region+Sex	4.400	916.000
ENSG00000112739	0.047	-0.503	-0.131	-0.876	PRPF4B	0.096	~amyloid_beta+(1|manifest)+cngeneson+pc_mito+brain_region+Sex	7.337	432.000
ENSG00000166839	0.263	-0.504	0.226	-1.234	ANKDD1A	0.317	~amyloid_beta+(1|manifest)+cngeneson+pc_mito+brain_region+Sex	20.647	45.000
ENSG00000182132	0.031	-0.506	-0.158	-0.854	KCNIP1	0.076	~amyloid_beta+(1|manifest)+cngeneson+pc_mito+brain_region+Sex	14.395	119.000
ENSG00000164989	0.010	-0.507	-0.208	-0.805	CCDC171	0.039	~amyloid_beta+(1|manifest)+cngeneson+pc_mito+brain_region+Sex	2.796	1670.000
ENSG00000144642	0.014	-0.508	-0.146	-0.870	RBMS3	0.048	~amyloid_beta+(1|manifest)+cngeneson+pc_mito+brain_region+Sex	0.444	5470.000
ENSG00000159140	0.014	-0.508	-0.119	-0.897	SON	0.048	~amyloid_beta+(1|manifest)+cngeneson+pc_mito+brain_region+Sex	11.483	198.000
ENSG00000178904	0.038	-0.508	-0.122	-0.894	DPY19L3	0.085	~amyloid_beta+(1|manifest)+cngeneson+pc_mito+brain_region+Sex	9.130	313.000
ENSG00000196776	0.054	-0.510	-0.114	-0.906	CD47	0.104	~amyloid_beta+(1|manifest)+cngeneson+pc_mito+brain_region+Sex	5.418	692.000
ENSG00000184903	0.001	-0.511	-0.218	-0.804	IMMP2L	0.009	~amyloid_beta+(1|manifest)+cngeneson+pc_mito+brain_region+Sex	5.714	644.000
ENSG00000172817	0.009	-0.513	-0.228	-0.798	CYP7B1	0.038	~amyloid_beta+(1|manifest)+cngeneson+pc_mito+brain_region+Sex	2.250	2156.000
ENSG00000178425	0.001	-0.515	-0.291	-0.739	NT5DC1	0.010	~amyloid_beta+(1|manifest)+cngeneson+pc_mito+brain_region+Sex	1.724	2791.000
ENSG00000144935	0.007	-0.515	-0.169	-0.862	TRPC1	0.033	~amyloid_beta+(1|manifest)+cngeneson+pc_mito+brain_region+Sex	4.750	832.000
ENSG00000104381	0.031	-0.517	-0.173	-0.862	GDAP1	0.075	~amyloid_beta+(1|manifest)+cngeneson+pc_mito+brain_region+Sex	2.794	1674.000
ENSG00000152217	0.000	-0.521	-0.355	-0.687	SETBP1	0.001	~amyloid_beta+(1|manifest)+cngeneson+pc_mito+brain_region+Sex	0.815	4585.000
ENSG00000138185	0.000	-0.523	-0.360	-0.686	ENTPD1	0.000	~amyloid_beta+(1|manifest)+cngeneson+pc_mito+brain_region+Sex	4.293	950.000
ENSG00000039319	0.000	-0.523	-0.280	-0.767	ZFYVE16	0.005	~amyloid_beta+(1|manifest)+cngeneson+pc_mito+brain_region+Sex	5.637	657.000
ENSG00000132122	0.025	-0.524	-0.123	-0.924	SPATA6	0.068	~amyloid_beta+(1|manifest)+cngeneson+pc_mito+brain_region+Sex	8.328	357.000
ENSG00000205336	0.193	-0.524	0.063	-1.110	ADGRG1	0.247	~amyloid_beta+(1|manifest)+cngeneson+pc_mito+brain_region+Sex	8.136	367.000
ENSG00000068078	0.185	-0.525	0.129	-1.180	FGFR3	0.240	~amyloid_beta+(1|manifest)+cngeneson+pc_mito+brain_region+Sex	21.692	41.000
ENSG00000176809	0.030	-0.527	-0.143	-0.912	LRRC37A3	0.075	~amyloid_beta+(1|manifest)+cngeneson+pc_mito+brain_region+Sex	12.320	166.000
ENSG00000204740	0.000	-0.536	-0.339	-0.734	MALRD1	0.004	~amyloid_beta+(1|manifest)+cngeneson+pc_mito+brain_region+Sex	0.402	5562.000
ENSG00000130413	0.006	-0.541	-0.191	-0.891	STK33	0.029	~amyloid_beta+(1|manifest)+cngeneson+pc_mito+brain_region+Sex	4.884	803.000
ENSG00000172260	0.010	-0.541	-0.220	-0.862	NEGR1	0.039	~amyloid_beta+(1|manifest)+cngeneson+pc_mito+brain_region+Sex	3.439	1283.000
ENSG00000083544	0.000	-0.545	-0.323	-0.766	TDRD3	0.000	~amyloid_beta+(1|manifest)+cngeneson+pc_mito+brain_region+Sex	6.312	546.000
ENSG00000179915	0.005	-0.545	-0.143	-0.946	NRXN1	0.028	~amyloid_beta+(1|manifest)+cngeneson+pc_mito+brain_region+Sex	13.089	147.000
ENSG00000149090	0.004	-0.545	-0.152	-0.938	PAMR1	0.025	~amyloid_beta+(1|manifest)+cngeneson+pc_mito+brain_region+Sex	11.190	212.000
ENSG00000148948	0.009	-0.546	-0.173	-0.919	LRRC4C	0.037	~amyloid_beta+(1|manifest)+cngeneson+pc_mito+brain_region+Sex	1.377	3362.000
ENSG00000196440	0.007	-0.550	-0.238	-0.862	ARMCX4	0.033	~amyloid_beta+(1|manifest)+cngeneson+pc_mito+brain_region+Sex	4.269	957.000
ENSG00000169251	0.010	-0.551	-0.219	-0.883	NMD3	0.039	~amyloid_beta+(1|manifest)+cngeneson+pc_mito+brain_region+Sex	2.856	1630.000
ENSG00000158019	0.000	-0.551	-0.292	-0.810	BABAM2	0.001	~amyloid_beta+(1|manifest)+cngeneson+pc_mito+brain_region+Sex	3.094	1487.000
ENSG00000172572	0.083	-0.554	-0.032	-1.076	PDE3A	0.138	~amyloid_beta+(1|manifest)+cngeneson+pc_mito+brain_region+Sex	7.199	452.000
ENSG00000188649	0.002	-0.555	-0.245	-0.864	CC2D2B	0.017	~amyloid_beta+(1|manifest)+cngeneson+pc_mito+brain_region+Sex	3.468	1265.000
ENSG00000226124	0.011	-0.555	-0.251	-0.859	FTCDNL1	0.042	~amyloid_beta+(1|manifest)+cngeneson+pc_mito+brain_region+Sex	2.542	1867.000
ENSG00000271447	0.045	-0.557	-0.176	-0.938	MMP28	0.094	~amyloid_beta+(1|manifest)+cngeneson+pc_mito+brain_region+Sex	4.642	857.000
ENSG00000181234	0.238	-0.560	0.065	-1.184	[illegible]	0.291	~amyloid_beta+(1|manifest)+cngeneson+pc_mito+brain_region+Sex	3.928	1078.000
ENSG00000135749	0.018	-0.563	-0.218	-0.909	PCNX2	0.055	~amyloid_beta+(1|manifest)+cngeneson+pc_mito+brain_region+Sex	1.036	4058.000
ENSG00000104427	0.047	-0.567	-0.150	-0.983	ZC2HC1A	0.096	~amyloid_beta+(1|manifest)+cngeneson+pc_mito+brain_region+Sex	1.465	3196.000
ENSG00000134201	0.002	-0.579	-0.301	-0.858	GSTM5	0.017	~amyloid_beta+(1|manifest)+cngeneson+pc_mito+brain_region+Sex	13.590	133.000
ENSG00000144455	0.001	-0.591	-0.266	-0.916	SUMF1	0.009	~amyloid_beta+(1|manifest)+cngeneson+pc_mito+brain_region+Sex	4.599	864.000
ENSG00000188803	0.036	-0.592	-0.204	-0.980	SHISA6	0.082	~amyloid_beta+(1|manifest)+cngeneson+pc_mito+brain_region+Sex	5.782	628.000
ENSG00000124406	0.006	-0.602	-0.229	-0.976	ATP8A1	0.030	~amyloid_beta+(1|manifest)+cngeneson+pc_mito+brain_region+Sex	4.986	780.000
ENSG00000216937	0.003	-0.603	-0.284	-0.922	CCDC7	0.019	~amyloid_beta+(1|manifest)+cngeneson+pc_mito+brain_region+Sex	2.990	1548.000
ENSG00000153930	0.023	-0.604	-0.201	-1.007	ANKFN1	0.064	~amyloid_beta+(1|manifest)+cngeneson+pc_mito+brain_region+Sex	2.344	2055.000
ENSG00000072952	0.035	-0.606	-0.075	-1.138	MRVI1	0.081	~amyloid_beta+(1|manifest)+cngeneson+pc_mito+brain_region+Sex	11.751	189.000
ENSG00000157103	0.101	-0.610	-0.005	-1.215	SLC6A1	0.156	~amyloid_beta+(1|manifest)+cngeneson+pc_mito+brain_region+Sex	11.982	177.000
ENSG00000144868	0.017	-0.610	-0.169	-1.051	TMEM108	0.054	~amyloid_beta+(1|manifest)+cngeneson+pc_mito+brain_region+Sex	4.440	906.000
ENSG00000132164	0.145	-0.610	-0.048	-1.173	SLC6A11	0.201	~amyloid_beta+(1|manifest)+cngeneson+pc_mito+brain_region+Sex	11.754	188.000
ENSG00000134376	0.000	-0.623	-0.321	-0.924	CRB1	0.000	~amyloid_beta+(1|manifest)+cngeneson+pc_mito+brain_region+Sex	8.209	361.000
ENSG00000197410	0.019	-0.635	-0.236	-1.033	DCHS2	0.057	~amyloid_beta+(1|manifest)+cngeneson+pc_mito+brain_region+Sex	3.115	1474.000
ENSG00000198822	0.059	-0.636	-0.103	-1.168	GRM3	0.111	~amyloid_beta+(1|manifest)+cngeneson+pc_mito+brain_region+Sex	3.792	1128.000
ENSG00000099250	0.140	-0.648	-0.044	-1.252	NRP1	0.196	~amyloid_beta+(1|manifest)+cngeneson+pc_mito+brain_region+Sex	20.234	52.000
ENSG00000165475	0.004	-0.649	-0.296	-1.002	CRYL1	0.025	~amyloid_beta+(1|manifest)+cngeneson+pc_mito+brain_region+Sex	4.238	970.000
ENSG00000032742	0.002	-0.649	-0.337	-0.961	IFT88	0.015	~amyloid_beta+(1|manifest)+cngeneson+pc_mito+brain_region+Sex	2.396	1989.000
ENSG00000007001	0.058	-0.656	-0.158	-1.155	UPP2	0.109	~amyloid_beta+(1|manifest)+cngeneson+pc_mito+brain_region+Sex	6.476	529.000
ENSG00000150477	0.000	-0.657	-0.319	-0.995	KIAA1328	0.004	~amyloid_beta+(1|manifest)+cngeneson+pc_mito+brain_region+Sex	3.897	1091.000
ENSG00000204442	0.013	-0.663	-0.067	-1.260	FAM155A	0.047	~amyloid_beta+(1|manifest)+cngeneson+pc_mito+brain_region+Sex	15.175	101.000
ENSG00000158296	0.028	-0.671	-0.168	-1.175	SLC13A3	0.071	~amyloid_beta+(1|manifest)+cngeneson+pc_mito+brain_region+Sex	7.180	454.000
ENSG00000177853	0.000	-0.673	-0.355	-0.991	ZNF518A	0.004	~amyloid_beta+(1|manifest)+cngeneson+pc_mito+brain_region+Sex	3.096	1486.000
ENSG00000172456	0.000	-0.687	-0.406	-0.969	FGGY	0.001	~amyloid_beta+(1|manifest)+cngeneson+pc_mito+brain_region+Sex	1.691	2842.000
ENSG00000063601	0.004	-0.702	-0.313	-1.092	MTMR1	0.022	~amyloid_beta+(1|manifest)+cngeneson+pc_mito+brain_region+Sex	5.869	613.000
ENSG00000115380	0.001	-0.707	-0.235	-1.178	EFEMP1	0.012	~amyloid_beta+(1|manifest)+cngeneson+pc_mito+brain_region+Sex	1.295	3514.000
ENSG00000137033	0.000	-0.708	-0.436	-0.980	IL33	0.005	~amyloid_beta+(1|manifest)+cngeneson+pc_mito+brain_region+Sex	3.669	1177.000
ENSG00000138639	0.011	-0.720	-0.132	-1.309	ARHGAP24	0.042	~amyloid_beta+(1|manifest)+cngeneson+pc_mito+brain_region+Sex	5.705	649.000
ENSG00000198797	0.011	-0.730	-0.282	-1.179	BRINP2	0.043	~amyloid_beta+(1|manifest)+cngeneson+pc_mito+brain_region+Sex	5.231	719.000
ENSG00000100033	0.190	-0.733	0.354	-1.821	PRODH	0.244	~amyloid_beta+(1|manifest)+cngeneson+pc_mito+brain_region+Sex	20.834	43.000
ENSG00000215252	0.073	-0.752	-0.192	-1.311	GOLGA8B	0.126	~amyloid_beta+(1|manifest)+cngeneson+pc_mito+brain_region+Sex	13.458	139.000
ENSG00000177694	0.001	-0.761	-0.263	-1.259	NAALADL2	0.008	~amyloid_beta+(1|manifest)+cngeneson+pc_mito+brain_region+Sex	10.952	224.000
ENSG00000008311	0.001	-0.782	-0.327	-1.237	AASS	0.009	~amyloid_beta+(1|manifest)+cngeneson+pc_mito+brain_region+Sex	8.404	352.000
ENSG00000165092	0.020	-0.783	-0.146	-1.420	ALDH1A1	0.058	~amyloid_beta+(1|manifest)+cngeneson+pc_mito+brain_region+Sex	12.743	159.000
ENSG00000105894	0.003	-0.823	-0.326	-1.320	PTN	0.019	~amyloid_beta+(1|manifest)+cngeneson+pc_mito+brain_region+Sex	7.514	412.000
ENSG00000139155	0.003	-0.830	-0.301	-1.359	SLCO1C1	0.019	~amyloid_beta+(1|manifest)+cngeneson+pc_mito+brain_region+Sex	11.795	187.000
ENSG00000175497	0.101	-0.847	0.064	-1.757	DPP10	0.156	~amyloid_beta+(1|manifest)+cngeneson+pc_mito+brain_region+Sex	12.193	171.000
ENSG00000120658	0.000	-0.897	-0.549	-1.245	ENOX1	0.000	~amyloid_beta+(1|manifest)+cngeneson+pc_mito+brain_region+Sex	4.457	902.000
ENSG00000145934	0.046	-0.900	-0.025	-1.775	TENM2	0.095	~amyloid_beta+(1|manifest)+cngeneson+pc_mito+brain_region+Sex	20.445	48.000
ENSG00000179796	0.000	-0.902	-0.430	-1.375	LRRC3B	0.004	~amyloid_beta+(1|manifest)+cngeneson+pc_mito+brain_region+Sex	5.106	753.000
ENSG00000186094	0.008	-0.954	-0.267	-1.642	AGBL4	0.035	~amyloid_beta+(1|manifest)+cngeneson+pc_mito+brain_region+Sex	5.422	688.000
ENSG00000120251	0.008	-0.972	-0.327	-1.617	GRIA2	0.036	~amyloid_beta+(1|manifest)+cngeneson+pc_mito+brain_region+Sex	13.566	134.000
ENSG00000265972	0.000	-1.021	-0.659	-1.383	TXNIP	0.004	~amyloid_beta+(1|manifest)+cngeneson+pc_mito+brain_region+Sex	0.953	4245.000
ENSG00000166432	0.001	-1.138	-0.481	-1.795	ZMAT1	0.011	~amyloid_beta+(1|manifest)+cngeneson+pc_mito+brain_region+Sex	6.903	477.000
ENSG00000167772	0.016	-1.173	-0.246	-2.100	ANGPTL4	0.053	~amyloid_beta+(1|manifest)+cngeneson+pc_mito+brain_region+Sex	14.445	116.000
ENSG00000099622	0.034	-1.177	-0.287	-2.067	CIRBP	0.080	~amyloid_beta+(1|manifest)+cngeneson+pc_mito+brain_region+Sex	22.761	36.000
ENSG00000117525	0.016	-1.214	-0.212	-2.216	F3	0.053	~amyloid_beta+(1|manifest)+cngeneson+pc_mito+brain_region+Sex	36.189	10.000
ENSG00000134146	0.000	-1.253	-0.717	-1.789	DPH6	0.002	~amyloid_beta+(1|manifest)+cngeneson+pc_mito+brain_region+Sex	7.286	444.000
ENSG00000228696	0.000	-2.469	-1.150	-3.788	ARL17B	0.002	~amyloid_beta+(1|manifest)+cngeneson+pc_mito+brain_region+Sex	51.289	4.000

Supplementary Table 7_Microglia and PVMs DGE
Contents

Sheet name	Description
Microglia_pTau	DGE analysis in Microglia using MAST and p_tau as contrast
PVM_pTau	DGE analysis in PVM using MAST and p_tau as contrast
Microglia_beta_amyloi	DGE analysis in Microglia using MAST and beta_amyloid as contrast
PVM_beta_amyloid	DGE analysis in PVM using MAST and beta_amyloid as contrast

ci.hi/lo: higher and lower confidence intervals of the logFC value, respectively

Supplementary Table 7_Microglia and PVMs DGE
Microglia pTau

DGE analysis in Total Microglia using MAST and p_tau as contrast

ensembl_gene_id	pval	logFC	ci.hi	ci.lo	gene	padj	model	variance	variance_rank
ENSG00000188641	0.010	1.544	2.434	0.654	DPYD	0.092	~p_tau+(1|manifest)+cngeneson+pc_mito+brain_region+Sex	9.581	116.000
ENSG00000133121	0.000	1.241	1.836	0.646	STARD13	0.006	~p_tau+(1|manifest)+cngeneson+pc_mito+brain_region+Sex	4.920	377.000
ENSG00000120694	0.054	1.125	2.302	-0.052	HSPH1	0.222	~p_tau+(1|manifest)+cngeneson+pc_mito+brain_region+Sex	32.066	5.000
ENSG00000130203	0.001	1.117	1.914	0.320	APOE	0.029	~p_tau+(1|manifest)+cngeneson+pc_mito+brain_region+Sex	14.739	47.000
ENSG00000080824	0.023	1.096	1.956	0.236	HSP90AA1	0.141	~p_tau+(1|manifest)+cngeneson+pc_mito+brain_region+Sex	21.166	22.000
ENSG00000135540	0.006	0.900	1.528	0.271	NHSL1	0.072	~p_tau+(1|manifest)+cngeneson+pc_mito+brain_region+Sex	4.216	488.000
ENSG00000145703	0.000	0.888	1.465	0.312	IQGAP2	0.014	~p_tau+(1|manifest)+cngeneson+pc_mito+brain_region+Sex	3.324	678.000
ENSG00000188906	0.001	0.887	1.442	0.331	LRRK2	0.037	~p_tau+(1|manifest)+cngeneson+pc_mito+brain_region+Sex	3.795	566.000
ENSG00000168010	0.000	0.885	1.242	0.528	ATG16L2	0.000	~p_tau+(1|manifest)+cngeneson+pc_mito+brain_region+Sex	2.810	838.000
ENSG00000106211	0.002	0.884	1.681	0.088	HSPB1	0.048	~p_tau+(1|manifest)+cngeneson+pc_mito+brain_region+Sex	11.752	81.000
ENSG00000127483	0.000	0.859	1.155	0.564	HP1BP3	0.000	~p_tau+(1|manifest)+cngeneson+pc_mito+brain_region+Sex	3.374	662.000
ENSG00000122574	0.008	0.846	1.660	0.031	WIPF3	0.080	~p_tau+(1|manifest)+cngeneson+pc_mito+brain_region+Sex	5.980	283.000
ENSG00000144724	0.000	0.831	1.433	0.228	PTPRG	0.020	~p_tau+(1|manifest)+cngeneson+pc_mito+brain_region+Sex	1.697	1456.000
ENSG00000177570	0.003	0.821	1.273	0.369	SAMD12	0.049	~p_tau+(1|manifest)+cngeneson+pc_mito+brain_region+Sex	3.681	600.000
ENSG00000007237	0.008	0.821	1.352	0.290	GAS7	0.081	~p_tau+(1|manifest)+cngeneson+pc_mito+brain_region+Sex	4.409	454.000
ENSG00000158201	0.006	0.781	1.405	0.156	ABHD3	0.072	~p_tau+(1|manifest)+cngeneson+pc_mito+brain_region+Sex	7.759	172.000
ENSG00000157483	0.000	0.760	1.061	0.459	MYO1E	0.003	~p_tau+(1|manifest)+cngeneson+pc_mito+brain_region+Sex	1.524	1632.000
ENSG00000115896	0.002	0.747	1.268	0.227	PLCL1	0.039	~p_tau+(1|manifest)+cngeneson+pc_mito+brain_region+Sex	3.991	522.000
ENSG00000104763	0.000	0.745	1.157	0.333	ASAH1	0.016	~p_tau+(1|manifest)+cngeneson+pc_mito+brain_region+Sex	2.329	1055.000
ENSG00000038945	0.068	0.739	1.519	-0.041	MSR1	0.246	~p_tau+(1|manifest)+cngeneson+pc_mito+brain_region+Sex	24.765	13.000
ENSG00000110172	0.047	0.728	1.593	-0.137	CHORDC1	0.202	~p_tau+(1|manifest)+cngeneson+pc_mito+brain_region+Sex	22.248	19.000
ENSG00000170921	0.000	0.707	0.988	0.427	TANC2	0.003	~p_tau+(1|manifest)+cngeneson+pc_mito+brain_region+Sex	2.220	1122.000
ENSG00000185829	0.000	0.706	1.113	0.299	ARL17A	0.003	~p_tau+(1|manifest)+cngeneson+pc_mito+brain_region+Sex	0.617	3472.000
ENSG00000145431	0.005	0.699	1.236	0.163	PDGFC	0.062	~p_tau+(1|manifest)+cngeneson+pc_mito+brain_region+Sex	5.734	301.000
ENSG00000114796	0.006	0.684	1.289	0.079	KLHL24	0.072	~p_tau+(1|manifest)+cngeneson+pc_mito+brain_region+Sex	7.154	203.000
ENSG00000105993	0.008	0.681	1.233	0.129	DNAJB6	0.082	~p_tau+(1|manifest)+cngeneson+pc_mito+brain_region+Sex	6.333	250.000
ENSG00000187098	0.002	0.669	1.017	0.320	MITF	0.038	~p_tau+(1|manifest)+cngeneson+pc_mito+brain_region+Sex	3.451	650.000
ENSG00000186047	0.023	0.647	1.279	0.015	DLEU7	0.140	~p_tau+(1|manifest)+cngeneson+pc_mito+brain_region+Sex	7.601	181.000
ENSG00000092531	0.001	0.644	1.117	0.172	SNAP23	0.031	~p_tau+(1|manifest)+cngeneson+pc_mito+brain_region+Sex	5.896	289.000
ENSG00000109458	0.052	0.644	1.277	0.011	GAB1	0.217	~p_tau+(1|manifest)+cngeneson+pc_mito+brain_region+Sex	8.895	131.000
ENSG00000197548	0.000	0.635	0.822	0.447	ATG7	0.000	~p_tau+(1|manifest)+cngeneson+pc_mito+brain_region+Sex	2.070	1204.000
ENSG00000085514	0.000	0.630	1.029	0.231	PILRA	0.015	~p_tau+(1|manifest)+cngeneson+pc_mito+brain_region+Sex	2.856	814.000
ENSG00000196141	0.115	0.629	1.340	-0.082	SPATS2L	0.328	~p_tau+(1|manifest)+cngeneson+pc_mito+brain_region+Sex	11.757	80.000
ENSG00000185950	0.027	0.627	1.208	0.045	IRS2	0.151	~p_tau+(1|manifest)+cngeneson+pc_mito+brain_region+Sex	4.402	456.000
ENSG00000170396	0.037	0.625	1.268	-0.019	ZNF804A	0.180	~p_tau+(1|manifest)+cngeneson+pc_mito+brain_region+Sex	2.790	846.000
ENSG00000166295	0.001	0.615	1.029	0.201	ANAPC16	0.027	~p_tau+(1|manifest)+cngeneson+pc_mito+brain_region+Sex	3.611	619.000
ENSG00000139793	0.052	0.613	1.230	-0.004	MBNL2	0.217	~p_tau+(1|manifest)+cngeneson+pc_mito+brain_region+Sex	10.495	97.000
ENSG00000156113	0.031	0.611	1.090	0.131	KCNMA1	0.162	~p_tau+(1|manifest)+cngeneson+pc_mito+brain_region+Sex	6.467	242.000
ENSG00000152518	0.028	0.609	1.088	0.130	ZFP36L2	0.153	~p_tau+(1|manifest)+cngeneson+pc_mito+brain_region+Sex	5.527	315.000
ENSG00000106066	0.105	0.599	1.270	-0.071	CPVL	0.311	~p_tau+(1|manifest)+cngeneson+pc_mito+brain_region+Sex	6.174	266.000
ENSG00000110958	0.094	0.592	1.304	-0.120	PTGES3	0.291	~p_tau+(1|manifest)+cngeneson+pc_mito+brain_region+Sex	14.789	46.000
ENSG00000136235	0.000	0.592	1.003	0.181	GPNMB	0.005	~p_tau+(1|manifest)+cngeneson+pc_mito+brain_region+Sex	2.352	1043.000
ENSG00000096384	0.039	0.587	1.229	-0.054	HSP90AB1	0.185	~p_tau+(1|manifest)+cngeneson+pc_mito+brain_region+Sex	8.326	147.000
ENSG00000086061	0.004	0.583	1.134	0.032	DNAJA1	0.060	~p_tau+(1|manifest)+cngeneson+pc_mito+brain_region+Sex	5.733	302.000
ENSG00000152689	0.004	0.577	1.029	0.126	RASGRP3	0.058	~p_tau+(1|manifest)+cngeneson+pc_mito+brain_region+Sex	0.464	4083.000
ENSG00000106617	0.015	0.571	0.977	0.165	PRKAG2	0.110	~p_tau+(1|manifest)+cngeneson+pc_mito+brain_region+Sex	3.890	544.000
ENSG00000137575	0.007	0.570	1.046	0.093	SDCBP	0.075	~p_tau+(1|manifest)+cngeneson+pc_mito+brain_region+Sex	3.547	629.000
ENSG00000164463	0.001	0.569	1.008	0.130	CREBRF	0.028	~p_tau+(1|manifest)+cngeneson+pc_mito+brain_region+Sex	4.216	489.000
ENSG00000140350	0.000	0.567	0.801	0.332	ANP32A	0.002	~p_tau+(1|manifest)+cngeneson+pc_mito+brain_region+Sex	0.600	3534.000
ENSG00000186635	0.000	0.566	0.846	0.286	ARAP1	0.016	~p_tau+(1|manifest)+cngeneson+pc_mito+brain_region+Sex	1.616	1535.000
ENSG00000147459	0.080	0.564	1.164	-0.036	DOCK5	0.269	~p_tau+(1|manifest)+cngeneson+pc_mito+brain_region+Sex	6.323	252.000
ENSG00000172530	0.000	0.563	0.960	0.166	BANP	0.015	~p_tau+(1|manifest)+cngeneson+pc_mito+brain_region+Sex	2.237	1111.000
ENSG00000172578	0.000	0.563	0.933	0.193	KLHL6	0.020	~p_tau+(1|manifest)+cngeneson+pc_mito+brain_region+Sex	2.358	1038.000
ENSG00000069849	0.000	0.562	0.904	0.219	ATP1B3	0.015	~p_tau+(1|manifest)+cngeneson+pc_mito+brain_region+Sex	0.595	3559.000
ENSG00000173068	0.002	0.556	0.864	0.247	BNC2	0.039	~p_tau+(1|manifest)+cngeneson+pc_mito+brain_region+Sex	2.487	976.000
ENSG00000144401	0.088	0.553	1.210	-0.105	METTL21A	0.282	~p_tau+(1|manifest)+cngeneson+pc_mito+brain_region+Sex	13.279	64.000
ENSG00000163491	0.034	0.549	1.207	-0.108	NEK10	0.172	~p_tau+(1|manifest)+cngeneson+pc_mito+brain_region+Sex	15.994	38.000
ENSG00000030582	0.000	0.548	0.934	0.163	GRN	0.015	~p_tau+(1|manifest)+cngeneson+pc_mito+brain_region+Sex	3.472	644.000
ENSG00000188559	0.000	0.542	0.831	0.253	RALGAPA2	0.015	~p_tau+(1|manifest)+cngeneson+pc_mito+brain_region+Sex	2.154	1152.000
ENSG00000144445	0.018	0.536	1.047	0.024	KANSL1L	0.124	~p_tau+(1|manifest)+cngeneson+pc_mito+brain_region+Sex	7.535	185.000
ENSG00000087460	0.001	0.532	0.871	0.194	GNAS	0.035	~p_tau+(1|manifest)+cngeneson+pc_mito+brain_region+Sex	3.700	589.000
ENSG00000133872	0.003	0.529	0.931	0.127	SARAF	0.050	~p_tau+(1|manifest)+cngeneson+pc_mito+brain_region+Sex	3.358	667.000
ENSG00000265972	0.046	0.527	1.192	-0.137	TXNIP	0.202	~p_tau+(1|manifest)+cngeneson+pc_mito+brain_region+Sex	8.363	146.000
ENSG00000159788	0.000	0.526	0.745	0.307	RGS12	0.010	~p_tau+(1|manifest)+cngeneson+pc_mito+brain_region+Sex	0.956	2535.000
ENSG00000151726	0.413	0.523	1.335	-0.288	ACSL1	0.658	~p_tau+(1|manifest)+cngeneson+pc_mito+brain_region+Sex	10.273	101.000
ENSG00000146070	0.001	0.520	0.836	0.205	PLA2G7	0.026	~p_tau+(1|manifest)+cngeneson+pc_mito+brain_region+Sex	3.726	584.000
ENSG00000133639	0.000	0.520	0.830	0.210	BTG1	0.019	~p_tau+(1|manifest)+cngeneson+pc_mito+brain_region+Sex	2.042	1221.000
ENSG00000126804	0.000	0.519	0.876	0.161	ZBTB1	0.015	~p_tau+(1|manifest)+cngeneson+pc_mito+brain_region+Sex	2.449	995.000
ENSG00000157657	0.008	0.516	0.946	0.086	ZNF618	0.079	~p_tau+(1|manifest)+cngeneson+pc_mito+brain_region+Sex	1.773	1406.000
ENSG00000172667	0.004	0.515	0.843	0.188	ZMAT3	0.057	~p_tau+(1|manifest)+cngeneson+pc_mito+brain_region+Sex	3.041	756.000
ENSG00000168439	0.001	0.513	0.996	0.030	STIP1	0.028	~p_tau+(1|manifest)+cngeneson+pc_mito+brain_region+Sex	4.045	515.000
ENSG00000165029	0.010	0.510	1.032	-0.013	ABCA1	0.089	~p_tau+(1|manifest)+cngeneson+pc_mito+brain_region+Sex	6.819	214.000
ENSG00000020577	0.026	0.505	0.949	0.061	SAMD4A	0.148	~p_tau+(1|manifest)+cngeneson+pc_mito+brain_region+Sex	3.858	555.000
ENSG00000147065	0.020	0.504	0.943	0.065	MSN	0.129	~p_tau+(1|manifest)+cngeneson+pc_mito+brain_region+Sex	4.694	406.000
ENSG00000136156	0.016	0.499	0.873	0.124	ITM2B	0.117	~p_tau+(1|manifest)+cngeneson+pc_mito+brain_region+Sex	8.497	140.000
ENSG00000187554	0.052	0.499	0.996	0.001	TLR5	0.216	~p_tau+(1|manifest)+cngeneson+pc_mito+brain_region+Sex	4.226	485.000
ENSG00000150991	0.227	0.498	1.158	-0.161	UBC	0.476	~p_tau+(1|manifest)+cngeneson+pc_mito+brain_region+Sex	7.384	193.000
ENSG00000170677	0.206	0.498	1.166	-0.170	SOCS6	0.449	~p_tau+(1|manifest)+cngeneson+pc_mito+brain_region+Sex	8.312	149.000
ENSG00000161011	0.000	0.498	0.779	0.216	SQSTM1	0.003	~p_tau+(1|manifest)+cngeneson+pc_mito+brain_region+Sex	1.318	1880.000
ENSG00000151623	0.012	0.495	0.835	0.155	NR3C2	0.100	~p_tau+(1|manifest)+cngeneson+pc_mito+brain_region+Sex	2.128	1165.000
ENSG00000110077	0.121	0.493	1.079	-0.093	MS4A6A	0.337	~p_tau+(1|manifest)+cngeneson+pc_mito+brain_region+Sex	10.226	102.000
ENSG00000198108	0.018	0.492	0.876	0.109	CHSY3	0.122	~p_tau+(1|manifest)+cngeneson+pc_mito+brain_region+Sex	3.229	705.000
ENSG00000110651	0.006	0.490	0.904	0.077	CD81	0.074	~p_tau+(1|manifest)+cngeneson+pc_mito+brain_region+Sex	3.963	528.000
ENSG00000167081	0.024	0.490	0.821	0.159	PBX3	0.144	~p_tau+(1|manifest)+cngeneson+pc_mito+brain_region+Sex	3.237	703.000
ENSG00000131446	0.023	0.488	0.880	0.095	MGAT1	0.141	~p_tau+(1|manifest)+cngeneson+pc_mito+brain_region+Sex	3.111	733.000
ENSG00000219481	0.000	0.485	0.744	0.225	NBPF1	0.003	~p_tau+(1|manifest)+cngeneson+pc_mito+brain_region+Sex	0.775	2998.000
ENSG00000152270	0.187	0.484	1.062	-0.094	PDE3B	0.427	~p_tau+(1|manifest)+cngeneson+pc_mito+brain_region+Sex	11.597	83.000
ENSG00000111790	0.000	0.483	0.713	0.254	FGFR1OP2	0.002	~p_tau+(1|manifest)+cngeneson+pc_mito+brain_region+Sex	0.802	2929.000
ENSG00000197520	0.022	0.483	1.064	-0.099	FAM177B	0.139	~p_tau+(1|manifest)+cngeneson+pc_mito+brain_region+Sex	8.387	144.000
ENSG00000141480	0.002	0.482	0.794	0.171	ARRB2	0.039	~p_tau+(1|manifest)+cngeneson+pc_mito+brain_region+Sex	2.333	1052.000
ENSG00000149927	0.000	0.482	0.846	0.119	DOC2A	0.002	~p_tau+(1|manifest)+cngeneson+pc_mito+brain_region+Sex	1.751	1420.000
ENSG00000143162	0.000	0.482	0.810	0.154	CREG1	0.015	~p_tau+(1|manifest)+cngeneson+pc_mito+brain_region+Sex	2.195	1130.000
ENSG00000121933	0.000	0.480	0.689	0.272	TMIGD3	0.015	~p_tau+(1|manifest)+cngeneson+pc_mito+brain_region+Sex	5.286	330.000
ENSG00000178573	0.094	0.480	0.944	0.017	MAF	0.291	~p_tau+(1|manifest)+cngeneson+pc_mito+brain_region+Sex	10.323	100.000
ENSG00000118855	0.009	0.477	0.844	0.109	MFSD1	0.085	~p_tau+(1|manifest)+cngeneson+pc_mito+brain_region+Sex	2.586	936.000
ENSG00000101752	0.075	0.476	0.978	-0.025	MIB1	0.260	~p_tau+(1|manifest)+cngeneson+pc_mito+brain_region+Sex	7.541	184.000
ENSG00000139946	0.180	0.475	0.995	-0.046	PELI2	0.419	~p_tau+(1|manifest)+cngeneson+pc_mito+brain_region+Sex	6.224	259.000
ENSG00000080546	0.030	0.475	0.888	0.061	SESN1	0.159	~p_tau+(1|manifest)+cngeneson+pc_mito+brain_region+Sex	2.524	957.000
ENSG00000138964	0.000	0.473	0.688	0.258	PARVG	0.003	~p_tau+(1|manifest)+cngeneson+pc_mito+brain_region+Sex	0.681	3263.000
ENSG00000145335	0.064	0.473	0.904	0.042	SNCA	0.241	~p_tau+(1|manifest)+cngeneson+pc_mito+brain_region+Sex	3.495	640.000
ENSG00000170873	0.145	0.468	0.923	0.014	MTSS1	0.370	~p_tau+(1|manifest)+cngeneson+pc_mito+brain_region+Sex	2.328	1056.000
ENSG00000173517	0.001	0.465	0.724	0.205	PEAK1	0.028	~p_tau+(1|manifest)+cngeneson+pc_mito+brain_region+Sex	2.729	880.000
ENSG00000244694	0.004	0.462	0.810	0.114	PTCHD4	0.056	~p_tau+(1|manifest)+cngeneson+pc_mito+brain_region+Sex	2.670	903.000
ENSG00000145390	0.117	0.456	1.019	-0.106	USP53	0.331	~p_tau+(1|manifest)+cngeneson+pc_mito+brain_region+Sex	10.096	104.000
ENSG00000164983	0.022	0.455	0.891	0.020	TMEM65	0.139	~p_tau+(1|manifest)+cngeneson+pc_mito+brain_region+Sex	3.179	716.000
ENSG00000151090	0.066	0.453	1.046	-0.140	THRB	0.244	~p_tau+(1|manifest)+cngeneson+pc_mito+brain_region+Sex	12.487	73.000
ENSG00000248905	0.063	0.449	0.825	0.073	FMN1	0.240	~p_tau+(1|manifest)+cngeneson+pc_mito+brain_region+Sex	9.782	114.000
ENSG00000168264	0.000	0.444	0.769	0.120	IRF2BP2	0.015	~p_tau+(1|manifest)+cngeneson+pc_mito+brain_region+Sex	3.212	708.000
ENSG00000186469	0.007	0.444	0.790	0.098	GNG2	0.074	~p_tau+(1|manifest)+cngeneson+pc_mito+brain_region+Sex	0.613	3483.000
ENSG00000117020	0.042	0.444	0.799	0.089	AKT3	0.192	~p_tau+(1|manifest)+cngeneson+pc_mito+brain_region+Sex	7.863	165.000
ENSG00000070882	0.004	0.442	0.699	0.186	OSBPL3	0.062	~p_tau+(1|manifest)+cngeneson+pc_mito+brain_region+Sex	1.298	1919.000
ENSG00000115875	0.010	0.442	0.860	0.024	SRSF7	0.088	~p_tau+(1|manifest)+cngeneson+pc_mito+brain_region+Sex	2.673	900.000
ENSG00000090376	0.077	0.442	0.918	-0.034	IRAK3	0.264	~p_tau+(1|manifest)+cngeneson+pc_mito+brain_region+Sex	4.542	427.000
ENSG00000150347	0.005	0.441	0.736	0.146	ARID5B	0.066	~p_tau+(1|manifest)+cngeneson+pc_mito+brain_region+Sex	2.467	984.000
ENSG00000171659	0.209	0.441	1.054	-0.173	GPR34	0.452	~p_tau+(1|manifest)+cngeneson+pc_mito+brain_region+Sex	6.219	261.000
ENSG00000185088	0.000	0.439	0.647	0.231	RPS27L	0.003	~p_tau+(1|manifest)+cngeneson+pc_mito+brain_region+Sex	0.830	2862.000
ENSG00000090238	0.000	0.439	0.845	0.033	YPEL3	0.016	~p_tau+(1|manifest)+cngeneson+pc_mito+brain_region+Sex	4.810	393.000
ENSG00000102172	0.000	0.439	0.631	0.246	SMS	0.003	~p_tau+(1|manifest)+cngeneson+pc_mito+brain_region+Sex	0.595	3557.000
ENSG00000134294	0.065	0.438	0.896	-0.021	SLC38A2	0.243	~p_tau+(1|manifest)+cngeneson+pc_mito+brain_region+Sex	1.410	1766.000
ENSG00000083312	0.001	0.437	0.701	0.173	TNPO1	0.028	~p_tau+(1|manifest)+cngeneson+pc_mito+brain_region+Sex	0.910	2649.000
ENSG00000133112	0.205	0.433	0.980	-0.114	TPT1	0.449	~p_tau+(1|manifest)+cngeneson+pc_mito+brain_region+Sex	5.755	300.000
ENSG00000147416	0.009	0.429	0.829	0.029	ATP6V1B2	0.085	~p_tau+(1|manifest)+cngeneson+pc_mito+brain_region+Sex	2.761	865.000
ENSG00000125703	0.001	0.428	0.754	0.102	ATG4C	0.030	~p_tau+(1|manifest)+cngeneson+pc_mito+brain_region+Sex	2.472	981.000
ENSG00000147408	0.298	0.426	1.007	-0.155	[illegible]	0.553	~p_tau+(1|manifest)+cngeneson+pc_mito+brain_region+Sex	20.145	27.000
ENSG00000138764	0.000	0.422	0.759	0.086	CCNG2	0.015	~p_tau+(1|manifest)+cngeneson+pc_mito+brain_region+Sex	6.329	251.000
ENSG00000127922	0.014	0.421	0.823	0.018	SEM1	0.107	~p_tau+(1|manifest)+cngeneson+pc_mito+brain_region+Sex	4.393	457.000
ENSG00000123933	0.005	0.418	0.840	-0.003	MXD4	0.064	~p_tau+(1|manifest)+cngeneson+pc_mito+brain_region+Sex	4.894	381.000
ENSG00000008083	0.006	0.418	0.652	0.183	JARID2	0.073	~p_tau+(1|manifest)+cngeneson+pc_mito+brain_region+Sex	2.704	889.000
ENSG00000185432	0.009	0.417	0.807	0.026	METTL7A	0.085	~p_tau+(1|manifest)+cngeneson+pc_mito+brain_region+Sex	3.834	562.000
ENSG00000164548	0.004	0.416	0.705	0.127	TRA2A	0.056	~p_tau+(1|manifest)+cngeneson+pc_mito+brain_region+Sex	2.660	906.000
ENSG00000075391	0.103	0.415	0.797	0.032	RASAL2	0.307	~p_tau+(1|manifest)+cngeneson+pc_mito+brain_region+Sex	4.555	424.000
ENSG00000159256	0.003	0.413	0.688	0.139	MORC3	0.055	~p_tau+(1|manifest)+cngeneson+pc_mito+brain_region+Sex	1.415	1761.000
ENSG00000049759	0.109	0.412	0.829	-0.005	NEDD4L	0.317	~p_tau+(1|manifest)+cngeneson+pc_mito+brain_region+Sex	3.789	568.000
ENSG00000114416	0.175	0.410	0.974	-0.155	FXR1	0.411	~p_tau+(1|manifest)+cngeneson+pc_mito+brain_region+Sex	9.525	118.000
ENSG00000143443	0.016	0.410	0.930	-0.111	C1orf56	0.117	~p_tau+(1|manifest)+cngeneson+pc_mito+brain_region+Sex	3.683	599.000
ENSG00000100380	0.042	0.408	0.891	-0.075	ST13	0.191	~p_tau+(1|manifest)+cngeneson+pc_mito+brain_region+Sex	10.337	99.000
ENSG00000088448	0.001	0.408	0.664	0.152	ANKRD10	0.037	~p_tau+(1|manifest)+cngeneson+pc_mito+brain_region+Sex	1.831	1368.000
ENSG00000196199	0.008	0.406	0.732	0.081	MPHOSPH8	0.080	~p_tau+(1|manifest)+cngeneson+pc_mito+brain_region+Sex	2.736	876.000
ENSG00000143507	0.001	0.406	0.710	0.103	DUSP10	0.035	~p_tau+(1|manifest)+cngeneson+pc_mito+brain_region+Sex	2.018	1235.000
ENSG00000132170	0.012	0.405	0.680	0.130	PPARG	0.097	~p_tau+(1|manifest)+cngeneson+pc_mito+brain_region+Sex	1.867	1339.000
ENSG00000143376	0.000	0.405	0.620	0.189	SNX27	0.006	~p_tau+(1|manifest)+cngeneson+pc_mito+brain_region+Sex	0.169	5336.000
ENSG00000148154	0.017	0.405	0.726	0.083	UGCG	0.121	~p_tau+(1|manifest)+cngeneson+pc_mito+brain_region+Sex	3.240	701.000
ENSG00000170017	0.026	0.404	0.675	0.132	ALCAM	0.148	~p_tau+(1|manifest)+cngeneson+pc_mito+brain_region+Sex	2.112	1178.000
ENSG00000181722	0.116	0.404	0.767	0.040	ZBTB20	0.330	~p_tau+(1|manifest)+cngeneson+pc_mito+brain_region+Sex	7.780	171.000
ENSG00000138760	0.008	0.404	0.715	0.093	SCARB2	0.079	~p_tau+(1|manifest)+cngeneson+pc_mito+brain_region+Sex	2.364	1032.000
ENSG00000117115	0.146	0.403	0.853	-0.047	PADI2	0.370	~p_tau+(1|manifest)+cngeneson+pc_mito+brain_region+Sex	5.149	347.000
ENSG00000114541	0.336	0.403	0.919	-0.113	FRMD4B	0.592	~p_tau+(1|manifest)+cngeneson+pc_mito+brain_region+Sex	7.982	161.000
ENSG00000170606	0.015	0.403	0.784	0.021	HSPA4	0.113	~p_tau+(1|manifest)+cngeneson+pc_mito+brain_region+Sex	4.672	410.000
ENSG00000013441	0.004	0.402	0.747	0.056	CLK1	0.058	~p_tau+(1|manifest)+cngeneson+pc_mito+brain_region+Sex	3.932	537.000
ENSG00000165113	0.007	0.398	0.736	0.060	GKAP1	0.074	~p_tau+(1|manifest)+cngeneson+pc_mito+brain_region+Sex	5.259	334.000
ENSG00000082898	0.004	0.395	0.690	0.099	XPO1	0.058	~p_tau+(1|manifest)+cngeneson+pc_mito+brain_region+Sex	2.470	982.000
ENSG00000172175	0.010	0.395	0.644	0.146	MALT1	0.089	~p_tau+(1|manifest)+cngeneson+pc_mito+brain_region+Sex	2.261	1095.000
ENSG00000148158	0.001	0.394	0.603	0.184	SNX30	0.033	~p_tau+(1|manifest)+cngeneson+pc_mito+brain_region+Sex	1.059	2328.000
ENSG00000130208	0.036	0.393	0.805	-0.019	APOC1	0.176	~p_tau+(1|manifest)+cngeneson+pc_mito+brain_region+Sex	4.989	370.000
ENSG00000078674	0.001	0.392	0.599	0.185	PCM1	0.031	~p_tau+(1|manifest)+cngeneson+pc_mito+brain_region+Sex	4.306	476.000
ENSG00000096746	0.000	0.392	0.609	0.174	HNRNPH3	0.015	~p_tau+(1|manifest)+cngeneson+pc_mito+brain_region+Sex	0.895	2678.000
ENSG00000221869	0.285	0.391	1.142	-0.360	CEBPD	0.539	~p_tau+(1|manifest)+cngeneson+pc_mito+brain_region+Sex	12.111	76.000
ENSG00000163131	0.000	0.390	0.594	0.187	CTSS	0.013	~p_tau+(1|manifest)+cngeneson+pc_mito+brain_region+Sex	0.929	2593.000
ENSG00000126821	0.024	0.380	0.690	0.070	SGPP1	0.142	~p_tau+(1|manifest)+cngeneson+pc_mito+brain_region+Sex	1.959	1271.000
ENSG00000197971	0.029	0.380	0.647	0.113	MBP	0.158	~p_tau+(1|manifest)+cngeneson+pc_mito+brain_region+Sex	2.685	896.000
ENSG00000102531	0.002	0.379	0.613	0.146	FNDC3A	0.040	~p_tau+(1|manifest)+cngeneson+pc_mito+brain_region+Sex	0.819	2892.000
ENSG00000136295	0.000	0.379	0.588	0.170	TTYH3	0.016	~p_tau+(1|manifest)+cngeneson+pc_mito+brain_region+Sex	1.222	2045.000
ENSG00000182185	0.085	0.378	0.700	0.055	RAD51B	0.277	~p_tau+(1|manifest)+cngeneson+pc_mito+brain_region+Sex	2.314	1061.000
ENSG00000166928	0.002	0.377	0.656	0.098	MS4A14	0.038	~p_tau+(1|manifest)+cngeneson+pc_mito+brain_region+Sex	2.815	834.000
ENSG00000186815	0.020	0.377	0.671	0.082	TPCN1	0.132	~p_tau+(1|manifest)+cngeneson+pc_mito+brain_region+Sex	2.422	1006.000
ENSG00000160179	0.010	0.376	0.680	0.073	ABCG1	0.091	~p_tau+(1|manifest)+cngeneson+pc_mito+brain_region+Sex	4.874	382.000
ENSG00000023171	0.053	0.376	0.758	-0.005	GRAMD1B	0.219	~p_tau+(1|manifest)+cngeneson+pc_mito+brain_region+Sex	5.672	306.000
ENSG00000120963	0.003	0.374	0.725	0.023	ZNF706	0.050	~p_tau+(1|manifest)+cngeneson+pc_mito+brain_region+Sex	2.873	811.000
ENSG00000139278	0.045	0.374	0.815	-0.068	GLIPR1	0.200	~p_tau+(1|manifest)+cngeneson+pc_mito+brain_region+Sex	4.543	426.000
ENSG00000135678	0.168	0.373	0.826	-0.080	CPM	0.401	~p_tau+(1|manifest)+cngeneson+pc_mito+brain_region+Sex	2.427	1004.000
ENSG00000139154	0.000	0.373	0.644	0.102	AEBP2	0.015	~p_tau+(1|manifest)+cngeneson+pc_mito+brain_region+Sex	1.232	2032.000
ENSG00000189067	0.000	0.373	0.594	0.151	LITAF	0.015	~p_tau+(1|manifest)+cngeneson+pc_mito+brain_region+Sex	1.896	1314.000
ENSG00000119655	0.003	0.371	0.632	0.111	NPC2	0.050	~p_tau+(1|manifest)+cngeneson+pc_mito+brain_region+Sex	2.998	774.000
ENSG00000151883	0.004	0.370	0.602	0.138	PARP8	0.061	~p_tau+(1|manifest)+cngeneson+pc_mito+brain_region+Sex	1.524	1634.000
ENSG00000164136	0.161	0.367	0.852	-0.117	IL15	0.391	~p_tau+(1|manifest)+cngeneson+pc_mito+brain_region+Sex	4.291	479.000
ENSG00000135968	0.013	0.367	0.621	0.113	GCC2	0.102	~p_tau+(1|manifest)+cngeneson+pc_mito+brain_region+Sex	1.628	1526.000
ENSG00000171860	0.002	0.367	0.645	0.089	C3AR1	0.038	~p_tau+(1|manifest)+cngeneson+pc_mito+brain_region+Sex	2.216	1127.000
ENSG00000107562	0.004	0.366	0.816	-0.083	CXCL12	0.058	~p_tau+(1|manifest)+cngeneson+pc_mito+brain_region+Sex	4.411	453.000
ENSG00000198286	0.419	0.366	0.950	-0.218	CARD11	0.662	~p_tau+(1|manifest)+cngeneson+pc_mito+brain_region+Sex	4.491	436.000
ENSG00000118689	0.084	0.365	0.731	-0.002	FOXO3	0.276	~p_tau+(1|manifest)+cngeneson+pc_mito+brain_region+Sex	2.788	847.000
ENSG00000171298	0.001	0.364	0.607	0.122	GAA	0.026	~p_tau+(1|manifest)+cngeneson+pc_mito+brain_region+Sex	2.926	791.000
ENSG00000140575	0.081	0.364	0.689	0.038	IQGAP1	0.270	~p_tau+(1|manifest)+cngeneson+pc_mito+brain_region+Sex	4.032	516.000
ENSG00000172766	0.039	0.359	0.787	-0.068	NAA16	0.185	~p_tau+(1|manifest)+cngeneson+pc_mito+brain_region+Sex	6.603	235.000
ENSG00000112576	0.548	0.359	1.084	-0.366	CCND3	0.755	~p_tau+(1|manifest)+cngeneson+pc_mito+brain_region+Sex	12.676	70.000
ENSG00000167280	0.001	0.358	0.651	0.064	ENGASE	0.033	~p_tau+(1|manifest)+cngeneson+pc_mito+brain_region+Sex	2.367	1031.000
ENSG00000100784	0.089	0.357	0.808	-0.095	RPS6KA5	0.284	~p_tau+(1|manifest)+cngeneson+pc_mito+brain_region+Sex	5.595	309.000
ENSG00000143641	0.050	0.355	0.641	0.070	GALNT2	0.210	~p_tau+(1|manifest)+cngeneson+pc_mito+brain_region+Sex	2.685	895.000
ENSG00000164713	0.000	0.353	0.519	0.187	BRI3	0.003	~p_tau+(1|manifest)+cngeneson+pc_mito+brain_region+Sex	1.191	2094.000
ENSG00000175471	0.015	0.353	0.684	0.021	MCTP1	0.112	~p_tau+(1|manifest)+cngeneson+pc_mito+brain_region+Sex	2.342	1047.000
ENSG00000154447	0.143	0.352	0.793	-0.089	SH3RF1	0.367	~p_tau+(1|manifest)+cngeneson+pc_mito+brain_region+Sex	3.794	567.000
ENSG00000116161	0.003	0.351	0.710	-0.007	CACYBP	0.055	~p_tau+(1|manifest)+cngeneson+pc_mito+brain_region+Sex	5.729	303.000
ENSG00000115524	0.011	0.351	0.650	0.051	SF3B1	0.094	~p_tau+(1|manifest)+cngeneson+pc_mito+brain_region+Sex	2.609	924.000
ENSG00000108669	0.006	0.351	0.566	0.135	CYTH1	0.073	~p_tau+(1|manifest)+cngeneson+pc_mito+brain_region+Sex	2.234	1112.000
ENSG00000135838	0.076	0.351	0.739	-0.038	NPL	0.262	~p_tau+(1|manifest)+cngeneson+pc_mito+brain_region+Sex	1.170	2126.000
ENSG00000163106	0.123	0.350	0.778	-0.077	HPGDS	0.339	~p_tau+(1|manifest)+cngeneson+pc_mito+brain_region+Sex	3.958	533.000
ENSG00000160209	0.057	0.350	0.710	-0.010	PDXK	0.227	~p_tau+(1|manifest)+cngeneson+pc_mito+brain_region+Sex	3.932	538.000
ENSG00000205517	0.001	0.349	0.568	0.129	RGL3	0.028	~p_tau+(1|manifest)+cngeneson+pc_mito+brain_region+Sex	1.520	1639.000
ENSG00000197063	0.000	0.347	0.550	0.144	MAFG	0.011	~p_tau+(1|manifest)+cngeneson+pc_mito+brain_region+Sex	1.158	2148.000
ENSG00000102908	0.014	0.347	0.654	0.040	NFAT5	0.110	~p_tau+(1|manifest)+cngeneson+pc_mito+brain_region+Sex	1.724	1436.000
ENSG00000186409	0.000	0.345	0.608	0.083	CCDC30	0.018	~p_tau+(1|manifest)+cngeneson+pc_mito+brain_region+Sex	3.284	689.000
ENSG00000182601	0.475	0.344	0.893	-0.204	HS3ST4	0.703	~p_tau+(1|manifest)+cngeneson+pc_mito+brain_region+Sex	20.918	24.000
ENSG00000172932	0.000	0.344	0.507	0.181	ANKRD13D	0.003	~p_tau+(1|manifest)+cngeneson+pc_mito+brain_region+Sex	1.002	2433.000
ENSG00000112335	0.009	0.344	0.713	-0.025	SNX3	0.084	~p_tau+(1|manifest)+cngeneson+pc_mito+brain_region+Sex	2.834	826.000
ENSG00000136720	0.001	0.343	0.568	0.119	HS6ST1	0.030	~p_tau+(1|manifest)+cngeneson+pc_mito+brain_region+Sex	1.833	1365.000
ENSG00000100100	0.013	0.343	0.645	0.041	PIK3IP1	0.103	~p_tau+(1|manifest)+cngeneson+pc_mito+brain_region+Sex	2.104	1183.000
ENSG00000108773	0.002	0.342	0.646	0.039	KAT2A	0.040	~p_tau+(1|manifest)+cngeneson+pc_mito+brain_region+Sex	2.244	1104.000
ENSG00000156931	0.001	0.342	0.530	0.154	VPS8	0.031	~p_tau+(1|manifest)+cngeneson+pc_mito+brain_region+Sex	0.687	3243.000
ENSG00000052126	0.024	0.341	0.634	0.048	PLEKHA5	0.142	~p_tau+(1|manifest)+cngeneson+pc_mito+brain_region+Sex	2.644	916.000
ENSG00000196950	0.008	0.341	0.629	0.053	SLC39A10	0.080	~p_tau+(1|manifest)+cngeneson+pc_mito+brain_region+Sex	2.115	1174.000
ENSG00000064225	0.140	0.340	0.717	-0.037	ST3GAL6	0.363	~p_tau+(1|manifest)+cngeneson+pc_mito+brain_region+Sex	2.736	877.000
ENSG00000017260	0.337	0.338	0.813	-0.137	ATP2C1	0.593	~p_tau+(1|manifest)+cngeneson+pc_mito+brain_region+Sex	14.390	50.000
ENSG00000245848	0.005	0.337	0.629	0.045	CEBPA	0.063	~p_tau+(1|manifest)+cngeneson+pc_mito+brain_region+Sex	2.376	1030.000
ENSG00000111144	0.000	0.336	0.542	0.131	LTA4H	0.015	~p_tau+(1|manifest)+cngeneson+pc_mito+brain_region+Sex	0.492	3975.000
ENSG00000145348	0.003	0.336	0.533	0.139	TBCK	0.054	~p_tau+(1|manifest)+cngeneson+pc_mito+brain_region+Sex	0.562	3678.000
ENSG00000099250	0.386	0.336	0.823	-0.152	NRP1	0.634	~p_tau+(1|manifest)+cngeneson+pc_mito+brain_region+Sex	1.622	1528.000
ENSG00000151322	0.096	0.335	0.847	-0.177	NPAS3	0.296	~p_tau+(1|manifest)+cngeneson+pc_mito+brain_region+Sex	2.385	1024.000
ENSG00000108306	0.343	0.334	0.779	-0.112	FBXL20	0.597	~p_tau+(1|manifest)+cngeneson+pc_mito+brain_region+Sex	5.529	314.000
ENSG00000197746	0.046	0.334	0.638	0.029	PSAP	0.201	~p_tau+(1|manifest)+cngeneson+pc_mito+brain_region+Sex	6.115	274.000
ENSG00000162869	0.005	0.333	0.542	0.125	PPP1R21	0.064	~p_tau+(1|manifest)+cngeneson+pc_mito+brain_region+Sex	0.953	2541.000
ENSG00000136732	0.000	0.333	0.498	0.168	GYPC	0.010	~p_tau+(1|manifest)+cngeneson+pc_mito+brain_region+Sex	0.329	4682.000
ENSG00000187474	0.039	0.333	0.668	-0.002	FPR3	0.185	~p_tau+(1|manifest)+cngeneson+pc_mito+brain_region+Sex	3.387	658.000
ENSG00000148090	0.009	0.332	0.600	0.065	AUH	0.084	~p_tau+(1|manifest)+cngeneson+pc_mito+brain_region+Sex	1.834	1363.000
ENSG00000141376	0.296	0.331	0.785	-0.123	BCAS3	0.550	~p_tau+(1|manifest)+cngeneson+pc_mito+brain_region+Sex	4.518	431.000
ENSG00000089327	0.000	0.331	0.620	0.042	FXYD5	0.016	~p_tau+(1|manifest)+cngeneson+pc_mito+brain_region+Sex	2.113	1176.000
ENSG00000114770	0.000	0.330	0.537	0.123	ABCC5	0.016	~p_tau+(1|manifest)+cngeneson+pc_mito+brain_region+Sex	0.960	2522.000
ENSG00000184922	0.010	0.330	0.614	0.045	FMNL1	0.091	~p_tau+(1|manifest)+cngeneson+pc_mito+brain_region+Sex	1.941	1286.000
ENSG00000248643	0.000	0.329	0.541	0.117	[illegible]	0.015	~p_tau+(1|manifest)+cngeneson+pc_mito+brain_region+Sex	1.085	2279.000
ENSG00000088854	0.066	0.329	0.599	0.059	C20orf194	0.244	~p_tau+(1|manifest)+cngeneson+pc_mito+brain_region+Sex	1.701	1453.000
ENSG00000011201	0.000	0.328	0.456	0.200	ANOS1	0.016	~p_tau+(1|manifest)+cngeneson+pc_mito+brain_region+Sex	1.164	2138.000
ENSG00000091831	0.078	0.328	0.677	-0.021	ESR1	0.266	~p_tau+(1|manifest)+cngeneson+pc_mito+brain_region+Sex	2.796	843.000
ENSG00000127947	0.001	0.328	0.523	0.132	PTPN12	0.035	~p_tau+(1|manifest)+cngeneson+pc_mito+brain_region+Sex	1.404	1767.000
ENSG00000106785	0.002	0.327	0.532	0.123	TRIM14	0.038	~p_tau+(1|manifest)+cngeneson+pc_mito+brain_region+Sex	0.879	2717.000
ENSG00000123329	0.000	0.327	0.578	0.075	ARHGAP9	0.020	~p_tau+(1|manifest)+cngeneson+pc_mito+brain_region+Sex	0.740	3085.000
ENSG00000148634	0.066	0.324	0.626	0.023	HERC4	0.244	~p_tau+(1|manifest)+cngeneson+pc_mito+brain_region+Sex	4.954	373.000
ENSG00000122884	0.373	0.324	0.811	-0.162	P4HA1	0.625	~p_tau+(1|manifest)+cngeneson+pc_mito+brain_region+Sex	5.001	367.000
ENSG00000153944	0.027	0.322	0.547	0.096	MSI2	0.151	~p_tau+(1|manifest)+cngeneson+pc_mito+brain_region+Sex	1.736	1426.000
ENSG00000167291	0.038	0.320	0.622	0.018	TBC1D16	0.182	~p_tau+(1|manifest)+cngeneson+pc_mito+brain_region+Sex	2.400	1021.000
ENSG00000107745	0.001	0.319	0.477	0.162	MICU1	0.037	~p_tau+(1|manifest)+cngeneson+pc_mito+brain_region+Sex	0.395	4380.000
ENSG00000275023	0.013	0.319	0.594	0.044	MLLT6	0.102	~p_tau+(1|manifest)+cngeneson+pc_mito+brain_region+Sex	2.764	860.000
ENSG00000090060	0.065	0.319	0.733	-0.095	PAPOLA	0.243	~p_tau+(1|manifest)+cngeneson+pc_mito+brain_region+Sex	4.464	442.000
ENSG00000164116	0.144	0.318	0.771	-0.136	GUCY1A1	0.367	~p_tau+(1|manifest)+cngeneson+pc_mito+brain_region+Sex	4.936	374.000
ENSG00000115904	0.067	0.316	0.619	0.012	SOS1	0.245	~p_tau+(1|manifest)+cngeneson+pc_mito+brain_region+Sex	3.181	714.000
ENSG00000104219	0.003	0.316	0.568	0.064	ZDHHC2	0.056	~p_tau+(1|manifest)+cngeneson+pc_mito+brain_region+Sex	1.460	1708.000
ENSG00000136436	0.033	0.315	0.699	-0.069	CALCOCO2	0.170	~p_tau+(1|manifest)+cngeneson+pc_mito+brain_region+Sex	3.473	643.000
ENSG00000134278	0.005	0.315	0.524	0.106	SPIRE1	0.063	~p_tau+(1|manifest)+cngeneson+pc_mito+brain_region+Sex	0.814	2906.000
ENSG00000178764	0.049	0.314	0.577	0.052	ZHX2	0.208	~p_tau+(1|manifest)+cngeneson+pc_mito+brain_region+Sex	1.440	1736.000
ENSG00000148842	0.001	0.314	0.487	0.142	CNNM2	0.028	~p_tau+(1|manifest)+cngeneson+pc_mito+brain_region+Sex	0.605	3513.000
ENSG00000214226	0.001	0.314	0.677	-0.049	C17orf67	0.035	~p_tau+(1|manifest)+cngeneson+pc_mito+brain_region+Sex	4.857	387.000
ENSG00000167216	0.002	0.313	0.539	0.087	KATNAL2	0.039	~p_tau+(1|manifest)+cngeneson+pc_mito+brain_region+Sex	1.634	1520.000
ENSG00000128487	0.163	0.313	0.680	-0.054	SPECC1	0.393	~p_tau+(1|manifest)+cngeneson+pc_mito+brain_region+Sex	1.939	1288.000
ENSG00000101194	0.001	0.312	0.562	0.061	SLC17A9	0.028	~p_tau+(1|manifest)+cngeneson+pc_mito+brain_region+Sex	1.490	1675.000
ENSG00000033627	0.015	0.311	0.603	0.020	ATP6V0A1	0.111	~p_tau+(1|manifest)+cngeneson+pc_mito+brain_region+Sex	1.607	1548.000
ENSG00000184903	0.034	0.311	0.528	0.095	IMMP2L	0.171	~p_tau+(1|manifest)+cngeneson+pc_mito+brain_region+Sex	1.877	1331.000
ENSG00000100906	0.004	0.311	0.540	0.082	NFKBIA	0.062	~p_tau+(1|manifest)+cngeneson+pc_mito+brain_region+Sex	1.579	1582.000
ENSG00000152818	0.231	0.310	0.676	-0.056	UTRN	0.480	~p_tau+(1|manifest)+cngeneson+pc_mito+brain_region+Sex	4.028	517.000
ENSG00000108654	0.037	0.310	0.553	0.066	DDX5	0.181	~p_tau+(1|manifest)+cngeneson+pc_mito+brain_region+Sex	3.546	631.000
ENSG00000116678	0.011	0.309	0.569	0.050	LEPR	0.094	~p_tau+(1|manifest)+cngeneson+pc_mito+brain_region+Sex	1.472	1692.000
ENSG00000275395	0.276	0.309	0.794	-0.176	FCGBP	0.529	~p_tau+(1|manifest)+cngeneson+pc_mito+brain_region+Sex	2.796	844.000
ENSG00000111666	0.145	0.308	0.847	-0.230	CHPT1	0.370	~p_tau+(1|manifest)+cngeneson+pc_mito+brain_region+Sex	3.337	673.000
ENSG00000213983	0.000	0.308	0.509	0.107	AP1G2	0.010	~p_tau+(1|manifest)+cngeneson+pc_mito+brain_region+Sex	1.287	1931.000
ENSG00000107798	0.016	0.307	0.575	0.038	LIPA	0.115	~p_tau+(1|manifest)+cngeneson+pc_mito+brain_region+Sex	1.529	1624.000
ENSG00000124422	0.000	0.306	0.437	0.176	USP22	0.005	~p_tau+(1|manifest)+cngeneson+pc_mito+brain_region+Sex	0.466	4077.000
ENSG00000120162	0.018	0.306	0.547	0.065	MOB3B	0.123	~p_tau+(1|manifest)+cngeneson+pc_mito+brain_region+Sex	1.134	2188.000
ENSG00000145088	0.047	0.305	0.616	-0.007	EAF2	0.202	~p_tau+(1|manifest)+cngeneson+pc_mito+brain_region+Sex	0.913	2641.000
ENSG00000164300	0.004	0.304	0.498	0.111	SERINC5	0.062	~p_tau+(1|manifest)+cngeneson+pc_mito+brain_region+Sex	0.755	3049.000
ENSG00000160953	0.004	0.304	0.575	0.034	PWWP3A	0.058	~p_tau+(1|manifest)+cngeneson+pc_mito+brain_region+Sex	1.945	1279.000
ENSG00000163539	0.002	0.304	0.475	0.133	CLASP2	0.047	~p_tau+(1|manifest)+cngeneson+pc_mito+brain_region+Sex	0.629	3423.000
ENSG00000137266	0.294	0.303	0.714	-0.107	SLC22A23	0.547	~p_tau+(1|manifest)+cngeneson+pc_mito+brain_region+Sex	5.498	318.000
ENSG00000145715	0.003	0.303	0.495	0.111	RASA1	0.055	~p_tau+(1|manifest)+cngeneson+pc_mito+brain_region+Sex	2.390	1022.000
ENSG00000162980	0.008	0.302	0.522	0.083	ARL5A	0.081	~p_tau+(1|manifest)+cngeneson+pc_mito+brain_region+Sex	1.347	1836.000
ENSG00000131149	0.002	0.300	0.484	0.117	GSE1	0.040	~p_tau+(1|manifest)+cngeneson+pc_mito+brain_region+Sex	0.470	4058.000
ENSG00000116679	0.002	0.300	0.533	0.067	IVNS1ABP	0.039	~p_tau+(1|manifest)+cngeneson+pc_mito+brain_region+Sex	1.422	1755.000
ENSG00000150995	0.204	0.300	0.647	-0.046	ITPR1	0.448	~p_tau+(1|manifest)+cngeneson+pc_mito+brain_region+Sex	0.499	3935.000
ENSG00000163468	0.004	0.299	0.625	-0.026	CCT3	0.058	~p_tau+(1|manifest)+cngeneson+pc_mito+brain_region+Sex	2.888	804.000
ENSG00000105193	0.030	0.299	0.624	-0.027	RPS16	0.159	~p_tau+(1|manifest)+cngeneson+pc_mito+brain_region+Sex	2.465	986.000
ENSG00000101040	0.007	0.298	0.507	0.088	ZMYND8	0.078	~p_tau+(1|manifest)+cngeneson+pc_mito+brain_region+Sex	1.098	2254.000
ENSG00000176974	0.016	0.297	0.543	0.052	SHMT1	0.115	~p_tau+(1|manifest)+cngeneson+pc_mito+brain_region+Sex	1.093	2270.000
ENSG00000171132	0.066	0.297	0.589	0.005	PRKCE	0.244	~p_tau+(1|manifest)+cngeneson+pc_mito+brain_region+Sex	1.511	1651.000
ENSG00000114554	0.001	0.297	0.533	0.061	PLXNA1	0.035	~p_tau+(1|manifest)+cngeneson+pc_mito+brain_region+Sex	1.091	2273.000
ENSG00000130303	0.036	0.297	0.703	-0.109	BST2	0.177	~p_tau+(1|manifest)+cngeneson+pc_mito+brain_region+Sex	2.264	1093.000
ENSG00000162367	0.036	0.297	0.568	0.026	TAL1	0.177	~p_tau+(1|manifest)+cngeneson+pc_mito+brain_region+Sex	2.054	1211.000
ENSG00000165416	0.013	0.296	0.646	-0.054	SUGT1	0.104	~p_tau+(1|manifest)+cngeneson+pc_mito+brain_region+Sex	4.968	371.000
ENSG00000147526	0.012	0.295	0.543	0.047	TACC1	0.096	~p_tau+(1|manifest)+cngeneson+pc_mito+brain_region+Sex	1.053	2344.000
ENSG00000121879	0.104	0.295	0.581	0.009	PIK3CA	0.309	~p_tau+(1|manifest)+cngeneson+pc_mito+brain_region+Sex	2.293	1077.000
ENSG00000107957	0.001	0.294	0.540	0.048	SH3PXD2A	0.027	~p_tau+(1|manifest)+cngeneson+pc_mito+brain_region+Sex	1.485	1679.000
ENSG00000114439	0.203	0.294	0.624	-0.036	BBX	0.446	~p_tau+(1|manifest)+cngeneson+pc_mito+brain_region+Sex	3.666	604.000
ENSG00000156508	0.169	0.293	0.684	-0.097	EEF1A1	0.401	~p_tau+(1|manifest)+cngeneson+pc_mito+brain_region+Sex	4.452	445.000
ENSG00000177463	0.005	0.293	0.530	0.056	NR2C2	0.063	~p_tau+(1|manifest)+cngeneson+pc_mito+brain_region+Sex	1.133	2189.000
ENSG00000244754	0.002	0.293	0.481	0.105	N4BP2L2	0.046	~p_tau+(1|manifest)+cngeneson+pc_mito+brain_region+Sex	3.699	590.000
ENSG00000103811	0.004	0.293	0.595	-0.010	CTSH	0.057	~p_tau+(1|manifest)+cngeneson+pc_mito+brain_region+Sex	1.580	1581.000
ENSG00000119314	0.007	0.292	0.475	0.109	PTBP3	0.076	~p_tau+(1|manifest)+cngeneson+pc_mito+brain_region+Sex	1.647	1503.000
ENSG00000197622	0.006	0.291	0.530	0.053	CDC42SE1	0.073	~p_tau+(1|manifest)+cngeneson+pc_mito+brain_region+Sex	1.314	1886.000
ENSG00000132002	0.192	0.290	0.712	-0.133	DNAJB1	0.433	~p_tau+(1|manifest)+cngeneson+pc_mito+brain_region+Sex	11.004	86.000
ENSG00000140548	0.046	0.288	0.504	0.072	ZNF710	0.202	~p_tau+(1|manifest)+cngeneson+pc_mito+brain_region+Sex	2.657	909.000
ENSG00000141506	0.024	0.286	0.517	0.056	PIK3R5	0.141	~p_tau+(1|manifest)+cngeneson+pc_mito+brain_region+Sex	1.260	1977.000
ENSG00000233927	0.013	0.286	0.603	-0.031	RPS28	0.106	~p_tau+(1|manifest)+cngeneson+pc_mito+brain_region+Sex	2.673	901.000
ENSG00000009413	0.006	0.286	0.447	0.124	REV3L	0.073	~p_tau+(1|manifest)+cngeneson+pc_mito+brain_region+Sex	0.284	4907.000
ENSG00000130522	0.004	0.285	0.473	0.098	JUND	0.060	~p_tau+(1|manifest)+cngeneson+pc_mito+brain_region+Sex	1.246	2009.000
ENSG00000091592	0.585	0.285	0.982	-0.413	NLRP1	0.778	~p_tau+(1|manifest)+cngeneson+pc_mito+brain_region+Sex	15.261	44.000
ENSG00000139117	0.038	0.283	0.544	0.023	CPNE8	0.182	~p_tau+(1|manifest)+cngeneson+pc_mito+brain_region+Sex	1.773	1407.000
ENSG00000119900	0.163	0.283	0.720	-0.155	OGFRL1	0.395	~p_tau+(1|manifest)+cngeneson+pc_mito+brain_region+Sex	4.997	368.000
ENSG00000150593	0.230	0.282	0.697	-0.133	PDCD4	0.479	~p_tau+(1|manifest)+cngeneson+pc_mito+brain_region+Sex	5.282	331.000
ENSG00000156482	0.023	0.282	0.610	-0.046	RPL30	0.141	~p_tau+(1|manifest)+cngeneson+pc_mito+brain_region+Sex	2.443	1000.000
ENSG00000102524	0.102	0.281	0.599	-0.036	TNFSF13B	0.305	~p_tau+(1|manifest)+cngeneson+pc_mito+brain_region+Sex	2.415	1009.000
ENSG00000184293	0.092	0.279	0.858	-0.301	CLECL1	0.289	~p_tau+(1|manifest)+cngeneson+pc_mito+brain_region+Sex	4.860	386.000
ENSG00000134574	0.047	0.278	0.510	0.046	DDB2	0.203	~p_tau+(1|manifest)+cngeneson+pc_mito+brain_region+Sex	1.573	1591.000
ENSG00000185305	0.069	0.278	0.498	0.058	ARL15	0.248	~p_tau+(1|manifest)+cngeneson+pc_mito+brain_region+Sex	1.660	1489.000
ENSG00000149256	0.391	0.278	0.693	-0.137	TENM4	0.638	~p_tau+(1|manifest)+cngeneson+pc_mito+brain_region+Sex	3.419	653.000
ENSG00000197006	0.094	0.278	0.631	-0.075	METTL9	0.290	~p_tau+(1|manifest)+cngeneson+pc_mito+brain_region+Sex	2.840	823.000
ENSG00000178695	0.043	0.278	0.538	0.017	KCTD12	0.193	~p_tau+(1|manifest)+cngeneson+pc_mito+brain_region+Sex	3.111	732.000
ENSG00000173715	0.005	0.278	0.490	0.065	C11orf80	0.063	~p_tau+(1|manifest)+cngeneson+pc_mito+brain_region+Sex	2.189	1135.000
ENSG00000119048	0.081	0.277	0.630	-0.075	UBE2B	0.271	~p_tau+(1|manifest)+cngeneson+pc_mito+brain_region+Sex	3.611	620.000
ENSG00000150093	0.022	0.276	0.487	0.065	ITGB1	0.137	~p_tau+(1|manifest)+cngeneson+pc_mito+brain_region+Sex	1.513	1648.000
ENSG00000161542	0.018	0.275	0.468	0.082	PRPSAP1	0.124	~p_tau+(1|manifest)+cngeneson+pc_mito+brain_region+Sex	0.978	2479.000
ENSG00000145246	0.075	0.275	0.599	-0.049	ATP10D	0.260	~p_tau+(1|manifest)+cngeneson+pc_mito+brain_region+Sex	1.886	1323.000
ENSG00000043093	0.002	0.275	0.460	0.089	DCUN1D1	0.041	~p_tau+(1|manifest)+cngeneson+pc_mito+brain_region+Sex	0.567	3656.000
ENSG00000147403	0.069	0.274	0.641	-0.092	RPL10	0.248	~p_tau+(1|manifest)+cngeneson+pc_mito+brain_region+Sex	5.924	286.000
ENSG00000146409	0.012	0.274	0.518	0.030	SLC18B1	0.098	~p_tau+(1|manifest)+cngeneson+pc_mito+brain_region+Sex	1.478	1685.000
ENSG00000173482	0.218	0.274	0.665	-0.117	PTPRM	0.463	~p_tau+(1|manifest)+cngeneson+pc_mito+brain_region+Sex	2.880	807.000
ENSG00000124496	0.153	0.273	0.597	-0.050	TRERF1	0.381	~p_tau+(1|manifest)+cngeneson+pc_mito+brain_region+Sex	7.878	164.000
ENSG00000205302	0.009	0.272	0.469	0.075	SNX2	0.084	~p_tau+(1|manifest)+cngeneson+pc_mito+brain_region+Sex	1.279	1949.000
ENSG00000162244	0.047	0.272	0.590	-0.046	RPL29	0.202	~p_tau+(1|manifest)+cngeneson+pc_mito+brain_region+Sex	1.951	1275.000
ENSG00000144746	0.000	0.271	0.457	0.084	ARL6IP5	0.015	~p_tau+(1|manifest)+cngeneson+pc_mito+brain_region+Sex	0.910	2648.000
ENSG00000082996	0.161	0.270	0.543	-0.004	RNF13	0.391	~p_tau+(1|manifest)+cngeneson+pc_mito+brain_region+Sex	2.787	849.000
ENSG00000170315	0.010	0.269	0.494	0.045	UBB	0.091	~p_tau+(1|manifest)+cngeneson+pc_mito+brain_region+Sex	1.155	2153.000
ENSG00000163755	0.005	0.269	0.460	0.078	HPS3	0.063	~p_tau+(1|manifest)+cngeneson+pc_mito+brain_region+Sex	0.795	2947.000
ENSG00000169826	0.000	0.268	0.381	0.154	[illegible]	0.010	~p_tau+(1|manifest)+cngeneson+pc_mito+brain_region+Sex	0.424	4246.000
ENSG00000171681	0.113	0.268	0.539	-0.004	ATF7IP	0.324	~p_tau+(1|manifest)+cngeneson+pc_mito+brain_region+Sex	2.950	785.000
ENSG00000101972	0.083	0.267	0.495	0.039	STAG2	0.275	~p_tau+(1|manifest)+cngeneson+pc_mito+brain_region+Sex	1.784	1401.000
ENSG00000206199	0.040	0.267	0.586	-0.053	ANKUB1	0.186	~p_tau+(1|manifest)+cngeneson+pc_mito+brain_region+Sex	3.546	630.000
ENSG00000159023	0.057	0.266	0.475	0.058	EPB41	0.227	~p_tau+(1|manifest)+cngeneson+pc_mito+brain_region+Sex	0.767	3020.000
ENSG00000136040	0.012	0.266	0.514	0.018	PLXNC1	0.098	~p_tau+(1|manifest)+cngeneson+pc_mito+brain_region+Sex	1.596	1563.000
ENSG00000137145	0.007	0.266	0.419	0.114	DENND4C	0.076	~p_tau+(1|manifest)+cngeneson+pc_mito+brain_region+Sex	1.577	1586.000
ENSG00000155111	0.044	0.266	0.531	0.000	CDK19	0.196	~p_tau+(1|manifest)+cngeneson+pc_mito+brain_region+Sex	2.269	1090.000
ENSG00000171497	0.012	0.266	0.534	-0.002	PPID	0.101	~p_tau+(1|manifest)+cngeneson+pc_mito+brain_region+Sex	2.705	887.000
ENSG00000104765	0.426	0.265	0.728	-0.198	BNIP3L	0.669	~p_tau+(1|manifest)+cngeneson+pc_mito+brain_region+Sex	5.181	344.000
ENSG00000029725	0.005	0.265	0.446	0.085	RABEP1	0.066	~p_tau+(1|manifest)+cngeneson+pc_mito+brain_region+Sex	0.905	2663.000
ENSG00000213096	0.000	0.265	0.379	0.150	ZNF254	0.015	~p_tau+(1|manifest)+cngeneson+pc_mito+brain_region+Sex	0.327	4700.000
ENSG00000135185	0.018	0.264	0.537	-0.009	TMEM243	0.124	~p_tau+(1|manifest)+cngeneson+pc_mito+brain_region+Sex	3.968	527.000
ENSG00000107679	0.008	0.264	0.442	0.085	PLEKHA1	0.079	~p_tau+(1|manifest)+cngeneson+pc_mito+brain_region+Sex	1.030	2382.000
ENSG00000011600	0.016	0.263	0.521	0.005	TYROBP	0.117	~p_tau+(1|manifest)+cngeneson+pc_mito+brain_region+Sex	2.489	975.000
ENSG00000197183	0.049	0.263	0.533	-0.008	NOL4L	0.207	~p_tau+(1|manifest)+cngeneson+pc_mito+brain_region+Sex	1.717	1445.000
ENSG00000152767	0.379	0.263	0.666	-0.141	FARP1	0.629	~p_tau+(1|manifest)+cngeneson+pc_mito+brain_region+Sex	6.925	207.000
ENSG00000039319	0.038	0.262	0.459	0.066	ZFYVE16	0.182	~p_tau+(1|manifest)+cngeneson+pc_mito+brain_region+Sex	0.970	2497.000
ENSG00000134243	0.237	0.262	0.606	-0.081	SORT1	0.487	~p_tau+(1|manifest)+cngeneson+pc_mito+brain_region+Sex	2.915	794.000
ENSG00000213918	0.000	0.262	0.414	0.110	DNASE1	0.015	~p_tau+(1|manifest)+cngeneson+pc_mito+brain_region+Sex	0.898	2674.000
ENSG00000189283	0.002	0.262	0.396	0.128	FHIT	0.046	~p_tau+(1|manifest)+cngeneson+pc_mito+brain_region+Sex	1.239	2018.000
ENSG00000138604	0.076	0.261	0.527	-0.004	GLCE	0.262	~p_tau+(1|manifest)+cngeneson+pc_mito+brain_region+Sex	1.559	1608.000
ENSG00000047346	0.110	0.261	0.565	-0.043	FAM214A	0.318	~p_tau+(1|manifest)+cngeneson+pc_mito+brain_region+Sex	3.601	623.000
ENSG00000135535	0.001	0.261	0.449	0.073	CD164	0.035	~p_tau+(1|manifest)+cngeneson+pc_mito+brain_region+Sex	0.817	2895.000
ENSG00000128872	0.066	0.261	0.629	-0.107	TMOD2	0.244	~p_tau+(1|manifest)+cngeneson+pc_mito+brain_region+Sex	3.224	706.000
ENSG00000261371	0.122	0.261	0.534	-0.012	PECAM1	0.338	~p_tau+(1|manifest)+cngeneson+pc_mito+brain_region+Sex	2.565	946.000
ENSG00000178852	0.034	0.260	0.617	-0.098	EFCAB13	0.171	~p_tau+(1|manifest)+cngeneson+pc_mito+brain_region+Sex	3.955	534.000
ENSG00000162104	0.000	0.258	0.387	0.128	ADCY9	0.015	~p_tau+(1|manifest)+cngeneson+pc_mito+brain_region+Sex	0.462	4099.000
ENSG00000142185	0.016	0.257	0.446	0.068	TRPM2	0.117	~p_tau+(1|manifest)+cngeneson+pc_mito+brain_region+Sex	2.225	1119.000
ENSG00000080503	0.014	0.257	0.464	0.050	SMARCA2	0.110	~p_tau+(1|manifest)+cngeneson+pc_mito+brain_region+Sex	1.160	2143.000
ENSG00000170153	0.796	0.257	1.109	-0.595	RNF150	0.901	~p_tau+(1|manifest)+cngeneson+pc_mito+brain_region+Sex	10.691	96.000
ENSG00000092439	0.001	0.257	0.409	0.104	TRPM7	0.029	~p_tau+(1|manifest)+cngeneson+pc_mito+brain_region+Sex	0.869	2746.000
ENSG00000175155	0.314	0.256	0.676	-0.163	YPEL2	0.570	~p_tau+(1|manifest)+cngeneson+pc_mito+brain_region+Sex	4.387	458.000
ENSG00000153179	0.007	0.256	0.400	0.112	RASSF3	0.075	~p_tau+(1|manifest)+cngeneson+pc_mito+brain_region+Sex	1.371	1804.000
ENSG00000134884	0.197	0.255	0.577	-0.067	ARGLU1	0.439	~p_tau+(1|manifest)+cngeneson+pc_mito+brain_region+Sex	2.726	881.000
ENSG00000114166	0.159	0.255	0.535	-0.026	KAT2B	0.389	~p_tau+(1|manifest)+cngeneson+pc_mito+brain_region+Sex	1.825	1376.000
ENSG00000166888	0.008	0.255	0.440	0.069	STAT6	0.079	~p_tau+(1|manifest)+cngeneson+pc_mito+brain_region+Sex	0.714	3156.000
ENSG00000136842	0.007	0.254	0.542	-0.035	TMOD1	0.075	~p_tau+(1|manifest)+cngeneson+pc_mito+brain_region+Sex	1.922	1298.000
ENSG00000154639	0.042	0.253	0.555	-0.048	CXADR	0.192	~p_tau+(1|manifest)+cngeneson+pc_mito+brain_region+Sex	1.566	1603.000
ENSG00000102032	0.000	0.253	0.395	0.111	RENBP	0.003	~p_tau+(1|manifest)+cngeneson+pc_mito+brain_region+Sex	0.656	3333.000
ENSG00000197580	0.143	0.253	0.648	-0.142	BCO2	0.366	~p_tau+(1|manifest)+cngeneson+pc_mito+brain_region+Sex	5.247	336.000
ENSG00000162704	0.002	0.253	0.401	0.105	ARPC5	0.039	~p_tau+(1|manifest)+cngeneson+pc_mito+brain_region+Sex	0.996	2444.000
ENSG00000102804	0.000	0.253	0.391	0.115	TSC22D1	0.019	~p_tau+(1|manifest)+cngeneson+pc_mito+brain_region+Sex	1.083	2281.000
ENSG00000154134	0.019	0.253	0.469	0.036	ROBO3	0.127	~p_tau+(1|manifest)+cngeneson+pc_mito+brain_region+Sex	1.793	1393.000
ENSG00000172292	0.505	0.253	0.680	-0.175	CERS6	0.725	~p_tau+(1|manifest)+cngeneson+pc_mito+brain_region+Sex	4.490	437.000
ENSG00000196664	0.009	0.252	0.525	-0.021	TLR7	0.084	~p_tau+(1|manifest)+cngeneson+pc_mito+brain_region+Sex	1.678	1470.000
ENSG00000131773	0.424	0.252	0.748	-0.245	KHDRBS3	0.667	~p_tau+(1|manifest)+cngeneson+pc_mito+brain_region+Sex	6.635	229.000
ENSG00000174748	0.029	0.252	0.474	0.029	RPL15	0.158	~p_tau+(1|manifest)+cngeneson+pc_mito+brain_region+Sex	3.672	602.000
ENSG00000197329	0.231	0.251	0.547	-0.046	PELI1	0.480	~p_tau+(1|manifest)+cngeneson+pc_mito+brain_region+Sex	3.042	755.000
ENSG00000126777	0.073	0.250	0.513	-0.013	KTN1	0.256	~p_tau+(1|manifest)+cngeneson+pc_mito+brain_region+Sex	2.250	1101.000
ENSG00000109323	0.167	0.250	0.499	0.001	MANBA	0.400	~p_tau+(1|manifest)+cngeneson+pc_mito+brain_region+Sex	2.690	893.000
ENSG00000150540	0.109	0.250	0.597	-0.098	HNMT	0.316	~p_tau+(1|manifest)+cngeneson+pc_mito+brain_region+Sex	3.883	547.000
ENSG00000051108	0.057	0.249	0.471	0.028	HERPUD1	0.227	~p_tau+(1|manifest)+cngeneson+pc_mito+brain_region+Sex	1.800	1391.000
ENSG00000183943	0.000	0.249	0.394	0.104	PRKX	0.016	~p_tau+(1|manifest)+cngeneson+pc_mito+brain_region+Sex	1.322	1874.000
ENSG00000126005	0.020	0.248	0.499	-0.004	MMP24OS	0.130	~p_tau+(1|manifest)+cngeneson+pc_mito+brain_region+Sex	1.506	1657.000
ENSG00000087086	0.631	0.247	0.762	-0.267	FTL	0.806	~p_tau+(1|manifest)+cngeneson+pc_mito+brain_region+Sex	16.304	37.000
ENSG00000169564	0.015	0.247	0.500	-0.005	PCBP1	0.114	~p_tau+(1|manifest)+cngeneson+pc_mito+brain_region+Sex	1.698	1455.000
ENSG00000119729	0.028	0.247	0.459	0.035	RHOQ	0.153	~p_tau+(1|manifest)+cngeneson+pc_mito+brain_region+Sex	1.095	2266.000
ENSG00000118260	0.024	0.247	0.444	0.049	CREB1	0.141	~p_tau+(1|manifest)+cngeneson+pc_mito+brain_region+Sex	0.886	2698.000
ENSG00000105810	0.397	0.246	0.594	-0.102	CDK6	0.644	~p_tau+(1|manifest)+cngeneson+pc_mito+brain_region+Sex	2.131	1164.000
ENSG00000158411	0.002	0.246	0.425	0.067	MITD1	0.039	~p_tau+(1|manifest)+cngeneson+pc_mito+brain_region+Sex	0.968	2505.000
ENSG00000162390	0.029	0.246	0.466	0.026	ACOT11	0.158	~p_tau+(1|manifest)+cngeneson+pc_mito+brain_region+Sex	1.828	1372.000
ENSG00000157985	0.014	0.246	0.462	0.030	AGAP1	0.106	~p_tau+(1|manifest)+cngeneson+pc_mito+brain_region+Sex	1.364	1815.000
ENSG00000088205	0.009	0.246	0.403	0.089	DDX18	0.085	~p_tau+(1|manifest)+cngeneson+pc_mito+brain_region+Sex	0.703	3198.000
ENSG00000165801	0.001	0.246	0.394	0.097	ARHGEF40	0.028	~p_tau+(1|manifest)+cngeneson+pc_mito+brain_region+Sex	0.647	3370.000
ENSG00000134480	0.407	0.245	0.637	-0.146	CCNH	0.654	~p_tau+(1|manifest)+cngeneson+pc_mito+brain_region+Sex	8.255	150.000
ENSG00000196323	0.009	0.245	0.471	0.019	ZBTB44	0.087	~p_tau+(1|manifest)+cngeneson+pc_mito+brain_region+Sex	1.611	1541.000
ENSG00000133104	0.000	0.245	0.406	0.084	SPART	0.018	~p_tau+(1|manifest)+cngeneson+pc_mito+brain_region+Sex	0.805	2925.000
ENSG00000048392	0.002	0.244	0.383	0.105	RRM2B	0.038	~p_tau+(1|manifest)+cngeneson+pc_mito+brain_region+Sex	0.481	4020.000
ENSG00000043143	0.020	0.244	0.477	0.011	JADE2	0.132	~p_tau+(1|manifest)+cngeneson+pc_mito+brain_region+Sex	1.674	1474.000
ENSG00000158467	0.003	0.244	0.366	0.122	AHCYL2	0.050	~p_tau+(1|manifest)+cngeneson+pc_mito+brain_region+Sex	0.190	5267.000
ENSG00000125633	0.003	0.244	0.412	0.075	CCDC93	0.052	~p_tau+(1|manifest)+cngeneson+pc_mito+brain_region+Sex	0.619	3466.000
ENSG00000089916	0.122	0.243	0.507	-0.022	GPATCH2L	0.338	~p_tau+(1|manifest)+cngeneson+pc_mito+brain_region+Sex	0.882	2707.000
ENSG00000147679	0.005	0.242	0.489	-0.004	UTP23	0.063	~p_tau+(1|manifest)+cngeneson+pc_mito+brain_region+Sex	3.208	709.000
ENSG00000085662	0.001	0.242	0.419	0.066	AKR1B1	0.032	~p_tau+(1|manifest)+cngeneson+pc_mito+brain_region+Sex	0.603	3522.000
ENSG00000164674	0.337	0.242	0.582	-0.098	SYTL3	0.593	~p_tau+(1|manifest)+cngeneson+pc_mito+brain_region+Sex	4.330	467.000
ENSG00000100591	0.006	0.242	0.503	-0.020	AHSA1	0.073	~p_tau+(1|manifest)+cngeneson+pc_mito+brain_region+Sex	1.445	1731.000
ENSG00000003402	0.099	0.241	0.527	-0.045	CFLAR	0.300	~p_tau+(1|manifest)+cngeneson+pc_mito+brain_region+Sex	2.779	853.000
ENSG00000169891	0.003	0.240	0.414	0.066	REPS2	0.055	~p_tau+(1|manifest)+cngeneson+pc_mito+brain_region+Sex	1.255	1988.000
ENSG00000106034	0.603	0.239	0.789	-0.311	CPED1	0.788	~p_tau+(1|manifest)+cngeneson+pc_mito+brain_region+Sex	21.519	21.000
ENSG00000174718	0.053	0.239	0.555	-0.077	RESF1	0.218	~p_tau+(1|manifest)+cngeneson+pc_mito+brain_region+Sex	2.194	1131.000
ENSG00000087250	0.013	0.239	0.407	0.071	MT3	0.106	~p_tau+(1|manifest)+cngeneson+pc_mito+brain_region+Sex	1.274	1957.000
ENSG00000148660	0.005	0.239	0.398	0.079	CAMK2G	0.064	~p_tau+(1|manifest)+cngeneson+pc_mito+brain_region+Sex	0.982	2467.000
ENSG00000135018	0.024	0.238	0.498	-0.021	UBQLN1	0.142	~p_tau+(1|manifest)+cngeneson+pc_mito+brain_region+Sex	1.304	1903.000
ENSG00000088970	0.043	0.238	0.449	0.028	KIZ	0.194	~p_tau+(1|manifest)+cngeneson+pc_mito+brain_region+Sex	2.050	1215.000
ENSG00000166501	0.063	0.238	0.423	0.053	PRKCB	0.239	~p_tau+(1|manifest)+cngeneson+pc_mito+brain_region+Sex	1.755	1416.000
ENSG00000113580	0.126	0.237	0.486	-0.011	NR3C1	0.343	~p_tau+(1|manifest)+cngeneson+pc_mito+brain_region+Sex	1.307	1899.000
ENSG00000071967	0.029	0.237	0.454	0.020	CYBRD1	0.157	~p_tau+(1|manifest)+cngeneson+pc_mito+brain_region+Sex	1.471	1693.000
ENSG00000151923	0.000	0.236	0.358	0.114	TIAL1	0.015	~p_tau+(1|manifest)+cngeneson+pc_mito+brain_region+Sex	0.382	4435.000
ENSG00000130584	0.013	0.236	0.437	0.034	ZBTB46	0.102	~p_tau+(1|manifest)+cngeneson+pc_mito+brain_region+Sex	1.190	2095.000
ENSG00000118257	0.028	0.236	0.427	0.044	NRP2	0.154	~p_tau+(1|manifest)+cngeneson+pc_mito+brain_region+Sex	0.561	3679.000
ENSG00000172456	0.077	0.235	0.481	-0.010	FGGY	0.264	~p_tau+(1|manifest)+cngeneson+pc_mito+brain_region+Sex	1.349	1833.000
ENSG00000175782	0.018	0.235	0.451	0.018	SLC35E3	0.123	~p_tau+(1|manifest)+cngeneson+pc_mito+brain_region+Sex	1.164	2136.000
ENSG00000138767	0.125	0.235	0.486	-0.017	CNOT6L	0.342	~p_tau+(1|manifest)+cngeneson+pc_mito+brain_region+Sex	2.094	1192.000
ENSG00000136048	0.004	0.235	0.412	0.057	DRAM1	0.062	~p_tau+(1|manifest)+cngeneson+pc_mito+brain_region+Sex	0.237	5122.000
ENSG00000157617	0.002	0.234	0.396	0.072	C2CD2	0.039	~p_tau+(1|manifest)+cngeneson+pc_mito+brain_region+Sex	0.545	3746.000
ENSG00000163406	0.332	0.233	0.715	-0.248	SLC15A2	0.589	~p_tau+(1|manifest)+cngeneson+pc_mito+brain_region+Sex	6.911	209.000
ENSG00000149311	0.015	0.233	0.382	0.085	ATM	0.110	~p_tau+(1|manifest)+cngeneson+pc_mito+brain_region+Sex	5.607	307.000
ENSG00000074054	0.063	0.233	0.463	0.004	CLASP1	0.240	~p_tau+(1|manifest)+cngeneson+pc_mito+brain_region+Sex	1.838	1359.000
ENSG00000128881	0.007	0.232	0.412	0.053	TTBK2	0.075	~p_tau+(1|manifest)+cngeneson+pc_mito+brain_region+Sex	0.640	3393.000
ENSG00000175029	0.113	0.232	0.442	0.022	CTBP2	0.323	~p_tau+(1|manifest)+cngeneson+pc_mito+brain_region+Sex	2.240	1107.000
ENSG00000196605	0.588	0.232	0.685	-0.221	ZNF846	0.780	~p_tau+(1|manifest)+cngeneson+pc_mito+brain_region+Sex	5.937	285.000
ENSG00000151240	0.006	0.231	0.429	0.034	DIP2C	0.073	~p_tau+(1|manifest)+cngeneson+pc_mito+brain_region+Sex	0.420	4267.000
ENSG00000158869	0.112	0.231	0.571	-0.108	FCER1G	0.322	~p_tau+(1|manifest)+cngeneson+pc_mito+brain_region+Sex	3.278	692.000
ENSG00000131669	0.011	0.231	0.430	0.032	NINJ1	0.093	~p_tau+(1|manifest)+cngeneson+pc_mito+brain_region+Sex	1.445	1730.000
ENSG00000079335	0.079	0.231	0.429	0.033	CDC14A	0.266	~p_tau+(1|manifest)+cngeneson+pc_mito+brain_region+Sex	1.247	2007.000
ENSG00000169045	0.008	0.231	0.438	0.024	HNRNPH1	0.080	~p_tau+(1|manifest)+cngeneson+pc_mito+brain_region+Sex	3.857	556.000
ENSG00000120868	0.050	0.231	0.446	0.015	APAF1	0.210	~p_tau+(1|manifest)+cngeneson+pc_mito+brain_region+Sex	0.719	3141.000
ENSG00000152953	0.304	0.230	0.530	-0.069	STK32B	0.560	~p_tau+(1|manifest)+cngeneson+pc_mito+brain_region+Sex	1.296	1920.000
ENSG00000075413	0.002	0.230	0.349	0.112	MARK3	0.038	~p_tau+(1|manifest)+cngeneson+pc_mito+brain_region+Sex	0.408	4326.000
ENSG00000100664	0.011	0.230	0.432	0.028	EIF5	0.094	~p_tau+(1|manifest)+cngeneson+pc_mito+brain_region+Sex	1.008	2419.000
ENSG00000130475	0.007	0.230	0.423	0.038	FCHO1	0.075	~p_tau+(1|manifest)+cngeneson+pc_mito+brain_region+Sex	0.667	3303.000
ENSG00000215301	0.012	0.230	0.402	0.059	DDX3X	0.098	~p_tau+(1|manifest)+cngeneson+pc_mito+brain_region+Sex	1.914	1303.000
ENSG00000155962	0.028	0.230	0.495	-0.035	CLIC2	0.155	~p_tau+(1|manifest)+cngeneson+pc_mito+brain_region+Sex	11.507	84.000
ENSG00000173812	0.231	0.230	0.559	-0.100	EIF1	0.480	~p_tau+(1|manifest)+cngeneson+pc_mito+brain_region+Sex	3.100	735.000
ENSG00000189403	0.078	0.229	0.519	-0.060	HMGB1	0.266	~p_tau+(1|manifest)+cngeneson+pc_mito+brain_region+Sex	2.757	867.000
ENSG00000134250	0.046	0.229	0.415	0.043	NOTCH2	0.201	~p_tau+(1|manifest)+cngeneson+pc_mito+brain_region+Sex	1.098	2255.000
ENSG00000116044	0.001	0.229	0.402	0.056	NFE2L2	0.036	~p_tau+(1|manifest)+cngeneson+pc_mito+brain_region+Sex	0.499	3938.000
ENSG00000250312	0.271	0.228	0.580	-0.125	ZNF718	0.525	~p_tau+(1|manifest)+cngeneson+pc_mito+brain_region+Sex	2.584	938.000
ENSG00000115107	0.037	0.227	0.454	0.001	STEAP3	0.180	~p_tau+(1|manifest)+cngeneson+pc_mito+brain_region+Sex	1.808	1386.000
ENSG00000169403	0.071	0.227	0.474	-0.020	PTAFR	0.251	~p_tau+(1|manifest)+cngeneson+pc_mito+brain_region+Sex	1.640	1512.000
ENSG00000178996	0.023	0.227	0.476	-0.022	SNX18	0.141	~p_tau+(1|manifest)+cngeneson+pc_mito+brain_region+Sex	1.307	1900.000
ENSG00000153094	0.002	0.227	0.401	0.053	BCL2L11	0.040	~p_tau+(1|manifest)+cngeneson+pc_mito+brain_region+Sex	1.143	2173.000
ENSG00000118816	0.001	0.227	0.407	0.046	CCNI	0.031	~p_tau+(1|manifest)+cngeneson+pc_mito+brain_region+Sex	0.693	3225.000
ENSG00000169967	0.092	0.227	0.445	0.009	MAP3K2	0.289	~p_tau+(1|manifest)+cngeneson+pc_mito+brain_region+Sex	1.742	1422.000
ENSG00000149289	0.068	0.226	0.440	0.012	ZC3H12C	0.246	~p_tau+(1|manifest)+cngeneson+pc_mito+brain_region+Sex	0.912	2646.000
ENSG00000198130	0.564	0.225	0.671	-0.222	HIBCH	0.764	~p_tau+(1|manifest)+cngeneson+pc_mito+brain_region+Sex	7.566	183.000
ENSG00000132424	0.071	0.224	0.479	-0.031	PNISR	0.251	~p_tau+(1|manifest)+cngeneson+pc_mito+brain_region+Sex	4.674	409.000
ENSG00000138443	0.004	0.223	0.384	0.063	ABI2	0.057	~p_tau+(1|manifest)+cngeneson+pc_mito+brain_region+Sex	0.889	2693.000
ENSG00000105223	0.039	0.222	0.403	0.042	PLD3	0.185	~p_tau+(1|manifest)+cngeneson+pc_mito+brain_region+Sex	1.888	1320.000
ENSG00000161016	0.377	0.222	0.668	-0.224	RPL8	0.628	~p_tau+(1|manifest)+cngeneson+pc_mito+brain_region+Sex	4.321	470.000
ENSG00000118263	0.164	0.222	0.453	-0.009	KLF7	0.395	~p_tau+(1|manifest)+cngeneson+pc_mito+brain_region+Sex	0.934	2582.000
ENSG00000121281	0.007	0.222	0.365	0.079	ADCY7	0.077	~p_tau+(1|manifest)+cngeneson+pc_mito+brain_region+Sex	1.274	1959.000
ENSG00000141519	0.007	0.221	0.478	-0.035	CCDC40	0.074	~p_tau+(1|manifest)+cngeneson+pc_mito+brain_region+Sex	1.723	1437.000
ENSG00000144381	0.195	0.221	0.605	-0.162	HSPD1	0.437	~p_tau+(1|manifest)+cngeneson+pc_mito+brain_region+Sex	5.146	348.000
ENSG00000131724	0.313	0.221	0.597	-0.155	IL13RA1	0.569	~p_tau+(1|manifest)+cngeneson+pc_mito+brain_region+Sex	4.511	432.000
ENSG00000047648	0.446	0.221	0.553	-0.111	ARHGAP6	0.683	~p_tau+(1|manifest)+cngeneson+pc_mito+brain_region+Sex	3.159	721.000
ENSG00000150760	0.025	0.221	0.427	0.015	DOCK1	0.147	~p_tau+(1|manifest)+cngeneson+pc_mito+brain_region+Sex	1.216	2057.000

ENSG00000146278	0.015	0.221	0.456	-0.015	PNRC1	0.113	~p_tau+(1|manifest)+cngeneson+pc_mito+brain_region+Sex	1.945	1281.000
ENSG00000214655	0.002	0.220	0.363	0.077	ZSWIM8	0.044	~p_tau+(1|manifest)+cngeneson+pc_mito+brain_region+Sex	1.122	2218.000
ENSG00000148180	0.212	0.220	0.538	-0.098	GSN	0.455	~p_tau+(1|manifest)+cngeneson+pc_mito+brain_region+Sex	3.505	636.000
ENSG00000108854	0.002	0.220	0.338	0.102	SMURF2	0.040	~p_tau+(1|manifest)+cngeneson+pc_mito+brain_region+Sex	0.895	2681.000
ENSG00000138814	0.351	0.220	0.509	-0.069	PPP3CA	0.605	~p_tau+(1|manifest)+cngeneson+pc_mito+brain_region+Sex	3.788	569.000
ENSG00000131375	0.007	0.220	0.384	0.056	CAPN7	0.076	~p_tau+(1|manifest)+cngeneson+pc_mito+brain_region+Sex	0.949	2546.000
ENSG00000148429	0.308	0.219	0.507	-0.069	USP6NL	0.564	~p_tau+(1|manifest)+cngeneson+pc_mito+brain_region+Sex	4.443	446.000
ENSG00000135976	0.019	0.219	0.367	0.071	ANKRD36	0.125	~p_tau+(1|manifest)+cngeneson+pc_mito+brain_region+Sex	0.726	3124.000
ENSG00000048471	0.097	0.219	0.411	0.027	SNX29	0.296	~p_tau+(1|manifest)+cngeneson+pc_mito+brain_region+Sex	5.271	333.000
ENSG00000168036	0.109	0.219	0.454	-0.017	CTNNB1	0.316	~p_tau+(1|manifest)+cngeneson+pc_mito+brain_region+Sex	0.929	2592.000
ENSG00000165209	0.149	0.218	0.454	-0.017	STRBP	0.375	~p_tau+(1|manifest)+cngeneson+pc_mito+brain_region+Sex	1.300	1913.000
ENSG00000109184	0.001	0.218	0.366	0.071	DCUN1D4	0.033	~p_tau+(1|manifest)+cngeneson+pc_mito+brain_region+Sex	0.732	3109.000
ENSG00000171791	0.169	0.218	0.438	-0.002	BCL2	0.401	~p_tau+(1|manifest)+cngeneson+pc_mito+brain_region+Sex	0.944	2560.000
ENSG00000163320	0.066	0.218	0.424	0.012	CGGBP1	0.244	~p_tau+(1|manifest)+cngeneson+pc_mito+brain_region+Sex	1.017	2401.000
ENSG00000117450	0.001	0.218	0.366	0.069	PRDX1	0.031	~p_tau+(1|manifest)+cngeneson+pc_mito+brain_region+Sex	0.647	3371.000
ENSG00000162434	0.062	0.217	0.438	-0.004	JAK1	0.238	~p_tau+(1|manifest)+cngeneson+pc_mito+brain_region+Sex	0.971	2494.000
ENSG00000124570	0.075	0.217	0.454	-0.021	SERPINB6	0.261	~p_tau+(1|manifest)+cngeneson+pc_mito+brain_region+Sex	2.759	866.000
ENSG00000164124	0.139	0.216	0.472	-0.039	TMEM144	0.362	~p_tau+(1|manifest)+cngeneson+pc_mito+brain_region+Sex	2.382	1027.000
ENSG00000173083	0.100	0.216	0.480	-0.047	HPSE	0.301	~p_tau+(1|manifest)+cngeneson+pc_mito+brain_region+Sex	3.343	672.000
ENSG00000178177	0.220	0.216	0.453	-0.021	LCORL	0.465	~p_tau+(1|manifest)+cngeneson+pc_mito+brain_region+Sex	1.738	1424.000
ENSG00000184677	0.000	0.216	0.354	0.078	ZBTB40	0.015	~p_tau+(1|manifest)+cngeneson+pc_mito+brain_region+Sex	0.538	3771.000
ENSG00000112624	0.142	0.216	0.459	-0.027	BICRAL	0.366	~p_tau+(1|manifest)+cngeneson+pc_mito+brain_region+Sex	1.180	2109.000
ENSG00000163116	0.150	0.216	0.534	-0.103	STPG2	0.377	~p_tau+(1|manifest)+cngeneson+pc_mito+brain_region+Sex	2.608	925.000
ENSG00000138385	0.001	0.215	0.337	0.094	SSB	0.028	~p_tau+(1|manifest)+cngeneson+pc_mito+brain_region+Sex	0.291	4864.000
ENSG00000122547	0.203	0.215	0.470	-0.040	EEPD1	0.446	~p_tau+(1|manifest)+cngeneson+pc_mito+brain_region+Sex	4.441	448.000
ENSG00000214013	0.005	0.215	0.334	0.096	GANC	0.066	~p_tau+(1|manifest)+cngeneson+pc_mito+brain_region+Sex	0.263	5013.000
ENSG00000151491	0.411	0.215	0.539	-0.109	EPS8	0.656	~p_tau+(1|manifest)+cngeneson+pc_mito+brain_region+Sex	2.482	977.000
ENSG00000075240	0.006	0.215	0.391	0.039	GRAMD4	0.073	~p_tau+(1|manifest)+cngeneson+pc_mito+brain_region+Sex	1.547	1613.000
ENSG00000075420	0.511	0.215	0.578	-0.148	FNDC3B	0.730	~p_tau+(1|manifest)+cngeneson+pc_mito+brain_region+Sex	8.478	141.000
ENSG00000064012	0.065	0.215	0.462	-0.033	CASP8	0.243	~p_tau+(1|manifest)+cngeneson+pc_mito+brain_region+Sex	0.823	2881.000
ENSG00000067141	0.005	0.214	0.355	0.073	NEO1	0.064	~p_tau+(1|manifest)+cngeneson+pc_mito+brain_region+Sex	1.058	2332.000
ENSG00000166272	0.004	0.214	0.347	0.081	WBP1L	0.062	~p_tau+(1|manifest)+cngeneson+pc_mito+brain_region+Sex	0.449	4147.000
ENSG00000113851	0.005	0.214	0.390	0.037	CRBN	0.068	~p_tau+(1|manifest)+cngeneson+pc_mito+brain_region+Sex	1.181	2107.000
ENSG00000091039	0.143	0.213	0.445	-0.018	OSBPL8	0.367	~p_tau+(1|manifest)+cngeneson+pc_mito+brain_region+Sex	1.518	1643.000
ENSG00000133789	0.132	0.213	0.429	-0.003	SWAP70	0.351	~p_tau+(1|manifest)+cngeneson+pc_mito+brain_region+Sex	1.833	1366.000
ENSG00000188846	0.282	0.213	0.547	-0.120	RPL14	0.535	~p_tau+(1|manifest)+cngeneson+pc_mito+brain_region+Sex	3.037	758.000
ENSG00000105856	0.303	0.213	0.567	-0.141	HBP1	0.559	~p_tau+(1|manifest)+cngeneson+pc_mito+brain_region+Sex	5.082	359.000
ENSG00000172243	0.428	0.213	0.541	-0.116	CLEC7A	0.669	~p_tau+(1|manifest)+cngeneson+pc_mito+brain_region+Sex	2.962	783.000
ENSG00000131507	0.016	0.213	0.403	0.022	NDFIP1	0.114	~p_tau+(1|manifest)+cngeneson+pc_mito+brain_region+Sex	1.300	1911.000
ENSG00000143198	0.006	0.213	0.399	0.026	MGST3	0.072	~p_tau+(1|manifest)+cngeneson+pc_mito+brain_region+Sex	1.630	1524.000
ENSG00000100207	0.004	0.213	0.362	0.063	TCF20	0.058	~p_tau+(1|manifest)+cngeneson+pc_mito+brain_region+Sex	0.564	3667.000
ENSG00000104852	0.262	0.212	0.534	-0.111	SNRNP70	0.516	~p_tau+(1|manifest)+cngeneson+pc_mito+brain_region+Sex	2.841	822.000
ENSG00000092931	0.056	0.211	0.414	0.008	MFSD11	0.224	~p_tau+(1|manifest)+cngeneson+pc_mito+brain_region+Sex	1.279	1950.000
ENSG00000168566	0.005	0.211	0.372	0.049	SNRNP48	0.067	~p_tau+(1|manifest)+cngeneson+pc_mito+brain_region+Sex	0.735	3099.000
ENSG00000090661	0.002	0.211	0.320	0.101	CERS4	0.038	~p_tau+(1|manifest)+cngeneson+pc_mito+brain_region+Sex	0.966	2510.000
ENSG00000157388	0.727	0.210	0.737	-0.317	CACNA1D	0.863	~p_tau+(1|manifest)+cngeneson+pc_mito+brain_region+Sex	5.102	356.000
ENSG00000057657	0.131	0.210	0.513	-0.094	PRDM1	0.350	~p_tau+(1|manifest)+cngeneson+pc_mito+brain_region+Sex	2.285	1081.000
ENSG00000035862	0.109	0.209	0.407	0.011	TIMP2	0.317	~p_tau+(1|manifest)+cngeneson+pc_mito+brain_region+Sex	1.159	2145.000
ENSG00000136404	0.257	0.208	0.503	-0.087	TM6SF1	0.511	~p_tau+(1|manifest)+cngeneson+pc_mito+brain_region+Sex	1.092	2271.000
ENSG00000004399	0.009	0.207	0.404	0.010	PLXND1	0.084	~p_tau+(1|manifest)+cngeneson+pc_mito+brain_region+Sex	0.946	2553.000
ENSG00000113719	0.069	0.207	0.436	-0.023	ERGIC1	0.247	~p_tau+(1|manifest)+cngeneson+pc_mito+brain_region+Sex	1.277	1954.000
ENSG00000160305	0.477	0.207	0.603	-0.190	DIP2A	0.704	~p_tau+(1|manifest)+cngeneson+pc_mito+brain_region+Sex	4.929	376.000
ENSG00000188921	0.130	0.206	0.514	-0.101	HACD4	0.349	~p_tau+(1|manifest)+cngeneson+pc_mito+brain_region+Sex	3.294	686.000
ENSG00000185862	0.005	0.206	0.401	0.011	EVI2B	0.066	~p_tau+(1|manifest)+cngeneson+pc_mito+brain_region+Sex	1.132	2191.000
ENSG00000109475	0.060	0.206	0.488	-0.076	RPL34	0.234	~p_tau+(1|manifest)+cngeneson+pc_mito+brain_region+Sex	2.652	911.000
ENSG00000068878	0.003	0.205	0.365	0.046	PSME4	0.054	~p_tau+(1|manifest)+cngeneson+pc_mito+brain_region+Sex	0.436	4191.000
ENSG00000121741	0.151	0.205	0.421	-0.011	ZMYM2	0.379	~p_tau+(1|manifest)+cngeneson+pc_mito+brain_region+Sex	1.649	1500.000
ENSG00000105366	0.117	0.205	0.525	-0.115	SIGLEC8	0.332	~p_tau+(1|manifest)+cngeneson+pc_mito+brain_region+Sex	3.094	737.000
ENSG00000136051	0.157	0.205	0.433	-0.023	WASHC4	0.385	~p_tau+(1|manifest)+cngeneson+pc_mito+brain_region+Sex	2.511	965.000
ENSG00000171587	0.685	0.204	0.827	-0.418	DSCAM	0.841	~p_tau+(1|manifest)+cngeneson+pc_mito+brain_region+Sex	8.566	137.000
ENSG00000150753	0.008	0.204	0.436	-0.028	CCT5	0.080	~p_tau+(1|manifest)+cngeneson+pc_mito+brain_region+Sex	1.247	2006.000
ENSG00000179833	0.119	0.204	0.418	-0.010	SERTAD2	0.334	~p_tau+(1|manifest)+cngeneson+pc_mito+brain_region+Sex	1.274	1958.000
ENSG00000065923	0.030	0.204	0.365	0.043	SLC9A7	0.159	~p_tau+(1|manifest)+cngeneson+pc_mito+brain_region+Sex	0.706	3188.000
ENSG00000130066	0.558	0.204	0.601	-0.194	SAT1	0.761	~p_tau+(1|manifest)+cngeneson+pc_mito+brain_region+Sex	17.863	32.000
ENSG00000137764	0.188	0.203	0.508	-0.102	MAP2K5	0.427	~p_tau+(1|manifest)+cngeneson+pc_mito+brain_region+Sex	1.945	1278.000
ENSG00000107890	0.019	0.203	0.343	0.063	ANKRD26	0.127	~p_tau+(1|manifest)+cngeneson+pc_mito+brain_region+Sex	0.574	3635.000
ENSG00000042493	0.000	0.203	0.349	0.056	CAPG	0.013	~p_tau+(1|manifest)+cngeneson+pc_mito+brain_region+Sex	1.775	1404.000
ENSG00000115484	0.140	0.202	0.526	-0.121	CCT4	0.363	~p_tau+(1|manifest)+cngeneson+pc_mito+brain_region+Sex	2.783	851.000
ENSG00000112851	0.057	0.202	0.364	0.041	ERBIN	0.227	~p_tau+(1|manifest)+cngeneson+pc_mito+brain_region+Sex	0.944	2559.000
ENSG00000134508	0.053	0.202	0.395	0.009	CABLES1	0.218	~p_tau+(1|manifest)+cngeneson+pc_mito+brain_region+Sex	0.710	3172.000
ENSG00000123106	0.222	0.202	0.422	-0.018	CCDC91	0.468	~p_tau+(1|manifest)+cngeneson+pc_mito+brain_region+Sex	1.918	1301.000
ENSG00000153551	0.064	0.202	0.412	-0.008	CMTM7	0.242	~p_tau+(1|manifest)+cngeneson+pc_mito+brain_region+Sex	1.073	2303.000
ENSG00000130396	0.003	0.202	0.365	0.039	AFDN	0.049	~p_tau+(1|manifest)+cngeneson+pc_mito+brain_region+Sex	1.277	1955.000
ENSG00000143669	0.267	0.202	0.466	-0.063	LYST	0.521	~p_tau+(1|manifest)+cngeneson+pc_mito+brain_region+Sex	3.925	541.000
ENSG00000099282	0.034	0.202	0.398	0.005	TSPAN15	0.171	~p_tau+(1|manifest)+cngeneson+pc_mito+brain_region+Sex	1.633	1521.000
ENSG00000168961	0.109	0.202	0.434	-0.031	LGALS9	0.317	~p_tau+(1|manifest)+cngeneson+pc_mito+brain_region+Sex	1.606	1550.000
ENSG00000204103	0.231	0.201	0.483	-0.080	MAFB	0.480	~p_tau+(1|manifest)+cngeneson+pc_mito+brain_region+Sex	4.505	434.000
ENSG00000169249	0.000	0.201	0.286	0.116	ZRSR2	0.015	~p_tau+(1|manifest)+cngeneson+pc_mito+brain_region+Sex	0.471	4057.000
ENSG00000197959	0.196	0.201	0.438	-0.036	DNM3	0.438	~p_tau+(1|manifest)+cngeneson+pc_mito+brain_region+Sex	2.128	1166.000
ENSG00000197601	0.038	0.200	0.380	0.021	FAR1	0.182	~p_tau+(1|manifest)+cngeneson+pc_mito+brain_region+Sex	1.501	1664.000
ENSG00000168906	0.159	0.200	0.454	-0.053	MAT2A	0.388	~p_tau+(1|manifest)+cngeneson+pc_mito+brain_region+Sex	1.503	1660.000
ENSG00000135473	0.003	0.200	0.354	0.046	PAN2	0.055	~p_tau+(1|manifest)+cngeneson+pc_mito+brain_region+Sex	1.418	1758.000
ENSG00000006652	0.003	0.200	0.313	0.086	IFRD1	0.055	~p_tau+(1|manifest)+cngeneson+pc_mito+brain_region+Sex	0.709	3175.000
ENSG00000203880	0.003	0.199	0.361	0.037	PCMTD2	0.054	~p_tau+(1|manifest)+cngeneson+pc_mito+brain_region+Sex	1.081	2288.000
ENSG00000151229	0.365	0.199	0.495	-0.097	SLC2A13	0.618	~p_tau+(1|manifest)+cngeneson+pc_mito+brain_region+Sex	1.419	1756.000
ENSG00000120438	0.001	0.199	0.403	-0.005	TCP1	0.033	~p_tau+(1|manifest)+cngeneson+pc_mito+brain_region+Sex	1.579	1583.000
ENSG00000006747	0.544	0.199	0.710	-0.312	SCIN	0.751	~p_tau+(1|manifest)+cngeneson+pc_mito+brain_region+Sex	9.071	126.000
ENSG00000062194	0.090	0.198	0.460	-0.064	GPBP1	0.285	~p_tau+(1|manifest)+cngeneson+pc_mito+brain_region+Sex	2.176	1142.000
ENSG00000168397	0.001	0.198	0.322	0.074	ATG4B	0.037	~p_tau+(1|manifest)+cngeneson+pc_mito+brain_region+Sex	1.077	2292.000
ENSG00000074416	0.077	0.198	0.448	-0.052	MGLL	0.264	~p_tau+(1|manifest)+cngeneson+pc_mito+brain_region+Sex	2.082	1196.000
ENSG00000134283	0.102	0.198	0.388	0.009	PPHLN1	0.305	~p_tau+(1|manifest)+cngeneson+pc_mito+brain_region+Sex	0.815	2902.000
ENSG00000143891	0.016	0.198	0.339	0.057	GALM	0.116	~p_tau+(1|manifest)+cngeneson+pc_mito+brain_region+Sex	1.075	2296.000
ENSG00000106479	0.023	0.198	0.406	-0.010	ZNF862	0.141	~p_tau+(1|manifest)+cngeneson+pc_mito+brain_region+Sex	1.735	1427.000
ENSG00000131080	0.036	0.198	0.395	0.000	EDA2R	0.178	~p_tau+(1|manifest)+cngeneson+pc_mito+brain_region+Sex	1.691	1461.000
ENSG00000087589	0.018	0.197	0.379	0.015	CASS4	0.124	~p_tau+(1|manifest)+cngeneson+pc_mito+brain_region+Sex	0.834	2844.000
ENSG00000136044	0.546	0.197	0.547	-0.152	APPL2	0.753	~p_tau+(1|manifest)+cngeneson+pc_mito+brain_region+Sex	4.478	439.000
ENSG00000110876	0.462	0.197	0.555	-0.161	SELPLG	0.694	~p_tau+(1|manifest)+cngeneson+pc_mito+brain_region+Sex	4.571	421.000
ENSG00000104907	0.044	0.197	0.433	-0.039	TRMT1	0.197	~p_tau+(1|manifest)+cngeneson+pc_mito+brain_region+Sex	1.789	1395.000
ENSG00000024048	0.030	0.196	0.358	0.035	UBR2	0.161	~p_tau+(1|manifest)+cngeneson+pc_mito+brain_region+Sex	1.009	2416.000
ENSG00000008256	0.168	0.196	0.458	-0.067	CYTH3	0.401	~p_tau+(1|manifest)+cngeneson+pc_mito+brain_region+Sex	1.521	1635.000
ENSG00000137818	0.387	0.196	0.635	-0.243	RPLP1	0.636	~p_tau+(1|manifest)+cngeneson+pc_mito+brain_region+Sex	4.709	403.000
ENSG00000240230	0.000	0.195	0.329	0.062	COX19	0.015	~p_tau+(1|manifest)+cngeneson+pc_mito+brain_region+Sex	0.610	3495.000
ENSG00000198771	0.353	0.195	0.458	-0.067	RCSD1	0.608	~p_tau+(1|manifest)+cngeneson+pc_mito+brain_region+Sex	3.087	743.000
ENSG00000204977	0.033	0.195	0.389	0.001	TRIM13	0.168	~p_tau+(1|manifest)+cngeneson+pc_mito+brain_region+Sex	1.301	1909.000
ENSG00000117266	0.005	0.195	0.338	0.052	CDK18	0.066	~p_tau+(1|manifest)+cngeneson+pc_mito+brain_region+Sex	0.798	2937.000
ENSG00000073060	0.076	0.195	0.432	-0.043	SCARB1	0.262	~p_tau+(1|manifest)+cngeneson+pc_mito+brain_region+Sex	1.344	1840.000
ENSG00000196531	0.364	0.194	0.526	-0.138	NACA	0.617	~p_tau+(1|manifest)+cngeneson+pc_mito+brain_region+Sex	2.846	820.000
ENSG00000140396	0.269	0.194	0.431	-0.043	NCOA2	0.523	~p_tau+(1|manifest)+cngeneson+pc_mito+brain_region+Sex	3.242	700.000
ENSG00000065060	0.000	0.194	0.321	0.066	UHRF1BP1	0.016	~p_tau+(1|manifest)+cngeneson+pc_mito+brain_region+Sex	0.363	4507.000
ENSG00000167526	0.440	0.194	0.573	-0.185	RPL13	0.680	~p_tau+(1|manifest)+cngeneson+pc_mito+brain_region+Sex	4.226	483.000
ENSG00000132669	0.383	0.194	0.537	-0.150	RIN2	0.632	~p_tau+(1|manifest)+cngeneson+pc_mito+brain_region+Sex	4.048	514.000
ENSG00000143772	0.056	0.194	0.356	0.031	ITPKB	0.225	~p_tau+(1|manifest)+cngeneson+pc_mito+brain_region+Sex	0.720	3140.000
ENSG00000185946	0.025	0.193	0.368	0.018	RNPC3	0.145	~p_tau+(1|manifest)+cngeneson+pc_mito+brain_region+Sex	0.742	3077.000
ENSG00000063177	0.218	0.193	0.561	-0.175	RPL18	0.463	~p_tau+(1|manifest)+cngeneson+pc_mito+brain_region+Sex	3.766	572.000
ENSG00000108298	0.663	0.193	0.621	-0.236	RPL19	0.827	~p_tau+(1|manifest)+cngeneson+pc_mito+brain_region+Sex	8.036	159.000
ENSG00000197555	0.863	0.192	0.951	-0.567	SIPA1L1	0.938	~p_tau+(1|manifest)+cngeneson+pc_mito+brain_region+Sex	22.341	18.000
ENSG00000110852	0.306	0.191	0.454	-0.072	CLEC2B	0.562	~p_tau+(1|manifest)+cngeneson+pc_mito+brain_region+Sex	3.030	762.000
ENSG00000108984	0.257	0.191	0.483	-0.101	MAP2K6	0.511	~p_tau+(1|manifest)+cngeneson+pc_mito+brain_region+Sex	3.634	614.000
ENSG00000165181	0.186	0.190	0.539	-0.159	SHOC1	0.426	~p_tau+(1|manifest)+cngeneson+pc_mito+brain_region+Sex	3.336	675.000
ENSG00000106723	0.189	0.189	0.430	-0.051	SPIN1	0.430	~p_tau+(1|manifest)+cngeneson+pc_mito+brain_region+Sex	1.204	2076.000
ENSG00000091409	0.100	0.189	0.456	-0.078	ITGA6	0.301	~p_tau+(1|manifest)+cngeneson+pc_mito+brain_region+Sex	4.832	390.000
ENSG00000154122	0.305	0.189	0.434	-0.055	ANKH	0.561	~p_tau+(1|manifest)+cngeneson+pc_mito+brain_region+Sex	0.923	2609.000
ENSG00000197020	0.444	0.189	0.486	-0.107	ZNF100	0.682	~p_tau+(1|manifest)+cngeneson+pc_mito+brain_region+Sex	3.158	722.000
ENSG00000171045	0.003	0.189	0.328	0.049	TSNARE1	0.052	~p_tau+(1|manifest)+cngeneson+pc_mito+brain_region+Sex	0.826	2872.000
ENSG00000133318	0.106	0.189	0.362	0.015	RTN3	0.311	~p_tau+(1|manifest)+cngeneson+pc_mito+brain_region+Sex	1.113	2232.000
ENSG00000070501	0.027	0.189	0.419	-0.042	POLB	0.151	~p_tau+(1|manifest)+cngeneson+pc_mito+brain_region+Sex	2.510	966.000
ENSG00000074590	0.405	0.188	0.474	-0.097	NUAK1	0.651	~p_tau+(1|manifest)+cngeneson+pc_mito+brain_region+Sex	1.828	1371.000
ENSG00000174738	0.099	0.188	0.412	-0.036	NR1D2	0.299	~p_tau+(1|manifest)+cngeneson+pc_mito+brain_region+Sex	1.847	1354.000
ENSG00000142676	0.570	0.188	0.622	-0.246	RPL11	0.768	~p_tau+(1|manifest)+cngeneson+pc_mito+brain_region+Sex	3.944	535.000
ENSG00000205542	0.694	0.188	0.616	-0.240	TMSB4X	0.845	~p_tau+(1|manifest)+cngeneson+pc_mito+brain_region+Sex	19.110	28.000
ENSG00000118200	0.081	0.188	0.349	0.027	CAMSAP2	0.271	~p_tau+(1|manifest)+cngeneson+pc_mito+brain_region+Sex	0.684	3255.000
ENSG00000178425	0.063	0.188	0.349	0.027	NT5DC1	0.240	~p_tau+(1|manifest)+cngeneson+pc_mito+brain_region+Sex	1.392	1779.000
ENSG00000088538	0.043	0.188	0.436	-0.061	DOCK3	0.195	~p_tau+(1|manifest)+cngeneson+pc_mito+brain_region+Sex	1.225	2040.000
ENSG00000166900	0.002	0.188	0.319	0.056	STX3	0.039	~p_tau+(1|manifest)+cngeneson+pc_mito+brain_region+Sex	0.453	4131.000
ENSG00000132952	0.027	0.187	0.389	-0.014	USPL1	0.151	~p_tau+(1|manifest)+cngeneson+pc_mito+brain_region+Sex	1.165	2133.000
ENSG00000111605	0.105	0.187	0.441	-0.067	CPSF6	0.310	~p_tau+(1|manifest)+cngeneson+pc_mito+brain_region+Sex	1.524	1633.000
ENSG00000134352	0.643	0.187	0.640	-0.267	IL6ST	0.813	~p_tau+(1|manifest)+cngeneson+pc_mito+brain_region+Sex	17.959	31.000
ENSG00000149806	0.142	0.187	0.438	-0.065	FAU	0.365	~p_tau+(1|manifest)+cngeneson+pc_mito+brain_region+Sex	2.755	868.000
ENSG00000104894	0.021	0.186	0.434	-0.061	CD37	0.132	~p_tau+(1|manifest)+cngeneson+pc_mito+brain_region+Sex	2.900	800.000
ENSG00000135679	0.175	0.186	0.380	-0.009	MDM2	0.411	~p_tau+(1|manifest)+cngeneson+pc_mito+brain_region+Sex	1.016	2405.000
ENSG00000180011	0.003	0.185	0.318	0.053	ZADH2	0.053	~p_tau+(1|manifest)+cngeneson+pc_mito+brain_region+Sex	1.135	2186.000
ENSG00000105967	0.507	0.185	0.510	-0.140	TFEC	0.727	~p_tau+(1|manifest)+cngeneson+pc_mito+brain_region+Sex	2.813	835.000
ENSG00000198053	0.106	0.185	0.411	-0.041	SIRPA	0.311	~p_tau+(1|manifest)+cngeneson+pc_mito+brain_region+Sex	1.470	1694.000
ENSG00000078699	0.072	0.185	0.334	0.036	CBFA2T2	0.254	~p_tau+(1|manifest)+cngeneson+pc_mito+brain_region+Sex	0.862	2762.000
ENSG00000120802	0.002	0.185	0.344	0.025	TMPO	0.045	~p_tau+(1|manifest)+cngeneson+pc_mito+brain_region+Sex	0.846	2804.000
ENSG00000138399	0.003	0.185	0.301	0.069	FASTKD1	0.056	~p_tau+(1|manifest)+cngeneson+pc_mito+brain_region+Sex	0.224	5162.000
ENSG00000169413	0.003	0.185	0.309	0.060	RNASE6	0.054	~p_tau+(1|manifest)+cngeneson+pc_mito+brain_region+Sex	0.793	2951.000
ENSG00000108256	0.361	0.184	0.521	-0.152	NUFIP2	0.615	~p_tau+(1|manifest)+cngeneson+pc_mito+brain_region+Sex	2.705	888.000
ENSG00000137449	0.001	0.184	0.300	0.069	CPEB2	0.037	~p_tau+(1|manifest)+cngeneson+pc_mito+brain_region+Sex	0.337	4644.000
ENSG00000134852	0.050	0.184	0.341	0.027	CLOCK	0.210	~p_tau+(1|manifest)+cngeneson+pc_mito+brain_region+Sex	0.359	4527.000
ENSG00000172216	0.229	0.184	0.526	-0.159	CEBPB	0.478	~p_tau+(1|manifest)+cngeneson+pc_mito+brain_region+Sex	6.772	219.000
ENSG00000182985	0.165	0.183	0.382	-0.015	CADM1	0.396	~p_tau+(1|manifest)+cngeneson+pc_mito+brain_region+Sex	3.167	717.000
ENSG00000111269	0.321	0.183	0.438	-0.073	CREBL2	0.578	~p_tau+(1|manifest)+cngeneson+pc_mito+brain_region+Sex	2.094	1191.000
ENSG00000145687	0.381	0.183	0.471	-0.106	SSBP2	0.630	~p_tau+(1|manifest)+cngeneson+pc_mito+brain_region+Sex	4.613	416.000
ENSG00000137161	0.119	0.182	0.401	-0.036	CNPY3	0.334	~p_tau+(1|manifest)+cngeneson+pc_mito+brain_region+Sex	1.508	1655.000
ENSG00000153317	0.510	0.182	0.510	-0.145	ASAP1	0.728	~p_tau+(1|manifest)+cngeneson+pc_mito+brain_region+Sex	3.661	606.000
ENSG00000262655	0.307	0.182	0.499	-0.134	SPON1	0.563	~p_tau+(1|manifest)+cngeneson+pc_mito+brain_region+Sex	1.939	1287.000
ENSG00000049860	0.009	0.182	0.292	0.072	HEXB	0.087	~p_tau+(1|manifest)+cngeneson+pc_mito+brain_region+Sex	0.285	4897.000
ENSG00000105122	0.076	0.182	0.373	-0.009	RASAL3	0.262	~p_tau+(1|manifest)+cngeneson+pc_mito+brain_region+Sex	1.057	2335.000
ENSG00000102699	0.186	0.182	0.391	-0.027	PARP4	0.426	~p_tau+(1|manifest)+cngeneson+pc_mito+brain_region+Sex	1.899	1312.000
ENSG00000038532	0.006	0.181	0.326	0.037	CLEC16A	0.073	~p_tau+(1|manifest)+cngeneson+pc_mito+brain_region+Sex	0.525	3817.000
ENSG00000171863	0.017	0.181	0.395	-0.033	RPS7	0.119	~p_tau+(1|manifest)+cngeneson+pc_mito+brain_region+Sex	1.539	1616.000
ENSG00000171105	0.583	0.181	0.531	-0.169	INSR	0.777	~p_tau+(1|manifest)+cngeneson+pc_mito+brain_region+Sex	4.149	498.000
ENSG00000168883	0.042	0.181	0.375	-0.014	USP39	0.192	~p_tau+(1|manifest)+cngeneson+pc_mito+brain_region+Sex	0.959	2524.000
ENSG00000107521	0.001	0.180	0.282	0.078	HPS1	0.036	~p_tau+(1|manifest)+cngeneson+pc_mito+brain_region+Sex	0.696	3218.000
ENSG00000213047	0.092	0.180	0.339	0.021	DENND1B	0.288	~p_tau+(1|manifest)+cngeneson+pc_mito+brain_region+Sex	1.125	2209.000
ENSG00000163960	0.056	0.180	0.350	0.010	UBXN7	0.225	~p_tau+(1|manifest)+cngeneson+pc_mito+brain_region+Sex	0.913	2640.000
ENSG00000115947	0.025	0.180	0.313	0.046	ORC4	0.145	~p_tau+(1|manifest)+cngeneson+pc_mito+brain_region+Sex	0.425	4240.000
ENSG00000105372	0.228	0.180	0.615	-0.256	RPS19	0.476	~p_tau+(1|manifest)+cngeneson+pc_mito+brain_region+Sex	2.676	899.000
ENSG00000109332	0.469	0.180	0.535	-0.176	UBE2D3	0.700	~p_tau+(1|manifest)+cngeneson+pc_mito+brain_region+Sex	3.125	731.000
ENSG00000140988	0.449	0.179	0.579	-0.220	RPS2	0.685	~p_tau+(1|manifest)+cngeneson+pc_mito+brain_region+Sex	6.045	279.000
ENSG00000078579	0.124	0.179	0.369	-0.010	FGF20	0.340	~p_tau+(1|manifest)+cngeneson+pc_mito+brain_region+Sex	1.815	1381.000
ENSG00000179454	0.005	0.179	0.289	0.069	KLHL28	0.064	~p_tau+(1|manifest)+cngeneson+pc_mito+brain_region+Sex	0.399	4363.000
ENSG00000168016	0.273	0.179	0.397	-0.039	TRANK1	0.527	~p_tau+(1|manifest)+cngeneson+pc_mito+brain_region+Sex	2.102	1188.000
ENSG00000157741	0.021	0.179	0.349	0.009	UBN2	0.134	~p_tau+(1|manifest)+cngeneson+pc_mito+brain_region+Sex	0.559	3687.000
ENSG00000147044	0.184	0.179	0.396	-0.039	CASK	0.424	~p_tau+(1|manifest)+cngeneson+pc_mito+brain_region+Sex	0.570	3650.000
ENSG00000197771	0.012	0.179	0.315	0.042	MCMBP	0.099	~p_tau+(1|manifest)+cngeneson+pc_mito+brain_region+Sex	0.674	3284.000
ENSG00000133943	0.185	0.178	0.393	-0.036	DGLUCY	0.425	~p_tau+(1|manifest)+cngeneson+pc_mito+brain_region+Sex	1.642	1510.000
ENSG00000109861	0.389	0.178	0.478	-0.122	CTSC	0.637	~p_tau+(1|manifest)+cngeneson+pc_mito+brain_region+Sex	3.303	682.000
ENSG00000196776	0.001	0.178	0.287	0.070	CD47	0.037	~p_tau+(1|manifest)+cngeneson+pc_mito+brain_region+Sex	0.920	2620.000
ENSG00000107863	0.510	0.178	0.510	-0.154	ARHGAP21	0.729	~p_tau+(1|manifest)+cngeneson+pc_mito+brain_region+Sex	6.783	218.000
ENSG00000196914	0.093	0.178	0.338	0.017	ARHGEF12	0.290	~p_tau+(1|manifest)+cngeneson+pc_mito+brain_region+Sex	0.300	4816.000
ENSG00000137601	0.044	0.178	0.323	0.033	NEK1	0.197	~p_tau+(1|manifest)+cngeneson+pc_mito+brain_region+Sex	0.313	4757.000
ENSG00000105698	0.025	0.178	0.321	0.035	USF2	0.146	~p_tau+(1|manifest)+cngeneson+pc_mito+brain_region+Sex	0.947	2549.000
ENSG00000120063	0.657	0.178	0.562	-0.207	GNA13	0.823	~p_tau+(1|manifest)+cngeneson+pc_mito+brain_region+Sex	6.215	262.000
ENSG00000065883	0.073	0.177	0.337	0.018	CDK13	0.256	~p_tau+(1|manifest)+cngeneson+pc_mito+brain_region+Sex	0.809	2915.000
ENSG00000164111	0.002	0.177	0.285	0.070	ANXA5	0.038	~p_tau+(1|manifest)+cngeneson+pc_mito+brain_region+Sex	0.509	3885.000
ENSG00000177000	0.009	0.177	0.311	0.044	MTHFR	0.083	~p_tau+(1|manifest)+cngeneson+pc_mito+brain_region+Sex	0.803	2927.000
ENSG00000176658	0.063	0.177	0.336	0.017	MYO1D	0.240	~p_tau+(1|manifest)+cngeneson+pc_mito+brain_region+Sex	0.765	3024.000
ENSG00000100284	0.008	0.176	0.306	0.047	TOM1	0.082	~p_tau+(1|manifest)+cngeneson+pc_mito+brain_region+Sex	0.655	3344.000
ENSG00000147324	0.001	0.176	0.264	0.089	MFHAS1	0.031	~p_tau+(1|manifest)+cngeneson+pc_mito+brain_region+Sex	0.640	3391.000
ENSG00000103994	0.047	0.176	0.343	0.010	ZNF106	0.202	~p_tau+(1|manifest)+cngeneson+pc_mito+brain_region+Sex	0.120	5436.000
ENSG00000105643	0.011	0.176	0.351	0.002	ARRDC2	0.094	~p_tau+(1|manifest)+cngeneson+pc_mito+brain_region+Sex	1.290	1929.000
ENSG00000153933	0.017	0.176	0.389	-0.037	DGKE	0.122	~p_tau+(1|manifest)+cngeneson+pc_mito+brain_region+Sex	2.647	913.000
ENSG00000068097	0.001	0.176	0.276	0.076	HEATR6	0.024	~p_tau+(1|manifest)+cngeneson+pc_mito+brain_region+Sex	0.318	4735.000
ENSG00000138449	0.024	0.176	0.399	-0.046	SLC40A1	0.143	~p_tau+(1|manifest)+cngeneson+pc_mito+brain_region+Sex	2.559	949.000
ENSG00000170802	0.187	0.176	0.510	-0.157	FOXN2	0.427	~p_tau+(1|manifest)+cngeneson+pc_mito+brain_region+Sex	1.260	1978.000
ENSG00000111275	0.151	0.176	0.376	-0.024	ALDH2	0.379	~p_tau+(1|manifest)+cngeneson+pc_mito+brain_region+Sex	1.319	1879.000
ENSG00000104093	0.227	0.176	0.386	-0.034	DMXL2	0.476	~p_tau+(1|manifest)+cngeneson+pc_mito+brain_region+Sex	3.009	770.000
ENSG00000114126	0.189	0.176	0.389	-0.037	TFDP2	0.429	~p_tau+(1|manifest)+cngeneson+pc_mito+brain_region+Sex	1.384	1786.000
ENSG00000081913	0.218	0.176	0.376	-0.025	PHLPP1	0.463	~p_tau+(1|manifest)+cngeneson+pc_mito+brain_region+Sex	1.613	1538.000
ENSG00000155307	0.440	0.176	0.439	-0.087	SAMSN1	0.680	~p_tau+(1|manifest)+cngeneson+pc_mito+brain_region+Sex	2.043	1220.000
ENSG00000127084	0.008	0.175	0.332	0.019	FGD3	0.080	~p_tau+(1|manifest)+cngeneson+pc_mito+brain_region+Sex	0.491	3977.000
ENSG00000177311	0.016	0.175	0.289	0.061	ZBTB38	0.117	~p_tau+(1|manifest)+cngeneson+pc_mito+brain_region+Sex	0.417	4285.000
ENSG00000101160	0.133	0.175	0.419	-0.069	CTSZ	0.354	~p_tau+(1|manifest)+cngeneson+pc_mito+brain_region+Sex	1.387	1782.000
ENSG00000137752	0.031	0.175	0.461	-0.111	CASP1	0.162	~p_tau+(1|manifest)+cngeneson+pc_mito+brain_region+Sex	2.687	894.000
ENSG00000135404	0.141	0.175	0.391	-0.042	CD63	0.364	~p_tau+(1|manifest)+cngeneson+pc_mito+brain_region+Sex	1.601	1558.000
ENSG00000119801	0.000	0.175	0.313	0.037	YPEL5	0.013	~p_tau+(1|manifest)+cngeneson+pc_mito+brain_region+Sex	0.970	2498.000
ENSG00000170634	0.286	0.174	0.390	-0.041	ACYP2	0.539	~p_tau+(1|manifest)+cngeneson+pc_mito+brain_region+Sex	0.345	4609.000
ENSG00000137642	0.030	0.174	0.313	0.036	SORL1	0.160	~p_tau+(1|manifest)+cngeneson+pc_mito+brain_region+Sex	8.379	145.000
ENSG00000141504	0.001	0.174	0.324	0.024	SAT2	0.033	~p_tau+(1|manifest)+cngeneson+pc_mito+brain_region+Sex	1.069	2306.000
ENSG00000198909	0.216	0.174	0.403	-0.054	MAP3K3	0.461	~p_tau+(1|manifest)+cngeneson+pc_mito+brain_region+Sex	1.431	1743.000
ENSG00000109685	0.051	0.174	0.309	0.039	NSD2	0.214	~p_tau+(1|manifest)+cngeneson+pc_mito+brain_region+Sex	0.332	4674.000
ENSG00000178980	0.002	0.174	0.330	0.017	SELENOW	0.039	~p_tau+(1|manifest)+cngeneson+pc_mito+brain_region+Sex	0.696	3222.000
ENSG00000039523	0.006	0.174	0.297	0.050	RIPOR1	0.070	~p_tau+(1|manifest)+cngeneson+pc_mito+brain_region+Sex	1.048	2351.000
ENSG00000156869	0.631	0.173	0.678	-0.331	FRRS1	0.806	~p_tau+(1|manifest)+cngeneson+pc_mito+brain_region+Sex	6.443	243.000
ENSG00000113269	0.089	0.173	0.334	0.012	RNF130	0.283	~p_tau+(1|manifest)+cngeneson+pc_mito+brain_region+Sex	2.889	803.000
ENSG00000011566	0.207	0.173	0.381	-0.035	MAP4K3	0.450	~p_tau+(1|manifest)+cngeneson+pc_mito+brain_region+Sex	1.327	1872.000
ENSG00000146425	0.014	0.173	0.311	0.034	DYNLT1	0.108	~p_tau+(1|manifest)+cngeneson+pc_mito+brain_region+Sex	0.874	2732.000
ENSG00000133103	0.026	0.173	0.293	0.052	COG6	0.150	~p_tau+(1|manifest)+cngeneson+pc_mito+brain_region+Sex	0.177	5304.000
ENSG00000166441	0.213	0.173	0.503	-0.158	RPL27A	0.456	~p_tau+(1|manifest)+cngeneson+pc_mito+brain_region+Sex	4.134	500.000
ENSG00000175895	0.009	0.172	0.298	0.045	PLEKHF2	0.085	~p_tau+(1|manifest)+cngeneson+pc_mito+brain_region+Sex	0.604	3518.000
ENSG00000171729	0.386	0.172	0.414	-0.070	TMEM51	0.635	~p_tau+(1|manifest)+cngeneson+pc_mito+brain_region+Sex	3.048	754.000
ENSG00000188001	0.344	0.172	0.428	-0.084	TPRG1	0.598	~p_tau+(1|manifest)+cngeneson+pc_mito+brain_region+Sex	4.734	400.000
ENSG00000182220	0.000	0.172	0.267	0.077	ATP6AP2	0.016	~p_tau+(1|manifest)+cngeneson+pc_mito+brain_region+Sex	0.293	4853.000
ENSG00000173166	0.289	0.172	0.422	-0.079	RAPH1	0.542	~p_tau+(1|manifest)+cngeneson+pc_mito+brain_region+Sex	1.209	2068.000
ENSG00000164031	0.053	0.171	0.362	-0.019	DNAJB14	0.218	~p_tau+(1|manifest)+cngeneson+pc_mito+brain_region+Sex	0.691	3229.000
ENSG00000172262	0.020	0.171	0.297	0.046	ZNF131	0.129	~p_tau+(1|manifest)+cngeneson+pc_mito+brain_region+Sex	0.210	5205.000
ENSG00000164830	0.460	0.171	0.435	-0.093	OXR1	0.692	~p_tau+(1|manifest)+cngeneson+pc_mito+brain_region+Sex	5.004	365.000
ENSG00000119899	0.000	0.171	0.255	0.087	SLC17A5	0.020	~p_tau+(1|manifest)+cngeneson+pc_mito+brain_region+Sex	0.312	4767.000
ENSG00000130254	0.003	0.171	0.290	0.053	SAFB2	0.055	~p_tau+(1|manifest)+cngeneson+pc_mito+brain_region+Sex	0.389	4401.000
ENSG00000103249	0.001	0.171	0.308	0.034	CLCN7	0.026	~p_tau+(1|manifest)+cngeneson+pc_mito+brain_region+Sex	0.336	4652.000
ENSG00000269743	0.001	0.171	0.296	0.046	SLC25A53	0.028	~p_tau+(1|manifest)+cngeneson+pc_mito+brain_region+Sex	0.653	3349.000
ENSG00000090615	0.000	0.170	0.241	0.100	GOLGA3	0.009	~p_tau+(1|manifest)+cngeneson+pc_mito+brain_region+Sex	0.294	4845.000
ENSG00000088986	0.003	0.170	0.283	0.057	DYNLL1	0.051	~p_tau+(1|manifest)+cngeneson+pc_mito+brain_region+Sex	0.691	3228.000
ENSG00000173020	0.075	0.170	0.457	-0.117	GRK2	0.261	~p_tau+(1|manifest)+cngeneson+pc_mito+brain_region+Sex	2.540	953.000
ENSG00000111670	0.257	0.170	0.375	-0.035	GNPTAB	0.511	~p_tau+(1|manifest)+cngeneson+pc_mito+brain_region+Sex	1.469	1696.000
ENSG00000173041	0.000	0.169	0.220	0.119	ZNF680	0.002	~p_tau+(1|manifest)+cngeneson+pc_mito+brain_region+Sex	0.451	4137.000
ENSG00000196705	0.005	0.169	0.260	0.078	ZNF431	0.063	~p_tau+(1|manifest)+cngeneson+pc_mito+brain_region+Sex	0.567	3659.000
ENSG00000171606	0.000	0.169	0.261	0.076	ZNF274	0.018	~p_tau+(1|manifest)+cngeneson+pc_mito+brain_region+Sex	0.399	4364.000
ENSG00000112759	0.019	0.168	0.338	-0.002	SLC29A1	0.127	~p_tau+(1|manifest)+cngeneson+pc_mito+brain_region+Sex	0.853	2780.000
ENSG00000168876	0.018	0.168	0.297	0.039	ANKRD49	0.124	~p_tau+(1|manifest)+cngeneson+pc_mito+brain_region+Sex	0.611	3491.000
ENSG00000205268	0.200	0.168	0.351	-0.015	PDE7A	0.442	~p_tau+(1|manifest)+cngeneson+pc_mito+brain_region+Sex	0.838	2835.000
ENSG00000121064	0.002	0.168	0.266	0.070	SCPEP1	0.044	~p_tau+(1|manifest)+cngeneson+pc_mito+brain_region+Sex	0.754	3052.000
ENSG00000111897	0.001	0.167	0.269	0.066	SERINC1	0.036	~p_tau+(1|manifest)+cngeneson+pc_mito+brain_region+Sex	0.761	3037.000
ENSG00000083642	0.270	0.167	0.398	-0.063	PDS5B	0.523	~p_tau+(1|manifest)+cngeneson+pc_mito+brain_region+Sex	0.926	2601.000
ENSG00000162836	0.001	0.167	0.261	0.073	ACP6	0.024	~p_tau+(1|manifest)+cngeneson+pc_mito+brain_region+Sex	0.334	4660.000
ENSG00000155097	0.144	0.167	0.349	-0.015	ATP6V1C1	0.368	~p_tau+(1|manifest)+cngeneson+pc_mito+brain_region+Sex	1.352	1829.000
ENSG00000135250	0.377	0.167	0.401	-0.067	SRPK2	0.628	~p_tau+(1|manifest)+cngeneson+pc_mito+brain_region+Sex	1.308	1896.000
ENSG00000140678	0.698	0.167	0.624	-0.291	ITGAX	0.847	~p_tau+(1|manifest)+cngeneson+pc_mito+brain_region+Sex	5.599	308.000
ENSG00000070759	0.042	0.167	0.308	0.025	TESK2	0.192	~p_tau+(1|manifest)+cngeneson+pc_mito+brain_region+Sex	0.670	3297.000
ENSG00000139832	0.213	0.167	0.429	-0.096	RAB20	0.457	~p_tau+(1|manifest)+cngeneson+pc_mito+brain_region+Sex	1.599	1559.000
ENSG00000176783	0.005	0.166	0.257	0.076	RUFY1	0.063	~p_tau+(1|manifest)+cngeneson+pc_mito+brain_region+Sex	0.506	3902.000
ENSG00000153339	0.136	0.166	0.339	-0.007	TRAPPC8	0.357	~p_tau+(1|manifest)+cngeneson+pc_mito+brain_region+Sex	0.921	2614.000
ENSG00000031823	0.010	0.166	0.303	0.029	RANBP3	0.089	~p_tau+(1|manifest)+cngeneson+pc_mito+brain_region+Sex	0.654	3346.000
ENSG00000113240	0.006	0.166	0.310	0.022	CLK4	0.073	~p_tau+(1|manifest)+cngeneson+pc_mito+brain_region+Sex	0.726	3125.000
ENSG00000112062	0.258	0.166	0.416	-0.084	MAPK14	0.512	~p_tau+(1|manifest)+cngeneson+pc_mito+brain_region+Sex	1.250	1998.000
ENSG00000116786	0.001	0.165	0.255	0.076	PLEKHM2	0.033	~p_tau+(1|manifest)+cngeneson+pc_mito+brain_region+Sex	0.672	3288.000
ENSG00000100612	0.000	0.165	0.272	0.059	DHRS7	0.015	~p_tau+(1|manifest)+cngeneson+pc_mito+brain_region+Sex	0.571	3644.000
ENSG00000105875	0.021	0.165	0.285	0.045	WDR91	0.132	~p_tau+(1|manifest)+cngeneson+pc_mito+brain_region+Sex	0.741	3081.000
ENSG00000133805	0.342	0.165	0.391	-0.061	AMPD3	0.597	~p_tau+(1|manifest)+cngeneson+pc_mito+brain_region+Sex	2.353	1042.000
ENSG00000139826	0.005	0.165	0.285	0.045	ABHD13	0.065	~p_tau+(1|manifest)+cngeneson+pc_mito+brain_region+Sex	0.636	3401.000
ENSG00000142303	0.060	0.165	0.363	-0.034	ADAMTS10	0.233	~p_tau+(1|manifest)+cngeneson+pc_mito+brain_region+Sex	1.226	2039.000
ENSG00000142784	0.019	0.165	0.325	0.004	WDTC1	0.126	~p_tau+(1|manifest)+cngeneson+pc_mito+brain_region+Sex	0.981	2473.000
ENSG00000124357	0.039	0.165	0.374	-0.045	NAGK	0.185	~p_tau+(1|manifest)+cngeneson+pc_mito+brain_region+Sex	2.734	878.000
ENSG00000135823	0.260	0.164	0.404	-0.076	STX6	0.514	~p_tau+(1|manifest)+cngeneson+pc_mito+brain_region+Sex	1.342	1844.000
ENSG00000166436	0.069	0.164	0.312	0.016	TRIM66	0.249	~p_tau+(1|manifest)+cngeneson+pc_mito+brain_region+Sex	2.357	1039.000
ENSG00000143353	0.046	0.164	0.396	-0.067	LYPLAL1	0.202	~p_tau+(1|manifest)+cngeneson+pc_mito+brain_region+Sex	0.866	2755.000
ENSG00000263956	0.013	0.164	0.310	0.018	NBPF11	0.102	~p_tau+(1|manifest)+cngeneson+pc_mito+brain_region+Sex	1.024	2388.000
ENSG00000112149	0.153	0.164	0.340	-0.012	CD83	0.381	~p_tau+(1|manifest)+cngeneson+pc_mito+brain_region+Sex	1.995	1251.000
ENSG00000157540	0.128	0.164	0.330	-0.003	DYRK1A	0.346	~p_tau+(1|manifest)+cngeneson+pc_mito+brain_region+Sex	1.232	2033.000
ENSG00000162599	0.527	0.164	0.448	-0.121	NFIA	0.739	~p_tau+(1|manifest)+cngeneson+pc_mito+brain_region+Sex	1.850	1351.000
ENSG00000178974	0.034	0.163	0.288	0.038	FBXO34	0.171	~p_tau+(1|manifest)+cngeneson+pc_mito+brain_region+Sex	0.538	3770.000
ENSG00000157107	0.472	0.163	0.453	-0.127	FCHO2	0.701	~p_tau+(1|manifest)+cngeneson+pc_mito+brain_region+Sex	2.286	1080.000
ENSG00000110324	0.558	0.163	0.597	-0.271	IL10RA	0.761	~p_tau+(1|manifest)+cngeneson+pc_mito+brain_region+Sex	3.603	622.000
ENSG00000196151	0.018	0.163	0.306	0.020	WDSUB1	0.123	~p_tau+(1|manifest)+cngeneson+pc_mito+brain_region+Sex	0.619	3469.000
ENSG00000112282	0.005	0.163	0.268	0.057	MED23	0.066	~p_tau+(1|manifest)+cngeneson+pc_mito+brain_region+Sex	0.597	3549.000
ENSG00000123643	0.006	0.163	0.272	0.053	SLC36A1	0.071	~p_tau+(1|manifest)+cngeneson+pc_mito+brain_region+Sex	1.361	1816.000
ENSG00000122862	0.870	0.162	0.914	-0.590	SRGN	0.941	~p_tau+(1|manifest)+cngeneson+pc_mito+brain_region+Sex	18.662	29.000
ENSG00000142875	0.336	0.162	0.450	-0.126	PRKACB	0.592	~p_tau+(1|manifest)+cngeneson+pc_mito+brain_region+Sex	2.449	996.000
ENSG00000214078	0.006	0.162	0.268	0.056	CPNE1	0.074	~p_tau+(1|manifest)+cngeneson+pc_mito+brain_region+Sex	0.609	3501.000
ENSG00000103489	0.662	0.162	0.520	-0.196	XYLT1	0.826	~p_tau+(1|manifest)+cngeneson+pc_mito+brain_region+Sex	2.861	812.000
ENSG00000108239	0.269	0.162	0.372	-0.049	TBC1D12	0.523	~p_tau+(1|manifest)+cngeneson+pc_mito+brain_region+Sex	1.493	1672.000
ENSG00000129625	0.014	0.161	0.292	0.031	REEP5	0.106	~p_tau+(1|manifest)+cngeneson+pc_mito+brain_region+Sex	0.578	3622.000
ENSG00000069493	0.430	0.161	0.434	-0.111	CLEC2D	0.670	~p_tau+(1|manifest)+cngeneson+pc_mito+brain_region+Sex	1.605	1552.000
ENSG00000105889	0.477	0.161	0.434	-0.111	STEAP1B	0.704	~p_tau+(1|manifest)+cngeneson+pc_mito+brain_region+Sex	2.764	863.000
ENSG00000116209	0.003	0.161	0.274	0.049	TMEM59	0.052	~p_tau+(1|manifest)+cngeneson+pc_mito+brain_region+Sex	0.331	4678.000
ENSG00000109971	0.005	0.161	0.336	-0.013	HSPA8	0.062	~p_tau+(1|manifest)+cngeneson+pc_mito+brain_region+Sex	0.765	3028.000
ENSG00000185896	0.035	0.161	0.315	0.007	LAMP1	0.175	~p_tau+(1|manifest)+cngeneson+pc_mito+brain_region+Sex	1.506	1658.000
ENSG00000160191	0.343	0.161	0.419	-0.096	PDE9A	0.597	~p_tau+(1|manifest)+cngeneson+pc_mito+brain_region+Sex	2.390	1023.000
ENSG00000175274	0.000	0.161	0.255	0.066	TP53I11	0.019	~p_tau+(1|manifest)+cngeneson+pc_mito+brain_region+Sex	0.825	2875.000
ENSG00000115084	0.094	0.161	0.359	-0.038	SLC35F5	0.291	~p_tau+(1|manifest)+cngeneson+pc_mito+brain_region+Sex	1.473	1691.000
ENSG00000112773	0.215	0.161	0.413	-0.092	TENT5A	0.459	~p_tau+(1|manifest)+cngeneson+pc_mito+brain_region+Sex	5.508	317.000
ENSG00000032444	0.039	0.160	0.353	-0.032	PNPLA6	0.185	~p_tau+(1|manifest)+cngeneson+pc_mito+brain_region+Sex	0.644	3380.000
ENSG00000198718	0.001	0.160	0.242	0.078	TOGARAM1	0.036	~p_tau+(1|manifest)+cngeneson+pc_mito+brain_region+Sex	0.289	4873.000
ENSG00000134207	0.273	0.160	0.412	-0.091	SYT6	0.527	~p_tau+(1|manifest)+cngeneson+pc_mito+brain_region+Sex	1.317	1882.000
ENSG00000108799	0.108	0.160	0.348	-0.028	EZH1	0.315	~p_tau+(1|manifest)+cngeneson+pc_mito+brain_region+Sex	1.435	1741.000
ENSG00000172915	0.444	0.159	0.400	-0.081	NBEA	0.682	~p_tau+(1|manifest)+cngeneson+pc_mito+brain_region+Sex	1.075	2299.000
ENSG00000182670	0.299	0.159	0.355	-0.037	TTC3	0.553	~p_tau+(1|manifest)+cngeneson+pc_mito+brain_region+Sex	1.666	1482.000
ENSG00000130844	0.047	0.159	0.288	0.031	ZNF331	0.202	~p_tau+(1|manifest)+cngeneson+pc_mito+brain_region+Sex	0.347	4597.000
ENSG00000066855	0.126	0.159	0.373	-0.054	MTFR1	0.343	~p_tau+(1|manifest)+cngeneson+pc_mito+brain_region+Sex	2.042	1222.000
ENSG00000109180	0.060	0.159	0.298	0.020	OCIAD1	0.234	~p_tau+(1|manifest)+cngeneson+pc_mito+brain_region+Sex	0.611	3492.000
ENSG00000117500	0.116	0.159	0.320	-0.003	TMED5	0.330	~p_tau+(1|manifest)+cngeneson+pc_mito+brain_region+Sex	0.637	3397.000
ENSG00000124299	0.000	0.158	0.222	0.095	PEPD	0.006	~p_tau+(1|manifest)+cngeneson+pc_mito+brain_region+Sex	0.365	4496.000
ENSG00000085365	0.005	0.158	0.254	0.063	SCAMP1	0.064	~p_tau+(1|manifest)+cngeneson+pc_mito+brain_region+Sex	0.280	4924.000
ENSG00000197381	0.007	0.158	0.256	0.060	ADARB1	0.075	~p_tau+(1|manifest)+cngeneson+pc_mito+brain_region+Sex	0.256	5048.000
ENSG00000122507	0.315	0.158	0.383	-0.067	BBS9	0.570	~p_tau+(1|manifest)+cngeneson+pc_mito+brain_region+Sex	1.553	1610.000
ENSG00000166167	0.266	0.158	0.382	-0.067	BTRC	0.520	~p_tau+(1|manifest)+cngeneson+pc_mito+brain_region+Sex	0.815	2900.000
ENSG00000081377	0.047	0.158	0.278	0.037	CDC14B	0.202	~p_tau+(1|manifest)+cngeneson+pc_mito+brain_region+Sex	0.298	4821.000
ENSG00000135686	0.000	0.158	0.248	0.068	KLHL36	0.016	~p_tau+(1|manifest)+cngeneson+pc_mito+brain_region+Sex	0.407	4328.000
ENSG00000258890	0.022	0.157	0.300	0.015	CEP95	0.139	~p_tau+(1|manifest)+cngeneson+pc_mito+brain_region+Sex	0.596	3554.000
ENSG00000106665	0.154	0.157	0.372	-0.058	CLIP2	0.382	~p_tau+(1|manifest)+cngeneson+pc_mito+brain_region+Sex	1.142	2175.000
ENSG00000158717	0.267	0.157	0.436	-0.122	RNF166	0.521	~p_tau+(1|manifest)+cngeneson+pc_mito+brain_region+Sex	3.080	745.000
ENSG00000166224	0.022	0.157	0.311	0.002	SGPL1	0.139	~p_tau+(1|manifest)+cngeneson+pc_mito+brain_region+Sex	0.602	3528.000
ENSG00000177030	0.013	0.156	0.270	0.043	DEAF1	0.103	~p_tau+(1|manifest)+cngeneson+pc_mito+brain_region+Sex	0.341	4628.000
ENSG00000124193	0.084	0.156	0.338	-0.026	SRSF6	0.277	~p_tau+(1|manifest)+cngeneson+pc_mito+brain_region+Sex	1.084	2280.000
ENSG00000143153	0.019	0.156	0.281	0.031	ATP1B1	0.125	~p_tau+(1|manifest)+cngeneson+pc_mito+brain_region+Sex	1.229	2035.000
ENSG00000182899	0.013	0.156	0.344	-0.032	RPL35A	0.105	~p_tau+(1|manifest)+cngeneson+pc_mito+brain_region+Sex	0.960	2520.000
ENSG00000138688	0.272	0.156	0.405	-0.093	KIAA1109	0.526	~p_tau+(1|manifest)+cngeneson+pc_mito+brain_region+Sex	2.579	941.000
ENSG00000109906	0.898	0.156	0.886	-0.574	ZBTB16	0.953	~p_tau+(1|manifest)+cngeneson+pc_mito+brain_region+Sex	5.308	328.000
ENSG00000134265	0.042	0.156	0.280	0.031	NAPG	0.191	~p_tau+(1|manifest)+cngeneson+pc_mito+brain_region+Sex	0.367	4489.000
ENSG00000087088	0.055	0.156	0.327	-0.016	BAX	0.224	~p_tau+(1|manifest)+cngeneson+pc_mito+brain_region+Sex	1.165	2135.000
ENSG00000100311	0.382	0.155	0.376	-0.067	PDGFB	0.631	~p_tau+(1|manifest)+cngeneson+pc_mito+brain_region+Sex	2.900	798.000
ENSG00000170836	0.036	0.155	0.272	0.037	PPM1D	0.178	~p_tau+(1|manifest)+cngeneson+pc_mito+brain_region+Sex	0.440	4175.000
ENSG00000124406	0.084	0.155	0.314	-0.005	ATP8A1	0.276	~p_tau+(1|manifest)+cngeneson+pc_mito+brain_region+Sex	0.866	2754.000
ENSG00000141837	0.240	0.154	0.591	-0.282	CACNA1A	0.491	~p_tau+(1|manifest)+cngeneson+pc_mito+brain_region+Sex	4.346	463.000
ENSG00000081154	0.001	0.154	0.234	0.075	PCNP	0.035	~p_tau+(1|manifest)+cngeneson+pc_mito+brain_region+Sex	0.402	4350.000
ENSG00000146676	0.014	0.154	0.272	0.036	PURB	0.110	~p_tau+(1|manifest)+cngeneson+pc_mito+brain_region+Sex	0.448	4154.000
ENSG00000141429	0.483	0.154	0.418	-0.110	GALNT1	0.709	~p_tau+(1|manifest)+cngeneson+pc_mito+brain_region+Sex	0.840	2827.000
ENSG00000154721	0.214	0.154	0.389	-0.081	JAM2	0.458	~p_tau+(1|manifest)+cngeneson+pc_mito+brain_region+Sex	2.125	1169.000
ENSG00000100354	0.329	0.154	0.368	-0.060	TNRC6B	0.587	~p_tau+(1|manifest)+cngeneson+pc_mito+brain_region+Sex	3.697	591.000
ENSG00000134744	0.146	0.154	0.317	-0.010	TUT4	0.370	~p_tau+(1|manifest)+cngeneson+pc_mito+brain_region+Sex	1.254	1991.000
ENSG00000135334	0.001	0.153	0.247	0.060	AKIRIN2	0.028	~p_tau+(1|manifest)+cngeneson+pc_mito+brain_region+Sex	0.369	4478.000
ENSG00000104427	0.003	0.153	0.242	0.064	ZC2HC1A	0.055	~p_tau+(1|manifest)+cngeneson+pc_mito+brain_region+Sex	0.301	4814.000
ENSG00000198431	0.054	0.153	0.286	0.020	TXNRD1	0.222	~p_tau+(1|manifest)+cngeneson+pc_mito+brain_region+Sex	1.072	2304.000
ENSG00000205531	0.050	0.153	0.282	0.024	NAP1L4	0.210	~p_tau+(1|manifest)+cngeneson+pc_mito+brain_region+Sex	0.754	3053.000
ENSG00000134759	0.059	0.153	0.369	-0.063	ELP2	0.231	~p_tau+(1|manifest)+cngeneson+pc_mito+brain_region+Sex	0.996	2446.000
ENSG00000112406	0.086	0.153	0.358	-0.052	HECA	0.280	~p_tau+(1|manifest)+cngeneson+pc_mito+brain_region+Sex	0.676	3281.000
ENSG00000121774	0.067	0.153	0.409	-0.103	KHDRBS1	0.245	~p_tau+(1|manifest)+cngeneson+pc_mito+brain_region+Sex	1.450	1718.000
ENSG00000146376	0.719	0.153	0.526	-0.220	ARHGAP18	0.859	~p_tau+(1|manifest)+cngeneson+pc_mito+brain_region+Sex	6.392	247.000
ENSG00000186951	0.020	0.153	0.271	0.034	PPARA	0.130	~p_tau+(1|manifest)+cngeneson+pc_mito+brain_region+Sex	0.870	2741.000
ENSG00000102710	0.110	0.153	0.300	0.005	SUPT20H	0.318	~p_tau+(1|manifest)+cngeneson+pc_mito+brain_region+Sex	0.947	2551.000
ENSG00000006715	0.036	0.152	0.339	-0.034	VPS41	0.177	~p_tau+(1|manifest)+cngeneson+pc_mito+brain_region+Sex	0.748	3068.000
ENSG00000115307	0.022	0.152	0.323	-0.018	AUP1	0.137	~p_tau+(1|manifest)+cngeneson+pc_mito+brain_region+Sex	1.042	2363.000
ENSG00000163931	0.023	0.152	0.306	-0.002	TKT	0.140	~p_tau+(1|manifest)+cngeneson+pc_mito+brain_region+Sex	0.458	4114.000
ENSG00000069974	0.120	0.152	0.379	-0.075	RAB27A	0.336	~p_tau+(1|manifest)+cngeneson+pc_mito+brain_region+Sex	1.281	1943.000
ENSG00000011295	0.133	0.152	0.372	-0.068	TTC19	0.354	~p_tau+(1|manifest)+cngeneson+pc_mito+brain_region+Sex	1.428	1747.000
ENSG00000133835	0.174	0.152	0.376	-0.073	HSD17B4	0.410	~p_tau+(1|manifest)+cngeneson+pc_mito+brain_region+Sex	1.908	1308.000
ENSG00000188529	0.067	0.152	0.306	-0.002	SRSF10	0.245	~p_tau+(1|manifest)+cngeneson+pc_mito+brain_region+Sex	0.631	3415.000
ENSG00000136874	0.004	0.152	0.275	0.028	STX17	0.062	~p_tau+(1|manifest)+cngeneson+pc_mito+brain_region+Sex	0.522	3827.000
ENSG00000118058	0.009	0.152	0.305	-0.002	KMT2A	0.083	~p_tau+(1|manifest)+cngeneson+pc_mito+brain_region+Sex	0.574	3637.000
ENSG00000119772	0.463	0.151	0.388	-0.085	DNMT3A	0.695	~p_tau+(1|manifest)+cngeneson+pc_mito+brain_region+Sex	1.581	1580.000
ENSG00000180098	0.004	0.151	0.253	0.049	TRNAU1AP	0.056	~p_tau+(1|manifest)+cngeneson+pc_mito+brain_region+Sex	0.583	3605.000
ENSG00000186184	0.026	0.151	0.265	0.038	POLR1D	0.148	~p_tau+(1|manifest)+cngeneson+pc_mito+brain_region+Sex	0.735	3098.000
ENSG00000169762	0.288	0.151	0.428	-0.126	TAPT1	0.540	~p_tau+(1|manifest)+cngeneson+pc_mito+brain_region+Sex	1.222	2047.000
ENSG00000188243	0.031	0.151	0.290	0.012	COMMD6	0.162	~p_tau+(1|manifest)+cngeneson+pc_mito+brain_region+Sex	0.687	3244.000
ENSG00000268043	0.001	0.151	0.228	0.074	NBPF12	0.031	~p_tau+(1|manifest)+cngeneson+pc_mito+brain_region+Sex	0.210	5208.000
ENSG00000120008	0.005	0.151	0.232	0.069	WDR11	0.062	~p_tau+(1|manifest)+cngeneson+pc_mito+brain_region+Sex	0.580	3616.000
ENSG00000139990	0.025	0.151	0.294	0.007	DCAF5	0.147	~p_tau+(1|manifest)+cngeneson+pc_mito+brain_region+Sex	0.734	3103.000
ENSG00000110031	0.058	0.151	0.424	-0.123	LPXN	0.230	~p_tau+(1|manifest)+cngeneson+pc_mito+brain_region+Sex	1.521	1636.000
ENSG00000083223	0.331	0.151	0.373	-0.072	TUT7	0.588	~p_tau+(1|manifest)+cngeneson+pc_mito+brain_region+Sex	1.575	1589.000
ENSG00000172349	0.029	0.151	0.305	-0.004	IL16	0.157	~p_tau+(1|manifest)+cngeneson+pc_mito+brain_region+Sex	1.047	2354.000
ENSG00000116754	0.195	0.150	0.323	-0.022	SRSF11	0.437	~p_tau+(1|manifest)+cngeneson+pc_mito+brain_region+Sex	1.034	2372.000
ENSG00000086619	0.329	0.150	0.355	-0.054	ERO1B	0.587	~p_tau+(1|manifest)+cngeneson+pc_mito+brain_region+Sex	1.196	2088.000
ENSG00000131374	0.036	0.150	0.259	0.041	TBC1D5	0.177	~p_tau+(1|manifest)+cngeneson+pc_mito+brain_region+Sex	2.402	1018.000
ENSG00000250479	0.009	0.150	0.277	0.023	CHCHD10	0.085	~p_tau+(1|manifest)+cngeneson+pc_mito+brain_region+Sex	1.374	1797.000
ENSG00000139132	0.134	0.149	0.292	0.007	FGD4	0.355	~p_tau+(1|manifest)+cngeneson+pc_mito+brain_region+Sex	3.862	554.000
ENSG00000101439	0.341	0.149	0.393	-0.094	CST3	0.597	~p_tau+(1|manifest)+cngeneson+pc_mito+brain_region+Sex	1.812	1384.000
ENSG00000164587	0.152	0.149	0.364	-0.065	RPS14	0.380	~p_tau+(1|manifest)+cngeneson+pc_mito+brain_region+Sex	1.732	1431.000
ENSG00000138468	0.182	0.149	0.303	-0.005	SENP7	0.421	~p_tau+(1|manifest)+cngeneson+pc_mito+brain_region+Sex	0.769	3012.000
ENSG00000117335	0.001	0.149	0.243	0.055	CD46	0.033	~p_tau+(1|manifest)+cngeneson+pc_mito+brain_region+Sex	0.242	5103.000
ENSG00000114013	0.633	0.149	0.483	-0.185	CD86	0.807	~p_tau+(1|manifest)+cngeneson+pc_mito+brain_region+Sex	3.758	573.000
ENSG00000144524	0.000	0.148	0.224	0.072	COPS7B	0.015	~p_tau+(1|manifest)+cngeneson+pc_mito+brain_region+Sex	0.349	4586.000
ENSG00000198380	0.027	0.148	0.278	0.018	GFPT1	0.151	~p_tau+(1|manifest)+cngeneson+pc_mito+brain_region+Sex	0.781	2984.000
ENSG00000165233	0.003	0.148	0.257	0.039	CARD19	0.050	~p_tau+(1|manifest)+cngeneson+pc_mito+brain_region+Sex	1.057	2334.000
ENSG00000169372	0.198	0.148	0.307	-0.011	CRADD	0.440	~p_tau+(1|manifest)+cngeneson+pc_mito+brain_region+Sex	0.524	3820.000
ENSG00000163235	0.084	0.148	0.279	0.017	TGFA	0.276	~p_tau+(1|manifest)+cngeneson+pc_mito+brain_region+Sex	0.625	3445.000
ENSG00000141279	0.081	0.147	0.302	-0.007	NPEPPS	0.271	~p_tau+(1|manifest)+cngeneson+pc_mito+brain_region+Sex	1.083	2282.000
ENSG00000120690	0.030	0.147	0.297	-0.002	ELF1	0.161	~p_tau+(1|manifest)+cngeneson+pc_mito+brain_region+Sex	1.171	2123.000
ENSG00000140264	0.761	0.147	0.562	-0.268	SERF2	0.880	~p_tau+(1|manifest)+cngeneson+pc_mito+brain_region+Sex	7.811	168.000
ENSG00000132323	0.022	0.147	0.299	-0.005	ILKAP	0.139	~p_tau+(1|manifest)+cngeneson+pc_mito+brain_region+Sex	0.607	3507.000
ENSG00000158864	0.001	0.147	0.237	0.056	NDUFS2	0.031	~p_tau+(1|manifest)+cngeneson+pc_mito+brain_region+Sex	0.260	5025.000
ENSG00000183597	0.182	0.147	0.351	-0.058	TANGO2	0.421	~p_tau+(1|manifest)+cngeneson+pc_mito+brain_region+Sex	1.876	1332.000
ENSG00000103024	0.068	0.146	0.359	-0.066	NME3	0.246	~p_tau+(1|manifest)+cngeneson+pc_mito+brain_region+Sex	2.005	1244.000
ENSG00000135709	0.009	0.146	0.284	0.009	KIAA0513	0.084	~p_tau+(1|manifest)+cngeneson+pc_mito+brain_region+Sex	0.655	3341.000
ENSG00000112232	0.456	0.146	0.408	-0.115	KHDRBS2	0.689	~p_tau+(1|manifest)+cngeneson+pc_mito+brain_region+Sex	0.867	2752.000
ENSG00000182022	0.789	0.146	0.676	-0.384	CHST15	0.896	~p_tau+(1|manifest)+cngeneson+pc_mito+brain_region+Sex	2.284	1082.000
ENSG00000187257	0.004	0.146	0.230	0.062	RSBN1L	0.061	~p_tau+(1|manifest)+cngeneson+pc_mito+brain_region+Sex	0.518	3844.000
ENSG00000105983	0.009	0.146	0.241	0.051	LMBR1	0.085	~p_tau+(1|manifest)+cngeneson+pc_mito+brain_region+Sex	0.353	4560.000
ENSG00000144935	0.054	0.146	0.285	0.007	TRPC1	0.222	~p_tau+(1|manifest)+cngeneson+pc_mito+brain_region+Sex	0.665	3313.000
ENSG00000093144	0.330	0.146	0.335	-0.044	ECHDC1	0.588	~p_tau+(1|manifest)+cngeneson+pc_mito+brain_region+Sex	0.820	2888.000
ENSG00000002016	0.001	0.145	0.230	0.061	RAD52	0.028	~p_tau+(1|manifest)+cngeneson+pc_mito+brain_region+Sex	0.490	3985.000
ENSG00000124226	0.040	0.145	0.265	0.025	RNF114	0.188	~p_tau+(1|manifest)+cngeneson+pc_mito+brain_region+Sex	1.109	2237.000
ENSG00000101290	0.007	0.145	0.238	0.053	CDS2	0.075	~p_tau+(1|manifest)+cngeneson+pc_mito+brain_region+Sex	0.671	3293.000
ENSG00000131469	0.228	0.145	0.471	-0.181	RPL27	0.476	~p_tau+(1|manifest)+cngeneson+pc_mito+brain_region+Sex	2.072	1203.000
ENSG00000165516	0.004	0.145	0.244	0.046	KLHDC2	0.060	~p_tau+(1|manifest)+cngeneson+pc_mito+brain_region+Sex	0.575	3632.000
ENSG00000175470	0.002	0.145	0.231	0.059	PPP2R2D	0.043	~p_tau+(1|manifest)+cngeneson+pc_mito+brain_region+Sex	0.196	5241.000
ENSG00000164985	0.034	0.145	0.298	-0.008	PSIP1	0.171	~p_tau+(1|manifest)+cngeneson+pc_mito+brain_region+Sex	1.296	1921.000
ENSG00000106459	0.259	0.145	0.347	-0.057	NRF1	0.513	~p_tau+(1|manifest)+cngeneson+pc_mito+brain_region+Sex	1.179	2110.000
ENSG00000120896	0.018	0.145	0.280	0.009	SORBS3	0.123	~p_tau+(1|manifest)+cngeneson+pc_mito+brain_region+Sex	1.020	2395.000
ENSG00000106952	0.002	0.144	0.232	0.057	TNFSF8	0.044	~p_tau+(1|manifest)+cngeneson+pc_mito+brain_region+Sex	0.231	5137.000
ENSG00000182287	0.001	0.144	0.245	0.044	AP1S2	0.028	~p_tau+(1|manifest)+cngeneson+pc_mito+brain_region+Sex	0.564	3669.000
ENSG00000058272	0.103	0.144	0.325	-0.036	PPP1R12A	0.307	~p_tau+(1|manifest)+cngeneson+pc_mito+brain_region+Sex	1.014	2409.000
ENSG00000123384	0.137	0.144	0.374	-0.085	LRP1	0.358	~p_tau+(1|manifest)+cngeneson+pc_mito+brain_region+Sex	1.699	1454.000
ENSG00000163328	0.363	0.144	0.463	-0.175	GPR155	0.617	~p_tau+(1|manifest)+cngeneson+pc_mito+brain_region+Sex	2.377	1029.000
ENSG00000185787	0.006	0.144	0.259	0.030	MORF4L1	0.073	~p_tau+(1|manifest)+cngeneson+pc_mito+brain_region+Sex	0.860	2770.000
ENSG00000180354	0.127	0.144	0.319	-0.031	MTURN	0.345	~p_tau+(1|manifest)+cngeneson+pc_mito+brain_region+Sex	1.479	1684.000
ENSG00000136111	0.779	0.144	0.548	-0.260	TBC1D4	0.890	~p_tau+(1|manifest)+cngeneson+pc_mito+brain_region+Sex	3.958	532.000
ENSG00000100813	0.056	0.144	0.302	-0.013	ACIN1	0.225	~p_tau+(1|manifest)+cngeneson+pc_mito+brain_region+Sex	0.363	4511.000
ENSG00000161921	0.275	0.144	0.328	-0.040	CXCL16	0.529	~p_tau+(1|manifest)+cngeneson+pc_mito+brain_region+Sex	1.411	1765.000
ENSG00000198586	0.198	0.144	0.302	-0.014	TLK1	0.440	~p_tau+(1|manifest)+cngeneson+pc_mito+brain_region+Sex	0.786	2973.000
ENSG00000184207	0.069	0.144	0.304	-0.016	PGP	0.248	~p_tau+(1|manifest)+cngeneson+pc_mito+brain_region+Sex	1.712	1447.000
ENSG00000187109	0.149	0.144	0.326	-0.039	NAP1L1	0.375	~p_tau+(1|manifest)+cngeneson+pc_mito+brain_region+Sex	0.926	2603.000
ENSG00000064115	0.114	0.144	0.284	0.003	TM7SF3	0.327	~p_tau+(1|manifest)+cngeneson+pc_mito+brain_region+Sex	1.113	2231.000
ENSG00000153201	0.244	0.143	0.378	-0.092	RANBP2	0.495	~p_tau+(1|manifest)+cngeneson+pc_mito+brain_region+Sex	1.046	2357.000
ENSG00000075303	0.002	0.143	0.250	0.037	SLC25A40	0.038	~p_tau+(1|manifest)+cngeneson+pc_mito+brain_region+Sex	0.267	4982.000
ENSG00000135951	0.084	0.143	0.287	-0.001	TSGA10	0.276	~p_tau+(1|manifest)+cngeneson+pc_mito+brain_region+Sex	0.737	3093.000
ENSG00000174720	0.047	0.143	0.280	0.006	LARP7	0.203	~p_tau+(1|manifest)+cngeneson+pc_mito+brain_region+Sex	0.592	3567.000
ENSG00000156232	0.001	0.143	0.218	0.068	WHAMM	0.036	~p_tau+(1|manifest)+cngeneson+pc_mito+brain_region+Sex	0.202	5226.000
ENSG00000138413	0.052	0.143	0.325	-0.040	IDH1	0.217	~p_tau+(1|manifest)+cngeneson+pc_mito+brain_region+Sex	1.385	1784.000
ENSG00000087095	0.290	0.143	0.343	-0.058	NLK	0.543	~p_tau+(1|manifest)+cngeneson+pc_mito+brain_region+Sex	1.008	2420.000
ENSG00000168944	0.000	0.142	0.227	0.058	CEP120	0.020	~p_tau+(1|manifest)+cngeneson+pc_mito+brain_region+Sex	0.226	5154.000
ENSG00000185215	0.232	0.142	0.374	-0.089	TNFAIP2	0.481	~p_tau+(1|manifest)+cngeneson+pc_mito+brain_region+Sex	3.233	704.000
ENSG00000130775	0.513	0.142	0.501	-0.217	THEMIS2	0.730	~p_tau+(1|manifest)+cngeneson+pc_mito+brain_region+Sex	6.351	248.000
ENSG00000165476	0.059	0.142	0.259	0.025	REEP3	0.230	~p_tau+(1|manifest)+cngeneson+pc_mito+brain_region+Sex	0.532	3792.000
ENSG00000167468	0.067	0.142	0.319	-0.036	GPX4	0.245	~p_tau+(1|manifest)+cngeneson+pc_mito+brain_region+Sex	1.734	1429.000
ENSG00000138744	0.003	0.142	0.231	0.052	NAAA	0.054	~p_tau+(1|manifest)+cngeneson+pc_mito+brain_region+Sex	0.497	3948.000
ENSG00000185158	0.017	0.141	0.258	0.025	LRRC37B	0.119	~p_tau+(1|manifest)+cngeneson+pc_mito+brain_region+Sex	0.626	3440.000
ENSG00000064652	0.474	0.141	0.375	-0.092	SNX24	0.703	~p_tau+(1|manifest)+cngeneson+pc_mito+brain_region+Sex	1.709	1448.000
ENSG00000105928	0.022	0.141	0.250	0.032	GSDME	0.139	~p_tau+(1|manifest)+cngeneson+pc_mito+brain_region+Sex	0.569	3653.000
ENSG00000101849	0.319	0.141	0.343	-0.061	TBL1X	0.575	~p_tau+(1|manifest)+cngeneson+pc_mito+brain_region+Sex	1.100	2252.000
ENSG00000023287	0.512	0.140	0.403	-0.122	RB1CC1	0.730	~p_tau+(1|manifest)+cngeneson+pc_mito+brain_region+Sex	2.088	1194.000
ENSG00000167766	0.055	0.140	0.316	-0.035	ZNF83	0.224	~p_tau+(1|manifest)+cngeneson+pc_mito+brain_region+Sex	0.819	2891.000
ENSG00000163814	0.134	0.140	0.291	-0.011	CDCP1	0.355	~p_tau+(1|manifest)+cngeneson+pc_mito+brain_region+Sex	2.880	806.000
ENSG00000059758	0.086	0.140	0.274	0.006	CDK17	0.280	~p_tau+(1|manifest)+cngeneson+pc_mito+brain_region+Sex	0.956	2534.000
ENSG00000164754	0.037	0.140	0.263	0.017	RAD21	0.181	~p_tau+(1|manifest)+cngeneson+pc_mito+brain_region+Sex	0.788	2964.000
ENSG00000172086	0.214	0.140	0.352	-0.072	KRCC1	0.458	~p_tau+(1|manifest)+cngeneson+pc_mito+brain_region+Sex	0.852	2788.000
ENSG00000154144	0.064	0.140	0.320	-0.041	TBRG1	0.241	~p_tau+(1|manifest)+cngeneson+pc_mito+brain_region+Sex	0.840	2828.000
ENSG00000009790	0.334	0.139	0.406	-0.127	TRAF3IP3	0.591	~p_tau+(1|manifest)+cngeneson+pc_mito+brain_region+Sex	1.936	1292.000
ENSG00000100811	0.060	0.139	0.260	0.019	YY1	0.233	~p_tau+(1|manifest)+cngeneson+pc_mito+brain_region+Sex	0.264	5001.000
ENSG00000180228	0.037	0.139	0.271	0.008	PRKRA	0.179	~p_tau+(1|manifest)+cngeneson+pc_mito+brain_region+Sex	0.521	3833.000
ENSG00000115839	0.167	0.139	0.278	0.000	RAB3GAP1	0.400	~p_tau+(1|manifest)+cngeneson+pc_mito+brain_region+Sex	0.500	3927.000
ENSG00000169714	0.028	0.139	0.262	0.017	CNBP	0.154	~p_tau+(1|manifest)+cngeneson+pc_mito+brain_region+Sex	0.889	2692.000
ENSG00000106733	0.039	0.139	0.284	-0.006	NMRK1	0.184	~p_tau+(1|manifest)+cngeneson+pc_mito+brain_region+Sex	0.954	2537.000
ENSG00000172171	0.086	0.139	0.275	0.002	TEFM	0.279	~p_tau+(1|manifest)+cngeneson+pc_mito+brain_region+Sex	1.049	2348.000
ENSG00000130985	0.015	0.139	0.251	0.026	UBA1	0.110	~p_tau+(1|manifest)+cngeneson+pc_mito+brain_region+Sex	0.696	3219.000
ENSG00000185129	0.009	0.139	0.281	-0.004	PURA	0.083	~p_tau+(1|manifest)+cngeneson+pc_mito+brain_region+Sex	0.437	4190.000
ENSG00000198668	0.426	0.139	0.348	-0.071	CALM1	0.669	~p_tau+(1|manifest)+cngeneson+pc_mito+brain_region+Sex	2.580	940.000
ENSG00000166801	0.320	0.139	0.351	-0.074	FAM111A	0.577	~p_tau+(1|manifest)+cngeneson+pc_mito+brain_region+Sex	1.153	2158.000
ENSG00000128829	0.035	0.138	0.246	0.030	EIF2AK4	0.176	~p_tau+(1|manifest)+cngeneson+pc_mito+brain_region+Sex	0.241	5105.000
ENSG00000164292	0.099	0.138	0.293	-0.017	RHOBTB3	0.300	~p_tau+(1|manifest)+cngeneson+pc_mito+brain_region+Sex	1.577	1584.000
ENSG00000119041	0.201	0.138	0.308	-0.031	GTF3C3	0.444	~p_tau+(1|manifest)+cngeneson+pc_mito+brain_region+Sex	1.127	2200.000
ENSG00000032219	0.464	0.138	0.399	-0.122	ARID4A	0.696	~p_tau+(1|manifest)+cngeneson+pc_mito+brain_region+Sex	2.984	775.000
ENSG00000179832	0.019	0.138	0.232	0.044	MROH1	0.127	~p_tau+(1|manifest)+cngeneson+pc_mito+brain_region+Sex	0.736	3095.000
ENSG00000150681	0.134	0.138	0.357	-0.081	RGS18	0.354	~p_tau+(1|manifest)+cngeneson+pc_mito+brain_region+Sex	1.691	1460.000
ENSG00000106524	0.000	0.138	0.272	0.004	ANKMY2	0.019	~p_tau+(1|manifest)+cngeneson+pc_mito+brain_region+Sex	1.466	1700.000
ENSG00000137710	0.020	0.138	0.248	0.028	RDX	0.130	~p_tau+(1|manifest)+cngeneson+pc_mito+brain_region+Sex	0.341	4624.000
ENSG00000149212	0.830	0.137	0.648	-0.373	SESN3	0.921	~p_tau+(1|manifest)+cngeneson+pc_mito+brain_region+Sex	7.939	162.000
ENSG00000019991	0.149	0.137	0.295	-0.020	HGF	0.376	~p_tau+(1|manifest)+cngeneson+pc_mito+brain_region+Sex	1.043	2361.000
ENSG00000019995	0.041	0.137	0.256	0.018	ZRANB1	0.189	~p_tau+(1|manifest)+cngeneson+pc_mito+brain_region+Sex	0.297	4827.000
ENSG00000121579	0.177	0.137	0.295	-0.020	NAA50	0.414	~p_tau+(1|manifest)+cngeneson+pc_mito+brain_region+Sex	0.671	3291.000
ENSG00000185359	0.005	0.137	0.232	0.042	HGS	0.066	~p_tau+(1|manifest)+cngeneson+pc_mito+brain_region+Sex	0.299	4818.000
ENSG00000167107	0.154	0.137	0.295	-0.021	ACSF2	0.381	~p_tau+(1|manifest)+cngeneson+pc_mito+brain_region+Sex	1.280	1948.000
ENSG00000138641	0.314	0.137	0.337	-0.063	HERC3	0.570	~p_tau+(1|manifest)+cngeneson+pc_mito+brain_region+Sex	1.174	2115.000
ENSG00000169193	0.028	0.137	0.243	0.031	CCDC126	0.153	~p_tau+(1|manifest)+cngeneson+pc_mito+brain_region+Sex	0.498	3943.000
ENSG00000032742	0.003	0.137	0.210	0.063	IFT88	0.054	~p_tau+(1|manifest)+cngeneson+pc_mito+brain_region+Sex	0.375	4458.000
ENSG00000140749	0.388	0.137	0.427	-0.154	IGSF6	0.636	~p_tau+(1|manifest)+cngeneson+pc_mito+brain_region+Sex	3.040	757.000
ENSG00000089041	0.526	0.136	0.438	-0.165	P2RX7	0.738	~p_tau+(1|manifest)+cngeneson+pc_mito+brain_region+Sex	0.639	3394.000
ENSG00000100650	0.326	0.136	0.378	-0.105	SRSF5	0.583	~p_tau+(1|manifest)+cngeneson+pc_mito+brain_region+Sex	2.340	1048.000
ENSG00000115170	0.003	0.136	0.227	0.045	ACVR1	0.055	~p_tau+(1|manifest)+cngeneson+pc_mito+brain_region+Sex	0.297	4830.000

ENSG00000027075	0.609	0.136	0.427	-0.155	PRKCH	0.792	~p_tau+(1|manifest)+cngeneson+pc_mito+brain_region+Sex	1.589	1569.000
ENSG00000123268	0.029	0.136	0.230	0.042	ATF1	0.157	~p_tau+(1|manifest)+cngeneson+pc_mito+brain_region+Sex	0.087	5456.000
ENSG00000180881	0.100	0.136	0.263	0.009	CAPS2	0.301	~p_tau+(1|manifest)+cngeneson+pc_mito+brain_region+Sex	0.498	3940.000
ENSG00000077254	0.053	0.136	0.254	0.017	USP33	0.218	~p_tau+(1|manifest)+cngeneson+pc_mito+brain_region+Sex	0.420	4270.000
ENSG00000122008	0.034	0.136	0.270	0.001	POLK	0.171	~p_tau+(1|manifest)+cngeneson+pc_mito+brain_region+Sex	0.631	3416.000
ENSG00000002834	0.125	0.135	0.319	-0.048	LASP1	0.342	~p_tau+(1|manifest)+cngeneson+pc_mito+brain_region+Sex	0.999	2439.000
ENSG00000173960	0.034	0.135	0.249	0.021	UBXN2A	0.172	~p_tau+(1|manifest)+cngeneson+pc_mito+brain_region+Sex	0.498	3942.000
ENSG00000072422	0.009	0.135	0.278	-0.008	RHOBTB1	0.084	~p_tau+(1|manifest)+cngeneson+pc_mito+brain_region+Sex	0.998	2441.000
ENSG00000001084	0.095	0.135	0.286	-0.016	GCLC	0.293	~p_tau+(1|manifest)+cngeneson+pc_mito+brain_region+Sex	1.299	1915.000
ENSG00000005893	0.017	0.135	0.231	0.039	LAMP2	0.119	~p_tau+(1|manifest)+cngeneson+pc_mito+brain_region+Sex	0.383	4431.000
ENSG00000148303	0.246	0.135	0.368	-0.098	RPL7A	0.497	~p_tau+(1|manifest)+cngeneson+pc_mito+brain_region+Sex	2.588	934.000
ENSG00000160199	0.009	0.135	0.243	0.027	PKNOX1	0.084	~p_tau+(1|manifest)+cngeneson+pc_mito+brain_region+Sex	0.370	4475.000
ENSG00000103769	0.360	0.135	0.411	-0.142	RAB11A	0.614	~p_tau+(1|manifest)+cngeneson+pc_mito+brain_region+Sex	2.648	912.000
ENSG00000013374	0.010	0.135	0.239	0.030	NUB1	0.091	~p_tau+(1|manifest)+cngeneson+pc_mito+brain_region+Sex	0.575	3630.000
ENSG00000104660	0.031	0.135	0.259	0.010	LEPROTL1	0.163	~p_tau+(1|manifest)+cngeneson+pc_mito+brain_region+Sex	0.287	4884.000
ENSG00000221983	0.378	0.134	0.531	-0.262	UBA52	0.629	~p_tau+(1|manifest)+cngeneson+pc_mito+brain_region+Sex	3.383	659.000
ENSG00000131018	0.063	0.134	0.271	-0.002	SYNE1	0.240	~p_tau+(1|manifest)+cngeneson+pc_mito+brain_region+Sex	0.731	3112.000
ENSG00000164306	0.070	0.134	0.306	-0.037	PRIMPOL	0.250	~p_tau+(1|manifest)+cngeneson+pc_mito+brain_region+Sex	1.049	2349.000
ENSG00000090273	0.000	0.134	0.298	-0.030	NUDC	0.015	~p_tau+(1|manifest)+cngeneson+pc_mito+brain_region+Sex	1.073	2302.000
ENSG00000107263	0.717	0.134	0.454	-0.185	RAPGEF1	0.859	~p_tau+(1|manifest)+cngeneson+pc_mito+brain_region+Sex	10.797	91.000
ENSG00000001461	0.024	0.134	0.246	0.022	NIPAL3	0.142	~p_tau+(1|manifest)+cngeneson+pc_mito+brain_region+Sex	0.346	4601.000
ENSG00000110880	0.591	0.134	0.421	-0.152	CORO1C	0.782	~p_tau+(1|manifest)+cngeneson+pc_mito+brain_region+Sex	6.068	277.000
ENSG00000122257	0.093	0.134	0.275	-0.006	RBBP6	0.290	~p_tau+(1|manifest)+cngeneson+pc_mito+brain_region+Sex	0.507	3897.000
ENSG00000144959	0.143	0.134	0.270	-0.002	NCEH1	0.366	~p_tau+(1|manifest)+cngeneson+pc_mito+brain_region+Sex	0.843	2812.000
ENSG00000135596	0.068	0.134	0.268	-0.001	MICAL1	0.246	~p_tau+(1|manifest)+cngeneson+pc_mito+brain_region+Sex	0.763	3031.000
ENSG00000070214	0.138	0.134	0.272	-0.004	SLC44A1	0.360	~p_tau+(1|manifest)+cngeneson+pc_mito+brain_region+Sex	0.713	3162.000
ENSG00000134815	0.006	0.134	0.231	0.036	DHX34	0.073	~p_tau+(1|manifest)+cngeneson+pc_mito+brain_region+Sex	0.217	5178.000
ENSG00000150756	0.012	0.134	0.275	-0.008	ATP5CKMT	0.098	~p_tau+(1|manifest)+cngeneson+pc_mito+brain_region+Sex	0.824	2880.000
ENSG00000162923	0.139	0.134	0.304	-0.036	WDR26	0.362	~p_tau+(1|manifest)+cngeneson+pc_mito+brain_region+Sex	1.032	2378.000
ENSG00000115525	0.021	0.133	0.241	0.026	ST3GAL5	0.134	~p_tau+(1|manifest)+cngeneson+pc_mito+brain_region+Sex	0.965	2513.000
ENSG00000113013	0.298	0.133	0.416	-0.149	HSPA9	0.553	~p_tau+(1|manifest)+cngeneson+pc_mito+brain_region+Sex	2.219	1124.000
ENSG00000198879	0.049	0.133	0.296	-0.030	SFMBT2	0.208	~p_tau+(1|manifest)+cngeneson+pc_mito+brain_region+Sex	6.541	238.000
ENSG00000158092	0.402	0.133	0.328	-0.061	NCK1	0.649	~p_tau+(1|manifest)+cngeneson+pc_mito+brain_region+Sex	0.311	4773.000
ENSG00000166974	0.202	0.133	0.276	-0.010	MAPRE2	0.444	~p_tau+(1|manifest)+cngeneson+pc_mito+brain_region+Sex	0.968	2501.000
ENSG00000006194	0.007	0.133	0.284	-0.017	ZNF263	0.076	~p_tau+(1|manifest)+cngeneson+pc_mito+brain_region+Sex	1.209	2066.000
ENSG00000110344	0.109	0.133	0.293	-0.028	UBE4A	0.317	~p_tau+(1|manifest)+cngeneson+pc_mito+brain_region+Sex	0.630	3420.000
ENSG00000128951	0.061	0.133	0.308	-0.043	DUT	0.236	~p_tau+(1|manifest)+cngeneson+pc_mito+brain_region+Sex	1.295	1922.000
ENSG00000127184	0.056	0.133	0.303	-0.038	COX7C	0.226	~p_tau+(1|manifest)+cngeneson+pc_mito+brain_region+Sex	1.489	1676.000
ENSG00000120457	0.025	0.132	0.276	-0.012	KCNJ5	0.146	~p_tau+(1|manifest)+cngeneson+pc_mito+brain_region+Sex	1.668	1478.000
ENSG00000105829	0.023	0.132	0.292	-0.028	BET1	0.140	~p_tau+(1|manifest)+cngeneson+pc_mito+brain_region+Sex	1.324	1873.000
ENSG00000198121	0.841	0.132	0.572	-0.309	LPAR1	0.925	~p_tau+(1|manifest)+cngeneson+pc_mito+brain_region+Sex	3.906	542.000
ENSG00000145675	0.193	0.132	0.271	-0.008	PIK3R1	0.434	~p_tau+(1|manifest)+cngeneson+pc_mito+brain_region+Sex	2.363	1035.000
ENSG00000165275	0.027	0.131	0.277	-0.014	TRMT10B	0.151	~p_tau+(1|manifest)+cngeneson+pc_mito+brain_region+Sex	0.918	2628.000
ENSG00000080371	0.004	0.131	0.252	0.011	RAB21	0.058	~p_tau+(1|manifest)+cngeneson+pc_mito+brain_region+Sex	0.358	4535.000
ENSG00000083896	0.292	0.131	0.305	-0.042	YTHDC1	0.545	~p_tau+(1|manifest)+cngeneson+pc_mito+brain_region+Sex	1.007	2421.000
ENSG00000182108	0.001	0.131	0.236	0.026	DEXI	0.033	~p_tau+(1|manifest)+cngeneson+pc_mito+brain_region+Sex	0.608	3503.000
ENSG00000042832	0.019	0.131	0.299	-0.038	TG	0.127	~p_tau+(1|manifest)+cngeneson+pc_mito+brain_region+Sex	2.663	905.000
ENSG00000066294	0.592	0.131	0.474	-0.212	CD84	0.783	~p_tau+(1|manifest)+cngeneson+pc_mito+brain_region+Sex	2.048	1216.000
ENSG00000092929	0.010	0.130	0.215	0.045	UNC13D	0.089	~p_tau+(1|manifest)+cngeneson+pc_mito+brain_region+Sex	0.421	4261.000
ENSG00000185278	0.070	0.130	0.251	0.009	ZBTB37	0.250	~p_tau+(1|manifest)+cngeneson+pc_mito+brain_region+Sex	0.275	4950.000
ENSG00000112419	0.782	0.130	0.497	-0.237	PHACTR2	0.891	~p_tau+(1|manifest)+cngeneson+pc_mito+brain_region+Sex	1.159	2144.000
ENSG00000255302	0.030	0.129	0.284	-0.026	EID1	0.160	~p_tau+(1|manifest)+cngeneson+pc_mito+brain_region+Sex	1.673	1475.000
ENSG00000106771	0.115	0.129	0.276	-0.018	TMEM245	0.328	~p_tau+(1|manifest)+cngeneson+pc_mito+brain_region+Sex	0.330	4681.000
ENSG00000127951	0.467	0.129	0.417	-0.159	FGL2	0.698	~p_tau+(1|manifest)+cngeneson+pc_mito+brain_region+Sex	2.583	939.000
ENSG00000198722	0.016	0.129	0.216	0.042	UNC13B	0.117	~p_tau+(1|manifest)+cngeneson+pc_mito+brain_region+Sex	0.509	3886.000
ENSG00000186314	0.311	0.129	0.297	-0.039	PRELID2	0.567	~p_tau+(1|manifest)+cngeneson+pc_mito+brain_region+Sex	1.033	2373.000
ENSG00000140470	0.579	0.129	0.390	-0.132	ADAMTS17	0.775	~p_tau+(1|manifest)+cngeneson+pc_mito+brain_region+Sex	6.285	255.000
ENSG00000150782	0.596	0.128	0.475	-0.219	IL18	0.785	~p_tau+(1|manifest)+cngeneson+pc_mito+brain_region+Sex	3.877	549.000
ENSG00000106049	0.165	0.128	0.261	-0.005	HIBADH	0.396	~p_tau+(1|manifest)+cngeneson+pc_mito+brain_region+Sex	0.546	3737.000
ENSG00000120742	0.052	0.128	0.283	-0.027	SERP1	0.217	~p_tau+(1|manifest)+cngeneson+pc_mito+brain_region+Sex	0.868	2747.000
ENSG00000138434	0.103	0.128	0.246	0.010	ITPRID2	0.307	~p_tau+(1|manifest)+cngeneson+pc_mito+brain_region+Sex	0.415	4292.000
ENSG00000138834	0.061	0.128	0.282	-0.026	MAPK8IP3	0.237	~p_tau+(1|manifest)+cngeneson+pc_mito+brain_region+Sex	0.952	2543.000
ENSG00000063854	0.008	0.128	0.243	0.013	HAGH	0.080	~p_tau+(1|manifest)+cngeneson+pc_mito+brain_region+Sex	0.582	3606.000
ENSG00000213551	0.025	0.128	0.224	0.032	DNAJC9	0.146	~p_tau+(1|manifest)+cngeneson+pc_mito+brain_region+Sex	0.383	4433.000
ENSG00000173992	0.016	0.128	0.271	-0.016	CCS	0.115	~p_tau+(1|manifest)+cngeneson+pc_mito+brain_region+Sex	1.313	1888.000
ENSG00000164506	0.446	0.128	0.326	-0.071	STXBP5	0.683	~p_tau+(1|manifest)+cngeneson+pc_mito+brain_region+Sex	1.088	2277.000
ENSG00000095066	0.004	0.128	0.222	0.033	HOOK2	0.057	~p_tau+(1|manifest)+cngeneson+pc_mito+brain_region+Sex	0.511	3877.000
ENSG00000187118	0.197	0.128	0.266	-0.011	CMC1	0.439	~p_tau+(1|manifest)+cngeneson+pc_mito+brain_region+Sex	0.304	4805.000
ENSG00000124588	0.041	0.127	0.258	-0.003	NQO2	0.191	~p_tau+(1|manifest)+cngeneson+pc_mito+brain_region+Sex	1.137	2185.000
ENSG00000198399	0.152	0.127	0.324	-0.069	ITSN2	0.380	~p_tau+(1|manifest)+cngeneson+pc_mito+brain_region+Sex	2.002	1246.000
ENSG00000075292	0.133	0.127	0.278	-0.023	ZNF638	0.354	~p_tau+(1|manifest)+cngeneson+pc_mito+brain_region+Sex	2.401	1019.000
ENSG00000068028	0.042	0.127	0.246	0.009	RASSF1	0.191	~p_tau+(1|manifest)+cngeneson+pc_mito+brain_region+Sex	0.533	3787.000
ENSG00000163703	0.030	0.127	0.304	-0.050	CRELD1	0.160	~p_tau+(1|manifest)+cngeneson+pc_mito+brain_region+Sex	1.337	1853.000
ENSG00000168066	0.228	0.127	0.346	-0.092	SF1	0.477	~p_tau+(1|manifest)+cngeneson+pc_mito+brain_region+Sex	2.257	1096.000
ENSG00000143947	0.731	0.127	0.447	-0.193	RPS27A	0.865	~p_tau+(1|manifest)+cngeneson+pc_mito+brain_region+Sex	4.008	519.000
ENSG00000152558	0.063	0.127	0.247	0.006	TMEM123	0.240	~p_tau+(1|manifest)+cngeneson+pc_mito+brain_region+Sex	0.459	4104.000
ENSG00000136451	0.001	0.127	0.240	0.013	VEZF1	0.026	~p_tau+(1|manifest)+cngeneson+pc_mito+brain_region+Sex	0.565	3665.000
ENSG00000153207	0.204	0.127	0.299	-0.046	AHCTF1	0.447	~p_tau+(1|manifest)+cngeneson+pc_mito+brain_region+Sex	0.914	2634.000
ENSG00000186350	0.016	0.126	0.263	-0.011	RXRA	0.117	~p_tau+(1|manifest)+cngeneson+pc_mito+brain_region+Sex	0.393	4390.000
ENSG00000128789	0.002	0.126	0.195	0.058	PSMG2	0.044	~p_tau+(1|manifest)+cngeneson+pc_mito+brain_region+Sex	0.170	5332.000
ENSG00000003400	0.044	0.126	0.298	-0.046	CASP10	0.196	~p_tau+(1|manifest)+cngeneson+pc_mito+brain_region+Sex	1.249	2001.000
ENSG00000070540	0.065	0.126	0.280	-0.029	WIPI1	0.243	~p_tau+(1|manifest)+cngeneson+pc_mito+brain_region+Sex	1.780	1402.000
ENSG00000116731	0.181	0.125	0.259	-0.008	PRDM2	0.420	~p_tau+(1|manifest)+cngeneson+pc_mito+brain_region+Sex	0.340	4632.000
ENSG00000068323	0.015	0.125	0.222	0.029	TFE3	0.110	~p_tau+(1|manifest)+cngeneson+pc_mito+brain_region+Sex	0.175	5312.000
ENSG00000146830	0.015	0.125	0.228	0.023	GIGYF1	0.110	~p_tau+(1|manifest)+cngeneson+pc_mito+brain_region+Sex	0.485	4000.000
ENSG00000144040	0.012	0.125	0.201	0.049	SFXN5	0.099	~p_tau+(1|manifest)+cngeneson+pc_mito+brain_region+Sex	0.550	3724.000
ENSG00000109445	0.002	0.125	0.204	0.046	ZNF330	0.040	~p_tau+(1|manifest)+cngeneson+pc_mito+brain_region+Sex	0.656	3337.000
ENSG00000153029	0.004	0.125	0.212	0.037	MR1	0.057	~p_tau+(1|manifest)+cngeneson+pc_mito+brain_region+Sex	0.376	4451.000
ENSG00000198853	0.003	0.125	0.191	0.058	RUSC2	0.054	~p_tau+(1|manifest)+cngeneson+pc_mito+brain_region+Sex	0.363	4512.000
ENSG00000067955	0.007	0.125	0.197	0.052	CBFB	0.075	~p_tau+(1|manifest)+cngeneson+pc_mito+brain_region+Sex	0.556	3698.000
ENSG00000134245	0.824	0.125	0.542	-0.293	WNT2B	0.918	~p_tau+(1|manifest)+cngeneson+pc_mito+brain_region+Sex	3.150	723.000
ENSG00000172845	0.218	0.124	0.281	-0.033	SP3	0.463	~p_tau+(1|manifest)+cngeneson+pc_mito+brain_region+Sex	0.849	2794.000
ENSG00000105819	0.032	0.124	0.287	-0.039	PMPCB	0.167	~p_tau+(1|manifest)+cngeneson+pc_mito+brain_region+Sex	1.175	2114.000
ENSG00000241399	0.000	0.124	0.253	-0.005	CD302	0.015	~p_tau+(1|manifest)+cngeneson+pc_mito+brain_region+Sex	0.768	3016.000
ENSG00000142541	0.201	0.124	0.356	-0.109	RPL13A	0.444	~p_tau+(1|manifest)+cngeneson+pc_mito+brain_region+Sex	3.143	727.000
ENSG00000168175	0.059	0.124	0.256	-0.009	MAPK1IP1L	0.231	~p_tau+(1|manifest)+cngeneson+pc_mito+brain_region+Sex	0.504	3911.000
ENSG00000122565	0.005	0.124	0.203	0.044	CBX3	0.064	~p_tau+(1|manifest)+cngeneson+pc_mito+brain_region+Sex	0.308	4786.000
ENSG00000198740	0.574	0.124	0.390	-0.143	ZNF652	0.771	~p_tau+(1|manifest)+cngeneson+pc_mito+brain_region+Sex	1.856	1346.000
ENSG00000156170	0.159	0.124	0.287	-0.040	NDUFAF6	0.389	~p_tau+(1|manifest)+cngeneson+pc_mito+brain_region+Sex	1.003	2425.000
ENSG00000147853	0.018	0.124	0.271	-0.024	AK3	0.124	~p_tau+(1|manifest)+cngeneson+pc_mito+brain_region+Sex	0.795	2946.000
ENSG00000146457	0.087	0.123	0.268	-0.021	WTAP	0.280	~p_tau+(1|manifest)+cngeneson+pc_mito+brain_region+Sex	0.934	2583.000
ENSG00000266338	0.001	0.123	0.218	0.029	NBPF15	0.033	~p_tau+(1|manifest)+cngeneson+pc_mito+brain_region+Sex	0.509	3887.000
ENSG00000277443	0.714	0.123	0.494	-0.248	MARCKS	0.857	~p_tau+(1|manifest)+cngeneson+pc_mito+brain_region+Sex	4.311	473.000
ENSG00000130304	0.047	0.123	0.225	0.021	SLC27A1	0.203	~p_tau+(1|manifest)+cngeneson+pc_mito+brain_region+Sex	0.542	3755.000
ENSG00000236383	0.024	0.123	0.246	0.000	CCDC200	0.142	~p_tau+(1|manifest)+cngeneson+pc_mito+brain_region+Sex	1.154	2157.000
ENSG00000135390	0.144	0.123	0.312	-0.066	ATP5MC2	0.367	~p_tau+(1|manifest)+cngeneson+pc_mito+brain_region+Sex	2.031	1228.000
ENSG00000100083	0.217	0.123	0.294	-0.048	GGA1	0.461	~p_tau+(1|manifest)+cngeneson+pc_mito+brain_region+Sex	1.218	2052.000
ENSG00000125148	0.383	0.123	0.315	-0.070	MT2A	0.632	~p_tau+(1|manifest)+cngeneson+pc_mito+brain_region+Sex	6.337	249.000
ENSG00000130997	0.003	0.123	0.187	0.058	POLN	0.048	~p_tau+(1|manifest)+cngeneson+pc_mito+brain_region+Sex	0.162	5351.000
ENSG00000144028	0.004	0.123	0.219	0.026	SNRNP200	0.056	~p_tau+(1|manifest)+cngeneson+pc_mito+brain_region+Sex	0.593	3563.000
ENSG00000155189	0.658	0.122	0.420	-0.175	AGPAT5	0.823	~p_tau+(1|manifest)+cngeneson+pc_mito+brain_region+Sex	2.822	831.000
ENSG00000117410	0.044	0.122	0.326	-0.081	ATP6V0B	0.198	~p_tau+(1|manifest)+cngeneson+pc_mito+brain_region+Sex	1.126	2205.000
ENSG00000154589	0.759	0.122	0.446	-0.202	LY96	0.878	~p_tau+(1|manifest)+cngeneson+pc_mito+brain_region+Sex	1.990	1253.000
ENSG00000123124	0.377	0.122	0.335	-0.091	WWP1	0.628	~p_tau+(1|manifest)+cngeneson+pc_mito+brain_region+Sex	1.056	2336.000
ENSG00000120709	0.001	0.122	0.194	0.050	FAM53C	0.026	~p_tau+(1|manifest)+cngeneson+pc_mito+brain_region+Sex	0.256	5044.000
ENSG00000104518	0.178	0.122	0.293	-0.049	GSDMD	0.415	~p_tau+(1|manifest)+cngeneson+pc_mito+brain_region+Sex	1.358	1819.000
ENSG00000153786	0.005	0.122	0.196	0.048	ZDHHC7	0.068	~p_tau+(1|manifest)+cngeneson+pc_mito+brain_region+Sex	0.356	4543.000
ENSG00000135299	0.018	0.122	0.203	0.040	ANKRD6	0.124	~p_tau+(1|manifest)+cngeneson+pc_mito+brain_region+Sex	0.509	3884.000
ENSG00000053900	0.000	0.121	0.172	0.071	ANAPC4	0.015	~p_tau+(1|manifest)+cngeneson+pc_mito+brain_region+Sex	0.293	4848.000
ENSG00000115977	0.164	0.121	0.241	0.002	AAK1	0.396	~p_tau+(1|manifest)+cngeneson+pc_mito+brain_region+Sex	0.500	3928.000
ENSG00000204161	0.047	0.121	0.373	-0.131	TMEM273	0.202	~p_tau+(1|manifest)+cngeneson+pc_mito+brain_region+Sex	2.155	1149.000
ENSG00000100485	0.315	0.121	0.279	-0.037	SOS2	0.570	~p_tau+(1|manifest)+cngeneson+pc_mito+brain_region+Sex	1.031	2381.000
ENSG00000138639	0.140	0.121	0.266	-0.024	ARHGAP24	0.363	~p_tau+(1|manifest)+cngeneson+pc_mito+brain_region+Sex	9.808	113.000
ENSG00000095787	0.412	0.121	0.294	-0.052	WAC	0.657	~p_tau+(1|manifest)+cngeneson+pc_mito+brain_region+Sex	1.834	1361.000
ENSG00000163191	0.753	0.121	0.444	-0.203	S100A11	0.875	~p_tau+(1|manifest)+cngeneson+pc_mito+brain_region+Sex	7.668	179.000
ENSG00000023228	0.024	0.121	0.207	0.034	NDUFS1	0.142	~p_tau+(1|manifest)+cngeneson+pc_mito+brain_region+Sex	0.112	5443.000
ENSG00000167895	0.310	0.120	0.354	-0.113	TMC8	0.565	~p_tau+(1|manifest)+cngeneson+pc_mito+brain_region+Sex	1.914	1304.000
ENSG00000143889	0.046	0.120	0.224	0.016	HNRNPLL	0.201	~p_tau+(1|manifest)+cngeneson+pc_mito+brain_region+Sex	0.358	4533.000
ENSG00000142192	0.310	0.120	0.276	-0.036	APP	0.565	~p_tau+(1|manifest)+cngeneson+pc_mito+brain_region+Sex	1.087	2278.000
ENSG00000107242	0.188	0.120	0.414	-0.174	PIP5K1B	0.427	~p_tau+(1|manifest)+cngeneson+pc_mito+brain_region+Sex	1.953	1274.000
ENSG00000143442	0.324	0.120	0.280	-0.041	POGZ	0.581	~p_tau+(1|manifest)+cngeneson+pc_mito+brain_region+Sex	1.510	1652.000
ENSG00000131389	0.450	0.120	0.398	-0.158	SLC6A6	0.685	~p_tau+(1|manifest)+cngeneson+pc_mito+brain_region+Sex	1.883	1324.000
ENSG00000122566	0.290	0.120	0.317	-0.077	HNRNPA2B1	0.543	~p_tau+(1|manifest)+cngeneson+pc_mito+brain_region+Sex	3.663	605.000
ENSG00000081087	0.026	0.120	0.223	0.016	OSTM1	0.149	~p_tau+(1|manifest)+cngeneson+pc_mito+brain_region+Sex	0.390	4399.000
ENSG00000155970	0.327	0.120	0.273	-0.034	MICU3	0.585	~p_tau+(1|manifest)+cngeneson+pc_mito+brain_region+Sex	0.942	2568.000
ENSG00000129071	0.116	0.120	0.234	0.005	MBD4	0.330	~p_tau+(1|manifest)+cngeneson+pc_mito+brain_region+Sex	0.371	4469.000
ENSG00000171206	0.044	0.119	0.244	-0.005	TRIM8	0.197	~p_tau+(1|manifest)+cngeneson+pc_mito+brain_region+Sex	0.555	3700.000
ENSG00000104205	0.543	0.119	0.353	-0.115	SGK3	0.751	~p_tau+(1|manifest)+cngeneson+pc_mito+brain_region+Sex	1.586	1573.000
ENSG00000145936	0.120	0.119	0.378	-0.139	KCNMB1	0.336	~p_tau+(1|manifest)+cngeneson+pc_mito+brain_region+Sex	3.729	583.000
ENSG00000101558	0.686	0.119	0.394	-0.156	VAPA	0.841	~p_tau+(1|manifest)+cngeneson+pc_mito+brain_region+Sex	2.202	1129.000
ENSG00000122707	0.307	0.119	0.311	-0.073	RECK	0.563	~p_tau+(1|manifest)+cngeneson+pc_mito+brain_region+Sex	0.907	2658.000
ENSG00000139636	0.142	0.119	0.243	-0.005	LMBR1L	0.366	~p_tau+(1|manifest)+cngeneson+pc_mito+brain_region+Sex	0.810	2909.000
ENSG00000121316	0.004	0.119	0.212	0.026	PLBD1	0.057	~p_tau+(1|manifest)+cngeneson+pc_mito+brain_region+Sex	0.494	3960.000
ENSG00000130024	0.068	0.119	0.243	-0.005	PHF10	0.246	~p_tau+(1|manifest)+cngeneson+pc_mito+brain_region+Sex	0.887	2697.000
ENSG00000135047	0.033	0.119	0.236	0.002	CTSL	0.168	~p_tau+(1|manifest)+cngeneson+pc_mito+brain_region+Sex	1.049	2350.000
ENSG00000164040	0.018	0.119	0.212	0.026	PGRMC2	0.124	~p_tau+(1|manifest)+cngeneson+pc_mito+brain_region+Sex	0.375	4457.000
ENSG00000048405	0.209	0.119	0.279	-0.042	ZNF800	0.452	~p_tau+(1|manifest)+cngeneson+pc_mito+brain_region+Sex	0.784	2976.000
ENSG00000178252	0.010	0.119	0.205	0.032	WDR6	0.089	~p_tau+(1|manifest)+cngeneson+pc_mito+brain_region+Sex	0.333	4668.000
ENSG00000163291	0.047	0.119	0.250	-0.013	PAQR3	0.202	~p_tau+(1|manifest)+cngeneson+pc_mito+brain_region+Sex	1.241	2015.000
ENSG00000132965	0.014	0.119	0.278	-0.041	ALOX5AP	0.109	~p_tau+(1|manifest)+cngeneson+pc_mito+brain_region+Sex	1.031	2380.000
ENSG00000133315	0.127	0.118	0.235	0.002	MACROD1	0.345	~p_tau+(1|manifest)+cngeneson+pc_mito+brain_region+Sex	0.497	3945.000
ENSG00000110321	0.105	0.118	0.273	-0.037	EIF4G2	0.311	~p_tau+(1|manifest)+cngeneson+pc_mito+brain_region+Sex	0.601	3530.000
ENSG00000086730	0.276	0.118	0.383	-0.147	LAT2	0.529	~p_tau+(1|manifest)+cngeneson+pc_mito+brain_region+Sex	2.078	1198.000
ENSG00000117592	0.006	0.118	0.208	0.028	PRDX6	0.073	~p_tau+(1|manifest)+cngeneson+pc_mito+brain_region+Sex	0.472	4049.000
ENSG00000067560	0.058	0.118	0.243	-0.007	RHOA	0.228	~p_tau+(1|manifest)+cngeneson+pc_mito+brain_region+Sex	0.902	2665.000
ENSG00000010295	0.058	0.118	0.261	-0.026	IFFO1	0.229	~p_tau+(1|manifest)+cngeneson+pc_mito+brain_region+Sex	1.733	1430.000
ENSG00000104695	0.040	0.118	0.249	-0.014	PPP2CB	0.186	~p_tau+(1|manifest)+cngeneson+pc_mito+brain_region+Sex	0.404	4341.000
ENSG00000109686	0.014	0.117	0.281	-0.046	SH3D19	0.110	~p_tau+(1|manifest)+cngeneson+pc_mito+brain_region+Sex	1.212	2063.000
ENSG00000083750	0.053	0.117	0.234	0.000	RRAGB	0.218	~p_tau+(1|manifest)+cngeneson+pc_mito+brain_region+Sex	0.544	3752.000
ENSG00000089053	0.064	0.117	0.230	0.005	ANAPC5	0.242	~p_tau+(1|manifest)+cngeneson+pc_mito+brain_region+Sex	0.636	3403.000
ENSG00000144029	0.044	0.117	0.225	0.009	MRPS5	0.196	~p_tau+(1|manifest)+cngeneson+pc_mito+brain_region+Sex	0.178	5302.000
ENSG00000113312	0.008	0.117	0.202	0.032	TTC1	0.082	~p_tau+(1|manifest)+cngeneson+pc_mito+brain_region+Sex	0.347	4591.000
ENSG00000075884	0.125	0.117	0.238	-0.004	ARHGAP15	0.342	~p_tau+(1|manifest)+cngeneson+pc_mito+brain_region+Sex	6.863	213.000
ENSG00000241127	0.000	0.117	0.218	0.015	YAE1	0.015	~p_tau+(1|manifest)+cngeneson+pc_mito+brain_region+Sex	0.566	3660.000
ENSG00000108840	0.036	0.116	0.219	0.013	HDAC5	0.177	~p_tau+(1|manifest)+cngeneson+pc_mito+brain_region+Sex	0.727	3121.000
ENSG00000126456	0.021	0.116	0.226	0.006	IRF3	0.134	~p_tau+(1|manifest)+cngeneson+pc_mito+brain_region+Sex	0.893	2686.000
ENSG00000270629	0.001	0.116	0.190	0.043	NBPF14	0.037	~p_tau+(1|manifest)+cngeneson+pc_mito+brain_region+Sex	0.348	4587.000
ENSG00000104365	0.121	0.116	0.283	-0.051	IKBKB	0.336	~p_tau+(1|manifest)+cngeneson+pc_mito+brain_region+Sex	0.753	3056.000
ENSG00000141002	0.476	0.116	0.376	-0.144	TCF25	0.703	~p_tau+(1|manifest)+cngeneson+pc_mito+brain_region+Sex	1.943	1284.000
ENSG00000082269	0.646	0.116	0.362	-0.131	FAM135A	0.815	~p_tau+(1|manifest)+cngeneson+pc_mito+brain_region+Sex	2.557	950.000
ENSG00000171867	0.099	0.116	0.262	-0.030	PRNP	0.300	~p_tau+(1|manifest)+cngeneson+pc_mito+brain_region+Sex	1.059	2329.000
ENSG00000177917	0.028	0.116	0.203	0.029	ARL6IP6	0.154	~p_tau+(1|manifest)+cngeneson+pc_mito+brain_region+Sex	0.512	3874.000
ENSG00000215114	0.775	0.116	0.490	-0.259	UBXN2B	0.888	~p_tau+(1|manifest)+cngeneson+pc_mito+brain_region+Sex	2.470	983.000
ENSG00000119820	0.014	0.116	0.220	0.011	YIPF4	0.106	~p_tau+(1|manifest)+cngeneson+pc_mito+brain_region+Sex	0.171	5325.000
ENSG00000148337	0.001	0.115	0.182	0.048	CIZ1	0.028	~p_tau+(1|manifest)+cngeneson+pc_mito+brain_region+Sex	0.421	4263.000
ENSG00000137486	0.627	0.115	0.354	-0.124	ARRB1	0.804	~p_tau+(1|manifest)+cngeneson+pc_mito+brain_region+Sex	1.642	1508.000
ENSG00000136603	0.574	0.115	0.348	-0.118	SKIL	0.770	~p_tau+(1|manifest)+cngeneson+pc_mito+brain_region+Sex	2.520	959.000
ENSG00000105323	0.033	0.115	0.206	0.024	HNRNPUL1	0.168	~p_tau+(1|manifest)+cngeneson+pc_mito+brain_region+Sex	0.286	4892.000
ENSG00000047644	0.006	0.115	0.204	0.026	WWC3	0.072	~p_tau+(1|manifest)+cngeneson+pc_mito+brain_region+Sex	0.179	5297.000
ENSG00000116604	0.046	0.115	0.250	-0.021	MEF2D	0.201	~p_tau+(1|manifest)+cngeneson+pc_mito+brain_region+Sex	0.942	2570.000
ENSG00000185436	0.369	0.114	0.285	-0.056	IFNLR1	0.622	~p_tau+(1|manifest)+cngeneson+pc_mito+brain_region+Sex	0.789	2961.000
ENSG00000171466	0.085	0.114	0.240	-0.011	ZNF562	0.278	~p_tau+(1|manifest)+cngeneson+pc_mito+brain_region+Sex	0.419	4276.000
ENSG00000169122	0.899	0.114	0.606	-0.378	FAM110B	0.953	~p_tau+(1|manifest)+cngeneson+pc_mito+brain_region+Sex	3.016	768.000
ENSG00000175104	0.080	0.114	0.286	-0.059	TRAF6	0.270	~p_tau+(1|manifest)+cngeneson+pc_mito+brain_region+Sex	2.038	1224.000
ENSG00000170145	0.107	0.114	0.216	0.012	SIK2	0.313	~p_tau+(1|manifest)+cngeneson+pc_mito+brain_region+Sex	0.219	5176.000
ENSG00000162642	0.006	0.114	0.201	0.027	C1orf52	0.073	~p_tau+(1|manifest)+cngeneson+pc_mito+brain_region+Sex	0.470	4062.000
ENSG00000138029	0.006	0.114	0.185	0.042	HADHB	0.073	~p_tau+(1|manifest)+cngeneson+pc_mito+brain_region+Sex	0.595	3556.000
ENSG00000100722	0.030	0.114	0.192	0.036	ZC3H14	0.160	~p_tau+(1|manifest)+cngeneson+pc_mito+brain_region+Sex	0.111	5444.000
ENSG00000169057	0.040	0.114	0.233	-0.006	MECP2	0.186	~p_tau+(1|manifest)+cngeneson+pc_mito+brain_region+Sex	0.346	4598.000
ENSG00000131504	0.309	0.114	0.265	-0.037	DIAPH1	0.565	~p_tau+(1|manifest)+cngeneson+pc_mito+brain_region+Sex	1.787	1397.000
ENSG00000147121	0.153	0.113	0.241	-0.015	KRBOX4	0.381	~p_tau+(1|manifest)+cngeneson+pc_mito+brain_region+Sex	0.492	3974.000
ENSG00000160445	0.184	0.113	0.246	-0.020	ZER1	0.424	~p_tau+(1|manifest)+cngeneson+pc_mito+brain_region+Sex	1.050	2347.000
ENSG00000088179	0.081	0.113	0.208	0.018	PTPN4	0.270	~p_tau+(1|manifest)+cngeneson+pc_mito+brain_region+Sex	0.196	5239.000
ENSG00000115419	0.601	0.113	0.371	-0.145	GLS	0.787	~p_tau+(1|manifest)+cngeneson+pc_mito+brain_region+Sex	2.659	907.000
ENSG00000116747	0.001	0.113	0.202	0.024	RO60	0.027	~p_tau+(1|manifest)+cngeneson+pc_mito+brain_region+Sex	0.249	5075.000
ENSG00000124491	0.211	0.113	0.378	-0.152	F13A1	0.454	~p_tau+(1|manifest)+cngeneson+pc_mito+brain_region+Sex	5.025	364.000
ENSG00000166716	0.042	0.113	0.200	0.026	ZNF592	0.192	~p_tau+(1|manifest)+cngeneson+pc_mito+brain_region+Sex	0.448	4155.000
ENSG00000092020	0.118	0.113	0.216	0.009	PPP2R3C	0.332	~p_tau+(1|manifest)+cngeneson+pc_mito+brain_region+Sex	0.618	3470.000
ENSG00000157538	0.002	0.113	0.169	0.056	VPS26C	0.044	~p_tau+(1|manifest)+cngeneson+pc_mito+brain_region+Sex	0.237	5124.000
ENSG00000249141	0.256	0.112	0.475	-0.250	AL159163.1	0.510	~p_tau+(1|manifest)+cngeneson+pc_mito+brain_region+Sex	3.162	719.000
ENSG00000180611	0.467	0.112	0.316	-0.092	MB21D2	0.698	~p_tau+(1|manifest)+cngeneson+pc_mito+brain_region+Sex	1.503	1661.000
ENSG00000162542	0.023	0.112	0.199	0.025	TMCO4	0.141	~p_tau+(1|manifest)+cngeneson+pc_mito+brain_region+Sex	0.498	3941.000
ENSG00000101901	0.761	0.112	0.418	-0.194	ALG13	0.879	~p_tau+(1|manifest)+cngeneson+pc_mito+brain_region+Sex	3.453	649.000
ENSG00000163788	0.207	0.112	0.243	-0.019	SNRK	0.450	~p_tau+(1|manifest)+cngeneson+pc_mito+brain_region+Sex	0.396	4376.000
ENSG00000167904	0.002	0.112	0.196	0.027	TMEM68	0.038	~p_tau+(1|manifest)+cngeneson+pc_mito+brain_region+Sex	0.514	3867.000
ENSG00000197405	0.509	0.112	0.316	-0.093	C5AR1	0.728	~p_tau+(1|manifest)+cngeneson+pc_mito+brain_region+Sex	3.370	663.000
ENSG00000106299	0.078	0.112	0.206	0.017	WASL	0.266	~p_tau+(1|manifest)+cngeneson+pc_mito+brain_region+Sex	0.335	4655.000
ENSG00000154655	0.654	0.112	0.367	-0.144	L3MBTL4	0.821	~p_tau+(1|manifest)+cngeneson+pc_mito+brain_region+Sex	2.270	1089.000
ENSG00000168724	0.017	0.112	0.207	0.016	DNAJC21	0.119	~p_tau+(1|manifest)+cngeneson+pc_mito+brain_region+Sex	0.351	4578.000
ENSG00000087274	0.214	0.111	0.239	-0.016	ADD1	0.458	~p_tau+(1|manifest)+cngeneson+pc_mito+brain_region+Sex	0.914	2638.000
ENSG00000169905	0.006	0.111	0.249	-0.026	TOR1AIP2	0.070	~p_tau+(1|manifest)+cngeneson+pc_mito+brain_region+Sex	0.353	4565.000
ENSG00000182180	0.002	0.111	0.194	0.028	MRPS16	0.039	~p_tau+(1|manifest)+cngeneson+pc_mito+brain_region+Sex	0.355	4548.000
ENSG00000104325	0.097	0.111	0.225	-0.003	DECR1	0.296	~p_tau+(1|manifest)+cngeneson+pc_mito+brain_region+Sex	0.451	4140.000
ENSG00000123908	0.158	0.111	0.276	-0.054	AGO2	0.388	~p_tau+(1|manifest)+cngeneson+pc_mito+brain_region+Sex	0.388	4409.000
ENSG00000160007	0.163	0.111	0.290	-0.068	ARHGAP35	0.394	~p_tau+(1|manifest)+cngeneson+pc_mito+brain_region+Sex	0.608	3502.000
ENSG00000227124	0.022	0.111	0.185	0.036	ZNF717	0.138	~p_tau+(1|manifest)+cngeneson+pc_mito+brain_region+Sex	0.326	4705.000
ENSG00000085491	0.279	0.111	0.254	-0.032	SLC25A24	0.533	~p_tau+(1|manifest)+cngeneson+pc_mito+brain_region+Sex	0.918	2626.000
ENSG00000089775	0.204	0.111	0.262	-0.040	ZBTB25	0.447	~p_tau+(1|manifest)+cngeneson+pc_mito+brain_region+Sex	0.314	4753.000
ENSG00000064601	0.006	0.111	0.230	-0.009	CTSA	0.071	~p_tau+(1|manifest)+cngeneson+pc_mito+brain_region+Sex	0.891	2689.000
ENSG00000101596	0.358	0.111	0.280	-0.059	SMCHD1	0.612	~p_tau+(1|manifest)+cngeneson+pc_mito+brain_region+Sex	0.731	3111.000
ENSG00000183808	0.046	0.110	0.218	0.003	RBM12B	0.202	~p_tau+(1|manifest)+cngeneson+pc_mito+brain_region+Sex	0.394	4383.000
ENSG00000106100	0.028	0.110	0.212	0.009	NOD1	0.154	~p_tau+(1|manifest)+cngeneson+pc_mito+brain_region+Sex	0.703	3196.000
ENSG00000095970	0.016	0.110	0.260	-0.040	TREM2	0.115	~p_tau+(1|manifest)+cngeneson+pc_mito+brain_region+Sex	1.533	1620.000
ENSG00000090266	0.003	0.110	0.182	0.039	NDUFB2	0.055	~p_tau+(1|manifest)+cngeneson+pc_mito+brain_region+Sex	0.699	3206.000
ENSG00000154473	0.001	0.110	0.187	0.033	BUB3	0.035	~p_tau+(1|manifest)+cngeneson+pc_mito+brain_region+Sex	0.182	5288.000
ENSG00000135315	0.010	0.110	0.193	0.027	CEP162	0.091	~p_tau+(1|manifest)+cngeneson+pc_mito+brain_region+Sex	0.297	4824.000
ENSG00000119042	0.051	0.110	0.208	0.012	SATB2	0.213	~p_tau+(1|manifest)+cngeneson+pc_mito+brain_region+Sex	0.180	5296.000
ENSG00000272602	0.014	0.110	0.192	0.028	ZNF595	0.108	~p_tau+(1|manifest)+cngeneson+pc_mito+brain_region+Sex	0.286	4893.000
ENSG00000119537	0.005	0.110	0.204	0.016	KDSR	0.063	~p_tau+(1|manifest)+cngeneson+pc_mito+brain_region+Sex	0.390	4398.000
ENSG00000157216	0.408	0.110	0.274	-0.055	SSBP3	0.654	~p_tau+(1|manifest)+cngeneson+pc_mito+brain_region+Sex	1.269	1965.000
ENSG00000162927	0.028	0.110	0.217	0.003	PUS10	0.155	~p_tau+(1|manifest)+cngeneson+pc_mito+brain_region+Sex	0.696	3221.000
ENSG00000175893	0.386	0.110	0.271	-0.052	ZDHHC21	0.634	~p_tau+(1|manifest)+cngeneson+pc_mito+brain_region+Sex	0.698	3213.000
ENSG00000115268	0.628	0.110	0.452	-0.233	RPS15	0.805	~p_tau+(1|manifest)+cngeneson+pc_mito+brain_region+Sex	3.132	729.000
ENSG00000146282	0.238	0.109	0.245	-0.027	RARS2	0.489	~p_tau+(1|manifest)+cngeneson+pc_mito+brain_region+Sex	0.601	3533.000
ENSG00000167601	0.773	0.109	0.455	-0.237	AXL	0.887	~p_tau+(1|manifest)+cngeneson+pc_mito+brain_region+Sex	2.811	837.000
ENSG00000168172	0.338	0.109	0.268	-0.050	HOOK3	0.594	~p_tau+(1|manifest)+cngeneson+pc_mito+brain_region+Sex	0.555	3699.000
ENSG00000173660	0.002	0.109	0.205	0.014	UQCRH	0.039	~p_tau+(1|manifest)+cngeneson+pc_mito+brain_region+Sex	0.361	4522.000
ENSG00000138756	0.034	0.109	0.193	0.026	BMP2K	0.172	~p_tau+(1|manifest)+cngeneson+pc_mito+brain_region+Sex	2.658	908.000
ENSG00000148400	0.093	0.109	0.268	-0.050	NOTCH1	0.289	~p_tau+(1|manifest)+cngeneson+pc_mito+brain_region+Sex	0.860	2769.000
ENSG00000134030	0.035	0.109	0.201	0.017	CTIF	0.174	~p_tau+(1|manifest)+cngeneson+pc_mito+brain_region+Sex	0.275	4949.000
ENSG00000188177	0.480	0.109	0.289	-0.071	ZC3H6	0.706	~p_tau+(1|manifest)+cngeneson+pc_mito+brain_region+Sex	1.029	2384.000
ENSG00000172296	0.391	0.109	0.385	-0.168	SPTLC3	0.638	~p_tau+(1|manifest)+cngeneson+pc_mito+brain_region+Sex	2.406	1015.000
ENSG00000119688	0.011	0.109	0.192	0.026	ABCD4	0.093	~p_tau+(1|manifest)+cngeneson+pc_mito+brain_region+Sex	0.482	4018.000
ENSG00000170234	0.130	0.109	0.277	-0.060	PWWP2A	0.348	~p_tau+(1|manifest)+cngeneson+pc_mito+brain_region+Sex	0.653	3351.000
ENSG00000114391	0.080	0.109	0.284	-0.067	RPL24	0.270	~p_tau+(1|manifest)+cngeneson+pc_mito+brain_region+Sex	0.990	2454.000
ENSG00000136527	0.078	0.109	0.345	-0.128	TRA2B	0.265	~p_tau+(1|manifest)+cngeneson+pc_mito+brain_region+Sex	1.572	1592.000
ENSG00000071246	0.805	0.108	0.482	-0.265	VASH1	0.906	~p_tau+(1|manifest)+cngeneson+pc_mito+brain_region+Sex	3.001	773.000
ENSG00000102780	0.074	0.108	0.232	-0.015	DGKH	0.259	~p_tau+(1|manifest)+cngeneson+pc_mito+brain_region+Sex	0.908	2656.000
ENSG00000178385	0.442	0.108	0.277	-0.061	PLEKHM3	0.681	~p_tau+(1|manifest)+cngeneson+pc_mito+brain_region+Sex	1.426	1750.000
ENSG00000126934	0.005	0.108	0.172	0.044	MAP2K2	0.064	~p_tau+(1|manifest)+cngeneson+pc_mito+brain_region+Sex	0.472	4051.000
ENSG00000123562	0.156	0.108	0.280	-0.064	MORF4L2	0.384	~p_tau+(1|manifest)+cngeneson+pc_mito+brain_region+Sex	0.654	3347.000
ENSG00000169660	0.211	0.108	0.249	-0.033	HEXD	0.454	~p_tau+(1|manifest)+cngeneson+pc_mito+brain_region+Sex	0.603	3521.000
ENSG00000166913	0.057	0.108	0.193	0.023	YWHAB	0.226	~p_tau+(1|manifest)+cngeneson+pc_mito+brain_region+Sex	0.565	3662.000
ENSG00000128654	0.004	0.108	0.181	0.034	MTX2	0.062	~p_tau+(1|manifest)+cngeneson+pc_mito+brain_region+Sex	0.324	4710.000
ENSG00000111011	0.062	0.107	0.225	-0.010	RSRC2	0.238	~p_tau+(1|manifest)+cngeneson+pc_mito+brain_region+Sex	0.328	4691.000
ENSG00000158863	0.004	0.107	0.175	0.040	FAM160B2	0.057	~p_tau+(1|manifest)+cngeneson+pc_mito+brain_region+Sex	0.395	4379.000
ENSG00000158604	0.008	0.107	0.181	0.034	TMED4	0.082	~p_tau+(1|manifest)+cngeneson+pc_mito+brain_region+Sex	0.689	3237.000
ENSG00000116198	0.003	0.107	0.165	0.049	CEP104	0.050	~p_tau+(1|manifest)+cngeneson+pc_mito+brain_region+Sex	0.264	5006.000
ENSG00000180530	0.912	0.107	0.602	-0.388	NRIP1	0.959	~p_tau+(1|manifest)+cngeneson+pc_mito+brain_region+Sex	6.526	239.000
ENSG00000104312	0.276	0.107	0.323	-0.109	RIPK2	0.529	~p_tau+(1|manifest)+cngeneson+pc_mito+brain_region+Sex	1.773	1405.000
ENSG00000198034	0.640	0.107	0.441	-0.227	RPS4X	0.811	~p_tau+(1|manifest)+cngeneson+pc_mito+brain_region+Sex	3.498	637.000
ENSG00000181220	0.002	0.107	0.179	0.035	ZNF746	0.038	~p_tau+(1|manifest)+cngeneson+pc_mito+brain_region+Sex	0.314	4756.000
ENSG00000171055	0.590	0.107	0.320	-0.106	FEZ2	0.781	~p_tau+(1|manifest)+cngeneson+pc_mito+brain_region+Sex	0.693	3224.000
ENSG00000124587	0.072	0.107	0.208	0.005	PEX6	0.254	~p_tau+(1|manifest)+cngeneson+pc_mito+brain_region+Sex	0.485	3998.000
ENSG00000197119	0.142	0.107	0.277	-0.064	SLC25A29	0.365	~p_tau+(1|manifest)+cngeneson+pc_mito+brain_region+Sex	1.224	2043.000
ENSG00000138801	0.004	0.106	0.160	0.052	PAPSS1	0.058	~p_tau+(1|manifest)+cngeneson+pc_mito+brain_region+Sex	0.136	5412.000
ENSG00000143624	0.012	0.106	0.207	0.005	INTS3	0.099	~p_tau+(1|manifest)+cngeneson+pc_mito+brain_region+Sex	0.553	3709.000
ENSG00000174282	0.066	0.106	0.217	-0.005	ZBTB4	0.244	~p_tau+(1|manifest)+cngeneson+pc_mito+brain_region+Sex	0.560	3686.000
ENSG00000163312	0.041	0.106	0.186	0.026	HELQ	0.190	~p_tau+(1|manifest)+cngeneson+pc_mito+brain_region+Sex	0.130	5421.000
ENSG00000198040	0.119	0.106	0.245	-0.033	ZNF84	0.334	~p_tau+(1|manifest)+cngeneson+pc_mito+brain_region+Sex	0.851	2790.000
ENSG00000196470	0.052	0.106	0.208	0.004	SIAH1	0.217	~p_tau+(1|manifest)+cngeneson+pc_mito+brain_region+Sex	0.520	3835.000
ENSG00000177156	0.021	0.106	0.204	0.008	TALDO1	0.133	~p_tau+(1|manifest)+cngeneson+pc_mito+brain_region+Sex	0.567	3655.000
ENSG00000130958	0.030	0.106	0.190	0.022	SLC35D2	0.161	~p_tau+(1|manifest)+cngeneson+pc_mito+brain_region+Sex	0.474	4041.000
ENSG00000133246	0.874	0.106	0.533	-0.322	PRAM1	0.942	~p_tau+(1|manifest)+cngeneson+pc_mito+brain_region+Sex	3.670	603.000
ENSG00000100852	0.605	0.106	0.322	-0.111	ARHGAP5	0.790	~p_tau+(1|manifest)+cngeneson+pc_mito+brain_region+Sex	1.945	1282.000
ENSG00000084676	0.208	0.106	0.237	-0.026	NCOA1	0.450	~p_tau+(1|manifest)+cngeneson+pc_mito+brain_region+Sex	0.601	3529.000
ENSG00000145833	0.223	0.105	0.226	-0.015	DDX46	0.469	~p_tau+(1|manifest)+cngeneson+pc_mito+brain_region+Sex	0.226	5158.000
ENSG00000161551	0.000	0.105	0.161	0.049	ZNF577	0.019	~p_tau+(1|manifest)+cngeneson+pc_mito+brain_region+Sex	0.312	4764.000
ENSG00000113638	0.009	0.105	0.175	0.034	TTC33	0.085	~p_tau+(1|manifest)+cngeneson+pc_mito+brain_region+Sex	0.226	5156.000
ENSG00000133612	0.048	0.105	0.238	-0.029	AGAP3	0.203	~p_tau+(1|manifest)+cngeneson+pc_mito+brain_region+Sex	0.178	5301.000
ENSG00000080298	0.641	0.105	0.344	-0.135	RFX3	0.811	~p_tau+(1|manifest)+cngeneson+pc_mito+brain_region+Sex	1.299	1914.000
ENSG00000141664	0.607	0.104	0.326	-0.117	ZCCHC2	0.790	~p_tau+(1|manifest)+cngeneson+pc_mito+brain_region+Sex	0.884	2702.000
ENSG00000127952	0.014	0.104	0.176	0.033	STYXL1	0.108	~p_tau+(1|manifest)+cngeneson+pc_mito+brain_region+Sex	0.431	4211.000
ENSG00000178585	0.018	0.104	0.179	0.030	CTNNBIP1	0.124	~p_tau+(1|manifest)+cngeneson+pc_mito+brain_region+Sex	0.530	3796.000
ENSG00000168994	0.012	0.104	0.179	0.029	PXDC1	0.100	~p_tau+(1|manifest)+cngeneson+pc_mito+brain_region+Sex	0.410	4312.000
ENSG00000066739	0.064	0.104	0.212	-0.004	ATG2B	0.242	~p_tau+(1|manifest)+cngeneson+pc_mito+brain_region+Sex	0.461	4102.000
ENSG00000120458	0.392	0.104	0.254	-0.046	MSANTD2	0.639	~p_tau+(1|manifest)+cngeneson+pc_mito+brain_region+Sex	0.501	3921.000
ENSG00000115318	0.512	0.104	0.363	-0.155	LOXL3	0.730	~p_tau+(1|manifest)+cngeneson+pc_mito+brain_region+Sex	2.013	1238.000
ENSG00000198730	0.033	0.104	0.215	-0.007	CTR9	0.168	~p_tau+(1|manifest)+cngeneson+pc_mito+brain_region+Sex	0.345	4611.000
ENSG00000099246	0.042	0.104	0.205	0.003	RAB18	0.192	~p_tau+(1|manifest)+cngeneson+pc_mito+brain_region+Sex	0.506	3904.000
ENSG00000269713	0.001	0.104	0.168	0.039	NBPF9	0.028	~p_tau+(1|manifest)+cngeneson+pc_mito+brain_region+Sex	0.229	5146.000
ENSG00000172493	0.634	0.104	0.313	-0.106	AFF1	0.807	~p_tau+(1|manifest)+cngeneson+pc_mito+brain_region+Sex	2.288	1079.000
ENSG00000164938	0.114	0.104	0.264	-0.057	TP53INP1	0.326	~p_tau+(1|manifest)+cngeneson+pc_mito+brain_region+Sex	1.820	1378.000
ENSG00000205213	0.038	0.103	0.182	0.024	LGR4	0.182	~p_tau+(1|manifest)+cngeneson+pc_mito+brain_region+Sex	0.502	3918.000
ENSG00000214941	0.157	0.103	0.245	-0.038	ZSWIM7	0.385	~p_tau+(1|manifest)+cngeneson+pc_mito+brain_region+Sex	0.553	3708.000
ENSG00000148362	0.129	0.103	0.311	-0.105	PAXX	0.348	~p_tau+(1|manifest)+cngeneson+pc_mito+brain_region+Sex	1.330	1864.000
ENSG00000162819	0.274	0.103	0.303	-0.097	BROX	0.528	~p_tau+(1|manifest)+cngeneson+pc_mito+brain_region+Sex	1.243	2013.000
ENSG00000196975	0.808	0.103	0.417	-0.210	ANXA4	0.907	~p_tau+(1|manifest)+cngeneson+pc_mito+brain_region+Sex	1.879	1327.000
ENSG00000058453	0.097	0.103	0.248	-0.042	CROCC	0.296	~p_tau+(1|manifest)+cngeneson+pc_mito+brain_region+Sex	1.346	1837.000
ENSG00000118579	0.034	0.103	0.213	-0.007	MED28	0.171	~p_tau+(1|manifest)+cngeneson+pc_mito+brain_region+Sex	0.258	5035.000
ENSG00000131748	0.001	0.103	0.176	0.030	STARD3	0.031	~p_tau+(1|manifest)+cngeneson+pc_mito+brain_region+Sex	0.276	4942.000
ENSG00000132182	0.130	0.103	0.270	-0.064	NUP210	0.349	~p_tau+(1|manifest)+cngeneson+pc_mito+brain_region+Sex	0.862	2764.000
ENSG00000163590	0.356	0.103	0.250	-0.044	PPM1L	0.611	~p_tau+(1|manifest)+cngeneson+pc_mito+brain_region+Sex	0.418	4281.000
ENSG00000106868	0.093	0.103	0.309	-0.103	SUSD1	0.290	~p_tau+(1|manifest)+cngeneson+pc_mito+brain_region+Sex	2.362	1036.000
ENSG00000198791	0.005	0.103	0.179	0.027	CNOT7	0.063	~p_tau+(1|manifest)+cngeneson+pc_mito+brain_region+Sex	0.309	4778.000
ENSG00000111653	0.018	0.103	0.190	0.015	ING4	0.124	~p_tau+(1|manifest)+cngeneson+pc_mito+brain_region+Sex	0.655	3340.000
ENSG00000137073	0.209	0.102	0.223	-0.018	UBAP2	0.451	~p_tau+(1|manifest)+cngeneson+pc_mito+brain_region+Sex	0.699	3209.000
ENSG00000104047	0.004	0.102	0.202	0.002	DTWD1	0.060	~p_tau+(1|manifest)+cngeneson+pc_mito+brain_region+Sex	0.605	3512.000
ENSG00000171100	0.200	0.102	0.221	-0.016	MTM1	0.442	~p_tau+(1|manifest)+cngeneson+pc_mito+brain_region+Sex	0.308	4785.000
ENSG00000163029	0.027	0.102	0.191	0.013	SMC6	0.152	~p_tau+(1|manifest)+cngeneson+pc_mito+brain_region+Sex	0.295	4838.000
ENSG00000162378	0.007	0.102	0.164	0.041	ZYG11B	0.075	~p_tau+(1|manifest)+cngeneson+pc_mito+brain_region+Sex	0.493	3968.000
ENSG00000099331	0.237	0.102	0.257	-0.053	MYO9B	0.487	~p_tau+(1|manifest)+cngeneson+pc_mito+brain_region+Sex	1.642	1509.000
ENSG00000197372	0.017	0.102	0.186	0.018	ZNF675	0.122	~p_tau+(1|manifest)+cngeneson+pc_mito+brain_region+Sex	0.275	4948.000
ENSG00000047365	0.846	0.102	0.452	-0.248	ARAP2	0.928	~p_tau+(1|manifest)+cngeneson+pc_mito+brain_region+Sex	3.658	609.000
ENSG00000158813	0.700	0.102	0.338	-0.134	EDA	0.848	~p_tau+(1|manifest)+cngeneson+pc_mito+brain_region+Sex	0.979	2476.000
ENSG00000174197	0.450	0.102	0.268	-0.064	MGA	0.685	~p_tau+(1|manifest)+cngeneson+pc_mito+brain_region+Sex	1.156	2150.000
ENSG00000169499	0.399	0.102	0.247	-0.043	PLEKHA2	0.645	~p_tau+(1|manifest)+cngeneson+pc_mito+brain_region+Sex	1.571	1593.000
ENSG00000120899	0.832	0.102	0.455	-0.251	PTK2B	0.922	~p_tau+(1|manifest)+cngeneson+pc_mito+brain_region+Sex	3.856	557.000
ENSG00000167658	0.205	0.102	0.271	-0.068	EEF2	0.449	~p_tau+(1|manifest)+cngeneson+pc_mito+brain_region+Sex	1.596	1564.000
ENSG00000123636	0.365	0.101	0.272	-0.069	BAZ2B	0.618	~p_tau+(1|manifest)+cngeneson+pc_mito+brain_region+Sex	3.243	699.000
ENSG00000043462	0.619	0.101	0.410	-0.207	LCP2	0.798	~p_tau+(1|manifest)+cngeneson+pc_mito+brain_region+Sex	2.576	942.000
ENSG00000006704	0.092	0.101	0.245	-0.043	GTF2IRD1	0.289	~p_tau+(1|manifest)+cngeneson+pc_mito+brain_region+Sex	1.338	1852.000
ENSG00000152464	0.086	0.101	0.207	-0.005	RPP38	0.278	~p_tau+(1|manifest)+cngeneson+pc_mito+brain_region+Sex	0.386	4413.000
ENSG00000117625	0.210	0.101	0.260	-0.058	RCOR3	0.453	~p_tau+(1|manifest)+cngeneson+pc_mito+brain_region+Sex	0.914	2637.000
ENSG00000060762	0.047	0.101	0.191	0.011	MPC1	0.203	~p_tau+(1|manifest)+cngeneson+pc_mito+brain_region+Sex	0.451	4139.000
ENSG00000135164	0.658	0.101	0.315	-0.113	DMTF1	0.823	~p_tau+(1|manifest)+cngeneson+pc_mito+brain_region+Sex	0.848	2801.000
ENSG00000109667	0.354	0.101	0.300	-0.098	SLC2A9	0.608	~p_tau+(1|manifest)+cngeneson+pc_mito+brain_region+Sex	0.252	5066.000
ENSG00000135677	0.007	0.101	0.195	0.006	GNS	0.076	~p_tau+(1|manifest)+cngeneson+pc_mito+brain_region+Sex	0.633	3410.000
ENSG00000161203	0.020	0.101	0.183	0.019	AP2M1	0.129	~p_tau+(1|manifest)+cngeneson+pc_mito+brain_region+Sex	0.526	3807.000
ENSG00000151743	0.456	0.101	0.262	-0.061	AMN1	0.690	~p_tau+(1|manifest)+cngeneson+pc_mito+brain_region+Sex	0.495	3956.000
ENSG00000150048	0.741	0.100	0.381	-0.180	CLEC1A	0.869	~p_tau+(1|manifest)+cngeneson+pc_mito+brain_region+Sex	13.468	61.000
ENSG00000197093	0.303	0.100	0.334	-0.133	GAL3ST4	0.559	~p_tau+(1|manifest)+cngeneson+pc_mito+brain_region+Sex	2.120	1172.000
ENSG00000236287	0.006	0.100	0.220	-0.020	ZBED5	0.073	~p_tau+(1|manifest)+cngeneson+pc_mito+brain_region+Sex	0.288	4876.000
ENSG00000156973	0.091	0.100	0.207	-0.007	PDE6D	0.288	~p_tau+(1|manifest)+cngeneson+pc_mito+brain_region+Sex	0.436	4192.000
ENSG00000006125	0.099	0.100	0.193	0.007	AP2B1	0.299	~p_tau+(1|manifest)+cngeneson+pc_mito+brain_region+Sex	0.454	4126.000
ENSG00000173221	0.511	0.100	0.332	-0.132	GLRX	0.729	~p_tau+(1|manifest)+cngeneson+pc_mito+brain_region+Sex	0.398	4368.000
ENSG00000176390	0.507	0.100	0.276	-0.076	CRLF3	0.727	~p_tau+(1|manifest)+cngeneson+pc_mito+brain_region+Sex	0.843	2816.000
ENSG00000171766	0.257	0.100	0.362	-0.163	GATM	0.511	~p_tau+(1|manifest)+cngeneson+pc_mito+brain_region+Sex	3.692	595.000
ENSG00000112079	0.259	0.100	0.255	-0.056	STK38	0.513	~p_tau+(1|manifest)+cngeneson+pc_mito+brain_region+Sex	0.557	3694.000
ENSG00000070476	0.187	0.100	0.245	-0.046	ZXDC	0.427	~p_tau+(1|manifest)+cngeneson+pc_mito+brain_region+Sex	0.476	4039.000
ENSG00000180376	0.123	0.099	0.216	-0.017	CCDC66	0.340	~p_tau+(1|manifest)+cngeneson+pc_mito+brain_region+Sex	0.411	4309.000
ENSG00000164924	0.239	0.099	0.271	-0.072	YWHAZ	0.489	~p_tau+(1|manifest)+cngeneson+pc_mito+brain_region+Sex	1.392	1778.000
ENSG00000173852	0.070	0.099	0.202	-0.003	DPY19L1	0.250	~p_tau+(1|manifest)+cngeneson+pc_mito+brain_region+Sex	0.551	3716.000
ENSG00000168067	0.003	0.099	0.157	0.041	MAP4K2	0.054	~p_tau+(1|manifest)+cngeneson+pc_mito+brain_region+Sex	0.258	5038.000
ENSG00000111348	0.169	0.099	0.271	-0.073	ARHGDIB	0.402	~p_tau+(1|manifest)+cngeneson+pc_mito+brain_region+Sex	0.411	4307.000
ENSG00000089737	0.147	0.099	0.208	-0.010	DDX24	0.373	~p_tau+(1|manifest)+cngeneson+pc_mito+brain_region+Sex	0.479	4030.000
ENSG00000011021	0.002	0.099	0.172	0.026	CLCN6	0.044	~p_tau+(1|manifest)+cngeneson+pc_mito+brain_region+Sex	0.375	4455.000
ENSG00000108946	0.164	0.099	0.231	-0.033	PRKAR1A	0.395	~p_tau+(1|manifest)+cngeneson+pc_mito+brain_region+Sex	0.957	2531.000
ENSG00000163945	0.678	0.099	0.325	-0.127	UVSSA	0.837	~p_tau+(1|manifest)+cngeneson+pc_mito+brain_region+Sex	1.061	2327.000
ENSG00000139508	0.051	0.099	0.214	-0.016	SLC46A3	0.213	~p_tau+(1|manifest)+cngeneson+pc_mito+brain_region+Sex	0.547	3734.000
ENSG00000213445	0.107	0.099	0.301	-0.103	SIPA1	0.313	~p_tau+(1|manifest)+cngeneson+pc_mito+brain_region+Sex	1.343	1843.000
ENSG00000197111	0.014	0.098	0.261	-0.064	PCBP2	0.109	~p_tau+(1|manifest)+cngeneson+pc_mito+brain_region+Sex	0.642	3386.000
ENSG00000099139	0.526	0.098	0.280	-0.083	PCSK5	0.738	~p_tau+(1|manifest)+cngeneson+pc_mito+brain_region+Sex	0.751	3063.000
ENSG00000104903	0.070	0.098	0.276	-0.079	LYL1	0.249	~p_tau+(1|manifest)+cngeneson+pc_mito+brain_region+Sex	2.030	1229.000
ENSG00000137074	0.167	0.098	0.226	-0.030	APTX	0.401	~p_tau+(1|manifest)+cngeneson+pc_mito+brain_region+Sex	0.734	3105.000
ENSG00000121989	0.048	0.098	0.209	-0.014	ACVR2A	0.205	~p_tau+(1|manifest)+cngeneson+pc_mito+brain_region+Sex	0.339	4636.000
ENSG00000102738	0.027	0.098	0.218	-0.022	MRPS31	0.152	~p_tau+(1|manifest)+cngeneson+pc_mito+brain_region+Sex	0.324	4715.000
ENSG00000172661	0.006	0.098	0.164	0.032	WASHC2C	0.073	~p_tau+(1|manifest)+cngeneson+pc_mito+brain_region+Sex	0.425	4241.000
ENSG00000172728	0.142	0.098	0.228	-0.032	FUT10	0.366	~p_tau+(1|manifest)+cngeneson+pc_mito+brain_region+Sex	0.838	2834.000
ENSG00000008513	0.795	0.098	0.381	-0.186	ST3GAL1	0.900	~p_tau+(1|manifest)+cngeneson+pc_mito+brain_region+Sex	2.143	1156.000
ENSG00000102125	0.152	0.097	0.211	-0.016	TAZ	0.380	~p_tau+(1|manifest)+cngeneson+pc_mito+brain_region+Sex	0.657	3332.000
ENSG00000114904	0.013	0.097	0.169	0.025	NEK4	0.101	~p_tau+(1|manifest)+cngeneson+pc_mito+brain_region+Sex	0.243	5098.000
ENSG00000015153	0.372	0.097	0.325	-0.130	YAF2	0.625	~p_tau+(1|manifest)+cngeneson+pc_mito+brain_region+Sex	0.824	2878.000
ENSG00000108107	0.898	0.097	0.537	-0.343	RPL28	0.953	~p_tau+(1|manifest)+cngeneson+pc_mito+brain_region+Sex	5.138	349.000
ENSG00000139921	0.268	0.097	0.238	-0.044	TMX1	0.522	~p_tau+(1|manifest)+cngeneson+pc_mito+brain_region+Sex	0.689	3239.000
ENSG00000196182	0.045	0.097	0.188	0.006	STK40	0.200	~p_tau+(1|manifest)+cngeneson+pc_mito+brain_region+Sex	0.270	4971.000
ENSG00000164430	0.175	0.097	0.337	-0.143	CGAS	0.411	~p_tau+(1|manifest)+cngeneson+pc_mito+brain_region+Sex	1.525	1631.000
ENSG00000072121	0.002	0.097	0.169	0.024	ZFYVE26	0.040	~p_tau+(1|manifest)+cngeneson+pc_mito+brain_region+Sex	0.223	5167.000
ENSG00000000419	0.049	0.096	0.190	0.003	DPM1	0.206	~p_tau+(1|manifest)+cngeneson+pc_mito+brain_region+Sex	0.419	4275.000
ENSG00000188647	0.517	0.096	0.270	-0.078	PTAR1	0.732	~p_tau+(1|manifest)+cngeneson+pc_mito+brain_region+Sex	0.958	2530.000
ENSG00000122741	0.471	0.096	0.249	-0.056	DCAF10	0.700	~p_tau+(1|manifest)+cngeneson+pc_mito+brain_region+Sex	0.583	3603.000
ENSG00000157426	0.004	0.096	0.165	0.027	AASDH	0.059	~p_tau+(1|manifest)+cngeneson+pc_mito+brain_region+Sex	0.160	5360.000
ENSG00000128805	0.691	0.096	0.324	-0.132	ARHGAP22	0.843	~p_tau+(1|manifest)+cngeneson+pc_mito+brain_region+Sex	7.226	200.000
ENSG00000071626	0.114	0.096	0.212	-0.020	DAZAP1	0.326	~p_tau+(1|manifest)+cngeneson+pc_mito+brain_region+Sex	0.949	2547.000
ENSG00000048649	0.264	0.096	0.220	-0.028	RSF1	0.517	~p_tau+(1|manifest)+cngeneson+pc_mito+brain_region+Sex	0.659	3325.000
ENSG00000155099	0.018	0.095	0.209	-0.019	PIP4P2	0.122	~p_tau+(1|manifest)+cngeneson+pc_mito+brain_region+Sex	0.493	3966.000
ENSG00000137513	0.378	0.095	0.259	-0.069	NARS2	0.629	~p_tau+(1|manifest)+cngeneson+pc_mito+brain_region+Sex	0.911	2647.000
ENSG00000218739	0.057	0.095	0.188	0.002	CEBPZOS	0.227	~p_tau+(1|manifest)+cngeneson+pc_mito+brain_region+Sex	0.290	4865.000
ENSG00000166503	0.009	0.095	0.217	-0.028	HDGFL3	0.084	~p_tau+(1|manifest)+cngeneson+pc_mito+brain_region+Sex	1.207	2070.000
ENSG00000139644	0.306	0.095	0.308	-0.119	TMBIM6	0.562	~p_tau+(1|manifest)+cngeneson+pc_mito+brain_region+Sex	1.223	2044.000
ENSG00000171109	0.003	0.095	0.141	0.048	MFN1	0.054	~p_tau+(1|manifest)+cngeneson+pc_mito+brain_region+Sex	0.259	5032.000
ENSG00000138593	0.125	0.094	0.193	-0.004	SECISBP2L	0.342	~p_tau+(1|manifest)+cngeneson+pc_mito+brain_region+Sex	0.277	4938.000
ENSG00000146247	0.514	0.094	0.262	-0.073	PHIP	0.731	~p_tau+(1|manifest)+cngeneson+pc_mito+brain_region+Sex	1.750	1421.000
ENSG00000112081	0.201	0.094	0.213	-0.024	SRSF3	0.444	~p_tau+(1|manifest)+cngeneson+pc_mito+brain_region+Sex	1.449	1721.000
ENSG00000140836	0.011	0.094	0.167	0.022	ZFHX3	0.092	~p_tau+(1|manifest)+cngeneson+pc_mito+brain_region+Sex	1.505	1659.000
ENSG00000198642	0.024	0.094	0.208	-0.019	KLHL9	0.142	~p_tau+(1|manifest)+cngeneson+pc_mito+brain_region+Sex	0.578	3621.000
ENSG00000205413	0.094	0.094	0.197	-0.008	SAMD9	0.291	~p_tau+(1|manifest)+cngeneson+pc_mito+brain_region+Sex	0.433	4207.000
ENSG00000120129	0.748	0.094	0.473	-0.285	DUSP1	0.873	~p_tau+(1|manifest)+cngeneson+pc_mito+brain_region+Sex	9.346	122.000
ENSG00000155849	0.131	0.094	0.222	-0.034	ELMO1	0.351	~p_tau+(1|manifest)+cngeneson+pc_mito+brain_region+Sex	7.518	186.000
ENSG00000111145	0.380	0.094	0.270	-0.082	ELK3	0.629	~p_tau+(1|manifest)+cngeneson+pc_mito+brain_region+Sex	0.497	3947.000
ENSG00000157954	0.327	0.094	0.257	-0.070	WIPI2	0.584	~p_tau+(1|manifest)+cngeneson+pc_mito+brain_region+Sex	2.296	1073.000
ENSG00000063244	0.052	0.094	0.184	0.004	U2AF2	0.216	~p_tau+(1|manifest)+cngeneson+pc_mito+brain_region+Sex	0.611	3493.000
ENSG00000145439	0.606	0.094	0.284	-0.096	CBR4	0.790	~p_tau+(1|manifest)+cngeneson+pc_mito+brain_region+Sex	0.590	3572.000
ENSG00000085760	0.000	0.094	0.138	0.049	MTIF2	0.015	~p_tau+(1|manifest)+cngeneson+pc_mito+brain_region+Sex	0.146	5394.000
ENSG00000122432	0.009	0.094	0.199	-0.012	SPATA1	0.085	~p_tau+(1|manifest)+cngeneson+pc_mito+brain_region+Sex	0.497	3949.000
ENSG00000004766	0.557	0.093	0.277	-0.090	VPS50	0.761	~p_tau+(1|manifest)+cngeneson+pc_mito+brain_region+Sex	1.002	2434.000
ENSG00000196187	0.004	0.093	0.146	0.040	TMEM63A	0.058	~p_tau+(1|manifest)+cngeneson+pc_mito+brain_region+Sex	0.216	5185.000
ENSG00000103091	0.002	0.093	0.138	0.048	WDR59	0.038	~p_tau+(1|manifest)+cngeneson+pc_mito+brain_region+Sex	0.136	5413.000
ENSG00000120306	0.012	0.093	0.192	-0.006	CYSTM1	0.101	~p_tau+(1|manifest)+cngeneson+pc_mito+brain_region+Sex	1.205	2074.000
ENSG00000241973	0.042	0.093	0.192	-0.006	PI4KA	0.192	~p_tau+(1|manifest)+cngeneson+pc_mito+brain_region+Sex	0.226	5160.000
ENSG00000176225	0.740	0.093	0.472	-0.287	RTTN	0.869	~p_tau+(1|manifest)+cngeneson+pc_mito+brain_region+Sex	4.306	477.000
ENSG00000140740	0.084	0.093	0.201	-0.016	UQCRC2	0.277	~p_tau+(1|manifest)+cngeneson+pc_mito+brain_region+Sex	0.267	4983.000
ENSG00000156261	0.125	0.093	0.220	-0.035	CCT8	0.342	~p_tau+(1|manifest)+cngeneson+pc_mito+brain_region+Sex	0.767	3021.000
ENSG00000188938	0.093	0.093	0.193	-0.008	---------	0.289	~p_tau+(1|manifest)+cngeneson+pc_mito+brain_region+Sex	0.539	3764.000
ENSG00000112893	0.736	0.093	0.370	-0.185	MAN2A1	0.867	~p_tau+(1|manifest)+cngeneson+pc_mito+brain_region+Sex	1.366	1811.000
ENSG00000171475	0.172	0.093	0.188	-0.003	WIPF2	0.407	~p_tau+(1|manifest)+cngeneson+pc_mito+brain_region+Sex	0.659	3324.000
ENSG00000161091	0.137	0.093	0.189	-0.004	MFSD12	0.358	~p_tau+(1|manifest)+cngeneson+pc_mito+brain_region+Sex	0.628	3431.000
ENSG00000145779	0.713	0.092	0.380	-0.195	TNFAIP8	0.856	~p_tau+(1|manifest)+cngeneson+pc_mito+brain_region+Sex	3.090	740.000
ENSG00000163743	0.039	0.092	0.172	0.013	RCHY1	0.185	~p_tau+(1|manifest)+cngeneson+pc_mito+brain_region+Sex	0.324	4709.000
ENSG00000138433	0.003	0.092	0.188	-0.004	CIR1	0.053	~p_tau+(1|manifest)+cngeneson+pc_mito+brain_region+Sex	0.418	4280.000
ENSG00000178691	0.064	0.092	0.176	0.008	SUZ12	0.242	~p_tau+(1|manifest)+cngeneson+pc_mito+brain_region+Sex	0.351	4577.000
ENSG00000156017	0.282	0.092	0.222	-0.038	CARNMT1	0.535	~p_tau+(1|manifest)+cngeneson+pc_mito+brain_region+Sex	1.340	1849.000
ENSG00000142546	0.096	0.092	0.180	0.004	NOSIP	0.294	~p_tau+(1|manifest)+cngeneson+pc_mito+brain_region+Sex	0.425	4242.000
ENSG00000113732	0.224	0.092	0.210	-0.026	ATP6V0E1	0.470	~p_tau+(1|manifest)+cngeneson+pc_mito+brain_region+Sex	0.448	4152.000
ENSG00000115816	0.148	0.092	0.190	-0.007	CEBPZ	0.373	~p_tau+(1|manifest)+cngeneson+pc_mito+brain_region+Sex	0.236	5126.000
ENSG00000074201	0.248	0.092	0.217	-0.034	CLNS1A	0.500	~p_tau+(1|manifest)+cngeneson+pc_mito+brain_region+Sex	0.445	4160.000
ENSG00000066926	0.123	0.092	0.183	0.000	FECH	0.340	~p_tau+(1|manifest)+cngeneson+pc_mito+brain_region+Sex	0.715	3150.000
ENSG00000116793	0.161	0.092	0.186	-0.003	PHTF1	0.391	~p_tau+(1|manifest)+cngeneson+pc_mito+brain_region+Sex	0.707	3184.000
ENSG00000106460	0.002	0.092	0.157	0.026	TMEM106B	0.040	~p_tau+(1|manifest)+cngeneson+pc_mito+brain_region+Sex	0.153	5376.000
ENSG00000203879	0.001	0.091	0.156	0.027	GDI1	0.031	~p_tau+(1|manifest)+cngeneson+pc_mito+brain_region+Sex	0.298	4820.000
ENSG00000143753	0.067	0.091	0.215	-0.033	DEGS1	0.245	~p_tau+(1|manifest)+cngeneson+pc_mito+brain_region+Sex	1.157	2149.000
ENSG00000111203	0.235	0.091	0.279	-0.096	ITFG2	0.485	~p_tau+(1|manifest)+cngeneson+pc_mito+brain_region+Sex	1.222	2046.000
ENSG00000116171	0.091	0.091	0.183	0.000	SCP2	0.288	~p_tau+(1|manifest)+cngeneson+pc_mito+brain_region+Sex	0.363	4513.000
ENSG00000147162	0.244	0.091	0.293	-0.110	OGT	0.494	~p_tau+(1|manifest)+cngeneson+pc_mito+brain_region+Sex	1.169	2127.000
ENSG00000062725	0.101	0.091	0.173	0.009	APPBP2	0.302	~p_tau+(1|manifest)+cngeneson+pc_mito+brain_region+Sex	0.138	5409.000
ENSG00000135297	0.002	0.091	0.142	0.040	MTO1	0.038	~p_tau+(1|manifest)+cngeneson+pc_mito+brain_region+Sex	0.232	5135.000
ENSG00000183091	0.169	0.091	0.256	-0.074	NEB	0.401	~p_tau+(1|manifest)+cngeneson+pc_mito+brain_region+Sex	2.183	1139.000
ENSG00000197081	0.417	0.091	0.388	-0.207	IGF2R	0.660	~p_tau+(1|manifest)+cngeneson+pc_mito+brain_region+Sex	2.167	1145.000
ENSG00000130177	0.109	0.091	0.188	-0.006	CDC16	0.316	~p_tau+(1|manifest)+cngeneson+pc_mito+brain_region+Sex	0.477	4038.000
ENSG00000135776	0.027	0.091	0.153	0.028	ABCB10	0.151	~p_tau+(1|manifest)+cngeneson+pc_mito+brain_region+Sex	0.368	4484.000
ENSG00000054983	0.038	0.091	0.157	0.024	GALC	0.182	~p_tau+(1|manifest)+cngeneson+pc_mito+brain_region+Sex	0.489	3986.000
ENSG00000196220	0.795	0.091	0.487	-0.306	SRGAP3	0.900	~p_tau+(1|manifest)+cngeneson+pc_mito+brain_region+Sex	4.611	417.000
ENSG00000137942	0.310	0.091	0.280	-0.098	FNBP1L	0.565	~p_tau+(1|manifest)+cngeneson+pc_mito+brain_region+Sex	0.598	3547.000
ENSG00000089220	0.196	0.090	0.198	-0.017	PEBP1	0.438	~p_tau+(1|manifest)+cngeneson+pc_mito+brain_region+Sex	1.249	2002.000
ENSG00000168273	0.028	0.090	0.196	-0.015	SMIM4	0.155	~p_tau+(1|manifest)+cngeneson+pc_mito+brain_region+Sex	0.715	3155.000
ENSG00000166925	0.027	0.090	0.162	0.019	TSC22D4	0.152	~p_tau+(1|manifest)+cngeneson+pc_mito+brain_region+Sex	0.592	3565.000
ENSG00000181904	0.045	0.090	0.194	-0.013	C5orf24	0.200	~p_tau+(1|manifest)+cngeneson+pc_mito+brain_region+Sex	0.552	3715.000
ENSG00000146067	0.101	0.090	0.241	-0.060	FAM193B	0.304	~p_tau+(1|manifest)+cngeneson+pc_mito+brain_region+Sex	0.953	2542.000
ENSG00000116095	0.018	0.090	0.159	0.022	PLEKHA3	0.123	~p_tau+(1|manifest)+cngeneson+pc_mito+brain_region+Sex	0.279	4928.000
ENSG00000139324	0.006	0.090	0.153	0.027	TMTC3	0.073	~p_tau+(1|manifest)+cngeneson+pc_mito+brain_region+Sex	0.210	5209.000
ENSG00000114353	0.276	0.090	0.221	-0.041	GNAI2	0.529	~p_tau+(1|manifest)+cngeneson+pc_mito+brain_region+Sex	1.015	2408.000
ENSG00000182118	0.102	0.090	0.219	-0.039	FAM89A	0.304	~p_tau+(1|manifest)+cngeneson+pc_mito+brain_region+Sex	1.152	2162.000
ENSG00000163156	0.034	0.090	0.225	-0.045	SCNM1	0.173	~p_tau+(1|manifest)+cngeneson+pc_mito+brain_region+Sex	0.950	2544.000
ENSG00000170852	0.060	0.090	0.180	0.000	KBTBD2	0.235	~p_tau+(1|manifest)+cngeneson+pc_mito+brain_region+Sex	0.437	4189.000
ENSG00000150316	0.011	0.090	0.162	0.017	CWC15	0.094	~p_tau+(1|manifest)+cngeneson+pc_mito+brain_region+Sex	0.531	3794.000
ENSG00000047410	0.500	0.090	0.242	-0.062	TPR	0.722	~p_tau+(1|manifest)+cngeneson+pc_mito+brain_region+Sex	0.523	3824.000
ENSG00000047230	0.066	0.090	0.194	-0.015	CTPS2	0.244	~p_tau+(1|manifest)+cngeneson+pc_mito+brain_region+Sex	0.265	4994.000
ENSG00000197302	0.007	0.090	0.161	0.018	ZNF720	0.078	~p_tau+(1|manifest)+cngeneson+pc_mito+brain_region+Sex	0.249	5073.000
ENSG00000019582	0.347	0.089	0.297	-0.118	CD74	0.600	~p_tau+(1|manifest)+cngeneson+pc_mito+brain_region+Sex	7.743	174.000
ENSG00000008311	0.209	0.089	0.289	-0.110	AASS	0.452	~p_tau+(1|manifest)+cngeneson+pc_mito+brain_region+Sex	1.620	1532.000
ENSG00000122958	0.165	0.089	0.200	-0.021	VPS26A	0.396	~p_tau+(1|manifest)+cngeneson+pc_mito+brain_region+Sex	0.894	2682.000
ENSG00000204217	0.348	0.089	0.286	-0.107	BMPR2	0.601	~p_tau+(1|manifest)+cngeneson+pc_mito+brain_region+Sex	2.095	1190.000
ENSG00000140199	0.564	0.089	0.270	-0.091	SLC12A6	0.764	~p_tau+(1|manifest)+cngeneson+pc_mito+brain_region+Sex	0.449	4150.000
ENSG00000179981	0.374	0.089	0.230	-0.051	TSHZ1	0.626	~p_tau+(1|manifest)+cngeneson+pc_mito+brain_region+Sex	2.589	931.000
ENSG00000153037	0.198	0.089	0.207	-0.029	SRP19	0.440	~p_tau+(1|manifest)+cngeneson+pc_mito+brain_region+Sex	0.748	3067.000
ENSG00000102755	0.429	0.089	0.345	-0.166	FLT1	0.670	~p_tau+(1|manifest)+cngeneson+pc_mito+brain_region+Sex	2.113	1177.000
ENSG00000134369	0.536	0.089	0.288	-0.110	NAV1	0.744	~p_tau+(1|manifest)+cngeneson+pc_mito+brain_region+Sex	0.830	2861.000
ENSG00000115548	0.748	0.089	0.341	-0.163	KDM3A	0.873	~p_tau+(1|manifest)+cngeneson+pc_mito+brain_region+Sex	2.594	929.000
ENSG00000137834	0.172	0.089	0.211	-0.033	SMAD6	0.406	~p_tau+(1|manifest)+cngeneson+pc_mito+brain_region+Sex	0.717	3144.000
ENSG00000060971	0.090	0.089	0.188	-0.010	ACAA1	0.285	~p_tau+(1|manifest)+cngeneson+pc_mito+brain_region+Sex	0.305	4803.000
ENSG00000183283	0.036	0.089	0.202	-0.024	DAZAP2	0.178	~p_tau+(1|manifest)+cngeneson+pc_mito+brain_region+Sex	0.795	2945.000
ENSG00000177728	0.009	0.089	0.153	0.025	TMEM94	0.087	~p_tau+(1|manifest)+cngeneson+pc_mito+brain_region+Sex	0.466	4078.000
ENSG00000096401	0.194	0.089	0.194	-0.016	CDC5L	0.435	~p_tau+(1|manifest)+cngeneson+pc_mito+brain_region+Sex	0.128	5425.000
ENSG00000120889	0.292	0.089	0.254	-0.076	TNFRSF10B	0.545	~p_tau+(1|manifest)+cngeneson+pc_mito+brain_region+Sex	1.256	1984.000
ENSG00000135870	0.398	0.089	0.232	-0.054	RC3H1	0.644	~p_tau+(1|manifest)+cngeneson+pc_mito+brain_region+Sex	0.492	3970.000
ENSG00000225921	0.052	0.089	0.202	-0.024	NOL7	0.218	~p_tau+(1|manifest)+cngeneson+pc_mito+brain_region+Sex	0.754	3054.000
ENSG00000107669	0.060	0.088	0.158	0.019	ATE1	0.233	~p_tau+(1|manifest)+cngeneson+pc_mito+brain_region+Sex	0.339	4633.000
ENSG00000119638	0.103	0.088	0.189	-0.012	NEK9	0.307	~p_tau+(1|manifest)+cngeneson+pc_mito+brain_region+Sex	0.415	4295.000
ENSG00000169902	0.133	0.088	0.191	-0.014	TPST1	0.354	~p_tau+(1|manifest)+cngeneson+pc_mito+brain_region+Sex	0.920	2617.000
ENSG00000143157	0.057	0.088	0.165	0.011	POGK	0.226	~p_tau+(1|manifest)+cngeneson+pc_mito+brain_region+Sex	0.487	3992.000

ENSG00000138386	0.171	0.088	0.190	-0.014	NAB1	0.406	~p_tau+(1|manifest)+cngeneson+pc_mito+brain_region+Sex	0.582	3607.000
ENSG00000182087	0.369	0.088	0.376	-0.200	TMEM259	0.622	~p_tau+(1|manifest)+cngeneson+pc_mito+brain_region+Sex	2.630	919.000
ENSG00000076554	0.086	0.088	0.176	0.000	TPD52	0.280	~p_tau+(1|manifest)+cngeneson+pc_mito+brain_region+Sex	0.572	3639.000
ENSG00000110075	0.592	0.088	0.261	-0.086	PPP6R3	0.782	~p_tau+(1|manifest)+cngeneson+pc_mito+brain_region+Sex	1.265	1972.000
ENSG00000118515	0.766	0.088	0.333	-0.157	SGK1	0.883	~p_tau+(1|manifest)+cngeneson+pc_mito+brain_region+Sex	2.135	1161.000
ENSG00000158636	0.459	0.087	0.274	-0.099	EMSY	0.692	~p_tau+(1|manifest)+cngeneson+pc_mito+brain_region+Sex	1.052	2345.000
ENSG00000116704	0.124	0.087	0.173	0.002	SLC35D1	0.340	~p_tau+(1|manifest)+cngeneson+pc_mito+brain_region+Sex	0.327	4696.000
ENSG00000230989	0.021	0.087	0.185	-0.010	HSBP1	0.136	~p_tau+(1|manifest)+cngeneson+pc_mito+brain_region+Sex	0.322	4719.000
ENSG00000161929	0.579	0.087	0.275	-0.100	SCIMP	0.775	~p_tau+(1|manifest)+cngeneson+pc_mito+brain_region+Sex	1.353	1828.000
ENSG00000118482	0.720	0.087	0.299	-0.125	PHF3	0.860	~p_tau+(1|manifest)+cngeneson+pc_mito+brain_region+Sex	1.153	2159.000
ENSG00000170085	0.175	0.087	0.177	-0.003	SIMC1	0.411	~p_tau+(1|manifest)+cngeneson+pc_mito+brain_region+Sex	0.268	4976.000
ENSG00000178913	0.085	0.087	0.182	-0.008	TAF7	0.278	~p_tau+(1|manifest)+cngeneson+pc_mito+brain_region+Sex	0.556	3696.000
ENSG00000185753	0.007	0.087	0.169	0.005	CXorf38	0.074	~p_tau+(1|manifest)+cngeneson+pc_mito+brain_region+Sex	0.640	3392.000
ENSG00000033867	0.874	0.087	0.448	-0.274	SLC4A7	0.942	~p_tau+(1|manifest)+cngeneson+pc_mito+brain_region+Sex	8.240	151.000
ENSG00000003056	0.053	0.087	0.195	-0.021	M6PR	0.218	~p_tau+(1|manifest)+cngeneson+pc_mito+brain_region+Sex	0.845	2809.000
ENSG00000100644	0.966	0.087	0.928	-0.754	HIF1A	0.984	~p_tau+(1|manifest)+cngeneson+pc_mito+brain_region+Sex	22.652	17.000
ENSG00000112137	0.762	0.087	0.482	-0.308	PHACTR1	0.880	~p_tau+(1|manifest)+cngeneson+pc_mito+brain_region+Sex	3.070	747.000
ENSG00000079950	0.529	0.087	0.246	-0.072	STX7	0.740	~p_tau+(1|manifest)+cngeneson+pc_mito+brain_region+Sex	1.873	1337.000
ENSG00000172869	0.092	0.087	0.169	0.005	DMXL1	0.288	~p_tau+(1|manifest)+cngeneson+pc_mito+brain_region+Sex	0.326	4703.000
ENSG00000130830	0.205	0.087	0.200	-0.027	MPP1	0.449	~p_tau+(1|manifest)+cngeneson+pc_mito+brain_region+Sex	1.285	1933.000
ENSG00000181982	0.627	0.087	0.266	-0.093	CCDC149	0.804	~p_tau+(1|manifest)+cngeneson+pc_mito+brain_region+Sex	1.380	1793.000
ENSG00000179021	0.011	0.087	0.163	0.010	C3orf38	0.094	~p_tau+(1|manifest)+cngeneson+pc_mito+brain_region+Sex	0.291	4858.000
ENSG00000111266	0.119	0.087	0.333	-0.160	DUSP16	0.334	~p_tau+(1|manifest)+cngeneson+pc_mito+brain_region+Sex	1.285	1932.000
ENSG00000173511	0.037	0.086	0.203	-0.030	VEGFB	0.180	~p_tau+(1|manifest)+cngeneson+pc_mito+brain_region+Sex	1.351	1830.000
ENSG00000100934	0.016	0.086	0.188	-0.015	SEC23A	0.117	~p_tau+(1|manifest)+cngeneson+pc_mito+brain_region+Sex	0.248	5080.000
ENSG00000196236	0.016	0.086	0.143	0.030	XPNPEP3	0.115	~p_tau+(1|manifest)+cngeneson+pc_mito+brain_region+Sex	0.144	5398.000
ENSG00000162959	0.142	0.086	0.223	-0.050	MEMO1	0.366	~p_tau+(1|manifest)+cngeneson+pc_mito+brain_region+Sex	0.805	2924.000
ENSG00000120451	0.467	0.086	0.274	-0.101	SNX19	0.699	~p_tau+(1|manifest)+cngeneson+pc_mito+brain_region+Sex	1.374	1798.000
ENSG00000038382	0.065	0.086	0.163	0.009	TRIO	0.243	~p_tau+(1|manifest)+cngeneson+pc_mito+brain_region+Sex	0.747	3070.000
ENSG00000177879	0.334	0.086	0.204	-0.032	AP3S1	0.591	~p_tau+(1|manifest)+cngeneson+pc_mito+brain_region+Sex	0.581	3614.000
ENSG00000143612	0.017	0.086	0.147	0.024	C1orf43	0.122	~p_tau+(1|manifest)+cngeneson+pc_mito+brain_region+Sex	0.181	5289.000
ENSG00000105793	0.024	0.086	0.145	0.026	GTPBP10	0.141	~p_tau+(1|manifest)+cngeneson+pc_mito+brain_region+Sex	0.183	5286.000
ENSG00000143499	0.235	0.086	0.192	-0.020	SMYD2	0.485	~p_tau+(1|manifest)+cngeneson+pc_mito+brain_region+Sex	0.253	5057.000
ENSG00000132485	0.607	0.086	0.258	-0.087	ZRANB2	0.790	~p_tau+(1|manifest)+cngeneson+pc_mito+brain_region+Sex	0.708	3178.000
ENSG00000182871	0.382	0.086	0.245	-0.074	COL18A1	0.631	~p_tau+(1|manifest)+cngeneson+pc_mito+brain_region+Sex	2.104	1185.000
ENSG00000184277	0.047	0.086	0.171	0.000	TM2D3	0.202	~p_tau+(1|manifest)+cngeneson+pc_mito+brain_region+Sex	0.373	4464.000
ENSG00000035115	0.080	0.085	0.168	0.002	SH3YL1	0.269	~p_tau+(1|manifest)+cngeneson+pc_mito+brain_region+Sex	0.280	4925.000
ENSG00000104687	0.047	0.085	0.189	-0.019	GSR	0.202	~p_tau+(1|manifest)+cngeneson+pc_mito+brain_region+Sex	0.699	3204.000
ENSG00000096717	0.265	0.085	0.202	-0.031	SIRT1	0.519	~p_tau+(1|manifest)+cngeneson+pc_mito+brain_region+Sex	0.525	3815.000
ENSG00000114742	0.155	0.085	0.206	-0.035	WDR48	0.383	~p_tau+(1|manifest)+cngeneson+pc_mito+brain_region+Sex	0.738	3088.000
ENSG00000087884	0.077	0.085	0.191	-0.021	AAMDC	0.263	~p_tau+(1|manifest)+cngeneson+pc_mito+brain_region+Sex	0.959	2527.000
ENSG00000165629	0.037	0.085	0.153	0.017	ATP5F1C	0.180	~p_tau+(1|manifest)+cngeneson+pc_mito+brain_region+Sex	0.215	5191.000
ENSG00000067182	0.408	0.085	0.294	-0.124	TNFRSF1A	0.654	~p_tau+(1|manifest)+cngeneson+pc_mito+brain_region+Sex	2.405	1016.000
ENSG00000177731	0.021	0.085	0.164	0.006	FLII	0.134	~p_tau+(1|manifest)+cngeneson+pc_mito+brain_region+Sex	0.440	4173.000
ENSG00000052795	0.725	0.085	0.290	-0.121	FNIP2	0.862	~p_tau+(1|manifest)+cngeneson+pc_mito+brain_region+Sex	1.980	1258.000
ENSG00000109220	0.541	0.085	0.234	-0.065	CHIC2	0.749	~p_tau+(1|manifest)+cngeneson+pc_mito+brain_region+Sex	0.780	2988.000
ENSG00000163389	0.338	0.085	0.203	-0.034	POGLUT1	0.594	~p_tau+(1|manifest)+cngeneson+pc_mito+brain_region+Sex	1.011	2413.000
ENSG00000065615	0.414	0.084	0.233	-0.064	CYB5R4	0.658	~p_tau+(1|manifest)+cngeneson+pc_mito+brain_region+Sex	0.428	4229.000
ENSG00000142552	0.070	0.084	0.214	-0.045	RCN3	0.250	~p_tau+(1|manifest)+cngeneson+pc_mito+brain_region+Sex	1.156	2152.000
ENSG00000267680	0.003	0.084	0.152	0.017	ZNF224	0.055	~p_tau+(1|manifest)+cngeneson+pc_mito+brain_region+Sex	0.297	4825.000
ENSG00000103197	0.004	0.084	0.132	0.037	TSC2	0.057	~p_tau+(1|manifest)+cngeneson+pc_mito+brain_region+Sex	0.226	5157.000
ENSG00000028528	0.179	0.084	0.257	-0.089	SNX1	0.416	~p_tau+(1|manifest)+cngeneson+pc_mito+brain_region+Sex	0.768	3017.000
ENSG00000132635	0.133	0.084	0.194	-0.025	PCED1A	0.354	~p_tau+(1|manifest)+cngeneson+pc_mito+brain_region+Sex	0.780	2989.000
ENSG00000089177	0.827	0.084	0.351	-0.182	KIF16B	0.919	~p_tau+(1|manifest)+cngeneson+pc_mito+brain_region+Sex	1.476	1688.000
ENSG00000163961	0.063	0.084	0.158	0.010	RNF168	0.240	~p_tau+(1|manifest)+cngeneson+pc_mito+brain_region+Sex	0.289	4874.000
ENSG00000101577	0.747	0.084	0.316	-0.148	LPIN2	0.873	~p_tau+(1|manifest)+cngeneson+pc_mito+brain_region+Sex	1.257	1983.000
ENSG00000204628	0.229	0.084	0.304	-0.137	RACK1	0.478	~p_tau+(1|manifest)+cngeneson+pc_mito+brain_region+Sex	1.548	1612.000
ENSG00000258227	0.600	0.084	0.356	-0.188	CLEC5A	0.787	~p_tau+(1|manifest)+cngeneson+pc_mito+brain_region+Sex	5.850	293.000
ENSG00000138119	0.658	0.084	0.290	-0.123	MYOF	0.823	~p_tau+(1|manifest)+cngeneson+pc_mito+brain_region+Sex	0.539	3763.000
ENSG00000077044	0.466	0.084	0.257	-0.090	DGKD	0.697	~p_tau+(1|manifest)+cngeneson+pc_mito+brain_region+Sex	0.584	3596.000
ENSG00000129292	0.655	0.084	0.261	-0.094	PHF20L1	0.822	~p_tau+(1|manifest)+cngeneson+pc_mito+brain_region+Sex	0.642	3383.000
ENSG00000130511	0.151	0.083	0.208	-0.041	SSBP4	0.378	~p_tau+(1|manifest)+cngeneson+pc_mito+brain_region+Sex	0.968	2502.000
ENSG00000118508	0.114	0.083	0.210	-0.043	RAB32	0.326	~p_tau+(1|manifest)+cngeneson+pc_mito+brain_region+Sex	0.924	2607.000
ENSG00000140463	0.010	0.083	0.132	0.034	BBS4	0.089	~p_tau+(1|manifest)+cngeneson+pc_mito+brain_region+Sex	0.081	5460.000
ENSG00000140497	0.384	0.083	0.258	-0.092	SCAMP2	0.632	~p_tau+(1|manifest)+cngeneson+pc_mito+brain_region+Sex	1.322	1876.000
ENSG00000182179	0.150	0.083	0.250	-0.084	UBA7	0.377	~p_tau+(1|manifest)+cngeneson+pc_mito+brain_region+Sex	2.104	1182.000
ENSG00000161800	0.016	0.083	0.142	0.024	RACGAP1	0.117	~p_tau+(1|manifest)+cngeneson+pc_mito+brain_region+Sex	0.276	4946.000
ENSG00000131373	0.193	0.083	0.173	-0.007	HACL1	0.434	~p_tau+(1|manifest)+cngeneson+pc_mito+brain_region+Sex	0.345	4606.000
ENSG00000131966	0.000	0.083	0.121	0.044	ACTR10	0.015	~p_tau+(1|manifest)+cngeneson+pc_mito+brain_region+Sex	0.244	5094.000
ENSG00000166333	0.146	0.083	0.185	-0.020	ILK	0.370	~p_tau+(1|manifest)+cngeneson+pc_mito+brain_region+Sex	0.706	3189.000
ENSG00000133030	0.613	0.083	0.300	-0.135	MPRIP	0.793	~p_tau+(1|manifest)+cngeneson+pc_mito+brain_region+Sex	0.918	2627.000
ENSG00000163866	0.027	0.083	0.167	-0.002	SMIM12	0.152	~p_tau+(1|manifest)+cngeneson+pc_mito+brain_region+Sex	0.404	4337.000
ENSG00000168386	0.162	0.083	0.239	-0.074	FILIP1L	0.392	~p_tau+(1|manifest)+cngeneson+pc_mito+brain_region+Sex	0.879	2719.000
ENSG00000163428	0.004	0.083	0.185	-0.020	LRRC58	0.057	~p_tau+(1|manifest)+cngeneson+pc_mito+brain_region+Sex	0.185	5278.000
ENSG00000163634	0.280	0.082	0.184	-0.019	THOC7	0.533	~p_tau+(1|manifest)+cngeneson+pc_mito+brain_region+Sex	0.414	4297.000
ENSG00000185963	0.087	0.082	0.216	-0.051	BICD2	0.280	~p_tau+(1|manifest)+cngeneson+pc_mito+brain_region+Sex	0.889	2694.000
ENSG00000179104	0.748	0.082	0.306	-0.141	TMTC2	0.873	~p_tau+(1|manifest)+cngeneson+pc_mito+brain_region+Sex	1.561	1606.000
ENSG00000156958	0.284	0.082	0.182	-0.017	GALK2	0.537	~p_tau+(1|manifest)+cngeneson+pc_mito+brain_region+Sex	0.332	4669.000
ENSG00000198947	0.156	0.082	0.169	-0.004	DMD	0.384	~p_tau+(1|manifest)+cngeneson+pc_mito+brain_region+Sex	0.712	3165.000
ENSG00000169813	0.243	0.082	0.250	-0.086	HNRNPF	0.494	~p_tau+(1|manifest)+cngeneson+pc_mito+brain_region+Sex	1.337	1854.000
ENSG00000154124	0.089	0.082	0.201	-0.036	OTULIN	0.283	~p_tau+(1|manifest)+cngeneson+pc_mito+brain_region+Sex	0.427	4235.000
ENSG00000186468	0.343	0.082	0.299	-0.136	RPS23	0.597	~p_tau+(1|manifest)+cngeneson+pc_mito+brain_region+Sex	1.604	1553.000
ENSG00000003756	0.018	0.082	0.253	-0.089	RBM5	0.124	~p_tau+(1|manifest)+cngeneson+pc_mito+brain_region+Sex	0.668	3302.000
ENSG00000165861	0.043	0.081	0.152	0.010	ZFYVE1	0.195	~p_tau+(1|manifest)+cngeneson+pc_mito+brain_region+Sex	0.580	3617.000
ENSG00000084070	0.747	0.081	0.335	-0.172	SMAP2	0.873	~p_tau+(1|manifest)+cngeneson+pc_mito+brain_region+Sex	6.794	216.000
ENSG00000168014	0.019	0.081	0.149	0.014	C2CD3	0.126	~p_tau+(1|manifest)+cngeneson+pc_mito+brain_region+Sex	0.283	4912.000
ENSG00000072364	0.466	0.081	0.231	-0.069	AFF4	0.697	~p_tau+(1|manifest)+cngeneson+pc_mito+brain_region+Sex	1.156	2151.000
ENSG00000090621	0.519	0.081	0.372	-0.209	PABPC4	0.733	~p_tau+(1|manifest)+cngeneson+pc_mito+brain_region+Sex	1.761	1415.000
ENSG00000121486	0.025	0.081	0.203	-0.041	TRMT1L	0.147	~p_tau+(1|manifest)+cngeneson+pc_mito+brain_region+Sex	0.627	3438.000
ENSG00000169087	0.761	0.081	0.392	-0.230	HSPBAP1	0.880	~p_tau+(1|manifest)+cngeneson+pc_mito+brain_region+Sex	2.288	1078.000
ENSG00000119004	0.226	0.081	0.202	-0.040	CYP20A1	0.473	~p_tau+(1|manifest)+cngeneson+pc_mito+brain_region+Sex	0.277	4939.000
ENSG00000162341	0.014	0.081	0.206	-0.044	TPCN2	0.109	~p_tau+(1|manifest)+cngeneson+pc_mito+brain_region+Sex	0.944	2561.000
ENSG00000133884	0.007	0.081	0.133	0.029	DPF2	0.075	~p_tau+(1|manifest)+cngeneson+pc_mito+brain_region+Sex	0.261	5019.000
ENSG00000151148	0.002	0.081	0.133	0.028	UBE3B	0.047	~p_tau+(1|manifest)+cngeneson+pc_mito+brain_region+Sex	0.180	5292.000
ENSG00000145741	0.050	0.081	0.192	-0.030	BTF3	0.210	~p_tau+(1|manifest)+cngeneson+pc_mito+brain_region+Sex	0.715	3148.000
ENSG00000125149	0.529	0.081	0.234	-0.073	C16orf70	0.740	~p_tau+(1|manifest)+cngeneson+pc_mito+brain_region+Sex	0.847	2802.000
ENSG00000136021	0.064	0.080	0.155	0.006	SCYL2	0.242	~p_tau+(1|manifest)+cngeneson+pc_mito+brain_region+Sex	0.341	4626.000
ENSG00000008130	0.007	0.080	0.145	0.016	NADK	0.075	~p_tau+(1|manifest)+cngeneson+pc_mito+brain_region+Sex	0.474	4043.000
ENSG00000186812	0.113	0.080	0.264	-0.103	ZNF397	0.324	~p_tau+(1|manifest)+cngeneson+pc_mito+brain_region+Sex	1.102	2249.000
ENSG00000113658	0.539	0.080	0.226	-0.065	SMAD5	0.747	~p_tau+(1|manifest)+cngeneson+pc_mito+brain_region+Sex	0.447	4157.000
ENSG00000113593	0.121	0.080	0.184	-0.024	PPWD1	0.336	~p_tau+(1|manifest)+cngeneson+pc_mito+brain_region+Sex	0.714	3157.000
ENSG00000068745	0.024	0.080	0.142	0.018	IP6K2	0.142	~p_tau+(1|manifest)+cngeneson+pc_mito+brain_region+Sex	0.276	4940.000
ENSG00000111802	0.038	0.080	0.154	0.006	TDP2	0.183	~p_tau+(1|manifest)+cngeneson+pc_mito+brain_region+Sex	0.249	5077.000
ENSG00000179152	0.387	0.080	0.194	-0.034	TCAIM	0.636	~p_tau+(1|manifest)+cngeneson+pc_mito+brain_region+Sex	0.236	5127.000
ENSG00000126775	0.023	0.080	0.161	-0.001	ATG14	0.140	~p_tau+(1|manifest)+cngeneson+pc_mito+brain_region+Sex	0.341	4622.000
ENSG00000077157	0.187	0.080	0.210	-0.051	PPP1R12B	0.427	~p_tau+(1|manifest)+cngeneson+pc_mito+brain_region+Sex	0.294	4841.000
ENSG00000130723	0.460	0.080	0.206	-0.047	PRRC2B	0.692	~p_tau+(1|manifest)+cngeneson+pc_mito+brain_region+Sex	1.122	2216.000
ENSG00000129534	0.696	0.080	0.601	-0.441	MIS18BP1	0.846	~p_tau+(1|manifest)+cngeneson+pc_mito+brain_region+Sex	11.023	85.000
ENSG00000102309	0.041	0.080	0.143	0.016	PIN4	0.189	~p_tau+(1|manifest)+cngeneson+pc_mito+brain_region+Sex	0.293	4852.000
ENSG00000005889	0.227	0.080	0.181	-0.022	ZFX	0.476	~p_tau+(1|manifest)+cngeneson+pc_mito+brain_region+Sex	2.333	1051.000
ENSG00000155846	0.557	0.080	0.225	-0.066	PPARGC1B	0.761	~p_tau+(1|manifest)+cngeneson+pc_mito+brain_region+Sex	1.122	2213.000
ENSG00000066136	0.390	0.080	0.220	-0.061	NFYC	0.638	~p_tau+(1|manifest)+cngeneson+pc_mito+brain_region+Sex	0.416	4288.000
ENSG00000116668	0.200	0.079	0.166	-0.008	SWT1	0.442	~p_tau+(1|manifest)+cngeneson+pc_mito+brain_region+Sex	0.626	3439.000
ENSG00000110696	0.133	0.079	0.170	-0.011	C11orf58	0.354	~p_tau+(1|manifest)+cngeneson+pc_mito+brain_region+Sex	0.336	4653.000
ENSG00000170502	0.057	0.079	0.212	-0.053	NUDT9	0.226	~p_tau+(1|manifest)+cngeneson+pc_mito+brain_region+Sex	1.357	1821.000
ENSG00000146731	0.270	0.079	0.247	-0.089	CCT6A	0.523	~p_tau+(1|manifest)+cngeneson+pc_mito+brain_region+Sex	0.915	2633.000
ENSG00000106608	0.193	0.079	0.166	-0.007	URGCP	0.434	~p_tau+(1|manifest)+cngeneson+pc_mito+brain_region+Sex	0.526	3810.000
ENSG00000135605	0.077	0.079	0.174	-0.016	TEC	0.264	~p_tau+(1|manifest)+cngeneson+pc_mito+brain_region+Sex	0.849	2799.000
ENSG00000101665	0.195	0.079	0.197	-0.039	SMAD7	0.437	~p_tau+(1|manifest)+cngeneson+pc_mito+brain_region+Sex	0.867	2753.000
ENSG00000186470	0.033	0.079	0.219	-0.061	BTN3A2	0.170	~p_tau+(1|manifest)+cngeneson+pc_mito+brain_region+Sex	1.106	2242.000
ENSG00000006451	0.003	0.079	0.151	0.006	RALA	0.055	~p_tau+(1|manifest)+cngeneson+pc_mito+brain_region+Sex	0.402	4348.000
ENSG00000090470	0.140	0.079	0.174	-0.017	PDCD7	0.363	~p_tau+(1|manifest)+cngeneson+pc_mito+brain_region+Sex	0.571	3645.000
ENSG00000136167	0.866	0.078	0.547	-0.390	LCP1	0.939	~p_tau+(1|manifest)+cngeneson+pc_mito+brain_region+Sex	4.693	407.000
ENSG00000101150	0.092	0.078	0.175	-0.018	TPD52L2	0.289	~p_tau+(1|manifest)+cngeneson+pc_mito+brain_region+Sex	0.492	3973.000
ENSG00000070950	0.072	0.078	0.161	-0.005	RAD18	0.252	~p_tau+(1|manifest)+cngeneson+pc_mito+brain_region+Sex	0.195	5247.000
ENSG00000101391	0.023	0.078	0.136	0.020	CDK5RAP1	0.141	~p_tau+(1|manifest)+cngeneson+pc_mito+brain_region+Sex	0.452	4133.000
ENSG00000044446	0.002	0.078	0.130	0.026	PHKA2	0.044	~p_tau+(1|manifest)+cngeneson+pc_mito+brain_region+Sex	0.062	5464.000
ENSG00000165795	0.226	0.078	0.226	-0.070	NDRG2	0.473	~p_tau+(1|manifest)+cngeneson+pc_mito+brain_region+Sex	1.517	1644.000
ENSG00000048028	0.004	0.078	0.133	0.023	USP28	0.058	~p_tau+(1|manifest)+cngeneson+pc_mito+brain_region+Sex	0.128	5428.000
ENSG00000147050	0.536	0.078	0.232	-0.077	KDM6A	0.744	~p_tau+(1|manifest)+cngeneson+pc_mito+brain_region+Sex	4.480	438.000
ENSG00000163166	0.177	0.077	0.192	-0.038	IWS1	0.414	~p_tau+(1|manifest)+cngeneson+pc_mito+brain_region+Sex	0.283	4909.000
ENSG00000136936	0.019	0.077	0.170	-0.015	XPA	0.127	~p_tau+(1|manifest)+cngeneson+pc_mito+brain_region+Sex	0.297	4828.000
ENSG00000188092	0.036	0.077	0.188	-0.033	GPR89B	0.178	~p_tau+(1|manifest)+cngeneson+pc_mito+brain_region+Sex	0.765	3027.000
ENSG00000166398	0.065	0.077	0.241	-0.087	KIAA0355	0.243	~p_tau+(1|manifest)+cngeneson+pc_mito+brain_region+Sex	1.199	2085.000
ENSG00000162852	0.158	0.077	0.156	-0.002	CNST	0.388	~p_tau+(1|manifest)+cngeneson+pc_mito+brain_region+Sex	0.306	4793.000
ENSG00000076242	0.033	0.077	0.169	-0.016	MLH1	0.169	~p_tau+(1|manifest)+cngeneson+pc_mito+brain_region+Sex	0.501	3925.000
ENSG00000001629	0.121	0.076	0.191	-0.039	ANKIB1	0.336	~p_tau+(1|manifest)+cngeneson+pc_mito+brain_region+Sex	0.621	3460.000
ENSG00000083457	0.014	0.076	0.140	0.013	ITGAE	0.106	~p_tau+(1|manifest)+cngeneson+pc_mito+brain_region+Sex	0.326	4701.000
ENSG00000100320	0.471	0.076	0.216	-0.064	RBFOX2	0.700	~p_tau+(1|manifest)+cngeneson+pc_mito+brain_region+Sex	1.212	2058.000
ENSG00000107779	0.767	0.076	0.284	-0.131	BMPR1A	0.884	~p_tau+(1|manifest)+cngeneson+pc_mito+brain_region+Sex	0.613	3481.000
ENSG00000174574	0.011	0.076	0.125	0.028	AKIRIN1	0.094	~p_tau+(1|manifest)+cngeneson+pc_mito+brain_region+Sex	0.162	5350.000
ENSG00000164941	0.055	0.076	0.140	0.012	INTS8	0.224	~p_tau+(1|manifest)+cngeneson+pc_mito+brain_region+Sex	0.081	5461.000
ENSG00000176978	0.308	0.076	0.287	-0.136	DPP7	0.564	~p_tau+(1|manifest)+cngeneson+pc_mito+brain_region+Sex	1.458	1709.000
ENSG00000110955	0.098	0.076	0.169	-0.017	ATP5F1B	0.298	~p_tau+(1|manifest)+cngeneson+pc_mito+brain_region+Sex	0.897	2676.000
ENSG00000067900	0.625	0.076	0.228	-0.076	ROCK1	0.802	~p_tau+(1|manifest)+cngeneson+pc_mito+brain_region+Sex	0.907	2657.000
ENSG00000165355	0.417	0.076	0.218	-0.066	FBXO33	0.660	~p_tau+(1|manifest)+cngeneson+pc_mito+brain_region+Sex	1.108	2239.000
ENSG00000196935	0.734	0.076	0.403	-0.252	SRGAP1	0.867	~p_tau+(1|manifest)+cngeneson+pc_mito+brain_region+Sex	6.027	281.000
ENSG00000197579	0.007	0.076	0.118	0.034	TOPORS	0.076	~p_tau+(1|manifest)+cngeneson+pc_mito+brain_region+Sex	0.165	5345.000
ENSG00000182149	0.107	0.076	0.151	0.000	IST1	0.313	~p_tau+(1|manifest)+cngeneson+pc_mito+brain_region+Sex	0.276	4941.000
ENSG00000048740	0.459	0.076	0.206	-0.055	CELF2	0.692	~p_tau+(1|manifest)+cngeneson+pc_mito+brain_region+Sex	5.961	284.000
ENSG00000232112	0.276	0.075	0.244	-0.093	TMA7	0.529	~p_tau+(1|manifest)+cngeneson+pc_mito+brain_region+Sex	2.187	1138.000
ENSG00000258643	0.084	0.075	0.167	-0.017	[illegible]	0.276	~p_tau+(1|manifest)+cngeneson+pc_mito+brain_region+Sex	0.639	3395.000
ENSG00000166783	0.380	0.075	0.249	-0.099	MARF1	0.629	~p_tau+(1|manifest)+cngeneson+pc_mito+brain_region+Sex	1.313	1887.000
ENSG00000100461	0.024	0.075	0.152	-0.002	RBM23	0.142	~p_tau+(1|manifest)+cngeneson+pc_mito+brain_region+Sex	0.346	4600.000
ENSG00000132300	0.208	0.075	0.175	-0.025	PTCD3	0.450	~p_tau+(1|manifest)+cngeneson+pc_mito+brain_region+Sex	0.349	4585.000
ENSG00000121310	0.216	0.075	0.217	-0.067	ECHDC2	0.460	~p_tau+(1|manifest)+cngeneson+pc_mito+brain_region+Sex	1.192	2090.000
ENSG00000176095	0.110	0.075	0.139	0.011	IP6K1	0.318	~p_tau+(1|manifest)+cngeneson+pc_mito+brain_region+Sex	0.239	5116.000
ENSG00000081803	0.881	0.075	0.452	-0.302	CADPS2	0.946	~p_tau+(1|manifest)+cngeneson+pc_mito+brain_region+Sex	1.298	1916.000
ENSG00000272886	0.097	0.075	0.143	0.007	DCP1A	0.297	~p_tau+(1|manifest)+cngeneson+pc_mito+brain_region+Sex	0.255	5054.000
ENSG00000109381	0.793	0.075	0.296	-0.146	ELF2	0.899	~p_tau+(1|manifest)+cngeneson+pc_mito+brain_region+Sex	1.316	1883.000
ENSG00000049246	0.764	0.075	0.293	-0.143	PER3	0.882	~p_tau+(1|manifest)+cngeneson+pc_mito+brain_region+Sex	1.832	1367.000
ENSG00000070610	0.005	0.075	0.123	0.026	GBA2	0.065	~p_tau+(1|manifest)+cngeneson+pc_mito+brain_region+Sex	0.255	5053.000
ENSG00000178951	0.394	0.075	0.253	-0.104	ZBTB7A	0.640	~p_tau+(1|manifest)+cngeneson+pc_mito+brain_region+Sex	1.305	1902.000
ENSG00000171115	0.361	0.075	0.383	-0.234	GIMAP8	0.615	~p_tau+(1|manifest)+cngeneson+pc_mito+brain_region+Sex	3.461	648.000
ENSG00000147592	0.248	0.074	0.167	-0.018	LACTB2	0.500	~p_tau+(1|manifest)+cngeneson+pc_mito+brain_region+Sex	0.426	4236.000
ENSG00000183066	0.251	0.074	0.234	-0.086	WBP2NL	0.503	~p_tau+(1|manifest)+cngeneson+pc_mito+brain_region+Sex	1.467	1698.000
ENSG00000094975	0.140	0.074	0.181	-0.032	SUCO	0.363	~p_tau+(1|manifest)+cngeneson+pc_mito+brain_region+Sex	0.715	3154.000
ENSG00000184497	0.165	0.074	0.189	-0.040	TMEM255B	0.397	~p_tau+(1|manifest)+cngeneson+pc_mito+brain_region+Sex	0.878	2721.000
ENSG00000117153	0.068	0.074	0.133	0.016	KLHL12	0.246	~p_tau+(1|manifest)+cngeneson+pc_mito+brain_region+Sex	0.257	5040.000
ENSG00000196591	0.155	0.074	0.176	-0.028	HDAC2	0.384	~p_tau+(1|manifest)+cngeneson+pc_mito+brain_region+Sex	0.434	4202.000
ENSG00000018189	0.505	0.074	0.218	-0.070	RUFY3	0.725	~p_tau+(1|manifest)+cngeneson+pc_mito+brain_region+Sex	0.627	3437.000
ENSG00000143727	0.009	0.074	0.132	0.016	ACP1	0.084	~p_tau+(1|manifest)+cngeneson+pc_mito+brain_region+Sex	0.138	5408.000
ENSG00000186153	0.727	0.074	0.253	-0.106	WWOX	0.863	~p_tau+(1|manifest)+cngeneson+pc_mito+brain_region+Sex	0.264	5004.000
ENSG00000160294	0.075	0.074	0.184	-0.036	MCM3AP	0.260	~p_tau+(1|manifest)+cngeneson+pc_mito+brain_region+Sex	0.592	3566.000
ENSG00000160752	0.015	0.074	0.144	0.003	FDPS	0.112	~p_tau+(1|manifest)+cngeneson+pc_mito+brain_region+Sex	0.799	2935.000
ENSG00000197008	0.008	0.074	0.124	0.023	ZNF138	0.081	~p_tau+(1|manifest)+cngeneson+pc_mito+brain_region+Sex	0.171	5328.000
ENSG00000198369	0.374	0.073	0.176	-0.029	SPRED2	0.626	~p_tau+(1|manifest)+cngeneson+pc_mito+brain_region+Sex	0.265	4993.000
ENSG00000185324	0.148	0.073	0.183	-0.036	CDK10	0.373	~p_tau+(1|manifest)+cngeneson+pc_mito+brain_region+Sex	0.679	3269.000
ENSG00000198429	0.389	0.073	0.182	-0.035	ZNF69	0.637	~p_tau+(1|manifest)+cngeneson+pc_mito+brain_region+Sex	0.746	3071.000
ENSG00000198814	0.786	0.073	0.283	-0.136	GK	0.894	~p_tau+(1|manifest)+cngeneson+pc_mito+brain_region+Sex	1.806	1387.000
ENSG00000101109	0.584	0.073	0.211	-0.064	STK4	0.777	~p_tau+(1|manifest)+cngeneson+pc_mito+brain_region+Sex	1.063	2320.000
ENSG00000005436	0.446	0.073	0.192	-0.046	GCFC2	0.683	~p_tau+(1|manifest)+cngeneson+pc_mito+brain_region+Sex	0.668	3300.000
ENSG00000164543	0.847	0.073	0.323	-0.177	STK17A	0.929	~p_tau+(1|manifest)+cngeneson+pc_mito+brain_region+Sex	1.055	2341.000
ENSG00000132155	0.491	0.073	0.191	-0.045	RAF1	0.714	~p_tau+(1|manifest)+cngeneson+pc_mito+brain_region+Sex	0.932	2584.000
ENSG00000066044	0.011	0.073	0.157	-0.011	ELAVL1	0.093	~p_tau+(1|manifest)+cngeneson+pc_mito+brain_region+Sex	0.622	3456.000
ENSG00000121892	0.448	0.073	0.212	-0.066	PDS5A	0.684	~p_tau+(1|manifest)+cngeneson+pc_mito+brain_region+Sex	0.870	2742.000
ENSG00000137177	0.678	0.073	0.263	-0.117	KIF13A	0.837	~p_tau+(1|manifest)+cngeneson+pc_mito+brain_region+Sex	1.255	1989.000
ENSG00000115902	0.429	0.073	0.213	-0.068	SLC1A4	0.670	~p_tau+(1|manifest)+cngeneson+pc_mito+brain_region+Sex	1.381	1789.000
ENSG00000137075	0.535	0.073	0.210	-0.065	RNF38	0.744	~p_tau+(1|manifest)+cngeneson+pc_mito+brain_region+Sex	0.697	3216.000
ENSG00000162688	0.011	0.073	0.124	0.021	AGL	0.093	~p_tau+(1|manifest)+cngeneson+pc_mito+brain_region+Sex	0.450	4146.000
ENSG00000041802	0.319	0.072	0.205	-0.060	LSG1	0.575	~p_tau+(1|manifest)+cngeneson+pc_mito+brain_region+Sex	2.044	1218.000
ENSG00000105383	0.046	0.072	0.173	-0.028	CD33	0.202	~p_tau+(1|manifest)+cngeneson+pc_mito+brain_region+Sex	0.542	3754.000
ENSG00000115652	0.133	0.072	0.150	-0.006	UXS1	0.354	~p_tau+(1|manifest)+cngeneson+pc_mito+brain_region+Sex	0.295	4839.000
ENSG00000135956	0.068	0.072	0.133	0.011	TMEM127	0.246	~p_tau+(1|manifest)+cngeneson+pc_mito+brain_region+Sex	0.342	4619.000
ENSG00000015285	0.051	0.072	0.182	-0.037	WAS	0.214	~p_tau+(1|manifest)+cngeneson+pc_mito+brain_region+Sex	0.516	3859.000
ENSG00000177600	0.882	0.072	0.484	-0.339	RPLP2	0.946	~p_tau+(1|manifest)+cngeneson+pc_mito+brain_region+Sex	4.310	474.000
ENSG00000135905	0.585	0.072	0.210	-0.066	DOCK10	0.778	~p_tau+(1|manifest)+cngeneson+pc_mito+brain_region+Sex	3.162	720.000
ENSG00000175662	0.359	0.072	0.188	-0.044	TOM1L2	0.613	~p_tau+(1|manifest)+cngeneson+pc_mito+brain_region+Sex	0.501	3926.000
ENSG00000136141	0.542	0.072	0.207	-0.062	LRCH1	0.751	~p_tau+(1|manifest)+cngeneson+pc_mito+brain_region+Sex	1.475	1690.000
ENSG00000109472	0.674	0.072	0.237	-0.093	CPE	0.834	~p_tau+(1|manifest)+cngeneson+pc_mito+brain_region+Sex	1.818	1380.000
ENSG00000154889	0.232	0.072	0.243	-0.099	MPPE1	0.481	~p_tau+(1|manifest)+cngeneson+pc_mito+brain_region+Sex	1.754	1417.000
ENSG00000164118	0.193	0.072	0.148	-0.004	CEP44	0.434	~p_tau+(1|manifest)+cngeneson+pc_mito+brain_region+Sex	0.226	5159.000
ENSG00000131626	0.344	0.072	0.163	-0.020	PPFIA1	0.598	~p_tau+(1|manifest)+cngeneson+pc_mito+brain_region+Sex	0.319	4731.000
ENSG00000154511	0.564	0.072	0.204	-0.061	DIPK1A	0.764	~p_tau+(1|manifest)+cngeneson+pc_mito+brain_region+Sex	0.648	3369.000
ENSG00000134330	0.000	0.072	0.166	-0.023	IAH1	0.015	~p_tau+(1|manifest)+cngeneson+pc_mito+brain_region+Sex	0.549	3728.000
ENSG00000068784	0.453	0.071	0.238	-0.095	SRBD1	0.688	~p_tau+(1|manifest)+cngeneson+pc_mito+brain_region+Sex	1.344	1842.000
ENSG00000088833	0.014	0.071	0.141	0.002	NSFL1C	0.108	~p_tau+(1|manifest)+cngeneson+pc_mito+brain_region+Sex	0.208	5217.000
ENSG00000166130	0.446	0.071	0.182	-0.039	IKBIP	0.683	~p_tau+(1|manifest)+cngeneson+pc_mito+brain_region+Sex	0.424	4247.000
ENSG00000197448	0.174	0.071	0.173	-0.030	GSTK1	0.409	~p_tau+(1|manifest)+cngeneson+pc_mito+brain_region+Sex	0.767	3019.000
ENSG00000134569	0.359	0.071	0.498	-0.355	LRP4	0.613	~p_tau+(1|manifest)+cngeneson+pc_mito+brain_region+Sex	9.912	111.000
ENSG00000054267	0.718	0.071	0.244	-0.101	ARID4B	0.859	~p_tau+(1|manifest)+cngeneson+pc_mito+brain_region+Sex	1.292	1927.000
ENSG00000163349	0.256	0.071	0.175	-0.033	HIPK1	0.510	~p_tau+(1|manifest)+cngeneson+pc_mito+brain_region+Sex	0.525	3814.000
ENSG00000130699	0.027	0.071	0.122	0.020	TAF4	0.152	~p_tau+(1|manifest)+cngeneson+pc_mito+brain_region+Sex	0.166	5343.000
ENSG00000163875	0.033	0.071	0.134	0.008	MEAF6	0.170	~p_tau+(1|manifest)+cngeneson+pc_mito+brain_region+Sex	0.538	3766.000
ENSG00000181827	0.487	0.071	0.230	-0.088	RFX7	0.712	~p_tau+(1|manifest)+cngeneson+pc_mito+brain_region+Sex	0.560	3685.000
ENSG00000110446	0.119	0.071	0.143	-0.001	SLC15A3	0.334	~p_tau+(1|manifest)+cngeneson+pc_mito+brain_region+Sex	0.478	4033.000
ENSG00000143756	0.125	0.071	0.194	-0.052	FBXO28	0.342	~p_tau+(1|manifest)+cngeneson+pc_mito+brain_region+Sex	0.336	4651.000
ENSG00000083535	0.771	0.071	0.295	-0.153	PIBF1	0.887	~p_tau+(1|manifest)+cngeneson+pc_mito+brain_region+Sex	1.717	1444.000
ENSG00000081923	0.309	0.071	0.250	-0.109	ATP8B1	0.565	~p_tau+(1|manifest)+cngeneson+pc_mito+brain_region+Sex	2.227	1116.000
ENSG00000010322	0.112	0.071	0.162	-0.020	NISCH	0.323	~p_tau+(1|manifest)+cngeneson+pc_mito+brain_region+Sex	0.712	3166.000
ENSG00000100401	0.120	0.071	0.176	-0.035	RANGAP1	0.336	~p_tau+(1|manifest)+cngeneson+pc_mito+brain_region+Sex	0.649	3366.000
ENSG00000172466	0.278	0.071	0.243	-0.101	ZNF24	0.531	~p_tau+(1|manifest)+cngeneson+pc_mito+brain_region+Sex	1.178	2112.000
ENSG00000100614	0.184	0.071	0.145	-0.003	PPM1A	0.424	~p_tau+(1|manifest)+cngeneson+pc_mito+brain_region+Sex	0.238	5119.000
ENSG00000168090	0.144	0.071	0.159	-0.018	COPS6	0.368	~p_tau+(1|manifest)+cngeneson+pc_mito+brain_region+Sex	0.557	3691.000
ENSG00000140992	0.039	0.071	0.156	-0.014	PDPK1	0.184	~p_tau+(1|manifest)+cngeneson+pc_mito+brain_region+Sex	0.710	3173.000
ENSG00000147894	0.428	0.071	0.225	-0.084	C9orf72	0.669	~p_tau+(1|manifest)+cngeneson+pc_mito+brain_region+Sex	1.268	1967.000
ENSG00000128534	0.244	0.070	0.192	-0.051	LSM8	0.495	~p_tau+(1|manifest)+cngeneson+pc_mito+brain_region+Sex	0.665	3314.000
ENSG00000141258	0.015	0.070	0.134	0.007	SGSM2	0.111	~p_tau+(1|manifest)+cngeneson+pc_mito+brain_region+Sex	0.425	4244.000
ENSG00000114670	0.260	0.070	0.158	-0.017	NEK11	0.514	~p_tau+(1|manifest)+cngeneson+pc_mito+brain_region+Sex	1.121	2219.000
ENSG00000047249	0.580	0.070	0.203	-0.062	ATP6V1H	0.775	~p_tau+(1|manifest)+cngeneson+pc_mito+brain_region+Sex	0.909	2652.000
ENSG00000111676	0.064	0.070	0.153	-0.012	ATN1	0.241	~p_tau+(1|manifest)+cngeneson+pc_mito+brain_region+Sex	0.371	4472.000
ENSG00000167553	0.018	0.070	0.140	0.001	TUBA1C	0.122	~p_tau+(1|manifest)+cngeneson+pc_mito+brain_region+Sex	0.253	5060.000
ENSG00000148737	0.666	0.070	0.235	-0.095	TCF7L2	0.829	~p_tau+(1|manifest)+cngeneson+pc_mito+brain_region+Sex	0.781	2983.000
ENSG00000112200	0.340	0.070	0.179	-0.038	ZNF451	0.596	~p_tau+(1|manifest)+cngeneson+pc_mito+brain_region+Sex	0.409	4317.000
ENSG00000004660	0.264	0.070	0.161	-0.020	CAMKK1	0.517	~p_tau+(1|manifest)+cngeneson+pc_mito+brain_region+Sex	0.546	3740.000
ENSG00000138777	0.256	0.070	0.260	-0.120	PPA2	0.510	~p_tau+(1|manifest)+cngeneson+pc_mito+brain_region+Sex	0.566	3661.000
ENSG00000140931	0.131	0.070	0.154	-0.014	CMTM3	0.351	~p_tau+(1|manifest)+cngeneson+pc_mito+brain_region+Sex	0.450	4145.000
ENSG00000120885	0.860	0.070	0.371	-0.231	CLU	0.937	~p_tau+(1|manifest)+cngeneson+pc_mito+brain_region+Sex	4.620	413.000
ENSG00000072864	0.298	0.070	0.182	-0.042	NDE1	0.553	~p_tau+(1|manifest)+cngeneson+pc_mito+brain_region+Sex	0.511	3880.000
ENSG00000138002	0.126	0.070	0.148	-0.008	IFT172	0.343	~p_tau+(1|manifest)+cngeneson+pc_mito+brain_region+Sex	0.463	4093.000
ENSG00000151327	0.002	0.070	0.104	0.035	FAM177A1	0.040	~p_tau+(1|manifest)+cngeneson+pc_mito+brain_region+Sex	0.171	5330.000
ENSG00000175806	0.555	0.070	0.255	-0.115	MSRA	0.760	~p_tau+(1|manifest)+cngeneson+pc_mito+brain_region+Sex	1.532	1621.000
ENSG00000034677	0.204	0.070	0.159	-0.019	RNF19A	0.447	~p_tau+(1|manifest)+cngeneson+pc_mito+brain_region+Sex	0.128	5424.000
ENSG00000159733	0.128	0.070	0.137	0.003	ZFYVE28	0.346	~p_tau+(1|manifest)+cngeneson+pc_mito+brain_region+Sex	0.161	5353.000
ENSG00000077721	0.067	0.070	0.150	-0.010	UBE2A	0.245	~p_tau+(1|manifest)+cngeneson+pc_mito+brain_region+Sex	0.439	4180.000
ENSG00000204767	0.049	0.070	0.179	-0.040	INSYN2B	0.208	~p_tau+(1|manifest)+cngeneson+pc_mito+brain_region+Sex	1.427	1749.000
ENSG00000164209	0.089	0.070	0.156	-0.017	SLC25A46	0.284	~p_tau+(1|manifest)+cngeneson+pc_mito+brain_region+Sex	0.294	4844.000
ENSG00000167792	0.208	0.070	0.209	-0.069	NDUFV1	0.450	~p_tau+(1|manifest)+cngeneson+pc_mito+brain_region+Sex	1.102	2248.000
ENSG00000096060	0.867	0.070	0.343	-0.203	FKBP5	0.939	~p_tau+(1|manifest)+cngeneson+pc_mito+brain_region+Sex	7.264	198.000
ENSG00000125753	0.046	0.070	0.169	-0.029	VASP	0.202	~p_tau+(1|manifest)+cngeneson+pc_mito+brain_region+Sex	0.580	3615.000
ENSG00000166323	0.473	0.070	0.188	-0.049	C11orf65	0.702	~p_tau+(1|manifest)+cngeneson+pc_mito+brain_region+Sex	1.070	2305.000
ENSG00000132294	0.398	0.070	0.225	-0.086	EFR3A	0.644	~p_tau+(1|manifest)+cngeneson+pc_mito+brain_region+Sex	0.832	2853.000
ENSG00000198964	0.692	0.069	0.230	-0.091	SGMS1	0.844	~p_tau+(1|manifest)+cngeneson+pc_mito+brain_region+Sex	1.040	2364.000
ENSG00000125971	0.067	0.069	0.133	0.006	DYNLRB1	0.245	~p_tau+(1|manifest)+cngeneson+pc_mito+brain_region+Sex	0.524	3819.000
ENSG00000198900	0.730	0.069	0.254	-0.115	TOP1	0.864	~p_tau+(1|manifest)+cngeneson+pc_mito+brain_region+Sex	0.640	3389.000
ENSG00000174579	0.230	0.069	0.165	-0.026	MSL2	0.479	~p_tau+(1|manifest)+cngeneson+pc_mito+brain_region+Sex	0.623	3453.000
ENSG00000197852	0.033	0.069	0.142	-0.003	INKA2	0.169	~p_tau+(1|manifest)+cngeneson+pc_mito+brain_region+Sex	1.331	1860.000
ENSG00000130338	0.627	0.069	0.211	-0.073	TULP4	0.804	~p_tau+(1|manifest)+cngeneson+pc_mito+brain_region+Sex	0.425	4243.000
ENSG00000175115	0.768	0.069	0.257	-0.119	PACS1	0.885	~p_tau+(1|manifest)+cngeneson+pc_mito+brain_region+Sex	1.263	1975.000
ENSG00000148908	0.851	0.069	0.380	-0.242	RGS10	0.932	~p_tau+(1|manifest)+cngeneson+pc_mito+brain_region+Sex	3.279	690.000
ENSG00000006459	0.857	0.069	0.314	-0.176	KDM7A	0.935	~p_tau+(1|manifest)+cngeneson+pc_mito+brain_region+Sex	2.004	1245.000
ENSG00000152223	0.097	0.069	0.156	-0.018	EPG5	0.296	~p_tau+(1|manifest)+cngeneson+pc_mito+brain_region+Sex	0.211	5203.000
ENSG00000147601	0.031	0.069	0.154	-0.016	TERF1	0.162	~p_tau+(1|manifest)+cngeneson+pc_mito+brain_region+Sex	0.195	5244.000
ENSG00000151893	0.559	0.069	0.193	-0.055	CACUL1	0.761	~p_tau+(1|manifest)+cngeneson+pc_mito+brain_region+Sex	0.874	2731.000
ENSG00000023734	0.085	0.069	0.150	-0.012	STRAP	0.277	~p_tau+(1|manifest)+cngeneson+pc_mito+brain_region+Sex	0.481	4019.000
ENSG00000127311	0.537	0.069	0.193	-0.056	HELB	0.746	~p_tau+(1|manifest)+cngeneson+pc_mito+brain_region+Sex	0.681	3262.000
ENSG00000157557	0.896	0.069	0.487	-0.349	ETS2	0.953	~p_tau+(1|manifest)+cngeneson+pc_mito+brain_region+Sex	0.989	2457.000
ENSG00000077713	0.358	0.069	0.198	-0.061	SLC25A43	0.612	~p_tau+(1|manifest)+cngeneson+pc_mito+brain_region+Sex	1.300	1912.000
ENSG00000129518	0.062	0.069	0.136	0.001	EAPP	0.238	~p_tau+(1|manifest)+cngeneson+pc_mito+brain_region+Sex	0.402	4345.000
ENSG00000184787	0.702	0.069	0.345	-0.208	UBE2G2	0.849	~p_tau+(1|manifest)+cngeneson+pc_mito+brain_region+Sex	2.596	928.000
ENSG00000138594	0.248	0.069	0.147	-0.010	TMOD3	0.500	~p_tau+(1|manifest)+cngeneson+pc_mito+brain_region+Sex	0.523	3825.000
ENSG00000165637	0.002	0.068	0.139	-0.003	VDAC2	0.044	~p_tau+(1|manifest)+cngeneson+pc_mito+brain_region+Sex	0.631	3419.000
ENSG00000116584	0.221	0.068	0.146	-0.010	ARHGEF2	0.468	~p_tau+(1|manifest)+cngeneson+pc_mito+brain_region+Sex	0.550	3725.000
ENSG00000174444	0.760	0.068	0.359	-0.222	RPL4	0.879	~p_tau+(1|manifest)+cngeneson+pc_mito+brain_region+Sex	3.030	761.000
ENSG00000138081	0.631	0.068	0.205	-0.069	FBXO11	0.806	~p_tau+(1|manifest)+cngeneson+pc_mito+brain_region+Sex	0.653	3352.000
ENSG00000177853	0.674	0.068	0.220	-0.084	ZNF518A	0.834	~p_tau+(1|manifest)+cngeneson+pc_mito+brain_region+Sex	0.457	4117.000
ENSG00000059728	0.315	0.068	0.166	-0.030	MXD1	0.570	~p_tau+(1|manifest)+cngeneson+pc_mito+brain_region+Sex	1.154	2155.000
ENSG00000127527	0.064	0.068	0.188	-0.052	EPS15L1	0.241	~p_tau+(1|manifest)+cngeneson+pc_mito+brain_region+Sex	0.384	4423.000
ENSG00000022567	0.405	0.068	0.171	-0.036	SLC45A4	0.651	~p_tau+(1|manifest)+cngeneson+pc_mito+brain_region+Sex	0.470	4059.000
ENSG00000068697	0.129	0.068	0.149	-0.013	LAPTM4A	0.348	~p_tau+(1|manifest)+cngeneson+pc_mito+brain_region+Sex	0.785	2974.000
ENSG00000155330	0.085	0.068	0.128	0.008	C16orf87	0.278	~p_tau+(1|manifest)+cngeneson+pc_mito+brain_region+Sex	0.222	5171.000
ENSG00000168137	0.547	0.068	0.256	-0.121	SETD5	0.753	~p_tau+(1|manifest)+cngeneson+pc_mito+brain_region+Sex	1.436	1738.000
ENSG00000123505	0.681	0.068	0.225	-0.089	AMD1	0.839	~p_tau+(1|manifest)+cngeneson+pc_mito+brain_region+Sex	0.750	3064.000
ENSG00000128791	0.075	0.068	0.134	0.001	TWSG1	0.261	~p_tau+(1|manifest)+cngeneson+pc_mito+brain_region+Sex	0.135	5416.000
ENSG00000074657	0.373	0.068	0.178	-0.043	ZNF532	0.625	~p_tau+(1|manifest)+cngeneson+pc_mito+brain_region+Sex	0.469	4064.000
ENSG00000072415	0.018	0.068	0.135	0.000	MPP5	0.124	~p_tau+(1|manifest)+cngeneson+pc_mito+brain_region+Sex	0.501	3922.000
ENSG00000132256	0.289	0.067	0.186	-0.051	TRIM5	0.542	~p_tau+(1|manifest)+cngeneson+pc_mito+brain_region+Sex	1.245	2011.000
ENSG00000145743	0.429	0.067	0.233	-0.098	FBXL17	0.670	~p_tau+(1|manifest)+cngeneson+pc_mito+brain_region+Sex	0.829	2866.000
ENSG00000104529	0.128	0.067	0.233	-0.098	EEF1D	0.346	~p_tau+(1|manifest)+cngeneson+pc_mito+brain_region+Sex	0.551	3717.000
ENSG00000069667	0.648	0.067	0.211	-0.077	RORA	0.817	~p_tau+(1|manifest)+cngeneson+pc_mito+brain_region+Sex	1.719	1441.000
ENSG00000118690	0.102	0.067	0.135	-0.001	ARMC2	0.306	~p_tau+(1|manifest)+cngeneson+pc_mito+brain_region+Sex	0.330	4680.000
ENSG00000274211	0.008	0.067	0.111	0.023	SOCS7	0.079	~p_tau+(1|manifest)+cngeneson+pc_mito+brain_region+Sex	0.121	5434.000
ENSG00000100099	0.054	0.067	0.143	-0.009	HPS4	0.222	~p_tau+(1|manifest)+cngeneson+pc_mito+brain_region+Sex	0.269	4974.000
ENSG00000068912	0.001	0.067	0.120	0.014	ERLEC1	0.028	~p_tau+(1|manifest)+cngeneson+pc_mito+brain_region+Sex	0.145	5397.000
ENSG00000105176	0.680	0.067	0.216	-0.082	URI1	0.839	~p_tau+(1|manifest)+cngeneson+pc_mito+brain_region+Sex	1.278	1951.000
ENSG00000181826	0.357	0.067	0.162	-0.028	RELL1	0.612	~p_tau+(1|manifest)+cngeneson+pc_mito+brain_region+Sex	0.943	2564.000
ENSG00000123349	0.406	0.067	0.273	-0.139	PFDN5	0.652	~p_tau+(1|manifest)+cngeneson+pc_mito+brain_region+Sex	2.051	1214.000
ENSG00000131143	0.341	0.067	0.222	-0.088	COX4I1	0.597	~p_tau+(1|manifest)+cngeneson+pc_mito+brain_region+Sex	1.556	1609.000
ENSG00000113845	0.195	0.067	0.149	-0.015	TIMMDC1	0.437	~p_tau+(1|manifest)+cngeneson+pc_mito+brain_region+Sex	0.359	4529.000
ENSG00000180398	0.075	0.067	0.127	0.007	MCFD2	0.261	~p_tau+(1|manifest)+cngeneson+pc_mito+brain_region+Sex	0.116	5441.000
ENSG00000164308	0.264	0.067	0.182	-0.048	ERAP2	0.517	~p_tau+(1|manifest)+cngeneson+pc_mito+brain_region+Sex	0.667	3305.000
ENSG00000205629	0.018	0.067	0.114	0.020	LCMT1	0.124	~p_tau+(1|manifest)+cngeneson+pc_mito+brain_region+Sex	0.327	4697.000
ENSG00000170417	0.109	0.067	0.128	0.006	TMEM182	0.316	~p_tau+(1|manifest)+cngeneson+pc_mito+brain_region+Sex	0.293	4851.000
ENSG00000123607	0.183	0.067	0.142	-0.008	TTC21B	0.423	~p_tau+(1|manifest)+cngeneson+pc_mito+brain_region+Sex	0.312	4766.000
ENSG00000172795	0.135	0.067	0.178	-0.044	DCP2	0.356	~p_tau+(1|manifest)+cngeneson+pc_mito+brain_region+Sex	0.305	4802.000
ENSG00000159082	0.230	0.067	0.141	-0.008	SYNJ1	0.479	~p_tau+(1|manifest)+cngeneson+pc_mito+brain_region+Sex	0.648	3367.000
ENSG00000132824	0.017	0.066	0.124	0.009	SERINC3	0.118	~p_tau+(1|manifest)+cngeneson+pc_mito+brain_region+Sex	0.143	5400.000
ENSG00000154217	0.321	0.066	0.271	-0.139	PITPNC1	0.578	~p_tau+(1|manifest)+cngeneson+pc_mito+brain_region+Sex	1.882	1326.000
ENSG00000061273	0.208	0.066	0.175	-0.042	HDAC7	0.450	~p_tau+(1|manifest)+cngeneson+pc_mito+brain_region+Sex	0.179	5299.000
ENSG00000155903	0.918	0.066	0.384	-0.252	RASA2	0.961	~p_tau+(1|manifest)+cngeneson+pc_mito+brain_region+Sex	2.001	1247.000
ENSG00000134452	0.070	0.066	0.137	-0.005	FBH1	0.250	~p_tau+(1|manifest)+cngeneson+pc_mito+brain_region+Sex	0.337	4647.000
ENSG00000090686	0.273	0.066	0.148	-0.016	USP48	0.527	~p_tau+(1|manifest)+cngeneson+pc_mito+brain_region+Sex	0.262	5016.000
ENSG00000165813	0.126	0.066	0.142	-0.010	CCDC186	0.343	~p_tau+(1|manifest)+cngeneson+pc_mito+brain_region+Sex	0.333	4667.000
ENSG00000112242	0.891	0.066	0.335	-0.203	E2F3	0.950	~p_tau+(1|manifest)+cngeneson+pc_mito+brain_region+Sex	2.444	998.000
ENSG00000053770	0.015	0.066	0.171	-0.039	AP5M1	0.111	~p_tau+(1|manifest)+cngeneson+pc_mito+brain_region+Sex	0.619	3468.000
ENSG00000115919	0.796	0.066	0.291	-0.159	KYNU	0.900	~p_tau+(1|manifest)+cngeneson+pc_mito+brain_region+Sex	2.502	969.000
ENSG00000135966	0.025	0.066	0.135	-0.004	TGFBRAP1	0.147	~p_tau+(1|manifest)+cngeneson+pc_mito+brain_region+Sex	0.288	4882.000
ENSG00000079459	0.383	0.066	0.163	-0.031	FDFT1	0.632	~p_tau+(1|manifest)+cngeneson+pc_mito+brain_region+Sex	1.513	1649.000
ENSG00000152332	0.182	0.066	0.208	-0.077	UHMK1	0.421	~p_tau+(1|manifest)+cngeneson+pc_mito+brain_region+Sex	0.689	3235.000
ENSG00000125691	0.673	0.065	0.392	-0.261	RPL23	0.833	~p_tau+(1|manifest)+cngeneson+pc_mito+brain_region+Sex	5.189	342.000
ENSG00000089335	0.066	0.065	0.173	-0.042	ZNF302	0.244	~p_tau+(1|manifest)+cngeneson+pc_mito+brain_region+Sex	0.513	3870.000
ENSG00000149273	0.773	0.065	0.402	-0.272	RPS3	0.887	~p_tau+(1|manifest)+cngeneson+pc_mito+brain_region+Sex	2.963	782.000
ENSG00000196814	0.860	0.065	0.299	-0.168	MVB12B	0.937	~p_tau+(1|manifest)+cngeneson+pc_mito+brain_region+Sex	2.494	972.000
ENSG00000008988	0.892	0.065	0.448	-0.317	RPS20	0.950	~p_tau+(1|manifest)+cngeneson+pc_mito+brain_region+Sex	7.452	188.000
ENSG00000122912	0.047	0.065	0.126	0.004	SLC25A16	0.202	~p_tau+(1|manifest)+cngeneson+pc_mito+brain_region+Sex	0.360	4526.000
ENSG00000211455	0.468	0.065	0.245	-0.114	STK38L	0.699	~p_tau+(1|manifest)+cngeneson+pc_mito+brain_region+Sex	0.465	4081.000
ENSG00000077380	0.370	0.065	0.191	-0.060	DYNC1I2	0.623	~p_tau+(1|manifest)+cngeneson+pc_mito+brain_region+Sex	0.849	2793.000
ENSG00000071054	0.713	0.065	0.222	-0.092	MAP4K4	0.856	~p_tau+(1|manifest)+cngeneson+pc_mito+brain_region+Sex	2.631	917.000
ENSG00000145414	0.364	0.065	0.253	-0.123	NAF1	0.617	~p_tau+(1|manifest)+cngeneson+pc_mito+brain_region+Sex	2.126	1168.000
ENSG00000143226	0.603	0.065	0.289	-0.159	FCGR2A	0.788	~p_tau+(1|manifest)+cngeneson+pc_mito+brain_region+Sex	1.661	1487.000
ENSG00000155096	0.586	0.065	0.223	-0.093	AZIN1	0.779	~p_tau+(1|manifest)+cngeneson+pc_mito+brain_region+Sex	1.054	2342.000
ENSG00000168917	0.302	0.065	0.179	-0.049	SLC35G2	0.557	~p_tau+(1|manifest)+cngeneson+pc_mito+brain_region+Sex	1.894	1316.000
ENSG00000112146	0.048	0.065	0.144	-0.014	FBXO9	0.204	~p_tau+(1|manifest)+cngeneson+pc_mito+brain_region+Sex	0.427	4234.000
ENSG00000113595	0.059	0.065	0.122	0.008	TRIM23	0.230	~p_tau+(1|manifest)+cngeneson+pc_mito+brain_region+Sex	0.283	4914.000
ENSG00000134086	0.159	0.065	0.134	-0.005	VHL	0.388	~p_tau+(1|manifest)+cngeneson+pc_mito+brain_region+Sex	0.315	4748.000
ENSG00000146776	0.698	0.065	0.214	-0.085	ATXN7L1	0.848	~p_tau+(1|manifest)+cngeneson+pc_mito+brain_region+Sex	0.448	4151.000
ENSG00000164168	0.068	0.065	0.126	0.003	TMEM184C	0.247	~p_tau+(1|manifest)+cngeneson+pc_mito+brain_region+Sex	0.304	4807.000
ENSG00000143324	0.653	0.065	0.296	-0.166	XPR1	0.820	~p_tau+(1|manifest)+cngeneson+pc_mito+brain_region+Sex	0.833	2850.000
ENSG00000111817	0.269	0.065	0.249	-0.120	DSE	0.523	~p_tau+(1|manifest)+cngeneson+pc_mito+brain_region+Sex	0.435	4201.000
ENSG00000117139	0.285	0.065	0.149	-0.020	KDM5B	0.538	~p_tau+(1|manifest)+cngeneson+pc_mito+brain_region+Sex	0.629	3424.000
ENSG00000159917	0.079	0.065	0.125	0.004	ZNF235	0.267	~p_tau+(1|manifest)+cngeneson+pc_mito+brain_region+Sex	0.205	5222.000
ENSG00000103381	0.850	0.065	0.289	-0.160	CPPED1	0.931	~p_tau+(1|manifest)+cngeneson+pc_mito+brain_region+Sex	1.443	1734.000
ENSG00000119689	0.088	0.064	0.136	-0.008	DLST	0.283	~p_tau+(1|manifest)+cngeneson+pc_mito+brain_region+Sex	0.408	4325.000
ENSG00000118418	0.291	0.064	0.231	-0.103	HMGN3	0.543	~p_tau+(1|manifest)+cngeneson+pc_mito+brain_region+Sex	0.770	3009.000
ENSG00000156467	0.713	0.064	0.365	-0.237	UQCRB	0.856	~p_tau+(1|manifest)+cngeneson+pc_mito+brain_region+Sex	4.620	412.000
ENSG00000138942	0.032	0.064	0.122	0.006	RNF185	0.164	~p_tau+(1|manifest)+cngeneson+pc_mito+brain_region+Sex	0.357	4537.000
ENSG00000169100	0.093	0.064	0.192	-0.063	SLC25A6	0.290	~p_tau+(1|manifest)+cngeneson+pc_mito+brain_region+Sex	1.611	1543.000
ENSG00000185728	0.656	0.064	0.204	-0.076	YTHDF3	0.822	~p_tau+(1|manifest)+cngeneson+pc_mito+brain_region+Sex	0.899	2669.000
ENSG00000165494	0.753	0.064	0.245	-0.117	PCF11	0.875	~p_tau+(1|manifest)+cngeneson+pc_mito+brain_region+Sex	0.438	4187.000
ENSG00000066629	0.943	0.064	0.467	-0.339	EML1	0.973	~p_tau+(1|manifest)+cngeneson+pc_mito+brain_region+Sex	5.038	362.000
ENSG00000136861	0.344	0.064	0.166	-0.038	CDK5RAP2	0.598	~p_tau+(1|manifest)+cngeneson+pc_mito+brain_region+Sex	0.170	5331.000
ENSG00000166439	0.388	0.064	0.184	-0.056	RNF169	0.636	~p_tau+(1|manifest)+cngeneson+pc_mito+brain_region+Sex	0.355	4552.000
ENSG00000153879	0.055	0.064	0.129	-0.001	CEBPG	0.224	~p_tau+(1|manifest)+cngeneson+pc_mito+brain_region+Sex	0.272	4959.000
ENSG00000154429	0.480	0.064	0.171	-0.044	CCSAP	0.707	~p_tau+(1|manifest)+cngeneson+pc_mito+brain_region+Sex	0.935	2581.000
ENSG00000115808	0.530	0.064	0.186	-0.058	STRN	0.740	~p_tau+(1|manifest)+cngeneson+pc_mito+brain_region+Sex	1.158	2146.000
ENSG00000107372	0.067	0.064	0.167	-0.040	ZFAND5	0.245	~p_tau+(1|manifest)+cngeneson+pc_mito+brain_region+Sex	0.684	3252.000
ENSG00000196418	0.904	0.064	0.348	-0.221	ZNF124	0.955	~p_tau+(1|manifest)+cngeneson+pc_mito+brain_region+Sex	1.814	1382.000
ENSG00000067369	0.062	0.064	0.123	0.004	TP53BP1	0.238	~p_tau+(1|manifest)+cngeneson+pc_mito+brain_region+Sex	0.345	4607.000
ENSG00000184060	0.960	0.064	0.502	-0.374	ADAP2	0.980	~p_tau+(1|manifest)+cngeneson+pc_mito+brain_region+Sex	2.891	801.000
ENSG00000057019	0.064	0.064	0.148	-0.021	DCBLD2	0.242	~p_tau+(1|manifest)+cngeneson+pc_mito+brain_region+Sex	0.311	4769.000
ENSG00000100442	0.026	0.064	0.139	-0.012	FKBP3	0.148	~p_tau+(1|manifest)+cngeneson+pc_mito+brain_region+Sex	0.229	5144.000
ENSG00000173575	0.633	0.063	0.222	-0.095	CHD2	0.807	~p_tau+(1|manifest)+cngeneson+pc_mito+brain_region+Sex	0.599	3541.000
ENSG00000100523	0.463	0.063	0.165	-0.038	DDHD1	0.695	~p_tau+(1|manifest)+cngeneson+pc_mito+brain_region+Sex	0.263	5012.000
ENSG00000197969	0.304	0.063	0.198	-0.072	VPS13A	0.560	~p_tau+(1|manifest)+cngeneson+pc_mito+brain_region+Sex	0.821	2885.000
ENSG00000125875	0.035	0.063	0.120	0.005	TBC1D20	0.173	~p_tau+(1|manifest)+cngeneson+pc_mito+brain_region+Sex	0.306	4798.000
ENSG00000160087	0.127	0.063	0.130	-0.005	UBE2J2	0.345	~p_tau+(1|manifest)+cngeneson+pc_mito+brain_region+Sex	0.459	4105.000
ENSG00000135932	0.590	0.063	0.182	-0.056	CAB39	0.781	~p_tau+(1|manifest)+cngeneson+pc_mito+brain_region+Sex	1.342	1846.000
ENSG00000138286	0.185	0.063	0.129	-0.003	FAM149B1	0.425	~p_tau+(1|manifest)+cngeneson+pc_mito+brain_region+Sex	0.188	5270.000
ENSG00000152700	0.094	0.063	0.124	0.001	SAR1B	0.292	~p_tau+(1|manifest)+cngeneson+pc_mito+brain_region+Sex	0.468	4070.000
ENSG00000113716	0.023	0.063	0.131	-0.006	HMGXB3	0.141	~p_tau+(1|manifest)+cngeneson+pc_mito+brain_region+Sex	0.744	3076.000
ENSG00000163738	0.517	0.063	0.178	-0.053	MTHFD2L	0.732	~p_tau+(1|manifest)+cngeneson+pc_mito+brain_region+Sex	0.913	2643.000
ENSG00000108578	0.120	0.063	0.123	0.002	BLMH	0.336	~p_tau+(1|manifest)+cngeneson+pc_mito+brain_region+Sex	0.159	5363.000
ENSG00000169252	0.158	0.063	0.249	-0.124	ADRB2	0.387	~p_tau+(1|manifest)+cngeneson+pc_mito+brain_region+Sex	1.726	1435.000
ENSG00000156304	0.690	0.062	0.216	-0.091	SCAF4	0.843	~p_tau+(1|manifest)+cngeneson+pc_mito+brain_region+Sex	1.138	2184.000
ENSG00000266412	0.248	0.062	0.244	-0.119	NCOA4	0.500	~p_tau+(1|manifest)+cngeneson+pc_mito+brain_region+Sex	0.975	2486.000
ENSG00000149308	0.531	0.062	0.171	-0.047	NPAT	0.740	~p_tau+(1|manifest)+cngeneson+pc_mito+brain_region+Sex	0.769	3015.000
ENSG00000132466	0.739	0.062	0.217	-0.093	ANKRD17	0.868	~p_tau+(1|manifest)+cngeneson+pc_mito+brain_region+Sex	1.848	1353.000
ENSG00000117448	0.593	0.062	0.269	-0.145	AKR1A1	0.783	~p_tau+(1|manifest)+cngeneson+pc_mito+brain_region+Sex	1.938	1291.000
ENSG00000087365	0.166	0.062	0.149	-0.025	SF3B2	0.397	~p_tau+(1|manifest)+cngeneson+pc_mito+brain_region+Sex	0.231	5138.000
ENSG00000100106	0.105	0.062	0.186	-0.062	TRIOBP	0.311	~p_tau+(1|manifest)+cngeneson+pc_mito+brain_region+Sex	0.921	2615.000
ENSG00000147889	0.037	0.062	0.124	0.000	CDKN2A	0.180	~p_tau+(1|manifest)+cngeneson+pc_mito+brain_region+Sex	0.237	5125.000
ENSG00000144744	0.479	0.062	0.161	-0.037	UBA3	0.706	~p_tau+(1|manifest)+cngeneson+pc_mito+brain_region+Sex	0.448	4153.000
ENSG00000077684	0.017	0.062	0.134	-0.010	JADE1	0.121	~p_tau+(1|manifest)+cngeneson+pc_mito+brain_region+Sex	0.436	4193.000
ENSG00000100292	0.811	0.062	0.275	-0.152	HMOX1	0.909	~p_tau+(1|manifest)+cngeneson+pc_mito+brain_region+Sex	3.278	691.000
ENSG00000175390	0.234	0.062	0.158	-0.035	EIF3F	0.485	~p_tau+(1|manifest)+cngeneson+pc_mito+brain_region+Sex	0.484	4006.000
ENSG00000134308	0.107	0.062	0.117	0.006	YWHAQ	0.313	~p_tau+(1|manifest)+cngeneson+pc_mito+brain_region+Sex	0.238	5120.000
ENSG00000121691	0.120	0.062	0.134	-0.011	CAT	0.336	~p_tau+(1|manifest)+cngeneson+pc_mito+brain_region+Sex	0.526	3811.000
ENSG00000183431	0.076	0.062	0.128	-0.004	SF3A3	0.262	~p_tau+(1|manifest)+cngeneson+pc_mito+brain_region+Sex	0.344	4613.000
ENSG00000115762	0.104	0.062	0.157	-0.034	PLEKHB2	0.309	~p_tau+(1|manifest)+cngeneson+pc_mito+brain_region+Sex	0.874	2735.000
ENSG00000105866	0.546	0.061	0.190	-0.067	SP4	0.753	~p_tau+(1|manifest)+cngeneson+pc_mito+brain_region+Sex	0.605	3511.000
ENSG00000122435	0.338	0.061	0.209	-0.086	TRMT13	0.594	~p_tau+(1|manifest)+cngeneson+pc_mito+brain_region+Sex	1.122	2215.000
ENSG00000135829	0.605	0.061	0.185	-0.063	DHX9	0.789	~p_tau+(1|manifest)+cngeneson+pc_mito+brain_region+Sex	0.601	3532.000
ENSG00000196262	0.244	0.061	0.219	-0.098	PPIA	0.495	~p_tau+(1|manifest)+cngeneson+pc_mito+brain_region+Sex	1.328	1869.000
ENSG00000005339	0.683	0.061	0.225	-0.103	CREBBP	0.840	~p_tau+(1|manifest)+cngeneson+pc_mito+brain_region+Sex	0.615	3478.000
ENSG00000160058	0.073	0.061	0.170	-0.049	BSDC1	0.255	~p_tau+(1|manifest)+cngeneson+pc_mito+brain_region+Sex	0.734	3106.000
ENSG00000142197	0.831	0.061	0.259	-0.137	DOP1B	0.921	~p_tau+(1|manifest)+cngeneson+pc_mito+brain_region+Sex	2.007	1243.000
ENSG00000206527	0.324	0.061	0.148	-0.026	HACD2	0.582	~p_tau+(1|manifest)+cngeneson+pc_mito+brain_region+Sex	0.441	4172.000
ENSG00000168904	0.312	0.061	0.151	-0.030	LRRC28	0.568	~p_tau+(1|manifest)+cngeneson+pc_mito+brain_region+Sex	0.338	4638.000
ENSG00000135317	0.331	0.061	0.157	-0.036	SNX14	0.588	~p_tau+(1|manifest)+cngeneson+pc_mito+brain_region+Sex	0.265	4996.000
ENSG00000153561	0.004	0.061	0.103	0.018	RMND5A	0.059	~p_tau+(1|manifest)+cngeneson+pc_mito+brain_region+Sex	0.130	5422.000
ENSG00000101493	0.521	0.061	0.231	-0.110	ZNF516	0.735	~p_tau+(1|manifest)+cngeneson+pc_mito+brain_region+Sex	1.402	1768.000
ENSG00000119929	0.190	0.061	0.138	-0.016	CUTC	0.430	~p_tau+(1|manifest)+cngeneson+pc_mito+brain_region+Sex	0.464	4089.000
ENSG00000196954	0.086	0.060	0.300	-0.179	CASP4	0.279	~p_tau+(1|manifest)+cngeneson+pc_mito+brain_region+Sex	0.859	2774.000
ENSG00000204406	0.755	0.060	0.222	-0.101	MBD5	0.876	~p_tau+(1|manifest)+cngeneson+pc_mito+brain_region+Sex	0.602	3527.000
ENSG00000157350	0.438	0.060	0.153	-0.033	ST3GAL2	0.678	~p_tau+(1|manifest)+cngeneson+pc_mito+brain_region+Sex	0.423	4255.000
ENSG00000177082	0.111	0.060	0.120	0.000	WDR73	0.320	~p_tau+(1|manifest)+cngeneson+pc_mito+brain_region+Sex	0.355	4549.000
ENSG00000183549	0.314	0.060	0.278	-0.157	ACSM5	0.570	~p_tau+(1|manifest)+cngeneson+pc_mito+brain_region+Sex	2.715	884.000
ENSG00000171311	0.009	0.060	0.130	-0.009	EXOSC1	0.083	~p_tau+(1|manifest)+cngeneson+pc_mito+brain_region+Sex	0.256	5046.000
ENSG00000173406	0.876	0.060	0.312	-0.191	DAB1	0.943	~p_tau+(1|manifest)+cngeneson+pc_mito+brain_region+Sex	3.272	693.000
ENSG00000145332	0.715	0.060	0.240	-0.119	KLHL8	0.857	~p_tau+(1|manifest)+cngeneson+pc_mito+brain_region+Sex	0.808	2916.000
ENSG00000115970	0.599	0.060	0.256	-0.136	THADA	0.786	~p_tau+(1|manifest)+cngeneson+pc_mito+brain_region+Sex	1.493	1671.000
ENSG00000198837	0.358	0.060	0.195	-0.075	DENND4B	0.612	~p_tau+(1|manifest)+cngeneson+pc_mito+brain_region+Sex	1.277	1952.000
ENSG00000198825	0.186	0.060	0.175	-0.055	INPP5F	0.427	~p_tau+(1|manifest)+cngeneson+pc_mito+brain_region+Sex	0.427	4233.000
ENSG00000136682	0.237	0.060	0.287	-0.167	CBWD2	0.488	~p_tau+(1|manifest)+cngeneson+pc_mito+brain_region+Sex	1.647	1504.000
ENSG00000151176	0.031	0.060	0.115	0.005	PLBD2	0.164	~p_tau+(1|manifest)+cngeneson+pc_mito+brain_region+Sex	0.341	4621.000
ENSG00000196911	0.478	0.060	0.179	-0.059	KPNA5	0.705	~p_tau+(1|manifest)+cngeneson+pc_mito+brain_region+Sex	0.424	4248.000
ENSG00000139718	0.069	0.060	0.114	0.005	SETD1B	0.247	~p_tau+(1|manifest)+cngeneson+pc_mito+brain_region+Sex	0.408	4324.000
ENSG00000285082	0.325	0.060	0.205	-0.086	AL160272.2	0.582	~p_tau+(1|manifest)+cngeneson+pc_mito+brain_region+Sex	1.496	1669.000
ENSG00000163110	0.397	0.060	0.146	-0.026	PDLIM5	0.644	~p_tau+(1|manifest)+cngeneson+pc_mito+brain_region+Sex	0.583	3602.000
ENSG00000131196	0.392	0.060	0.151	-0.032	NFATC1	0.639	~p_tau+(1|manifest)+cngeneson+pc_mito+brain_region+Sex	0.478	4035.000
ENSG00000178719	0.301	0.060	0.167	-0.048	GRINA	0.556	~p_tau+(1|manifest)+cngeneson+pc_mito+brain_region+Sex	0.781	2985.000
ENSG00000213741	0.393	0.060	0.231	-0.112	RPS29	0.639	~p_tau+(1|manifest)+cngeneson+pc_mito+brain_region+Sex	1.259	1979.000
ENSG00000173905	0.366	0.059	0.165	-0.046	GOLIM4	0.618	~p_tau+(1|manifest)+cngeneson+pc_mito+brain_region+Sex	0.764	3029.000
ENSG00000165983	0.043	0.059	0.133	-0.014	PTER	0.195	~p_tau+(1|manifest)+cngeneson+pc_mito+brain_region+Sex	0.256	5049.000
ENSG00000124155	0.079	0.059	0.143	-0.024	PIGT	0.268	~p_tau+(1|manifest)+cngeneson+pc_mito+brain_region+Sex	0.515	3863.000
ENSG00000146083	0.033	0.059	0.157	-0.038	RNF44	0.169	~p_tau+(1|manifest)+cngeneson+pc_mito+brain_region+Sex	0.762	3033.000
ENSG00000188186	0.169	0.059	0.120	-0.002	LAMTOR4	0.401	~p_tau+(1|manifest)+cngeneson+pc_mito+brain_region+Sex	0.288	4878.000
ENSG00000005812	0.485	0.059	0.160	-0.042	FBXL3	0.711	~p_tau+(1|manifest)+cngeneson+pc_mito+brain_region+Sex	0.350	4583.000
ENSG00000085511	0.154	0.059	0.118	0.000	MAP3K4	0.381	~p_tau+(1|manifest)+cngeneson+pc_mito+brain_region+Sex	0.286	4894.000
ENSG00000157625	0.144	0.059	0.120	-0.003	TAB3	0.367	~p_tau+(1|manifest)+cngeneson+pc_mito+brain_region+Sex	0.316	4742.000
ENSG00000066027	0.121	0.059	0.119	-0.002	PPP2R5A	0.337	~p_tau+(1|manifest)+cngeneson+pc_mito+brain_region+Sex	0.347	4595.000
ENSG00000113558	0.011	0.059	0.102	0.016	SKP1	0.092	~p_tau+(1|manifest)+cngeneson+pc_mito+brain_region+Sex	0.161	5355.000
ENSG00000138439	0.791	0.059	0.244	-0.126	FAM117B	0.897	~p_tau+(1|manifest)+cngeneson+pc_mito+brain_region+Sex	1.241	2016.000
ENSG00000149532	0.142	0.059	0.150	-0.033	CPSF7	0.366	~p_tau+(1|manifest)+cngeneson+pc_mito+brain_region+Sex	0.760	3038.000
ENSG00000109572	0.248	0.059	0.163	-0.046	CLCN3	0.500	~p_tau+(1|manifest)+cngeneson+pc_mito+brain_region+Sex	0.249	5074.000
ENSG00000126226	0.168	0.059	0.159	-0.042	PCID2	0.401	~p_tau+(1|manifest)+cngeneson+pc_mito+brain_region+Sex	0.421	4262.000
ENSG00000141378	0.047	0.059	0.152	-0.035	PTRH2	0.203	~p_tau+(1|manifest)+cngeneson+pc_mito+brain_region+Sex	0.806	2920.000
ENSG00000106609	0.053	0.058	0.102	0.015	TMEM248	0.218	~p_tau+(1|manifest)+cngeneson+pc_mito+brain_region+Sex	0.256	5050.000
ENSG00000005483	0.406	0.058	0.162	-0.045	KMT2E	0.652	~p_tau+(1|manifest)+cngeneson+pc_mito+brain_region+Sex	0.959	2526.000
ENSG00000048052	0.964	0.058	0.513	-0.396	HDAC9	0.983	~p_tau+(1|manifest)+cngeneson+pc_mito+brain_region+Sex	7.485	187.000
ENSG00000010270	0.056	0.058	0.105	0.012	STARD3NL	0.225	~p_tau+(1|manifest)+cngeneson+pc_mito+brain_region+Sex	0.332	4670.000
ENSG00000152520	0.605	0.058	0.184	-0.068	PAN3	0.790	~p_tau+(1|manifest)+cngeneson+pc_mito+brain_region+Sex	1.569	1596.000
ENSG00000119685	0.409	0.058	0.214	-0.098	TTLL5	0.654	~p_tau+(1|manifest)+cngeneson+pc_mito+brain_region+Sex	1.211	2065.000
ENSG00000057935	0.482	0.058	0.157	-0.041	MTA3	0.708	~p_tau+(1|manifest)+cngeneson+pc_mito+brain_region+Sex	0.392	4393.000
ENSG00000198700	0.413	0.058	0.149	-0.033	IPO9	0.658	~p_tau+(1|manifest)+cngeneson+pc_mito+brain_region+Sex	0.414	4300.000
ENSG00000172890	0.368	0.058	0.137	-0.022	NADSYN1	0.621	~p_tau+(1|manifest)+cngeneson+pc_mito+brain_region+Sex	0.277	4936.000
ENSG00000152601	0.417	0.058	0.155	-0.039	MBNL1	0.660	~p_tau+(1|manifest)+cngeneson+pc_mito+brain_region+Sex	5.329	326.000
ENSG00000102858	0.335	0.058	0.142	-0.026	MGRN1	0.592	~p_tau+(1|manifest)+cngeneson+pc_mito+brain_region+Sex	0.458	4113.000
ENSG00000149196	0.495	0.058	0.193	-0.078	HIKESHI	0.717	~p_tau+(1|manifest)+cngeneson+pc_mito+brain_region+Sex	0.774	3002.000
ENSG00000143761	0.106	0.058	0.193	-0.077	ARF1	0.311	~p_tau+(1|manifest)+cngeneson+pc_mito+brain_region+Sex	0.959	2529.000
ENSG00000155508	0.005	0.058	0.090	0.025	CNOT8	0.067	~p_tau+(1|manifest)+cngeneson+pc_mito+brain_region+Sex	0.203	5225.000
ENSG00000148730	0.162	0.057	0.160	-0.045	EIF4EBP2	0.393	~p_tau+(1|manifest)+cngeneson+pc_mito+brain_region+Sex	0.160	5357.000
ENSG00000137831	0.438	0.057	0.150	-0.035	UACA	0.678	~p_tau+(1|manifest)+cngeneson+pc_mito+brain_region+Sex	0.522	3828.000
ENSG00000083828	0.289	0.057	0.135	-0.021	ZNF586	0.542	~p_tau+(1|manifest)+cngeneson+pc_mito+brain_region+Sex	0.873	2738.000
ENSG00000197024	0.172	0.057	0.123	-0.008	ZNF398	0.406	~p_tau+(1|manifest)+cngeneson+pc_mito+brain_region+Sex	0.175	5314.000
ENSG00000023516	0.177	0.057	0.156	-0.042	AKAP11	0.414	~p_tau+(1|manifest)+cngeneson+pc_mito+brain_region+Sex	0.281	4919.000
ENSG00000176148	0.335	0.057	0.139	-0.025	TCP11L1	0.592	~p_tau+(1|manifest)+cngeneson+pc_mito+brain_region+Sex	0.076	5463.000
ENSG00000120656	0.013	0.057	0.090	0.023	TAF12	0.104	~p_tau+(1|manifest)+cngeneson+pc_mito+brain_region+Sex	0.263	5007.000
ENSG00000213654	0.093	0.057	0.160	-0.046	GPSM3	0.289	~p_tau+(1|manifest)+cngeneson+pc_mito+brain_region+Sex	0.588	3579.000
ENSG00000151151	0.573	0.057	0.162	-0.048	IPMK	0.770	~p_tau+(1|manifest)+cngeneson+pc_mito+brain_region+Sex	0.485	3999.000
ENSG00000139842	0.598	0.057	0.183	-0.069	CUL4A	0.786	~p_tau+(1|manifest)+cngeneson+pc_mito+brain_region+Sex	0.961	2518.000
ENSG00000070413	0.283	0.057	0.140	-0.027	DGCR2	0.536	~p_tau+(1|manifest)+cngeneson+pc_mito+brain_region+Sex	0.266	4988.000
ENSG00000003509	0.055	0.056	0.104	0.009	NDUFAF7	0.222	~p_tau+(1|manifest)+cngeneson+pc_mito+brain_region+Sex	0.145	5396.000
ENSG00000008294	0.502	0.056	0.149	-0.036	SPAG9	0.722	~p_tau+(1|manifest)+cngeneson+pc_mito+brain_region+Sex	0.649	3365.000
ENSG00000122729	0.005	0.056	0.104	0.008	ACO1	0.064	~p_tau+(1|manifest)+cngeneson+pc_mito+brain_region+Sex	0.174	5318.000
ENSG00000196214	0.086	0.056	0.118	-0.006	ZNF766	0.278	~p_tau+(1|manifest)+cngeneson+pc_mito+brain_region+Sex	0.336	4654.000
ENSG00000138279	0.018	0.056	0.091	0.021	ANXA7	0.122	~p_tau+(1|manifest)+cngeneson+pc_mito+brain_region+Sex	0.287	4888.000
ENSG00000179331	0.471	0.056	0.336	-0.224	RAB39A	0.700	~p_tau+(1|manifest)+cngeneson+pc_mito+brain_region+Sex	2.294	1075.000
ENSG00000101158	0.037	0.056	0.150	-0.038	NELFCD	0.181	~p_tau+(1|manifest)+cngeneson+pc_mito+brain_region+Sex	0.715	3153.000
ENSG00000140391	0.177	0.056	0.133	-0.021	TSPAN3	0.414	~p_tau+(1|manifest)+cngeneson+pc_mito+brain_region+Sex	0.221	5172.000

ENSG00000154767	0.175	0.056	0.159	-0.046	XPC	0.411	~p_tau+(1|manifest)+cngeneson+pc_mito+brain_region+Sex	0.517	3852.000
ENSG00000139437	0.286	0.056	0.164	-0.052	TCHP	0.539	~p_tau+(1|manifest)+cngeneson+pc_mito+brain_region+Sex	0.978	2478.000
ENSG00000102081	0.346	0.056	0.153	-0.041	FMR1	0.600	~p_tau+(1|manifest)+cngeneson+pc_mito+brain_region+Sex	0.381	4437.000
ENSG00000116205	0.062	0.056	0.122	-0.010	TCEANC2	0.238	~p_tau+(1|manifest)+cngeneson+pc_mito+brain_region+Sex	0.345	4605.000
ENSG00000100425	0.097	0.056	0.124	-0.012	BRD1	0.296	~p_tau+(1|manifest)+cngeneson+pc_mito+brain_region+Sex	0.964	2516.000
ENSG00000164975	0.089	0.056	0.117	-0.006	SNAPC3	0.283	~p_tau+(1|manifest)+cngeneson+pc_mito+brain_region+Sex	0.374	4460.000
ENSG00000116906	0.092	0.056	0.124	-0.013	GNPAT	0.289	~p_tau+(1|manifest)+cngeneson+pc_mito+brain_region+Sex	0.312	4763.000
ENSG00000115827	0.185	0.056	0.159	-0.048	DCAF17	0.426	~p_tau+(1|manifest)+cngeneson+pc_mito+brain_region+Sex	0.439	4182.000
ENSG00000117906	0.113	0.056	0.138	-0.027	RCN2	0.323	~p_tau+(1|manifest)+cngeneson+pc_mito+brain_region+Sex	0.569	3651.000
ENSG00000091428	0.365	0.055	0.227	-0.116	RAPGEF4	0.618	~p_tau+(1|manifest)+cngeneson+pc_mito+brain_region+Sex	0.874	2730.000
ENSG00000152102	0.237	0.055	0.145	-0.034	FAM168B	0.487	~p_tau+(1|manifest)+cngeneson+pc_mito+brain_region+Sex	0.268	4977.000
ENSG00000100393	0.600	0.055	0.162	-0.051	EP300	0.787	~p_tau+(1|manifest)+cngeneson+pc_mito+brain_region+Sex	0.458	4109.000
ENSG00000198952	0.067	0.055	0.111	0.000	SMG5	0.245	~p_tau+(1|manifest)+cngeneson+pc_mito+brain_region+Sex	0.354	4557.000
ENSG00000138380	0.432	0.055	0.161	-0.051	CARF	0.673	~p_tau+(1|manifest)+cngeneson+pc_mito+brain_region+Sex	0.470	4060.000
ENSG00000142534	0.965	0.055	0.481	-0.371	RPS11	0.984	~p_tau+(1|manifest)+cngeneson+pc_mito+brain_region+Sex	8.135	155.000
ENSG00000033327	0.371	0.055	0.158	-0.048	GAB2	0.624	~p_tau+(1|manifest)+cngeneson+pc_mito+brain_region+Sex	1.448	1726.000
ENSG00000124784	0.017	0.055	0.144	-0.034	RIOK1	0.118	~p_tau+(1|manifest)+cngeneson+pc_mito+brain_region+Sex	0.796	2942.000
ENSG00000125458	0.470	0.055	0.312	-0.202	NT5C	0.700	~p_tau+(1|manifest)+cngeneson+pc_mito+brain_region+Sex	2.339	1049.000
ENSG00000134070	0.619	0.055	0.166	-0.056	IRAK2	0.798	~p_tau+(1|manifest)+cngeneson+pc_mito+brain_region+Sex	0.439	4181.000
ENSG00000107362	0.345	0.055	0.131	-0.021	ABHD17B	0.599	~p_tau+(1|manifest)+cngeneson+pc_mito+brain_region+Sex	0.402	4349.000
ENSG00000143393	0.026	0.055	0.122	-0.013	PI4KB	0.149	~p_tau+(1|manifest)+cngeneson+pc_mito+brain_region+Sex	0.413	4301.000
ENSG00000152234	0.037	0.055	0.118	-0.008	ATP5F1A	0.179	~p_tau+(1|manifest)+cngeneson+pc_mito+brain_region+Sex	0.728	3120.000
ENSG00000165533	0.100	0.055	0.110	-0.001	TTC8	0.302	~p_tau+(1|manifest)+cngeneson+pc_mito+brain_region+Sex	0.464	4085.000
ENSG00000115109	0.197	0.055	0.125	-0.016	EPB41L5	0.439	~p_tau+(1|manifest)+cngeneson+pc_mito+brain_region+Sex	0.285	4900.000
ENSG00000131725	0.232	0.055	0.116	-0.007	WDR44	0.481	~p_tau+(1|manifest)+cngeneson+pc_mito+brain_region+Sex	0.323	4718.000
ENSG00000136100	0.060	0.054	0.100	0.009	VPS36	0.235	~p_tau+(1|manifest)+cngeneson+pc_mito+brain_region+Sex	0.187	5275.000
ENSG00000166532	0.284	0.054	0.124	-0.015	RIMKLB	0.538	~p_tau+(1|manifest)+cngeneson+pc_mito+brain_region+Sex	0.666	3306.000
ENSG00000105851	0.775	0.054	0.225	-0.117	PIK3CG	0.888	~p_tau+(1|manifest)+cngeneson+pc_mito+brain_region+Sex	0.315	4745.000
ENSG00000159202	0.205	0.054	0.140	-0.031	UBE2Z	0.449	~p_tau+(1|manifest)+cngeneson+pc_mito+brain_region+Sex	0.353	4558.000
ENSG00000139645	0.027	0.054	0.109	0.000	ANKRD52	0.151	~p_tau+(1|manifest)+cngeneson+pc_mito+brain_region+Sex	0.238	5117.000
ENSG00000054116	0.029	0.054	0.098	0.011	TRAPPC3	0.157	~p_tau+(1|manifest)+cngeneson+pc_mito+brain_region+Sex	0.486	3997.000
ENSG00000074266	0.456	0.054	0.170	-0.062	EED	0.689	~p_tau+(1|manifest)+cngeneson+pc_mito+brain_region+Sex	0.625	3442.000
ENSG00000029639	0.558	0.054	0.152	-0.044	TFB1M	0.761	~p_tau+(1|manifest)+cngeneson+pc_mito+brain_region+Sex	0.180	5295.000
ENSG00000187187	0.009	0.054	0.129	-0.022	ZNF546	0.085	~p_tau+(1|manifest)+cngeneson+pc_mito+brain_region+Sex	0.288	4879.000
ENSG00000213676	0.044	0.054	0.109	-0.001	ATF6B	0.197	~p_tau+(1|manifest)+cngeneson+pc_mito+brain_region+Sex	0.393	4386.000
ENSG00000177058	0.523	0.054	0.201	-0.093	SLC38A9	0.736	~p_tau+(1|manifest)+cngeneson+pc_mito+brain_region+Sex	0.505	3905.000
ENSG00000046651	0.711	0.054	0.216	-0.109	OFD1	0.855	~p_tau+(1|manifest)+cngeneson+pc_mito+brain_region+Sex	1.321	1877.000
ENSG00000089902	0.793	0.054	0.238	-0.131	RCOR1	0.899	~p_tau+(1|manifest)+cngeneson+pc_mito+brain_region+Sex	1.845	1356.000
ENSG00000066422	0.275	0.054	0.142	-0.034	ZBTB11	0.529	~p_tau+(1|manifest)+cngeneson+pc_mito+brain_region+Sex	0.498	3939.000
ENSG00000132005	0.198	0.054	0.122	-0.015	RFX1	0.440	~p_tau+(1|manifest)+cngeneson+pc_mito+brain_region+Sex	0.167	5340.000
ENSG00000153147	0.089	0.053	0.151	-0.044	SMARCA5	0.284	~p_tau+(1|manifest)+cngeneson+pc_mito+brain_region+Sex	0.586	3588.000
ENSG00000172943	0.010	0.053	0.092	0.015	PHF8	0.089	~p_tau+(1|manifest)+cngeneson+pc_mito+brain_region+Sex	0.177	5303.000
ENSG00000154305	0.258	0.053	0.150	-0.043	MIA3	0.512	~p_tau+(1|manifest)+cngeneson+pc_mito+brain_region+Sex	0.190	5266.000
ENSG00000132471	0.117	0.053	0.135	-0.028	WBP2	0.332	~p_tau+(1|manifest)+cngeneson+pc_mito+brain_region+Sex	0.806	2921.000
ENSG00000185246	0.145	0.053	0.123	-0.016	PRPF39	0.370	~p_tau+(1|manifest)+cngeneson+pc_mito+brain_region+Sex	0.275	4947.000
ENSG00000131238	0.287	0.053	0.300	-0.194	PPT1	0.539	~p_tau+(1|manifest)+cngeneson+pc_mito+brain_region+Sex	1.105	2243.000
ENSG00000132694	0.706	0.053	0.181	-0.075	ARHGEF11	0.852	~p_tau+(1|manifest)+cngeneson+pc_mito+brain_region+Sex	0.349	4584.000
ENSG00000157837	0.628	0.053	0.179	-0.073	SPPL3	0.804	~p_tau+(1|manifest)+cngeneson+pc_mito+brain_region+Sex	1.089	2276.000
ENSG00000103852	0.297	0.053	0.144	-0.038	TTC23	0.552	~p_tau+(1|manifest)+cngeneson+pc_mito+brain_region+Sex	0.880	2713.000
ENSG00000065665	0.023	0.053	0.111	-0.005	SEC61A2	0.141	~p_tau+(1|manifest)+cngeneson+pc_mito+brain_region+Sex	0.147	5387.000
ENSG00000162368	0.108	0.053	0.126	-0.020	CMPK1	0.315	~p_tau+(1|manifest)+cngeneson+pc_mito+brain_region+Sex	0.541	3757.000
ENSG00000152492	0.961	0.053	0.436	-0.330	CCDC50	0.981	~p_tau+(1|manifest)+cngeneson+pc_mito+brain_region+Sex	2.941	788.000
ENSG00000178567	0.067	0.053	0.176	-0.070	EPM2AIP1	0.245	~p_tau+(1|manifest)+cngeneson+pc_mito+brain_region+Sex	0.391	4395.000
ENSG00000066336	0.724	0.053	0.206	-0.100	SPI1	0.862	~p_tau+(1|manifest)+cngeneson+pc_mito+brain_region+Sex	1.292	1926.000
ENSG00000197594	0.780	0.053	0.236	-0.131	ENPP1	0.891	~p_tau+(1|manifest)+cngeneson+pc_mito+brain_region+Sex	1.239	2020.000
ENSG00000011258	0.802	0.053	0.221	-0.115	MBTD1	0.904	~p_tau+(1|manifest)+cngeneson+pc_mito+brain_region+Sex	1.047	2356.000
ENSG00000185298	0.117	0.053	0.120	-0.014	CCDC137	0.332	~p_tau+(1|manifest)+cngeneson+pc_mito+brain_region+Sex	0.313	4758.000
ENSG00000170430	0.857	0.053	0.316	-0.210	MGMT	0.935	~p_tau+(1|manifest)+cngeneson+pc_mito+brain_region+Sex	1.679	1469.000
ENSG00000060237	0.565	0.053	0.254	-0.149	WNK1	0.765	~p_tau+(1|manifest)+cngeneson+pc_mito+brain_region+Sex	2.682	897.000
ENSG00000145782	0.400	0.053	0.129	-0.024	ATG12	0.646	~p_tau+(1|manifest)+cngeneson+pc_mito+brain_region+Sex	0.162	5352.000
ENSG00000159461	0.024	0.053	0.127	-0.022	AMFR	0.142	~p_tau+(1|manifest)+cngeneson+pc_mito+brain_region+Sex	0.310	4775.000
ENSG00000095380	0.277	0.052	0.142	-0.037	NANS	0.531	~p_tau+(1|manifest)+cngeneson+pc_mito+brain_region+Sex	0.930	2587.000
ENSG00000116871	0.124	0.052	0.107	-0.003	MAP7D1	0.340	~p_tau+(1|manifest)+cngeneson+pc_mito+brain_region+Sex	0.342	4620.000
ENSG00000119705	0.011	0.052	0.164	-0.060	SLIRP	0.094	~p_tau+(1|manifest)+cngeneson+pc_mito+brain_region+Sex	0.769	3011.000
ENSG00000101146	0.176	0.052	0.111	-0.007	RAE1	0.413	~p_tau+(1|manifest)+cngeneson+pc_mito+brain_region+Sex	0.149	5383.000
ENSG00000184574	0.871	0.052	0.312	-0.208	LPAR5	0.941	~p_tau+(1|manifest)+cngeneson+pc_mito+brain_region+Sex	1.921	1299.000
ENSG00000157259	0.150	0.052	0.202	-0.097	GATAD1	0.377	~p_tau+(1|manifest)+cngeneson+pc_mito+brain_region+Sex	1.008	2418.000
ENSG00000111615	0.036	0.052	0.115	-0.011	KRR1	0.178	~p_tau+(1|manifest)+cngeneson+pc_mito+brain_region+Sex	0.237	5123.000
ENSG00000157827	0.449	0.052	0.174	-0.069	FMNL2	0.685	~p_tau+(1|manifest)+cngeneson+pc_mito+brain_region+Sex	4.552	425.000
ENSG00000117868	0.655	0.052	0.171	-0.067	ESYT2	0.822	~p_tau+(1|manifest)+cngeneson+pc_mito+brain_region+Sex	0.436	4194.000
ENSG00000273734	0.281	0.052	0.208	-0.104	AC005258.1	0.534	~p_tau+(1|manifest)+cngeneson+pc_mito+brain_region+Sex	1.280	1947.000
ENSG00000125952	0.853	0.052	0.237	-0.132	MAX	0.932	~p_tau+(1|manifest)+cngeneson+pc_mito+brain_region+Sex	1.122	2217.000
ENSG00000198146	0.073	0.052	0.132	-0.028	ZNF770	0.254	~p_tau+(1|manifest)+cngeneson+pc_mito+brain_region+Sex	0.470	4061.000
ENSG00000083844	0.152	0.052	0.105	-0.001	ZNF264	0.380	~p_tau+(1|manifest)+cngeneson+pc_mito+brain_region+Sex	0.402	4346.000
ENSG00000128245	0.316	0.052	0.226	-0.122	YWHAH	0.572	~p_tau+(1|manifest)+cngeneson+pc_mito+brain_region+Sex	1.650	1499.000
ENSG00000112304	0.008	0.052	0.137	-0.033	ACOT13	0.082	~p_tau+(1|manifest)+cngeneson+pc_mito+brain_region+Sex	0.215	5192.000
ENSG00000110429	0.077	0.052	0.102	0.001	FBXO3	0.264	~p_tau+(1|manifest)+cngeneson+pc_mito+brain_region+Sex	0.215	5193.000
ENSG00000102054	0.048	0.052	0.106	-0.003	RBBP7	0.204	~p_tau+(1|manifest)+cngeneson+pc_mito+brain_region+Sex	0.261	5018.000
ENSG00000124788	0.489	0.052	0.328	-0.224	ATXN1	0.713	~p_tau+(1|manifest)+cngeneson+pc_mito+brain_region+Sex	5.898	288.000
ENSG00000135698	0.383	0.052	0.175	-0.072	MPHOSPH6	0.632	~p_tau+(1|manifest)+cngeneson+pc_mito+brain_region+Sex	0.842	2820.000
ENSG00000165280	0.058	0.052	0.129	-0.026	VCP	0.228	~p_tau+(1|manifest)+cngeneson+pc_mito+brain_region+Sex	0.265	4997.000
ENSG00000008952	0.503	0.052	0.137	-0.034	SEC62	0.724	~p_tau+(1|manifest)+cngeneson+pc_mito+brain_region+Sex	0.195	5246.000
ENSG00000105483	0.675	0.051	0.193	-0.090	CARD8	0.834	~p_tau+(1|manifest)+cngeneson+pc_mito+brain_region+Sex	1.233	2030.000
ENSG00000072736	0.513	0.051	0.137	-0.034	NFATC3	0.730	~p_tau+(1|manifest)+cngeneson+pc_mito+brain_region+Sex	0.355	4546.000
ENSG00000174231	0.205	0.051	0.147	-0.045	PRPF8	0.449	~p_tau+(1|manifest)+cngeneson+pc_mito+brain_region+Sex	0.558	3689.000
ENSG00000133800	0.261	0.051	0.394	-0.291	LYVE1	0.515	~p_tau+(1|manifest)+cngeneson+pc_mito+brain_region+Sex	7.268	197.000
ENSG00000128294	0.155	0.051	0.126	-0.023	TPST2	0.383	~p_tau+(1|manifest)+cngeneson+pc_mito+brain_region+Sex	0.223	5165.000
ENSG00000017797	0.088	0.051	0.105	-0.002	RALBP1	0.282	~p_tau+(1|manifest)+cngeneson+pc_mito+brain_region+Sex	0.257	5041.000
ENSG00000144224	0.438	0.051	0.133	-0.030	UBXN4	0.678	~p_tau+(1|manifest)+cngeneson+pc_mito+brain_region+Sex	0.257	5039.000
ENSG00000105221	0.046	0.051	0.107	-0.005	AKT2	0.201	~p_tau+(1|manifest)+cngeneson+pc_mito+brain_region+Sex	0.217	5183.000
ENSG00000135930	0.028	0.051	0.112	-0.010	EIF4E2	0.153	~p_tau+(1|manifest)+cngeneson+pc_mito+brain_region+Sex	0.390	4397.000
ENSG00000243156	0.372	0.051	0.124	-0.022	MICAL3	0.625	~p_tau+(1|manifest)+cngeneson+pc_mito+brain_region+Sex	0.354	4553.000
ENSG00000154274	0.819	0.051	0.290	-0.189	C4orf19	0.915	~p_tau+(1|manifest)+cngeneson+pc_mito+brain_region+Sex	1.852	1349.000
ENSG00000180626	0.096	0.051	0.126	-0.025	ZNF594	0.295	~p_tau+(1|manifest)+cngeneson+pc_mito+brain_region+Sex	0.259	5030.000
ENSG00000109775	0.017	0.051	0.097	0.005	UFSP2	0.122	~p_tau+(1|manifest)+cngeneson+pc_mito+brain_region+Sex	0.216	5184.000
ENSG00000154814	0.236	0.051	0.115	-0.013	OXNAD1	0.487	~p_tau+(1|manifest)+cngeneson+pc_mito+brain_region+Sex	0.212	5200.000
ENSG00000105854	0.561	0.051	0.176	-0.075	PON2	0.763	~p_tau+(1|manifest)+cngeneson+pc_mito+brain_region+Sex	0.738	3087.000
ENSG00000108423	0.159	0.051	0.121	-0.020	TUBD1	0.389	~p_tau+(1|manifest)+cngeneson+pc_mito+brain_region+Sex	0.446	4159.000
ENSG00000134324	0.032	0.051	0.100	0.002	LPIN1	0.164	~p_tau+(1|manifest)+cngeneson+pc_mito+brain_region+Sex	0.278	4933.000
ENSG00000137509	0.612	0.050	0.151	-0.051	PRCP	0.793	~p_tau+(1|manifest)+cngeneson+pc_mito+brain_region+Sex	0.472	4053.000
ENSG00000139679	0.964	0.050	0.436	-0.335	LPAR6	0.983	~p_tau+(1|manifest)+cngeneson+pc_mito+brain_region+Sex	4.585	419.000
ENSG00000137497	0.829	0.050	0.213	-0.112	NUMA1	0.920	~p_tau+(1|manifest)+cngeneson+pc_mito+brain_region+Sex	1.542	1615.000
ENSG00000243317	0.220	0.050	0.151	-0.050	STMP1	0.466	~p_tau+(1|manifest)+cngeneson+pc_mito+brain_region+Sex	0.484	4002.000
ENSG00000115165	0.514	0.050	0.217	-0.117	CYTIP	0.731	~p_tau+(1|manifest)+cngeneson+pc_mito+brain_region+Sex	0.544	3748.000
ENSG00000118680	0.031	0.050	0.109	-0.009	MYL12B	0.162	~p_tau+(1|manifest)+cngeneson+pc_mito+brain_region+Sex	0.321	4723.000
ENSG00000101457	0.022	0.050	0.107	-0.007	DNTTIP1	0.138	~p_tau+(1|manifest)+cngeneson+pc_mito+brain_region+Sex	0.295	4835.000
ENSG00000122085	0.532	0.050	0.140	-0.040	MTERF4	0.741	~p_tau+(1|manifest)+cngeneson+pc_mito+brain_region+Sex	0.538	3773.000
ENSG00000081014	0.619	0.050	0.149	-0.050	AP4E1	0.798	~p_tau+(1|manifest)+cngeneson+pc_mito+brain_region+Sex	0.190	5263.000
ENSG00000117697	0.752	0.050	0.200	-0.100	NSL1	0.875	~p_tau+(1|manifest)+cngeneson+pc_mito+brain_region+Sex	0.491	3981.000
ENSG00000097033	0.650	0.050	0.201	-0.102	SH3GLB1	0.819	~p_tau+(1|manifest)+cngeneson+pc_mito+brain_region+Sex	0.850	2791.000
ENSG00000148450	0.083	0.050	0.104	-0.004	MSRB2	0.276	~p_tau+(1|manifest)+cngeneson+pc_mito+brain_region+Sex	0.259	5033.000
ENSG00000174106	0.825	0.050	0.212	-0.113	LEMD3	0.918	~p_tau+(1|manifest)+cngeneson+pc_mito+brain_region+Sex	0.689	3240.000
ENSG00000148690	0.433	0.050	0.122	-0.023	FRA10AC1	0.674	~p_tau+(1|manifest)+cngeneson+pc_mito+brain_region+Sex	0.340	4631.000
ENSG00000188677	0.687	0.049	0.297	-0.198	PARVB	0.841	~p_tau+(1|manifest)+cngeneson+pc_mito+brain_region+Sex	2.473	979.000
ENSG00000123575	0.270	0.049	0.126	-0.027	FAM199X	0.524	~p_tau+(1|manifest)+cngeneson+pc_mito+brain_region+Sex	0.508	3892.000
ENSG00000143155	0.070	0.049	0.105	-0.006	TIPRL	0.250	~p_tau+(1|manifest)+cngeneson+pc_mito+brain_region+Sex	0.177	5305.000
ENSG00000196683	0.266	0.049	0.198	-0.100	TOMM7	0.520	~p_tau+(1|manifest)+cngeneson+pc_mito+brain_region+Sex	2.230	1114.000
ENSG00000126698	0.057	0.049	0.097	0.001	DNAJC8	0.227	~p_tau+(1|manifest)+cngeneson+pc_mito+brain_region+Sex	0.309	4780.000
ENSG00000132589	0.750	0.049	0.356	-0.257	FLOT2	0.875	~p_tau+(1|manifest)+cngeneson+pc_mito+brain_region+Sex	1.451	1717.000
ENSG00000084093	0.342	0.049	0.120	-0.022	REST	0.597	~p_tau+(1|manifest)+cngeneson+pc_mito+brain_region+Sex	0.306	4792.000
ENSG00000116030	0.072	0.049	0.094	0.004	SUMO1	0.254	~p_tau+(1|manifest)+cngeneson+pc_mito+brain_region+Sex	0.291	4859.000
ENSG00000126214	0.321	0.049	0.158	-0.060	KLC1	0.578	~p_tau+(1|manifest)+cngeneson+pc_mito+brain_region+Sex	1.000	2437.000
ENSG00000178209	0.812	0.049	0.348	-0.250	PLEC	0.909	~p_tau+(1|manifest)+cngeneson+pc_mito+brain_region+Sex	3.651	611.000
ENSG00000179583	0.927	0.049	0.340	-0.242	CIITA	0.966	~p_tau+(1|manifest)+cngeneson+pc_mito+brain_region+Sex	2.969	780.000
ENSG00000054523	0.548	0.049	0.187	-0.090	KIF1B	0.755	~p_tau+(1|manifest)+cngeneson+pc_mito+brain_region+Sex	0.562	3676.000
ENSG00000122483	0.491	0.049	0.137	-0.039	CCDC18	0.714	~p_tau+(1|manifest)+cngeneson+pc_mito+brain_region+Sex	0.736	3097.000
ENSG00000065029	0.264	0.049	0.142	-0.045	ZNF76	0.517	~p_tau+(1|manifest)+cngeneson+pc_mito+brain_region+Sex	0.681	3264.000
ENSG00000147548	0.449	0.049	0.124	-0.027	NSD3	0.685	~p_tau+(1|manifest)+cngeneson+pc_mito+brain_region+Sex	0.334	4663.000
ENSG00000114999	0.458	0.049	0.141	-0.044	TTL	0.691	~p_tau+(1|manifest)+cngeneson+pc_mito+brain_region+Sex	0.364	4502.000
ENSG00000168438	0.290	0.049	0.136	-0.038	CDC40	0.543	~p_tau+(1|manifest)+cngeneson+pc_mito+brain_region+Sex	0.153	5374.000
ENSG00000172809	0.693	0.049	0.321	-0.223	RPL38	0.845	~p_tau+(1|manifest)+cngeneson+pc_mito+brain_region+Sex	4.338	465.000
ENSG00000163510	0.180	0.049	0.123	-0.026	CWC22	0.418	~p_tau+(1|manifest)+cngeneson+pc_mito+brain_region+Sex	0.266	4986.000
ENSG00000072134	0.822	0.048	0.201	-0.104	EPN2	0.917	~p_tau+(1|manifest)+cngeneson+pc_mito+brain_region+Sex	0.861	2766.000
ENSG00000132463	0.143	0.048	0.100	-0.003	GRSF1	0.367	~p_tau+(1|manifest)+cngeneson+pc_mito+brain_region+Sex	0.348	4590.000
ENSG00000114573	0.264	0.048	0.133	-0.036	ATP6V1A	0.517	~p_tau+(1|manifest)+cngeneson+pc_mito+brain_region+Sex	0.489	3988.000
ENSG00000160908	0.031	0.048	0.113	-0.016	ZNF394	0.161	~p_tau+(1|manifest)+cngeneson+pc_mito+brain_region+Sex	0.159	5365.000
ENSG00000265808	0.450	0.048	0.149	-0.053	SEC22B	0.685	~p_tau+(1|manifest)+cngeneson+pc_mito+brain_region+Sex	0.556	3695.000
ENSG00000173846	0.441	0.048	0.153	-0.056	PLK3	0.680	~p_tau+(1|manifest)+cngeneson+pc_mito+brain_region+Sex	1.257	1981.000
ENSG00000132950	0.133	0.048	0.101	-0.005	ZMYM5	0.354	~p_tau+(1|manifest)+cngeneson+pc_mito+brain_region+Sex	0.084	5458.000
ENSG00000066827	0.212	0.048	0.162	-0.066	ZFAT	0.455	~p_tau+(1|manifest)+cngeneson+pc_mito+brain_region+Sex	0.180	5293.000
ENSG00000143815	0.622	0.048	0.149	-0.052	LBR	0.800	~p_tau+(1|manifest)+cngeneson+pc_mito+brain_region+Sex	0.297	4829.000
ENSG00000166037	0.217	0.048	0.105	-0.009	CEP57	0.461	~p_tau+(1|manifest)+cngeneson+pc_mito+brain_region+Sex	0.217	5179.000
ENSG00000116406	0.669	0.048	0.246	-0.150	EDEM3	0.831	~p_tau+(1|manifest)+cngeneson+pc_mito+brain_region+Sex	0.973	2491.000
ENSG00000151348	0.394	0.048	0.133	-0.037	EXT2	0.640	~p_tau+(1|manifest)+cngeneson+pc_mito+brain_region+Sex	0.420	4266.000
ENSG00000100225	0.232	0.048	0.119	-0.023	FBXO7	0.481	~p_tau+(1|manifest)+cngeneson+pc_mito+brain_region+Sex	0.276	4945.000
ENSG00000072401	0.380	0.048	0.127	-0.031	UBE2D1	0.630	~p_tau+(1|manifest)+cngeneson+pc_mito+brain_region+Sex	0.350	4581.000
ENSG00000162928	0.345	0.048	0.111	-0.015	PEX13	0.599	~p_tau+(1|manifest)+cngeneson+pc_mito+brain_region+Sex	0.113	5442.000
ENSG00000197586	0.189	0.048	0.097	-0.002	ENTPD6	0.429	~p_tau+(1|manifest)+cngeneson+pc_mito+brain_region+Sex	0.222	5169.000
ENSG00000124228	0.089	0.048	0.096	-0.001	DDX27	0.284	~p_tau+(1|manifest)+cngeneson+pc_mito+brain_region+Sex	0.134	5417.000
ENSG00000196843	0.787	0.048	0.189	-0.094	ARID5A	0.895	~p_tau+(1|manifest)+cngeneson+pc_mito+brain_region+Sex	0.400	4359.000
ENSG00000176142	0.866	0.047	0.283	-0.188	TMEM39A	0.939	~p_tau+(1|manifest)+cngeneson+pc_mito+brain_region+Sex	3.294	687.000
ENSG00000159433	0.209	0.047	0.108	-0.013	STARD9	0.452	~p_tau+(1|manifest)+cngeneson+pc_mito+brain_region+Sex	0.362	4516.000
ENSG00000068308	0.055	0.047	0.122	-0.028	OTUD5	0.224	~p_tau+(1|manifest)+cngeneson+pc_mito+brain_region+Sex	0.522	3829.000
ENSG00000255872	0.159	0.047	0.118	-0.023	AL138752.2	0.389	~p_tau+(1|manifest)+cngeneson+pc_mito+brain_region+Sex	0.506	3898.000
ENSG00000108055	0.077	0.047	0.104	-0.009	SMC3	0.264	~p_tau+(1|manifest)+cngeneson+pc_mito+brain_region+Sex	0.188	5273.000
ENSG00000119335	0.287	0.047	0.151	-0.057	SET	0.540	~p_tau+(1|manifest)+cngeneson+pc_mito+brain_region+Sex	1.111	2235.000
ENSG00000183044	0.741	0.047	0.167	-0.073	ABAT	0.869	~p_tau+(1|manifest)+cngeneson+pc_mito+brain_region+Sex	0.882	2709.000
ENSG00000166734	0.782	0.047	0.184	-0.091	CASC4	0.891	~p_tau+(1|manifest)+cngeneson+pc_mito+brain_region+Sex	0.638	3396.000
ENSG00000101126	0.665	0.047	0.147	-0.054	ADNP	0.828	~p_tau+(1|manifest)+cngeneson+pc_mito+brain_region+Sex	0.202	5227.000
ENSG00000161638	0.245	0.047	0.188	-0.094	ITGA5	0.497	~p_tau+(1|manifest)+cngeneson+pc_mito+brain_region+Sex	0.288	4881.000
ENSG00000143093	0.041	0.047	0.088	0.005	STRIP1	0.191	~p_tau+(1|manifest)+cngeneson+pc_mito+brain_region+Sex	0.335	4658.000
ENSG00000122557	0.503	0.047	0.153	-0.059	HERPUD2	0.724	~p_tau+(1|manifest)+cngeneson+pc_mito+brain_region+Sex	0.870	2740.000
ENSG00000110367	0.544	0.047	0.129	-0.035	DDX6	0.751	~p_tau+(1|manifest)+cngeneson+pc_mito+brain_region+Sex	0.621	3462.000
ENSG00000114487	0.808	0.047	0.190	-0.097	MORC1	0.908	~p_tau+(1|manifest)+cngeneson+pc_mito+brain_region+Sex	1.118	2224.000
ENSG00000072609	0.234	0.047	0.166	-0.073	CHFR	0.484	~p_tau+(1|manifest)+cngeneson+pc_mito+brain_region+Sex	0.918	2625.000
ENSG00000089876	0.100	0.047	0.118	-0.025	DHX32	0.302	~p_tau+(1|manifest)+cngeneson+pc_mito+brain_region+Sex	0.334	4661.000
ENSG00000124333	0.003	0.046	0.076	0.017	VAMP7	0.050	~p_tau+(1|manifest)+cngeneson+pc_mito+brain_region+Sex	0.176	5310.000
ENSG00000111328	0.257	0.046	0.124	-0.031	CDK2AP1	0.511	~p_tau+(1|manifest)+cngeneson+pc_mito+brain_region+Sex	0.397	4372.000
ENSG00000036054	0.071	0.046	0.104	-0.011	TBC1D23	0.250	~p_tau+(1|manifest)+cngeneson+pc_mito+brain_region+Sex	0.323	4716.000
ENSG00000139684	0.047	0.046	0.139	-0.046	ESD	0.202	~p_tau+(1|manifest)+cngeneson+pc_mito+brain_region+Sex	0.488	3990.000
ENSG00000143919	0.527	0.046	0.312	-0.219	CAMKMT	0.738	~p_tau+(1|manifest)+cngeneson+pc_mito+brain_region+Sex	1.728	1433.000
ENSG00000101191	0.271	0.046	0.115	-0.022	DIDO1	0.525	~p_tau+(1|manifest)+cngeneson+pc_mito+brain_region+Sex	0.400	4358.000
ENSG00000119912	0.068	0.046	0.163	-0.071	IDE	0.246	~p_tau+(1|manifest)+cngeneson+pc_mito+brain_region+Sex	0.596	3553.000
ENSG00000134996	0.567	0.046	0.133	-0.041	OSTF1	0.766	~p_tau+(1|manifest)+cngeneson+pc_mito+brain_region+Sex	0.984	2464.000
ENSG00000136273	0.038	0.046	0.083	0.009	HUS1	0.182	~p_tau+(1|manifest)+cngeneson+pc_mito+brain_region+Sex	0.101	5451.000
ENSG00000163950	0.178	0.046	0.146	-0.053	SLBP	0.415	~p_tau+(1|manifest)+cngeneson+pc_mito+brain_region+Sex	0.534	3784.000
ENSG00000160075	0.186	0.046	0.115	-0.023	SSU72	0.427	~p_tau+(1|manifest)+cngeneson+pc_mito+brain_region+Sex	0.643	3381.000
ENSG00000141068	0.722	0.046	0.176	-0.084	KSR1	0.861	~p_tau+(1|manifest)+cngeneson+pc_mito+brain_region+Sex	1.375	1796.000
ENSG00000067596	0.012	0.046	0.104	-0.012	DHX8	0.098	~p_tau+(1|manifest)+cngeneson+pc_mito+brain_region+Sex	0.279	4927.000
ENSG00000144909	0.890	0.046	0.332	-0.240	OSBPL11	0.950	~p_tau+(1|manifest)+cngeneson+pc_mito+brain_region+Sex	1.859	1344.000
ENSG00000136478	0.162	0.046	0.137	-0.046	TEX2	0.393	~p_tau+(1|manifest)+cngeneson+pc_mito+brain_region+Sex	0.497	3946.000
ENSG00000107829	0.601	0.046	0.156	-0.065	FBXW4	0.787	~p_tau+(1|manifest)+cngeneson+pc_mito+brain_region+Sex	0.209	5212.000
ENSG00000107223	0.243	0.046	0.143	-0.052	EDF1	0.494	~p_tau+(1|manifest)+cngeneson+pc_mito+brain_region+Sex	0.881	2712.000
ENSG00000139726	0.229	0.046	0.117	-0.026	DENR	0.478	~p_tau+(1|manifest)+cngeneson+pc_mito+brain_region+Sex	0.429	4219.000
ENSG00000134627	0.561	0.046	0.184	-0.093	PIWIL4	0.763	~p_tau+(1|manifest)+cngeneson+pc_mito+brain_region+Sex	0.791	2956.000
ENSG00000070831	0.599	0.046	0.163	-0.072	CDC42	0.786	~p_tau+(1|manifest)+cngeneson+pc_mito+brain_region+Sex	0.805	2923.000
ENSG00000112234	0.428	0.045	0.180	-0.089	FBXL4	0.669	~p_tau+(1|manifest)+cngeneson+pc_mito+brain_region+Sex	0.423	4250.000
ENSG00000101639	0.737	0.045	0.159	-0.069	CEP192	0.867	~p_tau+(1|manifest)+cngeneson+pc_mito+brain_region+Sex	1.004	2424.000
ENSG00000264324	0.268	0.045	0.157	-0.066	AC006030.1	0.522	~p_tau+(1|manifest)+cngeneson+pc_mito+brain_region+Sex	2.591	930.000
ENSG00000184939	0.136	0.045	0.132	-0.041	ZFP90	0.358	~p_tau+(1|manifest)+cngeneson+pc_mito+brain_region+Sex	0.385	4419.000
ENSG00000104412	0.413	0.045	0.127	-0.036	EMC2	0.657	~p_tau+(1|manifest)+cngeneson+pc_mito+brain_region+Sex	0.628	3430.000
ENSG00000121390	0.295	0.045	0.171	-0.080	PSPC1	0.549	~p_tau+(1|manifest)+cngeneson+pc_mito+brain_region+Sex	0.311	4772.000
ENSG00000159692	0.140	0.045	0.122	-0.032	CTBP1	0.363	~p_tau+(1|manifest)+cngeneson+pc_mito+brain_region+Sex	0.528	3799.000
ENSG00000119396	0.531	0.045	0.240	-0.150	RAB14	0.740	~p_tau+(1|manifest)+cngeneson+pc_mito+brain_region+Sex	0.814	2903.000
ENSG00000188786	0.099	0.045	0.111	-0.021	MTF1	0.299	~p_tau+(1|manifest)+cngeneson+pc_mito+brain_region+Sex	0.362	4519.000
ENSG00000196116	0.867	0.045	0.216	-0.126	TDRD7	0.939	~p_tau+(1|manifest)+cngeneson+pc_mito+brain_region+Sex	0.894	2684.000
ENSG00000174799	0.278	0.045	0.109	-0.018	CEP135	0.531	~p_tau+(1|manifest)+cngeneson+pc_mito+brain_region+Sex	0.184	5285.000
ENSG00000109171	0.755	0.045	0.166	-0.076	SLAIN2	0.876	~p_tau+(1|manifest)+cngeneson+pc_mito+brain_region+Sex	0.562	3675.000
ENSG00000135972	0.208	0.045	0.110	-0.020	MRPS9	0.451	~p_tau+(1|manifest)+cngeneson+pc_mito+brain_region+Sex	0.251	5069.000
ENSG00000124795	0.736	0.045	0.238	-0.148	DEK	0.867	~p_tau+(1|manifest)+cngeneson+pc_mito+brain_region+Sex	1.497	1668.000
ENSG00000188316	0.075	0.045	0.152	-0.062	ENO4	0.259	~p_tau+(1|manifest)+cngeneson+pc_mito+brain_region+Sex	1.519	1640.000
ENSG00000174600	0.957	0.045	0.353	-0.263	CMKLR1	0.978	~p_tau+(1|manifest)+cngeneson+pc_mito+brain_region+Sex	2.316	1060.000
ENSG00000053524	0.553	0.045	0.384	-0.295	MCF2L2	0.759	~p_tau+(1|manifest)+cngeneson+pc_mito+brain_region+Sex	3.693	594.000
ENSG00000126882	0.583	0.045	0.163	-0.073	FAM78A	0.777	~p_tau+(1|manifest)+cngeneson+pc_mito+brain_region+Sex	1.065	2317.000
ENSG00000115841	0.353	0.045	0.120	-0.030	RMDN2	0.608	~p_tau+(1|manifest)+cngeneson+pc_mito+brain_region+Sex	0.274	4955.000
ENSG00000186298	0.427	0.045	0.139	-0.050	PPP1CC	0.669	~p_tau+(1|manifest)+cngeneson+pc_mito+brain_region+Sex	0.560	3684.000
ENSG00000093167	0.312	0.045	0.150	-0.061	LRRFIP2	0.567	~p_tau+(1|manifest)+cngeneson+pc_mito+brain_region+Sex	0.789	2958.000
ENSG00000079332	0.102	0.045	0.099	-0.010	SAR1A	0.305	~p_tau+(1|manifest)+cngeneson+pc_mito+brain_region+Sex	0.372	4467.000
ENSG00000171365	0.867	0.045	0.280	-0.191	CLCN5	0.939	~p_tau+(1|manifest)+cngeneson+pc_mito+brain_region+Sex	0.977	2482.000
ENSG00000057252	0.901	0.045	0.248	-0.159	SOAT1	0.953	~p_tau+(1|manifest)+cngeneson+pc_mito+brain_region+Sex	0.991	2453.000
ENSG00000126070	0.529	0.045	0.180	-0.091	AGO3	0.740	~p_tau+(1|manifest)+cngeneson+pc_mito+brain_region+Sex	0.832	2854.000
ENSG00000258472	0.084	0.045	0.090	-0.001	AC005726.1	0.277	~p_tau+(1|manifest)+cngeneson+pc_mito+brain_region+Sex	0.175	5313.000
ENSG00000180917	0.686	0.044	0.193	-0.105	CMTR2	0.841	~p_tau+(1|manifest)+cngeneson+pc_mito+brain_region+Sex	1.380	1791.000
ENSG00000155256	0.011	0.044	0.084	0.005	ZFYVE27	0.094	~p_tau+(1|manifest)+cngeneson+pc_mito+brain_region+Sex	0.231	5136.000
ENSG00000096063	0.196	0.044	0.116	-0.027	SRPK1	0.438	~p_tau+(1|manifest)+cngeneson+pc_mito+brain_region+Sex	0.299	4819.000
ENSG00000117533	0.017	0.044	0.092	-0.003	VAMP4	0.118	~p_tau+(1|manifest)+cngeneson+pc_mito+brain_region+Sex	0.517	3850.000
ENSG00000149357	0.357	0.044	0.113	-0.024	LAMTOR1	0.611	~p_tau+(1|manifest)+cngeneson+pc_mito+brain_region+Sex	0.336	4650.000
ENSG00000106028	0.068	0.044	0.189	-0.100	SSBP1	0.246	~p_tau+(1|manifest)+cngeneson+pc_mito+brain_region+Sex	0.455	4123.000
ENSG00000106780	0.455	0.044	0.168	-0.080	MEGF9	0.689	~p_tau+(1|manifest)+cngeneson+pc_mito+brain_region+Sex	0.613	3484.000
ENSG00000197958	0.342	0.044	0.338	-0.250	RPL12	0.597	~p_tau+(1|manifest)+cngeneson+pc_mito+brain_region+Sex	2.781	852.000
ENSG00000068903	0.139	0.044	0.096	-0.008	SIRT2	0.362	~p_tau+(1|manifest)+cngeneson+pc_mito+brain_region+Sex	0.253	5059.000
ENSG00000068724	0.945	0.044	0.450	-0.362	TTC7A	0.973	~p_tau+(1|manifest)+cngeneson+pc_mito+brain_region+Sex	10.089	105.000
ENSG00000166938	0.561	0.044	0.140	-0.052	DIS3L	0.763	~p_tau+(1|manifest)+cngeneson+pc_mito+brain_region+Sex	0.457	4116.000
ENSG00000173418	0.517	0.044	0.203	-0.115	NAA20	0.732	~p_tau+(1|manifest)+cngeneson+pc_mito+brain_region+Sex	0.645	3374.000
ENSG00000114650	0.497	0.044	0.139	-0.051	SCAP	0.720	~p_tau+(1|manifest)+cngeneson+pc_mito+brain_region+Sex	0.741	3080.000
ENSG00000166200	0.328	0.044	0.126	-0.038	COPS2	0.586	~p_tau+(1|manifest)+cngeneson+pc_mito+brain_region+Sex	0.360	4524.000
ENSG00000065526	0.876	0.044	0.222	-0.134	SPEN	0.943	~p_tau+(1|manifest)+cngeneson+pc_mito+brain_region+Sex	0.858	2775.000
ENSG00000127980	0.086	0.044	0.111	-0.023	PEX1	0.278	~p_tau+(1|manifest)+cngeneson+pc_mito+brain_region+Sex	0.249	5076.000
ENSG00000027697	0.962	0.044	0.355	-0.267	IFNGR1	0.981	~p_tau+(1|manifest)+cngeneson+pc_mito+brain_region+Sex	6.120	273.000
ENSG00000151135	0.092	0.044	0.090	-0.002	TMEM263	0.289	~p_tau+(1|manifest)+cngeneson+pc_mito+brain_region+Sex	0.350	4582.000
ENSG00000125814	0.131	0.044	0.085	0.003	NAPB	0.350	~p_tau+(1|manifest)+cngeneson+pc_mito+brain_region+Sex	0.396	4378.000
ENSG00000169592	0.090	0.044	0.127	-0.039	INO80E	0.285	~p_tau+(1|manifest)+cngeneson+pc_mito+brain_region+Sex	0.652	3356.000
ENSG00000196576	0.431	0.044	0.197	-0.109	PLXNB2	0.672	~p_tau+(1|manifest)+cngeneson+pc_mito+brain_region+Sex	0.625	3446.000
ENSG00000111785	0.446	0.044	0.157	-0.070	RIC8B	0.683	~p_tau+(1|manifest)+cngeneson+pc_mito+brain_region+Sex	0.274	4951.000
ENSG00000008086	0.529	0.044	0.122	-0.035	CDKL5	0.740	~p_tau+(1|manifest)+cngeneson+pc_mito+brain_region+Sex	0.308	4787.000
ENSG00000148834	0.311	0.044	0.128	-0.040	GSTO1	0.567	~p_tau+(1|manifest)+cngeneson+pc_mito+brain_region+Sex	0.692	3226.000
ENSG00000166226	0.091	0.044	0.132	-0.045	CCT2	0.288	~p_tau+(1|manifest)+cngeneson+pc_mito+brain_region+Sex	0.500	3930.000
ENSG00000276293	0.020	0.043	0.127	-0.040	PIP4K2B	0.129	~p_tau+(1|manifest)+cngeneson+pc_mito+brain_region+Sex	0.384	4426.000
ENSG00000145685	0.825	0.043	0.180	-0.093	LHFPL2	0.918	~p_tau+(1|manifest)+cngeneson+pc_mito+brain_region+Sex	1.882	1325.000
ENSG00000156976	0.452	0.043	0.227	-0.140	EIF4A2	0.687	~p_tau+(1|manifest)+cngeneson+pc_mito+brain_region+Sex	1.666	1480.000
ENSG00000115966	0.601	0.043	0.165	-0.078	ATF2	0.787	~p_tau+(1|manifest)+cngeneson+pc_mito+brain_region+Sex	0.794	2949.000
ENSG00000009844	0.223	0.043	0.191	-0.105	VTA1	0.469	~p_tau+(1|manifest)+cngeneson+pc_mito+brain_region+Sex	0.465	4082.000
ENSG00000104064	0.154	0.043	0.122	-0.036	GABPB1	0.382	~p_tau+(1|manifest)+cngeneson+pc_mito+brain_region+Sex	0.399	4365.000
ENSG00000141510	0.138	0.043	0.111	-0.025	TP53	0.361	~p_tau+(1|manifest)+cngeneson+pc_mito+brain_region+Sex	0.453	4130.000
ENSG00000142599	0.726	0.043	0.172	-0.086	RERE	0.863	~p_tau+(1|manifest)+cngeneson+pc_mito+brain_region+Sex	2.188	1137.000
ENSG00000100749	0.377	0.043	0.112	-0.026	VRK1	0.628	~p_tau+(1|manifest)+cngeneson+pc_mito+brain_region+Sex	0.142	5403.000
ENSG00000170266	0.648	0.043	0.134	-0.048	GLB1	0.817	~p_tau+(1|manifest)+cngeneson+pc_mito+brain_region+Sex	0.481	4023.000
ENSG00000275052	0.466	0.043	0.125	-0.039	PPP4R3B	0.697	~p_tau+(1|manifest)+cngeneson+pc_mito+brain_region+Sex	0.457	4115.000
ENSG00000165914	0.956	0.043	0.340	-0.254	TTC7B	0.978	~p_tau+(1|manifest)+cngeneson+pc_mito+brain_region+Sex	4.471	441.000
ENSG00000089048	0.928	0.043	0.282	-0.196	ESF1	0.967	~p_tau+(1|manifest)+cngeneson+pc_mito+brain_region+Sex	1.854	1347.000
ENSG00000198677	0.837	0.043	0.228	-0.143	TTC37	0.924	~p_tau+(1|manifest)+cngeneson+pc_mito+brain_region+Sex	0.774	3003.000
ENSG00000123091	0.248	0.043	0.097	-0.011	RNF11	0.500	~p_tau+(1|manifest)+cngeneson+pc_mito+brain_region+Sex	0.153	5378.000
ENSG00000279170	0.211	0.043	0.166	-0.080	TSTD3	0.454	~p_tau+(1|manifest)+cngeneson+pc_mito+brain_region+Sex	0.455	4125.000
ENSG00000104853	0.241	0.043	0.103	-0.017	CLPTM1	0.491	~p_tau+(1|manifest)+cngeneson+pc_mito+brain_region+Sex	0.261	5021.000
ENSG00000116514	0.754	0.043	0.170	-0.085	RNF19B	0.876	~p_tau+(1|manifest)+cngeneson+pc_mito+brain_region+Sex	0.646	3372.000
ENSG00000170275	0.071	0.042	0.083	0.002	CRTAP	0.251	~p_tau+(1|manifest)+cngeneson+pc_mito+brain_region+Sex	0.210	5207.000
ENSG00000175215	0.447	0.042	0.165	-0.080	CTDSP2	0.683	~p_tau+(1|manifest)+cngeneson+pc_mito+brain_region+Sex	0.121	5435.000
ENSG00000110888	0.405	0.042	0.121	-0.037	CAPRIN2	0.651	~p_tau+(1|manifest)+cngeneson+pc_mito+brain_region+Sex	0.331	4675.000
ENSG00000151131	0.383	0.042	0.137	-0.052	C12orf45	0.632	~p_tau+(1|manifest)+cngeneson+pc_mito+brain_region+Sex	0.831	2860.000
ENSG00000175048	0.842	0.042	0.418	-0.334	ZDHHC14	0.925	~p_tau+(1|manifest)+cngeneson+pc_mito+brain_region+Sex	2.764	862.000
ENSG00000158711	0.298	0.042	0.179	-0.095	ELK4	0.552	~p_tau+(1|manifest)+cngeneson+pc_mito+brain_region+Sex	0.672	3290.000
ENSG00000080822	0.007	0.042	0.101	-0.017	CLDND1	0.074	~p_tau+(1|manifest)+cngeneson+pc_mito+brain_region+Sex	0.263	5011.000
ENSG00000149313	0.210	0.042	0.105	-0.021	AASDHPPT	0.453	~p_tau+(1|manifest)+cngeneson+pc_mito+brain_region+Sex	0.374	4461.000
ENSG00000163694	0.468	0.042	0.138	-0.054	RBM47	0.699	~p_tau+(1|manifest)+cngeneson+pc_mito+brain_region+Sex	1.307	1898.000
ENSG00000085871	0.900	0.042	0.221	-0.138	MGST2	0.953	~p_tau+(1|manifest)+cngeneson+pc_mito+brain_region+Sex	0.735	3100.000
ENSG00000001167	0.515	0.042	0.117	-0.033	NFYA	0.732	~p_tau+(1|manifest)+cngeneson+pc_mito+brain_region+Sex	0.490	3983.000
ENSG00000151065	0.598	0.042	0.123	-0.039	DCP1B	0.786	~p_tau+(1|manifest)+cngeneson+pc_mito+brain_region+Sex	0.452	4134.000
ENSG00000144713	0.978	0.042	0.452	-0.369	RPL32	0.990	~p_tau+(1|manifest)+cngeneson+pc_mito+brain_region+Sex	3.717	586.000
ENSG00000133302	0.009	0.041	0.068	0.015	SLF1	0.084	~p_tau+(1|manifest)+cngeneson+pc_mito+brain_region+Sex	0.147	5390.000
ENSG00000117151	0.124	0.041	0.129	-0.046	CTBS	0.340	~p_tau+(1|manifest)+cngeneson+pc_mito+brain_region+Sex	0.370	4474.000
ENSG00000111707	0.144	0.041	0.093	-0.010	SUDS3	0.367	~p_tau+(1|manifest)+cngeneson+pc_mito+brain_region+Sex	0.096	5453.000
ENSG00000144747	0.379	0.041	0.125	-0.042	TMF1	0.629	~p_tau+(1|manifest)+cngeneson+pc_mito+brain_region+Sex	0.493	3969.000
ENSG00000186532	0.073	0.041	0.137	-0.054	SMYD4	0.255	~p_tau+(1|manifest)+cngeneson+pc_mito+brain_region+Sex	0.370	4473.000
ENSG00000162604	0.331	0.041	0.154	-0.072	TM2D1	0.588	~p_tau+(1|manifest)+cngeneson+pc_mito+brain_region+Sex	0.494	3957.000
ENSG00000100426	0.558	0.041	0.121	-0.039	ZBED4	0.761	~p_tau+(1|manifest)+cngeneson+pc_mito+brain_region+Sex	0.702	3200.000
ENSG00000123144	0.138	0.041	0.116	-0.034	TRIR	0.360	~p_tau+(1|manifest)+cngeneson+pc_mito+brain_region+Sex	0.674	3285.000
ENSG00000100567	0.079	0.041	0.103	-0.021	PSMA3	0.267	~p_tau+(1|manifest)+cngeneson+pc_mito+brain_region+Sex	0.482	4016.000
ENSG00000127334	0.468	0.041	0.142	-0.059	DYRK2	0.699	~p_tau+(1|manifest)+cngeneson+pc_mito+brain_region+Sex	0.925	2605.000
ENSG00000178761	0.019	0.041	0.138	-0.056	FAM219B	0.126	~p_tau+(1|manifest)+cngeneson+pc_mito+brain_region+Sex	0.286	4891.000
ENSG00000172159	0.697	0.041	0.212	-0.130	FRMD3	0.846	~p_tau+(1|manifest)+cngeneson+pc_mito+brain_region+Sex	2.227	1115.000
ENSG00000221909	0.125	0.041	0.128	-0.046	FAM200A	0.342	~p_tau+(1|manifest)+cngeneson+pc_mito+brain_region+Sex	0.860	2772.000
ENSG00000128604	0.549	0.041	0.240	-0.158	IRF5	0.755	~p_tau+(1|manifest)+cngeneson+pc_mito+brain_region+Sex	2.504	968.000
ENSG00000148200	0.524	0.041	0.271	-0.190	NR6A1	0.737	~p_tau+(1|manifest)+cngeneson+pc_mito+brain_region+Sex	1.660	1488.000
ENSG00000135341	0.610	0.041	0.143	-0.062	MAP3K7	0.793	~p_tau+(1|manifest)+cngeneson+pc_mito+brain_region+Sex	0.362	4518.000
ENSG00000125826	0.188	0.041	0.122	-0.040	RBCK1	0.428	~p_tau+(1|manifest)+cngeneson+pc_mito+brain_region+Sex	0.414	4298.000
ENSG00000077514	0.307	0.041	0.096	-0.014	POLD3	0.563	~p_tau+(1|manifest)+cngeneson+pc_mito+brain_region+Sex	0.161	5354.000
ENSG00000198689	0.015	0.041	0.080	0.002	SLC9A6	0.112	~p_tau+(1|manifest)+cngeneson+pc_mito+brain_region+Sex	0.208	5218.000
ENSG00000178607	0.216	0.041	0.088	-0.006	ERN1	0.461	~p_tau+(1|manifest)+cngeneson+pc_mito+brain_region+Sex	0.264	5005.000
ENSG00000160584	0.361	0.040	0.174	-0.093	SIK3	0.615	~p_tau+(1|manifest)+cngeneson+pc_mito+brain_region+Sex	0.475	4040.000
ENSG00000182400	0.093	0.040	0.082	-0.001	TRAPPC6B	0.290	~p_tau+(1|manifest)+cngeneson+pc_mito+brain_region+Sex	0.135	5415.000
ENSG00000187446	0.129	0.040	0.090	-0.010	CHP1	0.347	~p_tau+(1|manifest)+cngeneson+pc_mito+brain_region+Sex	0.253	5061.000
ENSG00000138660	0.096	0.040	0.099	-0.018	AP1AR	0.295	~p_tau+(1|manifest)+cngeneson+pc_mito+brain_region+Sex	0.447	4156.000
ENSG00000170142	0.332	0.040	0.198	-0.117	UBE2E1	0.588	~p_tau+(1|manifest)+cngeneson+pc_mito+brain_region+Sex	0.352	4571.000
ENSG00000173726	0.187	0.040	0.099	-0.019	TOMM20	0.427	~p_tau+(1|manifest)+cngeneson+pc_mito+brain_region+Sex	0.364	4505.000
ENSG00000238083	0.747	0.040	0.394	-0.313	LRRC37A2	0.873	~p_tau+(1|manifest)+cngeneson+pc_mito+brain_region+Sex	5.002	366.000
ENSG00000145354	0.201	0.040	0.115	-0.035	CISD2	0.444	~p_tau+(1|manifest)+cngeneson+pc_mito+brain_region+Sex	0.487	3991.000
ENSG00000159685	0.358	0.040	0.115	-0.035	CHCHD6	0.612	~p_tau+(1|manifest)+cngeneson+pc_mito+brain_region+Sex	0.355	4550.000
ENSG00000159459	0.773	0.040	0.154	-0.073	UBR1	0.887	~p_tau+(1|manifest)+cngeneson+pc_mito+brain_region+Sex	0.388	4407.000
ENSG00000159352	0.225	0.040	0.093	-0.013	PSMD4	0.472	~p_tau+(1|manifest)+cngeneson+pc_mito+brain_region+Sex	0.353	4562.000
ENSG00000128512	0.182	0.040	0.135	-0.055	DOCK4	0.421	~p_tau+(1|manifest)+cngeneson+pc_mito+brain_region+Sex	4.164	497.000
ENSG00000115364	0.039	0.040	0.103	-0.023	MRPL19	0.185	~p_tau+(1|manifest)+cngeneson+pc_mito+brain_region+Sex	0.428	4230.000
ENSG00000103479	0.709	0.040	0.146	-0.066	RBL2	0.854	~p_tau+(1|manifest)+cngeneson+pc_mito+brain_region+Sex	0.517	3854.000
ENSG00000196357	0.036	0.040	0.153	-0.074	ZNF565	0.177	~p_tau+(1|manifest)+cngeneson+pc_mito+brain_region+Sex	1.187	2100.000
ENSG00000166164	0.258	0.040	0.120	-0.040	BRD7	0.512	~p_tau+(1|manifest)+cngeneson+pc_mito+brain_region+Sex	0.640	3390.000
ENSG00000105821	0.591	0.040	0.126	-0.046	DNAJC2	0.782	~p_tau+(1|manifest)+cngeneson+pc_mito+brain_region+Sex	0.503	3915.000
ENSG00000135241	0.703	0.040	0.136	-0.056	PNPLA8	0.850	~p_tau+(1|manifest)+cngeneson+pc_mito+brain_region+Sex	0.392	4391.000
ENSG00000129197	0.063	0.040	0.113	-0.034	RPAIN	0.240	~p_tau+(1|manifest)+cngeneson+pc_mito+brain_region+Sex	0.381	4439.000
ENSG00000139597	0.752	0.040	0.263	-0.183	N4BP2L1	0.875	~p_tau+(1|manifest)+cngeneson+pc_mito+brain_region+Sex	1.849	1352.000
ENSG00000155008	0.080	0.040	0.098	-0.019	APOOL	0.270	~p_tau+(1|manifest)+cngeneson+pc_mito+brain_region+Sex	0.309	4783.000
ENSG00000198818	0.088	0.040	0.084	-0.005	SFT2D1	0.282	~p_tau+(1|manifest)+cngeneson+pc_mito+brain_region+Sex	0.270	4973.000
ENSG00000037749	0.567	0.040	0.118	-0.039	MFAP3	0.766	~p_tau+(1|manifest)+cngeneson+pc_mito+brain_region+Sex	0.385	4416.000
ENSG00000141646	0.207	0.039	0.105	-0.026	SMAD4	0.450	~p_tau+(1|manifest)+cngeneson+pc_mito+brain_region+Sex	0.401	4356.000
ENSG00000239521	0.427	0.039	0.117	-0.038	CASTOR3	0.669	~p_tau+(1|manifest)+cngeneson+pc_mito+brain_region+Sex	0.307	4790.000
ENSG00000011523	0.162	0.039	0.119	-0.040	CEP68	0.393	~p_tau+(1|manifest)+cngeneson+pc_mito+brain_region+Sex	0.611	3490.000
ENSG00000175792	0.252	0.039	0.142	-0.064	RUVBL1	0.504	~p_tau+(1|manifest)+cngeneson+pc_mito+brain_region+Sex	0.699	3208.000
ENSG00000106615	0.560	0.039	0.129	-0.050	RHEB	0.763	~p_tau+(1|manifest)+cngeneson+pc_mito+brain_region+Sex	0.394	4384.000
ENSG00000138175	0.254	0.039	0.093	-0.014	ARL3	0.507	~p_tau+(1|manifest)+cngeneson+pc_mito+brain_region+Sex	0.190	5264.000
ENSG00000150907	0.709	0.039	0.267	-0.189	FOXO1	0.854	~p_tau+(1|manifest)+cngeneson+pc_mito+brain_region+Sex	1.634	1518.000
ENSG00000140044	0.951	0.039	0.299	-0.221	JDP2	0.976	~p_tau+(1|manifest)+cngeneson+pc_mito+brain_region+Sex	1.626	1527.000
ENSG00000266472	0.142	0.039	0.123	-0.045	MRPS21	0.366	~p_tau+(1|manifest)+cngeneson+pc_mito+brain_region+Sex	0.691	3231.000
ENSG00000136560	0.916	0.039	0.278	-0.200	TANK	0.960	~p_tau+(1|manifest)+cngeneson+pc_mito+brain_region+Sex	1.681	1467.000
ENSG00000147364	0.180	0.039	0.100	-0.022	FBXO25	0.419	~p_tau+(1|manifest)+cngeneson+pc_mito+brain_region+Sex	0.341	4627.000
ENSG00000122042	0.571	0.039	0.321	-0.244	UBL3	0.769	~p_tau+(1|manifest)+cngeneson+pc_mito+brain_region+Sex	1.996	1250.000
ENSG00000142669	0.444	0.039	0.314	-0.236	SH3BGRL3	0.682	~p_tau+(1|manifest)+cngeneson+pc_mito+brain_region+Sex	4.903	380.000
ENSG00000115956	0.900	0.039	0.227	-0.150	PLEK	0.953	~p_tau+(1|manifest)+cngeneson+pc_mito+brain_region+Sex	0.251	5067.000
ENSG00000091527	0.581	0.039	0.153	-0.075	CDV3	0.776	~p_tau+(1|manifest)+cngeneson+pc_mito+brain_region+Sex	1.058	2333.000
ENSG00000108582	0.044	0.039	0.095	-0.018	CPD	0.196	~p_tau+(1|manifest)+cngeneson+pc_mito+brain_region+Sex	0.092	5454.000
ENSG00000186566	0.183	0.039	0.183	-0.106	GPATCH8	0.423	~p_tau+(1|manifest)+cngeneson+pc_mito+brain_region+Sex	0.505	3909.000
ENSG00000168522	0.133	0.038	0.073	0.004	FNTA	0.354	~p_tau+(1|manifest)+cngeneson+pc_mito+brain_region+Sex	0.116	5440.000
ENSG00000169020	0.842	0.038	0.215	-0.139	ATP5ME	0.926	~p_tau+(1|manifest)+cngeneson+pc_mito+brain_region+Sex	2.312	1062.000
ENSG00000120798	0.501	0.038	0.108	-0.031	NR2C1	0.722	~p_tau+(1|manifest)+cngeneson+pc_mito+brain_region+Sex	0.584	3600.000
ENSG00000112339	0.399	0.038	0.145	-0.068	HBS1L	0.645	~p_tau+(1|manifest)+cngeneson+pc_mito+brain_region+Sex	0.402	4351.000
ENSG00000106052	0.924	0.038	0.226	-0.150	TAX1BP1	0.965	~p_tau+(1|manifest)+cngeneson+pc_mito+brain_region+Sex	1.402	1769.000
ENSG00000129187	0.282	0.038	0.093	-0.016	DCTD	0.535	~p_tau+(1|manifest)+cngeneson+pc_mito+brain_region+Sex	0.485	4001.000
ENSG00000122359	0.738	0.038	0.145	-0.069	ANXA11	0.868	~p_tau+(1|manifest)+cngeneson+pc_mito+brain_region+Sex	0.651	3359.000
ENSG00000166889	0.125	0.038	0.093	-0.017	PATL1	0.342	~p_tau+(1|manifest)+cngeneson+pc_mito+brain_region+Sex	0.449	4148.000
ENSG00000148219	0.558	0.038	0.109	-0.033	ASTN2	0.761	~p_tau+(1|manifest)+cngeneson+pc_mito+brain_region+Sex	0.282	4916.000
ENSG00000145388	0.143	0.038	0.085	-0.009	METTL14	0.366	~p_tau+(1|manifest)+cngeneson+pc_mito+brain_region+Sex	0.140	5406.000
ENSG00000067533	0.510	0.038	0.132	-0.056	RRP15	0.729	~p_tau+(1|manifest)+cngeneson+pc_mito+brain_region+Sex	0.804	2926.000
ENSG00000124177	0.832	0.038	0.257	-0.181	CHD6	0.921	~p_tau+(1|manifest)+cngeneson+pc_mito+brain_region+Sex	1.383	1788.000
ENSG00000084090	0.453	0.038	0.115	-0.040	STARD7	0.688	~p_tau+(1|manifest)+cngeneson+pc_mito+brain_region+Sex	0.443	4167.000
ENSG00000125482	0.579	0.038	0.114	-0.038	TTF1	0.775	~p_tau+(1|manifest)+cngeneson+pc_mito+brain_region+Sex	0.354	4554.000
ENSG00000071553	0.162	0.038	0.130	-0.055	ATP6AP1	0.393	~p_tau+(1|manifest)+cngeneson+pc_mito+brain_region+Sex	1.104	2244.000
ENSG00000115756	0.821	0.038	0.157	-0.081	HPCAL1	0.916	~p_tau+(1|manifest)+cngeneson+pc_mito+brain_region+Sex	0.380	4441.000
ENSG00000139971	0.455	0.038	0.202	-0.127	ARMH4	0.689	~p_tau+(1|manifest)+cngeneson+pc_mito+brain_region+Sex	1.018	2398.000
ENSG00000141503	0.850	0.038	0.220	-0.145	MINK1	0.931	~p_tau+(1|manifest)+cngeneson+pc_mito+brain_region+Sex	2.320	1059.000
ENSG00000168374	0.362	0.038	0.109	-0.034	ARF4	0.616	~p_tau+(1|manifest)+cngeneson+pc_mito+brain_region+Sex	0.538	3769.000
ENSG00000131051	0.759	0.037	0.163	-0.088	RBM39	0.878	~p_tau+(1|manifest)+cngeneson+pc_mito+brain_region+Sex	1.301	1910.000
ENSG00000081019	0.267	0.037	0.121	-0.046	RSBN1	0.521	~p_tau+(1|manifest)+cngeneson+pc_mito+brain_region+Sex	0.240	5113.000
ENSG00000141458	0.918	0.037	0.263	-0.188	NPC1	0.961	~p_tau+(1|manifest)+cngeneson+pc_mito+brain_region+Sex	1.658	1491.000
ENSG00000166428	0.752	0.037	0.335	-0.260	PLD4	0.875	~p_tau+(1|manifest)+cngeneson+pc_mito+brain_region+Sex	3.961	529.000
ENSG00000181163	0.067	0.037	0.140	-0.065	NPM1	0.245	~p_tau+(1|manifest)+cngeneson+pc_mito+brain_region+Sex	1.033	2375.000
ENSG00000115216	0.085	0.037	0.095	-0.020	NRBP1	0.278	~p_tau+(1|manifest)+cngeneson+pc_mito+brain_region+Sex	0.198	5235.000
ENSG00000163683	0.839	0.037	0.173	-0.099	SMIM14	0.925	~p_tau+(1|manifest)+cngeneson+pc_mito+brain_region+Sex	0.910	2651.000
ENSG00000175203	0.033	0.037	0.079	-0.005	DCTN2	0.169	~p_tau+(1|manifest)+cngeneson+pc_mito+brain_region+Sex	0.222	5170.000
ENSG00000198160	0.301	0.037	0.122	-0.048	MIER1	0.556	~p_tau+(1|manifest)+cngeneson+pc_mito+brain_region+Sex	0.455	4122.000
ENSG00000127511	0.039	0.037	0.127	-0.053	SIN3B	0.185	~p_tau+(1|manifest)+cngeneson+pc_mito+brain_region+Sex	0.602	3526.000
ENSG00000099942	0.133	0.037	0.084	-0.010	CRKL	0.354	~p_tau+(1|manifest)+cngeneson+pc_mito+brain_region+Sex	0.173	5320.000
ENSG00000198265	0.558	0.037	0.177	-0.103	HELZ	0.761	~p_tau+(1|manifest)+cngeneson+pc_mito+brain_region+Sex	1.106	2241.000
ENSG00000130429	0.891	0.037	0.209	-0.135	ARPC1B	0.950	~p_tau+(1|manifest)+cngeneson+pc_mito+brain_region+Sex	1.282	1939.000
ENSG00000137817	0.016	0.037	0.092	-0.018	PARP6	0.118	~p_tau+(1|manifest)+cngeneson+pc_mito+brain_region+Sex	0.317	4738.000
ENSG00000196235	0.068	0.037	0.088	-0.014	SUPT5H	0.245	~p_tau+(1|manifest)+cngeneson+pc_mito+brain_region+Sex	0.301	4813.000
ENSG00000107566	0.134	0.037	0.090	-0.016	ERLIN1	0.354	~p_tau+(1|manifest)+cngeneson+pc_mito+brain_region+Sex	0.353	4563.000
ENSG00000103995	0.593	0.037	0.127	-0.053	CEP152	0.783	~p_tau+(1|manifest)+cngeneson+pc_mito+brain_region+Sex	0.467	4075.000
ENSG00000115520	0.082	0.037	0.073	0.001	COQ10B	0.273	~p_tau+(1|manifest)+cngeneson+pc_mito+brain_region+Sex	0.279	4926.000
ENSG00000165792	0.301	0.037	0.121	-0.047	METTL17	0.556	~p_tau+(1|manifest)+cngeneson+pc_mito+brain_region+Sex	0.505	3907.000
ENSG00000119559	0.453	0.037	0.120	-0.046	C19orf25	0.688	~p_tau+(1|manifest)+cngeneson+pc_mito+brain_region+Sex	0.544	3753.000
ENSG00000176853	0.080	0.037	0.109	-0.035	FAM91A1	0.269	~p_tau+(1|manifest)+cngeneson+pc_mito+brain_region+Sex	0.258	5034.000
ENSG00000129493	0.337	0.037	0.083	-0.009	HEATR5A	0.593	~p_tau+(1|manifest)+cngeneson+pc_mito+brain_region+Sex	0.146	5391.000
ENSG00000101751	0.553	0.037	0.136	-0.063	POLI	0.759	~p_tau+(1|manifest)+cngeneson+pc_mito+brain_region+Sex	0.571	3643.000
ENSG00000197056	0.356	0.037	0.105	-0.032	ZMYM1	0.611	~p_tau+(1|manifest)+cngeneson+pc_mito+brain_region+Sex	0.305	4804.000
ENSG00000165338	0.844	0.036	0.307	-0.234	HECTD2	0.926	~p_tau+(1|manifest)+cngeneson+pc_mito+brain_region+Sex	1.666	1481.000
ENSG00000136986	0.290	0.036	0.095	-0.022	DERL1	0.543	~p_tau+(1|manifest)+cngeneson+pc_mito+brain_region+Sex	0.278	4930.000
ENSG00000197798	0.467	0.036	0.119	-0.047	FAM118B	0.699	~p_tau+(1|manifest)+cngeneson+pc_mito+brain_region+Sex	0.516	3857.000
ENSG00000164961	0.745	0.036	0.135	-0.063	WASHC5	0.872	~p_tau+(1|manifest)+cngeneson+pc_mito+brain_region+Sex	0.595	3555.000
ENSG00000170185	0.016	0.036	0.075	-0.003	USP38	0.115	~p_tau+(1|manifest)+cngeneson+pc_mito+brain_region+Sex	0.128	5427.000
ENSG00000270647	0.886	0.036	0.252	-0.179	TAF15	0.948	~p_tau+(1|manifest)+cngeneson+pc_mito+brain_region+Sex	1.527	1627.000
ENSG00000007047	0.178	0.036	0.118	-0.046	MARK4	0.415	~p_tau+(1|manifest)+cngeneson+pc_mito+brain_region+Sex	0.451	4141.000
ENSG00000144426	0.863	0.036	0.176	-0.104	NBEAL1	0.938	~p_tau+(1|manifest)+cngeneson+pc_mito+brain_region+Sex	0.929	2591.000
ENSG00000164091	0.427	0.036	0.122	-0.050	WDR82	0.669	~p_tau+(1|manifest)+cngeneson+pc_mito+brain_region+Sex	0.680	3268.000
ENSG00000138303	0.866	0.036	0.202	-0.131	ASCC1	0.939	~p_tau+(1|manifest)+cngeneson+pc_mito+brain_region+Sex	0.263	5010.000
ENSG00000116266	0.726	0.036	0.233	-0.162	STXBP3	0.863	~p_tau+(1|manifest)+cngeneson+pc_mito+brain_region+Sex	1.169	2131.000
ENSG00000108559	0.368	0.036	0.127	-0.056	NUP88	0.621	~p_tau+(1|manifest)+cngeneson+pc_mito+brain_region+Sex	0.545	3744.000
ENSG00000163785	0.568	0.036	0.180	-0.108	RYK	0.766	~p_tau+(1|manifest)+cngeneson+pc_mito+brain_region+Sex	0.777	2996.000
ENSG00000185024	0.073	0.036	0.082	-0.011	BRF1	0.256	~p_tau+(1|manifest)+cngeneson+pc_mito+brain_region+Sex	0.174	5317.000
ENSG00000186111	0.015	0.036	0.073	-0.001	PIP5K1C	0.110	~p_tau+(1|manifest)+cngeneson+pc_mito+brain_region+Sex	0.337	4646.000
ENSG00000106415	0.519	0.036	0.211	-0.140	GLCCI1	0.733	~p_tau+(1|manifest)+cngeneson+pc_mito+brain_region+Sex	1.445	1733.000
ENSG00000131013	0.282	0.036	0.103	-0.032	PPIL4	0.535	~p_tau+(1|manifest)+cngeneson+pc_mito+brain_region+Sex	0.152	5380.000
ENSG00000147010	0.803	0.036	0.153	-0.081	SH3KBP1	0.904	~p_tau+(1|manifest)+cngeneson+pc_mito+brain_region+Sex	0.861	2765.000
ENSG00000119471	0.566	0.036	0.105	-0.034	HSDL2	0.766	~p_tau+(1|manifest)+cngeneson+pc_mito+brain_region+Sex	0.453	4129.000
ENSG00000100376	0.931	0.035	0.299	-0.228	FAM118A	0.967	~p_tau+(1|manifest)+cngeneson+pc_mito+brain_region+Sex	6.105	275.000
ENSG00000197694	0.573	0.035	0.114	-0.043	SPTAN1	0.770	~p_tau+(1|manifest)+cngeneson+pc_mito+brain_region+Sex	0.564	3668.000
ENSG00000151702	0.800	0.035	0.158	-0.087	FLI1	0.903	~p_tau+(1|manifest)+cngeneson+pc_mito+brain_region+Sex	1.463	1705.000
ENSG00000204842	0.857	0.035	0.167	-0.096	ATXN2	0.935	~p_tau+(1|manifest)+cngeneson+pc_mito+brain_region+Sex	0.954	2538.000
ENSG00000066654	0.337	0.035	0.095	-0.025	THUMPD1	0.593	~p_tau+(1|manifest)+cngeneson+pc_mito+brain_region+Sex	0.197	5238.000
ENSG00000140443	0.839	0.035	0.220	-0.150	IGF1R	0.925	~p_tau+(1|manifest)+cngeneson+pc_mito+brain_region+Sex	1.023	2389.000
ENSG00000204130	0.270	0.035	0.077	-0.007	RUFY2	0.524	~p_tau+(1|manifest)+cngeneson+pc_mito+brain_region+Sex	0.185	5280.000
ENSG00000089094	0.913	0.035	0.220	-0.151	KDM2B	0.959	~p_tau+(1|manifest)+cngeneson+pc_mito+brain_region+Sex	1.898	1313.000
ENSG00000151806	0.512	0.035	0.094	-0.024	GUF1	0.730	~p_tau+(1|manifest)+cngeneson+pc_mito+brain_region+Sex	0.243	5100.000
ENSG00000144118	0.263	0.035	0.098	-0.028	RALB	0.517	~p_tau+(1|manifest)+cngeneson+pc_mito+brain_region+Sex	0.226	5152.000
ENSG00000087008	0.244	0.035	0.075	-0.005	ACOX3	0.494	~p_tau+(1|manifest)+cngeneson+pc_mito+brain_region+Sex	0.365	4497.000
ENSG00000099783	0.899	0.035	0.185	-0.116	HNRNPM	0.953	~p_tau+(1|manifest)+cngeneson+pc_mito+brain_region+Sex	2.041	1223.000
ENSG00000090316	0.646	0.035	0.122	-0.052	MAEA	0.816	~p_tau+(1|manifest)+cngeneson+pc_mito+brain_region+Sex	0.822	2884.000
ENSG00000196369	0.941	0.035	0.231	-0.162	SRGAP2B	0.972	~p_tau+(1|manifest)+cngeneson+pc_mito+brain_region+Sex	6.184	265.000
ENSG00000131323	0.944	0.035	0.246	-0.177	TRAF3	0.973	~p_tau+(1|manifest)+cngeneson+pc_mito+brain_region+Sex	0.775	2999.000
ENSG00000154930	0.908	0.035	0.234	-0.165	ACSS1	0.957	~p_tau+(1|manifest)+cngeneson+pc_mito+brain_region+Sex	1.519	1641.000
ENSG00000143799	0.343	0.035	0.080	-0.011	PARP1	0.597	~p_tau+(1|manifest)+cngeneson+pc_mito+brain_region+Sex	0.203	5224.000
ENSG00000076108	0.136	0.035	0.174	-0.105	BAZ2A	0.357	~p_tau+(1|manifest)+cngeneson+pc_mito+brain_region+Sex	0.429	4220.000
ENSG00000196597	0.662	0.035	0.117	-0.048	ZNF782	0.826	~p_tau+(1|manifest)+cngeneson+pc_mito+brain_region+Sex	0.282	4917.000
ENSG00000067057	0.573	0.034	0.127	-0.058	PFKP	0.770	~p_tau+(1|manifest)+cngeneson+pc_mito+brain_region+Sex	0.172	5323.000
ENSG00000065559	0.619	0.034	0.123	-0.054	MAP2K4	0.798	~p_tau+(1|manifest)+cngeneson+pc_mito+brain_region+Sex	0.393	4389.000
ENSG00000102218	0.169	0.034	0.101	-0.033	RP2	0.401	~p_tau+(1|manifest)+cngeneson+pc_mito+brain_region+Sex	0.501	3924.000
ENSG00000151687	0.915	0.034	0.228	-0.160	ANKAR	0.960	~p_tau+(1|manifest)+cngeneson+pc_mito+brain_region+Sex	1.082	2287.000
ENSG00000133574	0.564	0.034	0.232	-0.163	GIMAP4	0.764	~p_tau+(1|manifest)+cngeneson+pc_mito+brain_region+Sex	1.876	1333.000
ENSG00000157916	0.139	0.034	0.089	-0.021	RER1	0.362	~p_tau+(1|manifest)+cngeneson+pc_mito+brain_region+Sex	0.586	3586.000
ENSG00000162601	0.801	0.034	0.138	-0.071	MYSM1	0.903	~p_tau+(1|manifest)+cngeneson+pc_mito+brain_region+Sex	0.290	4868.000
ENSG00000100422	0.699	0.034	0.173	-0.106	CERK	0.848	~p_tau+(1|manifest)+cngeneson+pc_mito+brain_region+Sex	1.123	2211.000
ENSG00000008018	0.473	0.034	0.129	-0.061	PSMB1	0.702	~p_tau+(1|manifest)+cngeneson+pc_mito+brain_region+Sex	0.350	4580.000
ENSG00000083845	0.241	0.034	0.189	-0.121	RPS5	0.492	~p_tau+(1|manifest)+cngeneson+pc_mito+brain_region+Sex	1.076	2294.000
ENSG00000120533	0.262	0.034	0.112	-0.045	ENY2	0.516	~p_tau+(1|manifest)+cngeneson+pc_mito+brain_region+Sex	0.384	4420.000
ENSG00000179387	0.189	0.034	0.090	-0.023	ELMOD2	0.429	~p_tau+(1|manifest)+cngeneson+pc_mito+brain_region+Sex	0.235	5129.000
ENSG00000137504	0.181	0.034	0.133	-0.066	CREBZF	0.420	~p_tau+(1|manifest)+cngeneson+pc_mito+brain_region+Sex	1.237	2024.000
ENSG00000173638	0.254	0.034	0.127	-0.060	SLC19A1	0.508	~p_tau+(1|manifest)+cngeneson+pc_mito+brain_region+Sex	0.425	4239.000
ENSG00000099219	0.207	0.034	0.070	-0.003	ERMP1	0.450	~p_tau+(1|manifest)+cngeneson+pc_mito+brain_region+Sex	0.154	5372.000
ENSG00000132640	0.526	0.034	0.097	-0.030	BTBD3	0.738	~p_tau+(1|manifest)+cngeneson+pc_mito+brain_region+Sex	0.241	5108.000
ENSG00000160948	0.156	0.033	0.121	-0.054	VPS28	0.384	~p_tau+(1|manifest)+cngeneson+pc_mito+brain_region+Sex	0.753	3057.000
ENSG00000205269	0.416	0.033	0.183	-0.116	TMEM170B	0.660	~p_tau+(1|manifest)+cngeneson+pc_mito+brain_region+Sex	0.527	3802.000
ENSG00000173436	0.906	0.033	0.382	-0.315	MICOS10	0.956	~p_tau+(1|manifest)+cngeneson+pc_mito+brain_region+Sex	5.388	323.000
ENSG00000164169	0.229	0.033	0.102	-0.035	PRMT9	0.477	~p_tau+(1|manifest)+cngeneson+pc_mito+brain_region+Sex	0.369	4479.000
ENSG00000105186	0.200	0.033	0.071	-0.004	ANKRD27	0.442	~p_tau+(1|manifest)+cngeneson+pc_mito+brain_region+Sex	0.318	4734.000
ENSG00000197102	0.309	0.033	0.141	-0.075	DYNC1H1	0.565	~p_tau+(1|manifest)+cngeneson+pc_mito+brain_region+Sex	0.849	2800.000
ENSG00000160299	0.493	0.033	0.129	-0.064	PCNT	0.716	~p_tau+(1|manifest)+cngeneson+pc_mito+brain_region+Sex	0.845	2807.000
ENSG00000183735	0.514	0.033	0.098	-0.032	TBK1	0.731	~p_tau+(1|manifest)+cngeneson+pc_mito+brain_region+Sex	0.391	4394.000
ENSG00000159592	0.695	0.033	0.144	-0.079	GPBP1L1	0.845	~p_tau+(1|manifest)+cngeneson+pc_mito+brain_region+Sex	0.635	3405.000
ENSG00000106266	0.310	0.033	0.118	-0.053	SNX8	0.565	~p_tau+(1|manifest)+cngeneson+pc_mito+brain_region+Sex	0.650	3362.000
ENSG00000143702	0.836	0.033	0.153	-0.088	CEP170	0.923	~p_tau+(1|manifest)+cngeneson+pc_mito+brain_region+Sex	1.139	2180.000
ENSG00000185619	0.525	0.033	0.120	-0.054	PCGF3	0.738	~p_tau+(1|manifest)+cngeneson+pc_mito+brain_region+Sex	0.499	3937.000
ENSG00000138757	0.400	0.032	0.095	-0.030	G3BP2	0.646	~p_tau+(1|manifest)+cngeneson+pc_mito+brain_region+Sex	0.401	4357.000
ENSG00000111846	0.669	0.032	0.358	-0.293	GCNT2	0.831	~p_tau+(1|manifest)+cngeneson+pc_mito+brain_region+Sex	1.727	1434.000
ENSG00000172270	0.618	0.032	0.099	-0.034	BSG	0.798	~p_tau+(1|manifest)+cngeneson+pc_mito+brain_region+Sex	0.235	5130.000
ENSG00000143420	0.501	0.032	0.129	-0.065	ENSA	0.722	~p_tau+(1|manifest)+cngeneson+pc_mito+brain_region+Sex	0.226	5153.000
ENSG00000183726	0.173	0.032	0.094	-0.030	TMEM50A	0.407	~p_tau+(1|manifest)+cngeneson+pc_mito+brain_region+Sex	0.377	4448.000
ENSG00000106682	0.185	0.032	0.103	-0.038	EIF4H	0.425	~p_tau+(1|manifest)+cngeneson+pc_mito+brain_region+Sex	0.502	3920.000
ENSG00000196312	0.763	0.032	0.138	-0.074	MFSD14C	0.881	~p_tau+(1|manifest)+cngeneson+pc_mito+brain_region+Sex	0.886	2699.000
ENSG00000104643	0.504	0.032	0.114	-0.050	MTMR9	0.725	~p_tau+(1|manifest)+cngeneson+pc_mito+brain_region+Sex	0.285	4903.000
ENSG00000074964	0.860	0.032	0.227	-0.163	ARHGEF10L	0.937	~p_tau+(1|manifest)+cngeneson+pc_mito+brain_region+Sex	2.076	1200.000
ENSG00000135929	0.414	0.032	0.604	-0.540	CYP27A1	0.658	~p_tau+(1|manifest)+cngeneson+pc_mito+brain_region+Sex	4.803	394.000
ENSG00000156531	0.120	0.032	0.119	-0.055	PHF6	0.336	~p_tau+(1|manifest)+cngeneson+pc_mito+brain_region+Sex	0.189	5268.000
ENSG00000133606	0.480	0.032	0.096	-0.033	MKRN1	0.706	~p_tau+(1|manifest)+cngeneson+pc_mito+brain_region+Sex	0.507	3894.000
ENSG00000166887	0.671	0.032	0.117	-0.054	VPS39	0.833	~p_tau+(1|manifest)+cngeneson+pc_mito+brain_region+Sex	0.522	3830.000
ENSG00000133997	0.089	0.032	0.061	0.002	MED6	0.284	~p_tau+(1|manifest)+cngeneson+pc_mito+brain_region+Sex	0.217	5181.000
ENSG00000112941	0.803	0.032	0.136	-0.073	TENT4A	0.905	~p_tau+(1|manifest)+cngeneson+pc_mito+brain_region+Sex	0.800	2932.000
ENSG00000112685	0.899	0.031	0.203	-0.140	EXOC2	0.953	~p_tau+(1|manifest)+cngeneson+pc_mito+brain_region+Sex	1.294	1924.000
ENSG00000122705	0.641	0.031	0.099	-0.037	CLTA	0.811	~p_tau+(1|manifest)+cngeneson+pc_mito+brain_region+Sex	0.840	2829.000
ENSG00000075539	0.745	0.031	0.173	-0.110	FRYL	0.872	~p_tau+(1|manifest)+cngeneson+pc_mito+brain_region+Sex	0.741	3082.000
ENSG00000167193	0.841	0.031	0.136	-0.074	CRK	0.925	~p_tau+(1|manifest)+cngeneson+pc_mito+brain_region+Sex	0.892	2688.000
ENSG00000183160	0.277	0.031	0.239	-0.177	TMEM119	0.530	~p_tau+(1|manifest)+cngeneson+pc_mito+brain_region+Sex	1.768	1411.000
ENSG00000158545	0.606	0.031	0.106	-0.043	ZC3H18	0.790	~p_tau+(1|manifest)+cngeneson+pc_mito+brain_region+Sex	0.445	4161.000
ENSG00000128585	0.931	0.031	0.192	-0.130	MKLN1	0.967	~p_tau+(1|manifest)+cngeneson+pc_mito+brain_region+Sex	1.596	1562.000
ENSG00000100697	0.911	0.031	0.271	-0.209	DICER1	0.959	~p_tau+(1|manifest)+cngeneson+pc_mito+brain_region+Sex	2.264	1092.000
ENSG00000137500	0.010	0.031	0.117	-0.055	CCDC90B	0.091	~p_tau+(1|manifest)+cngeneson+pc_mito+brain_region+Sex	0.527	3801.000
ENSG00000198408	0.917	0.031	0.307	-0.245	OGA	0.961	~p_tau+(1|manifest)+cngeneson+pc_mito+brain_region+Sex	2.246	1103.000
ENSG00000102879	0.566	0.031	0.279	-0.216	CORO1A	0.766	~p_tau+(1|manifest)+cngeneson+pc_mito+brain_region+Sex	2.416	1008.000
ENSG00000113387	0.140	0.031	0.089	-0.028	SUB1	0.363	~p_tau+(1|manifest)+cngeneson+pc_mito+brain_region+Sex	0.565	3663.000
ENSG00000115694	0.200	0.031	0.109	-0.047	STK25	0.442	~p_tau+(1|manifest)+cngeneson+pc_mito+brain_region+Sex	0.541	3761.000
ENSG00000169299	0.107	0.031	0.104	-0.042	PGM2	0.313	~p_tau+(1|manifest)+cngeneson+pc_mito+brain_region+Sex	0.468	4073.000
ENSG00000165632	0.771	0.031	0.200	-0.138	TAF3	0.887	~p_tau+(1|manifest)+cngeneson+pc_mito+brain_region+Sex	0.853	2781.000
ENSG00000181481	0.841	0.031	0.137	-0.075	RNF135	0.925	~p_tau+(1|manifest)+cngeneson+pc_mito+brain_region+Sex	0.623	3454.000
ENSG00000127948	0.067	0.031	0.081	-0.020	POR	0.245	~p_tau+(1|manifest)+cngeneson+pc_mito+brain_region+Sex	0.306	4797.000
ENSG00000156831	0.646	0.030	0.201	-0.140	NSMCE2	0.816	~p_tau+(1|manifest)+cngeneson+pc_mito+brain_region+Sex	0.853	2785.000

ENSG00000165669	0.491	0.030	0.081	-0.020	FAM204A	0.714	~p_tau+(1|manifest)+cngeneson+pc_mito+brain_region+Sex	0.283	4911.000
ENSG00000196712	0.630	0.030	0.147	-0.087	NF1	0.806	~p_tau+(1|manifest)+cngeneson+pc_mito+brain_region+Sex	1.582	1579.000
ENSG00000135801	0.377	0.030	0.099	-0.038	TAF5L	0.628	~p_tau+(1|manifest)+cngeneson+pc_mito+brain_region+Sex	0.347	4592.000
ENSG00000169895	0.599	0.030	0.098	-0.038	SYAP1	0.786	~p_tau+(1|manifest)+cngeneson+pc_mito+brain_region+Sex	0.368	4483.000
ENSG00000155363	0.593	0.030	0.103	-0.043	MOV10	0.783	~p_tau+(1|manifest)+cngeneson+pc_mito+brain_region+Sex	0.416	4290.000
ENSG00000163541	0.039	0.030	0.070	-0.009	SUCLG1	0.184	~p_tau+(1|manifest)+cngeneson+pc_mito+brain_region+Sex	0.194	5253.000
ENSG00000141644	0.066	0.030	0.075	-0.015	MBD1	0.244	~p_tau+(1|manifest)+cngeneson+pc_mito+brain_region+Sex	0.326	4704.000
ENSG00000149091	0.482	0.030	0.134	-0.074	DGKZ	0.708	~p_tau+(1|manifest)+cngeneson+pc_mito+brain_region+Sex	0.629	3427.000
ENSG00000012660	0.788	0.030	0.380	-0.320	ELOVL5	0.895	~p_tau+(1|manifest)+cngeneson+pc_mito+brain_region+Sex	2.824	828.000
ENSG00000122550	0.264	0.030	0.086	-0.026	KLHL7	0.517	~p_tau+(1|manifest)+cngeneson+pc_mito+brain_region+Sex	0.130	5420.000
ENSG00000112787	0.264	0.030	0.093	-0.033	FBRSL1	0.517	~p_tau+(1|manifest)+cngeneson+pc_mito+brain_region+Sex	0.555	3701.000
ENSG00000091542	0.102	0.030	0.111	-0.051	ALKBH5	0.305	~p_tau+(1|manifest)+cngeneson+pc_mito+brain_region+Sex	0.285	4898.000
ENSG00000176542	0.602	0.030	0.111	-0.052	USF3	0.787	~p_tau+(1|manifest)+cngeneson+pc_mito+brain_region+Sex	0.179	5298.000
ENSG00000040341	0.657	0.030	0.121	-0.061	STAU2	0.823	~p_tau+(1|manifest)+cngeneson+pc_mito+brain_region+Sex	0.500	3929.000
ENSG00000121749	0.728	0.030	0.165	-0.105	TBC1D15	0.863	~p_tau+(1|manifest)+cngeneson+pc_mito+brain_region+Sex	0.712	3167.000
ENSG00000130402	0.709	0.030	0.108	-0.049	ACTN4	0.854	~p_tau+(1|manifest)+cngeneson+pc_mito+brain_region+Sex	0.306	4794.000
ENSG00000021574	0.679	0.030	0.115	-0.056	SPAST	0.838	~p_tau+(1|manifest)+cngeneson+pc_mito+brain_region+Sex	0.230	5141.000
ENSG00000144228	0.931	0.030	0.226	-0.166	SPOPL	0.967	~p_tau+(1|manifest)+cngeneson+pc_mito+brain_region+Sex	0.849	2798.000
ENSG00000147140	0.094	0.030	0.087	-0.028	NONO	0.290	~p_tau+(1|manifest)+cngeneson+pc_mito+brain_region+Sex	0.537	3776.000
ENSG00000076321	0.332	0.029	0.111	-0.052	KLHL20	0.589	~p_tau+(1|manifest)+cngeneson+pc_mito+brain_region+Sex	0.166	5342.000
ENSG00000071127	0.502	0.029	0.097	-0.038	WDR1	0.723	~p_tau+(1|manifest)+cngeneson+pc_mito+brain_region+Sex	0.727	3122.000
ENSG00000175414	0.123	0.029	0.064	-0.005	ARL10	0.340	~p_tau+(1|manifest)+cngeneson+pc_mito+brain_region+Sex	0.195	5250.000
ENSG00000182903	0.350	0.029	0.107	-0.049	ZNF721	0.603	~p_tau+(1|manifest)+cngeneson+pc_mito+brain_region+Sex	0.310	4777.000
ENSG00000197724	0.164	0.029	0.082	-0.024	PHF2	0.395	~p_tau+(1|manifest)+cngeneson+pc_mito+brain_region+Sex	0.299	4817.000
ENSG00000161533	0.212	0.029	0.063	-0.005	ACOX1	0.455	~p_tau+(1|manifest)+cngeneson+pc_mito+brain_region+Sex	0.353	4564.000
ENSG00000142864	0.515	0.029	0.134	-0.076	SERBP1	0.731	~p_tau+(1|manifest)+cngeneson+pc_mito+brain_region+Sex	0.707	3183.000
ENSG00000196505	0.121	0.029	0.151	-0.093	GDAP2	0.337	~p_tau+(1|manifest)+cngeneson+pc_mito+brain_region+Sex	0.427	4231.000
ENSG00000198792	0.057	0.029	0.080	-0.022	TMEM184B	0.227	~p_tau+(1|manifest)+cngeneson+pc_mito+brain_region+Sex	0.251	5068.000
ENSG00000164190	0.852	0.029	0.140	-0.083	NIPBL	0.932	~p_tau+(1|manifest)+cngeneson+pc_mito+brain_region+Sex	3.616	617.000
ENSG00000169599	0.135	0.029	0.061	-0.004	NFU1	0.356	~p_tau+(1|manifest)+cngeneson+pc_mito+brain_region+Sex	0.195	5245.000
ENSG00000138668	0.706	0.029	0.216	-0.158	HNRNPD	0.852	~p_tau+(1|manifest)+cngeneson+pc_mito+brain_region+Sex	1.606	1549.000
ENSG00000183475	0.408	0.029	0.071	-0.013	ASB7	0.654	~p_tau+(1|manifest)+cngeneson+pc_mito+brain_region+Sex	0.139	5407.000
ENSG00000162645	0.773	0.029	0.224	-0.166	GBP2	0.887	~p_tau+(1|manifest)+cngeneson+pc_mito+brain_region+Sex	2.113	1175.000
ENSG00000213064	0.689	0.029	0.189	-0.131	SFT2D2	0.842	~p_tau+(1|manifest)+cngeneson+pc_mito+brain_region+Sex	1.276	1956.000
ENSG00000091164	0.231	0.029	0.083	-0.025	TXNL1	0.480	~p_tau+(1|manifest)+cngeneson+pc_mito+brain_region+Sex	0.321	4725.000
ENSG00000156136	0.266	0.029	0.106	-0.049	DCK	0.520	~p_tau+(1|manifest)+cngeneson+pc_mito+brain_region+Sex	0.612	3486.000
ENSG00000124356	0.384	0.029	0.080	-0.023	STAMBP	0.632	~p_tau+(1|manifest)+cngeneson+pc_mito+brain_region+Sex	0.196	5240.000
ENSG00000197162	0.482	0.029	0.119	-0.062	ZNF785	0.708	~p_tau+(1|manifest)+cngeneson+pc_mito+brain_region+Sex	0.711	3168.000
ENSG00000149716	0.649	0.029	0.098	-0.041	LTO1	0.817	~p_tau+(1|manifest)+cngeneson+pc_mito+brain_region+Sex	0.364	4503.000
ENSG00000023041	0.287	0.029	0.086	-0.029	ZDHHC6	0.539	~p_tau+(1|manifest)+cngeneson+pc_mito+brain_region+Sex	0.294	4842.000
ENSG00000103275	0.312	0.029	0.072	-0.015	UBE2I	0.567	~p_tau+(1|manifest)+cngeneson+pc_mito+brain_region+Sex	0.135	5414.000
ENSG00000100220	0.551	0.029	0.162	-0.105	RTCB	0.757	~p_tau+(1|manifest)+cngeneson+pc_mito+brain_region+Sex	0.755	3050.000
ENSG00000166619	0.786	0.029	0.110	-0.053	BLCAP	0.894	~p_tau+(1|manifest)+cngeneson+pc_mito+brain_region+Sex	0.411	4303.000
ENSG00000140464	0.135	0.029	0.079	-0.022	PML	0.355	~p_tau+(1|manifest)+cngeneson+pc_mito+brain_region+Sex	0.298	4822.000
ENSG00000122779	0.261	0.028	0.080	-0.023	TRIM24	0.515	~p_tau+(1|manifest)+cngeneson+pc_mito+brain_region+Sex	0.176	5307.000
ENSG00000074356	0.150	0.028	0.109	-0.052	NCBP3	0.378	~p_tau+(1|manifest)+cngeneson+pc_mito+brain_region+Sex	0.209	5214.000
ENSG00000135720	0.592	0.028	0.088	-0.031	DYNC1LI2	0.782	~p_tau+(1|manifest)+cngeneson+pc_mito+brain_region+Sex	0.358	4531.000
ENSG00000110719	0.126	0.028	0.181	-0.125	TCIRG1	0.343	~p_tau+(1|manifest)+cngeneson+pc_mito+brain_region+Sex	0.591	3570.000
ENSG00000177125	0.419	0.028	0.086	-0.029	ZBTB34	0.662	~p_tau+(1|manifest)+cngeneson+pc_mito+brain_region+Sex	0.346	4603.000
ENSG00000137947	0.694	0.028	0.095	-0.038	GTF2B	0.845	~p_tau+(1|manifest)+cngeneson+pc_mito+brain_region+Sex	0.234	5133.000
ENSG00000092203	0.176	0.028	0.098	-0.041	TOX4	0.412	~p_tau+(1|manifest)+cngeneson+pc_mito+brain_region+Sex	0.666	3312.000
ENSG00000117054	0.144	0.028	0.123	-0.066	ACADM	0.367	~p_tau+(1|manifest)+cngeneson+pc_mito+brain_region+Sex	0.306	4796.000
ENSG00000059691	0.432	0.028	0.078	-0.021	GATB	0.673	~p_tau+(1|manifest)+cngeneson+pc_mito+brain_region+Sex	0.173	5321.000
ENSG00000086189	0.801	0.028	0.114	-0.057	DIMT1	0.904	~p_tau+(1|manifest)+cngeneson+pc_mito+brain_region+Sex	0.691	3230.000
ENSG00000173889	0.908	0.028	0.154	-0.097	PHC3	0.957	~p_tau+(1|manifest)+cngeneson+pc_mito+brain_region+Sex	0.526	3808.000
ENSG00000197223	0.293	0.028	0.072	-0.016	C1D	0.546	~p_tau+(1|manifest)+cngeneson+pc_mito+brain_region+Sex	0.158	5367.000
ENSG00000072786	0.989	0.028	0.396	-0.340	STK10	0.993	~p_tau+(1|manifest)+cngeneson+pc_mito+brain_region+Sex	3.261	696.000
ENSG00000132383	0.235	0.028	0.096	-0.039	RPA1	0.485	~p_tau+(1|manifest)+cngeneson+pc_mito+brain_region+Sex	0.338	4639.000
ENSG00000179361	0.621	0.028	0.095	-0.038	ARID3B	0.800	~p_tau+(1|manifest)+cngeneson+pc_mito+brain_region+Sex	0.314	4755.000
ENSG00000177054	0.709	0.028	0.094	-0.039	ZDHHC13	0.854	~p_tau+(1|manifest)+cngeneson+pc_mito+brain_region+Sex	0.360	4525.000
ENSG00000120265	0.222	0.028	0.066	-0.011	PCMT1	0.468	~p_tau+(1|manifest)+cngeneson+pc_mito+brain_region+Sex	0.439	4183.000
ENSG00000141699	0.327	0.028	0.105	-0.049	RETREG3	0.585	~p_tau+(1|manifest)+cngeneson+pc_mito+brain_region+Sex	0.243	5095.000
ENSG00000172239	0.029	0.028	0.089	-0.033	PAIP1	0.155	~p_tau+(1|manifest)+cngeneson+pc_mito+brain_region+Sex	0.132	5418.000
ENSG00000072778	0.284	0.028	0.144	-0.088	ACADVL	0.537	~p_tau+(1|manifest)+cngeneson+pc_mito+brain_region+Sex	0.666	3309.000
ENSG00000166478	0.269	0.028	0.105	-0.049	ZNF143	0.523	~p_tau+(1|manifest)+cngeneson+pc_mito+brain_region+Sex	0.561	3681.000
ENSG00000162946	0.561	0.028	0.171	-0.115	DISC1	0.763	~p_tau+(1|manifest)+cngeneson+pc_mito+brain_region+Sex	3.746	578.000
ENSG00000060688	0.286	0.028	0.079	-0.024	SNRNP40	0.539	~p_tau+(1|manifest)+cngeneson+pc_mito+brain_region+Sex	0.263	5009.000
ENSG00000145901	0.365	0.028	0.155	-0.100	TNIP1	0.618	~p_tau+(1|manifest)+cngeneson+pc_mito+brain_region+Sex	0.291	4860.000
ENSG00000213523	0.310	0.028	0.101	-0.046	SRA1	0.565	~p_tau+(1|manifest)+cngeneson+pc_mito+brain_region+Sex	0.550	3722.000
ENSG00000124535	0.376	0.027	0.109	-0.054	WRNIP1	0.628	~p_tau+(1|manifest)+cngeneson+pc_mito+brain_region+Sex	0.684	3253.000
ENSG00000152404	0.455	0.027	0.175	-0.121	CWF19L2	0.689	~p_tau+(1|manifest)+cngeneson+pc_mito+brain_region+Sex	0.770	3010.000
ENSG00000111885	0.717	0.027	0.363	-0.308	MAN1A1	0.859	~p_tau+(1|manifest)+cngeneson+pc_mito+brain_region+Sex	3.203	712.000
ENSG00000118518	0.446	0.027	0.111	-0.056	RNF146	0.683	~p_tau+(1|manifest)+cngeneson+pc_mito+brain_region+Sex	0.306	4791.000
ENSG00000112679	0.202	0.027	0.151	-0.096	DUSP22	0.444	~p_tau+(1|manifest)+cngeneson+pc_mito+brain_region+Sex	0.463	4092.000
ENSG00000171720	0.212	0.027	0.086	-0.031	HDAC3	0.455	~p_tau+(1|manifest)+cngeneson+pc_mito+brain_region+Sex	0.407	4329.000
ENSG00000115145	0.781	0.027	0.115	-0.060	STAM2	0.891	~p_tau+(1|manifest)+cngeneson+pc_mito+brain_region+Sex	0.251	5070.000
ENSG00000168300	0.896	0.027	0.323	-0.269	PCMTD1	0.953	~p_tau+(1|manifest)+cngeneson+pc_mito+brain_region+Sex	4.148	499.000
ENSG00000123600	0.722	0.027	0.093	-0.039	METTL8	0.861	~p_tau+(1|manifest)+cngeneson+pc_mito+brain_region+Sex	0.385	4418.000
ENSG00000130560	0.751	0.027	0.118	-0.064	UBAC1	0.875	~p_tau+(1|manifest)+cngeneson+pc_mito+brain_region+Sex	1.267	1969.000
ENSG00000180776	0.494	0.027	0.166	-0.112	ZDHHC20	0.716	~p_tau+(1|manifest)+cngeneson+pc_mito+brain_region+Sex	0.595	3562.000
ENSG00000256525	0.132	0.027	0.059	-0.005	POLG2	0.353	~p_tau+(1|manifest)+cngeneson+pc_mito+brain_region+Sex	0.317	4737.000
ENSG00000181804	0.874	0.027	0.154	-0.100	SLC9A9	0.942	~p_tau+(1|manifest)+cngeneson+pc_mito+brain_region+Sex	4.615	415.000
ENSG00000119541	0.460	0.027	0.083	-0.029	VPS4B	0.692	~p_tau+(1|manifest)+cngeneson+pc_mito+brain_region+Sex	0.374	4462.000
ENSG00000067082	0.971	0.027	0.245	-0.192	KLF6	0.987	~p_tau+(1|manifest)+cngeneson+pc_mito+brain_region+Sex	1.610	1544.000
ENSG00000196367	0.662	0.027	0.089	-0.036	TRRAP	0.826	~p_tau+(1|manifest)+cngeneson+pc_mito+brain_region+Sex	0.205	5223.000
ENSG00000161813	0.833	0.027	0.205	-0.152	LARP4	0.922	~p_tau+(1|manifest)+cngeneson+pc_mito+brain_region+Sex	1.307	1901.000
ENSG00000189079	0.614	0.026	0.121	-0.068	ARID2	0.794	~p_tau+(1|manifest)+cngeneson+pc_mito+brain_region+Sex	0.464	4086.000
ENSG00000095319	0.048	0.026	0.057	-0.005	NUP188	0.205	~p_tau+(1|manifest)+cngeneson+pc_mito+brain_region+Sex	0.230	5139.000
ENSG00000111540	0.247	0.026	0.077	-0.024	RAB5B	0.499	~p_tau+(1|manifest)+cngeneson+pc_mito+brain_region+Sex	0.159	5366.000
ENSG00000123143	0.674	0.026	0.139	-0.086	PKN1	0.834	~p_tau+(1|manifest)+cngeneson+pc_mito+brain_region+Sex	0.620	3465.000
ENSG00000070367	0.223	0.026	0.143	-0.090	EXOC5	0.469	~p_tau+(1|manifest)+cngeneson+pc_mito+brain_region+Sex	0.595	3560.000
ENSG00000145730	0.735	0.026	0.181	-0.128	PAM	0.867	~p_tau+(1|manifest)+cngeneson+pc_mito+brain_region+Sex	2.153	1153.000
ENSG00000103061	0.409	0.026	0.132	-0.080	SLC7A6OS	0.654	~p_tau+(1|manifest)+cngeneson+pc_mito+brain_region+Sex	0.843	2813.000
ENSG00000164603	0.565	0.026	0.181	-0.128	BMT2	0.765	~p_tau+(1|manifest)+cngeneson+pc_mito+brain_region+Sex	0.942	2569.000
ENSG00000143457	0.776	0.026	0.126	-0.074	GOLPH3L	0.889	~p_tau+(1|manifest)+cngeneson+pc_mito+brain_region+Sex	0.160	5358.000
ENSG00000198420	0.365	0.026	0.062	-0.009	TCAF1	0.618	~p_tau+(1|manifest)+cngeneson+pc_mito+brain_region+Sex	0.136	5411.000
ENSG00000135108	0.195	0.026	0.059	-0.007	FBXO21	0.437	~p_tau+(1|manifest)+cngeneson+pc_mito+brain_region+Sex	0.103	5450.000
ENSG00000095794	0.882	0.026	0.243	-0.190	CREM	0.946	~p_tau+(1|manifest)+cngeneson+pc_mito+brain_region+Sex	1.592	1566.000
ENSG00000112763	0.172	0.026	0.091	-0.039	BTN2A1	0.406	~p_tau+(1|manifest)+cngeneson+pc_mito+brain_region+Sex	0.548	3732.000
ENSG00000159267	0.471	0.026	0.131	-0.079	HLCS	0.700	~p_tau+(1|manifest)+cngeneson+pc_mito+brain_region+Sex	0.824	2879.000
ENSG00000185515	0.431	0.026	0.064	-0.013	BRCC3	0.672	~p_tau+(1|manifest)+cngeneson+pc_mito+brain_region+Sex	0.185	5277.000
ENSG00000115685	0.271	0.026	0.068	-0.017	PPP1R7	0.525	~p_tau+(1|manifest)+cngeneson+pc_mito+brain_region+Sex	0.328	4689.000
ENSG00000166689	0.957	0.026	0.355	-0.303	PLEKHA7	0.978	~p_tau+(1|manifest)+cngeneson+pc_mito+brain_region+Sex	4.226	484.000
ENSG00000175764	0.883	0.026	0.128	-0.077	TTLL11	0.946	~p_tau+(1|manifest)+cngeneson+pc_mito+brain_region+Sex	0.945	2558.000
ENSG00000185947	0.297	0.026	0.094	-0.043	ZNF267	0.552	~p_tau+(1|manifest)+cngeneson+pc_mito+brain_region+Sex	0.598	3543.000
ENSG00000130227	0.680	0.026	0.149	-0.098	XPO7	0.839	~p_tau+(1|manifest)+cngeneson+pc_mito+brain_region+Sex	0.607	3509.000
ENSG00000131697	0.543	0.026	0.091	-0.040	NPHP4	0.751	~p_tau+(1|manifest)+cngeneson+pc_mito+brain_region+Sex	0.463	4091.000
ENSG00000171291	0.434	0.025	0.101	-0.051	ZNF439	0.674	~p_tau+(1|manifest)+cngeneson+pc_mito+brain_region+Sex	0.533	3789.000
ENSG00000149346	0.827	0.025	0.167	-0.116	SLX4IP	0.919	~p_tau+(1|manifest)+cngeneson+pc_mito+brain_region+Sex	0.175	5315.000
ENSG00000197050	0.286	0.025	0.092	-0.041	ZNF420	0.539	~p_tau+(1|manifest)+cngeneson+pc_mito+brain_region+Sex	0.326	4702.000
ENSG00000079482	0.927	0.025	0.166	-0.115	OPHN1	0.966	~p_tau+(1|manifest)+cngeneson+pc_mito+brain_region+Sex	0.229	5145.000
ENSG00000171928	0.146	0.025	0.097	-0.046	TVP23B	0.371	~p_tau+(1|manifest)+cngeneson+pc_mito+brain_region+Sex	0.574	3636.000
ENSG00000133226	0.923	0.025	0.162	-0.111	SRRM1	0.964	~p_tau+(1|manifest)+cngeneson+pc_mito+brain_region+Sex	0.550	3720.000
ENSG00000156110	0.691	0.025	0.177	-0.127	ADK	0.843	~p_tau+(1|manifest)+cngeneson+pc_mito+brain_region+Sex	0.677	3278.000
ENSG00000159658	0.404	0.025	0.110	-0.059	EFCAB14	0.650	~p_tau+(1|manifest)+cngeneson+pc_mito+brain_region+Sex	0.604	3515.000
ENSG00000221978	0.589	0.025	0.141	-0.091	CCNL2	0.781	~p_tau+(1|manifest)+cngeneson+pc_mito+brain_region+Sex	0.849	2796.000
ENSG00000164916	0.929	0.025	0.166	-0.116	FOXK1	0.967	~p_tau+(1|manifest)+cngeneson+pc_mito+brain_region+Sex	0.929	2590.000
ENSG00000100345	0.916	0.025	0.183	-0.133	MYH9	0.960	~p_tau+(1|manifest)+cngeneson+pc_mito+brain_region+Sex	0.945	2557.000
ENSG00000129317	0.834	0.025	0.113	-0.063	PUS7L	0.923	~p_tau+(1|manifest)+cngeneson+pc_mito+brain_region+Sex	0.578	3623.000
ENSG00000152683	0.558	0.025	0.104	-0.054	SLC30A6	0.761	~p_tau+(1|manifest)+cngeneson+pc_mito+brain_region+Sex	0.468	4068.000
ENSG00000152620	0.584	0.025	0.081	-0.030	NADK2	0.777	~p_tau+(1|manifest)+cngeneson+pc_mito+brain_region+Sex	0.257	5042.000
ENSG00000084754	0.330	0.025	0.095	-0.044	HADHA	0.588	~p_tau+(1|manifest)+cngeneson+pc_mito+brain_region+Sex	0.343	4617.000
ENSG00000124313	0.917	0.025	0.166	-0.116	IQSEC2	0.961	~p_tau+(1|manifest)+cngeneson+pc_mito+brain_region+Sex	1.487	1677.000
ENSG00000101413	0.087	0.025	0.079	-0.029	RPRD1B	0.281	~p_tau+(1|manifest)+cngeneson+pc_mito+brain_region+Sex	0.356	4544.000
ENSG00000013375	0.155	0.025	0.089	-0.039	PGM3	0.383	~p_tau+(1|manifest)+cngeneson+pc_mito+brain_region+Sex	0.451	4142.000
ENSG00000158435	0.088	0.025	0.060	-0.010	CNOT11	0.283	~p_tau+(1|manifest)+cngeneson+pc_mito+brain_region+Sex	0.130	5423.000
ENSG00000178338	0.256	0.025	0.085	-0.035	ZNF354B	0.510	~p_tau+(1|manifest)+cngeneson+pc_mito+brain_region+Sex	0.381	4438.000
ENSG00000248333	0.364	0.025	0.090	-0.040	CDK11B	0.617	~p_tau+(1|manifest)+cngeneson+pc_mito+brain_region+Sex	0.428	4228.000
ENSG00000235162	0.620	0.025	0.346	-0.297	C12orf75	0.799	~p_tau+(1|manifest)+cngeneson+pc_mito+brain_region+Sex	2.106	1181.000
ENSG00000284461	0.389	0.025	0.107	-0.057	AC027644.4	0.637	~p_tau+(1|manifest)+cngeneson+pc_mito+brain_region+Sex	1.020	2392.000
ENSG00000275342	0.147	0.025	0.195	-0.145	PRAG1	0.372	~p_tau+(1|manifest)+cngeneson+pc_mito+brain_region+Sex	0.668	3301.000
ENSG00000198589	0.885	0.025	0.128	-0.078	LRBA	0.947	~p_tau+(1|manifest)+cngeneson+pc_mito+brain_region+Sex	0.526	3809.000
ENSG00000150403	0.271	0.025	0.057	-0.008	TMCO3	0.525	~p_tau+(1|manifest)+cngeneson+pc_mito+brain_region+Sex	0.406	4332.000
ENSG00000205659	0.551	0.025	0.109	-0.060	LIN52	0.756	~p_tau+(1|manifest)+cngeneson+pc_mito+brain_region+Sex	0.143	5399.000
ENSG00000007341	0.887	0.025	0.157	-0.108	ST7L	0.948	~p_tau+(1|manifest)+cngeneson+pc_mito+brain_region+Sex	0.494	3962.000
ENSG00000127995	0.367	0.025	0.087	-0.038	CASD1	0.619	~p_tau+(1|manifest)+cngeneson+pc_mito+brain_region+Sex	0.483	4010.000
ENSG00000171503	0.371	0.025	0.084	-0.035	ETFDH	0.624	~p_tau+(1|manifest)+cngeneson+pc_mito+brain_region+Sex	0.145	5395.000
ENSG00000156599	0.251	0.024	0.069	-0.020	ZDHHC5	0.503	~p_tau+(1|manifest)+cngeneson+pc_mito+brain_region+Sex	0.243	5099.000
ENSG00000189266	0.445	0.024	0.082	-0.033	PNRC2	0.683	~p_tau+(1|manifest)+cngeneson+pc_mito+brain_region+Sex	0.200	5233.000
ENSG00000130023	0.073	0.024	0.072	-0.023	ERMARD	0.255	~p_tau+(1|manifest)+cngeneson+pc_mito+brain_region+Sex	0.148	5386.000
ENSG00000196937	0.902	0.024	0.152	-0.103	FAM3C	0.954	~p_tau+(1|manifest)+cngeneson+pc_mito+brain_region+Sex	1.282	1938.000
ENSG00000163904	0.363	0.024	0.084	-0.035	SENP2	0.617	~p_tau+(1|manifest)+cngeneson+pc_mito+brain_region+Sex	0.243	5096.000
ENSG00000130150	0.842	0.024	0.110	-0.062	MOSPD2	0.926	~p_tau+(1|manifest)+cngeneson+pc_mito+brain_region+Sex	0.601	3531.000
ENSG00000119715	0.816	0.024	0.107	-0.059	ESRRB	0.912	~p_tau+(1|manifest)+cngeneson+pc_mito+brain_region+Sex	0.402	4347.000
ENSG00000118961	0.909	0.024	0.166	-0.117	LDAH	0.958	~p_tau+(1|manifest)+cngeneson+pc_mito+brain_region+Sex	1.414	1762.000
ENSG00000110851	0.771	0.024	0.092	-0.044	PRDM4	0.887	~p_tau+(1|manifest)+cngeneson+pc_mito+brain_region+Sex	0.287	4887.000
ENSG00000071243	0.070	0.024	0.085	-0.037	ING3	0.250	~p_tau+(1|manifest)+cngeneson+pc_mito+brain_region+Sex	0.233	5134.000
ENSG00000081721	0.122	0.024	0.079	-0.031	DUSP12	0.338	~p_tau+(1|manifest)+cngeneson+pc_mito+brain_region+Sex	0.363	4514.000
ENSG00000104218	0.819	0.024	0.135	-0.087	CSPP1	0.915	~p_tau+(1|manifest)+cngeneson+pc_mito+brain_region+Sex	0.309	4782.000
ENSG00000170345	0.980	0.024	0.300	-0.252	FOS	0.990	~p_tau+(1|manifest)+cngeneson+pc_mito+brain_region+Sex	6.745	222.000
ENSG00000120594	0.856	0.024	0.115	-0.067	PLXDC2	0.935	~p_tau+(1|manifest)+cngeneson+pc_mito+brain_region+Sex	7.687	176.000
ENSG00000112237	0.023	0.024	0.121	-0.073	CCNC	0.140	~p_tau+(1|manifest)+cngeneson+pc_mito+brain_region+Sex	0.505	3910.000
ENSG00000051009	0.410	0.024	0.124	-0.076	FAM160A2	0.655	~p_tau+(1|manifest)+cngeneson+pc_mito+brain_region+Sex	0.913	2642.000
ENSG00000162402	0.958	0.024	0.203	-0.156	USP24	0.979	~p_tau+(1|manifest)+cngeneson+pc_mito+brain_region+Sex	0.739	3086.000
ENSG00000164919	0.461	0.024	0.080	-0.032	COX6C	0.693	~p_tau+(1|manifest)+cngeneson+pc_mito+brain_region+Sex	0.467	4074.000
ENSG00000165886	0.486	0.024	0.083	-0.036	UBTD1	0.711	~p_tau+(1|manifest)+cngeneson+pc_mito+brain_region+Sex	0.490	3984.000
ENSG00000137574	0.536	0.024	0.083	-0.036	TGS1	0.744	~p_tau+(1|manifest)+cngeneson+pc_mito+brain_region+Sex	0.169	5335.000
ENSG00000133704	0.855	0.024	0.129	-0.082	IPO8	0.935	~p_tau+(1|manifest)+cngeneson+pc_mito+brain_region+Sex	0.371	4470.000
ENSG00000111237	0.412	0.023	0.158	-0.111	VPS29	0.657	~p_tau+(1|manifest)+cngeneson+pc_mito+brain_region+Sex	1.431	1744.000
ENSG00000164631	0.343	0.023	0.084	-0.038	ZNF12	0.597	~p_tau+(1|manifest)+cngeneson+pc_mito+brain_region+Sex	0.211	5202.000
ENSG00000140395	0.111	0.023	0.082	-0.035	WDR61	0.320	~p_tau+(1|manifest)+cngeneson+pc_mito+brain_region+Sex	0.216	5189.000
ENSG00000155085	0.902	0.023	0.148	-0.102	AK9	0.954	~p_tau+(1|manifest)+cngeneson+pc_mito+brain_region+Sex	0.329	4687.000
ENSG00000197756	0.939	0.023	0.430	-0.384	RPL37A	0.971	~p_tau+(1|manifest)+cngeneson+pc_mito+brain_region+Sex	4.910	379.000
ENSG00000083099	0.319	0.023	0.056	-0.009	LYRM2	0.575	~p_tau+(1|manifest)+cngeneson+pc_mito+brain_region+Sex	0.127	5430.000
ENSG00000162971	0.130	0.023	0.079	-0.032	TYW5	0.349	~p_tau+(1|manifest)+cngeneson+pc_mito+brain_region+Sex	0.253	5058.000
ENSG00000178188	0.189	0.023	0.103	-0.056	SH2B1	0.429	~p_tau+(1|manifest)+cngeneson+pc_mito+brain_region+Sex	0.404	4340.000
ENSG00000099940	0.424	0.023	0.079	-0.032	SNAP29	0.667	~p_tau+(1|manifest)+cngeneson+pc_mito+brain_region+Sex	0.244	5091.000
ENSG00000075234	0.603	0.023	0.106	-0.060	TTC38	0.788	~p_tau+(1|manifest)+cngeneson+pc_mito+brain_region+Sex	0.400	4360.000
ENSG00000177646	0.262	0.023	0.062	-0.015	ACAD9	0.516	~p_tau+(1|manifest)+cngeneson+pc_mito+brain_region+Sex	0.292	4856.000
ENSG00000126705	0.603	0.023	0.095	-0.049	AHDC1	0.788	~p_tau+(1|manifest)+cngeneson+pc_mito+brain_region+Sex	0.288	4883.000
ENSG00000149262	0.639	0.023	0.079	-0.033	INTS4	0.811	~p_tau+(1|manifest)+cngeneson+pc_mito+brain_region+Sex	0.159	5364.000
ENSG00000155744	0.794	0.023	0.093	-0.047	FAM126B	0.899	~p_tau+(1|manifest)+cngeneson+pc_mito+brain_region+Sex	0.297	4831.000
ENSG00000033030	0.731	0.023	0.082	-0.036	ZCCHC8	0.865	~p_tau+(1|manifest)+cngeneson+pc_mito+brain_region+Sex	0.149	5382.000
ENSG00000118985	0.913	0.023	0.333	-0.287	ELL2	0.959	~p_tau+(1|manifest)+cngeneson+pc_mito+brain_region+Sex	5.360	325.000
ENSG00000197892	0.633	0.023	0.109	-0.063	KIF13B	0.807	~p_tau+(1|manifest)+cngeneson+pc_mito+brain_region+Sex	0.439	4179.000
ENSG00000165660	0.176	0.023	0.069	-0.023	ABRAXAS2	0.413	~p_tau+(1|manifest)+cngeneson+pc_mito+brain_region+Sex	0.261	5020.000
ENSG00000169018	0.082	0.023	0.071	-0.026	FEM1B	0.272	~p_tau+(1|manifest)+cngeneson+pc_mito+brain_region+Sex	0.248	5081.000
ENSG00000172716	0.753	0.023	0.134	-0.088	SLFN11	0.875	~p_tau+(1|manifest)+cngeneson+pc_mito+brain_region+Sex	0.939	2573.000
ENSG00000125107	0.952	0.023	0.165	-0.119	CNOT1	0.976	~p_tau+(1|manifest)+cngeneson+pc_mito+brain_region+Sex	0.981	2472.000
ENSG00000106560	0.278	0.023	0.102	-0.057	GIMAP2	0.532	~p_tau+(1|manifest)+cngeneson+pc_mito+brain_region+Sex	0.310	4774.000
ENSG00000137522	0.026	0.022	0.058	-0.013	RNF121	0.148	~p_tau+(1|manifest)+cngeneson+pc_mito+brain_region+Sex	0.293	4849.000
ENSG00000120925	0.407	0.022	0.103	-0.059	RNF170	0.654	~p_tau+(1|manifest)+cngeneson+pc_mito+brain_region+Sex	0.188	5272.000
ENSG00000089009	0.609	0.022	0.227	-0.182	RPL6	0.792	~p_tau+(1|manifest)+cngeneson+pc_mito+brain_region+Sex	2.220	1123.000
ENSG00000069329	0.823	0.022	0.133	-0.088	VPS35	0.917	~p_tau+(1|manifest)+cngeneson+pc_mito+brain_region+Sex	0.845	2805.000
ENSG00000174606	0.831	0.022	0.127	-0.083	ANGEL2	0.921	~p_tau+(1|manifest)+cngeneson+pc_mito+brain_region+Sex	0.590	3576.000
ENSG00000103148	0.564	0.022	0.096	-0.052	NPRL3	0.764	~p_tau+(1|manifest)+cngeneson+pc_mito+brain_region+Sex	0.190	5265.000
ENSG00000137714	0.755	0.022	0.110	-0.065	FDX1	0.876	~p_tau+(1|manifest)+cngeneson+pc_mito+brain_region+Sex	0.521	3831.000
ENSG00000165138	0.017	0.022	0.101	-0.057	ANKS6	0.119	~p_tau+(1|manifest)+cngeneson+pc_mito+brain_region+Sex	0.655	3339.000
ENSG00000067225	0.970	0.022	0.382	-0.338	PKM	0.986	~p_tau+(1|manifest)+cngeneson+pc_mito+brain_region+Sex	1.751	1419.000
ENSG00000094916	0.469	0.022	0.097	-0.053	CBX5	0.700	~p_tau+(1|manifest)+cngeneson+pc_mito+brain_region+Sex	0.291	4861.000
ENSG00000159189	0.865	0.022	0.522	-0.479	C1QC	0.939	~p_tau+(1|manifest)+cngeneson+pc_mito+brain_region+Sex	7.163	202.000
ENSG00000126561	0.611	0.022	0.106	-0.062	STAT5A	0.793	~p_tau+(1|manifest)+cngeneson+pc_mito+brain_region+Sex	0.624	3451.000
ENSG00000153006	0.756	0.022	0.083	-0.040	SREK1IP1	0.876	~p_tau+(1|manifest)+cngeneson+pc_mito+brain_region+Sex	0.049	5465.000
ENSG00000116497	0.868	0.022	0.193	-0.150	S100PBP	0.939	~p_tau+(1|manifest)+cngeneson+pc_mito+brain_region+Sex	0.462	4096.000
ENSG00000075415	0.199	0.021	0.097	-0.054	SLC25A3	0.441	~p_tau+(1|manifest)+cngeneson+pc_mito+brain_region+Sex	0.723	3133.000
ENSG00000089063	0.198	0.021	0.129	-0.086	TMEM230	0.440	~p_tau+(1|manifest)+cngeneson+pc_mito+brain_region+Sex	0.857	2776.000
ENSG00000164307	0.810	0.021	0.212	-0.170	ERAP1	0.909	~p_tau+(1|manifest)+cngeneson+pc_mito+brain_region+Sex	3.146	725.000
ENSG00000135912	0.612	0.021	0.231	-0.188	TTLL4	0.793	~p_tau+(1|manifest)+cngeneson+pc_mito+brain_region+Sex	0.311	4768.000
ENSG00000122299	0.740	0.021	0.106	-0.063	ZC3H7A	0.869	~p_tau+(1|manifest)+cngeneson+pc_mito+brain_region+Sex	0.678	3277.000
ENSG00000196968	0.448	0.021	0.144	-0.101	FUT11	0.684	~p_tau+(1|manifest)+cngeneson+pc_mito+brain_region+Sex	0.281	4920.000
ENSG00000141556	0.933	0.021	0.138	-0.096	TBCD	0.968	~p_tau+(1|manifest)+cngeneson+pc_mito+brain_region+Sex	0.818	2893.000
ENSG00000142002	0.784	0.021	0.106	-0.064	DPP9	0.893	~p_tau+(1|manifest)+cngeneson+pc_mito+brain_region+Sex	0.809	2913.000
ENSG00000170088	0.520	0.021	0.113	-0.071	TMEM192	0.734	~p_tau+(1|manifest)+cngeneson+pc_mito+brain_region+Sex	0.518	3847.000
ENSG00000145604	0.019	0.021	0.148	-0.106	SKP2	0.126	~p_tau+(1|manifest)+cngeneson+pc_mito+brain_region+Sex	0.975	2484.000
ENSG00000112659	0.512	0.021	0.081	-0.039	CUL9	0.730	~p_tau+(1|manifest)+cngeneson+pc_mito+brain_region+Sex	0.346	4599.000
ENSG00000175866	0.414	0.021	0.125	-0.083	BAIAP2	0.658	~p_tau+(1|manifest)+cngeneson+pc_mito+brain_region+Sex	0.380	4442.000
ENSG00000165934	0.679	0.021	0.081	-0.039	CPSF2	0.838	~p_tau+(1|manifest)+cngeneson+pc_mito+brain_region+Sex	0.162	5349.000
ENSG00000120727	0.433	0.021	0.139	-0.097	PAIP2	0.674	~p_tau+(1|manifest)+cngeneson+pc_mito+brain_region+Sex	0.787	2968.000
ENSG00000010244	0.518	0.021	0.175	-0.134	ZNF207	0.733	~p_tau+(1|manifest)+cngeneson+pc_mito+brain_region+Sex	0.384	4422.000
ENSG00000131067	0.571	0.021	0.119	-0.077	GGT7	0.769	~p_tau+(1|manifest)+cngeneson+pc_mito+brain_region+Sex	0.609	3497.000
ENSG00000121289	0.653	0.021	0.082	-0.040	CEP89	0.820	~p_tau+(1|manifest)+cngeneson+pc_mito+brain_region+Sex	0.357	4540.000
ENSG00000133059	0.627	0.021	0.077	-0.035	DSTYK	0.804	~p_tau+(1|manifest)+cngeneson+pc_mito+brain_region+Sex	0.291	4857.000
ENSG00000137992	0.027	0.021	0.065	-0.023	DBT	0.152	~p_tau+(1|manifest)+cngeneson+pc_mito+brain_region+Sex	0.153	5375.000
ENSG00000136193	0.602	0.021	0.095	-0.053	SCRN1	0.788	~p_tau+(1|manifest)+cngeneson+pc_mito+brain_region+Sex	0.369	4480.000
ENSG00000180448	0.331	0.021	0.233	-0.192	ARHGAP45	0.588	~p_tau+(1|manifest)+cngeneson+pc_mito+brain_region+Sex	1.065	2318.000
ENSG00000029364	0.340	0.021	0.067	-0.026	SLC39A9	0.596	~p_tau+(1|manifest)+cngeneson+pc_mito+brain_region+Sex	0.253	5056.000
ENSG00000141367	0.984	0.021	0.257	-0.215	CLTC	0.992	~p_tau+(1|manifest)+cngeneson+pc_mito+brain_region+Sex	2.240	1106.000
ENSG00000084073	0.851	0.021	0.094	-0.053	ZMPSTE24	0.932	~p_tau+(1|manifest)+cngeneson+pc_mito+brain_region+Sex	0.596	3552.000
ENSG00000012061	0.431	0.021	0.129	-0.088	ERCC1	0.672	~p_tau+(1|manifest)+cngeneson+pc_mito+brain_region+Sex	0.977	2480.000
ENSG00000107938	0.058	0.021	0.077	-0.036	EDRF1	0.230	~p_tau+(1|manifest)+cngeneson+pc_mito+brain_region+Sex	0.230	5142.000
ENSG00000186001	0.608	0.021	0.100	-0.059	LRCH3	0.791	~p_tau+(1|manifest)+cngeneson+pc_mito+brain_region+Sex	0.494	3961.000
ENSG00000114120	0.473	0.020	0.132	-0.091	SLC25A36	0.702	~p_tau+(1|manifest)+cngeneson+pc_mito+brain_region+Sex	0.844	2811.000
ENSG00000129128	0.776	0.020	0.211	-0.170	SPCS3	0.889	~p_tau+(1|manifest)+cngeneson+pc_mito+brain_region+Sex	1.924	1297.000
ENSG00000103449	0.987	0.020	0.277	-0.236	SALL1	0.993	~p_tau+(1|manifest)+cngeneson+pc_mito+brain_region+Sex	2.217	1126.000
ENSG00000087301	0.495	0.020	0.123	-0.083	TXNDC16	0.717	~p_tau+(1|manifest)+cngeneson+pc_mito+brain_region+Sex	0.898	2673.000
ENSG00000162882	0.661	0.020	0.123	-0.083	HAAO	0.825	~p_tau+(1|manifest)+cngeneson+pc_mito+brain_region+Sex	0.682	3259.000
ENSG00000074319	0.730	0.020	0.074	-0.034	TSG101	0.864	~p_tau+(1|manifest)+cngeneson+pc_mito+brain_region+Sex	0.176	5309.000
ENSG00000186088	0.753	0.020	0.212	-0.172	GSAP	0.875	~p_tau+(1|manifest)+cngeneson+pc_mito+brain_region+Sex	1.454	1714.000
ENSG00000184432	0.254	0.020	0.075	-0.036	COPB2	0.508	~p_tau+(1|manifest)+cngeneson+pc_mito+brain_region+Sex	0.363	4510.000
ENSG00000151233	0.518	0.020	0.092	-0.052	GXYLT1	0.733	~p_tau+(1|manifest)+cngeneson+pc_mito+brain_region+Sex	0.329	4686.000
ENSG00000160218	0.932	0.020	0.154	-0.114	TRAPPC10	0.967	~p_tau+(1|manifest)+cngeneson+pc_mito+brain_region+Sex	1.432	1742.000
ENSG00000104517	0.968	0.020	0.229	-0.190	UBR5	0.985	~p_tau+(1|manifest)+cngeneson+pc_mito+brain_region+Sex	2.588	933.000
ENSG00000011405	0.886	0.020	0.107	-0.067	PIK3C2A	0.948	~p_tau+(1|manifest)+cngeneson+pc_mito+brain_region+Sex	0.406	4333.000
ENSG00000005801	0.658	0.020	0.124	-0.084	ZNF195	0.823	~p_tau+(1|manifest)+cngeneson+pc_mito+brain_region+Sex	1.158	2147.000
ENSG00000273294	0.868	0.020	0.117	-0.077	[illegible]	0.939	~p_tau+(1|manifest)+cngeneson+pc_mito+brain_region+Sex	0.484	4003.000
ENSG00000059377	0.781	0.020	0.113	-0.074	TBXAS1	0.891	~p_tau+(1|manifest)+cngeneson+pc_mito+brain_region+Sex	3.584	625.000
ENSG00000080815	0.944	0.020	0.178	-0.138	PSEN1	0.973	~p_tau+(1|manifest)+cngeneson+pc_mito+brain_region+Sex	0.741	3078.000
ENSG00000185716	0.521	0.020	0.180	-0.140	MOSMO	0.735	~p_tau+(1|manifest)+cngeneson+pc_mito+brain_region+Sex	1.358	1818.000
ENSG00000154582	0.096	0.020	0.091	-0.052	ELOC	0.296	~p_tau+(1|manifest)+cngeneson+pc_mito+brain_region+Sex	0.565	3664.000
ENSG00000137413	0.043	0.020	0.059	-0.020	TAF8	0.195	~p_tau+(1|manifest)+cngeneson+pc_mito+brain_region+Sex	0.185	5279.000
ENSG00000184445	0.553	0.020	0.088	-0.048	KNTC1	0.759	~p_tau+(1|manifest)+cngeneson+pc_mito+brain_region+Sex	0.706	3192.000
ENSG00000163946	0.721	0.020	0.132	-0.093	TASOR	0.861	~p_tau+(1|manifest)+cngeneson+pc_mito+brain_region+Sex	0.293	4847.000
ENSG00000176986	0.447	0.019	0.059	-0.020	SEC24C	0.683	~p_tau+(1|manifest)+cngeneson+pc_mito+brain_region+Sex	0.192	5257.000
ENSG00000169155	0.206	0.019	0.082	-0.044	ZBTB43	0.450	~p_tau+(1|manifest)+cngeneson+pc_mito+brain_region+Sex	0.479	4029.000
ENSG00000011422	0.782	0.019	0.202	-0.163	PLAUR	0.892	~p_tau+(1|manifest)+cngeneson+pc_mito+brain_region+Sex	2.137	1159.000
ENSG00000101079	0.716	0.019	0.147	-0.109	NDRG3	0.858	~p_tau+(1|manifest)+cngeneson+pc_mito+brain_region+Sex	0.458	4112.000
ENSG00000229117	0.634	0.019	0.325	-0.287	RPL41	0.807	~p_tau+(1|manifest)+cngeneson+pc_mito+brain_region+Sex	3.551	627.000
ENSG00000138107	0.326	0.019	0.071	-0.033	ACTR1A	0.584	~p_tau+(1|manifest)+cngeneson+pc_mito+brain_region+Sex	0.394	4382.000
ENSG00000163104	0.933	0.019	0.143	-0.105	SMARCAD1	0.968	~p_tau+(1|manifest)+cngeneson+pc_mito+brain_region+Sex	0.722	3137.000
ENSG00000070061	0.632	0.019	0.074	-0.036	ELP1	0.807	~p_tau+(1|manifest)+cngeneson+pc_mito+brain_region+Sex	0.373	4465.000
ENSG00000114107	0.827	0.019	0.081	-0.043	CEP70	0.919	~p_tau+(1|manifest)+cngeneson+pc_mito+brain_region+Sex	0.169	5333.000
ENSG00000157181	0.748	0.019	0.108	-0.071	ODR4	0.873	~p_tau+(1|manifest)+cngeneson+pc_mito+brain_region+Sex	0.404	4339.000
ENSG00000108091	0.857	0.019	0.097	-0.060	CCDC6	0.935	~p_tau+(1|manifest)+cngeneson+pc_mito+brain_region+Sex	0.187	5276.000
ENSG00000213341	0.706	0.019	0.068	-0.031	CHUK	0.852	~p_tau+(1|manifest)+cngeneson+pc_mito+brain_region+Sex	0.357	4539.000
ENSG00000095139	0.491	0.019	0.062	-0.025	ARCN1	0.714	~p_tau+(1|manifest)+cngeneson+pc_mito+brain_region+Sex	0.293	4850.000
ENSG00000205581	0.779	0.019	0.206	-0.169	HMGN1	0.890	~p_tau+(1|manifest)+cngeneson+pc_mito+brain_region+Sex	3.180	715.000
ENSG00000022277	0.644	0.019	0.071	-0.033	RTF2	0.814	~p_tau+(1|manifest)+cngeneson+pc_mito+brain_region+Sex	0.137	5410.000
ENSG00000135148	0.593	0.019	0.102	-0.065	TRAFD1	0.783	~p_tau+(1|manifest)+cngeneson+pc_mito+brain_region+Sex	0.564	3666.000
ENSG00000111364	0.643	0.018	0.110	-0.073	DDX55	0.813	~p_tau+(1|manifest)+cngeneson+pc_mito+brain_region+Sex	0.837	2837.000
ENSG00000164066	0.727	0.018	0.077	-0.040	INTU	0.863	~p_tau+(1|manifest)+cngeneson+pc_mito+brain_region+Sex	0.374	4459.000
ENSG00000131269	0.439	0.018	0.109	-0.072	ABCB7	0.680	~p_tau+(1|manifest)+cngeneson+pc_mito+brain_region+Sex	0.655	3342.000
ENSG00000124532	0.098	0.018	0.064	-0.027	MRS2	0.298	~p_tau+(1|manifest)+cngeneson+pc_mito+brain_region+Sex	0.255	5051.000
ENSG00000163125	0.934	0.018	0.115	-0.079	RPRD2	0.969	~p_tau+(1|manifest)+cngeneson+pc_mito+brain_region+Sex	0.154	5373.000
ENSG00000232119	0.249	0.018	0.060	-0.024	MCTS1	0.501	~p_tau+(1|manifest)+cngeneson+pc_mito+brain_region+Sex	0.190	5261.000
ENSG00000120137	0.108	0.018	0.123	-0.086	PANK3	0.315	~p_tau+(1|manifest)+cngeneson+pc_mito+brain_region+Sex	0.321	4724.000
ENSG00000156313	0.489	0.018	0.049	-0.012	RPGR	0.713	~p_tau+(1|manifest)+cngeneson+pc_mito+brain_region+Sex	0.105	5449.000
ENSG00000133703	0.339	0.018	0.079	-0.043	KRAS	0.595	~p_tau+(1|manifest)+cngeneson+pc_mito+brain_region+Sex	0.469	4066.000
ENSG00000162623	0.025	0.018	0.086	-0.051	TYW3	0.146	~p_tau+(1|manifest)+cngeneson+pc_mito+brain_region+Sex	0.469	4067.000
ENSG00000189180	0.979	0.018	0.239	-0.204	ZNF33A	0.990	~p_tau+(1|manifest)+cngeneson+pc_mito+brain_region+Sex	0.786	2970.000
ENSG00000085382	0.435	0.018	0.178	-0.143	HACE1	0.674	~p_tau+(1|manifest)+cngeneson+pc_mito+brain_region+Sex	0.571	3642.000
ENSG00000184203	0.633	0.018	0.102	-0.066	PPP1R2	0.807	~p_tau+(1|manifest)+cngeneson+pc_mito+brain_region+Sex	0.503	3917.000
ENSG00000087502	0.058	0.018	0.082	-0.047	ERGIC2	0.230	~p_tau+(1|manifest)+cngeneson+pc_mito+brain_region+Sex	0.570	3649.000
ENSG00000170322	0.434	0.017	0.053	-0.018	NFRKB	0.674	~p_tau+(1|manifest)+cngeneson+pc_mito+brain_region+Sex	0.209	5210.000
ENSG00000127080	0.425	0.017	0.077	-0.042	IPPK	0.668	~p_tau+(1|manifest)+cngeneson+pc_mito+brain_region+Sex	0.552	3711.000
ENSG00000005020	0.955	0.017	0.133	-0.098	SKAP2	0.978	~p_tau+(1|manifest)+cngeneson+pc_mito+brain_region+Sex	1.367	1810.000
ENSG00000122122	0.133	0.017	0.112	-0.077	SASH3	0.354	~p_tau+(1|manifest)+cngeneson+pc_mito+brain_region+Sex	0.608	3504.000
ENSG00000047932	0.882	0.017	0.101	-0.066	GOPC	0.946	~p_tau+(1|manifest)+cngeneson+pc_mito+brain_region+Sex	0.278	4935.000
ENSG00000165733	0.835	0.017	0.074	-0.040	BMS1	0.923	~p_tau+(1|manifest)+cngeneson+pc_mito+brain_region+Sex	0.265	4991.000
ENSG00000105053	0.551	0.017	0.054	-0.019	VRK3	0.756	~p_tau+(1|manifest)+cngeneson+pc_mito+brain_region+Sex	0.142	5401.000
ENSG00000093072	0.878	0.017	0.211	-0.177	ADA2	0.944	~p_tau+(1|manifest)+cngeneson+pc_mito+brain_region+Sex	1.851	1350.000
ENSG00000115267	0.929	0.017	0.105	-0.071	IFIH1	0.967	~p_tau+(1|manifest)+cngeneson+pc_mito+brain_region+Sex	0.697	3217.000
ENSG00000182872	0.241	0.017	0.061	-0.028	RBM10	0.492	~p_tau+(1|manifest)+cngeneson+pc_mito+brain_region+Sex	0.098	5452.000
ENSG00000108587	0.410	0.017	0.119	-0.085	GOSR1	0.655	~p_tau+(1|manifest)+cngeneson+pc_mito+brain_region+Sex	0.498	3944.000
ENSG00000105738	0.850	0.017	0.138	-0.105	SIPA1L3	0.931	~p_tau+(1|manifest)+cngeneson+pc_mito+brain_region+Sex	0.379	4443.000
ENSG00000116729	0.924	0.017	0.181	-0.147	WLS	0.965	~p_tau+(1|manifest)+cngeneson+pc_mito+brain_region+Sex	0.979	2475.000
ENSG00000126970	0.086	0.017	0.071	-0.038	ZC4H2	0.279	~p_tau+(1|manifest)+cngeneson+pc_mito+brain_region+Sex	0.271	4965.000
ENSG00000042317	0.549	0.017	0.075	-0.042	SPATA7	0.756	~p_tau+(1|manifest)+cngeneson+pc_mito+brain_region+Sex	0.538	3772.000
ENSG00000049618	0.944	0.017	0.123	-0.089	ARID1B	0.973	~p_tau+(1|manifest)+cngeneson+pc_mito+brain_region+Sex	1.778	1403.000
ENSG00000094841	0.714	0.017	0.058	-0.025	UPRT	0.857	~p_tau+(1|manifest)+cngeneson+pc_mito+brain_region+Sex	0.195	5243.000
ENSG00000067704	0.685	0.017	0.057	-0.024	IARS2	0.841	~p_tau+(1|manifest)+cngeneson+pc_mito+brain_region+Sex	0.430	4215.000
ENSG00000151414	0.923	0.017	0.287	-0.254	NEK7	0.965	~p_tau+(1|manifest)+cngeneson+pc_mito+brain_region+Sex	2.850	817.000
ENSG00000148356	0.922	0.017	0.121	-0.088	LRSAM1	0.964	~p_tau+(1|manifest)+cngeneson+pc_mito+brain_region+Sex	0.721	3139.000
ENSG00000185049	0.493	0.017	0.061	-0.028	NELFA	0.716	~p_tau+(1|manifest)+cngeneson+pc_mito+brain_region+Sex	0.208	5219.000
ENSG00000050130	0.426	0.016	0.109	-0.076	JKAMP	0.669	~p_tau+(1|manifest)+cngeneson+pc_mito+brain_region+Sex	0.546	3741.000
ENSG00000168395	0.504	0.016	0.075	-0.042	ING5	0.724	~p_tau+(1|manifest)+cngeneson+pc_mito+brain_region+Sex	0.254	5055.000
ENSG00000215193	0.506	0.016	0.049	-0.016	PEX26	0.726	~p_tau+(1|manifest)+cngeneson+pc_mito+brain_region+Sex	0.142	5402.000
ENSG00000133114	0.883	0.016	0.082	-0.050	GPALPP1	0.946	~p_tau+(1|manifest)+cngeneson+pc_mito+brain_region+Sex	0.286	4890.000
ENSG00000140521	0.136	0.016	0.065	-0.032	POLG	0.357	~p_tau+(1|manifest)+cngeneson+pc_mito+brain_region+Sex	0.337	4642.000
ENSG00000118960	0.778	0.016	0.063	-0.030	HS1BP3	0.889	~p_tau+(1|manifest)+cngeneson+pc_mito+brain_region+Sex	0.260	5024.000
ENSG00000005007	0.406	0.016	0.110	-0.077	UPF1	0.653	~p_tau+(1|manifest)+cngeneson+pc_mito+brain_region+Sex	0.328	4693.000
ENSG00000111850	0.721	0.016	0.056	-0.023	SMIM8	0.860	~p_tau+(1|manifest)+cngeneson+pc_mito+brain_region+Sex	0.185	5282.000
ENSG00000118246	0.892	0.016	0.085	-0.052	FASTKD2	0.950	~p_tau+(1|manifest)+cngeneson+pc_mito+brain_region+Sex	0.662	3317.000
ENSG00000115993	0.912	0.016	0.092	-0.059	TRAK2	0.959	~p_tau+(1|manifest)+cngeneson+pc_mito+brain_region+Sex	0.118	5437.000
ENSG00000113532	0.912	0.016	0.226	-0.193	ST8SIA4	0.959	~p_tau+(1|manifest)+cngeneson+pc_mito+brain_region+Sex	0.991	2452.000
ENSG00000144791	0.721	0.016	0.223	-0.190	LIMD1	0.860	~p_tau+(1|manifest)+cngeneson+pc_mito+brain_region+Sex	0.676	3279.000
ENSG00000142687	0.956	0.016	0.128	-0.095	KIAA0319L	0.978	~p_tau+(1|manifest)+cngeneson+pc_mito+brain_region+Sex	0.617	3471.000
ENSG00000142937	0.996	0.016	0.453	-0.421	RPS8	0.998	~p_tau+(1|manifest)+cngeneson+pc_mito+brain_region+Sex	9.433	120.000
ENSG00000090857	0.659	0.016	0.172	-0.140	PDPR	0.823	~p_tau+(1|manifest)+cngeneson+pc_mito+brain_region+Sex	0.946	2555.000
ENSG00000152219	0.893	0.016	0.106	-0.074	ARL14EP	0.951	~p_tau+(1|manifest)+cngeneson+pc_mito+brain_region+Sex	0.220	5173.000
ENSG00000186141	0.834	0.016	0.093	-0.061	POLR3C	0.923	~p_tau+(1|manifest)+cngeneson+pc_mito+brain_region+Sex	0.396	4375.000
ENSG00000114956	0.610	0.016	0.088	-0.057	DGUOK	0.793	~p_tau+(1|manifest)+cngeneson+pc_mito+brain_region+Sex	0.629	3425.000
ENSG00000180902	0.860	0.016	0.159	-0.127	D2HGDH	0.937	~p_tau+(1|manifest)+cngeneson+pc_mito+brain_region+Sex	2.102	1186.000
ENSG00000115204	0.030	0.016	0.072	-0.041	MPV17	0.160	~p_tau+(1|manifest)+cngeneson+pc_mito+brain_region+Sex	0.439	4178.000
ENSG00000136636	0.581	0.016	0.065	-0.034	KCTD3	0.776	~p_tau+(1|manifest)+cngeneson+pc_mito+brain_region+Sex	0.270	4972.000
ENSG00000131100	0.719	0.016	0.054	-0.023	ATP6V1E1	0.859	~p_tau+(1|manifest)+cngeneson+pc_mito+brain_region+Sex	0.429	4222.000
ENSG00000108443	0.661	0.015	0.082	-0.051	RPS6KB1	0.826	~p_tau+(1|manifest)+cngeneson+pc_mito+brain_region+Sex	0.403	4343.000
ENSG00000101266	0.709	0.015	0.138	-0.107	CSNK2A1	0.854	~p_tau+(1|manifest)+cngeneson+pc_mito+brain_region+Sex	0.637	3400.000
ENSG00000163902	0.652	0.015	0.124	-0.094	RPN1	0.820	~p_tau+(1|manifest)+cngeneson+pc_mito+brain_region+Sex	0.666	3308.000
ENSG00000131127	0.898	0.015	0.083	-0.053	ZNF141	0.953	~p_tau+(1|manifest)+cngeneson+pc_mito+brain_region+Sex	0.385	4417.000
ENSG00000186625	0.707	0.015	0.068	-0.038	KATNA1	0.852	~p_tau+(1|manifest)+cngeneson+pc_mito+brain_region+Sex	0.509	3883.000
ENSG00000130332	0.742	0.015	0.123	-0.093	LSM7	0.870	~p_tau+(1|manifest)+cngeneson+pc_mito+brain_region+Sex	1.169	2128.000
ENSG00000013288	0.800	0.015	0.073	-0.042	MAN2B2	0.903	~p_tau+(1|manifest)+cngeneson+pc_mito+brain_region+Sex	0.264	4999.000
ENSG00000162086	0.811	0.015	0.084	-0.054	ZNF75A	0.909	~p_tau+(1|manifest)+cngeneson+pc_mito+brain_region+Sex	0.545	3743.000
ENSG00000152104	0.722	0.015	0.283	-0.252	PTPN14	0.861	~p_tau+(1|manifest)+cngeneson+pc_mito+brain_region+Sex	3.508	635.000
ENSG00000137802	0.278	0.015	0.081	-0.051	MAPKBP1	0.532	~p_tau+(1|manifest)+cngeneson+pc_mito+brain_region+Sex	0.266	4989.000
ENSG00000187676	0.932	0.015	0.123	-0.093	B3GLCT	0.967	~p_tau+(1|manifest)+cngeneson+pc_mito+brain_region+Sex	0.435	4199.000
ENSG00000163947	0.951	0.015	0.110	-0.080	ARHGEF3	0.976	~p_tau+(1|manifest)+cngeneson+pc_mito+brain_region+Sex	0.838	2832.000
ENSG00000182095	0.965	0.015	0.225	-0.196	TNRC18	0.983	~p_tau+(1|manifest)+cngeneson+pc_mito+brain_region+Sex	2.120	1171.000
ENSG00000164221	0.343	0.015	0.142	-0.113	CCDC112	0.597	~p_tau+(1|manifest)+cngeneson+pc_mito+brain_region+Sex	0.399	4362.000
ENSG00000111231	0.531	0.015	0.150	-0.120	GPN3	0.740	~p_tau+(1|manifest)+cngeneson+pc_mito+brain_region+Sex	0.762	3034.000
ENSG00000156171	0.398	0.015	0.223	-0.193	DRAM2	0.644	~p_tau+(1|manifest)+cngeneson+pc_mito+brain_region+Sex	1.069	2311.000
ENSG00000143256	0.653	0.015	0.092	-0.063	PFDN2	0.820	~p_tau+(1|manifest)+cngeneson+pc_mito+brain_region+Sex	0.536	3778.000
ENSG00000124275	0.915	0.015	0.085	-0.055	MTRR	0.960	~p_tau+(1|manifest)+cngeneson+pc_mito+brain_region+Sex	0.508	3893.000
ENSG00000109762	0.971	0.015	0.167	-0.138	SNX25	0.987	~p_tau+(1|manifest)+cngeneson+pc_mito+brain_region+Sex	0.367	4487.000
ENSG00000136161	0.900	0.014	0.270	-0.241	RCBTB2	0.953	~p_tau+(1|manifest)+cngeneson+pc_mito+brain_region+Sex	0.856	2778.000
ENSG00000069248	0.321	0.014	0.068	-0.040	NUP133	0.577	~p_tau+(1|manifest)+cngeneson+pc_mito+brain_region+Sex	0.265	4995.000
ENSG00000123095	0.835	0.014	0.283	-0.254	BHLHE41	0.923	~p_tau+(1|manifest)+cngeneson+pc_mito+brain_region+Sex	6.171	268.000
ENSG00000112992	0.625	0.014	0.088	-0.060	NNT	0.802	~p_tau+(1|manifest)+cngeneson+pc_mito+brain_region+Sex	0.581	3609.000
ENSG00000149658	0.484	0.014	0.058	-0.029	YTHDF1	0.710	~p_tau+(1|manifest)+cngeneson+pc_mito+brain_region+Sex	0.267	4980.000
ENSG00000198919	0.798	0.014	0.064	-0.035	DZIP3	0.902	~p_tau+(1|manifest)+cngeneson+pc_mito+brain_region+Sex	0.201	5232.000
ENSG00000115935	0.653	0.014	0.152	-0.124	WIPF1	0.820	~p_tau+(1|manifest)+cngeneson+pc_mito+brain_region+Sex	0.603	3524.000
ENSG00000105281	0.808	0.014	0.173	-0.144	SLC1A5	0.907	~p_tau+(1|manifest)+cngeneson+pc_mito+brain_region+Sex	1.248	2005.000
ENSG00000117682	0.410	0.014	0.069	-0.041	DHDDS	0.655	~p_tau+(1|manifest)+cngeneson+pc_mito+brain_region+Sex	0.738	3090.000
ENSG00000128915	0.273	0.014	0.170	-0.142	ICE2	0.527	~p_tau+(1|manifest)+cngeneson+pc_mito+brain_region+Sex	0.834	2845.000
ENSG00000022840	0.748	0.014	0.069	-0.040	RNF10	0.873	~p_tau+(1|manifest)+cngeneson+pc_mito+brain_region+Sex	0.402	4344.000
ENSG00000117528	0.830	0.014	0.081	-0.053	ABCD3	0.921	~p_tau+(1|manifest)+cngeneson+pc_mito+brain_region+Sex	0.217	5180.000
ENSG00000168411	0.397	0.014	0.085	-0.056	RFWD3	0.644	~p_tau+(1|manifest)+cngeneson+pc_mito+brain_region+Sex	0.334	4662.000
ENSG00000149932	0.044	0.014	0.081	-0.053	TMEM219	0.198	~p_tau+(1|manifest)+cngeneson+pc_mito+brain_region+Sex	0.538	3765.000
ENSG00000163399	0.975	0.014	0.140	-0.112	ATP1A1	0.989	~p_tau+(1|manifest)+cngeneson+pc_mito+brain_region+Sex	1.042	2362.000
ENSG00000110079	0.459	0.014	0.210	-0.182	MS4A4A	0.692	~p_tau+(1|manifest)+cngeneson+pc_mito+brain_region+Sex	2.058	1206.000
ENSG00000100997	0.729	0.014	0.320	-0.292	ABHD12	0.863	~p_tau+(1|manifest)+cngeneson+pc_mito+brain_region+Sex	5.137	350.000
ENSG00000141905	0.991	0.014	0.214	-0.186	NFIC	0.994	~p_tau+(1|manifest)+cngeneson+pc_mito+brain_region+Sex	1.983	1257.000
ENSG00000103319	0.867	0.014	0.102	-0.075	EEF2K	0.939	~p_tau+(1|manifest)+cngeneson+pc_mito+brain_region+Sex	0.781	2986.000
ENSG00000116641	0.987	0.014	0.195	-0.167	DOCK7	0.993	~p_tau+(1|manifest)+cngeneson+pc_mito+brain_region+Sex	1.632	1523.000
ENSG00000167232	0.955	0.014	0.117	-0.090	ZNF91	0.978	~p_tau+(1|manifest)+cngeneson+pc_mito+brain_region+Sex	0.725	3129.000
ENSG00000135336	0.681	0.014	0.090	-0.063	ORC3	0.839	~p_tau+(1|manifest)+cngeneson+pc_mito+brain_region+Sex	0.269	4975.000
ENSG00000178694	0.930	0.014	0.096	-0.069	NSUN3	0.967	~p_tau+(1|manifest)+cngeneson+pc_mito+brain_region+Sex	0.227	5150.000
ENSG00000160255	0.641	0.014	0.201	-0.174	ITGB2	0.811	~p_tau+(1|manifest)+cngeneson+pc_mito+brain_region+Sex	2.625	922.000
ENSG00000163558	0.611	0.014	0.050	-0.023	PRKCI	0.793	~p_tau+(1|manifest)+cngeneson+pc_mito+brain_region+Sex	0.174	5316.000
ENSG00000127914	0.982	0.014	0.231	-0.204	AKAP9	0.991	~p_tau+(1|manifest)+cngeneson+pc_mito+brain_region+Sex	1.653	1494.000
ENSG00000121766	0.770	0.013	0.051	-0.024	ZCCHC17	0.887	~p_tau+(1|manifest)+cngeneson+pc_mito+brain_region+Sex	0.365	4500.000
ENSG00000011304	0.264	0.013	0.094	-0.067	PTBP1	0.517	~p_tau+(1|manifest)+cngeneson+pc_mito+brain_region+Sex	0.373	4466.000
ENSG00000232593	0.936	0.013	0.205	-0.179	KANTR	0.970	~p_tau+(1|manifest)+cngeneson+pc_mito+brain_region+Sex	1.207	2069.000
ENSG00000083520	0.721	0.013	0.072	-0.045	DIS3	0.860	~p_tau+(1|manifest)+cngeneson+pc_mito+brain_region+Sex	0.408	4322.000
ENSG00000165699	0.719	0.013	0.083	-0.056	TSC1	0.859	~p_tau+(1|manifest)+cngeneson+pc_mito+brain_region+Sex	0.347	4596.000
ENSG00000198252	0.137	0.013	0.057	-0.030	STYX	0.358	~p_tau+(1|manifest)+cngeneson+pc_mito+brain_region+Sex	0.262	5017.000
ENSG00000121644	0.578	0.013	0.104	-0.077	DESI2	0.774	~p_tau+(1|manifest)+cngeneson+pc_mito+brain_region+Sex	0.271	4964.000
ENSG00000144635	0.916	0.013	0.084	-0.058	DYNC1LI1	0.960	~p_tau+(1|manifest)+cngeneson+pc_mito+brain_region+Sex	0.292	4855.000
ENSG00000130255	0.482	0.013	0.307	-0.280	RPL36	0.708	~p_tau+(1|manifest)+cngeneson+pc_mito+brain_region+Sex	2.173	1144.000
ENSG00000103043	0.563	0.013	0.083	-0.057	VAC14	0.764	~p_tau+(1|manifest)+cngeneson+pc_mito+brain_region+Sex	0.393	4387.000
ENSG00000078668	0.394	0.013	0.080	-0.054	VDAC3	0.641	~p_tau+(1|manifest)+cngeneson+pc_mito+brain_region+Sex	0.776	2997.000
ENSG00000143774	0.979	0.013	0.155	-0.129	GUK1	0.990	~p_tau+(1|manifest)+cngeneson+pc_mito+brain_region+Sex	1.601	1557.000
ENSG00000186432	0.948	0.013	0.122	-0.096	KPNA4	0.974	~p_tau+(1|manifest)+cngeneson+pc_mito+brain_region+Sex	0.346	4604.000
ENSG00000132849	0.015	0.013	0.248	-0.222	PATJ	0.110	~p_tau+(1|manifest)+cngeneson+pc_mito+brain_region+Sex	4.408	455.000
ENSG00000168092	0.860	0.013	0.061	-0.036	PAFAH1B2	0.937	~p_tau+(1|manifest)+cngeneson+pc_mito+brain_region+Sex	0.287	4885.000
ENSG00000006831	0.780	0.013	0.166	-0.140	ADIPOR2	0.891	~p_tau+(1|manifest)+cngeneson+pc_mito+brain_region+Sex	0.880	2714.000
ENSG00000034693	0.412	0.013	0.058	-0.032	PEX3	0.657	~p_tau+(1|manifest)+cngeneson+pc_mito+brain_region+Sex	0.226	5155.000
ENSG00000143569	0.476	0.013	0.099	-0.073	UBAP2L	0.703	~p_tau+(1|manifest)+cngeneson+pc_mito+brain_region+Sex	0.507	3896.000
ENSG00000196865	0.797	0.013	0.134	-0.108	NHLRC2	0.902	~p_tau+(1|manifest)+cngeneson+pc_mito+brain_region+Sex	0.215	5196.000
ENSG00000139163	0.716	0.013	0.217	-0.191	ETNK1	0.858	~p_tau+(1|manifest)+cngeneson+pc_mito+brain_region+Sex	1.193	2089.000
ENSG00000180304	0.597	0.013	0.077	-0.052	OAZ2	0.785	~p_tau+(1|manifest)+cngeneson+pc_mito+brain_region+Sex	0.615	3479.000
[truncated: 3,259,563 more chars]
